# Genes and gene expression modules associated with caloric restriction and aging in the laboratory mouse

**DOI:** 10.1186/1471-2164-10-585

**Published:** 2009-12-07

**Authors:** William R Swindell

**Affiliations:** 1 Department of Pathology, University of Michigan Medical School, Ann Arbor, MI 48109-2200, USA; 2 Department of Geriatrics, University of Michigan Medical School, Ann Arbor, MI 48109-2200, USA

## Abstract

**Background:**

Caloric restriction (CR) counters deleterious effects of aging and, for most mouse genotypes, increases mean and maximum lifespan. Previous analyses of microarray data have identified gene expression responses to CR that are shared among multiple mouse tissues, including the activation of anti-oxidant, tumor suppressor and anti-inflammatory pathways. These analyses have provided useful research directions, but have been restricted to a limited number of tissues, and have focused on individual genes, rather than whole-genome transcriptional networks. Furthermore, CR is thought to oppose age-associated gene expression patterns, but detailed statistical investigations of this hypothesis have not been carried out.

**Results:**

Systemic effects of CR and aging were identified by examining transcriptional responses to CR in 17 mouse tissue types, as well as responses to aging in 22 tissues. CR broadly induced the expression of genes known to inhibit oxidative stress (e.g., *Mt1*, *Mt2*), inflammation (e.g., *Nfkbia*, *Timp3*) and tumorigenesis (e.g., *Txnip*, *Zbtb16*). Additionally, a network-based investigation revealed that CR regulates a large co-expression module containing genes associated with the metabolism and splicing of mRNA (e.g., *Cpsf6*, *Sfpq*, *Sfrs18*). The effects of aging were, to a considerable degree, similar among groups of co-expressed genes. Age-related gene expression patterns characteristic of most mouse tissues were identified, including up regulation of granulin (*Grn*) and secreted phosphoprotein 1 (*Spp1*). The transcriptional association between CR and aging varied at different levels of analysis. With respect to gene subsets associated with certain biological processes (e.g., immunity and inflammation), CR opposed age-associated expression patterns. However, among all genes, global transcriptional effects of CR were only weakly related to those of aging.

**Conclusion:**

The study of aging, and of interventions thought to combat aging, has much to gain from data-driven and unbiased genomic investigations. Expression patterns identified in this analysis characterize a generalized response of mammalian cells to CR and/or aging. These patterns may be of importance in determining effects of CR on overall lifespan, or as factors that underlie age-related disease. The association between CR and aging warrants further study, but most evidence indicates that CR does not induce a genome-wide "reversal" of age-associated gene expression patterns.

## Background

The consumption of a low-calorie, but nutritionally sufficient, diet has been found to improve indices of disease risk in humans, and has been studied as an intervention that increases lifespan in mice of most genetic backgrounds. These effects of calorie restriction (CR) are hypothesized to reflect not only prevention of particular age-related diseases, but an actual interference with the process of aging [1-3]. The physiological effects of CR have therefore been of great interest from the standpoint of human health [4], and much effort has been devoted to development of drug compounds that "mimic" beneficial aspects of low-calorie diets, without requiring a reduction in overall caloric intake [5]. Despite decades of work examining rodent responses to CR, however, the key biochemical pathways underlying positive effects of this diet on aging and lifespan remain to be elucidated [6]. The most well-studied genes and pathways have emerged from work in lower invertebrates, including the insulin/insulin-like growth factor 1 system, the mammalian target of rapamycin (mTOR), forkhead transcription factors, and the sirtuin family of histone deacetylase genes [7]. Establishing the relative importance of these and other pathways in the mammalian CR effect provides avenues for development of CR mimetic compounds, but also advances a mechanistic understanding that should shed light on unanswered questions. For instance, it is unclear whether beneficial effects of CR arise from restriction of specific nutrients (e.g., fat, methionine) [1], and for unknown reasons, effects of CR on lifespan differ among genotypes [8,9]. These issues may be addressed, in part, by identifying pathways perturbed by CR and understanding their functional contribution to increased lifespan.

Gene expression analyses using DNA microarrays cannot reveal whether genes are causally involved in effects of CR on lifespan, but are nonetheless powerful tools for identifying new, and possibly unexpected, pathways that warrant further experimental investigation [10,11]. In mice, previous studies have found that CR brings about large shifts in gene expression, including tissue-specific responses to CR, as well as a generalized or common CR response that is shared among tissues [12,13]. Both tissue-specific and common responses to CR are of potential importance, but common responses to CR across multiple tissue types provide especially attractive targets for further study. Swindell (2008) [12] analyzed transcriptional responses to CR across 10 tissue types in the laboratory mouse, and found that common responses to CR include activation of tumor suppressor genes, as well as anti-oxidant and anti-inflammatory pathways. For instance, CR was found to increase expression of metallothionein-encoding genes (*Mt1 *and *Mt2*) in several tissues, including liver, heart, muscle, hypothalamus, colon and lung. Additionally, in multiple tissues, CR increased expression of *Nfkbia*, an inhibitor of NF-*κ*B, suggesting a potential anti-inflammatory mechanism of CR diets [13]. More recently, microarrays have been used to evaluate effects of CR in previously unexamined tissues, including thymus, gonads, mammary gland, spinal cord, cerebellum and striatum [14-16]. These data allow common responses to CR be refined, should lend greater confidence to lists of CR-regulated genes and pathways, and provide an opportunity for applying improved statistical methods. For instance, previous investigations have focused on how CR affects individual genes, but this may reveal only part of the story. A more comprehensive and informative approach is to identify regions within the genome-wide transcriptional network that are activated or inhibited by CR [13].

Historically, the effects of CR have been viewed as being associated with the aging process [1-3]. This standpoint argues that effects of CR extend beyond any one disease process (e.g., tumorigenesis), but that CR has multiplex effects on a range of physiological systems, ultimately amounting to an inhibitory effect on the progression of aging. The association between CR and aging, however, remains poorly understood, largely because the aging process itself remains poorly defined [17]. While an uncontroversial definition of aging may not be developed anytime soon, it should be possible to add rigor to the concept by generating quantitative models of aging that are operationally useful. In this regard, whole-genome microarray datasets would seem especially valuable [18], and can be used to generate models that test, quantitatively, the assertion that CR acts to oppose the progression of aging [11]. Conclusions generated from previous investigations conflict regarding the association between the effects of CR and aging. On the one hand, an early investigation revealed that age-associated expression patterns in muscle were "either completely or partially prevented by caloric restriction" [19], and this conclusion was supported in subsequent studies [14,20,21]. Other investigations, however, have yielded different conclusions. For instance, effects of CR were entirely unrelated to those of aging in muscle tissue from Rhesus monkeys [22], and in one aptly designed experiment examining mouse cardiac tissue, only 79 of 1075 age-responsive genes (7.3%) were significantly altered by CR [23]. Clearly, experimental design and statistical methodology are two important considerations for evaluating this diverse set of results. Many studies, for instance, have not evaluated whether the observed overlap between CR and aging effects is larger than expected by chance alone. This statistical evaluation would not be straight-forward in many cases, since experiments involved a shared control treatment that was used to evaluate the effects of both aging and CR (e.g., a young control treatment, an old control treatment, and an old CR treatment). Given this design, the effect of CR is not estimated independently of the effect of aging, and some correspondence between CR and aging effects would be expected by chance [12].

The present study integrates results from a large number of microarray experiments that have evaluated the effects of CR or aging in the laboratory mouse. Collectively, experiments analyzed in this report have evaluated the effects of CR in 17 mouse tissue types, and the effects of aging in 22 mouse tissue types. For many tissues, effects of CR and/or aging have been evaluated in multiple studies, and results from separate studies of the same tissue are combined within a meta-analytic framework. The overall approach was shaped by three main objectives. First, it was of interest to identify robust expression patterns associated with CR and aging, particularly common responses occurring within multiple mouse tissues. Secondly, to move beyond single-gene-centric analyses and to take full advantage of the discernable co-expression relationships among genes, regions of the transcriptional network activated or inhibited by CR or aging were identified (i.e., gene co-expression modules). Lastly, based on this cross-study comparison of multiple datasets, it was of interest to statistically evaluate whether CR acted to oppose age-associated gene expression patterns. The overlap between the effects of CR and aging was therefore evaluated using several different methods, including overlap between differential expression signatures [24] and similarity between ordered gene lists [25].

## Results

### Genes regulated by caloric restriction in the laboratory mouse

The CR dataset is a compilation of 40 experiments that, taken together, have evaluated the effects of CR in 17 tissue types (Table 1). For most tissues, data from just 1-3 experiments was available, but for well-studied tissues (liver, muscle and heart), data was available from as many as 10 separate experiments (Table 1). Additional File 1 provides detailed information regarding all experiments incorporated into the analysis, including mouse strains used in each study, sample sizes, array platforms, and whether experimental results from a given study were validated using RT-PCR. Differential expression results from independent studies were combined using Fisher's p-value combination method (see Methods). The symbol P_*u *_is used throughout to denote a p-value generated from a one-sided hypothesis test for up regulation by CR (or aging), while P_*d *_denotes a p-value generated from a one-sided hypothesis test for down regulation by CR (or aging). The customary P (without subscript) represents a two-sided p-value, generated by testing whether CR or aging altered gene expression in either direction (up or down). Unless otherwise indicated, p-values cited in the text have been adjusted to account for multiple testing of hypotheses among the 21,327 genes evaluated in the analysis.

**Table 1:** Mouse tissue and cell types.

Tissue	Symbol	No. Experiments (CR)	No. Experiments (Aging)
Liver	lvr	10	8

Heart	hrt	7	13

Muscle	msl	4	4

White Adipose Tissue	wat	3	-

Hippocampus	hip	2	4

Cortex	ctx	2	4

Hypothalamus	hyp	2	-

Cerebellum	cbm	1	7

Kidney	kid	1	4

Lung	lng	1	3

Thymus	thm	1	2

Spinal Cord	spc	1	1

Striatum	str	1	1

Cochlea	coc	1	1

Gonads	gon	1	1

Mammary Gland	mmy	1	-

Colon	cln	1	-

Eye	eye	-	3

Whole Brain	wbr	-	3

Spleen	spl	-	2

Aorta	art	-	1

Gametes	gam	-	1

Myoblast Progenitor Cells	myo	-	1

Hematopoetic Stem Cells	hsc	-	1

Bone Marrow	bmw	-	1

Adrenals	adr	-	1

The table lists the tissue and cell types for which array data was available and incorporated into the analysis. Each tissue or cell type is referenced by a three-letter symbol listed in the second columns. The final two columns list, for each tissue or cell type, the number of independent array experiments that have investigated response to CR or aging. Additional File 1 provides details regarding each experiment incorporated into the analysis (e.g., % CR, duration of CR, ages examined, genetic background, type of array utilized).

**Figure 1:**
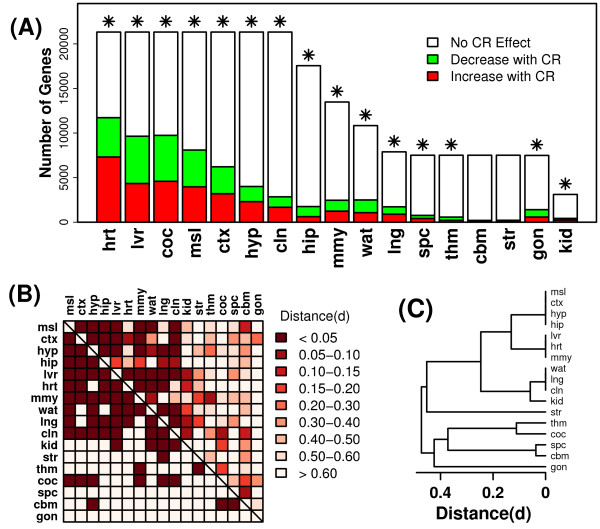
**Differential expression patterns associated with caloric restriction**. Differential expression signatures associated with CR in each tissue were generated based upon integration of results from one or several datasets (see Table 1). (A) The total height of each bar indicates the number of genes for which the effects of CR had been evaluated in at least one experiment (maximum of 21,327 genes). Colored sub-bars indicate the number of genes significantly up regulated (red) or down regulated (green) by CR within each tissue type (given a comparison-wise type I error rate of 0.05). Asterisk symbols (✴) indicate tissues for which a larger number of significant genes were identified than expected by chance. (B) The grid provides information on the pair-wise overlap between differential expression signatures associated with each tissue. The above-diagonal region is color-coded to reflect the distance between differential expression signatures (*d*). The value of *d *ranges from 0 to 1, where low values of *d *indicate that, between two signatures being compared, there exists an over-abundance of genes significantly regulated by CR in the same direction (see Methods). The region below the diagonal is coded in binary fashion, with dark squares indicating significant overlap between two differential expression signatures (P < 0.05) (see Methods). (C) An average linkage hierarchical cluster analysis was applied to differential expression signatures, where distance between signatures was based upon the measure *d *that is color-coded in the upper-diagonal region of the grid from part (B).

There was considerable heterogeneity among tissues in terms of the number of genes for which information was available, and with respect to the percentage of genes significantly influenced by CR. For heart, liver, cochlea, muscle, cortex, hypothalamus and colon, effects of CR had been evaluated, in one or more experiments, for nearly all 21,327 genes included in the analysis (Figure 1A). For other tissues, however, no data had been collected for most known genes and information was available for less than 4,000 genes (e.g., kidney) (Figure 1A). Based upon a nominal type I error rate of 0.05, it was expected that at least 5% of genes in any one tissue would be significantly altered by CR. This was the case for 15 of 17 tissues, in which CR altered more than 5% of the examined genes, with as many as 54% of genes altered in the case of heart (Figure 1A). For cerebellum and striatum, however, CR significantly altered only 2 - 3% of the examined genes, which is a smaller number of significant effects than expected on the basis of chance (Figure 1A). The effects of CR were characterized by constructing differential expression signatures associated with each tissue [24], and the overlap among signatures was examined. Differential expression signatures associated with CR in different tissues usually overlapped by less than 40%, but this overlap was often statistically significant (Figure 1B). A total of 136 pair-wise comparisons were made among 17 signatures, and in 54 of these cases (39.7%), the overlap was stronger than expected based on chance alone (P < 0.05). There was strong similarity between some segments of the central nervous system, such as spinal cord and cerebellum, as well as hypothalamus and hippocampus, but there was no overall tendency for signatures associated with the central nervous system to cluster together (Figure 1C). For instance, effects of CR in the striatum were not significantly similar to those in the cerebellum, spinal cord, hippocampus, cortex and hypothalamus (Figure 1B).

For liver, heart and muscle, effects of CR had been evaluated in more than four experiments, so detailed tissue-specific analyses were carried out (see Additional Files 2, 3 and 4). A surprising outcome of these analyses was that, although numerous genes emerged as significant in at least one experiment for each tissue, relatively few genes exhibited a consistent expression response to CR in multiple experiments. In liver, for instance, after collecting results across 10 experiments, only 10-15 genes were found to respond to CR in a consistent fashion (Additional File 2). These consistent responses provide liver-specific markers of CR response, and include up regulation of *Lpin1 *and *Lpin2 *(phosphatidate phosphatases involved in fat metabolism), which were both increased in each of 6 experiments for which data was available (P_*u *_< 4.12 × 10^-8^; P < 7.56 × 10^-8^). A second interesting aspect of the response to CR in liver was that several expression responses paralleled those resulting from growth hormone/insulin-like growth factor 1 (GH/IGF-1) mutations known to increase mouse lifespan [24]. For instance, in liver, CR decreased expression of leukemia inhibitor factor receptor (Lifr) (P_*d *_= 5.19 × 10^-4^; P = 9.39 × 10^-4^), insulin-like growth factor acid labile subunit (*Igfals*) (P_*d *_= 2.48 × 10^-4^; P = 4.20 × 10^-4^), kidney expressed gene 1 (*Keg1*) (P_*d *_= 8.64 × 10^-5^; P = 2.25 × 10^-4^), and the cytochrome P450 gene *Cyp2f2 *(P_*d *_= 2.47 × 10^-5^; P = 8.61 × 10^-5^). Additionally, CR increased expression of isocitrate dehydrogenase 2 (*Idh2*) (P_*u *_= 2.06 × 10^-5^; P = 9.74 × 10^-5^), insulin-like growth factor binding protein 1 (*Igfbp1*) (P_*u *_= 1.09 × 10^-3^; P = 6.90 × 10^-3^) and, very consistently, elevated expression of flavin-containing monooxygenase 3 (*Fmo3*) (P_*u *_= 2.81 × 10^-8^; P = 7.56 × 10^-8^) (see Additional File 2). Each of these expression responses was previously identified in association with multiple long-lived dwarf mutants, which have reduced levels of GH and/or IGF-1 in circulation [24].

Common responses to CR in multiple tissues included genes identified previously [12], but also new genes not uncovered in prior analyses. Given the large number of experiments considered, the analysis provided substantial statistical power, and overall, 29.7% of genes (6330/21327) were significantly up regulated by CR across tissues (P_*u *_< 0.05), while 27.6% of genes (5884/21327) were down regulated by CR across tissues (P_*d *_< 0.05). Given this large number of significant results, a more discerning criterion was to stratify genes according to the number of tissues in which a gene was significantly up or down regulated by CR. For instance, less than 1% of genes (118/21327) were up regulated by CR with respect to 8 or more tissue types, or down regulated by CR with respect to 8 or more tissues (61/21327).

**Figure 2:**
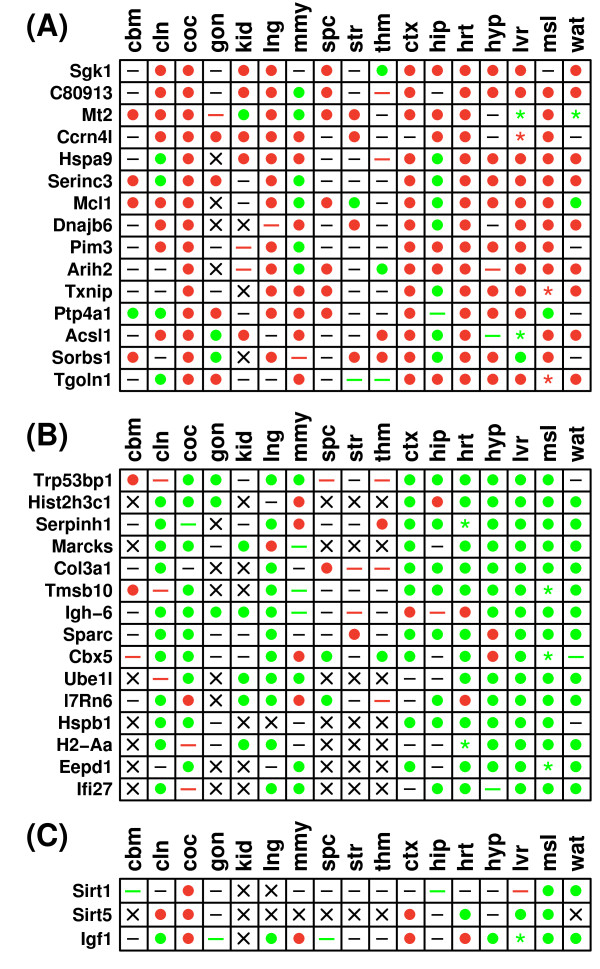
**Common gene expression responses to CR in the laboratory mouse**. Common gene expression responses to CR are ranked according to the number of tissue types in which genes were (A) significantly up regulated by CR or (B) significantly down regulated by CR. Additional File 5 provides a more comprehensive list of genes significantly regulated by CR across tissues, with genes ranked by p-values generated from Fisher's method. Genes shown in part (C) were not identified as common responses to CR, but have previously been associated with the mammalian CR response. Symbols shown within the grid reflect results of differential expression analyses for individual genes and tissues, given a comparison-wise type I error rate of 0.05 (i.e., no p-value adjustment for multiple testing of hypotheses). Filled circles indicate that a gene was significantly up regulated by CR (red circle) (P_*u *_< 0.05) or significantly down regulated by CR (green circle) (P_*d *_< 0.05) within a given tissue. The dash symbol is used to indicate cases in which the effects of CR are non-significant, although colored dash symbols indicate that expression is marginally increased by CR (red dash) (0.05 < P_*u *_< 0.10), or marginally decreased by CR (green dash) (0.05 < P_*d *_< 0.10). The asterisk symbol (✴) is used to designate instances where CR has significant effects, but there exists conflict among datasets. This may arise if at least two experiments exist, with at least one showing significant up regulation by CR (P_*u *_< 0.05), and at least one demonstrating significant down regulation by CR (P_*d *_< 0.05). Additionally, the asterisk is used when p-values, generated by combining results across experiments (Fisher's method), yield conflicting results in terms of whether a gene is up or down regulated (i.e., both P_*u *_< 0.05 and P_*d *_< 0.05). Asterisk symbols are colored red if there is stronger evidence that the gene is up regulated by CR (i.e., P_*u *_< 0.05 and P_*u *_< P_*d*_), and are colored green if there is stronger evidence that the gene is down regulated by CR (i.e., P_*d *_< 0.05 and P_*d *_< P_*u*_). The multiplication symbol (×) is used for cases in which no information was available regarding the effects of CR within a given tissue.

Figure 2 shows differential expression patterns associated with genes for which expression was most responsive to CR, in the same direction, across multiple tissue types. Genes shown in Figure 2A were up regulated significantly across all tissues (P_*u *_< 8.15 × 10^-7^; P < 3.38 × 10^-8^), and were each up regulated by CR significantly with respect to 9 or more individual tissues. Likewise, genes shown in Figure 2B were down regulated significantly across all tissues (P_*d *_< 4.93 × 10^-5^; P < 3.06 × 10^-6^), and were each down regulated by CR significantly with respect to 8 or more individual tissues. Aside from p-values generated using Fisher's method, the significance of genes shown in Figures 2A and 2B was confirmed by a second method, in which simulation was used to generate null distributions for a test statistic equal to the number of significant results across all tissue types (assuming random association of differential expression patterns among tissues) (P < 0.001 for each gene).

The most widespread effect of CR was up regulation of serum/glucocorticoid regulated kinase 1 (*Sgk1*) in 11 tissues, including colon, cochlea, kidney, lung, heart, liver and white adipose tissue, as well as four regions of the central nervous system (spinal cord, cortex, hippocampus and hypothalamus) (Figure 2A) (P_*u *_= 1.04 × 10^-10^; P = 1.31 × 10^-10^). Additionally, and consistent with previous analyses [12], CR increased expression of metallothionein 2 (*Mt2*) in 10 tissues (P_*u*_= 4.58 × 10^-10^; P = 1.74 × 10^-13^), and down regulated the collagen-specific molecular chaperone *Serpinh1 *in 8 tissues, including regions within the central nervous system (hypothalamus, hippocampus and striatum) (P_*d *_= 7.65 × 10^-9^; P = 1.98 × 10^-10^) (Figure 2B). A previous study has confirmed, by RT-PCR, that *Serpinh1 *is down regulated by CR in heart, liver and hypothalamus [26]. In Figure 2A and 2B, genes are ranked according to the total number of tissues in which CR increased or decreased expression significantly. Additional File 5 provides a comprehensive list using an alternative ranking scheme (based upon the Fisher p-value). This alternative approach highlighted some strong CR effects not shown in Figure 2, including increased expression of *Tsc22d3*, *Zbtb16*, *BC055107*, *Sult1a1*, *Per1*, *Timp3*, *S3-12*, *Pnpla2*, *Ppargc1a *and *Cp*, as well as decreased expression of *Ifitm3*, *Lasp1*, *Tia1*, *5830428H23Rik*, *Hsp90b1*, *Hist1h2bc*, *Cxcl12*, *Col6a2 *and *Dock4 *(see Additional File 5). Additional File 5 also provides analyses of gene ontology terms, KEGG pathways, associated microRNAs, and chromosomal locations. Overall, genes most strongly increased by CR across tissues were associated with fatty acid metabolism, the citrate cycle, PPAR signaling, oxidative phophorylation, amino acid degradation and metabolism, circadian rhythm, renal cell carcinoma, fatty acid elongation in mitochondria and the insulin signaling pathway (P < 0.01; over-represented KEGG pathways) (Additional File 5). Genes commonly down regulated by CR were associated with focal adhesion, antigen processing and presentation, ECM-receptor interaction, DNA replication, MAPK signaling, cell communication, VEGF signaling and natural killer cell mediated cytotoxicity (P < 0.01; over-represented KEGG pathways) (Additional File 5).

Genes listed in Figures 2A and 2B have not been well-studied in relation to the effect of CR in mouse. For instance, a series of PubMed searches was conducted (in September 2009), where each gene name was searched in conjunction with the phrase "caloric restriction". This search yielded one record associated with *Sgk1 *and *Hspb1*, respectively, but no PubMed records were retrieved for any other gene shown in Figures 2A and 2B. Figure 2C shows differential expression patterns of genes receiving greater research attention, such as sirtuin 1 (*Sirt1*), for which the above-mentioned search procedure retrieved 106 PubMed records. *Sirt1 *expression was significantly increased by CR in the cochlea, and significantly decreased in muscle and white adipose tissue, but overall, was not significantly regulated by CR across tissues (P_*u *_= 0.723; P_*d *_= 0.133; P = 0.068) (Figure 1C). Of the 7 sirtuin gene family members, CR had the strongest effect on the expression of sirtuin 5 (*Sirt5*), which was significantly increased by CR in colon, cochlea and cortex, and significantly decreased by CR in heart, liver and muscle (P_*u *_= 0.033; P_*d *_= 0.049; P = 3.81 × 10^-5^) (Figure 2C). Interestingly, the Igf1 transcript was significantly regulated by CR, but there was heterogeneity among tissues in the direction of this effect. In colon, lung, muscle, white adipose tissue and hypothalamus, Igf1 expression was decreased by CR, but the opposite trend was found in cochlea, heart, cortex and mammary gland (Figure 2C). Overall, across all tissues, there was significant evidence for decreased expression of *Igf1 *by CR (P_*u *_= 0.070; P_*d *_= 1.90 × 10^-4^; P = 1.71 × 10^-8^).

### Gene expression modules regulated by caloric restriction

The above analyses consider CR-responsiveness of individual genes, taken one at a time, but do not consider the organization among genes based upon their patterns of co-expression. For each gene, therefore, a "nearest-neighbor" co-expression module was generated by finding "neighbor" genes with correlated patterns of expression, based upon a reference set of 3,700 Affymetrix oligonucleotide arrays (see Methods). Nearest-neighbor modules of varying sizes were constructed for each gene included in the analysis (2, 3, 5, 10, 20 and 40 genes per module). Each module was then scored based upon an *M *statistic designed to quantify responsiveness of modules to CR, and the significance of observed *M *values was evaluated by simulations in which modules of a given size were formed at random (see Methods). A total of 3, 5, 22, 39 and 28 significant CR-responsive modules with 3, 5, 10, 20 and 40 genes, respectively, were identified. Table 2 lists the most significant CR-regulated co-expression modules of smaller size, and all significant modules of each size are shown in Additional File 6.

**Table 2:** CR-regulated co-expression modules

Modules	M (P-Value)	Minimal |*r*|
{*Fmo2*, *1200016E24Rik*}	10.7 (0.062)	0.703

{*Hist2h3c1*, *Hist2h3c2*}	10.5 (0.136)	0.817

{*Per1*, *Per2*}	10.1 (0.393)	0.597

{*Hist2h3c1*, *Hist2h3c2*, *Hist1h2bc*}	10.2 (0.004)	0.700

{*Per1*, *Per2*, *Zbtb16*}	10.0 (0.008)	0.596

{*Epm2aip1*, *Casc4*, *2810455D13Rik*}	9.73 (0.033)	0.568

{*Hist2h3c1*, *Hist2h3c2*, *Hist1h2bc*, *Hist2h2aa1*, *Hist1h1c*}	9.79 (< 0.001)	0.595

{*Per1*, *Per2*, *Zbtb16, Mm.34106, 1439537_at*}	9.29 (< 0.001)	0.564

{*Alkbh8*, *Zfp148*, *Rc3h2*, *Zdhhc21*, *Taok1*}	8.91 (0.006)	0.494

The table lists the most significant CR-regulated co-expression modules containing 2, 3, or 5 genes. The *M *statistic is an indicator of responsiveness to CR, with larger values suggesting stronger effects of CR on the expression of genes contained in a given module (see Methods). P-values associated with *M *statistics were generated by simulation, and reflect the likelihood of obtaining an equal or larger *M *value by randomly selecting genes to form a module of the same size (see Methods). The final column provides an indication of the degree to which module members are co-expressed with one another. This value is an element of the [0, 1] interval, and is obtained by calculating all possible pair-wise co-expression relationships among members of the module (all possible values of *r*), and identifying the weakest co-expression relationship between any two module members (i.e., the minimal value of |r|). Smaller minimal |r| values indicate more "diffuse" co-expression modules with weaker co-expression relationships, and larger minimal |r| values indicate "tighter" co-expression modules with stronger co-expression relationships. Additional File 6 provides a more detailed description of these and other co-expression modules that emerged as significantly associated with CR.

The approach identified 2-gene co-expression modules that were responsive to CR, some of which included functionally related gene pairs, such as {*Hist2h3c1*, *Hist2h3c2*} and {*Per1*, *Per2*} (*M *> 10.1) (Additional File 6). Of the 2-gene modules, only {*Fmo2, 1200016E24Rik*} was marginally significant (*M *= 10.7; P = 0.062), although others were often components of larger modules that were statistically significant (Additional File 6). For instance, significant 3-gene modules included {*Hist2h3c1*, *Hist2h3c2*, *Hist1h2bc*} and {*Per1*, *Per2*, *Zbtb16*} (*M *> 10.0; P < 0.008; Additional File 6). The composition of CR-responsive modules often revealed unexpected associations between genes. For instance, the most significant 10-gene module included six genes that encode histone cluster subunits, along with genes encoding an RNA binding protein (*Cstf2*) and a peroxisomal beta-oxidation system enzyme (*Acox1*) (*M *= 8.55; P < 0.001; see Additional File 6). The second-most significant 10-gene module included three period homologue genes (*Per1*, *Per2 *and *Per3*), a TSC22 domain family gene with anti-inflammatory effects (*Tsc22d3*) [27], and a gene known to negatively regulate tumorigenesis (*Zbtb16*) [28] (*M *= 8.40; P < 0.001; see Additional File 6). It was often the case that genes belonging to the same module exhibited similar responses to CR within the same tissues (Additional File 6).

**Figure 3:**
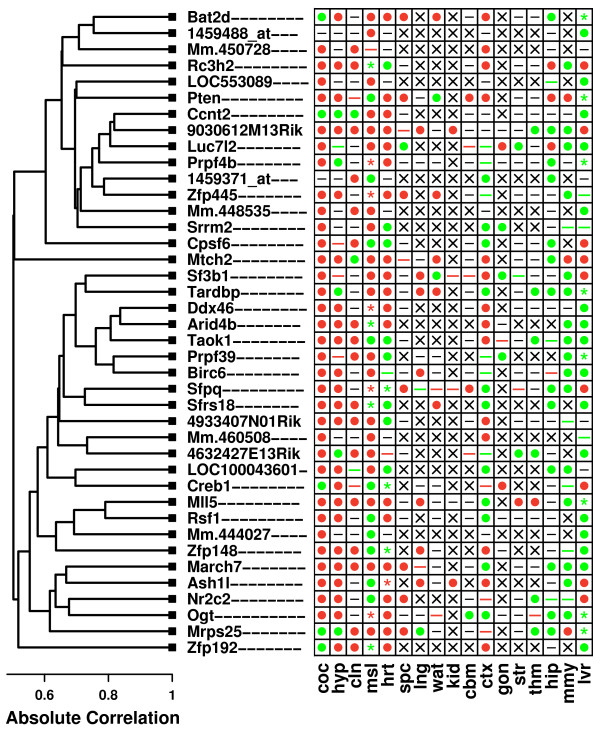
**Gene expression module regulated by CR**. The figure shows a 40-gene co-expression module that is regulated by CR across multiple tissue types. Symbols shown within the grid reflect results of differential expression analyses for individual genes and tissues, given a comparison-wise type I error rate of 0.05 (i.e., no p-value adjustment for multiple testing of hypotheses). These symbols have the same interpretation as those shown in Figure 2. The columns of the grid, corresponding to individual tissue types, have been ordered from those for which module genes are most strongly up regulated by CR, to those for which module genes are most strongly down regulated by CR. The dendrogram (left) reflects the co-expression patterns among the 40 genes belonging to this module, which are based upon the expression of genes across a set of 3,700 Affymetrix 430 2.0 oligonucleotide arrays (see Methods). The dendrogram was generated by average linkage hierarchical cluster analysis, with distance between genes based upon the absolute value of Pearson's correlation coefficient (i.e., distance = 1 - |*r*|). Other CR-regulated co-expression modules were identified in the analysis, and further detail on the most significant of these is provided in Additional File 6.

The most CR-responsive 40-gene module included genes associated with mRNA processing and transcriptional regulation (*M *= 7.61; P < 0.001; see Figure 3). The "center" of this module was the zinc finger protein 871 (*Zfp781*/*9030612M13Rik*), with the remaining 39 genes being nearest neighbors of *Zfp781*. Genes belonging to this module were highly responsive to CR, but the direction of response differed among tissue types (Figure 3). In cochlea, hypothalamus and colon, nearly all member genes were significantly up regulated by CR. However, in hippocampus, mammary gland and liver, genes in this module were usually down regulated by CR (Figure 3). Gene ontology analysis revealed that member genes were involved in transcriptional processes, particularly splicing, metabolism, processing and biosynthesis of RNA (P < 0.05). Along these lines, a disproportionately large number of module genes were associated with the nucleus, chromosome and spliceosome (P < 0.05).

### Genes regulated by aging in the laboratory mouse

**Figure 4:**
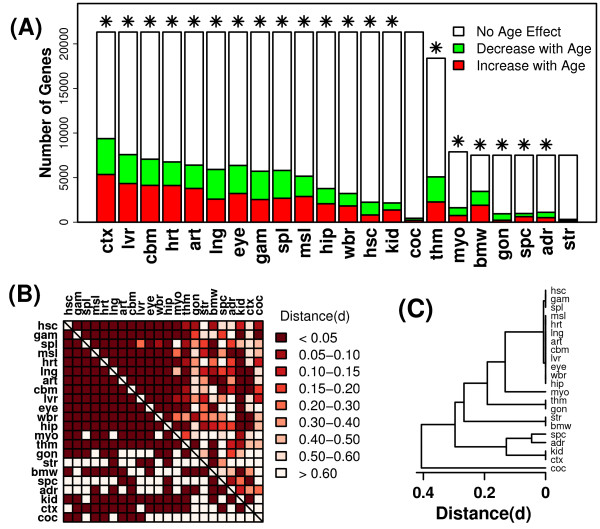
**Differential expression patterns associated with aging**. Differential expression signatures associated with aging in each tissue were generated based upon integration of results from one or several datasets (see Table 1). (A) The total height of each bar indicates the number of genes for which the effects of aging had been evaluated in at least one experiment (maximum of 21,327 genes). Colored sub-bars indicate the number of genes significantly up regulated (red) or down regulated (green) by aging within each tissue type (given a comparison-wise type I error rate of 0.05). Asterisk symbols (✴) indicate tissues for which a larger number of significant genes were identified than expected by chance. (B) The grid provides information on the pair-wise overlap between differential expression signatures associated with each tissue. The above-diagonal region is color-coded to reflect the distance between differential expression signatures (*d*). The value of *d *ranges from 0 to 1, where low values of *d *indicate that, between two signatures being compared, there exists an over-abundance of genes significantly regulated by aging in the same direction (see Methods). The region below the diagonal is coded in binary fashion, with dark squares indicating significant overlap between two differential expression signatures (P < 0.05) (see Methods). (C) An average linkage hierarchical cluster analysis was applied to differential expression signatures, where distance between signatures was based upon the measure *d *that is color-coded in the upper-diagonal region of the grid from part (B).

The effects of aging on gene expression had been evaluated in 67 experiments and a total of 22 tissue types (Table 1). On average, aging altered 22.9% of genes, with the strongest effects found in the cortex (44.0% of genes differentially expressed) (P < 0.05). Among those tissues with the strongest age effects (cortex, liver, cerebellum, heart, aorta), more genes increased with age than decreased with age (Figure 4). The weakest effects of aging were associated with cochlea and striatum, in which aging altered only 2-4% of genes, which is fewer than expected by chance at the nominal type I error rate of 0.05 (Figure 4A). For some tissues, aging had a larger impact on gene expression than observed in prior studies. In cerebellum, for example, at a type I error rate of 0.001, aging altered the expression of 2168 of 21327 genes (10.2%). This percentage is considerably higher than that observed in the AGEMAP project [29], in which aging altered the expression of only 17 of 8932 genes (0.19%) (P < 0.001). This apparent difference in effect size may reflect increased statistical power associated with a meta-analytic approach, or differences in the relative amount of noise influencing expression measurements generated using different array platforms. Differential expression signatures were constructed to characterize the effects of aging in each tissue, and comparisons of these signatures suggested that, relative to CR, effects of aging were more correspondent among different tissue types. For instance, among 231 pairwise comparisons between signatures, significant overlap was found in 158 cases (68.4% of comparisons) (Figure 4B), and a cluster analysis indicated that 12 of the 22 tissues formed a single group with similar patterns of differential expression (Figure 4C).

A side-by-side comparison of results from multiple experiments examining the same tissue highlighted robust tissue-specific markers of aging (Additional Files 7, 8 and 9). For instance, in each of seven experiments evaluating the effects of aging in liver, expression of immunoglobulin joining chain (*Igj*) increased (P_*u *_= 7.63 × 10^-9^; P = 1.04 × 10^-8^), while expression of kidney expressed gene 1 (*Keg1*) decreased (P_*d *_= 3.14 × 10^-7^; P = 3.73 × 10^-7^) (Additional File 7). The effects of aging in cardiac tissue had been examined in an especially large number of experiments (Additional File 8). Comparison of these experiments indicated that aging increased cardiac expression of phenylalanine hydroxylase (*Pah*) in all 12 experiments for which data was available (P_*u *_= 5.08 × 10^-16^; P = 4.87 × 10^-15^). Likewise, aging increased cardiac expression of complement component 4B (*C4b*) in 11 experiments for which data was available (P_*u*_= 5.08 × 10^-16^; P = 4.87 × 10^-15^). These results confirm and extend observations from a recent expression profiling study of age-associated gene expression patterns in cardiac tissue from multiple mouse strains, which identified both *Pah *and *C4b *as robust biomarkers of cardiac aging, and confirmed these age-related expression patterns using RT-PCR [11]. In muscle, an age-related increase of heat shock protein 90 (*Hsp90aa1*) expression was observed in four separate experiments (P_*u *_= 0.011; P = 0.019), as well as an age-associated decrease of *Col1a2 *expression (P_*d *_= 8.26 × 10^-3^; P = 4.73 × 10^-3^).

Genes with age-related expression patterns in multiple tissue types were identified using Fisher's method. This revealed that 7709 genes (36.1%) were significantly up regulated with age across all tissues (P_*u *_< 0.05), while 7872 genes (36.9%) were significantly down regulated with age across all tissues (P_*d *_< 0.05). There were strong functional differences between genes that increased with age in multiple tissues and genes that decreased with age in multiple tissues (Additional File 10). Genes up regulated with aging were frequently associated with immune response, and over represented gene ontology terms included antigen processing and presentation, lymphocyte mediated immunity, immunoglobulin mediated immune response, humoral immune response, lysosome organization/biogenesis, and regulation of adaptive immune response (P < 0.05; Gene Ontology biological process terms; Additional File 10). However, this was not generally true of genes decreased with age. Genes down regulated with aging in multiple tissues were more commonly associated with metabolic processes, chromosome organization/biogenesis, cell division and cycle, mRNA processing and splicing, and protein folding (P < 0.05) (Additional File 10).

**Figure 5:**
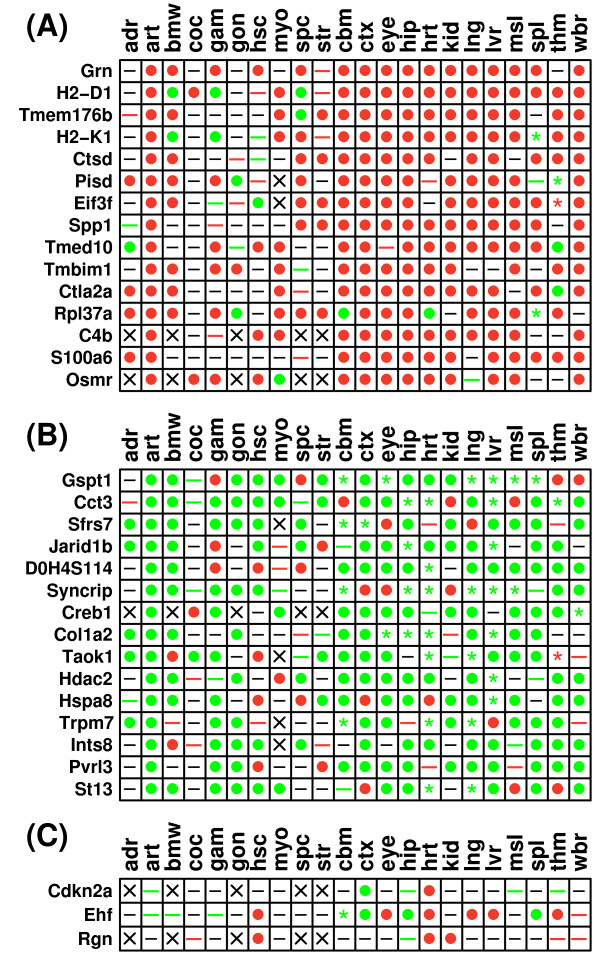
**Common gene expression responses to aging in the laboratory mouse**. Common gene expression responses to aging are ranked according to the number of tissue types in which genes were (A) significantly up regulated with age or (B) significantly down regulated with age. Genes shown in part (C) were not identified as common responses to aging, but have previously been associated with aging in mammals. Symbols shown within the grid have the same interpretation as those in Figure 2 (with p-values based upon a comparison-wise type I error rate of 0.05), except aging is the experimental factor (rather than CR). Additional File 10 provides a more comprehensive list of genes significantly regulated by aging across tissues, with genes ranked by p-values generated from Fisher's method.

Figure 5A displays differential expression patterns of genes significantly up regulated by age across all tissues (P_*u *_< 1.25 × 10^-9^; P = 7.38 × 10^-11^), with significant increases in 13 or more tissue types. Likewise, Figure 5B displays this information for genes significantly decreased by aging across tissues (P_*d *_< 1.47 × 10^-9^; P = 4.32 × 10^-11^), with significant effects in at least 11 tissues. The significance of all genes in Figures 5A and 5B was confirmed based upon a simulation analysis that treated the total number of significant results across all tissues as a test statistic (P < 0.001). Additional File 10 provides a more comprehensive list of genes with age-related expression in multiple tissues, with genes ranked according to Fisher p-values. Genes most strongly up regulated with age were often immune-related, including major histocompatability complex (MHC) genes (*H2-D1*, *H2-K1*, *H2-Q7*, *H2-L*, *H2-Aa*, *H2-T10*, *H2-M3*, *H2-T23*), and several genes encoding immunoglobulin chain components (e.g., *Igk-V1*, *Igh-6, Igj, Igh, Igl-V1*) (Figure 5A; Additional File 10). Expression of *H2-D1*, for instance, increased with age in 15 tissue types, including the central nervous system (whole brain, cortex, cerebellum, hippocampus), and several other major organ types (e.g., liver, heart, kidney) (P_*u *_= 1.25 × 10^-16^; P = 8.90 × 10^-18^) (Figure 5A). In both liver and thymus, a strong age-related increase in the expression of such immune-associated genes has previously been confirmed using RT-PCR [30,31]. Genes down regulated with age in multiple tissues were more diverse, and included heat shock proteins (*Hspa8*), along with genes involved in mRNA processing (*Sfrs7 *and *Syncrip*) (Figure 5B).

The age-related expression patterns of identified genes were more widespread than those of some genes previously associated mammalian aging and cellular senescence (Figure 5C). Based on previous investigations [32-34], for instance, it was expected that aging would increase expression of Cyclin-dependent kinase inhibitor 2A (*Cdkn2a/p16INK4A*) and Ets homologous factor (*Ehf*), and that aging would decrease expression of senescence marker protein 30 (*Smp30/Rgn*). For these genes, however, significant effects of aging were observed only in a few tissues, and often, the effect was inconsistent with prior expectations. For instance, *Cdkn2a/p16INK4A *is a regulator of the cell cycle, and it has been proposed that expression of *Cdkn2a *is a biomarker of aging in multiple mouse tissues [32]. But there was little indication that *Cdkn2a *expression increased with age in most tissues, and overall, the effect of age on *Cdkn2a *expression was nonsignificant among all tissue types (P_*u *_= 0.921; P_*d *_= 0.094; P = 0.178). The expression of *Cdkn2a *increased significantly only in cardiac tissue with age, and in cortex, *Cdkn2a *expression decreased with age (Figure 5C).

### Gene expression modules regulated by aging

Nearest-neighbor co-expression modules ranging in size from 2 to 40 genes were formed and the collective response of each module to aging across tissues was evaluated. The results demonstrate strong modularity of age-related expression patterns, with certain sets of co-expressed genes exhibiting correspondent differential expression patterns with age. A total of 5, 15, 43, 52 and 38 significant age-responsive modules with 3, 5, 10, 20 and 40 genes, respectively, were identified (Additional File 11). The most significant 3-gene module included two proteasome subunit genes (*Psmb8 *and *Psmb9*), along with the MHC antigen *H2-K1 *(M = 10.0; P < 0.001; see Table 3). The three genes contained in this module exhibited highly correspondent patterns of differential expression, with decreased expression occurring in spleen with age, and an age-related up regulation of expression across 13 tissues (Additional File 11). A similar pattern was present with respect to other 3-gene co-expression modules, such as {*Tyrobp*, *Mpeg1*, *Ctss*} and {*Sfi1*, *Pisd*, *4933439C20Rik*}, and with significant co-expression modules of larger size (Additional File 11). In each of these cases, genes belonging to the same module exhibited similar differential expression patterns in the same tissues, indicating that patterns of co-expression had considerable explanatory power in terms of age-related transcriptional effects.

**Table 3:** Age-regulated co-expression modules

Modules	M (P-Value)	Minimal |*r*|
{*Nckap1l*, *Evi2a*}	10.2 (0.101)	0.946

{*Plek*, *Ncf1*}	9.96 (0.208)	0.908

{*Sfi1*, *4933439C20Rik*}	9.93 (0.235)	0.780

{*Psmb8*, *Psmb9*, *H2-K1*}	10.0 (< 0.001)	0.859

{*Tyrobp*, *Mpeg1*, *Ctss*}	9.87 (< 0.001)	0.863

{*Sfi1*, *Pisd*, *4933439C20Rik*}	9.85 (< 0.001)	0.599

{*Tyrobp*, *Mpeg1*, *Fcgr3*, *Fcer1g*, *Plek*}	9.85 (< 0.001)	0.821

{*Psmb8*, *Psmb9*, *H2-M3*, *H2-K1*, *Tap2*}	9.29 (< 0.001)	0.813

{*Evi2a*, *Ly86*, *Pkib*, *March1*, *Lair1*}	9.16 (< 0.001)	0.713

The table lists the most significant age-regulated co-expression modules containing 2, 3, or 5 genes. Values in this table are interpreted in the same way as those listed in Table 2, except here the *M *statistic reflects responsiveness of a module to aging (rather than CR). Additional File 11 provides a more detailed description of these and other co-expression modules that emerged as significantly associated with aging.

**Figure 6:**
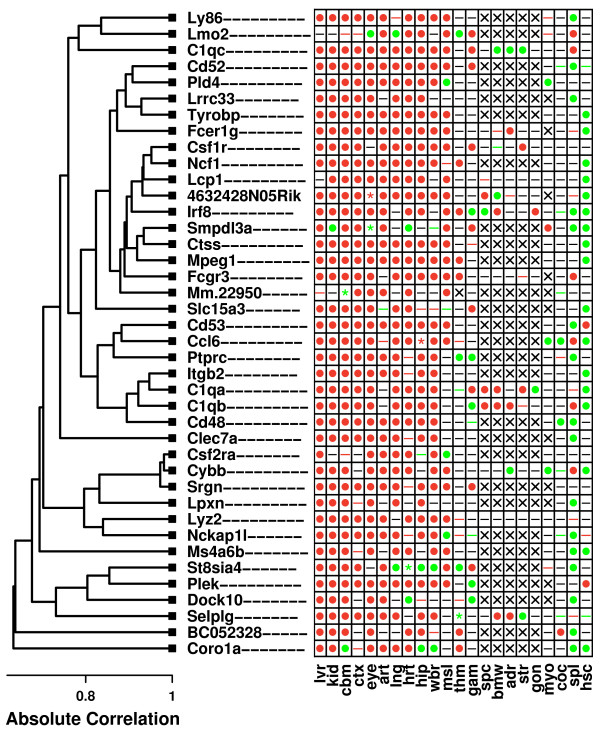
**Gene expression module regulated by aging**. The figure shows a 40-gene co-expression module that is regulated by aging across multiple tissue types. Symbols shown within the grid reflect results of differential expression analyses for individual genes and tissues, given a comparison-wise type I error rate of 0.05 (i.e., no p-value adjustment for multiple testing of hypotheses). These symbols have the same interpretation as those shown in Figure 2. The columns of the grid, corresponding to individual tissue types, have been ordered from those for which module genes are most strongly up regulated by age, to those for which module genes are most strongly down regulated by age. The dendrogram (left) reflects the co-expression patterns among the 40 genes belonging to this module, which are based upon the expression of genes across a set of 3,700 Affymetrix 430 2.0 oligonucleotide arrays (see Methods). The dendrogram was generated by average linkage hierarchical cluster analysis, with distance between genes based upon the absolute value of Pearson's correlation coefficient (i.e., distance = 1 - |*r*|). Other age-regulated co-expression modules were identified in the analysis, and further detail on the most of these is provided in Additional File 11.

The most significant 40-gene module was centered around cathepsin S (*Ctss*) (Figure 6). The rest of the module consisted of the 39 nearest neighbors of *Ctss*, which included genes encoding complement components (*C1qa*, *C1qb *and *C1qc*) and antigens (*Cd48*, *Cd52 *and *Cd53*). Strikingly, nearly every gene in this module exhibited an age-related expression increase in liver, kidney, cerebellum, cortex, eye, aorta, lung, heart, hippocampus and whole brain (Figure 6). Among genes belonging to the module, a gene ontology analysis revealed numerous over-represented biological processes, including activation of immune response, positive regulation of B cell mediated immunity, phagocytosis, inflammatory response and neutrophil chemotaxis (P < 0.01). Half of module genes were associated with membrane (20 and 40 genes; P < 0.001), particularly the plasma membrane (8 of 40 genes; P < 0.01). The second-most significant 40-gene module (see Additional File 11) was not associated with inflammation or immune response, but interestingly, included genes with roles in mRNA processing, RNA transport and RNA localization. Most of the genes in this module were associated with the nucleus (24 of 40 genes) and several were among the most age-responsive genes identified in the analysis, such as *Gspt1*, *Ints8*, *Syncrip *and *Creb1 *(Figure 5B). In contrast to the module shown in Figure 6, most member genes were predominantly decreased with aging, particularly in aorta, gametes and cardiac tissue (Additional File 11).

### Relationship between caloric restriction and aging

**Figure 7:**
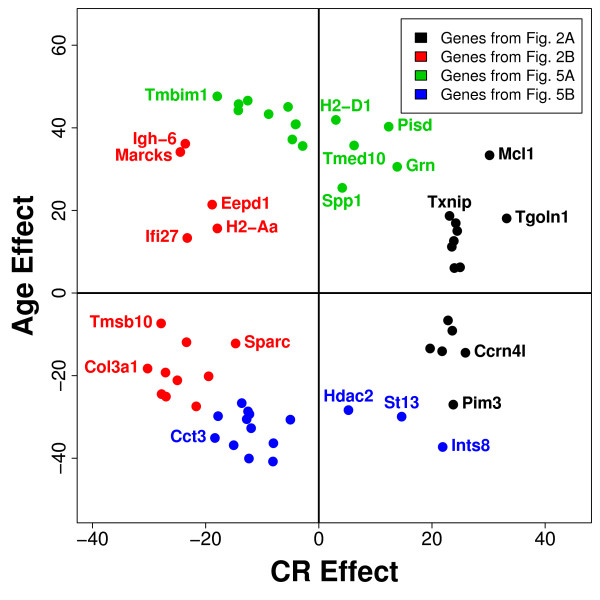
**Relationship between caloric restriction and aging**. The association between CR and aging was evaluated for genes most strongly increased CR across tissues (Figure 2A), genes most strongly decreased by CR (Figure 2B), genes most strongly increased by aging (Figure 5A), and genes most strongly decreased by aging (Figure 5B). The "CR effect" is positive for genes up regulated by CR across tissues and negative for genes down regulated by CR across tissues. Likewise, the "age effect" is positive for genes up regulated with age across tissues and negative for genes down regulated with age across tissues. The magnitude of the CR effect and age effect reflects the degree of statistical significance. In particular, the CR effect is equal to I * -log[min(*P*_*u*_, *P*_*d*_)], where *P*_*u *_and *P*_*d *_are p-values generated by one-sided hypothesis tests of up and down regulation by CR across tissues, while *I *is equal to 1 if *P*_*u *_<*P*_*d *_and is equal to -1 otherwise. Similarly, the age effect is equal to I * -log[min(*P*_*u*_, *P*_*d*_)], where P_*u *_and P_*d *_are p-values generated by one-sided hypothesis tests of up and down regulation by aging, while *I *is equal to 1 if P_*u *_< P_*d *_and is equal to -1 otherwise.

CR is thought to inhibit the progression of aging and to oppose age-related gene expression patterns. It was therefore expected that a negative association would exist between transcriptional effects of CR and aging, and several approaches were taken to evaluate whether such an association was present. First, genes most strongly regulated by CR or aging across multiple tissue types were considered (see Figures 2 and 5). There was no general tendency for genes increased by CR across tissues to be decreased by aging, and likewise, genes decreased by CR across tissues were not disproportionately increased by aging (Figure 7). Individual genes can be identified that either support or contradict the expected pattern. For instance, CR decreased expression of *Igh-6 *and *Marcks*, and this effect does indeed oppose increased expression of these genes with age across tissues. On the other hand, CR decreased expression of *Col3a1*, and this effect would seem to mimic, rather than oppose, age-related decreases in *Col3a1 *expression (Figure 7).

**Figure 8:**
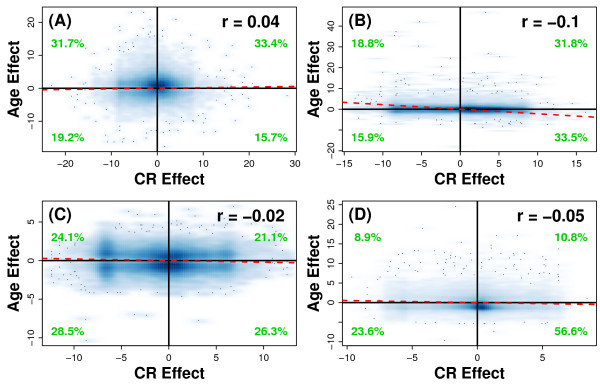
**Relationship between caloric restriction and aging in liver, heart, muscle and the central nervous system**. The association between CR and aging was evaluated for the (A) liver, (B) heart, (C) muscle and (D) central nervous system (hippocampus + cortex). The CR effect is positive for genes up regulated by CR and negative for genes down regulated by CR (see Figure 7 legend). Likewise, the age effect is positive for genes up regulated with age and negative for genes down regulated with age (see Figure 7 legend). The abundance of genes in relation to the CR and age effect is reflected by the color intensity, with deep blue colors corresponding to regions with the largest number of genes. The dashed red line is based upon a least-squares regression fit that quantifies the overall relationship between the CR and aging effects. In each panel, the estimated Pearson correlation is shown in the upper-right, and the percentage values (green font) indicate the fraction of genes that belong to each quadrant. The effects of CR and aging were computed in each organ system based upon p-values generated by combining results from at least 3 independent experiments. In liver, CR and aging effects are based upon 9 and 7 experiments, respectively. In heart, CR and aging effects are based upon 5 and 10 experiments, respectively. For muscle and central nervous system, CR and aging effects are each based upon 3 -6 experiments. For each organ, distinct sets of data were used to estimate the CR and aging effects, such that CR and aging effects are *a priori *independent.

The association between CR and aging was next examined at the global scale, among all genes, and also with respect to each of the four most well-studied tissue types (liver, heart, muscle and central nervous system) (Figure 8). In liver, there was a slight, positive association between the effects of CR and aging (*r *= 0.04) (Figure 8A). This association was significant (P < 6.72 × 10^-12^), although given the large number of genes involved in the comparison, this significance test was not too informative. In the heart, muscle and central nervous system, the expected negative association between CR and aging did emerge, albeit weakly, with the estimated correlation coefficient less than or equal to -0.10 in each case. The strongest association was found in heart (Figure 8B), in which age-related expression patterns were weakly opposed by CR (*r *= -0.096; P = 2.20 × 10^-16^). In muscle and central nervous system (Figures 8C and 8D), the association between CR and aging was again weak (*r *< -0.048), and non-significant in the case of muscle (P = 0.054), despite the large number of genes upon which the association was based. With respect to central nervous system, a large fraction of genes (56.6%) were both increased by CR and decreased with age (i.e., within the lower-right quadrant of Figure 8D), although very few genes (8.9%) were decreased by CR and increased with age (i.e., within the upper-left quadrant of Figure 8D).

**Figure 9:**
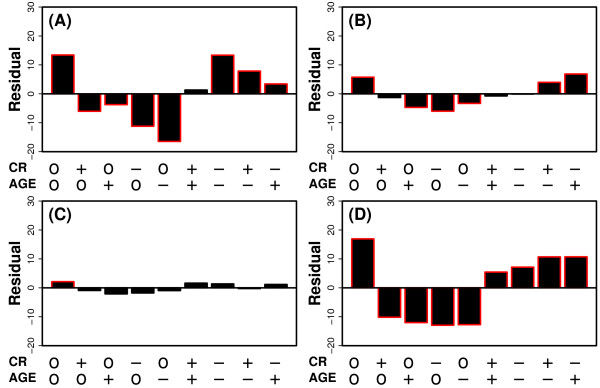
**Relationship between caloric restriction and aging in liver, heart, muscle and the central nervous system. Association between differential expression signatures**. The association between CR and aging was evaluated for the (A) liver, (B) heart, (C) muscle and (D) central nervous system (hippocampus + cortex). For each tissue, a differential expression signature was constructed corresponding to the effects of CR, and another signature was constructed corresponding to the effects of aging. Both signatures were based upon 3 or more experiments (see Figure 8 legend). CR and aging signatures were cross-tabulated, to place component genes into 9 separate categories, depending upon whether genes were increased by CR and/or aging (+), decreased by CR and/or aging (-), or whether CR and/or aging had no significant effect on gene expression (o). For each category, bars represent the adjusted Pearson residual (see Methods) (94). The absolute value of this quantity reflects the degree to which gene counts differ from what is expected based upon a random association between the CR and aging differential expression signatures. Positive residuals indicate that more genes belong to that category than expected by chance, given a random association between the effects of CR and aging. Negative residuals indicate that fewer genes belong to that category than expected by chance, given a random association between the effects of CR and aging. The adjusted Pearson residuals follow a standard normal distribution, with residuals larger than 3 in absolute value suggestive of significant, non-random associations between the effects of CR and aging [94]. The bars outlined in red indicate a significantly large or small residual (P < 0.05).

**Figure 10:**
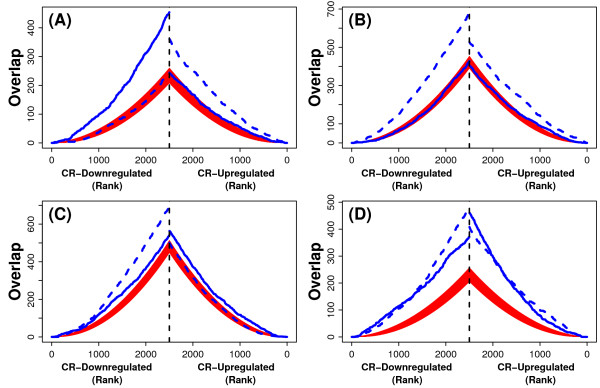
**Relationship between caloric restriction and aging in liver, heart, muscle and the central nervous system. Association between ordered gene lists**. The association between the effects of CR and aging were evaluated in (A) liver, (B) heart, (C) muscle and (D) central nervous system (hippocampus + cortex). The association was evaluated following the approach of Lottaz et al. (2006) [25]. For each tissue type, four ordered lists of genes were generated (abbreviated as: CR_u_, CR_D_, AGE_u_, AGE_D_). The CR_u _list contained the 2,500 genes most strongly up regulated by CR, ordered according to the significance of this effect (i.e., most-to-least significant). Likewise, the CR_D _list contained the 2,500 genes most strongly down regulated by CR, ordered according to the significance of this effect (i.e., most-to-least significant). Similarly, the AGE_U _list contained the 2,500 genes most strongly up regulated with age, and the AGE_D _list contained the 2,500 genes most strongly down regulated with age (ordered from most-to-least significant). In each panel, blue lines indicate the degree of overlap between CR and AGE gene lists, with respect to gene ranks between 1 and 2,500. On the right side of each panel, solid blue lines represent the overlap between CR_U _and AGE_U_, while dotted blue lines represent the overlap between CR_U_and AGE_D_. On the left side of each panel, solid blue lines represent the overlap between CR_D _and AGE_D_, while dotted blue lines represent the overlap between CR_D _and AGE_U_. Red bands in each plot correspond to 95% confidence intervals for the degree of overlap expected by chance (based on the hypergeometric distribution) (25). Given an inverse association between CR and aging, it is expected that dotted blue lines would fall above the region shown in red, whereas the solid blue lines would fall within (or below) the region shown in red.

The global effects of CR and aging were then compared by evaluating the association between differential expression signatures. In this analysis, it was expected that genes significantly increased by CR would tend to be decreased significantly with age, and conversely, that genes significantly decreased by CR would increase significantly with age. Associations between differential expression patterns were found (Figure 9), and were moderately strong in liver (*χ*^2 ^= 295.8; P < 2.2 × 10^-16^), weaker in heart (*χ*^2 ^= 64.22; P = 2.94 × 10^-13^), weakest in muscle (*χ*^2 ^= 6.56; P = 0.161), and strongest in the central nervous system (*χ*^2 ^= 330; P < 2.2 × 10^-16^). The pattern of association, however, varied among tissues, and was often not consistent with the expectation. In liver and central nervous system, CR did oppose age-related expression patterns, but these trends were complicated by the fact that, in each tissue type, there was a significant tendency for CR to actually reinforce the effects of aging, which is in complete contrast to the expectation stated above (Figures 9A and 9D). In muscle, the relationship between CR and age-related differential expression patterns was effectively random, with little or no evidence of any association (Figure 9C). The strongest evidence for the expected CR-aging relationship was again found with respect to heart. In cardiac tissue, there was a significant abundance of genes increased by CR and decreased by age, as well as an abundance of genes decreased by CR and increased by age (Figure 9B). These conclusions were reinforced by results from an alternative analytical method, based upon correspondence between ranked gene lists [25], which also showed conflicting associations between CR and aging in liver, muscle and central nervous system (Figures 10ACD), with a stronger and more clear-cut relationship in heart (Figure 10B).

**Figure 11:**
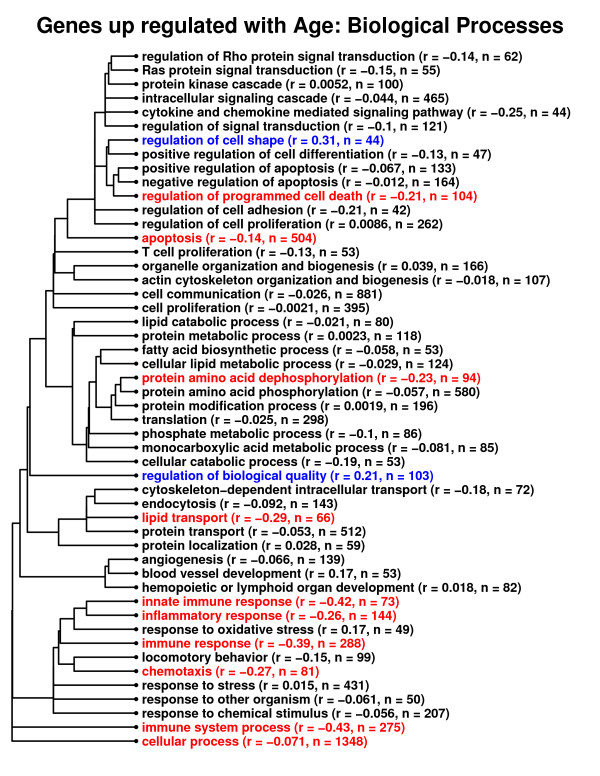
**Relationship between caloric restriction and aging. Gene ontology biological process terms associated with genes up regulated by aging across all mouse tissues**. A total of 2554 genes were identified as significantly increased by aging across the 22 mouse tissue types, based upon stringent significance criteria (P_*u *_< 0.001 and up regulated by age in at least five mouse tissues). Among these genes, significantly over-represented gene ontology (GO) biological process terms were identified (P < 0.05; hypergeometric test). These significant GO terms are shown in the chart and have been clustered according to the number of ancestor terms shared between any two GO biological processes. For each term, a subset of *n *associated genes was identified, and among these *n *genes, the correlation (*r*) between the transcriptional effects of CR and aging was evaluated. Transcriptional effects of CR and aging across tissues were defined as explained in Figure 7. For terms displayed in red font, the transcriptional association between CR and aging was negative (*r *< 0) and significant (P < 0.05). For terms displayed in blue font, the transcriptional association between CR and aging effects was positive (*r *> 0) and significant (P < 0.05). For all other terms (black font), there was no significant relationship between transcriptional effects of CR and aging (P > 0.05).

**Figure 12:**
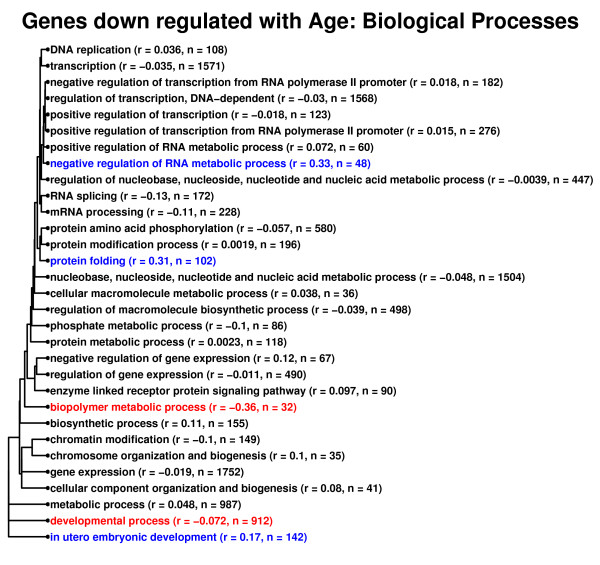
**Relationship between caloric restriction and aging. Gene ontology biological process terms associated with genes down regulated by aging across all mouse tissues**. A total of 663 genes were identified as significantly down regulated by aging across the 22 mouse tissue types, based upon stringent significance criteria (P_*d *_< 0.01 and down regulated by age in at least five mouse tissues). Among these genes, significantly over-represented gene ontology biological process terms were identified (P < 0.05; hypergeometric test), and these terms are displayed in the figure. The same analysis described in Figure 11 was applied to each term. Colors indicate whether, among genes associated with each term, there was a significant negative relationship between transcriptional effects of CR and aging (red font; *r *< 0; P < 0.05), a significant positive relationship (blue font; *r *> 0; P < 0.05), or no significant relationship (black font; P > 0.05).

The above results suggest that, among all genes, the transcriptional association between effects of CR and aging is limited, with a stronger relationship in heart relative to other tissue types. One possibility is that the transcriptional association between CR and aging is stronger among genes associated with some biological processes, and weaker among certain genes associated with other processes. The CR-aging association was thus examined with respect to gene ontology biological process terms that were associated with genes increased or decreased by aging across all mouse tissues (Figures 11 and 12). This analysis revealed definite differences among gene categorizations with respect to the transcriptional relationship between CR and aging. In particular, among genes involved in immune response, innate immune response and immune system processes, the effects of CR were negatively associated with those of aging (Figure 11; *r *≤ -0.39). These results are in agreement with the RT-PCR investigation of Park et al. (2009) [11], which found that, in heart and cerebellum, CR opposed the effects of aging for certain genes associated with immunity and defense response (e.g., *C4*, *C1qa*, *Lzp-s*, *Cxcl14*). Figures 11 and 12 also show that CR opposes age-associated expression patterns among other classes of genes as well, such as groups of genes with roles in apoptosis, protein amino acid dephosphorylation, lipid transport, chemotaxis, biopolymer metabolic process and inflammation. At the same time, for some processes, the opposite pattern of association was found (e.g., negative regulation of RNA metabolic process; Figure 12), and for many gene groups, the association between CR and aging was non-significant and weakly inverse, in agreement with analyses based upon global transcriptional patterns among all genes.

## Discussion

Caloric restriction (CR) and aging have been found to influence the expression of numerous genes in a variety of mouse tissues, but there has been considerable heterogeneity in results generated from different laboratories. This study has identified genes and gene expression modules that, based upon currently available data, are most robustly associated with CR and/or aging in the laboratory mouse. These transcriptional effects provide a well-supported basis for the generation of new research hypotheses, which can serve as starting points for experimental studies into mechanisms of CR and aging. The identified expression patterns influence a wide range of tissue and cell types, and thus represent the most systemic transcriptional effects of CR and aging, which may be associated with pathways required for positive effects of CR on survivorship, or linked to mechanisms that drive increased disease and mortality risk with age. These transcriptional effects were often modular, with groups of co-expressed genes responding to CR or aging in coordination across multiple tissues. For instance, the analysis uncovered a CR-responsive group of co-expressed genes encoding histone clusters (*Hist2h3c1*, *Hist2h3c2*, *Hist1h2bc*, *Hist2h2aa1*and *Hist1h1c*), as well as a large module that contains genes involved in mRNA processing (e.g., *Sfrs18*, *Sfpq*, *Prpf4b*, *Prpf39*). Age-related expression patterns affecting multiple tissues were, to a considerable degree, explained by the modularity of the transcriptome. This was best demonstrated by a large module containing 40 inflammation and immune response genes (e.g., *C1qa*, *C1qb*, *C1qc*, *Cd48*, *Cd52 *and *Cd53*), nearly all of which increased expression with age in ten mouse tissues. It was expected that effects of CR and aging would be associated, with CR acting of oppose age-related changes in gene expression. This was the case among genes associated with certain biological processes (e.g., immune response and inflammation), but at the genome-wide level among all genes, the transcriptional association between CR and aging was limited.

Common responses to CR represent systemic effects of low-calorie diets that influence multiple organ systems [12]. A previous study found that CR increased expression of genes encoding the anti-oxidant metallothioneins (*Mt1 *and *Mt2*), the NF-*κ*B inhibitor I*κ*B*α *(*Nfkbia*), as well as the tumor suppressor P21 (*Cdkn1a*) [12]. The present investigation has revealed that these effects are even more widespread than previously thought, each occurring in at least seven mouse tissues (Additional File 5). Additionally, widespread transcriptional effects of CR were identified that could influence the predisposition of mice to develop cancers, which is thought to be a main factor underlying pro-longevity effects of the CR diet [35,36]. These expression patterns were associated with a broad range of tissue types, and include increased expression of thioredoxin interacting protein (*Txnip*/*Vdup1*), zinc finger and BTB domain containing 16 (*Zbtb16*/*PLZF*) and tissue inhibitor of metalloproteinase 3 (*Timp3*) (see Figure 2 and Additional File 5). Furthermore, CR reduced expression of thymosin (*Tmsb10*), secreted acidic cysteine rich glycoprotein (*Sparc*) and heat shock protein 1 (*Hspb1*) (see Figure 2), and each of these effects could also inhibit transformation or cellular proliferation and migration [37-40]. Increased expression of *Txnip *and *Zbtb16 *expression by CR could reflect an anti-proliferative effect of low calorie diets on a common pathway, since *Txnip *encodes a protein known to induce expression of *Zbtb16 *[41], and both genes encode proteins that can promote apoptosis or limit the invasive capacity of tumor cells [28,41,42]. Widespread effects of CR on *Txnip *expression are especially noteworthy, since *Txnip*-deficient mice exhibit several abnormal responses to fasting [43], and because Txnip can regulate activation of the PTEN tumor suppressor protein (also elevated by CR; see Figure 3) [44]. Like *Txnip *and *Zbtb16*, *Timp3 *has tumor-suppressive properties [45], and its expression limits tumor invasiveness and has been correlated with survivorship in cancer patients [46,47]. Additionally, expression of *Timp3 *negatively controls tumor necrosis factor alpha (TNF-*α*) levels, leading to reduced NF-*κ*B activation and inflammation [48,49]. Taken together, these results begin to outline a generalized response of the mammalian cell to CR. This response involves increased expression of genes with tumor-suppressive properties (e.g., *Txnip*, *Zbtb16*, *Timp3 *and *Cdkn1a*), decreased expression of genes associated with cellular transformation and proliferation (e.g., *Tmsb10, Sparc *and *Hspb1*), as well as the induction of genes with anti-inflammatory (e.g., *Nfkbia*, *Timp3*) or anti-oxidative effects (e.g., *Mt1*, *Mt2*).

The beneficial effects of CR on survivorship may be related to loss of insulin-like growth factor 1 (IGF-1) signaling [50]. This type of mechanism has been shown to increase lifespan and decrease cancer incidence in mutant dwarf mice, in which serum IGF-1 levels are greatly diminished [24], or in which the localized bioavailability of IGF-1 within tissues is reduced [51]. Similarly, it was recently shown that mouse lifespan can be increased by late-life administration of rapamycin, which inhibits mTOR, an element downstream of the IGF-1/PI3-K pathway [52]. A major question in aging biology, therefore, is whether pro-survival effects of the CR diet are dependent upon inhibition of IGF-1 signals in a manner that, to some degree, parallels mechanisms underlying increased lifespan in long-lived dwarf mutants and/or mice treated with rapamycin. Along these lines, it is interesting to note that the most widespread effect of CR to emerge from this analysis was increased expression of serum/glucocorticoid regulated kinase 1 (*Sgk1*), which occurred in 11 of the 17 mouse tissues examined (Figure 2A). The Sgk1 kinase is activated by IGF-1/phosphatidylinositol-3 kinase (PI3-K) signals, and data from several microarray analyses indicate that expression of *Sgk1 *is up regulated by mutations and treatments that inhibit the IGF-1/PI3-K pathway [53-56]. Another widespread effect of CR was increased expression of phosphatase and tensin homolog (*Pten*) (Figure 4), which is a tumor suppressor that inhibits IGF-1 signals by preventing activation of the PI3K/Akt signaling pathway [57]. These effects of CR on *Sgk1 *and *Pten *expression may reflect attenuation of IGF-1 signals across a number of tissue types, possibly in a manner that, to some degree, mimics the influence of dwarf longevity mutations or rapamycin treatment. Such expression patterns thus raise new questions for further work, which can contribute to a developing model of interactions between CR and the IGF-1 pathway [50].

The metabolism of mRNA is a fundamental process that underlies virtually all cellular activities, and perturbation of mRNA processing can have important effects on gene regulation, homeostasis and cell cycle control. This study has found that, in multiple tissues, CR influences the expression of genes involved in the splicing and processing of messenger RNA (Figure 3). These genes are co-expressed and organized into a single large module that includes splicing factors (*Sf3b1*, *Sfpq*, *Sfrs18*), pre-mRNA processing factors (*Prpf4b*, *Prpf39*), pre-mRNA cleavage factor I (*Cpsf6*), an RNA/DNA binding protein associated with splicing regulation and mRNA stability (*Tardbp*) [58], a co-activator of pre-mRNA splicing (*Srrm2*/*SRm300*) [59], and a yeast homolog of Luc7p that associates with the spliceosomal subunit U1 snRNP (*Luc7l2*) [60]. The possibility that nutrient signals, and in particular CR, can have important effects on mRNA processing is consistent with previous investigations. For example, glucose-6-phosphate dehydrogenase (G6PD) protein levels and activity are positively regulated by intake of carbohydrates, but without any alteration in transcription rate of the associated *G6pdx *gene [61]. Carbohydrate intake instead appears to increase the efficiency of splicing events within the nucleus, leading to increased abundance of mature mRNA transcript and G6PD protein [61]. Nutritional regulation of mRNA processing has also been reported for thyroid hormone responsive SPOT14 homolog (*Thrsp*) [62], as well as insulin-like growth factor I (*Igf1*) [63]. These examples suggest that nutritional effects on mRNA processing are not altogether unusual, and that some widespread effects of CR could result from an influence on the multi-step process by which mRNAs proceed from transcription to translation. This effect of CR may be consequential for disease processes, since proteins that regulate splicing events can contribute to cell growth and the proliferation of cancer cells [64-66]. It is interesting to note that expression of genes associated with mRNA processing, RNA transport or RNA localization are decreased by aging in many tissues (e.g., *Syncrip *and *Sfrs7*; Figure 5; Additional File 11). This consistent effect of aging contrasted with that of CR, which variably influenced the expression of functionally similar genes, promoting increased expression in some tissues (e.g., cochlea and hypothalamus) but decreased expression in others (e.g., mammary gland and liver) (see Figure 3).

There is much evidence to suggest that certain mechanisms of aging are conserved across species, with similar pathways regulating life span and the progression of aging in worms, flies and mammals [67]. This notion suggests that there may exist common features of aging that are evolutionarily conserved, which are reflected by age-related patterns of gene expression that are shared among mammalian cell types within diverse tissues. The present study has identified robust age-related expression patterns that appear to be characteristic of cells within many or most mammalian tissues. It is not expected that all such age-related expression patterns will be associated with deleterious pathologies, since some modifications of gene expression with age are likely to reflect favorable compensatory mechanisms. Nevertheless, some age-regulated genes identified in this analysis are known to modulate inflammatory processes and contribute to cellular proliferation. For instance, two widespread effects of aging were up regulation of granulin (*Grn*) and secreted phosphoprotein 1 (*Spp1*) (see Figure 5). Increased expression of *Grn *was in fact the most widespread effect of aging identified, occurring in nearly all of the tissues examined (16 of 22). *Grn *encodes the granulin protein (also referred to as progranulin, granulin-epithelin precursor, proepithelin or acrogranin), which can inhibit TNF-induced inflammatory processes and promote vascularization necessary for wound and tissue repair [68], but can also give rise to cleavage products that stimulate cytokine secretion leading to the exacerbation of inflammatory processes [69]. Additionally, granulin has been identified as potent growth factor that stimulates aggressive cell proliferation, and in at least some cell lines, this mitogenic effect depends upon the expression level of *Grn *[70]. Increased expression of *Spp1 *with age was identified in 14 of 22 mouse tissue types, with the strongest increase observed in the cerebellum. *Spp1 *encodes osteopontin, a protein involved in localized recruitment of macrophages and in the production of cytokines within certain immune cells, which can contribute to a state of chronic inflammation [71]. Osteopontin is also a regulator of tumor progression and its abundance within tumors has emerged as an impressive biomarker for numerous types of cancer [72]. Expression of osteopontin within tissues and in circulation has also been associated with a wide range of other conditions, including obesity, diabetes and cardiovascular disease [73-75]. An important component of future work will be RT-PCR investigation of *Grn *and *Spp1 *expression patterns with age, both across mouse tissues and among different mouse strains [11].

A widespread effect of aging was increased expression of inflammation and immune response genes, which involved class I MHC genes (e.g., *H2-D1*, *H2-K1*), immunoglobulin chain components (e.g., *Igk-V1*, *Igh-6*), and a large module of 40 co-expressed genes known to participate in inflammation and immune processes (see Figures 5 and 6; Additional File 10). These observations are consistent with those from previous investigations that have evaluated genome-wide expression patterns in aging mice [30,31], and thus provide further evidence that heightened expression of inflammation and immune response genes is a systemic feature of aging that influences a broad range of organ systems. Indeed, exceptions to this pattern may represent the most interesting cases. For example, only 4 of 22 tissues *did not *show an age-related increase in the expression of either *H2-D1 *or *H2-K1 *(adrenals, bone marrow, gametes and gonads). It is likely that, at least in part, increased expression of inflammation and immune response genes reflects age-related increases in immune cell populations within aging tissues. In liver, a recent histological analysis demonstrated that several immune cell types (i.e., macrophages, T and B cells, neutrophils and natural killer cells) infiltrate perivascular regions with aging to establish proliferating cell clusters [31]. This type of mechanism is likely to explain, for example, the increased expression of *Igk-V1 *and *Igh-6 *described in this analysis, which could be driven by B-cell lymphocyte infiltration of aging tissues. Such immune cell infiltration could be driven by chronic inflammation, possibly in a manner that parallels tertiary lymphoid neogenesis [76], and it is further possible that such inflammation is exacerbated by an age-associated accumulation of fat cells [31]. An important direction for future studies will be to evaluate mechanisms by which immune cells, such as lymphocytes, are drawn to aging tissues and whether such mechanisms are comparable in various organ systems. An inspection of current data, for example, revealed that the B-cell attractants *Ccl19 *and *Cxcl13 *increase with age in some tissues [31,77]. In particular, expression of *Ccl19 *increased with age in 5 tissues (cerebellum, eye, heart, kidney and liver), while an age-related increase in *Cxcl13 *expression was observed in 8 tissues (aorta, cerebellum, heart, kidney, lung, liver, muscle, whole brain).

The identification of robust age-related gene expression patterns may lead to the development of useful "biomarkers", which could provide tools for tracking the progression of the aging in experimental studies [17]. Development of such indicators is requisite for making objective statements regarding the effects of interventions, or of particular mutations, on the deceleration (or acceleration) of aging. The age-related expression patterns highlighted in this analysis represent systemic or near-systemic effects, which have repeatedly emerged in multiple experimental settings. Such transcriptional effects may thus be suitable for further evaluation as aging biomarkers, or as components of a quantitative model whose output serves as an aging biomarker [78]. The current data, for example, suggest that increased expression of immune response genes (e.g., *H2-D1*, *H2-K1*, *Igk-V1 *or *Igh-6*) is a reliable feature of normal aging, perhaps representing a widespread outcome of aging that compares with the prominent but localized effects of aging in the thymus (i.e., thymic involution). Localized expression of such genes may be either a cause of consequence of multiple age-related pathologies [79,80], particularly if increased expression arises from sustained inflammation in certain tissues [31]. Consequently, such age-related effects may provide informative endpoints that, in combination with other indicators of aging and healthspan, will eventually lead to surrogates for lifespan in experimental studies of anti-aging interventions using the laboratory mouse [81]. In this sense, other aspects of the aging immune system have also proven informative, such as T-cell subsets, which have been found to predict mouse lifespan within experimental cohorts [82-84]. Along these lines, extended lifespan of mutant mice lacking the pregnancy-associated plasma protein A (*Pappa*) gene is reflected by a delayed rate of thymic atrophy [85].

A conceptual association between CR and aging has been maintained for decades, and numerous investigations have been designed around the expectation that CR interferes with the progression of aging. The alternative view is that CR and aging are in fact disparate phenomena, with CR having many favorable phenotypic effects (e.g., decreased adipose tissue, improved insulin sensitivity, increased survivorship), but not having effects broad enough to be characterized in terms of the actual process of "aging". Gene expression data is not, by itself, sufficient to discriminate between these two views, and it is clear that any sufficient definition of aging must include measurements from multiple physiological domains [17]. Moreover, it is certainly possible that deleterious aspects of aging are prevented by CR in ways that are not reflected by gene expression patterns, and many age-associated expression patterns may indeed reflect favorable compensatory mechanisms rather than pathological processes. Nevertheless, gene expression data reflect the behavior of numerous biochemical pathways, and such data are also well-suited to quantitative investigation. Genome-wide expression data can, in this regard, provide at least some insight into the conceptual links between CR and aging. There are elements of this analysis that support an association between CR and aging, and elements that do not. On the one hand, individual genes can be identified for which the effects of CR oppose those of aging in multiple tissues (e.g., *Igh-6*; see Figure 7), and this also appears to be true among genes associated with certain types of biological processes (e.g., immune response, inflammation, biopolymer metabolic process; see Figures 11 and 12). At a global scale, however, among all genes, this association is fairly weak. This is most clearly shown by the scatterplots in Figure 8, which indicate that genome-wide correlations between CR and aging effects within particular tissues do not exceed 0.10 in magnitude, and this basic conclusion is supported by analyses shown in Figures 9 and 10. Among different tissues, the genome-wide association was stronger in cardiac tissue, but weaker in liver, muscle and central nervous system. Taken together, therefore, these analyses show that CR counters certain types of age-associated expression patterns, but not others, with a fairly weak global association among all genes, and some degree of variability in the strength of the association among mouse tissue types.

## Conclusion

CR and aging have widespread biochemical effects and systems-oriented approaches are needed to develop comprehensive *in silico *models that link these effects conceptually. Microarray analyses provide a data-driven approach for judging which pathways serve as "hubs" that connect the diverse processes modulated by CR and aging, and this can lead to the construction of large gene interaction networks, with scope substantially greater than those generated by standard methods. This study has identified sub-networks, embedded within the genome-wide transcriptional network, that are responsive to either dietary intake or aging across a variety of mouse tissues. CR influenced small co-expression modules containing genes that encode histone clusters (e.g., *Hist2h3c1*, *Hist2h3c2*, *Hist1h2bc*, *Hist2h2aa1*and *Hist1h1c*), period homologues (*Per1*, *Per2 *and *Per3*), as well as a large module containing genes involved in the splicing and processing of mRNA (e.g., *Sfrs18*, *Sfpq*, *Prpf4b*, *Prpf39*). Aging increased expression of granulin (*Grn*) and secreted phosphoprotein 1 (*Spp1*) in more than 14 tissue types, and led to elevated expression of genes and modules associated with inflammation and immune response (e.g., *H2-D1*, *H2-K1*, *Igk-V1 *or *Igh-6*). In general, many age-associated differential expression patterns were explained by the underlying modularity of the transcriptome. It was expected that the transcriptional effects of CR and aging would be negatively associated, and this was true for genes associated with certain biological processes (e.g., immune response and inflammation). At the genome-wide level, however, CR and aging were found to have weak association, which was stronger in heart relative to liver, muscle and central nervous system.

## Methods

### Gene Expression Datasets

Gene expression datasets were obtained from the Gene Expression Omnibus or ArrayExpress public databases [86,87], or were received from contact authors of published studies upon request [23,88,89]. The analyzed data represent a comprehensive collection of most or all experiments that have evaluated the effects of CR and/or aging in the laboratory mouse, including those generated as part of the AGEMAP project [29]. Of 40 experiments that evaluated the effects of CR, 82% (33/40) were derived from studies in which array data had been validated by RT-PCR (Additional File 1). Of 67 experiments that evaluated the effects of aging, 72% (48/67) were derived from studies in which array data had been validated by RT-PCR (Additional File 1). The majority of experiments incorporated into the analysis had thus been shown to generate reliable results in agreement with PCR-based methods.

Most data were generated using Affymetrix oligonucleotide microarray platforms, but Illumina, Agilent or non-commercial arrays were also used in some experiments (Additional File 1). The general strategy followed in linking all datasets was to assign transcripts to one or more probe sets included on the Affymetrix 430 2.0 array. This approach was taken because the 430 2.0 array, with more than 39,000 transcripts, is the most comprehensive mouse oligonucleotide array currently available. It was therefore likely that transcripts represented on less comprehensive platforms would be a subset of those represented on the Affymetrix 430 2.0 array. Additionally, comprehensive and updated annotation information was available for the Affymetrix 430 2.0 array, and this platform was also used to generate many of the datasets considered in the analysis. Data from early generation Affymetrix platforms (e.g., MOE430, MG-U74, Mullk), or from Illumina Mouseref arrays were linked to Affymetrix 430 2.0 identifiers according to best match tables published by array manufacturers. For non-commercial arrays, less annotation information was available, so best match tables were generated from Unigene ids, Entrez ids, or alignment scores between probe sequences. In a small fraction of cases, multiple transcripts from one platform corresponded with the same probeset on the Affymetrix 430 2.0 array, and no annotation was available to determine which transcript better matched the Affymetrix 430 2.0 array transcript. In these cases, the transcript associated with the most significant CR (or aging) effect was selected, and the remaining (less significant) transcripts were discarded from further analyses. This selection procedure ensured that significant results were not overlooked, but may be regarded as non-conservative, because tests used to select an appropriate transcript are not accounted for when adjusting p-values to control the false discovery rate among all genes included in the analysis (described below). The number of excess tests performed was small, however, and the significance of identified genes is not altered when the false discovery rate is controlled for a number of tests that greatly exceeds the number of genes considered in the overall analysis. Methods for normalization and calculation of expression scores varied, depending upon whether raw data was available from corresponding authors. For Affymetrix datasets in which CEL files were available, the gcRMA algorithm was used for normalization and calculation of expression scores. For other platforms, or when raw data was not available, methods included RMA, average difference, quantile normalization, and calculation of Z-scores. While these differences in pre-processing were not ideal, the deleterious effect of such differences was minimized by analyzing all experiments independently, without combining expression scores from different studies (see below).

### Statistical Methods

Statistical methods for detection of differentially expressed genes were similar to those described previously [12]. For a small number of datasets, it was not possible to obtain raw data for analysis. In these cases, the only option was to generate differential expression signatures based upon supplemental data files provided in original research reports, in which significant CR and age-regulated genes were identified based upon standard t-tests. In most cases, however, raw data was available, and in these instances, differential expression was evaluated based upon linear model analysis. Most experiments utilized a standard two-treatment design (e.g., CR versus *ad lib*, or young versus old). To analyze these experiments, the limma linear modeling package was used [90], which bases statistical inference upon an empirical Bayes moderated t-statistic. The denominator of this statistic is shrunk towards a prior value that is set, in part, using information borrowed across all transcripts, which stabilizes variance estimates associated with individual genes [90]. For some experiments, the design was more complex, and there were additional experimental groups or covariates to be taken account of (e.g., gender and age). For such experiments, linear models included both gender, along with age, in order to calculate a treatment effect adjusted for these factors. For some aging experiments involving more than just "young" and "old" treatments, the effect of age was treated as a continuous factor embedded within a standard regression model, along with any other potentially relevant covariates (e.g., gender).

Fisher's method of p-value combination was used to integrate differential expression results involving the same tissue type, as well as results from different types of tissues [91]. Given this meta-analytic approach, risk of false-positive identification is considerably reduced relative to that associated with analysis of a single dataset [92]. For individual tissues, p-values were integrated among *i *= 1,..., *n *separate experiments using the statistic -2Σlog(*P*_*i*_), which has a *χ*^2 ^distribution with 2*n *degrees of freedom if each of the *n *null hypotheses being considered is true. For some tissues, data from multiple experiments was not available (Table 1) and it was therefore not necessary to apply Fisher's method. Once p-values for each individual tissue type had been obtained, the statistic -2Σlog(*P*_*j*_) was used to evaluate significance of expression patterns among the *j *= 1,..., *m *tissues (*m *= 17 and 22 for the CR and aging analyses, respectively). In applying Fisher's method, a consideration was whether to combine p-values generated by one-sided tests for up regulation, one-sided tests for down regulation, or two-sided tests for either up or down regulation. In some cases, the interest was to identify genes consistently up regulated or down regulated, either among replicate experiments evaluating the same tissue, or among experiments evaluating different tissues (e.g., see Figures 2 and 5). For this purpose, Fisher's method was applied to p-values generated from one-sided hypothesis tests, where the *P*_*i *_and *P*_*j *_correspond to specific tests for an expression shift in a particular direction (up or down). For other analyses, the purpose was to extract differential expression signatures for making comparisons among tissues (e.g., Figures 1 and 4), or between the effects of CR and aging (e.g., Figures 9 and 10). In these cases, Fisher's method was used to calculate a one-sided p-value for up regulation (P_*u*_), as well as a one-sided p-value for down regulation (P_*d*_), and then the overall p-value was taken to be the lower of these quantities, multiplied by a factor of 2 (i.e., 2 × min(P_*u*_, P_*d*_)) [29]. As an additional criterion, for a transcript to be considered significantly up regulated, it was required that P_*d *_> 0.05. Likewise, for a transcript to be considered significantly down regulated, it was required that P_*u *_> 0.05. Fisher's method assigns a disproportionate weight to very small p-values, which can lead to a significant overall p-value, even when most p-values being combined suggest a weak treatment effect. To guard against this possibility, p-values less than 10^-3 ^were set equal to 10^-3^. This measure introduced a degree of conservatism in results and ensured that p-values were not disproportionately influenced by exceptionally low p-values associated with certain experiments. As a final step, Fisher p-values generated from the across-tissue meta-analysis were adjusted for multiple testing using the Benjamini-Hochberg method [93]. P-values generated by Fisher's method that have been cited in text are therefore adjusted to account for multiple hypothesis testing among all genes considered in the analysis. Symbols corresponding to differential expression results in Figures 2 -5, however, are based upon comparison-wise type I error rates of 0.05 (i.e., symbols represent p-values that are not adjusted to control the experiment-wise false discovery rate).

It was common for arrays to include multiple transcripts annotated with the same gene symbol. To eliminate redundancy in the results, and in comparisons among differential expression signatures, it was necessary to pool results among those transcripts associated with the same gene symbol. This was done by selecting the transcript for which effects of CR (or aging) were most significant. By summarizing information in this fashion, a risk is that alternative transcripts associated with the same gene symbol could exhibit differential responses to CR or aging, which might be overlooked. This was, however, unusual, and in tissue-specific analyses (Additional Files 2, 3, 4, 7, 8 and 9), conflicts among multiple transcripts associated with the same gene symbol are indicated. This procedure led to a total of 21, 327 genes upon which most analyses were based. The Affymetrix 430 2.0 array also included 5,794 transcripts that were not associated with any gene symbol. These transcripts seldom had matches with transcripts from other arrays, and were therefore often eliminated from analyses. In this paper and in Additional data files, such transcripts are identified based upon a Unigene ID or an Affymetrix 430 2.0 probe set ID.

### Distance between Differential Expression Signatures

The distance between differential expression signatures was evaluated based upon adjusted residuals (e.g., see Figures 1, 4 and 7). In the analysis of contingency tables, adjusted residuals reflect the magnitude of deviation from expected cell counts, where expected cell counts are calculated under the assumption that the categorical variables being cross-tabulated are independent. For a cell in row *i *and column *j*, with observed cell count *n*_*ij*_, and expected cell count *μ*_*ij*_, the adjusted residual *R*_*ij *_is defined by Equation (1) [94].(1)

The values *p*_*i*+ _and *p*_+*j *_represent marginal frequencies based upon row and column totals of the contingency table. For instance, if all cell counts sum to *N*, the value of *p*_*i*+ _is , and the value of *p*_+*j *_is . For large samples, adjusted residuals are expected to follow a standard normal distribution, such that values greater than 3 in absolute value are suggestive of a significant deviation between observed and expected cell counts [94]. For a comparison between two differential expression signatures, a contingency table is formed that includes a total of nine cells (see Figure 7), and for each cell, the adjusted residual indicates whether there is an over or under-abundance of genes within that particular category.

In Figures 1 and 4, a distance measure (*d*) was used to compare differential signatures, where *d *is an element of the interval [0, 1]. The value of *d *is based upon two adjusted residual values (*R*_1 _and *R*_2_). The value of *R*_1 _is the adjusted residual associated with a contingency table cell that contains the number of genes up regulated by CR (or aging) in both signatures. The value of *R*_2 _is the adjusted residual associated with a cell containing the number of genes down regulated by CR (or aging) in both signatures. Since both *R*_1 _and *R*_2_, follow the standard normal distribution for large samples, the value of *d *was calculated by applying the standard normal cumulative distribution function, Φ(•), to both *R*_1 _and *R*_2_.(2)

The distance *d *is therefore the average of two p-values. The first p-value, 1-Φ(*R*_1_), addresses whether there exists an over-abundance of genes up regulated with respect to both signatures, and the second p-value, 1-Φ(*R*_2_), addresses whether there exists an under-abundance of genes down regulated with respect to both signatures. Values of *d *less than 0.05 are therefore suggestive of non-random associations between differential expression signatures. This measure corresponded well with results from significance tests of differential expression association using methods that have been described previously [24], based upon the binomial distribution approximation (Figures 1B and 4B; below-diagonal region).

### Gene Co-Expression Patterns

The co-expression of genes was evaluated based upon patterns across a large database of 3,700 Affymetrix 430 2.0 oligonucleotide arrays. These arrays included hybridizations from a wide range of RNA sources extracted in different tissue types and under various experimental conditions. This expression profiling approach has previously been used in mouse and other organisms to identify co-expression patterns and to infer transcriptional networks [95-98], and several helpful reviews are available that describe concepts and methodologies associated with this systems-level approach [99,100]. To obtain the 3,700 arrays for expression profiling, an initial batch of 5,700 CEL files was downloaded from Gene Expression Omnibus, and each file was evaluated according to four quality control criteria (average background, scale factor, percent present, RNA degradation) [101]. The average background score provides an indication of the signal strength associated with the lowest-intensity probes on each array. It was desirable that all arrays used for expression profiling have similar background scores, so background scores were computed for all 5,700 arrays, and those with scores below percentile 7.5 and above percentile 92.5 were eliminated. The scale factor is a measure of the global intensity difference between a given array and a baseline target intensity. To ensure that all arrays used for expression profiling had similar scale factors, and thus similar overall intensity, arrays with factors below percentile 7.5 and above percentile 92.5 were removed from analyses. The percent present refers to the percentage of probe sets on an array for which intensity is sufficient to be called "present", or expressed above background, by a detection algorithm. Arrays with percent present calls below the 15th percentile were removed from analyses. Lastly, arrays that potentially involved hybridization with low-quality RNA were eliminated based upon a genome-wide RNA degradation score. This score is calculated based upon the degree of intensity difference between probes associated with the 5' end of transcripts and probes associated with the 3' end of transcripts. Arrays with degradation scores above the 85th percentile were removed from the analysis. After arrays had been filtered according to the above-described quality control measures, there remained 3,700 arrays and these were used for expression profiling.

The 3,700 arrays were randomly assigned to 37 batches, and for each batch separately, expression scores were calculated using the RMA algorithm. The gcRMA algorithm was not used, as in differential expression analyses (see above), because a prior study has reported that gcRMA normalization may lead to over-estimation of co-expression relationships [102]. This procedure generated a total of 37 independent datasets, each with expression values corresponding to 100 separate conditions. To evaluate the co-expression of two transcripts, say *X *and *Y*, the Pearson correlation coefficient (*r*) between *X *and *Y *was calculated with respect to each of the 37 datasets individually, and the 37 values of *r *were then averaged to generate a single measure of co-expression between probe sets *X *and *Y*. For all co-expression and network analyses (e.g., Figures 3 and 6), the distance between gene elements was based upon the absolute value of the Pearson correlation coefficient (i.e., 1 - |*r*|). All co-expression calculations were performed after eliminating 4547 Affymetrix probe sets (10% of those represented on the Affymetrix 430 2.0 Mouse Genome Array), which were associated with identifiers that included an "_s" or an "_x" suffix. Based upon probe sequence information, such probe sets could cross-hybridize with multiple transcripts from two separate genes. It was therefore necessary to remove these probe sets, prior to co-expression analyses, to ensure that some co-expression estimates were not upwardly biased by probe sets that cross-hybridize with multiple mRNA transcripts in a non-specific fashion [103,104].

It was often necessary to evaluate the co-expression between gene symbols or Unigene identifiers for which there were multiple transcripts represented on the Affymetrix 430 2.0 array. As an illustrative example, suppose that gene symbol *X *is represented by transcripts *x*_1 _and *x*_2_, while gene symbol *Y *is represented by transcripts *y*_1 _and *y*_2_. A summary distance measure was used for evaluating the co-expression of *X *and *Y*, based on all combinations of transcripts associated with X and Y. In this example, three values of *r *were calculated, one for the association between *x*_1 _and *y*_1_, one for the association between *x*_1 _versus *y*_2_, and one for *x*_2 _and *y*_2_. The overall correlation between *X *and *Y *was then taken as the single value of *r *that was largest in absolute value. This approach was applied for all such cases involving multiple transcripts associated with a given gene symbol.

Co-expression modules of varying size (2, 3, 5, 10, 20 and 40 genes) were identified based upon the "nearest neighbor" concept. In this approach, a co-expression module was formed for each gene included in the analysis, by identifying the 1 to 39 other genes with expression patterns that were most similar. This led to a total of 21,327 co-expression modules, and as expected, there was some degree of overlap in the composition of closely related modules. After co-expression modules had been identified, each was scored to determine which was most strongly associated with modified expression under CR or aging. This was done using the statistic *M*, which is based upon one-sided p-values used to test for up and downregulation of particular genes (P_*u *_and P_*d*_). For a module containing *i *= 1,..., *G *genes, the *M *statistic integrated results from significance analyses among *j *= 1,..., *T *tissue types in a fashion similar to Fisher's method.(3)

Large values of *M *suggest strong regulation of the module by CR (or aging), whilesmaller values of *M *suggest weak regulation of the module by CR (or aging). The value of *M *for a given module is inversely related to the average value of min(P_*u*_, P_*d*_) among the *G *genes included in a module. The quantity min(P_*u*_, P_*d*_) is calculated for each gene according to Equation (4), based upon *T *tissues for each gene, where *T *may not be identical for all genes within a module, since data was often not available for every tissue. Because Equation (3) is scaled by the factor 1/*G*, while Equation (4) is scaled by the factor (1/*T*), values of *M *should be comparable among modules that differ in size or in the number of tissue types for which data is available. In general, however, the data suggested that *M *decreased as module size increased.

The significance of the *M *values associated with each co-expression module was assessed by simulation. The simulation approach addressed whether co-expression patterns contribute meaningful information, by evaluating whether groups of transcripts formed using coexpression patterns are, collectively, more responsive to CR or aging than groups of transcripts that have been formed at random. In each simulation trial, a total of *N *= 21,327 modules of size *G *were formed by randomly assigning *G*-1 transcripts to each of the *N *total transcripts, and calculating the value of *M *for each of the 1,..., *N *randomly generated modules. The maximum value of *M *among all 21,327 randomly formed modules, referred to as M*, was then determined. This process was repeated in 2,000 simulation trials, and for each trial, the value of M* was recorded, providing a null distribution that reflected the largest value of *M *likely to arise among *N *modules formed randomly, without any reference to the observed co-expression patterns. Observed values of *M *were then compared to the M* null distribution in order to generate p-values and evaluate the significance of individual co-expression modules. The final step in the analysis was to screen all modules, and retain those modules most responsive to CR (or aging), while discarding less responsive modules that overlapped with more highly ranked modules. This was done by ranking all modules from most to least responsive to CR (or aging) (i.e., rank modules by *M*), and discarding any module sharing at least one gene with any more responsive module of higher rank.

## Supplementary Material

Additional file 1 **Gene expression datasets**. Description of the CR and aging datasets included in this analysis. Click here for file

Additional file 2 **Genes regulated by caloric restriction in liver**. Results from 10 experiments are analyzed to identify genes significantly up and down regulated by CR in liver. This file also includes analysis of associated gene ontology terms, KEGG pathways, microRNA targets and chromosomal locations of CR-regulated genes. Click here for file

Additional file 3 **Genes regulated by caloric restriction in heart**. Results from 7 experiments are analyzed to identify genes significantly up and down regulated by CR in heart. This file also includes analysis of associated gene ontology terms, KEGG pathways, microRNA targets and chromosomal locations of CR-regulated genes. Click here for file

Additional file 4 **Genes regulated by caloric restriction in muscle**. Results from 4 experiments are analyzed to identify genes significantly up and down regulated by CR in muscle. This file also includes analysis of associated gene ontology terms, KEGG pathways, microRNA targets and chromosomal locations of CR-regulated genes. Click here for file

Additional file 5 **Genes regulated by caloric restriction in multiple mouse tissues**. A gene chart is presented that provides a comprehensive listing of the genes most strongly increased by CR across tissues, most strongly decreased by CR across tissues, and most strongly regulated by CR (in either direction) across tissues. The chart is comparable to those shown in Figure 2, except genes are ranked based upon a p-value generated using Fisher's method, rather than the total number of tissue types in which a gene is up or down regulated by CR. This file also includes analysis of associated gene ontology terms, KEGG pathways, microRNA targets and chromosomal locations of CR-regulated genes. Click here for file

Additional file 6 **Gene expression modules regulated by caloric restriction in multiple mouse tissues**. This file provides a description of the most significant CR-regulated co-expression modules. Co-expression modules of varying size are shown (2, 3, 5, 10, 20 and 40 genes), along with their patterns of differential expression across the mouse tissues examined (e.g., see Figure 3). Click here for file

Additional file 7 **Genes regulated by aging in liver**. Results from 8 experiments are analyzed to identify genes significantly up and down regulated by aging in liver. This file also includes analysis of associated gene ontology terms, KEGG pathways, microRNA targets and chromosomal locations of age-regulated genes. Click here for file

Additional file 8 **Genes regulated by aging in heart**. Results from 5 experiments are analyzed to identify genes significantly up and down regulated by aging in heart. This file also includes analysis of associated gene ontology terms, KEGG pathways, microRNA targets and chromosomal locations of age-regulated genes. Click here for file

Additional file 9 **Genes regulated by aging in muscle**. Results from 4 experiments are analyzed to identify genes significantly up and down regulated by aging in muscle. This file also includes analysis of associated gene ontology terms, KEGG pathways, microRNA targets and chromosomal locations of age-regulated genes. Click here for file

Additional file 10 **Genes regulated by aging in multiple mouse tissues**. A gene chart is presented that provides a comprehensive listing of the genes most strongly increased by age across tissues, most strongly decreased by age across tissues, and most strongly regulated by age (in either direction) across tissues. The chart is comparable to those shown in Figure 5, except genes are ranked based upon a p-value generated using Fisher's method, rather than the total number of tissue types in which a gene is up or down regulated by age. This file also includes analysis of associated gene ontology terms, KEGG pathways, microRNA targets and chromosomal locations of age-regulated genes. Click here for file

Additional file 11 **Gene expression modules regulated by aging in multiple mouse tissues**. This file provides a description of the most significant age-regulated co-expression modules. Co-expression modules of varying size are shown (2, 3, 5, 10, 20 and 40 genes), along with their patterns of differential expression across the mouse tissues examined (e.g., see Figure 6). Click here for file
