# Supplementary material for: Genes and gene expression modules associated with caloric restriction and aging in the laboratory mouse
Source: BMC Genomics. 2009 Dec 7;10:585. doi: 10.1186/1471-2164-10-585 (PMC2795771; doi:10.1186/1471-2164-10-585)
Supplement: Additional file 2 — Genes regulated by caloric restriction in liver. Results from 10 experiments are analyzed to identify genes significantly up and down regulated by CR in liver. This file also includes analysis of associated gene ontology terms, KEGG pathways, microRNA targets and chromosomal locations of CR-regulated genes. [file 1471-2164-10-585-S2.PDF]

Additional File 2

Genes and Gene Expression Modules Associated with Caloric Restriction and Aging in the Laboratory Mouse

William R. Swindell
University of Michigan, Departments of Pathology and Geriatrics

---

Genes Regulated by Caloric Restriction in Liver

This file provides information on genes significantly influenced by CR in the liver. The first set of charts displays differential expression results of the 200 genes most strongly up regulated by CR in liver, while the second set of charts displays differential expression results for the 200 genes most strongly down regulated by CR in liver. Each row corresponds to an individual gene and each column corresponds to a separate experiment (see Additional File 1). Symbols are interpreted as follows:

- ● Gene is significantly up regulated by CR ($P_u < 0.05$)
- ● Gene is significantly down regulated by CR ($P_d < 0.05$)
- ▬ Gene is marginally up regulated by CR ($0.05 < P_u < 0.10$)
- ▬ Gene is marginally down regulated by CR ($0.05 < P_d < 0.10$)
- ▬ Non-significant CR effect ($P_u > 0.10$ and $P_d > 0.10$)
- × No data (gene not represented in experiment or array annotation was limiting)
- ✳ Evidence conflicts but favors up regulation by CR
- ✳ Evidence conflicts, but favors down regulation by CR

The last two categories (✳ and ✳) indicate significant effects with conflicting evidence. This can arise if multiple transcripts associated with the same gene symbol yield opposite conclusions. Alternatively, a conflict may arise if $P_u < 0.05$ and also $P_d < 0.05$. Symbols shown in charts are based upon a comparison-wise type I error rate of 0.05. The final column in each chart lists meta-analysis p-values generated using Fisher's method, which have been adjusted using the Benjamini-Hochberg method to control the false discovery rate among all 21,327 genes.

The remainder of the file includes lists of over-represented gene ontology terms, over-represented KEGG pathways, and over-represented KEGG pathways defined based upon IP domain signatures (see Hahne et al. 2008, BMC Bioinformatics 9:3). Genes were also analyzed to determine if there existed an over-abundance of targets for certain microRNAs (see Betel et al. 2008, Nucleic Acids Res. 36: D149-153), and a list of associated microRNAs is provided based upon this analysis. Lastly, tests for over-representation of identified genes with respect to each chromosome were performed, and an idiogram mapping of identified genes to chromosomal locations is shown.

Contact: William R. Swindell, wswindel@umich.edu

↑ CR

Genes up regulated by CR

	lvr1	lvr4a	lvr4b	lvr5	lvr10	lvr13	lvr14	lvr16	lvr20	lvr22	P_u
Fmo3	●	–	●	●	×	●	●	×	×	●	2.81e−08
Slc37a4	●	●	●	●	×	●	×	●	×	●	2.81e−08
Lpin2	●	●	●	●	×	●	×	×	×	●	2.86e−08
Lpin1	●	●	●	●	×	●	×	×	×	●	4.12e−08
Glrx	●	●	●	●	×	●	×	×	×	×	5.8e−07
Eif1	●	●	●	●	×	●	×	×	×	●	1.28e−06
Map1lc3b	*	●	●	●	×	×	×	● (green)	×	●	2.08e−06
Akr1b7	●	●	–	●	×	●	×	–	×	●	2.08e−06
Rbm3	*	●	●	●	●	●	×	×	×	–	2.34e−06
Samm50	– (red)	●	●	●	×	●	×	×	×	●	3.59e−06
Fkbp5	●	●	–	●	×	●	×	● (green)	●	●	5.44e−06
Rgs16	●	●	●	–	×	●	×	–	×	●	5.44e−06
Sin3b	●	●	●	●	×	●	×	×	×	–	6.86e−06
St3gal5	●	●	– (red)	●	●	●	×	×	●	×	6.86e−06
Txnip	●	●	–	●	×	●	×	×	×	●	7.02e−06
Lepr	●	●	●	●	×	●	×	–	×	●	1.22e−05
Slc19a1	●	●	●	●	×	●	–	● (green)	×	–	1.5e−05
Slc38a2	●	●	–	– (red)	×	●	×	●	×	●	1.5e−05
Cyp4a14	●	●	–	– (red)	●	●	×	×	×	●	1.5e−05
Cebpd	●	–	●	–	×	×	×	●	×	●	1.58e−05
Mdh2	●	●	●	●	×	●	●	×	×	×	1.58e−05
Gstt2	●	●	●	–	×	●	×	●	×	– (green)	1.58e−05
Idh2	●	●	–	●	×	●	×	●	●	–	2.06e−05
Gne	●	●	●	●	×	●	×	● (green)	×	●	2.35e−05
Zbtb16	●	●	●	●	×	×	×	–	×	●	3.39e−05
Il1r1	●	●	●	●	×	×	×	×	×	●	3.93e−05
Slc20a1	●	●	●	–	×	×	×	–	×	●	3.93e−05
Fmo5	●	●	●	●	×	●	×	×	×	–	4.12e−05
Usp2	●	●	– (red)	●	×	●	×	×	×	×	4.51e−05
Etf1	●	●	–	●	×	●	×	×	×	●	4.59e−05

↑ CR

Genes up regulated by CR

	lvr1	lvr4a	lvr4b	lvr5	lvr10	lvr13	lvr14	lvr16	lvr20	lvr22	P_u
Serinc3	●	●	–	●	×	×	×	×	●	●	4.59e−05
Rpl22l1	●	●	●	●	×	×	×	×	×	●	4.99e−05
Cyhr1	*	●	–	●	×	●	×	×	×	–	5.03e−05
Gch1	●	●	●	●	×	●	×	×	×	–	5.23e−05
Gfra1	●	–	–	●	×	×	●	×	×	●	5.23e−05
D16H22S680E	–	●	●	●	×	×	×	×	●	●	6.33e−05
Per1	●	–	●	●	×	●	×	×	×	●	6.61e−05
Rps18	●	–	●	●	×	●	×	×	×	●	6.66e−05
Slc7a2	●	–	–	●	×	●	×	×	×	●	6.77e−05
Ehhadh	●	●	–	–	×	●	×	●	×	–	7.27e−05
Igfbp2	●	●	●	●	×	●	●	×	×	×	7.27e−05
Rps10	●	●	●	●	×	●	×	×	×	●	7.65e−05
Clta	*	●	–	●	×	●	×	–	×	–	7.75e−05
Enpp1	●	●	–	●	×	●	–	×	×	–	7.75e−05
Pck1	●	●	●	●	×	●	●	●	×	–	7.75e−05
Rps16	–	●	●	●	×	×	×	×	×	●	7.75e−05
Grem2	●	●	●	–	×	●	×	–	×	●	7.75e−05
Ctgf	–	●	●	●	×	●	●	×	×	●	8.17e−05
Ahcy	●	–	–	●	×	●	×	×	●	●	8.58e−05
Pnpla2	●	●	●	●	×	●	×	–	×	●	9e−05
Bcl2l11	●	●	●	–	×	●	×	×	×	●	9.97e−05
Rpl13	●	●	–	●	×	●	×	×	×	●	9.97e−05
Herpud1	●	●	●	–	×	●	×	×	●	●	0.000116
Mfsd2	●	×	×	×	×	×	×	●	×	●	0.000119
Rnf166	●	●	–	●	×	●	×	×	×	●	0.000122
Hsd17b11	●	●	●	●	×	×	×	–	×	×	0.000136
[illegible]USG00000022086	●	●	●	●	×	×	×	×	×	×	0.000149
Rab4a	●	●	–	●	×	●	×	●	×	×	0.00015
Rps12	●	●	●	●	×	×	×	×	×	●	0.00015
Map3k5	●	●	–	●	×	×	×	×	×	●	0.000152

↑ CR

Genes up regulated by CR

	lvr1	lvr4a	lvr4b	lvr5	lvr10	lvr13	lvr14	lvr16	lvr20	lvr22	P_u
Map2k6	●	●	●	–	×	●	×	– (red)	×	●	0.000154
Zfand6	●	●	●	●	×	×	×	● (green)	×	×	0.000157
Rps15a	●	●	–	●	×	×	×	–	×	×	0.00016
Ndel1	●	●	–	●	×	●	×	–	×	–	0.000175
Cyp4a10	●	●	–	–	●	●	×	×	×	×	0.000177
Tef	●	●	●	●	×	●	×	×	×	● (green)	0.000179
2410015N17Rik	●	– (red)	– (red)	●	×	●	×	–	×	●	0.000193
Man2a1	●	●	●	●	×	×	×	– (red)	×	●	0.000213
Angptl4	●	●	–	●	×	×	×	×	×	●	0.00025
Grsf1	●	●	–	●	×	●	×	– (green)	×	×	0.00025
Rad51l1	●	●	–	–	●	●	×	×	×	–	0.00025
Parp16	●	×	×	×	×	×	×	●	×	●	0.00025
Ell2	●	●	– (red)	– (green)	×	×	×	●	×	●	0.000253
Dnajb6	●	– (red)	●	*	×	×	×	×	×	–	0.000284
Gnb2l1	● (green)	●	– (red)	●	×	×	×	–	×	●	0.00029
Klhl21	●	●	–	●	×	●	×	● (green)	×	●	0.00029
Cyp2c39	●	–	–	●	×	●	×	●	×	–	0.00029
Abcc2	●	–	●	–	×	●	×	●	×	●	0.000317
Cyp2b9	●	–	– (green)	–	×	●	×	×	●	●	0.00035
Cyp2b13	●	–	– (green)	●	×	●	×	×	●	●	0.000357
Cry1	●	●	●	●	×	×	×	×	×	●	0.000366
Mfn1	●	●	●	●	×	●	×	×	×	●	0.000424
Sdhd	●	●	–	●	×	●	×	●	×	×	0.000441
Rab9	●	●	–	●	×	×	×	×	×	×	0.000444
Alas1	●	●	●	– (red)	×	●	×	×	×	●	0.000457
Rps5	●	●	●	●	×	●	–	×	×	●	0.000457
Hspa9	●	●	●	●	×	●	×	×	×	×	0.000526
F5	–	●	●	●	×	×	●	×	×	– (red)	0.000555
Hadhb	*	●	– (red)	●	×	×	×	×	×	–	0.000561
Rad9b	●	●	●	–	×	●	×	● (green)	×	×	0.000561

↑ CR

Genes up regulated by CR

	lvr1	lvr4a	lvr4b	lvr5	lvr10	lvr13	lvr14	lvr16	lvr20	lvr22	P_u
Uqcrc1	●	●	–	●	×	●	×	●	×	– (red)	0.000566
1110038D17Rik	– (green)	●	–	●	×	×	×	×	×	●	0.000572
Cyp2a5	●	–	–	●	×	●	●	×	×	● (green)	0.000626
Pfkfb3	●	●	●	●	×	×	×	×	×	●	0.000639
Timm9	●	●	●	●	×	×	×	×	×	–	0.000642
Chchd7	●	●	–	●	×	●	×	–	×	●	0.000647
Mat1a	●	×	×	×	×	×	×	●	×	●	0.000663
Nudt19	●	●	–	● (green)	×	●	×	●	×	×	0.000672
Chd1	● (green)	●	– (green)	●	×	×	●	–	×	●	0.000672
Dus1l	– (red)	●	–	●	×	●	×	×	×	×	0.000672
AI132487	●	●	–	– (red)	×	●	×	–	×	×	0.000672
Rpl27a	– (red)	●	●	●	×	×	×	×	×	×	0.000691
Sgk1	●	●	– (red)	–	×	●	×	● (green)	×	●	0.000715
Bckdhb	–	●	●	●	×	●	×	×	×	–	0.000723
Unc84b	* (green)	●	●	●	×	×	×	×	×	×	0.000744
Gpi1	– (green)	●	●	●	×	●	×	×	×	●	0.000804
0610005C13Rik	●	–	●	●	×	●	×	×	×	×	0.000816
1110008F13Rik	* (red)	●	–	●	×	●	×	–	×	–	0.000836
Hspa2	●	–	–	–	×	●	– (green)	●	×	●	0.000836
Fh1	●	–	–	●	×	●	– (red)	×	×	●	0.00087
Ncl	●	–	–	●	×	●	×	– (green)	×	–	0.00091
Nfkbia	●	●	– (red)	–	×	●	×	×	×	●	0.00091
1110002B05Rik	●	●	●	–	×	●	×	–	×	–	0.00106
Eno1	● (green)	●	●	●	×	●	×	×	×	– (red)	0.00108
Sult1d1	●	– (green)	– (red)	–	●	●	×	×	×	●	0.00108
Rps3	●	●	–	●	×	×	×	×	×	●	0.00108
Tnfrsf1b	●	–	●	–	×	×	●	×	×	●	0.00116
Aco2	●	–	–	–	×	●	×	●	×	●	0.00122
Rnf145	●	●	●	●	×	×	×	–	×	–	0.00122
Por	●	–	–	●	×	●	×	×	●	×	0.00126

↑ CR

Genes up regulated by CR

	lvr1	lvr4a	lvr4b	lvr5	lvr10	lvr13	lvr14	lvr16	lvr20	lvr22	P_u
**Ndufv1**	– (red)	– (red)	–	● (red)	×	● (red)	– (red)	● (red)	×	● (red)	**0.00135**
**Kat2b**	* (red)	● (red)	● (red)	–	×	×	×	×	×	–	**0.0015**
**Rpl30**	● (red)	● (red)	–	● (red)	×	×	×	×	×	● (red)	**0.00158**
**Fgf1**	● (red)	×	×	×	×	×	● (red)	×	×	● (red)	**0.00159**
**Rps27**	● (red)	● (red)	–	● (red)	×	×	×	×	×	×	**0.00163**
**Nt5e**	* (red)	● (green)	● (green)	● (red)	×	● (red)	–	×	×	● (red)	**0.00166**
**Pcsk4**	● (red)	● (red)	● (red)	● (green)	×	● (red)	● (green)	–	×	–	**0.00184**
**Rps27a**	● (red)	● (red)	–	● (red)	×	×	×	×	×	● (red)	**0.00189**
**Tial1**	● (green)	● (red)	– (red)	● (red)	×	×	– (green)	×	×	● (red)	**0.0019**
**Igfbp1**	● (red)	– (red)	● (red)	● (red)	×	×	×	×	×	×	**0.0019**
**Srpr**	– (red)	● (red)	● (red)	● (red)	×	×	×	×	×	×	**0.00192**
**Nrtn**	● (red)	● (red)	–	● (red)	×	×	×	–	×	● (red)	**0.00192**
**Eif5**	● (red)	● (red)	–	– (green)	×	● (red)	● (red)	×	×	×	**0.00194**
**Npm1**	● (red)	● (red)	–	● (red)	×	×	×	×	×	–	**0.00197**
**Strn3**	* (green)	● (red)	–	–	● (red)	● (red)	×	×	×	● (red)	**0.00201**
**Baiap2l1**	● (red)	● (red)	–	● (red)	×	×	×	–	×	● (red)	**0.00202**
**Rpl36**	–	● (red)	– (red)	● (red)	×	×	×	×	×	● (red)	**0.00203**
**Btg3**	– (red)	● (red)	● (red)	–	×	×	×	×	×	● (red)	**0.0021**
**Abcd2**	● (red)	–	–	–	● (red)	● (red)	×	–	×	–	**0.0021**
**Ak3l1**	● (red)	● (red)	– (red)	–	×	● (red)	×	×	×	● (green)	**0.0021**
**Atf6**	● (red)	– (red)	–	–	×	×	×	×	×	● (red)	**0.0021**
**C230093N12Rik**	● (red)	– (green)	– (red)	–	×	×	×	● (red)	×	×	**0.0021**
**Dact2**	● (red)	×	×	×	×	×	×	–	×	● (red)	**0.0021**
**Dusp3**	● (red)	×	×	×	×	×	×	×	×	● (red)	**0.0021**
**Fmo2**	● (red)	×	×	×	×	×	×	×	×	● (red)	**0.0021**
**Foxo1**	● (red)	×	×	×	×	×	×	–	×	● (red)	**0.0021**
**Gpr123**	● (red)	×	×	×	×	×	×	● (red)	×	×	**0.0021**
**Gsta2**	● (red)	● (red)	– (red)	–	×	● (red)	×	×	×	● (green)	**0.0021**
**Hkdc1**	● (red)	×	×	×	×	×	×	● (red)	×	×	**0.0021**
**Il6ra**	● (red)	● (green)	– (green)	–	×	×	● (red)	×	×	×	**0.0021**

↑ CR

Genes up regulated by CR

	lvr1	lvr4a	lvr4b	lvr5	lvr10	lvr13	lvr14	lvr16	lvr20	lvr22	P_u
Mapk8ip2	● (red)	×	×	×	×	×	×	● (red)	×	×	0.0021
Mpzl2	● (red)	– (green)	–	– (red)	×	×	● (red)	×	×	×	0.0021
Mycn	● (red)	–	–	–	×	×	● (red)	×	×	×	0.0021
Npal1	● (red)	×	×	×	×	×	×	×	×	● (red)	0.0021
Nudt4	● (red)	– (red)	–	–	×	×	×	×	×	● (red)	0.0021
Pabpc4	● (red)	– (red)	● (red)	–	×	×	×	×	×	● (red)	0.0021
Pdk1	● (red)	–	–	– (red)	×	● (red)	×	● (red)	×	×	0.0021
Pear1	● (red)	×	×	×	×	×	×	×	×	● (red)	0.0021
Plcb1	● (red)	● (green)	– (green)	● (red)	×	×	×	–	×	● (red)	0.0021
Ppargc1a	● (red)	×	×	×	×	×	×	– (red)	×	● (red)	0.0021
Ppm1a	* (red)	● (red)	–	● (red)	×	● (red)	×	×	×	–	0.0021
Prei4	● (red)	×	×	×	×	×	×	×	×	● (red)	0.0021
Ptp4a1	● (red)	–	–	● (green)	● (red)	×	×	×	×	● (red)	0.0021
Ranbp2	● (red)	×	×	×	×	×	×	● (red)	×	×	0.0021
Sf3b1	● (red)	● (green)	● (green)	● (green)	×	● (red)	×	×	×	● (red)	0.0021
Sfpq	● (red)	–	● (red)	–	×	×	×	×	×	● (red)	0.0021
Slc25a25	● (red)	×	×	×	×	×	×	● (green)	×	● (red)	0.0021
Slc25a29	● (red)	×	×	×	×	×	×	×	×	● (red)	0.0021
Slc25a32	● (red)	×	×	×	×	×	×	–	×	● (red)	0.0021
Tbc1d15	● (red)	● (red)	–	● (green)	×	×	×	×	×	● (red)	0.0021
Tbc1d8	● (red)	×	×	×	×	×	×	–	×	● (red)	0.0021
Tmem38b	● (red)	● (red)	–	–	×	● (red)	×	×	×	–	0.0021
Tmtc2	● (red)	×	×	×	×	×	×	– (red)	×	● (red)	0.0021
Tob2	● (red)	×	×	×	×	×	×	–	×	● (red)	0.0021
Tsc22d3	● (red)	–	● (red)	–	×	×	×	×	×	● (red)	0.0021
Usf2	● (red)	–	–	● (red)	×	● (red)	● (red)	×	×	–	0.0021
1110003O08Rik	● (red)	×	×	×	×	×	×	×	×	● (red)	0.0021
1450373_at	● (red)	–	● (red)	● (red)	×	×	×	×	×	×	0.0021
Aass	● (red)	–	–	● (red)	×	×	×	–	×	● (red)	0.0021
Abcg5	● (red)	×	×	×	×	×	×	● (red)	×	– (red)	0.0021

↑ CR

Genes up regulated by CR

	lvr1	lvr4a	lvr4b	lvr5	lvr10	lvr13	lvr14	lvr16	lvr20	lvr22	P_u
**Acot3**	● (red)	✕	✕	✕	✕	✕	✕	● (red)	✕	✕	**0.0021**
**Arrdc2**	● (red)	✕	✕	✕	✕	✕	✕	✕	✕	● (red)	**0.0021**
**Asl**	● (red)	✕	✕	✕	✕	✕	✕	✕	✕	● (red)	**0.0021**
**Car2**	– (green)	● (red)	● (red)	● (red)	✕	● (red)	● (green)	✕	✕	● (green)	**0.0021**
**Cyp39a1**	● (red)	✕	✕	✕	✕	✕	✕	✕	✕	● (red)	**0.0021**
**Etnk2**	● (red)	✕	✕	✕	✕	✕	● (red)	✕	✕	✕	**0.0021**
**Ppp1r3g**	● (red)	✕	✕	✕	✕	✕	✕	✕	✕	● (red)	**0.0021**
**Prpsap1**	–	● (red)	● (red)	● (red)	✕	✕	✕	● (red)	✕	– (green)	**0.0021**
**Slc16a7**	● (red)	– (red)	–	● (green)	✕	● (red)	● (red)	✕	✕	–	**0.0021**
**Slco1a4**	● (red)	✕	✕	✕	✕	✕	✕	✕	✕	● (red)	**0.0021**
**Smad9**	● (red)	✕	✕	✕	✕	✕	✕	✕	✕	● (red)	**0.0021**
**Ttc23**	● (red)	✕	✕	✕	✕	✕	✕	● (red)	✕	✕	**0.0021**
**Slc5a6**	● (red)	● (red)	–	– (green)	✕	● (red)	✕	–	✕	✕	**0.00214**
**Rb1cc1**	● (red)	✕	✕	✕	✕	✕	✕	✕	✕	● (red)	**0.00218**
**Rere**	● (green)	● (red)	● (red)	● (red)	✕	● (red)	✕	– (red)	✕	✕	**0.00218**
**Lmo7**	● (red)	● (red)	–	● (green)	✕	● (red)	✕	✕	✕	✕	**0.0023**
**Imp3**	● (red)	● (red)	–	● (red)	✕	✕	✕	✕	✕	✕	**0.0023**
**Rcor2**	● (red)	✕	✕	✕	✕	✕	✕	● (red)	✕	✕	**0.0023**
**Paics**	● (red)	✕	✕	✕	✕	✕	✕	–	✕	● (red)	**0.00232**
**Rhoq**	● (red)	● (red)	–	–	✕	● (red)	✕	● (red)	✕	✕	**0.00234**

↓ CR

Genes down regulated by CR

	lvr1	lvr4a	lvr4b	lvr5	lvr10	lvr13	lvr14	lvr16	lvr20	lvr22	P_d
Lasp1	●	●	●	●	×	×	×	×	×	●	2.47e−05
Ube2d2	*	●	●	●	×	×	×	×	×	−	2.47e−05
Cyp2f2	−	●	●	●	●	●	×	−	●	−	2.47e−05
Ifi47	●	●	−	●	×	●	●	●	×	●	2.47e−05
Ifit3	●	●	−	●	×	×	●	×	×	●	2.47e−05
Iigp2	●	●	−	●	×	×	×	●	×	●	2.47e−05
Nudt1	●	●	●	●	×	×	×	×	×	●	2.47e−05
Sept9	●	●	●	●	×	×	×	−	×	●	2.47e−05
Hsd17b2	●	●	●	−	●	●	−	×	●	−	2.72e−05
Fzd8	●	●	—	●	×	×	×	×	×	●	3.72e−05
Ddc	●	●	●	●	×	●	×	×	×	−	3.96e−05
Pnkd	●	●	●	—	×	●	×	×	×	●	3.96e−05
2900086B20Rik	●	●	●	●	×	×	×	×	×	−	3.96e−05
Aqp8	●	●	—	●	×	×	−	×	×	●	3.96e−05
C730048C13Rik	●	−	●	●	●	●	×	×	●	×	3.96e−05
Tfpi2	●	●	●	—	×	×	●	×	×	●	3.96e−05
Srebf1	●	●	●	●	×	×	×	×	×	●	3.98e−05
Rhou	●	−	●	●	×	×	×	●	●	●	4.1e−05
Cyp2j5	*	●	●	●	×	×	−	×	●	●	4.21e−05
Ifit1	●	●	−	●	×	×	●	×	×	×	4.39e−05
Adipor2	●	●	●	−	×	●	×	×	×	●	4.81e−05
Uck1	●	●	●	—	×	●	—	×	×	●	5.7e−05
C9	●	●	−	●	●	●	−	×	●	●	5.72e−05
Igtp	●	●	−	●	×	×	×	●	×	●	5.72e−05
Gjb2	●	●	●	●	×	×	×	×	●	—	6.36e−05
Hsd3b7	●	—	—	●	×	●	×	●	×	●	6.36e−05
Car3	●	—	—	●	●	●	−	—	×	●	7.33e−05
Alas2	●	●	−	●	●	●	−	×	×	●	7.38e−05
Csrp3	●	●	●	●	×	●	×	×	×	−	7.38e−05
1110031B06Rik	●	●	−	●	×	●	×	−	×	×	7.56e−05

↓ CR

Genes down regulated by CR

	lvr1	lvr4a	lvr4b	lvr5	lvr10	lvr13	lvr14	lvr16	lvr20	lvr22	P_d
2410014A08Rik	●	●	–	●	×	●	×	×	×	×	8.64e−05
Rrbp1	●	●	●	●	×	●	×	●	×	–	8.64e−05
Keg1	– (green)	●	–	●	×	●	×	×	×	●	8.64e−05
Ela1	●	●	–	–	●	●	×	●	×	–	9.94e−05
Phlda1	●	●	●	–	×	●	×	×	×	●	0.000105
Pole4	●	●	●	●	×	×	×	–	×	×	0.000111
Tgm2	●	●	–	●	×	×	●	●	●	●	0.000112
Cyp7b1	●	–	– (red)	– (green)	●	●	×	●	●	●	0.000121
Mbl1	–	●	●	●	●	×	● (red)	×	●	●	0.000122
Cml1	●	●	●	–	●	●	×	×	×	●	0.000126
Pdcd4	●	–	–	●	×	×	●	×	×	●	0.000136
Dhrs3	●	–	●	●	×	●	– (green)	–	×	●	0.000138
Pdia4	●	●	–	●	×	×	×	●	×	×	0.000138
Anp32e	●	●	– (green)	●	×	×	×	–	×	●	0.000139
Hspa5	* (red)	●	–	●	●	×	×	–	●	●	0.000139
4930528F23Rik	●	×	×	×	×	×	×	●	×	●	0.000139
2310008M10Rik	●	– (green)	–	●	×	×	×	×	×	●	0.000142
Elovl6	●	●	●	●	×	×	×	● (red)	×	●	0.000163
Col15a1	●	●	●	●	×	×	–	–	×	×	0.000165
Serpina10	–	●	●	●	×	●	×	●	●	×	0.000165
Acly	●	●	–	●	×	×	×	● (red)	×	●	0.000165
1600012H06Rik	●	●	●	●	×	×	×	×	×	×	0.000169
G0s2	●	●	●	–	×	×	×	×	×	●	0.000171
Arpp19	●	–	–	●	×	×	×	●	×	●	0.000174
Nrp1	●	●	–	●	×	●	×	–	×	●	0.000177
Gadd45a	●	●	–	●	×	×	×	×	×	●	0.000193
Creld2	●	●	–	●	●	●	×	×	×	–	0.000207
Mpv17	–	●	– (green)	●	×	×	●	×	×	●	0.000216
S100a10	●	– (green)	–	●	●	●	×	–	×	–	0.000229
Serpina12	●	–	– (red)	●	×	●	×	–	×	●	0.000238

↓ CR

Genes down regulated by CR

	lvr1	lvr4a	lvr4b	lvr5	lvr10	lvr13	lvr14	lvr16	lvr20	lvr22	P_d
Fermt2	green ●	−	−	green ●	×	green ●	×	green ●	×	×	0.000241
Igfals	−	green ●	green ●	−	×	green ●	−	green ●	×	green ●	0.000248
Cxcl1	green ●	−	−	green ●	green ●	×	−	×	×	green ●	0.000249
Dhrs7b	green ●	green ●	−	green ●	×	×	×	×	×	green ●	0.000269
Ly6a	green ●	−	−	green ●	green ●	×	×	green ●	×	green ●	0.000269
Smarca4	green ●	green ●	green −	green ●	×	×	×	−	×	green ●	0.000295
St3gal1	green ●	green ●	green ●	green ●	×	×	×	×	×	red ●	0.000295
Tle3	green ●	green ●	−	green ●	×	×	×	×	×	green ●	0.000295
2200001I15Rik	green ●	×	×	×	×	×	×	green ●	×	green ●	0.000295
Aox3	green ●	×	×	×	×	×	×	green ●	×	green ●	0.000295
Lgals3bp	green ●	−	−	green ●	×	green ●	×	green ●	×	green ●	0.000295
Sh3bp2	green ●	green ●	−	green ●	×	×	×	−	×	green ●	0.000295
Tmem19	green ●	×	×	×	×	×	×	green ●	×	green ●	0.000296
Irf5	green ●	green ●	−	−	×	green ●	×	green ●	×	green ●	0.000296
Irgm1	green ●	green −	−	green ●	×	×	×	×	×	green ●	0.000316
Snrpa	green ●	green ●	−	green ●	×	×	×	×	×	green ●	0.000316
Psmb9	green ●	green ●	−	green ●	×	×	−	green ●	×	green ●	0.000324
Zfp706	green ●	green −	green ●	green ●	×	×	×	×	×	×	0.000329
Sdf2l1	green ●	×	×	×	×	×	×	green ●	×	green ●	0.000329
Samhd1	green ●	green −	−	green ●	×	×	×	×	×	green ●	0.00033
Ttc30b	green ●	green ●	−	green ●	×	green ●	×	×	×	×	0.000377
Ccng1	green ●	−	−	green ●	×	×	×	×	×	green ●	0.000405
Jarid1b	green ●	green ●	red ●	green ●	×	×	×	green ●	×	×	0.000405
Dmap1	green ●	×	×	×	×	×	×	green ●	×	green ●	0.000405
Nit2	green −	green −	green ●	green ●	×	×	×	×	×	green ●	0.000405
Mtif2	green ●	green ●	green ●	green ●	×	×	×	−	×	green −	0.000462
Stat1	green ●	−	−	green ●	×	×	×	×	×	green ●	0.000482
Idi1	green ●	−	−	green ●	green ●	×	×	red ●	×	green ●	0.000496
G3bp2	green ●	green ●	−	green ●	×	×	×	×	×	−	0.000519
Lifr	green ●	green ●	green −	green ●	green ●	green ●	×	×	×	−	0.000519

↓ CR

Genes down regulated by CR

	lvr1	lvr4a	lvr4b	lvr5	lvr10	lvr13	lvr14	lvr16	lvr20	lvr22	P_d
Pdia3	●	●	−	●	×	×	●	−	●	×	0.000519
Siva1	●	−	●	●	×	×	×	×	×	●	0.000519
Cdcp1	●	●	●	−	×	×	×	×	×	×	0.000519
Cyp4a12a	●	−	−	●	●	●	×	×	×	×	0.000519
Lipc	−	●	●	●	×	×	●	−	●	−	0.000519
Nthl1	●	●	−	−	×	×	●	−	×	−	0.000519
Ube2d3	*	−	−	●	×	×	×	−	×	●	0.000527
St5	●	●	−	−	×	●	×	×	×	×	0.000539
Cldn2	●	●	●	−	×	×	×	●	×	●	0.000545
Lgals8	●	●	●	●	×	×	×	×	×	×	0.00062
Zc3hav1	●	×	×	×	×	×	×	●	×	●	0.00062
Dio1	−	●	−	●	●	●	×	×	×	×	0.00062
Antxr2	●	●	−	●	×	×	×	×	×	●	0.000659
Med28	●	●	−	●	×	×	×	●	×	×	0.00066
Sec62	*	−	−	●	×	●	×	×	×	×	0.00066
Tsc22d1	●	●	−	●	●	×	×	●	×	×	0.000668
Atg5	●	●	●	●	×	×	×	×	×	×	0.000674
Ide	*	●	●	*	×	×	×	×	×	−	0.000674
Frag1	●	●	−	●	×	×	×	×	×	●	0.000676
Bcl3	●	−	−	●	×	×	×	●	×	●	0.000687
Cyp2c70	●	●	−	−	×	×	×	●	×	×	0.000699
Tmed9	●	●	●	●	×	×	×	×	×	●	0.000729
3010026O09Rik	●	×	×	×	×	×	×	●	×	●	0.000769
Sppl3	●	●	●	−	×	×	×	●	×	×	0.000792
5033414D02Rik	●	−	−	●	×	×	×	×	×	●	0.000808
Timd2	●	●	●	●	×	●	×	×	×	×	0.000828
Adrb3	●	●	−	●	×	●	×	−	×	×	0.000838
Cited2	●	●	−	●	×	×	×	×	×	−	0.000854
Mt2	*	●	●	●	×	×	×	●	×	×	0.000857
Fasn	●	●	−	−	●	×	×	×	×	●	0.000857

↓ CR

Genes down regulated by CR

	lvr1	lvr4a	lvr4b	lvr5	lvr10	lvr13	lvr14	lvr16	lvr20	lvr22	P_d
Mfsd1	* (red)	●	●	●	×	×	×	●	×	×	0.000872
Centd2	●	×	×	×	×	×	×	●	×	●	0.000899
Mtmr9	●	●	●	●	×	×	×	–	×	–	0.00094
Hus1	●	●	–	–	×	×	●	×	×	–	0.000946
Rpn1	●	●	–	●	×	×	×	×	×	●	0.000946
Cmas	●	●	–	●	×	×	×	●	×	–	0.000946
LOC100048541	●	●	–	●	×	×	×	×	×	×	0.000946
Pafah1b1	●	●	–	●	×	×	×	×	×	×	0.000969
Ccnf	●	– (green)	–	– (red)	×	●	×	×	×	●	0.000985
Acad9	●	–	–	●	×	×	×	×	×	●	0.000988
Anapc11	●	●	–	●	×	×	×	×	×	– (green)	0.000988
Anp32a	●	●	●	●	×	×	×	×	×	–	0.000988
Arhgdia	●	●	●	– (green)	×	×	×	×	×	×	0.000988
Ergic1	* (green)	●	●	–	×	×	×	–	×	×	0.000988
Lyrm2	●	●	–	●	×	×	×	● (red)	×	×	0.000988
St6gal1	●	–	●	●	×	×	×	●	×	×	0.000988
2700029M09Rik	●	– (green)	–	●	×	×	×	–	×	●	0.000988
Cwf19l1	●	●	–	●	×	×	×	×	×	– (green)	0.00101
Elovl5	●	●	●	–	×	×	×	● (red)	×	●	0.00106
Krt8	●	–	–	●	×	×	●	×	×	–	0.00106
Pter	●	–	– (green)	●	×	×	×	–	×	●	0.00106
Copz1	–	●	– (green)	●	×	×	×	×	×	●	0.00106
Ipo11	●	●	–	●	×	×	×	×	×	×	0.00117
Sec23b	●	●	–	●	×	×	×	×	×	×	0.00117
Atp11a	●	●	–	●	×	●	×	×	×	●	0.00125
Gck	●	●	–	–	●	×	×	● (red)	×	●	0.00125
Ssr1	●	●	●	●	×	×	×	×	×	●	0.00125
Ints3	●	●	–	●	×	×	×	–	×	–	0.00127
Aox1	●	–	●	●	×	×	×	×	×	●	0.00129
Ndst1	●	●	●	–	×	×	×	×	×	●	0.00133

↓ CR

Genes down regulated by CR

	lvr1	lvr4a	lvr4b	lvr5	lvr10	lvr13	lvr14	lvr16	lvr20	lvr22	P_d
1300018I05Rik	●	–	–	●	×	×	×	●	×	×	0.00138
Pctp	● (red)	●	●	●	×	×	×	● (red)	×	●	0.00138
Whsc2	●	– (green)	–	–	×	●	×	●	×	●	0.00138
Casp3	●	–	–	●	×	×	●	×	×	●	0.00139
Fpgs	●	●	–	–	●	●	×	×	×	–	0.00139
Mapre1	* (green)	●	● (red)	●	×	×	×	●	×	–	0.00142
Jarid2	●	●	–	●	×	×	×	×	×	●	0.00144
Mdfic	●	●	–	●	×	×	●	–	×	–	0.00149
Eif1a	●	–	–	●	×	×	●	●	×	×	0.0015
Lrig1	●	×	×	×	×	×	×	●	×	– (green)	0.0015
Crebl1	●	●	– (green)	●	×	●	×	– (green)	×	–	0.00151
1700007B13Rik	●	×	×	×	×	×	×	●	×	– (green)	0.00153
2310079N02Rik	●	–	–	●	×	● (red)	×	–	×	●	0.00153
5730494M16Rik	●	–	●	●	×	×	×	×	×	×	0.00153
Ccnd1	●	● (red)	–	●	×	×	×	×	×	●	0.00153
M6pr	– (green)	–	–	●	×	×	×	●	×	●	0.00153
Zfr	●	●	●	●	×	×	×	×	×	– (red)	0.00153
Slc35b1	●	– (green)	–	●	×	●	×	– (green)	×	●	0.00156
Mm.352037	●	●	–	●	×	×	×	×	×	×	0.00156
Pik3r1	● (red)	●	●	●	×	● (red)	×	×	×	●	0.00158
Mterfd3	●	●	–	●	×	×	×	×	×	×	0.00159
Acbd4	●	×	×	×	×	×	×	●	×	×	0.00165
Anapc10	●	×	×	×	×	×	×	×	×	●	0.00165
Ap2a2	●	– (green)	●	–	×	×	–	●	×	● (red)	0.00165
Arsg	●	×	×	×	×	×	×	●	×	×	0.00165
Bcar1	●	–	–	– (red)	×	×	×	●	×	●	0.00165
Bet1	●	– (green)	–	●	×	×	×	×	×	●	0.00165
Col5a3	●	×	×	×	×	×	×	×	×	●	0.00165
Commd10	●	×	×	×	×	×	×	● (red)	×	●	0.00165
Cxcl11	●	×	×	×	×	×	×	×	×	●	0.00165

↓ CR

Genes down regulated by CR

	lvr1	lvr4a	lvr4b	lvr5	lvr10	lvr13	lvr14	lvr16	lvr20	lvr22	P_d
Cyp2u1	*	×	×	×	×	×	×	×	×	●	0.00165
D430042O09Rik	●	×	×	×	×	×	×	●	×	×	0.00165
D5Wsu178e	●	×	×	×	×	×	×	–	×	●	0.00165
Dnajc1	●	●	–	●	×	×	×	×	×	●	0.00165
Dot1l	●	×	×	×	×	×	●	–	×	×	0.00165
Dut	●	–	●	–	×	×	×	●	×	●	0.00165
Esd	●	–	–	–	×	×	×	×	×	●	0.00165
Exoc4	●	×	×	×	×	×	×	–	×	●	0.00165
Fzd7	●	–	–	–	×	×	×	×	×	●	0.00165
Hes6	●	●	●	● (red)	×	×	×	×	×	●	0.00165
Hspa4l	●	●	–	● (red)	×	●	×	–	×	–	0.00165
Igh−6	*	●	–	● (red)	×	●	×	×	×	● (red)	0.00165
Kynu	●	×	×	×	×	×	×	●	×	×	0.00165
Ldlr	●	–	–	●	×	×	×	● (red)	×	●	0.00165
Mmab	●	×	×	×	×	×	×	×	×	●	0.00165
Mmp15	●	–	–	–	×	●	●	×	×	●	0.00165
Mmp19	●	×	×	×	×	×	×	–	×	●	0.00165
Mpdz	●	● (red)	–	–	×	×	×	–	×	●	0.00165
Nans	●	–	–	–	×	×	×	–	×	●	0.00165
Nmral1	●	×	×	×	×	×	×	●	×	×	0.00165

↑ CR

Overrepresented Biological Processes

GO Term	P−Value
translation	7.61e−11
cofactor metabolic process	3.25e−06
tricarboxylic acid cycle	1.24e−05
coenzyme catabolic process	6.41e−05
acetyl−CoA metabolic process	8.05e−05
generation of precursor metabolites and energy	8.98e−05
cellular respiration	1e−04
acyl−CoA metabolic process	0.000167
biosynthetic process	0.000512
respiratory burst	0.000774
oxidation reduction	0.00102
gluconeogenesis	0.00201
negative regulation of hydrolase activity	0.00222
sulfur compound catabolic process	0.00228
electron transport chain	0.00243
catabolic process	0.00275
ATP synthesis coupled electron transport	0.00391
mitochondrial electron transport, ubiquinol to cytochrome c	0.00447
synaptic growth at neuromuscular junction	0.00447
circadian rhythm	0.00471
organic acid metabolic process	0.0048
monosaccharide biosynthetic process	0.00544
mitochondrion organization and biogenesis	0.00594
glycolysis	0.00657
monocarboxylic acid metabolic process	0.0075
negative regulation of transcription factor activity	0.0108
protein import into mitochondrial inner membrane	0.0108
hexose catabolic process	0.0134
protein targeting to mitochondrion	0.0148
post−embryonic organ development	0.0148

↑ CR

Overrepresented Biological Processes

GO Term	P−Value
T cell homeostasis	0.0149
negative regulation of myeloid cell differentiation	0.0149
cellular macromolecule metabolic process	0.0167
segment specification	0.0194
alcohol catabolic process	0.0195
leukocyte homeostasis	0.0207
transmembrane receptor protein tyrosine kinase signaling pathway	0.0214
carboxylic acid catabolic process	0.0223
chromatin remodeling	0.0226
MAPKKK cascade	0.0235
lipid metabolic process	0.0241
cell migration	0.0245
stem cell division	0.0245
regulation of epidermal growth factor receptor signaling pathway	0.0245
macrophage activation	0.0245
alcohol metabolic process	0.025
pteridine and derivative biosynthetic process	0.03
regulation of fat cell differentiation	0.03
negative regulation of binding	0.03
cellular metabolic process	0.0305
localization of cell	0.0307
triacylglycerol metabolic process	0.0313
lipid homeostasis	0.0313
cellular carbohydrate catabolic process	0.0326
nucleotide metabolic process	0.0338
transmembrane transport	0.0347
axon cargo transport	0.036
response to dsRNA	0.036
in utero embryonic development	0.0362
positive regulation of MAP kinase activity	0.0386

↑ CR

Overrepresented Biological Processes

GO Term	P−Value
primary metabolic process	0.0396
regulation of cell size	0.04
hexose metabolic process	0.0412
cerebellar cortex formation	0.0425
neutrophil chemotaxis	0.0425
neuron maturation	0.0425
cellular lipid catabolic process	0.0441
protein metabolic process	0.0453
liver development	0.0459
regulation of tissue remodeling	0.0459
nucleobase, nucleoside and nucleotide metabolic process	0.0469
long−chain fatty acid metabolic process	0.0493
cell differentiation in hindbrain	0.0493
regulation of RNA stability	0.0493
dicarboxylic acid metabolic process	0.0493
neutral lipid metabolic process	0.05
regulation of inflammatory response	0.05

↑ CR

Overrepresented Cell Components

GO Term	P−Value
ribosome	1.21e−09
intracellular	1.44e−07
mitochondrial envelope	4.25e−07
envelope	1.32e−06
organelle membrane	2.43e−06
microsome	5.3e−05
intracellular organelle	5.63e−05
membrane fraction	0.000143
cytoplasm	0.000397
mitochondrial inner membrane	0.000486
cell fraction	0.000669
mitochondrion	0.000765
small ribosomal subunit	0.00197
intracellular organelle part	0.00399
mitochondrial respiratory chain	0.00872
mitochondrial intermembrane space protein transporter complex	0.00876
trans−Golgi network transport vesicle membrane	0.0121
organelle envelope lumen	0.019
cytoplasmic part	0.0211
intrinsic to endoplasmic reticulum membrane	0.0238
nuclear speck	0.0246
cell	0.025
Golgi−associated vesicle	0.0264
intracellular membrane−bounded organelle	0.0278
cytosolic ribosome	0.0349
coated pit	0.0351
nuclear envelope−endoplasmic reticulum network	0.0359
transport vesicle	0.0406
non−membrane−bounded organelle	0.0487

↑ CR

Overrepresented Molecular Functions

GO Term	P−Value
structural constituent of ribosome	9.68e−13
monooxygenase activity	1.38e−05
electron carrier activity	7.62e−05
rRNA binding	7.66e−05
flavin−containing monooxygenase activity	0.000206
heme binding	0.000334
iron ion binding	0.000339
oxidoreductase activity, acting on paired donors, with incorporation or reduction of molecular oxygen	0.000544
aconitate hydratase activity	0.000774
tyrosine−ester sulfotransferase activity	0.000774
alkane 1−monooxygenase activity	0.000774
carbohydrate kinase activity	0.00165
unspecific monooxygenase activity	0.00177
insulin−like growth factor binding	0.00201
dodecenoyl−CoA delta−isomerase activity	0.00228
carbon−oxygen lyase activity	0.0028
aryl sulfotransferase activity	0.00447
phosphoric ester hydrolase activity	0.00463
catalytic activity	0.0058
NADP binding	0.00621
3−hydroxyacyl−CoA dehydrogenase activity	0.00732
carboxylesterase activity	0.0078
cofactor binding	0.0103
steroid hormone receptor activity	0.0105
carbon−nitrogen lyase activity	0.0148
4 iron, 4 sulfur cluster binding	0.0149
intramolecular oxidoreductase activity	0.0173
ubiquinol−cytochrome−c reductase activity	0.0194
oxidoreductase activity, acting on diphenols and related substances as donors	0.0194
glutathione transferase activity	0.0223

↑ CR

Overrepresented Molecular Functions

GO Term	P−Value
phosphatidate phosphatase activity	0.0245
protein kinase activator activity	0.0245
oxidoreductase activity, acting on CH−OH group of donors	0.0261
palmitoyl−CoA hydrolase activity	0.03
symporter activity	0.0326
metal cluster binding	0.036
FMN binding	0.0361
RNA polymerase II transcription factor activity, enhancer binding	0.0383
active transmembrane transporter activity	0.0398
two−component sensor activity	0.0459

↓CR

Overrepresented Biological Processes

GO Term	P−Value
cholesterol biosynthetic process	0.000135
intracellular protein transport	0.000163
alcohol metabolic process	0.000277
activation of plasma proteins during acute inflammatory response	0.000651
response to unfolded protein	0.00081
establishment of protein localization	0.000833
complement activation, alternative pathway	0.00129
complement activation, classical pathway	0.00164
response to biotic stimulus	0.00264
calcium−independent cell−cell adhesion	0.00265
macromolecule localization	0.0028
fatty acid elongation	0.00282
CMP−N−acetylneuraminate metabolic process	0.00282
regulation of cell cycle	0.00289
establishment of localization in cell	0.00305
activation of immune response	0.00335
B cell mediated immunity	0.00413
humoral immune response mediated by circulating immunoglobulin	0.00436
response to virus	0.00539
cellular lipid metabolic process	0.0062
response to exogenous dsRNA	0.00686
amino sugar metabolic process	0.00766
meiotic spindle organization and biogenesis	0.00817
re−entry into mitotic cell cycle	0.00817
base conversion or substitution editing	0.00817
ISG15−protein conjugation	0.00817
homologous chromosome segregation	0.00817
regulation of transforming growth factor beta receptor signaling pathway	0.00864
cholesterol metabolic process	0.00972
ER to Golgi vesicle−mediated transport	0.0108

↓ CR

Overrepresented Biological Processes

GO Term	P−Value
protein catabolic process	0.0119
positive regulation of MAPKKK cascade	0.0128
lipid biosynthetic process	0.0132
adaptive immune response	0.0148
mRNA modification	0.0158
response to lipopolysaccharide	0.0194
B cell receptor signaling pathway	0.023
macromolecule catabolic process	0.0237
regulation of systemic arterial blood pressure by norepinephrine−epinephrine	0.0254
pyrimidine base metabolic process	0.0254
methylation−dependent chromatin silencing	0.0254
deoxyribonucleoside metabolic process	0.0254
benzene and derivative metabolic process	0.0254
positive regulation of chemokine biosynthetic process	0.0254
decidualization	0.0254
one−carbon compound metabolic process	0.0257
steroid biosynthetic process	0.0265
response to DNA damage stimulus	0.0267
cellular macromolecular complex assembly	0.0274
immune effector process	0.0275
oxidation reduction	0.0284
primary metabolic process	0.0286
epithelial cell development	0.0287
positive regulation of immune system process	0.0302
cell death	0.0323
DNA metabolic process	0.0341
glycerol−3−phosphate metabolic process	0.0367
regulation of mitotic metaphase/anaphase transition	0.0367
heat generation	0.0367
regulation of Rho GTPase activity	0.0367

↓ CR

Overrepresented Biological Processes

GO Term	P−Value
neurotransmitter receptor metabolic process	0.0367
focal adhesion formation	0.0367
spindle assembly	0.0367
embryonic process involved in female pregnancy	0.0367
metabolic process	0.0377
cellular macromolecule catabolic process	0.0394
protein import into nucleus, docking	0.0422
isoprenoid biosynthetic process	0.0422
spleen development	0.0422
microtubule cytoskeleton organization and biogenesis	0.0451
regulation of B cell proliferation	0.0467
apoptosis	0.0474
defense response	0.0474
multi−organism process	0.0479
macromolecular complex subunit organization	0.0481
protein complex assembly	0.0484
aromatic compound metabolic process	0.0487
sterol biosynthetic process	0.0488
myoblast fusion	0.0496
induction of apoptosis via death domain receptors	0.0496
type I interferon biosynthetic process	0.0496
chemokine metabolic process	0.0496
pyrimidine nucleotide biosynthetic process	0.05

↓ CR

Overrepresented Cell Components

GO Term	P−Value
endoplasmic reticulum	1.58e−12
cytoplasm	2.28e−10
organelle	1.61e−05
soluble fraction	0.000329
intracellular membrane−bounded organelle	0.000776
endoplasmic reticulum membrane	0.00102
membrane attack complex	0.00135
integral to endoplasmic reticulum membrane	0.00252
Golgi membrane	0.00372
ubiquitin ligase complex	0.00676
glycerol−3−phosphate dehydrogenase complex	0.00804
replisome	0.00965
nuclear replication fork	0.00965
cytosol	0.0136
perinuclear region of cytoplasm	0.0155
lamin filament	0.0155
alpha DNA polymerase:primase complex	0.0155
stress fiber	0.0158
microsome	0.0168
cell−substrate adherens junction	0.0218
exosome (RNase complex)	0.0225
cullin−RING ubiquitin ligase complex	0.0225
cell	0.0235
Golgi apparatus	0.0283
intrinsic to organelle membrane	0.0348
early endosome	0.0351
intracellular part	0.0393
endosome	0.0403
anaphase−promoting complex	0.0489

↓ CR

Overrepresented Molecular Functions

GO Term	P−Value
catalytic activity	0.00162
actin filament binding	0.00169
lyase activity	0.00187
left−handed Z−DNA binding	0.00263
thymidylate kinase activity	0.00263
hydrolase activity, acting on acid anhydrides, in phosphorus−containing anhydrides	0.00295
oxidoreductase activity, acting on paired donors, with incorporation or reduction of molecular oxygen	0.00386
hydrolase activity	0.00581
RNA binding	0.00611
small protein activating enzyme activity	0.00619
molybdenum ion binding	0.00619
carboxy−lyase activity	0.00658
double−stranded RNA adenosine deaminase activity	0.00761
DNA (cytosine−5−)−methyltransferase activity	0.00761
aldehyde oxidase activity	0.00761
3−beta−hydroxy−delta5−steroid dehydrogenase activity	0.00894
steroid dehydrogenase activity	0.00936
monooxygenase activity	0.0122
binding	0.0123
ubiquitin−protein ligase activity	0.0124
pyridoxal phosphate binding	0.0132
inositol or phosphatidylinositol kinase activity	0.0149
nucleoside−triphosphatase activity	0.0186
hydro−lyase activity	0.0193
acid−amino acid ligase activity	0.0201
deaminase activity	0.0204
unspecific monooxygenase activity	0.0223
inositol−polyphosphate 5−phosphatase activity	0.0237
chaperone regulator activity	0.0237
ribosome binding	0.0237

↓ CR

Overrepresented Molecular Functions

GO Term	P−Value
transferase activity, transferring acyl groups, acyl groups converted into alkyl on transfer	0.0237
unfolded protein binding	0.0258
electron carrier activity	0.0271
CDP−alcohol phosphatidyltransferase activity	0.0343
beta−1,3−galactosyltransferase activity	0.0343
chaperone binding	0.0343
intramolecular oxidoreductase activity	0.0353
protein transporter activity	0.0358
isomerase activity	0.0372
acyl−CoA dehydrogenase activity	0.0385
SNAP receptor activity	0.0385
oxidoreductase activity, acting on the CH−CH group of donors, NAD or NADP as acceptor	0.0385
protein disulfide isomerase activity	0.0464
nucleotide diphosphatase activity	0.0464
dolichyl−diphosphooligosaccharide−protein glycotransferase activity	0.0464
pheromone binding	0.0464
transferase activity	0.047
oxidoreductase activity, acting on the CH−OH group of donors, NAD or NADP as acceptor	0.0487

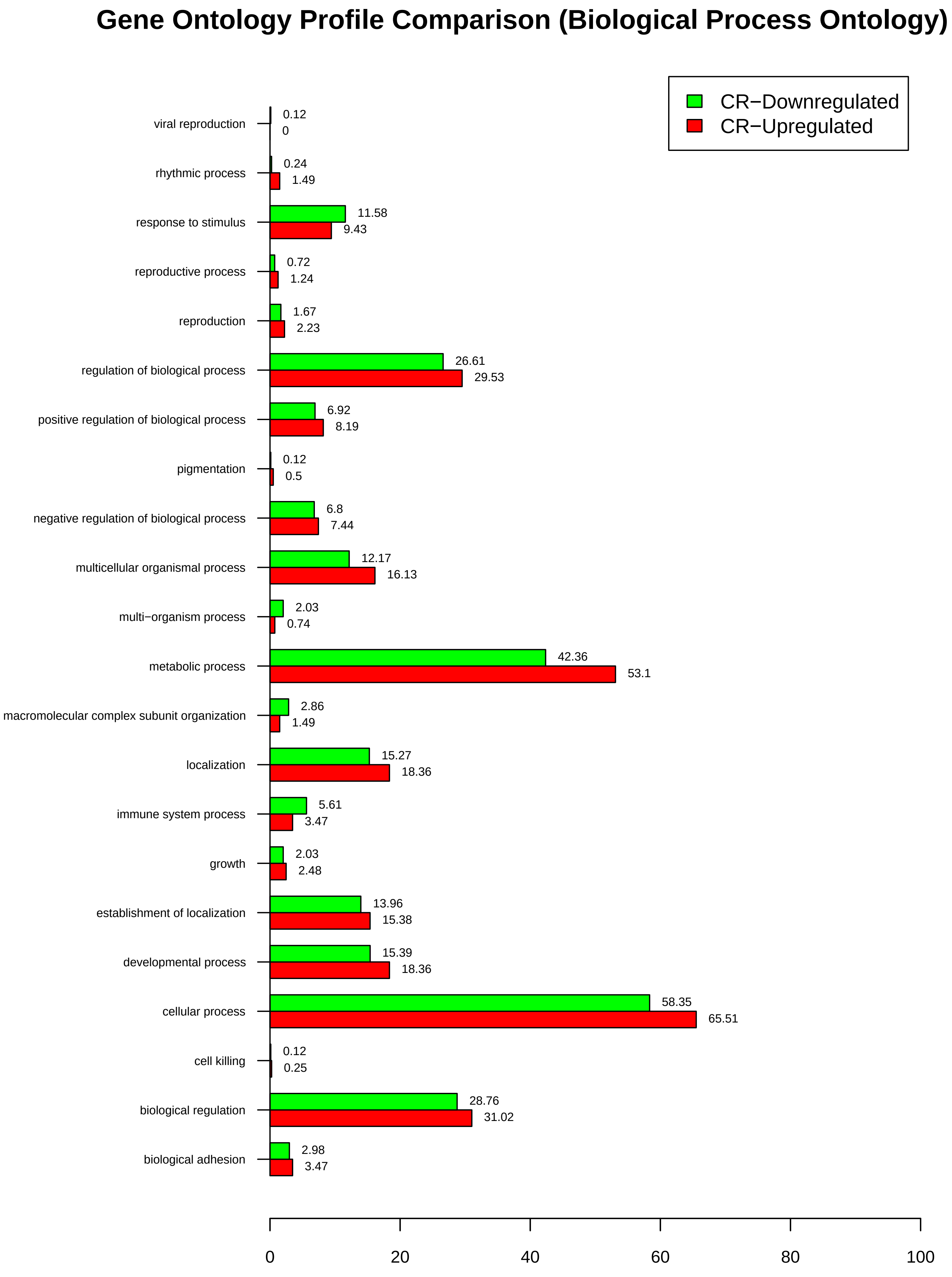
Gene Ontology Profile Comparison (Biological Process Ontology)
CR−Downregulated
CR−Upregulated
viral reproduction
0.12
0
rhythmic process
0.24
1.49
response to stimulus
11.58
9.43
reproductive process
0.72
1.24
reproduction
1.67
2.23
regulation of biological process
26.61
29.53
positive regulation of biological process
6.92
8.19
pigmentation
0.12
0.5
negative regulation of biological process
6.8
7.44
multicellular organismal process
12.17
16.13
multi−organism process
2.03
0.74
metabolic process
42.36
53.1
macromolecular complex subunit organization
2.86
1.49
localization
15.27
18.36
immune system process
5.61
3.47
growth
2.03
2.48
establishment of localization
13.96
15.38
developmental process
15.39
18.36
cellular process
58.35
65.51
cell killing
0.12
0.25
biological regulation
28.76
31.02
biological adhesion
2.98
3.47
0
20
40
60
80
100

Gene Ontology Profile Comparison (Cell Component Ontology)

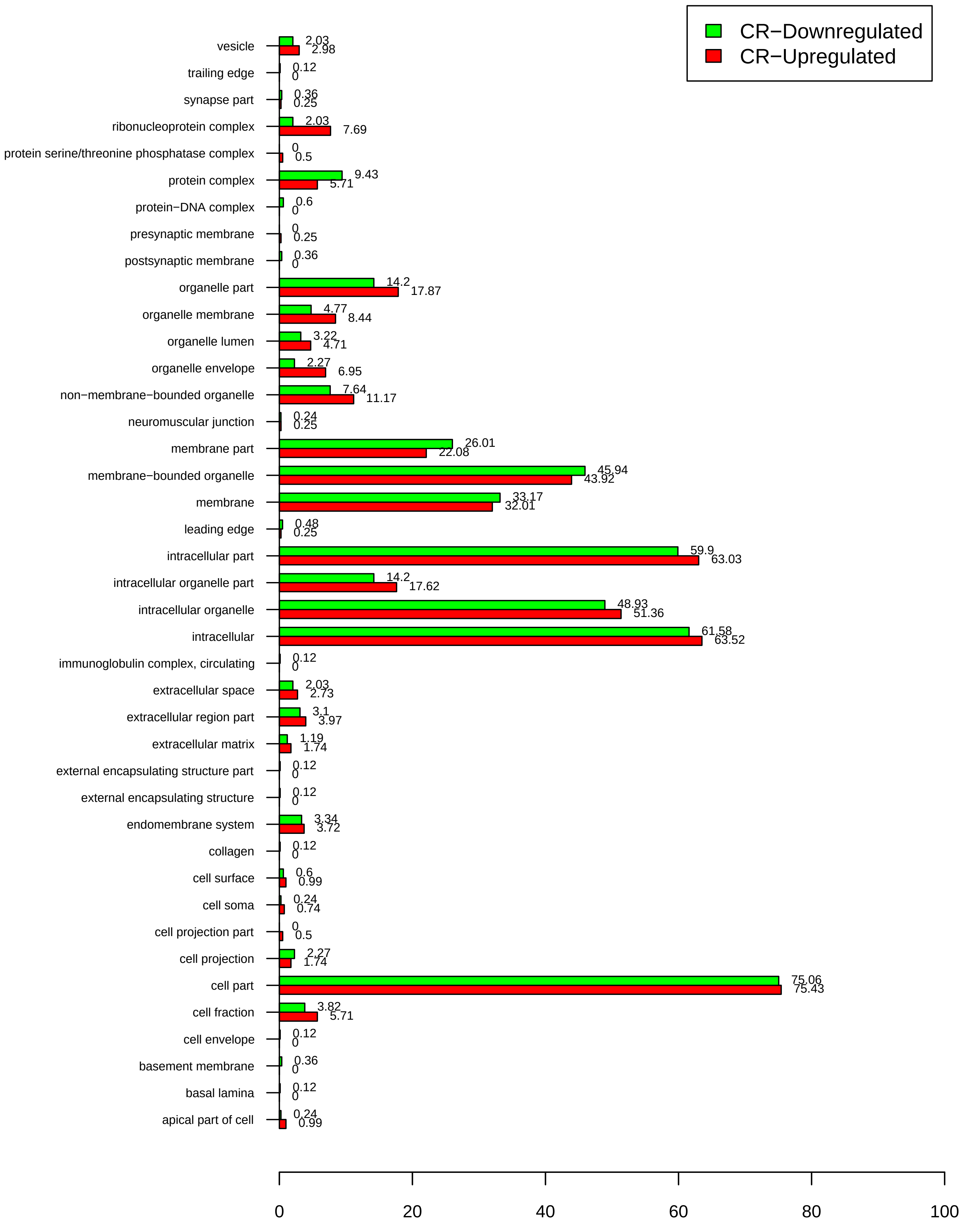

Gene Ontology Profile Comparison (Molecular Function Ontology)

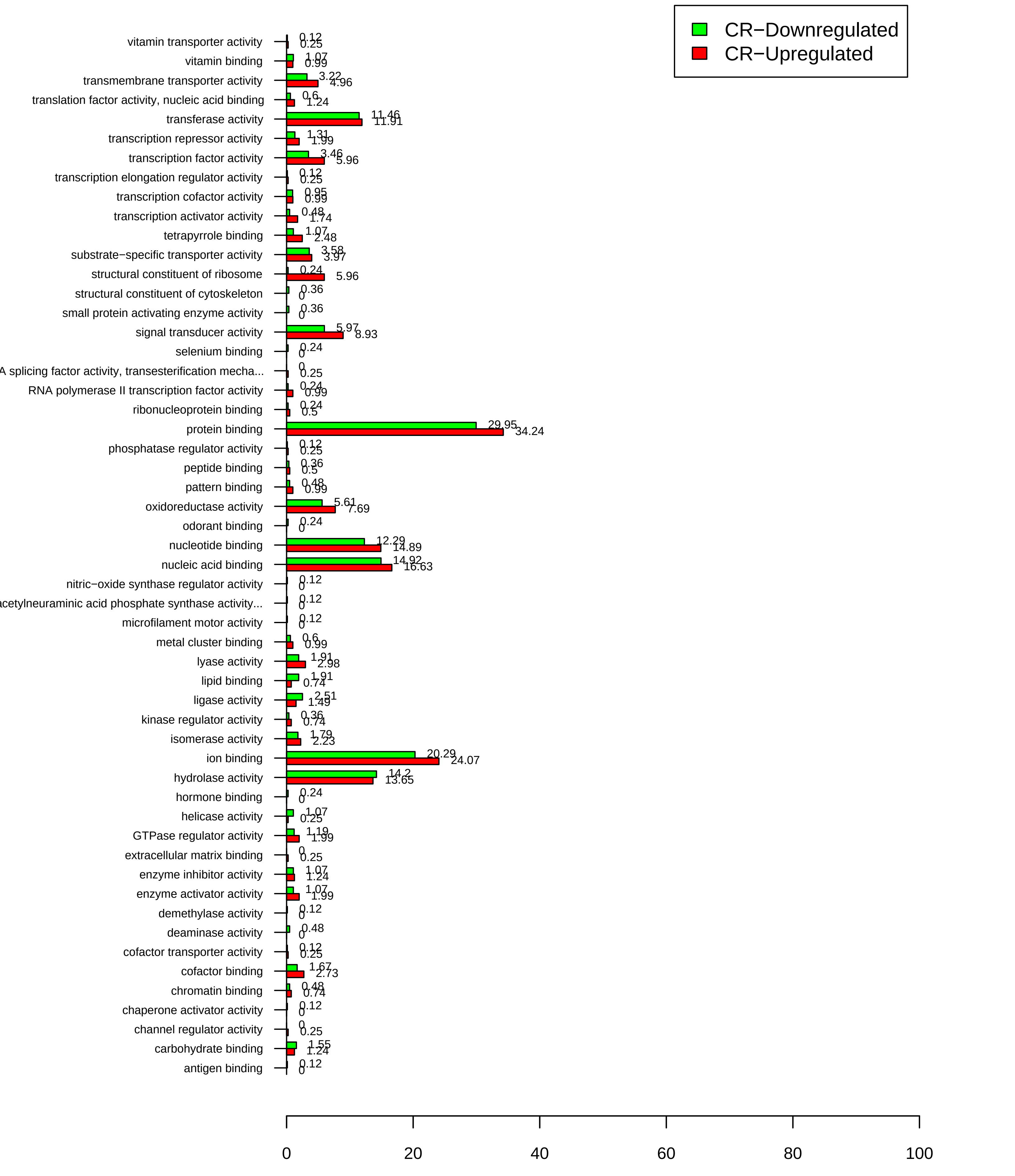

↑ CR

Overrepresented KEGG Pathways

GO Term	P−Value
Ribosome	4.24e−15
Reductive carboxylate cycle (CO2 fixation)	3.08e−05
Citrate cycle (TCA cycle)	3.95e−05
Fatty acid metabolism	0.000963
Metabolism of xenobiotics by cytochrome P450	0.00191
Arachidonic acid metabolism	0.00424
Circadian rhythm	0.0121
Glyoxylate and dicarboxylate metabolism	0.015
Nicotinate and nicotinamide metabolism	0.0397

↓ CR

Overrepresented KEGG Pathways

GO Term	P−Value
Biosynthesis of steroids	7.45e−07
SNARE interactions in vesicular transport	0.00135
Toll−like receptor signaling pathway	0.00143
Biosynthesis of unsaturated fatty acids	0.00397
Cell cycle	0.0112
Ubiquitin mediated proteolysis	0.0113
Aminosugars metabolism	0.0124
Apoptosis	0.017
Pancreatic cancer	0.0194
Colorectal cancer	0.0218
Pyrimidine metabolism	0.0237
p53 signaling pathway	0.0241
Atrazine degradation	0.0241
Regulation of autophagy	0.0259
Terpenoid biosynthesis	0.0384
Huntington's disease	0.0397
Phosphatidylinositol signaling system	0.0435
Antigen processing and presentation	0.047

↑ CR

Overrepresented KEGG Pathways

(Based on InterPro Domain Signatures)

GO Term	P−Value
Ribosome	1e−04
Glyoxylate and dicarboxylate metabolism	0.0017
Reductive carboxylate cycle (CO2 fixation)	0.0019
gamma−Hexachlorocyclohexane degradation	0.0058
Fructose and mannose metabolism	0.0068
Cysteine metabolism	0.0074
Linoleic acid metabolism	0.015
Citrate cycle (TCA cycle)	0.0167
Drug metabolism − cytochrome P450	0.0182
Riboflavin metabolism	0.0185
Carbon fixation	0.0285
Porphyrin and chlorophyll metabolism	0.0304
Arachidonic acid metabolism	0.0329
Neurodegenerative Diseases	0.0442
Galactose metabolism	0.0457
Retinol metabolism	0.049

↓ CR

Overrepresented KEGG Pathways

(Based on InterPro Domain Signatures)

GO Term	P−Value
Biosynthesis of steroids	1e−04
Toll−like receptor signaling pathway	2e−04
Ribosome	0.0013
Vitamin B6 metabolism	0.0034
SNARE interactions in vesicular transport	0.0037
Drug metabolism − other enzymes	0.0043
Tryptophan metabolism	0.0155
Biosynthesis of unsaturated fatty acids	0.0163
Antigen processing and presentation	0.0163
Methionine metabolism	0.0168
Apoptosis	0.026
Geraniol degradation	0.0303
Alzheimer's disease	0.0377
C21−Steroid hormone metabolism	0.0379
Regulation of autophagy	0.0425

↑ CR

Abundance of miRNA Targets

miRNA	Freq(Obs)	Freq(Exp)	Obs/Exp	P−value	P−Value(Adj)
miR−615−3p	0.0518	0.032	1.62	0.0222	1
miR−127	0.0453	0.0272	1.66	0.023	1
miR−384−3p	0.146	0.112	1.3	0.0264	1
miR−99b	0.0227	0.013	1.75	0.0494	1
miR−574−3p	0.0583	0.0423	1.38	0.0669	1
miR−764−5p	0.0906	0.0706	1.28	0.0726	1
miR−652	0.0324	0.0228	1.42	0.0982	1
miR−504	0.0939	0.0765	1.23	0.107	1
miR−671−3p	0.0421	0.0316	1.33	0.115	1
miR−375	0.0324	0.0237	1.37	0.118	1

↓ CR

Abundance of miRNA Targets

miRNA	Freq(Obs)	Freq(Exp)	Obs/Exp	P−value	P−Value(Adj)
miR−504	0.101	0.0765	1.32	0.00997	1
miR−345−5p	0.082	0.0638	1.29	0.0271	1
miR−714	0.0379	0.0263	1.44	0.0308	1
miR−339−3p	0.0426	0.0313	1.36	0.0442	1
miR−296−5p	0.0615	0.0498	1.23	0.0757	1
miR−148b	0.147	0.129	1.14	0.0768	1
miR−877	0.125	0.108	1.16	0.0771	1
miR−540−5p	0.0994	0.086	1.16	0.101	1
miR−293	0.0331	0.0266	1.24	0.127	1
miR−546	0.082	0.0715	1.15	0.133	1

Tests for Chromosome Over−representation

Chromosome	CR−upregulated Genes	CR−downregulated Genes
1	0.37	0.896
2	0.422	0.0588
3	0.778	0.0378*
4	0.267	0.539
5	0.0102*	0.867
6	0.989	0.679
7	0.335	0.855
8	0.487	0.184
9	0.261	0.85
10	0.0671	0.793
11	0.888	0.00218**
12	0.021*	0.721
13	0.876	0.34
14	0.982	0.592
15	0.143	0.21
16	0.446	0.707
17	0.186	0.606
18	0.786	0.245
19	0.337	0.215
X	0.866	0.973
Y	1.00	1.00

The table lists p−values generated from a test that evaluates whether there exists an over−abundance of identified genes with respect to a given chromosome. The null hypothesis assumes that the set of genes has been selected at random from those represented on the Affymetrix 430 2.0 array. A significant test indicates that a chromosome contains more of the identified genes than would be expected if the gene set had been chosen at random.

* = significant p−value, without multiple test adjustment

** = significant p−value, with multiple test adjustment

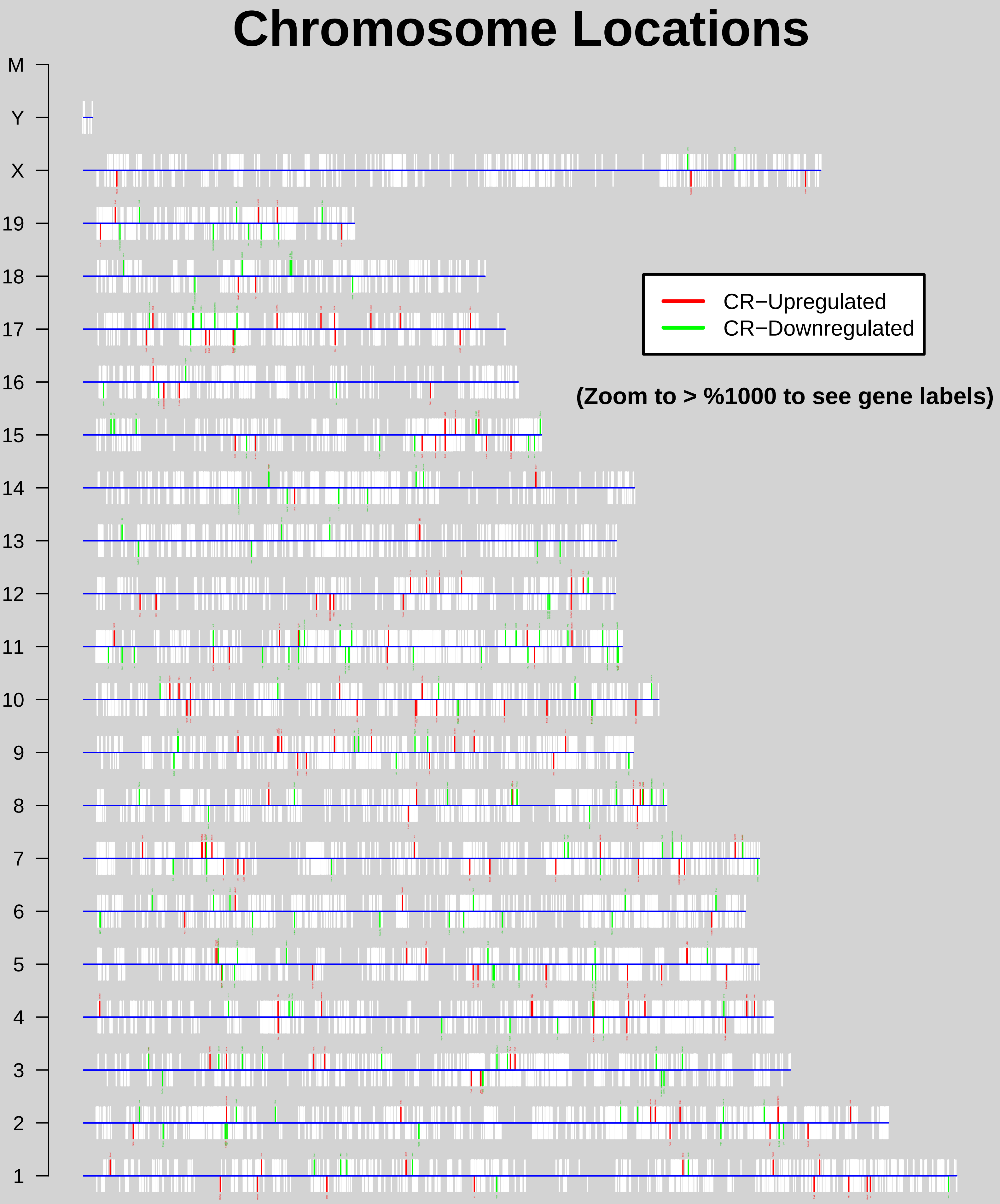
Chromosome Locations
M
Y
X
19
18
17
16
15
14
13
12
11
10
9
8
7
6
5
4
3
2
1
CR−Upregulated
CR−Downregulated
(Zoom to > %1000 to see gene labels)
