# Supplementary material for: Genes and gene expression modules associated with caloric restriction and aging in the laboratory mouse
Source: BMC Genomics. 2009 Dec 7;10:585. doi: 10.1186/1471-2164-10-585 (PMC2795771; doi:10.1186/1471-2164-10-585)

Additional File 3

Genes and Gene Expression Modules Associated with Caloric Restriction and Aging in the Laboratory Mouse

William R. Swindell
University of Michigan, Departments of Pathology and Geriatrics

---

Genes Regulated by Caloric Restriction in Heart

This file provides information on genes significantly influenced by CR in the heart. The first set of charts displays differential expression results of the 200 genes most strongly up regulated by CR in heart, while the second set of charts displays differential expression results for the 200 genes most strongly down regulated by CR in heart. Each row corresponds to an individual gene and each column corresponds to a separate experiment (see Additional File 1). Symbols are interpreted as follows:

- ● Gene is significantly up regulated by CR ($P_u < 0.05$)
- ● Gene is significantly down regulated by CR ($P_d < 0.05$)
- ▬ Gene is marginally up regulated by CR ($0.05 < P_u < 0.10$)
- ▬ Gene is marginally down regulated by CR ($0.05 < P_d < 0.10$)
- ▬ Non-significant CR effect ($P_u > 0.10$ and $P_d > 0.10$)
- × No data (gene not represented in experiment or array annotation was limiting)
- ✳ Evidence conflicts but favors up regulation by CR
- ✳ Evidence conflicts, but favors down regulation by CR

The last two categories (✳ and ✳) indicate significant effects with conflicting evidence. This can arise if multiple transcripts associated with the same gene symbol yield opposite conclusions. Alternatively, a conflict may arise if $P_u < 0.05$ and also $P_d < 0.05$. Symbols shown in charts are based upon a comparison-wise type I error rate of 0.05. The final column in each chart lists meta-analysis p-values generated using Fisher's method, which have been adjusted using the Benjamini-Hochberg method to control the false discovery rate among all 21,327 genes.

The remainder of the file includes lists of over-represented gene ontology terms, over-represented KEGG pathways, and over-represented KEGG pathways defined based upon IP domain signatures (see Hahne et al. 2008, BMC Bioinformatics 9:3). Genes were also analyzed to determine if there existed an over-abundance of targets for certain microRNAs (see Betel et al. 2008, Nucleic Acids Res. 36: D149-153), and a list of associated microRNAs is provided based upon this analysis. Lastly, tests for over-representation of identified genes with respect to each chromosome were performed, and an idiogram mapping of identified genes to chromosomal locations is shown.

Contact: William R. Swindell, wswindel@umich.edu

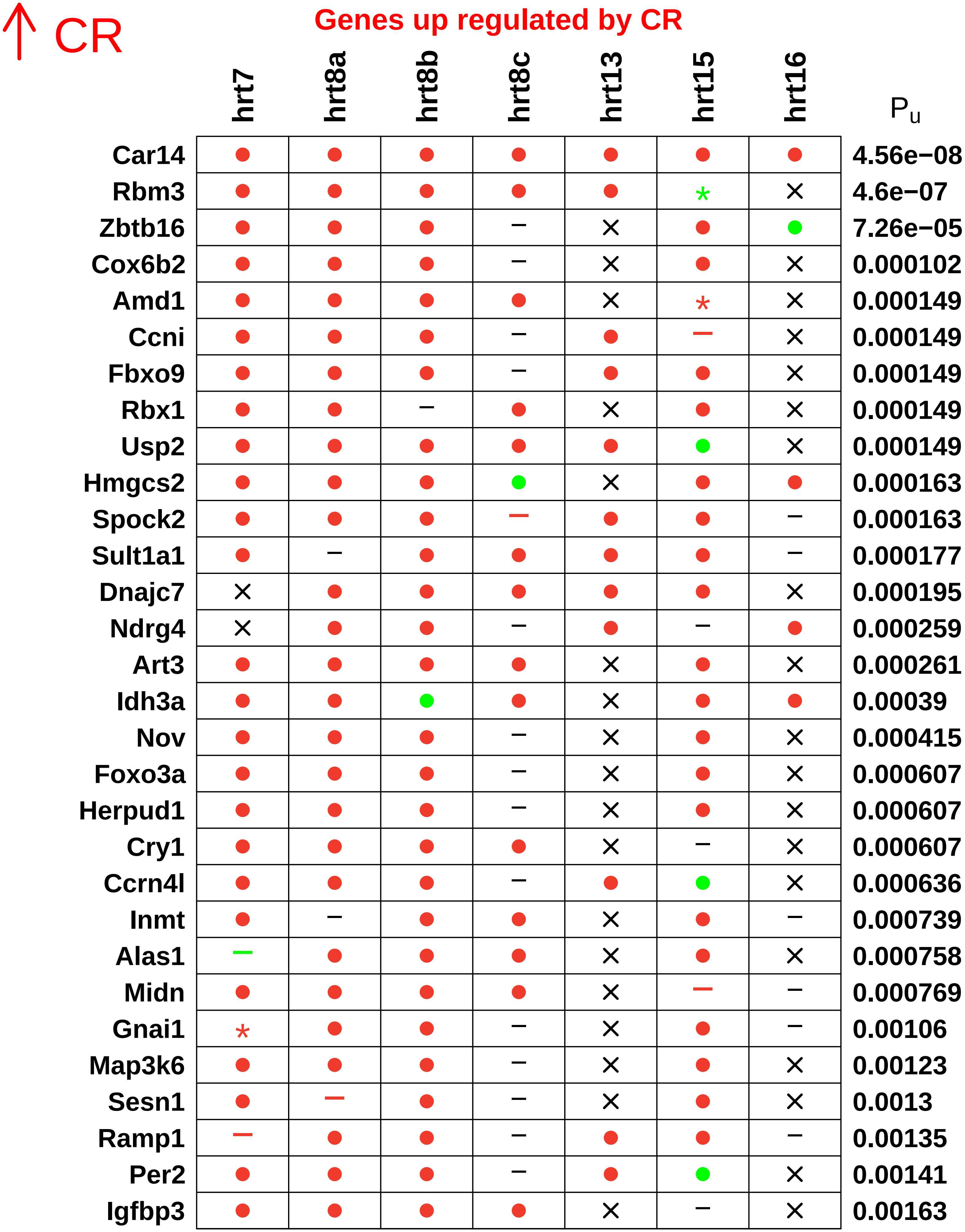
↑ CR
Genes up regulated by CR
hrt7
hrt8a
hrt8b
hrt8c
hrt13
hrt15
hrt16
P_u
Car14
Rbm3
Zbtb16
Cox6b2
Amd1
Ccni
Fbxo9
Rbx1
Usp2
Hmgcs2
Spock2
Sult1a1
Dnajc7
Ndrg4
Art3
Idh3a
Nov
Foxo3a
Herpud1
Cry1
Ccrn4l
Inmt
Alas1
Midn
Gnai1
Map3k6
Sesn1
Ramp1
Per2
Igfbp3
4.56e−08
4.6e−07
7.26e−05
0.000102
0.000149
0.000149
0.000149
0.000149
0.000149
0.000163
0.000163
0.000177
0.000195
0.000259
0.000261
0.00039
0.000415
0.000607
0.000607
0.000607
0.000636
0.000739
0.000758
0.000769
0.00106
0.00123
0.0013
0.00135
0.00141
0.00163

↑ CR

Genes up regulated by CR

	hrt7	hrt8a	hrt8b	hrt8c	hrt13	hrt15	hrt16	P_u
**Man2a1**	🔴	🔴	−	−	×	🔴	−	**0.0017**
**Perp**	−	🔴	🔴	🔴	×	−	−	**0.00192**
**3110003A17Rik**	−	🔴	🔴	−	×	🔴	×	**0.00204**
**Peci**	🔴	🔴	🔴	−	🔴	−	×	**0.00224**
**Ntn1**	🔴	🔴	🔴	−	🔴	🔴	×	**0.00226**
**3300001P08Rik**	×	🔴	🔴	− (red)	×	🟢	🔴	**0.0024**
**D730040F13Rik**	🔴	🔴	−	−	🔴	🔴	×	**0.0024**
**Pfkfb1**	🔴	−	− (red)	🔴	×	🔴	−	**0.00246**
**Tubb6**	×	🔴	🔴	− (red)	×	🟢	🔴	**0.00249**
**Tuba8**	🟢	🔴	🔴	🔴	×	🟢	🔴	**0.00253**
**Fkbp5**	🔴	−	🔴	−	×	🔴	− (green)	**0.00259**
**Gcom1**	×	🔴	🔴	🔴	×	− (red)	×	**0.00265**
**Ppara**	−	🔴	🔴	🔴	×	🟢	×	**0.00317**
**Btg3**	🔴	🔴	🔴	−	🔴	− (red)	×	**0.00317**
**Rgs2**	🔴	🔴	−	−	🔴	🔴	×	**0.0032**
**Hnrpll**	− (green)	🔴	🔴	−	×	🔴	🔴	**0.0036**
**Ddit4**	🔴	🔴	− (red)	−	×	🔴	×	**0.0036**
**Tmem11**	🔴	🔴	−	−	×	🔴	🔴	**0.0036**
**Csrp2**	🟢	🔴	🔴	🟢	×	🔴	🔴	**0.0038**
**Abcd3**	🔴	🔴	−	− (red)	×	🔴	×	**0.00418**
**Lifr**	🔴	🔴	🔴	−	×	🔴	×	**0.00418**
**Tuba3a**	🟢	🔴	🔴	🔴	×	−	×	**0.00418**
**D7Wsu130e**	🔴	− (red)	−	−	🔴	🔴	×	**0.0044**
**Cish**	🔴	🔴	🔴	🔴	×	−	−	**0.00444**
**Vdac2**	×	🔴	🔴	🔴	×	− (red)	×	**0.00444**
**Flcn**	×	🔴	🔴	−	×	🔴	−	**0.00478**
**Rpl3**	−	🔴	🔴	🔴	🔴	−	×	**0.00549**
**Glul**	🔴	−	🔴	−	×	🔴	−	**0.0058**
**Slc25a17**	🔴	−	🔴	−	×	🔴	−	**0.0058**
**Ide**	🔴	🔴	🔴	🔴	×	🔴	×	**0.00614**

↑ CR

Genes up regulated by CR

	hrt7	hrt8a	hrt8b	hrt8c	hrt13	hrt15	hrt16	P_u
Gata6	–	●	●	–	×	●	–	0.00618
Mtmr1	–	–	–	–	×	●	●	0.00618
2410124H12Rik	×	×	×	×	×	●	●	0.00618
6530404N21Rik	×	×	×	×	×	●	●	0.00618
Acot1	●	●	●	–	×	–	×	0.00618
Dhx36	●	–	●	–	×	●	–	0.0064
Ccr1	–	● (green)	–	●	×	●	●	0.0067
Pcdh7	●	–	●	–	●	●	×	0.0067
Fmo3	●	–	●	– (green)	×	●	×	0.0067
Tuba4a	×	×	×	×	×	●	●	0.00704
Isca1	–	●	–	●	×	●	×	0.00734
Txndc1	●	–	– (red)	●	●	●	– (green)	0.00748
Atp6v1d	●	●	●	– (red)	×	●	–	0.00748
0610010K06Rik	–	●	– (green)	–	×	●	×	0.00758
0610037D15Rik	×	×	×	×	×	*	–	0.00758
0610038F07Rik	● (green)	–	–	–	×	●	●	0.00758
0610040A22Rik	×	×	×	×	×	●	×	0.00758
100039204	●	● (green)	–	–	×	●	×	0.00758
100042277	×	×	×	×	×	●	×	0.00758
100042286	×	×	×	×	×	●	×	0.00758
1110003E01Rik	– (green)	●	● (green)	–	×	*	●	0.00758
1110004M10Rik	×	×	×	×	×	●	×	0.00758
1110005A03Rik	×	×	×	×	×	*	×	0.00758
1110014N23Rik	×	×	×	×	×	*	–	0.00758
1110017I16Rik	×	×	×	×	×	●	×	0.00758
1110018G07Rik	×	×	×	×	×	●	–	0.00758
1110021J02Rik	×	×	×	×	×	*	×	0.00758
1110034G24Rik	×	×	×	×	×	●	×	0.00758
1110057K04Rik	– (green)	–	● (green)	–	×	*	×	0.00758
1110067D22Rik	×	–	●	–	×	●	×	0.00758

↑ CR

Genes up regulated by CR

	hrt7	hrt8a	hrt8b	hrt8c	hrt13	hrt15	hrt16	P_u
1200015N20Rik	×	×	×	×	×	*	–	0.00758
1300018I17Rik	×	×	×	×	×	●	×	0.00758
1500005I02Rik	×	–	–	–	×	●	×	0.00758
1500010J02Rik	×	●	–	–	×	●	●	0.00758
1500032O14Rik	×	×	×	×	×	●	×	0.00758
1520401O13Rik	×	×	×	×	×	●	×	0.00758
1600014C10Rik	×	×	×	×	×	*	–	0.00758
1600027N09Rik	×	×	×	×	×	●	×	0.00758
1600029D21Rik	–	–	–	–	×	●	×	0.00758
1700001C19Rik	×	×	×	×	×	●	×	0.00758
1700003F12Rik	×	×	×	×	×	●	×	0.00758
1700003H04Rik	×	×	×	×	×	●	×	0.00758
1700008I05Rik	×	×	×	×	×	●	×	0.00758
1700010H22Rik	×	×	×	×	×	●	×	0.00758
1700010I14Rik	×	×	×	×	×	●	×	0.00758
1700021K02Rik	×	×	×	×	×	●	×	0.00758
1700023B13Rik	×	×	×	×	×	●	×	0.00758
1700023L04Rik	×	×	×	×	×	●	×	0.00758
1700026H06Rik	×	×	×	×	×	●	×	0.00758
1700029I08Rik	×	×	×	×	×	●	×	0.00758
1700029M03Rik	×	×	×	×	×	●	×	0.00758
1700034F02Rik	×	×	×	×	×	●	×	0.00758
1700036D21Rik	×	×	×	×	×	●	×	0.00758
1700054O13Rik	×	×	×	×	×	●	×	0.00758
1700061J05Rik	×	×	×	×	×	●	–	0.00758
1700073E17Rik	×	×	×	×	×	●	×	0.00758
1700081D17Rik	×	×	×	×	×	●	×	0.00758
1700101I19Rik	×	×	×	×	×	●	×	0.00758
1700105P06Rik	×	×	×	×	×	●	×	0.00758
1700112M01Rik	×	×	×	×	×	●	×	0.00758

↑ CR

Genes up regulated by CR

	hrt7	hrt8a	hrt8b	hrt8c	hrt13	hrt15	hrt16	P_u
1810010M01Rik	×	−	−	−	×	● (red)	−	0.00758
1810011H11Rik	×	×	×	×	×	● (red)	×	0.00758
1810012K16Rik	×	×	×	×	×	● (red)	×	0.00758
1810015A16Rik	×	×	×	×	×	● (red)	×	0.00758
1810015C04Rik	● (red)	−	● (red)	−	×	● (red)	● (green)	0.00758
1810020G14Rik	×	−	−	−	×	● (red)	×	0.00758
1810026J23Rik	×	×	×	×	×	● (red)	×	0.00758
1810030O07Rik	−	− (red)	−	● (green)	×	* (red)	×	0.00758
2010000I03Rik	×	×	×	×	×	● (red)	×	0.00758
2010003H20Rik	×	×	×	×	×	● (red)	×	0.00758
2010106G01Rik	×	×	×	×	×	* (red)	×	0.00758
2010109A12Rik	×	×	×	×	×	● (red)	×	0.00758
2210008N01Rik	×	×	×	×	×	● (red)	×	0.00758
2210012G02Rik	×	×	×	×	×	● (red)	−	0.00758
2210409E12Rik	×	×	×	×	×	● (red)	×	0.00758
2210409O19Rik	×	×	×	×	×	● (red)	×	0.00758
2210411G17Rik	×	×	×	×	×	● (red)	×	0.00758
2300004M11Rik	×	×	×	×	×	● (red)	×	0.00758
2300010F08Rik	×	×	×	×	×	● (red)	×	0.00758
2310005N03Rik	×	×	×	×	×	* (red)	×	0.00758
2310033K02Rik	×	×	×	×	×	● (red)	×	0.00758
2310035K24Rik	● (green)	− (red)	−	−	×	● (red)	● (red)	0.00758
2310038E17Rik	×	×	×	×	×	● (red)	×	0.00758
2310038H17Rik	×	×	×	×	×	● (red)	− (red)	0.00758
2310040A07Rik	×	×	×	×	×	● (red)	×	0.00758
2310040C09Rik	−	● (green)	−	−	×	● (red)	×	0.00758
2310045A20Rik	×	×	×	×	×	● (red)	−	0.00758
2310047O13Rik	×	×	×	×	×	● (red)	×	0.00758
2310051E17Rik	● (red)	−	−	−	×	● (red)	×	0.00758
2310076G13Rik	×	×	×	×	×	* (red)	×	0.00758

↑ CR

Genes up regulated by CR

	hrt7	hrt8a	hrt8b	hrt8c	hrt13	hrt15	hrt16	P_u
2410015M20Rik	–	●	–	–	×	●	–	0.00758
2410017P07Rik	×	×	×	×	×	●	×	0.00758
2410018L13Rik	×	×	×	×	×	●	×	0.00758
2410025L10Rik	×	×	×	×	×	●	×	0.00758
2410049M19Rik	×	×	×	×	×	●	×	0.00758
2410076I21Rik	×	×	×	×	×	●	–	0.00758
2500003M10Rik	–	●	–	–	×	●	×	0.00758
2600010E01Rik	×	×	×	×	×	●	×	0.00758
2610015P09Rik	×	×	×	×	×	●	×	0.00758
2610021K21Rik	×	×	×	×	×	●	×	0.00758
2610024D14Rik	×	×	×	×	×	●	×	0.00758
2610035D17Rik	×	×	×	×	×	●	×	0.00758
2610037D02Rik	×	×	×	×	×	●	×	0.00758
2610105M22Rik	×	×	×	×	×	●	×	0.00758
2610207I05Rik	×	×	×	×	×	●	×	0.00758
2610301B20Rik	–	–	–	–	×	●	–	0.00758
2610301F02Rik	×	×	×	×	×	●	×	0.00758
2700008E08Rik	×	×	×	×	×	●	×	0.00758
2810022L02Rik	×	×	×	×	×	*	×	0.00758
2810403D21Rik	×	×	×	×	×	●	×	0.00758
2810407C02Rik	–	●	–	–	×	●	×	0.00758
2810408A11Rik	×	×	×	×	×	●	×	0.00758
2810410P21Rik	×	×	×	×	×	●	×	0.00758
2810433K01Rik	×	×	×	×	×	●	×	0.00758
2810442I21Rik	×	×	×	×	×	●	×	0.00758
2810449G22Rik	×	×	×	×	×	●	×	0.00758
2810485I05Rik	●	–	–	–	●	●	–	0.00758
2900011F02Rik	×	×	×	×	×	●	×	0.00758
2900018N21Rik	×	×	×	×	×	●	×	0.00758
2900042E19Rik	×	×	×	×	×	●	×	0.00758

↑ CR

Genes up regulated by CR

	hrt7	hrt8a	hrt8b	hrt8c	hrt13	hrt15	hrt16	P_u
2900060K15Rik	×	×	×	×	×	●	×	0.00758
2900069G24Rik	×	×	×	×	×	●	×	0.00758
2900078I11Rik	×	×	×	×	×	●	×	0.00758
2900092N22Rik	×	×	×	×	×	●	×	0.00758
2900097C17Rik	–	–	–	–	×	*	×	0.00758
3010026O09Rik	×	×	×	×	×	●	–	0.00758
3110002H16Rik	×	–	●	–	×	●	●	0.00758
3110007F17Rik	×	×	×	×	×	●	×	0.00758
3110021A11Rik	×	×	×	×	×	●	×	0.00758
3110032G18Rik	×	×	×	×	×	●	×	0.00758
3110049I03Rik	×	×	×	×	×	●	×	0.00758
3110054G05Rik	×	×	×	×	×	●	×	0.00758
3110057O12Rik	×	×	×	×	×	*	–	0.00758
3110062G12Rik	×	×	×	×	×	●	×	0.00758
3222402N08Rik	×	×	×	×	×	●	×	0.00758
3222402P14Rik	×	×	×	×	×	●	×	0.00758
3300002A11Rik	×	×	×	×	×	●	×	0.00758
3300002I10Rik	×	×	×	×	×	●	×	0.00758
3321401G04Rik	×	×	×	×	×	●	×	0.00758
3830422I06Rik	×	×	×	×	×	●	×	0.00758

↓ CR

Genes down regulated by CR

	hrt7	hrt8a	hrt8b	hrt8c	hrt13	hrt15	hrt16	P_d
Ifi203	●	●	●	●	●	●	×	5.29e−07
1500005K14Rik	●	–	●	●	●	●	×	1.43e−05
Col4a1	●	–	●	●	●	●	●	1.43e−05
Ube1l	●	●	●	●	×	●	–	1.62e−05
Cpxm2	●	●	●	–	●	–	×	1.62e−05
Col15a1	●	–	●	●	●	●	–	1.62e−05
Cx3cl1	●	●	●	–	●	●	–	1.81e−05
Rcan1	●	–	●	●	●	*	×	1.81e−05
Cd74	–	●	●	●	●	●	×	1.81e−05
Pltp	●	●	●	●	×	●	×	2.01e−05
Rps6ka2	●	●	●	–	●	*	×	2.19e−05
AW112010	●	–	●	●	●	●	×	2.38e−05
Fbn1	●	–	●	●	●	●	×	2.53e−05
Tie1	●	●	●	●	×	●	×	2.53e−05
Clpb	●	●	●	–	×	●	–	2.64e−05
Cd93	–	–	●	●	●	●	●	5.71e−05
Ly6a	●	●	●	●	●	●	–	5.71e−05
Ets1	–	●	●	●	●	●	×	6.98e−05
Grk5	●	●	●	●	×	●	×	6.98e−05
Mapt	●	●	●	●	×	●	×	6.98e−05
Ccl7	●	●	●	●	×	●	×	6.98e−05
Cyb5r1	●	●	●	●	×	●	●	6.98e−05
Col3a1	●	–	●	●	●	●	–	7.18e−05
Gimap4	●	–	●	●	×	●	×	7.32e−05
Col1a1	●	–	●	●	●	●	×	7.63e−05
Cdh5	●	–	●	●	×	●	●	7.74e−05
Hexa	●	–	●	●	×	●	●	9.12e−05
Cyb5b	●	–	●	●	×	●	–	9.72e−05
Armet	●	–	●	–	●	●	–	0.00012
Nppb	●	●	●	●	×	●	●	0.000135

↓ CR

Genes down regulated by CR

	hrt7	hrt8a	hrt8b	hrt8c	hrt13	hrt15	hrt16	P_d
Sept4	green ●	green ●	green ●	green ●	green ●	green ●	−	0.000139
H2−Aa	−	green ●	green ●	green ●	green ●	red ●	−	0.000194
Col4a2	green ●	−	green ●	green ●	×	green ●	×	0.000194
Uck2	−	green ●	green ●	green ●	×	green *	×	0.000242
Dynll1	green ●	red ●	green ●	green ●	×	green ●	×	0.000286
St8sia4	green ●	green ●	green −	green ●	×	green *	red ●	0.000359
Ubfd1	green ●	green −	green ●	−	×	green ●	×	0.000359
Acads	green ●	−	green ●	green −	×	green ●	×	0.00036
Csf1	green ●	green ●	green ●	−	×	green ●	−	0.000365
Gbp3	green ●	green ●	−	green −	×	green ●	×	0.000365
Sms	green ●	green ●	−	green −	×	green ●	×	0.000384
Coq4	green ●	green ●	green ●	green −	×	green ●	×	0.000423
Nr1h3	green ●	−	−	green ●	×	green ●	green ●	0.000423
Hsph1	green ●	red ●	green ●	−	green ●	green ●	×	0.000445
Sipa1	green ●	−	green ●	green −	×	green ●	green ●	0.000453
Acta1	green ●	−	−	green ●	green ●	green ●	−	0.000486
Serpinh1	green ●	red ●	green ●	green ●	green ●	green ●	−	0.000495
Usp18	−	green ●	green −	green ●	×	green ●	×	0.000537
Atf5	green ●	−	green ●	−	green ●	green ●	×	0.000631
Icam1	green ●	green ●	green −	−	×	green ●	×	0.000631
Nqo1	green −	green ●	green ●	red ●	×	green ●	×	0.000631
Kdr	green ●	−	green −	green −	green ●	green ●	×	0.000633
H13	green ●	−	green −	green ●	×	green *	green ●	0.000639
2010111I01Rik	green ●	−	green ●	−	×	green *	×	0.00065
Notch3	red −	green ●	green ●	green ●	×	green ●	green ●	0.00065
2810453I06Rik	green ●	green ●	green ●	green −	green ●	red ●	×	0.000699
Tmsb10	green ●	−	green ●	green ●	×	green ●	×	0.000715
AU020206	−	green ●	green ●	−	×	green ●	×	0.000715
Cacna1s	green ●	green ●	green ●	−	×	green ●	×	0.000963
Ivd	green ●	green ●	green ●	red ●	×	−	green ●	0.000971

↓ CR

Genes down regulated by CR

	hrt7	hrt8a	hrt8b	hrt8c	hrt13	hrt15	hrt16	P_d
Marcks	–	green ●	green ●	green ●	green ●	red ●	–	0.000971
Irf7	green ●	green ●	–	green ●	×	green ●	–	0.000971
Sirpa	green ●	green ●	green ●	–	×	green *	–	0.000996
Psmb8	green ●	green ●	green ●	green –	×	green ●	green –	0.001
Gbp2	green –	green ●	green ●	green –	green ●	green ●	–	0.00105
Serpinb9	green ●	–	green ●	green ●	×	green ●	×	0.00105
Shisa5	–	green –	green ●	green ●	×	green ●	×	0.00105
Col6a3	green ●	–	green ●	green ●	×	–	×	0.00106
Rexo2	green ●	green ●	green ●	green ●	×	green ●	×	0.00123
Tubb2a	green ●	red ●	green ●	–	×	green ●	×	0.00128
100040213	green ●	green ●	green ●	–	×	green ●	×	0.00128
Nrap	×	green ●	green ●	–	×	green ●	green –	0.00128
2410014A08Rik	green ●	–	green ●	–	green ●	green *	×	0.0015
Pfn2	–	green ●	green ●	–	×	green ●	×	0.0015
Vegfc	green ●	green ●	–	green ●	×	green ●	–	0.0015
Fbln1	green ●	red –	green ●	green –	×	green *	×	0.00163
Ptrf	green ●	green ●	–	green ●	×	green ●	×	0.00163
Junb	green ●	–	green ●	green –	×	green ●	green ●	0.00163
Ppm1g	green ●	–	green ●	–	×	green ●	green ●	0.00181
Rrad	green ●	green ●	green –	–	×	green ●	×	0.00183
Nrd1	green ●	green ●	green ●	–	×	green ●	–	0.00188
Bckdha	green ●	–	green ●	red ●	×	green ●	green ●	0.00188
C1qc	green ●	green ●	green ●	–	green ●	–	–	0.00188
Lcp1	green –	green ●	green ●	–	×	green ●	red ●	0.00204
Arntl	green ●	green –	–	green ●	green ●	red –	green ●	0.00208
Col6a2	green ●	–	green ●	green ●	×	–	green ●	0.00208
Cxcl14	green ●	green ●	green ●	–	×	red ●	green ●	0.00208
Hspa8	green ●	red ●	green ●	green ●	×	green ●	×	0.00208
Map4k4	green ●	–	green ●	green ●	×	green ●	×	0.00208
Sparc	green ●	–	–	green ●	×	green ●	×	0.00208

↓ CR

Genes down regulated by CR

	hrt7	hrt8a	hrt8b	hrt8c	hrt13	hrt15	hrt16	P_d
Txnrd1	●	●	−	−	×	●	−	0.00208
Atn1	●	−	● (red)	−	×	●	●	0.00208
H2−Eb1	− (green)	●	●	●	×	−	●	0.00208
C1qb	●	●	●	− (green)	●	−	−	0.00211
Cd82	−	●	●	−	×	●	●	0.00211
P4ha1	●	● (red)	●	− (green)	×	●	−	0.00211
Psmb9	●	●	−	●	×	* (green)	− (green)	0.00211
Cmtm3	●	−	− (green)	●	×	●	×	0.00211
Dpp3	●	●	●	−	×	●	×	0.00211
Iigp2	−	●	−	−	×	●	●	0.00211
Tnks1bp1	●	●	−	−	×	●	●	0.00211
Myh9	●	−	●	− (green)	×	●	×	0.00216
Pcolce	●	−	●	●	×	●	−	0.00216
H2−K1	●	●	●	●	×	* (green)	×	0.00224
Ctsd	●	●	●	−	×	− (green)	×	0.00224
Tars2	●	−	−	−	×	●	●	0.00227
Arhgef5	×	●	●	−	×	●	− (green)	0.00227
Gja1	●	●	●	− (green)	×	* (green)	● (red)	0.00239
H2−Ab1	●	●	●	− (green)	●	− (green)	×	0.00239
Itpkb	− (green)	− (green)	●	−	●	●	×	0.00239
Slfn2	●	●	●	−	×	●	−	0.00247
Ugdh	●	−	●	●	×	●	×	0.00247
Rrbp1	●	−	●	−	×	●	− (green)	0.00248
H2−Q7	●	− (green)	−	− (green)	×	●	×	0.00248
1700027J05Rik	×	×	×	×	×	* (red)	●	0.00248
Acbd4	×	×	×	×	×	●	●	0.00248
Agrn	−	●	●	●	×	●	−	0.00248
C130038G02Rik	×	×	×	×	×	* (green)	●	0.00248
Cpt2	−	− (green)	●	●	×	●	×	0.00248
Dis3l2	×	×	×	×	×	* (red)	●	0.00248

↓ CR

Genes down regulated by CR

	hrt7	hrt8a	hrt8b	hrt8c	hrt13	hrt15	hrt16	P_d
Fkbp10	–	red ●	–	–	×	green ●	green ●	0.00248
H2−D1	green ●	green –	–	green ●	×	green *	×	0.00248
Hsp90b1	×	–	green ●	green ●	×	green *	–	0.00248
Ifi205	red –	green ●	green ●	green ●	×	red ●	×	0.00248
Kat2a	×	–	green –	–	×	green ●	green ●	0.00248
Kcnk3	–	–	red ●	–	×	green ●	green ●	0.00248
Klhl22	×	×	×	×	×	green *	green ●	0.00248
Lama4	green ●	red ●	green ●	green ●	×	red *	×	0.00248
Lama5	red –	–	–	–	×	green ●	green ●	0.00248
Lipa	red ●	green ●	green –	green ●	×	green ●	×	0.00248
Mical3	×	×	×	×	×	green ●	green ●	0.00248
Nfic	red –	–	green ●	–	×	green ●	green ●	0.00248
Pea15a	green ●	–	green ●	green ●	×	green ●	×	0.00248
Phf20l1	green ●	green ●	–	green ●	green ●	green ●	×	0.00248
Ppp1r16a	×	×	×	×	×	green ●	green ●	0.00248
Purb	red ●	–	–	–	×	green ●	green ●	0.00248
Rgs12	×	×	×	×	×	red *	green ●	0.00248
Scly	×	×	×	×	×	green ●	green ●	0.00248
Wdr6	green ●	green ●	–	–	×	green ●	green ●	0.00248
Zfp629	×	×	×	×	×	green ●	green ●	0.00248
2310044H10Rik	green ●	red ●	green ●	green –	×	green ●	×	0.00248
2610027L16Rik	×	×	×	×	×	green ●	green ●	0.00248
Amy1	red ●	green ●	green ●	red ●	×	green –	green ●	0.00248
BC039210	×	×	×	×	×	green ●	green ●	0.00248
Ctps	green ●	–	green ●	–	×	green ●	×	0.00248
Dhx57	×	×	×	×	×	green ●	green ●	0.00248
Ifitm3	×	green ●	green ●	green ●	×	green ●	–	0.00248
Mbd3	×	×	×	×	×	green ●	green ●	0.00248
Nfe2l1	×	green –	green ●	–	×	green ●	green ●	0.00248
Perld1	×	×	×	×	×	green ●	green ●	0.00248

↓ CR

Genes down regulated by CR

	hrt7	hrt8a	hrt8b	hrt8c	hrt13	hrt15	hrt16	P_d
Ppil2	● (green)	–	● (green)	–	×	● (red)	● (green)	0.00248
Prkcdbp	–	–	● (green)	● (green)	×	● (green)	×	0.00248
Prr13	● (green)	● (green)	– (green)	–	×	● (green)	–	0.00248
Rasip1	×	×	×	×	×	● (green)	● (green)	0.00248
Rce1	● (green)	–	● (green)	– (green)	×	● (green)	● (green)	0.00248
Rnf31	×	×	×	×	×	● (green)	● (green)	0.00248
Scaf1	×	×	×	×	×	● (green)	● (green)	0.00248
Slc12a4	● (green)	–	–	–	×	● (green)	● (green)	0.00248
Smarcc2	×	×	×	×	×	● (green)	● (green)	0.00248
Tbc1d13	×	×	×	×	×	● (green)	● (green)	0.00248
Unk	×	×	×	×	×	● (green)	● (green)	0.00248
Zhx2	×	×	×	×	×	● (green)	● (green)	0.00248
Mesdc2	● (green)	–	● (green)	–	×	● (green)	–	0.00251
Arsa	● (green)	–	● (green)	● (green)	×	● (green)	– (green)	0.00252
P2ry2	● (green)	● (green)	–	– (green)	×	● (green)	×	0.00259
2310066E14Rik	×	×	×	×	×	● (green)	● (green)	0.00262
Dnajc2	● (red)	● (green)	● (green)	– (red)	×	* (green)	● (green)	0.00262
Eng	–	– (red)	– (green)	● (green)	×	● (green)	● (green)	0.00262
Ganab	● (green)	–	–	● (green)	×	● (green)	×	0.00262
Card10	×	×	×	×	×	● (green)	● (green)	0.00262
Cdc34	● (green)	– (green)	– (green)	–	×	● (green)	×	0.00262
Copz2	● (green)	● (green)	–	–	● (green)	● (green)	×	0.00262
Frmd6	● (green)	–	● (green)	–	×	● (green)	×	0.00262
Itgb1bp1	● (green)	● (green)	–	–	×	● (green)	×	0.00262
Plekho2	×	×	×	×	×	● (green)	● (green)	0.00268
Rpn1	● (green)	● (red)	● (green)	–	×	● (green)	×	0.0027
Gprasp1	×	×	×	×	×	* (green)	● (green)	0.00276
Anapc2	● (green)	–	– (green)	● (green)	×	● (green)	×	0.00276
Cldn5	● (green)	● (red)	–	● (green)	● (green)	–	– (green)	0.00276
Csnk2a1	* (red)	– (red)	* (green)	● (green)	×	● (green)	–	0.00281

↓ CR

Genes down regulated by CR

	hrt7	hrt8a	hrt8b	hrt8c	hrt13	hrt15	hrt16	P_d
Wscd1	×	×	×	×	×	●	●	0.00291
Abca2	●	–	–	–	×	●	●	0.00291
Cerk	●	●	●	●	×	●	×	0.00291
P4ha2	●	–	●	–	×	●	×	0.00295
4931406P16Rik	×	×	×	×	×	*	●	0.00299
Nkain1	–	●	●	–	×	●	–	0.00309
Hspa5	●	–	●	–	×	●	–	0.0031
Bcam	×	×	×	×	×	●	●	0.0031
Kbtbd5	×	×	×	×	×	●	●	0.0031
Pprc1	●	–	–	–	×	●	●	0.00312
Ccdc124	×	×	×	×	×	●	●	0.0032
Pias4	×	×	×	×	×	*	●	0.00321
Jak3	●	–	–	–	×	*	×	0.00322
Drg2	×	×	×	×	×	●	●	0.00323
Ptpn21	–	–	●	–	×	●	●	0.00343
Myo18a	×	×	×	×	×	●	●	0.00343
Comt1	●	●	●	–	×	●	×	0.00348
Scn1b	●	●	–	–	×	●	×	0.00348
Loxl1	●	–	–	–	×	●	–	0.00358
Slc25a36	×	●	●	–	×	*	–	0.00363

↑ CR

Overrepresented Biological Processes

GO Term	P−Value
reproductive behavior	0.00131
positive regulation of mononuclear cell proliferation	0.00193
positive regulation of biological process	0.00202
positive regulation of erythrocyte differentiation	0.00228
cell−matrix adhesion	0.00257
cellular copper ion homeostasis	0.00293
negative regulation of T cell mediated cytotoxicity	0.00344
positive regulation of transforming growth factor beta receptor signaling pathway	0.00344
maternal behavior	0.00344
negative regulation of myeloid cell differentiation	0.00423
mitotic cell cycle	0.00424
negative regulation of osteoclast differentiation	0.00578
DNA replication	0.00702
lymphocyte mediated immunity	0.00734
B cell activation	0.00734
regulation of B cell differentiation	0.00776
positive regulation of B cell proliferation	0.00855
regulation of T cell proliferation	0.00947
negative regulation of adaptive immune response based on somatic recombination of immune receptors bu	0.0101
positive regulation of leukocyte activation	0.0104
regulation of cell activation	0.0118
nuclear export	0.0118
response to pheromone	0.0122
signal peptide processing	0.0122
olfactory bulb interneuron differentiation	0.0122
regulation of cholesterol absorption	0.0122
regulation of myeloid cell differentiation	0.0122
acetyl−CoA metabolic process	0.0143
cellular response to nutrient levels	0.0157
response to starvation	0.0157

↑ CR

Overrepresented Biological Processes

GO Term	P−Value
response to extracellular stimulus	0.016
regulation of nucleotide metabolic process	0.0163
proton transport	0.0164
positive regulation of signal transduction	0.0168
T cell homeostasis	0.0168
regulation of adaptive immune response	0.0184
adaptive immune response based on somatic recombination of immune receptors built from immunoglobul	0.0195
reproduction	0.0216
negative regulation of hydrolase activity	0.0218
copper ion transport	0.0218
positive regulation of activated T cell proliferation	0.0218
positive regulation of alpha−beta T cell proliferation	0.0218
ventricular cardiac muscle morphogenesis	0.0218
positive regulation of humoral immune response mediated by circulating immunoglobulin	0.0228
acetyl−CoA biosynthetic process from pyruvate	0.0228
nitric oxide mediated signal transduction	0.0228
asymmetric cell division	0.0228
embryonic olfactory bulb interneuron precursor migration	0.0228
fatty acid elongation	0.0228
positive regulation of cGMP biosynthetic process	0.0228
positive regulation of Rac protein signal transduction	0.0228
leg morphogenesis	0.0228
embryonic arm morphogenesis	0.0228
positive regulation of NF−kappaB import into nucleus	0.0228
natural killer cell degranulation	0.0228
glycerol−3−phosphate catabolic process	0.0228
polyol catabolic process	0.0228
aldehyde catabolic process	0.0228
negative regulation of hair follicle development	0.0228
atrial cardiac muscle morphogenesis	0.0228

↑ CR

Overrepresented Biological Processes

GO Term	P−Value
negative regulation of lipase activity	0.0228
DNA damage response, signal transduction by p53 class mediator	0.0242
negative regulation of immune effector process	0.0245
regulation of T cell mediated immunity	0.0245
cell cycle process	0.0254
positive regulation of T cell activation	0.0264
cellular response to stimulus	0.0267
G1 phase of mitotic cell cycle	0.0271
regulation of lipid kinase activity	0.0271
positive regulation of phosphoinositide 3−kinase activity	0.0271
lipid digestion	0.0271
oxidation reduction	0.0295
mitotic sister chromatid segregation	0.0328
negative regulation of immune response	0.0328
regulation of lymphocyte proliferation	0.0345
negative regulation of B cell proliferation	0.0347
negative thymic T cell selection	0.0347
cellular localization	0.0349
positive regulation of lymphocyte differentiation	0.0353
mitosis	0.036
M phase	0.0367
immunoglobulin production	0.0369
regulation of myeloid leukocyte differentiation	0.0379
respiratory gaseous exchange	0.0379
response to DNA damage stimulus	0.0384
regulation of alpha−beta T cell activation	0.041
microtubule−based movement	0.0419
positive regulation of apoptosis	0.0419
regulation of cell cycle	0.0426
immune effector process	0.0434

↑ CR

Overrepresented Biological Processes

GO Term	P−Value
mating	0.0479
macroautophagy	0.0479
regulation of fibroblast proliferation	0.0479
response to tumor cell	0.0482
polyamine biosynthetic process	0.0482
cellular zinc ion homeostasis	0.0482
carnitine metabolic process	0.0482
response to glucose stimulus	0.0482
negative regulation of cell killing	0.0482
response to cytokine stimulus	0.0482
response to monosaccharide stimulus	0.0482
cholesterol metabolic process	0.0491
immunoglobulin mediated immune response	0.0491

↑ CR

Overrepresented Cell Components

GO Term	P−Value
spindle pole	0.00836
nuclear speck	0.00836
lateral plasma membrane	0.0105
vacuolar part	0.018
prefoldin complex	0.0187
signal peptidase complex	0.0208
microvillus membrane	0.0208
male pronucleus	0.0239
mitochondrial inner membrane	0.0272
chromosome	0.0399
female pronucleus	0.0428
intracellular part	0.0467

↑ CR

Overrepresented Molecular Functions

GO Term	P−Value
flavin−containing monooxygenase activity	0.00209
pheromone receptor activity	0.00328
oxidoreductase activity, acting on CH−OH group of donors	0.0041
structural constituent of ribosome	0.00669
acid−thiol ligase activity	0.00694
rRNA binding	0.00756
copper ion binding	0.0078
copper ion transmembrane transporter activity	0.0114
6−phosphofructo−2−kinase activity	0.0114
fructose−2,6−bisphosphate 2−phosphatase activity	0.0114
histone deacetylase binding	0.0114
estradiol 17−beta−dehydrogenase activity	0.0202
glycerol−3−phosphate dehydrogenase (NAD+) activity	0.0218
alpha−L−fucosidase activity	0.0218
growth hormone receptor binding	0.0218
interleukin−5 receptor binding	0.0218
N−acetyllactosaminide beta−1,6−N−acetylglucosaminyltransferase activity	0.0218
oxidoreductase activity, acting on paired donors, with incorporation or reduction of molecular oxygen, anot[illegible]	0.0218
tyrosine−ester sulfotransferase activity	0.0218
protein tyrosine kinase activator activity	0.0218
vitamin D receptor binding	0.0218
ribosomal large subunit binding	0.0218
3−hydroxyacyl−CoA dehydrogenase activity	0.0255
alcohol dehydrogenase activity	0.0255
iron ion transmembrane transporter activity	0.0255
ornithine decarboxylase regulator activity	0.0255
inorganic cation transmembrane transporter activity	0.028
lipid transporter activity	0.031
metalloexopeptidase activity	0.0322
hydrogen ion transporting ATP synthase activity, rotational mechanism	0.0377

↑ CR

Overrepresented Molecular Functions

GO Term	P−Value
hydrogen ion transporting ATPase activity, rotational mechanism	0.0377
receptor activity	0.0409
DNA−(apurinic or apyrimidinic site) lyase activity	0.0454
high−density lipoprotein binding	0.0454
neurexin binding	0.0454
lipid phosphatase activity	0.0475

↓ CR

Overrepresented Biological Processes

GO Term	P−Value
phosphate transport	9.77e−06
regulation of cell migration	1.97e−05
antigen processing and presentation of exogenous antigen	0.000179
antigen processing and presentation of peptide antigen	0.000339
antigen processing and presentation of exogenous peptide antigen via MHC class II	0.000356
immune response	0.000452
regulation of cell adhesion	0.000545
antigen processing and presentation of peptide antigen via MHC class I	0.000598
antigen processing and presentation of peptide or polysaccharide antigen via MHC class II	0.000598
anion transport	0.000643
biological adhesion	0.000888
protein metabolic process	0.00121
cellular macromolecule metabolic process	0.00158
peptidyl−proline hydroxylation to 4−hydroxy−L−proline	0.00169
ISG15−protein conjugation	0.00169
sequestering of actin monomers	0.00169
chaperone cofactor−dependent protein folding	0.00254
peptidyl−proline modification	0.00332
negative regulation of protein polymerization	0.00332
cell maturation	0.00368
'de novo' protein folding	0.00406
plasma membrane fusion	0.00499
cell adhesion mediated by integrin	0.00544
blood vessel development	0.0055
negative regulation of angiogenesis	0.00603
skeletal muscle fiber development	0.00695
anatomical structure formation	0.00695
regeneration	0.0072
regulation of embryonic development	0.00798
response to retinoic acid	0.00804

↓ CR

Overrepresented Biological Processes

GO Term	P−Value
response to vitamin	0.00804
cell motility	0.00845
regulation of nitrogen compound metabolic process	0.00848
negative regulation of cytoskeleton organization and biogenesis	0.00939
cell−substrate adhesion	0.0107
myoblast fusion	0.0111
metaphase	0.0111
blood vessel morphogenesis	0.0112
syncytium formation	0.0145
positive thymic T cell selection	0.0145
organ development	0.0149
developmental growth	0.0162
negative regulation of cellular component organization and biogenesis	0.0162
odontogenesis	0.0162
proteolysis	0.0166
myoblast development	0.0169
neurite regeneration	0.0184
positive regulation of Ras protein signal transduction	0.0184
antigen processing and presentation	0.0214
regulation of lymphocyte differentiation	0.0222
negative regulation of cellular metabolic process	0.0225
negative regulation of microtubule depolymerization	0.0226
regulation of synapse organization and biogenesis	0.0226
neuromuscular process controlling posture	0.0226
positive regulation of T cell differentiation	0.0233
activation of protein kinase C activity	0.0272
response to axon injury	0.0272
cell−cell adhesion	0.0292
protein folding	0.0297
muscle development	0.0301

↓ CR

Overrepresented Biological Processes

GO Term	P−Value
positive regulation of cell differentiation	0.0314
membrane organization and biogenesis	0.0321
positive regulation of biological process	0.033
regulation of angiogenesis	0.0368
vasculogenesis	0.0372
protein complex assembly	0.0393
in utero embryonic development	0.0398
regulation of microtubule polymerization or depolymerization	0.043
protein kinase cascade	0.0454
regulation of catalytic activity	0.0455
negative regulation of protein metabolic process	0.0466
biopolymer catabolic process	0.0477
amino acid derivative metabolic process	0.0487
polyamine metabolic process	0.0488
lysosome organization and biogenesis	0.0488

↓CR

Overrepresented Cell Components

GO Term	P−Value
extracellular matrix	1.66e−05
collagen	4.61e−05
external side of plasma membrane	0.000697
cytoplasm	0.000783
MHC class II protein complex	0.000815
basal lamina	0.00155
MHC class I protein complex	0.00325
lysosome	0.00437
laminin−1 complex	0.00466
protein complex	0.00626
collagen type IV	0.0069
vacuole	0.00845
cytosol	0.00946
multivesicular body	0.0125
plasma membrane	0.0282
endomembrane system	0.0314
basement membrane	0.0318
extracellular space	0.0423

↓ CR

Overrepresented Molecular Functions

GO Term	P−Value
extracellular matrix structural constituent	1.2e−05
unfolded protein binding	0.00173
hydrolase activity, acting on glycosyl bonds	0.00214
protein C−terminus binding	0.00215
procollagen−proline 4−dioxygenase activity	0.00296
peptide antigen binding	0.00296
aminopeptidase activity	0.00296
GTPase activity	0.00343
adenyl nucleotide binding	0.00363
purine ribonucleotide binding	0.00427
ATP binding	0.00612
nucleotide binding	0.00672
hydrolase activity, acting on acid anhydrides	0.00688
glucosidase activity	0.00717
L−ascorbic acid binding	0.00843
manganese ion binding	0.00926
integrin binding	0.00976
peptidyl−proline dioxygenase activity	0.00989
protein binding	0.0109
vascular endothelial growth factor receptor activity	0.013
pyrophosphatase activity	0.0132
catalytic activity	0.0141
actin filament binding	0.0202
diacylglycerol kinase activity	0.0289
mannosidase activity	0.0336
calmodulin binding	0.0355
beta−catenin binding	0.0386
peptidyl−prolyl cis−trans isomerase activity	0.0407
mRNA binding	0.0438
chemokine activity	0.0438

Gene Ontology Profile Comparison (Biological Process Ontology)

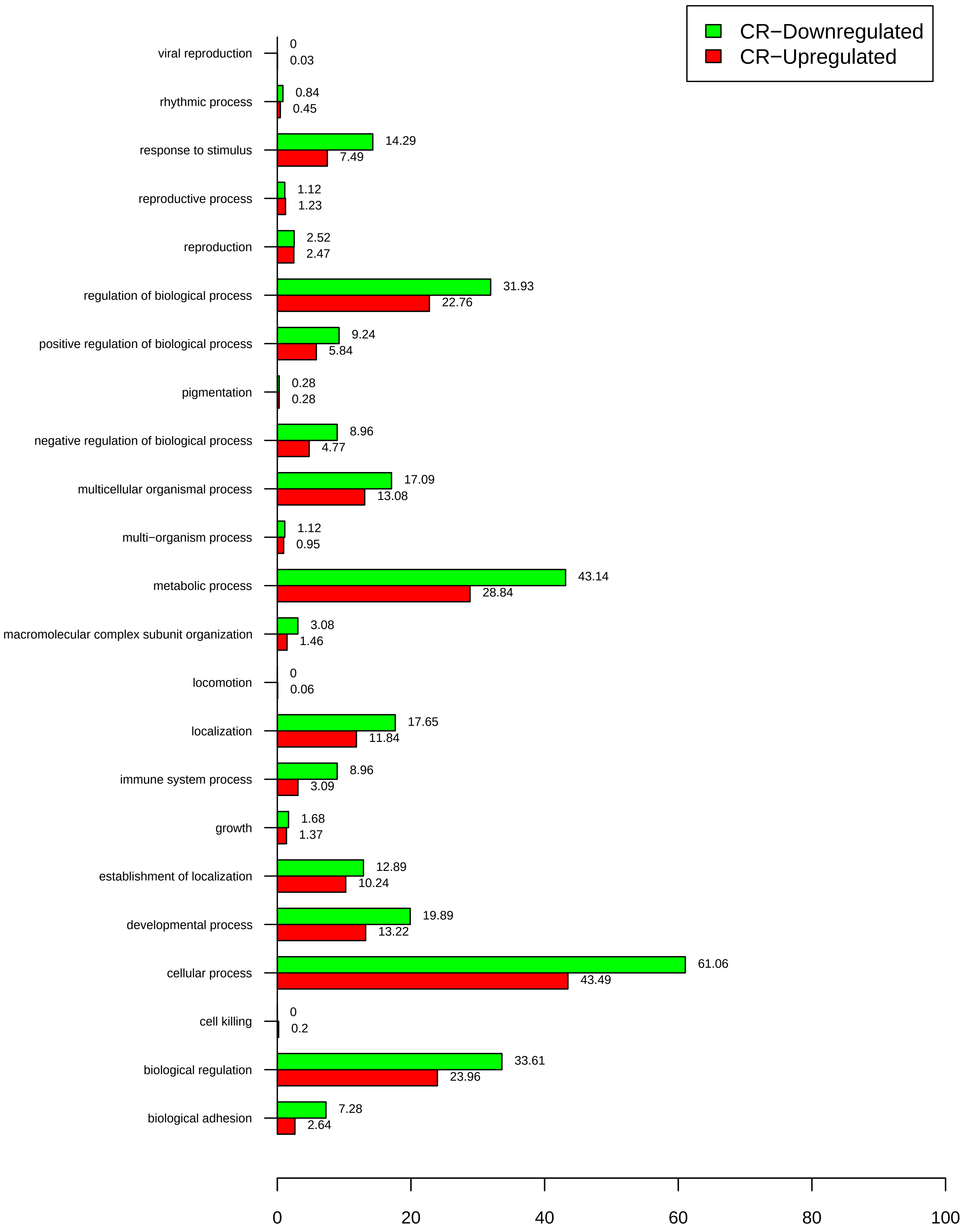

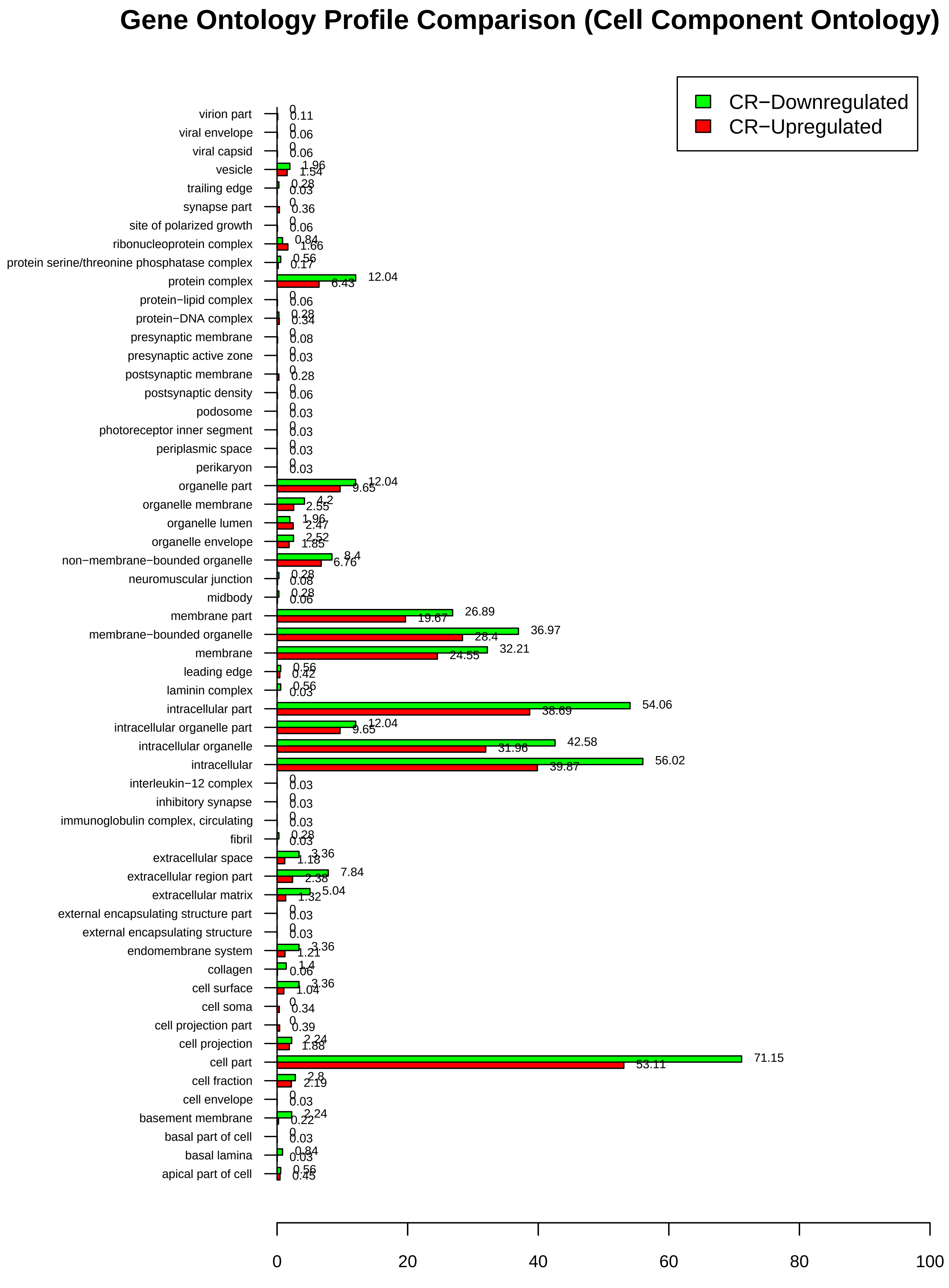
Gene Ontology Profile Comparison (Cell Component Ontology)
CR−Downregulated
CR−Upregulated
virion part 0 0.11
viral envelope 0 0.06
viral capsid 0 0.06
vesicle 1.96 1.54
trailing edge 0.28 0.03
synapse part 0 0.36
site of polarized growth 0 0.06
ribonucleoprotein complex 0.84 1.66
protein serine/threonine phosphatase complex 0.56 0.17
protein complex 12.04 6.43
protein−lipid complex 0 0.06
protein−DNA complex 0.28 0.34
presynaptic membrane 0 0.08
presynaptic active zone 0 0.03
postsynaptic membrane 0 0.28
postsynaptic density 0 0.06
podosome 0 0.03
photoreceptor inner segment 0 0.03
periplasmic space 0 0.03
perikaryon 0 0.03
organelle part 12.04 9.65
organelle membrane 4.2 2.55
organelle lumen 1.96 2.47
organelle envelope 2.52 1.85
non−membrane−bounded organelle 8.4 6.76
neuromuscular junction 0.28 0.08
midbody 0.28 0.06
membrane part 26.89 19.67
membrane−bounded organelle 36.97 28.4
membrane 32.21 24.55
leading edge 0.56 0.42
laminin complex 0.56 0.03
intracellular part 54.06 38.69
intracellular organelle part 12.04 9.65
intracellular organelle 42.58 31.96
intracellular 56.02 39.87
interleukin−12 complex 0 0.03
inhibitory synapse 0 0.03
immunoglobulin complex, circulating 0 0.03
fibril 0.28 0.03
extracellular space 3.36 1.18
extracellular region part 7.84 2.38
extracellular matrix 5.04 1.32
external encapsulating structure part 0 0.03
external encapsulating structure 0 0.03
endomembrane system 3.36 1.21
collagen 1.4 0.06
cell surface 3.36 1.04
cell soma 0 0.34
cell projection part 0 0.39
cell projection 2.24 1.88
cell part 71.15 53.11
cell fraction 2.8 2.19
cell envelope 0 0.03
basement membrane 2.24 0.22
basal part of cell 0 0.03
basal lamina 0.84 0.03
apical part of cell 0.56 0.45
0
20
40
60
80
100

Gene Ontology Profile Comparison (Molecular Function Ontology)

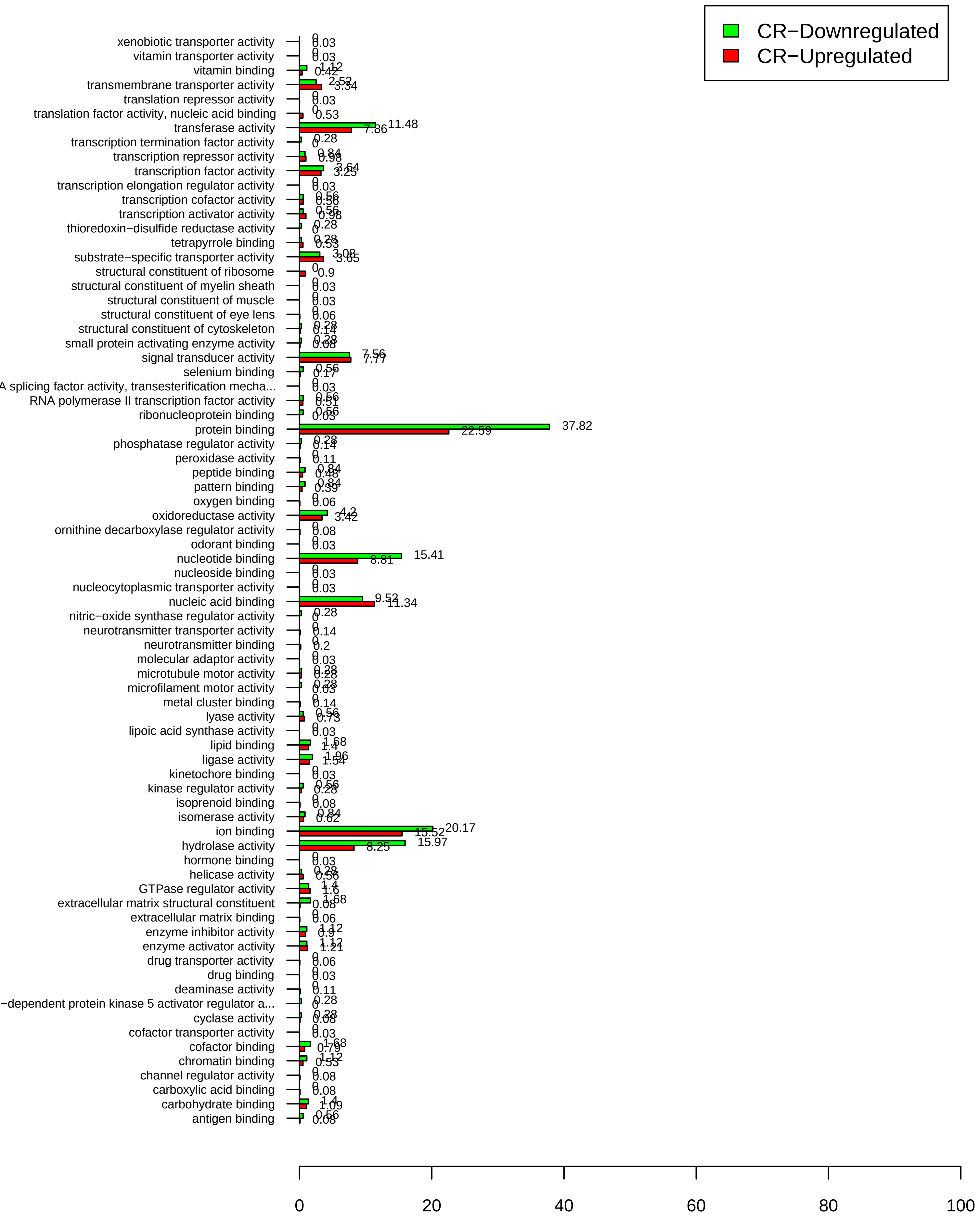

↑ CR

Overrepresented KEGG Pathways

GO Term	P−Value
p53 signaling pathway	0.000624
Cell cycle	0.00134
Butanoate metabolism	0.00592
T cell receptor signaling pathway	0.00768
Maturity onset diabetes of the young	0.00953
Ribosome	0.0218
PPAR signaling pathway	0.024
Long−term potentiation	0.0253
Chronic myeloid leukemia	0.0262
Wnt signaling pathway	0.0275
Phosphatidylinositol signaling system	0.0278
Valine, leucine and isoleucine degradation	0.0279
Olfactory transduction	0.029
Glioma	0.0313
B cell receptor signaling pathway	0.0363
Melanoma	0.045
Colorectal cancer	0.0472
Long−term depression	0.0479

↓ CR

Overrepresented KEGG Pathways

GO Term	P−Value
ECM−receptor interaction	5.09e−07
Antigen processing and presentation	6.82e−07
Cell Communication	4.16e−05
Focal adhesion	0.000202
Cell adhesion molecules (CAMs)	0.0148
Type I diabetes mellitus	0.0218
Small cell lung cancer	0.0277
Neurodegenerative Diseases	0.0404

↑ CR

Overrepresented KEGG Pathways

(Based on InterPro Domain Signatures)

GO Term	P−Value
Pyruvate metabolism	0.0099
Glycolysis / Gluconeogenesis	0.0134
Cell cycle	0.0192
Axon guidance	0.0292
Allograft rejection	0.0309
p53 signaling pathway	0.0339
Glycosylphosphatidylinositol(GPI)−anchor biosynthesis	0.034
One carbon pool by folate	0.0461

↓ CR

Overrepresented KEGG Pathways

(Based on InterPro Domain Signatures)

GO Term	P−Value
ECM−receptor interaction	0.0035
Cell Communication	0.0078
Ribosome	0.0081
N−Glycan biosynthesis	0.0086
Glycan structures − degradation	0.0086
Antigen processing and presentation	0.0107
N−Glycan degradation	0.0131
Cell adhesion molecules (CAMs)	0.015
Oxidative phosphorylation	0.0233
Prion disease	0.0298
Notch signaling pathway	0.0307
Starch and sucrose metabolism	0.0328
Glycosphingolipid biosynthesis − globoseries	0.0334
Asthma	0.036
Glycosaminoglycan degradation	0.0373
p53 signaling pathway	0.038
Glycan structures − biosynthesis 1	0.0394
Glycosylphosphatidylinositol(GPI)−anchor biosynthesis	0.0459

↑ CR

Abundance of miRNA Targets

miRNA	Freq(Obs)	Freq(Exp)	Obs/Exp	P−value	P−Value(Adj)
miR−340−3p	0.0521	0.0429	1.21	0.0134	1
miR−92a	0.0907	0.0788	1.15	0.0149	1
miR−369−3p	0.122	0.11	1.11	0.0314	1
miR−341	0.00629	0.00419	1.5	0.0456	1
miR−92b	0.0831	0.0753	1.1	0.0711	1
miR−365	0.07	0.063	1.11	0.0722	1
miR−208a	0.0539	0.0481	1.12	0.084	1
miR−25	0.103	0.0952	1.08	0.0841	1
miR−142−5p	0.192	0.182	1.06	0.0904	1
miR−19a	0.179	0.169	1.06	0.0941	1

↓ CR

Abundance of miRNA Targets

miRNA	Freq(Obs)	Freq(Exp)	Obs/Exp	P−value	P−Value(Adj)
miR−705	0.193	0.14	1.39	0.00554	1
miR−449b	0.171	0.123	1.39	0.00868	1
miR−1188	0.126	0.0873	1.45	0.0113	1
miR−423−5p	0.186	0.141	1.32	0.0153	1
miR−34a	0.182	0.138	1.32	0.0173	1
miR−540−3p	0.115	0.0811	1.42	0.0188	1
miR−682	0.138	0.103	1.34	0.0269	1
miR−149	0.175	0.136	1.29	0.0286	1
miR−615−5p	0.1	0.0721	1.39	0.0325	1
miR−770−3p	0.0892	0.0634	1.41	0.0359	1
miR−29b	0.16	0.125	1.28	0.0366	1
miR−532−3p	0.13	0.0988	1.32	0.0377	1
miR−23b	0.178	0.142	1.25	0.0399	1
miR−685	0.123	0.0928	1.32	0.0402	1
miR−670	0.134	0.103	1.3	0.0428	1
miR−1224	0.145	0.113	1.28	0.0433	1
miR−29c	0.141	0.111	1.28	0.0481	1
miR−485	0.16	0.127	1.25	0.0488	1

Tests for Chromosome Over−representation

Chromosome	CR−upregulated Genes	CR−downregulated Genes
1	0.997	0.845
2	1.00	0.663
3	1.00	0.889
4	1.00	0.508
5	0.995	0.495
6	0.894	0.961
7	1.00	0.00768*
8	1.00	0.027*
9	1.00	0.214
10	0.98	0.032*
11	1.00	0.0386*
12	0.969	0.982
13	0.972	0.972
14	0.998	0.845
15	0.985	0.197
16	0.997	0.708
17	1.00	0.115
18	0.933	0.931
19	0.999	0.0149*
X	0.849	0.842
Y	0.378	1.00

The table lists p−values generated from a test that evaluates whether there exists an over−abundance of identified genes with respect to a given chromosome. The null hypothesis assumes that the set of genes has been selected at random from those represented on the Affymetrix 430 2.0 array. A significant test indicates that a chromosome contains more of the identified genes than would be expected if the gene set had been chosen at random.

* = significant p−value, without multiple test adjustment

** = significant p−value, with multiple test adjustment

Chromosome Locations

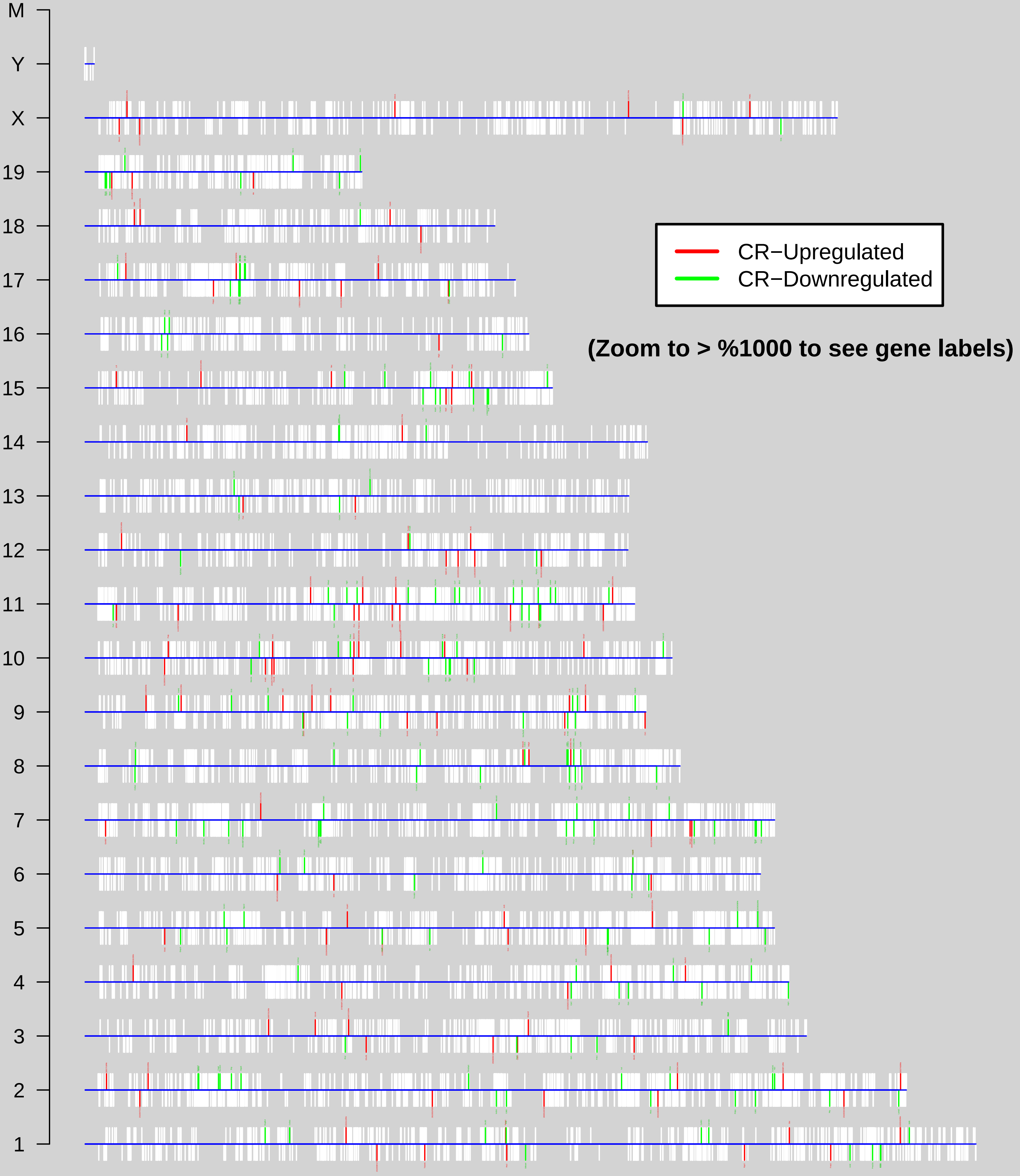

Supplement: Additional file 3 — Genes regulated by caloric restriction in heart. Results from 7 experiments are analyzed to identify genes significantly up and down regulated by CR in heart. This file also includes analysis of associated gene ontology terms, KEGG pathways, microRNA targets and chromosomal locations of CR-regulated genes. [file 1471-2164-10-585-S3.PDF]
