# Supplementary material for: Genes and gene expression modules associated with caloric restriction and aging in the laboratory mouse
Source: BMC Genomics. 2009 Dec 7;10:585. doi: 10.1186/1471-2164-10-585 (PMC2795771; doi:10.1186/1471-2164-10-585)
Supplement: Additional file 4 — Genes regulated by caloric restriction in muscle. Results from 4 experiments are analyzed to identify genes significantly up and down regulated by CR in muscle. This file also includes analysis of associated gene ontology terms, KEGG pathways, microRNA targets and chromosomal locations of CR-regulated genes. [file 1471-2164-10-585-S4.PDF]

# Additional File 4

## Genes and Gene Expression Modules Associated with Caloric Restriction and Aging in the Laboratory Mouse

*William R. Swindell*
*University of Michigan, Departments of Pathology and Geriatrics*

---

## Genes Regulated by Caloric Restriction in Muscle

This file provides information on genes significantly influenced by CR in muscle. The first set of charts displays differential expression results of the 200 genes most strongly up regulated by CR in muscle, while the second set of charts displays differential expression results for the 200 genes most strongly down regulated by CR in muscle. Each row corresponds to an individual gene and each column corresponds to a separate experiment (see Additional File 1). Symbols are interpreted as follows:

- ● (red) Gene is significantly up regulated by CR ($P_u < 0.05$)
- ● (green) Gene is significantly down regulated by CR ($P_d < 0.05$)
- — (red) Gene is marginally up regulated by CR ($0.05 < P_u < 0.10$)
- — (green) Gene is marginally down regulated by CR ($0.05 < P_d < 0.10$)
- — (black) Non-significant CR effect ($P_u > 0.10$ and $P_d > 0.10$)
- × No data (gene not represented in experiment or array annotation was limiting)
- ✳ (red) Evidence conflicts but favors up regulation by CR
- ✳ (green) Evidence conflicts, but favors down regulation by CR

The last two categories (✳ and ✳) indicate significant effects with conflicting evidence. This can arise if multiple transcripts associated with the same gene symbol yield opposite conclusions. Alternatively, a conflict may arise if $P_u < 0.05$ and also $P_d < 0.05$. Symbols shown in charts are based upon a comparison-wise type I error rate of 0.05. The final column in each chart lists meta-analysis p-values generated using Fisher's method, which have been adjusted using the Benjamini-Hochberg method to control the false discovery rate among all 21,327 genes.

The remainder of the file includes lists of over-represented gene ontology terms, over-represented KEGG pathways, and over-represented KEGG pathways defined based upon IP domain signatures (see Hahne et al. 2008, BMC Bioinformatics 9:3). Genes were also analyzed to determine if there existed an over-abundance of targets for certain microRNAs (see Betel et al. 2008, Nucleic Acids Res. 36: D149-153), and a list of associated microRNAs is provided based upon this analysis. Lastly, tests for over-representation of identified genes with respect to each chromosome were performed, and an idiogram mapping of identified genes to chromosomal locations is shown.

---

**Contact: William R. Swindell, wswindel@umich.edu**

↑ CR

## Genes up regulated by CR

| | msl1 | msl3 | msl15 | msl16 | $P_u$ |
|---|---|---|---|---|---|
| Mylc2pl | ● | ● | ● | ● | 0.000704 |
| Snw1 | ● | ● | ● | − | 0.00214 |
| Acads | ● | ● | ● | × | 0.00214 |
| Nfs1 | ● | ● | * (red) | × | 0.00221 |
| Trabd | ● | ● | ● | × | 0.00291 |
| Aqp4 | ● | ● | * (green) | ● | 0.00312 |
| Atp6v1h | ● | ● | ● | × | 0.00312 |
| Mea1 | ● | ● | ● | − | 0.00312 |
| Mul1 | ● | ● | * (green) | × | 0.00312 |
| Rad23a | ● | ● | ● | × | 0.00312 |
| Snai3 | ● | ● | ● | − | 0.00312 |
| Tsc22d3 | ● | ● | * (green) | × | 0.00312 |
| Adh1 | ● | ● | ● | × | 0.00312 |
| Ap2s1 | ● | ● | ● | × | 0.00312 |
| Gabarap | ● | ● | ● | − | 0.00312 |
| 1110034G24Rik | ● | × | ● | × | 0.00331 |
| 1110059G02Rik | ● | × | ● | × | 0.00331 |
| 2310035K24Rik | ● | ● | ● | − (red) | 0.00331 |
| 2310050B05Rik | ● | × | ● | × | 0.00331 |
| 2310076L09Rik | ● | − | ● | × | 0.00331 |
| 2810001A02Rik | ● | × | ● | × | 0.00331 |
| 4833442J19Rik | ● | − | ● | × | 0.00331 |
| 8430408G22Rik | ● | × | ● | × | 0.00331 |
| 9030612M13Rik | ● | ● (green) | * (green) | × | 0.00331 |
| A430108E01Rik | ● | × | ● | × | 0.00331 |
| Abcf2 | ● | ● | − (red) | × | 0.00331 |
| Acly | ● | ● | * (green) | − | 0.00331 |
| Acot2 | ● | ● | ● | × | 0.00331 |
| Add1 | ● | ● | * (green) | × | 0.00331 |
| Ankrd39 | ● | × | ● | − | 0.00331 |

↑ CR

## Genes up regulated by CR

| | msl1 | msl3 | msl15 | msl16 | $P_u$ |
|---|---|---|---|---|---|
| Arrdc3 | ● (red) | × | ● (red) | × | 0.00331 |
| Art5 | ● (red) | ● (red) | ● (red) | × | 0.00331 |
| BC055107 | ● (red) | × | ● (red) | – | 0.00331 |
| Bcl2l1 | ● (red) | ● (red) | ● (red) | – | 0.00331 |
| Camk2a | ● (green) | ● (red) | * (red) | ● (red) | 0.00331 |
| Cblb | ● (red) | × | ● (red) | × | 0.00331 |
| Cops6 | ● (red) | ● (red) | ● (red) | – | 0.00331 |
| Cpne3 | ● (red) | – (green) | ● (red) | × | 0.00331 |
| D16H22S680E | ● (red) | – | ● (red) | × | 0.00331 |
| D19Wsu162e | ● (red) | ● (red) | – (red) | × | 0.00331 |
| D1Ertd53e | ● (red) | – (green) | ● (red) | × | 0.00331 |
| Dnm2 | ● (red) | ● (red) | ● (red) | × | 0.00331 |
| Elmod3 | ● (red) | – | ● (red) | × | 0.00331 |
| Epc1 | ● (red) | – | ● (red) | × | 0.00331 |
| Ercc8 | ● (red) | ● (red) | * (red) | × | 0.00331 |
| Glb1l2 | ● (red) | × | ● (red) | – | 0.00331 |
| Gpd1 | ● (red) | ● (red) | ● (red) | × | 0.00331 |
| Jph1 | ● (red) | ● (red) | ● (green) | ● (red) | 0.00331 |
| Keap1 | ● (red) | ● (red) | ● (red) | × | 0.00331 |
| Klhdc6 | ● (red) | × | ● (red) | × | 0.00331 |
| Ksr1 | ● (red) | ● (red) | * (green) | × | 0.00331 |
| Lonrf3 | ● (red) | × | ● (red) | × | 0.00331 |
| Manbal | ● (red) | ● (red) | ● (red) | × | 0.00331 |
| Mef2b | ● (red) | – (red) | ● (red) | × | 0.00331 |
| Mettl1 | ● (red) | – (red) | ● (red) | × | 0.00331 |
| Mrps5 | ● (red) | ● (red) | * (green) | × | 0.00331 |
| Ndufs4 | * (red) | ● (red) | ● (red) | × | 0.00331 |
| Nrg4 | ● (red) | ● (red) | ● (red) | × | 0.00331 |
| Pde4dip | ● (red) | ● (red) | * (red) | – | 0.00331 |
| Phtf2 | ● (red) | ● (green) | * (red) | – | 0.00331 |

↑ CR

## Genes up regulated by CR

| | msl1 | msl3 | msl15 | msl16 | $P_u$ |
|---|---|---|---|---|---|
| Ppp1r11 | * | ● | ● | – | 0.00331 |
| Prep | ● | ● | * | × | 0.00331 |
| Rbm12 | ● | ● | * (green) | × | 0.00331 |
| Rbm8a | ● | ● | ● | – | 0.00331 |
| Rell1 | ● | ● (green) | ● | ● | 0.00331 |
| Sap30bp | ● | – (red) | ● | – | 0.00331 |
| Slc44a2 | ● | ● | * (green) | × | 0.00331 |
| Srp72 | ● | × | * | – | 0.00331 |
| Stx8 | ● | ● | ● | × | 0.00331 |
| Timp3 | ● | * | ● | × | 0.00331 |
| Tkt | ● | ● | ● | × | 0.00331 |
| Twistnb | ● | × | ● | – | 0.00331 |
| Ube2g2 | ● | ● | ● | × | 0.00331 |
| Ubl4 | ● | ● | ● | – | 0.00331 |
| Ucp3 | ● | ● | ● | × | 0.00331 |
| Wtap | ● | ● | ● | × | 0.00331 |
| Zfand5 | ● | ● (green) | * | × | 0.00331 |
| Zkscan1 | ● | – | * | × | 0.00331 |
| 1420312_s_at | ● | × | ● | × | 0.00331 |
| 1435833_at | ● | – (red) | ● | × | 0.00331 |
| 1444750_at | ● | × | ● | × | 0.00331 |
| 1457438_at | ● | × | ● | × | 0.00331 |
| 1459557_at | ● | × | ● | × | 0.00331 |
| 1700021F05Rik | – | ● | ● | ● | 0.00331 |
| 2310015A10Rik | ● | × | ● | × | 0.00331 |
| 2700078K21Rik | ● | ● | ● | × | 0.00331 |
| 2810427A07Rik | ● | × | ● | × | 0.00331 |
| 2900010J23Rik | – | ● | ● | × | 0.00331 |
| 4933414I06Rik | ● | × | ● | × | 0.00331 |
| 545228 | ● | × | ● | × | 0.00331 |

↑ CR

## Genes up regulated by CR

| | msl1 | msl3 | msl15 | msl16 | $P_u$ |
|---|---|---|---|---|---|
| 6030422H21Rik | ● | × | ● | × | 0.00331 |
| 6330548G22Rik | ● | × | ● | × | 0.00331 |
| Arrdc2 | ● | × | ● | × | 0.00331 |
| Asna1 | ● | ● | ● | – | 0.00331 |
| Atp5e | – | ● | ● | ● | 0.00331 |
| BC050254 | ● | × | ● | × | 0.00331 |
| Bnip1 | ● | ● | ● | – | 0.00331 |
| C230066K19Rik | ● | × | ● | × | 0.00331 |
| Dusp26 | ● | ● | – | ● | 0.00331 |
| Eapp | ● | ● | ● | – | 0.00331 |
| Gnmt | ● | ● | ● | × | 0.00331 |
| Gpihbp1 | ● | × | ● | × | 0.00331 |
| H2−Ke2 | ● | ● | ● | × | 0.00331 |
| H2−Q10 | ● | ● | ● | × | 0.00331 |
| Lgals4 | ● | ● | ● | × | 0.00331 |
| LOC100039864 | ● | × | ● | × | 0.00331 |
| Lrrc40 | ● | ● | ● | – | 0.00331 |
| Mcf2l | ● | × | ● | × | 0.00331 |
| Mm.152838 | ● | × | ● | × | 0.00331 |
| Mm.427936 | ● | × | ● | × | 0.00331 |
| Mm.440621 | ● | × | ● | × | 0.00331 |
| Mm.445012 | ● | × | ● | × | 0.00331 |
| Mm.453308 | ● | × | ● | × | 0.00331 |
| Mrpl2 | ● | ● | ● | × | 0.00331 |
| Mterfd2 | ● | ● | ● | × | 0.00331 |
| Phpt1 | ● | ● | ● | – | 0.00331 |
| Ptprr | ● | ● | ● | × | 0.00331 |
| Sat1 | ● | ● | ● | × | 0.00331 |
| Slc25a34 | ● | × | ● | × | 0.00331 |
| Stk11ip | ● | ● | ● | × | 0.00331 |

↑ CR

## Genes up regulated by CR

| | msl1 | msl3 | msl15 | msl16 | $P_u$ |
|---|---|---|---|---|---|
| Syde2 | ● | × | ● | × | 0.00331 |
| Trit1 | ● | ● | ● | × | 0.00331 |
| Ube2t | ● | ● | ● | × | 0.00331 |
| 4833418N02Rik | ● | × | ● | × | 0.00341 |
| Hemk1 | ● | ● | − | ● | 0.00341 |
| Mdh1 | * | − | * | × | 0.00341 |
| Prpf39 | ● | ● | * | − | 0.00341 |
| Tbrg4 | ● | ● | * | × | 0.00343 |
| D10Ertd641e | * | − | ● | − | 0.00353 |
| 5730411F24Rik | ● | × | ● | × | 0.00353 |
| Thrsp | ● | ● | − | − | 0.00358 |
| 1445443_at | ● | × | ● | × | 0.00358 |
| AI607873 | ● | × | ● | × | 0.00358 |
| Mm.411941 | ● | × | ● | × | 0.00358 |
| 1810011O10Rik | ● | ● | ● | − | 0.0036 |
| S100a1 | − | ● | ● | − | 0.00371 |
| Smcr7 | ● | × | ● | × | 0.00371 |
| Slc25a25 | ● | ● | ● | − | 0.00373 |
| 1444376_at | ● | × | ● | × | 0.0038 |
| Mpa2l | − | ● | ● | × | 0.00388 |
| LOC100047583 | ● | × | ● | × | 0.00388 |
| Arfgap2 | ● | ● | ● | × | 0.00394 |
| 2900002J02Rik | ● | × | ● | × | 0.00394 |
| Selenbp2 | ● | ● | − | × | 0.00394 |
| 1200016E24Rik | ● | − | * | × | 0.00402 |
| Gna13 | ● | ● | * | × | 0.00416 |
| N6amt1 | ● | × | * | × | 0.00416 |
| Dgat1 | ● | − | ● | × | 0.00418 |
| Rpo1−1 | ● | ● | ● | − | 0.00435 |
| Cd163 | ● | − | ● | × | 0.00435 |

↑ CR

## Genes up regulated by CR

| | msl1 | msl3 | msl15 | msl16 | $P_u$ |
|---|---|---|---|---|---|
| Fusip1 | ● (red) | ● (red) | * (red) | × | 0.00436 |
| Vamp5 | ● (green) | ● (red) | * (red) | × | 0.00437 |
| Ppp1r1a | ● (red) | ● (red) | – | ● (red) | 0.00448 |
| Atad3a | ● (red) | – | ● (red) | × | 0.0045 |
| Snord22 | ● (red) | – (red) | ● (red) | × | 0.0045 |
| 9630025I21Rik | ● (red) | × | ● (red) | × | 0.0045 |
| Msl31 | – | ● (red) | ● (red) | × | 0.0045 |
| Pcx | ● (red) | ● (red) | – | × | 0.0045 |
| Cenpb | ● (red) | ● (red) | ● (red) | – | 0.0045 |
| 1810032O08Rik | ● (red) | – | ● (red) | × | 0.00454 |
| 9430098F02Rik | ● (red) | × | ● (red) | × | 0.00454 |
| Jmjd3 | ● (red) | × | ● (red) | × | 0.00468 |
| 1441722_at | ● (red) | × | ● (red) | × | 0.00472 |
| Spnb2 | ● (red) | ● (red) | * (red) | – | 0.00473 |
| Nmb | ● (red) | – | ● (red) | – | 0.00473 |
| Gas5 | ● (red) | – (green) | * (red) | × | 0.00484 |
| Hmgcl | ● (red) | ● (red) | ● (red) | × | 0.0049 |
| 100039204 | – | * (red) | ● (red) | × | 0.00498 |
| Sorbs3 | ● (red) | ● (red) | ● (red) | – | 0.00499 |
| Mm.441301 | ● (red) | × | ● (red) | × | 0.00502 |
| 2410006H16Rik | ● (red) | × | ● (red) | × | 0.00502 |
| Mm.442478 | ● (red) | × | ● (red) | × | 0.00502 |
| Mm.442564 | ● (red) | × | ● (red) | × | 0.00502 |
| 1444591_at | ● (red) | × | ● (red) | × | 0.00526 |
| C920006O11Rik | ● (red) | × | ● (red) | × | 0.00536 |
| Dclre1b | ● (red) | ● (red) | ● (red) | × | 0.00536 |
| Slc2a4 | ● (red) | ● (red) | ● (red) | ● (red) | 0.00536 |
| Tmem109 | * (green) | ● (red) | * (red) | – | 0.00536 |
| Plekhj1 | ● (red) | ● (red) | ● (green) | ● (red) | 0.00536 |
| Araf | * (red) | ● (red) | ● (green) | × | 0.00536 |

↑ CR

## Genes up regulated by CR

| | msl1 | msl3 | msl15 | msl16 | $P_u$ |
|---|---|---|---|---|---|
| **Mgll** | ● | ● | ● | × | **0.00536** |
| **Cnih4** | ● | − | ● | × | **0.00538** |
| **D1Bwg0212e** | ● | × | * | × | **0.00538** |
| **Glrx2** | ● | − | ● | × | **0.00538** |
| **Ptrh2** | ● | ● | ● | × | **0.00538** |
| **Plcb3** | ● | ● | ● | × | **0.00538** |
| **Pxmp2** | ● | ● | ● | − | **0.00538** |
| **Exosc8** | ● | × | ● | − | **0.00545** |
| **Cdk5** | ● | ● | − | − | **0.00552** |
| **Mm.474787** | ● | × | ● | × | **0.00552** |
| **Mm.442565** | ● | × | ● | × | **0.00556** |
| **Reep2** | − (red) | ● | ● | × | **0.00556** |
| **BC054059** | ● | ● | − | − | **0.00558** |
| **Dusp18** | ● | ● | ● | − | **0.00569** |
| **Glul** | ● | − (red) | ● | − | **0.00569** |
| **Dctn2** | ● | ● | ● | × | **0.00578** |
| **Kcnj11** | ● | ● | * (green) | × | **0.00578** |
| **Scd1** | ● | ● | − | − (red) | **0.00578** |
| **Zbtb8os** | ● (green) | ● | ● | × | **0.00578** |
| **1441765_at** | ● | × | ● | × | **0.00578** |

↓ CR

## Genes down regulated by CR

| | msl1 | msl3 | msl15 | msl16 | $P_d$ |
|---|---|---|---|---|---|
| Slc38a4 | ● | ● | ● | ● | 0.000443 |
| Acvr1 | ● | ● | ● | − | 0.00187 |
| Adcy9 | ● | ● | ● | − | 0.00187 |
| Akirin1 | ● | ● | ● | ● | 0.00187 |
| Arhgap24 | ● | ● | ● | × | 0.00187 |
| Arntl | ● | ● | ● | ● | 0.00187 |
| Cpeb4 | ● | ● | ● | × | 0.00187 |
| Dag1 | * | ● | ● | − | 0.00187 |
| Ets1 | ● | ● | ● | × | 0.00187 |
| Itpr1 | ● | ● | ● | − | 0.00187 |
| Npnt | ● | * | ● | × | 0.00187 |
| Nrarp | ● | ● | ● | ● | 0.00187 |
| Nrp1 | ● | ● | ● | − | 0.00187 |
| Nudt4 | ● | ● | ● | × | 0.00187 |
| Prnp | ● | ● | ● | ● | 0.00187 |
| Siah2 | ● | ● | ● | − | 0.00187 |
| Cpeb1 | ● | − | ● | ● | 0.00187 |
| Actr3 | ● | ● | ● | × | 0.00195 |
| Aurka | ● | ● | ● | ● | 0.00195 |
| Calm1 | ● | ● | ● | × | 0.00195 |
| Cd164 | ● | ● | ● | − | 0.00195 |
| Mtmr1 | − | ● | ● | ● | 0.00195 |
| Ppm1a | ● | ● | * | × | 0.00195 |
| Stam2 | ● | * | * | × | 0.00195 |
| Styx | ● | ● | ● | × | 0.00195 |
| En1 | ● | ● | ● | × | 0.00195 |
| Klhl30 | ● | × | ● | ● | 0.00195 |
| Six2 | ● | ● | ● | × | 0.00195 |
| Aplnr | ● | ● | ● | × | 0.00202 |
| Tmem123 | ● | ● | ● | × | 0.00208 |

↓ CR

## Genes down regulated by CR

| | msl1 | msl3 | msl15 | msl16 | $P_d$ |
|---|---|---|---|---|---|
| Galnt7 | ● | ● | ● | × | 0.00228 |
| Amd1 | ● | ● | ● | × | 0.00262 |
| Arfgef1 | ● | ● | * (red) | × | 0.00262 |
| Arhgef12 | ● | ● | ● | × | 0.00262 |
| Dnajc3 | ● | ● | ● | × | 0.00262 |
| Igf1 | ● | ● | ● | – | 0.00262 |
| Rrad | ● | ● | ● | × | 0.00262 |
| Fbxo28 | ● | ● | ● | ● | 0.00262 |
| Marcks | ● | ● | * (green) | – | 0.00262 |
| Ranbp10 | * (red) | ● | ● | ● | 0.00265 |
| Samd8 | ● | ● | ● | × | 0.00265 |
| Exoc7 | – | ● | ● | ● | 0.00265 |
| Cxxc5 | ● | ● | ● | × | 0.00273 |
| Lamc1 | ● | ● | ● | – | 0.00273 |
| Larp5 | ● | ● | ● | × | 0.00273 |
| Pofut2 | * (green) | * (red) | ● | – | 0.00273 |
| Rras2 | ● | ● | ● | × | 0.00273 |
| Sertad2 | ● | ● | ● | × | 0.00273 |
| Sh3bp5 | ● | ● | ● | – | 0.00273 |
| Sap30 | ● | ● | ● | – | 0.00273 |
| Cdyl | ● | ● | ● | × | 0.00305 |
| Psd3 | ● | ● | ● | × | 0.00305 |
| Slc2a3 | – | ● | ● | ● | 0.00305 |
| Trio | ● | ● | * (green) | × | 0.00305 |
| Mm.393827 | ● | ● | ● | × | 0.00305 |
| Rfwd2 | ● | ● | ● | × | 0.00305 |
| 1110002E22Rik | ● | × | ● | × | 0.00307 |
| 1110021J02Rik | ● | – | * (red) | × | 0.00307 |
| 1190002N15Rik | ● | ● | ● | × | 0.00307 |
| 1500005K14Rik | ● | – (green) | ● | × | 0.00307 |

↓ CR

## Genes down regulated by CR

| | msl1 | msl3 | msl15 | msl16 | $P_d$ |
|---|---|---|---|---|---|
| 2310076G13Rik | ● | × | * | × | 0.00307 |
| 2610030H06Rik | * | ● | ● | × | 0.00307 |
| 5430433E21Rik | ● | × | ● | × | 0.00307 |
| App | – (green) | ● | ● | × | 0.00307 |
| Atg5 | ● (red) | ● | ● | × | 0.00307 |
| Auts2 | ● | – (black) | ● | × | 0.00307 |
| BB001228 | ● | ● | * | × | 0.00307 |
| Btbd7 | ● | × | * (red) | × | 0.00307 |
| Casp12 | ● | ● | ● | × | 0.00307 |
| Ccdc69 | ● | × | ● | × | 0.00307 |
| Cdk2ap1 | ● | ● | * | × | 0.00307 |
| Clint1 | – (green) | ● | ● | × | 0.00307 |
| Clock | ● | ● | * (red) | × | 0.00307 |
| Commd3 | ● | × | ● | – (black) | 0.00307 |
| Cpd | ● (red) | * | ● | × | 0.00307 |
| Ctsc | ● | ● | ● | × | 0.00307 |
| Ddx3y | ● | ● | ● | × | 0.00307 |
| Dyrk2 | ● | × | ● | × | 0.00307 |
| Etl4 | ● | – (black) | * (red) | ● | 0.00307 |
| Fchsd2 | ● | × | ● | × | 0.00307 |
| Fgd4 | ● | ● (red) | ● | × | 0.00307 |
| Fkbp9 | ● | ● | * | ● | 0.00307 |
| Golph3 | * | ● (red) | ● | × | 0.00307 |
| Ibtk | ● | * | ● | × | 0.00307 |
| Igfbp5 | ● | ● | * | × | 0.00307 |
| Ints3 | – (black) | ● | ● | ● | 0.00307 |
| Lphn1 | – (green) | ● | ● | × | 0.00307 |
| Map2k6 | ● | ● (red) | ● | ● (red) | 0.00307 |
| Mast4 | ● | × | ● | ● | 0.00307 |
| Neto2 | ● | × | ● | × | 0.00307 |

↓ CR

## Genes down regulated by CR

| | msl1 | msl3 | msl15 | msl16 | $P_d$ |
|---|---|---|---|---|---|
| Nfib | ● | ● | * | × | 0.00307 |
| Nup153 | ● (red) | ● | ● | × | 0.00307 |
| Pik3ca | ● | ● | * | × | 0.00307 |
| Ppm1b | * | ● | ● | × | 0.00307 |
| Psme4 | ● | ● | * | × | 0.00307 |
| Rnf11 | ● | ● | ● | × | 0.00307 |
| Rragd | * (red) | ● (red) | ● | × | 0.00307 |
| Runx1 | ● | ● | ● | ● | 0.00307 |
| Setd8 | ● | ● | ● | − | 0.00307 |
| Six4 | ● | ● | ● | − | 0.00307 |
| Slc16a10 | ● | × | * | × | 0.00307 |
| Tbc1d8b | ● | − | ● | × | 0.00307 |
| Thap2 | ● | − | ● | − | 0.00307 |
| Tiam1 | ● | ● | ● | − | 0.00307 |
| Tnfsf10 | ● | − | ● | × | 0.00307 |
| Trip11 | ● | ● | ● | × | 0.00307 |
| Tspan8 | ● (red) | ● | * | ● | 0.00307 |
| Usp24 | ● | − (red) | ● | × | 0.00307 |
| Vps37c | − | ● | ● | ● | 0.00307 |
| Yy1 | * | ● | ● | × | 0.00307 |
| Zc3h12c | ● | − (green) | ● | × | 0.00307 |
| Zfp260 | ● | ● | ● | × | 0.00307 |
| 2310014D11Rik | ● | × | ● | × | 0.00307 |
| 2310014F06Rik | ● | × | ● | × | 0.00307 |
| 4921524J17Rik | ● | ● | ● | × | 0.00307 |
| Antxr2 | ● | ● | ● | × | 0.00307 |
| B230337E12Rik | ● | × | ● | × | 0.00307 |
| BC022623 | − (green) | ● | ● | × | 0.00307 |
| Cyp4a29 | ● | × | ● | × | 0.00307 |
| Hs3st5 | ● | × | ● | × | 0.00307 |

↓ CR

## Genes down regulated by CR

| | msl1 | msl3 | msl15 | msl16 | $P_d$ |
|---|---|---|---|---|---|
| Lrrc38 | ● | × | ● | × | 0.00307 |
| Mif4gd | ● | ● | ● | × | 0.00307 |
| Mm.38405 | ● | × | ● | × | 0.00307 |
| Mm.427239 | ● | × | ● | × | 0.00307 |
| Mm.476300 | ● | × | ● | × | 0.00307 |
| Mm.76761 | ● | × | ● | × | 0.00307 |
| USG00000014994 | ● | ● | ● | × | 0.00307 |
| Slc9a2 | ● | × | ● | × | 0.00307 |
| Uso1 | – | ● | ● | ● | 0.00307 |
| Zfp275 | ● | – | ● | ● | 0.00307 |
| Zhx2 | ● | × | ● | – (green) | 0.00307 |
| Map3k4 | ● | ● | ● | – | 0.00309 |
| Apbb2 | – (red) | ● | ● | × | 0.0031 |
| Fam174a | ● | ● | ● | × | 0.0031 |
| Rnf103 | ● (red) | ● | ● (red) | ● | 0.0031 |
| Trim35 | * (red) | ● (red) | ● | × | 0.0031 |
| Mm.285452 | ● | × | ● | × | 0.0031 |
| Lcor | * (green) | – (red) | ● | – | 0.0031 |
| St7l | ● (red) | ● | ● | – (green) | 0.0031 |
| Ppp2r5c | ● (red) | ● | ● | × | 0.00319 |
| Prrg1 | ● | × | ● | × | 0.00326 |
| Atf2 | * (green) | ● | ● | × | 0.00334 |
| Cdc2l6 | ● | × | ● | ● | 0.0034 |
| Otub2 | ● | ● | – | × | 0.0034 |
| Plcd4 | ● | × | ● | ● | 0.0034 |
| Traf3 | ● | ● | ● | × | 0.0034 |
| Zeb1 | – | ● | ● | ● | 0.0034 |
| Dennd2c | ● | × | ● | × | 0.0034 |
| Nsmaf | ● | ● | ● | – (green) | 0.0034 |
| Dopey2 | – | ● | ● | × | 0.00344 |

↓ CR

## Genes down regulated by CR

| | msl1 | msl3 | msl15 | msl16 | $P_d$ |
|---|---|---|---|---|---|
| Slc44a1 | – | ● (green) | ● (green) | × | 0.00344 |
| Agxt2l2 | * (green) | ● (green) | * (red) | – | 0.00346 |
| 2900097C17Rik | * (red) | ● (green) | ● (green) | × | 0.0035 |
| Sqle | ● (green) | – | ● (green) | × | 0.0035 |
| BC023105 | ● (green) | ● (green) | – | × | 0.00352 |
| Sos2 | ● (green) | – (green) | ● (green) | × | 0.00353 |
| Rap1gds1 | – | ● (green) | ● (green) | × | 0.0036 |
| Nkain1 | – (red) | ● (green) | * (red) | – | 0.00365 |
| Rab11fip3 | – (green) | × | ● (green) | ● (green) | 0.00367 |
| Phf17 | ● (green) | ● (green) | ● (green) | × | 0.00376 |
| Cish | ● (green) | ● (green) | ● (green) | – (green) | 0.00385 |
| Nek9 | – (green) | ● (green) | ● (green) | × | 0.00385 |
| BC010304 | * (red) | ● (green) | ● (green) | – | 0.00387 |
| E130308A19Rik | ● (green) | × | ● (green) | – | 0.00387 |
| Tshz1 | ● (green) | ● (green) | ● (green) | – (green) | 0.00387 |
| Lrrc58 | ● (green) | ● (green) | ● (green) | × | 0.00392 |
| Kif3c | ● (green) | – (green) | ● (green) | × | 0.00392 |
| Ncoa4 | ● (red) | ● (green) | ● (green) | × | 0.00403 |
| Heatr5a | ● (green) | × | ● (green) | × | 0.00412 |
| Ky | ● (green) | – | ● (green) | × | 0.00412 |
| Smad3 | ● (green) | ● (red) | ● (green) | × | 0.00412 |
| Mrgprh | ● (green) | ● (green) | ● (green) | – | 0.00412 |
| Pum2 | ● (red) | ● (green) | ● (green) | – | 0.00414 |
| Slc11a2 | ● (green) | * (red) | ● (green) | – | 0.00422 |
| Angptl1 | ● (green) | – | ● (green) | × | 0.00425 |
| Kctd7 | ● (green) | × | ● (green) | – | 0.00425 |
| Maf | ● (green) | – | * (green) | × | 0.00425 |
| Nfatc3 | ● (red) | ● (green) | ● (green) | × | 0.00425 |
| Mm.414514 | ● (green) | × | ● (green) | × | 0.00427 |
| Bace1 | ● (green) | ● (green) | ● (green) | – | 0.00432 |

↓ CR

## Genes down regulated by CR

| | msl1 | msl3 | msl15 | msl16 | $P_d$ |
|---|---|---|---|---|---|
| **Mark2** | ● (green) | ● (green) | ● (green) | × | **0.00438** |
| **Edc4** | – | ● (green) | ● (green) | ● (green) | **0.00438** |
| **Impad1** | – | ● (green) | * (red) | × | **0.00438** |
| **Ptp4a2** | ● (green) | – (green) | ● (green) | × | **0.00439** |
| **Ccndbp1** | ● (red) | ● (green) | ● (green) | ● (green) | **0.00443** |
| **Mm.384967** | ● (green) | × | ● (green) | × | **0.00443** |
| **Nckap1** | – | ● (green) | ● (green) | × | **0.00443** |
| **Daxx** | ● (red) | ● (green) | ● (green) | ● (green) | **0.00446** |
| **BC023829** | ● (green) | ● (green) | ● (green) | × | **0.0045** |
| **B3galnt2** | ● (green) | ● (green) | * (red) | × | **0.00459** |
| **Rnf115** | ● (green) | – | ● (green) | – | **0.00462** |
| **Mapk14** | * (red) | ● (green) | * (green) | – | **0.00463** |
| **Pten** | ● (green) | ● (green) | ● (green) | × | **0.00465** |
| **Tex2** | ● (green) | ● (green) | ● (green) | × | **0.00465** |
| **Myo18a** | – | – | ● (green) | ● (green) | **0.00465** |
| **BC004044** | ● (green) | – (green) | ● (green) | – | **0.00466** |
| **Cep350** | ● (red) | ● (green) | ● (green) | × | **0.00466** |
| **Grsf1** | ● (green) | ● (green) | ● (green) | – | **0.00466** |
| **Metap2** | ● (green) | ● (green) | ● (green) | × | **0.00466** |
| **Serpinh1** | ● (green) | – (red) | ● (green) | – | **0.00466** |

↑ CR

# Overrepresented Biological Processes

| GO Term | P−Value |
|---|---|
| RNA splicing | 9.61e−05 |
| mRNA processing | 0.000547 |
| insulin receptor signaling pathway | 0.00071 |
| glycerol metabolic process | 0.00203 |
| nuclear export | 0.00358 |
| proton transport | 0.00372 |
| monocarboxylic acid metabolic process | 0.00487 |
| protein targeting | 0.0086 |
| negative regulation of transport | 0.00872 |
| glucose transport | 0.0111 |
| regulation of Notch signaling pathway | 0.012 |
| negative regulation of T cell receptor signaling pathway | 0.012 |
| monosaccharide transport | 0.0125 |
| rhythmic process | 0.0181 |
| glucose homeostasis | 0.019 |
| ncRNA processing | 0.0195 |
| gluconeogenesis | 0.02 |
| response to organic cyclic substance | 0.02 |
| glycine metabolic process | 0.0216 |
| cotranslational protein targeting to membrane | 0.0216 |
| glutamine family amino acid catabolic process | 0.0216 |
| cortical actin cytoskeleton organization and biogenesis | 0.0216 |
| regulation of protein ubiquitination | 0.0216 |
| response to exogenous dsRNA | 0.0216 |
| amine catabolic process | 0.0227 |
| electron transport chain | 0.0228 |
| regulation of intracellular transport | 0.0228 |
| ribonucleoside triphosphate metabolic process | 0.0229 |
| regulation of localization | 0.0238 |
| alcohol catabolic process | 0.0243 |

↑ CR

# Overrepresented Biological Processes

| GO Term | P−Value |
|---|---|
| nucleotide−excision repair | 0.0259 |
| mRNA export from nucleus | 0.0273 |
| purine nucleoside triphosphate metabolic process | 0.0296 |
| generation of precursor metabolites and energy | 0.0331 |
| ovulation from ovarian follicle | 0.0334 |
| cellular process | 0.0334 |
| translation | 0.0352 |
| acyl−CoA metabolic process | 0.0401 |
| axon cargo transport | 0.0401 |
| monosaccharide biosynthetic process | 0.0401 |
| transmembrane transport | 0.0401 |
| establishment of localization in cell | 0.0426 |
| positive regulation of MAPKKK cascade | 0.0442 |
| ATP metabolic process | 0.0442 |
| retinoid metabolic process | 0.0471 |
| lipopolysaccharide−mediated signaling pathway | 0.0471 |
| regulation of antigen receptor−mediated signaling pathway | 0.0471 |
| carboxylic acid metabolic process | 0.0472 |
| primary metabolic process | 0.0475 |

↑ CR

# Overrepresented Cell Components

| GO Term | P−Value |
|---|---|
| intracellular | 1.32e−08 |
| mitochondrion | 4.71e−08 |
| cytoplasm | 5.18e−08 |
| intracellular organelle part | 5.02e−07 |
| mitochondrial inner membrane | 1.96e−06 |
| organelle membrane | 4.63e−06 |
| membrane−bounded organelle | 4.87e−06 |
| macromolecular complex | 6.16e−06 |
| mitochondrial envelope | 1.04e−05 |
| envelope | 8.55e−05 |
| intracellular organelle | 0.000166 |
| cytosol | 0.000191 |
| spliceosome | 0.000482 |
| ribosome | 0.000661 |
| prefoldin complex | 0.00111 |
| nucleus | 0.00116 |
| mitochondrial matrix | 0.00171 |
| sarcoplasmic reticulum | 0.00561 |
| dynactin complex | 0.00744 |
| proton−transporting two−sector ATPase complex, catalytic domain | 0.011 |
| cell−substrate junction | 0.0161 |
| peroxisome | 0.0165 |
| growth cone | 0.0176 |
| mediator complex | 0.0201 |
| signalosome | 0.0249 |
| organelle lumen | 0.0306 |
| mitochondrial membrane part | 0.0336 |
| focal adhesion | 0.0391 |
| cell | 0.0414 |
| exosome (RNase complex) | 0.0431 |

↑ CR

# Overrepresented Cell Components

| GO Term | P−Value |
| --- | --- |
| filopodium | 0.0431 |
| cortical actin cytoskeleton | 0.0431 |

# Overrepresented Molecular Functions

| GO Term | P−Value |
|---|---|
| vitamin D receptor binding | 0.000814 |
| structural constituent of ribosome | 0.00116 |
| hydrogen ion transporting ATP synthase activity, rotational mechanism | 0.00197 |
| hydrogen ion transporting ATPase activity, rotational mechanism | 0.00197 |
| bisphosphoglycerate mutase activity | 0.00239 |
| bisphosphoglycerate 2−phosphatase activity | 0.00239 |
| diacylglycerol O−acyltransferase activity | 0.00239 |
| phosphoglycerate mutase activity | 0.00239 |
| 2−acylglycerol O−acyltransferase activity | 0.0047 |
| transferase activity, transferring acyl groups | 0.00533 |
| unfolded protein binding | 0.00554 |
| hormone receptor binding | 0.00665 |
| p53 binding | 0.00768 |
| RNA splicing factor activity, transesterification mechanism | 0.00768 |
| SNAP receptor activity | 0.00814 |
| monovalent inorganic cation transmembrane transporter activity | 0.00832 |
| P−P−bond−hydrolysis−driven transmembrane transporter activity | 0.00925 |
| exonuclease activity, active with either ribo− or deoxyribonucleic acids and producing 5'−phosphomonoest | 0.00981 |
| oxidoreductase activity, acting on paired donors, with oxidation of a pair of donors resulting in the reductio | 0.0113 |
| phosphoric ester hydrolase activity | 0.0131 |
| ATPase activity, coupled to transmembrane movement of ions | 0.0138 |
| DNA−directed RNA polymerase activity | 0.0139 |
| 3'−5'−exoribonuclease activity | 0.0155 |
| glycerophosphodiester phosphodiesterase activity | 0.0155 |
| catalytic activity | 0.0194 |
| hydrolase activity, acting on acid anhydrides, catalyzing transmembrane movement of substances | 0.0199 |
| metalloendopeptidase inhibitor activity | 0.0203 |
| ATPase activity, coupled to movement of substances | 0.021 |
| methyltransferase activity | 0.0233 |
| RNA binding | 0.0238 |

↑ CR

# Overrepresented Molecular Functions

| GO Term | P−Value |
| --- | --- |
| acyltransferase activity | 0.0241 |
| carbohydrate phosphatase activity | 0.0256 |
| transcription coactivator activity | 0.028 |
| palmitoyl−CoA hydrolase activity | 0.0315 |
| exoribonuclease activity | 0.0377 |
| insulin receptor binding | 0.0444 |

↓CR

# Overrepresented Biological Processes

| GO Term | P−Value |
|---|---|
| embryonic skeletal morphogenesis | 7.87e−05 |
| protein modification process | 8.6e−05 |
| skeletal development | 9.71e−05 |
| organ morphogenesis | 0.000117 |
| tube development | 0.000149 |
| pattern specification process | 0.000174 |
| mesoderm formation | 0.000193 |
| intracellular signaling cascade | 0.000222 |
| transforming growth factor beta receptor signaling pathway | 0.000228 |
| negative regulation of transcription from RNA polymerase II promoter | 0.000544 |
| blood vessel development | 0.000556 |
| modification−dependent macromolecule catabolic process | 0.000558 |
| chordate embryonic development | 0.000688 |
| proteolysis involved in cellular protein catabolic process | 0.000708 |
| phosphate metabolic process | 0.000717 |
| middle ear morphogenesis | 0.000788 |
| biopolymer metabolic process | 0.000971 |
| enzyme linked receptor protein signaling pathway | 0.00103 |
| G2 phase of mitotic cell cycle | 0.00143 |
| myoblast migration | 0.00143 |
| ureteric bud branching | 0.00178 |
| phosphate transport | 0.00184 |
| metanephros development | 0.00231 |
| regulation of binding | 0.00231 |
| protein catabolic process | 0.00266 |
| negative regulation of RNA metabolic process | 0.00301 |
| mesoderm development | 0.00322 |
| gastrulation | 0.00354 |
| embryonic development | 0.00364 |
| paraxial mesoderm morphogenesis | 0.00382 |

↓ CR

# Overrepresented Biological Processes

| GO Term | P−Value |
|---|---|
| anterior/posterior pattern formation | 0.00391 |
| positive regulation of macromolecule metabolic process | 0.00392 |
| positive regulation of biological process | 0.00404 |
| regulation of biological process | 0.00406 |
| acrosome formation | 0.00418 |
| voluntary musculoskeletal movement | 0.00418 |
| ubiquitin−dependent protein catabolic process | 0.00435 |
| positive regulation of transcription | 0.00437 |
| regulation of small GTPase mediated signal transduction | 0.00444 |
| protein amino acid phosphorylation | 0.00511 |
| urogenital system development | 0.00597 |
| regulation of protein polymerization | 0.00607 |
| regulation of protein metabolic process | 0.00626 |
| anatomical structure development | 0.0063 |
| cellular developmental process | 0.00647 |
| regulation of gene expression | 0.00649 |
| heart development | 0.00669 |
| positive regulation of cellular metabolic process | 0.00741 |
| striated muscle development | 0.00765 |
| Ras protein signal transduction | 0.00811 |
| spermine metabolic process | 0.00812 |
| primary metabolic process | 0.00814 |
| positive regulation of actin filament polymerization | 0.00816 |
| response to osmotic stress | 0.00919 |
| positive regulation of transcription from RNA polymerase II promoter | 0.00948 |
| cell morphogenesis | 0.0103 |
| positive regulation of biosynthetic process | 0.0106 |
| negative regulation of macromolecule metabolic process | 0.011 |
| negative regulation of cellular metabolic process | 0.0111 |
| regulation of Rho protein signal transduction | 0.0112 |

↓ CR

# Overrepresented Biological Processes

| GO Term | P−Value |
|---|---|
| macromolecule catabolic process | 0.0113 |
| protein amino acid O−linked glycosylation | 0.0116 |
| vacuolar transport | 0.0116 |
| steroid hormone receptor signaling pathway | 0.0116 |
| cartilage development | 0.0121 |
| actin filament−based process | 0.0131 |
| polyamine biosynthetic process | 0.0132 |
| histone ubiquitination | 0.0133 |
| regulation of catalytic activity | 0.0139 |
| negative regulation of biosynthetic process | 0.0143 |
| mating | 0.0144 |
| RNA biosynthetic process | 0.0153 |
| gastrulation with mouth forming second | 0.0157 |
| positive regulation of RNA metabolic process | 0.0157 |
| regulation of cell growth | 0.016 |
| negative regulation of cell cycle | 0.016 |
| system development | 0.0161 |
| regulation of transcription, DNA−dependent | 0.0164 |
| negative regulation of cell differentiation | 0.0164 |
| palate development | 0.0174 |
| lamellipodium biogenesis | 0.0175 |
| cell communication | 0.0182 |
| regulation of actin polymerization and/or depolymerization | 0.0186 |
| regulation of neuron differentiation | 0.0186 |
| negative regulation of transcription | 0.0188 |
| protein kinase B signaling cascade | 0.0192 |
| in utero embryonic development | 0.0193 |
| response to retinoic acid | 0.0194 |
| response to vitamin | 0.0194 |
| regulation of protein kinase B signaling cascade | 0.0194 |

↓ CR

# Overrepresented Biological Processes

| GO Term | P−Value |
| --- | --- |
| cellular protein metabolic process | 0.0204 |
| neural tube formation | 0.0204 |
| cell development | 0.0209 |
| positive regulation of mesenchymal cell proliferation | 0.0209 |
| regulation of transforming growth factor beta receptor signaling pathway | 0.0209 |
| regulation of macromolecule biosynthetic process | 0.0212 |
| tissue morphogenesis | 0.0212 |
| regulation of Ras GTPase activity | 0.0219 |
| angiogenesis | 0.0224 |
| regulation of actin cytoskeleton organization and biogenesis | 0.0224 |
| anion transport | 0.0239 |
| regulation of cell shape | 0.0246 |
| proteasomal ubiquitin−dependent protein catabolic process | 0.0247 |
| protein import into nucleus | 0.0254 |
| response to unfolded protein | 0.0255 |
| protein amino acid dephosphorylation | 0.0259 |
| negative regulation of developmental process | 0.0262 |
| rostrocaudal neural tube patterning | 0.0265 |
| reproductive behavior in a multicellular organism | 0.0265 |
| positive regulation of angiogenesis | 0.0265 |
| regulation of cellular component size | 0.0268 |
| regulation of anatomical structure morphogenesis | 0.0274 |
| regulation of nucleobase, nucleoside, nucleotide and nucleic acid metabolic process | 0.0286 |
| regulation of cellular metabolic process | 0.0288 |
| neural plate development | 0.0292 |
| regulation of translation | 0.0315 |
| negative regulation of apoptosis | 0.0329 |
| multicellular organismal process | 0.033 |
| endoderm development | 0.0331 |
| suckling behavior | 0.0344 |

↓ CR

# Overrepresented Biological Processes

| GO Term | P−Value |
|---|---|
| cellular copper ion homeostasis | 0.0344 |
| cerebral cortex radially oriented cell migration | 0.0344 |
| chromatin modification | 0.0354 |
| regulation of kinase activity | 0.0383 |
| ear development | 0.0396 |
| cellular protein complex assembly | 0.0414 |
| protein ubiquitination | 0.0415 |
| protein localization | 0.043 |
| regulation of cellular component organization and biogenesis | 0.0437 |
| establishment and/or maintenance of chromatin architecture | 0.0441 |
| regulation of T cell differentiation | 0.046 |
| positive regulation of cellular component organization and biogenesis | 0.0461 |
| branching morphogenesis of a tube | 0.047 |
| morphogenesis of an epithelium | 0.0477 |
| patterning of blood vessels | 0.0487 |
| positive regulation of cell migration | 0.0487 |

↓ CR

# Overrepresented Cell Components

| GO Term | P−Value |
|---|---|
| cytoplasm | 2.8e−05 |
| collagen | 4.3e−05 |
| intracellular | 4.45e−05 |
| transcription factor complex | 0.000242 |
| nuclear lumen | 0.000406 |
| nucleus | 0.000573 |
| cell | 0.00114 |
| heterochromatin | 0.00242 |
| membrane−enclosed lumen | 0.00283 |
| calcineurin complex | 0.00394 |
| protein phosphatase type 2A complex | 0.00652 |
| PcG protein complex | 0.00652 |
| lamellipodium | 0.00873 |
| nucleoplasm part | 0.0168 |
| insoluble fraction | 0.0252 |
| intracellular organelle | 0.0258 |
| membrane−bounded organelle | 0.0279 |
| extracellular matrix | 0.028 |
| basement membrane | 0.0332 |
| macromolecular complex | 0.0374 |
| ciliary rootlet | 0.0409 |

↓ CR

# Overrepresented Molecular Functions

| GO Term | P−Value |
|---|---|
| protein binding | 3.54e−06 |
| ubiquitin−protein ligase activity | 9.3e−05 |
| manganese ion binding | 0.000124 |
| transcription factor binding | 0.000201 |
| extracellular matrix structural constituent | 0.000221 |
| magnesium ion binding | 0.000418 |
| transcription factor activity | 0.00142 |
| acid−amino acid ligase activity | 0.00164 |
| protein serine/threonine kinase activity | 0.0018 |
| aminopeptidase activity | 0.00315 |
| transmembrane receptor protein serine/threonine kinase activity | 0.00719 |
| protein transporter activity | 0.00818 |
| activin receptor activity | 0.00824 |
| mRNA binding | 0.00988 |
| phosphotransferase activity, alcohol group as acceptor | 0.0125 |
| sequence−specific DNA binding | 0.0125 |
| receptor signaling protein activity | 0.0144 |
| acetylgalactosaminyltransferase activity | 0.0159 |
| MAP kinase kinase kinase activity | 0.0177 |
| solute:hydrogen antiporter activity | 0.0177 |
| phosphoric ester hydrolase activity | 0.0186 |
| phosphoprotein binding | 0.0195 |
| sodium:hydrogen antiporter activity | 0.0196 |
| semaphorin receptor activity | 0.0196 |
| chaperone binding | 0.0196 |
| kinase activity | 0.0213 |
| ligase activity | 0.0218 |
| ubiquitin thiolesterase activity | 0.0224 |
| small GTPase regulator activity | 0.0237 |
| transcription coactivator activity | 0.026 |

↓ CR

# Overrepresented Molecular Functions

| GO Term | P−Value |
|---|---|
| Rho guanyl−nucleotide exchange factor activity | 0.026 |
| small conjugating protein−specific protease activity | 0.026 |
| histone acetyltransferase activity | 0.0292 |
| L−ascorbic acid binding | 0.0337 |
| protein phosphatase type 2A regulator activity | 0.0348 |
| protein phosphorylated amino acid binding | 0.0348 |
| ion binding | 0.0372 |
| double−stranded DNA binding | 0.0386 |
| solute:solute antiporter activity | 0.0386 |
| kinase inhibitor activity | 0.0386 |
| protein tyrosine/serine/threonine phosphatase activity | 0.039 |
| phosphatidate phosphatase activity | 0.0436 |
| metalloexopeptidase activity | 0.0436 |
| Rab GTPase activator activity | 0.0467 |

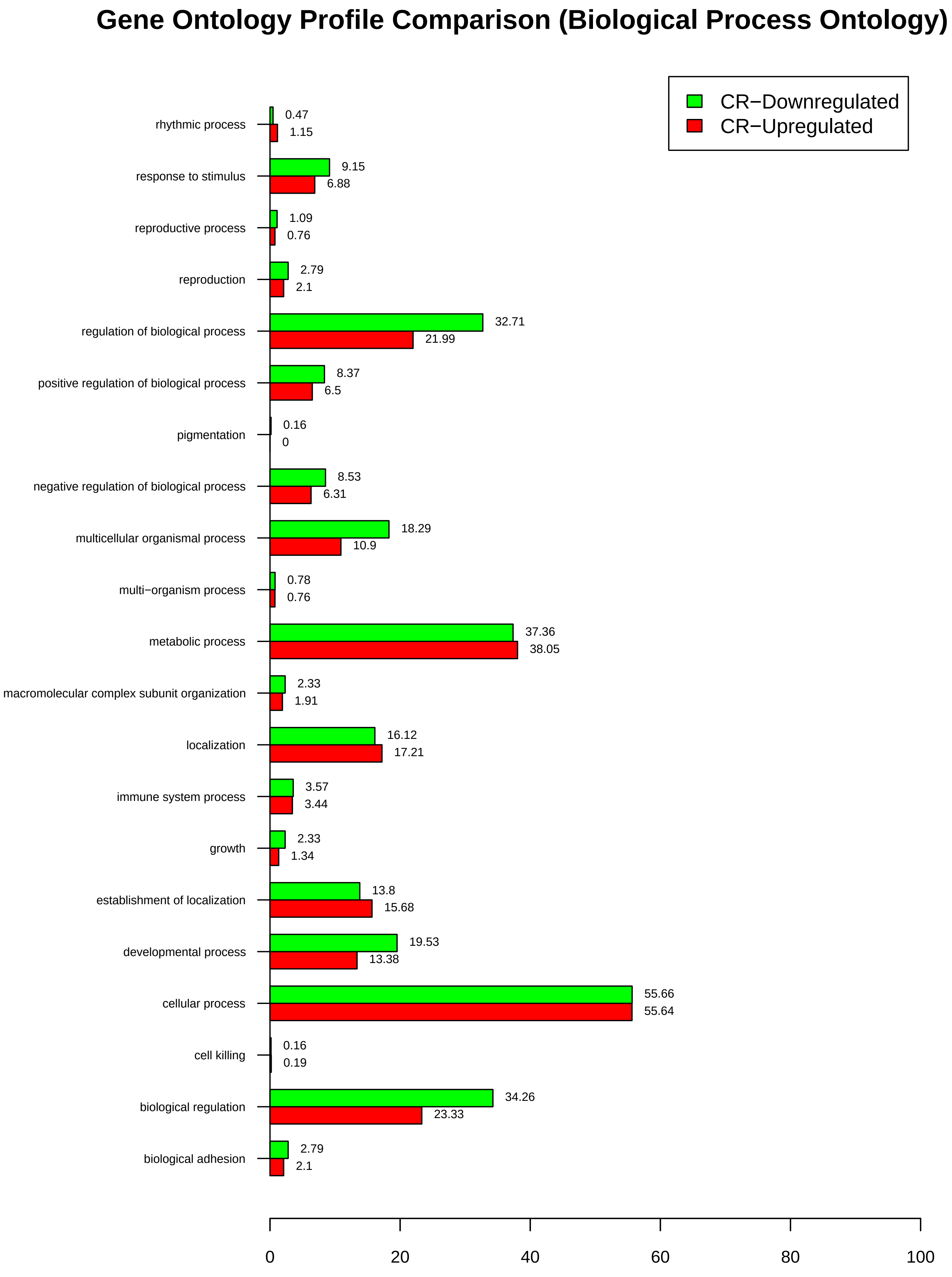

Gene Ontology Profile Comparison (Biological Process Ontology)
CR−Downregulated
CR−Upregulated
rhythmic process
0.47
1.15
response to stimulus
9.15
6.88
reproductive process
1.09
0.76
reproduction
2.79
2.1
regulation of biological process
32.71
21.99
positive regulation of biological process
8.37
6.5
pigmentation
0.16
0
negative regulation of biological process
8.53
6.31
multicellular organismal process
18.29
10.9
multi−organism process
0.78
0.76
metabolic process
37.36
38.05
macromolecular complex subunit organization
2.33
1.91
localization
16.12
17.21
immune system process
3.57
3.44
growth
2.33
1.34
establishment of localization
13.8
15.68
developmental process
19.53
13.38
cellular process
55.66
55.64
cell killing
0.16
0.19
biological regulation
34.26
23.33
biological adhesion
2.79
2.1
0
20
40
60
80
100

## Gene Ontology Profile Comparison (Cell Component Ontology)

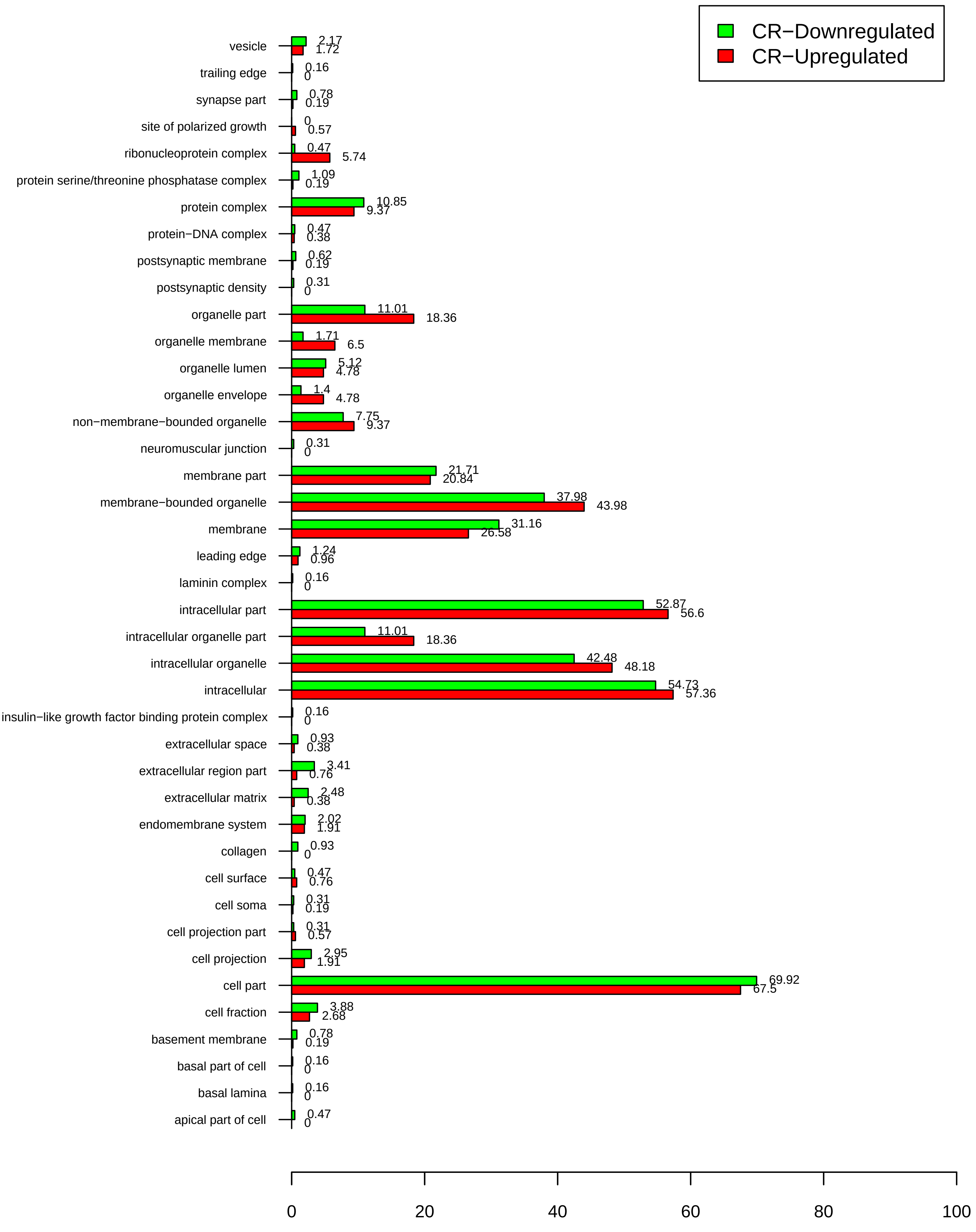

# Gene Ontology Profile Comparison (Molecular Function Ontology)

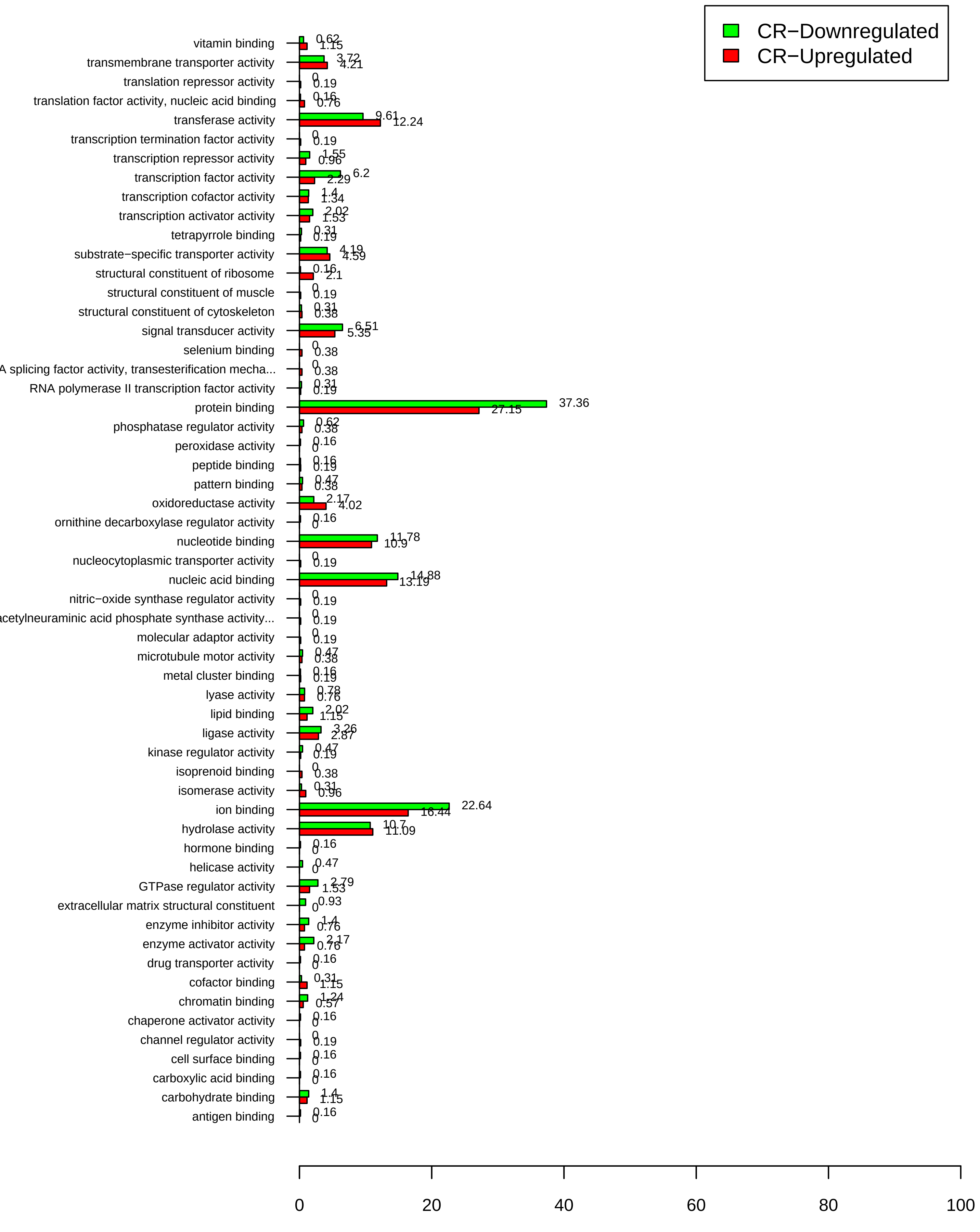

↑ CR

# Overrepresented KEGG Pathways

| GO Term | P−Value |
|---|---|
| Insulin signaling pathway | 2.88e−06 |
| Biosynthesis of unsaturated fatty acids | 0.000341 |
| SNARE interactions in vesicular transport | 0.00165 |
| ErbB signaling pathway | 0.00544 |
| Endometrial cancer | 0.0141 |
| Valine, leucine and isoleucine degradation | 0.0196 |
| Geraniol degradation | 0.0201 |
| Cyanoamino acid metabolism | 0.0201 |
| Fatty acid metabolism | 0.0216 |
| Acute myeloid leukemia | 0.0232 |
| Chronic myeloid leukemia | 0.0246 |
| Oxidative phosphorylation | 0.0278 |
| Type II diabetes mellitus | 0.0283 |
| Long−term potentiation | 0.0406 |
| mTOR signaling pathway | 0.0453 |

↓ CR

# Overrepresented KEGG Pathways

| GO Term | P−Value |
|---|---|
| TGF−beta signaling pathway | 0.000192 |
| ECM−receptor interaction | 0.000506 |
| MAPK signaling pathway | 0.0018 |
| Focal adhesion | 0.00463 |
| GnRH signaling pathway | 0.00528 |
| Phosphatidylinositol signaling system | 0.00873 |
| Circadian rhythm | 0.015 |
| Wnt signaling pathway | 0.016 |
| Huntington's disease | 0.0307 |
| Fc epsilon RI signaling pathway | 0.0377 |
| Regulation of actin cytoskeleton | 0.039 |
| Ubiquitin mediated proteolysis | 0.0432 |
| Glioma | 0.0459 |

↑ CR

# Overrepresented KEGG Pathways

## (Based on InterPro Domain Signatures)

| GO Term | P−Value |
|---|---|
| Citrate cycle (TCA cycle) | 1e−04 |
| Biosynthesis of unsaturated fatty acids | 1e−04 |
| Insulin signaling pathway | 1e−04 |
| Reductive carboxylate cycle (CO2 fixation) | 0.0023 |
| 1− and 2−Methylnaphthalene degradation | 0.0027 |
| Fatty acid biosynthesis | 0.0034 |
| Propanoate metabolism | 0.0078 |
| Valine, leucine and isoleucine degradation | 0.009 |
| Aminophosphonate metabolism | 0.0098 |
| Glyoxylate and dicarboxylate metabolism | 0.0098 |
| Geraniol degradation | 0.01 |
| Jak−STAT signaling pathway | 0.0113 |
| Carbon fixation | 0.0118 |
| Fatty acid elongation in mitochondria | 0.0119 |
| Selenoamino acid metabolism | 0.0126 |
| alpha−Linolenic acid metabolism | 0.0164 |
| VEGF signaling pathway | 0.021 |
| Thiamine metabolism | 0.0219 |
| Pentose phosphate pathway | 0.0227 |
| Histidine metabolism | 0.0234 |
| Pyruvate metabolism | 0.0237 |
| Fatty acid metabolism | 0.0244 |
| SNARE interactions in vesicular transport | 0.029 |
| Fructose and mannose metabolism | 0.0313 |
| ErbB signaling pathway | 0.0332 |
| Butanoate metabolism | 0.0357 |
| Benzoate degradation via CoA ligation | 0.037 |
| Nitrogen metabolism | 0.0402 |
| Alanine and aspartate metabolism | 0.0431 |
| Glycerophospholipid metabolism | 0.0487 |

↓ CR

# Overrepresented KEGG Pathways

## (Based on InterPro Domain Signatures)

| GO Term | P−Value |
|---|---|
| B cell receptor signaling pathway | 1e−04 |
| Chronic myeloid leukemia | 1e−04 |
| MAPK signaling pathway | 2e−04 |
| Non−small cell lung cancer | 3e−04 |
| T cell receptor signaling pathway | 6e−04 |
| Phosphatidylinositol signaling system | 9e−04 |
| Renal cell carcinoma | 9e−04 |
| Endometrial cancer | 0.0011 |
| ErbB signaling pathway | 0.0022 |
| Pancreatic cancer | 0.0024 |
| Small cell lung cancer | 0.0027 |
| Prostate cancer | 0.0041 |
| p53 signaling pathway | 0.0045 |
| mTOR signaling pathway | 0.0048 |
| Alzheimer's disease | 0.0051 |
| Glioma | 0.0058 |
| Fc epsilon RI signaling pathway | 0.0066 |
| TGF−beta signaling pathway | 0.0071 |
| Focal adhesion | 0.0082 |
| Acute myeloid leukemia | 0.0084 |
| VEGF signaling pathway | 0.0089 |
| Inositol phosphate metabolism | 0.0122 |
| Huntington's disease | 0.0126 |
| GnRH signaling pathway | 0.0147 |
| Natural killer cell mediated cytotoxicity | 0.0166 |
| Regulation of autophagy | 0.0178 |
| Colorectal cancer | 0.0184 |
| Adherens junction | 0.0225 |
| Insulin signaling pathway | 0.0268 |
| Dentatorubropallidoluysian atrophy (DRPLA) | 0.0297 |

↓ CR

# Overrepresented KEGG Pathways

## (Based on InterPro Domain Signatures)

| GO Term | P−Value |
|---|---|
| Ubiquitin mediated proteolysis | 0.0318 |
| Prion disease | 0.0334 |
| Regulation of actin cytoskeleton | 0.0361 |
| Neurodegenerative Diseases | 0.0395 |
| Wnt signaling pathway | 0.0426 |
| Tight junction | 0.0448 |
| Aminoacyl−tRNA biosynthesis | 0.0466 |
| Terpenoid biosynthesis | 0.048 |
| Leukocyte transendothelial migration | 0.048 |
| Porphyrin and chlorophyll metabolism | 0.0484 |

↑ CR

# Abundance of miRNA Targets

| miRNA | Freq(Obs) | Freq(Exp) | Obs/Exp | P−value | P−Value(Adj) |
|---|---|---|---|---|---|
| miR−1188 | 0.12 | 0.0873 | 1.37 | 0.0116 | 1 |
| miR−329 | 0.115 | 0.092 | 1.25 | 0.0548 | 1 |
| miR−504 | 0.0938 | 0.0765 | 1.22 | 0.0879 | 1 |
| miR−365 | 0.0781 | 0.063 | 1.24 | 0.0943 | 1 |
| miR−339−3p | 0.0417 | 0.0313 | 1.33 | 0.0976 | 1 |
| miR−675−5p | 0.0339 | 0.0252 | 1.34 | 0.109 | 1 |
| miR−147 | 0.0443 | 0.0347 | 1.28 | 0.122 | 1 |
| miR−540−3p | 0.0938 | 0.0811 | 1.16 | 0.156 | 1 |
| miR−423−3p | 0.0391 | 0.0325 | 1.2 | 0.188 | 1 |
| miR−701 | 0.0208 | 0.0167 | 1.25 | 0.196 | 1 |

↓ CR

# Abundance of miRNA Targets

| miRNA | Freq(Obs) | Freq(Exp) | Obs/Exp | P−value | P−Value(Adj) |
|---|---|---|---|---|---|
| miR−291b−3p | 0.266 | 0.157 | 1.69 | 9.62e−12 | 4.73e−09 |
| miR−590−3p | 0.374 | 0.261 | 1.43 | 9.03e−10 | 2.22e−07 |
| miR−384−5p | 0.271 | 0.178 | 1.52 | 1.09e−08 | 1.79e−06 |
| miR−338−5p | 0.2 | 0.126 | 1.59 | 1.82e−07 | 2.24e−05 |
| miR−669b | 0.277 | 0.192 | 1.44 | 3.06e−07 | 3e−05 |
| miR−30e | 0.23 | 0.156 | 1.48 | 1.01e−06 | 8.15e−05 |
| miR−30a | 0.218 | 0.146 | 1.49 | 1.47e−06 | 8.15e−05 |
| miR−32 | 0.167 | 0.104 | 1.6 | 1.83e−06 | 8.15e−05 |
| miR−1192 | 0.246 | 0.171 | 1.44 | 1.88e−06 | 8.15e−05 |
| miR−466a−3p | 0.261 | 0.184 | 1.41 | 2.38e−06 | 8.15e−05 |
| miR−466b−3−3p | 0.261 | 0.184 | 1.41 | 2.38e−06 | 8.15e−05 |
| miR−466b−3p | 0.261 | 0.184 | 1.41 | 2.38e−06 | 8.15e−05 |
| miR−466c−3p | 0.261 | 0.184 | 1.41 | 2.38e−06 | 8.15e−05 |
| miR−466e−3p | 0.261 | 0.184 | 1.41 | 2.38e−06 | 8.15e−05 |
| miR−30b | 0.22 | 0.149 | 1.47 | 2.49e−06 | 8.15e−05 |
| miR−301a | 0.232 | 0.162 | 1.44 | 4.55e−06 | 0.000138 |
| miR−367 | 0.156 | 0.0981 | 1.59 | 4.76e−06 | 0.000138 |
| miR−374 | 0.19 | 0.127 | 1.5 | 6.7e−06 | 0.000183 |
| miR−466f−3p | 0.262 | 0.19 | 1.38 | 8.77e−06 | 0.000216 |
| miR−105 | 0.181 | 0.12 | 1.51 | 8.8e−06 | 0.000216 |
| miR−92a | 0.129 | 0.0788 | 1.64 | 1.08e−05 | 0.000244 |
| miR−30c | 0.22 | 0.154 | 1.43 | 1.09e−05 | 0.000244 |
| miR−25 | 0.149 | 0.0952 | 1.56 | 1.49e−05 | 0.000318 |
| miR−301b | 0.22 | 0.156 | 1.41 | 2.48e−05 | 0.000507 |
| miR−126−5p | 0.195 | 0.135 | 1.44 | 2.59e−05 | 0.000508 |
| miR−568 | 0.225 | 0.161 | 1.39 | 2.74e−05 | 0.000517 |
| miR−706 | 0.19 | 0.131 | 1.44 | 3.19e−05 | 0.000579 |
| miR−873 | 0.179 | 0.123 | 1.46 | 3.81e−05 | 0.000668 |
| miR−20a | 0.238 | 0.174 | 1.37 | 4.19e−05 | 0.000705 |
| miR−224 | 0.177 | 0.122 | 1.46 | 4.31e−05 | 0.000705 |

↓ CR

# Abundance of miRNA Targets

| miRNA | Freq(Obs) | Freq(Exp) | Obs/Exp | P−value | P−Value(Adj) |
|---|---|---|---|---|---|
| miR−340−5p | 0.264 | 0.198 | 1.33 | 4.92e−05 | 0.000779 |
| miR−675−3p | 0.0851 | 0.0481 | 1.77 | 5.28e−05 | 0.00081 |
| miR−148b | 0.184 | 0.129 | 1.43 | 5.46e−05 | 0.000812 |
| miR−466l | 0.363 | 0.29 | 1.25 | 5.76e−05 | 0.000832 |
| miR−294 | 0.195 | 0.138 | 1.41 | 6.11e−05 | 0.000851 |
| miR−335−5p | 0.199 | 0.141 | 1.41 | 6.37e−05 | 0.000851 |
| miR−467g | 0.238 | 0.175 | 1.35 | 6.41e−05 | 0.000851 |
| miR−466d−3p | 0.223 | 0.163 | 1.37 | 6.64e−05 | 0.000858 |
| miR−132 | 0.17 | 0.118 | 1.45 | 7.56e−05 | 0.000951 |
| miR−20b | 0.22 | 0.16 | 1.37 | 7.75e−05 | 0.000951 |
| miR−152 | 0.174 | 0.121 | 1.44 | 8.28e−05 | 0.000992 |
| miR−92b | 0.119 | 0.0753 | 1.58 | 8.48e−05 | 0.000992 |
| miR−297b−3p | 0.238 | 0.177 | 1.34 | 9.11e−05 | 0.00102 |
| miR−883b−3p | 0.181 | 0.127 | 1.42 | 9.12e−05 | 0.00102 |
| miR−106a | 0.218 | 0.16 | 1.37 | 9.5e−05 | 0.00104 |
| miR−429 | 0.207 | 0.151 | 1.38 | 0.000108 | 0.00112 |
| miR−669h−3p | 0.206 | 0.149 | 1.38 | 0.000109 | 0.00112 |
| miR−465a−5p | 0.262 | 0.2 | 1.31 | 0.000112 | 0.00112 |
| miR−29b | 0.177 | 0.125 | 1.42 | 0.000112 | 0.00112 |
| miR−30d | 0.188 | 0.134 | 1.4 | 0.000121 | 0.00118 |
| miR−335−3p | 0.282 | 0.218 | 1.29 | 0.00013 | 0.00125 |
| miR−141 | 0.222 | 0.164 | 1.35 | 0.000132 | 0.00125 |
| miR−382 | 0.177 | 0.126 | 1.41 | 0.000148 | 0.00137 |
| miR−448 | 0.179 | 0.127 | 1.41 | 0.000152 | 0.00139 |
| miR−218 | 0.158 | 0.11 | 1.44 | 0.000167 | 0.00149 |
| miR−148a | 0.177 | 0.126 | 1.4 | 0.000172 | 0.0015 |
| miR−467d | 0.0674 | 0.0373 | 1.81 | 0.000181 | 0.00156 |
| miR−18a | 0.174 | 0.123 | 1.41 | 0.000187 | 0.00158 |
| miR−421 | 0.195 | 0.143 | 1.37 | 0.000241 | 0.00201 |
| miR−694 | 0.248 | 0.19 | 1.3 | 0.000245 | 0.00201 |

↓ CR

# Abundance of miRNA Targets

| miRNA | Freq(Obs) | Freq(Exp) | Obs/Exp | P−value | P−Value(Adj) |
|---|---|---|---|---|---|
| miR−200b | 0.2 | 0.148 | 1.35 | 0.000291 | 0.00234 |
| miR−669k | 0.165 | 0.118 | 1.4 | 0.000312 | 0.00247 |
| miR−871 | 0.227 | 0.172 | 1.32 | 0.00032 | 0.0025 |
| miR−544 | 0.207 | 0.155 | 1.34 | 0.000326 | 0.0025 |
| miR−495 | 0.216 | 0.163 | 1.33 | 0.000348 | 0.00263 |
| miR−669e | 0.158 | 0.112 | 1.41 | 0.000373 | 0.00275 |
| miR−29c | 0.156 | 0.111 | 1.41 | 0.000375 | 0.00275 |
| miR−139−5p | 0.168 | 0.121 | 1.39 | 0.000391 | 0.00282 |
| miR−17 | 0.222 | 0.168 | 1.32 | 4e−04 | 0.00284 |
| miR−1198 | 0.167 | 0.12 | 1.39 | 0.000426 | 0.00299 |
| miR−106b | 0.19 | 0.14 | 1.35 | 0.000441 | 0.00305 |
| miR−29a | 0.152 | 0.108 | 1.41 | 0.000463 | 0.00316 |
| miR−465c−5p | 0.204 | 0.153 | 1.33 | 0.00048 | 0.0032 |
| miR−363 | 0.131 | 0.0905 | 1.45 | 0.000482 | 0.0032 |
| miR−669f | 0.277 | 0.219 | 1.26 | 0.000509 | 0.00333 |
| miR−155 | 0.168 | 0.123 | 1.37 | 0.000524 | 0.00338 |
| miR−466c−5p | 0.202 | 0.152 | 1.33 | 0.000537 | 0.00342 |
| miR−200c | 0.197 | 0.148 | 1.33 | 0.000543 | 0.00342 |
| miR−212 | 0.154 | 0.111 | 1.39 | 0.00057 | 0.00354 |
| miR−883a−3p | 0.174 | 0.128 | 1.36 | 6e−04 | 0.00368 |
| miR−19b | 0.206 | 0.156 | 1.32 | 0.000615 | 0.0037 |
| miR−124 | 0.16 | 0.115 | 1.38 | 0.000617 | 0.0037 |
| miR−342−3p | 0.152 | 0.111 | 1.38 | 0.000874 | 0.00517 |
| miR−93 | 0.209 | 0.161 | 1.3 | 0.000903 | 0.00528 |
| miR−130a | 0.186 | 0.14 | 1.33 | 0.000928 | 0.00536 |
| miR−103 | 0.191 | 0.145 | 1.32 | 0.000962 | 0.00543 |
| miR−107 | 0.191 | 0.145 | 1.32 | 0.000962 | 0.00543 |
| miR−292−3p | 0.137 | 0.0974 | 1.4 | 0.00102 | 0.00568 |
| miR−345−3p | 0.142 | 0.102 | 1.39 | 0.00113 | 0.00621 |
| miR−1188 | 0.124 | 0.0873 | 1.42 | 0.00114 | 0.00621 |

↓ CR

# Abundance of miRNA Targets

| miRNA | Freq(Obs) | Freq(Exp) | Obs/Exp | P−value | P−Value(Adj) |
|---|---|---|---|---|---|
| miR−542−3p | 0.181 | 0.137 | 1.32 | 0.0012 | 0.00649 |
| miR−467a | 0.124 | 0.0879 | 1.41 | 0.00138 | 0.00733 |
| miR−101b | 0.158 | 0.117 | 1.35 | 0.00139 | 0.00733 |
| miR−18b | 0.158 | 0.117 | 1.35 | 0.00145 | 0.00757 |
| miR−19a | 0.216 | 0.169 | 1.28 | 0.00147 | 0.0076 |
| miR−320 | 0.229 | 0.181 | 1.27 | 0.0015 | 0.00769 |
| miR−743a | 0.25 | 0.2 | 1.25 | 0.00152 | 0.00772 |
| miR−669i | 0.14 | 0.102 | 1.37 | 0.00155 | 0.00778 |
| miR−380−3p | 0.129 | 0.0931 | 1.39 | 0.00169 | 0.00838 |
| miR−16 | 0.232 | 0.185 | 1.26 | 0.00174 | 0.00843 |
| miR−465b−5p | 0.223 | 0.177 | 1.27 | 0.00175 | 0.00843 |
| miR−200a | 0.195 | 0.151 | 1.29 | 0.00175 | 0.00843 |
| miR−376a | 0.0408 | 0.022 | 1.85 | 0.00177 | 0.00846 |
| miR−693−3p | 0.259 | 0.209 | 1.24 | 0.00179 | 0.00846 |
| miR−682 | 0.14 | 0.103 | 1.36 | 0.00186 | 0.00872 |
| miR−467c | 0.0709 | 0.045 | 1.58 | 0.00191 | 0.00884 |
| miR−10b | 0.105 | 0.0727 | 1.44 | 0.00199 | 0.00912 |
| miR−805 | 0.105 | 0.0727 | 1.44 | 0.00203 | 0.00917 |
| miR−541 | 0.209 | 0.164 | 1.27 | 0.00204 | 0.00917 |
| miR−122 | 0.126 | 0.0909 | 1.39 | 0.00209 | 0.00932 |
| miR−494 | 0.193 | 0.15 | 1.28 | 0.00222 | 0.00984 |
| miR−1186 | 0.168 | 0.128 | 1.31 | 0.00229 | 0.01 |
| miR−290−3p | 0.129 | 0.0943 | 1.37 | 0.00234 | 0.0102 |
| miR−878−3p | 0.128 | 0.093 | 1.37 | 0.00248 | 0.0107 |
| miR−721 | 0.154 | 0.116 | 1.33 | 0.00252 | 0.0108 |
| miR−340−3p | 0.0674 | 0.0429 | 1.57 | 0.00256 | 0.0108 |
| miR−146b | 0.158 | 0.12 | 1.32 | 0.00268 | 0.0112 |
| miR−293 | 0.0461 | 0.0266 | 1.73 | 0.00271 | 0.0112 |
| miR−23a | 0.183 | 0.142 | 1.29 | 0.00274 | 0.0112 |
| miR−369−3p | 0.147 | 0.11 | 1.33 | 0.00274 | 0.0112 |

↓ CR

# Abundance of miRNA Targets

| miRNA | Freq(Obs) | Freq(Exp) | Obs/Exp | P−value | P−Value(Adj) |
|---|---|---|---|---|---|
| miR−217 | 0.16 | 0.121 | 1.31 | 0.00278 | 0.0113 |
| miR−467b | 0.117 | 0.0844 | 1.39 | 0.00283 | 0.0114 |
| miR−743b−5p | 0.144 | 0.108 | 1.33 | 0.00312 | 0.0125 |
| miR−1191 | 0.121 | 0.0879 | 1.37 | 0.00325 | 0.0128 |
| miR−10a | 0.106 | 0.0758 | 1.4 | 0.00325 | 0.0128 |
| miR−497 | 0.232 | 0.188 | 1.24 | 0.00328 | 0.0128 |
| miR−425 | 0.105 | 0.0744 | 1.41 | 0.00336 | 0.013 |
| miR−883b−5p | 0.202 | 0.161 | 1.26 | 0.00366 | 0.014 |
| miR−143 | 0.154 | 0.118 | 1.31 | 0.00393 | 0.015 |
| miR−708 | 0.17 | 0.132 | 1.29 | 0.00399 | 0.0151 |
| miR−509−3p | 0.0833 | 0.0572 | 1.46 | 0.00406 | 0.0152 |
| miR−136 | 0.181 | 0.142 | 1.27 | 0.00409 | 0.0152 |
| miR−486 | 0.0887 | 0.0617 | 1.44 | 0.00415 | 0.0153 |
| miR−713 | 0.124 | 0.0919 | 1.35 | 0.00417 | 0.0153 |
| miR−503 | 0.119 | 0.0874 | 1.36 | 0.00422 | 0.0153 |
| miR−350 | 0.197 | 0.157 | 1.26 | 0.00422 | 0.0153 |
| miR−302b | 0.209 | 0.168 | 1.24 | 0.0043 | 0.0154 |
| miR−489 | 0.122 | 0.0907 | 1.35 | 0.00456 | 0.0162 |
| miR−802 | 0.144 | 0.109 | 1.31 | 0.00459 | 0.0162 |
| miR−704 | 0.108 | 0.0787 | 1.37 | 0.0048 | 0.0168 |
| miR−182 | 0.197 | 0.158 | 1.25 | 0.00535 | 0.0186 |
| miR−592 | 0.131 | 0.0992 | 1.32 | 0.00552 | 0.0191 |
| miR−743b−3p | 0.254 | 0.211 | 1.2 | 0.00564 | 0.0194 |
| miR−300 | 0.142 | 0.109 | 1.3 | 0.00604 | 0.0206 |
| miR−302a | 0.2 | 0.162 | 1.24 | 0.00617 | 0.0209 |
| miR−881 | 0.183 | 0.146 | 1.25 | 0.00637 | 0.0214 |
| let−7a | 0.154 | 0.12 | 1.28 | 0.00643 | 0.0215 |
| miR−532−5p | 0.128 | 0.0969 | 1.32 | 0.00658 | 0.0218 |
| miR−137 | 0.149 | 0.116 | 1.28 | 0.00696 | 0.0229 |
| miR−501−5p | 0.154 | 0.121 | 1.28 | 0.00702 | 0.023 |

↓ CR

# Abundance of miRNA Targets

| miRNA | Freq(Obs) | Freq(Exp) | Obs/Exp | P−value | P−Value(Adj) |
|---|---|---|---|---|---|
| miR−130b | 0.172 | 0.137 | 1.25 | 0.00752 | 0.0245 |
| miR−343 | 0.126 | 0.0959 | 1.31 | 0.0076 | 0.0245 |
| miR−324−5p | 0.0869 | 0.0622 | 1.4 | 0.00775 | 0.0247 |
| miR−669a | 0.152 | 0.12 | 1.27 | 0.00775 | 0.0247 |
| miR−582−3p | 0.115 | 0.0868 | 1.33 | 0.00805 | 0.0249 |
| miR−199a−5p | 0.165 | 0.131 | 1.26 | 0.00808 | 0.0249 |
| miR−466a−5p | 0.19 | 0.154 | 1.24 | 0.00808 | 0.0249 |
| miR−466b−5p | 0.19 | 0.154 | 1.24 | 0.00808 | 0.0249 |
| miR−466e−5p | 0.19 | 0.154 | 1.24 | 0.00808 | 0.0249 |
| let−7d | 0.149 | 0.117 | 1.28 | 0.00812 | 0.0249 |
| miR−23b | 0.177 | 0.142 | 1.25 | 0.00821 | 0.025 |
| miR−763 | 0.181 | 0.146 | 1.24 | 0.00872 | 0.0264 |
| miR−297a | 0.183 | 0.148 | 1.24 | 0.00886 | 0.0267 |
| miR−142−5p | 0.22 | 0.182 | 1.21 | 0.00894 | 0.0267 |
| miR−192 | 0.0709 | 0.0493 | 1.44 | 0.00901 | 0.0267 |
| miR−1−2−as | 0.137 | 0.106 | 1.29 | 0.00903 | 0.0267 |
| miR−298 | 0.246 | 0.207 | 1.19 | 0.0092 | 0.0269 |
| miR−709 | 0.213 | 0.175 | 1.21 | 0.0092 | 0.0269 |
| miR−302c | 0.176 | 0.142 | 1.24 | 0.00964 | 0.028 |
| miR−190b | 0.128 | 0.0986 | 1.3 | 0.00976 | 0.0282 |
| miR−291a−3p | 0.172 | 0.138 | 1.24 | 0.00988 | 0.0284 |
| miR−669c | 0.137 | 0.107 | 1.28 | 0.01 | 0.0286 |
| miR−679 | 0.126 | 0.0972 | 1.29 | 0.0103 | 0.0292 |
| miR−466j | 0.179 | 0.145 | 1.23 | 0.0106 | 0.0297 |
| miR−195 | 0.222 | 0.185 | 1.2 | 0.0106 | 0.0297 |
| miR−142−3p | 0.131 | 0.102 | 1.29 | 0.0106 | 0.0297 |
| miR−683 | 0.163 | 0.131 | 1.25 | 0.0108 | 0.03 |
| miR−381 | 0.177 | 0.144 | 1.23 | 0.0112 | 0.0309 |
| miR−451 | 0.0514 | 0.0341 | 1.51 | 0.0116 | 0.0317 |
| let−7c | 0.156 | 0.125 | 1.25 | 0.0116 | 0.0317 |

↓ CR

# Abundance of miRNA Targets

| miRNA | Freq(Obs) | Freq(Exp) | Obs/Exp | P−value | P−Value(Adj) |
|---|---|---|---|---|---|
| miR−219 | 0.0691 | 0.0487 | 1.42 | 0.0118 | 0.0321 |
| miR−153 | 0.126 | 0.098 | 1.28 | 0.0122 | 0.0328 |
| miR−466k | 0.186 | 0.153 | 1.22 | 0.0124 | 0.0332 |
| miR−183 | 0.135 | 0.106 | 1.27 | 0.0125 | 0.0333 |
| miR−144 | 0.179 | 0.146 | 1.22 | 0.0129 | 0.0343 |
| miR−101a | 0.142 | 0.113 | 1.26 | 0.0131 | 0.0343 |
| miR−197 | 0.101 | 0.0764 | 1.32 | 0.0131 | 0.0343 |
| miR−302d | 0.197 | 0.163 | 1.21 | 0.0131 | 0.0343 |
| miR−703 | 0.154 | 0.124 | 1.24 | 0.0135 | 0.0352 |
| miR−598 | 0.0567 | 0.039 | 1.46 | 0.014 | 0.0362 |
| miR−1194 | 0.137 | 0.108 | 1.26 | 0.0146 | 0.0374 |
| miR−543 | 0.145 | 0.116 | 1.25 | 0.0146 | 0.0374 |
| miR−297b−5p | 0.172 | 0.141 | 1.22 | 0.0149 | 0.0379 |
| miR−666−5p | 0.0691 | 0.0495 | 1.4 | 0.015 | 0.038 |
| miR−1196 | 0.0851 | 0.0633 | 1.34 | 0.0157 | 0.0395 |
| miR−511 | 0.167 | 0.136 | 1.22 | 0.0158 | 0.0395 |
| miR−380−5p | 0.0922 | 0.0696 | 1.32 | 0.0163 | 0.0407 |
| miR−804 | 0.11 | 0.0852 | 1.29 | 0.0164 | 0.0407 |
| miR−488 | 0.188 | 0.156 | 1.2 | 0.017 | 0.0419 |
| miR−490 | 0.106 | 0.0822 | 1.29 | 0.0171 | 0.0419 |
| miR−214 | 0.213 | 0.179 | 1.19 | 0.0173 | 0.0422 |
| miR−186 | 0.241 | 0.206 | 1.17 | 0.0177 | 0.043 |
| miR−338−3p | 0.152 | 0.124 | 1.23 | 0.0181 | 0.0437 |
| miR−28 | 0.147 | 0.119 | 1.24 | 0.0181 | 0.0437 |
| miR−190 | 0.17 | 0.14 | 1.21 | 0.0185 | 0.0442 |
| miR−322 | 0.23 | 0.196 | 1.17 | 0.0186 | 0.0442 |
| miR−653 | 0.129 | 0.103 | 1.25 | 0.0186 | 0.0442 |
| miR−15a | 0.223 | 0.19 | 1.18 | 0.0199 | 0.0469 |
| miR−672 | 0.147 | 0.12 | 1.23 | 0.0202 | 0.0475 |
| miR−467h | 0.168 | 0.139 | 1.21 | 0.0204 | 0.0478 |

↓ CR

# Abundance of miRNA Targets

| miRNA | Freq(Obs) | Freq(Exp) | Obs/Exp | P−value | P−Value(Adj) |
|---|---|---|---|---|---|
| miR−297c | 0.183 | 0.152 | 1.2 | 0.0206 | 0.0479 |
| miR−410 | 0.11 | 0.0863 | 1.27 | 0.021 | 0.0483 |
| miR−196a | 0.138 | 0.112 | 1.24 | 0.0211 | 0.0483 |
| miR−154 | 0.0975 | 0.0753 | 1.29 | 0.0212 | 0.0483 |
| miR−325 | 0.156 | 0.128 | 1.22 | 0.0213 | 0.0483 |
| miR−466h | 0.156 | 0.128 | 1.22 | 0.0213 | 0.0483 |
| miR−467e | 0.152 | 0.125 | 1.22 | 0.0217 | 0.0489 |
| miR−146a | 0.145 | 0.118 | 1.23 | 0.0217 | 0.0489 |
| miR−879 | 0.119 | 0.0944 | 1.26 | 0.0219 | 0.0492 |
| miR−876−3p | 0.167 | 0.138 | 1.21 | 0.0222 | 0.0492 |
| miR−761 | 0.207 | 0.176 | 1.18 | 0.0222 | 0.0492 |
| miR−15b | 0.222 | 0.189 | 1.17 | 0.0223 | 0.0492 |
| miR−666−3p | 0.16 | 0.132 | 1.21 | 0.0224 | 0.0492 |
| miR−539 | 0.207 | 0.176 | 1.18 | 0.023 | 0.0504 |
| miR−98 | 0.149 | 0.122 | 1.22 | 0.0234 | 0.051 |
| miR−327 | 0.113 | 0.09 | 1.26 | 0.0237 | 0.0515 |
| miR−674 | 0.181 | 0.152 | 1.19 | 0.0241 | 0.0521 |
| miR−201 | 0.138 | 0.113 | 1.23 | 0.0249 | 0.0535 |
| miR−34b−3p | 0.0869 | 0.0668 | 1.3 | 0.0255 | 0.0548 |
| miR−690 | 0.128 | 0.103 | 1.24 | 0.0258 | 0.0551 |
| miR−378 | 0.122 | 0.0985 | 1.24 | 0.026 | 0.0553 |
| miR−452 | 0.167 | 0.139 | 1.2 | 0.0265 | 0.056 |
| miR−191 | 0.0869 | 0.067 | 1.3 | 0.027 | 0.0569 |
| miR−467f | 0.145 | 0.12 | 1.21 | 0.0279 | 0.0584 |
| miR−574−5p | 0.168 | 0.141 | 1.19 | 0.0279 | 0.0584 |
| miR−717 | 0.168 | 0.141 | 1.19 | 0.0287 | 0.0598 |
| miR−291a−5p | 0.16 | 0.133 | 1.2 | 0.0303 | 0.0622 |
| miR−291b−5p | 0.16 | 0.133 | 1.2 | 0.0303 | 0.0622 |
| miR−376c | 0.158 | 0.132 | 1.2 | 0.0304 | 0.0622 |
| miR−491 | 0.124 | 0.101 | 1.23 | 0.0306 | 0.0622 |

↓CR

# Abundance of miRNA Targets

| miRNA | Freq(Obs) | Freq(Exp) | Obs/Exp | P−value | P−Value(Adj) |
|---|---|---|---|---|---|
| miR−379 | 0.0638 | 0.0475 | 1.34 | 0.0306 | 0.0622 |
| miR−9 | 0.206 | 0.176 | 1.17 | 0.0307 | 0.0622 |
| miR−669d | 0.158 | 0.132 | 1.2 | 0.0314 | 0.0634 |
| miR−181c | 0.176 | 0.149 | 1.18 | 0.0326 | 0.0656 |
| miR−187 | 0.0851 | 0.0664 | 1.28 | 0.0335 | 0.0672 |
| miR−202−3p | 0.0993 | 0.0791 | 1.26 | 0.0339 | 0.0676 |
| miR−181d | 0.204 | 0.175 | 1.16 | 0.0342 | 0.0679 |
| miR−464 | 0.152 | 0.128 | 1.19 | 0.0352 | 0.0696 |
| miR−483 | 0.0851 | 0.0668 | 1.27 | 0.0362 | 0.0713 |
| miR−377 | 0.197 | 0.169 | 1.16 | 0.0364 | 0.0714 |
| miR−129−5p | 0.149 | 0.125 | 1.19 | 0.0372 | 0.0727 |
| miR−695 | 0.0816 | 0.0638 | 1.28 | 0.0373 | 0.0727 |
| miR−194 | 0.126 | 0.104 | 1.21 | 0.0381 | 0.0739 |
| miR−883a−5p | 0.179 | 0.153 | 1.17 | 0.0388 | 0.075 |
| miR−140 | 0.129 | 0.107 | 1.21 | 0.0394 | 0.0757 |
| miR−466f−5p | 0.0904 | 0.0719 | 1.26 | 0.0395 | 0.0757 |
| miR−875−3p | 0.218 | 0.19 | 1.15 | 0.0401 | 0.0763 |
| miR−582−5p | 0.144 | 0.12 | 1.19 | 0.0403 | 0.0763 |
| miR−876−5p | 0.14 | 0.117 | 1.2 | 0.0403 | 0.0763 |
| miR−295 | 0.181 | 0.155 | 1.17 | 0.0406 | 0.0768 |
| miR−26b | 0.152 | 0.129 | 1.18 | 0.0418 | 0.0787 |
| let−7i | 0.147 | 0.124 | 1.19 | 0.042 | 0.0787 |
| miR−493 | 0.117 | 0.0963 | 1.22 | 0.0426 | 0.0796 |
| miR−33 | 0.119 | 0.0981 | 1.21 | 0.0443 | 0.0824 |
| miR−22 | 0.131 | 0.11 | 1.2 | 0.0448 | 0.083 |
| miR−1187 | 0.191 | 0.166 | 1.15 | 0.0466 | 0.086 |
| let−7e | 0.163 | 0.139 | 1.17 | 0.0467 | 0.086 |
| miR−872 | 0.112 | 0.0921 | 1.21 | 0.0474 | 0.0868 |
| miR−684 | 0.151 | 0.128 | 1.18 | 0.0481 | 0.0878 |

# Tests for Chromosome Over−representation

| Chromosome | CR−upregulated Genes | CR−downregulated Genes |
|---|---|---|
| 1 | 0.774 | 0.126 |
| 2 | 0.339 | 0.85 |
| 3 | 0.766 | 0.364 |
| 4 | 0.541 | 0.315 |
| 5 | 0.0436* | 0.717 |
| 6 | 0.617 | 0.997 |
| 7 | 0.551 | 0.948 |
| 8 | 0.174 | 0.123 |
| 9 | 0.818 | 0.648 |
| 10 | 0.342 | 0.274 |
| 11 | 0.844 | 0.356 |
| 12 | 0.821 | 0.0345* |
| 13 | 0.913 | 0.708 |
| 14 | 0.938 | 0.485 |
| 15 | 0.217 | 0.899 |
| 16 | 0.91 | 0.476 |
| 17 | 0.301 | 0.487 |
| 18 | 0.984 | 0.081 |
| 19 | 0.148 | 0.891 |
| X | 0.917 | 0.632 |
| Y | 1.00 | 0.139 |

The table lists p−values generated from a test that evaluates whether there exists an over−abundance of identified genes with respect to a given chromosome. The null hypothesis assumes that the set of genes has been selected at random from those represented on the Affymetrix 430 2.0 array. A significant test indicates that a chromosome contains more of the identified genes than would be expected if the gene set had been chosen at random.

* = significant p−value, without multiple test adjustment

** = significant p−value, with multiple test adjustment

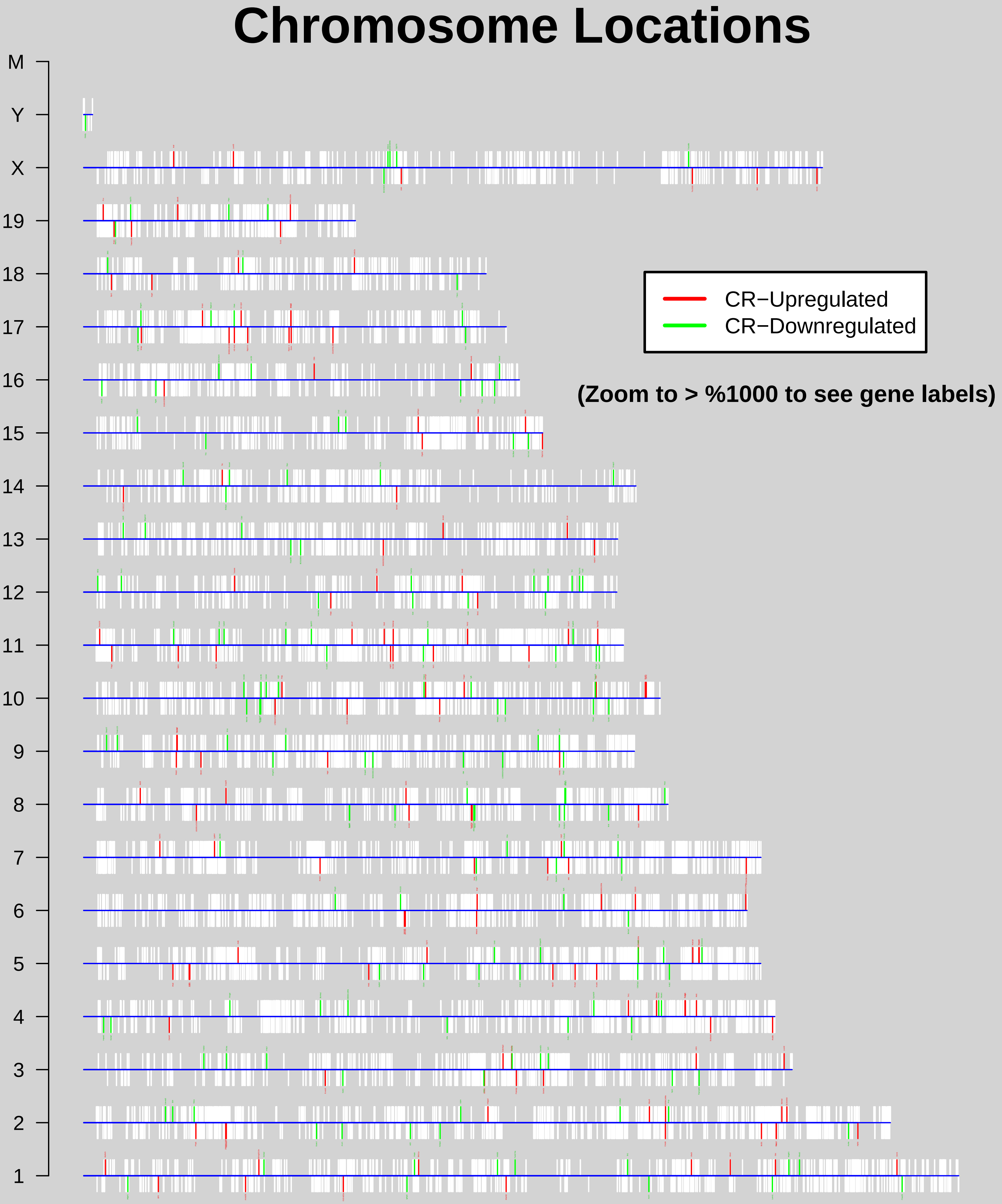

Chromosome Locations
M
Y
X
19
18
17
16
15
14
13
12
11
10
9
8
7
6
5
4
3
2
1
CR−Upregulated
CR−Downregulated
(Zoom to > %1000 to see gene labels)
