# Supplementary material for: Genes and gene expression modules associated with caloric restriction and aging in the laboratory mouse
Source: BMC Genomics. 2009 Dec 7;10:585. doi: 10.1186/1471-2164-10-585 (PMC2795771; doi:10.1186/1471-2164-10-585)
Supplement: Additional file 5 — Genes regulated by caloric restriction in multiple mouse tissues. A gene chart is presented that provides a comprehensive listing of the genes most strongly increased by CR across tissues, most strongly decreased by CR across tissues, and most strongly regulated by CR (in either direction) across tissues. The chart is comparable to those shown in Figure 2, except genes are ranked based upon a p-value generated using Fisher's method, rather than the total number of tissue types in which a gene is up or down regulated by CR. This file also includes analysis of associated gene ontology terms, KEGG pathways, microRNA targets and chromosomal locations of CR-regulated genes. [file 1471-2164-10-585-S5.PDF]

Additional File 5

Genes and Gene Expression Modules Associated with Caloric Restriction and Aging in the Laboratory Mouse

William R. Swindell
University of Michigan, Departments of Pathology and Geriatrics

---

Common Responses to CR across 17 Mouse Tissues

This file provides information on genes regulated by CR significantly among all 17 tissues included in the analysis. The first set of charts displays differential expression results of the 300 genes most strongly up regulated by CR across tissues, while the second set of charts displays differential expression results for the 300 genes most strongly down regulated by CR across tissues. An additional set of charts shows the top 300 most significant genes based upon a two-sided significance test for regulation by CR across tissues (either up or down in each tissue). Each row corresponds to an individual gene and each column corresponds to one of 17 tissues analyzed. Symbols are interpreted as follows:

- ● Gene is significantly up regulated by CR ($P_u < 0.05$)
- ● Gene is significantly down regulated by CR ($P_d < 0.05$)
- ▬ Gene is marginally up regulated by CR ($0.05 < P_u < 0.10$)
- ▬ Gene is marginally down regulated by CR ($0.05 < P_d < 0.10$)
- ▬ Non-significant CR effect ($P_u > 0.10$ and $P_d > 0.10$)
- × No data (gene not represented for a given tissue or array annotation was limiting)
- ✳ Evidence conflicts, but favors up regulation by CR
- ✳ Evidence conflicts, but favors down regulation by CR

The last two categories (✳ and ✳) indicate significant effects with conflicting evidence. This can arise if there is significant up regulation by CR in one experiment, and significant down regulation by CR in another experiment that has examined the same tissue. Alternatively, a conflict may arise if $P_u < 0.05$ and also $P_d < 0.05$ for a given tissue type. Symbols shown in charts are based upon a comparison-wise type I error rate of 0.05. The final column in each chart lists meta-analysis p-values generated using Fisher's method, which have been adjusted using the Benjamini-Hochberg method to control the false discovery rate among all 21,327 genes.

The remainder of the file includes lists of over-represented gene ontology terms, over-represented KEGG pathways, and over-represented KEGG pathways defined based upon IP domain signatures (see Hahne et al. 2008, BMC Bioinformatics 9:3). Genes were also analyzed to determine if there existed an over-abundance of targets for certain microRNAs (see Betel et al. 2008, Nucleic Acids Res. 36: D149-153), and a list of associated microRNAs is provided based upon this analysis. Lastly, tests for over-representation of identified genes with respect to each chromosome were performed, and an idiogram mapping of identified genes to chromosomal locations is shown.

Contact: William R. Swindell, wswindel@umich.edu

↑ CR

Genes up regulated by CR

	cbm	cln	coc	gon	kid	lng	mmy	spc	str	thm	ctx	hip	hrt	hyp	lvr	msl	wat	P_u
Sgk1	−	red ●	red ●	−	red ●	red ●	−	red ●	−	green ●	red ●	red ●	red ●	red ●	red ●	−	red ●	1.04e−10
Tsc22d3	×	red ●	red ●	×	×	−	red ●	×	×	×	red ●	−	red ●	red ●	red ●	red ●	red −	1.72e−10
Zbtb16	×	red ●	red ●	−	×	red ●	−	×	×	×	red ●	−	red ●	red −	red ●	red ●	red ●	3.35e−10
BC055107	×	red ●	red ●	×	×	×	×	×	×	×	red ●	×	red ●	red ●	red ●	red ●	−	4.58e−10
Mt2	red ●	red ●	red ●	red −	green ●	red ●	green ●	red ●	red ●	−	red ●	red ●	red ●	−	green *	red ●	green *	4.58e−10
Sult1a1	×	−	red ●	−	green ●	red ●	−	×	×	×	red ●	×	red ●	red ●	red ●	red ●	red ●	9.05e−10
Per1	×	red ●	red ●	red ●	×	red ●	green ●	×	×	×	green ●	−	red ●	red −	red ●	red ●	red ●	1.96e−09
Timp3	−	−	red ●	−	×	×	red ●	−	red ●	green ●	red ●	−	red ●	red ●	red ●	red ●	×	8.72e−09
S3−12	×	red ●	red ●	×	×	red −	green −	×	×	×	red ●	red ●	red ●	red ●	red ●	red *	green ●	1.08e−08
Dnajb6	−	red ●	red ●	×	×	red −	red ●	−	red ●	−	red ●	green ●	red ●	−	red ●	red ●	red ●	1.38e−08
Pnpla2	green ●	red ●	red ●	×	×	red ●	−	−	−	red −	−	red −	red ●	red ●	red ●	red ●	red ●	1.38e−08
Ppargc1a	×	red ●	red ●	red −	×	×	red ●	×	×	×	red ●	−	red ●	red ●	red ●	red ●	−	1.49e−08
Cp	−	green ●	red ●	×	−	red ●	green ●	green ●	−	−	red ●	red ●	red ●	−	red ●	red ●	red ●	1.86e−08
1200016E24Rik	×	green −	red ●	×	×	×	red ●	×	×	×	red ●	×	red ●	red −	red ●	red ●	×	2e−08
Ddit4	×	−	red ●	×	−	red ●	−	×	×	×	red ●	red −	red ●	red ●	red ●	red ●	−	2e−08
Pim3	−	red ●	red ●	−	red −	red ●	green ●	−	−	−	red ●	red ●	red ●	red ●	red ●	red ●	−	2.42e−08
Cdkn1a	×	red −	red ●	×	−	red ●	−	×	×	×	red ●	−	red ●	red ●	red ●	red ●	green ●	2.9e−08
Arih2	−	−	red ●	×	red −	red ●	green ●	red ●	−	green ●	red ●	red ●	red ●	red −	red ●	red ●	red ●	3.6e−08
Mpzl2	×	red ●	red ●	×	×	green −	red ●	×	×	×	red ●	−	red −	−	red ●	red ●	red ●	4.35e−08
Txnip	−	−	red ●	−	×	red ●	red ●	red ●	−	−	red ●	green ●	red ●	red ●	red ●	red *	red ●	4.35e−08
Hspa9	−	green ●	red ●	×	red ●	red ●	red ●	−	−	red −	red ●	green ●	red ●	red ●	red ●	red ●	red ●	4.88e−08
Rora	×	green −	red ●	−	−	red ●	−	×	×	×	red ●	−	red ●	red ●	red ●	red *	red ●	4.88e−08
Ccrn4l	−	red ●	red ●	red ●	red ●	red ●	red ●	−	red ●	−	−	red ●	red ●	−	red *	red ●	−	4.88e−08
Map3k6	×	−	red −	×	×	red ●	−	×	×	×	red ●	−	red ●	red −	red ●	red ●	red ●	4.88e−08
Ptp4a1	green ●	green ●	red ●	red ●	−	red ●	red ●	red ●	−	−	red ●	green −	red ●	red ●	red ●	green ●	−	5.21e−08
Acsl1	−	red ●	red ●	green ●	red ●	−	red ●	−	−	red ●	red ●	green ●	red ●	green −	green *	red ●	red ●	5.95e−08
Fbxo9	×	red −	red ●	red ●	×	−	red ●	×	×	×	red ●	−	red ●	red ●	red ●	red *	−	6.23e−08
Serinc3	red ●	green ●	red ●	red ●	−	red ●	green ●	−	−	−	red ●	green ●	red ●	red ●	red ●	red ●	red ●	6.23e−08
Klf9	−	red ●	red ●	×	×	×	green ●	−	−	−	red ●	−	red ●	red ●	red ●	red ●	×	7.3e−08
Phip	red −	−	red ●	red ●	×	×	green ●	green −	red ●	−	red ●	green ●	red ●	red ●	green ●	red ●	×	7.3e−08

↑ CR

Genes up regulated by CR

	cbm	cln	coc	gon	kid	lng	mmy	spc	str	thm	ctx	hip	hrt	hyp	lvr	msl	wat	P_u
Wdr45	×	red ●	red –	×	red ●	red ●	red –	×	×	×	red ●	×	green *	green –	red ●	red *	red ●	7.3e−08
2310051E17Rik	–	red ●	red ●	×	×	red ●	green ●	–	–	–	red ●	–	red ●	red –	green ●	red ●	red ●	7.32e−08
5730469M10Rik	–	red –	red ●	×	red ●	red ●	–	green ●	red –	green ●	red –	red –	red ●	red ●	red –	red ●	red ●	7.62e−08
Pdk1	–	red ●	red ●	×	×	red –	red ●	–	red –	–	red ●	–	red ●	red ●	red ●	green *	red ●	7.62e−08
Sorbs1	red ●	–	red ●	green ●	×	red ●	red –	–	red ●	red ●	red ●	green ●	red ●	red ●	green ●	red ●	–	7.62e−08
Cirbp	–	red –	red ●	×	×	red ●	green ●	green ●	green –	–	red ●	green –	red ●	red ●	red ●	red ●	red ●	7.62e−08
Mt1	–	red ●	red ●	×	×	red ●	green –	–	–	–	–	red –	red ●	red ●	red ●	red ●	red ●	8.41e−08
Tgoln1	–	green ●	red ●	red ●	–	–	red ●	–	green –	green –	red ●	red ●	red ●	red ●	red ●	red *	red ●	8.7e−08
Wee1	×	red ●	green ●	×	×	green –	red ●	×	×	×	red ●	–	red ●	–	red ●	red ●	red ●	9.28e−08
Errfi1	–	red –	red ●	×	red ●	red ●	red ●	–	–	–	red ●	green ●	red ●	–	red ●	red *	–	1.14e−07
Serinc1	–	green –	red ●	red ●	×	red ●	red ●	–	–	–	red –	–	red ●	red ●	red ●	red *	green ●	1.2e−07
Selenbp1	×	–	red –	–	red ●	red ●	–	×	×	×	–	×	red ●	red ●	red ●	red ●	red ●	1.2e−07
Per2	×	red ●	red ●	–	×	–	red –	×	×	×	green ●	green ●	red ●	red ●	red ●	red ●	red ●	1.71e−07
Zwint	–	red ●	red ●	×	×	–	red ●	–	red –	–	red ●	–	red ●	red ●	red ●	red ●	red ●	1.75e−07
Fmo1	×	green ●	red ●	×	red ●	red –	–	×	×	×	red ●	–	red ●	–	red ●	red ●	red ●	1.77e−07
Nfkbia	green –	–	red ●	red –	–	red ●	green ●	red ●	–	–	red ●	–	red ●	red ●	red ●	red ●	–	1.77e−07
Rnf14	red ●	–	red ●	green ●	–	–	red ●	green ●	–	red ●	red ●	red ●	red ●	red –	red ●	red ●	–	1.77e−07
Syncrip	red –	–	red ●	×	red ●	red ●	–	red ●	–	–	red ●	–	red ●	red ●	red ●	red *	red ●	1.77e−07
C80913	–	red ●	red ●	–	red ●	red ●	green ●	red ●	–	red –	red ●	–	red ●	red ●	red ●	red ●	red ●	1.9e−07
4833442J19Rik	×	red ●	red ●	×	×	×	–	×	×	×	–	×	red ●	–	red ●	red ●	×	1.99e−07
Fkbp5	–	red ●	red ●	green ●	green ●	red ●	–	–	–	–	red ●	–	red ●	red ●	red ●	red ●	red ●	1.99e−07
0610007C21Rik	×	red ●	red ●	×	×	–	red ●	×	×	×	red ●	–	red ●	–	red ●	red ●	red ●	1.99e−07
Chchd7	×	red ●	red ●	green ●	–	–	–	×	×	×	red ●	–	red ●	–	red ●	red ●	–	2.13e−07
Fmo2	×	red ●	red ●	×	×	×	red ●	×	×	×	red ●	–	red ●	green ●	red ●	red ●	×	2.19e−07
Glul	–	red –	red ●	–	×	red ●	–	–	green –	red ●	green ●	green ●	red ●	red ●	red ●	red ●	red ●	2.4e−07
Gna13	green –	red ●	red ●	×	–	red ●	green ●	–	–	–	red ●	red –	green *	red ●	red ●	red ●	green ●	2.4e−07
Pdha1	–	–	red ●	red –	–	–	red ●	red –	–	red –	red ●	red ●	red ●	red ●	red ●	red *	red ●	2.4e−07
Lyrm5	×	red ●	red ●	×	×	×	–	×	×	×	red ●	–	red ●	red ●	green ●	red ●	×	2.62e−07
Nedd4	–	green ●	red ●	×	–	red ●	red ●	green ●	–	–	green –	red ●	red ●	red ●	red ●	red *	–	2.64e−07
Angptl4	×	red –	–	green –	red ●	red ●	–	×	×	×	red ●	–	red ●	–	red ●	red ●	–	2.66e−07

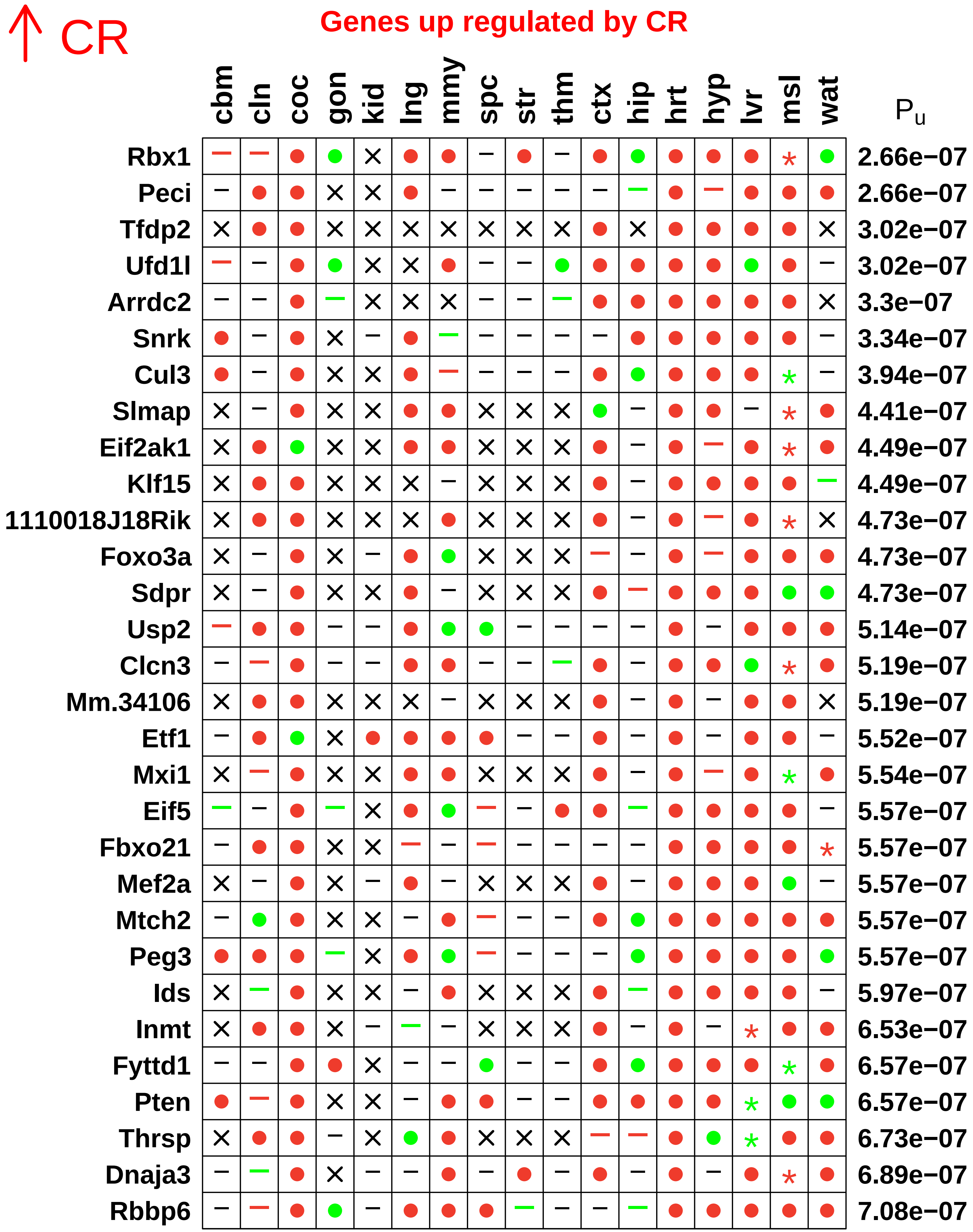

↑ CR
Genes up regulated by CR
cbm cln coc gon kid lng mmy spc str thm ctx hip hrt hyp lvr msl wat
Pu
Rbx1 2.66e−07
Peci 2.66e−07
Tfdp2 3.02e−07
Ufd1l 3.02e−07
Arrdc2 3.3e−07
Snrk 3.34e−07
Cul3 3.94e−07
Slmap 4.41e−07
Eif2ak1 4.49e−07
Klf15 4.49e−07
1110018J18Rik 4.73e−07
Foxo3a 4.73e−07
Sdpr 4.73e−07
Usp2 5.14e−07
Clcn3 5.19e−07
Mm.34106 5.19e−07
Etf1 5.52e−07
Mxi1 5.54e−07
Eif5 5.57e−07
Fbxo21 5.57e−07
Mef2a 5.57e−07
Mtch2 5.57e−07
Peg3 5.57e−07
Ids 5.97e−07
Inmt 6.53e−07
Fyttd1 6.57e−07
Pten 6.57e−07
Thrsp 6.73e−07
Dnaja3 6.89e−07
Rbbp6 7.08e−07

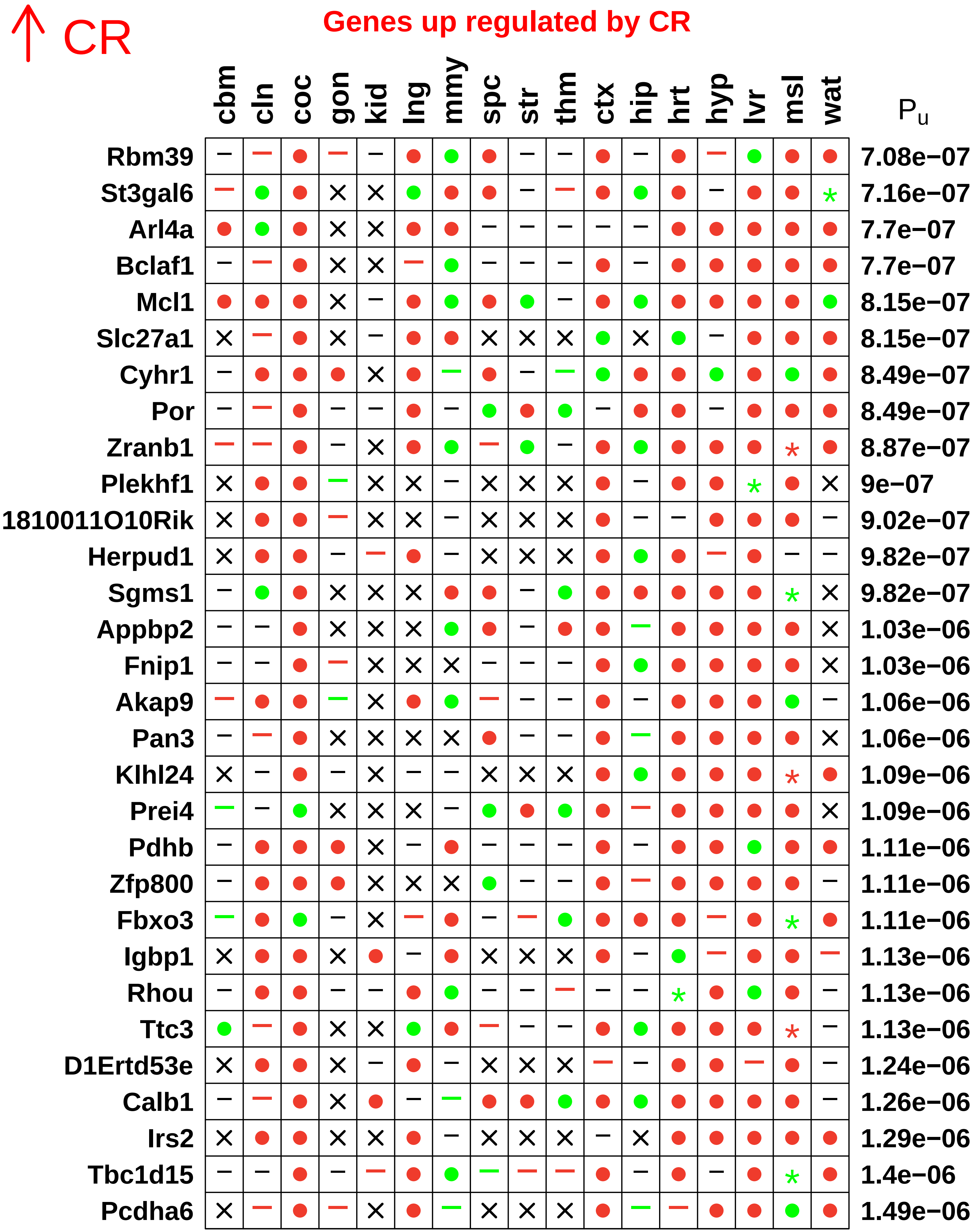

↑ CR
Genes up regulated by CR
cbm
cln
coc
gon
kid
lng
mmy
spc
str
thm
ctx
hip
hrt
hyp
lvr
msl
wat
Pu
Rbm39
St3gal6
Arl4a
Bclaf1
Mcl1
Slc27a1
Cyhr1
Por
Zranb1
Plekhf1
1810011O10Rik
Herpud1
Sgms1
Appbp2
Fnip1
Akap9
Pan3
Klhl24
Prei4
Pdhb
Zfp800
Fbxo3
Igbp1
Rhou
Ttc3
D1Ertd53e
Calb1
Irs2
Tbc1d15
Pcdha6
7.08e−07
7.16e−07
7.7e−07
7.7e−07
8.15e−07
8.15e−07
8.49e−07
8.49e−07
8.87e−07
9e−07
9.02e−07
9.82e−07
9.82e−07
1.03e−06
1.03e−06
1.06e−06
1.06e−06
1.09e−06
1.09e−06
1.11e−06
1.11e−06
1.11e−06
1.13e−06
1.13e−06
1.13e−06
1.24e−06
1.26e−06
1.29e−06
1.4e−06
1.49e−06

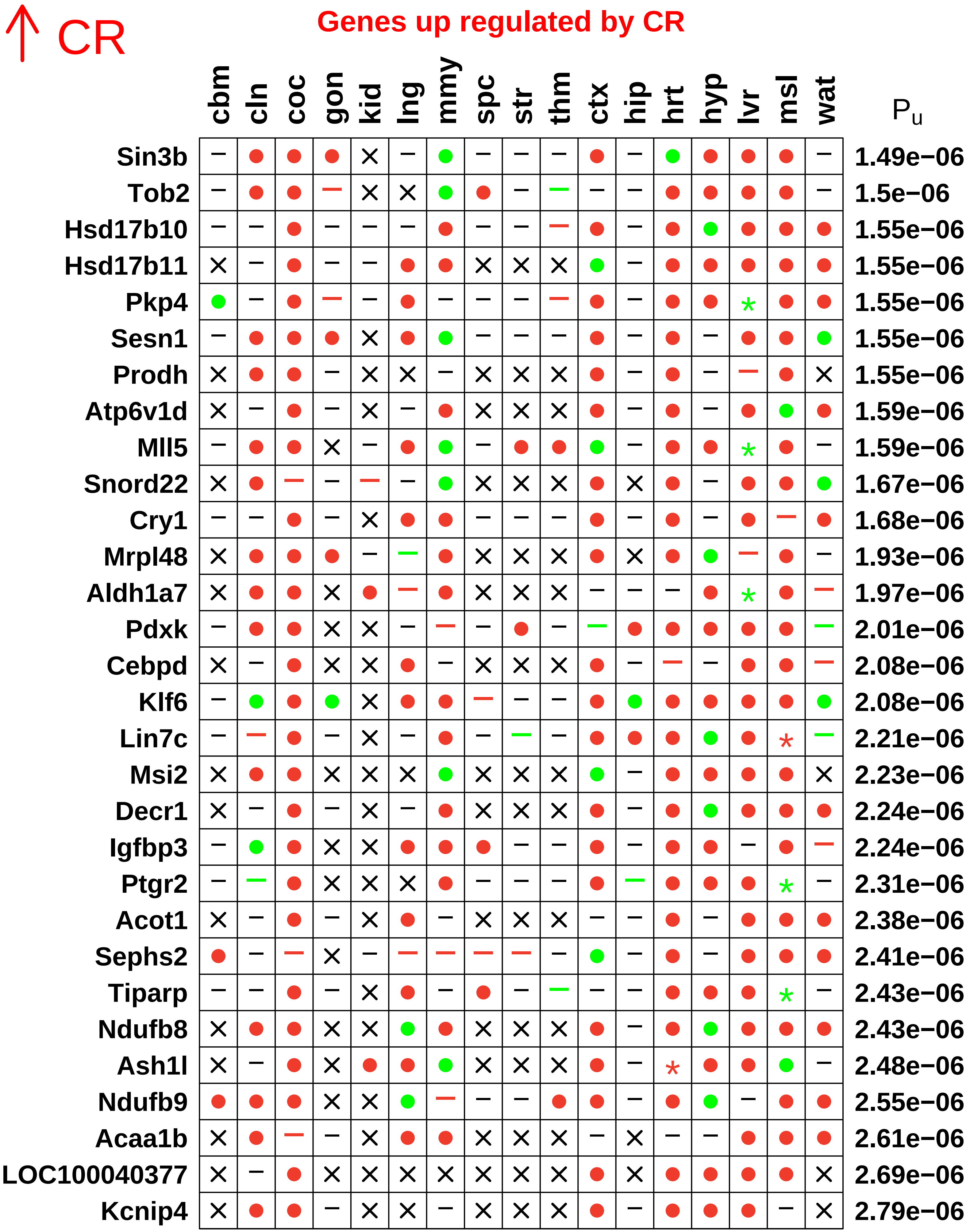

↑ CR
Genes up regulated by CR
cbm
cln
coc
gon
kid
lng
mmy
spc
str
thm
ctx
hip
hrt
hyp
lvr
msl
wat
P_u
Sin3b
1.49e−06
Tob2
1.5e−06
Hsd17b10
1.55e−06
Hsd17b11
1.55e−06
Pkp4
1.55e−06
Sesn1
1.55e−06
Prodh
1.55e−06
Atp6v1d
1.59e−06
Mll5
1.59e−06
Snord22
1.67e−06
Cry1
1.68e−06
Mrpl48
1.93e−06
Aldh1a7
1.97e−06
Pdxk
2.01e−06
Cebpd
2.08e−06
Klf6
2.08e−06
Lin7c
2.21e−06
Msi2
2.23e−06
Decr1
2.24e−06
Igfbp3
2.24e−06
Ptgr2
2.31e−06
Acot1
2.38e−06
Sephs2
2.41e−06
Tiparp
2.43e−06
Ndufb8
2.43e−06
Ash1l
2.48e−06
Ndufb9
2.55e−06
Acaa1b
2.61e−06
LOC100040377
2.69e−06
Kcnip4
2.79e−06

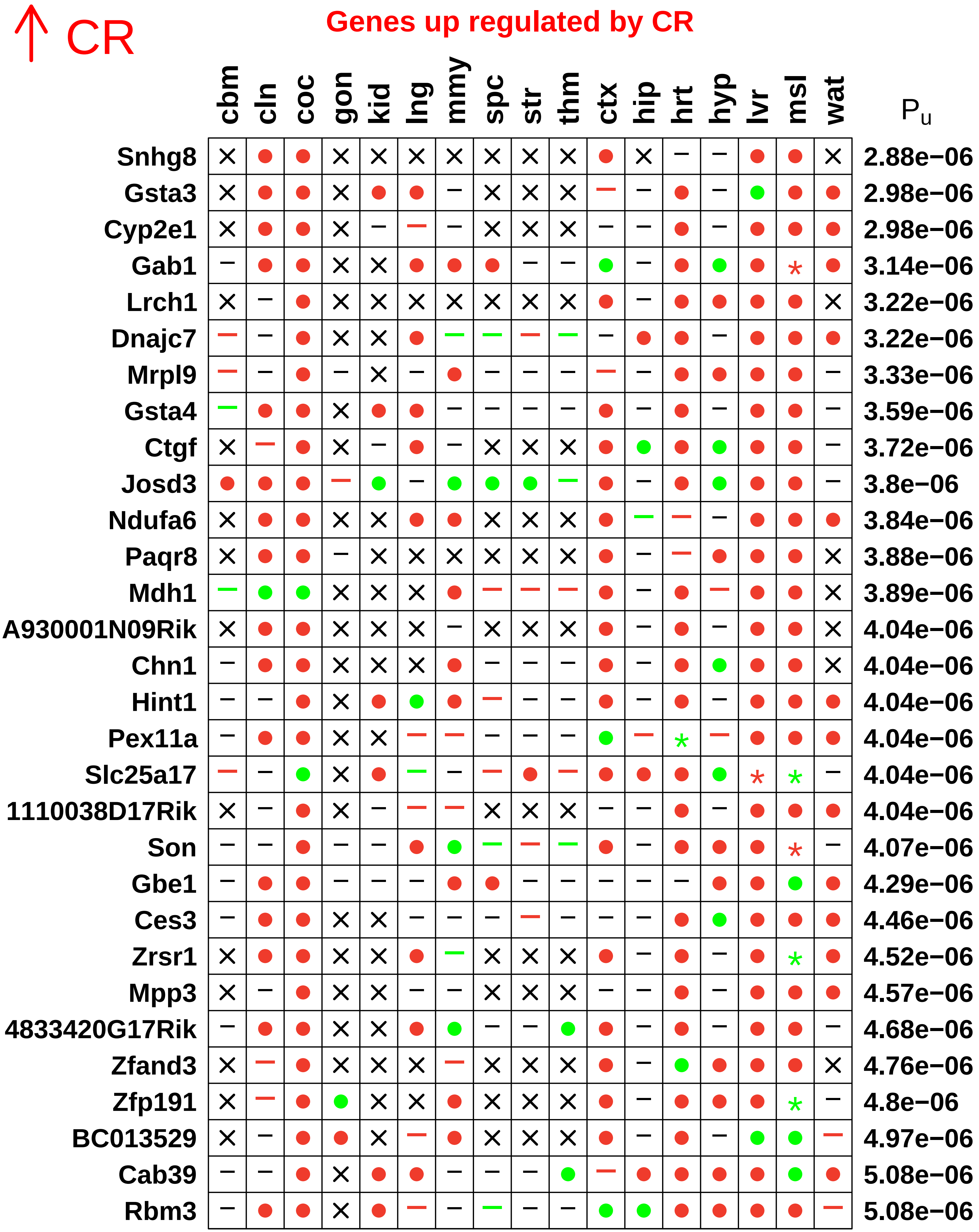
↑ CR
Genes up regulated by CR
cbm
cln
coc
gon
kid
lng
mmy
spc
str
thm
ctx
hip
hrt
hyp
lvr
msl
wat
P_u
Snhg8 2.88e−06
Gsta3 2.98e−06
Cyp2e1 2.98e−06
Gab1 3.14e−06
Lrch1 3.22e−06
Dnajc7 3.22e−06
Mrpl9 3.33e−06
Gsta4 3.59e−06
Ctgf 3.72e−06
Josd3 3.8e−06
Ndufa6 3.84e−06
Paqr8 3.88e−06
Mdh1 3.89e−06
A930001N09Rik 4.04e−06
Chn1 4.04e−06
Hint1 4.04e−06
Pex11a 4.04e−06
Slc25a17 4.04e−06
1110038D17Rik 4.04e−06
Son 4.07e−06
Gbe1 4.29e−06
Ces3 4.46e−06
Zrsr1 4.52e−06
Mpp3 4.57e−06
4833420G17Rik 4.68e−06
Zfand3 4.76e−06
Zfp191 4.8e−06
BC013529 4.97e−06
Cab39 5.08e−06
Rbm3 5.08e−06

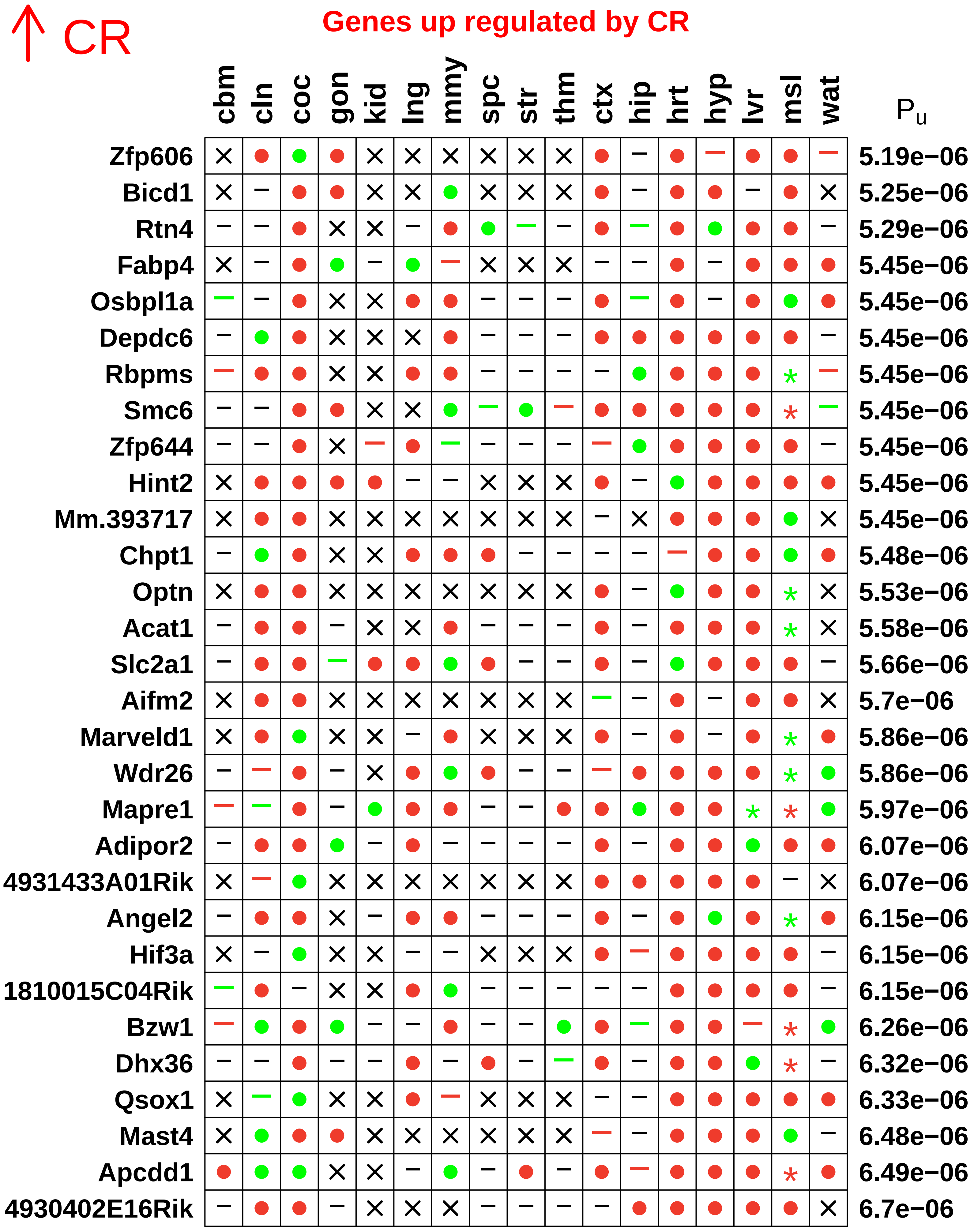
↑ CR
Genes up regulated by CR
cbm
cln
coc
gon
kid
lng
mmy
spc
str
thm
ctx
hip
hrt
hyp
lvr
msl
wat
P_u
Zfp606 5.19e−06
Bicd1 5.25e−06
Rtn4 5.29e−06
Fabp4 5.45e−06
Osbpl1a 5.45e−06
Depdc6 5.45e−06
Rbpms 5.45e−06
Smc6 5.45e−06
Zfp644 5.45e−06
Hint2 5.45e−06
Mm.393717 5.45e−06
Chpt1 5.48e−06
Optn 5.53e−06
Acat1 5.58e−06
Slc2a1 5.66e−06
Aifm2 5.7e−06
Marveld1 5.86e−06
Wdr26 5.86e−06
Mapre1 5.97e−06
Adipor2 6.07e−06
4931433A01Rik 6.07e−06
Angel2 6.15e−06
Hif3a 6.15e−06
1810015C04Rik 6.15e−06
Bzw1 6.26e−06
Dhx36 6.32e−06
Qsox1 6.33e−06
Mast4 6.48e−06
Apcdd1 6.49e−06
4930402E16Rik 6.7e−06

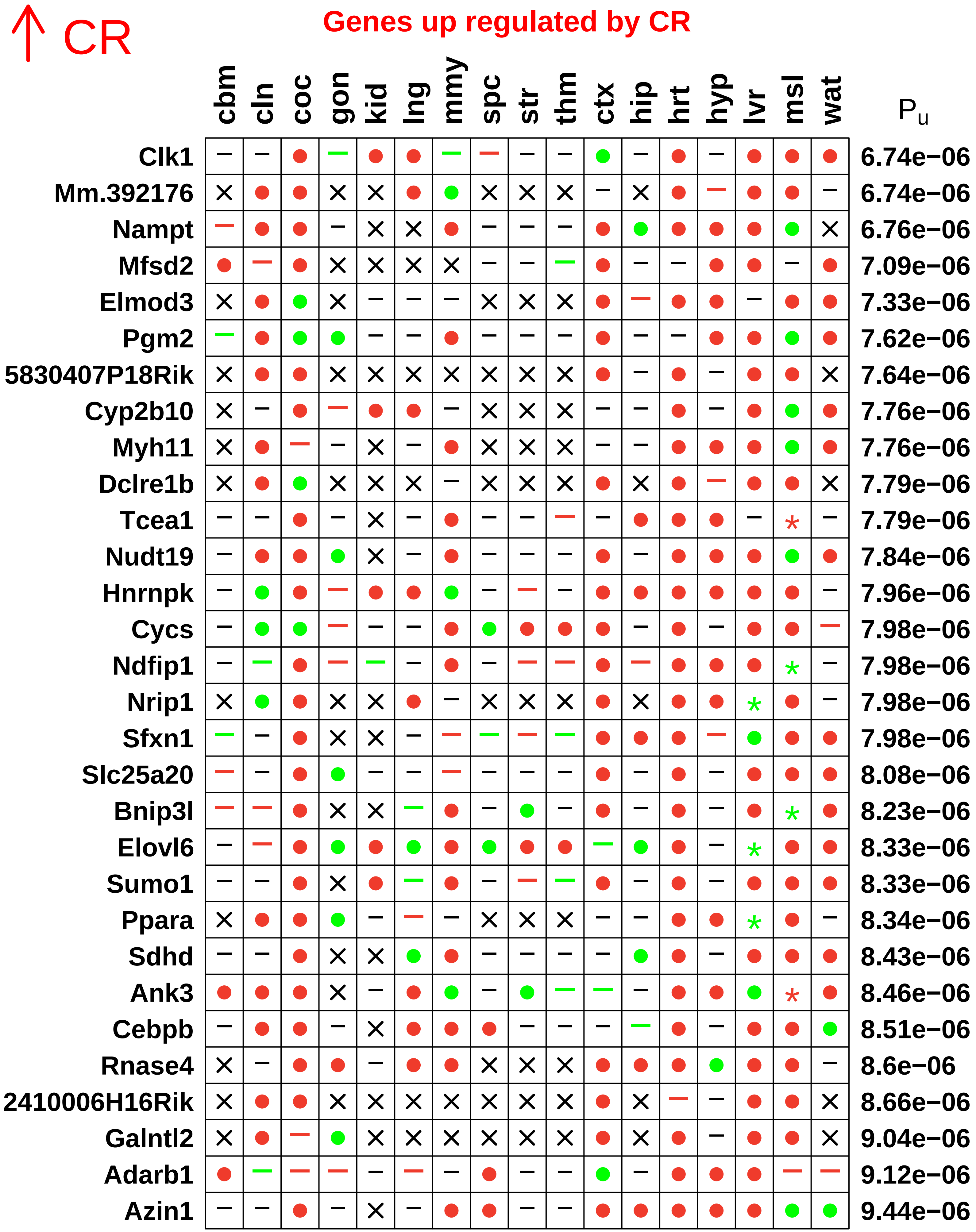
↑ CR
Genes up regulated by CR
cbm
cln
coc
gon
kid
lng
mmy
spc
str
thm
ctx
hip
hrt
hyp
lvr
msl
wat
P_u
Clk1 6.74e−06
Mm.392176 6.74e−06
Nampt 6.76e−06
Mfsd2 7.09e−06
Elmod3 7.33e−06
Pgm2 7.62e−06
5830407P18Rik 7.64e−06
Cyp2b10 7.76e−06
Myh11 7.76e−06
Dclre1b 7.79e−06
Tcea1 7.79e−06
Nudt19 7.84e−06
Hnrnpk 7.96e−06
Cycs 7.98e−06
Ndfip1 7.98e−06
Nrip1 7.98e−06
Sfxn1 7.98e−06
Slc25a20 8.08e−06
Bnip3l 8.23e−06
Elovl6 8.33e−06
Sumo1 8.33e−06
Ppara 8.34e−06
Sdhd 8.43e−06
Ank3 8.46e−06
Cebpb 8.51e−06
Rnase4 8.6e−06
2410006H16Rik 8.66e−06
Galntl2 9.04e−06
Adarb1 9.12e−06
Azin1 9.44e−06

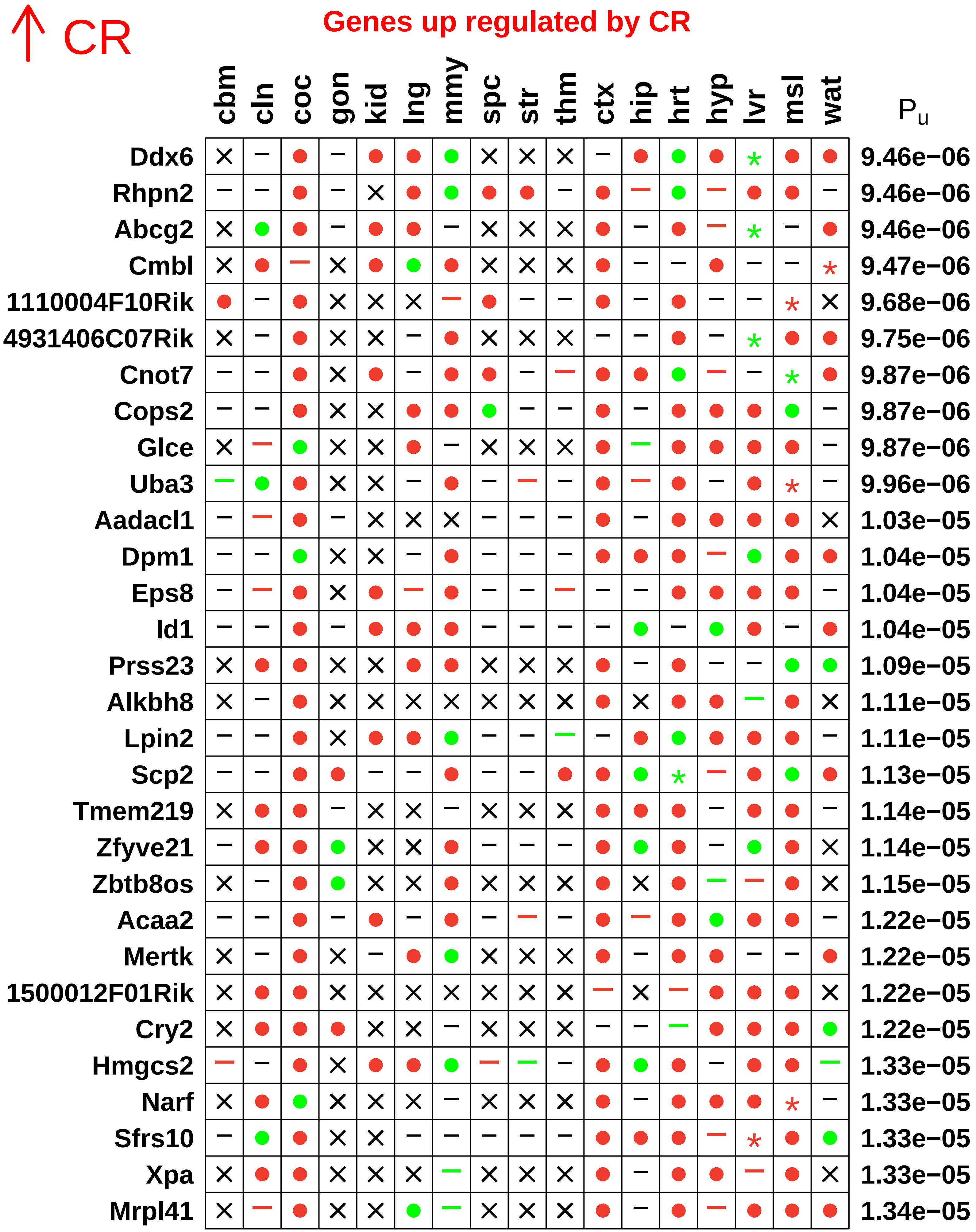
↑ CR
Genes up regulated by CR
cbm
cln
coc
gon
kid
lng
mmy
spc
str
thm
ctx
hip
hrt
hyp
lvr
msl
wat
P_u
Ddx6 9.46e−06
Rhpn2 9.46e−06
Abcg2 9.46e−06
Cmbl 9.47e−06
1110004F10Rik 9.68e−06
4931406C07Rik 9.75e−06
Cnot7 9.87e−06
Cops2 9.87e−06
Glce 9.87e−06
Uba3 9.96e−06
Aadacl1 1.03e−05
Dpm1 1.04e−05
Eps8 1.04e−05
Id1 1.04e−05
Prss23 1.09e−05
Alkbh8 1.11e−05
Lpin2 1.11e−05
Scp2 1.13e−05
Tmem219 1.14e−05
Zfyve21 1.14e−05
Zbtb8os 1.15e−05
Acaa2 1.22e−05
Mertk 1.22e−05
1500012F01Rik 1.22e−05
Cry2 1.22e−05
Hmgcs2 1.33e−05
Narf 1.33e−05
Sfrs10 1.33e−05
Xpa 1.33e−05
Mrpl41 1.34e−05

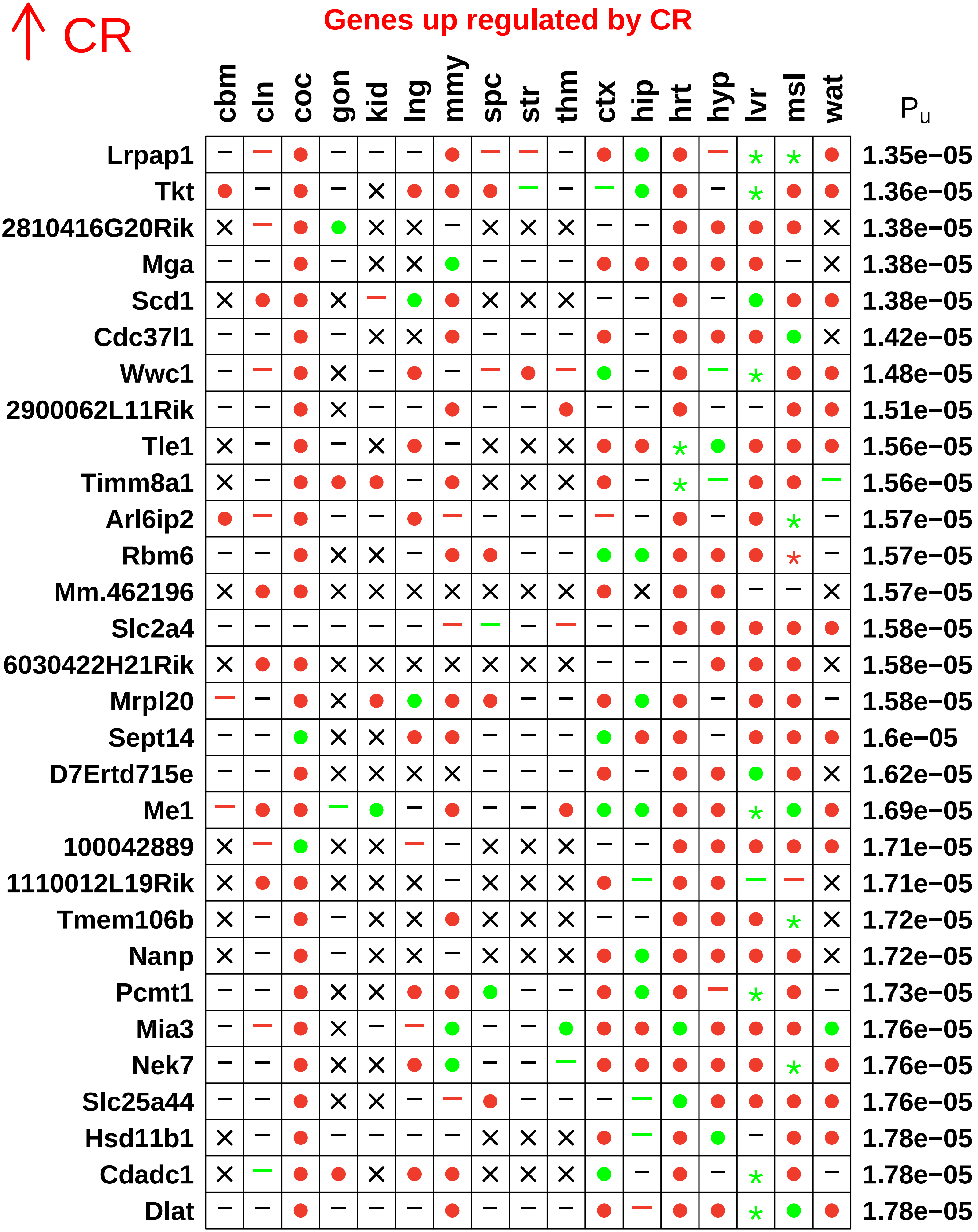
↑ CR
Genes up regulated by CR
cbm cln coc gon kid lng mmy spc str thm ctx hip hrt hyp lvr msl wat
Pu
Lrpap1 1.35e−05
Tkt 1.36e−05
2810416G20Rik 1.38e−05
Mga 1.38e−05
Scd1 1.38e−05
Cdc37l1 1.42e−05
Wwc1 1.48e−05
2900062L11Rik 1.51e−05
Tle1 1.56e−05
Timm8a1 1.56e−05
Arl6ip2 1.57e−05
Rbm6 1.57e−05
Mm.462196 1.57e−05
Slc2a4 1.58e−05
6030422H21Rik 1.58e−05
Mrpl20 1.58e−05
Sept14 1.6e−05
D7Ertd715e 1.62e−05
Me1 1.69e−05
100042889 1.71e−05
1110012L19Rik 1.71e−05
Tmem106b 1.72e−05
Nanp 1.72e−05
Pcmt1 1.73e−05
Mia3 1.76e−05
Nek7 1.76e−05
Slc25a44 1.76e−05
Hsd11b1 1.78e−05
Cdadc1 1.78e−05
Dlat 1.78e−05

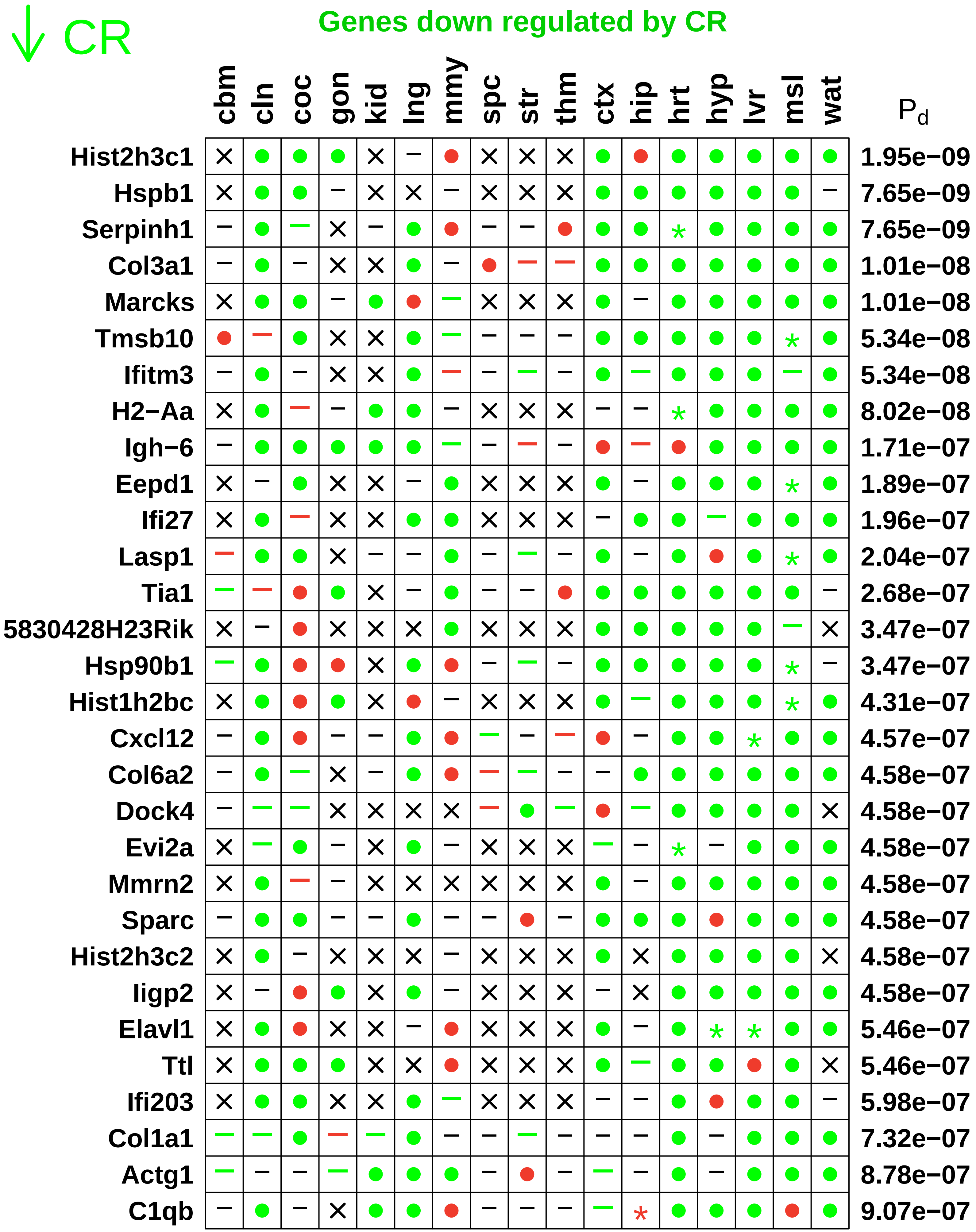

↓ CR
Genes down regulated by CR
cbm
cln
coc
gon
kid
lng
mmy
spc
str
thm
ctx
hip
hrt
hyp
lvr
msl
wat
P_d
Hist2h3c1 1.95e−09
Hspb1 7.65e−09
Serpinh1 7.65e−09
Col3a1 1.01e−08
Marcks 1.01e−08
Tmsb10 5.34e−08
Ifitm3 5.34e−08
H2−Aa 8.02e−08
Igh−6 1.71e−07
Eepd1 1.89e−07
Ifi27 1.96e−07
Lasp1 2.04e−07
Tia1 2.68e−07
5830428H23Rik 3.47e−07
Hsp90b1 3.47e−07
Hist1h2bc 4.31e−07
Cxcl12 4.57e−07
Col6a2 4.58e−07
Dock4 4.58e−07
Evi2a 4.58e−07
Mmrn2 4.58e−07
Sparc 4.58e−07
Hist2h3c2 4.58e−07
Iigp2 4.58e−07
Elavl1 5.46e−07
Ttl 5.46e−07
Ifi203 5.98e−07
Col1a1 7.32e−07
Actg1 8.78e−07
C1qb 9.07e−07

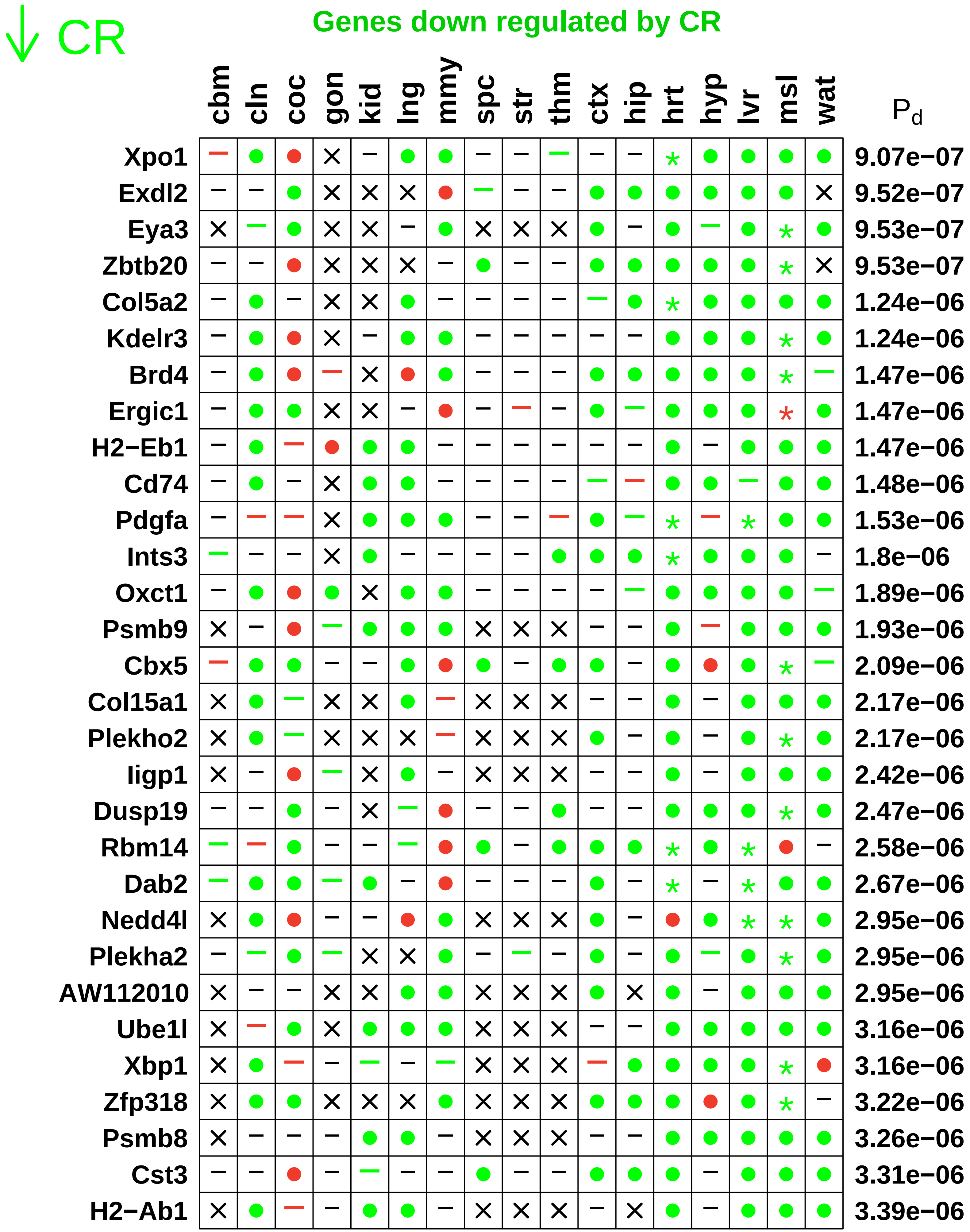
↓ CR
Genes down regulated by CR
cbm
cln
coc
gon
kid
lng
mmy
spc
str
thm
ctx
hip
hrt
hyp
lvr
msl
wat
Pd
Xpo1 9.07e−07
Exdl2 9.52e−07
Eya3 9.53e−07
Zbtb20 9.53e−07
Col5a2 1.24e−06
Kdelr3 1.24e−06
Brd4 1.47e−06
Ergic1 1.47e−06
H2−Eb1 1.47e−06
Cd74 1.48e−06
Pdgfa 1.53e−06
Ints3 1.8e−06
Oxct1 1.89e−06
Psmb9 1.93e−06
Cbx5 2.09e−06
Col15a1 2.17e−06
Plekho2 2.17e−06
Iigp1 2.42e−06
Dusp19 2.47e−06
Rbm14 2.58e−06
Dab2 2.67e−06
Nedd4l 2.95e−06
Plekha2 2.95e−06
AW112010 2.95e−06
Ube1l 3.16e−06
Xbp1 3.16e−06
Zfp318 3.22e−06
Psmb8 3.26e−06
Cst3 3.31e−06
H2−Ab1 3.39e−06

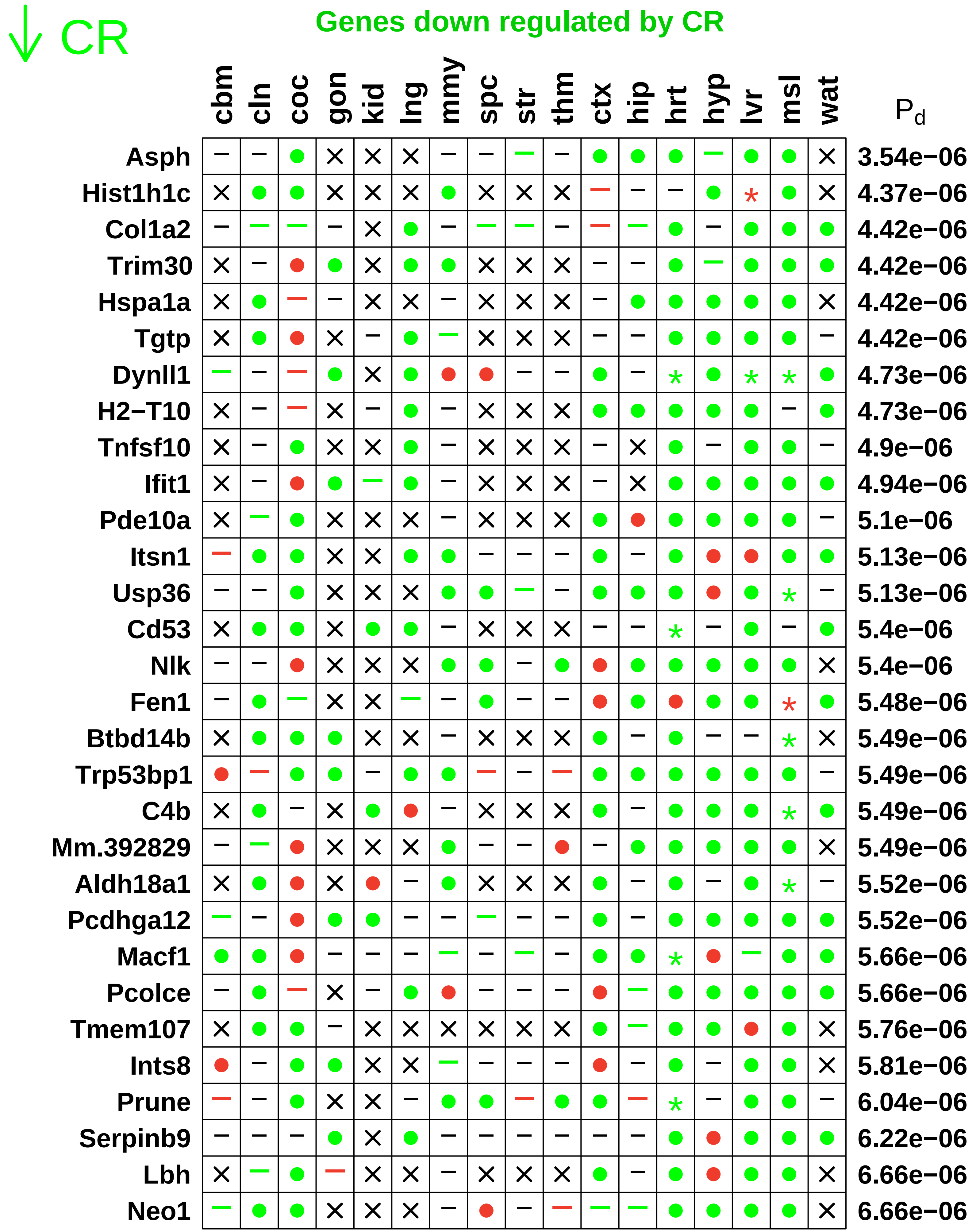
↓ CR
Genes down regulated by CR
cbm
cln
coc
gon
kid
lng
mmy
spc
str
thm
ctx
hip
hrt
hyp
lvr
msl
wat
P_d
Asph 3.54e−06
Hist1h1c 4.37e−06
Col1a2 4.42e−06
Trim30 4.42e−06
Hspa1a 4.42e−06
Tgtp 4.42e−06
Dynll1 4.73e−06
H2−T10 4.73e−06
Tnfsf10 4.9e−06
Ifit1 4.94e−06
Pde10a 5.1e−06
Itsn1 5.13e−06
Usp36 5.13e−06
Cd53 5.4e−06
Nlk 5.4e−06
Fen1 5.48e−06
Btbd14b 5.49e−06
Trp53bp1 5.49e−06
C4b 5.49e−06
Mm.392829 5.49e−06
Aldh18a1 5.52e−06
Pcdhga12 5.52e−06
Macf1 5.66e−06
Pcolce 5.66e−06
Tmem107 5.76e−06
Ints8 5.81e−06
Prune 6.04e−06
Serpinb9 6.22e−06
Lbh 6.66e−06
Neo1 6.66e−06

↓ CR

Genes down regulated by CR

	cbm	cln	coc	gon	kid	lng	mmy	spc	str	thm	ctx	hip	hrt	hyp	lvr	msl	wat	P_d
Ly86	×	–g	–	–	●g	●g	–	×	×	×	●g	●r	●g	●g	●g	–	●g	6.74e−06
5730470L24Rik	×	●g	●r	×	×	×	●r	×	×	×	●g	●g	●g	●g	●g	●g	×	6.81e−06
4933428G20Rik	×	–	●g	●r	×	×	–	×	×	×	●g	–	●g	–	●g	–	●g	7.33e−06
Mdn1	–	–g	●g	×	×	–	●g	●g	–	–	●g	●g	●r	●g	–	●g	●r	7.33e−06
Pmepa1	×	–g	●r	×	×	×	●g	×	×	×	●g	–	●g	●r	●g	●g	×	7.33e−06
1810063B05Rik	×	–r	●r	×	×	●g	–g	×	×	×	●g	●g	●g	–g	●r	●g	–	7.42e−06
Sirpa	–	–	–	–g	×	●g	–	–	–g	–r	●g	●g	●g	–	–g	●g	●g	7.53e−06
Hfe2	×	●g	●g	×	×	×	×	×	×	×	–	–	●g	–g	●g	●g	×	7.67e−06
Tcf4	–	–	●r	×	×	–	●g	–	–r	–	●r	–	●g	●g	*g	*g	●g	7.96e−06
Lima1	–	–r	●r	×	×	●g	●g	–r	–g	–	–	●g	●g	●g	●g	*g	●r	8.19e−06
Speg	–	–g	●g	×	×	–	–g	–	●g	–g	●g	–	*g	●g	●g	*g	●r	8.54e−06
Tmcc1	–r	–g	●g	×	×	×	×	–	–	–	●r	–g	●g	●g	●g	*g	●g	8.54e−06
P4ha1	●g	–g	●g	×	–	–	●r	–	–	–	–	●g	*g	●g	●r	*g	●g	8.74e−06
Hexa	–	–	–	●g	×	●g	●r	–	–r	–r	●g	–	●g	●g	●g	●g	●g	8.74e−06
Nrp1	–	–	●r	●r	–	●g	–	–	–g	●r	●r	–g	●g	●g	●g	●g	●g	9.18e−06
Commd7	–	–	●r	●g	×	●g	–	–	–g	–	–	●g	●g	–	●g	●g	●g	9.19e−06
Numa1	–	–	●g	–	×	–r	●g	●g	●r	–	●g	●g	*g	●g	*r	●g	–r	9.42e−06
Plekho1	×	–	–	×	×	●g	–	×	×	×	●g	–	●g	●g	●g	*g	–	9.42e−06
Col4a1	–	●g	–	–	●g	●r	–g	●r	–	–	●g	–	●g	–	●g	●g	●g	9.75e−06
Iqsec2	×	●r	●g	×	×	×	×	×	×	×	●g	×	●g	●g	●r	*g	×	9.85e−06
Cdc42ep2	×	●r	–	×	×	×	×	×	×	×	●g	×	–	●g	●g	●g	●g	9.85e−06
Gas7	×	–	●r	●g	●g	●g	–	×	×	×	●g	–	●r	●g	●g	*g	●g	9.99e−06
Trim25	×	–	●g	–	–	●r	●g	×	×	×	●r	–	●g	–g	●g	*g	●g	9.99e−06
Mett10d	×	–	●r	–	×	×	×	×	×	×	●g	●g	●g	–	●g	●g	×	9.99e−06
Ubfd1	–g	●g	●g	●r	×	●g	–	–	–r	–	●g	–r	●g	–g	*g	*g	●g	1.01e−05
Dmtf1	–	●r	●r	●g	×	×	●g	●r	–	–	●g	–	●g	–g	●g	●g	×	1.02e−05
Heatr6	×	●r	●g	×	×	×	–g	×	×	×	●r	–	●g	●g	●g	*g	×	1.02e−05
Opa3	×	–g	●g	×	×	×	–	×	×	×	–	–	●g	●g	●g	●g	×	1.03e−05
Col6a3	×	●g	–	–	–	●g	●r	×	×	×	●g	–	●g	–	●g	●g	●g	1.21e−05
Ctsc	●r	–	●g	–	●g	–	●r	●r	●g	–	–	–g	●g	●g	*g	●g	●g	1.21e−05

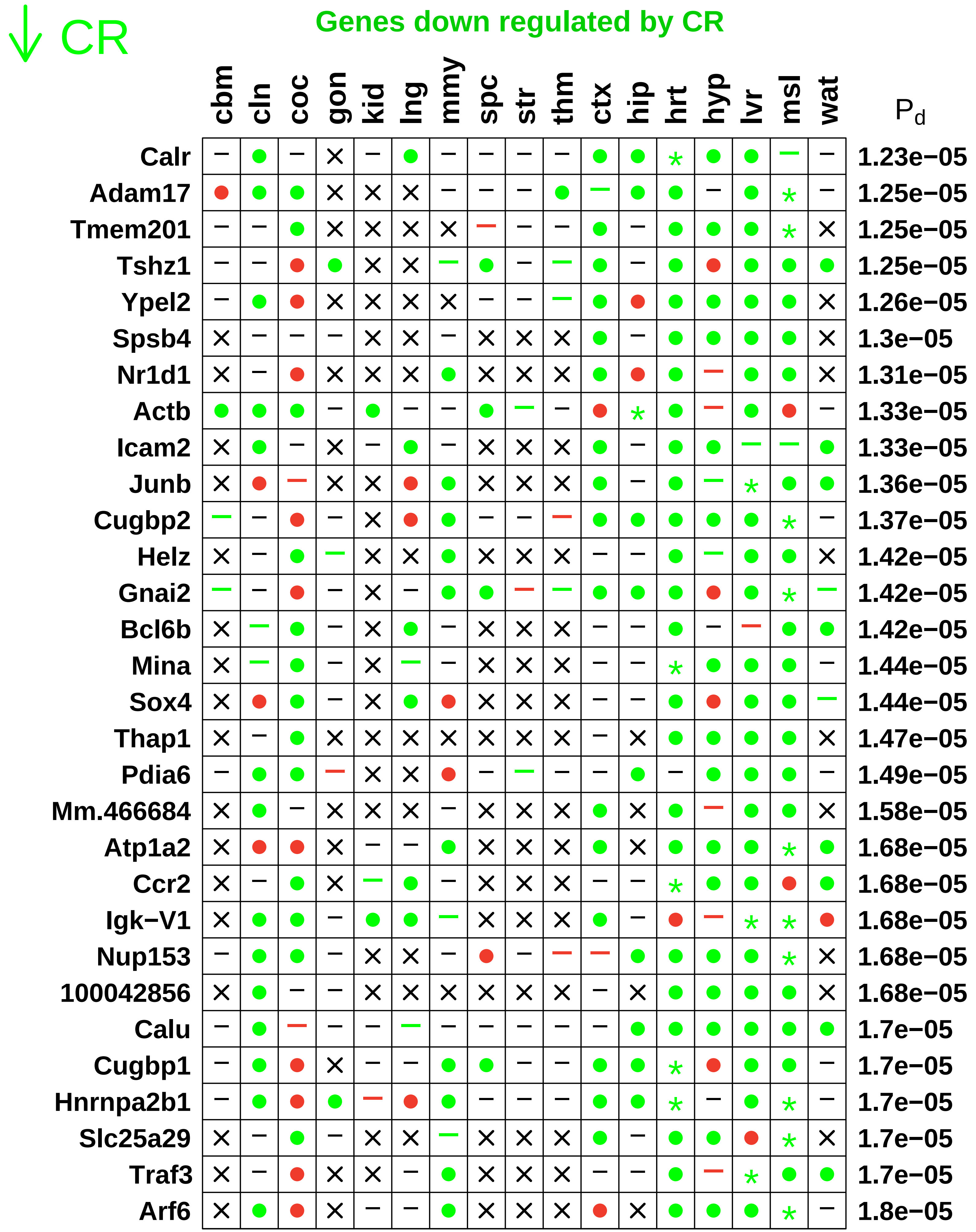
↓ CR
Genes down regulated by CR
cbm cln coc gon kid lng mmy spc str thm ctx hip hrt hyp lvr msl wat
Pd
Calr 1.23e−05
Adam17 1.25e−05
Tmem201 1.25e−05
Tshz1 1.25e−05
Ypel2 1.26e−05
Spsb4 1.3e−05
Nr1d1 1.31e−05
Actb 1.33e−05
Icam2 1.33e−05
Junb 1.36e−05
Cugbp2 1.37e−05
Helz 1.42e−05
Gnai2 1.42e−05
Bcl6b 1.42e−05
Mina 1.44e−05
Sox4 1.44e−05
Thap1 1.47e−05
Pdia6 1.49e−05
Mm.466684 1.58e−05
Atp1a2 1.68e−05
Ccr2 1.68e−05
Igk−V1 1.68e−05
Nup153 1.68e−05
100042856 1.68e−05
Calu 1.7e−05
Cugbp1 1.7e−05
Hnrnpa2b1 1.7e−05
Slc25a29 1.7e−05
Traf3 1.7e−05
Arf6 1.8e−05

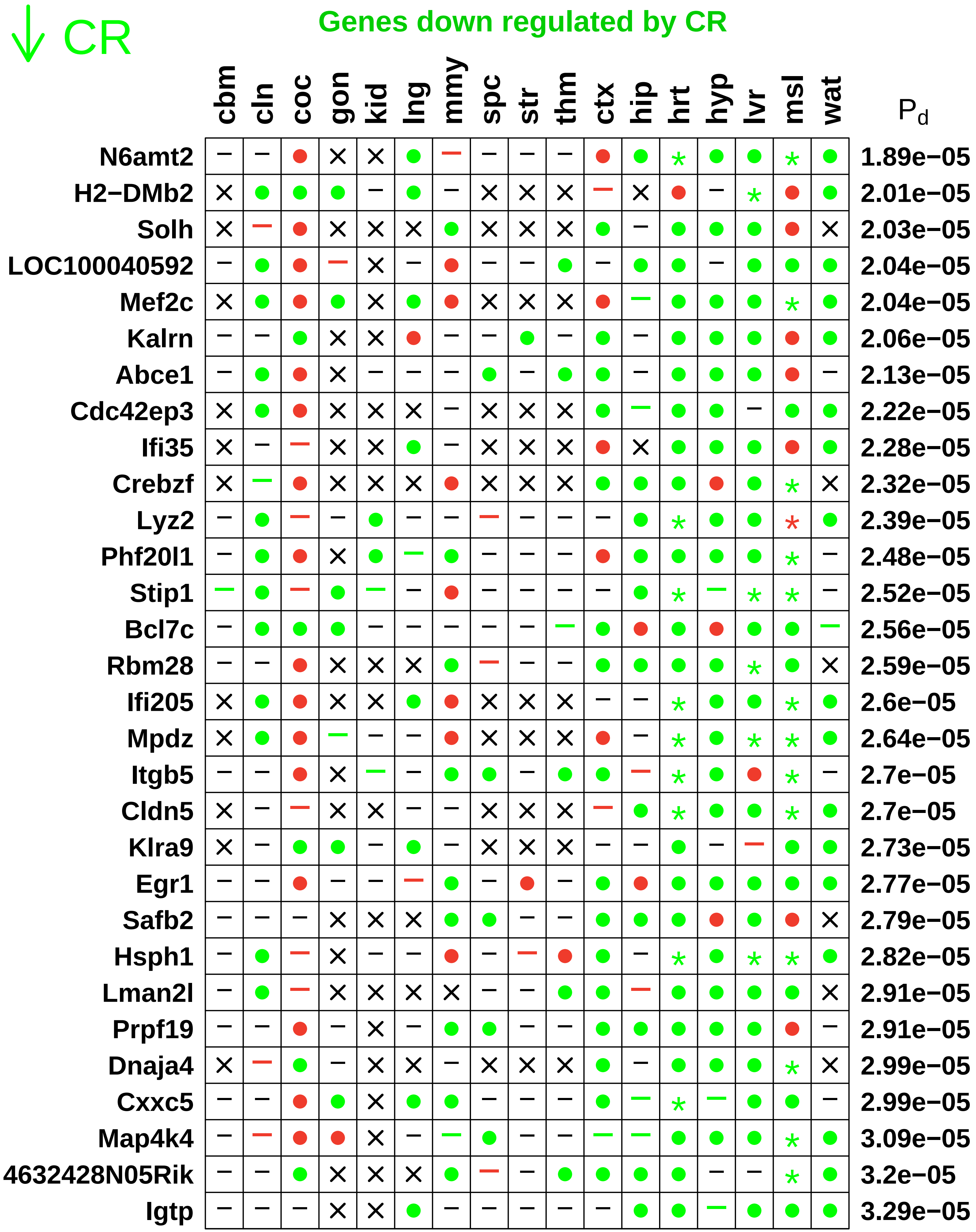

↓ CR
Genes down regulated by CR
cbm
cln
coc
gon
kid
lng
mmy
spc
str
thm
ctx
hip
hrt
hyp
lvr
msl
wat
Pd
N6amt2 1.89e−05
H2−DMb2 2.01e−05
Solh 2.03e−05
LOC100040592 2.04e−05
Mef2c 2.04e−05
Kalrn 2.06e−05
Abce1 2.13e−05
Cdc42ep3 2.22e−05
Ifi35 2.28e−05
Crebzf 2.32e−05
Lyz2 2.39e−05
Phf20l1 2.48e−05
Stip1 2.52e−05
Bcl7c 2.56e−05
Rbm28 2.59e−05
Ifi205 2.6e−05
Mpdz 2.64e−05
Itgb5 2.7e−05
Cldn5 2.7e−05
Klra9 2.73e−05
Egr1 2.77e−05
Safb2 2.79e−05
Hsph1 2.82e−05
Lman2l 2.91e−05
Prpf19 2.91e−05
Dnaja4 2.99e−05
Cxxc5 2.99e−05
Map4k4 3.09e−05
4632428N05Rik 3.2e−05
Igtp 3.29e−05

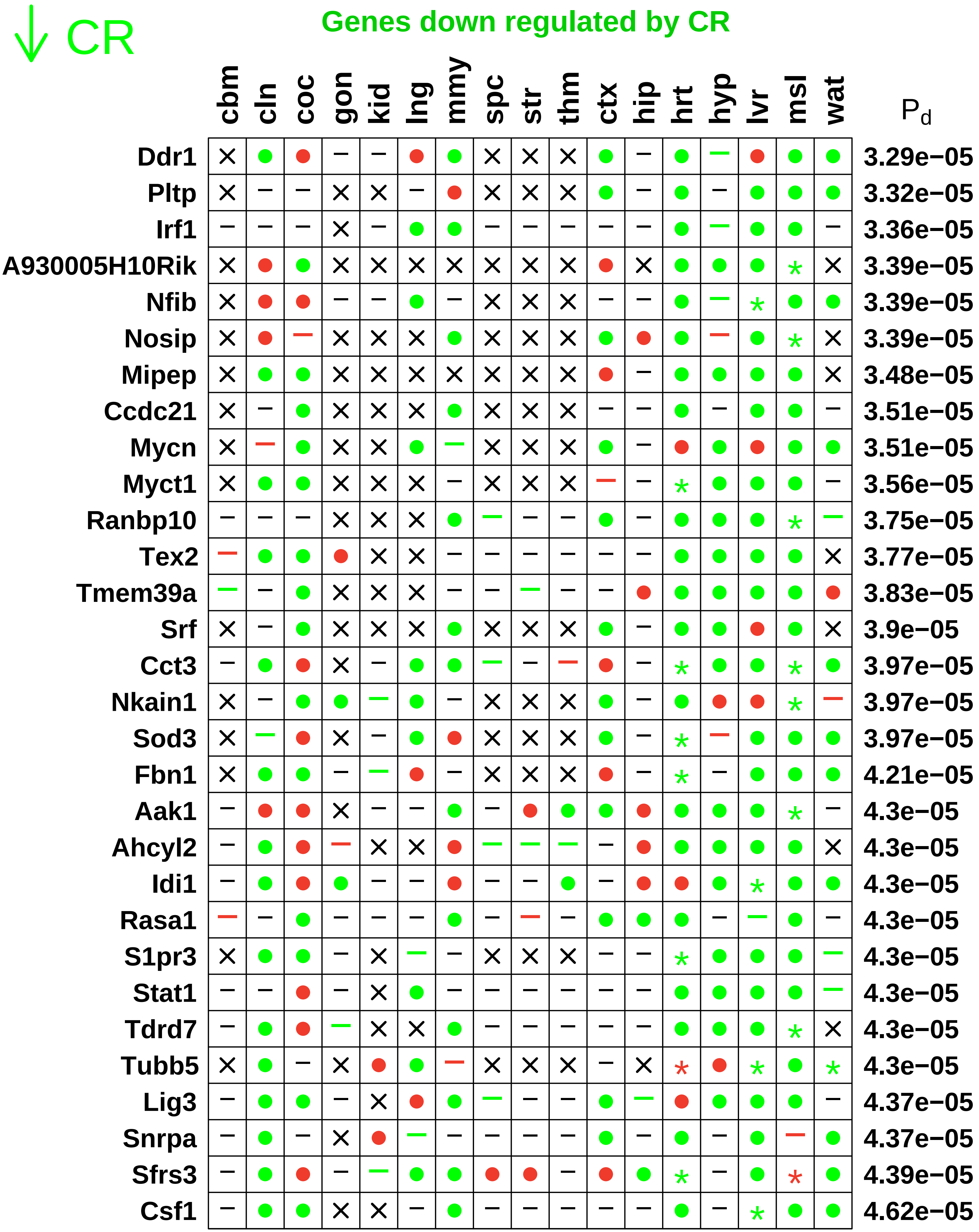
↓ CR
Genes down regulated by CR
cbm
cln
coc
gon
kid
lng
mmy
spc
str
thm
ctx
hip
hrt
hyp
lvr
msl
wat
P_d
Ddr1 3.29e−05
Pltp 3.32e−05
Irf1 3.36e−05
A930005H10Rik 3.39e−05
Nfib 3.39e−05
Nosip 3.39e−05
Mipep 3.48e−05
Ccdc21 3.51e−05
Mycn 3.51e−05
Myct1 3.56e−05
Ranbp10 3.75e−05
Tex2 3.77e−05
Tmem39a 3.83e−05
Srf 3.9e−05
Cct3 3.97e−05
Nkain1 3.97e−05
Sod3 3.97e−05
Fbn1 4.21e−05
Aak1 4.3e−05
Ahcyl2 4.3e−05
Idi1 4.3e−05
Rasa1 4.3e−05
S1pr3 4.3e−05
Stat1 4.3e−05
Tdrd7 4.3e−05
Tubb5 4.3e−05
Lig3 4.37e−05
Snrpa 4.37e−05
Sfrs3 4.39e−05
Csf1 4.62e−05

↓ CR

Genes down regulated by CR

	cbm	cln	coc	gon	kid	lng	mmy	spc	str	thm	ctx	hip	hrt	hyp	lvr	msl	wat	P_d
Rangap1	−	● green	−	×	×	−	−	−	−	−	− green	● green	* green	● red	● green	● green	● green	4.62e−05
Smr2	×	− green	● green	×	×	−	−	×	×	×	● red	−	* green	● green	● green	− green	● green	4.64e−05
Kdr	×	−	● green	×	● green	● green	−	×	×	×	−	−	● green	● green	− green	● green	−	4.64e−05
Rnf11	−	● green	● red	×	×	−	● red	−	− green	− red	● green	● green	● green	● red	● green	● green	− green	4.68e−05
Dalrd3	×	● green	● red	×	×	● red	−	×	×	×	● green	−	● green	● green	● green	● green	● red	4.79e−05
Pign	×	● green	● green	×	×	−	−	×	×	×	− red	−	● green	−	● green	● green	● green	4.84e−05
Ccnd1	×	−	● red	− red	−	● green	● green	×	×	×	● green	● green	● green	● green	● green	● red	−	4.9e−05
Pex6	−	− green	● red	− red	− red	−	● green	− red	−	−	● green	−	● green	● green	* green	* green	−	4.9e−05
I7Rn6	−	● green	● red	×	● green	● green	● red	● green	−	− red	−	● green	● red	● green	● green	● green	● green	4.93e−05
Ctss	×	−	−	● green	● green	−	−	×	×	×	−	● red	* green	−	− green	* green	● green	4.95e−05
Gats	×	● green	● green	×	×	×	− green	×	×	×	− green	−	● green	● red	● green	* green	×	4.97e−05
2610528G24Rik	−	● green	− red	×	−	● green	−	● green	−	−	−	− red	● green	− green	● green	● green	● green	5.15e−05
Sec16a	−	−	− red	×	×	● red	● green	−	− green	−	● green	−	● green	● green	● green	● green	−	5.17e−05
Sdf2l1	×	● green	−	×	×	×	−	×	×	×	−	● green	● green	● green	● green	−	● red	5.17e−05
Smyd1	−	−	● green	● green	×	● green	− red	− green	−	● red	− red	● red	* green	− red	● green	* green	● green	5.28e−05
Fcho2	−	−	● red	● green	×	×	×	−	−	−	● green	− green	● green	−	● green	● green	− green	5.38e−05
Mdfic	×	−	− red	−	− green	−	−	×	×	×	● red	−	* green	● green	● green	● green	● green	5.44e−05
Orc4l	● green	− red	● green	×	×	−	● green	− red	−	−	● red	● green	● green	● red	* green	* green	− green	5.45e−05
Apobec1	−	−	● green	×	−	−	● green	● green	−	−	−	−	* green	−	● green	−	● green	5.45e−05
Ifit3	×	−	● red	− green	● green	● green	−	×	×	×	− red	− green	● green	−	● green	−	● green	5.45e−05
Tln2	×	● green	● green	● red	×	×	×	×	×	×	● green	− green	● green	● red	● green	● green	×	5.47e−05
Dpp9	×	−	−	−	×	×	×	×	×	×	● green	● green	● green	−	● green	● green	×	5.64e−05
Arid1a	−	−	● red	● green	− green	● red	● green	● green	−	● red	● green	● red	* green	● red	* green	* green	−	5.65e−05
Dcx	×	● green	● green	×	×	−	−	×	×	×	● green	−	● red	● green	● green	−	−	5.65e−05
Jarid2	−	−	● red	● red	● red	− green	● green	● green	● green	● red	− green	−	● green	−	● green	* green	−	5.65e−05
Tardbp	−	−	● red	×	×	● red	● green	−	−	● green	● green	● green	● red	● green	* green	● red	● red	5.71e−05
Tmem150	×	− red	− green	×	×	● green	−	×	×	×	● green	−	● green	− red	* green	● green	● green	5.71e−05
A530088I07Rik	×	−	● green	● green	×	×	− green	×	×	×	● green	−	● red	● green	● green	− green	×	5.92e−05
Rras2	−	− green	● red	● green	×	×	● green	− green	−	−	● red	● green	● green	● green	−	● green	×	5.95e−05
Rrbp1	−	−	● red	×	−	−	−	● green	− red	−	● green	● green	● green	● red	● green	● green	● green	5.99e−05

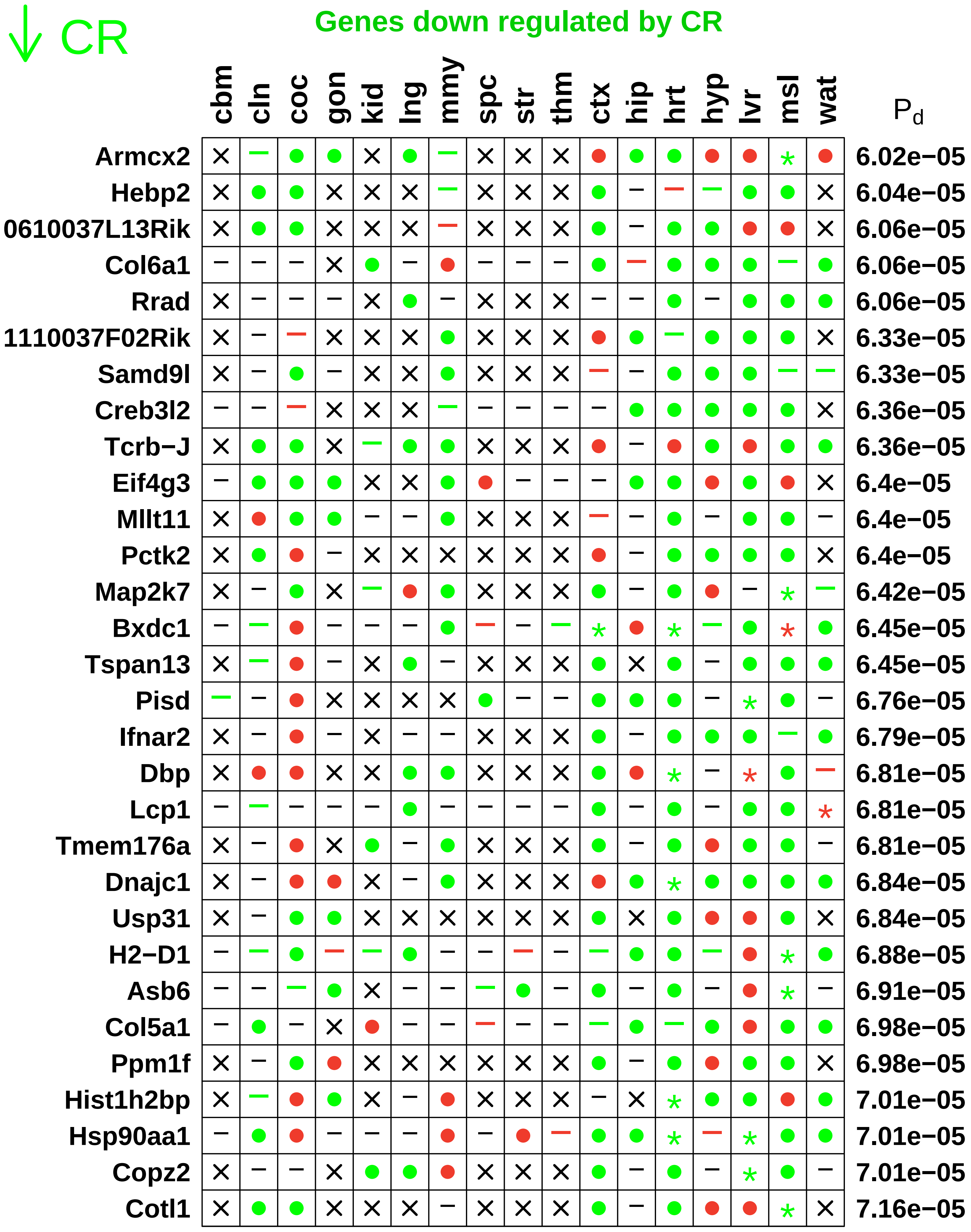
↓ CR
Genes down regulated by CR
cbm
cln
coc
gon
kid
lng
mmy
spc
str
thm
ctx
hip
hrt
hyp
lvr
msl
wat
P_d
Armcx2 6.02e−05
Hebp2 6.04e−05
0610037L13Rik 6.06e−05
Col6a1 6.06e−05
Rrad 6.06e−05
1110037F02Rik 6.33e−05
Samd9l 6.33e−05
Creb3l2 6.36e−05
Tcrb−J 6.36e−05
Eif4g3 6.4e−05
Mllt11 6.4e−05
Pctk2 6.4e−05
Map2k7 6.42e−05
Bxdc1 6.45e−05
Tspan13 6.45e−05
Pisd 6.76e−05
Ifnar2 6.79e−05
Dbp 6.81e−05
Lcp1 6.81e−05
Tmem176a 6.81e−05
Dnajc1 6.84e−05
Usp31 6.84e−05
H2−D1 6.88e−05
Asb6 6.91e−05
Col5a1 6.98e−05
Ppm1f 6.98e−05
Hist1h2bp 7.01e−05
Hsp90aa1 7.01e−05
Copz2 7.01e−05
Cotl1 7.16e−05

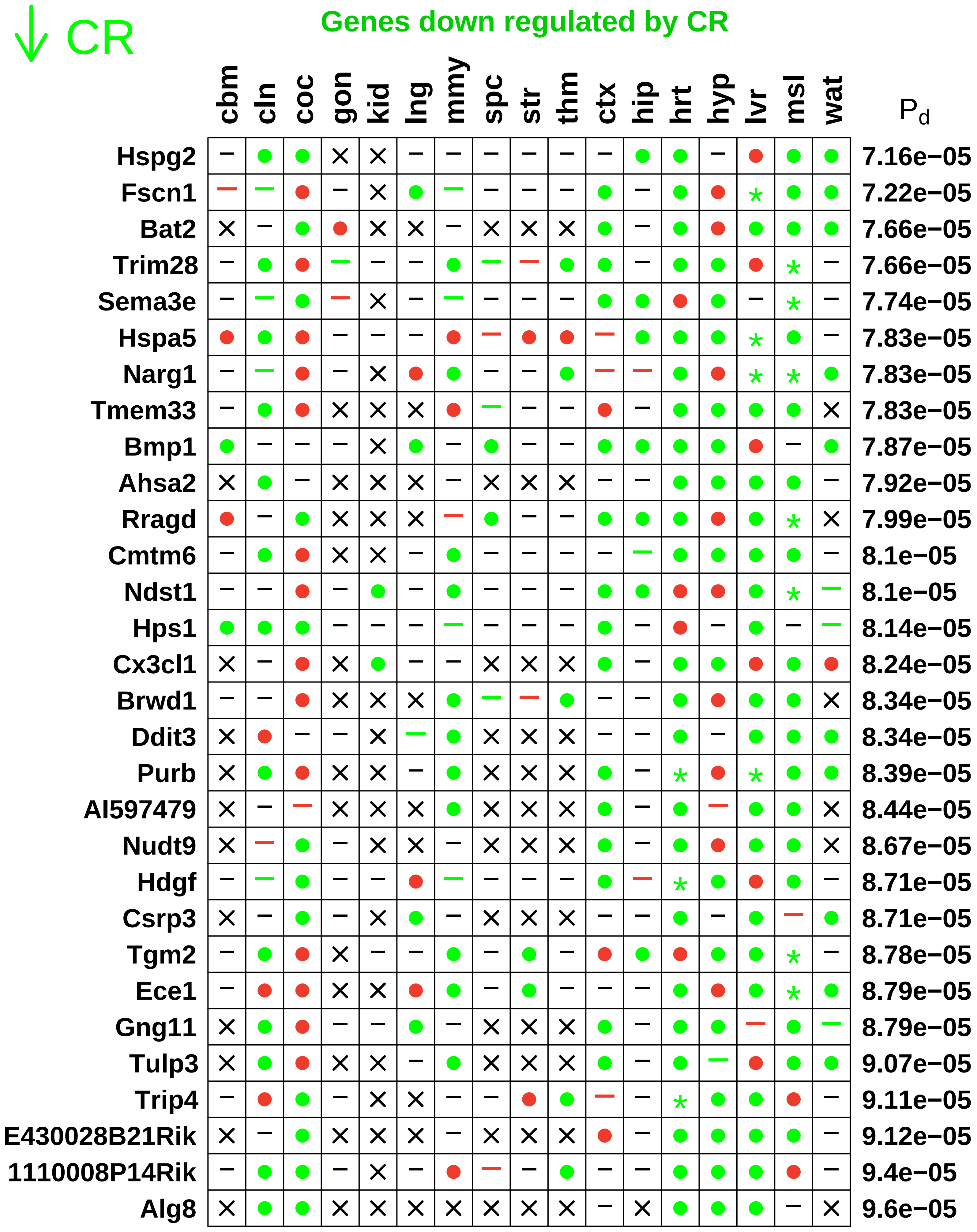

↓ CR
Genes down regulated by CR
cbm
cln
coc
gon
kid
lng
mmy
spc
str
thm
ctx
hip
hrt
hyp
lvr
msl
wat
P_d
Hspg2 7.16e−05
Fscn1 7.22e−05
Bat2 7.66e−05
Trim28 7.66e−05
Sema3e 7.74e−05
Hspa5 7.83e−05
Narg1 7.83e−05
Tmem33 7.83e−05
Bmp1 7.87e−05
Ahsa2 7.92e−05
Rragd 7.99e−05
Cmtm6 8.1e−05
Ndst1 8.1e−05
Hps1 8.14e−05
Cx3cl1 8.24e−05
Brwd1 8.34e−05
Ddit3 8.34e−05
Purb 8.39e−05
AI597479 8.44e−05
Nudt9 8.67e−05
Hdgf 8.71e−05
Csrp3 8.71e−05
Tgm2 8.78e−05
Ece1 8.79e−05
Gng11 8.79e−05
Tulp3 9.07e−05
Trip4 9.11e−05
E430028B21Rik 9.12e−05
1110008P14Rik 9.4e−05
Alg8 9.6e−05

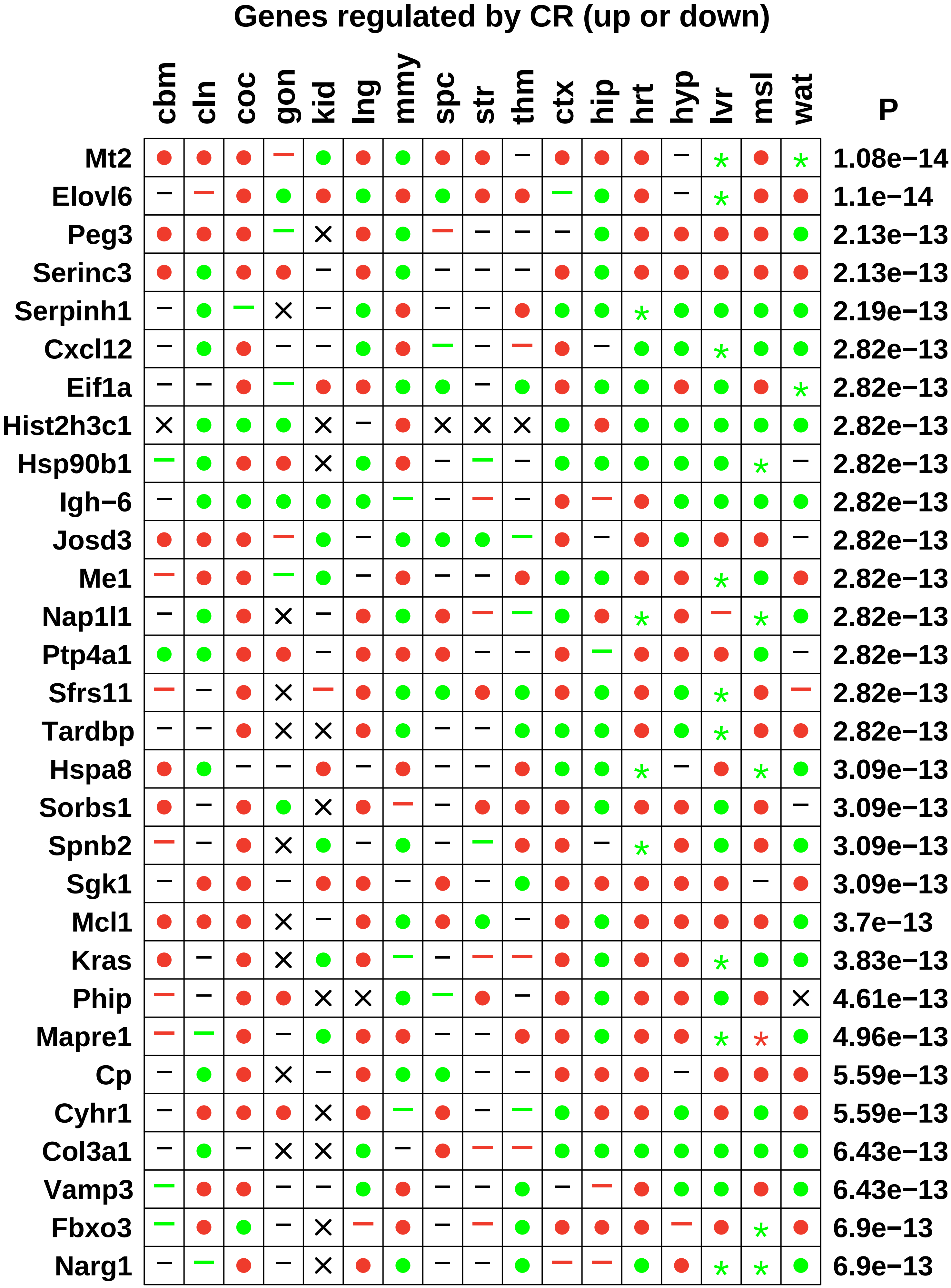
Genes regulated by CR (up or down)
cbm
cln
coc
gon
kid
lng
mmy
spc
str
thm
ctx
hip
hrt
hyp
lvr
msl
wat
P
Mt2
1.08e−14
Elovl6
1.1e−14
Peg3
2.13e−13
Serinc3
2.13e−13
Serpinh1
2.19e−13
Cxcl12
2.82e−13
Eif1a
2.82e−13
Hist2h3c1
2.82e−13
Hsp90b1
2.82e−13
Igh−6
2.82e−13
Josd3
2.82e−13
Me1
2.82e−13
Nap1l1
2.82e−13
Ptp4a1
2.82e−13
Sfrs11
2.82e−13
Tardbp
2.82e−13
Hspa8
3.09e−13
Sorbs1
3.09e−13
Spnb2
3.09e−13
Sgk1
3.09e−13
Mcl1
3.7e−13
Kras
3.83e−13
Phip
4.61e−13
Mapre1
4.96e−13
Cp
5.59e−13
Cyhr1
5.59e−13
Col3a1
6.43e−13
Vamp3
6.43e−13
Fbxo3
6.9e−13
Narg1
6.9e−13

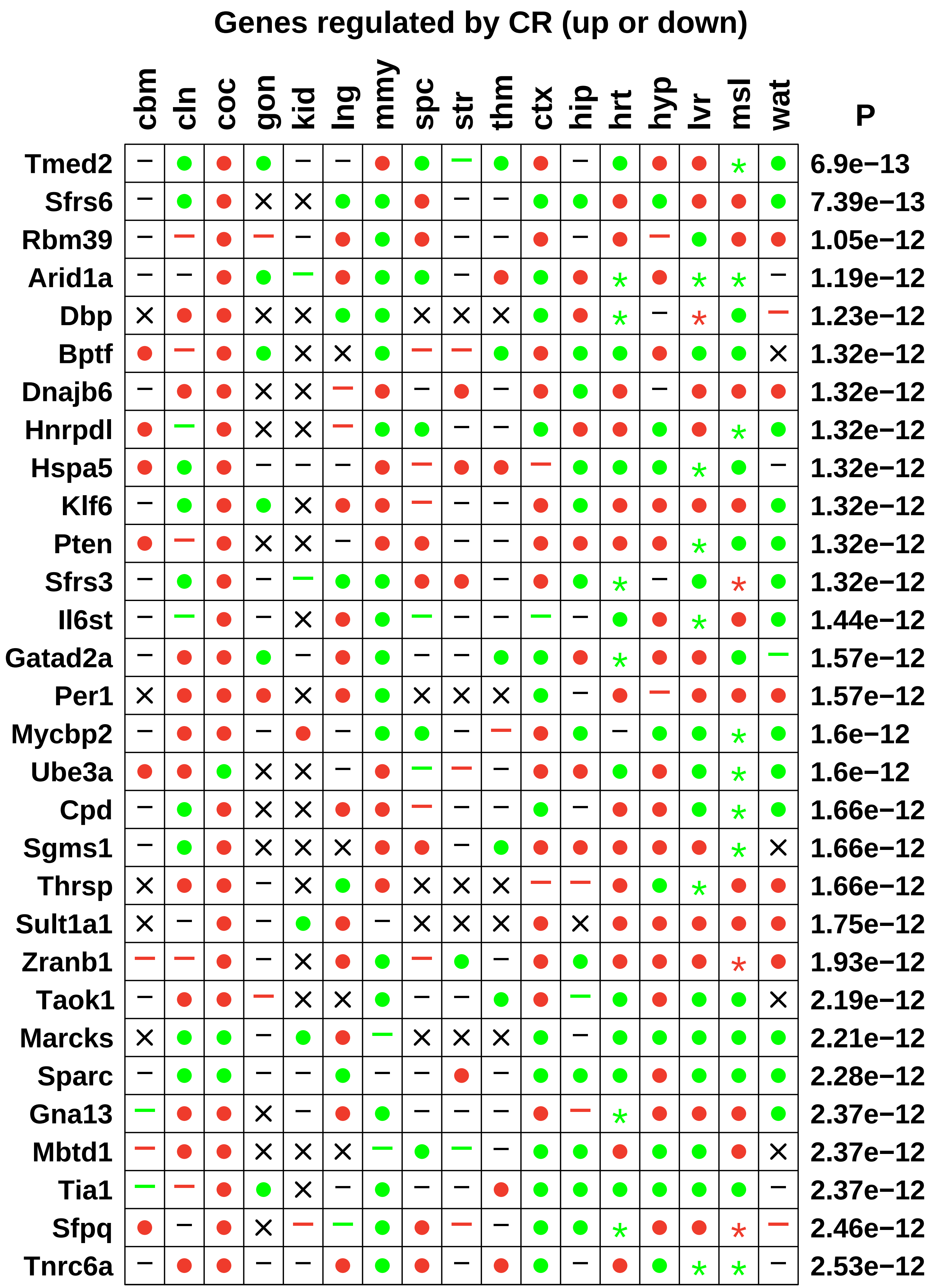
Genes regulated by CR (up or down)
cbm
cln
coc
gon
kid
lng
mmy
spc
str
thm
ctx
hip
hrt
hyp
lvr
msl
wat
P
Tmed2
Sfrs6
Rbm39
Arid1a
Dbp
Bptf
Dnajb6
Hnrpdl
Hspa5
Klf6
Pten
Sfrs3
Il6st
Gatad2a
Per1
Mycbp2
Ube3a
Cpd
Sgms1
Thrsp
Sult1a1
Zranb1
Taok1
Marcks
Sparc
Gna13
Mbtd1
Tia1
Sfpq
Tnrc6a
6.9e−13
7.39e−13
1.05e−12
1.19e−12
1.23e−12
1.32e−12
1.32e−12
1.32e−12
1.32e−12
1.32e−12
1.32e−12
1.32e−12
1.44e−12
1.57e−12
1.57e−12
1.6e−12
1.6e−12
1.66e−12
1.66e−12
1.66e−12
1.75e−12
1.93e−12
2.19e−12
2.21e−12
2.28e−12
2.37e−12
2.37e−12
2.37e−12
2.46e−12
2.53e−12

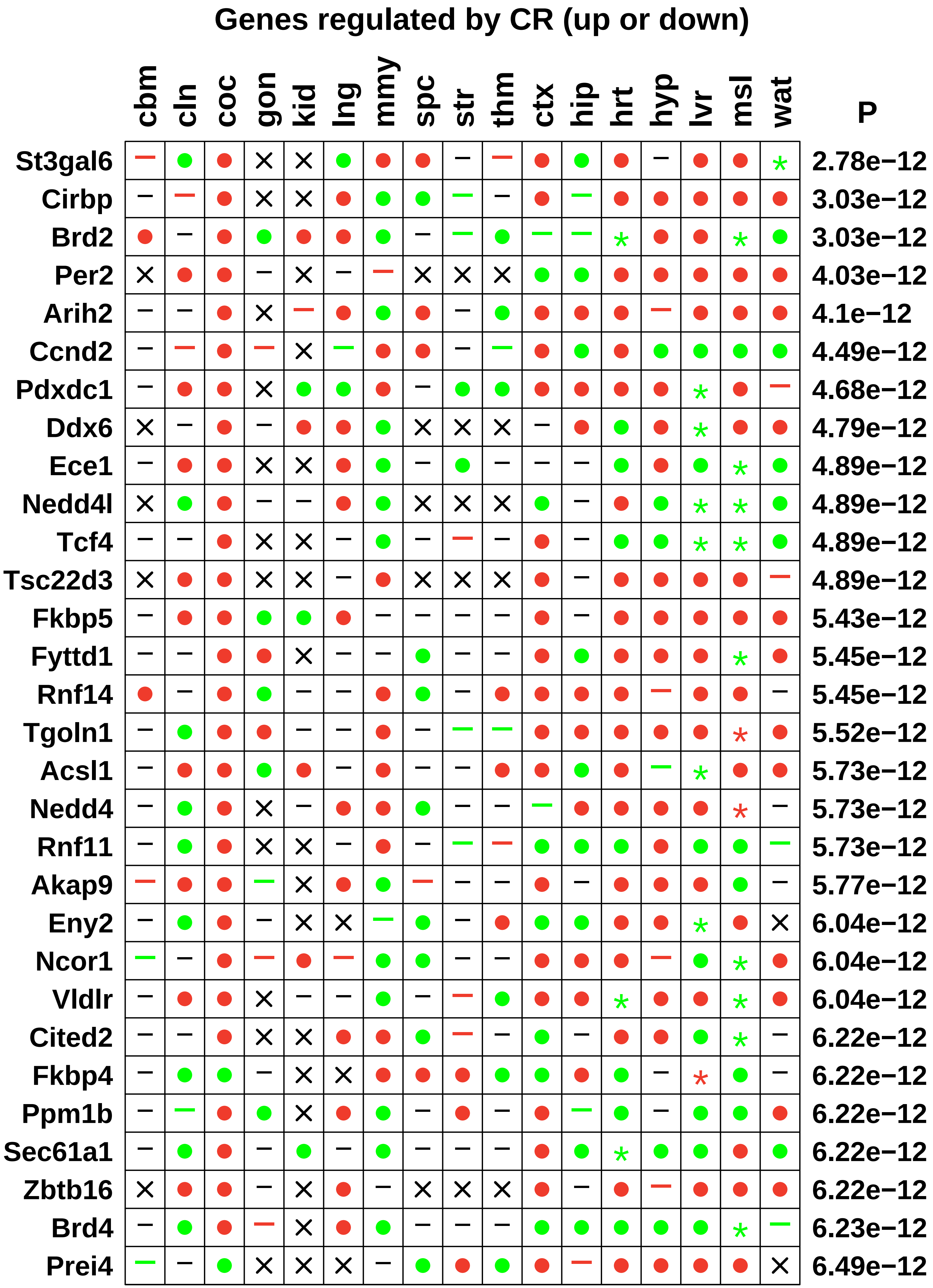
Genes regulated by CR (up or down)
cbm
cln
coc
gon
kid
lng
mmy
spc
str
thm
ctx
hip
hrt
hyp
lvr
msl
wat
P
St3gal6 2.78e−12
Cirbp 3.03e−12
Brd2 3.03e−12
Per2 4.03e−12
Arih2 4.1e−12
Ccnd2 4.49e−12
Pdxdc1 4.68e−12
Ddx6 4.79e−12
Ece1 4.89e−12
Nedd4l 4.89e−12
Tcf4 4.89e−12
Tsc22d3 4.89e−12
Fkbp5 5.43e−12
Fyttd1 5.45e−12
Rnf14 5.45e−12
Tgoln1 5.52e−12
Acsl1 5.73e−12
Nedd4 5.73e−12
Rnf11 5.73e−12
Akap9 5.77e−12
Eny2 6.04e−12
Ncor1 6.04e−12
Vldlr 6.04e−12
Cited2 6.22e−12
Fkbp4 6.22e−12
Ppm1b 6.22e−12
Sec61a1 6.22e−12
Zbtb16 6.22e−12
Brd4 6.23e−12
Prei4 6.49e−12

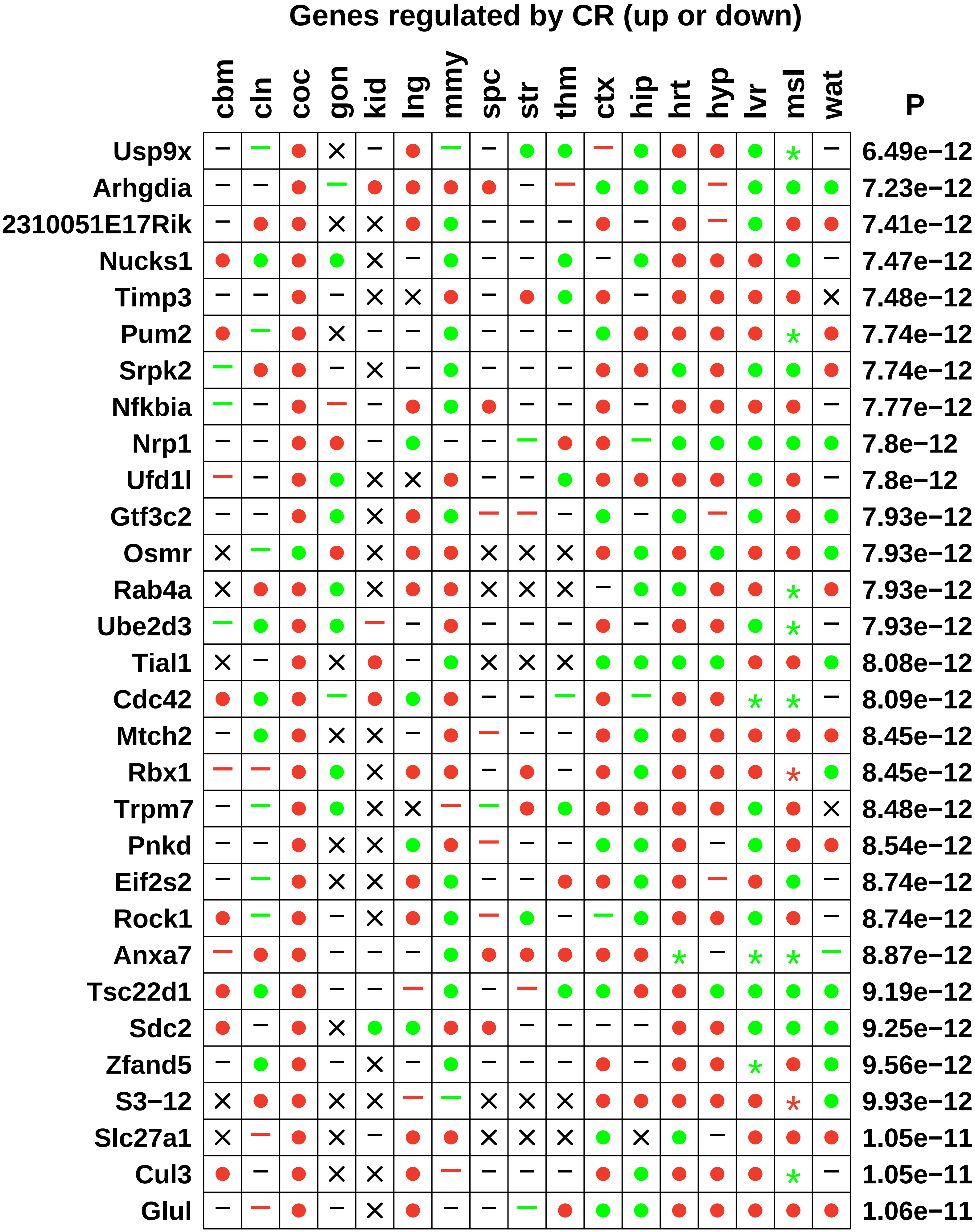

Genes regulated by CR (up or down)
cbm
cln
coc
gon
kid
lng
mmy
spc
str
thm
ctx
hip
hrt
hyp
lvr
msl
wat
P
Usp9x 6.49e−12
Arhgdia 7.23e−12
2310051E17Rik 7.41e−12
Nucks1 7.47e−12
Timp3 7.48e−12
Pum2 7.74e−12
Srpk2 7.74e−12
Nfkbia 7.77e−12
Nrp1 7.8e−12
Ufd1l 7.8e−12
Gtf3c2 7.93e−12
Osmr 7.93e−12
Rab4a 7.93e−12
Ube2d3 7.93e−12
Tial1 8.08e−12
Cdc42 8.09e−12
Mtch2 8.45e−12
Rbx1 8.45e−12
Trpm7 8.48e−12
Pnkd 8.54e−12
Eif2s2 8.74e−12
Rock1 8.74e−12
Anxa7 8.87e−12
Tsc22d1 9.19e−12
Sdc2 9.25e−12
Zfand5 9.56e−12
S3−12 9.93e−12
Slc27a1 1.05e−11
Cul3 1.05e−11
Glul 1.06e−11

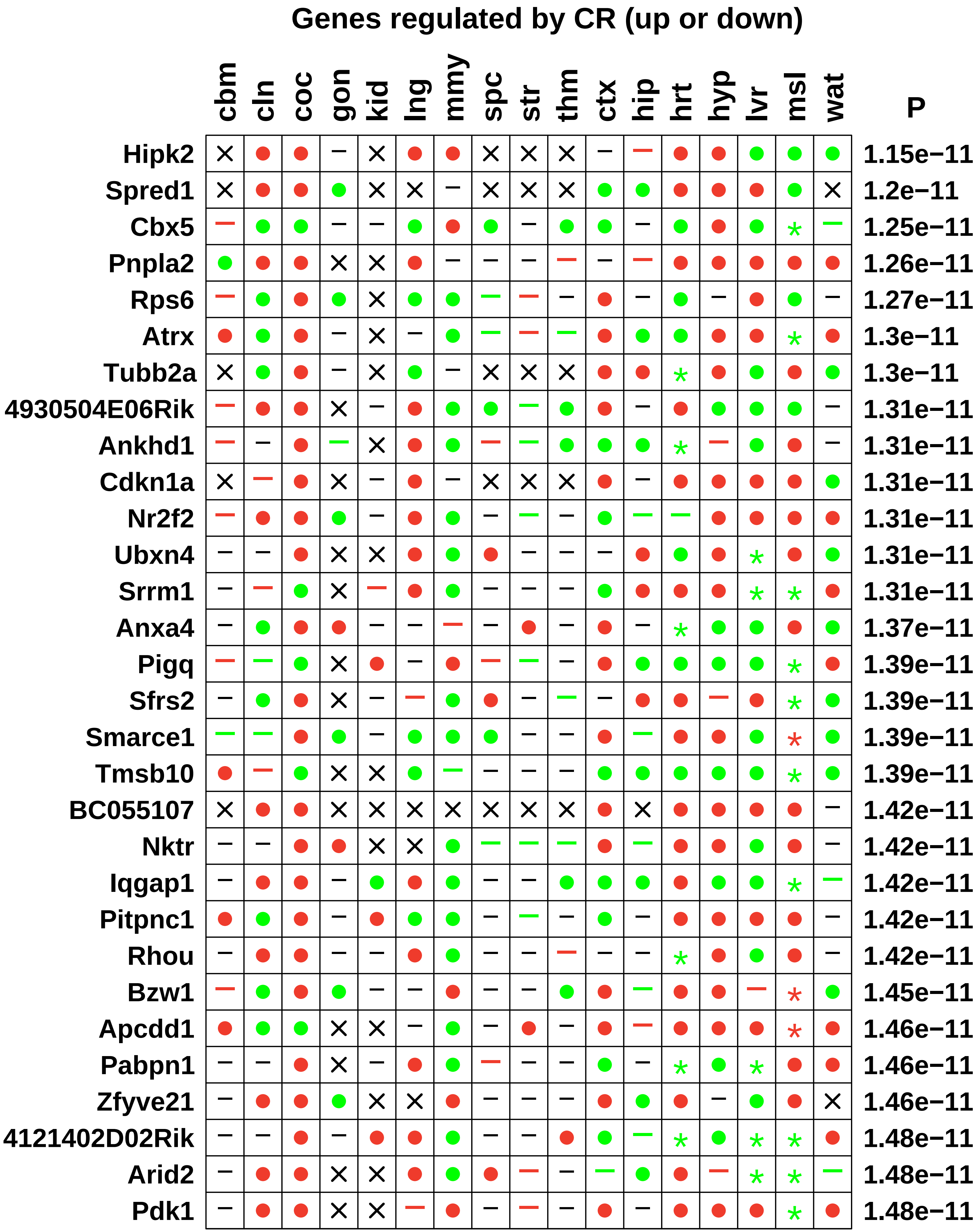
Genes regulated by CR (up or down)
cbm
cln
coc
gon
kid
lng
mmy
spc
str
thm
ctx
hip
hrt
hyp
lvr
msl
wat
P
Hipk2 1.15e−11
Spred1 1.2e−11
Cbx5 1.25e−11
Pnpla2 1.26e−11
Rps6 1.27e−11
Atrx 1.3e−11
Tubb2a 1.3e−11
4930504E06Rik 1.31e−11
Ankhd1 1.31e−11
Cdkn1a 1.31e−11
Nr2f2 1.31e−11
Ubxn4 1.31e−11
Srrm1 1.31e−11
Anxa4 1.37e−11
Pigq 1.39e−11
Sfrs2 1.39e−11
Smarce1 1.39e−11
Tmsb10 1.39e−11
BC055107 1.42e−11
Nktr 1.42e−11
Iqgap1 1.42e−11
Pitpnc1 1.42e−11
Rhou 1.42e−11
Bzw1 1.45e−11
Apcdd1 1.46e−11
Pabpn1 1.46e−11
Zfyve21 1.46e−11
4121402D02Rik 1.48e−11
Arid2 1.48e−11
Pdk1 1.48e−11

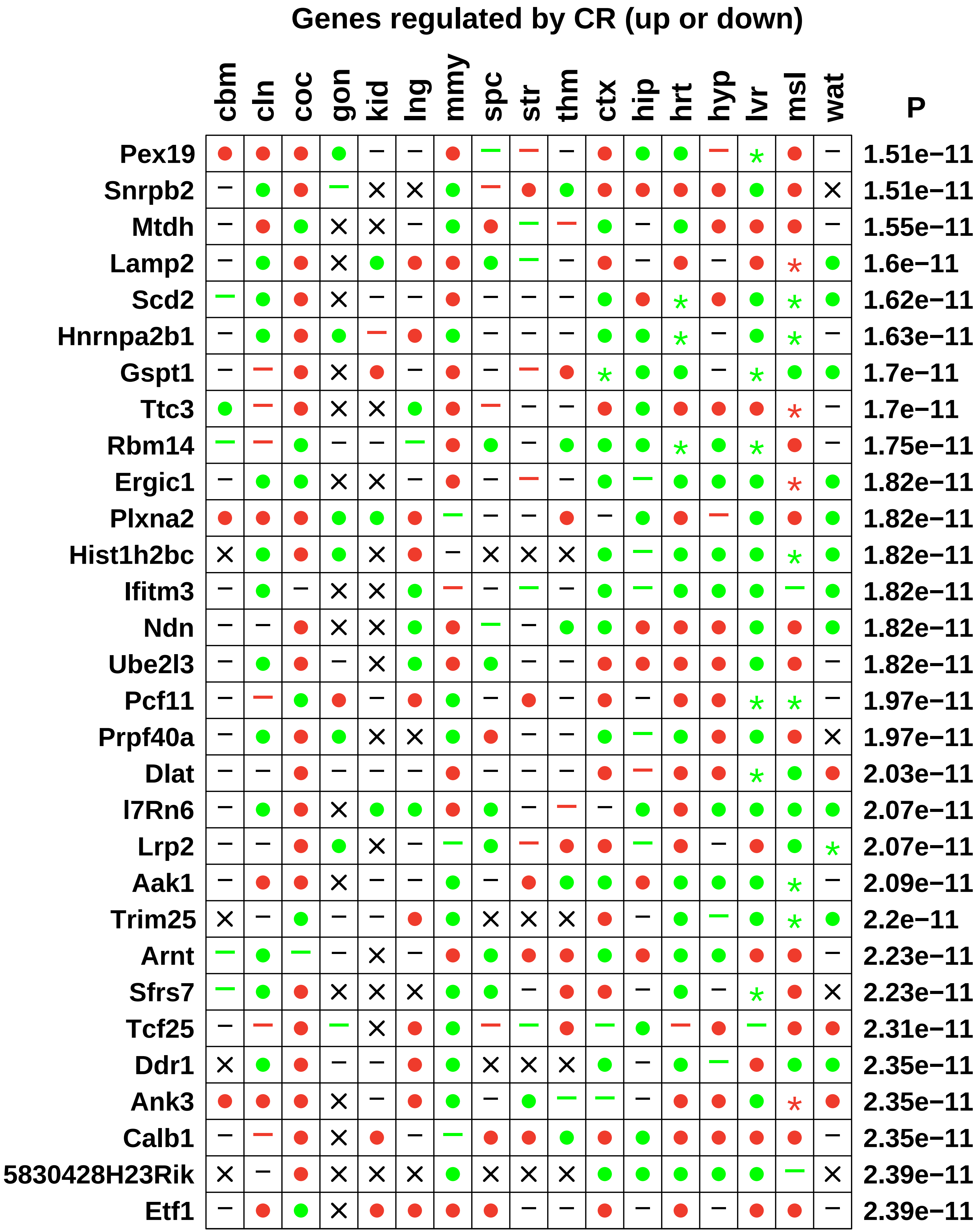
Genes regulated by CR (up or down)
cbm
cln
coc
gon
kid
lng
mmy
spc
str
thm
ctx
hip
hrt
hyp
lvr
msl
wat
P
Pex19
1.51e−11
Snrpb2
1.51e−11
Mtdh
1.55e−11
Lamp2
1.6e−11
Scd2
1.62e−11
Hnrnpa2b1
1.63e−11
Gspt1
1.7e−11
Ttc3
1.7e−11
Rbm14
1.75e−11
Ergic1
1.82e−11
Plxna2
1.82e−11
Hist1h2bc
1.82e−11
Ifitm3
1.82e−11
Ndn
1.82e−11
Ube2l3
1.82e−11
Pcf11
1.97e−11
Prpf40a
1.97e−11
Dlat
2.03e−11
l7Rn6
2.07e−11
Lrp2
2.07e−11
Aak1
2.09e−11
Trim25
2.2e−11
Arnt
2.23e−11
Sfrs7
2.23e−11
Tcf25
2.31e−11
Ddr1
2.35e−11
Ank3
2.35e−11
Calb1
2.35e−11
5830428H23Rik
2.39e−11
Etf1
2.39e−11

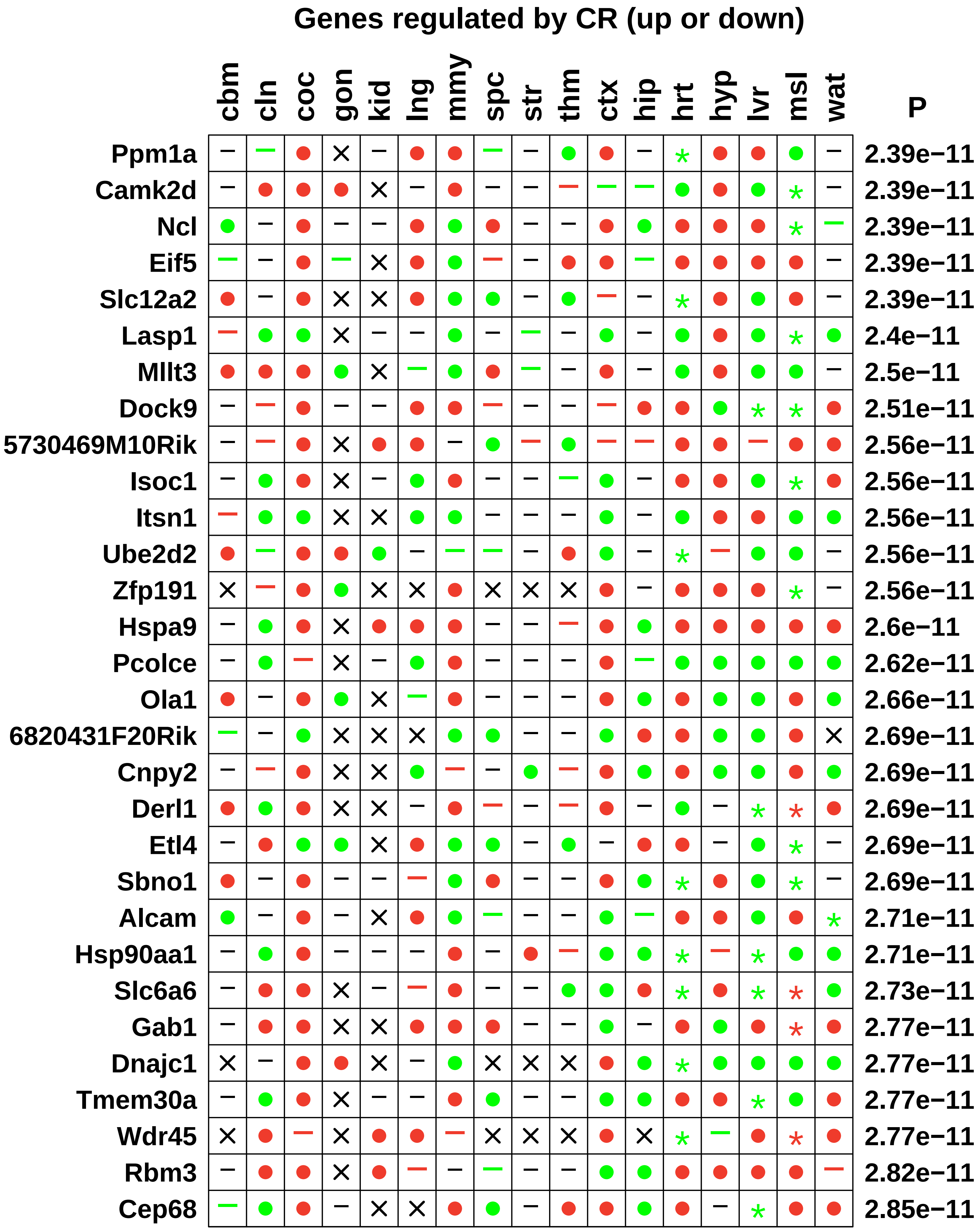
Genes regulated by CR (up or down)
cbm
cln
coc
gon
kid
lng
mmy
spc
str
thm
ctx
hip
hrt
hyp
lvr
msl
wat
P
Ppm1a 2.39e−11
Camk2d 2.39e−11
Ncl 2.39e−11
Eif5 2.39e−11
Slc12a2 2.39e−11
Lasp1 2.4e−11
Mllt3 2.5e−11
Dock9 2.51e−11
5730469M10Rik 2.56e−11
Isoc1 2.56e−11
Itsn1 2.56e−11
Ube2d2 2.56e−11
Zfp191 2.56e−11
Hspa9 2.6e−11
Pcolce 2.62e−11
Ola1 2.66e−11
6820431F20Rik 2.69e−11
Cnpy2 2.69e−11
Derl1 2.69e−11
Etl4 2.69e−11
Sbno1 2.69e−11
Alcam 2.71e−11
Hsp90aa1 2.71e−11
Slc6a6 2.73e−11
Gab1 2.77e−11
Dnajc1 2.77e−11
Tmem30a 2.77e−11
Wdr45 2.77e−11
Rbm3 2.82e−11
Cep68 2.85e−11

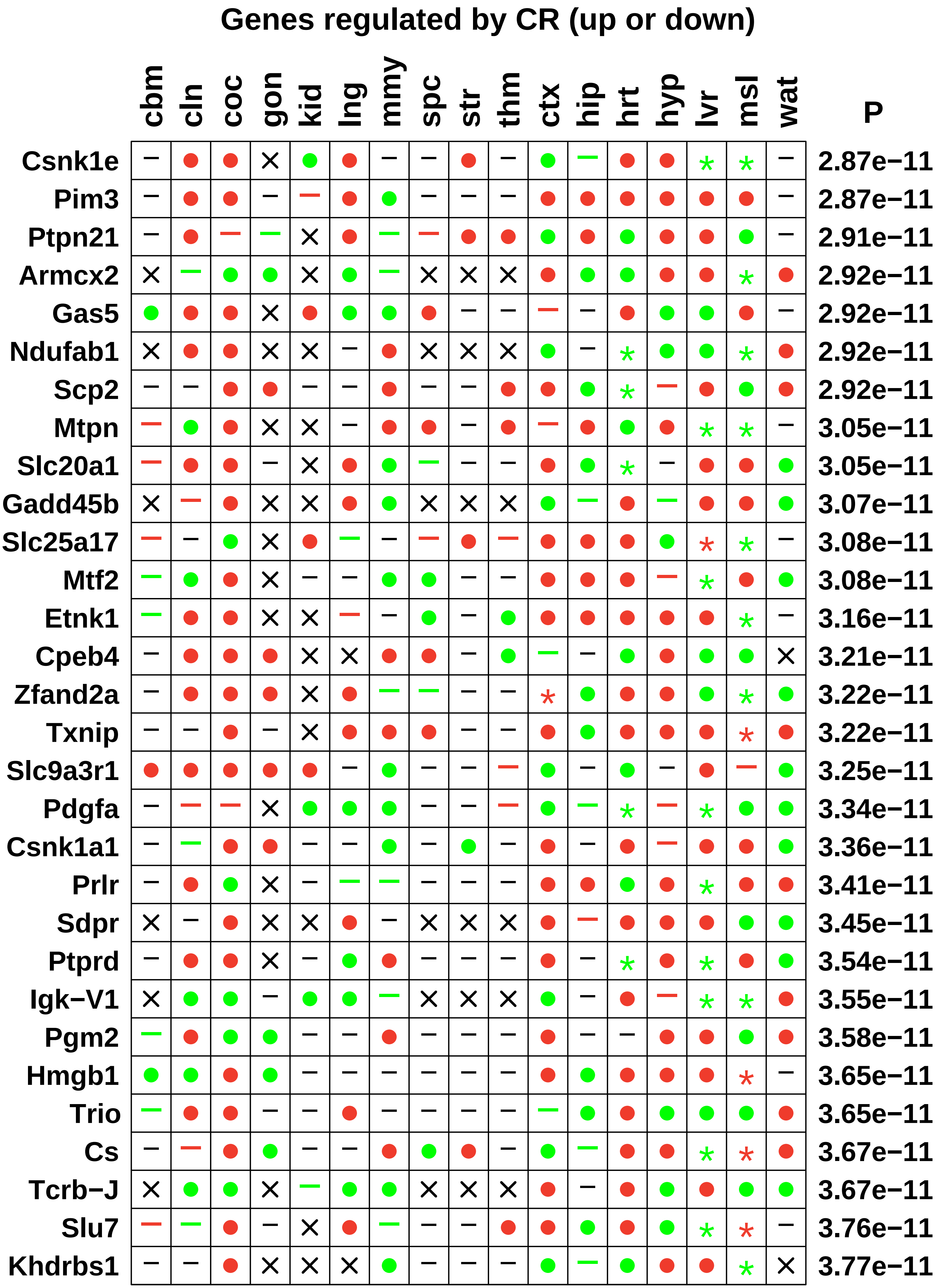
Genes regulated by CR (up or down)
cbm
cln
coc
gon
kid
lng
mmy
spc
str
thm
ctx
hip
hrt
hyp
lvr
msl
wat
P
Csnk1e 2.87e−11
Pim3 2.87e−11
Ptpn21 2.91e−11
Armcx2 2.92e−11
Gas5 2.92e−11
Ndufab1 2.92e−11
Scp2 2.92e−11
Mtpn 3.05e−11
Slc20a1 3.05e−11
Gadd45b 3.07e−11
Slc25a17 3.08e−11
Mtf2 3.08e−11
Etnk1 3.16e−11
Cpeb4 3.21e−11
Zfand2a 3.22e−11
Txnip 3.22e−11
Slc9a3r1 3.25e−11
Pdgfa 3.34e−11
Csnk1a1 3.36e−11
Prlr 3.41e−11
Sdpr 3.45e−11
Ptprd 3.54e−11
Igk−V1 3.55e−11
Pgm2 3.58e−11
Hmgb1 3.65e−11
Trio 3.65e−11
Cs 3.67e−11
Tcrb−J 3.67e−11
Slu7 3.76e−11
Khdrbs1 3.77e−11

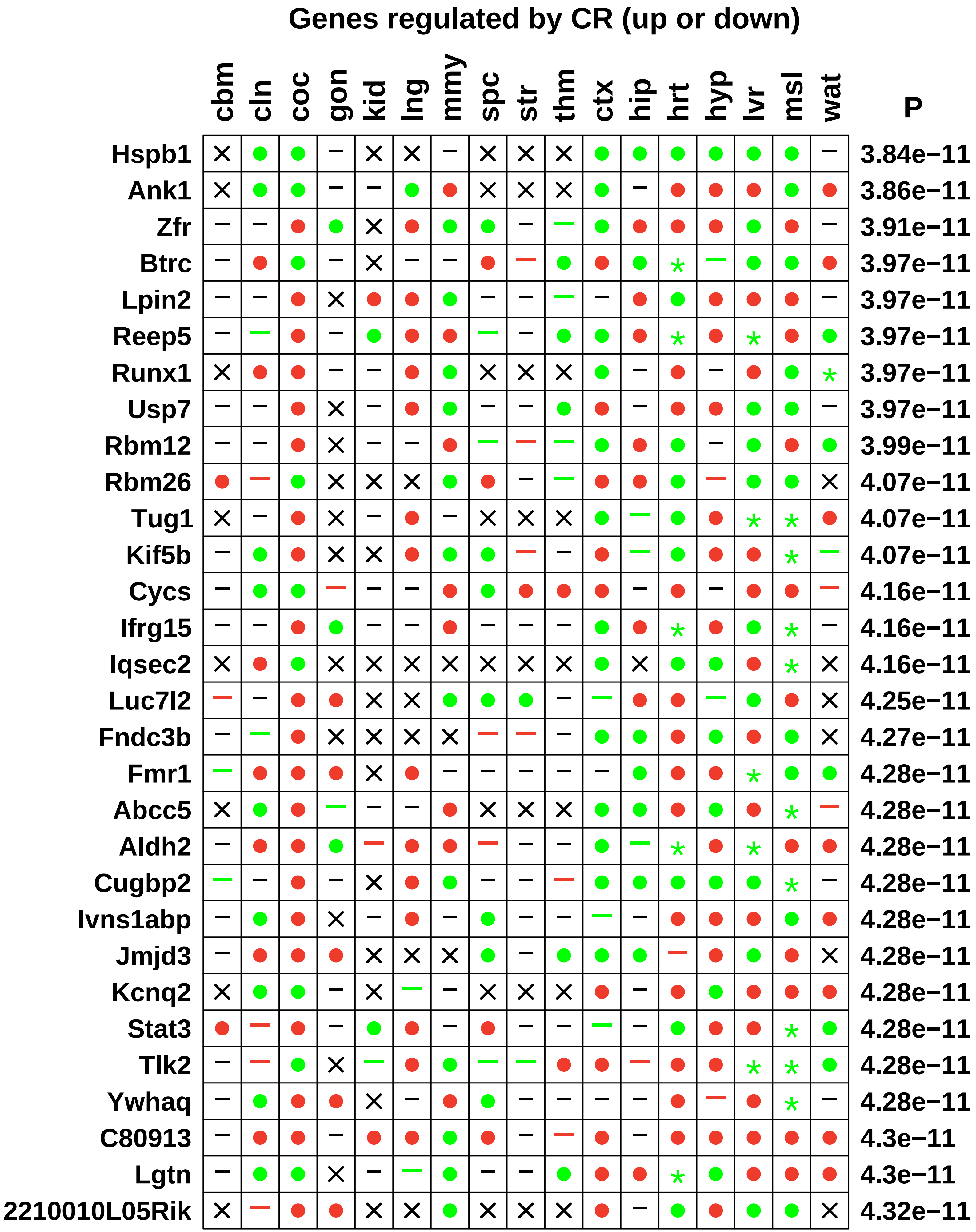
Genes regulated by CR (up or down)
cbm
cln
coc
gon
kid
lng
mmy
spc
str
thm
ctx
hip
hrt
hyp
lvr
msl
wat
P
Hspb1
Ank1
Zfr
Btrc
Lpin2
Reep5
Runx1
Usp7
Rbm12
Rbm26
Tug1
Kif5b
Cycs
Ifrg15
Iqsec2
Luc7l2
Fndc3b
Fmr1
Abcc5
Aldh2
Cugbp2
Ivns1abp
Jmjd3
Kcnq2
Stat3
Tlk2
Ywhaq
C80913
Lgtn
2210010L05Rik
3.84e−11
3.86e−11
3.91e−11
3.97e−11
3.97e−11
3.97e−11
3.97e−11
3.97e−11
3.99e−11
4.07e−11
4.07e−11
4.07e−11
4.16e−11
4.16e−11
4.16e−11
4.25e−11
4.27e−11
4.28e−11
4.28e−11
4.28e−11
4.28e−11
4.28e−11
4.28e−11
4.28e−11
4.28e−11
4.28e−11
4.28e−11
4.3e−11
4.3e−11
4.32e−11

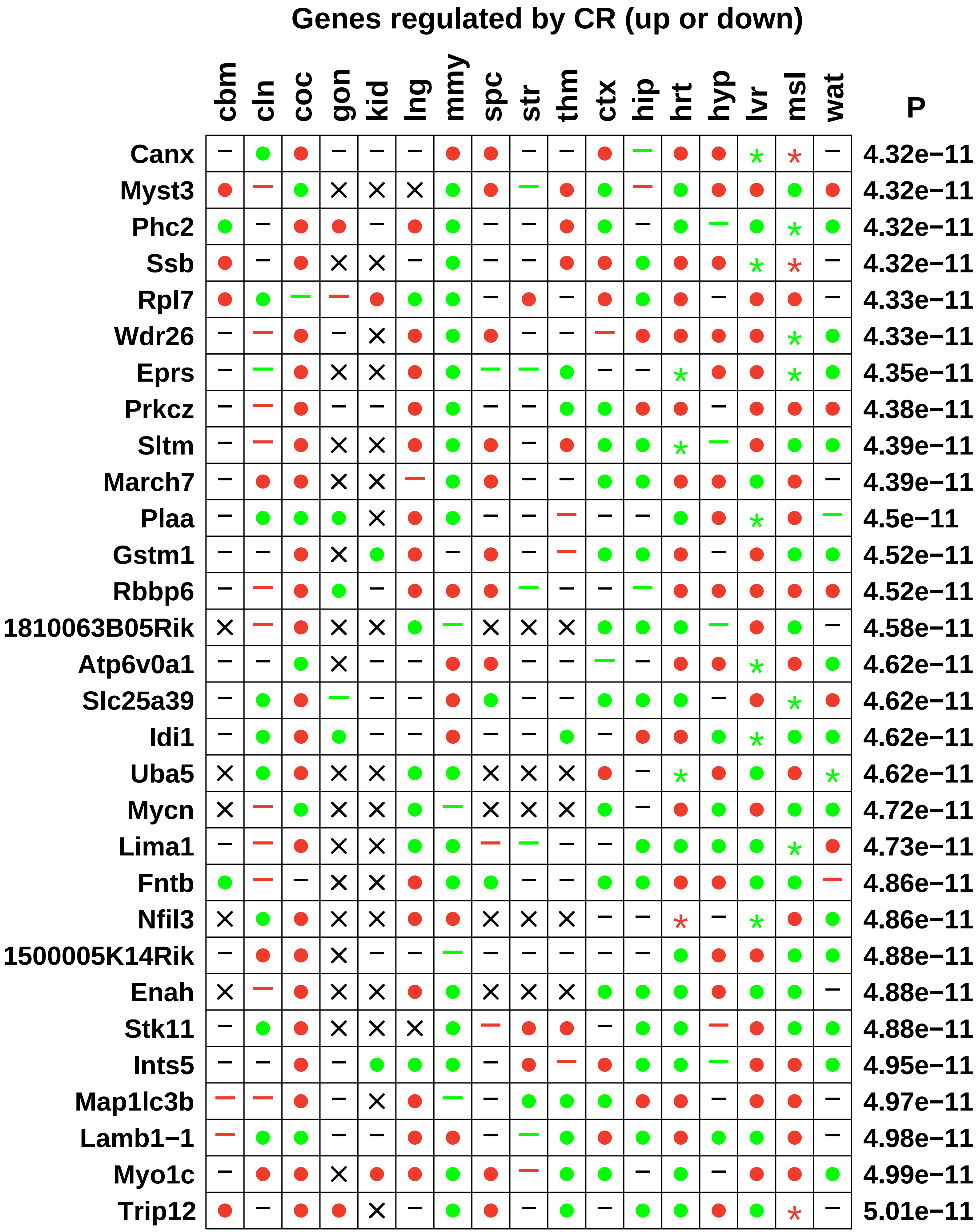
Genes regulated by CR (up or down)
cbm
cln
coc
gon
kid
lng
mmy
spc
str
thm
ctx
hip
hrt
hyp
lvr
msl
wat
P
Canx 4.32e−11
Myst3 4.32e−11
Phc2 4.32e−11
Ssb 4.32e−11
Rpl7 4.33e−11
Wdr26 4.33e−11
Eprs 4.35e−11
Prkcz 4.38e−11
Sltm 4.39e−11
March7 4.39e−11
Plaa 4.5e−11
Gstm1 4.52e−11
Rbbp6 4.52e−11
1810063B05Rik 4.58e−11
Atp6v0a1 4.62e−11
Slc25a39 4.62e−11
Idi1 4.62e−11
Uba5 4.62e−11
Mycn 4.72e−11
Lima1 4.73e−11
Fntb 4.86e−11
Nfil3 4.86e−11
1500005K14Rik 4.88e−11
Enah 4.88e−11
Stk11 4.88e−11
Ints5 4.95e−11
Map1lc3b 4.97e−11
Lamb1−1 4.98e−11
Myo1c 4.99e−11
Trip12 5.01e−11

↑ CR

Overrepresented Biological Processes

GO Term	P−Value
generation of precursor metabolites and energy	6.62e−05
ATP synthesis coupled electron transport	0.000113
oxidation reduction	0.00018
cellular lipid metabolic process	0.000299
regulation of programmed cell death	0.000312
negative regulation of transcription	0.000835
negative regulation of biosynthetic process	0.000857
acyl−CoA metabolic process	0.00106
phosphorus metabolic process	0.00131
negative regulation of cell cycle	0.00166
mitochondrial electron transport, NADH to ubiquinone	0.00166
negative regulation of RNA metabolic process	0.00186
monocarboxylic acid metabolic process	0.0019
nitric oxide mediated signal transduction	0.00205
phosphorylation	0.00242
coenzyme metabolic process	0.00329
metabolic process	0.00341
positive regulation of developmental process	0.00342
glucose metabolic process	0.00432
positive regulation of apoptosis	0.00459
circadian rhythm	0.00467
detoxification of copper ion	0.00595
regulation of glucocorticoid metabolic process	0.00595
peroxisome organization and biogenesis	0.00617
negative regulation of macromolecule metabolic process	0.00775
negative regulation of developmental process	0.00846
cofactor catabolic process	0.00899
monosaccharide metabolic process	0.00918
negative regulation of myeloid cell differentiation	0.00935
protein modification process	0.00984

↑ CR

Overrepresented Biological Processes

GO Term	P−Value
cellular macromolecule metabolic process	0.0102
protein metabolic process	0.0103
primary metabolic process	0.011
tricarboxylic acid cycle	0.0113
mitochondrial electron transport, ubiquinol to cytochrome c	0.0115
cell migration in hindbrain	0.0115
regulation of lipase activity	0.0115
glycolysis	0.0123
alcohol catabolic process	0.0127
cell death	0.0128
negative regulation of cellular metabolic process	0.0139
protein folding	0.0141
regulation of cell motility	0.0145
positive regulation of cell migration	0.0159
ubiquitin−dependent protein catabolic process	0.0169
erythrocyte differentiation	0.0173
negative regulation of neuron apoptosis	0.0175
negative regulation of transcription from RNA polymerase II promoter	0.0182
protein modification by small protein conjugation	0.0186
acetyl−CoA biosynthetic process	0.0187
histone ubiquitination	0.0187
triacylglycerol catabolic process	0.0187
glycerol ether catabolic process	0.0187
neutral lipid catabolic process	0.0187
glycerolipid catabolic process	0.0187
long−chain fatty acid metabolic process	0.0188
modification−dependent macromolecule catabolic process	0.0189
regulation of heart contraction	0.0197
homeostasis of number of cells	0.0217
proteolysis involved in cellular protein catabolic process	0.0223

↑ CR

Overrepresented Biological Processes

GO Term	P−Value
induction of apoptosis	0.0226
protein import into nucleus, translocation	0.0231
negative regulation of apoptosis	0.0238
carbohydrate metabolic process	0.024
RNA splicing, via transesterification reactions with bulged adenosine as nucleophile	0.0247
insulin receptor signaling pathway	0.0247
protein amino acid phosphorylation	0.0268
proton transport	0.0268
hexose catabolic process	0.0268
vitamin metabolic process	0.027
mitochondrial genome maintenance	0.0272
epithelial cell maturation	0.0272
malate metabolic process	0.0272
cellular zinc ion homeostasis	0.0272
response to cytokine stimulus	0.0272
protein import into mitochondrial inner membrane	0.0272
isoprenoid biosynthetic process	0.0279
transmembrane transport	0.0281
organic acid metabolic process	0.0292
protein targeting	0.0297
response to oxidative stress	0.0317
regulation of lipid metabolic process	0.0319
mitochondrial membrane organization and biogenesis	0.0333
DNA damage response, signal transduction by p53 class mediator	0.0333
translation	0.0347
cellular process	0.0352
cellular respiration	0.0359
covalent chromatin modification	0.0365
protein targeting to mitochondrion	0.0369
enucleate erythrocyte differentiation	0.0369

↑ CR

Overrepresented Biological Processes

GO Term	P−Value
negative regulation of fibroblast proliferation	0.0369
protein import	0.0384
vitamin A metabolic process	0.0391
liver development	0.0402
glucose transport	0.0448
respiratory electron transport chain	0.047
negative regulation of biological process	0.0471
suckling behavior	0.0478
behavioral response to pain	0.0478
monosaccharide transport	0.0497

↑ CR

Overrepresented Cell Components

GO Term	P−Value
organelle membrane	2.08e−12
mitochondrial envelope	5.39e−10
mitochondrion	5.61e−10
envelope	2.25e−09
organelle	1.08e−07
cytoplasm	9.07e−07
mitochondrial respiratory chain	7.98e−06
intracellular organelle part	1.05e−05
mitochondrial inner membrane	6.05e−05
cytosol	0.000167
intracellular membrane−bounded organelle	0.000295
intracellular part	0.000502
cell	0.000623
nuclear envelope−endoplasmic reticulum network	0.00104
glycogen granule	0.00183
ubiquitin ligase complex	0.00279
endomembrane system	0.00622
peroxisome	0.0068
stress fiber	0.00769
nucleus	0.00779
vacuolar part	0.0093
lipid particle	0.00994
cytoplasmic vesicle membrane	0.0118
organelle lumen	0.0152
cytoplasmic part	0.0183
proton−transporting two−sector ATPase complex	0.0198
macromolecular complex	0.0218
intrinsic to endoplasmic reticulum membrane	0.0243
mitochondrial intermembrane space protein transporter complex	0.0244
large ribosomal subunit	0.0266

↑ CR

Overrepresented Cell Components

GO Term	P−Value
peroxisomal membrane	0.0288
PML body	0.0288
lysosomal membrane	0.0333
mitochondrial large ribosomal subunit	0.0339
mitochondrial matrix	0.0418
prefoldin complex	0.0431
integral to endoplasmic reticulum membrane	0.0453
microbody part	0.0453
cytoplasmic vesicle	0.047

↑ CR

Overrepresented Molecular Functions

GO Term	P−Value
transferase activity	9.87e−06
nucleotide binding	9.81e−05
kinase activity	0.000441
transcription corepressor activity	0.000562
RNA splicing factor activity, transesterification mechanism	0.000771
oxidoreductase activity	0.00113
magnesium ion binding	0.00151
transcription repressor activity	0.00172
DNA photolyase activity	0.0019
tyrosine−ester sulfotransferase activity	0.0019
monovalent inorganic cation transmembrane transporter activity	0.00264
phosphotransferase activity, alcohol group as acceptor	0.00282
unfolded protein binding	0.00311
protein dimerization activity	0.00505
acetyl−CoA C−acyltransferase activity	0.00552
heat shock protein binding	0.00564
protein serine/threonine kinase activity	0.00566
ATPase activity, coupled to transmembrane movement of ions	0.00999
FAD binding	0.0105
transferase activity, transferring acyl groups, acyl groups converted into alkyl on transfer	0.0107
aryl sulfotransferase activity	0.0107
retinoid X receptor binding	0.0107
microtubule plus−end binding	0.0107
hydrogen ion transporting ATP synthase activity, rotational mechanism	0.0117
hydrogen ion transporting ATPase activity, rotational mechanism	0.0117
glutathione transferase activity	0.014
prenyltransferase activity	0.017
3−hydroxyacyl−CoA dehydrogenase activity	0.0174
flavin−containing monooxygenase activity	0.0174
S−acyltransferase activity	0.0174

↑ CR

Overrepresented Molecular Functions

GO Term	P−Value
lyase activity	0.0174
RNA binding	0.0181
coenzyme binding	0.0185
hormone receptor binding	0.0209
zinc ion binding	0.023
structural constituent of ribosome	0.0253
specific transcriptional repressor activity	0.0253
oxidoreductase activity, acting on the CH−CH group of donors, NAD or NADP as acceptor	0.0253
protein methyltransferase activity	0.0262
transcription factor binding	0.0266
ion binding	0.0286
small conjugating protein ligase activity	0.03
CoA hydrolase activity	0.0302
electron carrier activity	0.0335
aldehyde dehydrogenase (NAD) activity	0.0344
malate dehydrogenase activity	0.0344
oxidoreductase activity, acting on the aldehyde or oxo group of donors, disulfide as acceptor	0.0344
P−P−bond−hydrolysis−driven transmembrane transporter activity	0.0356
two−component sensor activity	0.0357
adenyl nucleotide binding	0.0444
adenylate kinase activity	0.0446
ubiquinol−cytochrome−c reductase activity	0.0446
metalloendopeptidase inhibitor activity	0.0446
oxidoreductase activity, acting on diphenols and related substances as donors	0.0446
purine ribonucleotide binding	0.0481

↓ CR

Overrepresented Biological Processes

GO Term	P−Value
macromolecular complex subunit organization	3.18e−05
antigen processing and presentation of exogenous peptide antigen via MHC class II	7.47e−05
phosphate transport	0.000114
antigen processing and presentation of peptide or polysaccharide antigen via MHC class II	0.000146
cellular macromolecule metabolic process	0.00022
DNA replication	0.000303
chaperone cofactor−dependent protein folding	0.000414
nuclear import	0.000674
antigen processing and presentation of exogenous antigen	0.000678
'de novo' protein folding	0.000796
nucleosome assembly	0.00098
protein import into nucleus, docking	0.00106
protein metabolic process	0.00123
protein import	0.00169
antigen processing and presentation of peptide antigen	0.0032
regulation of biological process	0.0033
cellular component assembly	0.0035
cellular process	0.00351
DNA packaging	0.00424
antigen processing and presentation	0.00428
cellular macromolecular complex assembly	0.00456
regulation of cellular metabolic process	0.00538
chromosome organization and biogenesis	0.00554
mRNA stabilization	0.00585
regulation of astrocyte differentiation	0.00585
chromatin assembly or disassembly	0.00654
steroid hormone receptor signaling pathway	0.00729
biopolymer metabolic process	0.00828
posttranscriptional regulation of gene expression	0.00908
nuclear transport	0.0103

↓CR

Overrepresented Biological Processes

GO Term	P−Value
regulation of transcription from RNA polymerase II promoter	0.0107
regulation of biosynthetic process	0.0109
protein folding	0.0129
tissue homeostasis	0.0129
regulation of RNA metabolic process	0.0131
mRNA splice site selection	0.014
ER overload response	0.014
response to unfolded protein	0.0146
vasculogenesis	0.0163
cytoskeleton organization and biogenesis	0.017
nucleobase, nucleoside, nucleotide and nucleic acid metabolic process	0.0174
anion transport	0.0175
T cell differentiation	0.0188
regulation of mRNA stability	0.019
regulation of transcription	0.0201
regulation of cellular component organization and biogenesis	0.0207
cellular protein complex assembly	0.0228
protein targeting	0.0239
intracellular signaling cascade	0.0239
regulation of protein metabolic process	0.0245
central nervous system projection neuron axonogenesis	0.0251
positive regulation of neuron apoptosis	0.0251
biopolymer biosynthetic process	0.0252
immune response	0.0254
actin filament organization	0.027
gene expression	0.0282
leukocyte differentiation	0.0302
peptidyl−amino acid modification	0.0309
regulation of Ras protein signal transduction	0.0315
regulation of protein localization	0.0316

↓CR

Overrepresented Biological Processes

GO Term	P−Value
protein targeting to ER	0.0316
regulation of nitric oxide biosynthetic process	0.0316
germ cell development	0.0322
regulation of anatomical structure morphogenesis	0.0322
lung development	0.0324
glial cell differentiation	0.035
GPI anchor biosynthetic process	0.0357
actin filament polymerization	0.0357
axon extension	0.0357
transcription, DNA−dependent	0.0377
regulation of macromolecule metabolic process	0.0379
regulation of cell−matrix adhesion	0.0387
regulation of gliogenesis	0.0387
endoplasmic reticulum unfolded protein response	0.0387
positive regulation of cell differentiation	0.039
actin cytoskeleton organization and biogenesis	0.0401
negative regulation of cellular process	0.0413
blastocyst development	0.0442
blood vessel development	0.0446
protein modification process	0.0455
trophectodermal cell differentiation	0.0463
insulin−like growth factor receptor signaling pathway	0.0463

↓ CR

Overrepresented Cell Components

GO Term	P−Value
intracellular	5.18e−08
collagen	1e−06
intracellular organelle	9.1e−06
nucleus	1.29e−05
cytoplasm	4.02e−05
MHC class II protein complex	0.000104
extracellular matrix	0.000166
cell	0.000198
membrane−bounded organelle	0.000239
tubulin complex	0.000973
multivesicular body	0.00151
basement membrane	0.002
replisome	0.00221
nuclear replication fork	0.00221
chromosome	0.00357
soluble fraction	0.0054
alpha DNA polymerase:primase complex	0.0056
protein serine/threonine phosphatase complex	0.00931
non−membrane−bounded organelle	0.00956
ubiquitin ligase complex	0.0127
collagen type IV	0.0134
SWI/SNF complex	0.0134
nucleosome	0.0151
ruffle	0.0207
filamentous actin	0.024
macromolecular complex	0.0256
endoplasmic reticulum part	0.0388
actin filament	0.0441
septin complex	0.0444
cell cortex	0.0465

↓ CR

Overrepresented Molecular Functions

GO Term	P−Value
extracellular matrix structural constituent	3.48e−07
protein binding	3.58e−06
unfolded protein binding	5.34e−05
nucleotide binding	8.45e−05
AU−rich element binding	0.000112
DNA binding	0.00103
purine ribonucleotide binding	0.0032
peptide antigen binding	0.0054
GTPase activity	0.00665
DNA−directed DNA polymerase activity	0.00762
ribosome binding	0.00882
pyrophosphatase activity	0.00905
hydrolase activity, acting on acid anhydrides	0.0101
hydrolase activity, acting on ether bonds	0.013
GTP binding	0.013
guanyl nucleotide binding	0.0155
heat shock protein binding	0.0181
enzyme regulator activity	0.0185
vascular endothelial growth factor receptor activity	0.0232
low−density lipoprotein receptor binding	0.0232
magnesium ion binding	0.0232
non−membrane spanning protein tyrosine kinase activity	0.0283
cytoskeletal protein binding	0.0332
structural constituent of cytoskeleton	0.0359
Rho guanyl−nucleotide exchange factor activity	0.0364
ATP binding	0.0383
protein transporter activity	0.0387
ligase activity	0.0399
transcription regulator activity	0.0404
oxidoreductase activity, acting on single donors with incorporation of molecular oxygen, incorporation of tw	0.0411

↓ CR

Overrepresented Molecular Functions

GO Term	P−Value
receptor signaling protein activity	0.044
actin filament binding	0.0441
adenyl nucleotide binding	0.0461

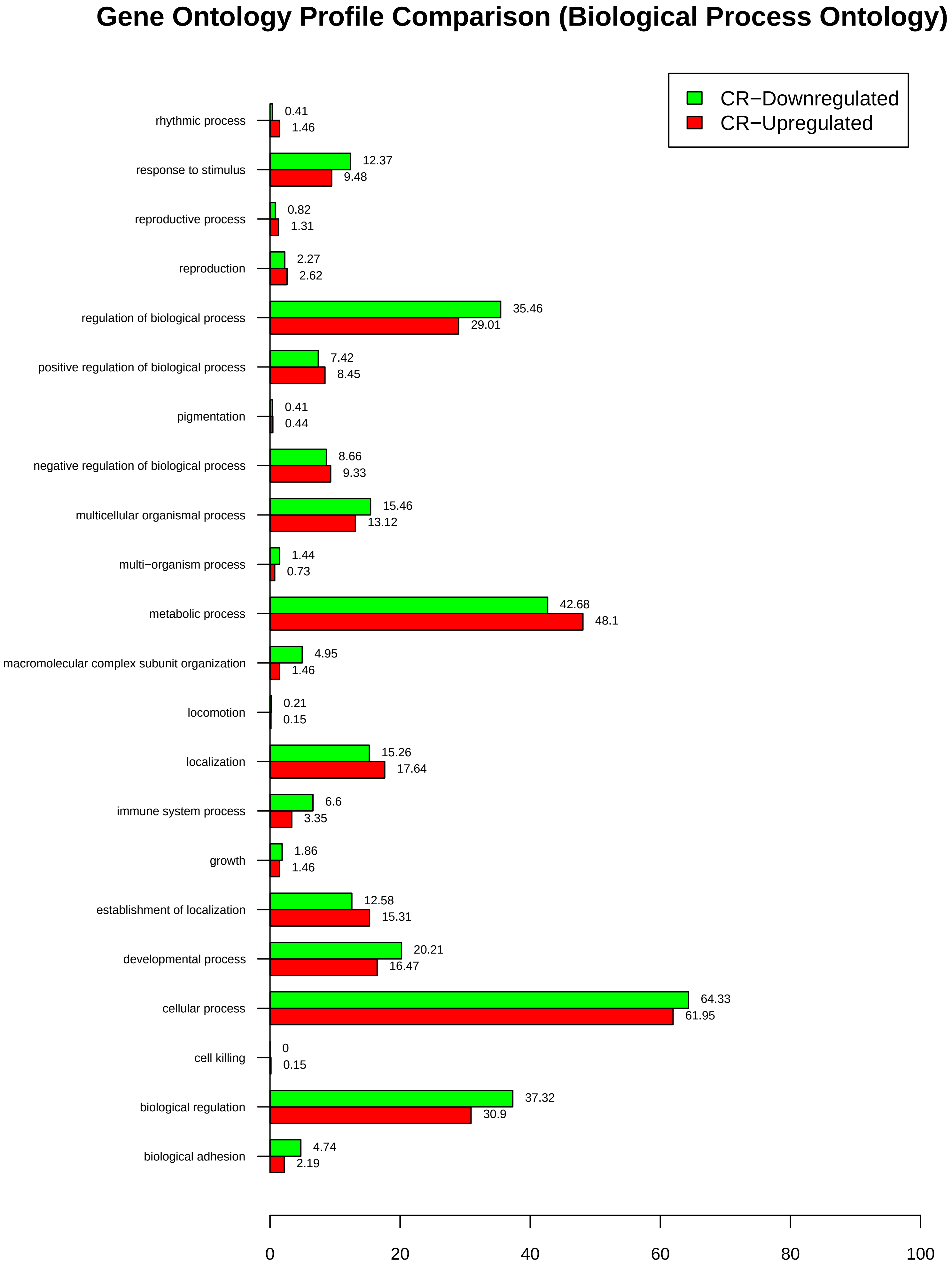

Gene Ontology Profile Comparison (Biological Process Ontology)
CR−Downregulated
CR−Upregulated
rhythmic process
0.41
1.46
response to stimulus
12.37
9.48
reproductive process
0.82
1.31
reproduction
2.27
2.62
regulation of biological process
35.46
29.01
positive regulation of biological process
7.42
8.45
pigmentation
0.41
0.44
negative regulation of biological process
8.66
9.33
multicellular organismal process
15.46
13.12
multi−organism process
1.44
0.73
metabolic process
42.68
48.1
macromolecular complex subunit organization
4.95
1.46
locomotion
0.21
0.15
localization
15.26
17.64
immune system process
6.6
3.35
growth
1.86
1.46
establishment of localization
12.58
15.31
developmental process
20.21
16.47
cellular process
64.33
61.95
cell killing
0
0.15
biological regulation
37.32
30.9
biological adhesion
4.74
2.19
0
20
40
60
80
100

Gene Ontology Profile Comparison (Cell Component Ontology)

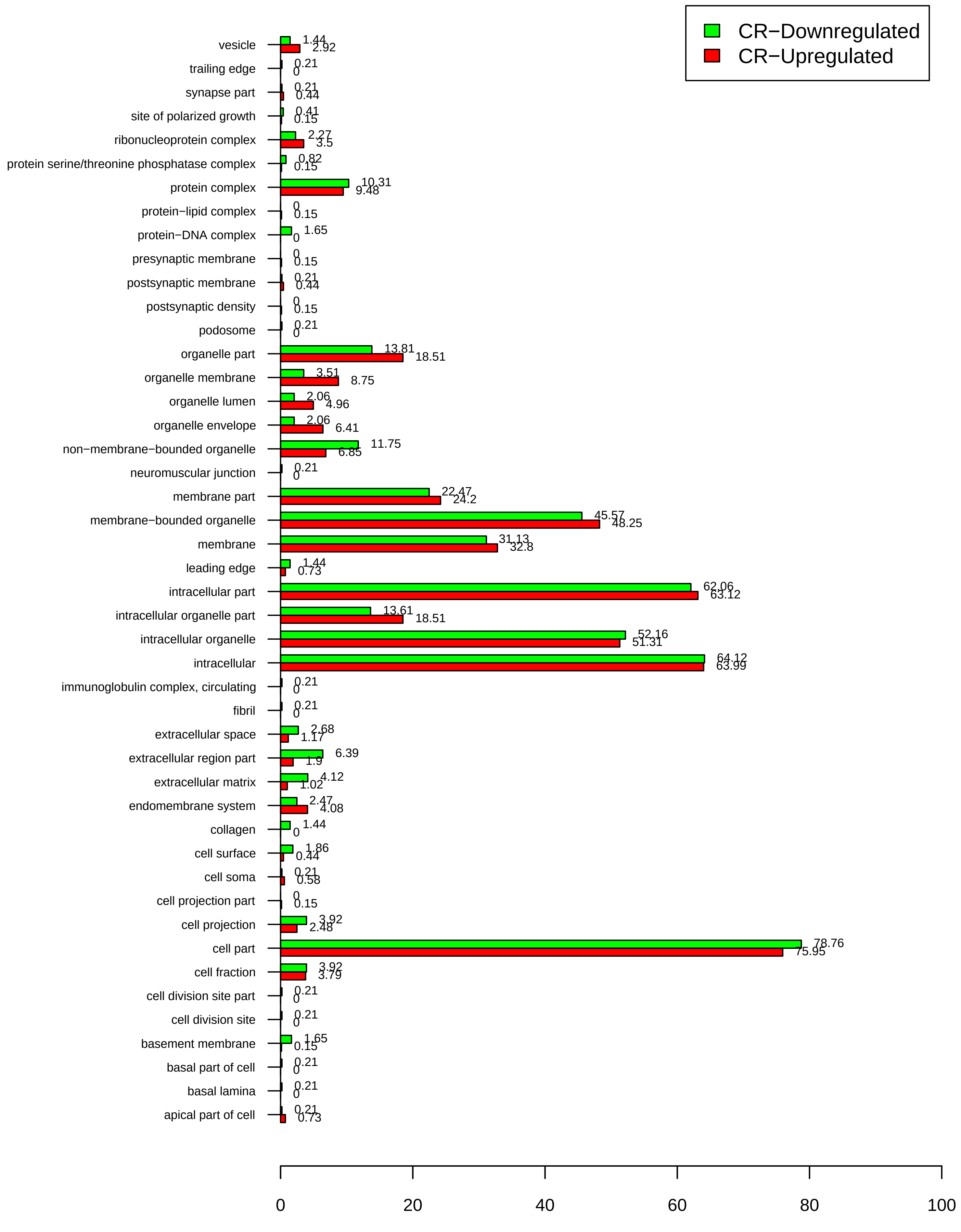

Gene Ontology Profile Comparison (Molecular Function Ontology)

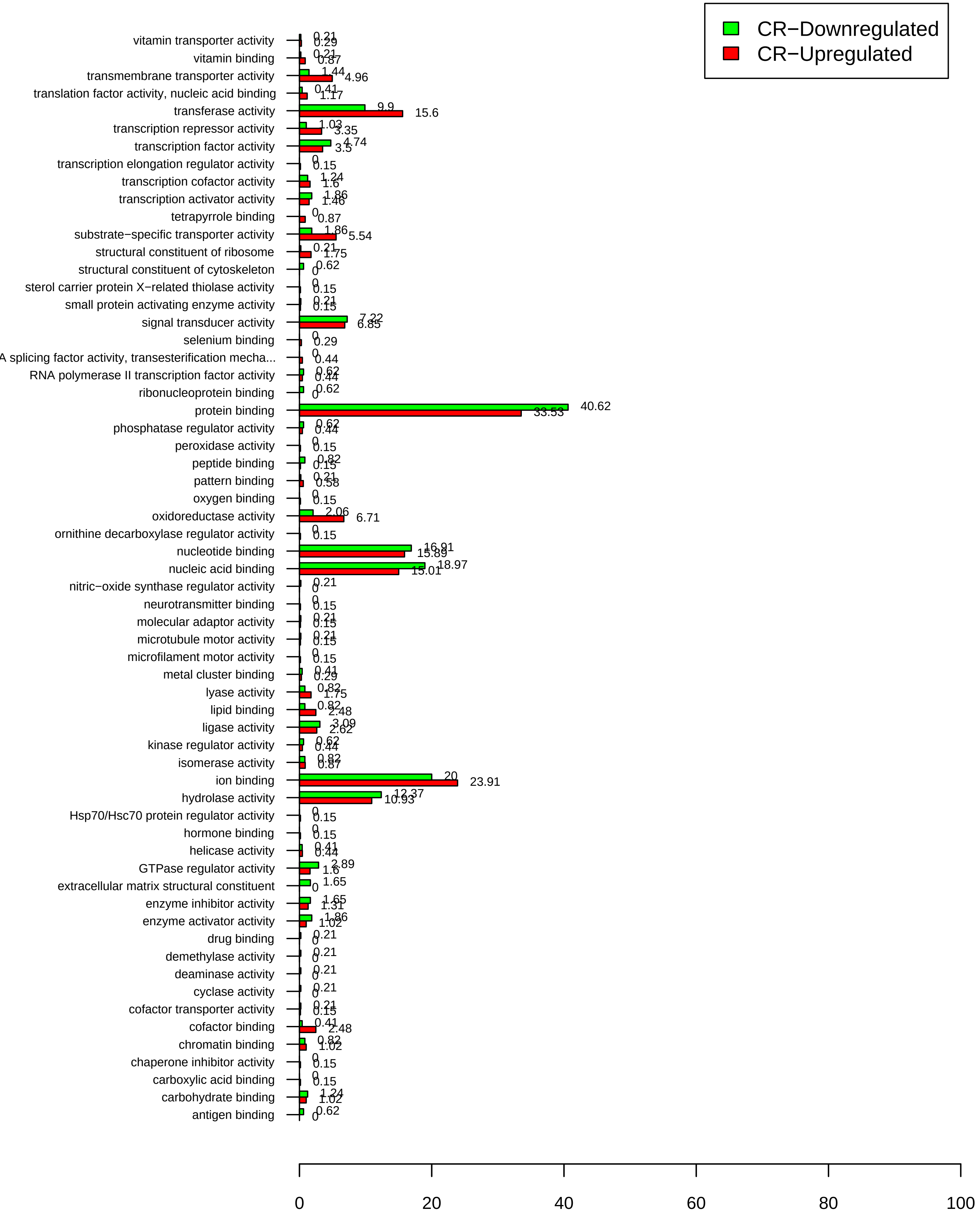

↑ CR

Overrepresented KEGG Pathways

GO Term	P−Value
Fatty acid metabolism	9.9e−06
Citrate cycle (TCA cycle)	4.75e−05
PPAR signaling pathway	8.24e−05
Oxidative phosphorylation	9.91e−05
Valine, leucine and isoleucine degradation	0.000285
Pyruvate metabolism	0.000285
Circadian rhythm	0.000424
Renal cell carcinoma	0.00114
Fatty acid elongation in mitochondria	0.00142
Insulin signaling pathway	0.00257
Geraniol degradation	0.00288
Adipocytokine signaling pathway	0.00484
Lysine degradation	0.00691
Biosynthesis of unsaturated fatty acids	0.0119
Retinol metabolism	0.0268
Terpenoid biosynthesis	0.0268
Carbon fixation	0.0295
Butanoate metabolism	0.0379
Adherens junction	0.0447

↓ CR

Overrepresented KEGG Pathways

GO Term	P−Value
Focal adhesion	4.8e−06
Antigen processing and presentation	1.21e−05
ECM−receptor interaction	0.000218
DNA replication	0.00113
MAPK signaling pathway	0.00245
Cell Communication	0.00379
VEGF signaling pathway	0.00719
Natural killer cell mediated cytotoxicity	0.00941
Glioma	0.0139
Type I diabetes mellitus	0.0155
Gap junction	0.0182
Prostate cancer	0.0205
Cell adhesion molecules (CAMs)	0.0229
Non−small cell lung cancer	0.025
Synthesis and degradation of ketone bodies	0.031
Protein export	0.0454

↑ CR

Overrepresented KEGG Pathways

(Based on InterPro Domain Signatures)

GO Term	P−Value
Fatty acid metabolism	1e−04
Biosynthesis of unsaturated fatty acids	1e−04
PPAR signaling pathway	1e−04
Citrate cycle (TCA cycle)	2e−04
Valine, leucine and isoleucine degradation	3e−04
Lysine degradation	3e−04
Fatty acid elongation in mitochondria	0.0011
Pyruvate metabolism	0.0011
Tryptophan metabolism	0.0012
Caprolactam degradation	0.0013
Glycolysis / Gluconeogenesis	0.0017
Adherens junction	0.0025
Reductive carboxylate cycle (CO2 fixation)	0.0026
Tight junction	0.0028
Drug metabolism − cytochrome P450	0.0036
Butanoate metabolism	0.0056
Glycerophospholipid metabolism	0.0075
Carbon fixation	0.0092
Geraniol degradation	0.0097
Riboflavin metabolism	0.0106
Glyoxylate and dicarboxylate metabolism	0.0119
Vitamin B6 metabolism	0.0129
Terpenoid biosynthesis	0.0156
Glycerolipid metabolism	0.0181
Aminosugars metabolism	0.019
Caffeine metabolism	0.0209
Prostate cancer	0.0222
Glutathione metabolism	0.0224
Nicotinate and nicotinamide metabolism	0.0308
Notch signaling pathway	0.0361

↑ CR

Overrepresented KEGG Pathways

(Based on InterPro Domain Signatures)

GO Term	P−Value
Base excision repair	0.0385
Pantothenate and CoA biosynthesis	0.0431
alpha−Linolenic acid metabolism	0.0438
Melanoma	0.0457
Renal cell carcinoma	0.0463
Huntington's disease	0.0488

↓ CR

Overrepresented KEGG Pathways

(Based on InterPro Domain Signatures)

GO Term	P−Value
DNA replication	1e−04
Base excision repair	1e−04
Mismatch repair	0.0012
Small cell lung cancer	0.0016
Nucleotide excision repair	0.0024
MAPK signaling pathway	0.0028
VEGF signaling pathway	0.0042
Non−homologous end−joining	0.0054
Toll−like receptor signaling pathway	0.0056
Apoptosis	0.0064
Natural killer cell mediated cytotoxicity	0.0064
Protein export	0.0073
Focal adhesion	0.0129
Antigen processing and presentation	0.0137
p53 signaling pathway	0.0141
Glycosphingolipid biosynthesis − globoseries	0.015
Tight junction	0.0173
Non−small cell lung cancer	0.0208
Glycosphingolipid biosynthesis − ganglioseries	0.0212
Acute myeloid leukemia	0.0224
Axon guidance	0.0266
Systemic lupus erythematosus	0.0278
Leukocyte transendothelial migration	0.028
Huntington's disease	0.0286
Adipocytokine signaling pathway	0.0308
Pyrimidine metabolism	0.0343
Prostate cancer	0.0343
Dentatorubropallidoluysian atrophy (DRPLA)	0.0382
Cell adhesion molecules (CAMs)	0.0416
Atrazine degradation	0.0477

↓ CR

Overrepresented KEGG Pathways

(Based on InterPro Domain Signatures)

GO Term	P−Value
Glutamate metabolism	0.0479
Oxidative phosphorylation	0.048

↑ CR

Abundance of miRNA Targets

miRNA	Freq(Obs)	Freq(Exp)	Obs/Exp	P−value	P−Value(Adj)
miR−19a	0.254	0.169	1.5	6.16e−08	3.02e−05
miR−703	0.191	0.124	1.54	1.29e−06	0.000144
miR−590−3p	0.347	0.261	1.33	1.29e−06	0.000144
miR−466a−3p	0.259	0.184	1.41	2.34e−06	0.000144
miR−466b−3−3p	0.259	0.184	1.41	2.34e−06	0.000144
miR−466b−3p	0.259	0.184	1.41	2.34e−06	0.000144
miR−466c−3p	0.259	0.184	1.41	2.34e−06	0.000144
miR−466e−3p	0.259	0.184	1.41	2.34e−06	0.000144
miR−19b	0.223	0.156	1.43	6.09e−06	0.000332
miR−467g	0.242	0.175	1.38	1.54e−05	0.000758
miR−466d−3p	0.227	0.163	1.39	2.18e−05	0.000974
miR−543	0.172	0.116	1.48	2.55e−05	0.000984
miR−466l	0.366	0.29	1.26	2.61e−05	0.000984
miR−186	0.273	0.206	1.33	3.62e−05	0.00127
miR−301b	0.216	0.156	1.38	4.53e−05	0.00148
miR−297b−3p	0.239	0.177	1.35	5.34e−05	0.00158
miR−466f−5p	0.115	0.0719	1.6	5.46e−05	0.00158
miR−17	0.229	0.168	1.36	6.63e−05	0.00181
miR−694	0.253	0.19	1.33	7.45e−05	0.00193
miR−568	0.22	0.161	1.36	7.91e−05	0.00194
miR−669f	0.284	0.219	1.29	9.89e−05	0.00231
miR−466i	0.208	0.153	1.36	0.000125	0.0028
miR−23b	0.196	0.142	1.38	0.000137	0.0029
miR−1192	0.229	0.171	1.33	0.000142	0.0029
miR−23a	0.194	0.142	1.37	0.000181	0.00352
miR−148b	0.179	0.129	1.39	0.000191	0.00352
miR−301a	0.216	0.162	1.34	0.000193	0.00352
miR−1191	0.129	0.0879	1.47	0.00033	0.00565
miR−743b−3p	0.268	0.211	1.27	0.000341	0.00565
miR−375	0.0464	0.0237	1.96	0.000356	0.00565

↑ CR

Abundance of miRNA Targets

miRNA	Freq(Obs)	Freq(Exp)	Obs/Exp	P−value	P−Value(Adj)
miR−92b	0.113	0.0753	1.51	0.000359	0.00565
miR−106a	0.211	0.16	1.32	0.000369	0.00565
miR−291b−3p	0.208	0.157	1.32	0.000444	0.00652
miR−26a	0.168	0.123	1.37	0.000451	0.00652
miR−539	0.229	0.176	1.3	0.00048	0.00659
miR−93	0.211	0.161	1.31	0.000483	0.00659
miR−590−5p	0.134	0.0942	1.42	0.000615	0.00816
miR−335−3p	0.273	0.218	1.25	0.000633	0.00818
miR−338−5p	0.17	0.126	1.35	0.000759	0.00955
miR−410	0.124	0.0863	1.43	0.000803	0.00986
miR−292−5p	0.134	0.0953	1.41	0.000857	0.0103
miR−26b	0.172	0.129	1.33	0.00106	0.0124
miR−743a	0.251	0.2	1.25	0.00111	0.0127
miR−105	0.162	0.12	1.35	0.00115	0.0128
miR−142−5p	0.23	0.182	1.27	0.00124	0.0133
miR−367	0.136	0.0981	1.38	0.00127	0.0133
miR−466h	0.17	0.128	1.33	0.00127	0.0133
miR−361	0.144	0.106	1.36	0.00141	0.0145
miR−144	0.189	0.146	1.29	0.00184	0.0184
miR−384−5p	0.223	0.178	1.26	0.00203	0.0196
miR−152	0.16	0.121	1.32	0.00204	0.0196
miR−876−3p	0.179	0.138	1.29	0.00226	0.0204
miR−1187	0.21	0.166	1.26	0.00231	0.0204
miR−20a	0.218	0.174	1.26	0.00231	0.0204
miR−467f	0.158	0.12	1.32	0.00232	0.0204
miR−495	0.206	0.163	1.26	0.0024	0.0204
miR−200c	0.189	0.148	1.28	0.00241	0.0204
miR−505	0.136	0.1	1.35	0.00243	0.0204
miR−878−3p	0.127	0.093	1.37	0.00245	0.0204
miR−130b	0.177	0.137	1.29	0.00256	0.0208

↑ CR

Abundance of miRNA Targets

miRNA	Freq(Obs)	Freq(Exp)	Obs/Exp	P−value	P−Value(Adj)
miR−29b	0.163	0.125	1.31	0.00258	0.0208
miR−130a	0.18	0.14	1.29	0.00267	0.0212
miR−706	0.17	0.131	1.29	0.00285	0.0222
miR−181a	0.203	0.161	1.26	0.00293	0.0225
miR−409−3p	0.131	0.0967	1.35	0.00298	0.0225
miR−21	0.117	0.0851	1.37	0.00319	0.0229
miR−466a−5p	0.194	0.154	1.26	0.00322	0.0229
miR−466b−5p	0.194	0.154	1.26	0.00322	0.0229
miR−466e−5p	0.194	0.154	1.26	0.00322	0.0229
miR−1−2−as	0.141	0.106	1.33	0.00327	0.0229
miR−875−3p	0.234	0.19	1.23	0.00343	0.0237
miR−200a	0.191	0.151	1.26	0.00357	0.0242
miR−218	0.144	0.11	1.32	0.00362	0.0242
miR−30e	0.196	0.156	1.26	0.00364	0.0242
miR−199a−5p	0.168	0.131	1.29	0.00371	0.0243
miR−106b	0.179	0.14	1.27	0.0039	0.0249
miR−696	0.0567	0.0358	1.58	0.00391	0.0249
miR−34b−3p	0.0945	0.0668	1.41	0.00397	0.025
miR−141	0.204	0.164	1.25	0.00402	0.025
miR−1	0.179	0.141	1.27	0.00411	0.0252
miR−181c	0.187	0.149	1.26	0.00422	0.0256
miR−431	0.0876	0.0614	1.43	0.00445	0.0266
miR−429	0.189	0.151	1.25	0.0048	0.0284
miR−148a	0.162	0.126	1.28	0.00509	0.0297
miR−466g	0.17	0.134	1.27	0.00514	0.0297
miR−206	0.168	0.133	1.27	0.00562	0.0321
miR−721	0.149	0.116	1.29	0.00602	0.0336
miR−363	0.12	0.0905	1.33	0.00603	0.0336
miR−32	0.136	0.104	1.3	0.0064	0.0349
miR−25	0.125	0.0952	1.32	0.00641	0.0349

↑ CR

Abundance of miRNA Targets

miRNA	Freq(Obs)	Freq(Exp)	Obs/Exp	P−value	P−Value(Adj)
miR−92a	0.107	0.0788	1.35	0.00653	0.0349
miR−30b	0.186	0.149	1.24	0.00662	0.0349
miR−122	0.12	0.0909	1.32	0.00668	0.0349
miR−217	0.155	0.121	1.27	0.00671	0.0349
miR−30a	0.182	0.146	1.25	0.00676	0.0349
miR−20b	0.198	0.16	1.23	0.00689	0.035
miR−139−5p	0.155	0.121	1.27	0.00691	0.035
miR−464	0.162	0.128	1.26	0.00698	0.035
miR−136	0.177	0.142	1.25	0.00752	0.0373
miR−674	0.187	0.152	1.24	0.00776	0.0381
miR−377	0.206	0.169	1.22	0.00812	0.0395
miR−350	0.192	0.157	1.23	0.00825	0.0397
miR−30c	0.189	0.154	1.23	0.00866	0.0411
miR−140	0.137	0.107	1.28	0.00875	0.0411
miR−669d	0.165	0.132	1.25	0.00879	0.0411
miR−369−3p	0.141	0.11	1.28	0.00894	0.0412
miR−669h−3p	0.184	0.149	1.23	0.00898	0.0412
miR−1197	0.156	0.124	1.26	0.00918	0.0417
miR−342−3p	0.141	0.111	1.27	0.00941	0.0424
miR−337−5p	0.0292	0.0167	1.75	0.00956	0.0427
miR−208a	0.0687	0.0481	1.43	0.00976	0.0432
miR−203	0.204	0.169	1.21	0.00993	0.0435
miR−466f	0.124	0.0959	1.29	0.0109	0.0465
miR−295	0.189	0.155	1.22	0.0109	0.0465
miR−199a−3p	0.134	0.105	1.27	0.011	0.0465
miR−199b	0.134	0.105	1.27	0.011	0.0465
miR−687	0.103	0.0779	1.32	0.0111	0.0465
miR−320	0.216	0.181	1.2	0.0113	0.0472
miR−881	0.179	0.146	1.23	0.0115	0.0475
miR−302b	0.203	0.168	1.21	0.0117	0.0477

↑ CR

Abundance of miRNA Targets

miRNA	Freq(Obs)	Freq(Exp)	Obs/Exp	P−value	P−Value(Adj)
miR−297c	0.186	0.152	1.22	0.0119	0.0481
miR−146b	0.149	0.12	1.25	0.0123	0.0496
miR−33	0.125	0.0981	1.28	0.0127	0.0504
miR−872	0.119	0.0921	1.29	0.0127	0.0504
miR−290−5p	0.12	0.0938	1.28	0.0132	0.0511
miR−29c	0.139	0.111	1.26	0.0132	0.0511
miR−489	0.117	0.0907	1.29	0.0133	0.0511
miR−411	0.0722	0.0519	1.39	0.0133	0.0511
miR−871	0.206	0.172	1.2	0.0138	0.0524
miR−653	0.131	0.103	1.27	0.0139	0.0525
miR−381	0.175	0.144	1.22	0.0145	0.0537
miR−466f−3p	0.225	0.19	1.18	0.0146	0.0537
miR−201	0.141	0.113	1.25	0.0147	0.0537
miR−467c	0.0636	0.045	1.41	0.0147	0.0537
miR−669c	0.134	0.107	1.26	0.0148	0.0537
miR−1190	0.143	0.115	1.25	0.0154	0.0556
miR−382	0.155	0.126	1.23	0.0164	0.0586
miR−291a−3p	0.168	0.138	1.22	0.0167	0.0595
miR−499	0.137	0.11	1.25	0.0169	0.0599
miR−683	0.16	0.131	1.22	0.0176	0.0615
miR−200b	0.179	0.148	1.21	0.0177	0.0615
miR−551b	0.0447	0.03	1.49	0.0179	0.0615
miR−7a	0.192	0.161	1.2	0.0179	0.0615
miR−101b	0.144	0.117	1.23	0.0184	0.0624
miR−465a−5p	0.234	0.2	1.17	0.0184	0.0624
miR−494	0.18	0.15	1.2	0.0196	0.0658
miR−22	0.136	0.11	1.24	0.0199	0.0664
miR−294	0.167	0.138	1.21	0.0206	0.0684
miR−669k	0.144	0.118	1.23	0.0209	0.0689
miR−804	0.108	0.0852	1.27	0.0212	0.069

↑ CR

Abundance of miRNA Targets

miRNA	Freq(Obs)	Freq(Exp)	Obs/Exp	P−value	P−Value(Adj)
miR−29a	0.134	0.108	1.24	0.0212	0.069
miR−345−3p	0.127	0.102	1.24	0.0222	0.0717
miR−188−3p	0.129	0.104	1.24	0.0225	0.0724
miR−190	0.168	0.14	1.2	0.023	0.0733
miR−669j	0.127	0.103	1.24	0.0233	0.0737
miR−669b	0.223	0.192	1.16	0.024	0.0754
miR−434−3p	0.0687	0.0511	1.35	0.0242	0.0756
miR−452	0.167	0.139	1.2	0.0247	0.0768
miR−195	0.215	0.185	1.16	0.027	0.0834
miR−883b−3p	0.153	0.127	1.2	0.0282	0.086
miR−466k	0.18	0.153	1.18	0.0282	0.086
miR−323−3p	0.108	0.0867	1.25	0.0291	0.0873
miR−202−5p	0.0842	0.0653	1.29	0.0291	0.0873
miR−181b	0.203	0.174	1.17	0.0292	0.0873
miR−449c	0.136	0.112	1.22	0.0294	0.0875
miR−181d	0.204	0.175	1.17	0.0297	0.0878
miR−669g	0.148	0.123	1.2	0.0303	0.0891
miR−380−3p	0.115	0.0931	1.24	0.0307	0.0898
miR−146a	0.143	0.118	1.2	0.032	0.0925
miR−669e	0.136	0.112	1.21	0.0323	0.0925
miR−212	0.134	0.111	1.21	0.0323	0.0925
miR−185	0.22	0.191	1.15	0.0324	0.0925
miR−684	0.153	0.128	1.19	0.0328	0.0925
miR−467e	0.149	0.125	1.2	0.0328	0.0925
miR−124	0.139	0.115	1.21	0.0331	0.0929
miR−500	0.0773	0.0598	1.29	0.0333	0.0929
miR−1198	0.144	0.12	1.2	0.0335	0.0929
miR−497	0.216	0.188	1.15	0.0344	0.0946
miR−1186	0.153	0.128	1.19	0.0345	0.0946
miR−465b−5p	0.204	0.177	1.16	0.0348	0.095

↑ CR

Abundance of miRNA Targets

miRNA	Freq(Obs)	Freq(Exp)	Obs/Exp	P−value	P−Value(Adj)
miR−132	0.141	0.118	1.2	0.0359	0.0974
miR−322	0.225	0.196	1.15	0.0363	0.0979
miR−574−3p	0.0567	0.0423	1.34	0.0367	0.0986
miR−126−5p	0.16	0.135	1.18	0.0374	0.0995
miR−7b	0.186	0.159	1.16	0.0375	0.0995
miR−878−5p	0.101	0.0818	1.24	0.0383	0.101
miR−10b	0.0911	0.0727	1.25	0.0388	0.102
miR−669a	0.143	0.12	1.19	0.0392	0.102
miR−425	0.0928	0.0744	1.25	0.0404	0.105
miR−469	0.201	0.174	1.15	0.0405	0.105
miR−511	0.16	0.136	1.17	0.0428	0.11
miR−302a	0.187	0.162	1.16	0.043	0.11
miR−466c−5p	0.177	0.152	1.16	0.0433	0.11
miR−693−3p	0.237	0.209	1.13	0.0439	0.11
miR−451	0.0464	0.0341	1.36	0.0441	0.11
miR−374	0.149	0.127	1.18	0.0441	0.11
miR−340−3p	0.0567	0.0429	1.32	0.0443	0.11
miR−101a	0.134	0.113	1.19	0.0457	0.113
miR−291a−5p	0.156	0.133	1.17	0.0461	0.113
miR−291b−5p	0.156	0.133	1.17	0.0461	0.113
miR−654−5p	0.0808	0.0643	1.26	0.0464	0.113
miR−378	0.119	0.0985	1.2	0.0464	0.113
miR−712	0.125	0.105	1.19	0.048	0.116
miR−365	0.079	0.063	1.25	0.0488	0.116
miR−154	0.0928	0.0753	1.23	0.0491	0.116
miR−448	0.149	0.127	1.17	0.0491	0.116
miR−541	0.189	0.164	1.15	0.0491	0.116

↓ CR

Abundance of miRNA Targets

miRNA	Freq(Obs)	Freq(Exp)	Obs/Exp	P−value	P−Value(Adj)
miR−1188	0.154	0.0873	1.76	4.27e−06	0.00209
miR−455	0.146	0.0924	1.58	0.000159	0.0343
miR−331−3p	0.124	0.0772	1.61	0.000343	0.0343
miR−540−5p	0.134	0.086	1.56	0.00044	0.0343
miR−339−3p	0.062	0.0313	1.98	0.000458	0.0343
miR−300	0.161	0.109	1.48	0.000507	0.0343
miR−124	0.169	0.115	1.46	0.000535	0.0343
miR−298	0.273	0.207	1.32	0.000559	0.0343
miR−878−5p	0.127	0.0818	1.55	0.00074	0.0382
miR−324−5p	0.102	0.0622	1.63	0.00078	0.0382
miR−483	0.107	0.0668	1.6	0.000942	0.042
miR−23a	0.194	0.142	1.36	0.00159	0.0569
miR−762	0.208	0.155	1.34	0.00163	0.0569
miR−185	0.248	0.191	1.3	0.00168	0.0569
miR−709	0.231	0.175	1.32	0.00182	0.0569
miR−743a	0.258	0.2	1.29	0.00186	0.0569
miR−532−3p	0.141	0.0988	1.43	0.00233	0.0605
miR−700	0.0298	0.0129	2.31	0.00236	0.0605
miR−369−5p	0.0372	0.0178	2.1	0.00252	0.0605
miR−770−5p	0.0968	0.0624	1.55	0.00261	0.0605
miR−679	0.139	0.0972	1.43	0.00262	0.0605
miR−149	0.184	0.136	1.35	0.00275	0.0605
miR−485	0.174	0.127	1.36	0.00284	0.0605
miR−365	0.0968	0.063	1.54	0.00309	0.0631
miR−327	0.129	0.09	1.43	0.00343	0.0644
miR−491	0.141	0.101	1.4	0.00372	0.0644
miR−330	0.196	0.149	1.32	0.00372	0.0644
miR−486	0.0943	0.0617	1.53	0.00372	0.0644
miR−339−5p	0.144	0.103	1.39	0.00381	0.0644
miR−761	0.226	0.176	1.28	0.00415	0.0653

↓ CR

Abundance of miRNA Targets

miRNA	Freq(Obs)	Freq(Exp)	Obs/Exp	P−value	P−Value(Adj)
miR−184	0.0794	0.0504	1.58	0.00447	0.0653
miR−883a−3p	0.171	0.128	1.34	0.00449	0.0653
miR−760	0.176	0.132	1.33	0.00457	0.0653
miR−18a	0.166	0.123	1.35	0.00463	0.0653
miR−764−5p	0.104	0.0706	1.48	0.00466	0.0653
miR−335−5p	0.186	0.141	1.32	0.00484	0.0658
miR−214	0.228	0.179	1.27	0.00496	0.0658
miR−741	0.176	0.133	1.33	0.00527	0.0679
miR−710	0.181	0.138	1.32	0.00559	0.069
miR−879	0.132	0.0944	1.39	0.00564	0.069
miR−22	0.149	0.11	1.36	0.00584	0.0698
miR−592	0.136	0.0992	1.38	0.0063	0.0735
miR−704	0.112	0.0787	1.42	0.00713	0.0798
miR−125a−3p	0.179	0.137	1.3	0.00719	0.0798
miR−31	0.149	0.111	1.34	0.00733	0.0798
miR−324−3p	0.117	0.0831	1.4	0.00755	0.0804
miR−484	0.141	0.105	1.35	0.00779	0.0812
miR−23b	0.184	0.142	1.29	0.00847	0.0865
miR−883b−3p	0.166	0.127	1.31	0.00895	0.0895
miR−688	0.0546	0.0334	1.64	0.00936	0.0903
miR−743b−5p	0.144	0.108	1.34	0.0094	0.0903
miR−325	0.166	0.128	1.3	0.0103	0.0947
miR−684	0.166	0.128	1.3	0.0104	0.0947
miR−705	0.179	0.14	1.28	0.0111	0.0947
miR−331−5p	0.0943	0.0661	1.43	0.0111	0.0947
miR−742	0.208	0.167	1.25	0.0116	0.0947
miR−96	0.151	0.116	1.31	0.0119	0.0947
miR−126−3p	0.0174	0.00755	2.3	0.0119	0.0947
miR−27b	0.199	0.158	1.26	0.0119	0.0947
miR−125b−5p	0.124	0.0919	1.35	0.0122	0.0947

↓ CR

Abundance of miRNA Targets

miRNA	Freq(Obs)	Freq(Exp)	Obs/Exp	P−value	P−Value(Adj)
miR−145	0.179	0.14	1.27	0.0123	0.0947
miR−706	0.169	0.131	1.28	0.0124	0.0947
miR−9	0.218	0.176	1.24	0.0125	0.0947
miR−582−5p	0.156	0.12	1.3	0.0126	0.0947
miR−680	0.134	0.101	1.33	0.0126	0.0947
miR−351	0.156	0.12	1.3	0.0128	0.0947
let−7g	0.159	0.123	1.29	0.0129	0.0947
miR−877	0.141	0.108	1.31	0.014	0.101
miR−202−5p	0.0918	0.0653	1.41	0.0149	0.105
miR−203	0.208	0.169	1.24	0.0154	0.106
miR−409−5p	0.067	0.0449	1.49	0.0156	0.106
miR−338−3p	0.159	0.124	1.28	0.0156	0.106
miR−342−5p	0.102	0.0741	1.37	0.016	0.108
miR−99b	0.0248	0.013	1.91	0.0168	0.11
miR−290−5p	0.124	0.0938	1.32	0.0172	0.11
miR−133a	0.0968	0.0702	1.38	0.0174	0.11
miR−133b	0.0968	0.0702	1.38	0.0174	0.11
miR−376a	0.0372	0.022	1.69	0.0175	0.11
miR−449c	0.144	0.112	1.29	0.0184	0.114
miR−1	0.176	0.141	1.25	0.0187	0.115
miR−670	0.134	0.103	1.3	0.0191	0.115
miR−453	0.104	0.0771	1.35	0.0192	0.115
miR−297c	0.189	0.152	1.24	0.0196	0.116
miR−882	0.199	0.162	1.23	0.0204	0.117
miR−1190	0.146	0.115	1.28	0.0205	0.117
miR−141	0.201	0.164	1.23	0.0205	0.117
miR−362−3p	0.127	0.0971	1.3	0.0211	0.119
miR−18b	0.149	0.117	1.27	0.0218	0.121
miR−26b	0.161	0.129	1.25	0.0236	0.128
miR−468	0.127	0.0977	1.29	0.0236	0.128

↓ CR

Abundance of miRNA Targets

miRNA	Freq(Obs)	Freq(Exp)	Obs/Exp	P−value	P−Value(Adj)
miR−103	0.179	0.145	1.23	0.0256	0.133
miR−107	0.179	0.145	1.23	0.0256	0.133
miR−699	0.127	0.0982	1.29	0.0257	0.133
miR−296−3p	0.156	0.125	1.25	0.0259	0.133
miR−804	0.112	0.0852	1.31	0.0259	0.133
miR−504	0.102	0.0765	1.33	0.026	0.133
miR−873	0.154	0.123	1.25	0.0265	0.134
miR−27a	0.189	0.155	1.22	0.027	0.135
miR−872	0.119	0.0921	1.29	0.0274	0.136
miR−295	0.189	0.155	1.22	0.0285	0.139
miR−654−5p	0.0868	0.0643	1.35	0.0292	0.142
miR−208b	0.067	0.0475	1.41	0.0297	0.143
miR−470	0.159	0.128	1.24	0.0303	0.144
let−7i	0.154	0.124	1.24	0.0308	0.145
miR−292−3p	0.124	0.0974	1.27	0.0321	0.147
miR−433	0.0943	0.0712	1.32	0.0322	0.147
miR−125a−5p	0.132	0.104	1.26	0.0323	0.147
miR−693−3p	0.246	0.209	1.17	0.0323	0.147
miR−490	0.107	0.0822	1.3	0.0331	0.149
miR−106b	0.171	0.14	1.22	0.0341	0.152
miR−34a	0.169	0.138	1.22	0.0347	0.153
miR−291a−3p	0.169	0.138	1.22	0.0352	0.154
miR−770−3p	0.0844	0.0634	1.33	0.037	0.159
miR−466i	0.184	0.153	1.2	0.0371	0.159
miR−717	0.171	0.141	1.21	0.0377	0.16
miR−763	0.176	0.146	1.21	0.038	0.16
miR−186	0.241	0.206	1.17	0.0381	0.16
miR−329	0.117	0.092	1.27	0.0386	0.16
miR−541	0.196	0.164	1.19	0.0389	0.16
miR−449b	0.151	0.123	1.23	0.0393	0.16

↓ CR

Abundance of miRNA Targets

miRNA	Freq(Obs)	Freq(Exp)	Obs/Exp	P−value	P−Value(Adj)
miR−540−3p	0.104	0.0811	1.29	0.0396	0.16
miR−874	0.117	0.0922	1.27	0.0399	0.16
miR−206	0.161	0.133	1.21	0.0417	0.166
miR−148b	0.156	0.129	1.22	0.0427	0.169
miR−467c	0.062	0.045	1.38	0.0431	0.169
miR−871	0.203	0.172	1.18	0.0433	0.169
miR−665	0.171	0.143	1.2	0.0448	0.173
miR−7a	0.191	0.161	1.19	0.0452	0.173
miR−188−5p	0.0993	0.0777	1.28	0.0469	0.178
miR−216a	0.134	0.109	1.23	0.0477	0.178
miR−134	0.114	0.0911	1.25	0.0477	0.178
miR−466g	0.161	0.134	1.2	0.0482	0.178
miR−337−3p	0.127	0.102	1.24	0.0482	0.178
miR−467d	0.0521	0.0373	1.4	0.049	0.179
miR−880	0.114	0.0913	1.25	0.0492	0.179

Tests for Chromosome Over−representation

Chromosome	CR−upregulated Genes	CR−downregulated Genes
1	0.227	0.935
2	0.764	0.843
3	0.262	0.331
4	0.812	0.108
5	0.741	0.798
6	0.609	0.252
7	0.727	0.858
8	0.784	0.226
9	0.227	0.557
10	0.105	0.698
11	0.459	0.00767*
12	0.189	0.584
13	0.551	0.475
14	0.556	0.437
15	0.364	0.689
16	0.0703	0.454
17	0.566	0.0122*
18	0.633	0.286
19	0.234	0.0958
X	0.529	0.999
Y	1.00	0.0841

The table lists p−values generated from a test that evaluates whether there exists an over−abundance of identified genes with respect to a given chromosome. The null hypothesis assumes that the set of genes has been selected at random from those represented on the Affymetrix 430 2.0 array. A significant test indicates that a chromosome contains more of the identified genes than would be expected if the gene set had been chosen at random.

* = significant p−value, without multiple test adjustment

** = significant p−value, with multiple test adjustment

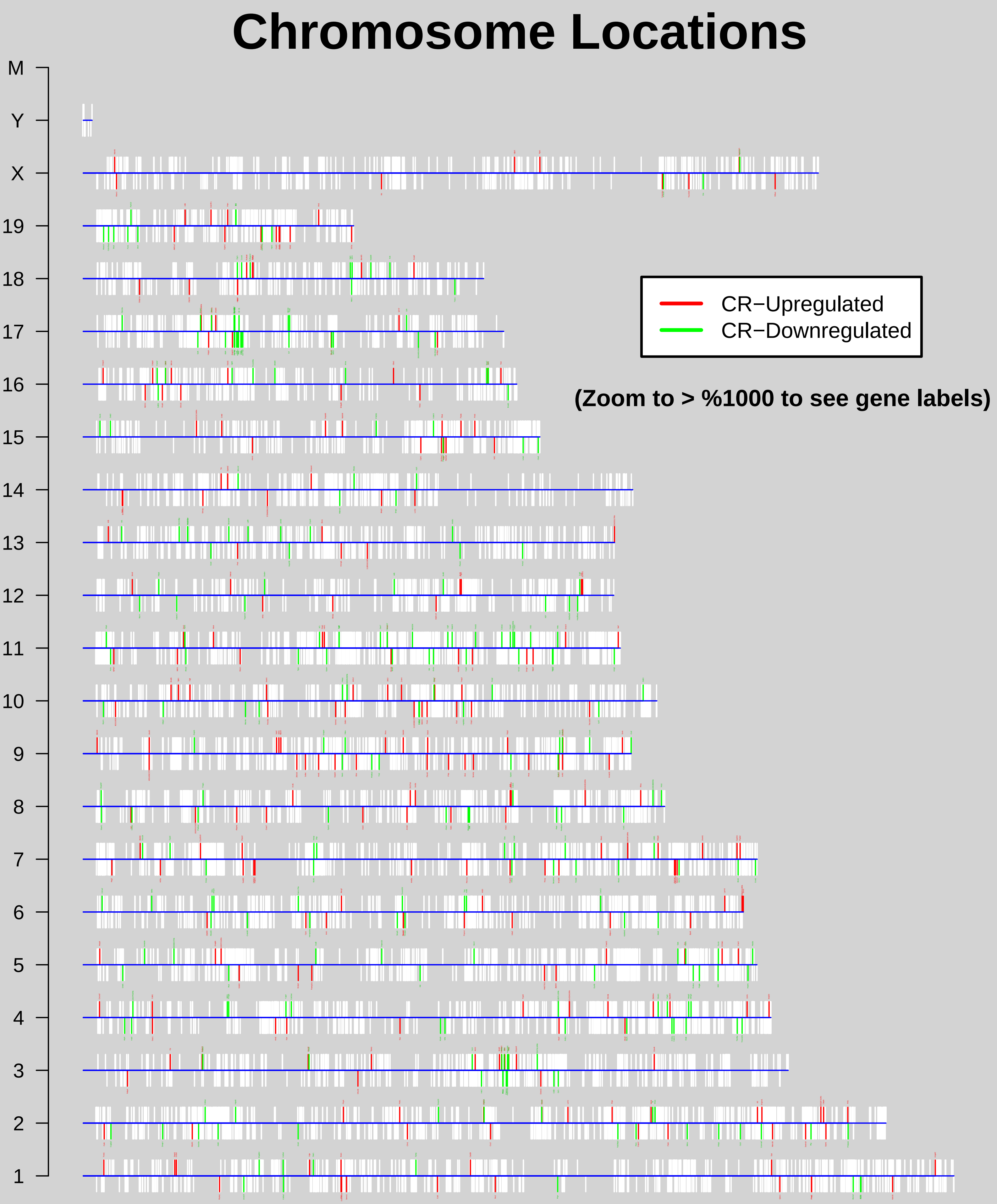
Chromosome Locations
M
Y
X
19
18
17
16
15
14
13
12
11
10
9
8
7
6
5
4
3
2
1
CR−Upregulated
CR−Downregulated
(Zoom to > %1000 to see gene labels)
