# Supplementary material for: Genes and gene expression modules associated with caloric restriction and aging in the laboratory mouse
Source: BMC Genomics. 2009 Dec 7;10:585. doi: 10.1186/1471-2164-10-585 (PMC2795771; doi:10.1186/1471-2164-10-585)

Additional File 6

Genes and Gene Expression Modules Associated with Caloric Restriction and Aging in the Laboratory Mouse

William R. Swindell
University of Michigan, Departments of Pathology and Geriatrics

---

Gene Expression Modules Regulated by CR

This file provides a description of gene expression modules regulated by CR in multiple tissue types. Gene expression modules of varying size are displayed, including those with 2, 3, 5, 10, 20 and 40 member genes. The value of *M* associated with each module is directly related to the overall responsiveness of member genes to CR across tissue types (see Methods). The p-value associated with each value of *M* is generated by a simulation analysis, in which modules of the same size are formed at random, without reference to observed co-expression patterns (see Methods).

A dendrogram is shown for each module, which represents the co-expression patterns of member genes. This was generated based upon an average linkage hierarchical cluster analysis, in which similarity between genes was based upon the absolute value of Pearson's correlation coefficient (*r*) (see Methods). Additionally, for each module, a grid is shown that displays the differential expression patterns associated with member genes. Symbols have the following interpretation.

● Gene is significantly up regulated by CR ($P_u < 0.05$)
● Gene is significantly down regulated by CR ($P_d < 0.05$)
— Gene is marginally up regulated by CR ($0.05 < P_u < 0.10$)
— Gene is marginally down regulated by CR ($0.05 < P_d < 0.10$)
— Non-significant CR effect ($P_u > 0.10$ and $P_d > 0.10$)
× No data (gene not represented for a given tissue or array annotation was limiting)
✳ Evidence conflicts, but favors up regulation by CR
✳ Evidence conflicts, but favors down regulation by CR

The last two categories (✳ and ✳) indicate significant effects with conflicting evidence. This can arise if there is significant up regulation by CR in one experiment, and significant down regulation by CR in another experiment that has examined the same tissue. Alternatively, a conflict may arise if $P_u < 0.05$ and also $P_d < 0.05$ for a given tissue type. Symbols shown in charts are based upon a comparison-wise type I error rate of 0.05.

Contact: William R. Swindell, wswindel@umich.edu

CR−Regulated Modules (2 Genes)

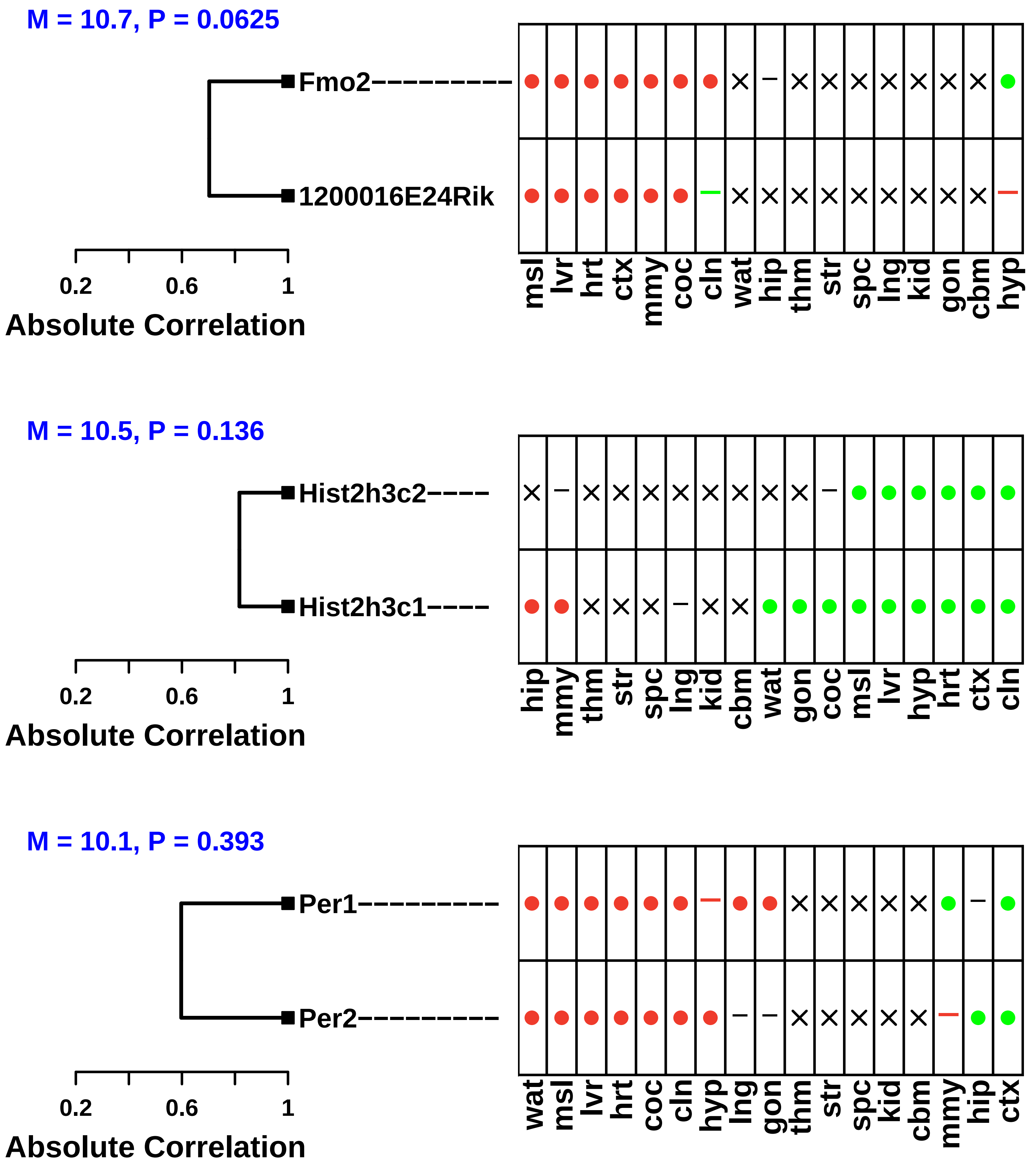

CR−Regulated Modules (2 Genes)

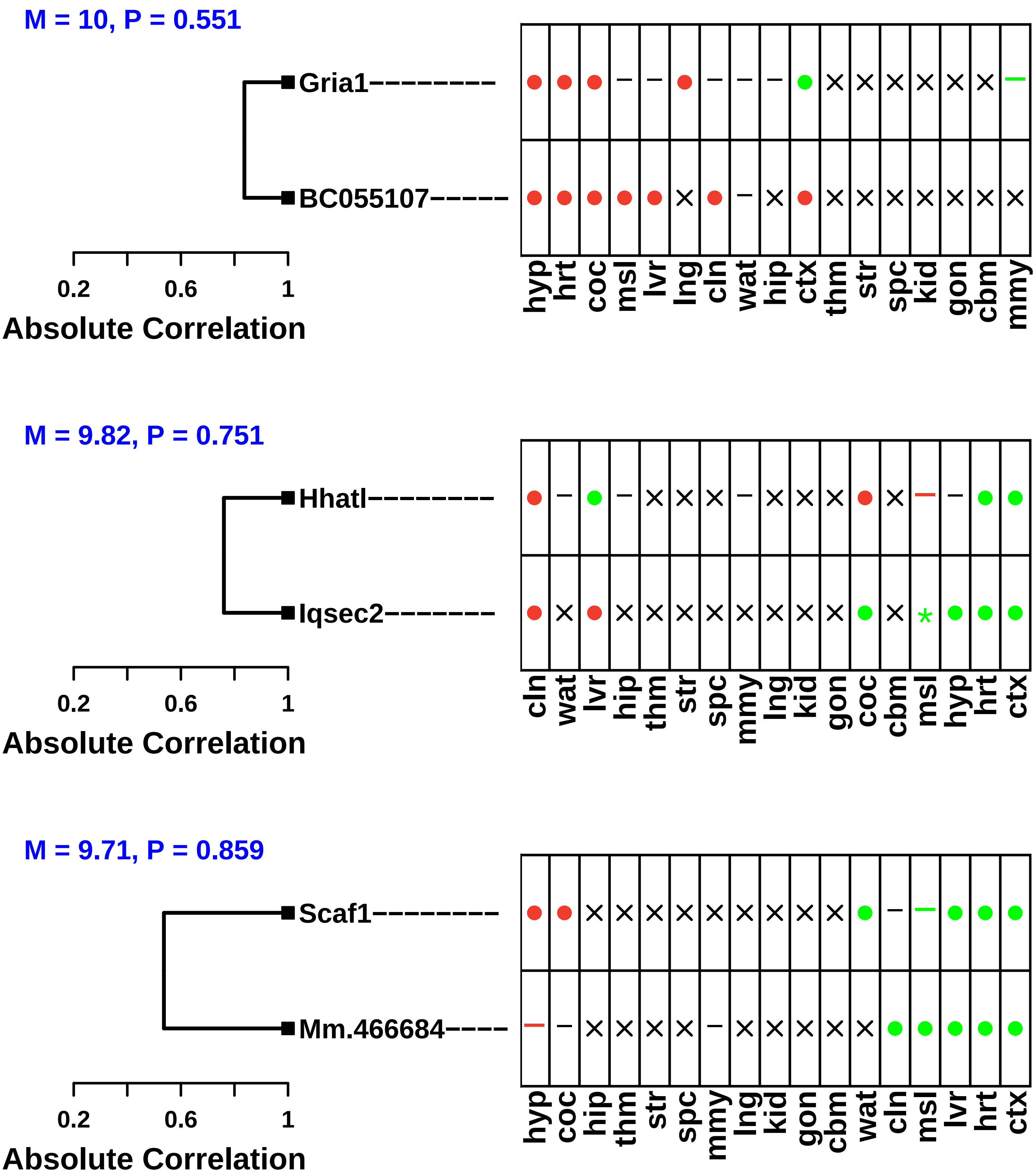

CR−Regulated Modules (2 Genes)

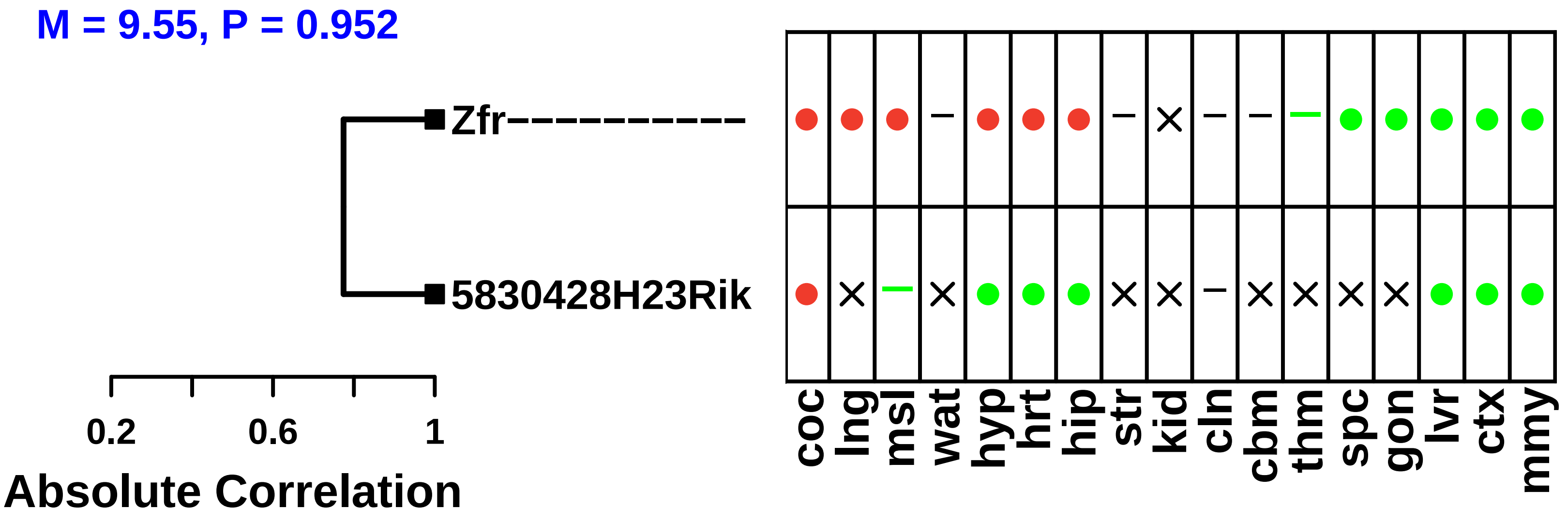

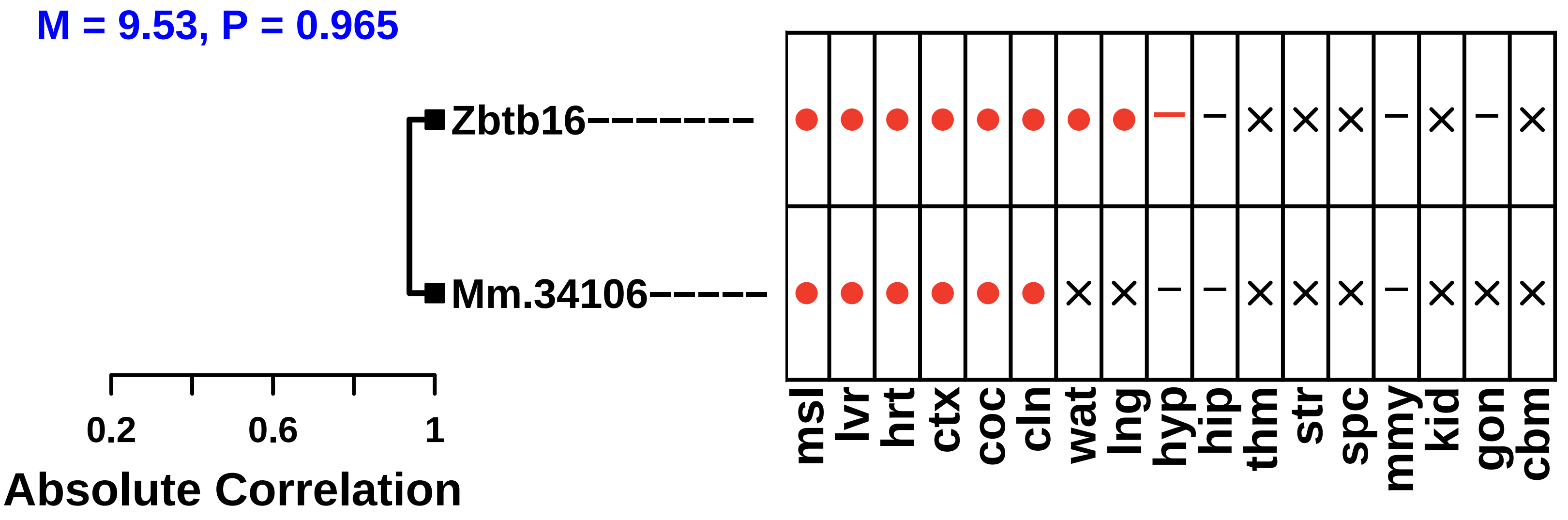

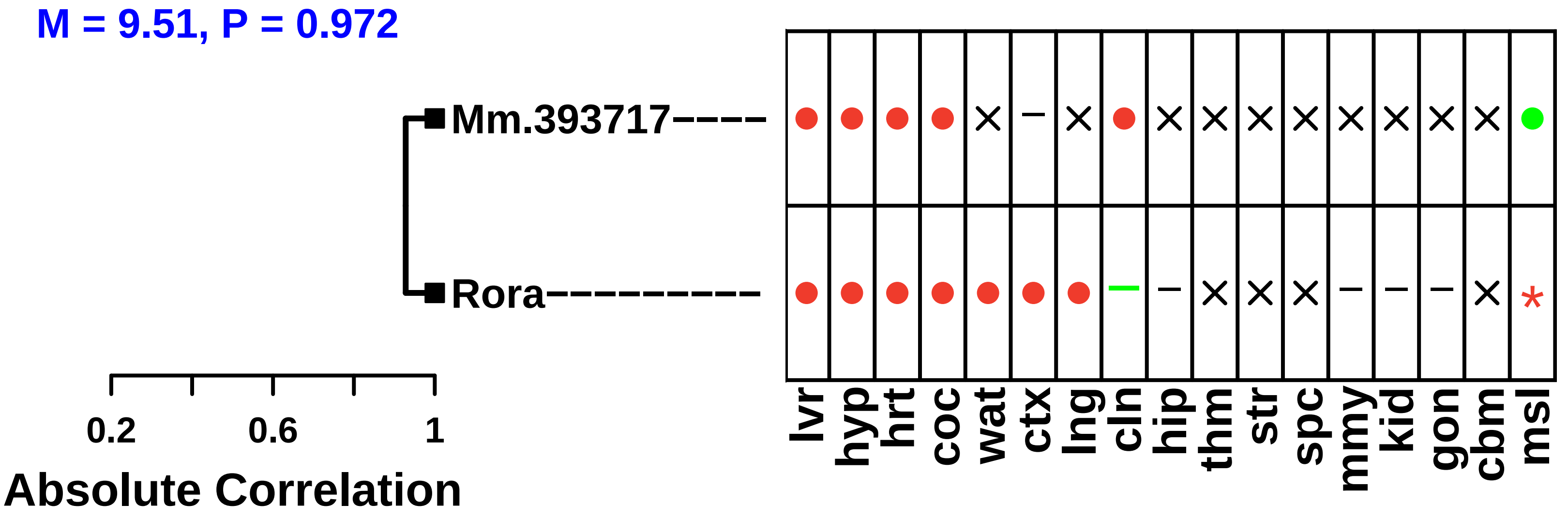

CR−Regulated Modules (2 Genes)

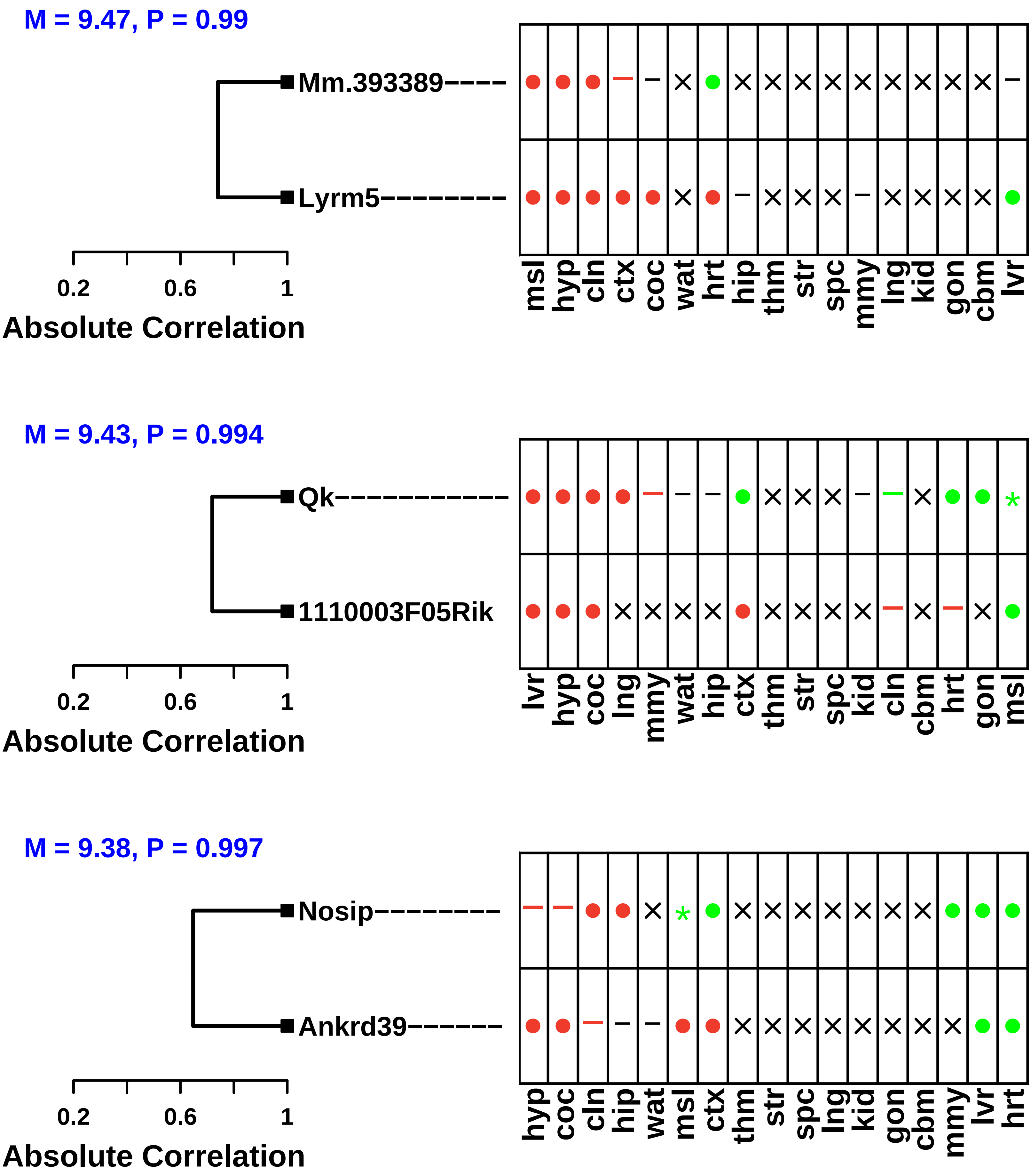

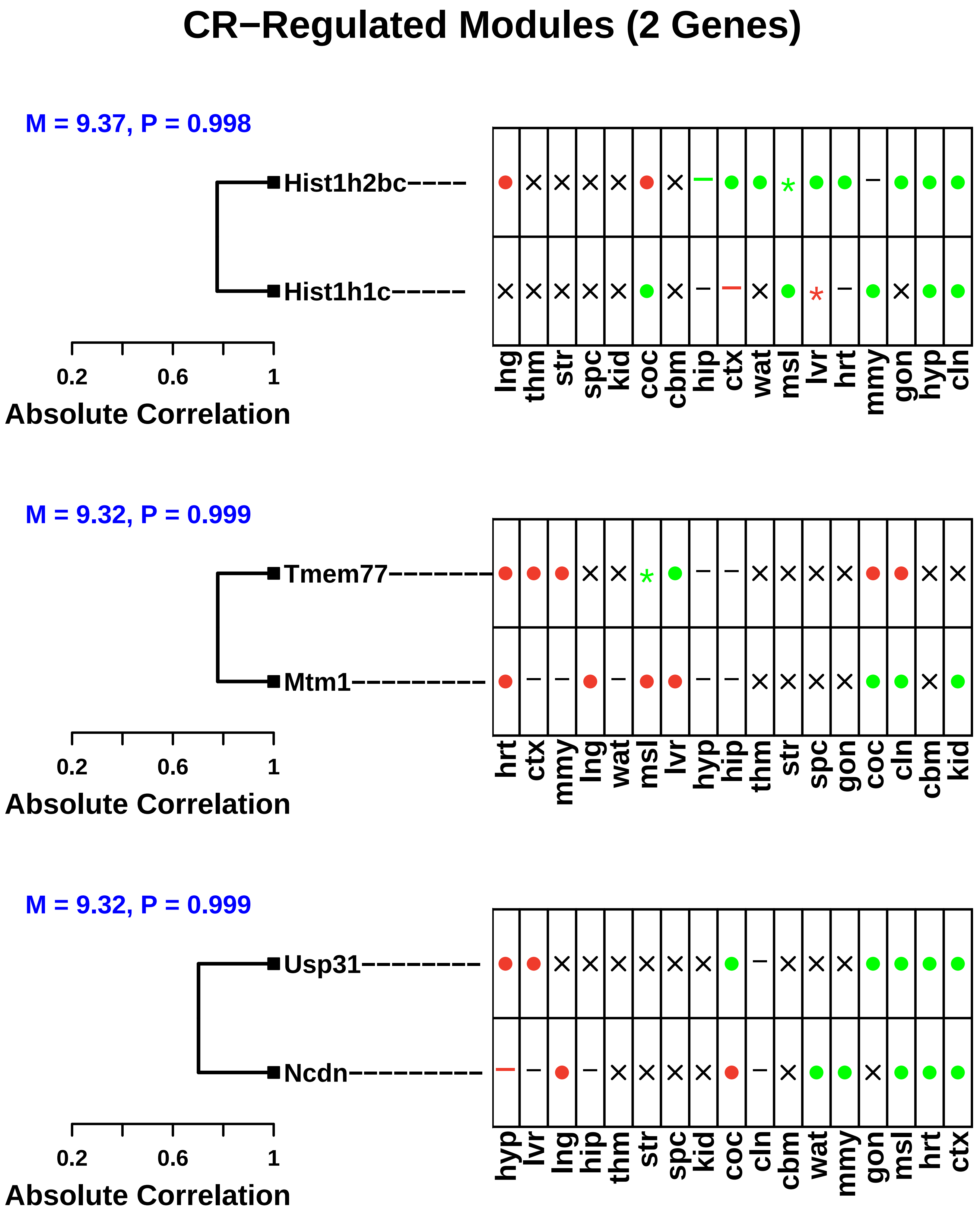
CR−Regulated Modules (2 Genes)
M = 9.37, P = 0.998
Hist1h2bc
Hist1h1c
0.2
0.6
1
Absolute Correlation
lng thm str spc kid coc cbm hip ctx wat msl lvr hrt mmy gon hyp cln
M = 9.32, P = 0.999
Tmem77
Mtm1
0.2
0.6
1
Absolute Correlation
hrt ctx mmy lng wat msl lvr hyp hip thm str spc gon coc cln cbm kid
M = 9.32, P = 0.999
Usp31
Ncdn
0.2
0.6
1
Absolute Correlation
hyp lvr lng hip thm str spc kid coc cln cbm wat mmy gon msl hrt ctx

CR−Regulated Modules (2 Genes)

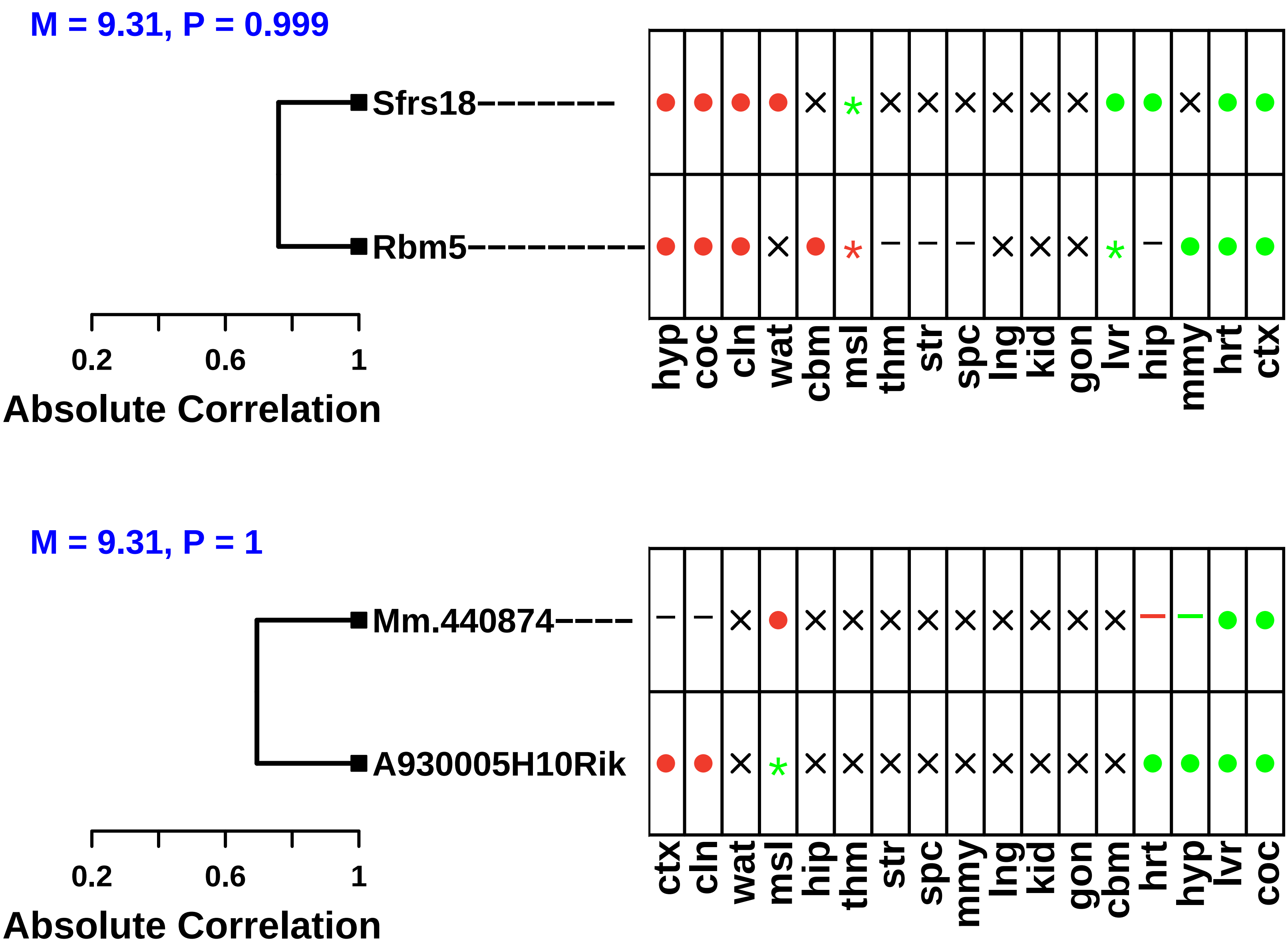

CR−Regulated Modules (3 Genes)

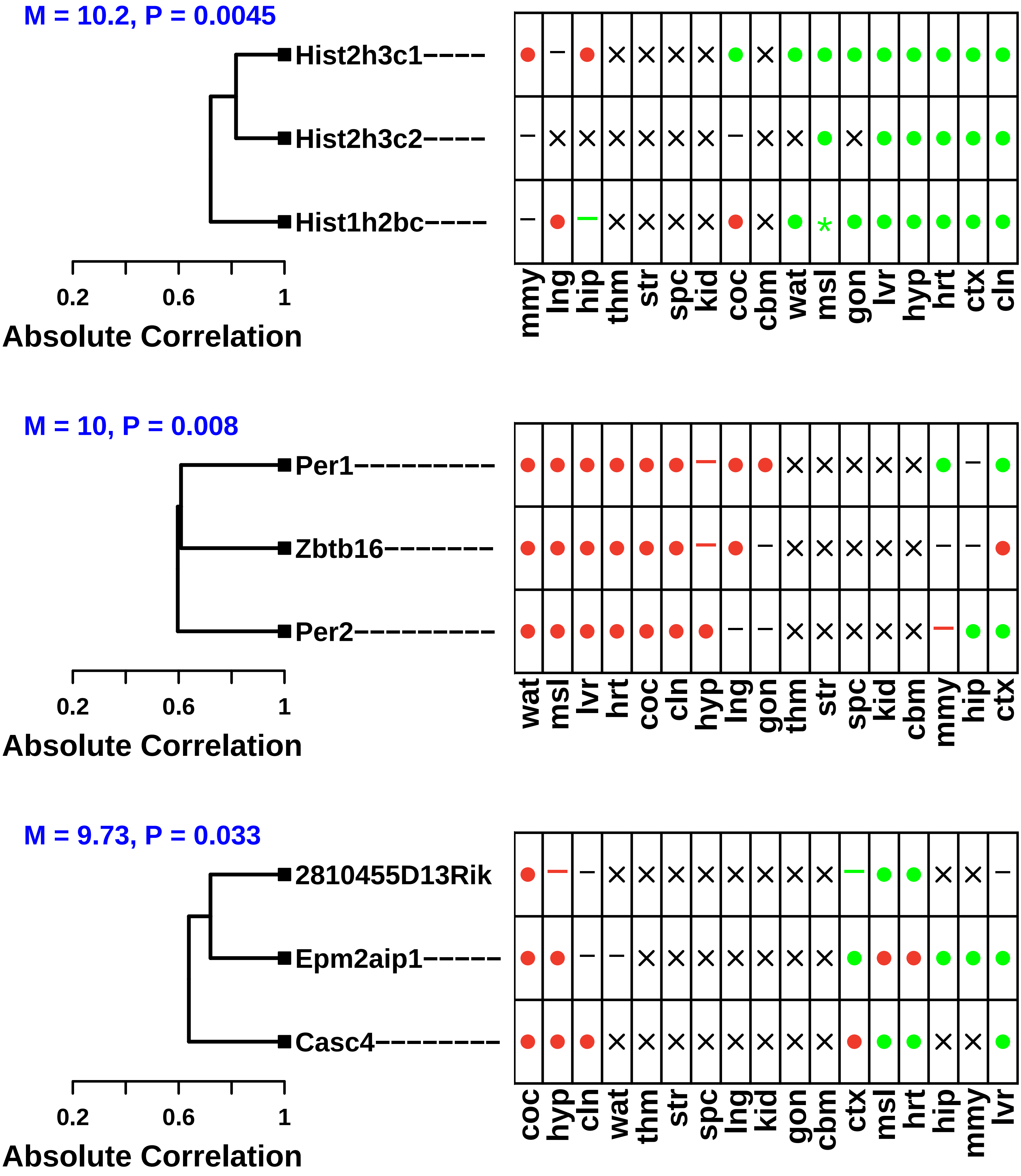

CR−Regulated Modules (3 Genes)

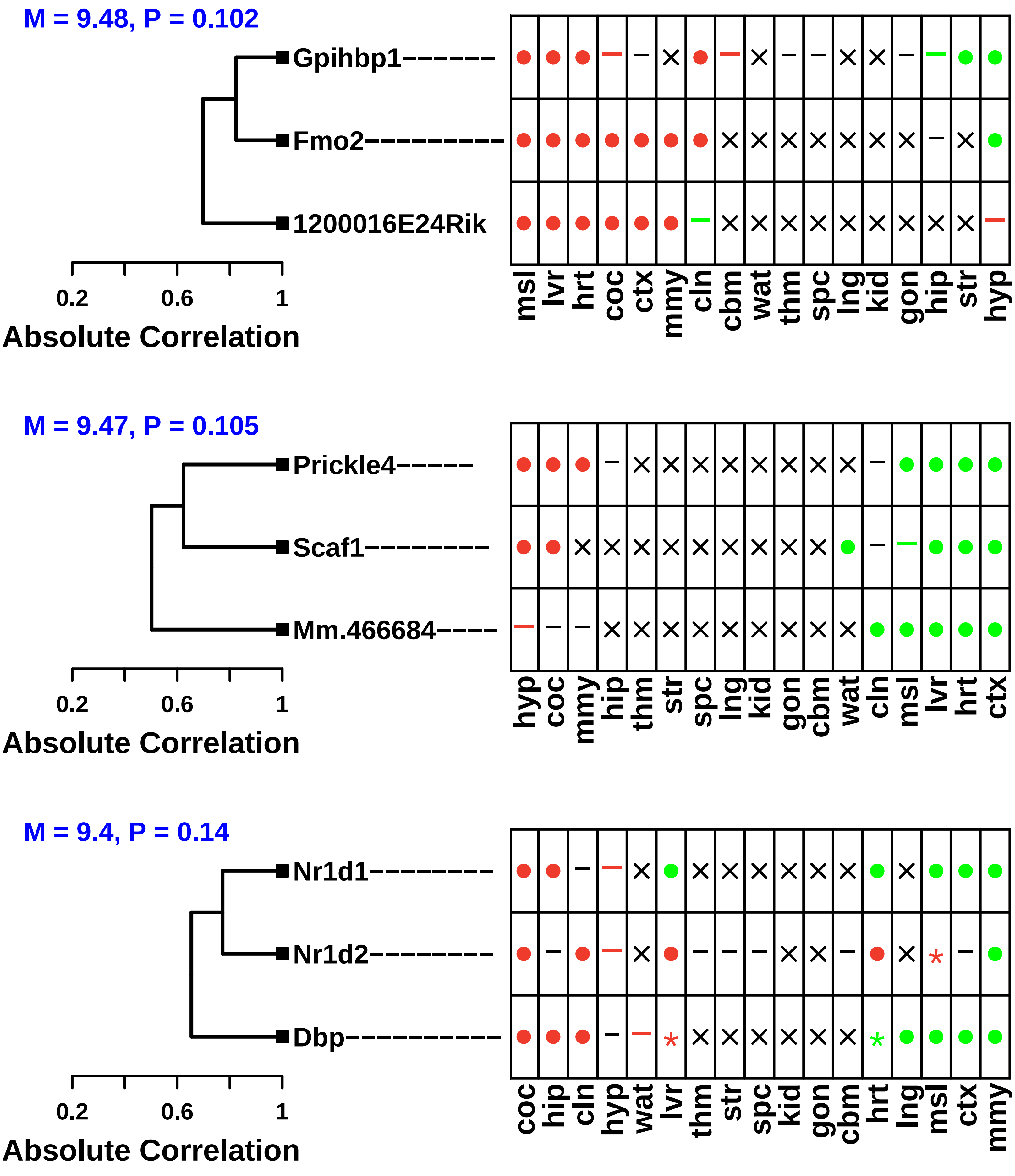

CR−Regulated Modules (5 Genes)

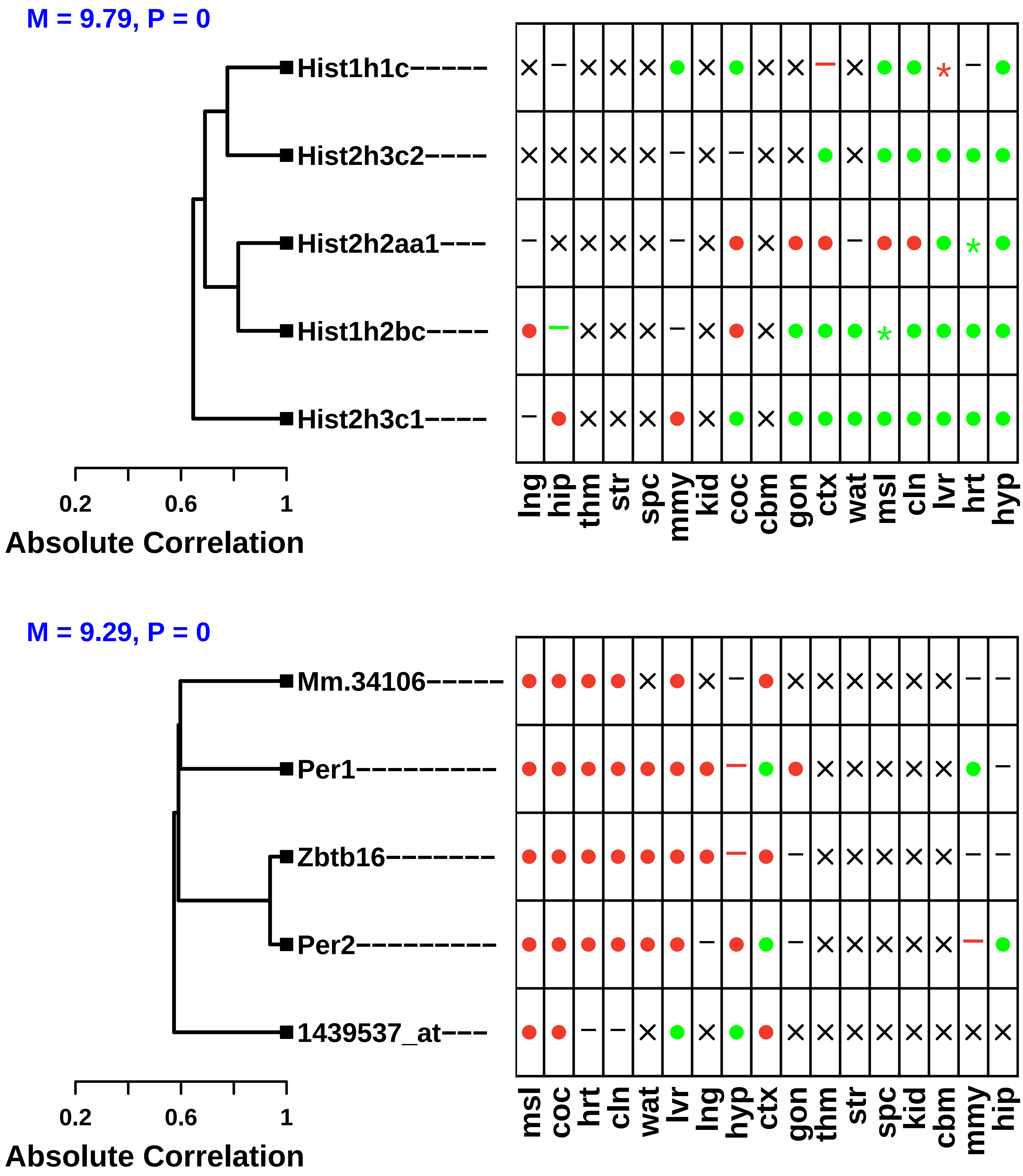

CR−Regulated Modules (5 Genes)

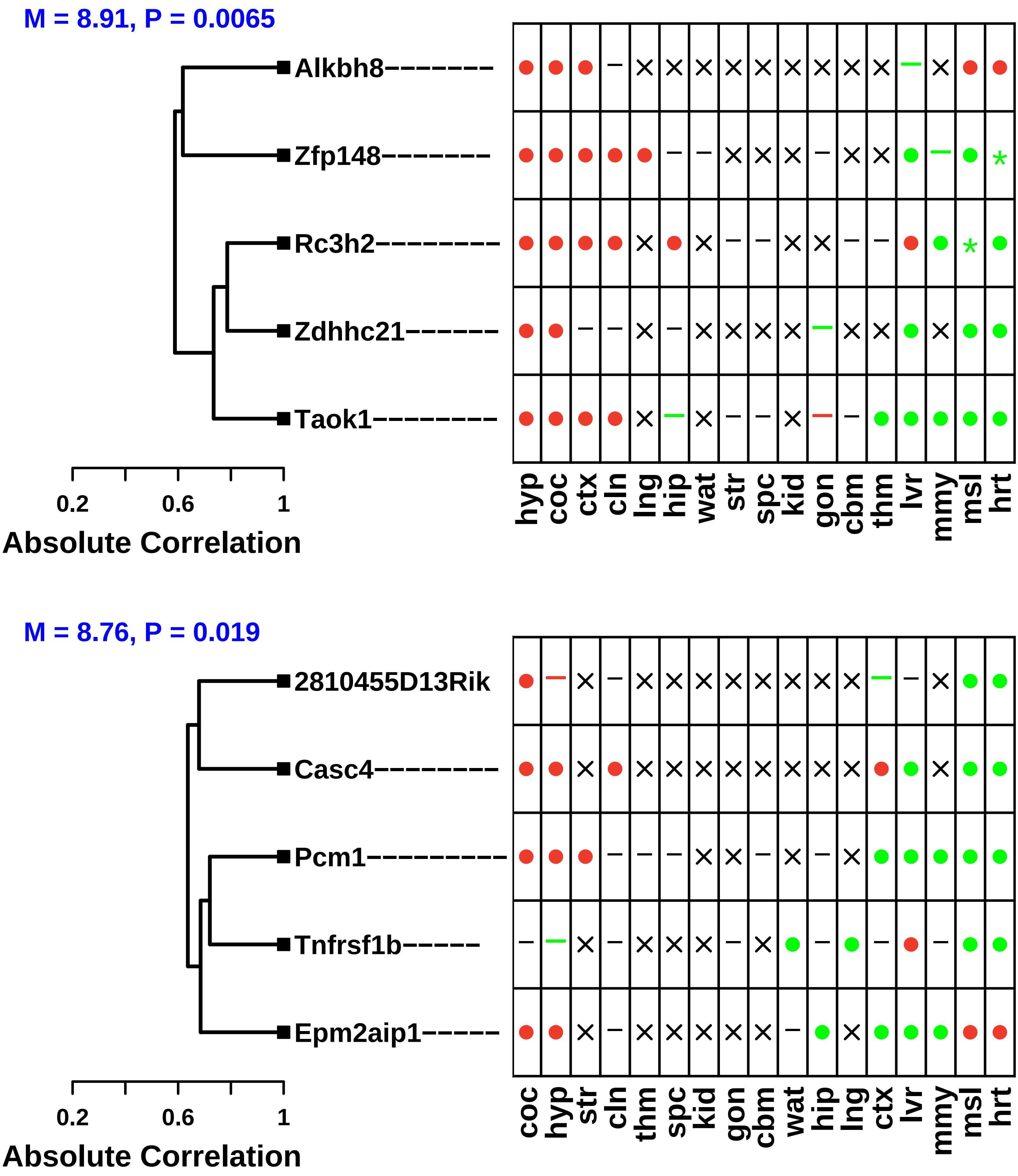

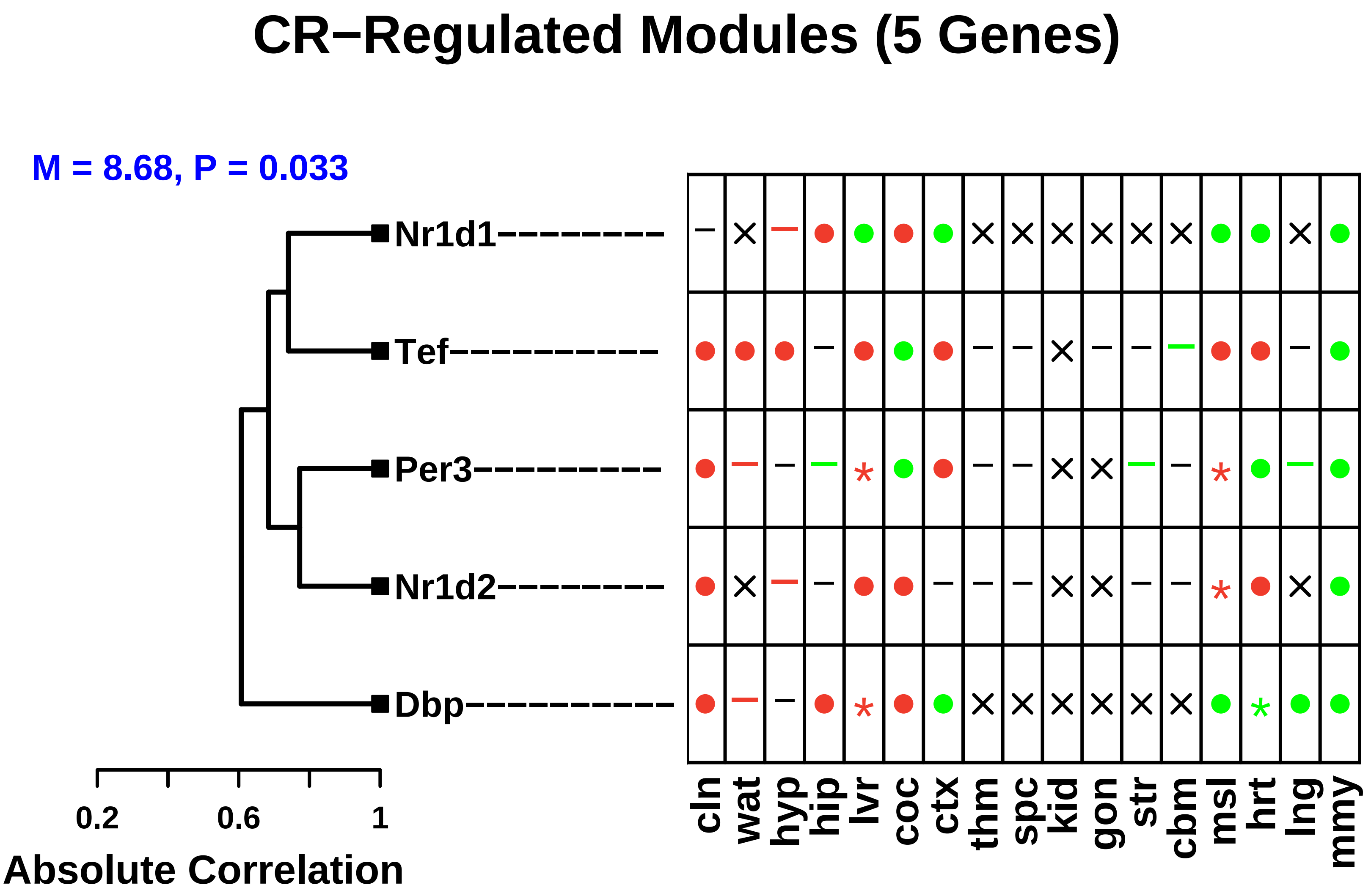

CR−Regulated Modules (5 Genes)
M = 8.68, P = 0.033
Nr1d1
Tef
Per3
Nr1d2
Dbp
0.2
0.6
1
Absolute Correlation
cln
wat
hyp
hip
lvr
coc
ctx
thm
spc
kid
gon
str
cbm
msl
hrt
lng
mmy

CR−Regulated Modules (10 Genes)

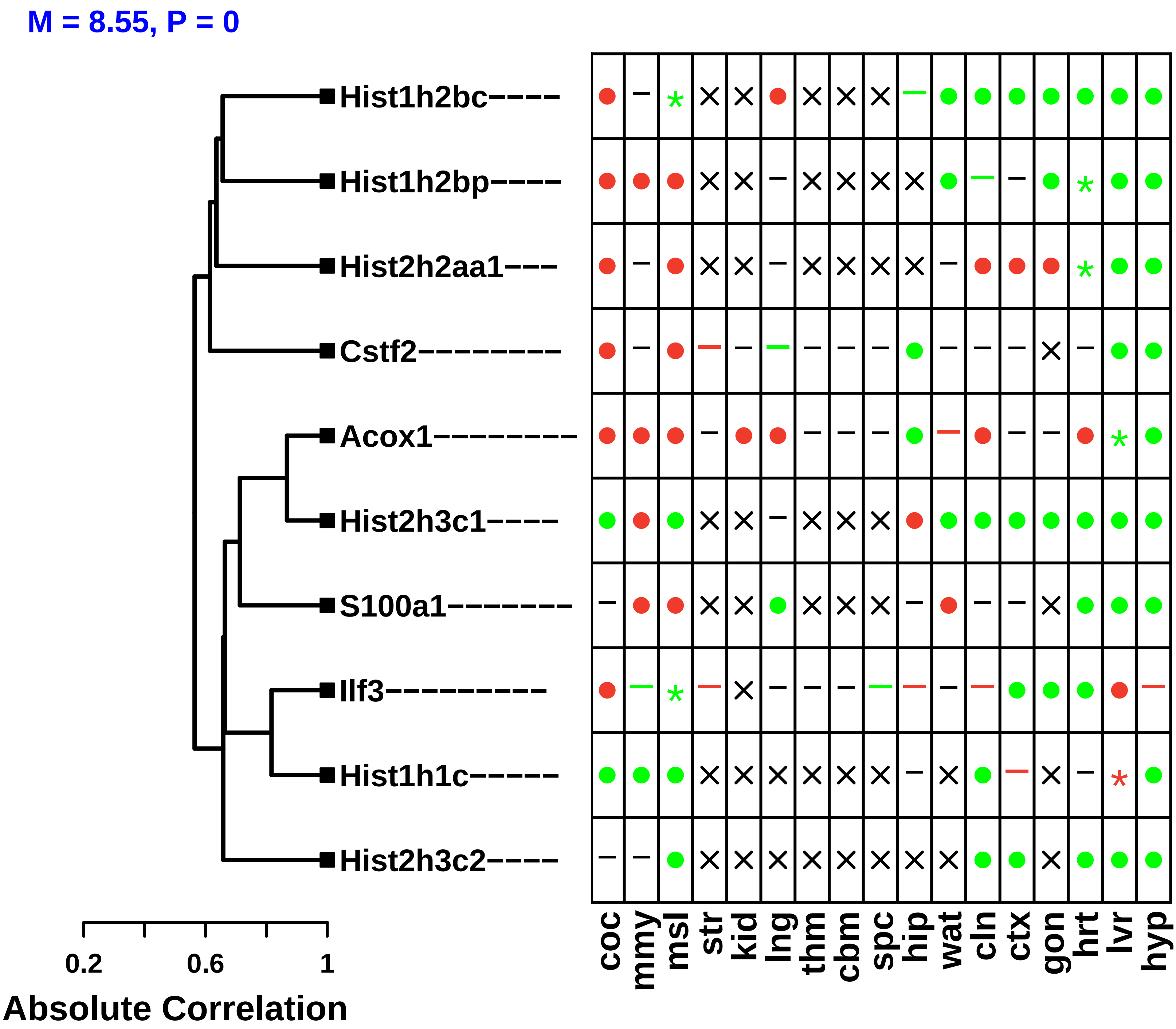

CR−Regulated Modules (10 Genes)

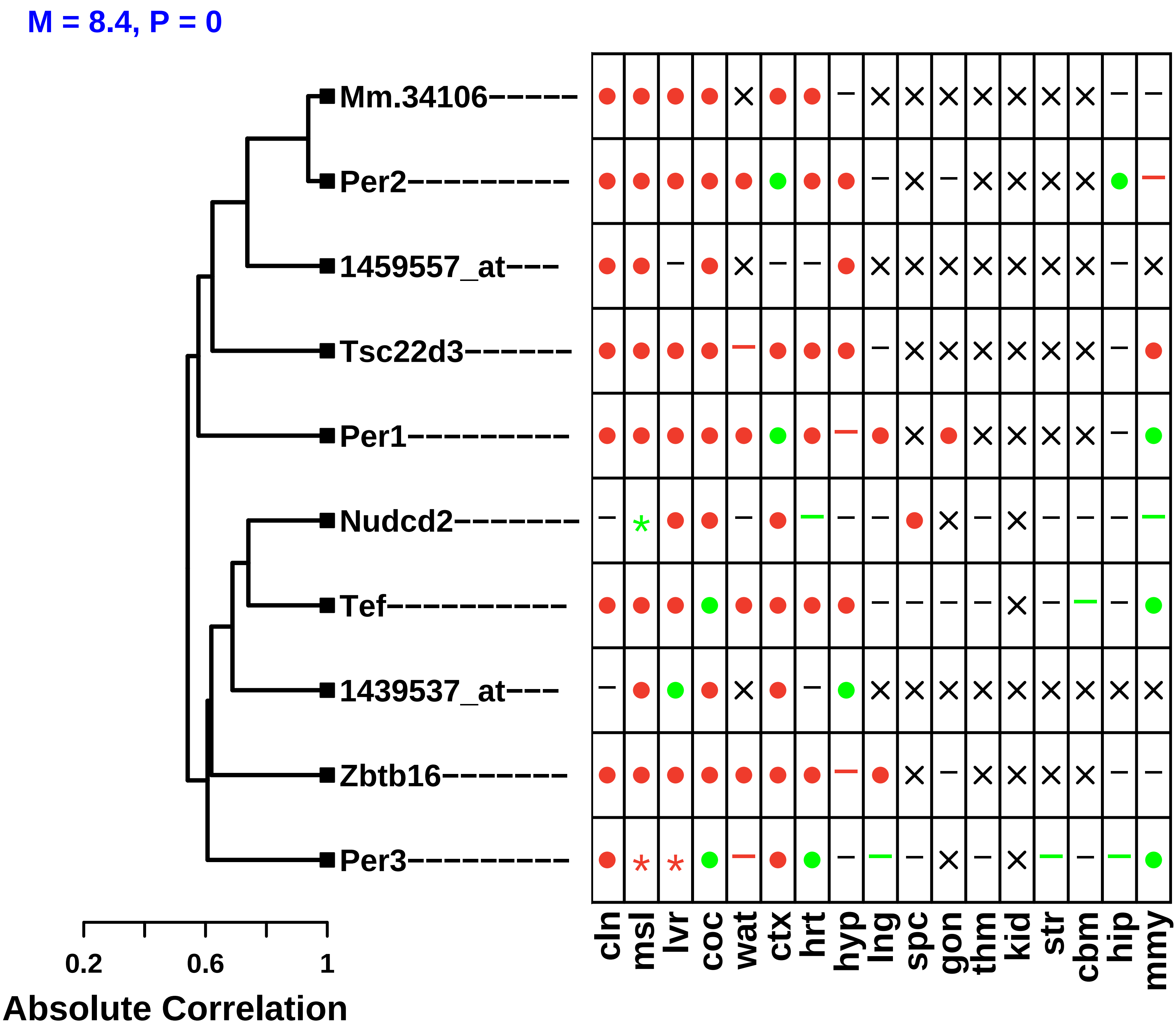

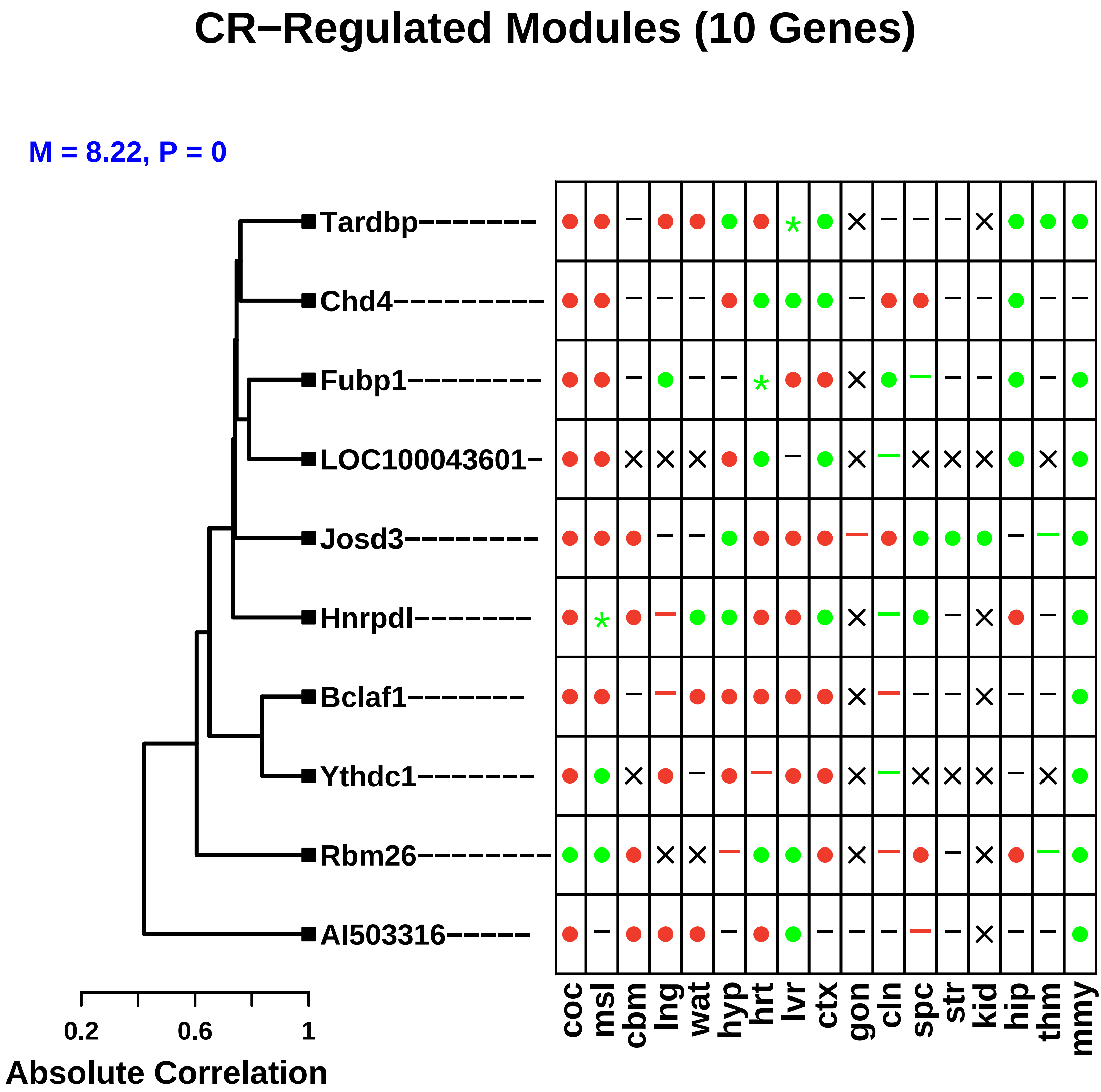

CR−Regulated Modules (10 Genes)
M = 8.22, P = 0
Tardbp
Chd4
Fubp1
LOC100043601
Josd3
Hnrpdl
Bclaf1
Ythdc1
Rbm26
AI503316
0.2
0.6
1
Absolute Correlation
coc
msl
cbm
lng
wat
hyp
hrt
lvr
ctx
gon
cln
spc
str
kid
hip
thm
mmy

CR−Regulated Modules (10 Genes)

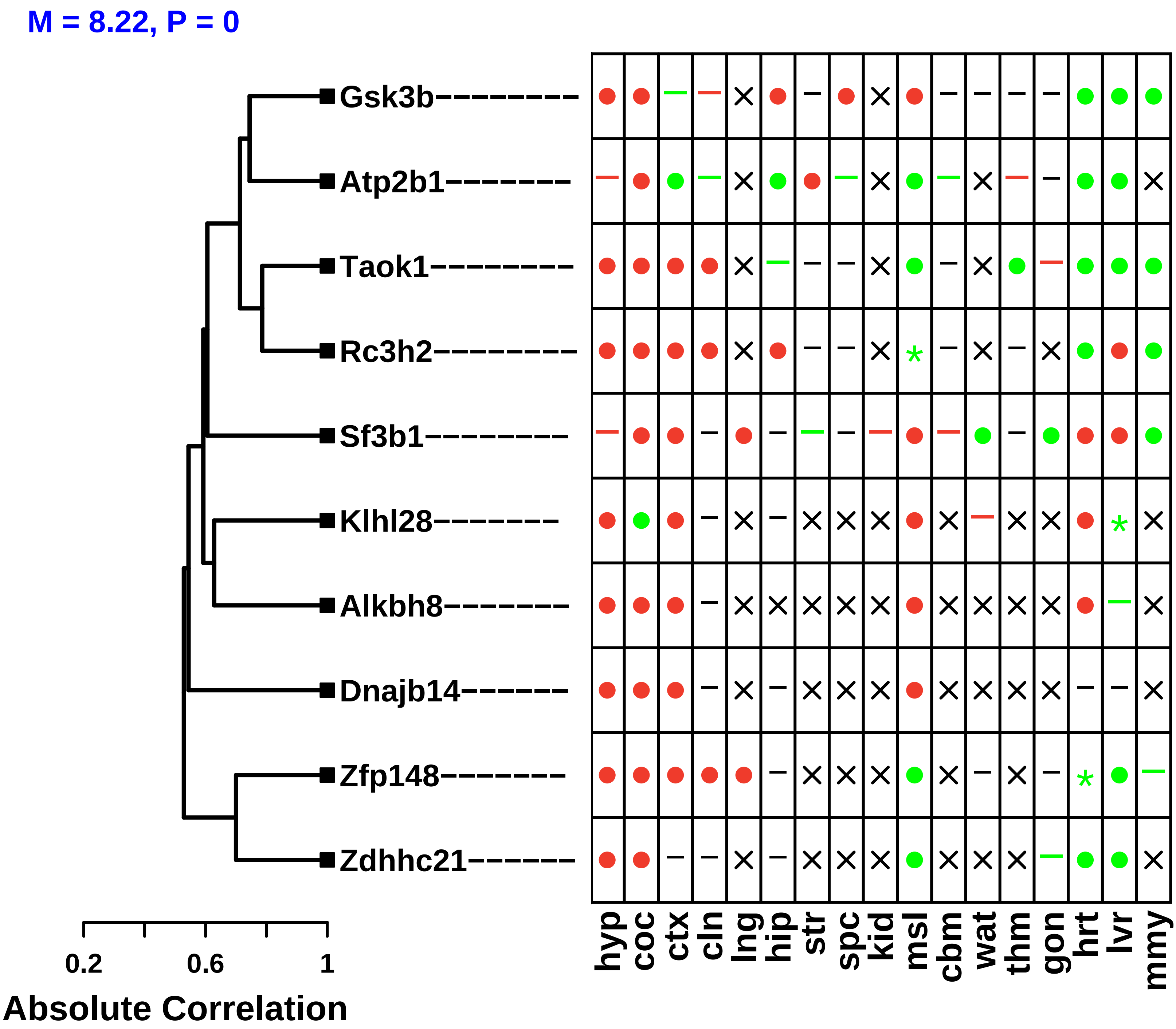

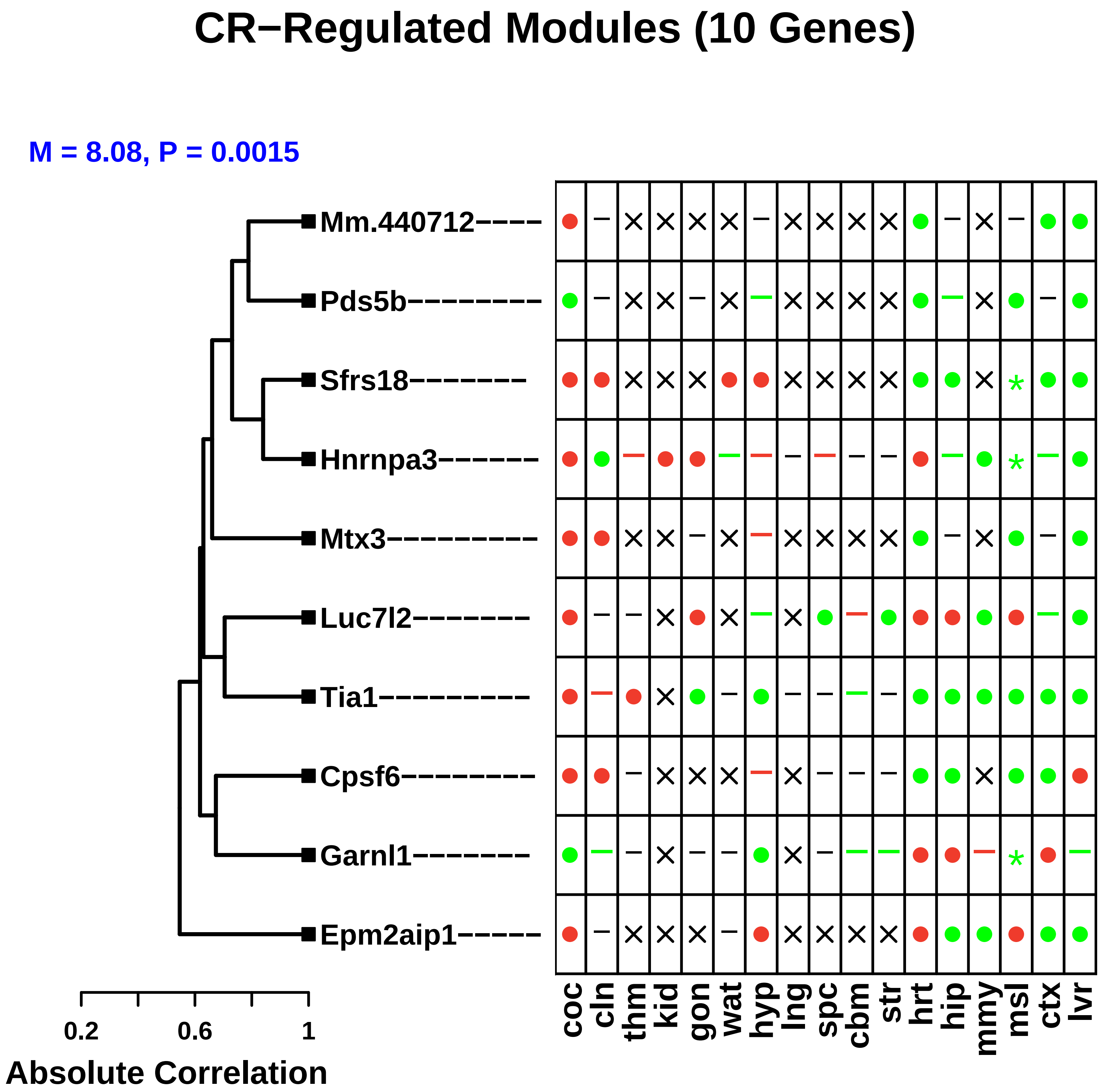
CR−Regulated Modules (10 Genes)
M = 8.08, P = 0.0015
Mm.440712
Pds5b
Sfrs18
Hnrnpa3
Mtx3
Luc7l2
Tia1
Cpsf6
Garnl1
Epm2aip1
coc
cln
thm
kid
gon
wat
hyp
lng
spc
cbm
str
hrt
hip
mmy
msl
ctx
lvr
0.2
0.6
1
Absolute Correlation

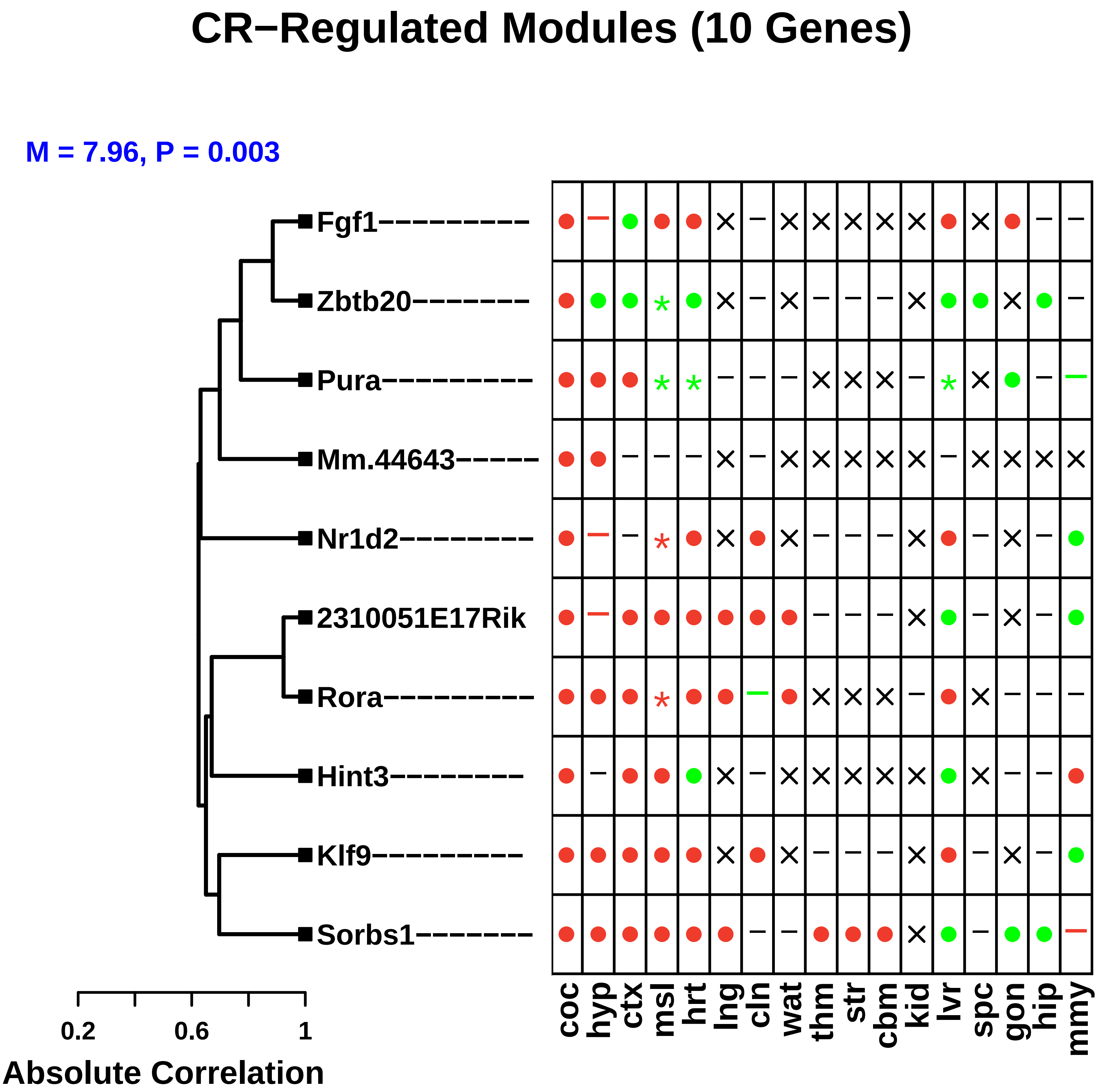

CR−Regulated Modules (10 Genes)
M = 7.96, P = 0.003
Fgf1
Zbtb20
Pura
Mm.44643
Nr1d2
2310051E17Rik
Rora
Hint3
Klf9
Sorbs1
coc
hyp
ctx
msl
hrt
lng
cln
wat
thm
str
cbm
kid
lvr
spc
gon
hip
mmy
0.2
0.6
1
Absolute Correlation

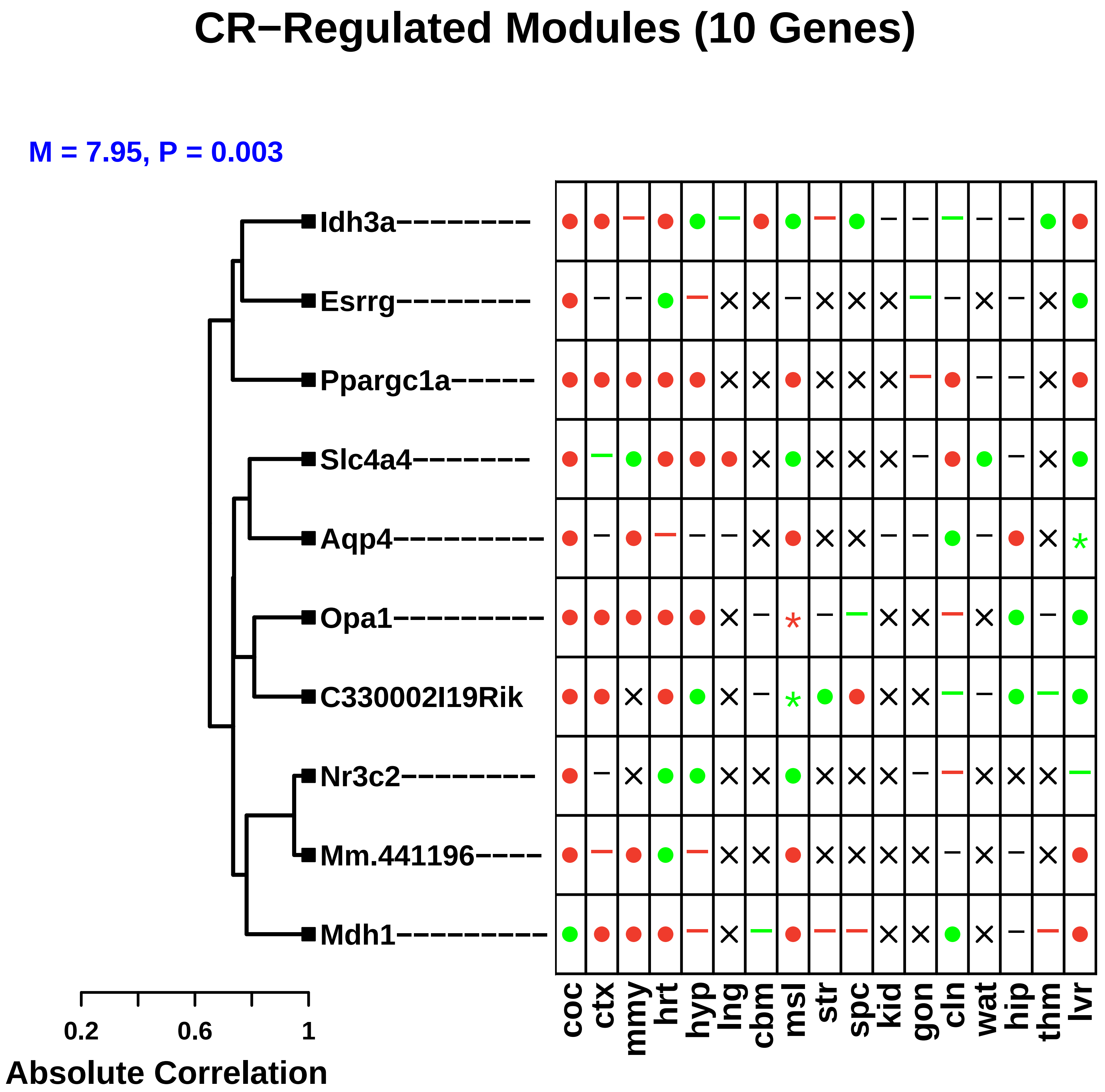

CR−Regulated Modules (10 Genes)
M = 7.95, P = 0.003
Idh3a
Esrrg
Ppargc1a
Slc4a4
Aqp4
Opa1
C330002I19Rik
Nr3c2
Mm.441196
Mdh1
0.2
0.6
1
Absolute Correlation
coc
ctx
mmy
hrt
hyp
lng
cbm
msl
str
spc
kid
gon
cln
wat
hip
thm
lvr

CR−Regulated Modules (10 Genes)

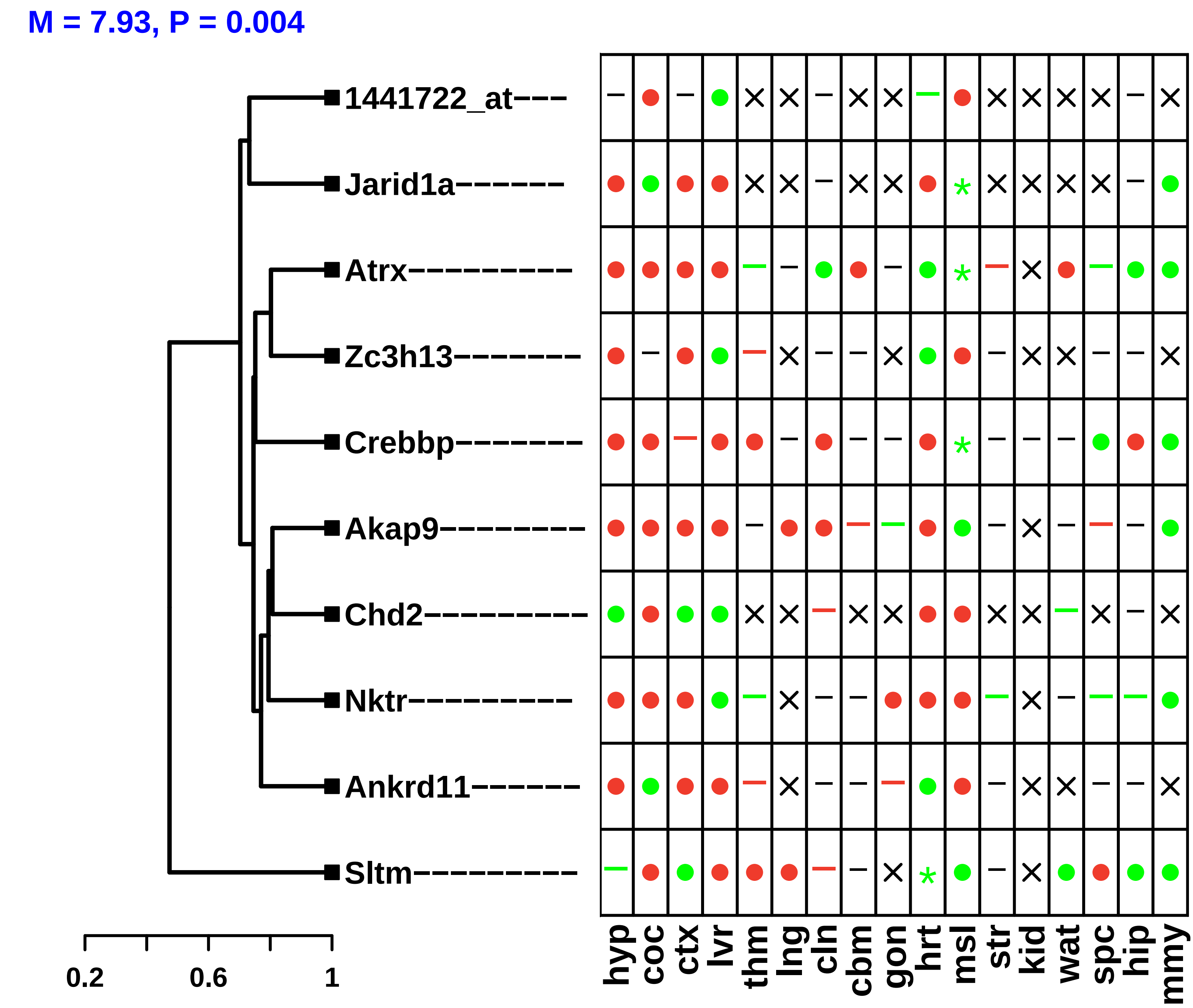

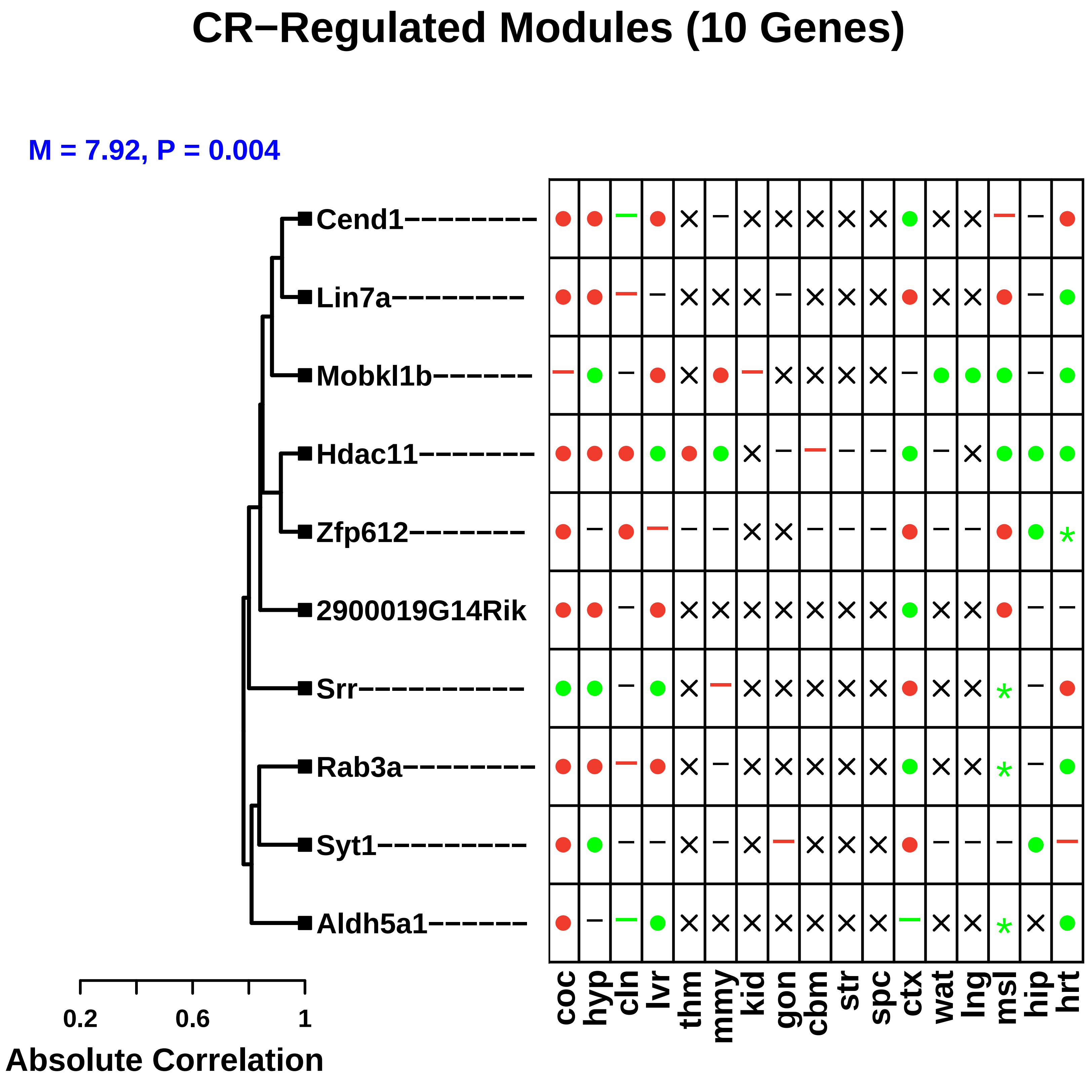
CR−Regulated Modules (10 Genes)
M = 7.92, P = 0.004
Cend1
Lin7a
Mobkl1b
Hdac11
Zfp612
2900019G14Rik
Srr
Rab3a
Syt1
Aldh5a1
0.2
0.6
1
Absolute Correlation
coc
hyp
cln
lvr
thm
mmy
kid
gon
cbm
str
spc
ctx
wat
lng
msl
hip
hrt

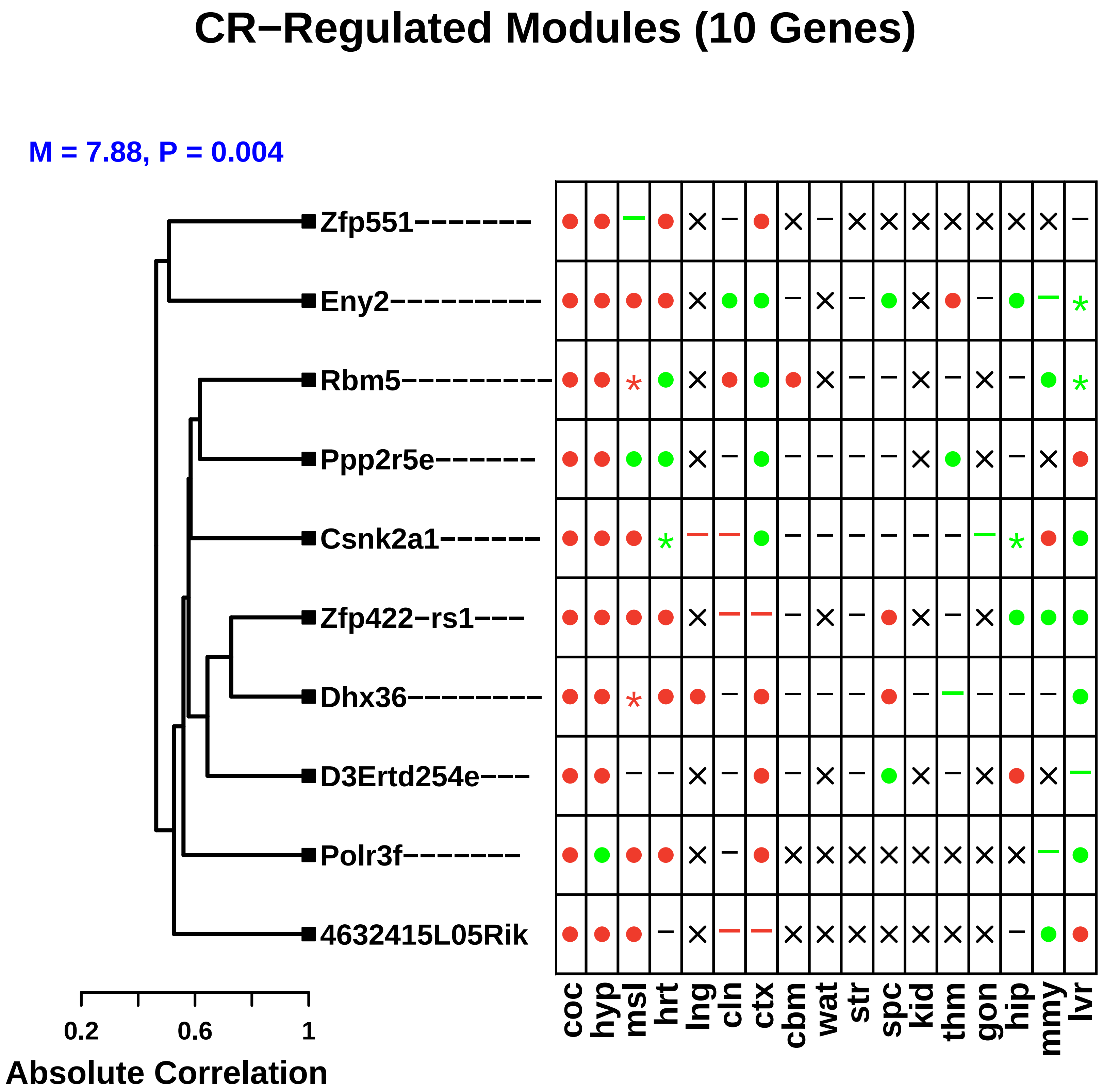

CR−Regulated Modules (10 Genes)
M = 7.88, P = 0.004
Zfp551
Eny2
Rbm5
Ppp2r5e
Csnk2a1
Zfp422−rs1
Dhx36
D3Ertd254e
Polr3f
4632415L05Rik
0.2
0.6
1
Absolute Correlation
coc
hyp
msl
hrt
lng
cln
ctx
cbm
wat
str
spc
kid
thm
gon
hip
mmy
lvr

CR−Regulated Modules (10 Genes)

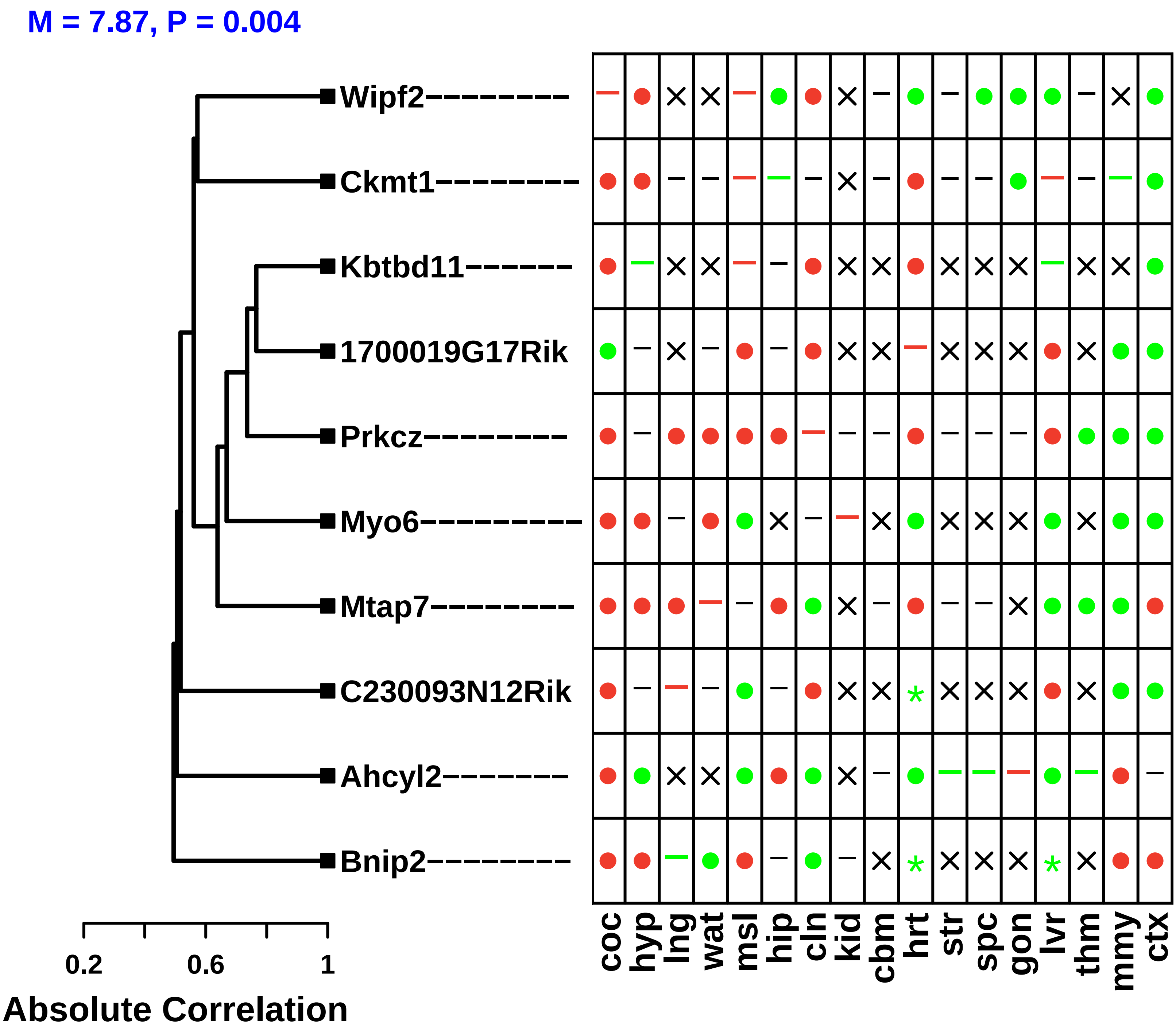

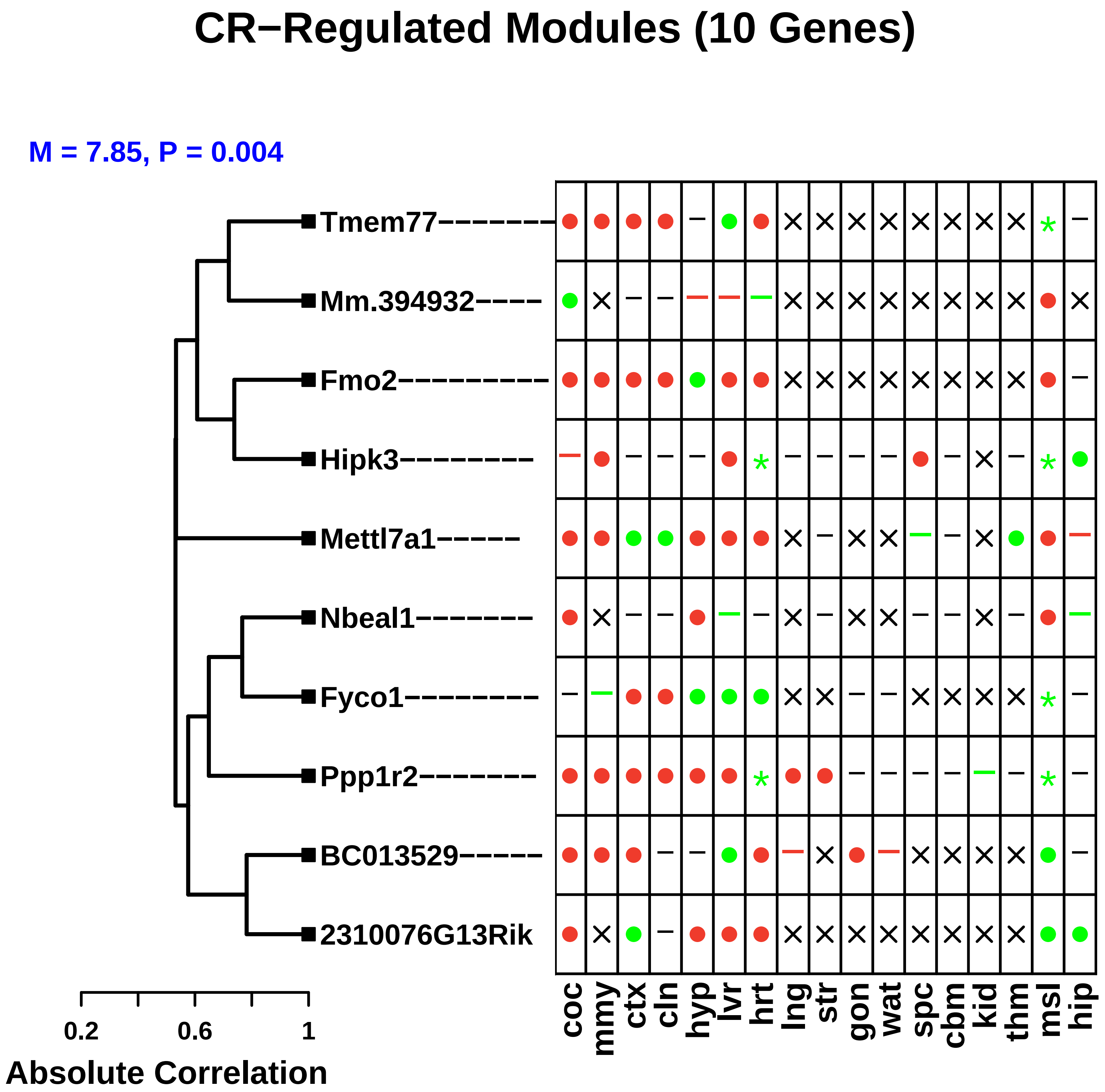
CR−Regulated Modules (10 Genes)
M = 7.85, P = 0.004
Tmem77
Mm.394932
Fmo2
Hipk3
Mettl7a1
Nbeal1
Fyco1
Ppp1r2
BC013529
2310076G13Rik
coc
mmy
ctx
cln
hyp
lvr
hrt
lng
str
gon
wat
spc
cbm
kid
thm
msl
hip
0.2
0.6
1
Absolute Correlation

CR−Regulated Modules (10 Genes)

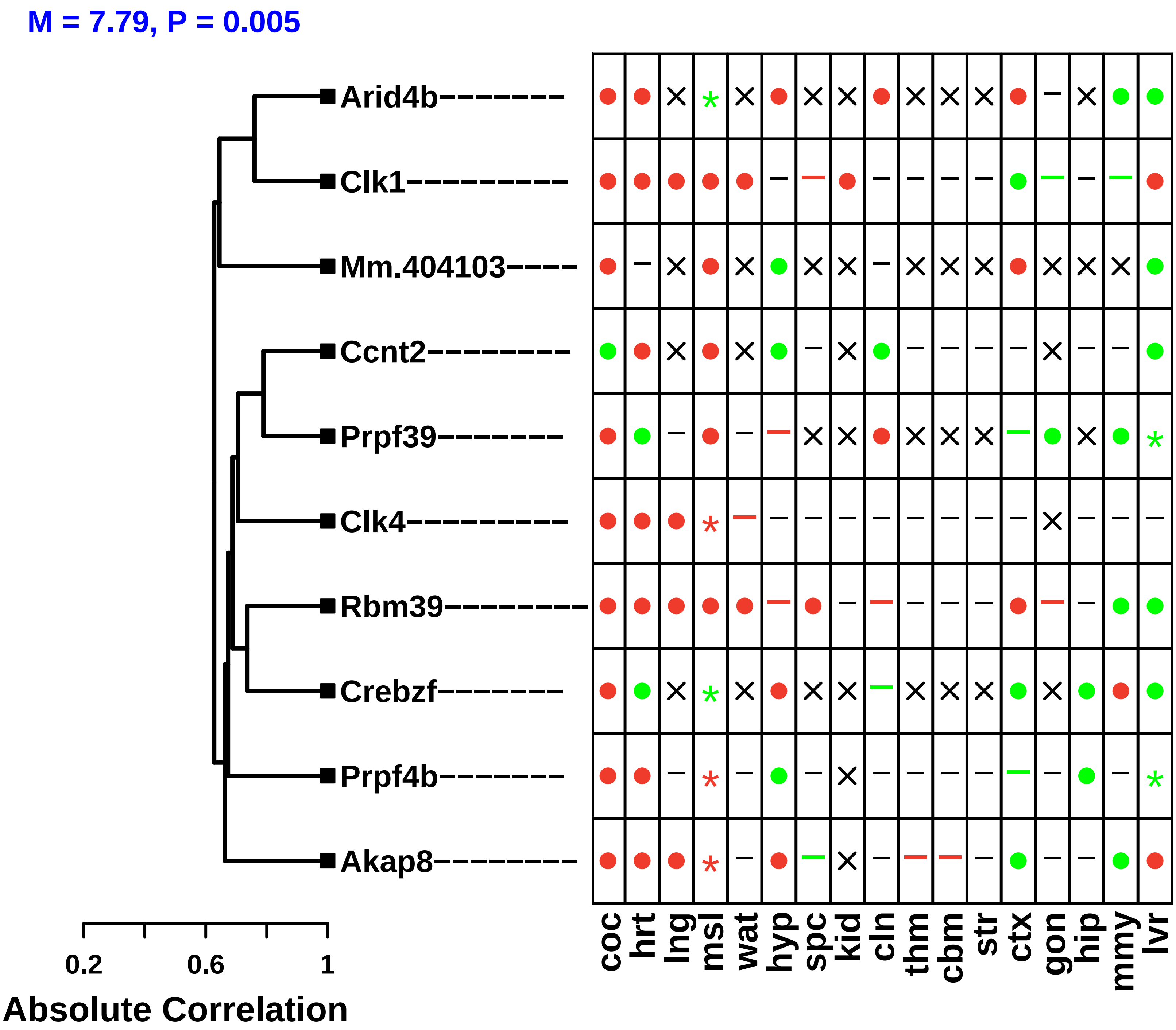

CR−Regulated Modules (10 Genes)

M = 7.78, P = 0.0055

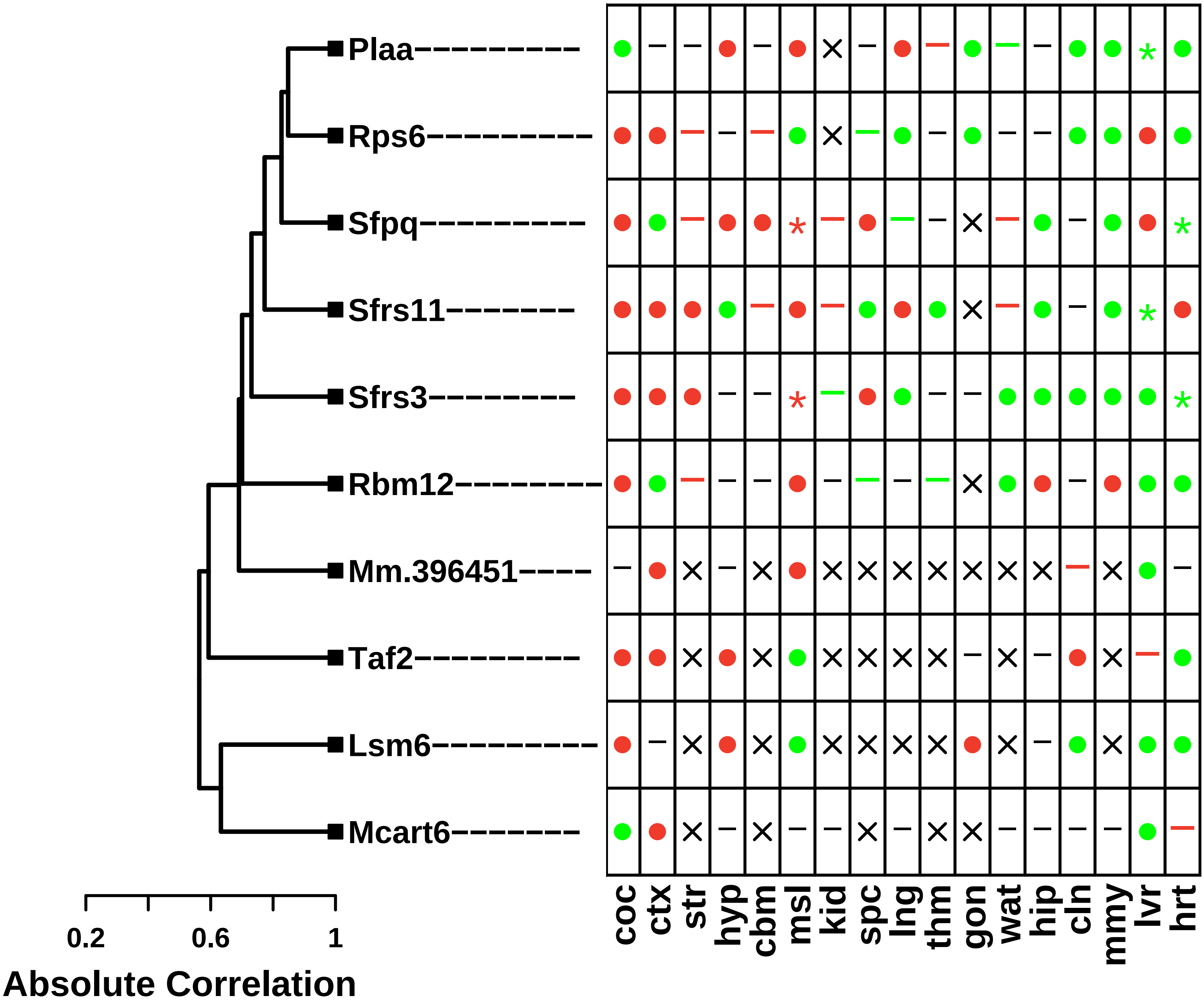

CR−Regulated Modules (10 Genes)

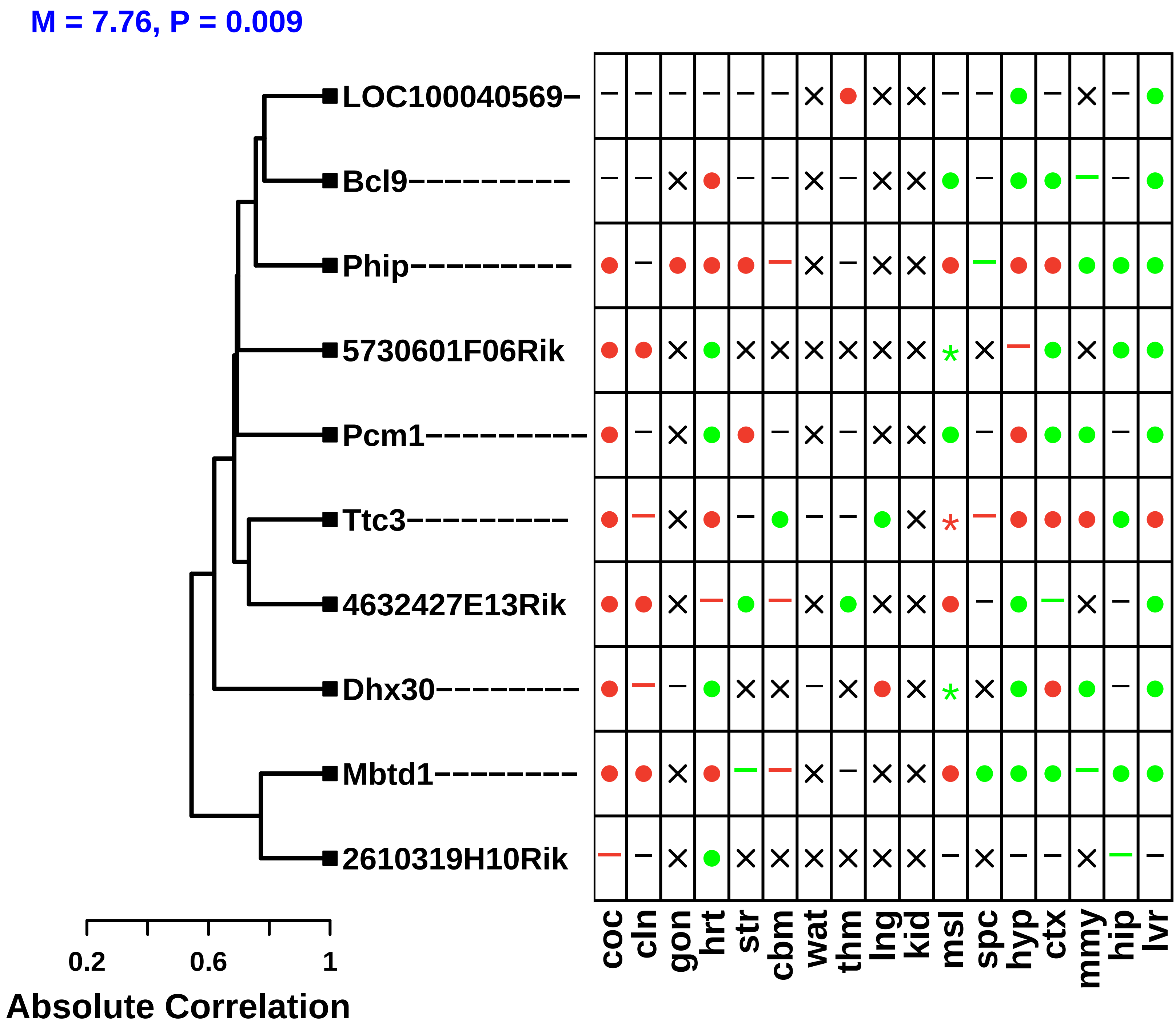

CR−Regulated Modules (10 Genes)

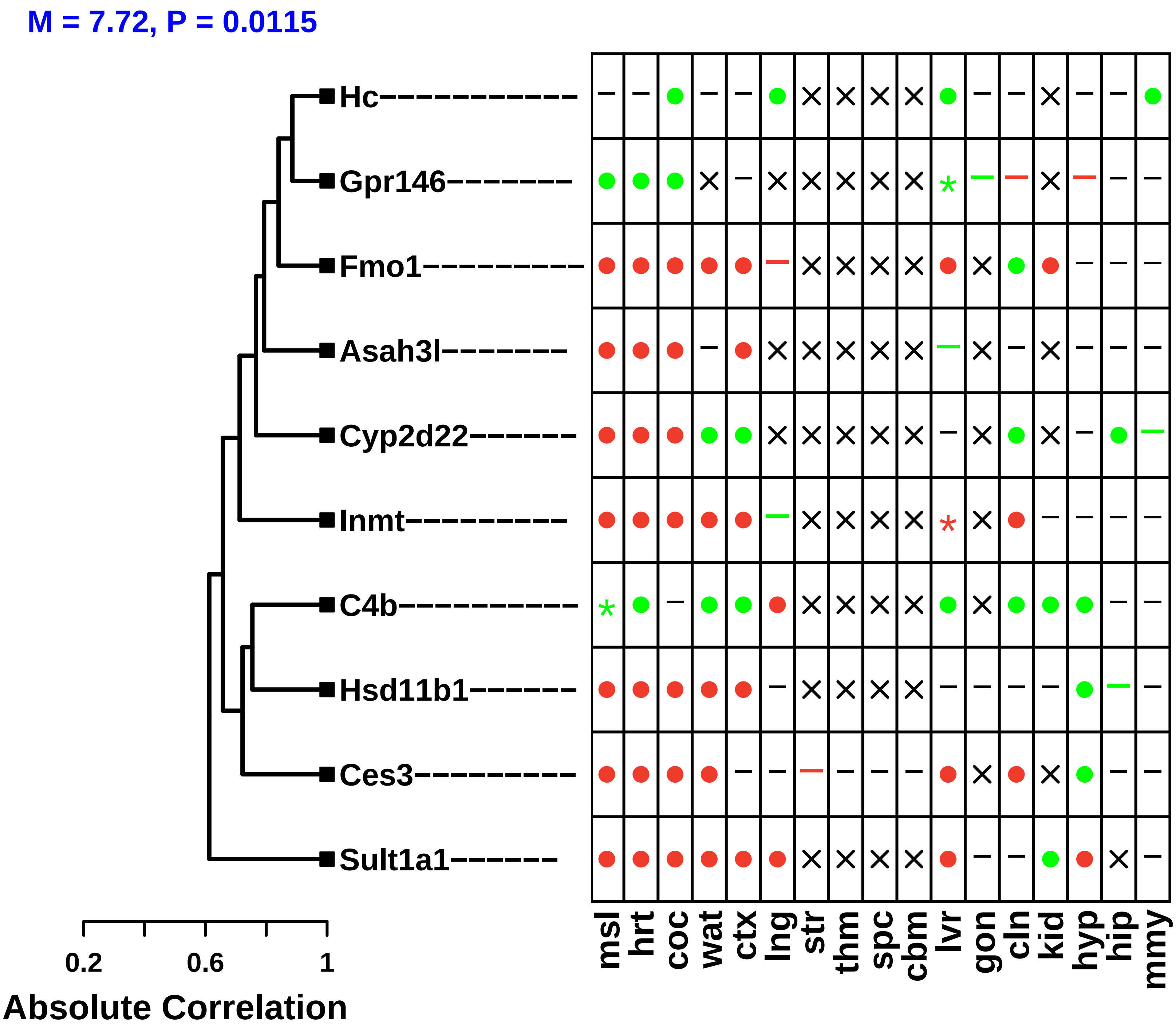

CR−Regulated Modules (10 Genes)

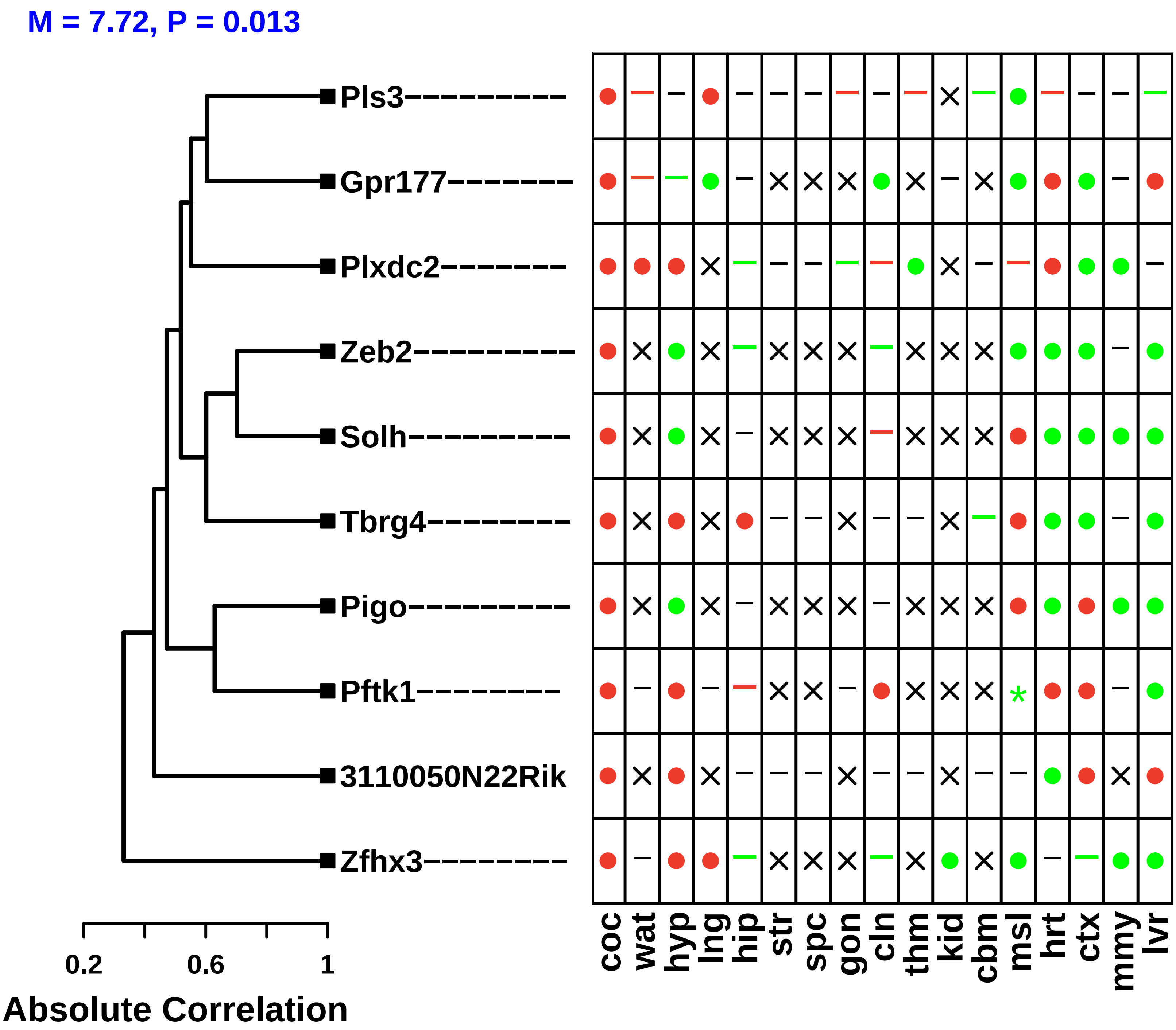

CR−Regulated Modules (10 Genes)

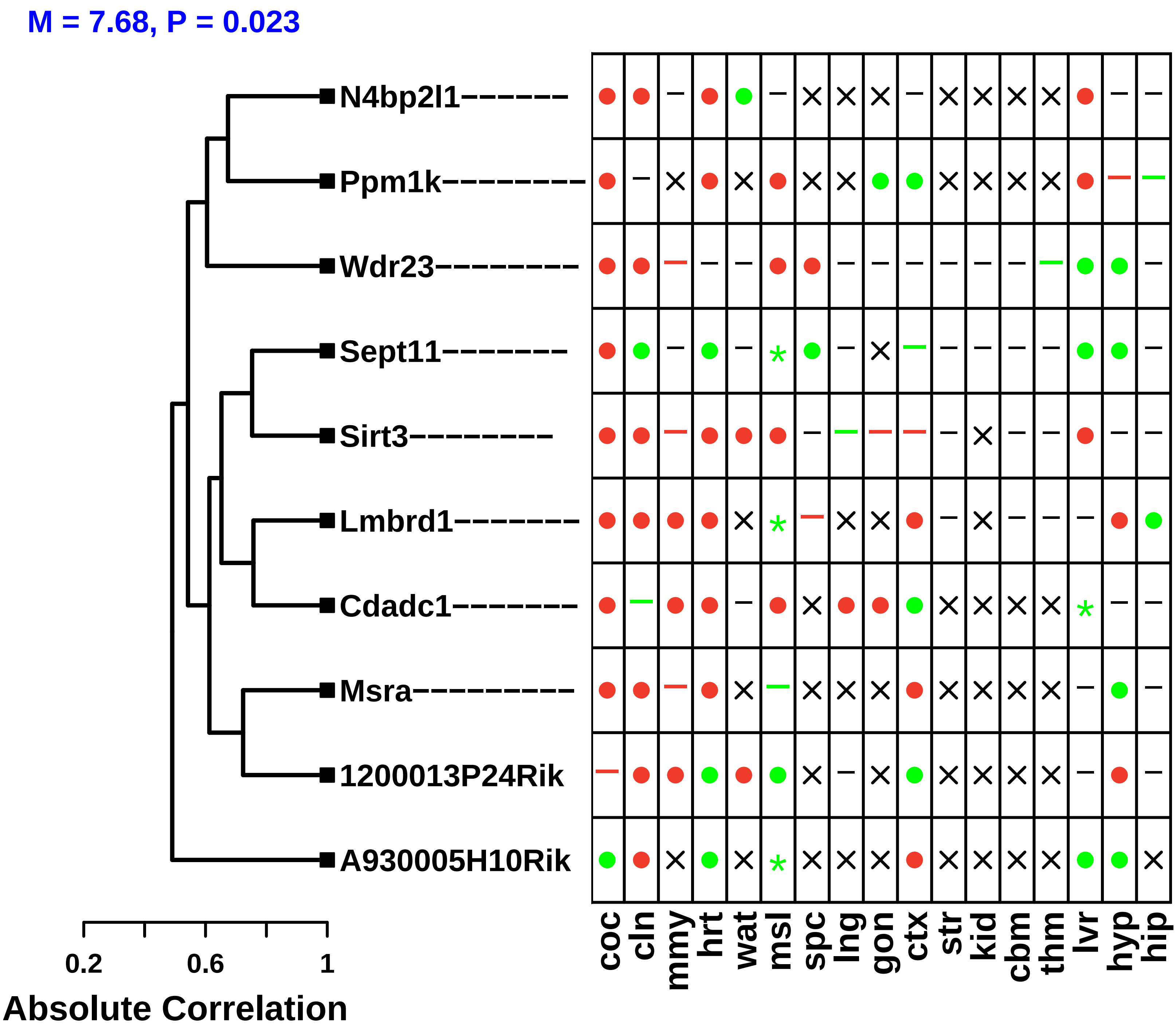

CR−Regulated Modules (10 Genes)

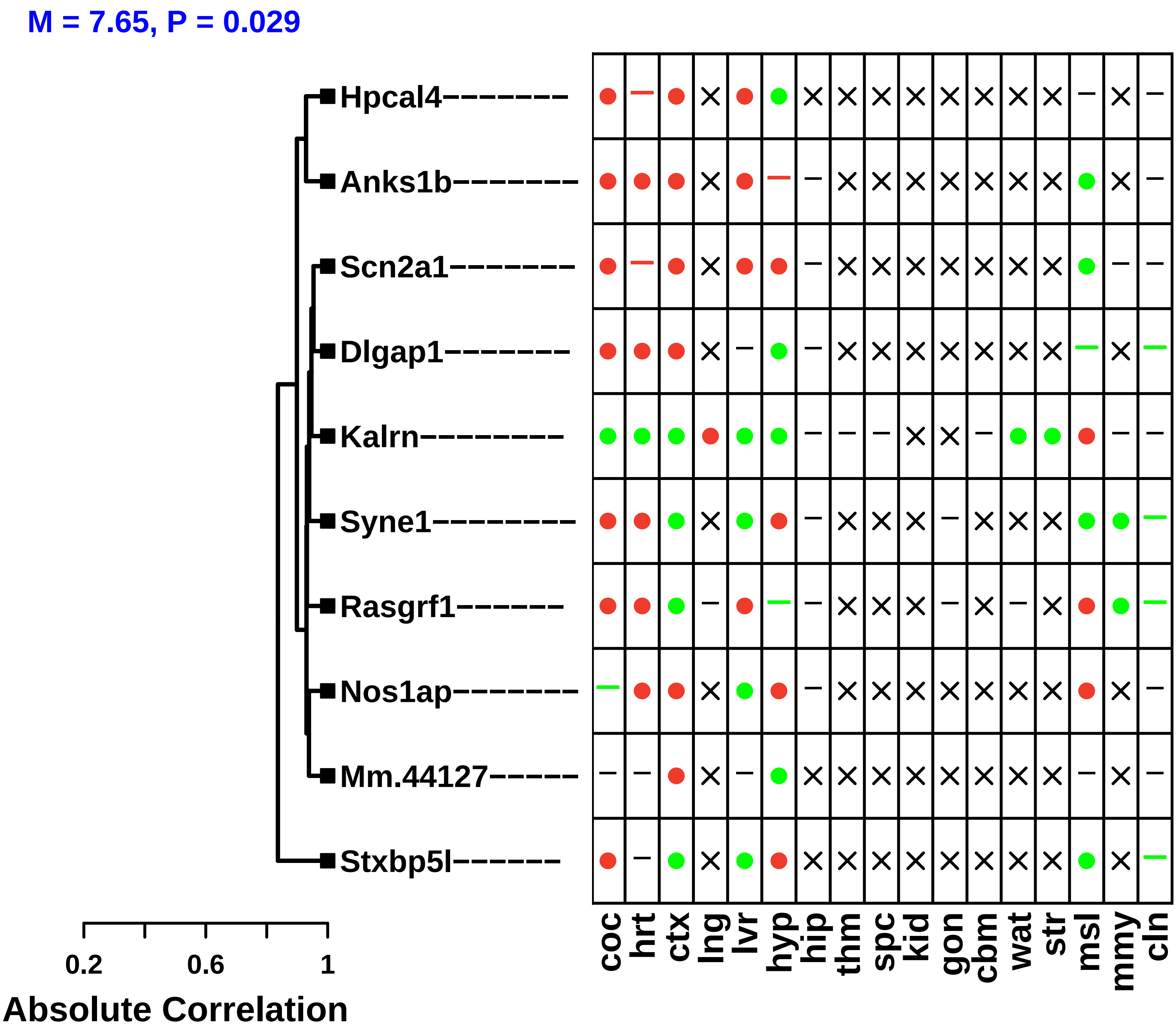

CR−Regulated Modules (10 Genes)

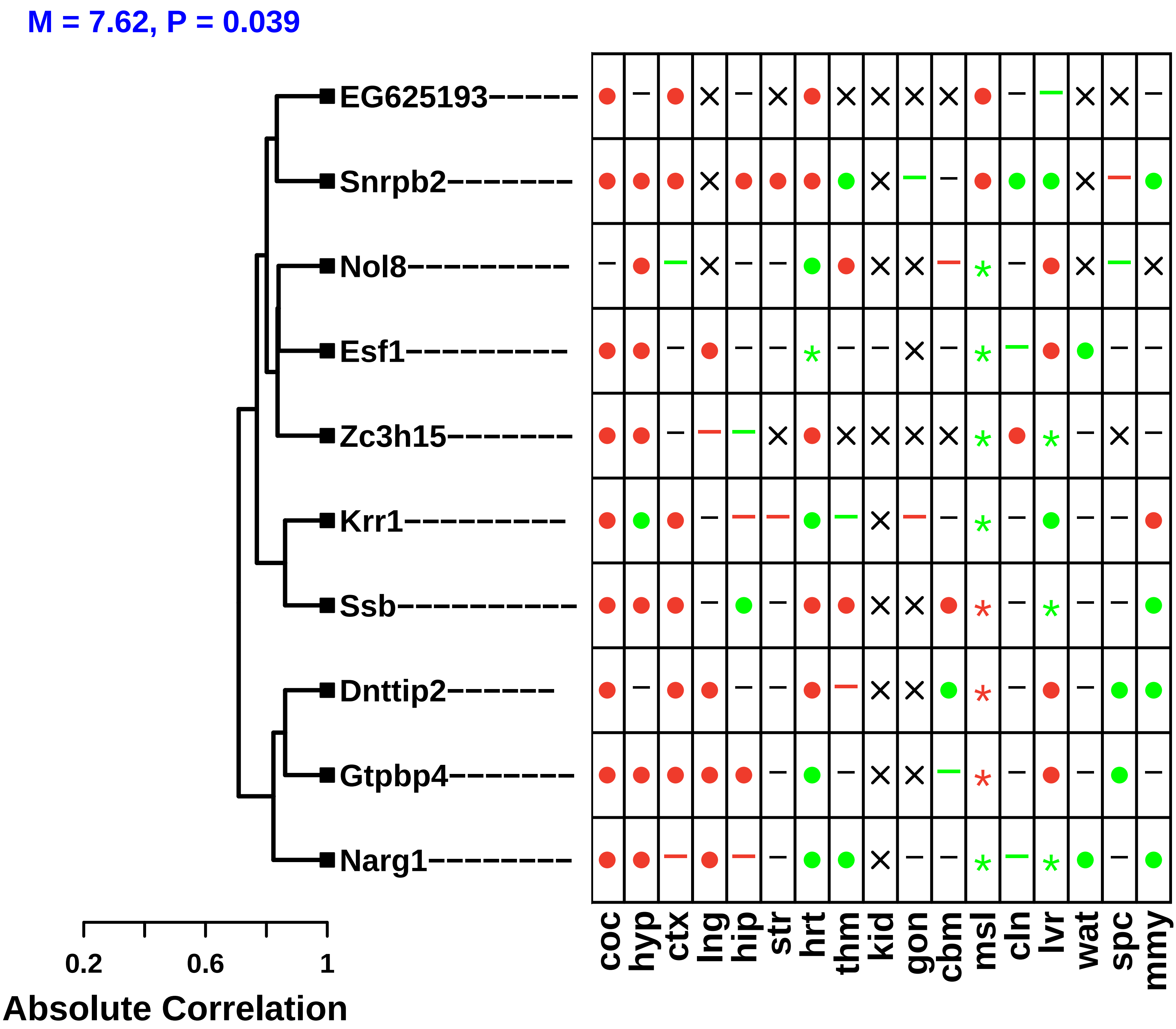

CR−Regulated Modules (10 Genes)

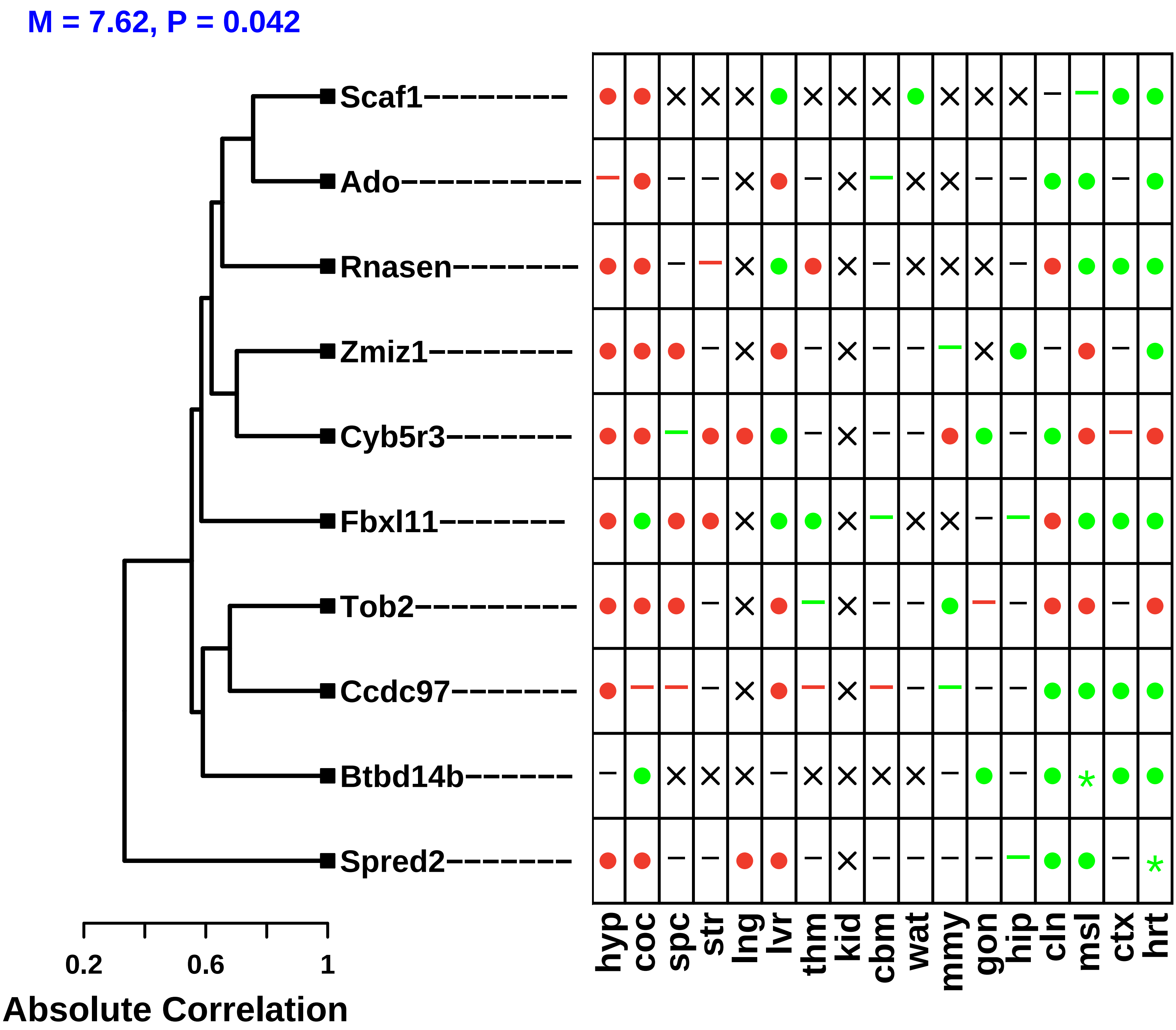

CR−Regulated Modules (10 Genes)

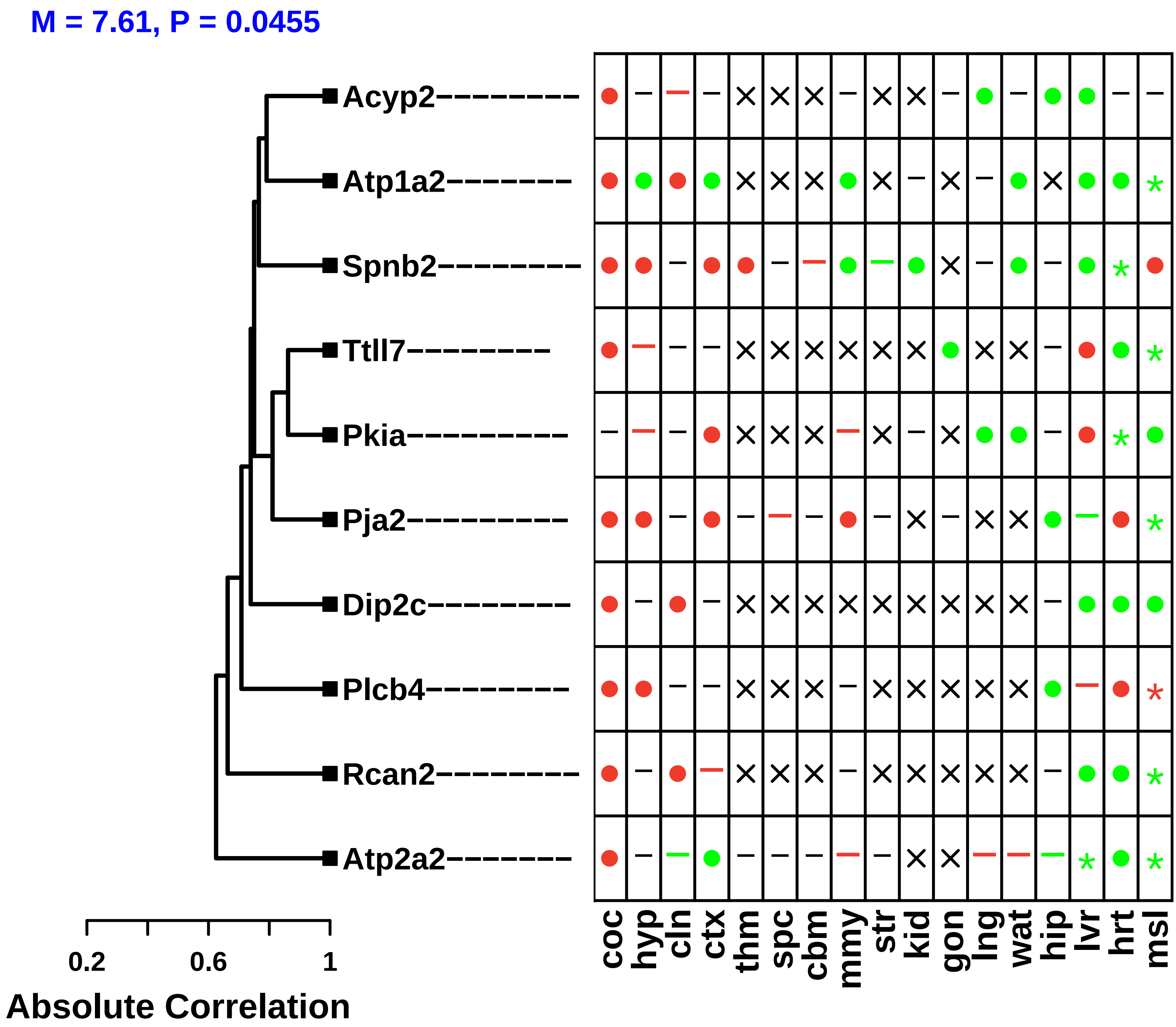

CR−Regulated Modules (20 Genes)

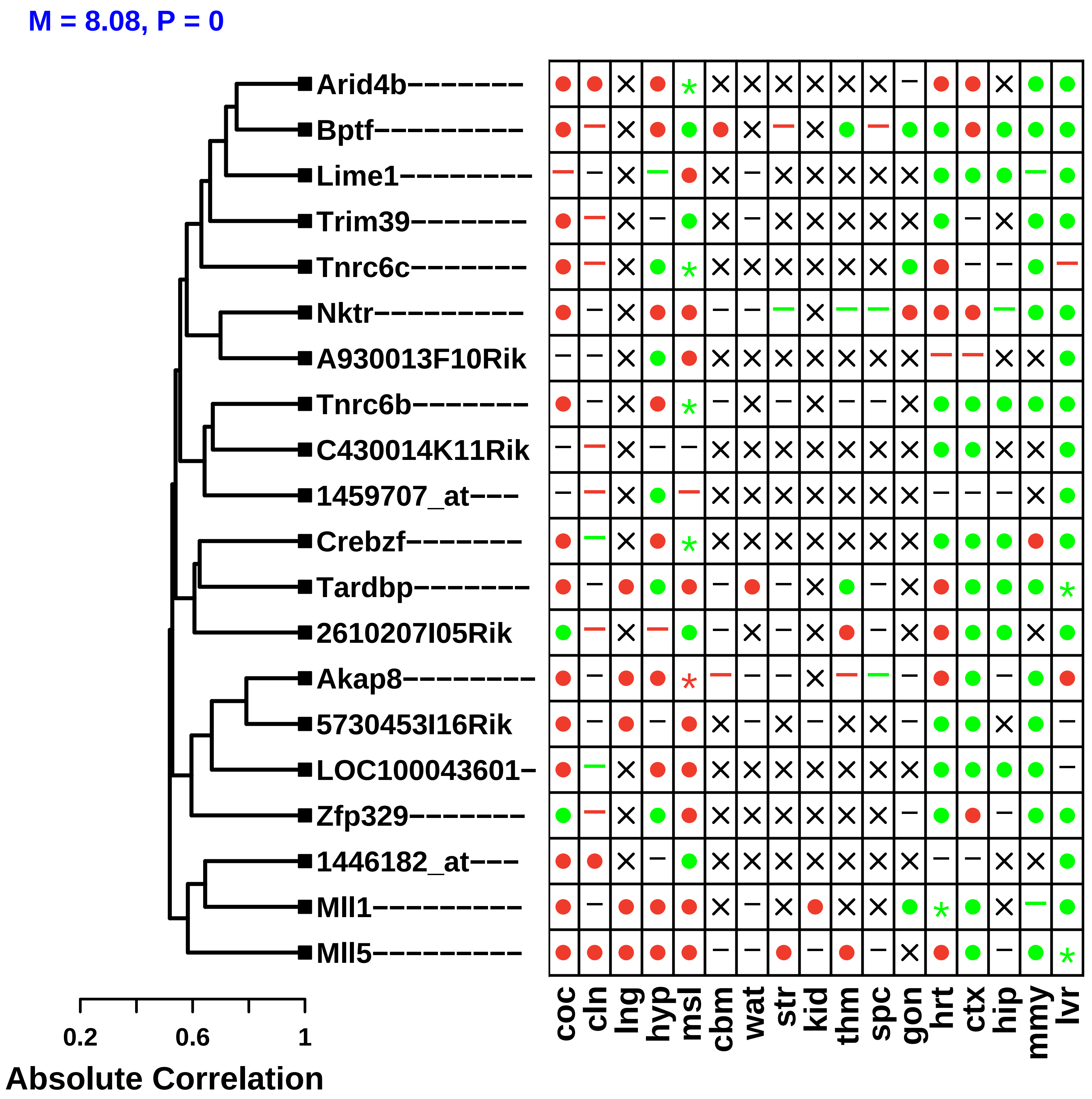

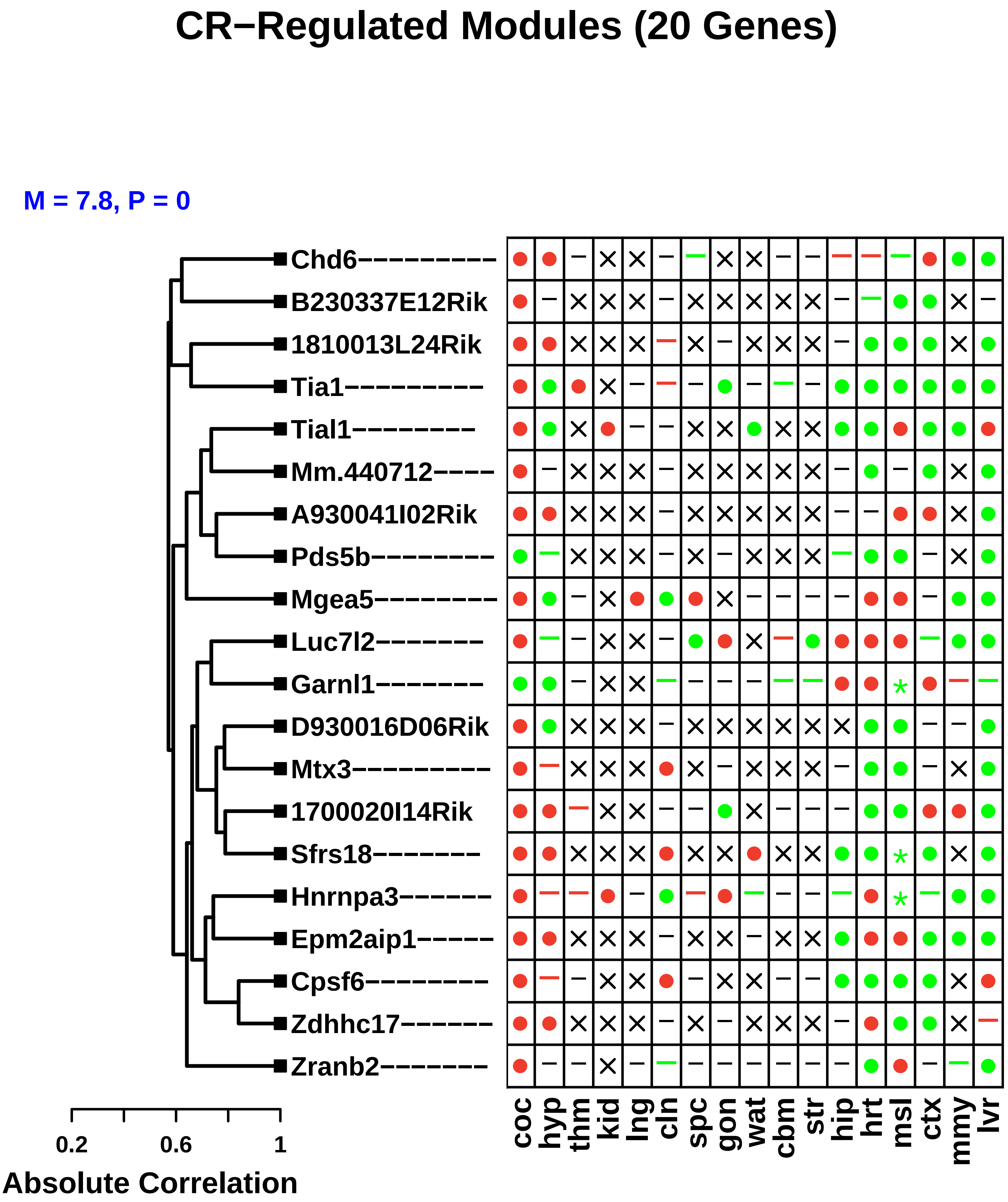
CR−Regulated Modules (20 Genes)
M = 7.8, P = 0
Chd6
B230337E12Rik
1810013L24Rik
Tia1
Tial1
Mm.440712
A930041I02Rik
Pds5b
Mgea5
Luc7l2
Garnl1
D930016D06Rik
Mtx3
1700020I14Rik
Sfrs18
Hnrnpa3
Epm2aip1
Cpsf6
Zdhhc17
Zranb2
coc
hyp
thm
kid
lng
cln
spc
gon
wat
cbm
str
hip
hrt
msl
ctx
mmy
lvr
0.2
0.6
1
Absolute Correlation

CR−Regulated Modules (20 Genes)

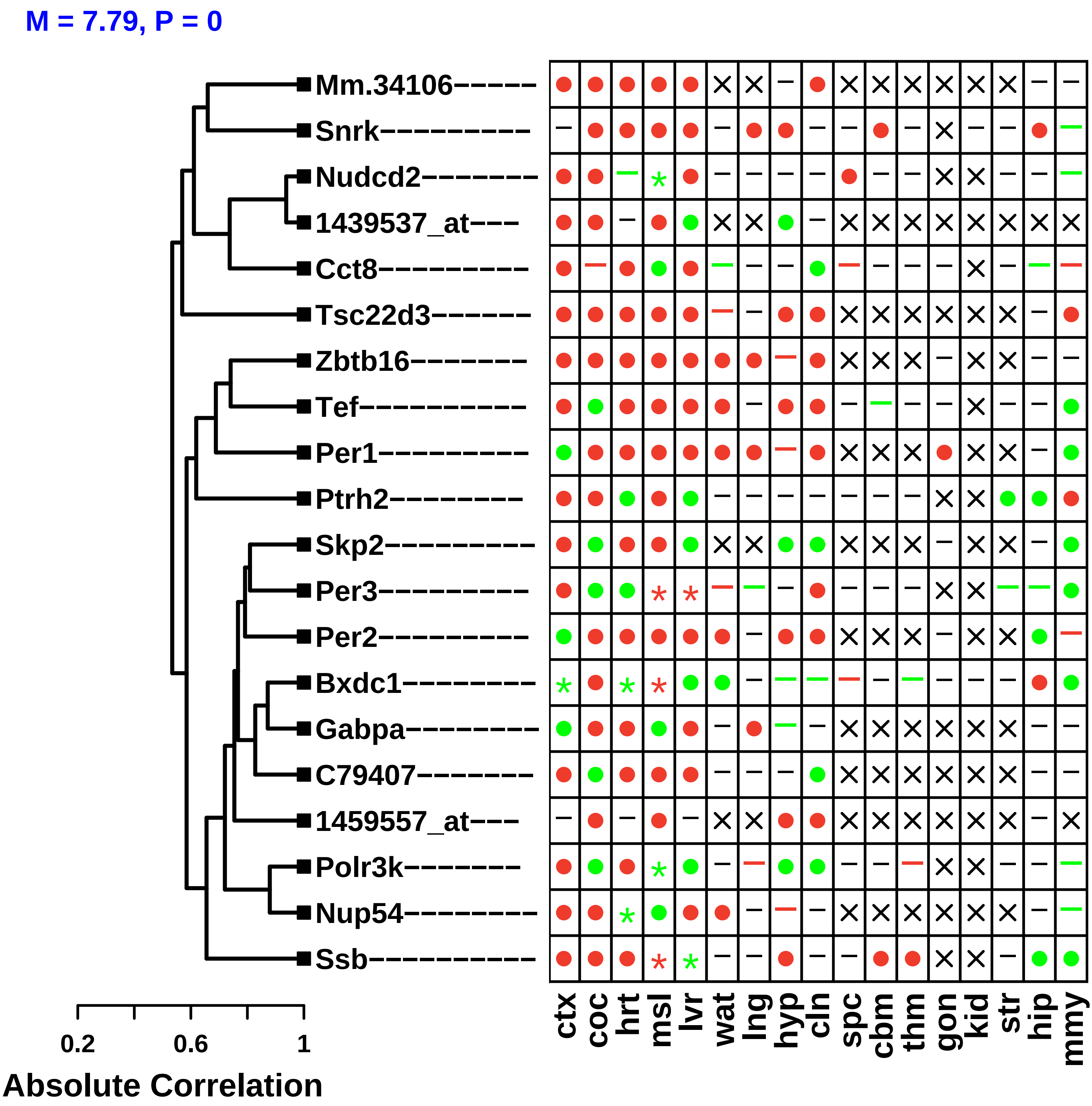

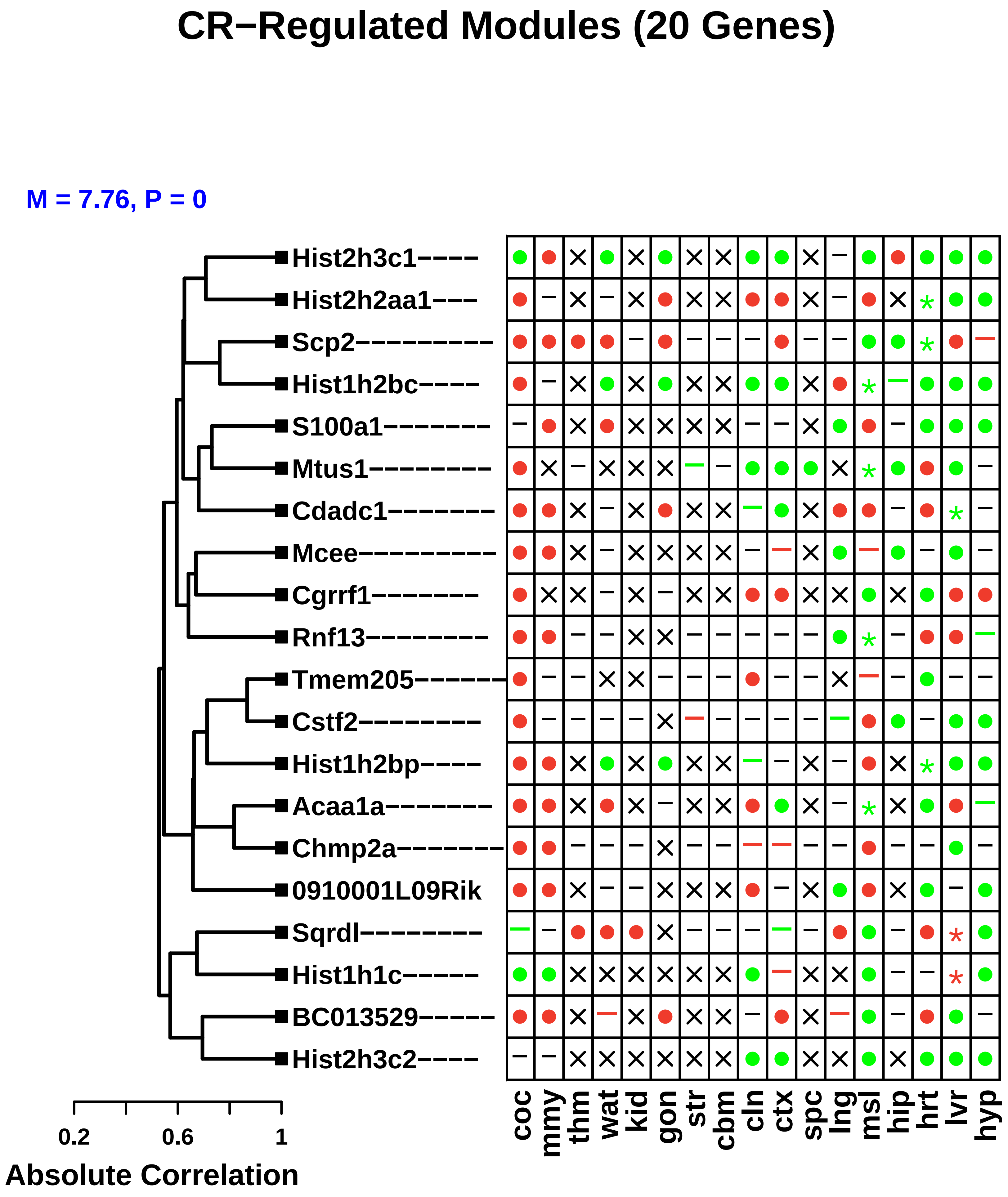
CR−Regulated Modules (20 Genes)
M = 7.76, P = 0
Hist2h3c1
Hist2h2aa1
Scp2
Hist1h2bc
S100a1
Mtus1
Cdadc1
Mcee
Cgrrf1
Rnf13
Tmem205
Cstf2
Hist1h2bp
Acaa1a
Chmp2a
0910001L09Rik
Sqrdl
Hist1h1c
BC013529
Hist2h3c2
0.2
0.6
1
Absolute Correlation
coc
mmy
thm
wat
kid
gon
str
cbm
cln
ctx
spc
lng
msl
hip
hrt
lvr
hyp

CR−Regulated Modules (20 Genes)

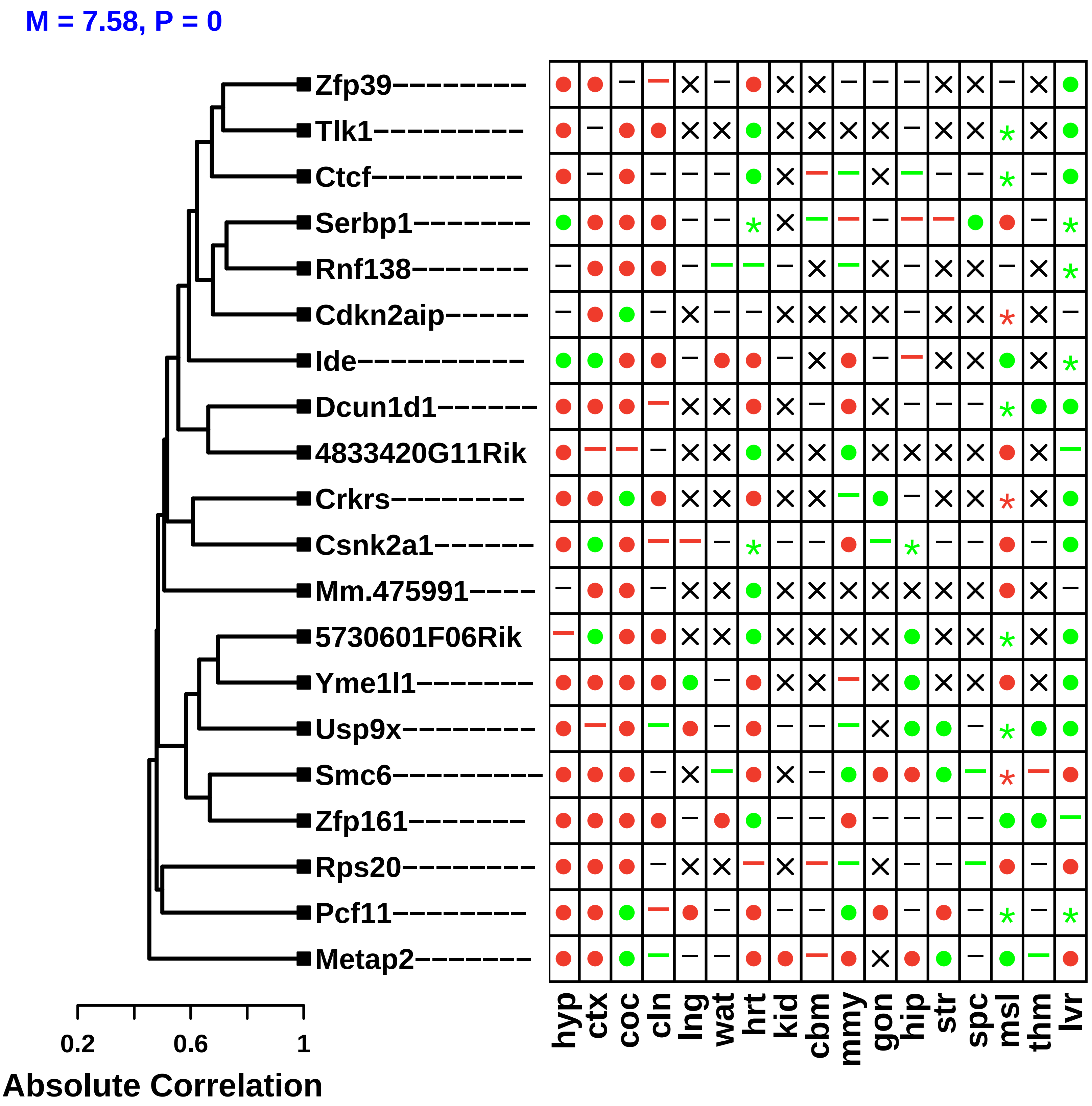

CR−Regulated Modules (20 Genes)

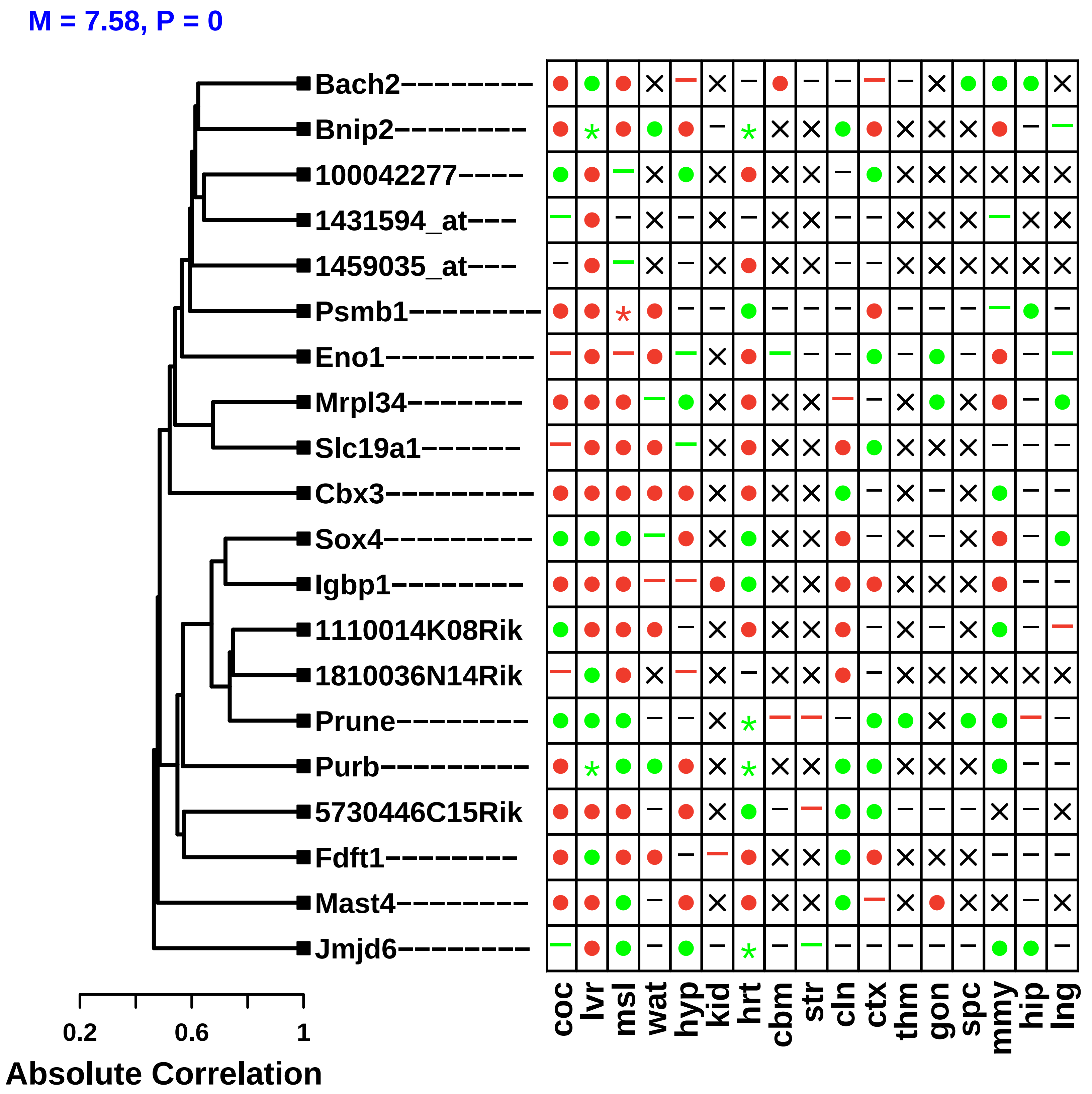

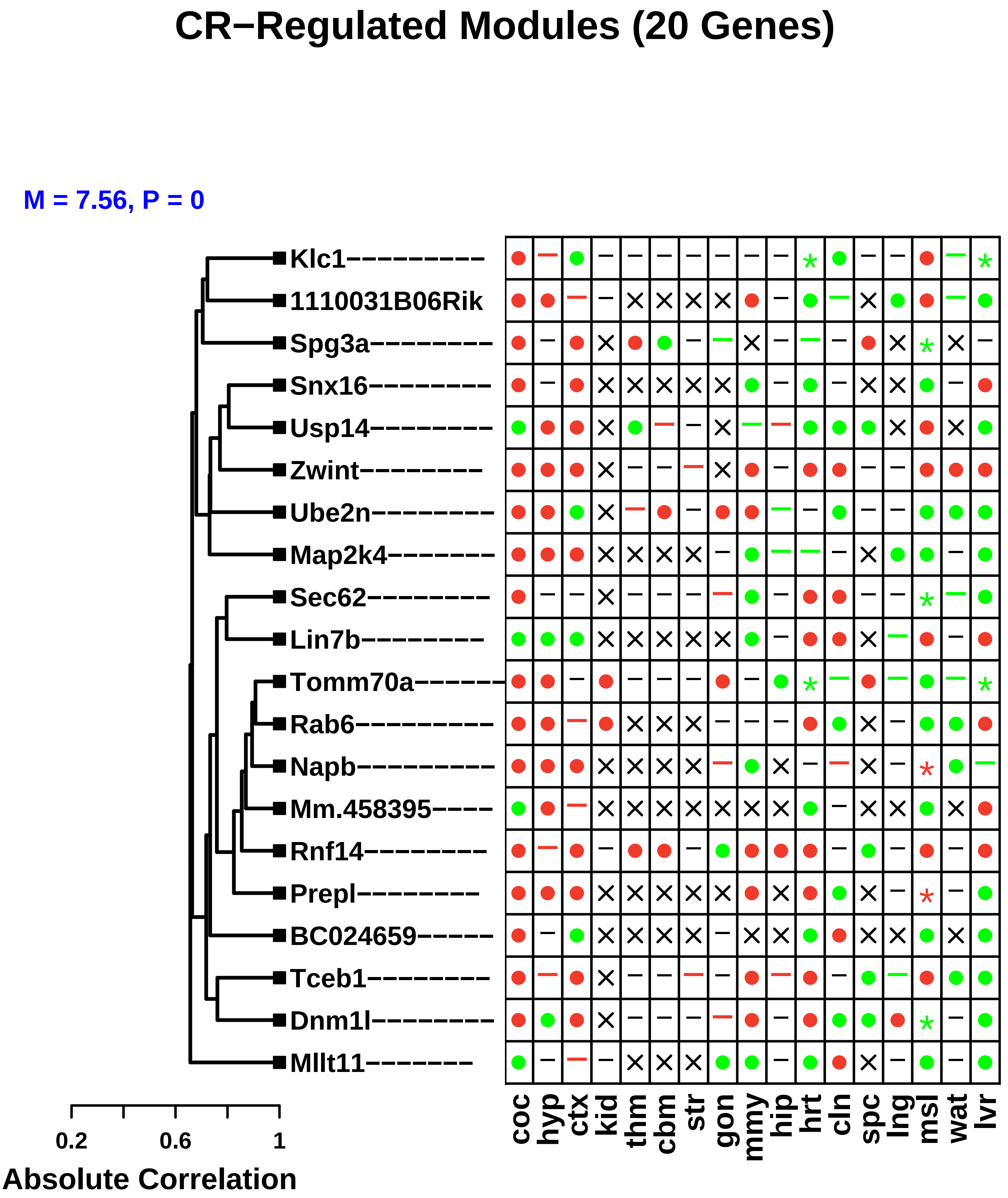
CR−Regulated Modules (20 Genes)
M = 7.56, P = 0
Klc1
1110031B06Rik
Spg3a
Snx16
Usp14
Zwint
Ube2n
Map2k4
Sec62
Lin7b
Tomm70a
Rab6
Napb
Mm.458395
Rnf14
Prepl
BC024659
Tceb1
Dnm1l
Mllt11
coc
hyp
ctx
kid
thm
cbm
str
gon
mmy
hip
hrt
cln
spc
lng
msl
wat
lvr
0.2
0.6
1
Absolute Correlation

CR−Regulated Modules (20 Genes)

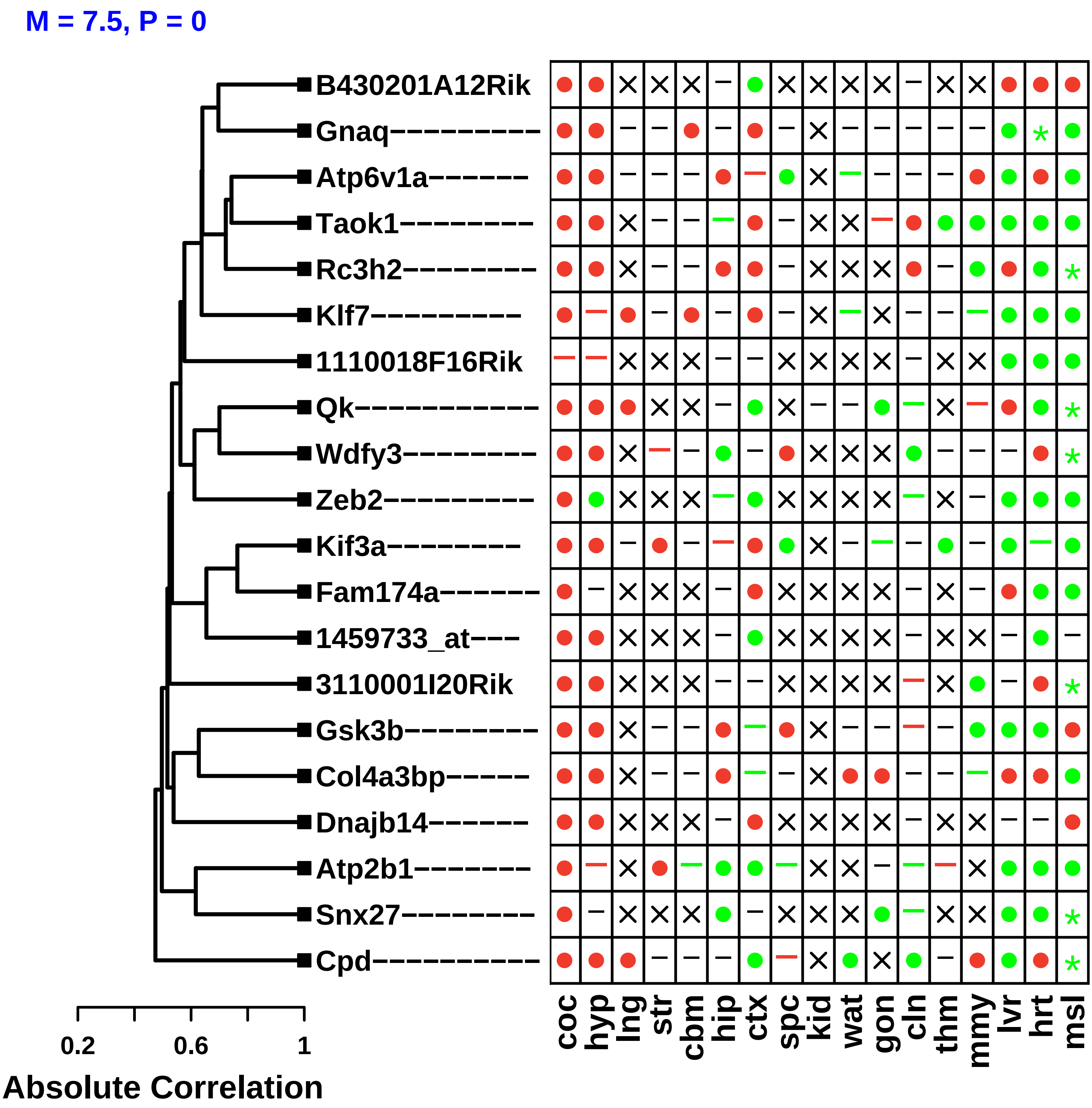

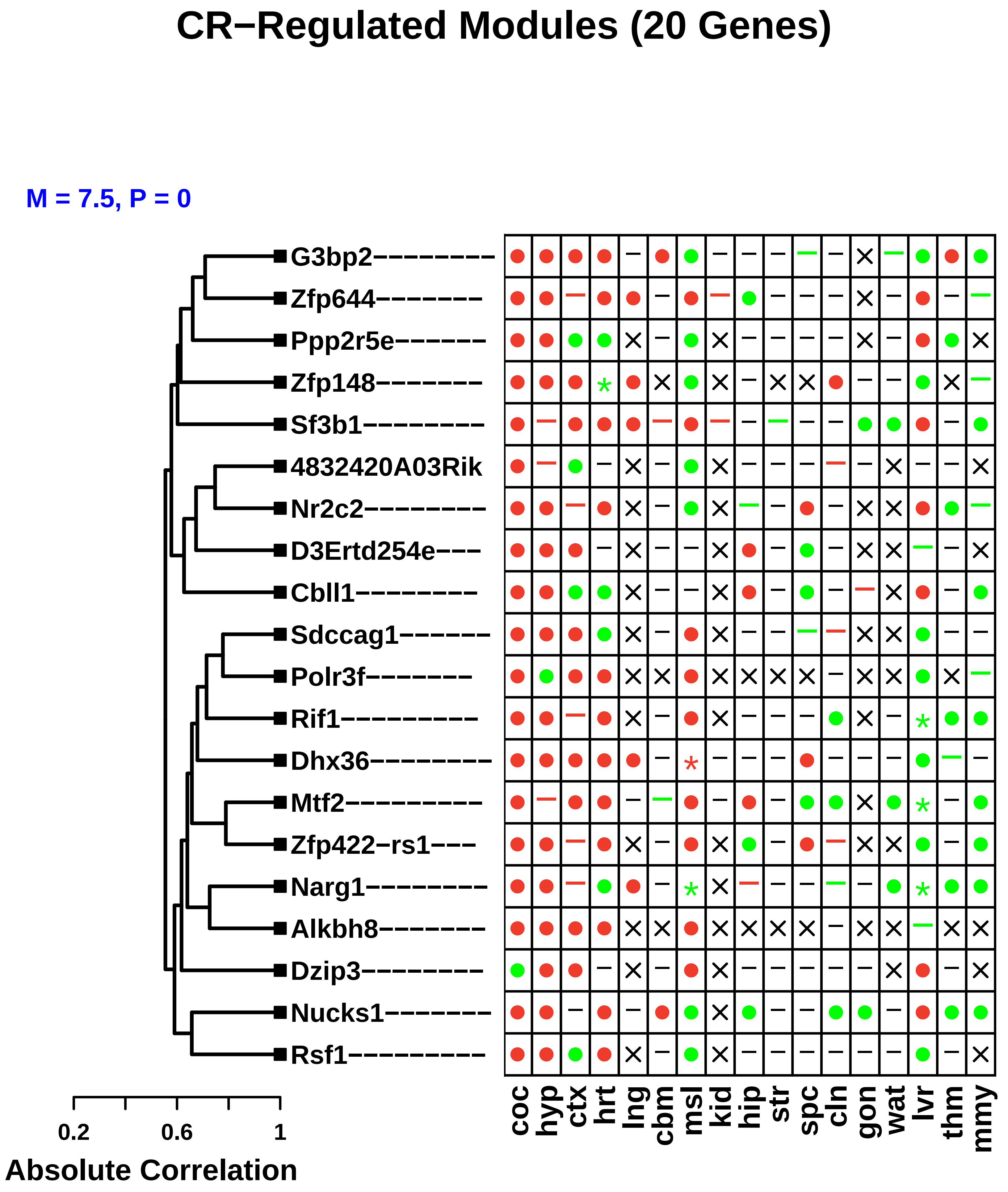
CR−Regulated Modules (20 Genes)
M = 7.5, P = 0
G3bp2
Zfp644
Ppp2r5e
Zfp148
Sf3b1
4832420A03Rik
Nr2c2
D3Ertd254e
Cbll1
Sdccag1
Polr3f
Rif1
Dhx36
Mtf2
Zfp422−rs1
Narg1
Alkbh8
Dzip3
Nucks1
Rsf1
coc
hyp
ctx
hrt
lng
cbm
msl
kid
hip
str
spc
cln
gon
wat
lvr
thm
mmy
0.2
0.6
1
Absolute Correlation

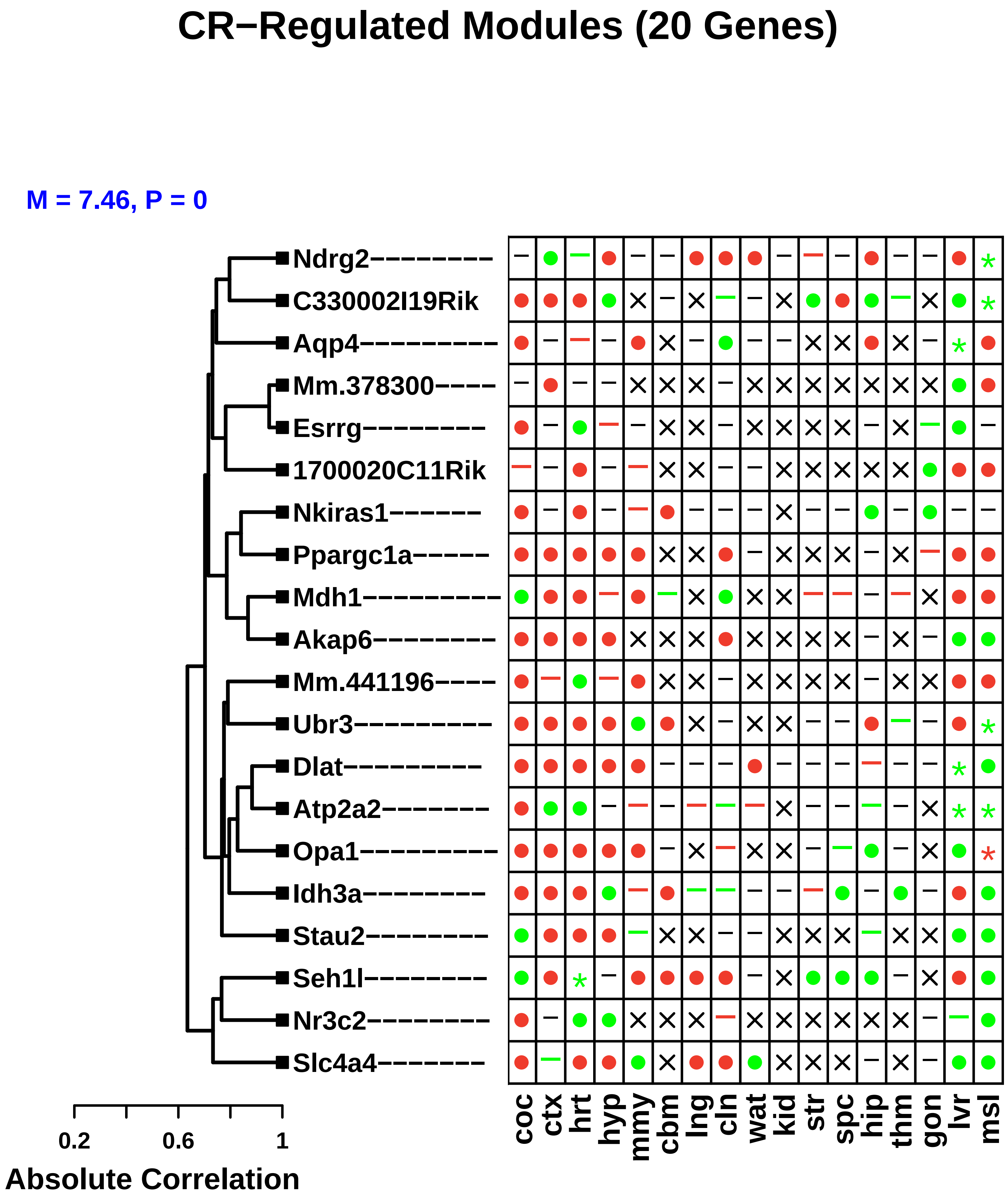

CR−Regulated Modules (20 Genes)
M = 7.46, P = 0
Ndrg2
C330002I19Rik
Aqp4
Mm.378300
Esrrg
1700020C11Rik
Nkiras1
Ppargc1a
Mdh1
Akap6
Mm.441196
Ubr3
Dlat
Atp2a2
Opa1
Idh3a
Stau2
Seh1l
Nr3c2
Slc4a4
coc
ctx
hrt
hyp
mmy
cbm
lng
cln
wat
kid
str
spc
hip
thm
gon
lvr
msl
0.2
0.6
1
Absolute Correlation

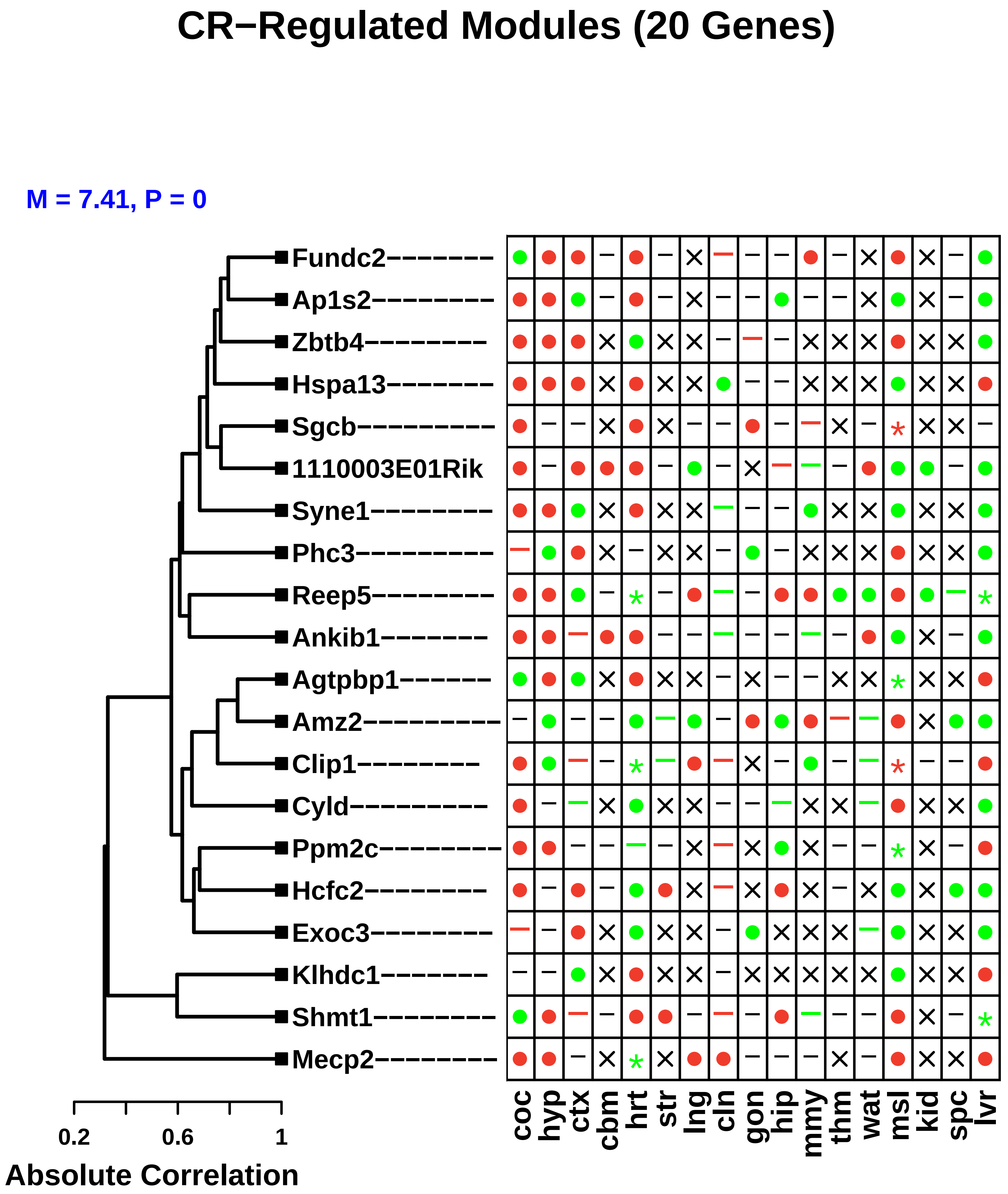
CR−Regulated Modules (20 Genes)
M = 7.41, P = 0
Fundc2
Ap1s2
Zbtb4
Hspa13
Sgcb
1110003E01Rik
Syne1
Phc3
Reep5
Ankib1
Agtpbp1
Amz2
Clip1
Cyld
Ppm2c
Hcfc2
Exoc3
Klhdc1
Shmt1
Mecp2
coc
hyp
ctx
cbm
hrt
str
lng
cln
gon
hip
mmy
thm
wat
msl
kid
spc
lvr
0.2
0.6
1
Absolute Correlation

CR−Regulated Modules (20 Genes)

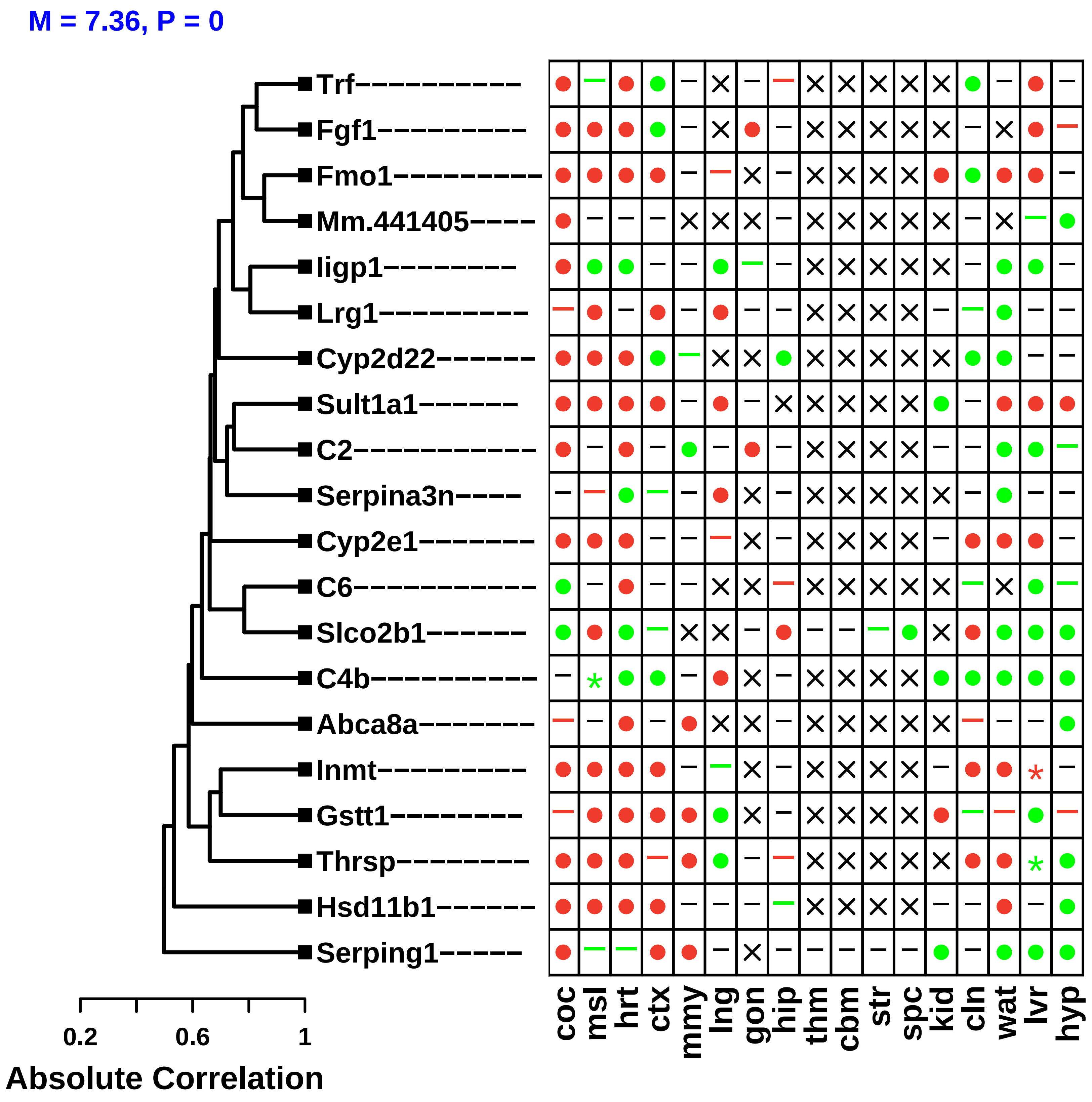

CR−Regulated Modules (20 Genes)

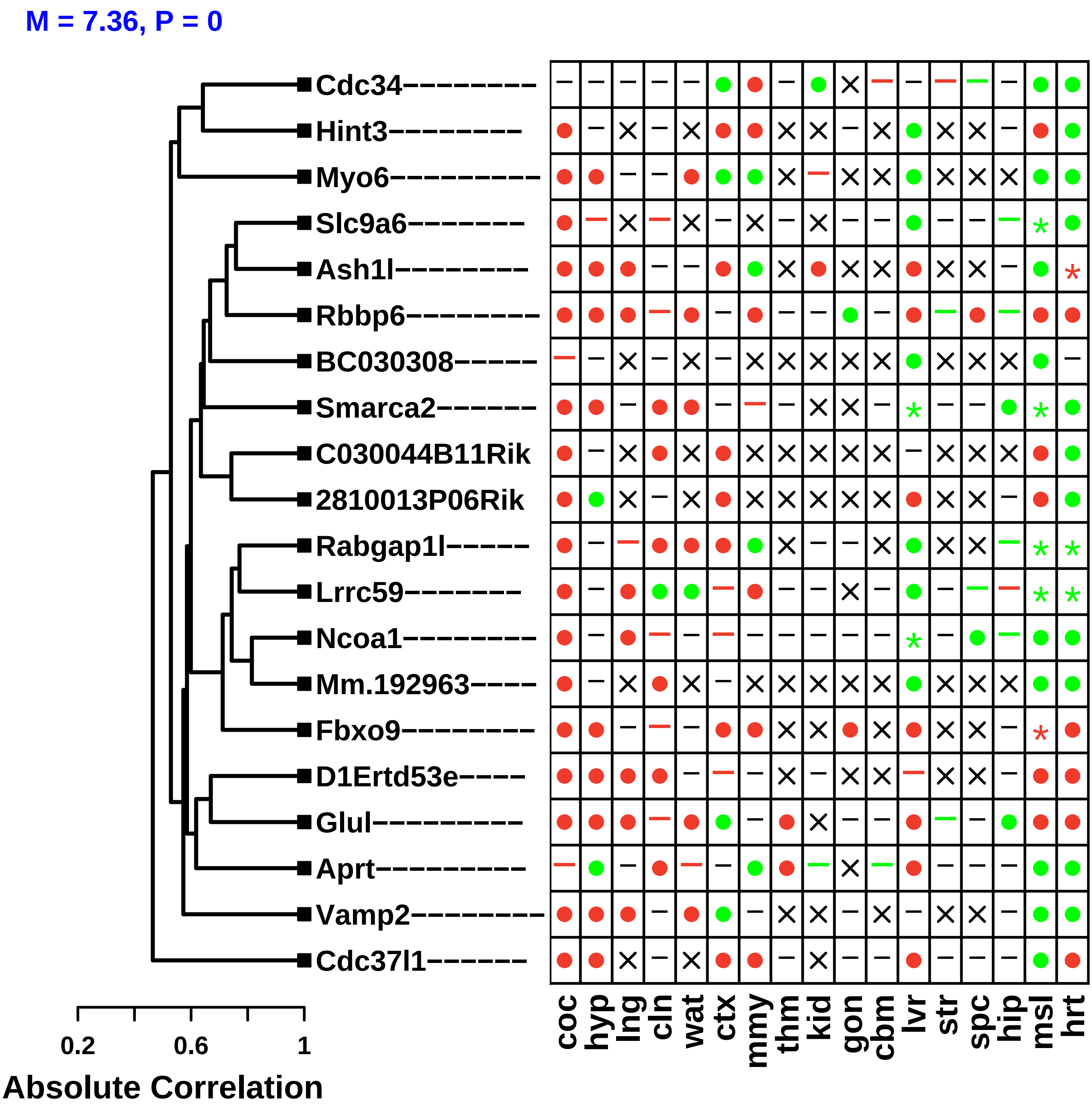

CR−Regulated Modules (20 Genes)

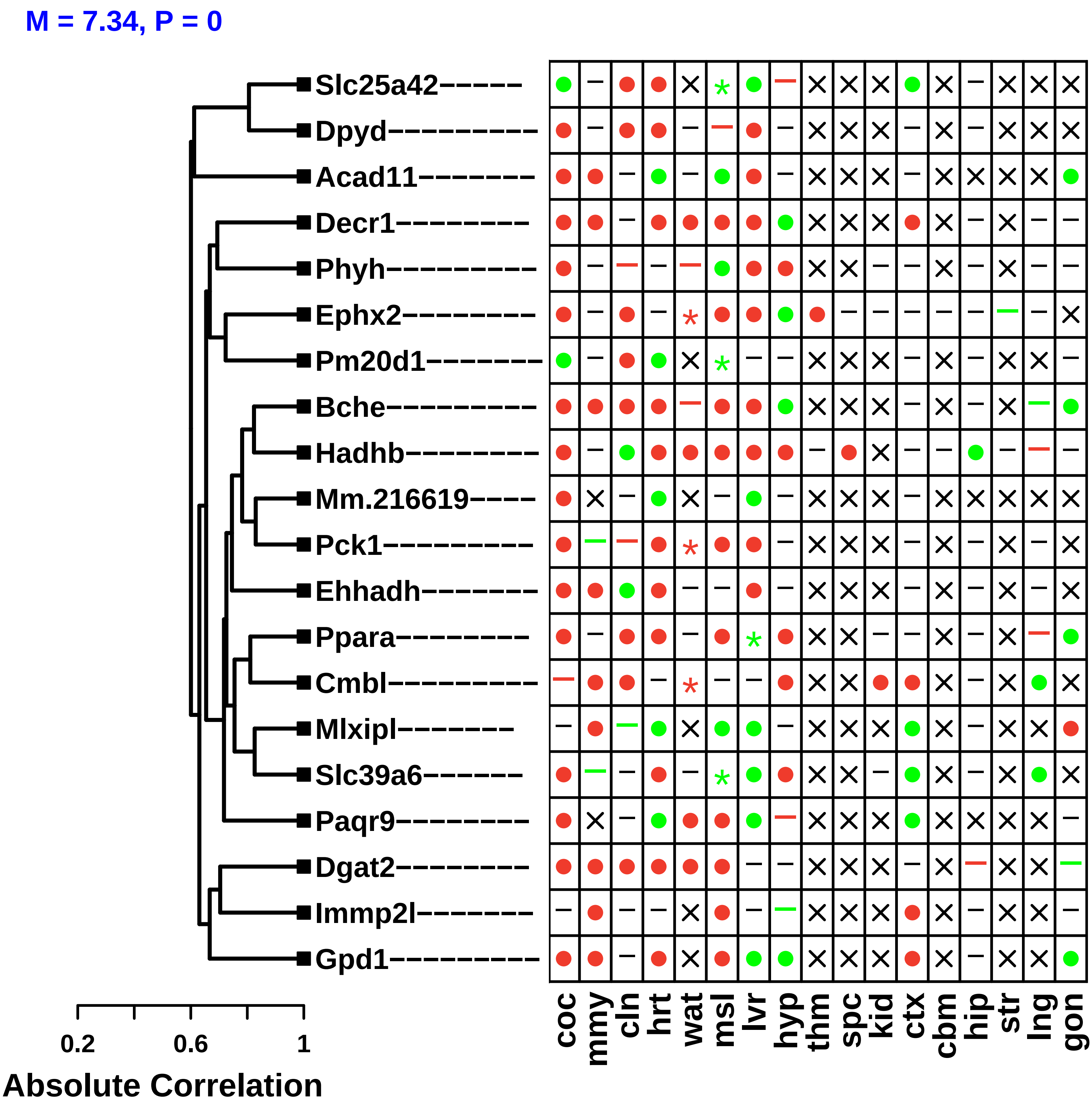

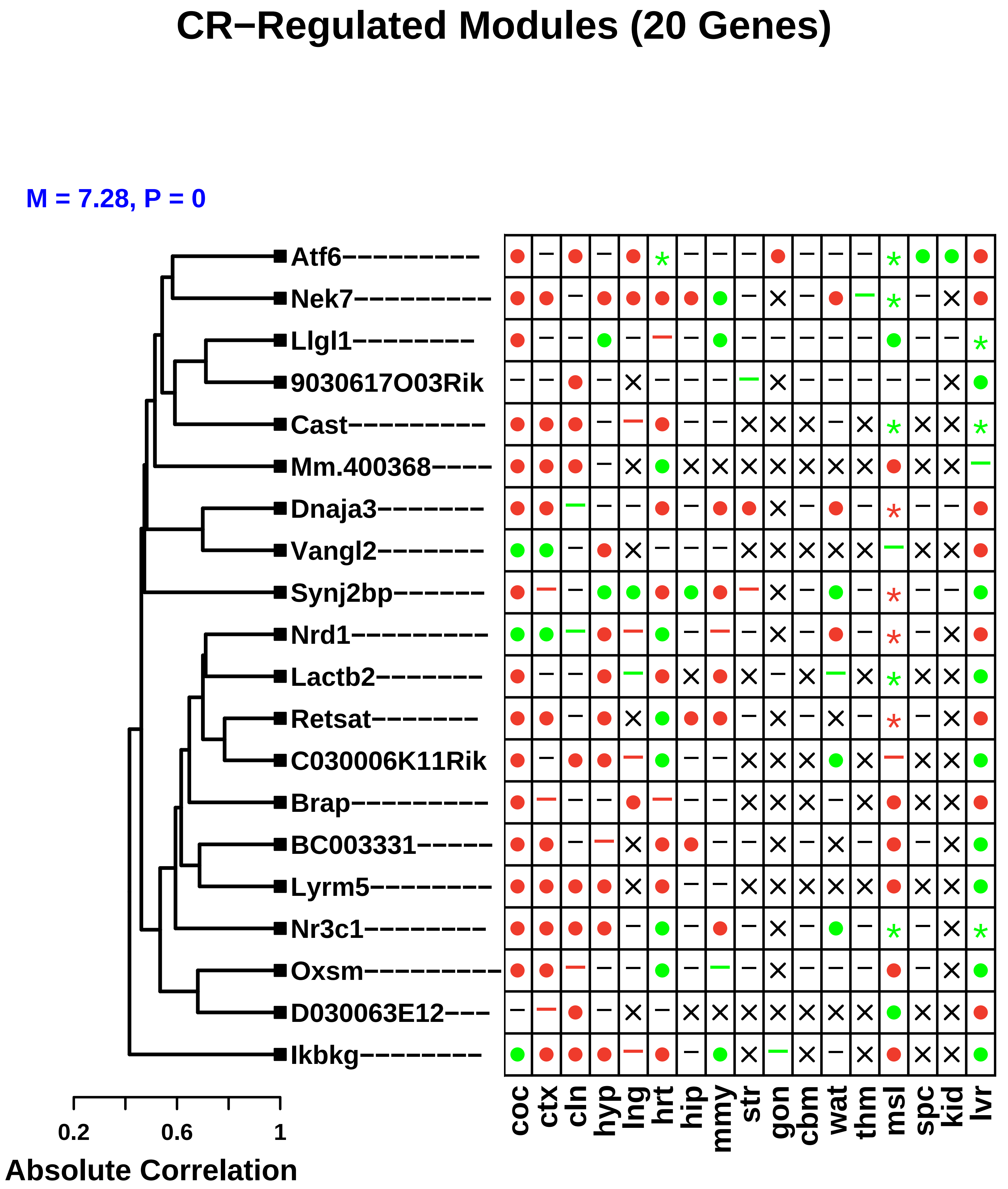
CR−Regulated Modules (20 Genes)
M = 7.28, P = 0
Atf6
Nek7
Llgl1
9030617O03Rik
Cast
Mm.400368
Dnaja3
Vangl2
Synj2bp
Nrd1
Lactb2
Retsat
C030006K11Rik
Brap
BC003331
Lyrm5
Nr3c1
Oxsm
D030063E12
Ikbkg
coc
ctx
cln
hyp
lng
hrt
hip
mmy
str
gon
cbm
wat
thm
msl
spc
kid
lvr
0.2
0.6
1
Absolute Correlation

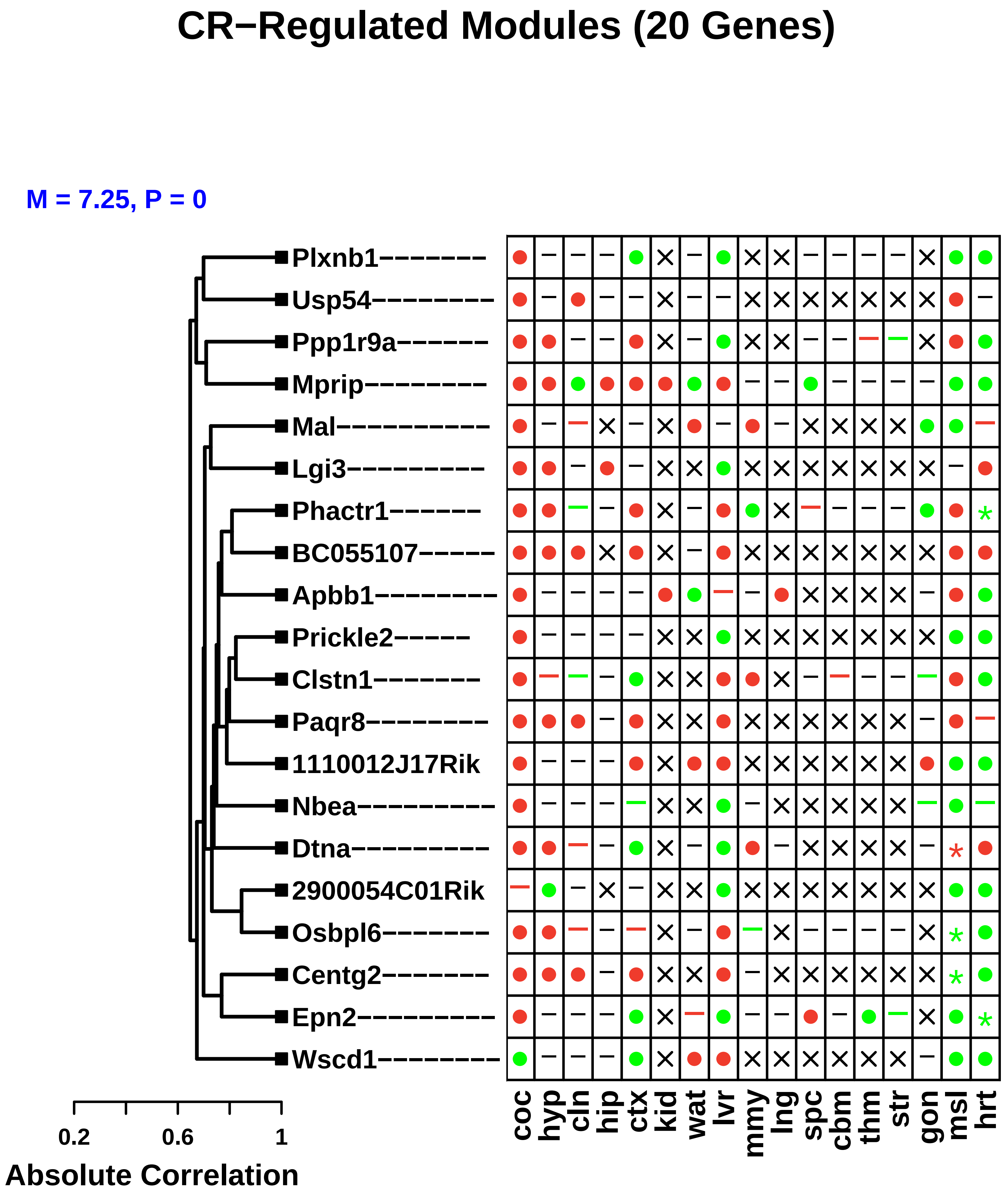

CR−Regulated Modules (20 Genes)
M = 7.25, P = 0
Plxnb1
Usp54
Ppp1r9a
Mprip
Mal
Lgi3
Phactr1
BC055107
Apbb1
Prickle2
Clstn1
Paqr8
1110012J17Rik
Nbea
Dtna
2900054C01Rik
Osbpl6
Centg2
Epn2
Wscd1
coc
hyp
cln
hip
ctx
kid
wat
lvr
mmy
lng
spc
cbm
thm
str
gon
msl
hrt
0.2
0.6
1
Absolute Correlation

CR−Regulated Modules (20 Genes)

M = 7.25, P = 0

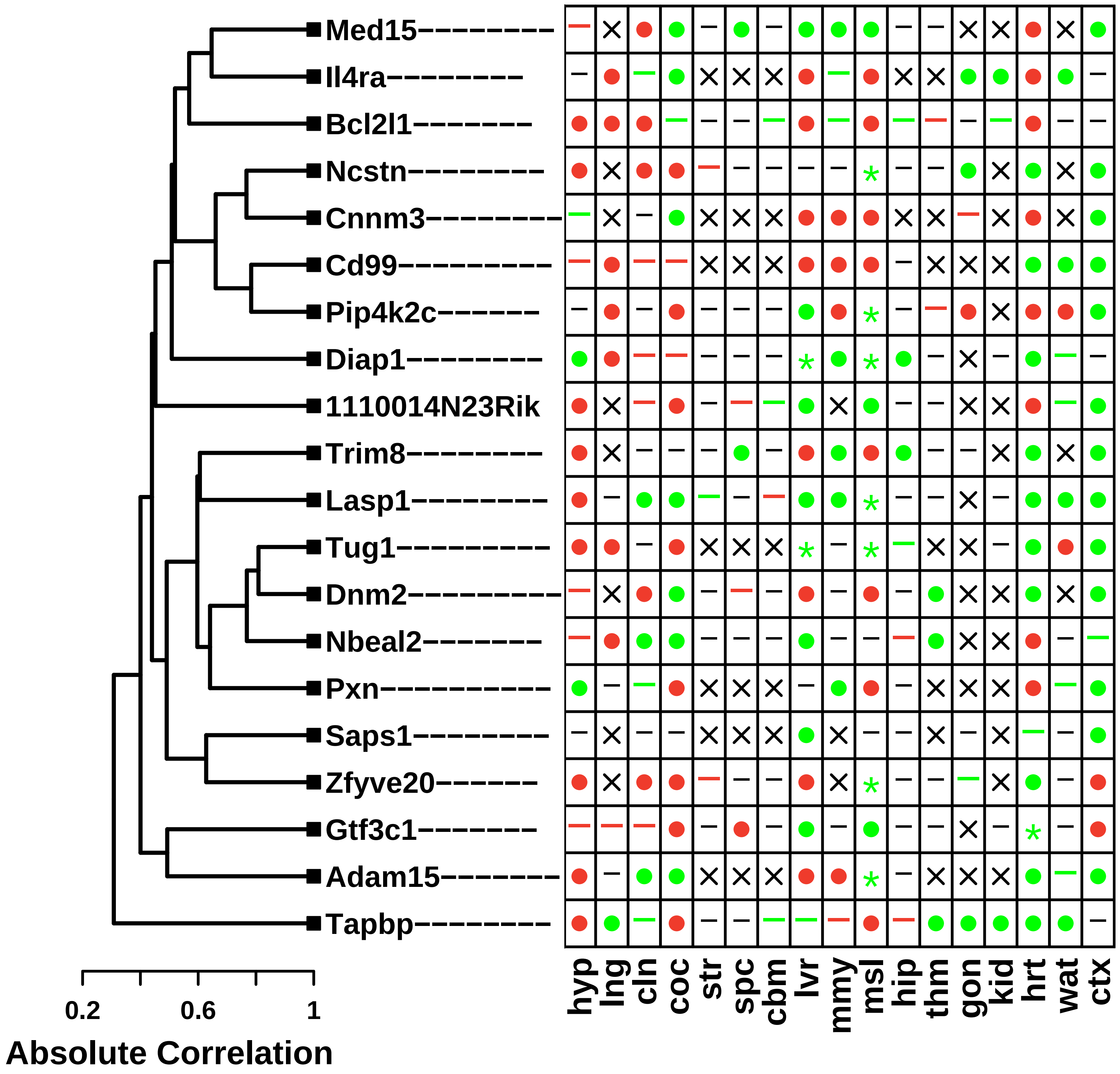

CR−Regulated Modules (20 Genes)

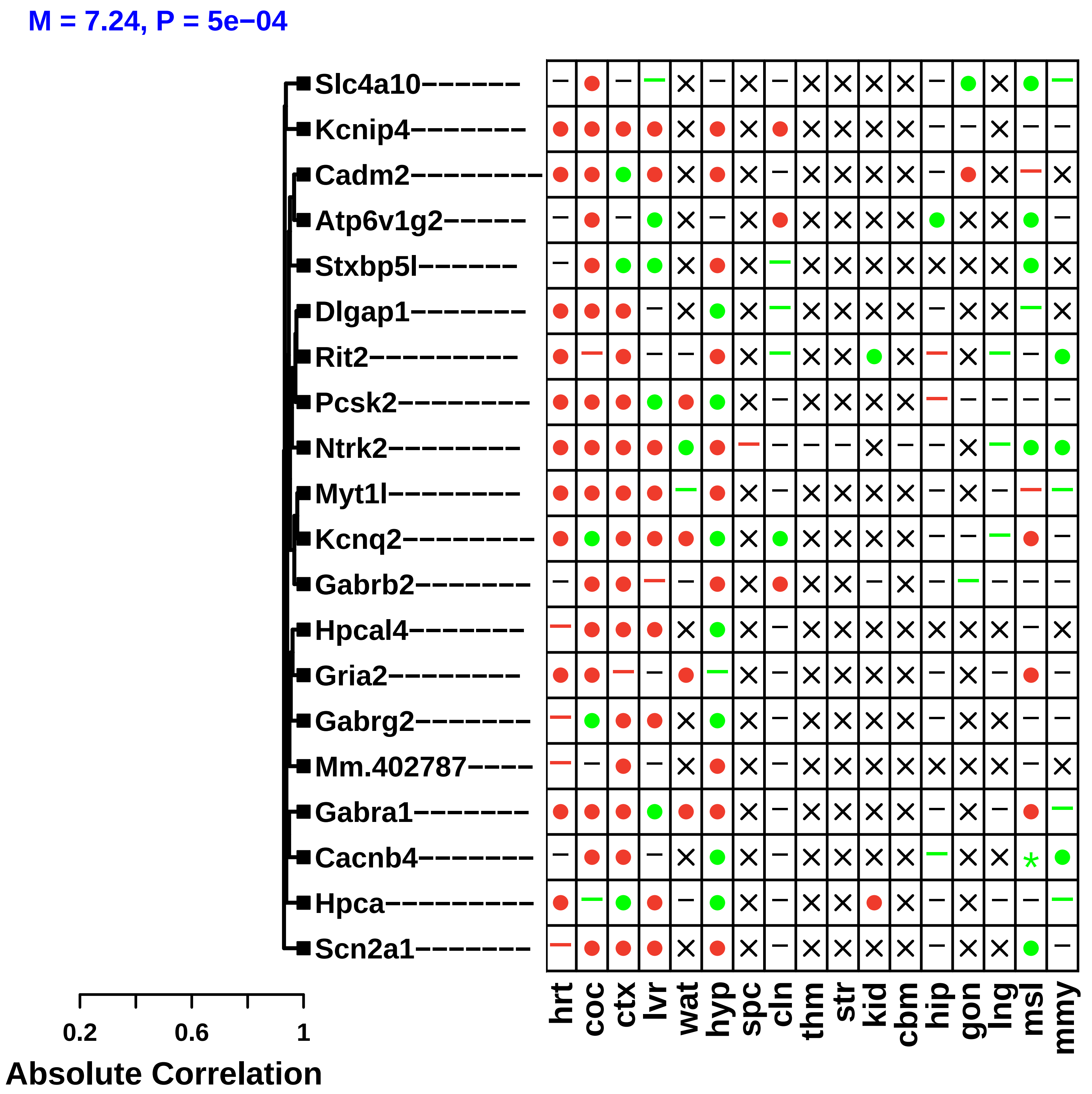

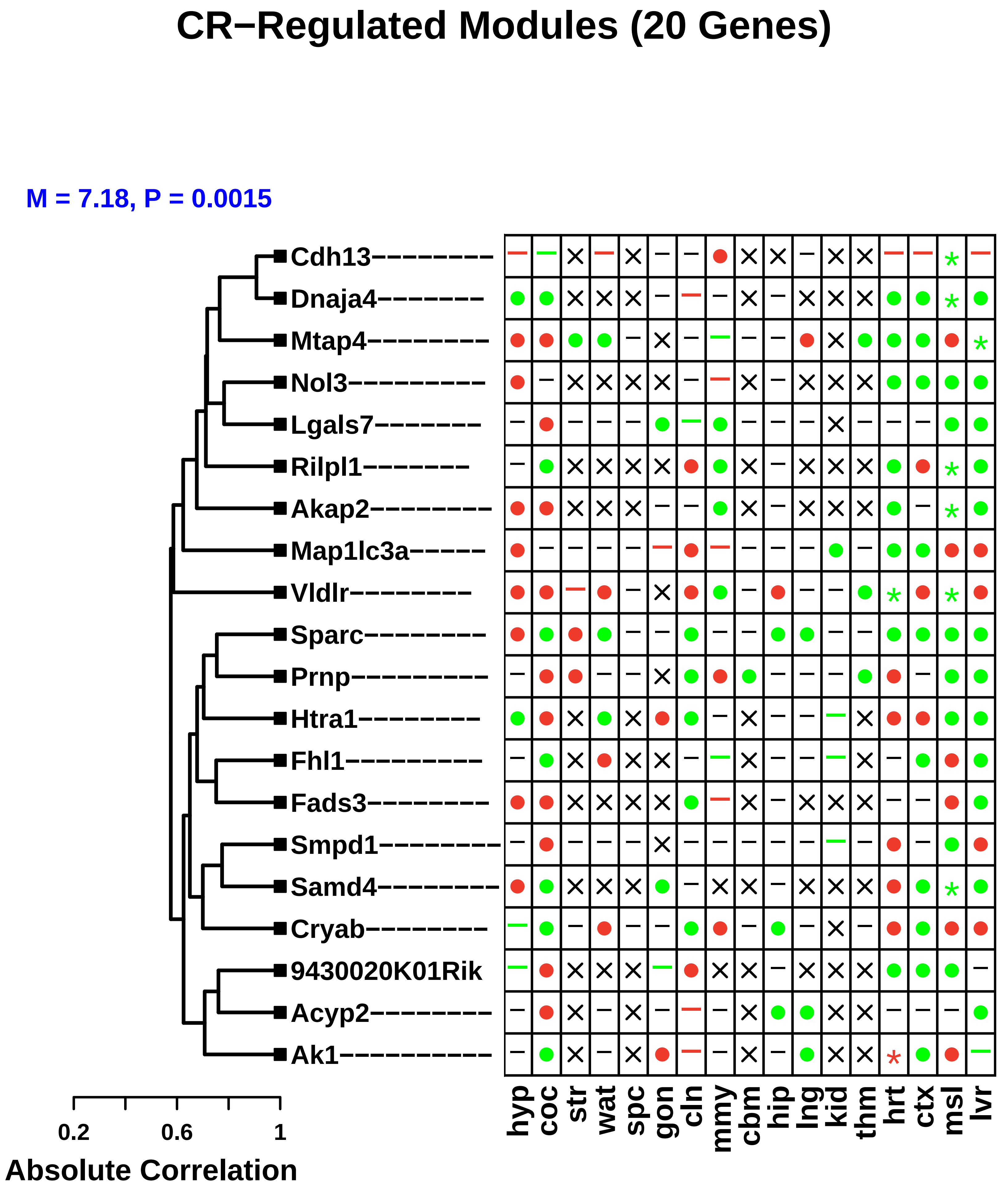
CR−Regulated Modules (20 Genes)
M = 7.18, P = 0.0015
Cdh13
Dnaja4
Mtap4
Nol3
Lgals7
Rilpl1
Akap2
Map1lc3a
Vldlr
Sparc
Prnp
Htra1
Fhl1
Fads3
Smpd1
Samd4
Cryab
9430020K01Rik
Acyp2
Ak1
hyp
coc
str
wat
spc
gon
cln
mmy
cbm
hip
lng
kid
thm
hrt
ctx
msl
lvr
0.2
0.6
1
Absolute Correlation

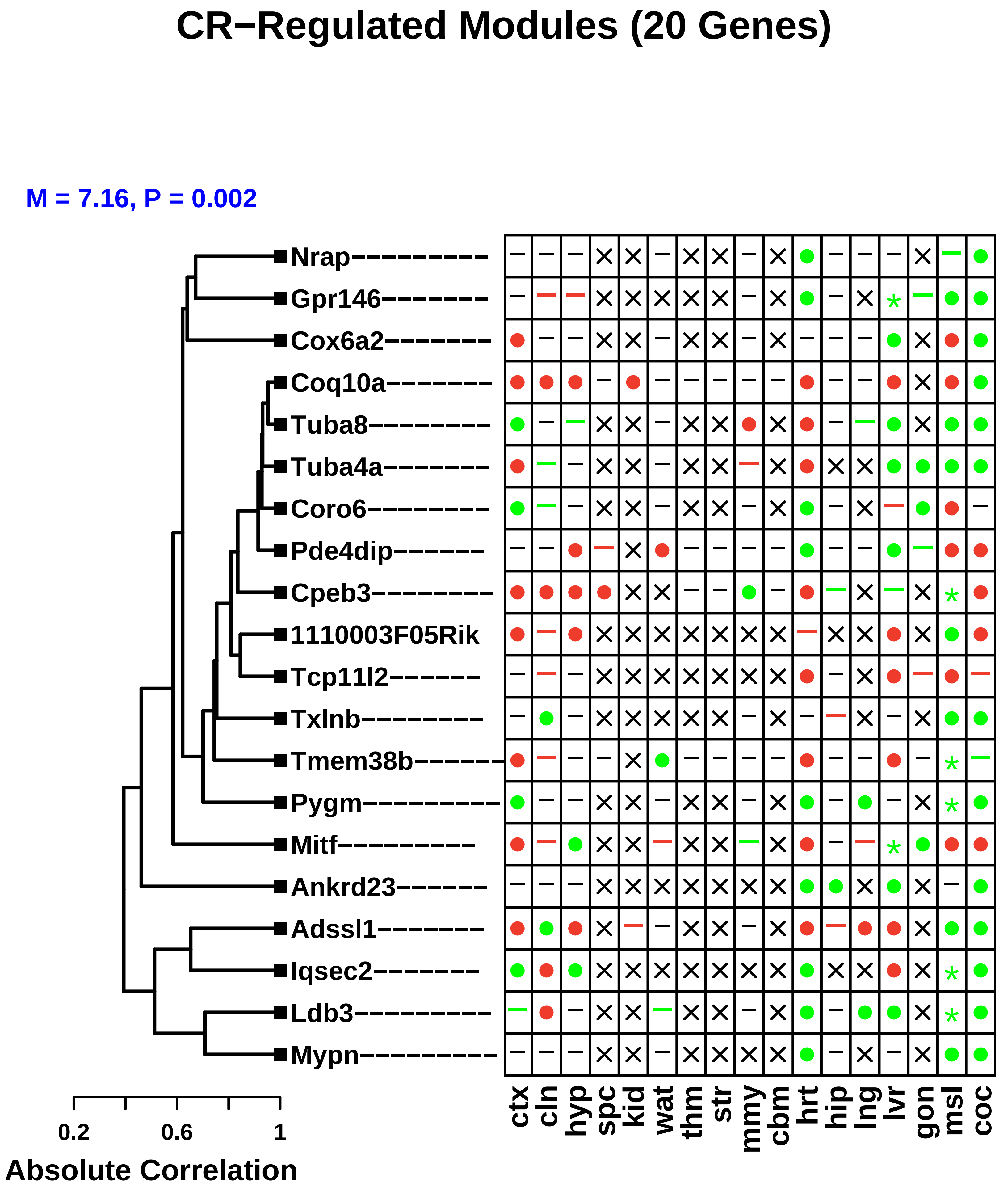
CR−Regulated Modules (20 Genes)
M = 7.16, P = 0.002
Nrap
Gpr146
Cox6a2
Coq10a
Tuba8
Tuba4a
Coro6
Pde4dip
Cpeb3
1110003F05Rik
Tcp11l2
Txlnb
Tmem38b
Pygm
Mitf
Ankrd23
Adssl1
Iqsec2
Ldb3
Mypn
ctx
cln
hyp
spc
kid
wat
thm
str
mmy
cbm
hrt
hip
lng
lvr
gon
msl
coc
0.2
0.6
1
Absolute Correlation

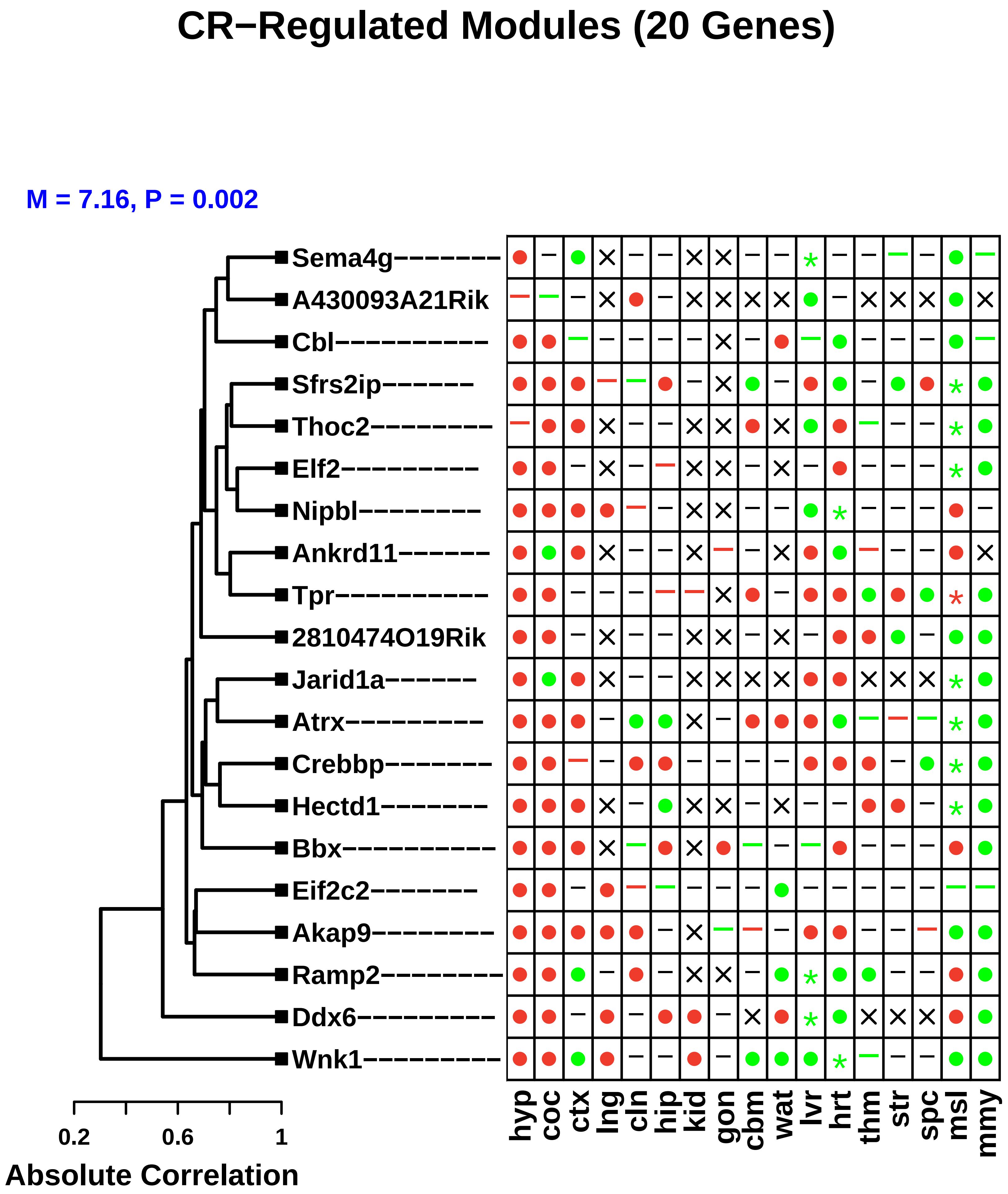
CR−Regulated Modules (20 Genes)
M = 7.16, P = 0.002
Sema4g
A430093A21Rik
Cbl
Sfrs2ip
Thoc2
Elf2
Nipbl
Ankrd11
Tpr
2810474O19Rik
Jarid1a
Atrx
Crebbp
Hectd1
Bbx
Eif2c2
Akap9
Ramp2
Ddx6
Wnk1
hyp
coc
ctx
lng
cln
hip
kid
gon
cbm
wat
lvr
hrt
thm
str
spc
msl
mmy
0.2
0.6
1
Absolute Correlation

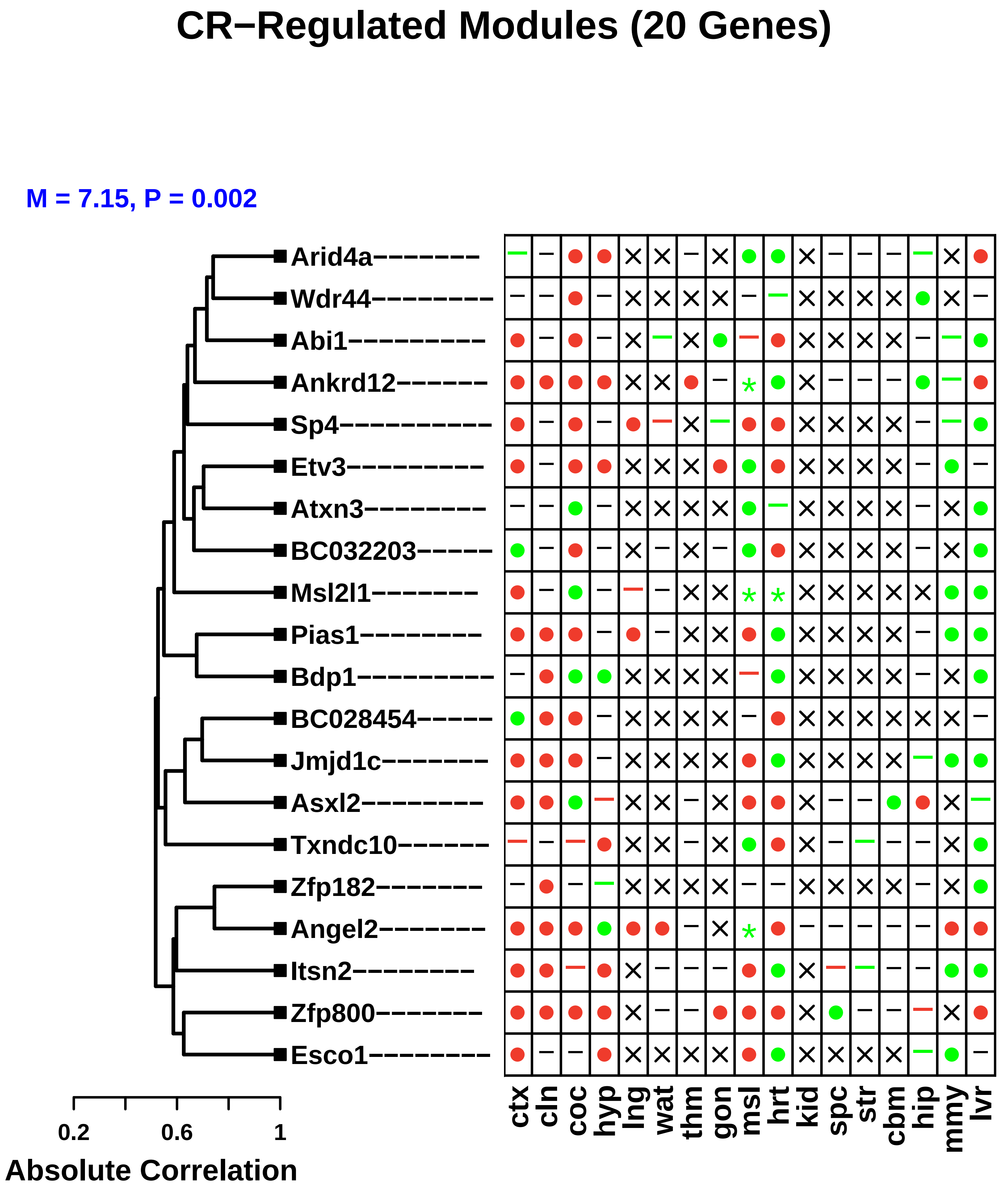
CR−Regulated Modules (20 Genes)
M = 7.15, P = 0.002
Arid4a
Wdr44
Abi1
Ankrd12
Sp4
Etv3
Atxn3
BC032203
Msl2l1
Pias1
Bdp1
BC028454
Jmjd1c
Asxl2
Txndc10
Zfp182
Angel2
Itsn2
Zfp800
Esco1
ctx
cln
coc
hyp
lng
wat
thm
gon
msl
hrt
kid
spc
str
cbm
hip
mmy
lvr
0.2
0.6
1
Absolute Correlation

CR−Regulated Modules (20 Genes)

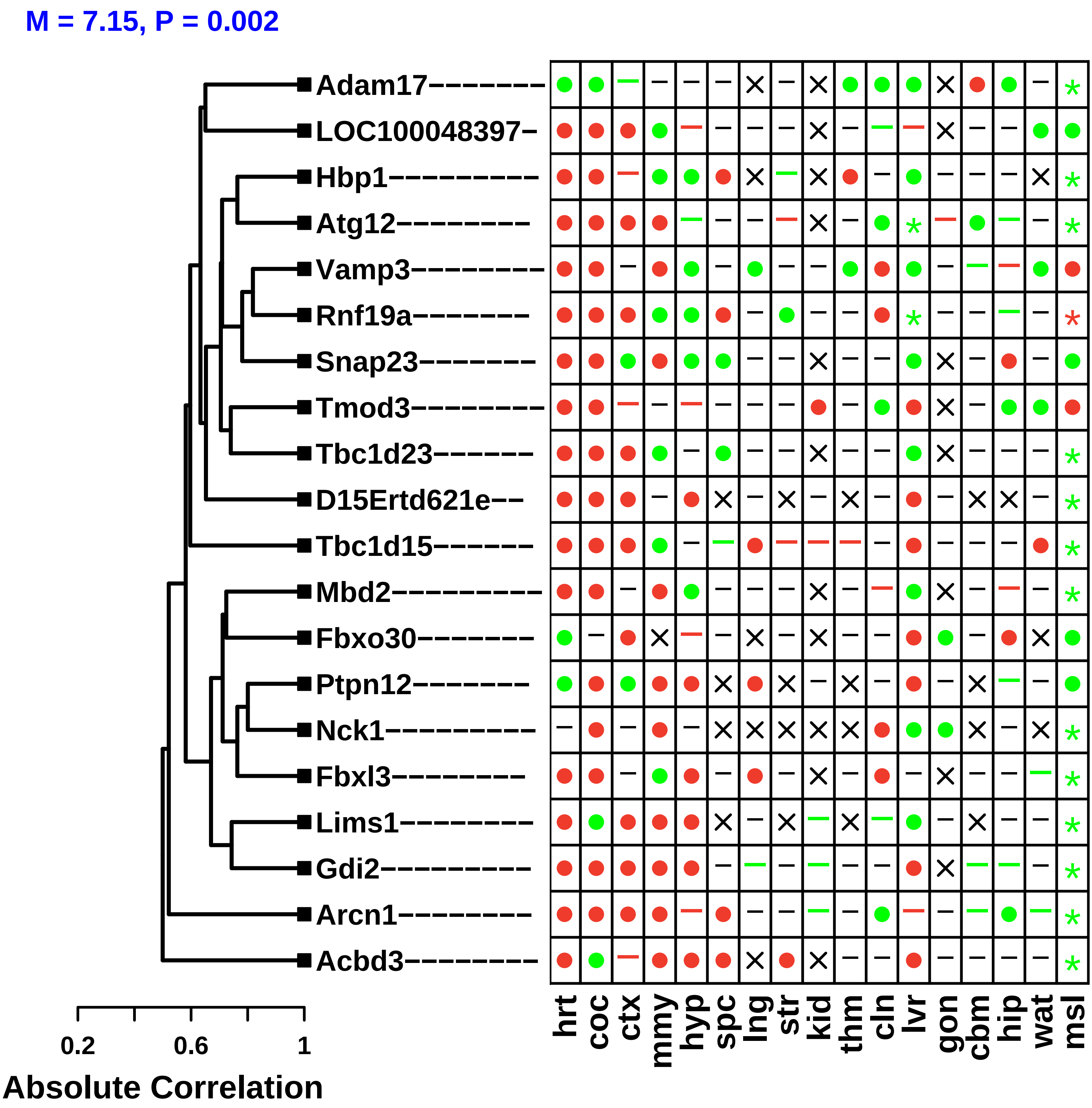

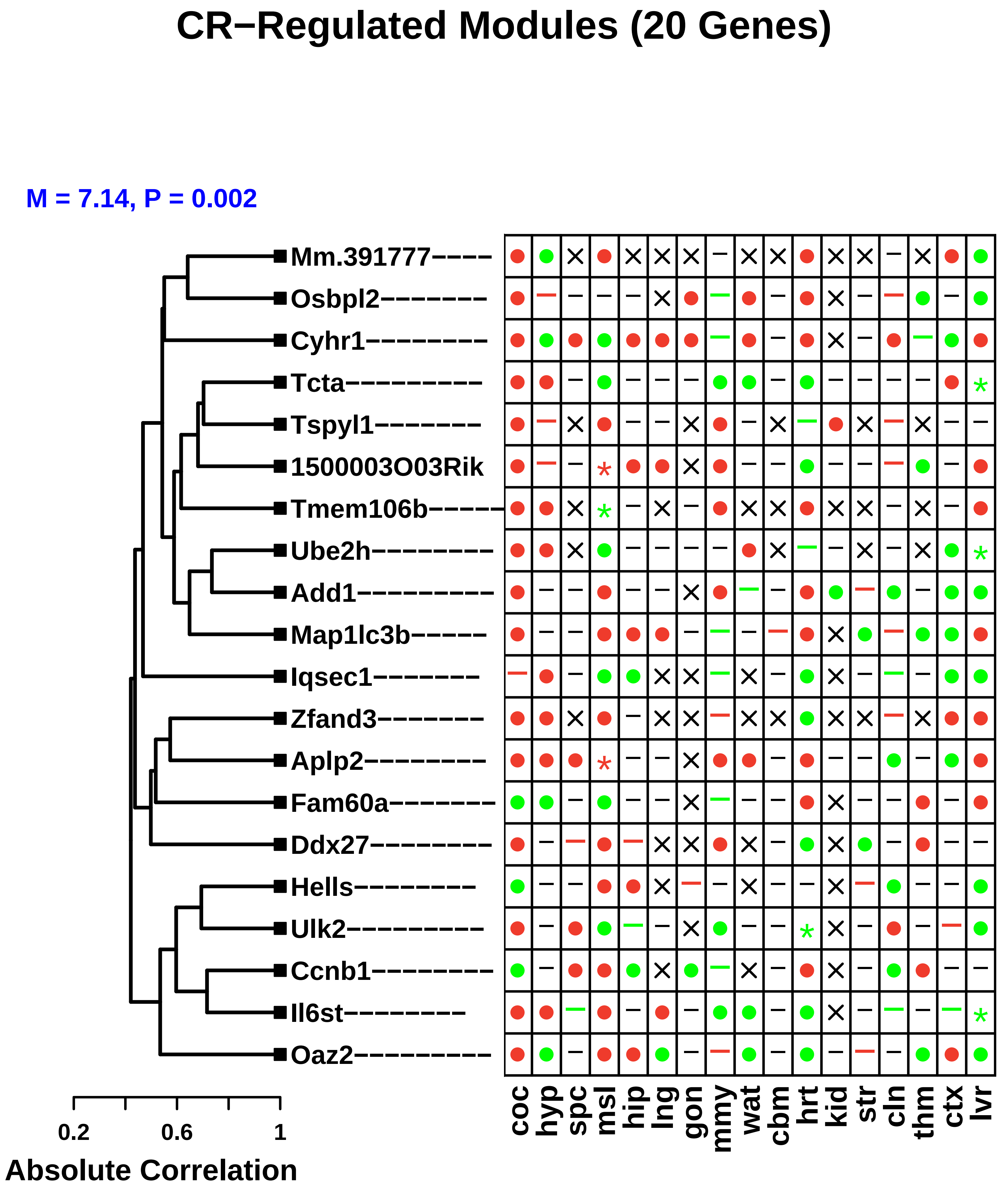
CR−Regulated Modules (20 Genes)
M = 7.14, P = 0.002
Mm.391777
Osbpl2
Cyhr1
Tcta
Tspyl1
1500003O03Rik
Tmem106b
Ube2h
Add1
Map1lc3b
Iqsec1
Zfand3
Aplp2
Fam60a
Ddx27
Hells
Ulk2
Ccnb1
Il6st
Oaz2
coc
hyp
spc
msl
hip
lng
gon
mmy
wat
cbm
hrt
kid
str
cln
thm
ctx
lvr
0.2
0.6
1
Absolute Correlation

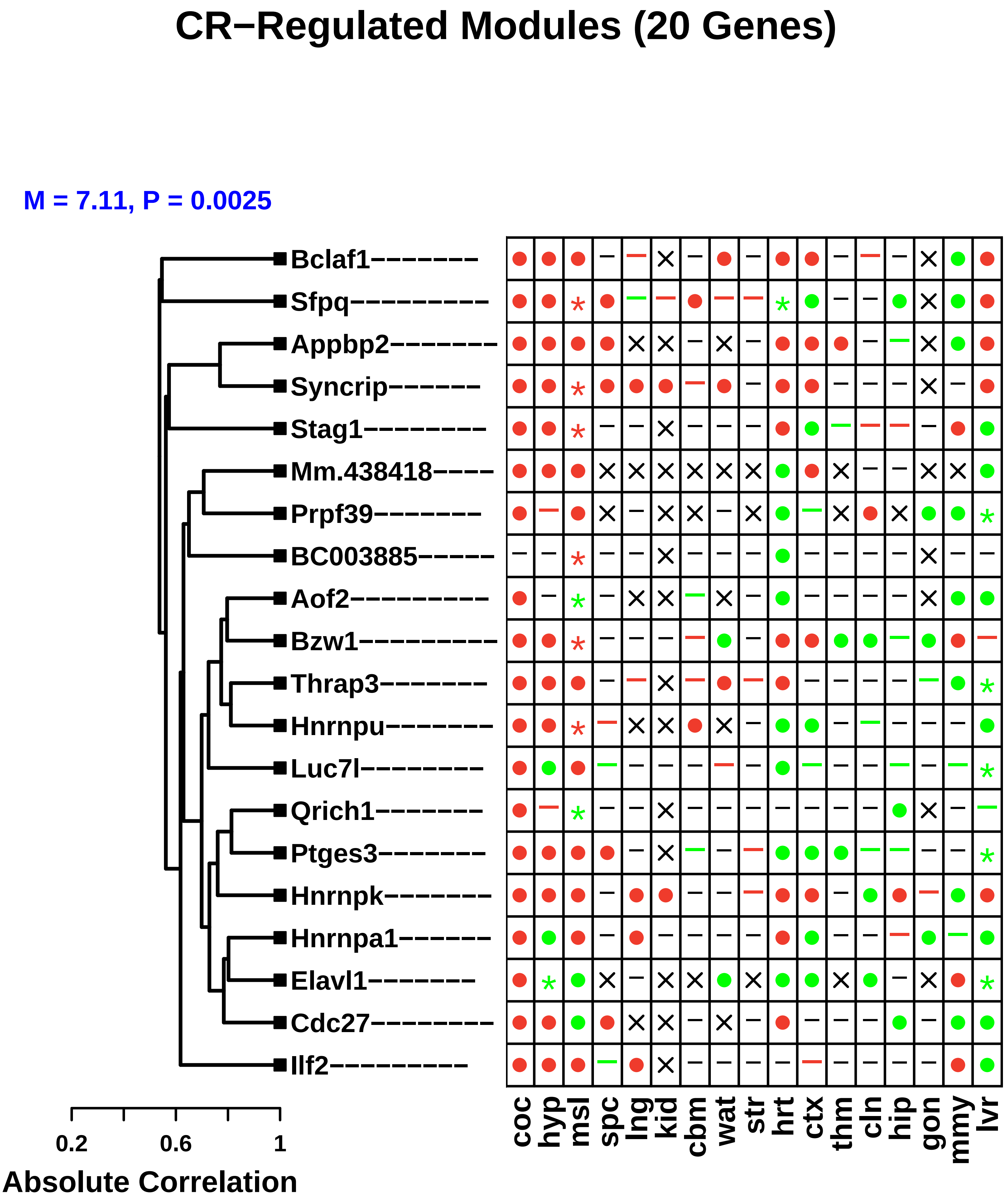
CR−Regulated Modules (20 Genes)
M = 7.11, P = 0.0025
Bclaf1
Sfpq
Appbp2
Syncrip
Stag1
Mm.438418
Prpf39
BC003885
Aof2
Bzw1
Thrap3
Hnrnpu
Luc7l
Qrich1
Ptges3
Hnrnpk
Hnrnpa1
Elavl1
Cdc27
Ilf2
coc
hyp
msl
spc
lng
kid
cbm
wat
str
hrt
ctx
thm
cln
hip
gon
mmy
lvr
0.2
0.6
1
Absolute Correlation

CR−Regulated Modules (20 Genes)

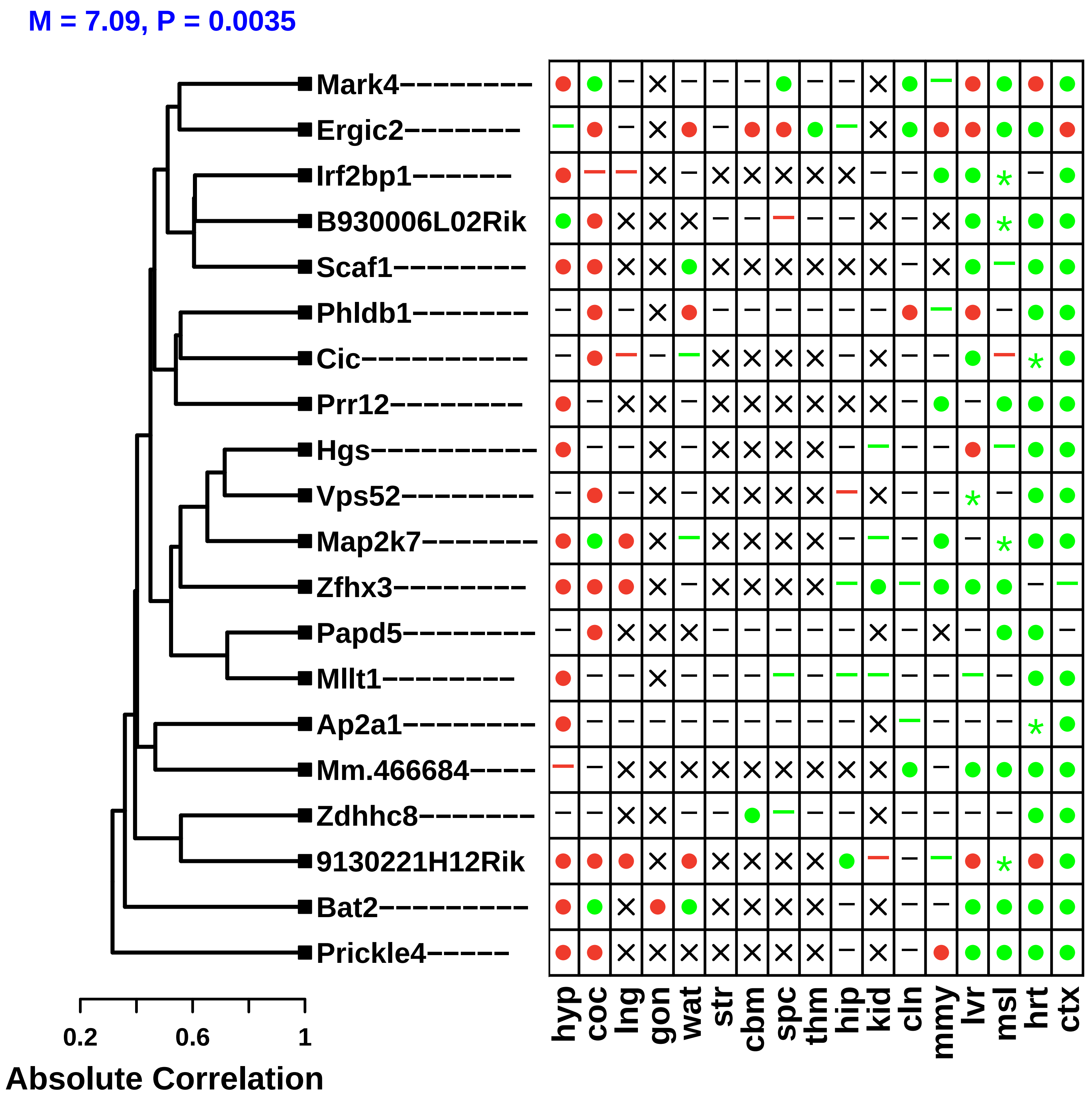

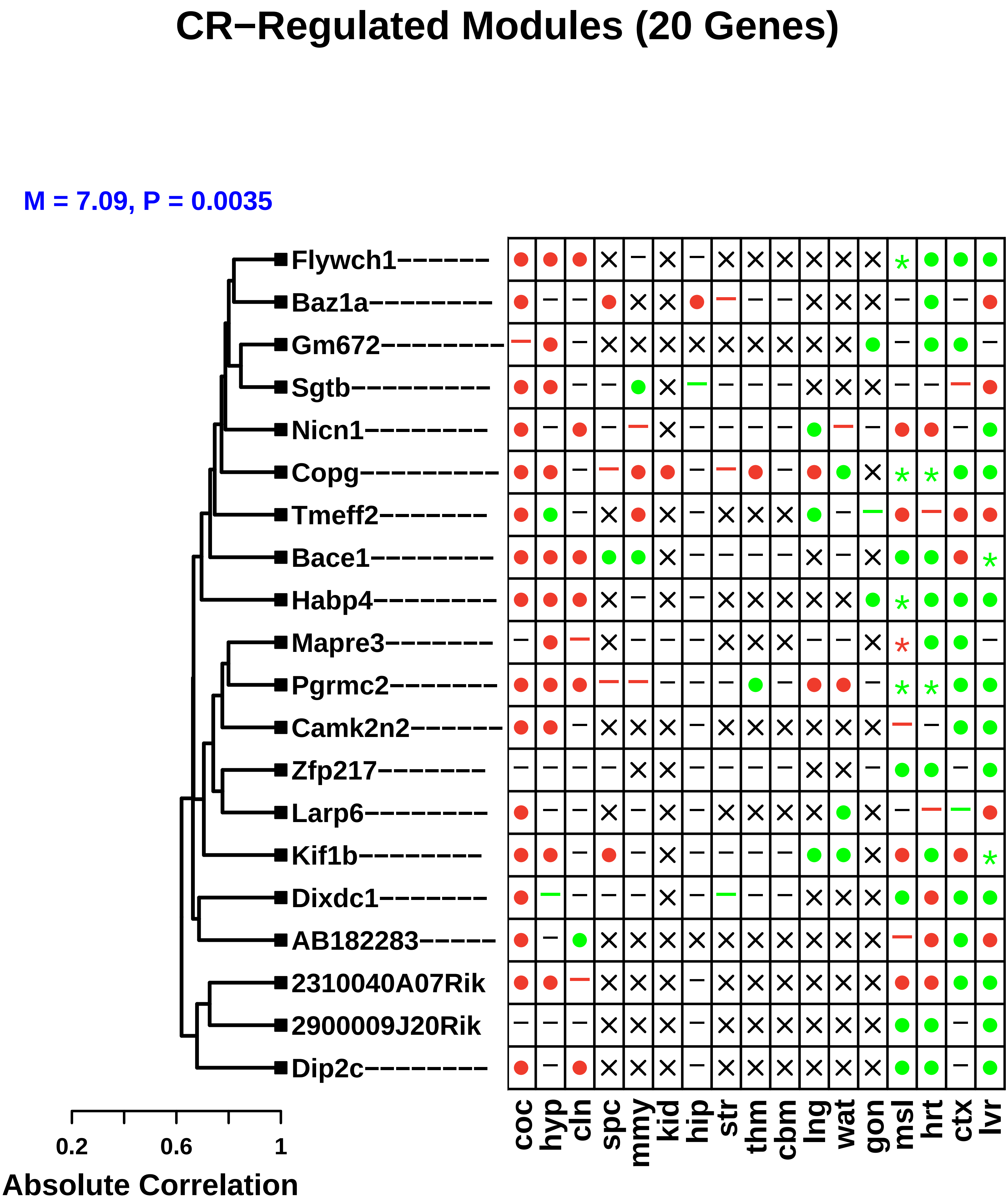
CR−Regulated Modules (20 Genes)
M = 7.09, P = 0.0035
Flywch1
Baz1a
Gm672
Sgtb
Nicn1
Copg
Tmeff2
Bace1
Habp4
Mapre3
Pgrmc2
Camk2n2
Zfp217
Larp6
Kif1b
Dixdc1
AB182283
2310040A07Rik
2900009J20Rik
Dip2c
coc
hyp
cln
spc
mmy
kid
hip
str
thm
cbm
lng
wat
gon
msl
hrt
ctx
lvr
0.2
0.6
1
Absolute Correlation

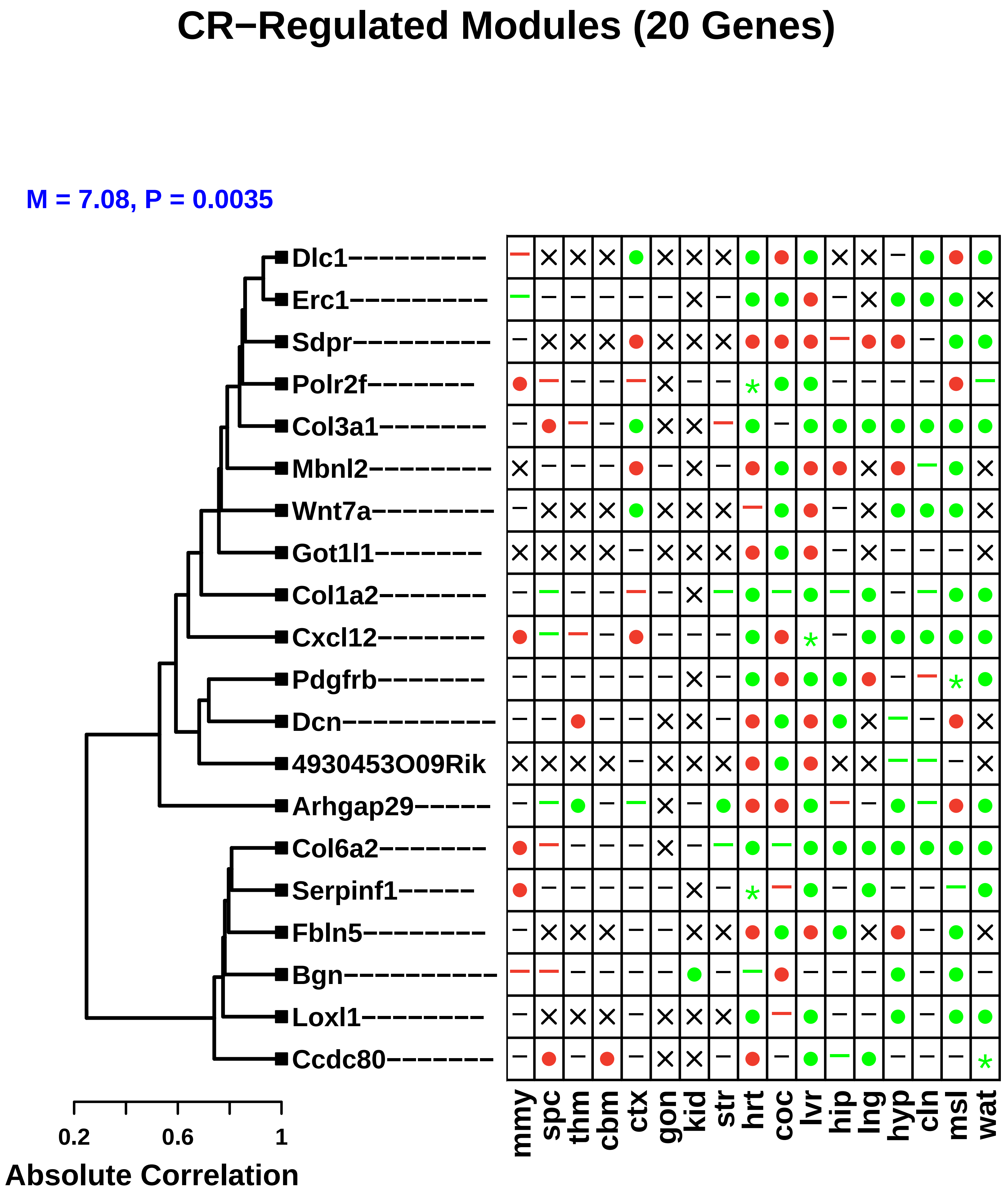

CR−Regulated Modules (20 Genes)
M = 7.08, P = 0.0035
Dlc1
Erc1
Sdpr
Polr2f
Col3a1
Mbnl2
Wnt7a
Got1l1
Col1a2
Cxcl12
Pdgfrb
Dcn
4930453O09Rik
Arhgap29
Col6a2
Serpinf1
Fbln5
Bgn
Loxl1
Ccdc80
mmy spc thm cbm ctx gon kid str hrt coc lvr hip lng hyp cln msl wat
0.2
0.6
1
Absolute Correlation

CR−Regulated Modules (20 Genes)

M = 7.05, P = 0.007

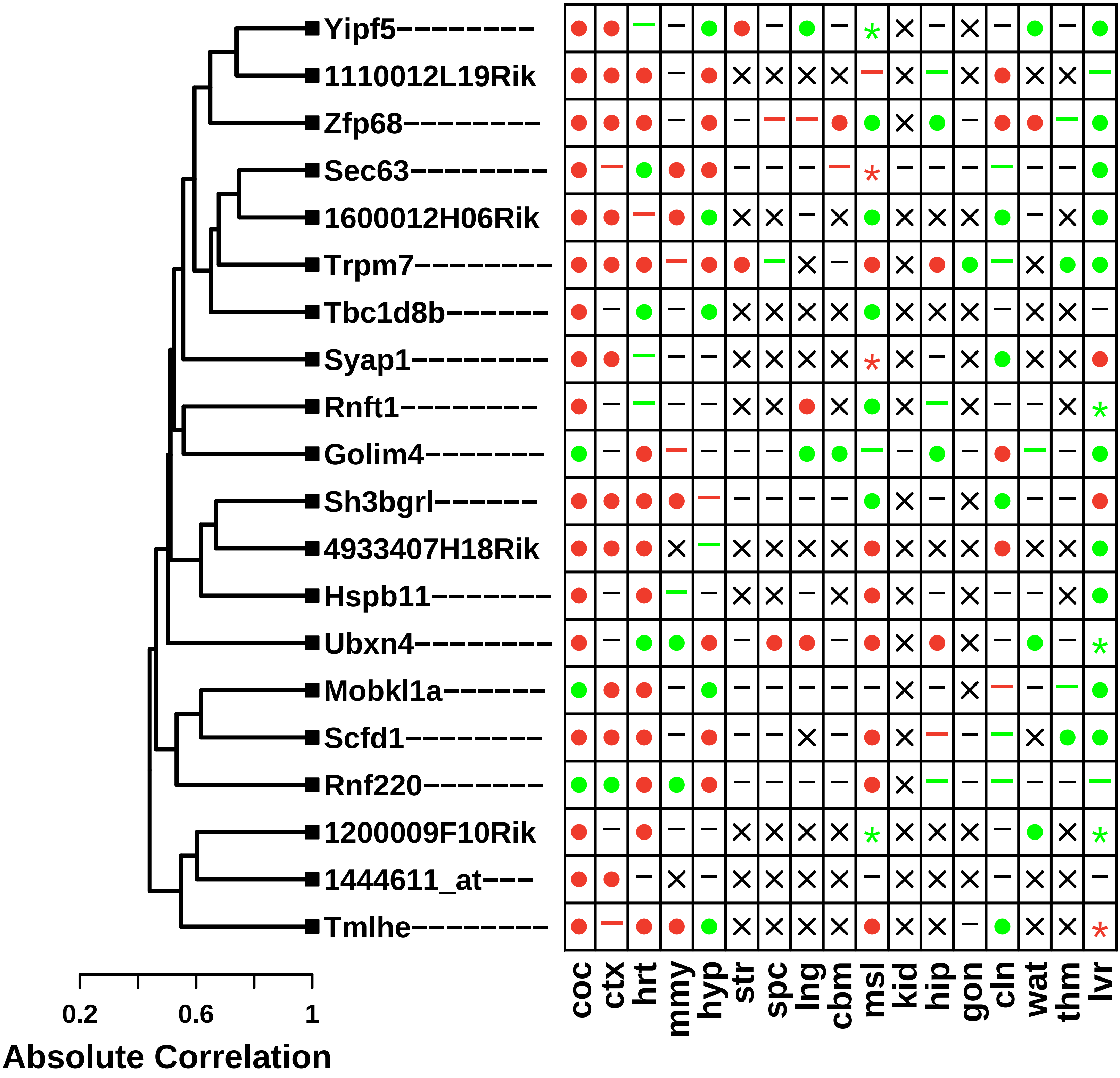

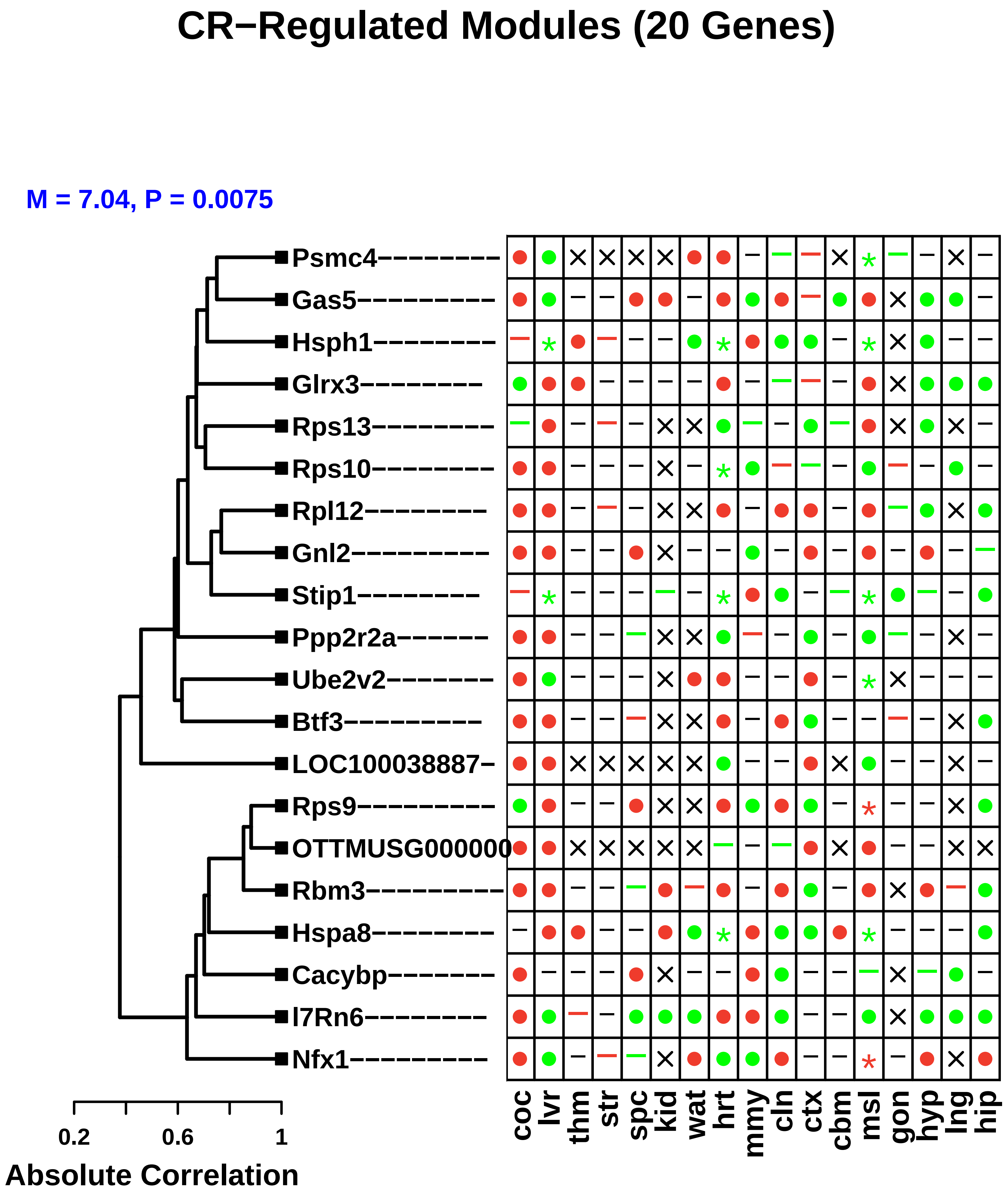

CR−Regulated Modules (20 Genes)
M = 7.04, P = 0.0075
Psmc4
Gas5
Hsph1
Glrx3
Rps13
Rps10
Rpl12
Gnl2
Stip1
Ppp2r2a
Ube2v2
Btf3
LOC100038887
Rps9
OTTMUSG000000
Rbm3
Hspa8
Cacybp
I7Rn6
Nfx1
coc
lvr
thm
str
spc
kid
wat
hrt
mmy
cln
ctx
cbm
msl
gon
hyp
lng
hip
0.2
0.6
1
Absolute Correlation

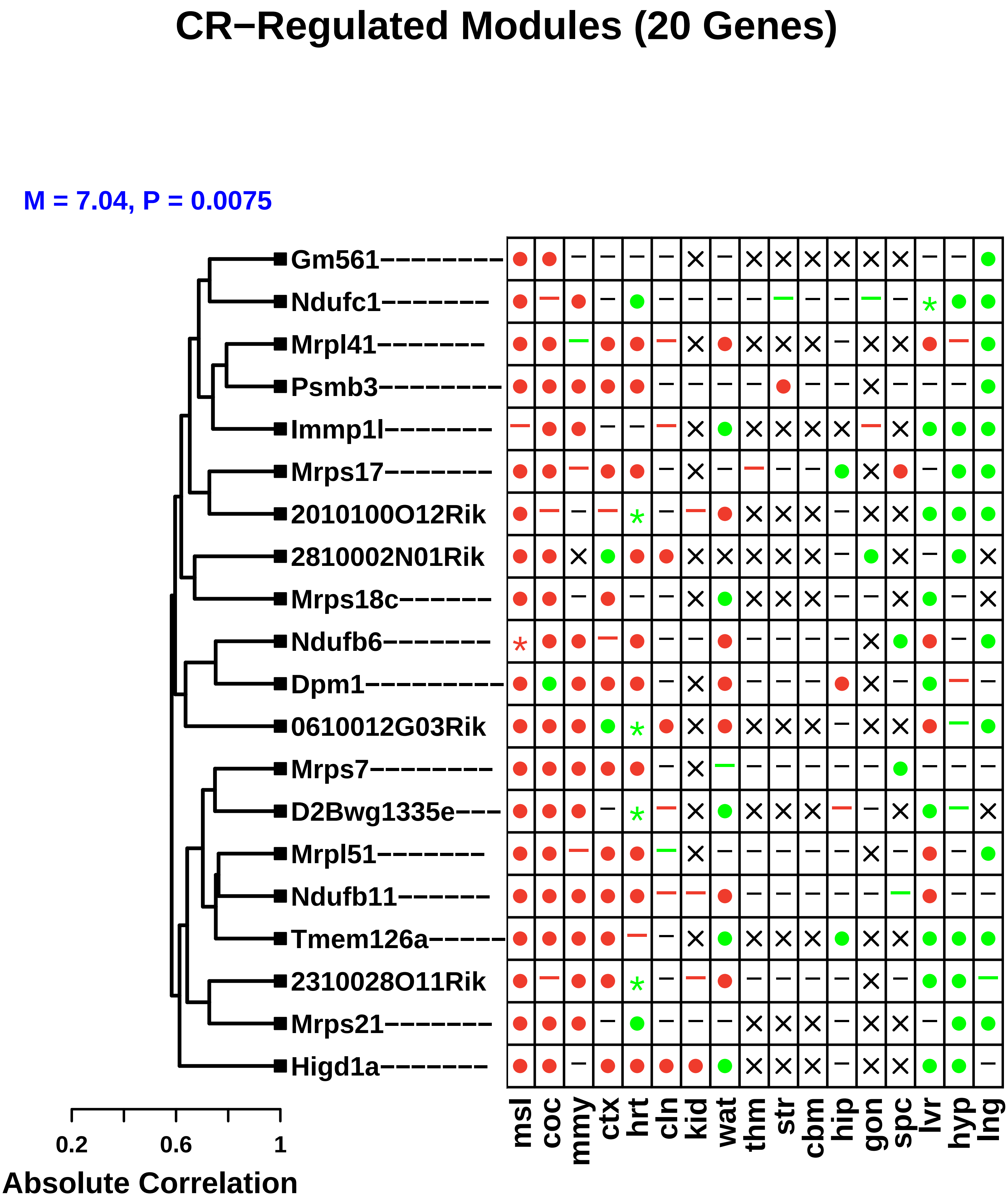
CR−Regulated Modules (20 Genes)
M = 7.04, P = 0.0075
Gm561
Ndufc1
Mrpl41
Psmb3
Immp1l
Mrps17
2010100O12Rik
2810002N01Rik
Mrps18c
Ndufb6
Dpm1
0610012G03Rik
Mrps7
D2Bwg1335e
Mrpl51
Ndufb11
Tmem126a
2310028O11Rik
Mrps21
Higd1a
msl
coc
mmy
ctx
hrt
cln
kid
wat
thm
str
cbm
hip
gon
spc
lvr
hyp
lng
0.2
0.6
1
Absolute Correlation

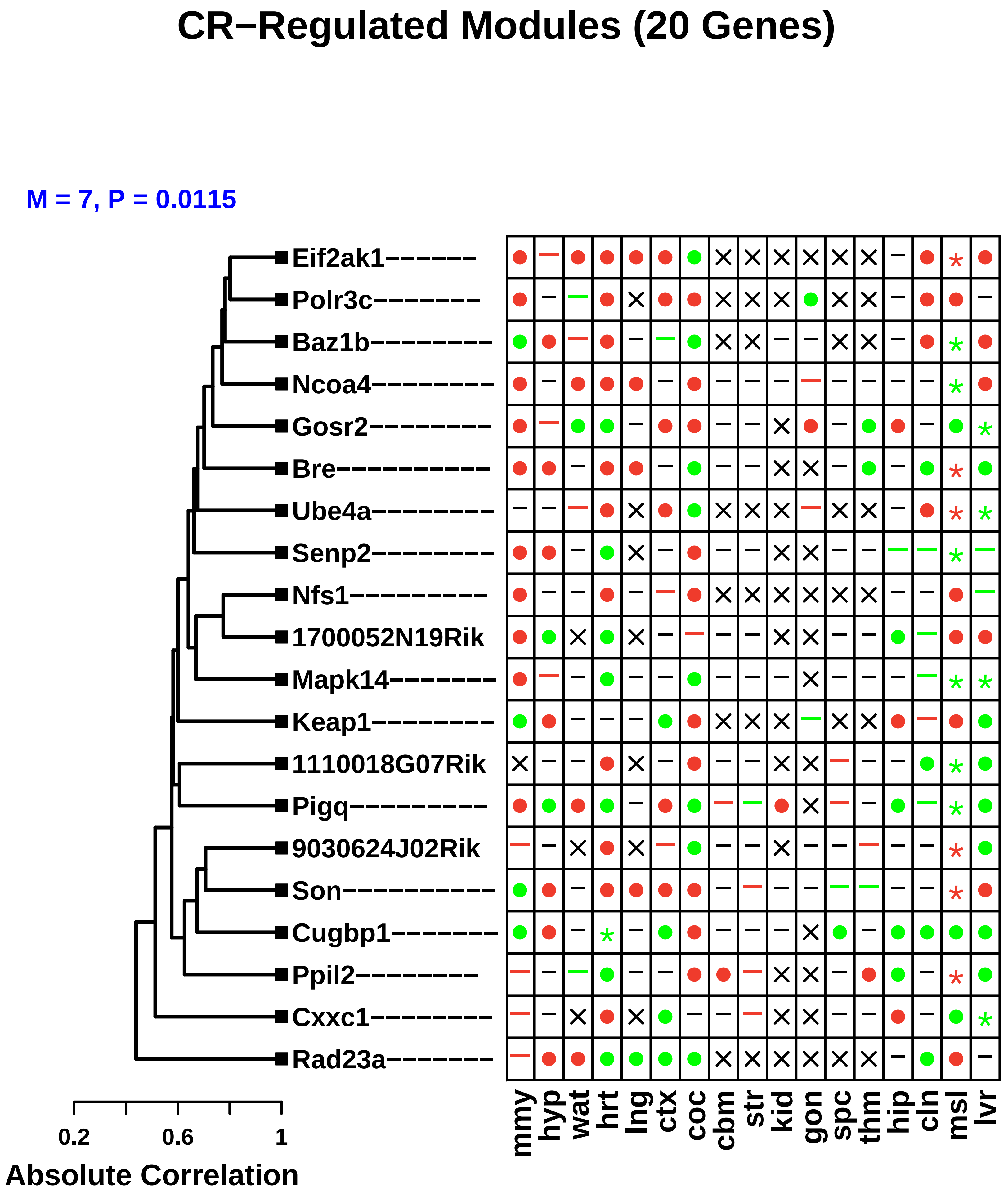
CR−Regulated Modules (20 Genes)
M = 7, P = 0.0115
Eif2ak1
Polr3c
Baz1b
Ncoa4
Gosr2
Bre
Ube4a
Senp2
Nfs1
1700052N19Rik
Mapk14
Keap1
1110018G07Rik
Pigq
9030624J02Rik
Son
Cugbp1
Ppil2
Cxxc1
Rad23a
mmy
hyp
wat
hrt
lng
ctx
coc
cbm
str
kid
gon
spc
thm
hip
cln
msl
lvr
0.2
0.6
1
Absolute Correlation

CR−Regulated Modules (20 Genes)

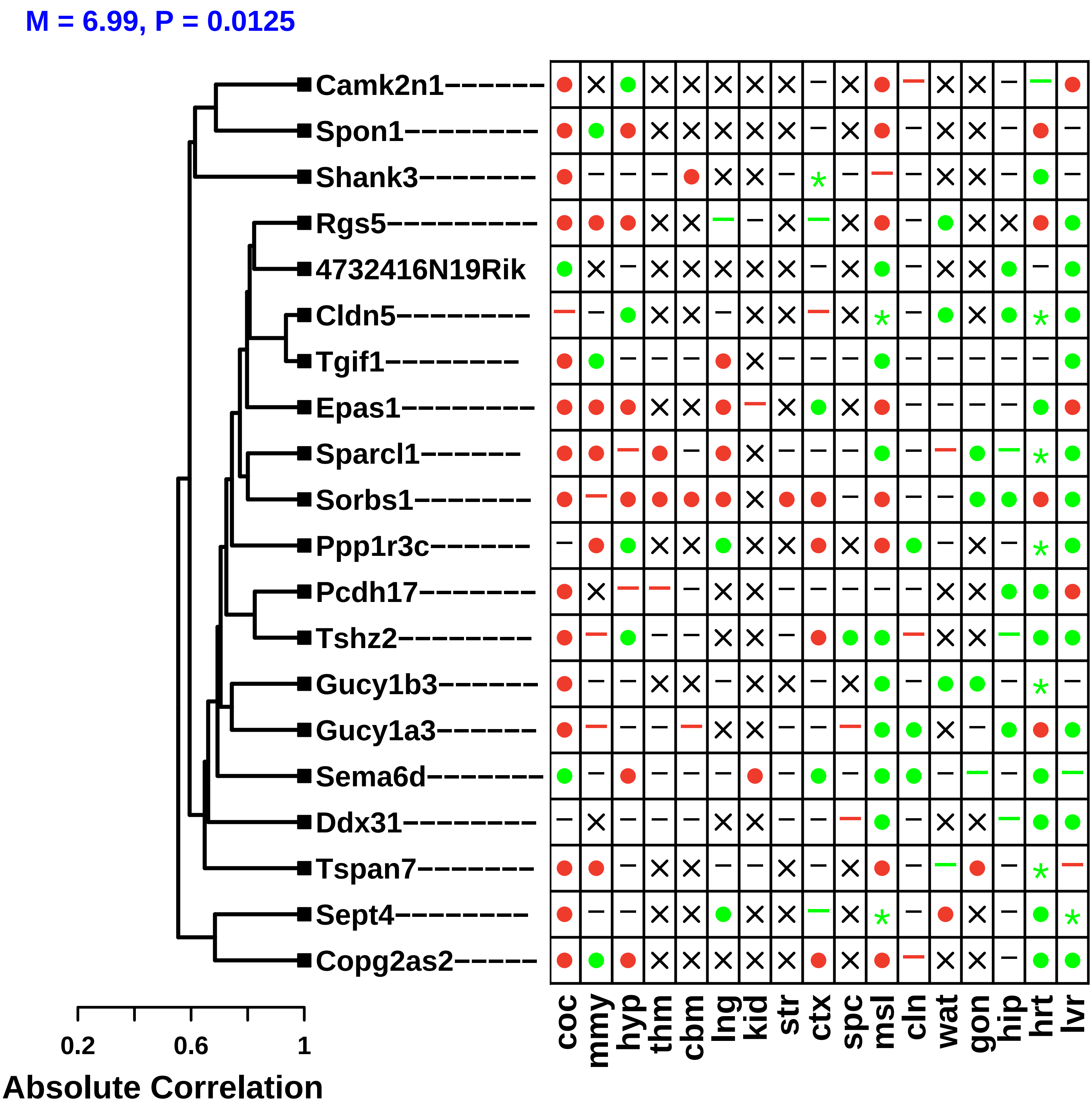

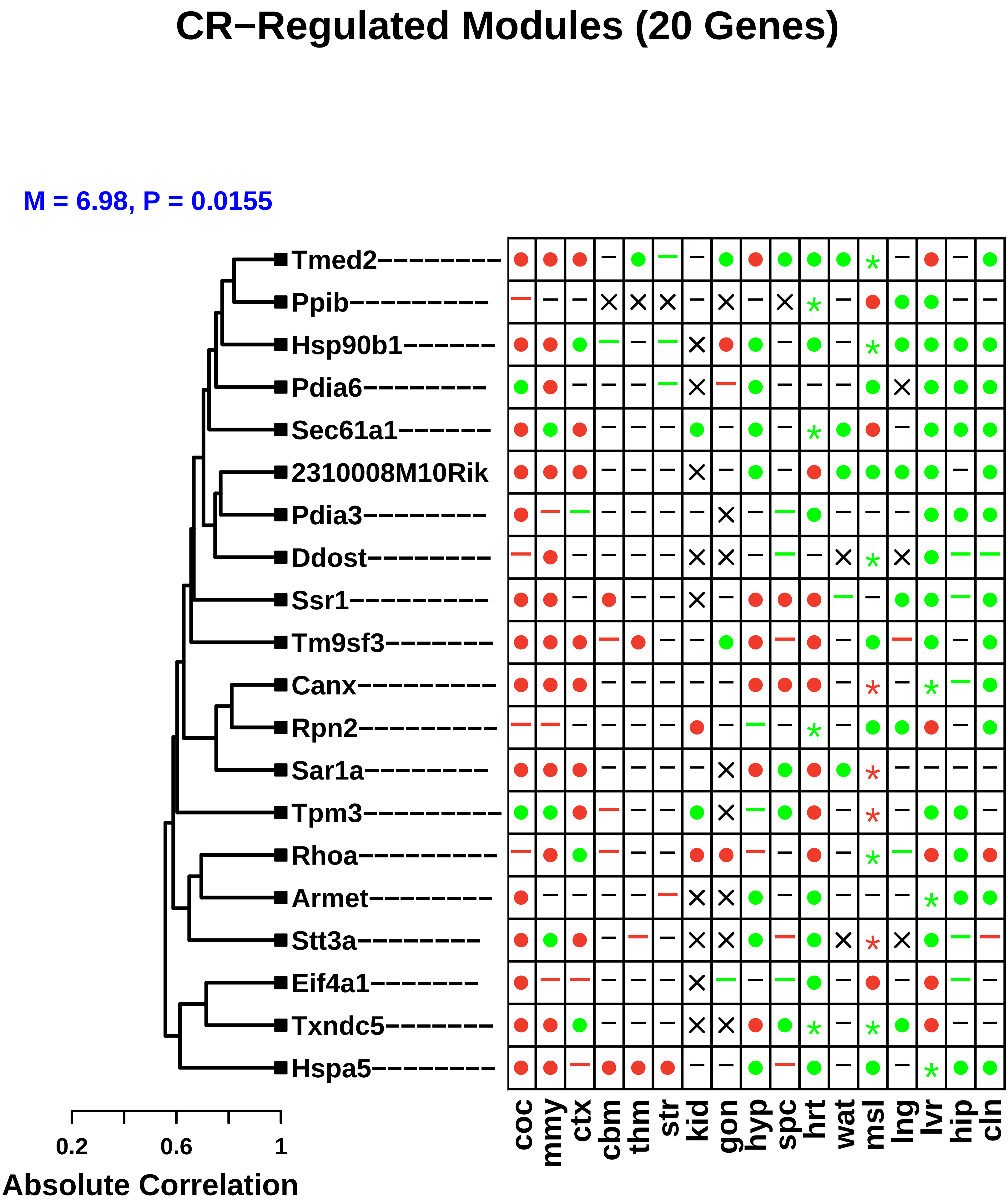
CR−Regulated Modules (20 Genes)
M = 6.98, P = 0.0155
Tmed2
Ppib
Hsp90b1
Pdia6
Sec61a1
2310008M10Rik
Pdia3
Ddost
Ssr1
Tm9sf3
Canx
Rpn2
Sar1a
Tpm3
Rhoa
Armet
Stt3a
Eif4a1
Txndc5
Hspa5
coc
mmy
ctx
cbm
thm
str
kid
gon
hyp
spc
hrt
wat
msl
lng
lvr
hip
cln
0.2
0.6
1
Absolute Correlation

CR−Regulated Modules (20 Genes)

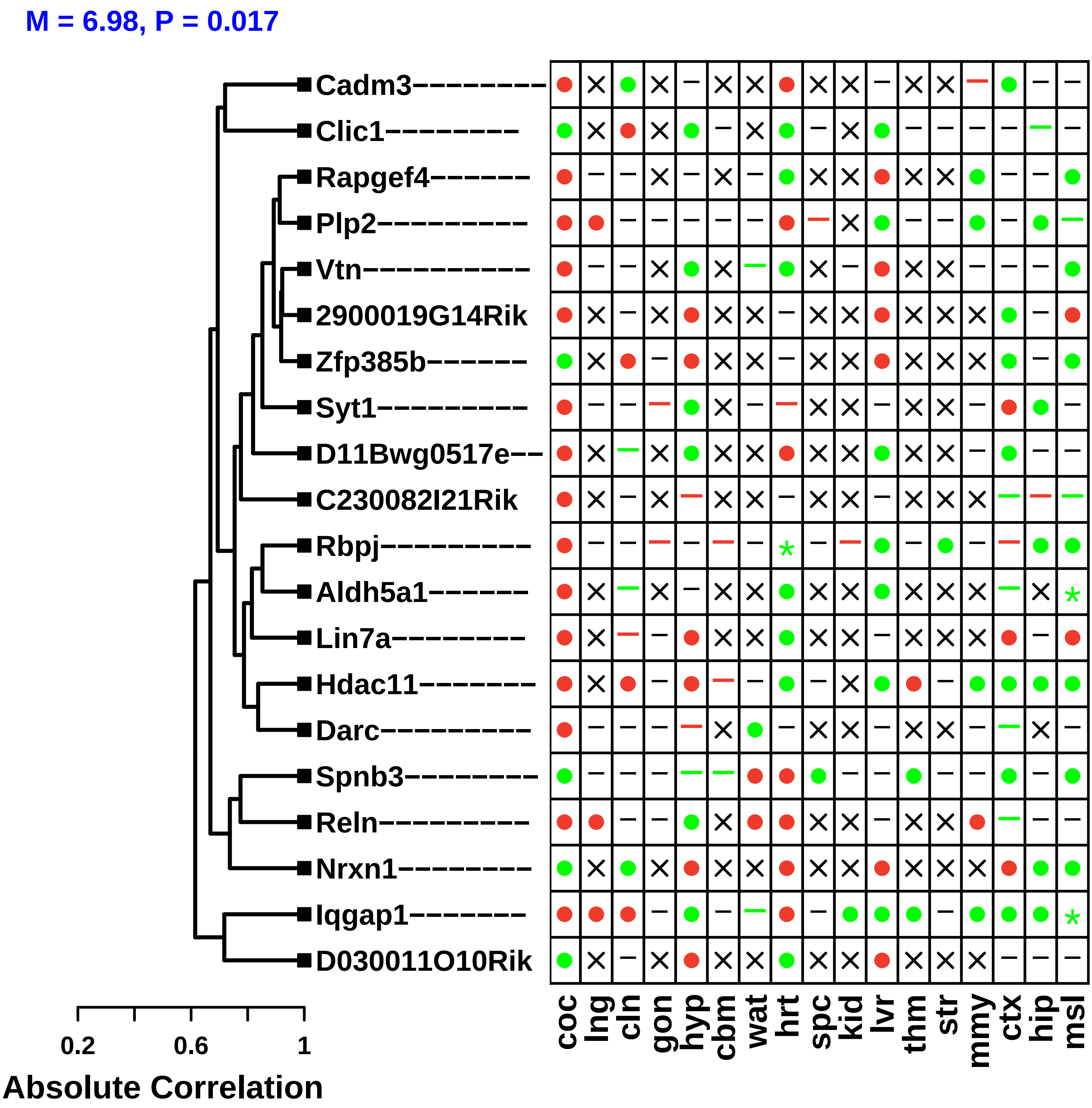

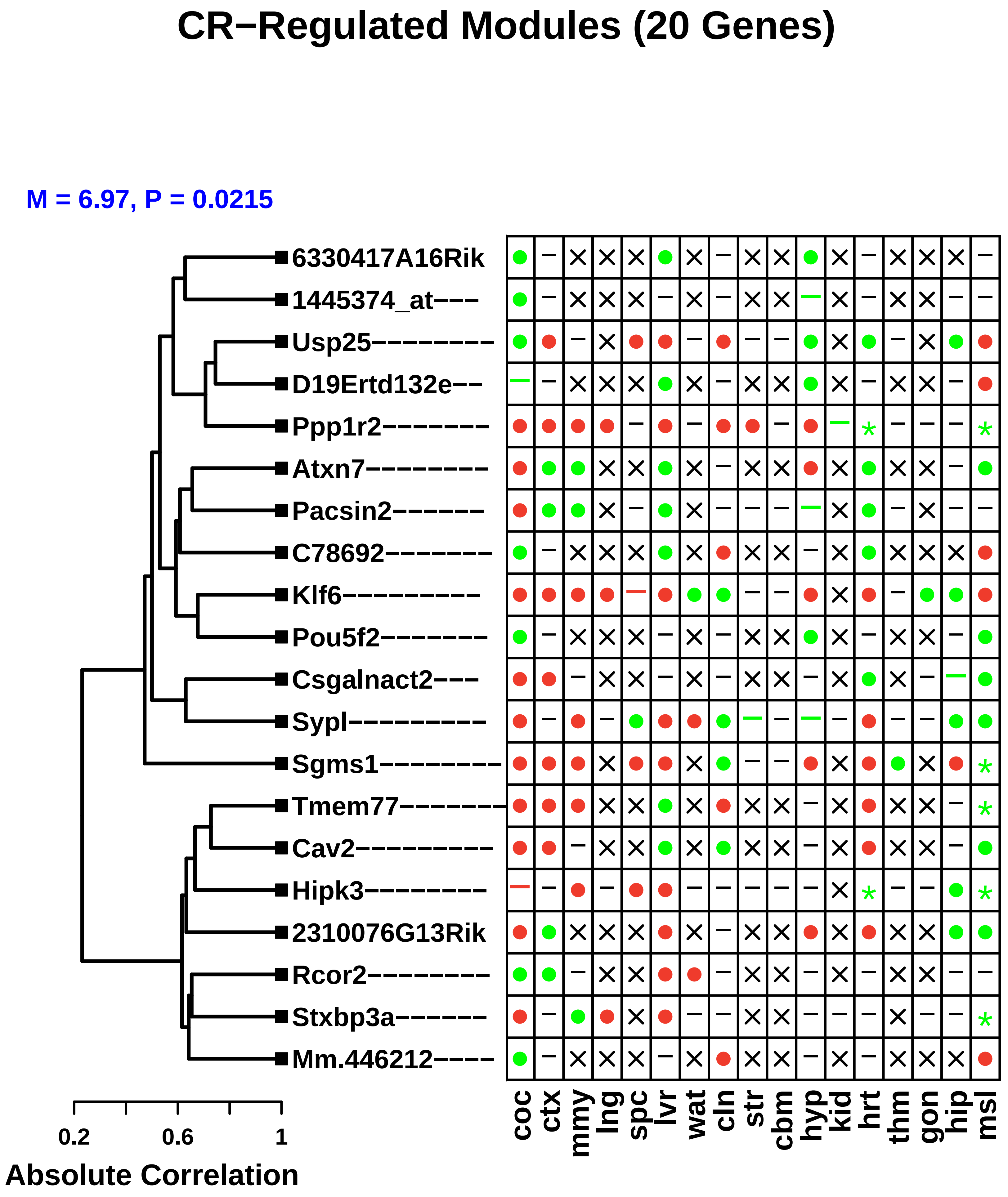
CR−Regulated Modules (20 Genes)
M = 6.97, P = 0.0215
6330417A16Rik
1445374_at
Usp25
D19Ertd132e
Ppp1r2
Atxn7
Pacsin2
C78692
Klf6
Pou5f2
Csgalnact2
Sypl
Sgms1
Tmem77
Cav2
Hipk3
2310076G13Rik
Rcor2
Stxbp3a
Mm.446212
coc
ctx
mmy
lng
spc
lvr
wat
cln
str
cbm
hyp
kid
hrt
thm
gon
hip
msl
0.2
0.6
1
Absolute Correlation

CR−Regulated Modules (20 Genes)

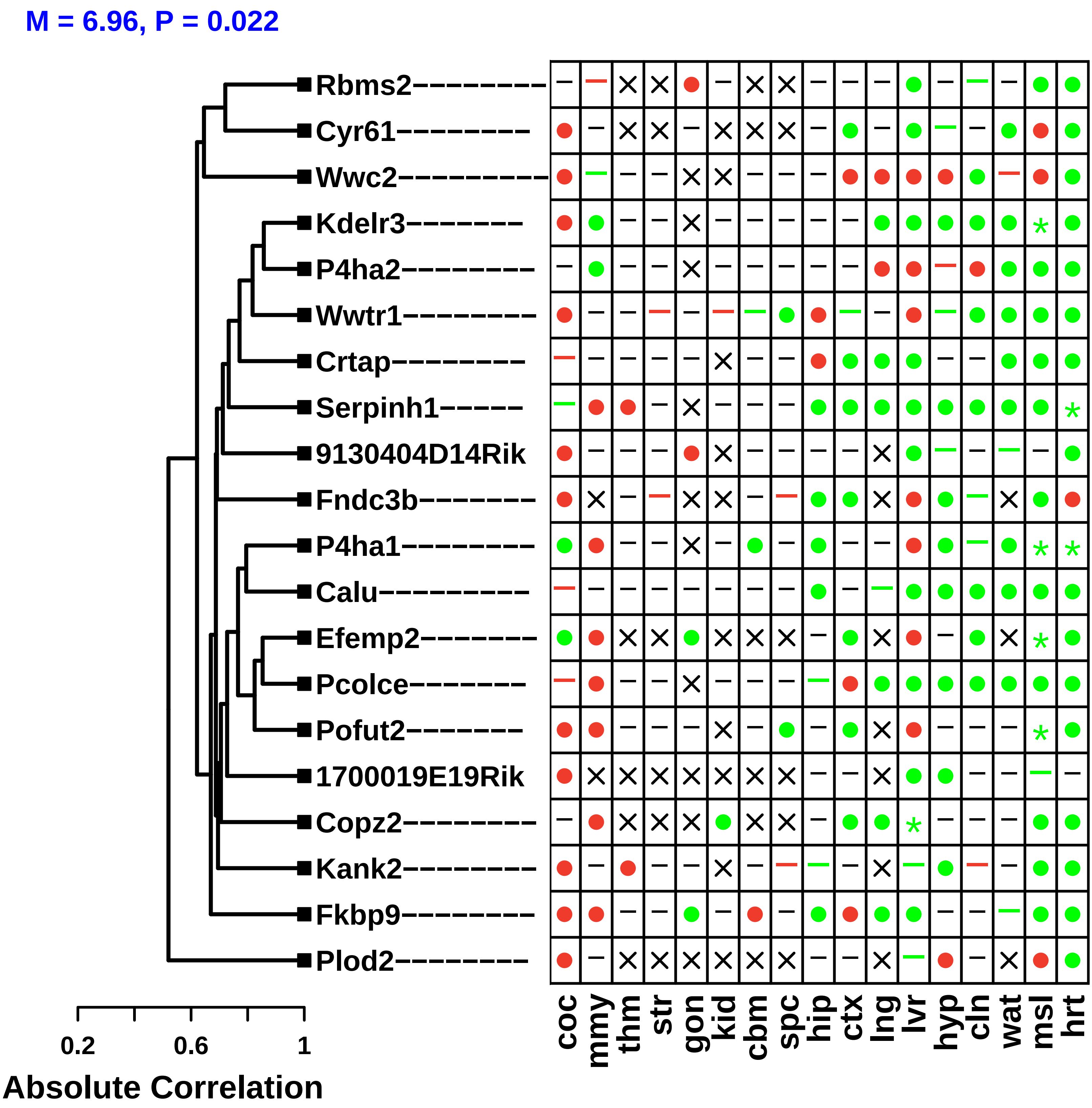

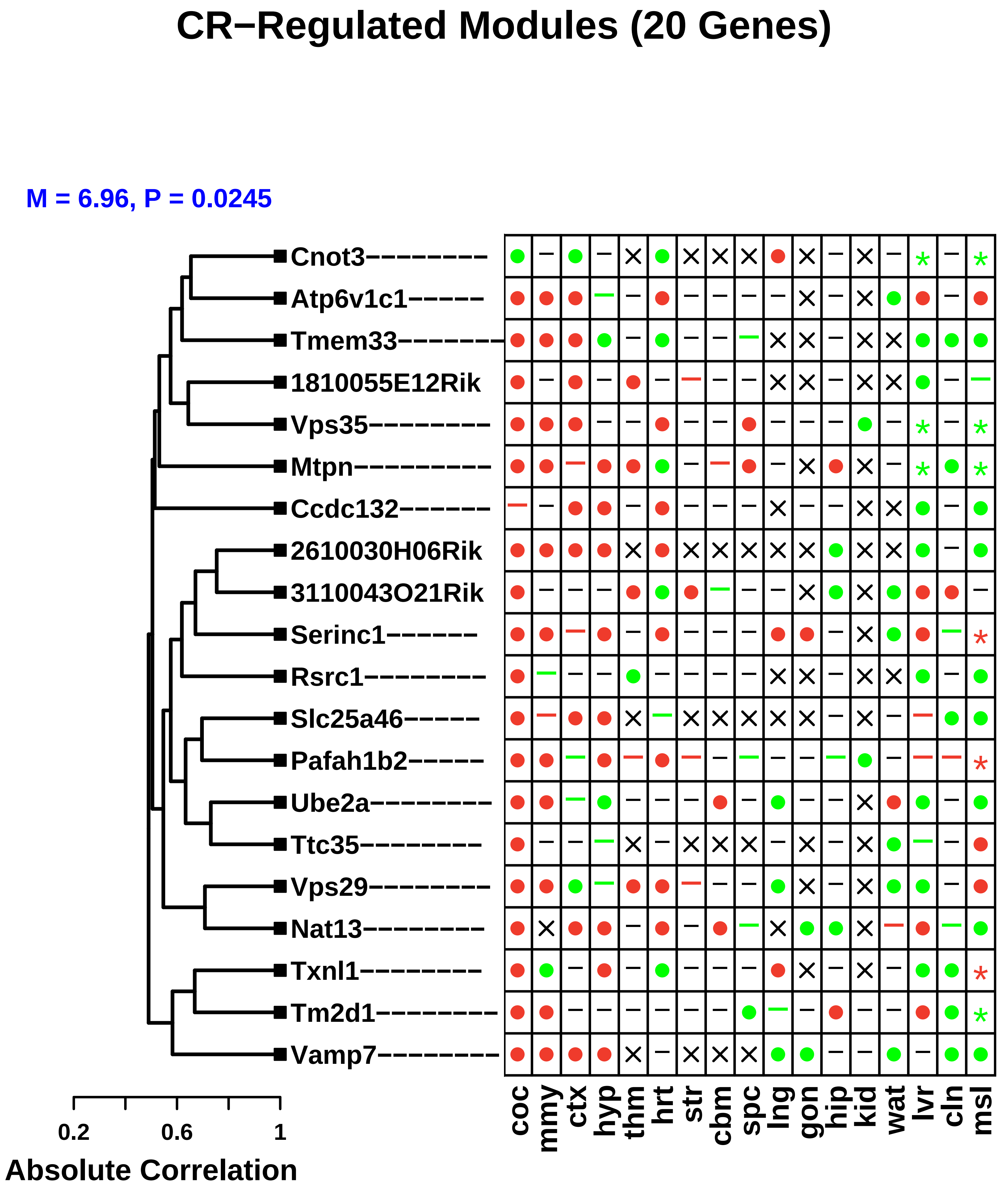
CR−Regulated Modules (20 Genes)
M = 6.96, P = 0.0245
Cnot3
Atp6v1c1
Tmem33
1810055E12Rik
Vps35
Mtpn
Ccdc132
2610030H06Rik
3110043O21Rik
Serinc1
Rsrc1
Slc25a46
Pafah1b2
Ube2a
Ttc35
Vps29
Nat13
Txnl1
Tm2d1
Vamp7
coc
mmy
ctx
hyp
thm
hrt
str
cbm
spc
lng
gon
hip
kid
wat
lvr
cln
msl
0.2
0.6
1
Absolute Correlation

CR−Regulated Modules (20 Genes)

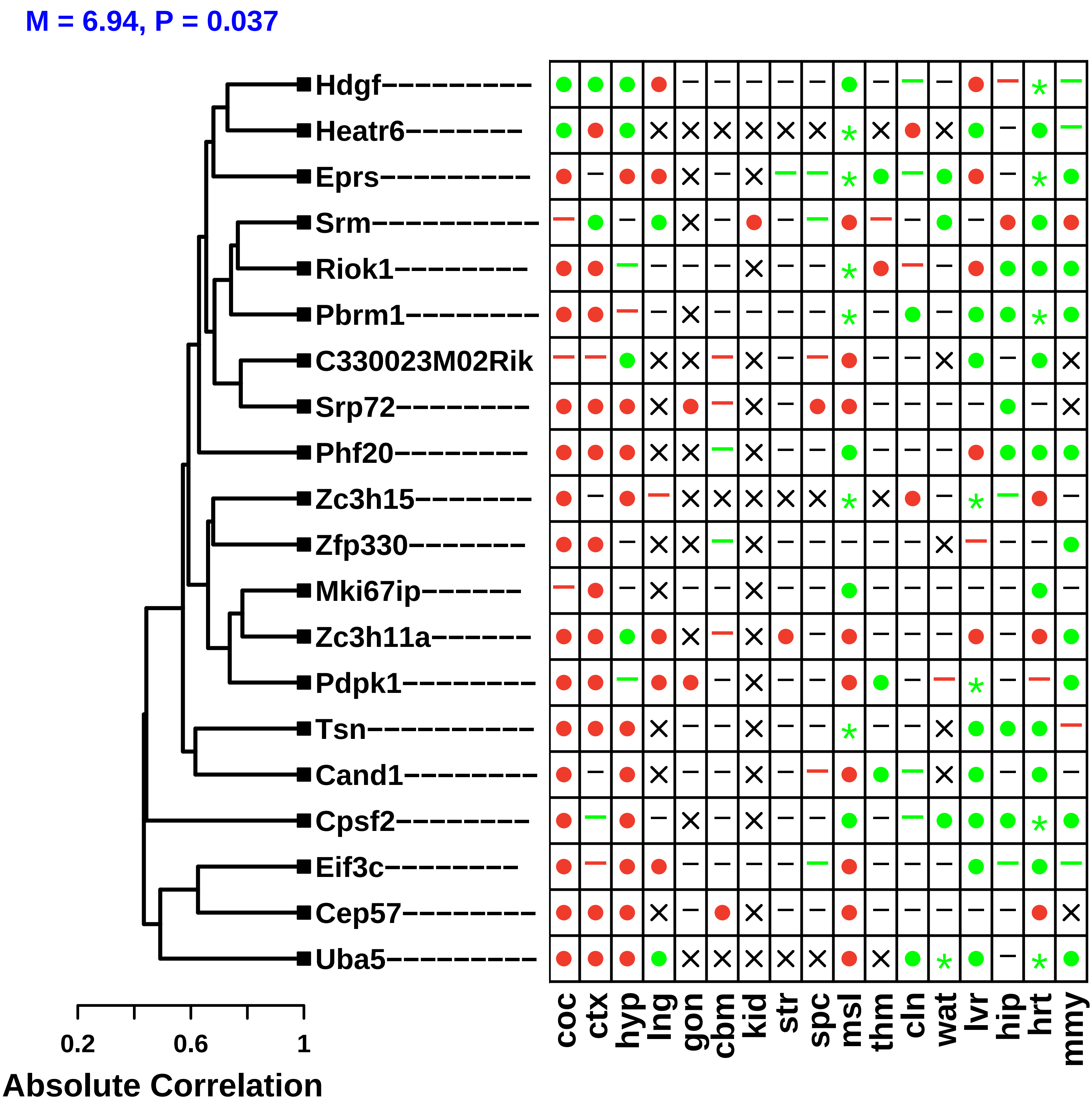

CR−Regulated Modules (40 Genes)

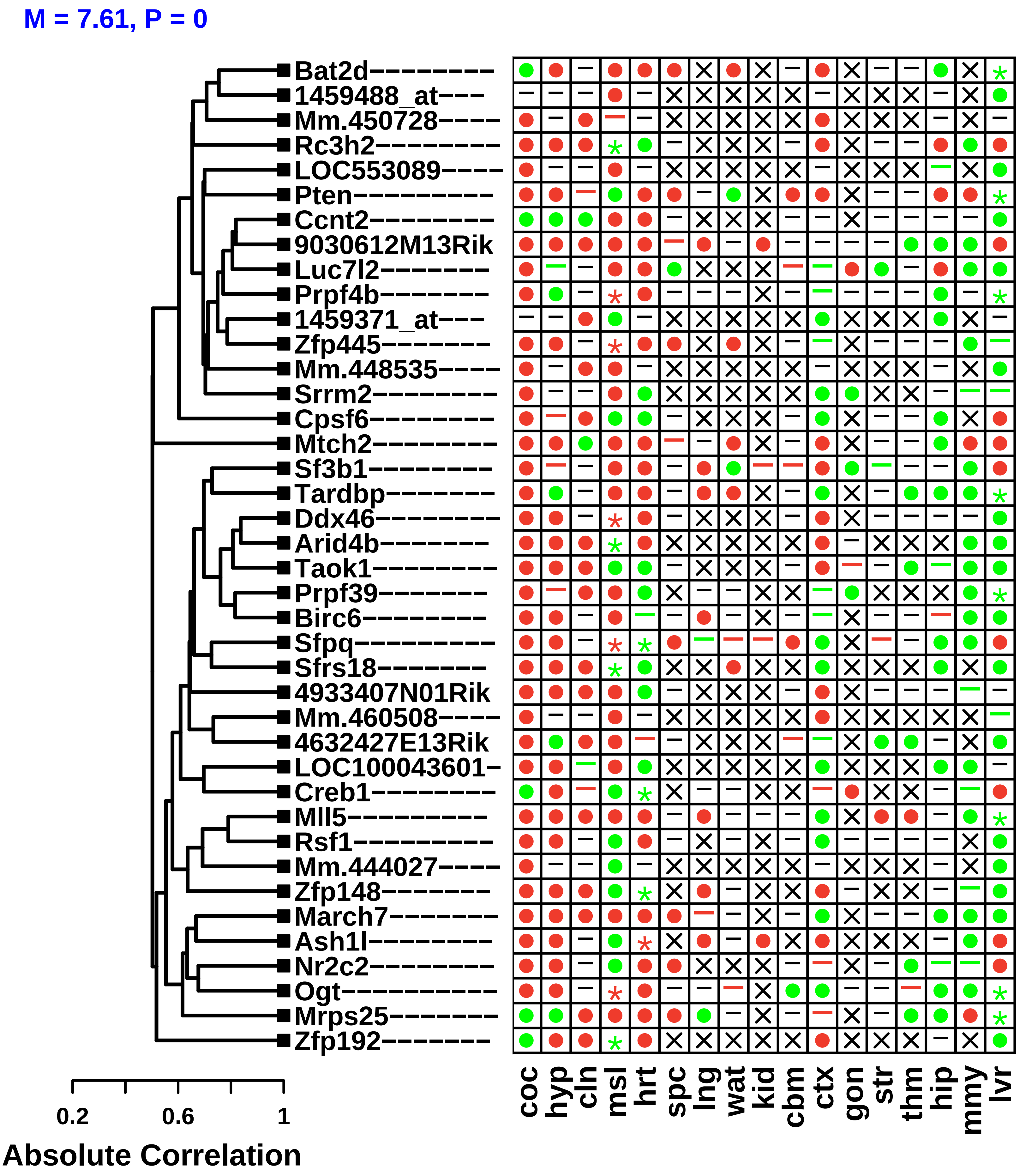

CR−Regulated Modules (40 Genes)

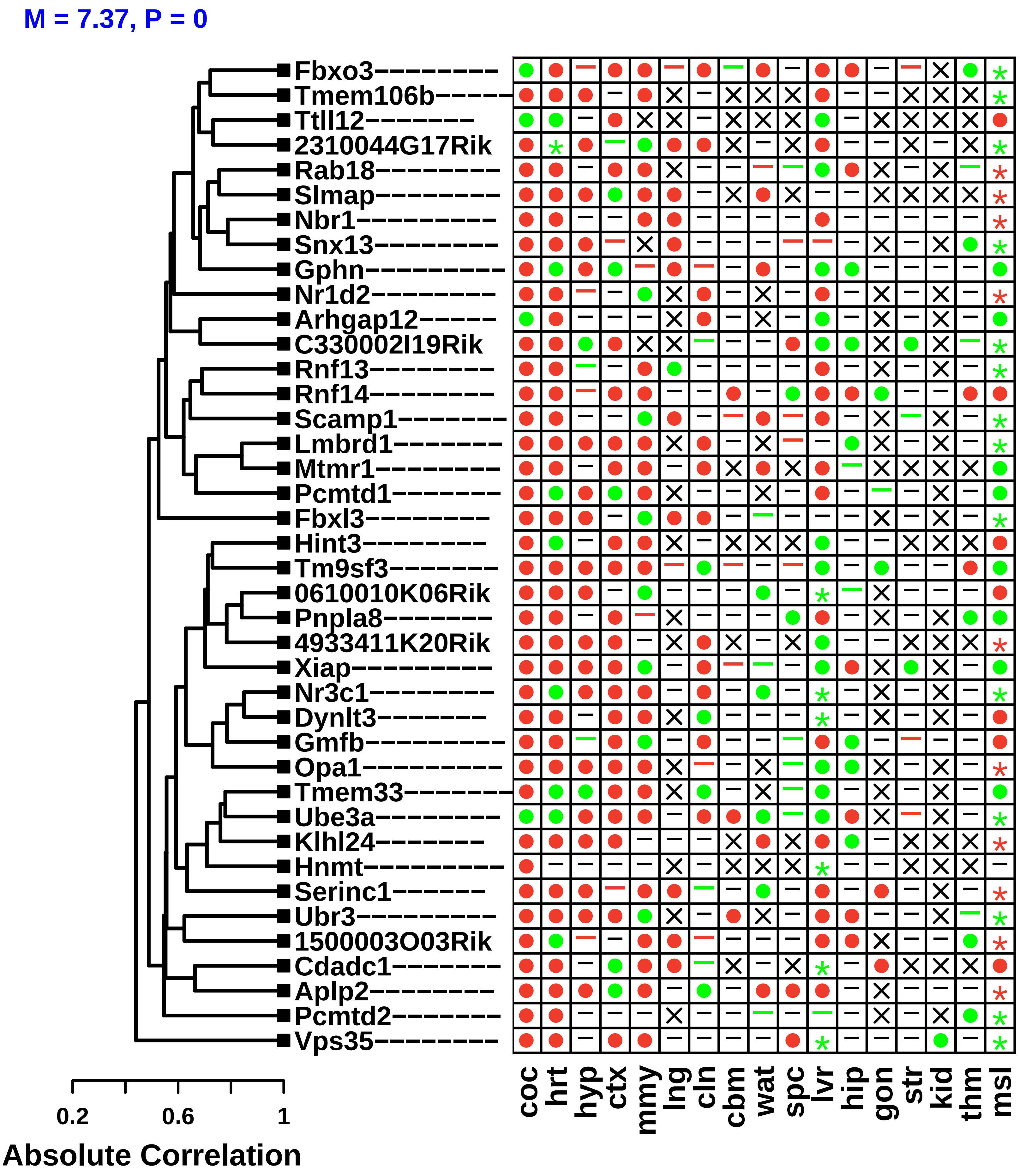

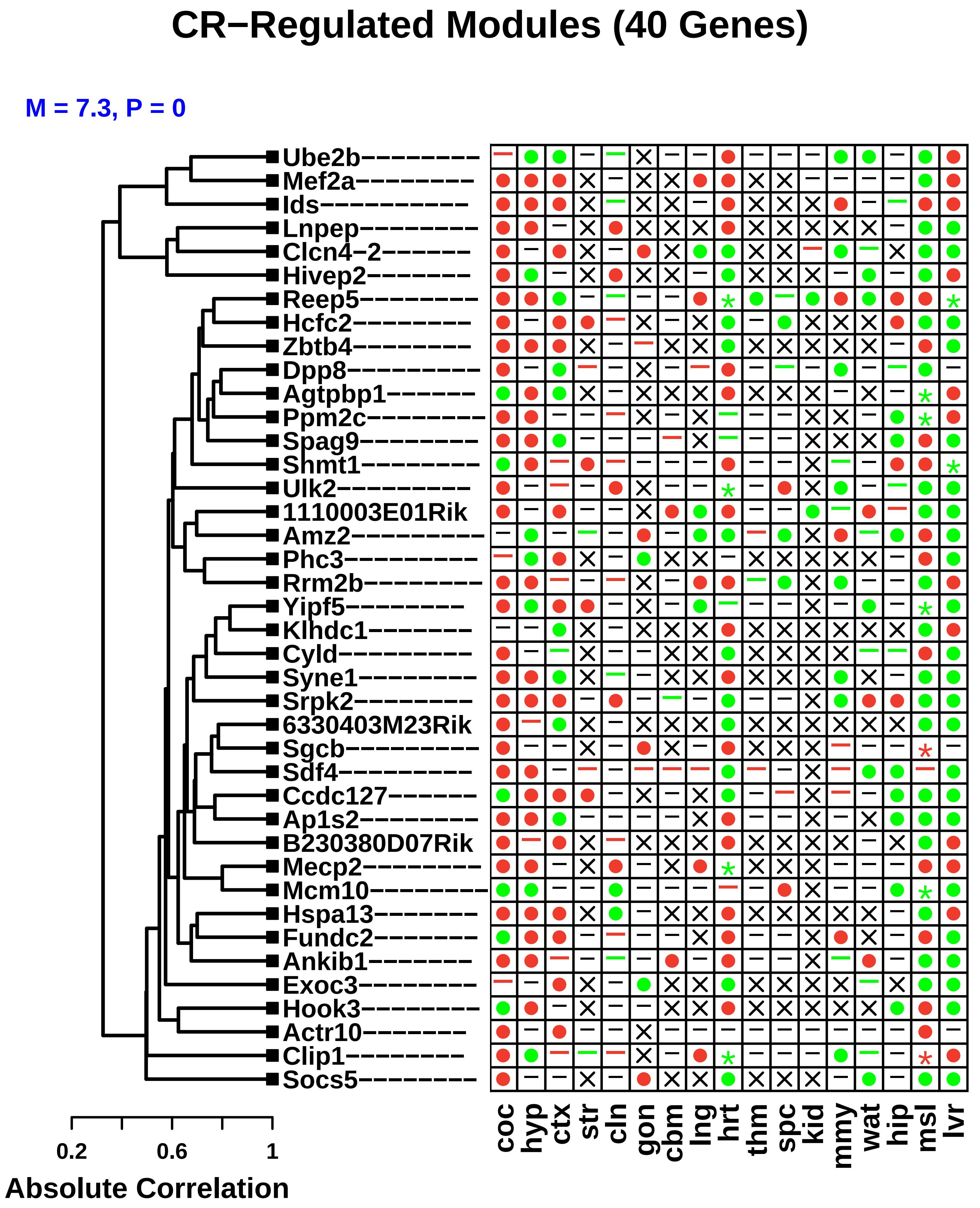
CR−Regulated Modules (40 Genes)
M = 7.3, P = 0
Ube2b
Mef2a
Ids
Lnpep
Clcn4−2
Hivep2
Reep5
Hcfc2
Zbtb4
Dpp8
Agtpbp1
Ppm2c
Spag9
Shmt1
Ulk2
1110003E01Rik
Amz2
Phc3
Rrm2b
Yipf5
Klhdc1
Cyld
Syne1
Srpk2
6330403M23Rik
Sgcb
Sdf4
Ccdc127
Ap1s2
B230380D07Rik
Mecp2
Mcm10
Hspa13
Fundc2
Ankib1
Exoc3
Hook3
Actr10
Clip1
Socs5
coc
hyp
ctx
str
cln
gon
cbm
lng
hrt
thm
spc
kid
mmy
wat
hip
msl
lvr
0.2
0.6
1
Absolute Correlation

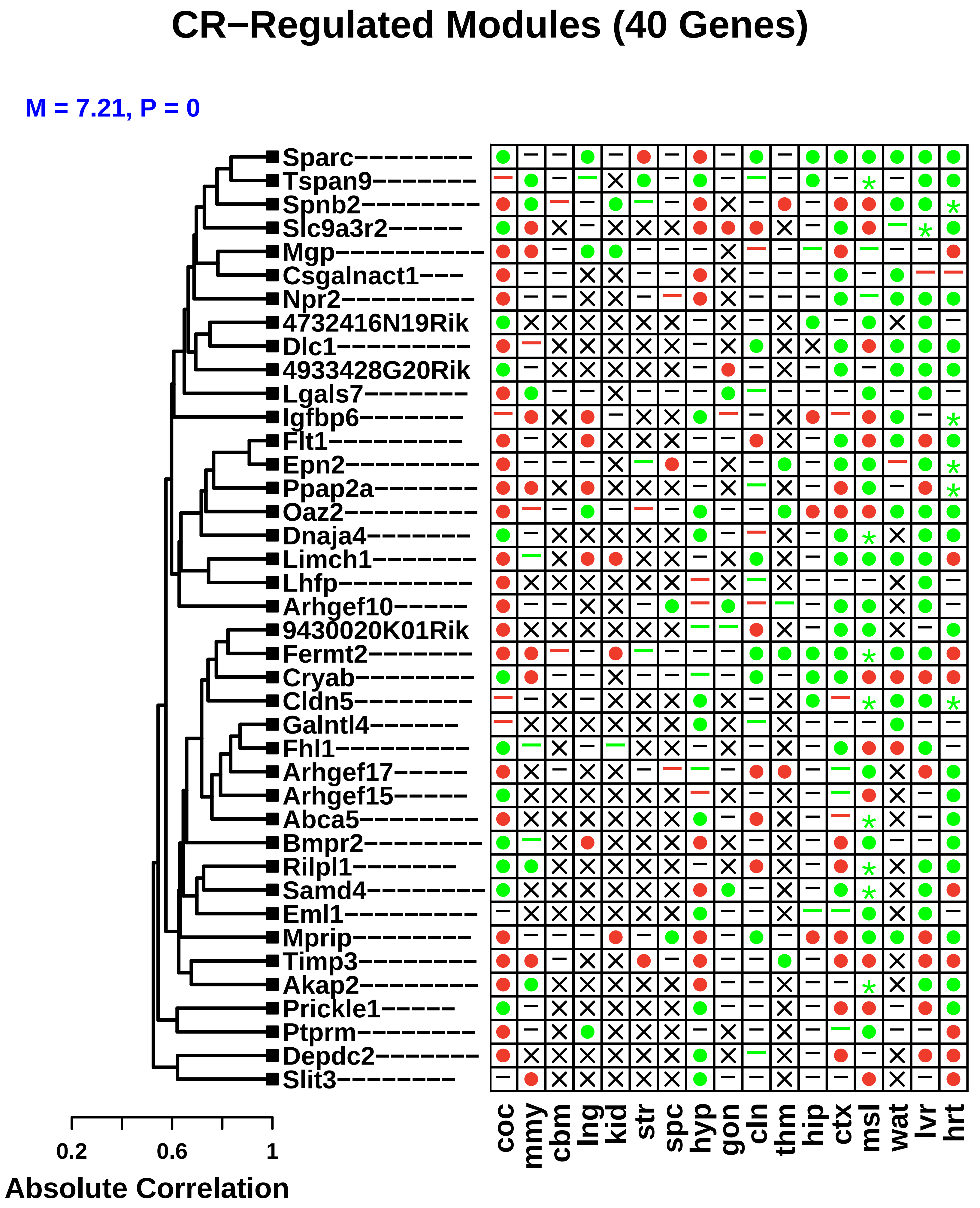

CR−Regulated Modules (40 Genes)
M = 7.21, P = 0
Sparc
Tspan9
Spnb2
Slc9a3r2
Mgp
Csgalnact1
Npr2
4732416N19Rik
Dlc1
4933428G20Rik
Lgals7
Igfbp6
Flt1
Epn2
Ppap2a
Oaz2
Dnaja4
Limch1
Lhfp
Arhgef10
9430020K01Rik
Fermt2
Cryab
Cldn5
Galntl4
Fhl1
Arhgef17
Arhgef15
Abca5
Bmpr2
Rilpl1
Samd4
Eml1
Mprip
Timp3
Akap2
Prickle1
Ptprm
Depdc2
Slit3
coc
mmy
cbm
lng
kid
str
spc
hyp
gon
cln
thm
hip
ctx
msl
wat
lvr
hrt
0.2
0.6
1
Absolute Correlation

CR−Regulated Modules (40 Genes)

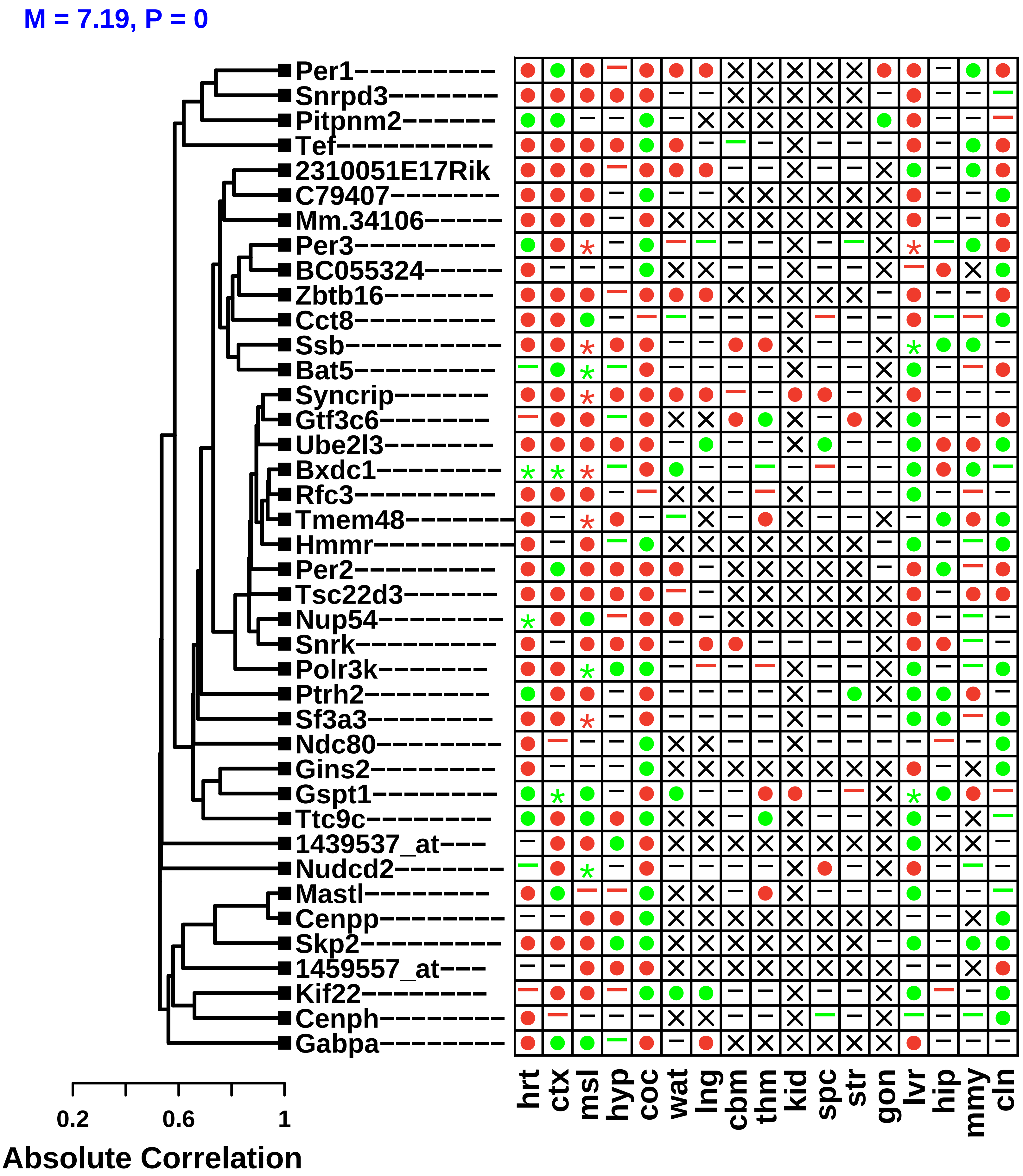

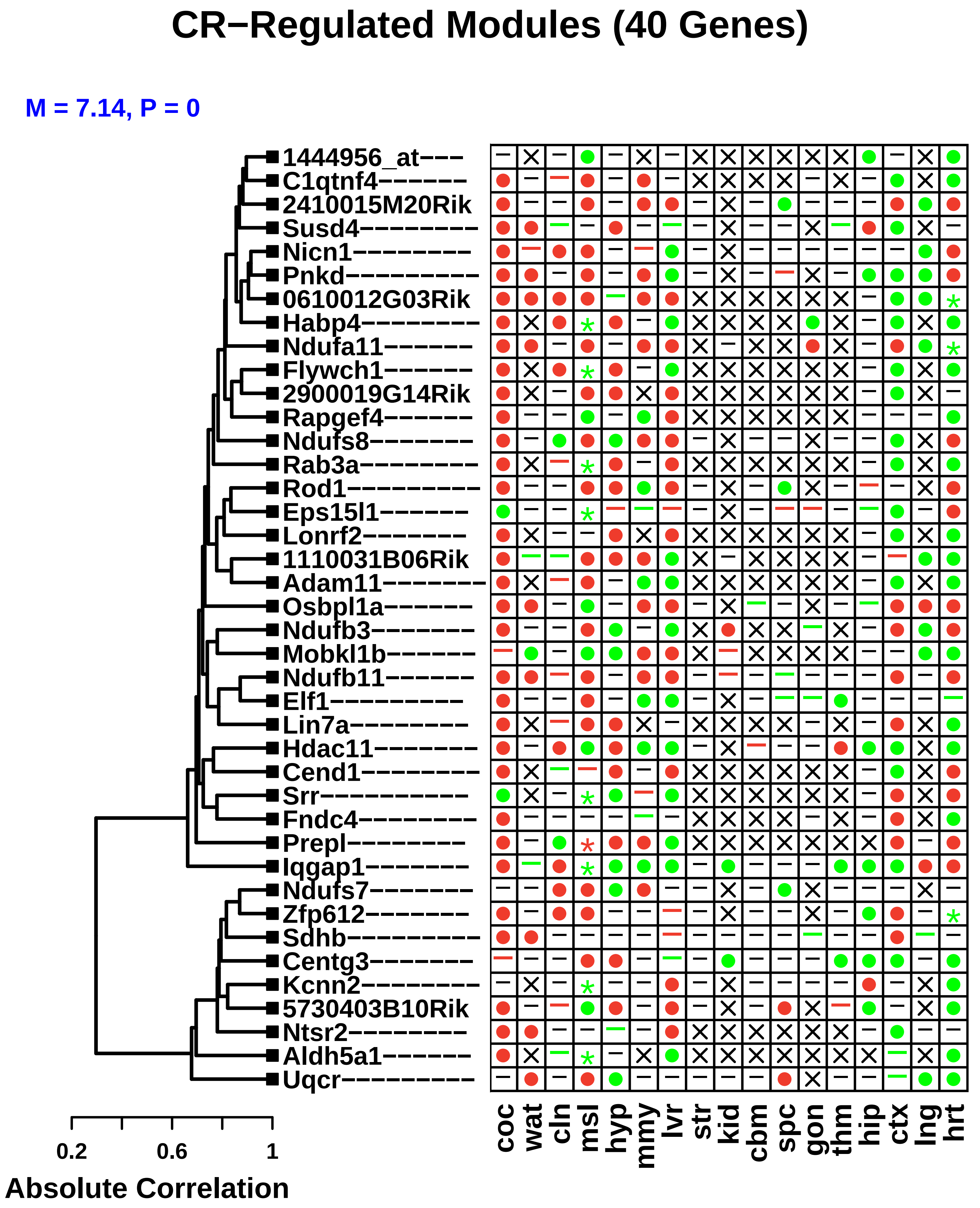
CR−Regulated Modules (40 Genes)
M = 7.14, P = 0
1444956_at
C1qtnf4
2410015M20Rik
Susd4
Nicn1
Pnkd
0610012G03Rik
Habp4
Ndufa11
Flywch1
2900019G14Rik
Rapgef4
Ndufs8
Rab3a
Rod1
Eps15l1
Lonrf2
1110031B06Rik
Adam11
Osbpl1a
Ndufb3
Mobkl1b
Ndufb11
Elf1
Lin7a
Hdac11
Cend1
Srr
Fndc4
Prepl
Iqgap1
Ndufs7
Zfp612
Sdhb
Centg3
Kcnn2
5730403B10Rik
Ntsr2
Aldh5a1
Uqcr
coc
wat
cln
msl
hyp
mmy
lvr
str
kid
cbm
spc
gon
thm
hip
ctx
lng
hrt
0.2
0.6
1
Absolute Correlation

CR−Regulated Modules (40 Genes)

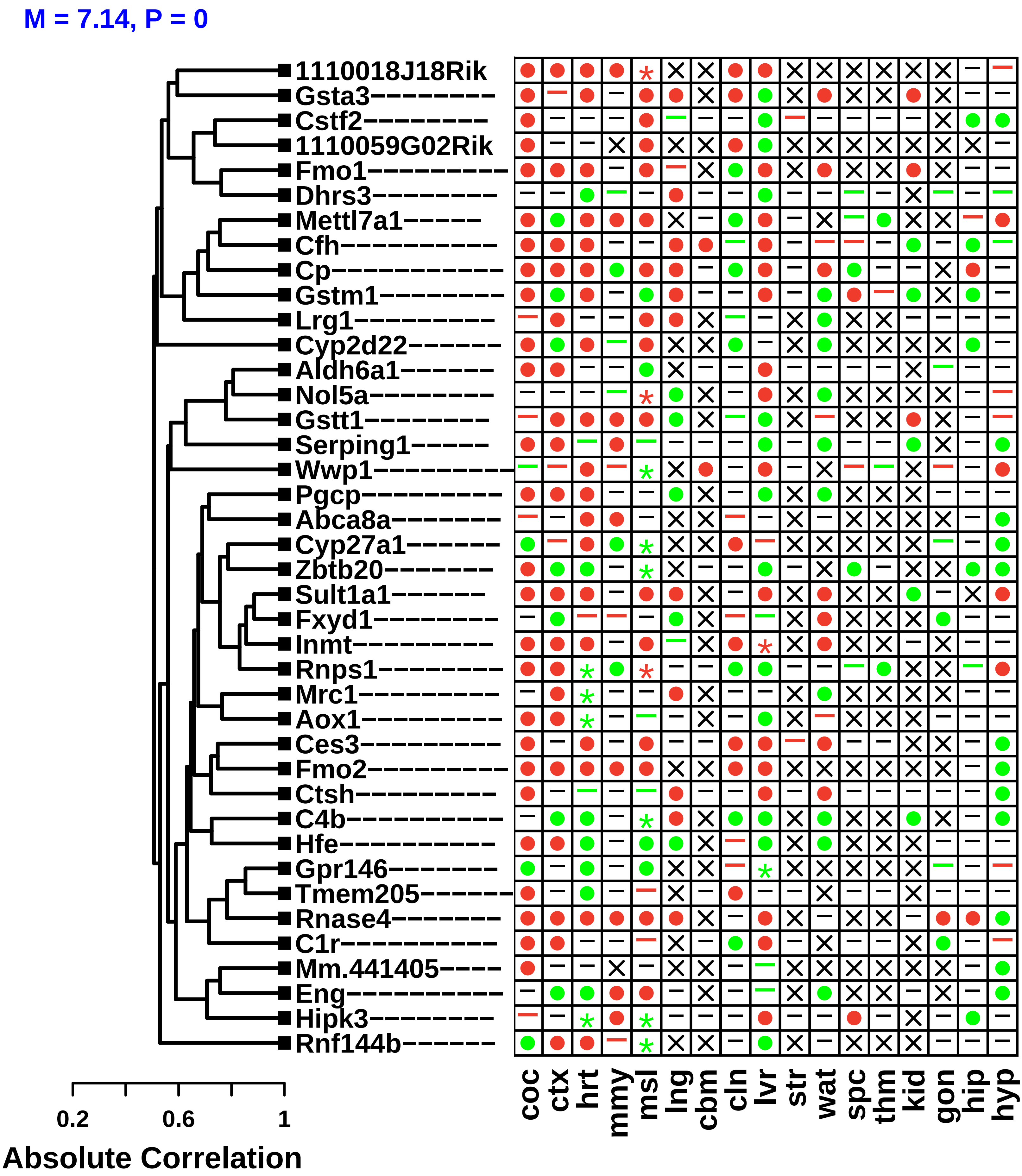

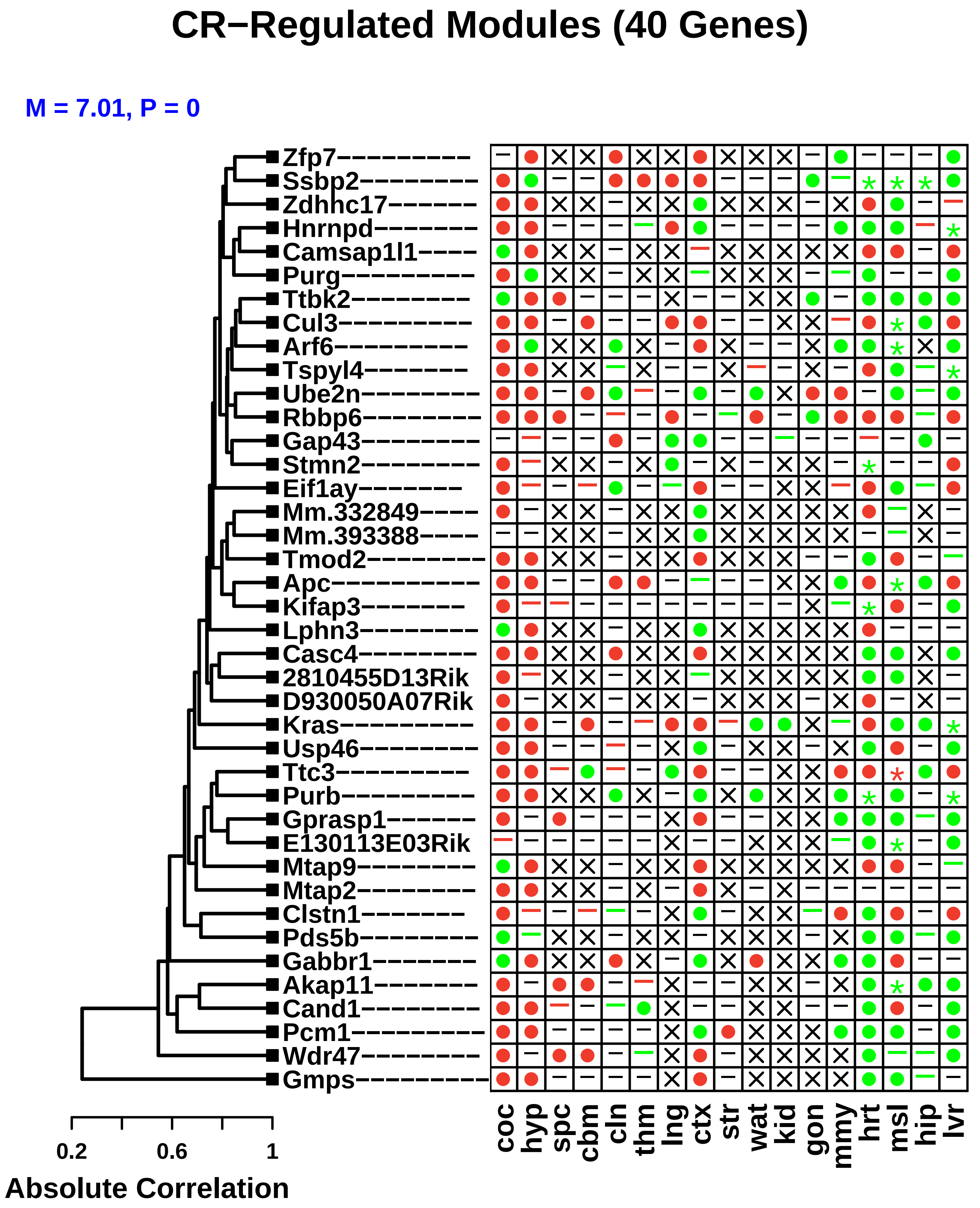
CR−Regulated Modules (40 Genes)
M = 7.01, P = 0
Zfp7
Ssbp2
Zdhhc17
Hnrnpd
Camsap1l1
Purg
Ttbk2
Cul3
Arf6
Tspyl4
Ube2n
Rbbp6
Gap43
Stmn2
Eif1ay
Mm.332849
Mm.393388
Tmod2
Apc
Kifap3
Lphn3
Casc4
2810455D13Rik
D930050A07Rik
Kras
Usp46
Ttc3
Purb
Gprasp1
E130113E03Rik
Mtap9
Mtap2
Clstn1
Pds5b
Gabbr1
Akap11
Cand1
Pcm1
Wdr47
Gmps
coc
hyp
spc
cbm
cln
thm
lng
ctx
str
wat
kid
gon
mmy
hrt
msl
hip
lvr
0.2
0.6
1
Absolute Correlation

CR−Regulated Modules (40 Genes)

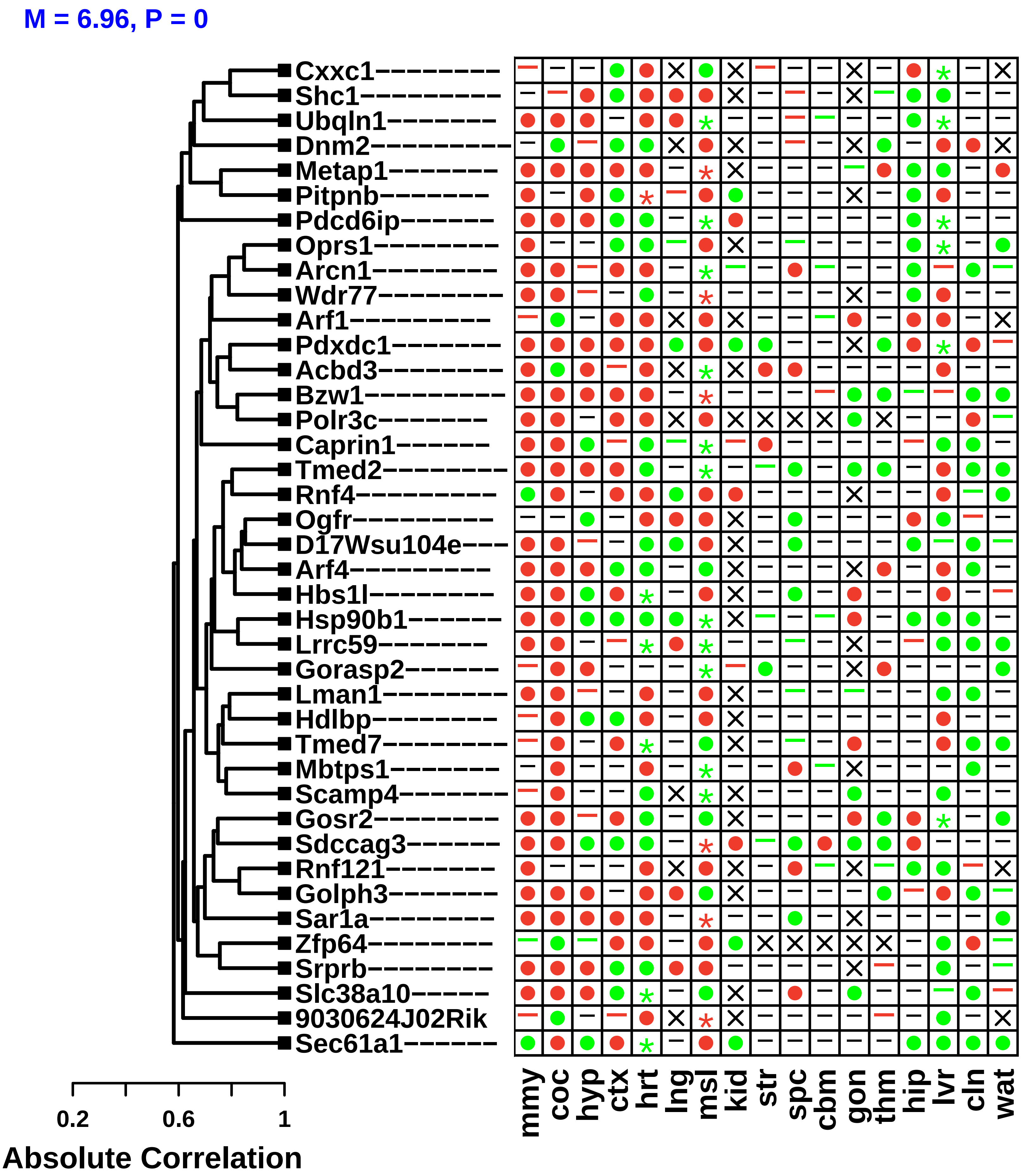

CR−Regulated Modules (40 Genes)

M = 6.96, P = 0

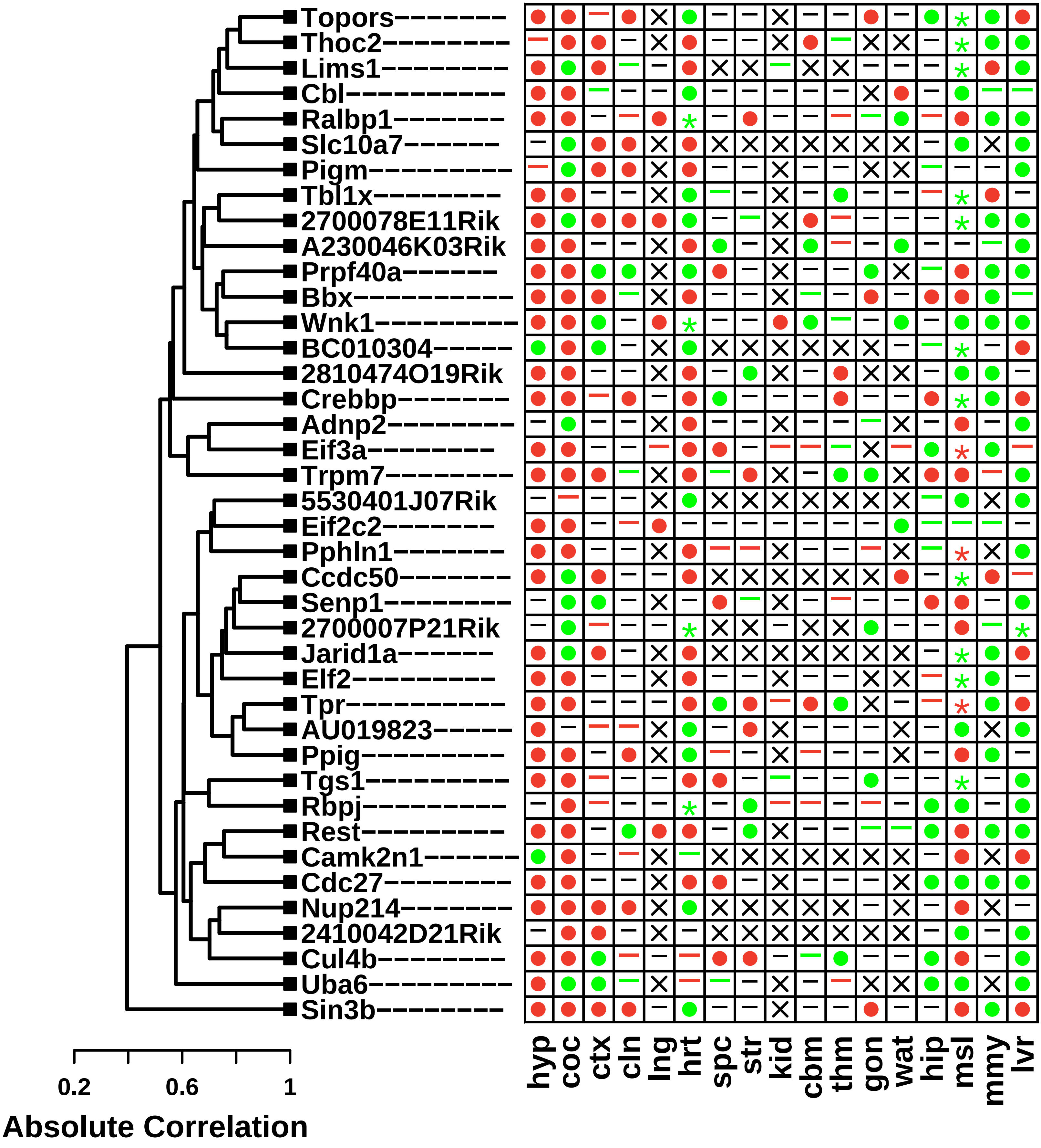

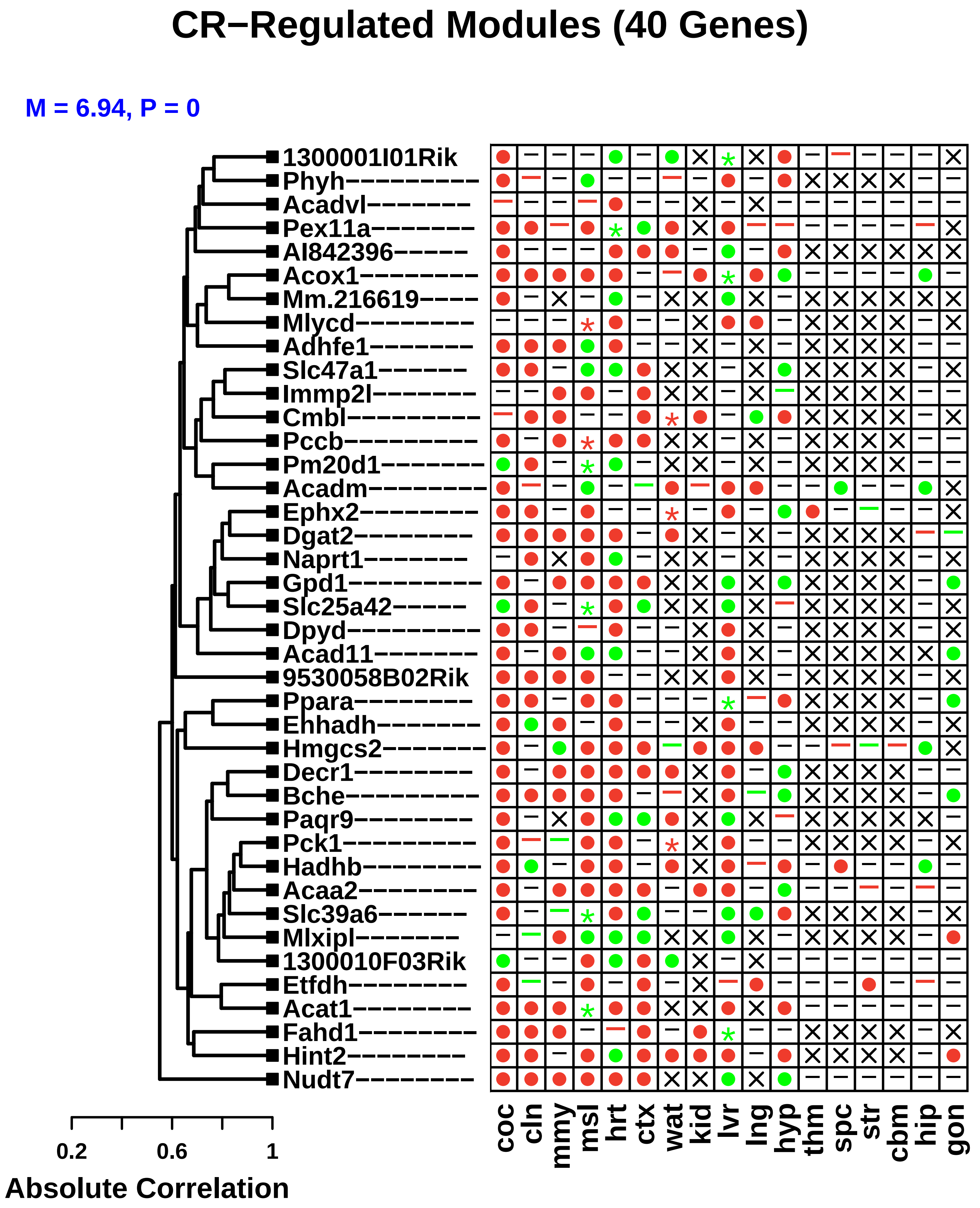
CR−Regulated Modules (40 Genes)
M = 6.94, P = 0
1300001I01Rik
Phyh
Acadvl
Pex11a
AI842396
Acox1
Mm.216619
Mlycd
Adhfe1
Slc47a1
Immp2l
Cmbl
Pccb
Pm20d1
Acadm
Ephx2
Dgat2
Naprt1
Gpd1
Slc25a42
Dpyd
Acad11
9530058B02Rik
Ppara
Ehhadh
Hmgcs2
Decr1
Bche
Paqr9
Pck1
Hadhb
Acaa2
Slc39a6
Mlxipl
1300010F03Rik
Etfdh
Acat1
Fahd1
Hint2
Nudt7
coc
cln
mmy
msl
hrt
ctx
wat
kid
lvr
lng
hyp
thm
spc
str
cbm
hip
gon
0.2
0.6
1
Absolute Correlation

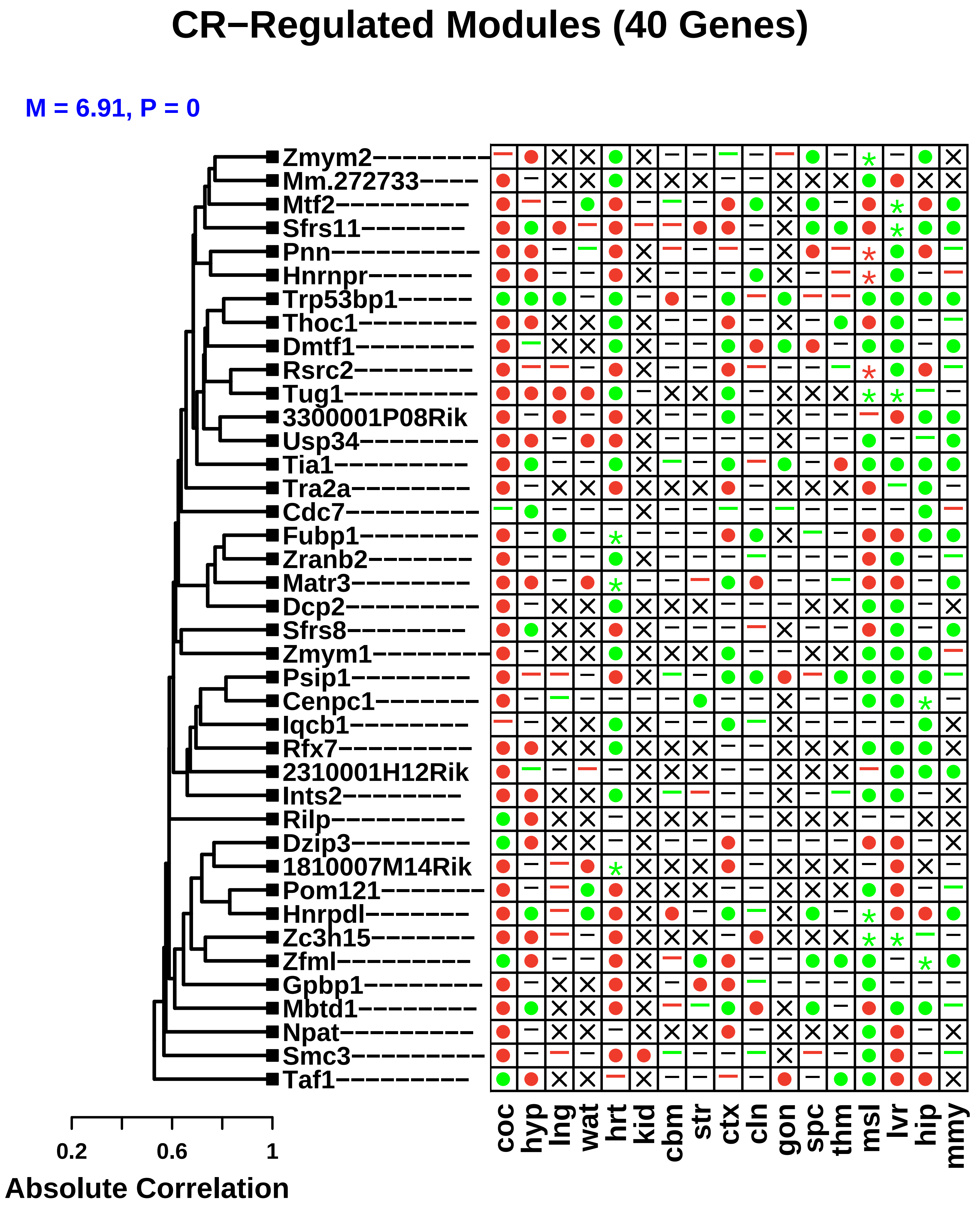
CR−Regulated Modules (40 Genes)
M = 6.91, P = 0
Zmym2
Mm.272733
Mtf2
Sfrs11
Pnn
Hnrnpr
Trp53bp1
Thoc1
Dmtf1
Rsrc2
Tug1
3300001P08Rik
Usp34
Tia1
Tra2a
Cdc7
Fubp1
Zranb2
Matr3
Dcp2
Sfrs8
Zmym1
Psip1
Cenpc1
Iqcb1
Rfx7
2310001H12Rik
Ints2
Rilp
Dzip3
1810007M14Rik
Pom121
Hnrpdl
Zc3h15
Zfml
Gpbp1
Mbtd1
Npat
Smc3
Taf1
coc
hyp
lng
wat
hrt
kid
cbm
str
ctx
cln
gon
spc
thm
msl
lvr
hip
mmy
0.2
0.6
1
Absolute Correlation

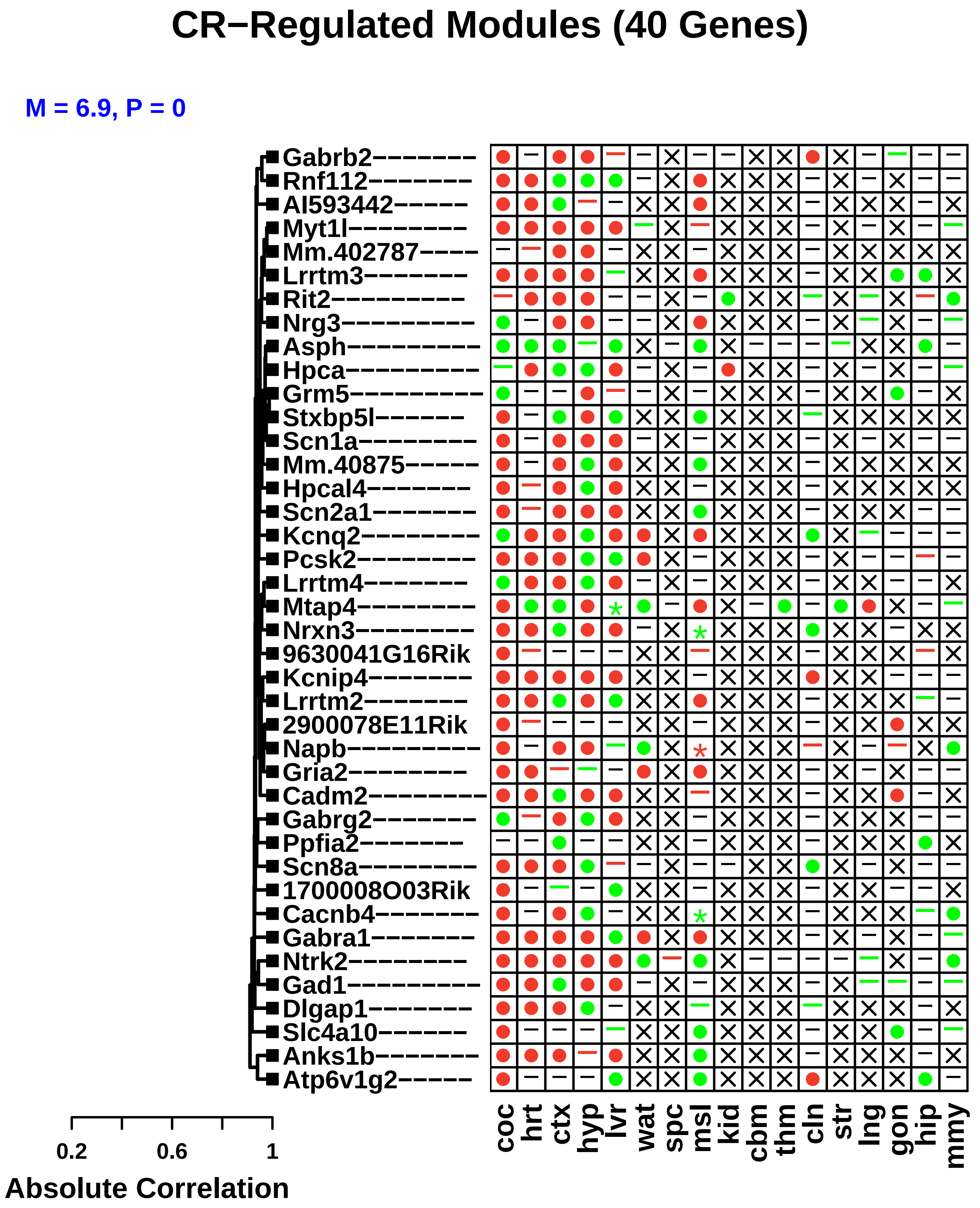
CR−Regulated Modules (40 Genes)
M = 6.9, P = 0
Gabrb2
Rnf112
AI593442
Myt1l
Mm.402787
Lrrtm3
Rit2
Nrg3
Asph
Hpca
Grm5
Stxbp5l
Scn1a
Mm.40875
Hpcal4
Scn2a1
Kcnq2
Pcsk2
Lrrtm4
Mtap4
Nrxn3
9630041G16Rik
Kcnip4
Lrrtm2
2900078E11Rik
Napb
Gria2
Cadm2
Gabrg2
Ppfia2
Scn8a
1700008O03Rik
Cacnb4
Gabra1
Ntrk2
Gad1
Dlgap1
Slc4a10
Anks1b
Atp6v1g2
coc
hrt
ctx
hyp
lvr
wat
spc
msl
kid
cbm
thm
cln
str
lng
gon
hip
mmy
0.2
0.6
1
Absolute Correlation

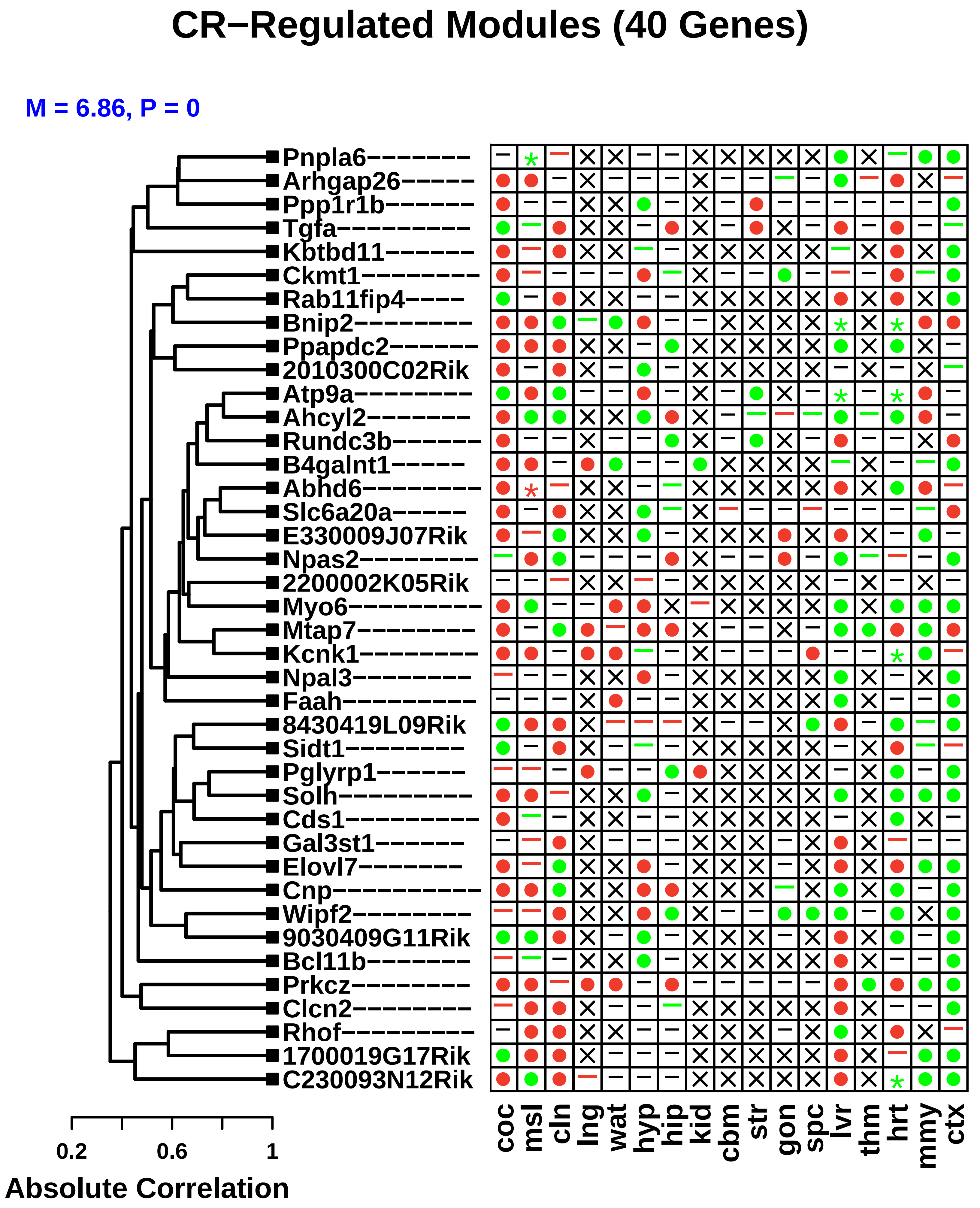
CR−Regulated Modules (40 Genes)
M = 6.86, P = 0
Pnpla6
Arhgap26
Ppp1r1b
Tgfa
Kbtbd11
Ckmt1
Rab11fip4
Bnip2
Ppapdc2
2010300C02Rik
Atp9a
Ahcyl2
Rundc3b
B4galnt1
Abhd6
Slc6a20a
E330009J07Rik
Npas2
2200002K05Rik
Myo6
Mtap7
Kcnk1
Npal3
Faah
8430419L09Rik
Sidt1
Pglyrp1
Solh
Cds1
Gal3st1
Elovl7
Cnp
Wipf2
9030409G11Rik
Bcl11b
Prkcz
Clcn2
Rhof
1700019G17Rik
C230093N12Rik
coc
msl
cln
lng
wat
hyp
hip
kid
cbm
str
gon
spc
lvr
thm
hrt
mmy
ctx
0.2
0.6
1
Absolute Correlation

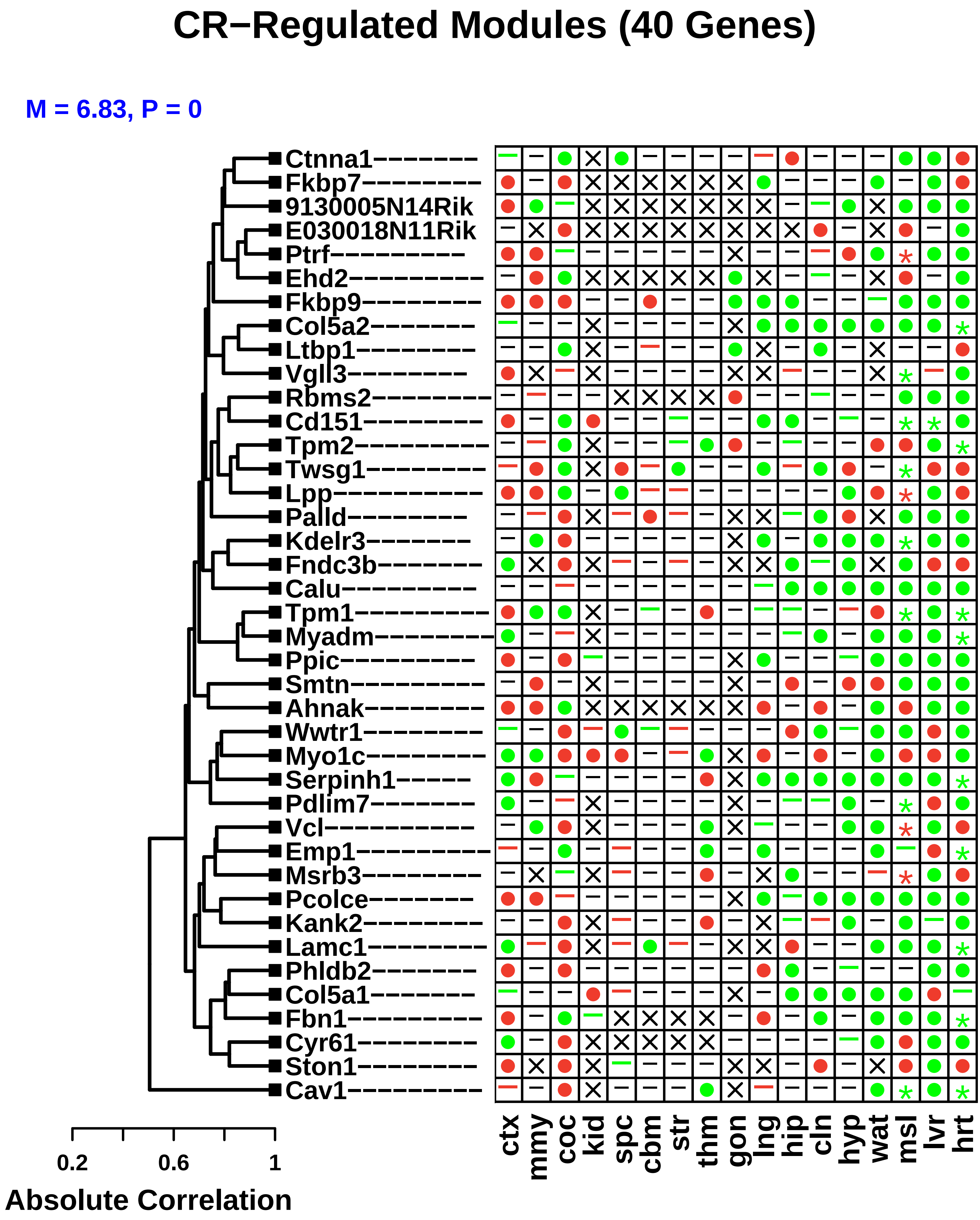

CR−Regulated Modules (40 Genes)
M = 6.83, P = 0
Ctnna1
Fkbp7
9130005N14Rik
E030018N11Rik
Ptrf
Ehd2
Fkbp9
Col5a2
Ltbp1
Vgll3
Rbms2
Cd151
Tpm2
Twsg1
Lpp
Palld
Kdelr3
Fndc3b
Calu
Tpm1
Myadm
Ppic
Smtn
Ahnak
Wwtr1
Myo1c
Serpinh1
Pdlim7
Vcl
Emp1
Msrb3
Pcolce
Kank2
Lamc1
Phldb2
Col5a1
Fbn1
Cyr61
Ston1
Cav1
ctx
mmy
coc
kid
spc
cbm
str
thm
gon
lng
hip
cln
hyp
wat
msl
lvr
hrt
0.2
0.6
1
Absolute Correlation

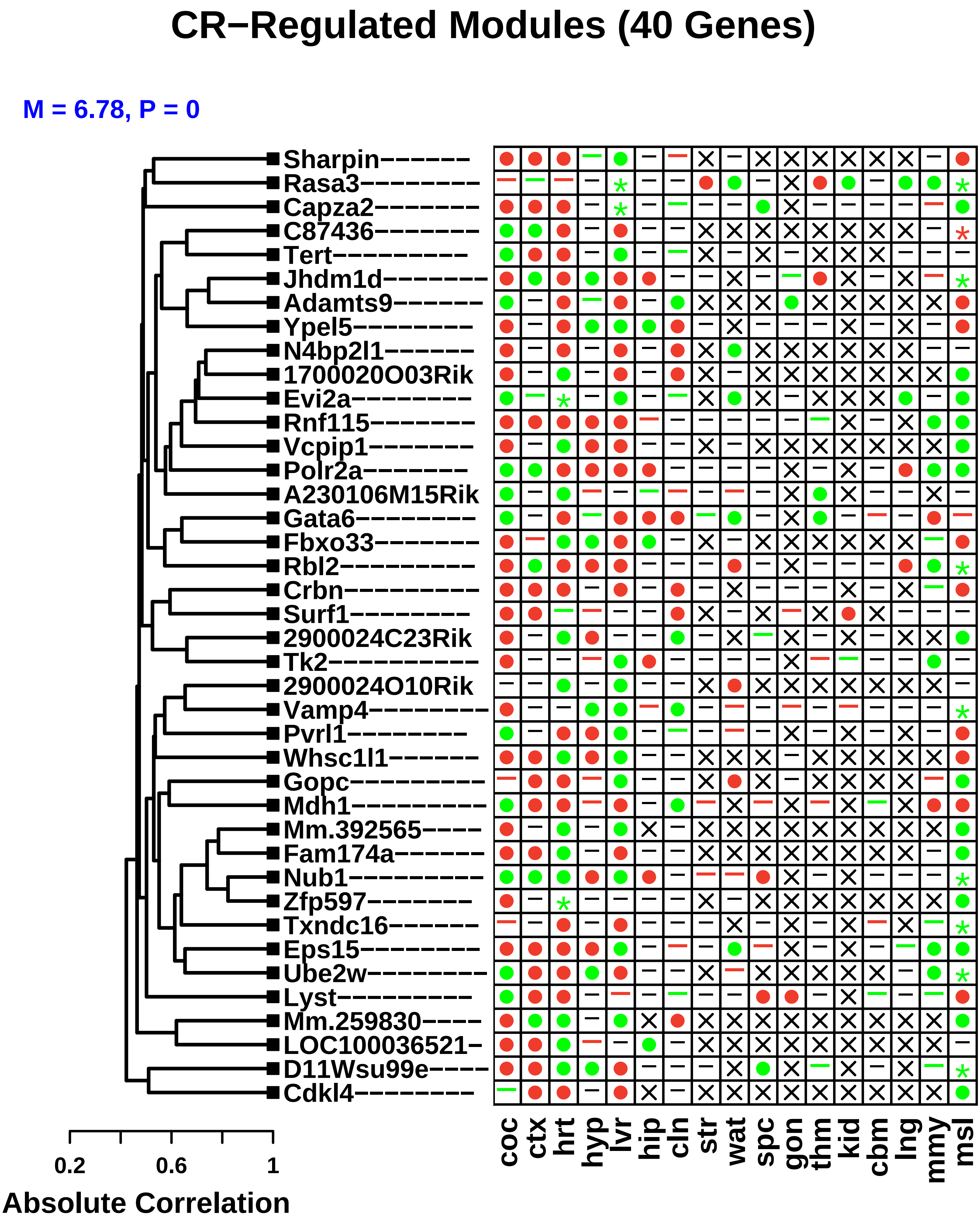

CR−Regulated Modules (40 Genes)
M = 6.78, P = 0
Sharpin
Rasa3
Capza2
C87436
Tert
Jhdm1d
Adamts9
Ypel5
N4bp2l1
1700020O03Rik
Evi2a
Rnf115
Vcpip1
Polr2a
A230106M15Rik
Gata6
Fbxo33
Rbl2
Crbn
Surf1
2900024C23Rik
Tk2
2900024O10Rik
Vamp4
Pvrl1
Whsc1l1
Gopc
Mdh1
Mm.392565
Fam174a
Nub1
Zfp597
Txndc16
Eps15
Ube2w
Lyst
Mm.259830
LOC100036521
D11Wsu99e
Cdkl4
0.2
0.6
1
Absolute Correlation
coc
ctx
hrt
hyp
lvr
hip
cln
str
wat
spc
gon
thm
kid
cbm
lng
mmy
msl

CR−Regulated Modules (40 Genes)

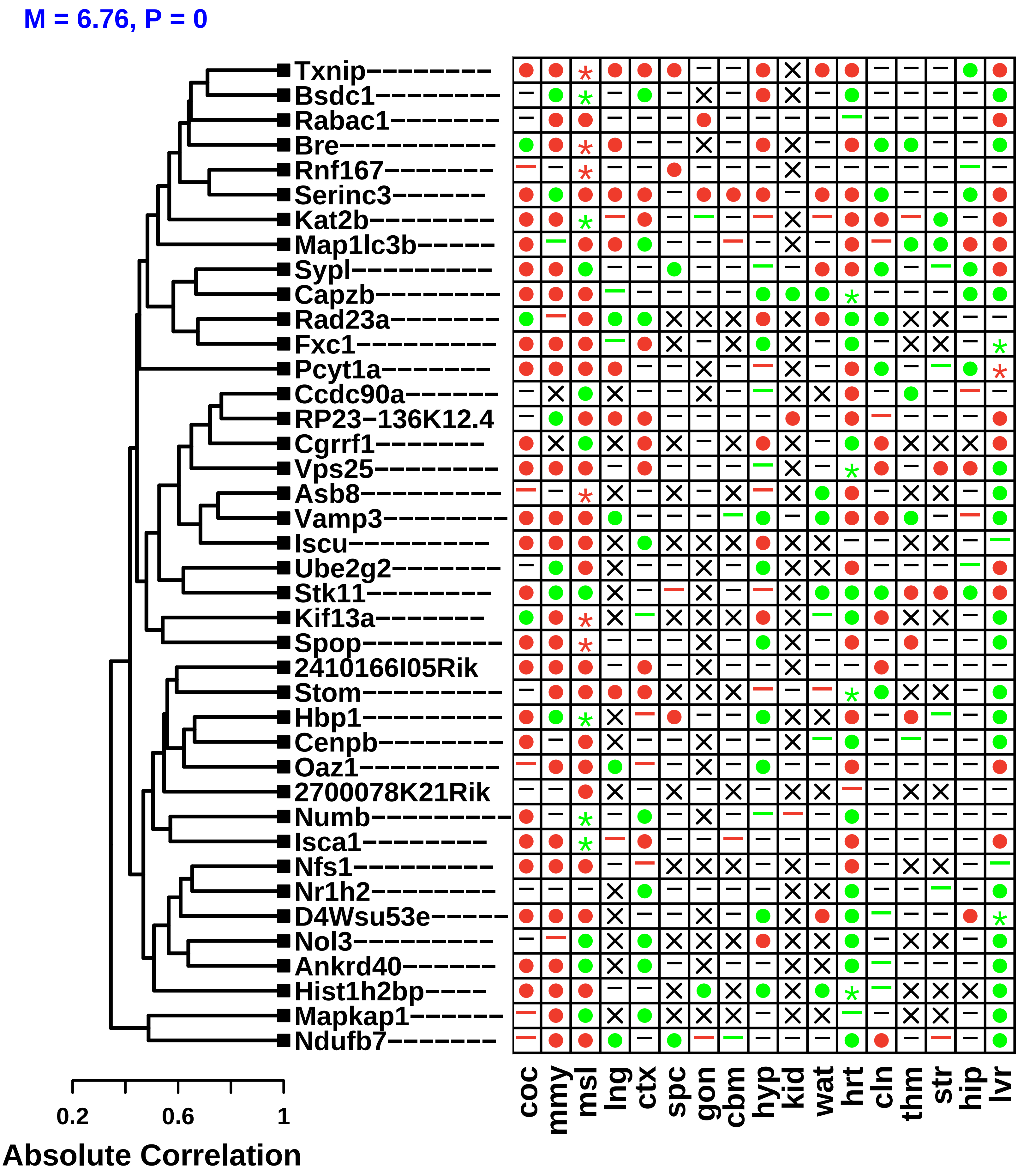

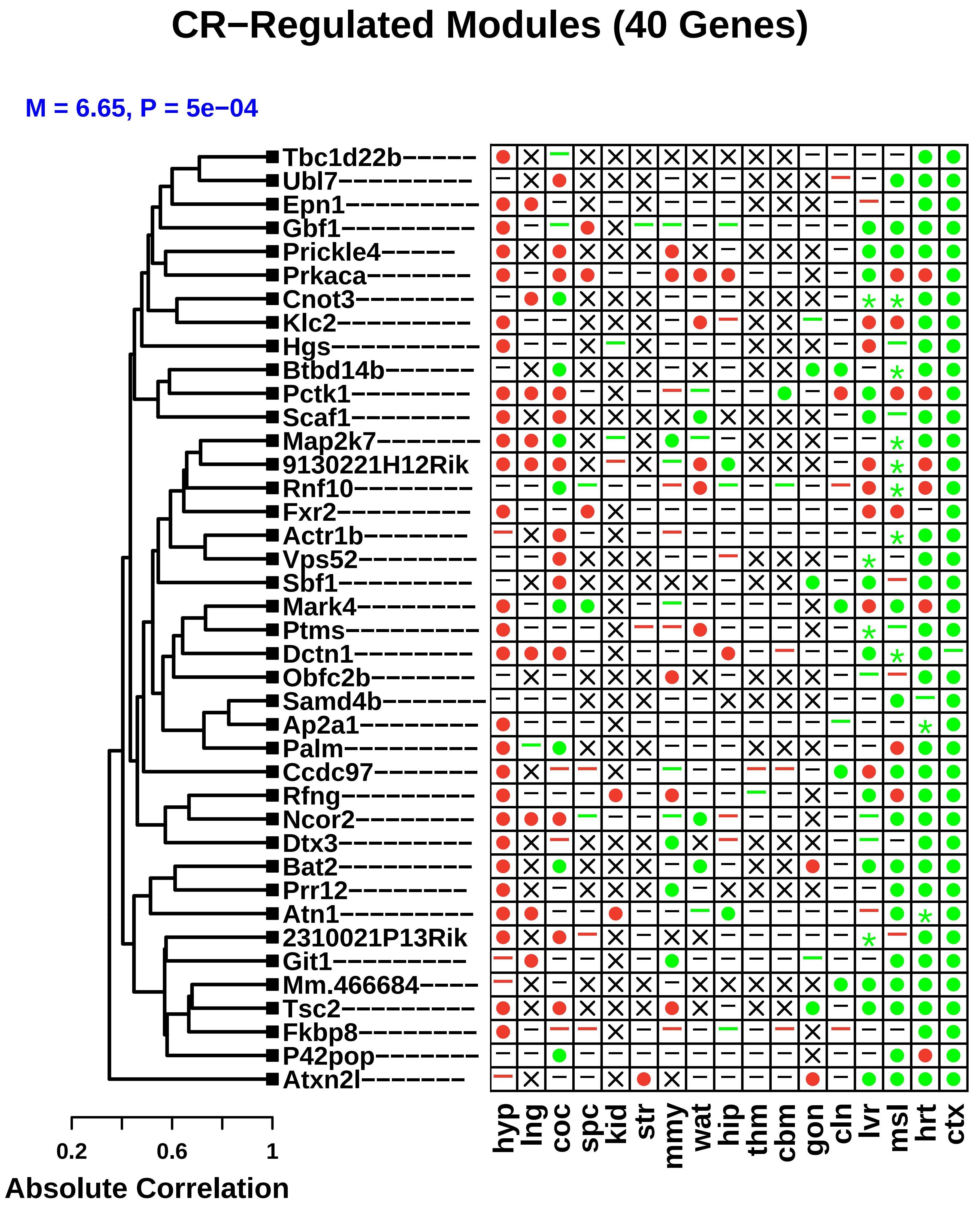
CR−Regulated Modules (40 Genes)
M = 6.65, P = 5e−04
Tbc1d22b
Ubl7
Epn1
Gbf1
Prickle4
Prkaca
Cnot3
Klc2
Hgs
Btbd14b
Pctk1
Scaf1
Map2k7
9130221H12Rik
Rnf10
Fxr2
Actr1b
Vps52
Sbf1
Mark4
Ptms
Dctn1
Obfc2b
Samd4b
Ap2a1
Palm
Ccdc97
Rfng
Ncor2
Dtx3
Bat2
Prr12
Atn1
2310021P13Rik
Git1
Mm.466684
Tsc2
Fkbp8
P42pop
Atxn2l
hyp
lng
coc
spc
kid
str
mmy
wat
hip
thm
cbm
gon
cln
lvr
msl
hrt
ctx
0.2
0.6
1
Absolute Correlation

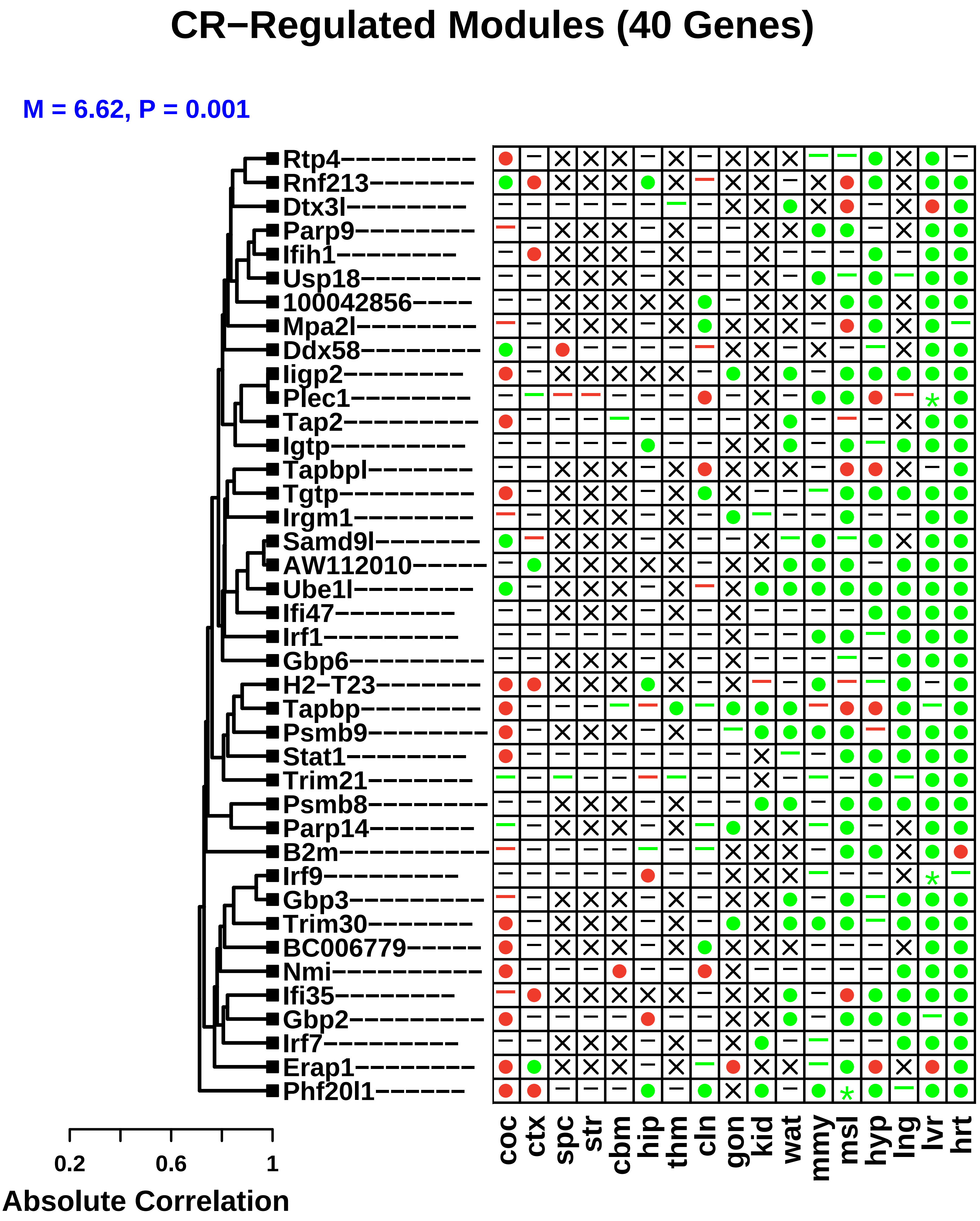
CR−Regulated Modules (40 Genes)
M = 6.62, P = 0.001
Rtp4
Rnf213
Dtx3l
Parp9
Ifih1
Usp18
100042856
Mpa2l
Ddx58
Iigp2
Plec1
Tap2
Igtp
Tapbpl
Tgtp
Irgm1
Samd9l
AW112010
Ube1l
Ifi47
Irf1
Gbp6
H2−T23
Tapbp
Psmb9
Stat1
Trim21
Psmb8
Parp14
B2m
Irf9
Gbp3
Trim30
BC006779
Nmi
Ifi35
Gbp2
Irf7
Erap1
Phf20l1
coc
ctx
spc
str
cbm
hip
thm
cln
gon
kid
wat
mmy
msl
hyp
lng
lvr
hrt
0.2
0.6
1
Absolute Correlation

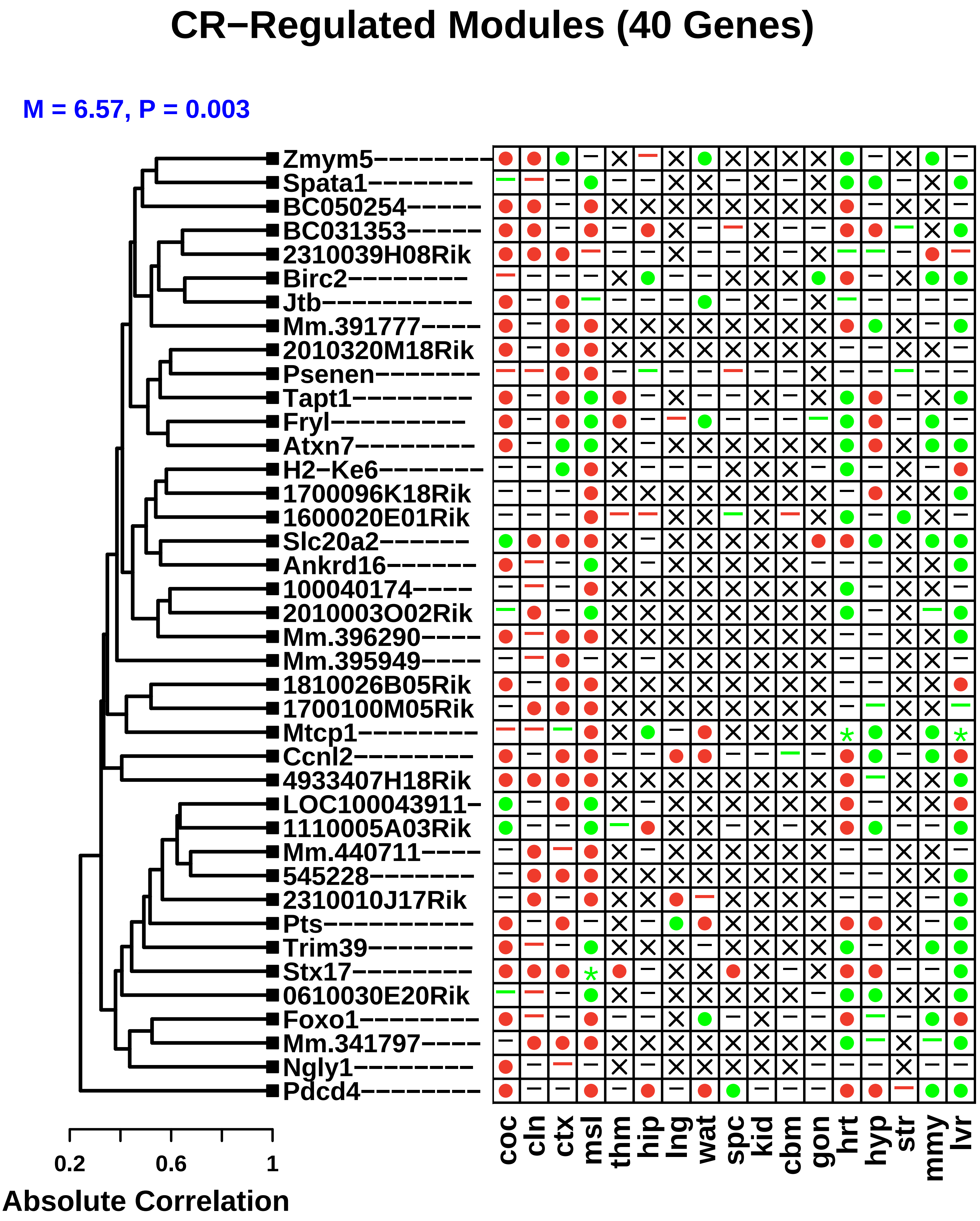
CR−Regulated Modules (40 Genes)
M = 6.57, P = 0.003
Zmym5
Spata1
BC050254
BC031353
2310039H08Rik
Birc2
Jtb
Mm.391777
2010320M18Rik
Psenen
Tapt1
Fryl
Atxn7
H2−Ke6
1700096K18Rik
1600020E01Rik
Slc20a2
Ankrd16
100040174
2010003O02Rik
Mm.396290
Mm.395949
1810026B05Rik
1700100M05Rik
Mtcp1
Ccnl2
4933407H18Rik
LOC100043911
1110005A03Rik
Mm.440711
545228
2310010J17Rik
Pts
Trim39
Stx17
0610030E20Rik
Foxo1
Mm.341797
Ngly1
Pdcd4
coc
cln
ctx
msl
thm
hip
lng
wat
spc
kid
cbm
gon
hrt
hyp
str
mmy
lvr
0.2
0.6
1
Absolute Correlation

CR−Regulated Modules (40 Genes)

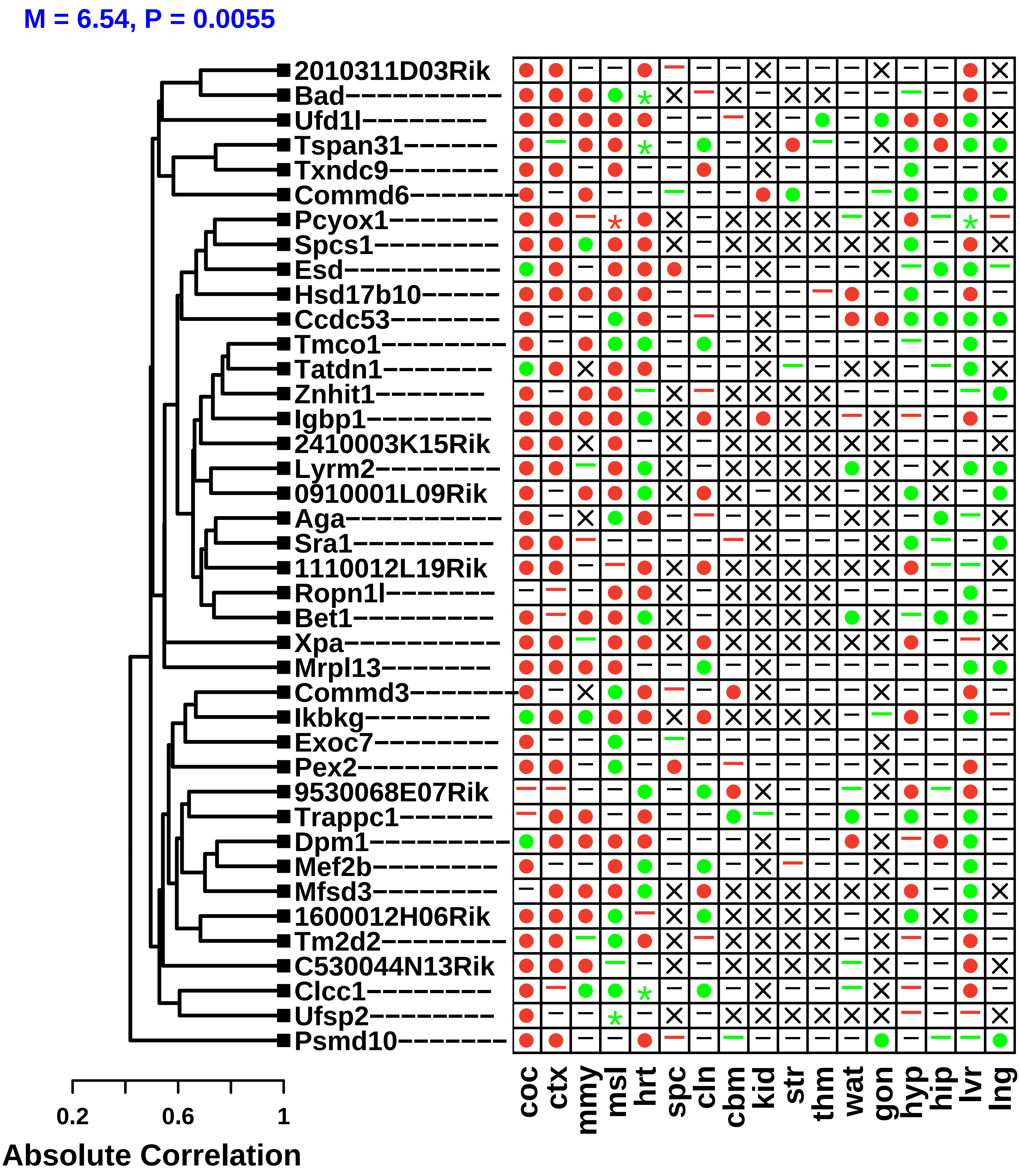

CR−Regulated Modules (40 Genes)

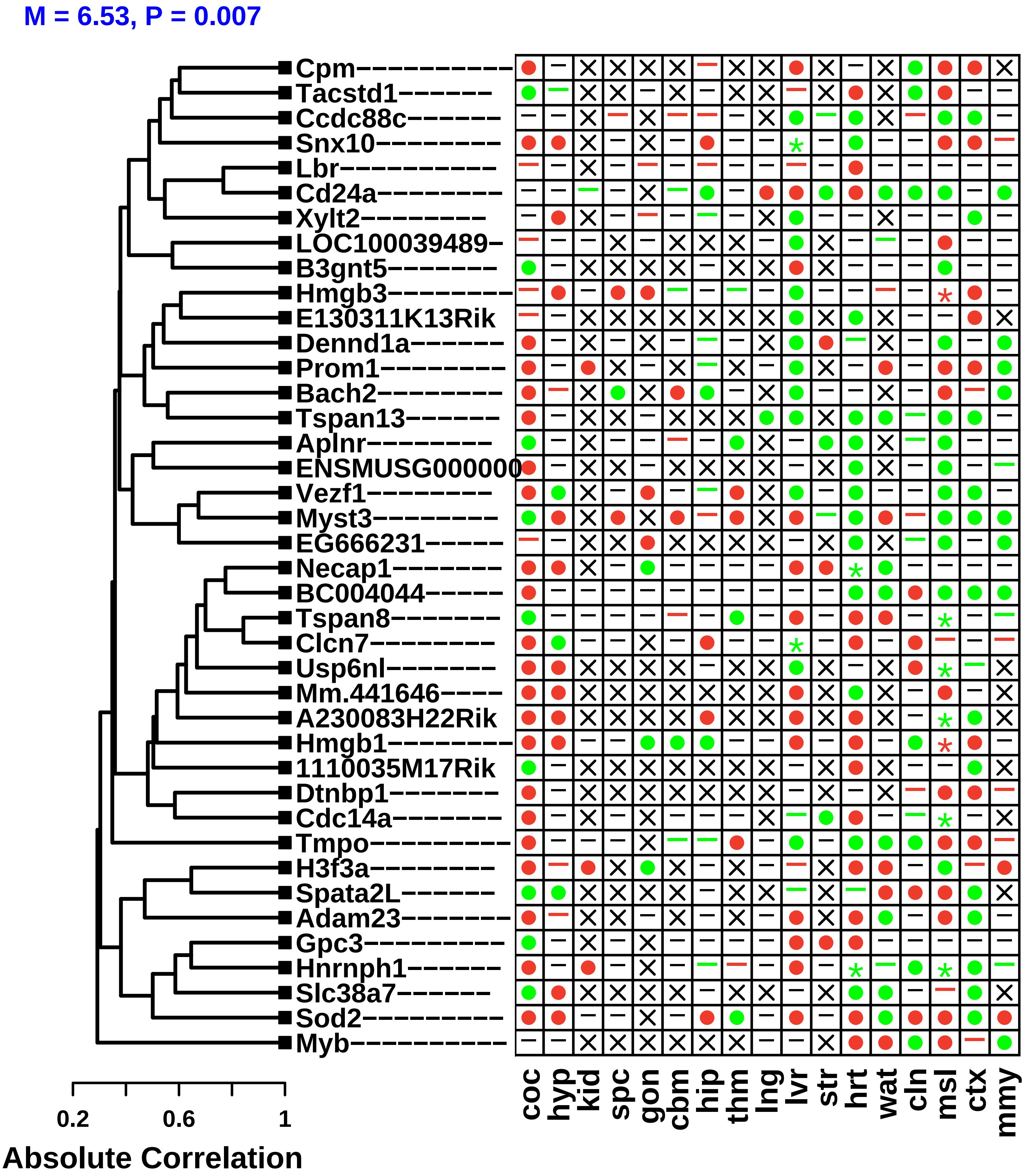

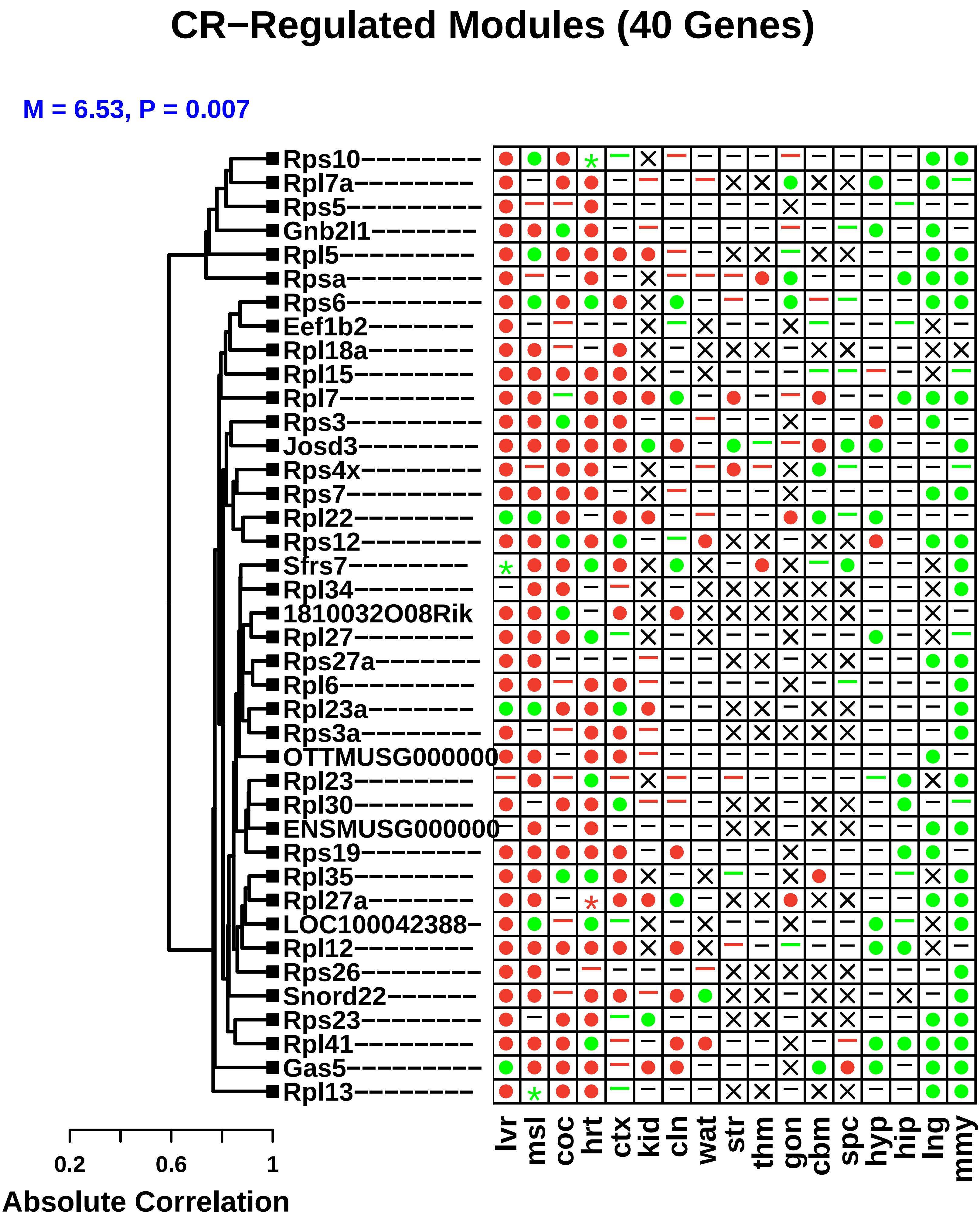
CR−Regulated Modules (40 Genes)
M = 6.53, P = 0.007
Rps10
Rpl7a
Rps5
Gnb2l1
Rpl5
Rpsa
Rps6
Eef1b2
Rpl18a
Rpl15
Rpl7
Rps3
Josd3
Rps4x
Rps7
Rpl22
Rps12
Sfrs7
Rpl34
1810032O08Rik
Rpl27
Rps27a
Rpl6
Rpl23a
Rps3a
OTTMUSG000000
Rpl23
Rpl30
ENSMUSG000000
Rps19
Rpl35
Rpl27a
LOC100042388
Rpl12
Rps26
Snord22
Rps23
Rpl41
Gas5
Rpl13
lvr
msl
coc
hrt
ctx
kid
cln
wat
str
thm
gon
cbm
spc
hyp
hip
lng
mmy
0.2
0.6
1
Absolute Correlation

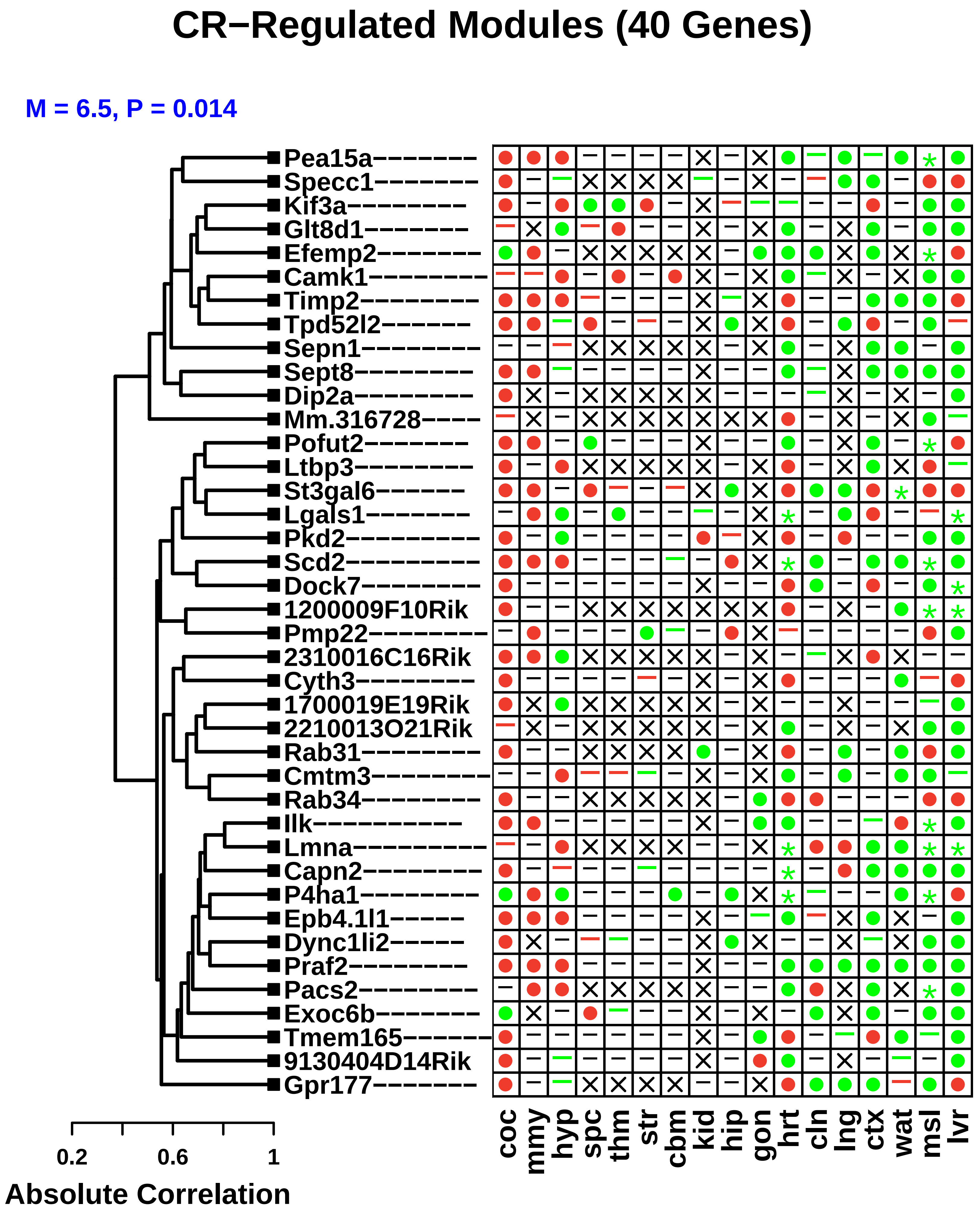

CR−Regulated Modules (40 Genes)
M = 6.5, P = 0.014
Pea15a
Specc1
Kif3a
Glt8d1
Efemp2
Camk1
Timp2
Tpd52l2
Sepn1
Sept8
Dip2a
Mm.316728
Pofut2
Ltbp3
St3gal6
Lgals1
Pkd2
Scd2
Dock7
1200009F10Rik
Pmp22
2310016C16Rik
Cyth3
1700019E19Rik
2210013O21Rik
Rab31
Cmtm3
Rab34
Ilk
Lmna
Capn2
P4ha1
Epb4.1l1
Dync1li2
Praf2
Pacs2
Exoc6b
Tmem165
9130404D14Rik
Gpr177
coc
mmy
hyp
spc
thm
str
cbm
kid
hip
gon
hrt
cln
lng
ctx
wat
msl
lvr
0.2
0.6
1
Absolute Correlation

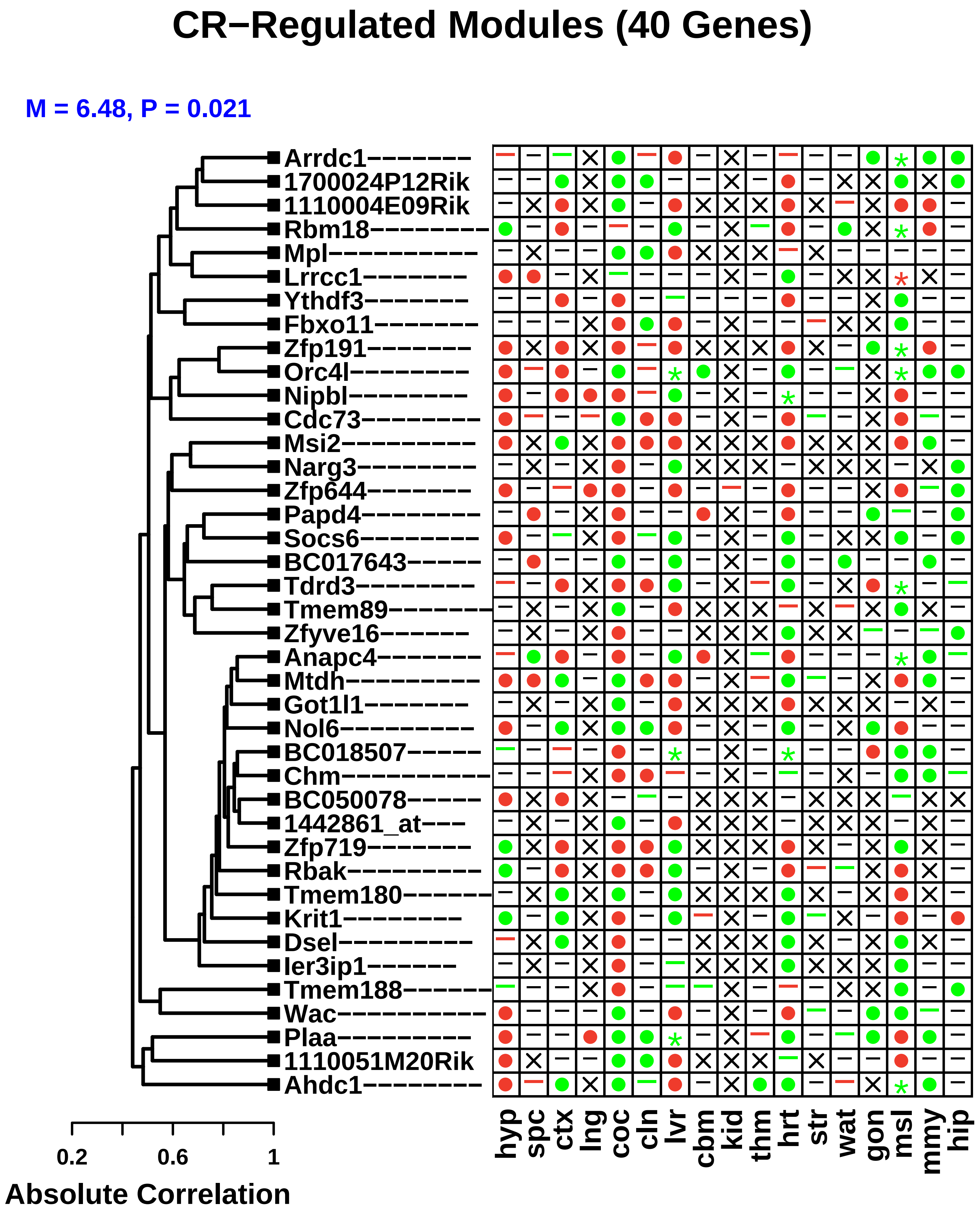
CR−Regulated Modules (40 Genes)
M = 6.48, P = 0.021
Arrdc1
1700024P12Rik
1110004E09Rik
Rbm18
Mpl
Lrrcc1
Ythdf3
Fbxo11
Zfp191
Orc4l
Nipbl
Cdc73
Msi2
Narg3
Zfp644
Papd4
Socs6
BC017643
Tdrd3
Tmem89
Zfyve16
Anapc4
Mtdh
Got1l1
Nol6
BC018507
Chm
BC050078
1442861_at
Zfp719
Rbak
Tmem180
Krit1
Dsel
Ier3ip1
Tmem188
Wac
Plaa
1110051M20Rik
Ahdc1
hyp
spc
ctx
lng
coc
cln
lvr
cbm
kid
thm
hrt
str
wat
gon
msl
mmy
hip
0.2
0.6
1
Absolute Correlation

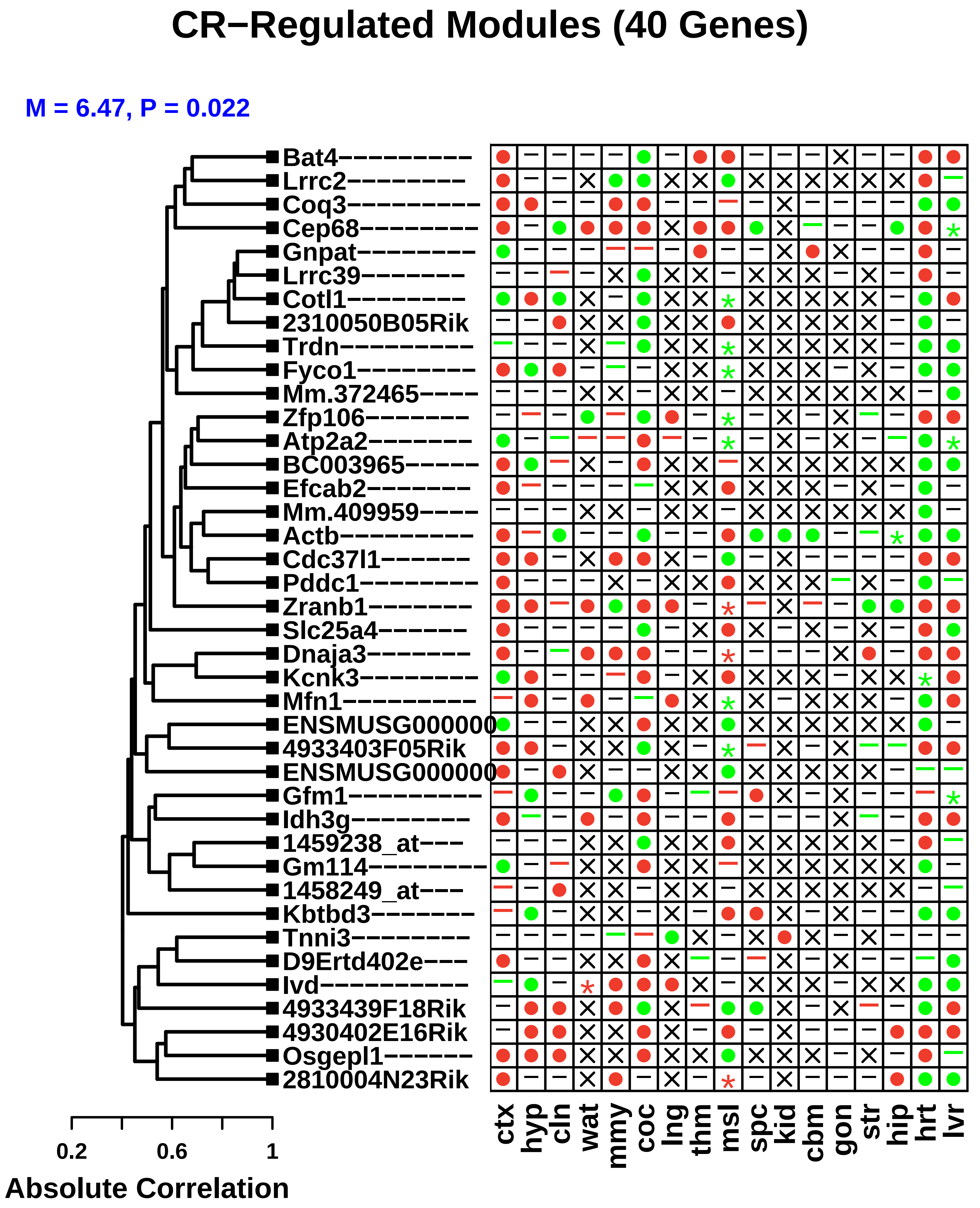
CR−Regulated Modules (40 Genes)
M = 6.47, P = 0.022
Bat4
Lrrc2
Coq3
Cep68
Gnpat
Lrrc39
Cotl1
2310050B05Rik
Trdn
Fyco1
Mm.372465
Zfp106
Atp2a2
BC003965
Efcab2
Mm.409959
Actb
Cdc37l1
Pddc1
Zranb1
Slc25a4
Dnaja3
Kcnk3
Mfn1
ENSMUSG000000
4933403F05Rik
ENSMUSG000000
Gfm1
Idh3g
1459238_at
Gm114
1458249_at
Kbtbd3
Tnni3
D9Ertd402e
Ivd
4933439F18Rik
4930402E16Rik
Osgepl1
2810004N23Rik
ctx
hyp
cln
wat
mmy
coc
lng
thm
msl
spc
kid
cbm
gon
str
hip
hrt
lvr
0.2
0.6
1
Absolute Correlation

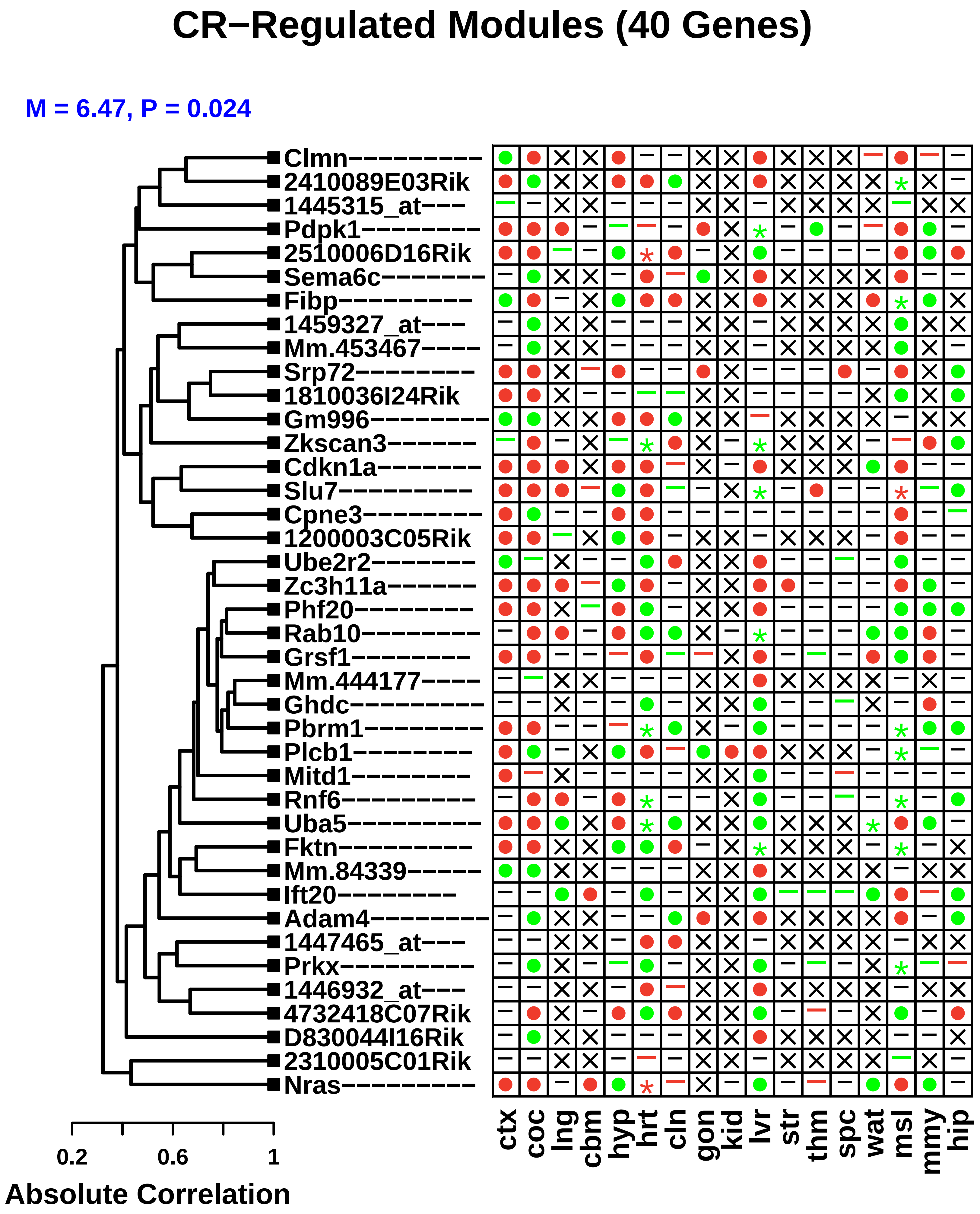
CR−Regulated Modules (40 Genes)
M = 6.47, P = 0.024
Clmn
2410089E03Rik
1445315_at
Pdpk1
2510006D16Rik
Sema6c
Fibp
1459327_at
Mm.453467
Srp72
1810036I24Rik
Gm996
Zkscan3
Cdkn1a
Slu7
Cpne3
1200003C05Rik
Ube2r2
Zc3h11a
Phf20
Rab10
Grsf1
Mm.444177
Ghdc
Pbrm1
Plcb1
Mitd1
Rnf6
Uba5
Fktn
Mm.84339
Ift20
Adam4
1447465_at
Prkx
1446932_at
4732418C07Rik
D830044I16Rik
2310005C01Rik
Nras
ctx
coc
lng
cbm
hyp
hrt
cln
gon
kid
lvr
str
thm
spc
wat
msl
mmy
hip
0.2
0.6
1
Absolute Correlation

CR−Regulated Modules (40 Genes)

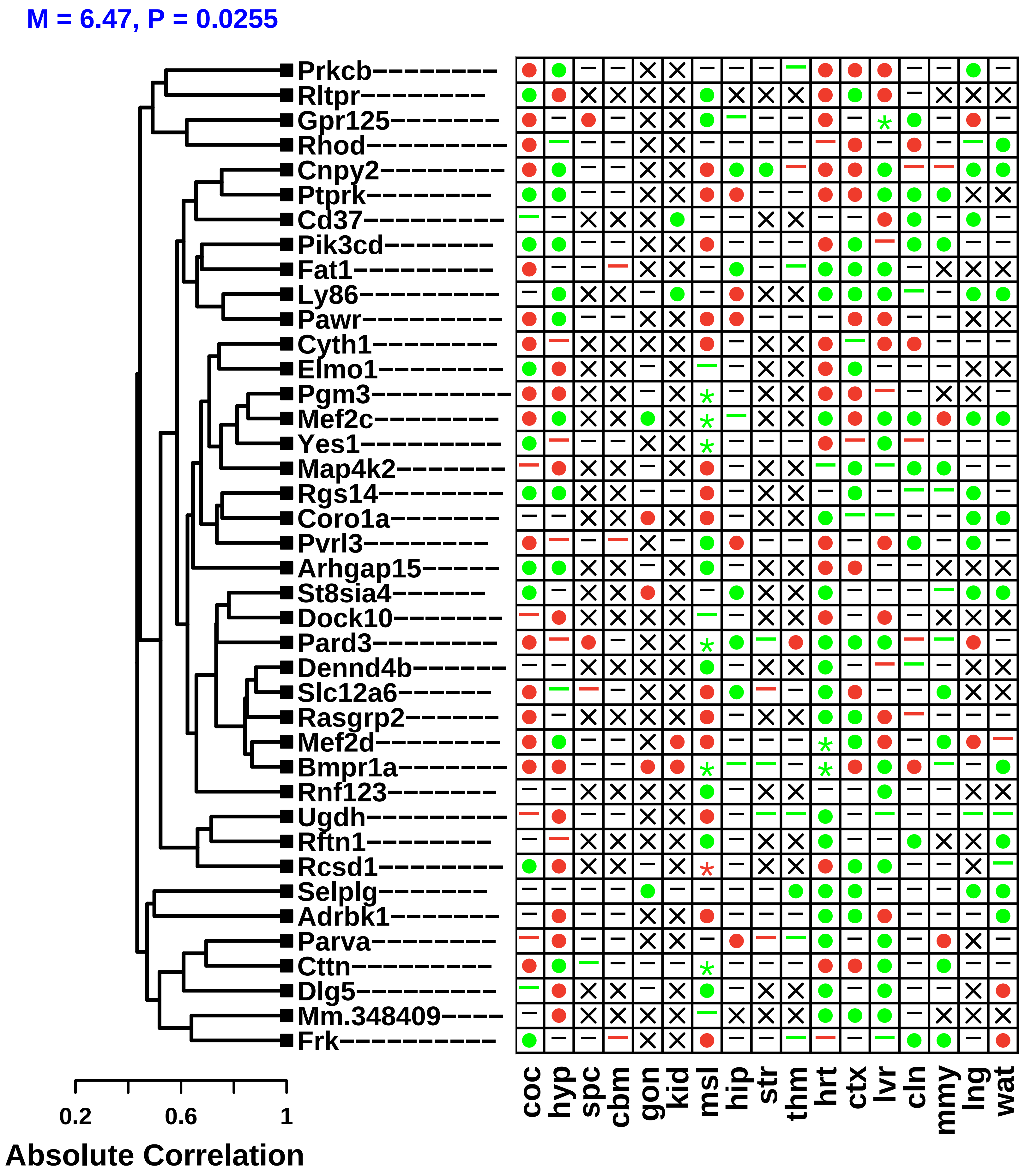

Supplement: Additional file 6 — Gene expression modules regulated by caloric restriction in multiple mouse tissues. This file provides a description of the most significant CR-regulated co-expression modules. Co-expression modules of varying size are shown (2, 3, 5, 10, 20 and 40 genes), along with their patterns of differential expression across the mouse tissues examined (e.g., see Figure 3). [file 1471-2164-10-585-S6.PDF]
