# Supplementary material for: Genes and gene expression modules associated with caloric restriction and aging in the laboratory mouse
Source: BMC Genomics. 2009 Dec 7;10:585. doi: 10.1186/1471-2164-10-585 (PMC2795771; doi:10.1186/1471-2164-10-585)
Supplement: Additional file 7 — Genes regulated by aging in liver. Results from 8 experiments are analyzed to identify genes significantly up and down regulated by aging in liver. This file also includes analysis of associated gene ontology terms, KEGG pathways, microRNA targets and chromosomal locations of age-regulated genes. [file 1471-2164-10-585-S7.PDF]

Additional File 7

Genes and Gene Expression Modules Associated with Caloric Restriction and Aging in the Laboratory Mouse

William R. Swindell
University of Michigan, Departments of Pathology and Geriatrics

---

Genes Regulated by Aging in Liver

This file provides information on genes significantly influenced by aging in the liver. The first set of charts displays differential expression results of the 200 genes most strongly up regulated by aging in liver, while the second set of charts displays differential expression results for the 200 genes most strongly down regulated by aging in liver. Each row corresponds to an individual gene and each column corresponds to a separate experiment (see Additional File 1). Symbols are interpreted as follows:

- ● Gene is significantly up regulated by age ($P_u < 0.05$)
- ● Gene is significantly down regulated by age ($P_d < 0.05$)
- ▬ Gene is marginally up regulated by age ($0.05 < P_u < 0.10$)
- ▬ Gene is marginally down regulated by age ($0.05 < P_d < 0.10$)
- ▬ Non-significant age effect ($P_u > 0.10$ and $P_d > 0.10$)
- × No data (gene not represented in experiment or array annotation was limiting)
- ✳ Evidence conflicts but favors up regulation by age
- ✳ Evidence conflicts, but favors down regulation by age

The last two categories (✳ and ✳) indicate significant effects with conflicting evidence. This can arise if multiple transcripts associated with the same gene symbol yield opposite conclusions. Alternatively, a conflict may arise if $P_u < 0.05$ and also $P_d < 0.05$. Symbols shown in charts are based upon a comparison-wise type I error rate of 0.05. The final column in each chart lists meta-analysis p-values generated using Fisher's method, which have been adjusted using the Benjamini-Hochberg method to control the false discovery rate among all 21,327 genes.

The remainder of the file includes lists of over-represented gene ontology terms, over-represented KEGG pathways, and over-represented KEGG pathways defined based upon IP domain signatures (see Hahne et al. 2008, BMC Bioinformatics 9:3). Genes were also analyzed to determine if there existed an over-abundance of targets for certain microRNAs (see Betel et al. 2008, Nucleic Acids Res. 36: D149-153), and a list of associated microRNAs is provided based upon this analysis. Lastly, tests for over-representation of identified genes with respect to each chromosome were performed, and an idiogram mapping of identified genes to chromosomal locations is shown.

Contact: William R. Swindell, wswindel@umich.edu

↑ Age

Genes up regulated by Age

	lvr2	lvr3	lvr10a	lvr10b	lvr11	lvr20	lvr22	lvr27	P_u
Igh−6	●	●	●	●	●	−	●	●	6.3e−09
Igj	●	●	●	●	●	×	●	●	7.63e−09
Igk−V1	●	●	●	●	●	×	●	−	1.04e−08
Lyz2	●	●	●	●	●	−	●	×	3.91e−08
Tmem176a	●	●	●	●	●	×	●	●	3.91e−08
C1qb	●	●	●	●	●	−	●	●	5.39e−08
Ptprc	●	●	●	●	−	×	●	●	1.53e−07
Pros1	●	●	●	●	●	−	●	●	1.53e−07
Lgmn	●	●	●	●	×	−	●	●	1.62e−07
Csprs	●	●	●	●	×	×	●	●	1.65e−07
LOC100047628	●	●	●	●	×	×	●	×	1.65e−07
100043821	●	●	●	●	●	−	●	×	1.73e−07
C1qa	●	●	●	●	●	−	●	●	1.88e−07
Prg4	●	●	●	●	×	×	●	●	1.88e−07
Steap4	●	●	●	●	×	×	●	●	2.85e−07
Igh	●	●	●	●	*	−	−	×	3.32e−07
Rbm3	●	●	●	●	●	−	●	−	3.32e−07
Vcam1	●	●	−	●	●	×	●	●	3.32e−07
C1qc	●	●	●	●	●	−	●	●	3.32e−07
Cd68	●	●	●	●	−	×	●	●	3.32e−07
Ctss	●	●	−	●	●	×	●	●	3.32e−07
Lgals3	●	●	●	●	●	×	●	×	3.32e−07
Mpeg1	●	●	●	●	●	×	●	−	3.32e−07
Slc3a1	●	−	●	●	−	×	●	●	3.32e−07
Tmem176b	−	●	●	●	−	−	●	●	3.32e−07
Tmsb4x	−	●	−	●	●	−	●	●	3.32e−07
Atp11a	●	●	●	●	−	●	●	●	4.24e−07
Lyz1	●	●	●	●	×	×	●	×	4.24e−07
Ccl6	●	●	−	●	−	×	●	●	4.5e−07
Lcn2	●	●	●	●	●	−	●	×	4.73e−07

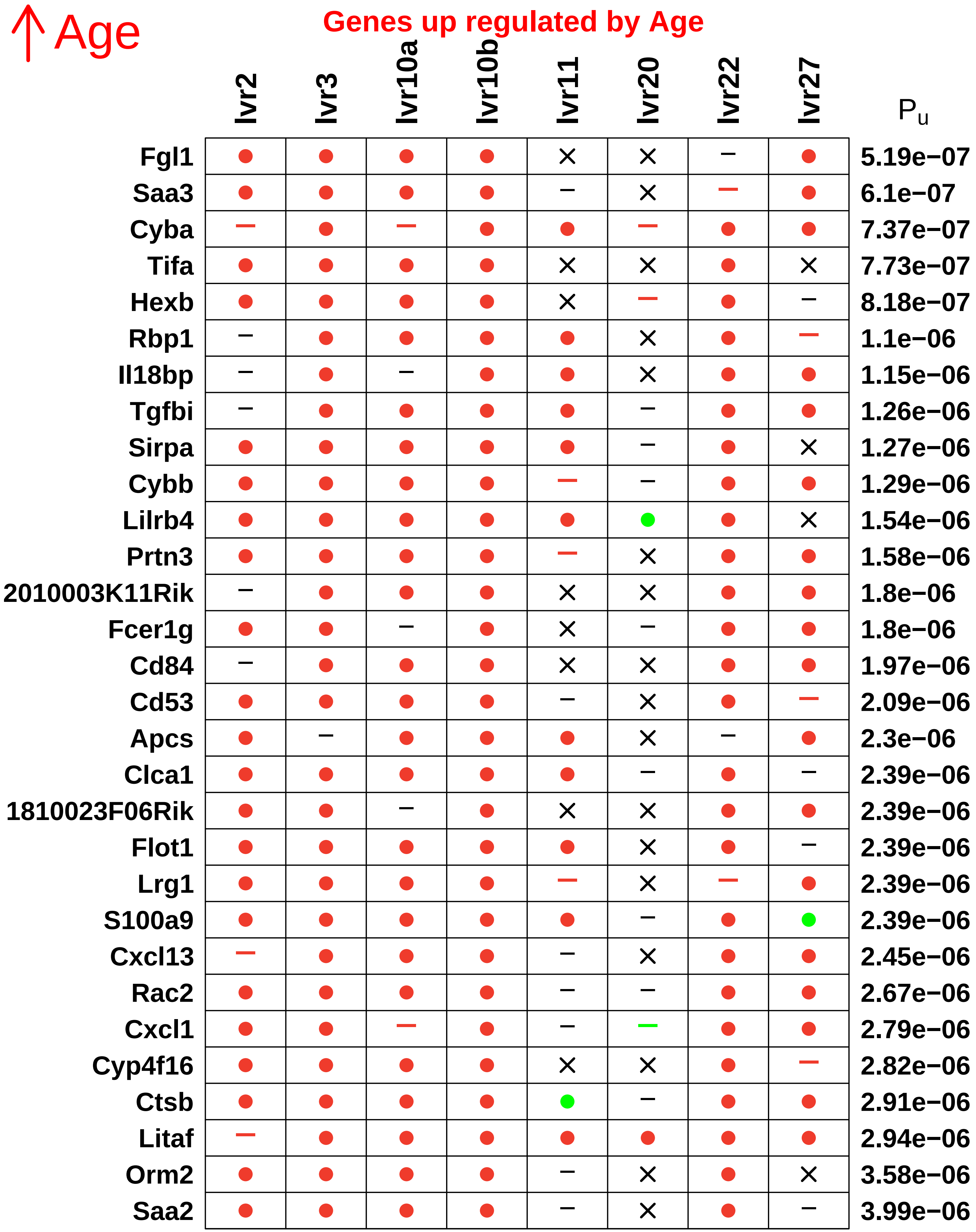

↑ Age
Genes up regulated by Age
lvr2
lvr3
lvr10a
lvr10b
lvr11
lvr20
lvr22
lvr27
Pu
Fgl1
Saa3
Cyba
Tifa
Hexb
Rbp1
Il18bp
Tgfbi
Sirpa
Cybb
Lilrb4
Prtn3
2010003K11Rik
Fcer1g
Cd84
Cd53
Apcs
Clca1
1810023F06Rik
Flot1
Lrg1
S100a9
Cxcl13
Rac2
Cxcl1
Cyp4f16
Ctsb
Litaf
Orm2
Saa2
5.19e−07
6.1e−07
7.37e−07
7.73e−07
8.18e−07
1.1e−06
1.15e−06
1.26e−06
1.27e−06
1.29e−06
1.54e−06
1.58e−06
1.8e−06
1.8e−06
1.97e−06
2.09e−06
2.3e−06
2.39e−06
2.39e−06
2.39e−06
2.39e−06
2.39e−06
2.45e−06
2.67e−06
2.79e−06
2.82e−06
2.91e−06
2.94e−06
3.58e−06
3.99e−06

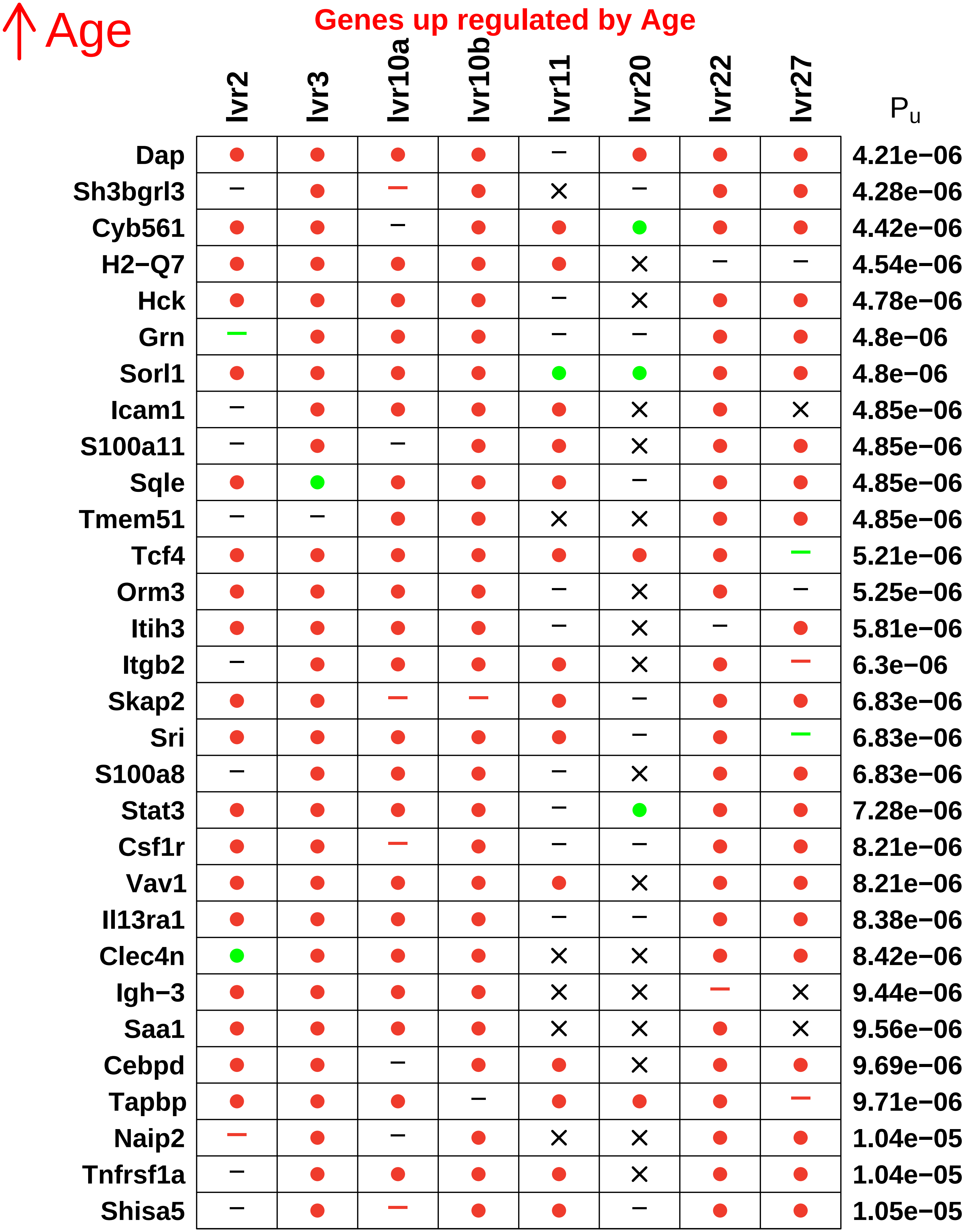
↑ Age
Genes up regulated by Age
lvr2
lvr3
lvr10a
lvr10b
lvr11
lvr20
lvr22
lvr27
P_u
Dap 4.21e−06
Sh3bgrl3 4.28e−06
Cyb561 4.42e−06
H2−Q7 4.54e−06
Hck 4.78e−06
Grn 4.8e−06
Sorl1 4.8e−06
Icam1 4.85e−06
S100a11 4.85e−06
Sqle 4.85e−06
Tmem51 4.85e−06
Tcf4 5.21e−06
Orm3 5.25e−06
Itih3 5.81e−06
Itgb2 6.3e−06
Skap2 6.83e−06
Sri 6.83e−06
S100a8 6.83e−06
Stat3 7.28e−06
Csf1r 8.21e−06
Vav1 8.21e−06
Il13ra1 8.38e−06
Clec4n 8.42e−06
Igh−3 9.44e−06
Saa1 9.56e−06
Cebpd 9.69e−06
Tapbp 9.71e−06
Naip2 1.04e−05
Tnfrsf1a 1.04e−05
Shisa5 1.05e−05

↑ Age

Genes up regulated by Age

	lvr2	lvr3	lvr10a	lvr10b	lvr11	lvr20	lvr22	lvr27	P_u
Cd5l	●	●	–	●	●	×	— (red)	●	1.33e−05
Ctsl	— (red)	●	●	●	●	–	●	●	1.47e−05
Pdgfra	*	●	–	●	●	● (green)	●	— (red)	1.5e−05
Mgat5	●	●	–	–	×	×	●	●	1.54e−05
Tyrobp	●	●	–	●	–	×	●	●	1.54e−05
Coro1a	*	●	●	●	–	×	●	–	1.62e−05
Cttn	●	●	— (red)	●	●	–	● (green)	●	1.8e−05
Cd44	●	●	— (red)	●	–	–	●	●	1.95e−05
Parp3	●	●	●	●	×	×	●	● (green)	1.95e−05
Efemp1	●	●	— (red)	●	×	×	●	–	1.96e−05
Slc13a3	●	●	●	●	×	×	●	●	2.13e−05
USG00000073624	–	●	●	●	×	×	●	×	2.25e−05
Naip5	●	●	–	●	×	×	●	×	2.35e−05
Serpina3n	●	●	–	●	–	×	— (red)	●	2.4e−05
Ms4a4d	●	●	— (red)	●	●	×	●	● (green)	2.4e−05
Plek	–	●	–	●	×	×	●	●	2.4e−05
Ifitm2	●	–	●	●	–	● (green)	●	●	2.55e−05
Mustn1	–	●	–	●	×	×	●	●	2.62e−05
Il1rn	●	●	— (red)	●	●	×	●	●	2.73e−05
Asah3l	●	●	–	●	×	×	●	* (green)	2.74e−05
Dmpk	●	●	●	●	— (red)	×	●	–	2.92e−05
Slc41a2	●	●	●	●	×	×	●	×	2.97e−05
Csrp1	●	●	— (green)	●	●	–	●	●	3.05e−05
Cd48	●	●	–	●	–	×	●	●	3.05e−05
Npc2	●	●	●	●	●	●	●	● (green)	3.06e−05
Igkv1−117	*	●	●	●	●	×	–	×	3.12e−05
Cd52	— (red)	●	— (red)	●	–	×	●	●	3.16e−05
Msn	●	●	●	●	–	— (red)	●	–	3.19e−05
Ear2	–	●	●	●	×	×	●	×	3.31e−05
BC048546	●	●	×	×	×	●	●	×	3.58e−05

↑ Age

Genes up regulated by Age

	lvr2	lvr3	lvr10a	lvr10b	lvr11	lvr20	lvr22	lvr27	P_u
Clu	red ●	red ●	red ●	red ●	–	–	–	red ●	3.71e−05
Cfp	red ●	red ●	–	red ●	red ●	×	red ●	×	3.71e−05
Serpinf2	red ●	–	red ●	red ●	red ●	×	–	red ●	3.75e−05
Sult1e1	red ●	red ●	red ●	–	×	×	red ●	–	3.75e−05
Clec4f	red ●	red ●	–	red ●	red ●	×	red ●	red ●	3.86e−05
Adam11	red ●	red ●	red –	red –	×	×	red ●	red ●	3.95e−05
Plscr1	red ●	–	red ●	red ●	red ●	×	red ●	red ●	4.1e−05
Ccl5	–	red ●	–	red ●	red ●	×	red ●	red ●	4.19e−05
Fcgr4	red ●	red ●	–	red ●	×	×	red ●	×	4.23e−05
Dnajc12	red ●	red ●	red ●	red ●	×	×	red ●	green ●	4.31e−05
Cyp3a13	red ●	red –	red ●	red ●	–	×	–	red ●	4.46e−05
Cp	red ●	red ●	red ●	red ●	–	–	red ●	red ●	4.8e−05
Mm.417626	red ●	red ●	×	×	×	×	red ●	×	4.82e−05
Isyna1	red ●	red ●	–	–	red –	green –	red ●	red ●	4.86e−05
Serpinb1a	red ●	red ●	red ●	red ●	×	–	–	green ●	5.07e−05
Rpap3	red ●	–	red ●	–	–	×	red ●	red ●	5.23e−05
Mmd2	red ●	red ●	red ●	red ●	×	–	red ●	–	5.84e−05
Cnp	red ●	red ●	red ●	red ●	×	×	red –	×	5.84e−05
Ms4a7	–	red ●	red ●	red ●	×	×	red ●	×	5.84e−05
Ncf4	red ●	red ●	red –	red ●	red ●	×	red ●	green ●	5.84e−05
Tbc1d24	red ●	red ●	red ●	red ●	red –	red –	red ●	×	6.05e−05
Sult1a1	red ●	–	red ●	red ●	red ●	×	–	–	6.47e−05
Ly86	–	red ●	red –	red ●	–	×	red ●	red ●	6.83e−05
Stx3	red ●	red ●	red ●	red ●	–	red ●	red ●	green –	7.01e−05
Hmox1	red –	red ●	red ●	red ●	green ●	green ●	red ●	red ●	7.01e−05
Vim	–	red ●	–	red ●	×	–	red ●	red ●	7.2e−05
4933439C20Rik	red ●	red ●	red ●	red ●	×	–	red ●	–	7.71e−05
Plekhb1	red ●	–	red ●	red ●	×	×	red ●	–	7.71e−05
Fcgr3	red ●	red ●	–	red ●	×	–	red ●	red ●	8.15e−05
100040213	red ●	red ●	red ●	red ●	red ●	×	red ●	green –	8.22e−05

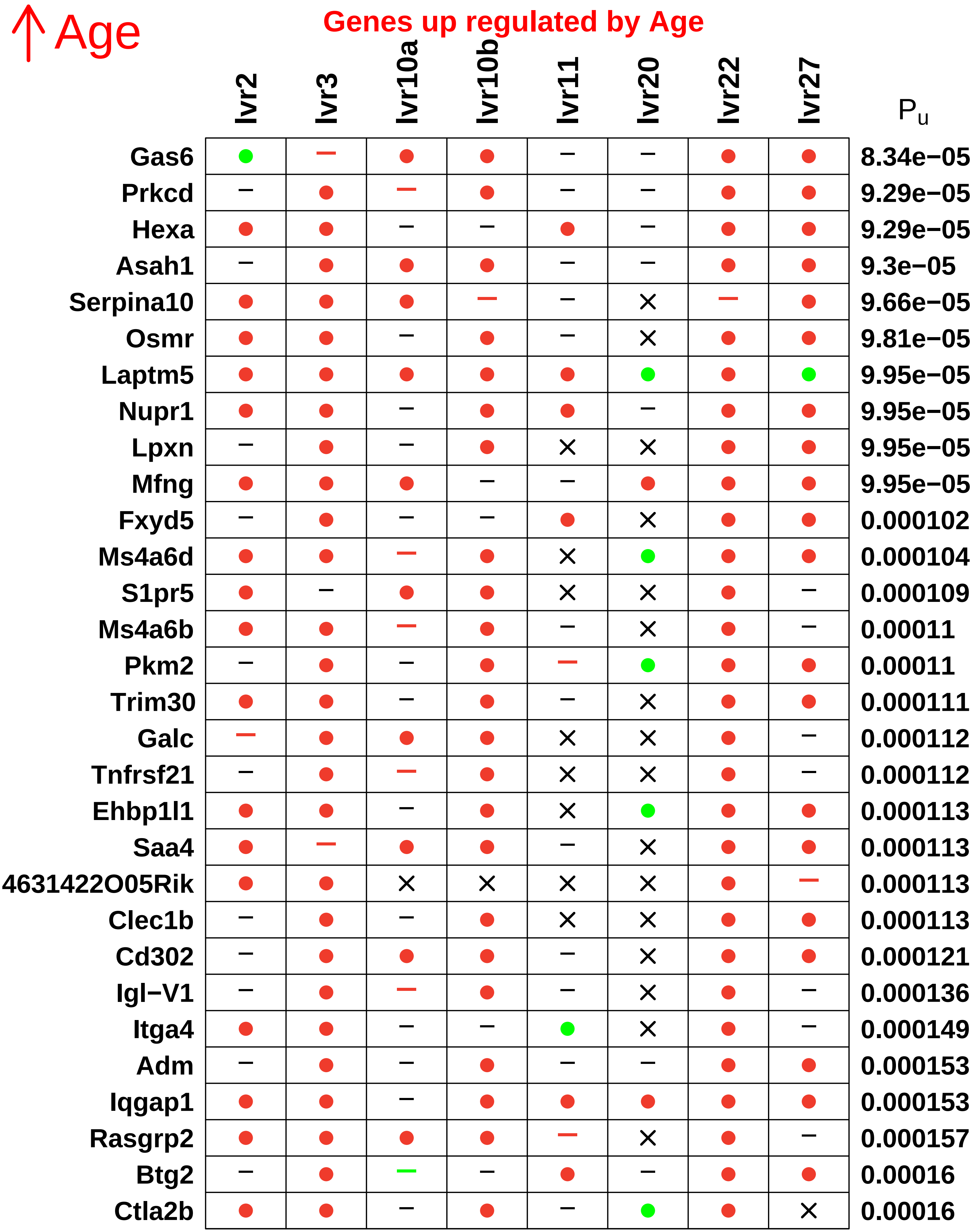

↑ Age
Genes up regulated by Age
lvr2
lvr3
lvr10a
lvr10b
lvr11
lvr20
lvr22
lvr27
P u
Gas6 8.34e−05
Prkcd 9.29e−05
Hexa 9.29e−05
Asah1 9.3e−05
Serpina10 9.66e−05
Osmr 9.81e−05
Laptm5 9.95e−05
Nupr1 9.95e−05
Lpxn 9.95e−05
Mfng 9.95e−05
Fxyd5 0.000102
Ms4a6d 0.000104
S1pr5 0.000109
Ms4a6b 0.00011
Pkm2 0.00011
Trim30 0.000111
Galc 0.000112
Tnfrsf21 0.000112
Ehbp1l1 0.000113
Saa4 0.000113
4631422O05Rik 0.000113
Clec1b 0.000113
Cd302 0.000121
Igl−V1 0.000136
Itga4 0.000149
Adm 0.000153
Iqgap1 0.000153
Rasgrp2 0.000157
Btg2 0.00016
Ctla2b 0.00016

↑ Age

Genes up regulated by Age

	lvr2	lvr3	lvr10a	lvr10b	lvr11	lvr20	lvr22	lvr27	P_u
**Esm1**	–	●	–	–	×	×	●	●	**0.00016**
**Ifi204**	–	●	–	●	●	×	●	●	**0.00016**
**Ppp1r14a**	–	–	●	–	×	×	●	●	**0.00016**
**Emp2**	●	–	●	●	–	×	●	×	**0.00016**
**1451626_x_at**	–	●	–	●	×	×	●	×	**0.000163**
**Ctsc**	●	●	●	●	–	–	●	–	**0.00017**
**Srgn**	–	●	–	●	×	×	●	●	**0.000175**
**Ms4a8a**	●	●	–	–	×	×	●	●	**0.000176**
**Tmem86a**	–	●	×	×	×	×	●	●	**0.000178**
**St6gal1**	●	●	●	●	–	–	–	●	**0.00019**
**Crlf2**	–	●	●	●	×	×	●	–	**0.000192**
**Bin3**	–	●	●	●	×	×	●	●	**0.000193**
**Lbr**	●	–	–	●	●	●	●	●	**0.000193**
**Tmsb10**	●	●	●	●	–	–	●	–	**0.000196**
**Rgs2**	●	●	–	–	–	●	●	●	**2e−04**
**Ctsh**	●	●	●	●	–	–	–	●	**0.000202**
**S100a6**	–	●	–	●	●	–	●	–	**0.000202**
**Cytip**	●	●	–	●	–	×	●	–	**0.000209**
**S100a4**	●	●	–	●	–	×	●	●	**0.000215**
**Tnfaip3**	●	●	–	–	×	×	●	●	**0.000217**

↓ Age

Genes down regulated by Age

	lvr2	lvr3	lvr10a	lvr10b	lvr11	lvr20	lvr22	lvr27	P_d
Keg1	●	●	●	●	●	×	●	●	3.14e−07
Acot1	●	●	●	●	●	×	●	−	1.16e−06
Ces2	●	●	●	●	×	×	●	●	1.16e−06
Car14	●	●	●	●	●	−	●	×	2.63e−06
Mm.466684	●	●	●	●	×	×	●	×	2.63e−06
Hsd3b3	●	●	−	●	●	×	●	●	4.95e−06
Acot2	●	●	●	●	×	×	●	×	7.22e−06
Chchd7	●	●	●	●	−	×	●	×	7.85e−06
Abcd3	●	●	−	●	●	−	●	●	8.01e−06
Slco1a1	−	●	−	●	×	×	●	●	8.01e−06
Ugt2b5	●	●	●	●	−	×	●	×	1.15e−05
Aadac	●	●	●	●	−	×	●	●	1.6e−05
Cdh1	●	●	●	●	●	−	●	●	1.77e−05
Hao1	−	●	●	●	×	×	●	●	1.77e−05
Fabp2	●	●	−	−	●	×	●	●	2.22e−05
Agxt2l1	●	●	×	×	×	×	●	●	2.69e−05
Ehhadh	●	●	−	−	●	×	●	●	2.92e−05
Lifr	−	●	●	●	●	×	●	●	3.38e−05
Acaa1a	●	●	●	●	−	×	●	×	3.54e−05
Acsl1	●	●	●	●	●	−	●	●	3.54e−05
Cpt2	*	●	−	●	●	●	●	●	3.54e−05
Smyd2	●	●	●	●	−	×	●	−	3.54e−05
Abhd6	●	●	−	●	×	×	●	●	3.88e−05
Inmt	−	●	●	●	−	×	−	●	4.63e−05
Pex11a	●	●	−	−	●	−	●	●	6.02e−05
Chchd3	*	●	●	−	×	●	●	●	6.74e−05
Abcg2	●	−	●	●	●	×	−	−	6.74e−05
Lonp2	●	●	−	−	●	−	●	●	6.82e−05
Ndufs3	●	●	−	−	−	−	●	●	6.99e−05
Ndufb3	●	●	−	●	−	×	●	●	7.3e−05

↓ Age

Genes down regulated by Age

	lvr2	lvr3	lvr10a	lvr10b	lvr11	lvr20	lvr22	lvr27	P_d
**Fmo1**	●	●	−	●	●	×	− (green)	●	**7.86e−05**
**Hsd3b2**	−	●	−	●	●	×	●	●	**7.96e−05**
**D0H4S114**	−	●	●	●	−	−	●	●	**9.82e−05**
**BC024137**	●	●	●	●	×	×	●	×	**9.82e−05**
**Acaa1b**	●	●	●	●	●	×	●	×	**0.000122**
**C730048C13Rik**	− (green)	●	−	●	− (green)	×	●	×	**0.000124**
**Aco2**	●	●	●	−	●	− (green)	●	●	**0.000129**
**Dci**	●	− (green)	−	●	●	−	●	●	**0.000139**
**Akr1c20**	− (green)	●	−	●	×	×	●	* (green)	**0.000151**
**Mm.397724**	●	●	×	×	×	×	●	×	**0.00016**
**Cyp4v3**	−	●	●	●	×	×	●	●	**0.000175**
**Me1**	●	●	●	●	−	●	●	×	**0.000202**
**Cyc1**	●	●	●	− (green)	−	− (green)	−	●	**0.000202**
**Mgll**	●	●	−	− (green)	− (green)	●	●	●	**0.000205**
**Rps4y2**	●	−	●	●	−	×	●	●	**0.00026**
**Ndufa10**	●	●	− (green)	−	−	●	●	●	**0.000276**
**Car3**	●	●	●	●	−	×	●	●	**0.000364**
**Chmp7**	●	●	− (green)	−	−	●	●	×	**0.000364**
**Ecsit**	●	●	−	●	×	×	−	●	**0.000367**
**Lactb2**	●	●	−	●	●	×	●	●	**0.00037**
**Ppara**	* (red)	●	●	−	●	×	●	●	**0.000396**
**Mal2**	●	●	− (green)	●	×	×	●	×	**0.000444**
**Dio1**	●	●	●	●	− (green)	×	●	−	**0.000468**
**Timd2**	●	●	−	−	●	−	−	●	**0.00054**
**Ugt2b1**	−	●	●	●	×	×	●	×	**0.00054**
**Cideb**	●	− (green)	−	− (red)	●	×	●	●	**0.00059**
**Fabp1**	●	●	●	●	−	×	−	●	**0.000635**
**Cyp2a12**	●	●	●	− (green)	●	×	−	●	**0.000641**
**Mrpl38**	−	●	−	●	−	●	−	●	**0.000665**
**Ndufa12**	* (green)	●	●	●	×	−	●	−	**0.000665**

↓ Age

Genes down regulated by Age

	lvr2	lvr3	lvr10a	lvr10b	lvr11	lvr20	lvr22	lvr27	P_d
9530058B02Rik	green ●	green ●	green –	green ●	×	×	–	green ●	0.000665
Aadat	–	green ●	green ●	–	green ●	×	green ●	×	0.000665
Aldh3a2	green ●	green ●	green –	–	green ●	–	green ●	green ●	0.000665
Cyp8b1	green ●	green ●	green –	green ●	green ●	×	green ●	–	0.000665
1200015F23Rik	green –	green ●	green –	green ●	–	×	–	green ●	0.000693
Afmid	red ●	green ●	–	–	×	×	green ●	green ●	0.000693
Rangap1	red ●	green ●	green ●	green ●	–	–	green ●	–	0.000716
Fkbp4	–	green ●	green ●	green ●	×	red ●	–	green ●	0.000726
Atp5k	green ●	green ●	–	–	×	green ●	green ●	green ●	0.000745
Gsta3	green ●	green ●	–	–	green –	×	–	green ●	0.000745
Cyp2d13	red ●	green ●	green ●	green ●	×	×	green ●	red ●	0.000871
1810009N02Rik	–	green ●	–	green ●	×	×	green ●	green ●	0.00093
Ak2	green ●	green ●	–	–	–	green –	green ●	green ●	0.000981
Fzd4	green ●	green ●	–	–	green ●	×	–	green ●	0.000981
Letm1	green ●	green ●	green –	green ●	×	–	green ●	green ●	0.000981
AW111846	green ●	green ●	×	×	×	×	green ●	×	0.000985
Srd5a1	green ●	green ●	×	×	×	×	green ●	red –	0.00104
8430406I07Rik	green ●	green ●	–	green ●	×	–	green –	green ●	0.00112
Rab14	red ●	green ●	red –	green ●	–	–	green ●	green ●	0.00114
Pdk2	green ●	green ●	–	–	×	×	–	green ●	0.00121
Aifm2	red ●	green ●	×	×	×	×	green ●	green ●	0.00122
Acsm2	–	green ●	green ●	green ●	–	×	green ●	×	0.00132
Gclc	green ●	green ●	–	green ●	green ●	–	green ●	green ●	0.00132
Pvrl2	green ●	green ●	–	green ●	–	green ●	green ●	green –	0.00132
Retsat	green ●	green ●	–	green ●	×	red ●	green ●	×	0.00132
Ubxn4	green ●	red ●	green ●	green ●	green ●	green ●	green –	×	0.00132
Usp10	green ●	green ●	–	green –	–	green ●	green ●	green ●	0.00132
Onecut1	–	green ●	green –	green ●	–	–	green ●	green ●	0.00138
2810007J24Rik	green –	green ●	×	×	×	×	green ●	green ●	0.00138
Hsd17b2	green –	green ●	green ●	green ●	red ●	×	green ●	–	0.00138

↓ Age

Genes down regulated by Age

	lvr2	lvr3	lvr10a	lvr10b	lvr11	lvr20	lvr22	lvr27	P_d
2310061I04Rik	– (green)	● (green)	–	● (green)	● (green)	×	● (green)	×	0.00143
Decr1	● (green)	● (green)	–	● (green)	– (red)	×	● (green)	● (green)	0.00143
Prpf6	– (green)	● (green)	● (green)	● (green)	–	–	● (green)	● (green)	0.00143
Ndufa5	● (green)	● (green)	–	–	–	×	● (green)	● (green)	0.0015
Nudt7	● (green)	● (green)	● (green)	● (green)	×	–	● (green)	● (green)	0.0015
Mup5	– (green)	● (green)	● (green)	● (green)	–	×	● (green)	–	0.0015
Hsd3b6	–	● (green)	● (green)	● (green)	● (green)	×	● (green)	×	0.00154
Cox7c	* (red)	● (green)	– (red)	–	● (green)	–	● (green)	● (green)	0.00154
Lman1	* (green)	● (green)	● (green)	● (green)	–	–	● (green)	–	0.00154
Zfp109	● (green)	● (red)	–	● (green)	×	×	● (green)	● (green)	0.00154
Epb4.1l4b	● (green)	● (green)	–	– (green)	–	● (green)	● (green)	–	0.00158
Gas1	● (green)	● (green)	● (green)	–	● (green)	–	● (green)	● (green)	0.00166
Uqcrc1	–	● (green)	● (green)	● (green)	–	–	● (green)	● (green)	0.00168
Rap2a	– (green)	● (green)	–	● (green)	×	×	● (green)	–	0.00176
1700007B13Rik	● (red)	● (green)	×	×	×	×	● (green)	● (green)	0.00178
Mfn2	● (green)	● (green)	–	–	×	×	–	● (green)	0.00178
Timm9	– (red)	● (green)	● (green)	● (green)	–	–	● (green)	● (green)	0.00178
Zmiz1	* (red)	● (green)	– (green)	–	×	● (green)	● (green)	×	0.00178
Dcakd	● (green)	● (green)	–	● (green)	×	– (green)	● (green)	● (green)	0.00178
Ghr	● (green)	● (green)	● (green)	– (green)	● (green)	–	● (green)	–	0.0018
Pcgf3	● (green)	● (green)	×	×	×	● (green)	–	×	0.0018
Pebp1	● (green)	* (red)	–	–	×	×	● (green)	● (green)	0.00189
Atp5c1	* (green)	● (green)	–	–	● (green)	–	● (green)	–	0.002
Cyp2d9	● (green)	–	● (green)	● (green)	–	×	● (green)	×	0.002
Comt1	– (green)	● (green)	● (green)	● (green)	–	–	–	● (green)	0.002
Hes6	– (green)	● (green)	● (green)	● (green)	● (red)	×	–	● (green)	0.002
Ephx2	● (green)	● (green)	–	● (green)	● (green)	–	–	● (green)	0.002
Mpst	● (green)	● (green)	–	– (red)	×	×	–	● (green)	0.002
Slc25a22	● (green)	–	–	● (green)	×	● (green)	– (green)	● (green)	0.002
Ephx1	● (green)	–	–	● (green)	● (green)	–	–	● (green)	0.00209

↓ Age

Genes down regulated by Age

	lvr2	lvr3	lvr10a	lvr10b	lvr11	lvr20	lvr22	lvr27	P_d
Nr1i3	−	🟢	−	−	🟢	×	🟢	🟢	0.00216
Prox1	− (green)	🟢	−	−	−	×	🟢	🔴	0.00216
AU018778	🟢	🟢	− (green)	−	×	−	🟢	🟢	0.00216
Rorc	🟢	🟢	−	🟢	🟢	×	−	🟢	0.00218
Serpina4−ps1	− (green)	🟢	×	×	×	×	🟢	×	0.00224
Ugt2b34	🟢	🟢	−	🟢	− (green)	×	🟢	×	0.00224
Lyrm5	🟢	🟢	−	−	×	×	🟢	×	0.00228
Sdhb	🟢	🟢	−	−	− (green)	−	🟢	🟢	0.0023
Cog4	− (red)	🟢	−	🟢	×	🟢	🟢	🟢	0.00244
Abca8a	−	🟢	−	🟢	×	×	🟢	×	0.00249
Mm.393484	−	🟢	🟢	− (green)	×	×	🟢	🟢	0.00249
Kynu	🔴	🟢	−	−	×	×	🟢	🟢	0.00251
Mycbp	🟢	🟢	−	🟢	−	−	🔴	🟢	0.00251
Slc25a30	🟢	🟢	−	− (green)	×	×	🟢	×	0.00251
Cyp4a14	−	−	−	−	🟢	×	🟢	🟢	0.00251
Mm.132956	− (green)	🟢	×	×	×	×	🟢	×	0.00251
Bri3bp	🟢	🟢	×	×	×	×	🟢	− (green)	0.00254
Hmgcs2	−	🟢	− (green)	− (green)	🟢	−	−	🟢	0.00254
Hsd3b5	−	🟢	−	🟢	−	×	🟢	− (green)	0.00254
Ugt2b37	−	🟢	🟢	🟢	×	×	🟢	×	0.00254
3110049J23Rik	−	🟢	− (green)	🟢	×	×	− (green)	🟢	0.00255
Ces5	− (green)	🟢	🟢	−	×	×	🟢	🟢	0.00261
Brd3	🟢	🟢	−	−	×	−	🟢	−	0.00262
Idh3b	🟢	🟢	−	🟢	×	−	🟢	🟢	0.00262
Sdha	🟢	🟢	−	🟢	×	−	🟢	🟢	0.00262
Slc25a20	🟢	🟢	−	−	🟢	−	🟢	🟢	0.00262
Cpox	🟢	🟢	−	−	−	−	− (green)	🟢	0.00265
Aox3	−	🟢	− (green)	🟢	×	−	−	🟢	0.00267
Cml5	🟢	🟢	−	🟢	×	×	−	− (green)	0.0027
Nek4	🟢	🟢	🟢	− (green)	−	×	− (green)	−	0.00286

↓ Age

Genes down regulated by Age

	lvr2	lvr3	lvr10a	lvr10b	lvr11	lvr20	lvr22	lvr27	P_d
Elovl2	–	● (green)	–	–	–	×	● (green)	● (green)	0.00286
Mrpl37	–	● (green)	— (green)	● (green)	–	×	— (green)	● (green)	0.00286
Suclg1	● (green)	● (green)	–	–	–	–	● (green)	● (green)	0.00322
Ccdc84	— (green)	● (green)	–	— (green)	×	×	● (green)	×	0.00332
Cryl1	— (red)	* (red)	–	–	● (green)	–	— (green)	● (green)	0.00332
Elp4	● (red)	● (green)	–	● (green)	×	–	● (green)	● (green)	0.00332
Svs5	● (green)	— (green)	● (green)	● (green)	● (green)	×	● (green)	● (red)	0.00334
Dnase2a	● (green)	● (green)	–	— (green)	×	● (green)	— (red)	● (red)	0.00334
Paqr7	● (green)	● (green)	–	–	● (green)	×	● (green)	×	0.00348
Nat12	— (green)	● (green)	–	–	×	–	● (green)	● (green)	0.00352
Bysl	— (green)	● (green)	● (green)	● (green)	● (green)	● (green)	–	● (green)	0.0036
Myo1e	–	● (green)	–	–	— (red)	● (green)	● (green)	● (green)	0.00365
Bbs7	● (green)	● (green)	×	×	×	— (green)	● (green)	–	0.00369
Cyp2j5	–	● (green)	–	–	–	×	● (green)	● (green)	0.00369
Mrpl39	— (green)	● (green)	–	–	● (green)	×	● (green)	● (green)	0.00369
Ociad1	–	● (green)	● (green)	–	— (green)	— (green)	● (green)	— (green)	0.00369
Sap18	● (red)	* (green)	–	● (green)	–	● (red)	● (green)	● (green)	0.00369
Ube2h	● (green)	● (green)	–	–	–	×	● (green)	● (green)	0.00369
1300001I01Rik	–	● (green)	–	● (green)	×	● (green)	— (green)	● (green)	0.00369
Atrn	● (green)	● (green)	–	–	×	— (green)	● (green)	● (green)	0.00375
D1Ertd53e	● (green)	* (green)	–	— (green)	● (green)	×	● (green)	×	0.00378
Nelf	● (green)	● (green)	–	● (green)	×	×	● (green)	● (green)	0.00378
Suds3	–	● (green)	–	–	● (green)	×	–	● (green)	0.00378
Mrpl47	–	● (green)	–	● (green)	×	×	● (green)	● (green)	0.00384
Cecr2	— (red)	● (green)	×	×	×	— (green)	— (green)	● (green)	0.00387
Mm.473985	● (green)	● (green)	×	×	×	×	● (green)	×	0.00391
2410001C21Rik	● (green)	— (green)	–	–	–	● (green)	● (green)	● (green)	0.00401
1300010F03Rik	–	● (green)	–	–	×	— (red)	● (green)	● (green)	0.00405
Map1lc3a	● (green)	● (green)	–	● (green)	● (red)	–	— (green)	×	0.0042
Slc25a17	— (green)	● (green)	● (green)	● (green)	–	–	— (green)	● (green)	0.00424

↓ Age

Genes down regulated by Age

	lvr2	lvr3	lvr10a	lvr10b	lvr11	lvr20	lvr22	lvr27	P_d
Baat	●	●	●	−	−	×	—	●	0.00434
Nfic	●	●	—	●	−	−	—	●	0.00434
Accn5	−	●	−	●	●	×	—	−	0.00436
Atp5l	●	●	−	−	−	−	●	●	0.00436
G6pc	−	−	−	—	●	×	●	●	0.00438
Adipor2	●	●	●	●	—	−	●	×	0.00439
EG13909	—	●	●	●	−	×	●	×	0.00442
Pgrmc1	●	●	−	−	—	−	−	●	0.00442
Sec14l2	—	●	−	●	●	−	—	●	0.00449
Smcr7l	●	●	×	×	×	×	−	×	0.00453
Cebpa	●	●	●	−	−	×	−	●	0.00453
Ulk2	●	●	—	●	●	●	●	−	0.00455
Cyp1a2	−	●	−	−	−	×	●	●	0.00455
Ppme1	−	−	—	●	×	−	—	●	0.00455
Rg9mtd1	—	●	−	−	×	—	—	●	0.00466
Hspa1b	−	−	●	●	● (red)	×	●	●	0.0048
Sult5a1	−	●	●	−	—	×	●	−	0.00484
Lad1	●	−	−	●	×	−	●	—	0.00487
Atp5j	●	●	−	−	●	−	—	●	0.0049
[illegible]USG00000073738	−	●	×	×	×	×	●	×	0.0049

↑Age

Overrepresented Biological Processes

GO Term	P−Value
immune response	1.16e−11
positive regulation of response to stimulus	7.4e−10
immune effector process	4.38e−09
activation of immune response	5.92e−09
positive regulation of phagocytosis	1.58e−08
lymphocyte mediated immunity	1.94e−08
positive regulation of leukocyte activation	4.29e−08
leukocyte adhesion	9.61e−08
adaptive immune response based on somatic recombination of immune receptors built from immunoglobul	2.16e−07
antigen processing and presentation of peptide or polysaccharide antigen via MHC class II	2.55e−07
antigen processing and presentation of exogenous antigen	3.07e−07
acute−phase response	1.04e−06
positive regulation of adaptive immune response based on somatic recombination of immune receptors bu	1.17e−06
antigen processing and presentation of exogenous peptide antigen via MHC class II	1.6e−06
regulation of adaptive immune response	2.13e−06
activation of plasma proteins during acute inflammatory response	2.96e−06
regulation of endocytosis	5.5e−06
inflammatory response	7.01e−06
cytokine and chemokine mediated signaling pathway	7.07e−06
complement activation, classical pathway	8.26e−06
positive regulation of inflammatory response to antigenic stimulus	8.72e−06
regulation of acute inflammatory response to antigenic stimulus	8.72e−06
regulation of defense response	1.45e−05
regulation of cell activation	1.89e−05
positive regulation of type IIa hypersensitivity	1.9e−05
regulation of type II hypersensitivity	1.9e−05
cell death	1.99e−05
positive regulation of immune effector process	2.11e−05
antigen processing and presentation of peptide antigen	2.55e−05
positive regulation of transport	3.19e−05

↑Age

Overrepresented Biological Processes

GO Term	P−Value
cell activation	3.22e−05
regulation of response to external stimulus	3.36e−05
cholesterol biosynthetic process	4.46e−05
locomotory behavior	5.07e−05
regulation of lymphocyte activation	5.07e−05
regulation of localization	5.48e−05
positive regulation of acute inflammatory response	5.49e−05
defense response to Gram−positive bacterium	5.49e−05
membrane organization and biogenesis	5.65e−05
mast cell activation	6.32e−05
regulation of B cell mediated immunity	6.43e−05
positive regulation of cellular component organization and biogenesis	8.41e−05
phagocytosis, engulfment	0.000106
positive regulation of lymphocyte mediated immunity	0.000107
regulation of leukocyte mediated immunity	0.000121
negative regulation of fibroblast proliferation	0.000124
localization of cell	0.000149
positive regulation of immune system process	0.000166
apoptosis	0.000182
innate immune response	0.000252
response to wounding	0.000298
positive regulation of type I hypersensitivity	0.000342
negative regulation of fat cell differentiation	0.000342
defense response to Gram−negative bacterium	0.000342
positive regulation of T cell activation	0.000361
phagocytosis, recognition	0.000414
negative thymic T cell selection	0.000414
defense response to virus	0.000414
immunoglobulin mediated immune response	0.000466
sphingolipid metabolic process	0.000484

↑Age

Overrepresented Biological Processes

GO Term	P−Value
sterol metabolic process	0.00052
cytolysis	0.000521
immune response−regulating signal transduction	0.000545
glycolipid catabolic process	0.000828
ceramide metabolic process	0.00089
immune response−activating cell surface receptor signaling pathway	0.000908
regulation of body fluid levels	0.00115
T cell homeostasis	0.0012
T cell selection	0.0012
leukocyte homeostasis	0.00128
regulation of T cell mediated immunity	0.00146
neutrophil chemotaxis	0.00146
myeloid leukocyte activation	0.00146
chemotaxis	0.00147
defense response	0.00163
regulation of immune response	0.00171
positive regulation of programmed cell death	0.00177
positive regulation of myeloid leukocyte mediated immunity	0.00198
nitric oxide mediated signal transduction	0.00199
positive regulation of isotype switching to IgA isotypes	0.00199
negative regulation of astrocyte differentiation	0.00199
antigen processing and presentation of peptide antigen via MHC class I	0.002
positive regulation of cell adhesion	0.00204
regulation of actin filament length	0.00235
cell migration	0.00246
positive regulation of tumor necrosis factor production	0.00271
type I interferon biosynthetic process	0.00271
immune system process	0.00275
regulated secretory pathway	0.00275
positive regulation of kinase activity	0.00332

↑Age

Overrepresented Biological Processes

GO Term	P−Value
regulation of cellular protein metabolic process	0.00336
regulation of protein kinase activity	0.00343
endocytosis	0.00343
glycosaminoglycan metabolic process	0.00358
positive regulation of lymphocyte proliferation	0.00359
lysosome organization and biogenesis	0.00362
positive regulation of MAP kinase activity	0.00401
T cell costimulation	0.00419
myeloid dendritic cell differentiation	0.00419
positive thymic T cell selection	0.00419
positive regulation of alpha−beta T cell proliferation	0.00419
negative regulation of mononuclear cell proliferation	0.0044
B cell proliferation	0.00459
regulation of mononuclear cell proliferation	0.00461
regulation of T cell differentiation	0.00479
negative regulation of cell activation	0.00479
negative regulation of lymphocyte activation	0.00479
cellular lipid metabolic process	0.00506
actin polymerization and/or depolymerization	0.0051
regulation of transferase activity	0.00546
cytokine metabolic process	0.00549
I−kappaB kinase/NF−kappaB cascade	0.0055
acute inflammatory response	0.00552
immune system development	0.00572
response to bacterium	0.00573
positive regulation of immunoglobulin mediated immune response	0.00573
negative regulation of protein polymerization	0.00577
antigen processing and presentation of exogenous peptide antigen via MHC class I	0.00577
response to external stimulus	0.00579
positive regulation of type III hypersensitivity	0.0058

↑Age

Overrepresented Biological Processes

GO Term	P−Value
negative regulation of endothelial cell proliferation	0.0058
ganglioside catabolic process	0.0058
detoxification of copper ion	0.0058
regulation of mast cell activation	0.0058
sequestering of actin monomers	0.0058
positive regulation of mast cell degranulation	0.0058
regulatory T cell differentiation	0.0058
positive regulation of gamma−delta T cell differentiation	0.0058
regulation of gamma−delta T cell activation	0.0058
regulation of actin filament polymerization	0.00588
induction of apoptosis	0.0061
positive regulation of MAPKKK cascade	0.00622
regulation of alpha−beta T cell activation	0.00622
negative regulation of immune system process	0.00716
regulation of cell migration	0.00735
regulation of molecular function	0.00773
antigen processing and presentation	0.00781
mononuclear cell proliferation	0.00795
regulation of cytoskeleton organization and biogenesis	0.00827
tumor necrosis factor production	0.00828
complement activation, alternative pathway	0.0084
activation of JNK activity	0.0084
positive regulation of DNA recombination	0.0084
regulation of tissue remodeling	0.0085
positive regulation of cell proliferation	0.00863
negative regulation of T cell proliferation	0.00887
N−acetylglucosamine metabolic process	0.00892
oxygen and reactive oxygen species metabolic process	0.00984
positive regulation of hydrolase activity	0.00984
blood coagulation	0.00998

↑Age

Overrepresented Biological Processes

GO Term	P−Value
lymphocyte activation	0.0101
maintenance of location	0.0106
induction of apoptosis by extracellular signals	0.0108
response to lipopolysaccharide	0.0108
response to molecule of bacterial origin	0.0111
ganglioside metabolic process	0.0112
sequestering of lipid	0.0112
sphingolipid catabolic process	0.0112
positive regulation of calcium−mediated signaling	0.0112
serotonin secretion	0.0112
regulation of cytokine and chemokine mediated signaling pathway	0.0112
inositol biosynthetic process	0.0112
membrane budding	0.0112
positive regulation of interleukin−6 production	0.0112
positive regulation of tumor necrosis factor biosynthetic process	0.0112
regulation of leukocyte degranulation	0.0112
release of sequestered calcium ion into cytosol	0.0112
sequestering of metal ion	0.0112
regulation of sequestering of calcium ion	0.0112
integrin−mediated signaling pathway	0.0115
cellular ion homeostasis	0.0127
caspase activation	0.0128
peptidyl−tyrosine phosphorylation	0.0142
response to fungus	0.0144
platelet activation	0.0144
B cell receptor signaling pathway	0.0144
regulation of antigen receptor−mediated signaling pathway	0.0144
protein complex assembly	0.015
positive regulation of protein metabolic process	0.0156
regulation of cytokine production	0.0164

↑Age

Overrepresented Biological Processes

GO Term	P−Value
hemopoiesis	0.0167
steroid biosynthetic process	0.017
phosphate transport	0.017
humoral immune response	0.0173
cell adhesion	0.0178
positive regulation of inflammatory response	0.0179
response to metal ion	0.0181
regulation of isotype switching	0.0181
negative regulation of bone remodeling	0.0181
tolerance induction	0.0182
hyaluronan metabolic process	0.0182
regulation of interleukin−1 production	0.0182
signal transduction	0.0186
negative regulation of cytoskeleton organization and biogenesis	0.0187
response to other organism	0.0189
di−, tri−valent inorganic cation transport	0.0195
positive regulation of lymphocyte differentiation	0.0203
amino sugar metabolic process	0.0205
phosphorylation	0.0205
actin filament−based process	0.0207
T cell proliferation	0.0214
T cell differentiation in the thymus	0.0233
somatic recombination of immunoglobulin genes during immune response	0.0236
negative regulation of cell adhesion	0.0236
regulation of phosphate metabolic process	0.0243
cell−matrix adhesion	0.0253
negative regulation of multicellular organismal process	0.0253
negative regulation of protein kinase activity	0.0259
positive regulation of B cell activation	0.0261
regulation of B cell differentiation	0.0263

↑Age

Overrepresented Biological Processes

GO Term	P−Value
positive regulation of T cell mediated cytotoxicity	0.0265
regulation of response to biotic stimulus	0.0265
cellular zinc ion homeostasis	0.0265
negative regulation of neuroblast proliferation	0.0265
peptide cross−linking	0.0265
regulation of Rac protein signal transduction	0.0265
negative regulation of protein import into nucleus	0.0265
positive regulation of ion transport	0.0265
cellular extravasation	0.0265
regulation of bone resorption	0.0265
positive regulation of exocytosis	0.0265
cytokine production	0.027
isoprenoid biosynthetic process	0.027
positive regulation of alpha−beta T cell differentiation	0.027
activated T cell proliferation	0.027
regulation of protein amino acid phosphorylation	0.0273
regulation of cell proliferation	0.028
cellular metal ion homeostasis	0.0293
regulation of apoptosis	0.0304
chemical homeostasis	0.0305
leukocyte mediated cytotoxicity	0.0305
negative regulation of transferase activity	0.0316
phospholipid transport	0.0321
actin filament−based movement	0.0321
regulation of JNK activity	0.0321
lipid biosynthetic process	0.0329
regulation of T cell proliferation	0.0335
positive regulation of catalytic activity	0.0348
humoral immune response mediated by circulating immunoglobulin	0.0351
leukocyte chemotaxis	0.0356

↑ Age

Overrepresented Biological Processes

GO Term	P−Value
mast cell mediated immunity	0.036
negative regulation of intracellular transport	0.036
interleukin−10 production	0.036
positive regulation of B cell differentiation	0.036
regulation of mitochondrial membrane potential	0.036
phosphorus metabolic process	0.0363
di−, tri−valent inorganic cation homeostasis	0.0364
secretion	0.0377
sphingolipid biosynthetic process	0.0378
positive regulation of B cell proliferation	0.0378
multicellular organismal protein catabolic process	0.0378
multicellular organismal macromolecule metabolic process	0.0378
lymph node development	0.0378
regulation of protein kinase cascade	0.0386
lymphocyte differentiation	0.0407
myelination	0.043
heterophilic cell adhesion	0.0438
regulation of intracellular protein transport	0.0438
cytosolic calcium ion homeostasis	0.0438
production of molecular mediator of immune response	0.0452
glycolipid metabolic process	0.0461
negative regulation of gliogenesis	0.0466
response to exogenous dsRNA	0.0466
negative regulation of MAPKKK cascade	0.0466
positive regulation of neuron apoptosis	0.0466
homeostasis of number of cells within a tissue	0.0466
regulation of cytokine secretion	0.0466
somatic diversification of immunoglobulins	0.0476
positive regulation of biological process	0.0477
multi−organism process	0.0483

↑Age

Overrepresented Biological Processes

GO Term	P−Value
stress−activated protein kinase signaling pathway	0.049
regulation of developmental process	0.0491
lung development	0.0496

↑Age

Overrepresented Cell Components

GO Term	P−Value
extracellular region	5.74e−11
external side of plasma membrane	9.35e−11
vacuole	5.89e−10
lysosome	9.93e−10
plasma membrane	1.82e−07
membrane part	3.85e−06
MHC class II protein complex	1.76e−05
integral to membrane	0.000293
immunoglobulin complex, circulating	0.00193
membrane raft	0.00217
MHC class I protein complex	0.00261
integrin complex	0.00282
immunological synapse	0.00437
B cell receptor complex	0.0109
cell fraction	0.011
anchored to membrane	0.014
uropod	0.0177
membrane fraction	0.0252
calcium− and calmodulin−dependent protein kinase complex	0.0258
clathrin coat of trans−Golgi network vesicle	0.0258
integral to plasma membrane	0.0273
Golgi membrane	0.0275
focal adhesion	0.0291
extracellular space	0.0295
proteinaceous extracellular matrix	0.0308
nuclear lamina	0.0454
multivesicular body	0.0454

↑Age

Overrepresented Molecular Functions

GO Term	P−Value
enzyme inhibitor activity	2.87e−07
serine−type endopeptidase inhibitor activity	5.3e−07
antigen binding	2.74e−06
receptor activity	1.83e−05
lipid transporter activity	5.05e−05
molecular transducer activity	5.89e−05
scavenger receptor activity	0.000138
growth factor binding	0.000203
pancreatic ribonuclease activity	0.000867
cytokine binding	0.00154
sugar binding	0.00171
intracellular calcium activated chloride channel activity	0.00184
cysteine−type endopeptidase activity	0.00292
polysaccharide binding	0.00308
chemokine activity	0.0034
hematopoietin/interferon−class (D200−domain) cytokine receptor activity	0.00359
endonuclease activity, active with either ribo− or deoxyribonucleic acids and producing 3'−phosphomonoes	0.00403
Rho GDP−dissociation inhibitor activity	0.00535
protein serine/threonine/tyrosine kinase activity	0.00543
hydrolase activity, hydrolyzing O−glycosyl compounds	0.00588
lipopolysaccharide binding	0.0104
aldehyde dehydrogenase [NAD(P)+] activity	0.0104
immunoglobulin receptor activity	0.0104
peptide antigen binding	0.0104
lipid binding	0.0122
endopeptidase inhibitor activity	0.013
protein complex binding	0.0132
heparin binding	0.0139
beta−N−acetylhexosaminidase activity	0.0168
receptor signaling protein tyrosine kinase activity	0.0168

↑Age

Overrepresented Molecular Functions

GO Term	P−Value
apoptotic protease activator activity	0.0168
IgE binding	0.0168
IgG binding	0.0168
UDP−glycosyltransferase activity	0.0181
non−membrane spanning protein tyrosine kinase activity	0.0222
G−protein−coupled receptor binding	0.0231
protein kinase C activity	0.0243
ceramidase activity	0.0245
ATP−gated cation channel activity	0.0245
galactosyltransferase activity	0.0303
oxidoreductase activity, oxidizing metal ions	0.0334
interleukin receptor activity	0.0394
enzyme activator activity	0.0395
protein binding	0.0413
endoribonuclease activity	0.0421
receptor signaling protein serine/threonine kinase activity	0.0424
protein−glutamine gamma−glutamyltransferase activity	0.0433
intramolecular oxidoreductase activity, interconverting aldoses and ketoses	0.0433
Rac GTPase activator activity	0.0433

↓Age

Overrepresented Biological Processes

GO Term	P−Value
oxidation reduction	3.79e−14
cellular respiration	6.35e−08
generation of precursor metabolites and energy	2.96e−07
carboxylic acid metabolic process	7.64e−07
acyl−CoA metabolic process	1.5e−06
cofactor catabolic process	1.54e−06
tricarboxylic acid cycle	2.2e−06
lipid oxidation	3.03e−06
fatty acid metabolic process	3.73e−06
coenzyme metabolic process	6.43e−05
carboxylic acid catabolic process	7.08e−05
fatty acid beta−oxidation	0.00015
C21−steroid hormone biosynthetic process	0.00015
ATP synthesis coupled proton transport	0.000264
response to toxin	0.000335
peroxisome organization and biogenesis	0.00034
transmembrane ion transport	0.00034
metabolic process	0.000481
catabolic process	0.000912
regulation of mitochondrial depolarization	0.00125
xenobiotic metabolic process	0.00127
hormone metabolic process	0.00196
ATP metabolic process	0.00213
cellular lipid catabolic process	0.00298
biogenic amine catabolic process	0.00328
purine ribonucleoside triphosphate biosynthetic process	0.00359
tryptophan catabolic process	0.00407
indole derivative catabolic process	0.00407
nucleoside triphosphate biosynthetic process	0.00452
very−long−chain fatty acid metabolic process	0.00602

↓Age

Overrepresented Biological Processes

GO Term	P−Value
mitochondrial electron transport, NADH to ubiquinone	0.00602
steroid biosynthetic process	0.00622
ribonucleoside triphosphate metabolic process	0.00684
glutathione biosynthetic process	0.00831
regulation of mitochondrial membrane potential	0.00831
electron transport chain	0.00876
purine nucleoside triphosphate metabolic process	0.00902
cellular lipid metabolic process	0.00944
negative regulation of transforming growth factor beta receptor signaling pathway	0.0109
aromatic compound catabolic process	0.0137
ribonucleotide biosynthetic process	0.0157
purine nucleotide biosynthetic process	0.0169
secondary metabolic process	0.0169
regulation of cell−matrix adhesion	0.0171
nucleotide metabolic process	0.0173
mitochondrial transport	0.0194
indolalkylamine metabolic process	0.0206
indole and derivative metabolic process	0.0206
proton transport	0.0207
liver development	0.0213
purine ribonucleotide metabolic process	0.0221
nucleobase, nucleoside and nucleotide metabolic process	0.0239
endocrine pancreas development	0.0244
ATP synthesis coupled electron transport	0.0244
translational initiation	0.0255
regulation of caspase activity	0.0277
long−chain fatty acid metabolic process	0.0285
epithelial cell development	0.0285
regulation of peptidase activity	0.03
porphyrin biosynthetic process	0.0328

↓Age

Overrepresented Biological Processes

GO Term	P−Value
hydrogen peroxide metabolic process	0.0421
spermatid development	0.0431
heme metabolic process	0.047
gastrulation	0.0472

↓Age

Overrepresented Cell Components

GO Term	P−Value
mitochondrion	8.27e−35
mitochondrial envelope	1.32e−28
envelope	2.83e−23
mitochondrial inner membrane	1.96e−21
organelle membrane	4.42e−20
cytoplasm	2.76e−19
peroxisome	4.37e−11
microsome	2.79e−09
mitochondrial respiratory chain	3.7e−09
endoplasmic reticulum	1.46e−08
intracellular	3.14e−07
intracellular organelle part	1e−05
membrane−bounded organelle	1.37e−05
microbody part	2.78e−05
intracellular organelle	3.4e−05
cell fraction	0.000182
peroxisomal membrane	0.000231
glutamate−cysteine ligase complex	0.000394
proton−transporting ATP synthase complex, coupling factor F(o)	0.000843
membrane fraction	0.000895
mitochondrial ribosome	0.00398
cell	0.00474
proton−transporting two−sector ATPase complex	0.00604
mitochondrial lumen	0.013

↓Age

Overrepresented Molecular Functions

GO Term	P−Value
electron carrier activity	7.37e−12
catalytic activity	2.29e−08
steroid dehydrogenase activity	3.86e−08
iron ion binding	4.95e−07
oxidoreductase activity	5.22e−07
heme binding	7.55e−07
NADH dehydrogenase activity	2.46e−06
NADH dehydrogenase (ubiquinone) activity	2.46e−06
monooxygenase activity	3.96e−06
oxidoreductase activity, acting on NADH or NADPH, quinone or similar compound as acceptor	6.08e−06
carboxylesterase activity	6.95e−06
acetyl−CoA C−acyltransferase activity	9.18e−06
dodecenoyl−CoA delta−isomerase activity	9.18e−06
3−beta−hydroxy−delta5−steroid dehydrogenase activity	2.21e−05
steroid delta−isomerase activity	3.61e−05
3−hydroxyacyl−CoA dehydrogenase activity	8.89e−05
intramolecular oxidoreductase activity	9.33e−05
metal cluster binding	0.000333
oxidoreductase activity, acting on the CH−CH group of donors	0.000369
aconitate hydratase activity	0.00044
epoxide hydrolase activity	0.00044
succinate dehydrogenase (ubiquinone) activity	0.00044
estradiol 17−beta−dehydrogenase activity	0.000475
unspecific monooxygenase activity	0.000499
4 iron, 4 sulfur cluster binding	0.000576
pyridoxal phosphate binding	0.000595
oxidoreductase activity, acting on the CH−OH group of donors, NAD or NADP as acceptor	0.000675
aldo−keto reductase activity	0.000987
palmitoyl−CoA hydrolase activity	0.000987
oxidoreductase activity, acting on paired donors, with incorporation or reduction of molecular oxygen	0.00108

↓Age

Overrepresented Molecular Functions

GO Term	P−Value
acetyl−CoA C−acetyltransferase activity	0.0013
monovalent inorganic cation transmembrane transporter activity	0.00155
CoA hydrolase activity	0.00278
acyltransferase activity	0.00421
sterol carrier activity	0.00422
cytochrome−c oxidase activity	0.00501
glucuronosyltransferase activity	0.00501
oxidoreductase activity, acting on heme group of donors	0.00501
2 iron, 2 sulfur cluster binding	0.00501
hydrolase activity, acting on ether bonds	0.00624
acetyltransferase activity	0.00784
transferase activity, transferring acyl groups	0.00786
coenzyme binding	0.00796
pheromone binding	0.00862
lyase activity	0.00869
molybdenum ion binding	0.0113
steroid binding	0.0134
NAD binding	0.015
ATPase activity	0.017
non−G−protein coupled 7TM receptor activity	0.0213
fatty acid binding	0.0213
FMN binding	0.0213
acid−thiol ligase activity	0.0251
long−chain−fatty−acid−CoA ligase activity	0.0253
selenium binding	0.0267
hydrogen ion transporting ATP synthase activity, rotational mechanism	0.029
hydrogen ion transporting ATPase activity, rotational mechanism	0.029
N−acyltransferase activity	0.0292
nuclear hormone receptor binding	0.0295
hydrolase activity, acting on acid anhydrides, in phosphorus−containing anhydrides	0.0309

↓Age

Overrepresented Molecular Functions

GO Term	P−Value
hydro−lyase activity	0.0469
ATPase activity, coupled to transmembrane movement of substances	0.0497

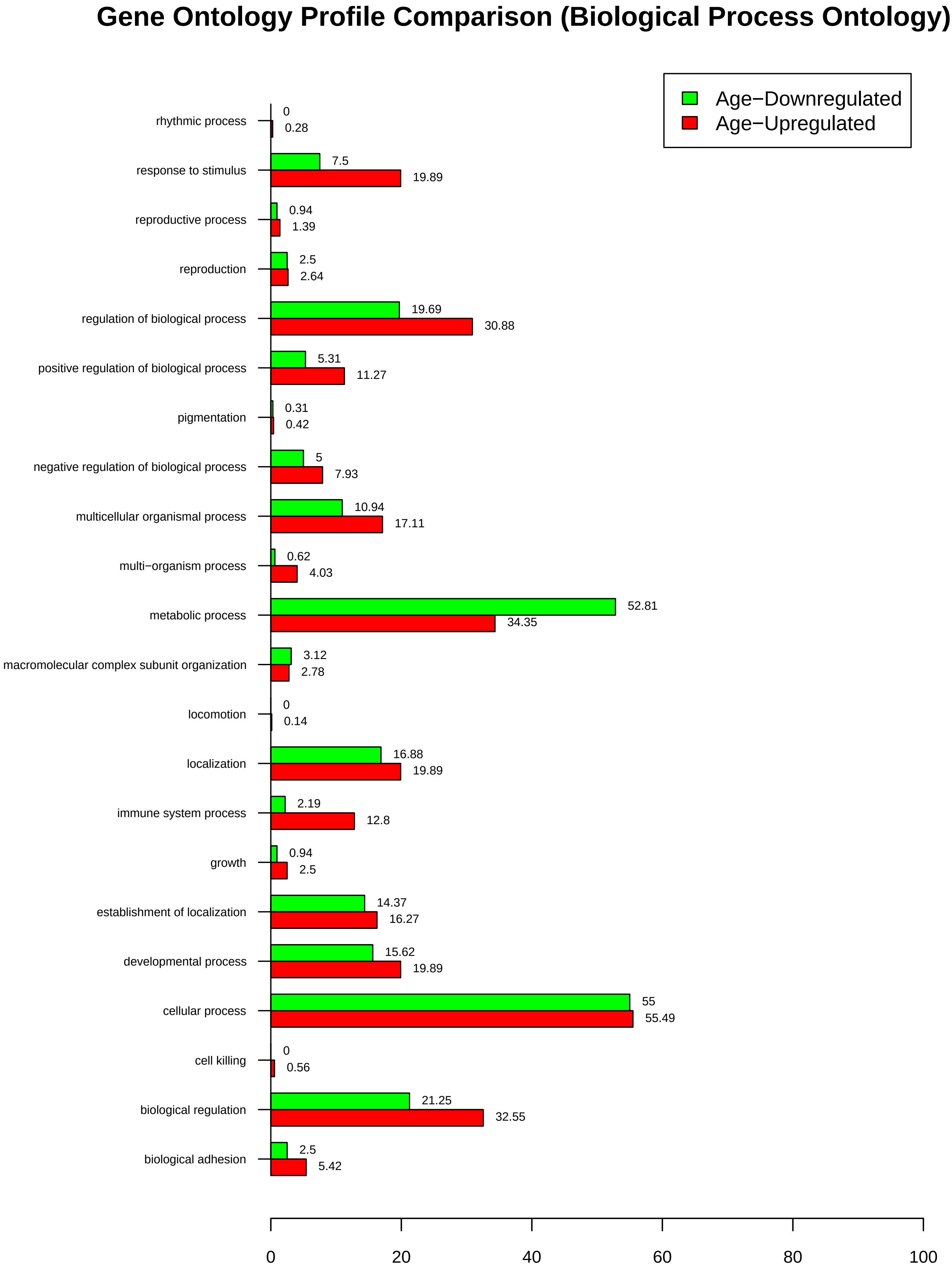

Gene Ontology Profile Comparison (Biological Process Ontology)
Age−Downregulated
Age−Upregulated
rhythmic process
0
0.28
response to stimulus
7.5
19.89
reproductive process
0.94
1.39
reproduction
2.5
2.64
regulation of biological process
19.69
30.88
positive regulation of biological process
5.31
11.27
pigmentation
0.31
0.42
negative regulation of biological process
5
7.93
multicellular organismal process
10.94
17.11
multi−organism process
0.62
4.03
metabolic process
52.81
34.35
macromolecular complex subunit organization
3.12
2.78
locomotion
0
0.14
localization
16.88
19.89
immune system process
2.19
12.8
growth
0.94
2.5
establishment of localization
14.37
16.27
developmental process
15.62
19.89
cellular process
55
55.49
cell killing
0
0.56
biological regulation
21.25
32.55
biological adhesion
2.5
5.42
0
20
40
60
80
100

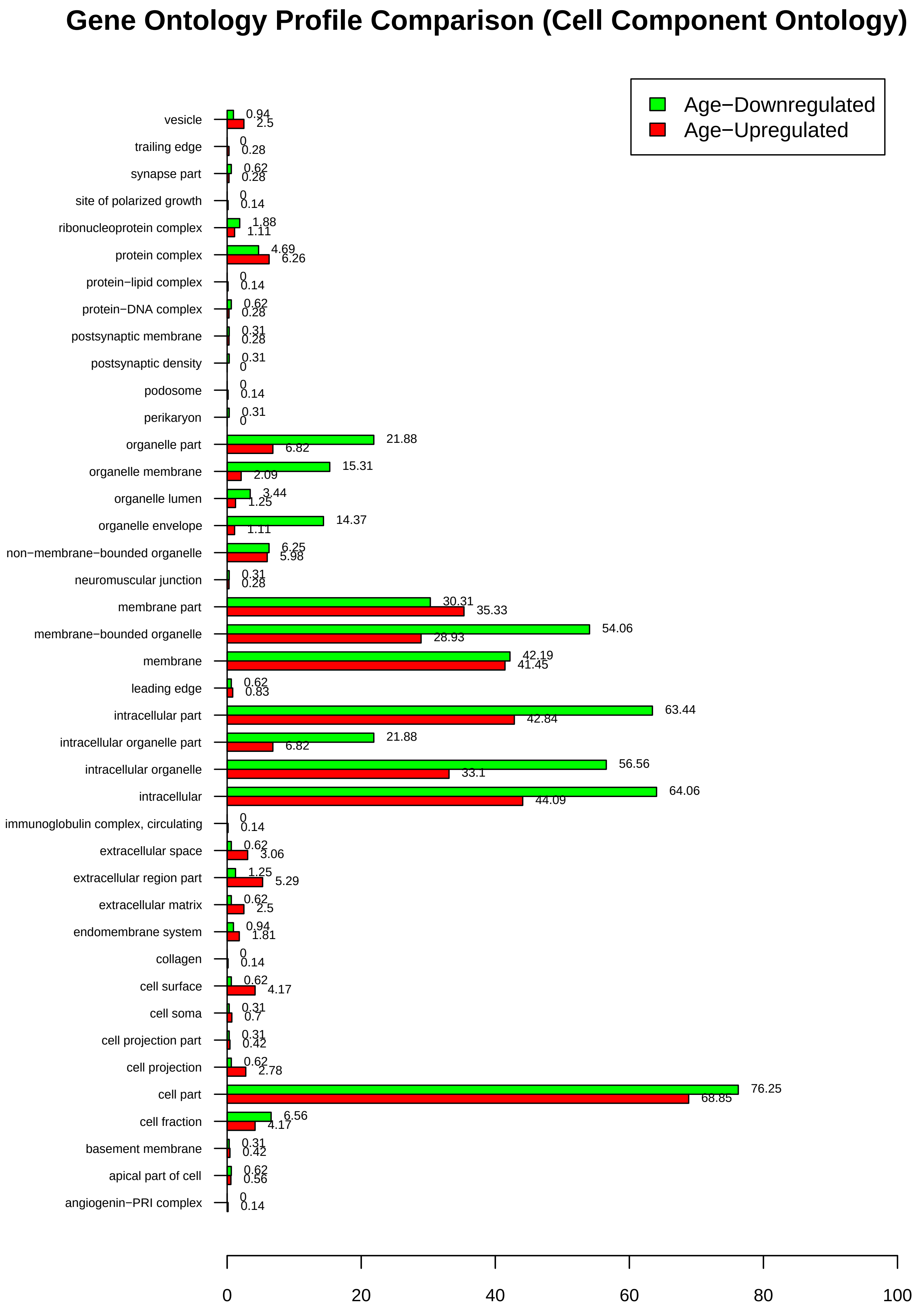

Gene Ontology Profile Comparison (Cell Component Ontology)
Age−Downregulated
Age−Upregulated
vesicle
0.94
2.5
trailing edge
0
0.28
synapse part
0.62
0.28
site of polarized growth
0
0.14
ribonucleoprotein complex
1.88
1.11
protein complex
4.69
6.26
protein−lipid complex
0
0.14
protein−DNA complex
0.62
0.28
postsynaptic membrane
0.31
0.28
postsynaptic density
0.31
0
podosome
0
0.14
perikaryon
0.31
0
organelle part
21.88
6.82
organelle membrane
15.31
2.09
organelle lumen
3.44
1.25
organelle envelope
14.37
1.11
non−membrane−bounded organelle
6.25
5.98
neuromuscular junction
0.31
0.28
membrane part
30.31
35.33
membrane−bounded organelle
54.06
28.93
membrane
42.19
41.45
leading edge
0.62
0.83
intracellular part
63.44
42.84
intracellular organelle part
21.88
6.82
intracellular organelle
56.56
33.1
intracellular
64.06
44.09
immunoglobulin complex, circulating
0
0.14
extracellular space
0.62
3.06
extracellular region part
1.25
5.29
extracellular matrix
0.62
2.5
endomembrane system
0.94
1.81
collagen
0
0.14
cell surface
0.62
4.17
cell soma
0.31
0.7
cell projection part
0.31
0.42
cell projection
0.62
2.78
cell part
76.25
68.85
cell fraction
6.56
4.17
basement membrane
0.31
0.42
apical part of cell
0.62
0.56
angiogenin−PRI complex
0
0.14
0
20
40
60
80
100

Gene Ontology Profile Comparison (Molecular Function Ontology)

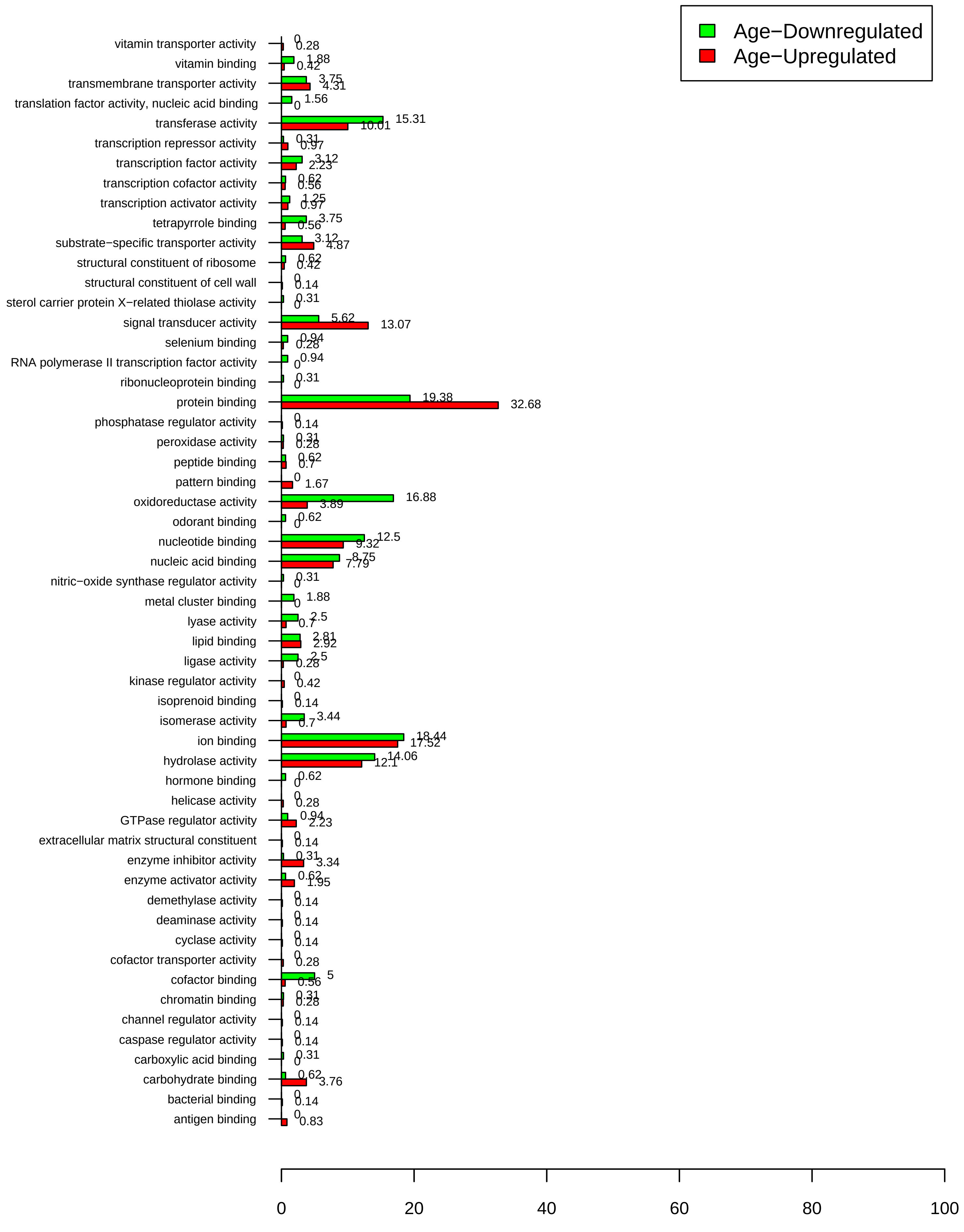

↑Age

Overrepresented KEGG Pathways

GO Term	P−Value
Leukocyte transendothelial migration	3.76e−05
Biosynthesis of steroids	4.51e−05
Antigen processing and presentation	0.00011
Natural killer cell mediated cytotoxicity	0.000201
Cell adhesion molecules (CAMs)	0.000459
Complement and coagulation cascades	0.00086
Sphingolipid metabolism	0.000914
Cytokine−cytokine receptor interaction	0.00143
Terpenoid biosynthesis	0.0022
Hematopoietic cell lineage	0.00327
Type I diabetes mellitus	0.00782
Glycosaminoglycan degradation	0.00908
Aminosugars metabolism	0.00938
Regulation of actin cytoskeleton	0.0129
Glycosphingolipid biosynthesis − ganglioseries	0.015
Glycan structures − degradation	0.0174
Alzheimer's disease	0.0239
B cell receptor signaling pathway	0.0443
Fc epsilon RI signaling pathway	0.0458

↓Age

Overrepresented KEGG Pathways

GO Term	P−Value
Fatty acid metabolism	2.87e−12
Oxidative phosphorylation	1.55e−10
PPAR signaling pathway	5.39e−09
Tryptophan metabolism	9.26e−08
Valine, leucine and isoleucine degradation	1.29e−07
Androgen and estrogen metabolism	1.2e−06
Butanoate metabolism	4.18e−06
Lysine degradation	2.4e−05
Bile acid biosynthesis	2.98e−05
Citrate cycle (TCA cycle)	6.87e−05
Fatty acid elongation in mitochondria	0.000116
Geraniol degradation	0.000427
Glyoxylate and dicarboxylate metabolism	0.000507
Metabolism of xenobiotics by cytochrome P450	0.00142
Caprolactam degradation	0.00414
C21−Steroid hormone metabolism	0.00527
Porphyrin and chlorophyll metabolism	0.00865
Propanoate metabolism	0.00976
Biosynthesis of unsaturated fatty acids	0.0115
Alkaloid biosynthesis II	0.0158
Caffeine metabolism	0.0203
Synthesis and degradation of ketone bodies	0.0256
Tetrachloroethene degradation	0.0256
Linoleic acid metabolism	0.0331
Reductive carboxylate cycle (CO2 fixation)	0.0376
Arachidonic acid metabolism	0.0436

↑Age

Overrepresented KEGG Pathways

(Based on InterPro Domain Signatures)

GO Term	P−Value
Hematopoietic cell lineage	1e−04
Leukocyte transendothelial migration	1e−04
Biosynthesis of steroids	3e−04
Terpenoid biosynthesis	0.0016
Complement and coagulation cascades	0.0031
Sphingolipid metabolism	0.0058
Natural killer cell mediated cytotoxicity	0.0084
ECM−receptor interaction	0.0094
Glycan structures − biosynthesis 1	0.0115
Glycosphingolipid biosynthesis − lactoseries	0.0138
Glycosphingolipid biosynthesis − globoseries	0.0144
O−Glycan biosynthesis	0.0162
Huntington's disease	0.019
N−Glycan degradation	0.022
Aminosugars metabolism	0.0244
Alzheimer's disease	0.0249
Glycan structures − degradation	0.027
Fc epsilon RI signaling pathway	0.0287
Focal adhesion	0.0299
Glycosphingolipid biosynthesis − ganglioseries	0.0337
B cell receptor signaling pathway	0.0345
Glutamate metabolism	0.0349
Cell Communication	0.0466
Jak−STAT signaling pathway	0.0483
Primary immunodeficiency	0.0483

↓Age

Overrepresented KEGG Pathways

(Based on InterPro Domain Signatures)

GO Term	P−Value
Fatty acid metabolism	1e−04
Androgen and estrogen metabolism	1e−04
Tryptophan metabolism	1e−04
Tetrachloroethene degradation	1e−04
Butanoate metabolism	1e−04
Metabolism of xenobiotics by cytochrome P450	1e−04
Drug metabolism − cytochrome P450	1e−04
PPAR signaling pathway	1e−04
Retinol metabolism	2e−04
C21−Steroid hormone metabolism	3e−04
Oxidative phosphorylation	3e−04
Histidine metabolism	5e−04
Lysine degradation	7e−04
Fatty acid elongation in mitochondria	8e−04
Bile acid biosynthesis	8e−04
Glyoxylate and dicarboxylate metabolism	0.0011
Caprolactam degradation	0.0012
Aminophosphonate metabolism	0.0029
Alkaloid biosynthesis II	0.0033
Valine, leucine and isoleucine degradation	0.0045
Synthesis and degradation of ketone bodies	0.0046
Ascorbate and aldarate metabolism	0.0049
Selenoamino acid metabolism	0.0052
Linoleic acid metabolism	0.0053
Bisphenol A degradation	0.006
Arachidonic acid metabolism	0.0066
Benzoate degradation via CoA ligation	0.0068
Limonene and pinene degradation	0.0079
Phenylalanine metabolism	0.0106
Tyrosine metabolism	0.0109

↓Age

Overrepresented KEGG Pathways

(Based on InterPro Domain Signatures)

GO Term	P−Value
Reductive carboxylate cycle (CO2 fixation)	0.0117
Taurine and hypotaurine metabolism	0.0155
Citrate cycle (TCA cycle)	0.016
Porphyrin and chlorophyll metabolism	0.0274
Inositol metabolism	0.0289
Caffeine metabolism	0.0295
beta−Alanine metabolism	0.0302
Pentose and glucuronate interconversions	0.0317
Biosynthesis of unsaturated fatty acids	0.0328
Geraniol degradation	0.0338
Glycine, serine and threonine metabolism	0.0346
Drug metabolism − other enzymes	0.0358
Carbon fixation	0.0359

↑Age

Abundance of miRNA Targets

miRNA	Freq(Obs)	Freq(Exp)	Obs/Exp	P−value	P−Value(Adj)
miR−133a	0.0849	0.0702	1.21	0.0822	1
miR−133b	0.0849	0.0702	1.21	0.0822	1
miR−701	0.0232	0.0167	1.39	0.0969	1
miR−696	0.0444	0.0358	1.24	0.122	1
miR−409−5p	0.0541	0.0449	1.2	0.133	1
miR−128	0.158	0.143	1.11	0.136	1
miR−293	0.0328	0.0266	1.23	0.154	1
miR−153	0.11	0.098	1.12	0.157	1
miR−671−5p	0.143	0.129	1.1	0.161	1
miR−325	0.141	0.128	1.1	0.17	1

↓Age

Abundance of miRNA Targets

miRNA	Freq(Obs)	Freq(Exp)	Obs/Exp	P−value	P−Value(Adj)
miR−689	0.05	0.0304	1.65	0.0371	1
miR−466f−5p	0.0955	0.0719	1.33	0.0733	1
miR−210	0.0591	0.0432	1.37	0.0963	1
miR−487b	0.0273	0.0194	1.41	0.138	1
miR−879	0.114	0.0944	1.2	0.138	1
miR−490	0.1	0.0822	1.22	0.139	1
miR−574−3p	0.0545	0.0423	1.29	0.142	1
miR−878−3p	0.109	0.093	1.17	0.172	1
miR−1187	0.186	0.166	1.12	0.182	1
miR−434−5p	0.0364	0.0289	1.26	0.187	1

Tests for Chromosome Over−representation

Chromosome	Age−upregulated Genes	Age−downregulated Genes
1	0.615	0.184
2	0.909	0.222
3	0.534	0.365
4	0.975	0.163
5	0.552	0.706
6	0.693	0.224
7	0.0636	0.936
8	0.675	0.0543
9	0.425	0.00416*
10	0.239	0.951
11	0.953	0.973
12	0.586	0.603
13	0.529	0.663
14	0.103	0.165
15	0.228	0.363
16	0.352	0.729
17	0.057	0.71
18	0.944	0.57
19	0.274	0.945
X	0.2	0.927
Y	0.162	1.00

The table lists p−values generated from a test that evaluates whether there exists an over−abundance of identified genes with respect to a given chromosome. The null hypothesis assumes that the set of genes has been selected at random from those represented on the Affymetrix 430 2.0 array. A significant test indicates that a chromosome contains more of the identified genes than would be expected if the gene set had been chosen at random.

* = significant p−value, without multiple test adjustment

** = significant p−value, with multiple test adjustment

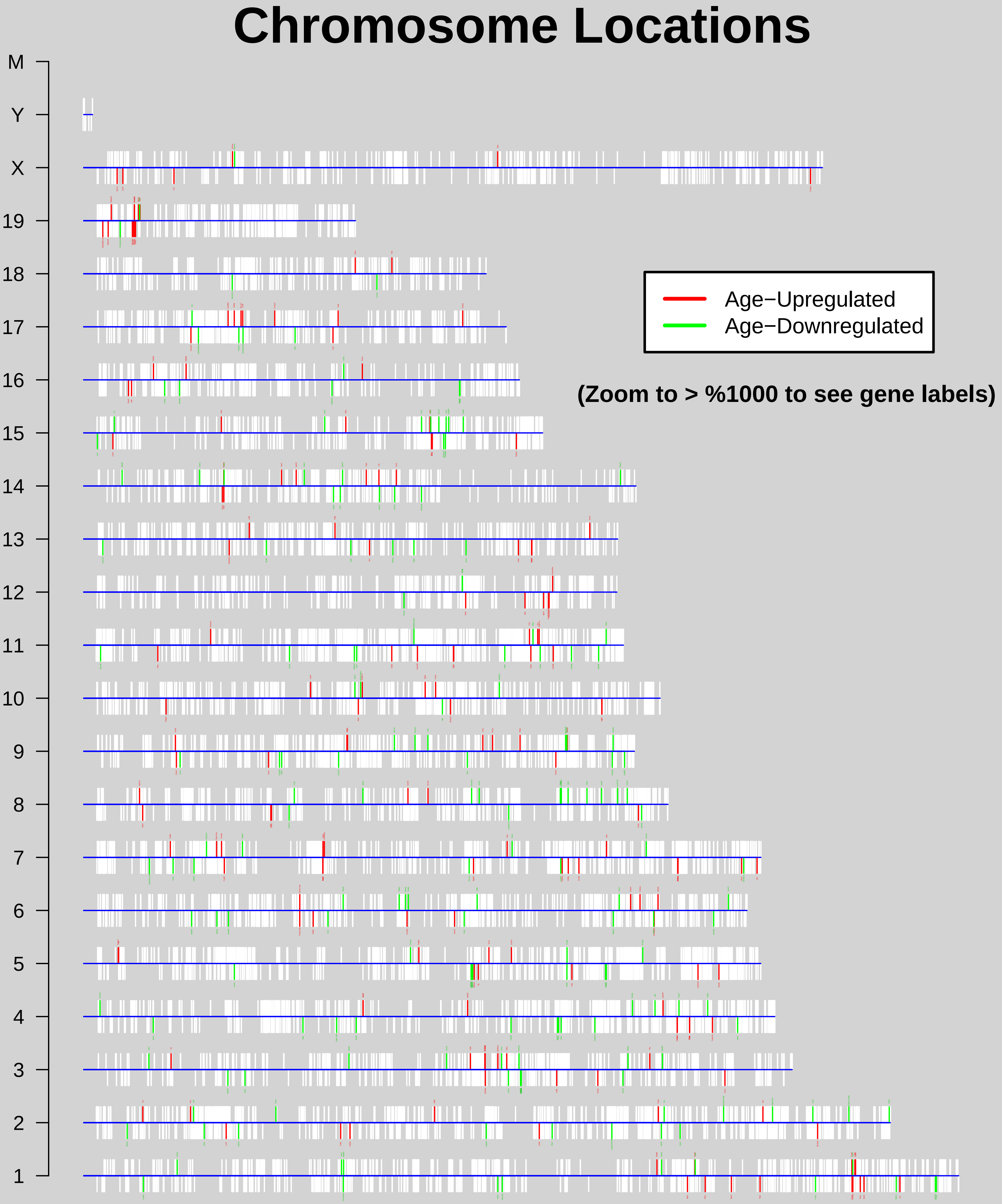

Chromosome Locations
M
Y
X
19
18
17
16
15
14
13
12
11
10
9
8
7
6
5
4
3
2
1
Age−Upregulated
Age−Downregulated
(Zoom to > %1000 to see gene labels)
