# Supplementary material for: Genes and gene expression modules associated with caloric restriction and aging in the laboratory mouse
Source: BMC Genomics. 2009 Dec 7;10:585. doi: 10.1186/1471-2164-10-585 (PMC2795771; doi:10.1186/1471-2164-10-585)
Supplement: Additional file 8 — Genes regulated by aging in heart. Results from 5 experiments are analyzed to identify genes significantly up and down regulated by aging in heart. This file also includes analysis of associated gene ontology terms, KEGG pathways, microRNA targets and chromosomal locations of age-regulated genes. [file 1471-2164-10-585-S8.PDF]

Additional File 8

Genes and Gene Expression Modules Associated with Caloric Restriction and Aging in the Laboratory Mouse

William R. Swindell
University of Michigan, Departments of Pathology and Geriatrics

---

Genes Regulated by Aging in Heart

This file provides information on genes significantly influenced by aging in the heart. The first set of charts displays differential expression results of the 200 genes most strongly up regulated by aging in heart, while the second set of charts displays differential expression results for the 200 genes most strongly down regulated by aging in heart. Each row corresponds to an individual gene and each column corresponds to a separate experiment (see Additional File 1). Symbols are interpreted as follows:

● Gene is significantly up regulated by age ($P_u < 0.05$)
● Gene is significantly down regulated by age ($P_d < 0.05$)
— Gene is marginally up regulated by age ($0.05 < P_u < 0.10$)
— Gene is marginally down regulated by age ($0.05 < P_d < 0.10$)
— Non-significant age effect ($P_u > 0.10$ and $P_d > 0.10$)
× No data (gene not represented in experiment or array annotation was limiting)
✳ Evidence conflicts but favors up regulation by age
✳ Evidence conflicts, but favors down regulation by age

The last two categories (✳ and ✳) indicate significant effects with conflicting evidence. This can arise if multiple transcripts associated with the same gene symbol yield opposite conclusions. Alternatively, a conflict may arise if $P_u < 0.05$ and also $P_d < 0.05$. Symbols shown in charts are based upon a comparison-wise type I error rate of 0.05. The final column in each chart lists meta-analysis p-values generated using Fisher's method, which have been adjusted using the Benjamini-Hochberg method to control the false discovery rate among all 21,327 genes.

The remainder of the file includes lists of over-represented gene ontology terms, over-represented KEGG pathways, and over-represented KEGG pathways defined based upon IP domain signatures (see Hahne et al. 2008, BMC Bioinformatics 9:3). Genes were also analyzed to determine if there existed an over-abundance of targets for certain microRNAs (see Betel et al. 2008, Nucleic Acids Res. 36: D149-153), and a list of associated microRNAs is provided based upon this analysis. Lastly, tests for over-representation of identified genes with respect to each chromosome were performed, and an idiogram mapping of identified genes to chromosomal locations is shown.

Contact: William R. Swindell, wswindel@umich.edu

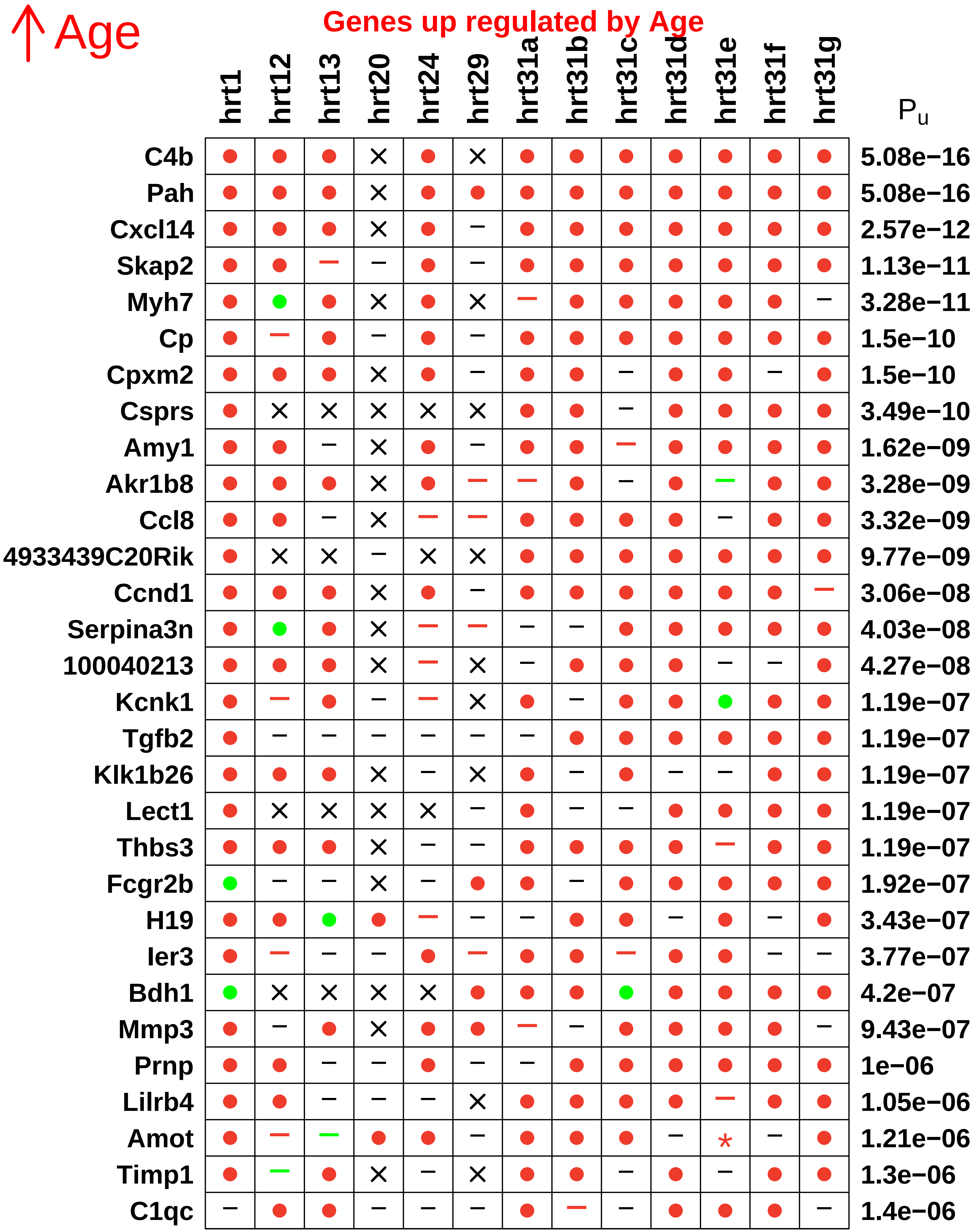

↑ Age
Genes up regulated by Age
hrt1
hrt12
hrt13
hrt20
hrt24
hrt29
hrt31a
hrt31b
hrt31c
hrt31d
hrt31e
hrt31f
hrt31g
Pu
C4b 5.08e−16
Pah 5.08e−16
Cxcl14 2.57e−12
Skap2 1.13e−11
Myh7 3.28e−11
Cp 1.5e−10
Cpxm2 1.5e−10
Csprs 3.49e−10
Amy1 1.62e−09
Akr1b8 3.28e−09
Ccl8 3.32e−09
4933439C20Rik 9.77e−09
Ccnd1 3.06e−08
Serpina3n 4.03e−08
100040213 4.27e−08
Kcnk1 1.19e−07
Tgfb2 1.19e−07
Klk1b26 1.19e−07
Lect1 1.19e−07
Thbs3 1.19e−07
Fcgr2b 1.92e−07
H19 3.43e−07
Ier3 3.77e−07
Bdh1 4.2e−07
Mmp3 9.43e−07
Prnp 1e−06
Lilrb4 1.05e−06
Amot 1.21e−06
Timp1 1.3e−06
C1qc 1.4e−06

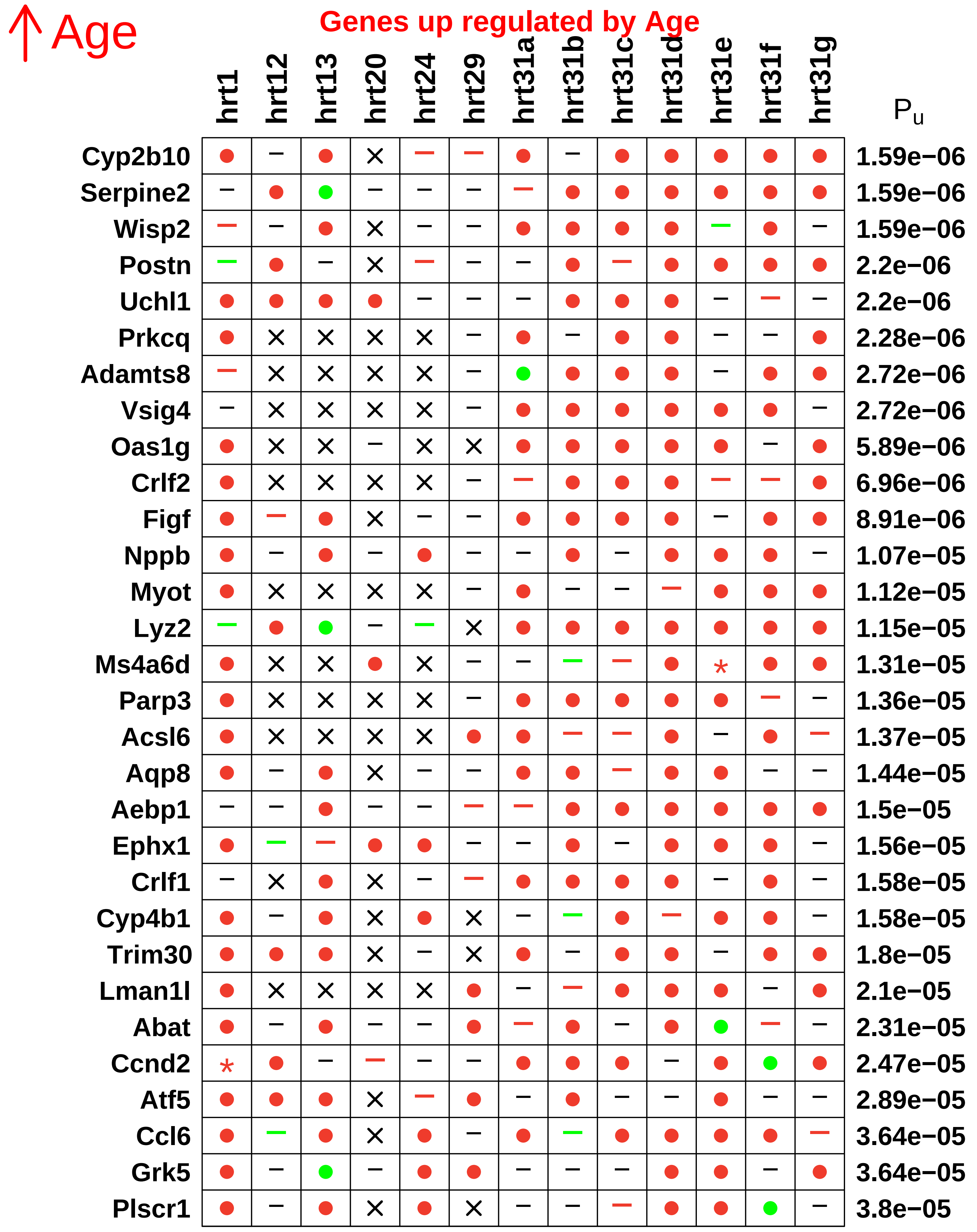
↑ Age
Genes up regulated by Age
hrt1
hrt12
hrt13
hrt20
hrt24
hrt29
hrt31a
hrt31b
hrt31c
hrt31d
hrt31e
hrt31f
hrt31g
P_u
Cyp2b10 1.59e−06
Serpine2 1.59e−06
Wisp2 1.59e−06
Postn 2.2e−06
Uchl1 2.2e−06
Prkcq 2.28e−06
Adamts8 2.72e−06
Vsig4 2.72e−06
Oas1g 5.89e−06
Crlf2 6.96e−06
Figf 8.91e−06
Nppb 1.07e−05
Myot 1.12e−05
Lyz2 1.15e−05
Ms4a6d 1.31e−05
Parp3 1.36e−05
Acsl6 1.37e−05
Aqp8 1.44e−05
Aebp1 1.5e−05
Ephx1 1.56e−05
Crlf1 1.58e−05
Cyp4b1 1.58e−05
Trim30 1.8e−05
Lman1l 2.1e−05
Abat 2.31e−05
Ccnd2 2.47e−05
Atf5 2.89e−05
Ccl6 3.64e−05
Grk5 3.64e−05
Plscr1 3.8e−05

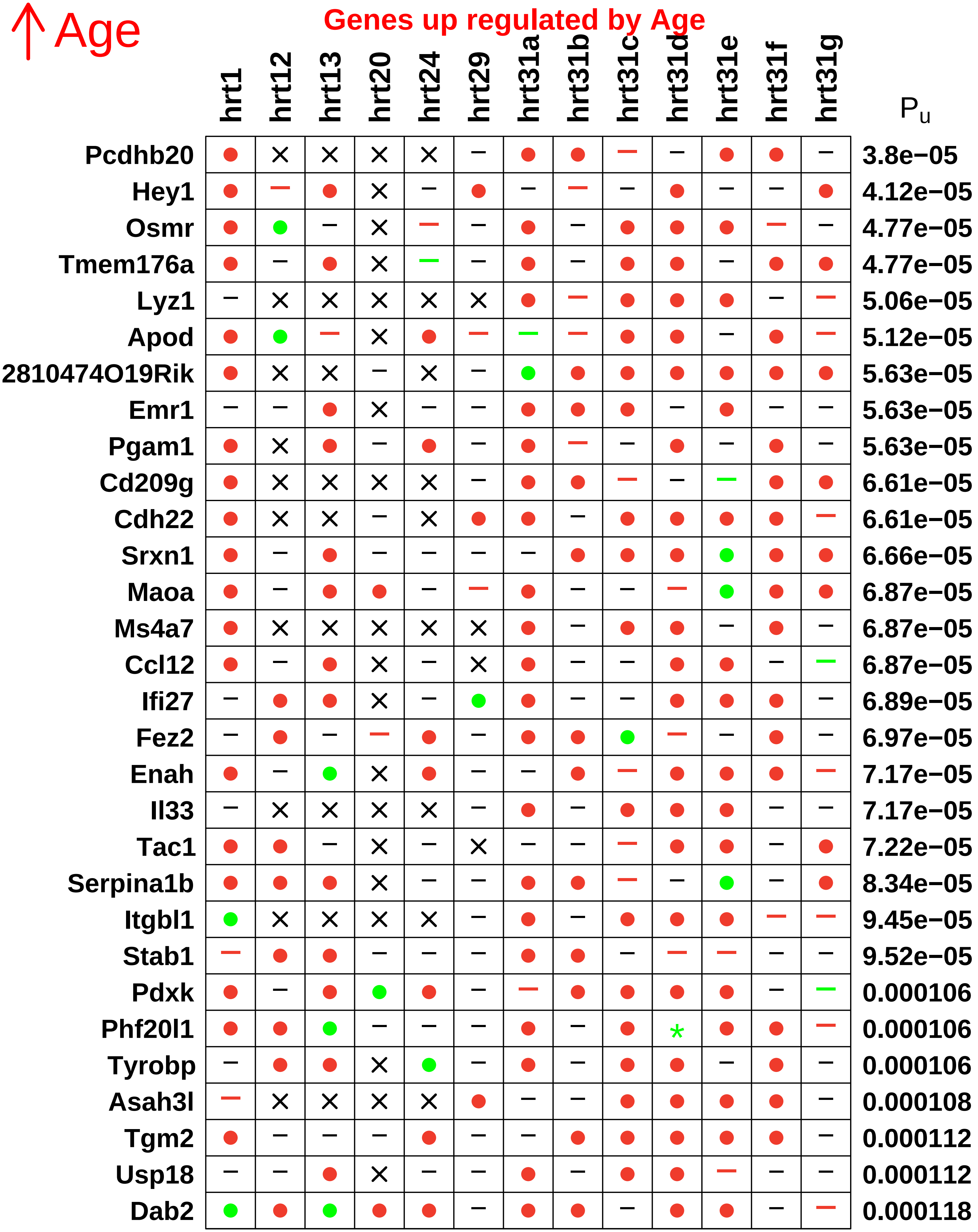
↑ Age
Genes up regulated by Age
hrt1
hrt12
hrt13
hrt20
hrt24
hrt29
hrt31a
hrt31b
hrt31c
hrt31d
hrt31e
hrt31f
hrt31g
Pu
Pcdhb20 3.8e−05
Hey1 4.12e−05
Osmr 4.77e−05
Tmem176a 4.77e−05
Lyz1 5.06e−05
Apod 5.12e−05
2810474O19Rik 5.63e−05
Emr1 5.63e−05
Pgam1 5.63e−05
Cd209g 6.61e−05
Cdh22 6.61e−05
Srxn1 6.66e−05
Maoa 6.87e−05
Ms4a7 6.87e−05
Ccl12 6.87e−05
Ifi27 6.89e−05
Fez2 6.97e−05
Enah 7.17e−05
Il33 7.17e−05
Tac1 7.22e−05
Serpina1b 8.34e−05
Itgbl1 9.45e−05
Stab1 9.52e−05
Pdxk 0.000106
Phf20l1 0.000106
Tyrobp 0.000106
Asah3l 0.000108
Tgm2 0.000112
Usp18 0.000112
Dab2 0.000118

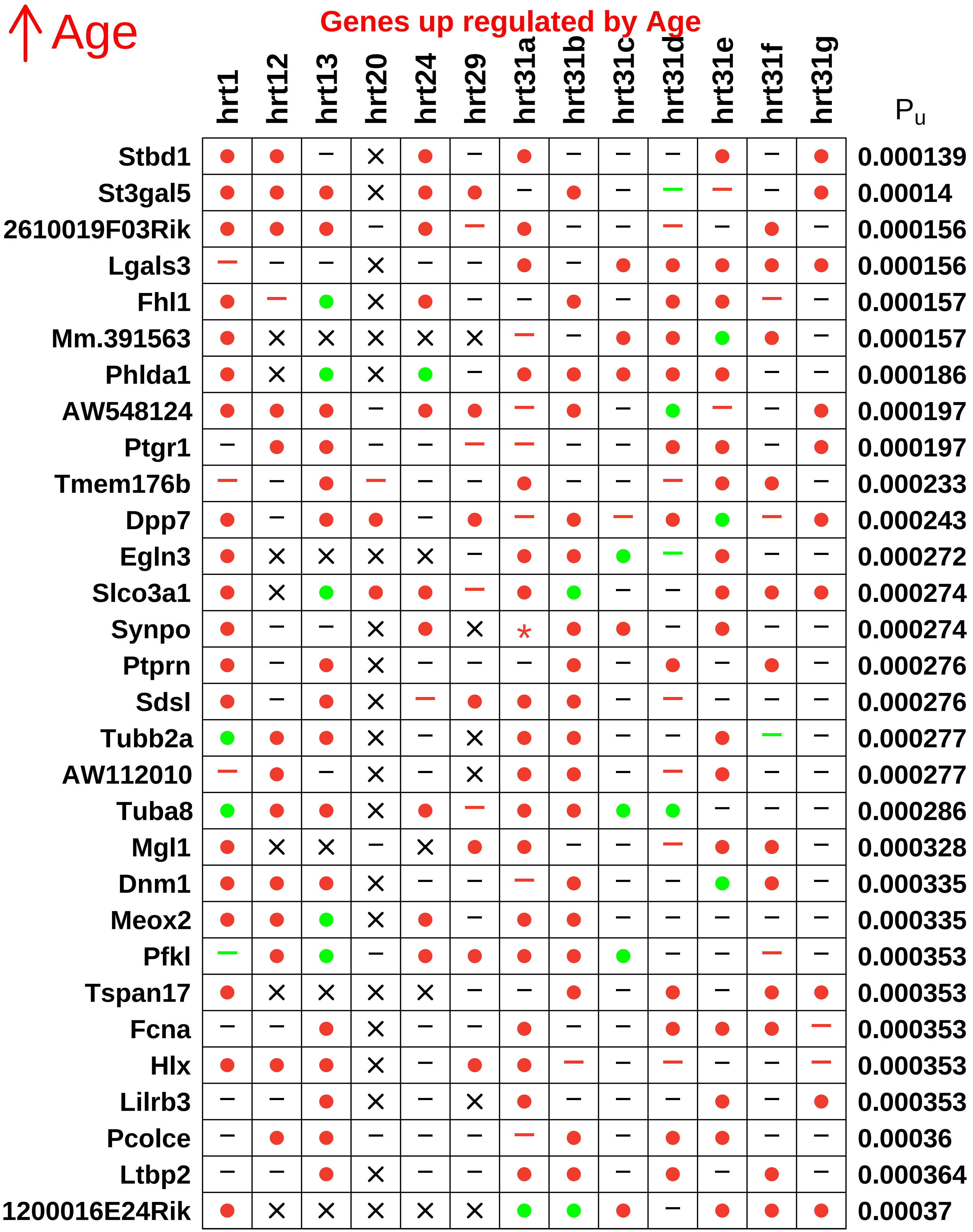

↑ Age
Genes up regulated by Age
hrt1
hrt12
hrt13
hrt20
hrt24
hrt29
hrt31a
hrt31b
hrt31c
hrt31d
hrt31e
hrt31f
hrt31g
P_u
Stbd1 0.000139
St3gal5 0.00014
2610019F03Rik 0.000156
Lgals3 0.000156
Fhl1 0.000157
Mm.391563 0.000157
Phlda1 0.000186
AW548124 0.000197
Ptgr1 0.000197
Tmem176b 0.000233
Dpp7 0.000243
Egln3 0.000272
Slco3a1 0.000274
Synpo 0.000274
Ptprn 0.000276
Sdsl 0.000276
Tubb2a 0.000277
AW112010 0.000277
Tuba8 0.000286
Mgl1 0.000328
Dnm1 0.000335
Meox2 0.000335
Pfkl 0.000353
Tspan17 0.000353
Fcna 0.000353
Hlx 0.000353
Lilrb3 0.000353
Pcolce 0.00036
Ltbp2 0.000364
1200016E24Rik 0.00037

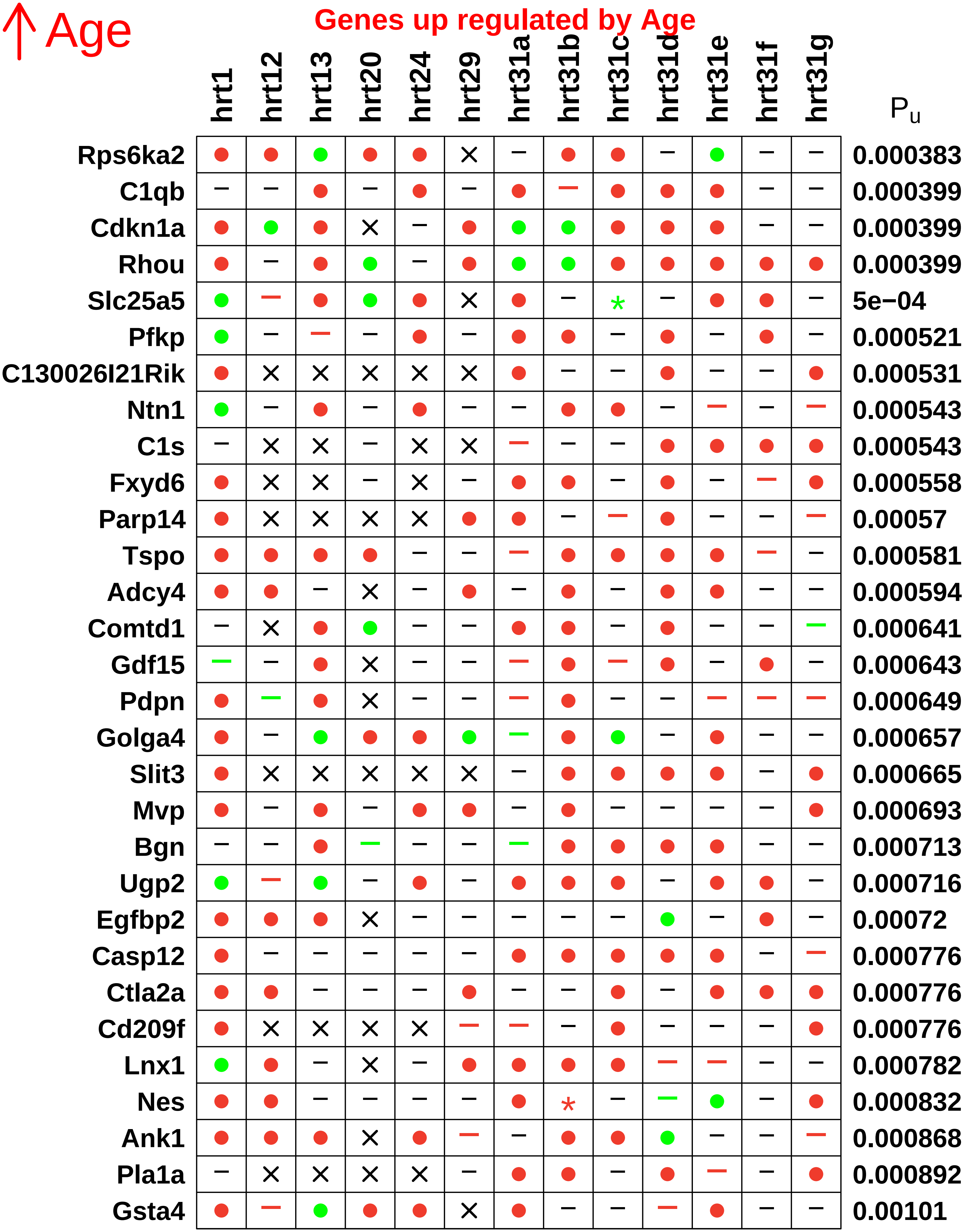
↑ Age
Genes up regulated by Age
hrt1
hrt12
hrt13
hrt20
hrt24
hrt29
hrt31a
hrt31b
hrt31c
hrt31d
hrt31e
hrt31f
hrt31g
P_u
Rps6ka2 0.000383
C1qb 0.000399
Cdkn1a 0.000399
Rhou 0.000399
Slc25a5 5e−04
Pfkp 0.000521
C130026I21Rik 0.000531
Ntn1 0.000543
C1s 0.000543
Fxyd6 0.000558
Parp14 0.00057
Tspo 0.000581
Adcy4 0.000594
Comtd1 0.000641
Gdf15 0.000643
Pdpn 0.000649
Golga4 0.000657
Slit3 0.000665
Mvp 0.000693
Bgn 0.000713
Ugp2 0.000716
Egfbp2 0.00072
Casp12 0.000776
Ctla2a 0.000776
Cd209f 0.000776
Lnx1 0.000782
Nes 0.000832
Ank1 0.000868
Pla1a 0.000892
Gsta4 0.00101

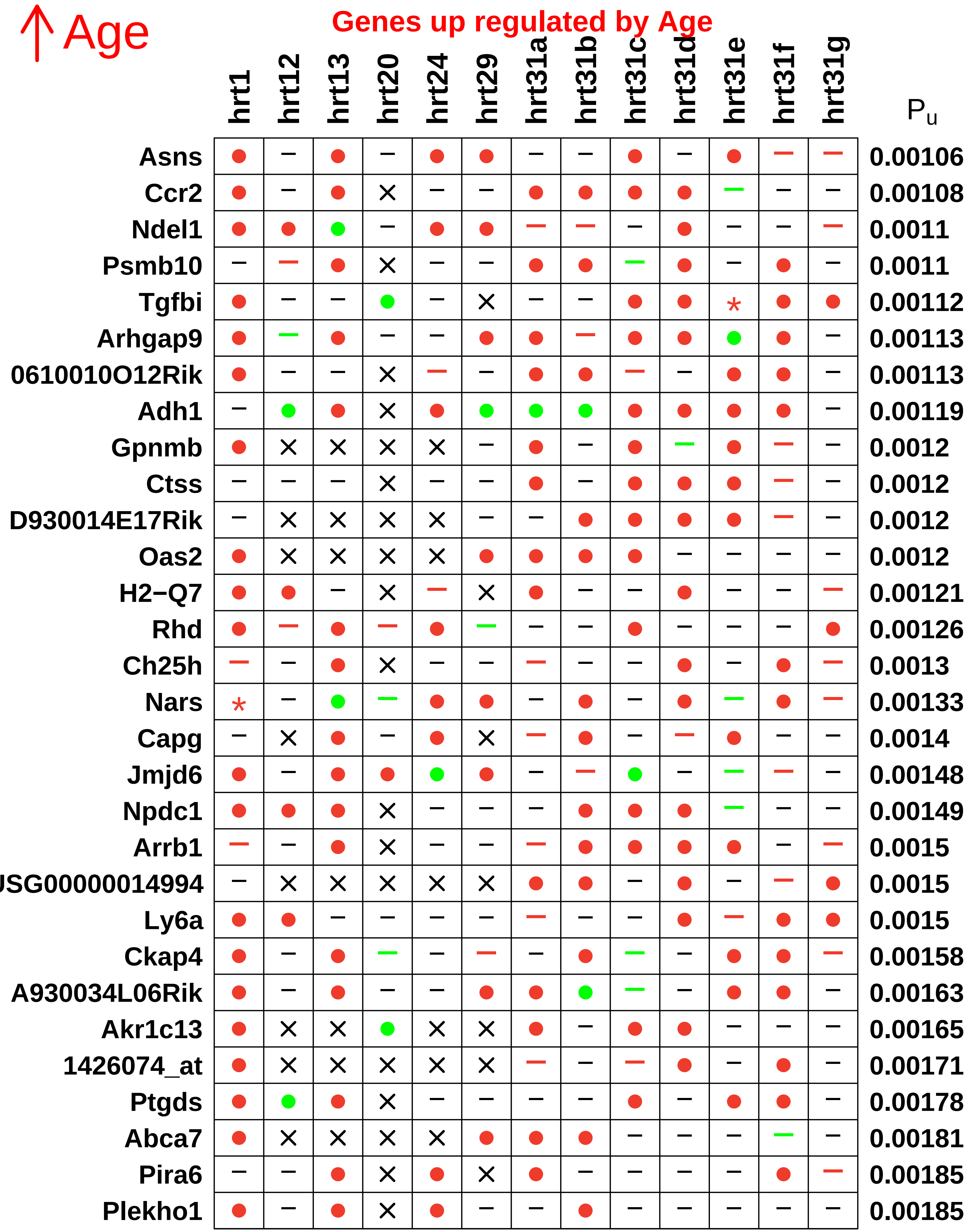
↑ Age
Genes up regulated by Age
hrt1
hrt12
hrt13
hrt20
hrt24
hrt29
hrt31a
hrt31b
hrt31c
hrt31d
hrt31e
hrt31f
hrt31g
P_u
Asns 0.00106
Ccr2 0.00108
Ndel1 0.0011
Psmb10 0.0011
Tgfbi 0.00112
Arhgap9 0.00113
0610010O12Rik 0.00113
Adh1 0.00119
Gpnmb 0.0012
Ctss 0.0012
D930014E17Rik 0.0012
Oas2 0.0012
H2−Q7 0.00121
Rhd 0.00126
Ch25h 0.0013
Nars 0.00133
Capg 0.0014
Jmjd6 0.00148
Npdc1 0.00149
Arrb1 0.0015
JSG00000014994 0.0015
Ly6a 0.0015
Ckap4 0.00158
A930034L06Rik 0.00163
Akr1c13 0.00165
1426074_at 0.00171
Ptgds 0.00178
Abca7 0.00181
Pira6 0.00185
Plekho1 0.00185

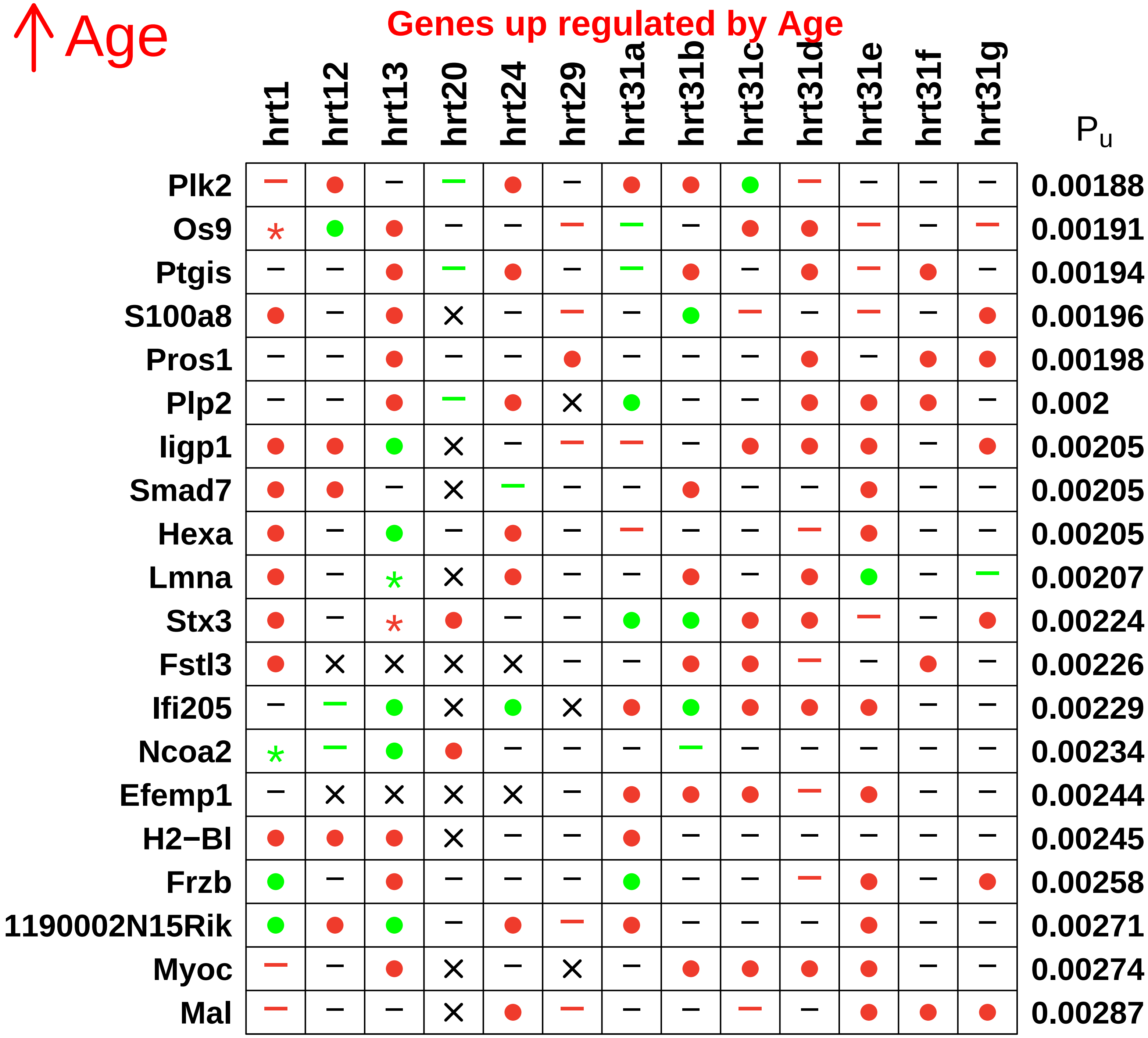

Age
Genes up regulated by Age
hrt1
hrt12
hrt13
hrt20
hrt24
hrt29
hrt31a
hrt31b
hrt31c
hrt31d
hrt31e
hrt31f
hrt31g
P_u
Plk2 0.00188
Os9 0.00191
Ptgis 0.00194
S100a8 0.00196
Pros1 0.00198
Plp2 0.002
Iigp1 0.00205
Smad7 0.00205
Hexa 0.00205
Lmna 0.00207
Stx3 0.00224
Fstl3 0.00226
Ifi205 0.00229
Ncoa2 0.00234
Efemp1 0.00244
H2−Bl 0.00245
Frzb 0.00258
1190002N15Rik 0.00271
Myoc 0.00274
Mal 0.00287

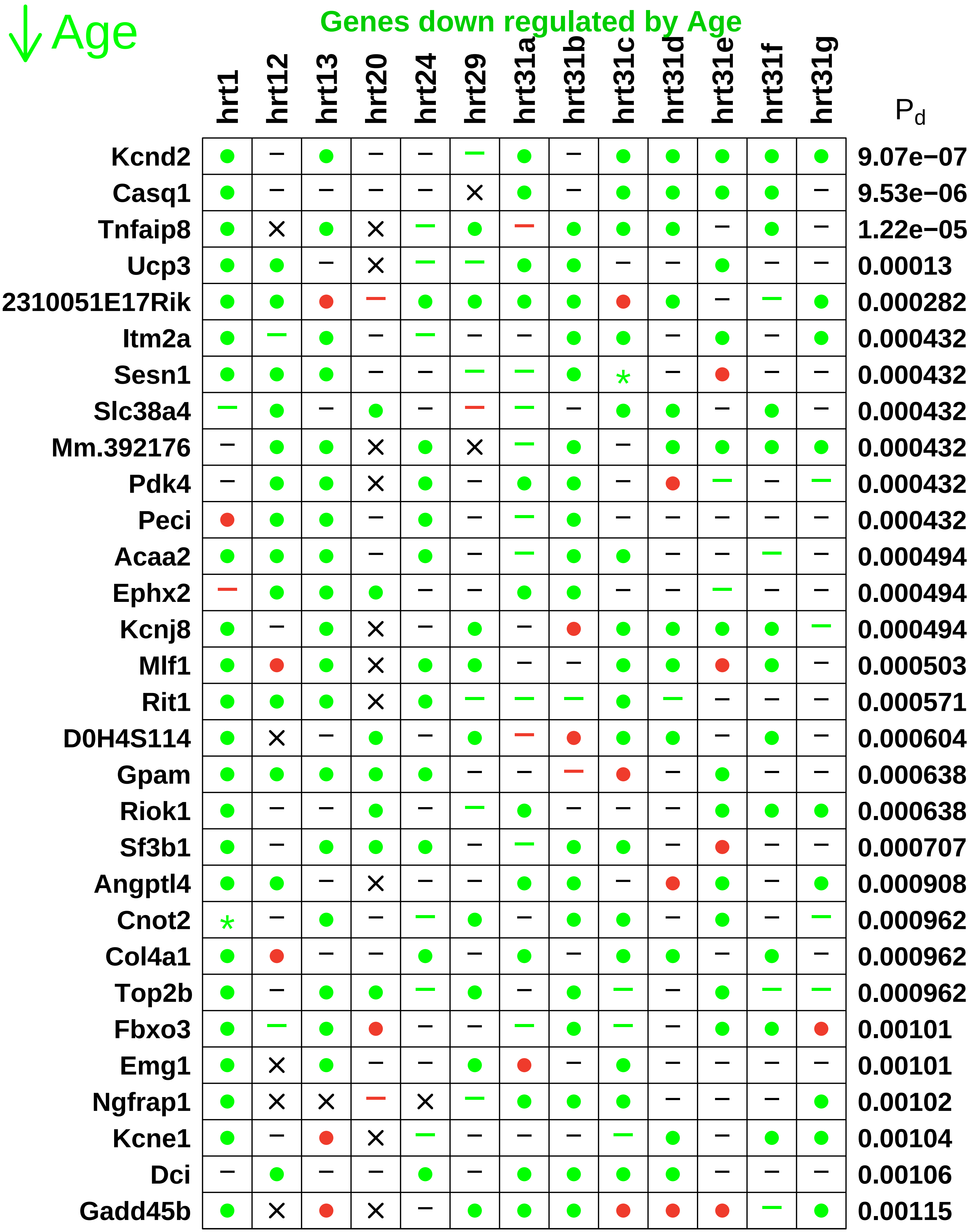

↓Age
Genes down regulated by Age
hrt1
hrt12
hrt13
hrt20
hrt24
hrt29
hrt31a
hrt31b
hrt31c
hrt31d
hrt31e
hrt31f
hrt31g
P_d
Kcnd2 9.07e−07
Casq1 9.53e−06
Tnfaip8 1.22e−05
Ucp3 0.00013
2310051E17Rik 0.000282
Itm2a 0.000432
Sesn1 0.000432
Slc38a4 0.000432
Mm.392176 0.000432
Pdk4 0.000432
Peci 0.000432
Acaa2 0.000494
Ephx2 0.000494
Kcnj8 0.000494
Mlf1 0.000503
Rit1 0.000571
D0H4S114 0.000604
Gpam 0.000638
Riok1 0.000638
Sf3b1 0.000707
Angptl4 0.000908
Cnot2 0.000962
Col4a1 0.000962
Top2b 0.000962
Fbxo3 0.00101
Emg1 0.00101
Ngfrap1 0.00102
Kcne1 0.00104
Dci 0.00106
Gadd45b 0.00115

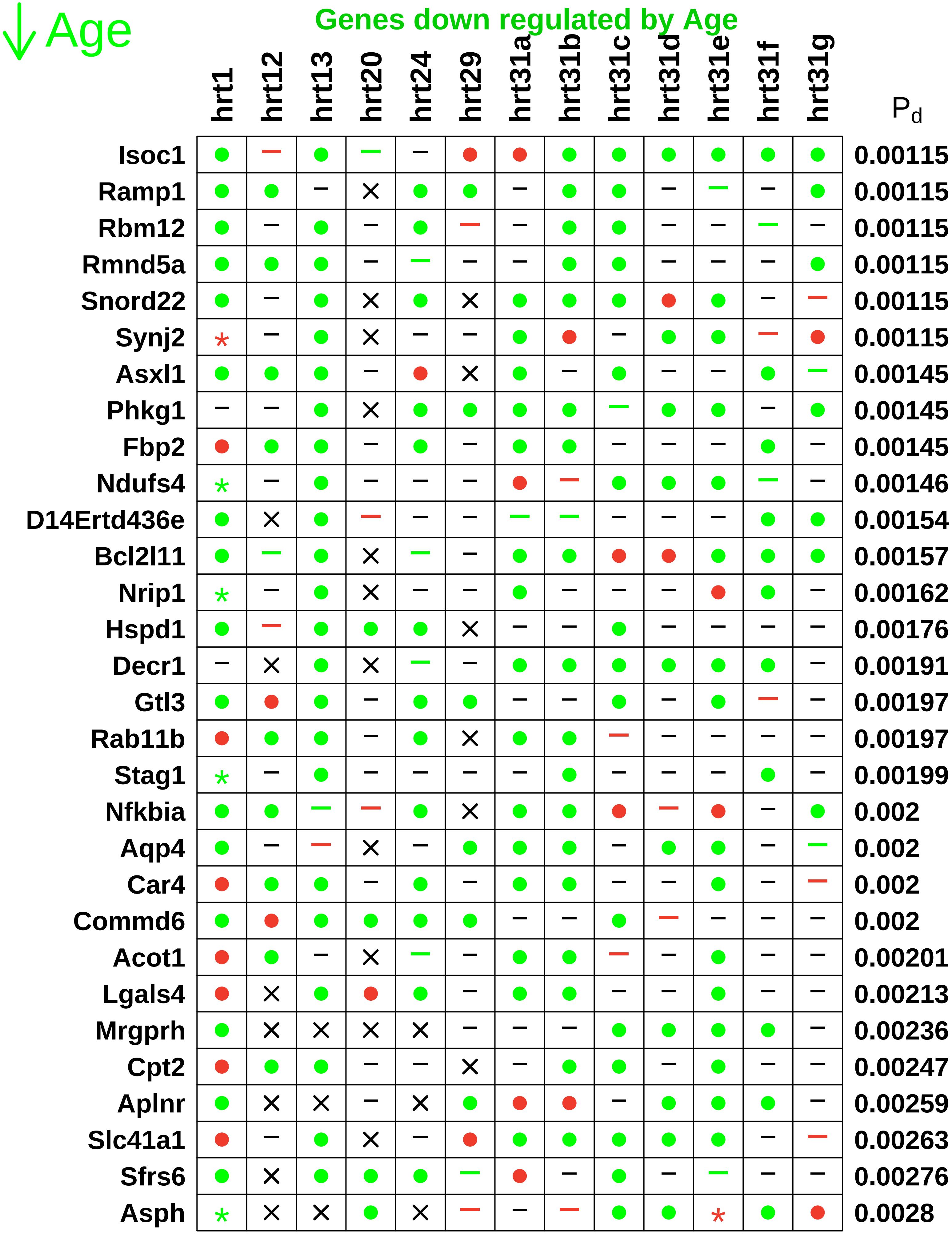

↓Age
Genes down regulated by Age
hrt1
hrt12
hrt13
hrt20
hrt24
hrt29
hrt31a
hrt31b
hrt31c
hrt31d
hrt31e
hrt31f
hrt31g
P_d
Isoc1 0.00115
Ramp1 0.00115
Rbm12 0.00115
Rmnd5a 0.00115
Snord22 0.00115
Synj2 0.00115
Asxl1 0.00145
Phkg1 0.00145
Fbp2 0.00145
Ndufs4 0.00146
D14Ertd436e 0.00154
Bcl2l11 0.00157
Nrip1 0.00162
Hspd1 0.00176
Decr1 0.00191
Gtl3 0.00197
Rab11b 0.00197
Stag1 0.00199
Nfkbia 0.002
Aqp4 0.002
Car4 0.002
Commd6 0.002
Acot1 0.00201
Lgals4 0.00213
Mrgprh 0.00236
Cpt2 0.00247
Aplnr 0.00259
Slc41a1 0.00263
Sfrs6 0.00276
Asph 0.0028

↓ Age

Genes down regulated by Age

	hrt1	hrt12	hrt13	hrt20	hrt24	hrt29	hrt31a	hrt31b	hrt31c	hrt31d	hrt31e	hrt31f	hrt31g	P_d
Slc25a20	●(red)	●(green)	●(green)	–	●(green)	–	●(green)	●(green)	–	–	–	●(green)	–	0.0028
Zfp36	●(green)	●(green)	–	×	–	×	●(green)	●(green)	–(green)	–	–	–	–	0.0028
Meg3	●(green)	×	●(red)	–	●(green)	–	●(green)	–	●(green)	●(green)	●(green)	–(green)	–(green)	0.00305
Hdac2	●(green)	×	●(green)	●(green)	●(green)	●(green)	–	–	●(green)	–	–	–	–	0.00315
Peg3	●(red)	●(green)	–	–	●(green)	–	●(green)	●(green)	–(green)	–	●(green)	–	●(green)	0.00315
Rnf166	●(green)	●(red)	●(green)	–	●(green)	–(green)	–	●(green)	–	●(green)	–	–	–	0.00315
Ubl5	●(green)	●(red)	●(green)	–	●(green)	–	–(green)	●(green)	●(green)	–(green)	●(green)	–	–	0.00315
Wasf2	●(green)	×	×	–	×	–	●(green)	●(green)	●(green)	–	–	–	–(green)	0.00315
Mrps33	●(green)	–(red)	●(green)	×	●(green)	●(green)	●(red)	–	●(green)	–	–	–	–	0.00317
Ccng2	●(green)	–(green)	●(green)	×	●(green)	–	–	●(green)	–(green)	–(red)	●(green)	–(red)	●(green)	0.00351
Epha4	●(green)	–	●(green)	×	–	–	–	–	●(red)	●(green)	●(green)	–	–	0.00351
Mlycd	●(red)	●(green)	●(red)	×	●(green)	–	●(green)	●(green)	–	–	●(green)	–	–	0.00351
Ak1	●(green)	●(red)	●(green)	×	●(green)	●(green)	–(red)	●(red)	●(green)	–(green)	–	–	–	0.00361
Ramp2	●(red)	–	●(green)	–	●(green)	●(green)	●(green)	–(green)	–(green)	–	●(red)	–	–	0.00377
Rora	●(green)	●(green)	●(green)	×	–	–	●(green)	–(green)	–	●(green)	●(green)	–	–	0.00377
Pdcd4	●(green)	–	●(green)	●(red)	●(green)	–	–	●(green)	●(green)	–	–	–	–	0.00383
Tmem16k	●(green)	×	×	×	×	–	–	–	●(green)	●(green)	–	–(green)	●(green)	0.00391
Ap1m1	●(green)	●(green)	●(green)	–	●(green)	×	–	–	●(green)	–	●(green)	–(red)	–	0.00408
Errfi1	●(green)	●(green)	–	●(red)	●(green)	–	●(green)	●(green)	–(green)	–	–(green)	–	–	0.00408
Gas5	–(green)	●(green)	●(green)	●(green)	●(green)	–	●(green)	●(green)	●(green)	●(red)	–	–	●(green)	0.00408
Jarid2	–	–	–(green)	●(red)	–	–	–	●(green)	●(green)	●(green)	–	●(green)	–	0.00408
Ireb2	●(green)	–(red)	●(green)	–	–	×	–	●(green)	●(green)	–(green)	–	–	–	0.00408
Cdk2	●(green)	–	●(red)	–	●(green)	–(green)	–	●(green)	–(green)	–	●(green)	–	–	0.00414
Eif4b	●(red)	●(green)	●(green)	●(red)	●(red)	●(green)	●(green)	●(green)	●(green)	–(red)	●(green)	–	–	0.00428
Lpin1	●(green)	●(red)	●(green)	×	●(red)	–	–(green)	●(green)	●(green)	●(green)	–	–(green)	–	0.00428
Pim3	●(green)	●(green)	●(green)	–	–	●(green)	●(green)	●(green)	–	–	–	–(green)	–	0.00428
Hadh	●(red)	–(green)	●(green)	–	●(green)	●(green)	–	–(green)	*(green)	–	●(red)	●(green)	–	0.00459
LOC100045629	–	–	●(green)	●(green)	●(green)	×	●(green)	●(green)	–	–	●(green)	–	–	0.00459
Armcx3	●(green)	×	×	–	×	●(green)	–	●(green)	–(green)	–	–	●(green)	–(green)	0.0046
Npm3	●(green)	–	●(red)	–	●(green)	×	–	●(green)	●(green)	●(green)	–	–	–(green)	0.00478

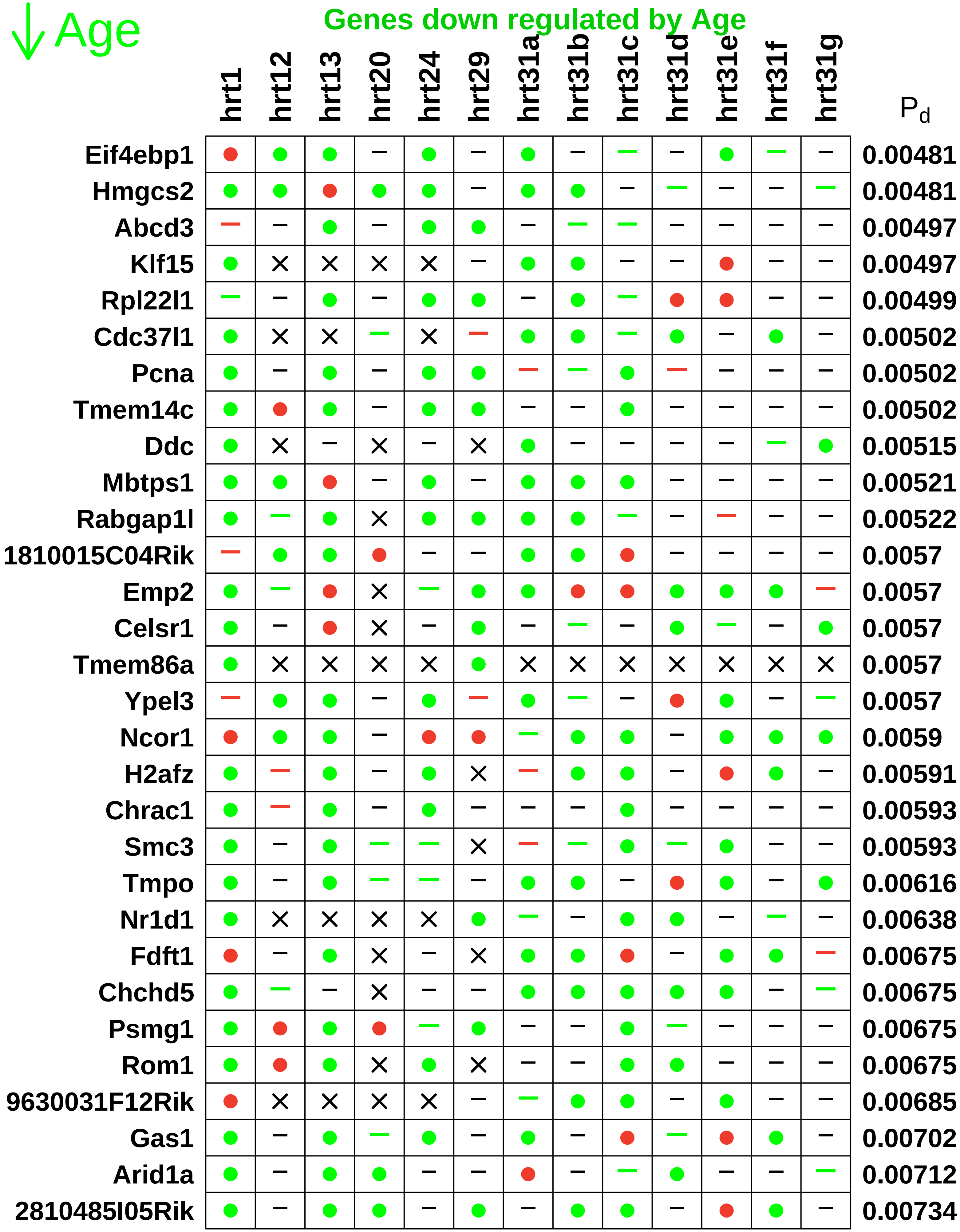

Age
Genes down regulated by Age
hrt1
hrt12
hrt13
hrt20
hrt24
hrt29
hrt31a
hrt31b
hrt31c
hrt31d
hrt31e
hrt31f
hrt31g
P_d
Eif4ebp1 0.00481
Hmgcs2 0.00481
Abcd3 0.00497
Klf15 0.00497
Rpl22l1 0.00499
Cdc37l1 0.00502
Pcna 0.00502
Tmem14c 0.00502
Ddc 0.00515
Mbtps1 0.00521
Rabgap1l 0.00522
1810015C04Rik 0.0057
Emp2 0.0057
Celsr1 0.0057
Tmem86a 0.0057
Ypel3 0.0057
Ncor1 0.0059
H2afz 0.00591
Chrac1 0.00593
Smc3 0.00593
Tmpo 0.00616
Nr1d1 0.00638
Fdft1 0.00675
Chchd5 0.00675
Psmg1 0.00675
Rom1 0.00675
9630031F12Rik 0.00685
Gas1 0.00702
Arid1a 0.00712
2810485I05Rik 0.00734

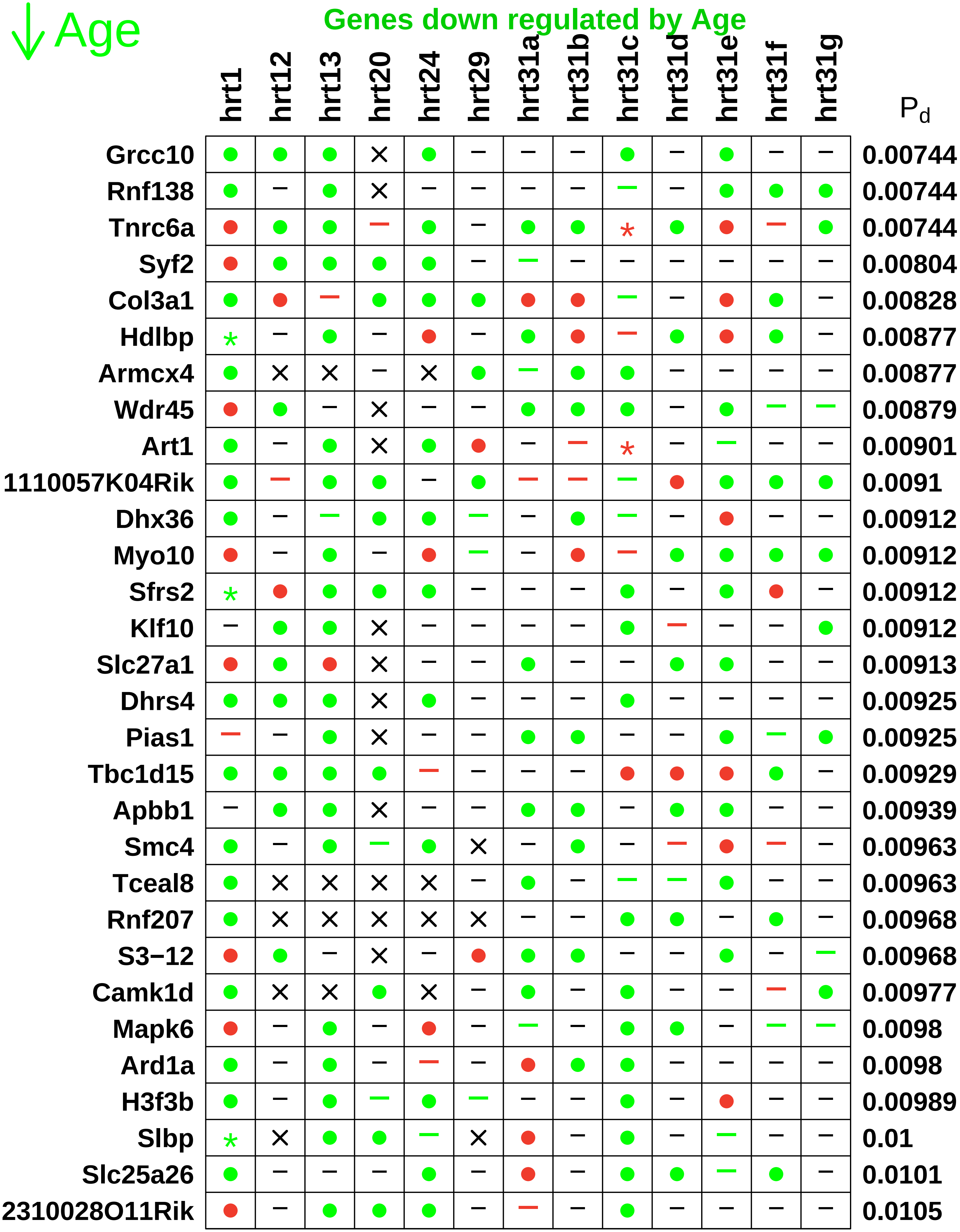
↓Age
Genes down regulated by Age
hrt1
hrt12
hrt13
hrt20
hrt24
hrt29
hrt31a
hrt31b
hrt31c
hrt31d
hrt31e
hrt31f
hrt31g
P_d
Grcc10 0.00744
Rnf138 0.00744
Tnrc6a 0.00744
Syf2 0.00804
Col3a1 0.00828
Hdlbp 0.00877
Armcx4 0.00877
Wdr45 0.00879
Art1 0.00901
1110057K04Rik 0.0091
Dhx36 0.00912
Myo10 0.00912
Sfrs2 0.00912
Klf10 0.00912
Slc27a1 0.00913
Dhrs4 0.00925
Pias1 0.00925
Tbc1d15 0.00929
Apbb1 0.00939
Smc4 0.00963
Tceal8 0.00963
Rnf207 0.00968
S3−12 0.00968
Camk1d 0.00977
Mapk6 0.0098
Ard1a 0.0098
H3f3b 0.00989
Slbp 0.01
Slc25a26 0.0101
2310028O11Rik 0.0105

↓ Age

Genes down regulated by Age

	hrt1	hrt12	hrt13	hrt20	hrt24	hrt29	hrt31a	hrt31b	hrt31c	hrt31d	hrt31e	hrt31f	hrt31g	P_d
Eral1	red ●	−	green ●	−	−	green −	green ●	−	green ●	green ●	green ●	green ●	green ●	0.0105
BC043118	red ●	×	×	green ●	×	green ●	−	−	−	green ●	−	green ●	−	0.0105
Hsdl2	green ●	green ●	green ●	−	green −	−	green −	green ●	−	−	−	−	−	0.0105
Tfrc	green ●	red ●	red ●	green ●	−	−	red ●	red ●	green ●	green ●	−	green ●	−	0.0107
Chkb	red ●	green ●	−	×	green ●	−	−	green −	green ●	−	−	−	−	0.0107
Fbxo9	green ●	−	green ●	×	green ●	green ●	green −	−	−	−	−	red ●	−	0.0107
Ift81	green ●	red ●	green ●	−	−	−	−	green ●	green ●	green ●	−	green ●	−	0.0107
Matr3	red ●	−	green ●	−	green −	×	green −	green ●	red −	−	red ●	green ●	green ●	0.0107
Nsmce1	red −	−	green ●	green ●	green ●	×	green ●	−	green ●	−	green −	green ●	−	0.0107
Nsun3	green ●	×	×	green ●	×	green −	×	×	×	×	×	×	×	0.0107
Lgi1	green ●	×	×	×	×	red ●	−	−	−	−	green ●	green ●	−	0.0107
Sfrs10	green ●	×	green ●	−	green ●	×	−	−	green ●	−	−	−	−	0.0108
Slc35b4	green ●	×	×	green ●	×	−	−	green ●	green ●	−	green ●	−	−	0.0113
Erh	green ●	red ●	green ●	×	green ●	×	−	−	green ●	−	−	−	−	0.0113
Frat2	green ●	−	red ●	−	−	−	−	green ●	−	green ●	green ●	green ●	−	0.0113
Rere	red ●	green ●	green ●	−	−	−	green ●	green −	green −	−	green ●	−	−	0.0113
Zfpm2	green ●	−	−	×	−	green −	green ●	−	red ●	green −	green ●	green ●	−	0.0113
Rgs4	green ●	−	−	×	−	−	−	red −	red *	green −	green ●	−	green ●	0.0116
Btg1	green ●	−	green ●	red −	green −	×	red −	−	green ●	−	−	−	−	0.0116
Rfc4	green ●	red −	green −	×	green −	green ●	red −	green −	−	−	green ●	−	−	0.0117
Trp53inp1	green ●	×	×	−	×	−	green ●	green ●	−	green ●	red −	−	green −	0.0117
Nit1	green ●	×	green ●	−	green ●	green ●	−	−	−	−	green ●	red *	−	0.0118
Tmem35	green ●	×	×	×	×	red ●	green ●	−	green ●	green −	green ●	−	green ●	0.0118
1190002H23Rik	green ●	×	−	×	green ●	green ●	green ●	green ●	red ●	red ●	red ●	−	−	0.0118
Atp1a2	red ●	−	−	×	−	−	green ●	green ●	red ●	green ●	green −	green ●	−	0.0118
Bub1b	green ●	×	×	green ●	×	×	−	green ●	green −	−	green −	−	green ●	0.0118
Dsg2	green *	green ●	green ●	−	−	green ●	green ●	green −	red ●	−	red ●	green ●	−	0.0118
Pex11a	red ●	green ●	red ●	−	green ●	−	green ●	green ●	−	−	green ●	−	−	0.0118
Slc25a17	green ●	−	red ●	green ●	green ●	−	green ●	green ●	−	red −	green ●	−	−	0.0118
Ttll1	green *	×	×	×	×	−	green ●	−	green ●	green ●	−	green ●	−	0.0118

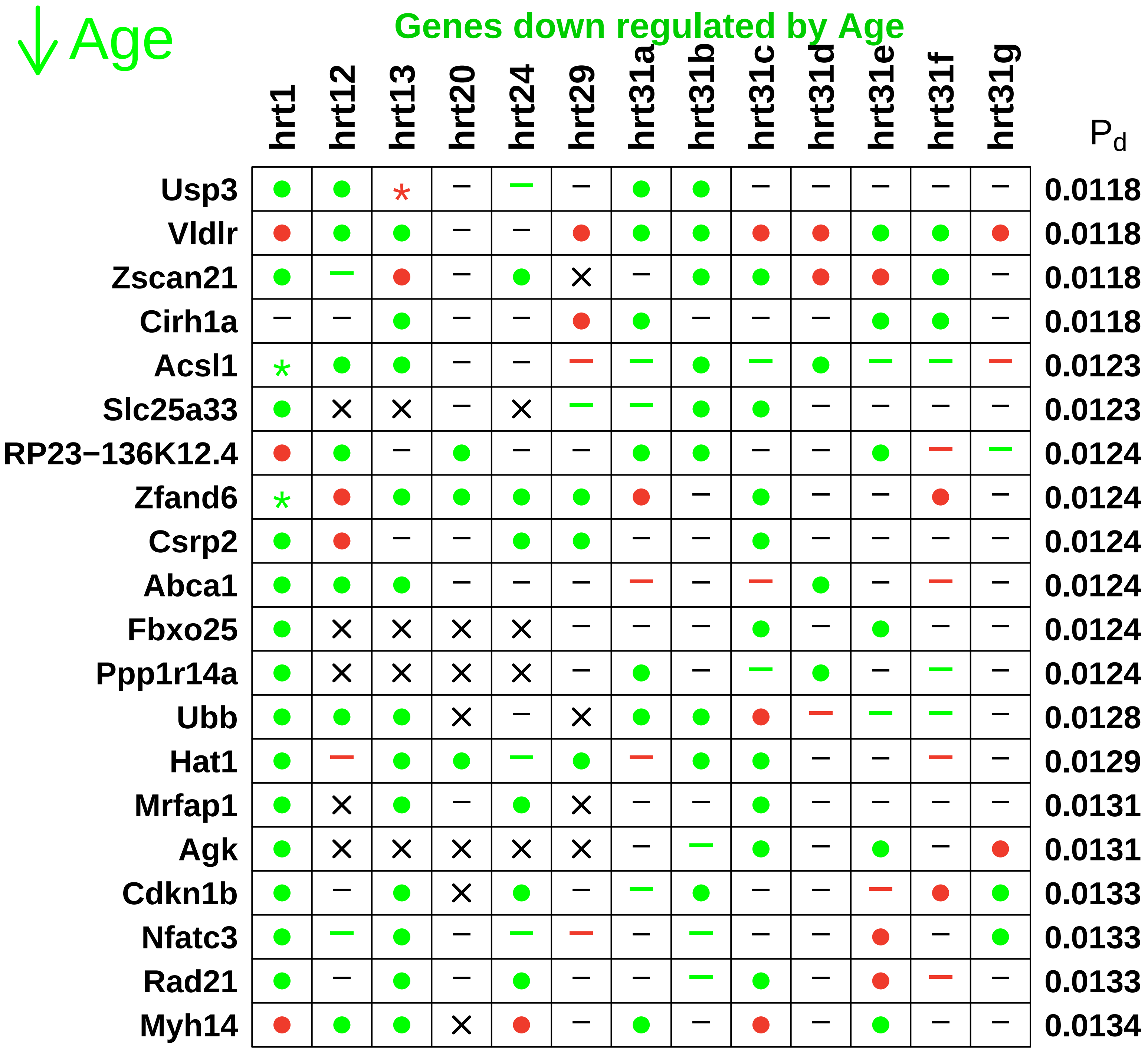

↓Age
Genes down regulated by Age
hrt1
hrt12
hrt13
hrt20
hrt24
hrt29
hrt31a
hrt31b
hrt31c
hrt31d
hrt31e
hrt31f
hrt31g
P_d
Usp3 0.0118
Vldlr 0.0118
Zscan21 0.0118
Cirh1a 0.0118
Acsl1 0.0123
Slc25a33 0.0123
RP23−136K12.4 0.0124
Zfand6 0.0124
Csrp2 0.0124
Abca1 0.0124
Fbxo25 0.0124
Ppp1r14a 0.0124
Ubb 0.0128
Hat1 0.0129
Mrfap1 0.0131
Agk 0.0131
Cdkn1b 0.0133
Nfatc3 0.0133
Rad21 0.0133
Myh14 0.0134

↑Age

Overrepresented Biological Processes

GO Term	P−Value
humoral immune response	5.93e−06
inflammatory response	2.04e−05
activation of plasma proteins during acute inflammatory response	3.65e−05
response to external stimulus	0.000123
positive regulation of phagocytosis	0.000149
complement activation, classical pathway	0.000198
positive regulation of leukocyte activation	0.000341
regulation of lymphocyte proliferation	0.000402
regulation of B cell activation	0.000463
positive regulation of transport	0.000824
activation of immune response	0.000855
regulation of cell activation	0.00123
ISG15−protein conjugation	0.00147
positive regulation of mononuclear cell proliferation	0.00209
prostanoid metabolic process	0.0021
phagocytosis, engulfment	0.0021
erythrocyte development	0.0021
regulation of immune response	0.00261
long−chain fatty acid biosynthetic process	0.0029
defense response to Gram−negative bacterium	0.0029
regulation of cell migration	0.003
leukocyte mediated immunity	0.00349
cell adhesion	0.00372
positive regulation of response to stimulus	0.00382
fibroblast growth factor receptor signaling pathway	0.00413
regulation of endocytosis	0.00479
negative regulation of angiogenesis	0.005
microtubule−based movement	0.00576
positive regulation of cellular component organization and biogenesis	0.00595
positive regulation of B cell proliferation	0.00597

↑Age

Overrepresented Biological Processes

GO Term	P−Value
response to stress	0.00671
somatic stem cell division	0.00705
defense response to Gram−positive bacterium	0.00705
blood vessel morphogenesis	0.00732
regulation of leukocyte mediated immunity	0.00743
regulation of cell proliferation	0.00802
regulation of T cell differentiation	0.00822
positive regulation of immune system process	0.00828
immune response	0.00844
positive regulation of B cell differentiation	0.00972
innate immune response	0.00973
fatty acid metabolic process	0.0102
hemopoiesis	0.0106
catabolic process	0.011
phagocytosis, recognition	0.0127
prostaglandin biosynthetic process	0.0128
regulation of acute inflammatory response	0.0128
regulation of hypersensitivity	0.0128
negative regulation of axon extension	0.0128
skeletal development	0.0128
icosanoid biosynthetic process	0.0141
sphingolipid metabolic process	0.0165
cytokine and chemokine mediated signaling pathway	0.0165
ceramide metabolic process	0.0177
chemotaxis	0.0179
negative regulation of lymphocyte mediated immunity	0.0199
oligopeptide transport	0.0199
complement activation, alternative pathway	0.0199
endoplasmic reticulum unfolded protein response	0.0199
neurotransmitter catabolic process	0.0199

↑ Age

Overrepresented Biological Processes

GO Term	P−Value
positive regulation of T cell activation	0.0213
cellular macromolecule catabolic process	0.0235
regulation of immunoglobulin mediated immune response	0.024
positive regulation of immune effector process	0.024
regulation of adaptive immune response based on somatic recombination of immune receptors built from i[illegible]	0.024
carboxylic acid biosynthetic process	0.0249
lymphocyte activation	0.0251
cellular developmental process	0.0257
glucose catabolic process	0.0269
monosaccharide catabolic process	0.0269
anatomical structure formation	0.027
negative regulation of immune effector process	0.0284
regulation of inflammatory response to antigenic stimulus	0.0284
water transport	0.0284
regulation of defense response	0.0287
response to unfolded protein	0.0288
blood vessel development	0.0289
immune system development	0.0307
alcohol metabolic process	0.0314
vasculogenesis	0.0314
oxidation reduction	0.032
negative regulation of inflammatory response	0.0331
positive regulation of T cell proliferation	0.0341
antigen processing and presentation of peptide antigen	0.0341
cell death	0.0355
myeloid leukocyte mediated immunity	0.038
cell wall organization and biogenesis	0.038
cell wall catabolic process	0.038
aromatic compound catabolic process	0.038
mast cell activation	0.038

↑Age

Overrepresented Biological Processes

GO Term	P−Value
immunoglobulin mediated immune response	0.0397
adaptive immune response based on somatic recombination of immune receptors built from immunoglobul	0.0414
cytoskeleton organization and biogenesis	0.0417
positive regulation of developmental process	0.0424
positive regulation of alpha−beta T cell differentiation	0.0432
regulation of apoptosis	0.0447
cell motility	0.0448
carbohydrate metabolic process	0.0452
anatomical structure development	0.0455
negative regulation of cell activation	0.046
negative regulation of lymphocyte activation	0.046
nervous system development	0.0462
membrane organization and biogenesis	0.0467
tube morphogenesis	0.0485
cartilage condensation	0.0487
organic anion transport	0.0487
negative regulation of response to stimulus	0.0492
negative regulation of cellular component organization and biogenesis	0.0498

↑Age

Overrepresented Cell Components

GO Term	P−Value
extracellular region	1.05e−06
extracellular space	0.000261
6−phosphofructokinase complex	0.00128
cell surface	0.00405
cytosol	0.00567
external side of plasma membrane	0.00636
proteinaceous extracellular matrix	0.0068
cyclin−dependent protein kinase holoenzyme complex	0.00846
extracellular region part	0.00977
plasma membrane	0.0113
intrinsic to endoplasmic reticulum membrane	0.0146
axon	0.0232
filopodium	0.0248
vacuolar membrane	0.0379
proton−transporting two−sector ATPase complex	0.0412
microtubule	0.0466
cytoplasm	0.0478

↑Age

Overrepresented Molecular Functions

GO Term	P−Value
heparin binding	0.000233
polysaccharide binding	0.000349
protease inhibitor activity	0.000573
integrin binding	0.000679
chemokine activity	0.000727
6−phosphofructokinase activity	0.00127
folic acid transporter activity	0.00413
G−protein−coupled receptor binding	0.00437
O−methyltransferase activity	0.00611
glutathione transferase activity	0.0102
metalloendopeptidase inhibitor activity	0.0111
lysozyme activity	0.0141
immunoglobulin binding	0.0141
catalytic activity	0.0149
carbohydrate binding	0.0164
aldo−keto reductase activity	0.0173
oligopeptide transporter activity	0.0173
serine−type endopeptidase inhibitor activity	0.0198
water channel activity	0.0209
protein binding	0.0222
protein homodimerization activity	0.0241
GTPase activity	0.0271
hydrogen ion transporting ATP synthase activity, rotational mechanism	0.0282
hydrogen ion transporting ATPase activity, rotational mechanism	0.0282
cyclin−dependent protein kinase regulator activity	0.0288
monooxygenase activity	0.0293
phosphotransferase activity, nitrogenous group as acceptor	0.0306
hydrogen−exporting ATPase activity, phosphorylative mechanism	0.0332
NADP binding	0.0332
hormone receptor binding	0.0332

↓Age

Overrepresented Biological Processes

GO Term	P−Value
lipid metabolic process	4.25e−05
negative regulation of S phase of mitotic cell cycle	0.000271
fatty acid metabolic process	0.000485
carboxylic acid metabolic process	0.000486
cell cycle arrest	0.000953
regulation of S phase	0.00133
response to exogenous dsRNA	0.00245
sterol metabolic process	0.00602
mitotic cell cycle	0.00655
long−chain fatty acid metabolic process	0.00662
isoprenoid biosynthetic process	0.0088
spleen development	0.0088
actin filament−based movement	0.00999
regulation of G−protein coupled receptor protein signaling pathway	0.0113
posttranscriptional regulation of gene expression	0.0117
thymus development	0.0126
cerebellum morphogenesis	0.014
negative regulation of myeloid cell differentiation	0.014
response to lipopolysaccharide	0.0154
cholesterol biosynthetic process	0.0202
chromosome organization and biogenesis	0.0213
mitosis	0.0216
response to toxin	0.0332
metencephalon development	0.0332
intracellular transport	0.0338
DNA packaging	0.0426
potassium ion transport	0.0444
ear development	0.0466
negative regulation of cell cycle	0.0494

↓Age

Overrepresented Cell Components

GO Term	P−Value
mitochondrial inner membrane	9.59e−06
intracellular	1.74e−05
mitochondrial envelope	5.22e−05
envelope	6.03e−05
mitochondrion	6.48e−05
peroxisome	0.000244
membrane−bounded organelle	3e−04
intracellular organelle	0.000447
organelle membrane	0.00119
cyclin−dependent protein kinase holoenzyme complex	0.00176
collagen	0.0133
cell part	0.0156
nucleus	0.0183
histone deacetylase complex	0.0192
replication fork	0.0192
early endosome	0.0209
cytoplasm	0.023
sarcoplasm	0.0298
heterochromatin	0.0489
chromosomal part	0.0492

↓Age

Overrepresented Molecular Functions

GO Term	P−Value
dodecenoyl−CoA delta−isomerase activity	0.000233
coreceptor, soluble ligand activity	0.000233
translation repressor activity	0.000767
extracellular matrix structural constituent	0.019
transferase activity, transferring acyl groups	0.0216
nucleic acid binding	0.037
voltage−gated potassium channel activity	0.0398
Rab GTPase activator activity	0.0404
carbon−carbon lyase activity	0.0404
intramolecular oxidoreductase activity	0.0404

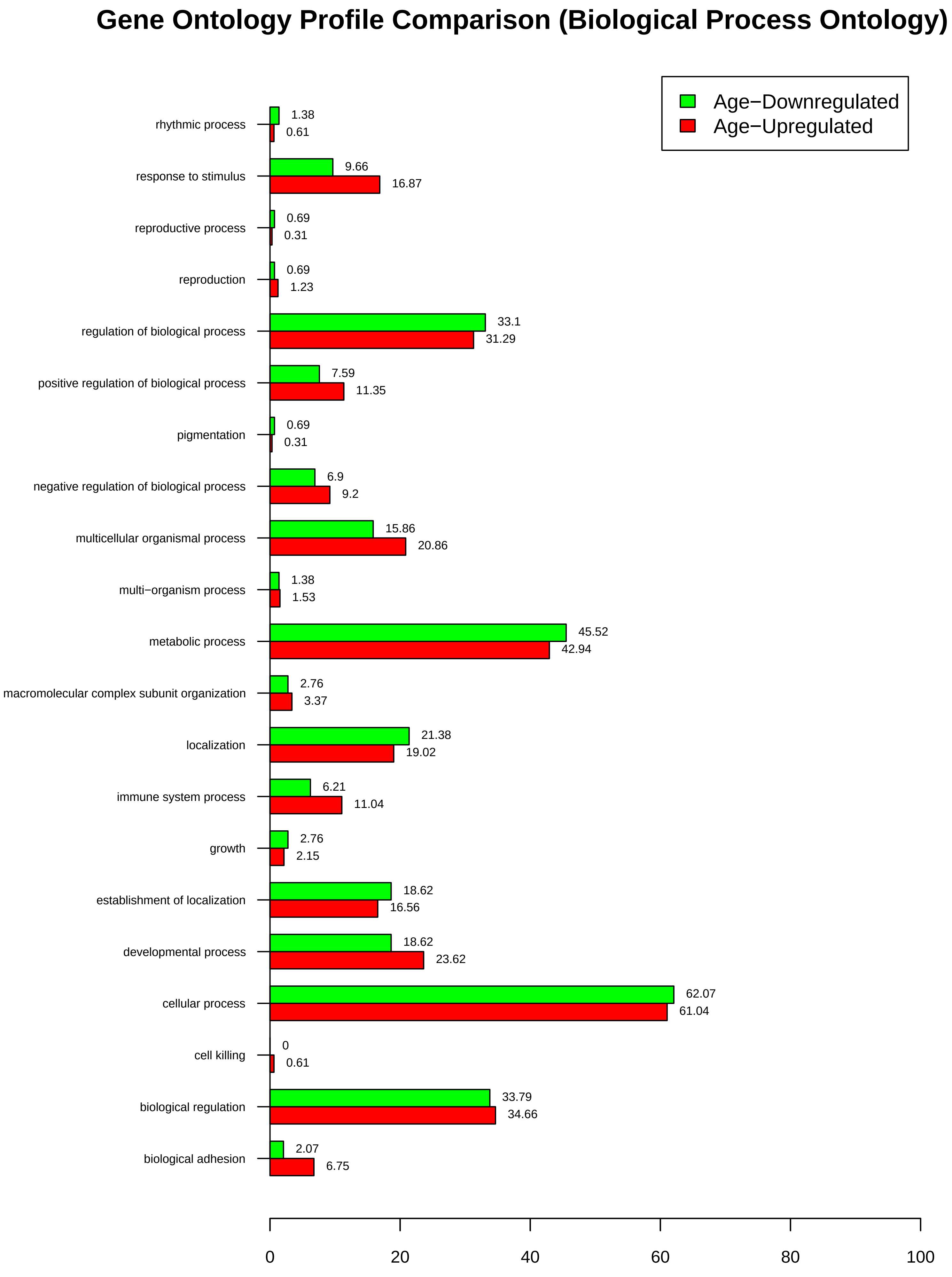
Gene Ontology Profile Comparison (Biological Process Ontology)
Age−Downregulated
Age−Upregulated
rhythmic process
1.38
0.61
response to stimulus
9.66
16.87
reproductive process
0.69
0.31
reproduction
0.69
1.23
regulation of biological process
33.1
31.29
positive regulation of biological process
7.59
11.35
pigmentation
0.69
0.31
negative regulation of biological process
6.9
9.2
multicellular organismal process
15.86
20.86
multi−organism process
1.38
1.53
metabolic process
45.52
42.94
macromolecular complex subunit organization
2.76
3.37
localization
21.38
19.02
immune system process
6.21
11.04
growth
2.76
2.15
establishment of localization
18.62
16.56
developmental process
18.62
23.62
cellular process
62.07
61.04
cell killing
0
0.61
biological regulation
33.79
34.66
biological adhesion
2.07
6.75
0
20
40
60
80
100

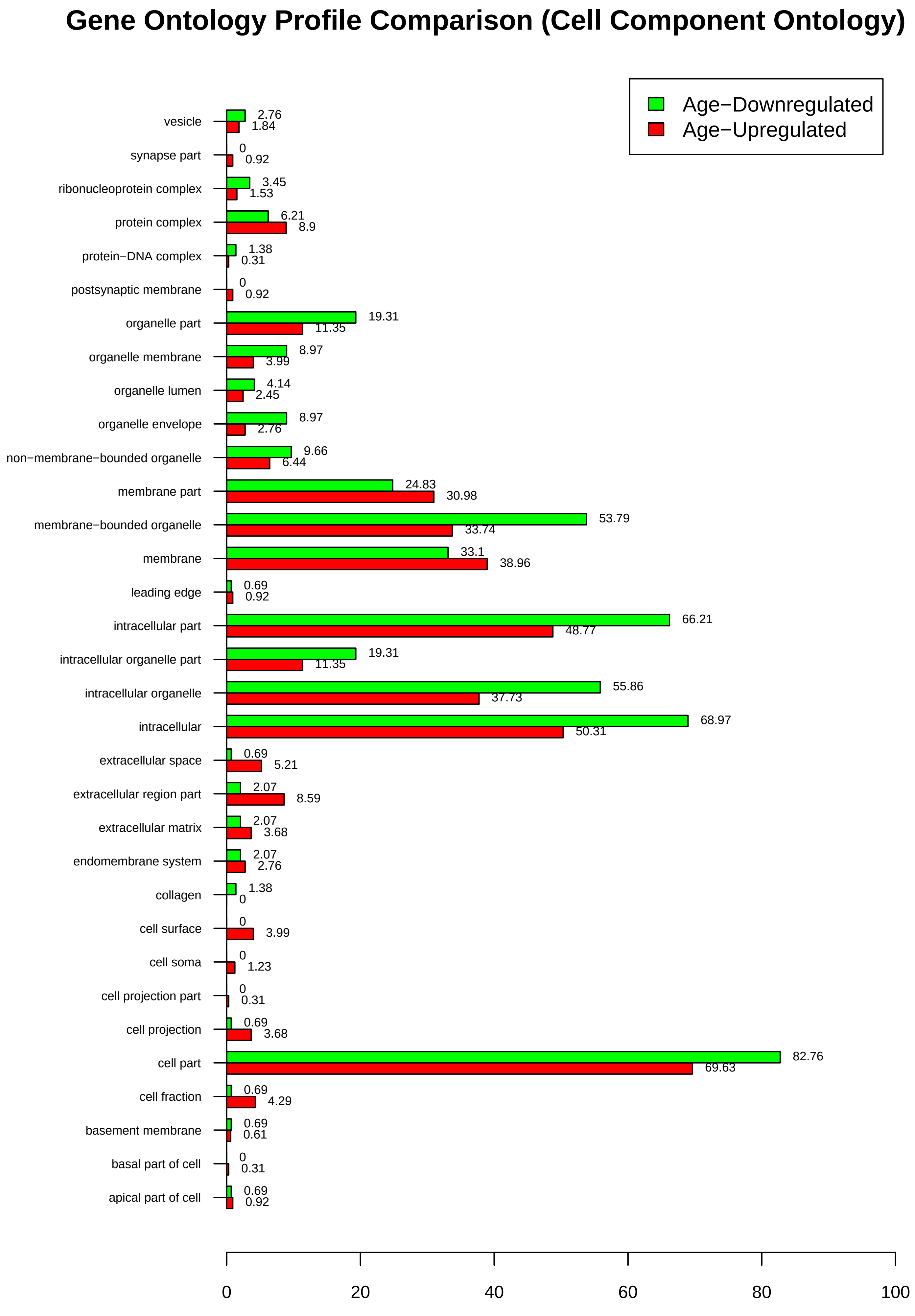
Gene Ontology Profile Comparison (Cell Component Ontology)
Age−Downregulated
Age−Upregulated
vesicle
2.76
1.84
synapse part
0
0.92
ribonucleoprotein complex
3.45
1.53
protein complex
6.21
8.9
protein−DNA complex
1.38
0.31
postsynaptic membrane
0
0.92
organelle part
19.31
11.35
organelle membrane
8.97
3.99
organelle lumen
4.14
2.45
organelle envelope
8.97
2.76
non−membrane−bounded organelle
9.66
6.44
membrane part
24.83
30.98
membrane−bounded organelle
53.79
33.74
membrane
33.1
38.96
leading edge
0.69
0.92
intracellular part
66.21
48.77
intracellular organelle part
19.31
11.35
intracellular organelle
55.86
37.73
intracellular
68.97
50.31
extracellular space
0.69
5.21
extracellular region part
2.07
8.59
extracellular matrix
2.07
3.68
endomembrane system
2.07
2.76
collagen
1.38
0
cell surface
0
3.99
cell soma
0
1.23
cell projection part
0
0.31
cell projection
0.69
3.68
cell part
82.76
69.63
cell fraction
0.69
4.29
basement membrane
0.69
0.61
basal part of cell
0
0.31
apical part of cell
0.69
0.92
0
20
40
60
80
100

Gene Ontology Profile Comparison (Molecular Function Ontology)

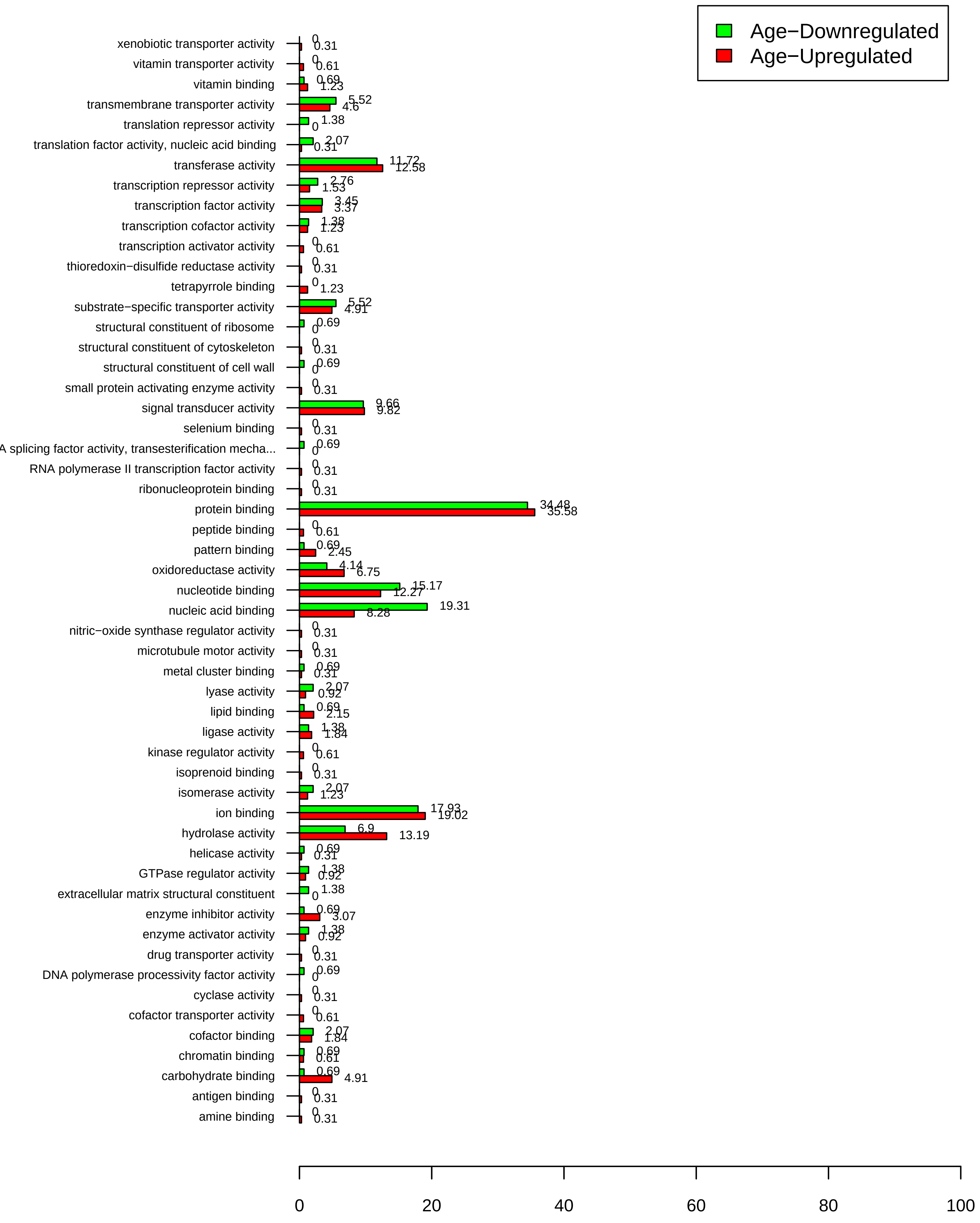

↑Age

Overrepresented KEGG Pathways

GO Term	P−Value
Galactose metabolism	0.00191
Glycolysis / Gluconeogenesis	0.00436
Metabolism of xenobiotics by cytochrome P450	0.00804
Type I diabetes mellitus	0.0119
Complement and coagulation cascades	0.0145
Neurodegenerative Diseases	0.0248
Fructose and mannose metabolism	0.0428
Alzheimer's disease	0.0441
Butanoate metabolism	0.0458

↓Age

Overrepresented KEGG Pathways

GO Term	P−Value
Fatty acid metabolism	8.23e−05
Fatty acid elongation in mitochondria	0.00509
p53 signaling pathway	0.00511
PPAR signaling pathway	0.00703
Valine, leucine and isoleucine degradation	0.0101
Small cell lung cancer	0.0143
Cell cycle	0.0304
Phenylalanine metabolism	0.0353

↑Age

Overrepresented KEGG Pathways

(Based on InterPro Domain Signatures)

GO Term	P−Value
Metabolism of xenobiotics by cytochrome P450	0.0015
Drug metabolism − cytochrome P450	0.0032
Ribosome	0.0065
Glycosphingolipid biosynthesis − globoseries	0.0102
Parkinson's disease	0.0106
Alzheimer's disease	0.0123
Glycosphingolipid biosynthesis − ganglioseries	0.0148
Fructose and mannose metabolism	0.0156
Glycolysis / Gluconeogenesis	0.0158
Starch and sucrose metabolism	0.0296

↓Age

Overrepresented KEGG Pathways

(Based on InterPro Domain Signatures)

GO Term	P−Value
Butanoate metabolism	0.0016
Fatty acid elongation in mitochondria	0.0047
Terpenoid biosynthesis	0.0049
Lysine degradation	0.0073
Caprolactam degradation	0.0082
Taurine and hypotaurine metabolism	0.0162
Tetrachloroethene degradation	0.0264
PPAR signaling pathway	0.0277
Synthesis and degradation of ketone bodies	0.029
Valine, leucine and isoleucine degradation	0.036
Benzoate degradation via CoA ligation	0.0367
Pentose phosphate pathway	0.0381
C5−Branched dibasic acid metabolism	0.0391
Circadian rhythm	0.0414
Thiamine metabolism	0.043
Nitrogen metabolism	0.0434

↑Age

Abundance of miRNA Targets

miRNA	Freq(Obs)	Freq(Exp)	Obs/Exp	P−value	P−Value(Adj)
miR−293	0.0528	0.0266	1.98	0.00658	1
miR−423−5p	0.175	0.141	1.24	0.0538	1
miR−127	0.0407	0.0272	1.49	0.0748	1
miR−483	0.0854	0.0668	1.28	0.1	1
miR−296−5p	0.065	0.0498	1.31	0.109	1
miR−711	0.0407	0.0303	1.34	0.129	1
miR−762	0.179	0.155	1.15	0.131	1
miR−296−3p	0.146	0.125	1.17	0.133	1
miR−193	0.0935	0.0768	1.22	0.135	1
miR−299	0.0976	0.0809	1.21	0.141	1

↓Age

Abundance of miRNA Targets

miRNA	Freq(Obs)	Freq(Exp)	Obs/Exp	P−value	P−Value(Adj)
miR−183	0.177	0.106	1.67	0.00542	0.995
miR−148a	0.194	0.126	1.53	0.0116	0.995
miR−148b	0.194	0.129	1.51	0.0143	0.995
miR−715	0.0484	0.0207	2.34	0.0147	0.995
miR−1188	0.137	0.0873	1.57	0.0221	0.995
miR−540−3p	0.129	0.0811	1.59	0.0227	0.995
miR−668	0.113	0.0703	1.61	0.0277	0.995
miR−125b−3p	0.0645	0.036	1.79	0.0356	0.995
miR−152	0.169	0.121	1.4	0.0412	0.995
miR−291b−3p	0.21	0.157	1.33	0.0463	0.995
miR−125a−3p	0.185	0.137	1.35	0.0486	0.995

Tests for Chromosome Over−representation

Chromosome	Age−upregulated Genes	Age−downregulated Genes
1	0.667	0.199
2	0.218	0.642
3	0.202	0.232
4	0.908	0.539
5	0.933	0.985
6	0.567	0.425
7	0.241	0.587
8	0.418	0.328
9	0.513	0.264
10	0.216	0.383
11	0.716	0.881
12	0.709	0.48
13	0.0416*	0.225
14	0.859	0.293
15	0.919	0.139
16	0.377	0.586
17	0.1	0.472
18	0.133	0.113
19	0.184	0.0719
X	0.217	0.117
Y	0.0423*	1.00

The table lists p−values generated from a test that evaluates whether there exists an over−abundance of identified genes with respect to a given chromosome. The null hypothesis assumes that the set of genes has been selected at random from those represented on the Affymetrix 430 2.0 array. A significant test indicates that a chromosome contains more of the identified genes than would be expected if the gene set had been chosen at random.

* = significant p−value, without multiple test adjustment

** = significant p−value, with multiple test adjustment

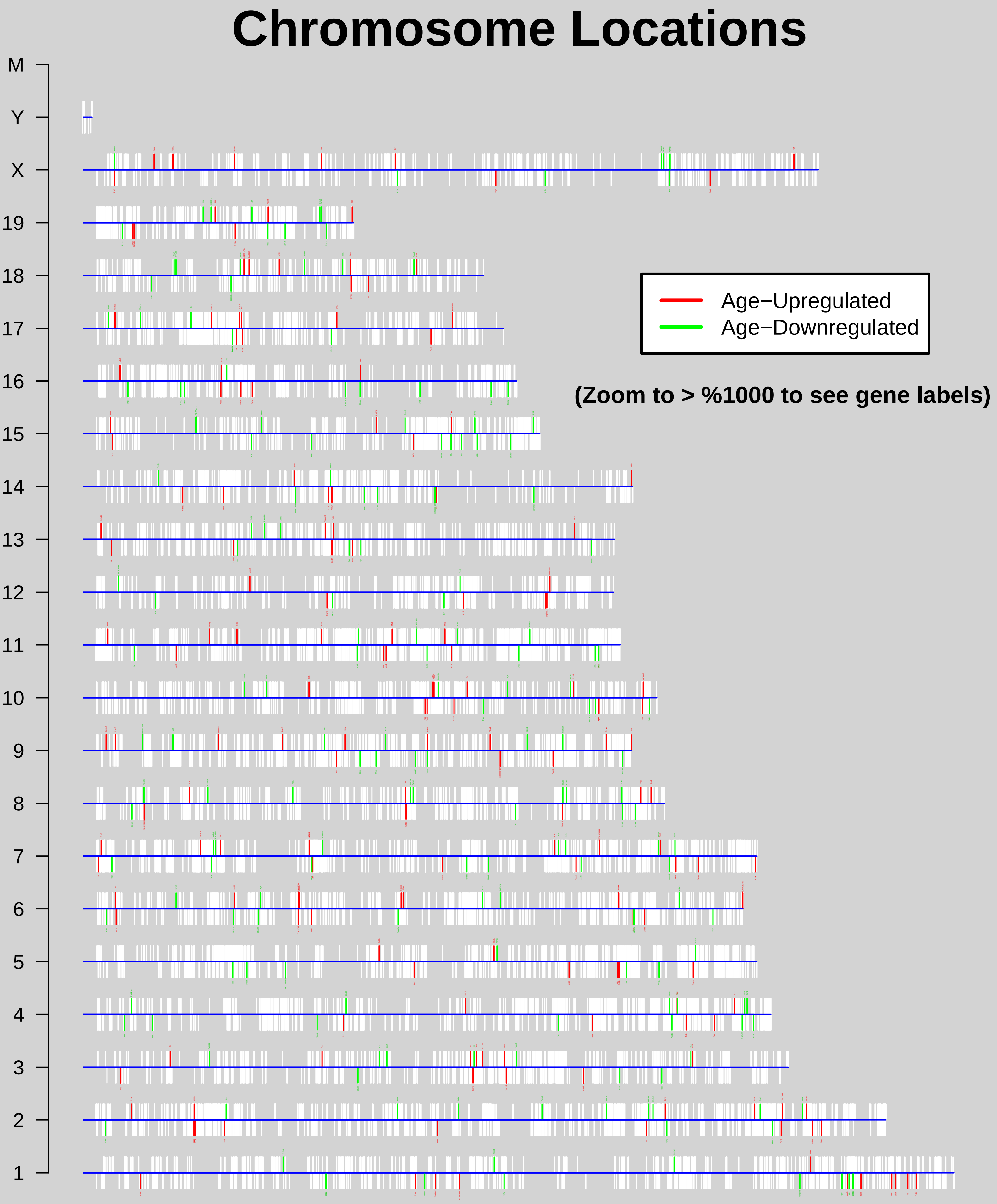
Chromosome Locations
M
Y
X
19
18
17
16
15
14
13
12
11
10
9
8
7
6
5
4
3
2
1
Age−Upregulated
Age−Downregulated
(Zoom to > %1000 to see gene labels)
