# Supplementary material for: Genes and gene expression modules associated with caloric restriction and aging in the laboratory mouse
Source: BMC Genomics. 2009 Dec 7;10:585. doi: 10.1186/1471-2164-10-585 (PMC2795771; doi:10.1186/1471-2164-10-585)

Additional File 9

Genes and Gene Expression Modules Associated with Caloric Restriction and Aging in the Laboratory Mouse

William R. Swindell
University of Michigan, Departments of Pathology and Geriatrics

---

Genes Regulated by Aging in Muscle

This file provides information on genes significantly influenced by aging in muscle. The first set of charts displays differential expression results of the 200 genes most strongly up regulated by aging in muscle, while the second set of charts displays differential expression results for the 200 genes most strongly down regulated by aging in muscle. Each row corresponds to an individual gene and each column corresponds to a separate experiment (see Additional File 1). Symbols are interpreted as follows:

- ● Gene is significantly up regulated by age ($P_u < 0.05$)
- ● Gene is significantly down regulated by age ($P_d < 0.05$)
- ▬ Gene is marginally up regulated by age ($0.05 < P_u < 0.10$)
- ▬ Gene is marginally down regulated by age ($0.05 < P_d < 0.10$)
- ▬ Non-significant age effect ($P_u > 0.10$ and $P_d > 0.10$)
- × No data (gene not represented in experiment or array annotation was limiting)
- ✳ Evidence conflicts but favors up regulation by age
- ✳ Evidence conflicts, but favors down regulation by age

The last two categories (✳ and ✳) indicate significant effects with conflicting evidence. This can arise if multiple transcripts associated with the same gene symbol yield opposite conclusions. Alternatively, a conflict may arise if $P_u < 0.05$ and also $P_d < 0.05$. Symbols shown in charts are based upon a comparison-wise type I error rate of 0.05. The final column in each chart lists meta-analysis p-values generated using Fisher's method, which have been adjusted using the Benjamini-Hochberg method to control the false discovery rate among all 21,327 genes.

The remainder of the file includes lists of over-represented gene ontology terms, over-represented KEGG pathways, and over-represented KEGG pathways defined based upon IP domain signatures (see Hahne et al. 2008, BMC Bioinformatics 9:3). Genes were also analyzed to determine if there existed an over-abundance of targets for certain microRNAs (see Betel et al. 2008, Nucleic Acids Res. 36: D149-153), and a list of associated microRNAs is provided based upon this analysis. Lastly, tests for over-representation of identified genes with respect to each chromosome were performed, and an idiogram mapping of identified genes to chromosomal locations is shown.

Contact: William R. Swindell, wswindel@umich.edu

↑ Age

Genes up regulated by Age

	msl1	msl9	msl20	msl21	P_u
Igk−V1	●	●	×	●	0.00214
Apod	●	●	×	●	0.00214
Hoxc4	●	●	×	●	0.00214
Chrna1	●	●	×	●	0.00293
Gadd45a	●	●	×	●	0.00293
C4b	●	●	×	●	0.00353
Amy1	●	●	×	●	0.00685
Lgals3	●	●	×	●	0.0095
Relb	●	●	×	●	0.0095
Hsp90aa1	●	●	●	●	0.0111
Kcnab1	●	●	×	●	0.0116
Cdkn1a	●	●	×	●	0.0145
Dab2	●	●	●	●	0.0145
Dapk1	●	●	×	×	0.0145
Fcgr2b	●	●	×	–	0.0145
Gprc5b	●	●	●	×	0.0145
Hpgd	●	–	×	●	0.0145
Lepr	●	●	×	●	0.0145
Mapt	*	–	●	●	0.0145
Rpl13	●	●	×	×	0.0145
Slc44a1	●	●	●	×	0.0145
LOC100047628	●	●	×	×	0.0145
Aldh1a1	●	●	●	●	0.0145
Btg3	●	●	×	●	0.0145
Ccl8	●	●	×	×	0.0145
Cpne2	●	●	×	×	0.0145
Ctss	●	●	×	●	0.0145
Efemp1	●	●	×	×	0.0145
Igj	●	●	×	×	0.0145
Uso1	●	●	●	×	0.0145

↑ Age

Genes up regulated by Age

	msl1	msl9	msl20	msl21	P_u
Eif5b	●	●	–	×	0.0156
Rasd2	●	●	●	×	0.0159
4933415F23Rik	●	●	×	×	0.0159
1190002H23Rik	●	●	×	×	0.0159
Angel2	* (red)	● (green)	–	●	0.0159
Ankrd1	●	●	×	×	0.0159
App	●	●	–	●	0.0159
Atp13a3	●	●	– (red)	×	0.0159
C80913	●	●	●	×	0.0159
Cadm1	●	●	×	×	0.0159
Cd53	●	●	×	●	0.0159
Clic4	●	●	–	●	0.0159
Eprs	* (green)	●	●	●	0.0159
Fst	●	●	×	–	0.0159
Hsp90ab1	●	● (green)	●	●	0.0159
Mt1	●	●	–	●	0.0159
Mt2	●	●	–	●	0.0159
Ncor1	●	●	●	● (green)	0.0159
Ndrg1	●	* (green)	–	●	0.0159
Npnt	●	●	– (red)	×	0.0159
Ppap2a	●	●	×	●	0.0159
Ppp2ca	●	●	–	●	0.0159
Prpf19	●	●	●	×	0.0159
Ptgs1	●	● (green)	×	●	0.0159
Runx1	●	● (green)	×	●	0.0159
Sema4a	●	●	–	●	0.0159
Sptlc2	●	–	×	●	0.0159
Trim30	●	●	×	–	0.0159
Ankrd32	●	●	– (red)	×	0.0159
Anxa11	●	●	–	●	0.0159

↑ Age

Genes up regulated by Age

	msl1	msl9	msl20	msl21	P_u
B230312A22Rik	● (red)	● (red)	×	×	0.0159
Cacng1	● (red)	● (red)	×	● (red)	0.0159
Irx3	● (red)	● (red)	– (red)	×	0.0159
Krt18	● (red)	● (red)	–	● (red)	0.0159
Mrc1	● (red)	–	×	● (red)	0.0159
Myh3	● (red)	● (red)	×	×	0.0159
Nbea	● (red)	● (red)	×	×	0.0159
Rasa3	● (red)	● (red)	● (green)	● (red)	0.0159
Rras	● (red)	–	×	● (red)	0.0159
Tuba1b	● (red)	– (red)	×	● (red)	0.0159
Ablim1	● (red)	● (red)	–	×	0.0167
H2−Aa	● (red)	● (red)	×	● (red)	0.0178
Clock	● (red)	● (red)	×	×	0.0178
Dpyd	● (red)	● (red)	×	×	0.0178
Igh−6	● (red)	● (red)	–	● (red)	0.0178
Mknk2	* (green)	● (red)	– (red)	–	0.018
Pmpcb	● (red)	– (green)	×	● (red)	0.018
Cma1	● (red)	● (red)	×	– (red)	0.018
Derl1	● (red)	● (red)	–	×	0.0189
Ankrd6	● (red)	● (green)	● (red)	×	0.0194
Akr1b8	● (red)	–	×	● (red)	0.0195
Gprasp1	● (red)	● (red)	– (red)	×	0.0197
Btf3l4	● (red)	● (red)	● (red)	×	0.0197
Rab2a	– (green)	● (red)	● (red)	● (red)	0.0197
Rnf187	● (red)	● (red)	–	×	0.0197
Sept7	● (red)	● (red)	–	● (red)	0.0197
Synpo	● (red)	● (red)	×	×	0.0197
Ugt1a6a	● (red)	● (red)	– (red)	×	0.0197
Zfp598	● (red)	● (red)	–	×	0.0197
Acsl6	● (red)	● (red)	×	×	0.02

↑ Age

Genes up regulated by Age

	msl1	msl9	msl20	msl21	P_u
Mib1	●	–	●	×	0.02
Rac1	–	●	●	●	0.0203
Acadm	●	– (red)	●	–	0.0203
Ccdc97	●	●	– (red)	×	0.0212
Dusp3	*	●	×	×	0.0212
Ahcyl2	*	●	–	×	0.0221
Htatip2	●	●	×	×	0.0221
Kpnb1	●	● (green)	– (red)	●	0.0221
March5	●	●	–	×	0.0221
Sav1	●	●	×	×	0.0221
Sln	●	●	×	×	0.0221
Atp6ap1	●	– (red)	×	●	0.0227
S100a11	●	–	×	●	0.0242
Fryl	●	●	–	×	0.0244
Rab4a	●	●	×	– (red)	0.0252
Slc25a28	●	●	×	–	0.0252
Rrad	●	●	×	×	0.0255
Ccl9	●	●	×	●	0.0257
Slc10a3	●	–	×	●	0.0263
Aof2	●	●	–	×	0.0266
Sim2	●	●	×	–	0.0266
Dhx32	●	●	×	×	0.0268
Rpl5	●	●	×	●	0.0268
Lamp1	●	●	–	×	0.0268
Mdn1	–	●	●	×	0.0272
Bmi1	●	●	–	●	0.0273
Copb1	●	●	×	×	0.0275
4933439C20Rik	●	●	–	×	0.0275
Crk	●	●	– (red)	×	0.0275
Cstb	●	– (red)	–	●	0.0275

↑ Age

Genes up regulated by Age

	msl1	msl9	msl20	msl21	P_u
Fut8	●	●	×	×	0.0275
Spint2	●	●	−	×	0.0275
Csf2rb2	●	●	×	●	0.0275
AW049604	●	●	×	×	0.0276
Gpnmb	●	●	×	×	0.0276
Hes6	●	−	×	●	0.0276
1420172_at	●	●	×	×	0.0276
Tmem176a	●	●	×	×	0.0278
Tbcb	●	●	−	●	0.0286
Cd2ap	●	− (green)	●	×	0.0295
H2−D1	●	− (red)	−	●	0.0295
Rpl36a	●	●	×	×	0.0295
Tubb5	●	−	×	●	0.0295
Gltp	●	●	−	×	0.0295
Pdcd2	●	●	×	×	0.0299
Cpa3	●	− (red)	×	●	0.0299
Figf	●	●	×	●	0.03
Npepps	●	●	−	●	0.03
Arl5a	*	●	● (green)	×	0.03
Ppp1r3b	●	×	●	×	0.0303
Lyz2	●	−	− (green)	●	0.0308
Lilrb4	●	●	− (red)	−	0.0309
Klhl5	●	●	−	×	0.031
Atp6ap2	●	●	−	×	0.0312
Cbx4	●	●	×	×	0.0312
Mnt	●	● (green)	×	●	0.0312
Gns	●	●	×	×	0.0314
Kifap3	● (green)	●	−	●	0.0314
Usp21	●	−	−	●	0.0314
Sfrs17b	●	−	●	×	0.0318

↑ Age

Genes up regulated by Age

	msl1	msl9	msl20	msl21	P_u
Usp10	●	–	–	●	0.0325
Dnajc5	●	●	–	×	0.0328
1434569_at	●	●	×	×	0.0328
Tnnt2	●	–	×	●	0.033
Adamts12	●	×	●	×	0.0337
Sh3bgrl	●	●	–	×	0.0341
Tmem205	●	–	●	×	0.0341
1200016E24Rik	*	●	×	×	0.0343
Cp	●	●	–	–	0.0346
Lmo2	●	–	×	●	0.0346
Frap1	●	●	–	●	0.0348
Sash1	●	●	×	×	0.0348
Sfxn1	●	–	●	×	0.0348
Wnt5b	●	–	●	●	0.0348
Flywch1	*	●	×	×	0.0348
Slc4a3	●	–	–	●	0.0349
Eml5	●	●	×	×	0.0349
D730040F13Rik	●	●	×	×	0.0353
G0s2	●	●	×	–	0.0353
Aebp2	●	●	×	×	0.0354
Ankrd17	*	●	–	×	0.0354
Anxa7	–	●	–	●	0.0354
Arf6	●	●	×	×	0.0354
Asah3l	●	●	×	×	0.0354
Dag1	*	●	●	●	0.0354
Samd10	●	●	×	×	0.0354
Syne1	*	●	×	×	0.0354
Ddx3x	–	●	●	●	0.0354
Sertad2	●	●	–	×	0.0356
Ss18	●	●	–	●	0.0356

↑ Age

Genes up regulated by Age

	msl1	msl9	msl20	msl21	P_u
C78339	●	●	–	×	0.0363
Cugbp1	*	●	–	–	0.0364
Sept4	●	●	×	●	0.0364
Tex261	●	●	–	–	0.0364
Riok3	●	●	●	×	0.0372
Cd209d	●	●	×	×	0.0372
Abcd3	●	–	–	●	0.0373
Psat1	●	–	–	●	0.0376
Rpsa	●	–	–	●	0.0376
Sh3d19	●	*	–	×	0.0376
Rpl7a	●	–	×	●	0.0376
Kctd2	●	●	–	×	0.0377
Gcap14	*	●	–	×	0.0386
1110008F13Rik	●	●	×	×	0.0389
Btbd1	●	●	–	×	0.0389
Commd9	–	●	●	×	0.0389
Ktn1	–	●	–	●	0.0389
Lin7c	●	●	●	×	0.0389
Nxn	●	●	–	×	0.0389
Rab21	●	●	–	●	0.0389

↓ Age

Genes down regulated by Age

	msl1	msl9	msl20	msl21	P_d
Col1a2	●	●	●	●	0.00826
Cacng6	●	●	×	×	0.0241
Ccdc50	●	●	×	×	0.0241
Gpd1	●	●	×	×	0.0241
Itgb6	●	●	×	×	0.0241
Lman1	●	●	–	×	0.0241
Ndufc1	●	●	●	×	0.0241
Nfs1	●	●	×	×	0.0241
Otub1	●	●	×	×	0.0241
Rpl7l1	●	●	–	×	0.0241
Scd2	●	●	–	–	0.0241
Serpinf1	●	●	–	●	0.0241
Syt11	●	*	–	●	0.0241
Trappc5	●	●	×	–	0.0241
Ube2l3	●	●	–	●	0.0241
Zfp191	*	●	×	×	0.0241
Pin1	●	●	–	●	0.0241
Usp5	●	●	–	●	0.0241
Pvr	●	●	–	●	0.0241
Stom	●	●	×	●	0.0241
2810405K02Rik	●	●	×	×	0.0242
Ap2m1	●	●	●	●	0.0242
1200011I18Rik	*	●	×	×	0.0308
Abcb8	●	●	–	×	0.0308
Acvrl1	●	●	×	–	0.0308
Angptl1	●	●	×	×	0.0308
Art5	●	●	×	●	0.0308
Cacnb1	●	●	×	×	0.0308
Clcn3	●	●	–	–	0.0308
Cttn	*	●	–	●	0.0308

↓ Age

Genes down regulated by Age

	msl1	msl9	msl20	msl21	P_d
D4Wsu53e	●	●	●	×	0.0308
Dnm2	●	●	– (green)	×	0.0308
Epas1	●	●	×	×	0.0308
Etv1	●	–	×	●	0.0308
Fbln1	●	●	×	●	0.0308
Fbxw11	●	*	–	×	0.0308
Gys1	●	●	×	–	0.0308
Hbs1l	●	●	– (red)	–	0.0308
Hr	●	– (green)	×	●	0.0308
Ibtk	●	●	–	×	0.0308
Ifi35	–	●	×	●	0.0308
Il6st	●	●	– (red)	–	0.0308
Kcnj11	●	●	×	●	0.0308
LOC67527	●	●	×	×	0.0308
Map2k7	*	●	×	×	0.0308
Mat2a	●	● (red)	●	×	0.0308
Mbp	●	*	–	–	0.0308
Meg3	●	●	–	×	0.0308
Myl6	● (red)	●	●	×	0.0308
Narf	●	●	×	×	0.0308
Pcyt1a	●	●	–	×	0.0308
Pde4dip	●	●	– (green)	×	0.0308
Pdgfra	●	●	●	–	0.0308
Pdlim5	●	*	–	×	0.0308
Pfkfb3	●	●	–	×	0.0308
Pfkl	●	●	–	●	0.0308
Pld2	●	●	–	×	0.0308
Polr3f	●	●	×	×	0.0308
Prpf39	●	●	×	×	0.0308
Prrx1	●	●	×	● (red)	0.0308

↓ Age

Genes down regulated by Age

	msl1	msl9	msl20	msl21	P_d
Sec61a1	● (green)	● (green)	–	● (red)	0.0308
Sema6c	● (green)	● (green)	×	×	0.0308
Slc2a4	● (green)	● (green)	● (green)	● (green)	0.0308
Slc44a2	* (green)	● (green)	–	×	0.0308
Slc9a3r2	● (green)	● (green)	×	×	0.0308
Smad3	● (green)	● (green)	–	×	0.0308
Sox6	● (green)	● (green)	–	–	0.0308
Sparc	–	● (green)	● (green)	– (green)	0.0308
Spnb2	* (green)	● (green)	– (green)	● (red)	0.0308
Tfrc	● (green)	● (green)	–	● (red)	0.0308
Thrap3	● (green)	● (green)	–	×	0.0308
Timp3	● (green)	● (green)	–	×	0.0308
Tmem109	* (green)	● (green)	–	×	0.0308
Tmem183a	● (green)	● (green)	–	×	0.0308
Tmem38a	● (green)	● (green)	×	×	0.0308
Tmod3	● (green)	● (green)	– (green)	×	0.0308
Trip12	* (red)	● (green)	● (green)	×	0.0308
Twf2	● (green)	● (green)	×	×	0.0308
Ubr7	● (green)	● (green)	–	×	0.0308
Wwp1	* (green)	● (green)	–	×	0.0308
Zc3h11a	● (green)	* (green)	–	×	0.0308
Zfp346	● (green)	● (green)	×	×	0.0308
100039840	● (green)	– (green)	×	● (green)	0.0308
Actc1	–	● (green)	×	● (green)	0.0308
Asna1	● (green)	● (green)	×	×	0.0308
Bnip1	● (green)	● (green)	– (green)	×	0.0308
H19	–	● (green)	● (green)	–	0.0308
Magt1	● (green)	● (green)	–	×	0.0308
Nrbp1	● (green)	● (green)	● (green)	×	0.0308
Bcas3	* (green)	● (green)	– (red)	×	0.0308

↓ Age

Genes down regulated by Age

	msl1	msl9	msl20	msl21	P_d
Ak3	●	●	– (green)	×	0.0312
Cux1	*	●	–	–	0.0312
Hspb7	●	●	×	×	0.0312
Ogfr	●	●	–	×	0.0312
Hdlbp	*	●	– (red)	×	0.0315
6330578E17Rik	*	●	–	×	0.0324
Sspn	●	●	×	–	0.0325
Tapbp	●	●	–	×	0.0325
Cd2bp2	●	●	–	×	0.0328
Gosr2	●	●	– (red)	×	0.0328
Klc1	●	●	–	● (red)	0.0328
2610507B11Rik	●	●	–	–	0.0328
Ak1	●	●	×	–	0.0328
Mtg1	●	●	–	×	0.0328
1427798_x_at	●	●	×	×	0.0337
Sdf4	●	●	–	×	0.0341
Asb15	●	●	×	×	0.0349
Camk2a	●	●	×	● (red)	0.035
Snurf	●	●	×	×	0.035
Tug1	●	●	×	● (red)	0.0357
Pmm2	●	●	–	×	0.0362
Sod2	●	●	●	–	0.0362
Scn4a	●	●	×	×	0.0365
1427797_s_at	●	●	×	×	0.0365
Pdpk1	*	–	–	●	0.0373
Wdr42a	●	●	– (green)	×	0.0373
Zfp294	●	●	–	×	0.0373
Cenpb	●	●	–	×	0.0373
Cuedc1	●	●	×	×	0.0374
Eif4g1	●	–	●	×	0.0374

↓ Age

Genes down regulated by Age

	msl1	msl9	msl20	msl21	P_d
Ksr1	🟢	🟢	×	🔴	0.0374
Rnf114	🟢	* (green)	–	×	0.0374
Steap3	🟢	🟢	×	×	0.0374
2700078K21Rik	🟢	🟢	×	×	0.0374
Homer1	🟢	🟢	×	×	0.0375
Metapl1	🟢	🟢	×	×	0.0375
Pknox2	🟢	🟢	–	×	0.0379
Angptl2	🟢	🟢	×	×	0.0382
Bcl6	🟢	🟢	×	🔴	0.0382
Ppp1r11	🟢	🟢	×	×	0.0382
Vps36	🟢	🟢	×	×	0.0392
Cep68	🟢	🟢	🔴	×	0.0399
Etv5	🟢	🟢	–	×	0.0399
Snai3	🟢	🟢	×	×	0.0402
Atrx	🟢	🟢	🔴	×	0.0407
Gli1	🟢	🟢	×	×	0.0407
Tor1b	🟢	🟢	×	×	0.0408
Ubac2	🟢	🟢	–	×	0.0408
Akt3	🟢	🟢	×	×	0.041
Srp72	🟢	×	🟢	×	0.041
Mmab	🟢	🟢	×	×	0.042
P4ha1	🟢	🟢	–	– (green)	0.042
Cops7a	🟢	🟢	–	×	0.042
Zfp106	* (red)	🟢	🔴	×	0.042
Abcc5	🟢	🟢	×	🔴	0.0431
Acp2	🟢	🟢	×	–	0.0431
Atp6v0b	🟢	🟢	–	×	0.0431
Baz1b	🟢	* (red)	×	×	0.0431
Cacna2d1	🟢	🟢	🟢	×	0.0431
Dnajb5	🟢	🟢	×	×	0.0431

↓ Age

Genes down regulated by Age

	msl1	msl9	msl20	msl21	P_d
Elk1	● (green)	● (green)	×	– (green)	0.0431
Fert2	● (green)	● (green)	×	– (green)	0.0431
Ikbkg	● (green)	● (green)	×	×	0.0431
Mtm1	● (green)	● (green)	×	×	0.0431
Phkg1	● (green)	● (green)	×	–	0.0431
Rad23a	● (green)	● (green)	×	● (red)	0.0431
Slc31a2	● (green)	● (green)	–	×	0.0431
Trim26	* (green)	● (green)	×	×	0.0431
Ube2h	● (green)	● (green)	×	×	0.0431
Xrcc2	● (green)	● (green)	×	×	0.0431
Ptprr	● (green)	● (green)	×	×	0.0431
Gpam	● (green)	● (green)	–	– (green)	0.0432
Col3a1	–	● (green)	–	● (green)	0.0433
Cnot4	● (green)	* (red)	×	×	0.0446
Sgcb	● (green)	● (green)	×	×	0.0446
Dhdds	● (green)	● (green)	–	×	0.0449
Slc25a19	–	● (green)	–	● (green)	0.0449
Uqcc	● (green)	● (green)	×	×	0.0449
Ppp3r1	● (green)	● (red)	● (green)	×	0.0462
Ykt6	● (green)	● (green)	● (red)	×	0.0463
Ddx19b	● (green)	● (green)	×	×	0.0465
Fli1	● (green)	● (green)	×	– (green)	0.0465
Slc41a3	● (green)	● (green)	–	×	0.0465
Ppapdc3	● (green)	● (green)	×	×	0.0465
Klk1	● (green)	– (green)	×	● (green)	0.0476
Btbd3	● (green)	● (green)	×	×	0.048
Jam2	● (green)	● (green)	×	×	0.048
Sar1a	● (green)	● (green)	● (red)	● (red)	0.048
Sel1l	● (green)	● (green)	×	×	0.048
Eif2s3x	● (green)	● (green)	–	● (red)	0.0485

↓ Age

Genes down regulated by Age

	msl1	msl9	msl20	msl21	P_d
Rab8a	●	●	–	×	0.0485
Add1	*	●	–	×	0.049
Mbtps1	*	●	–	×	0.049
Syn3	●	●	×	×	0.0494
Ing4	●	●	–	×	0.0496
AU040320	●	●	–	×	0.0499
Glo1	●	●	–	×	0.0499
Grwd1	●	●	–	×	0.0499
Klhdc3	●	●	–	×	0.0499
Mrps5	●	●	×	×	0.0499
Oaz2	● (red)	●	–	×	0.0499
Slk	●	●	–	×	0.0499
Icam4	●	●	×	×	0.0499
Mlf1	●	●	×	×	0.0499
Sytl2	●	●	×	×	0.0499
Slc37a3	●	●	–	×	0.0501
Csrp1	●	●	– (red)	●	0.0509
Sars	●	●	● (red)	– (red)	0.0509
Tnxb	–	●	×	●	0.0516
St3gal5	●	●	×	–	0.0517

↑Age

Overrepresented Biological Processes

GO Term	P−Value
antigen processing and presentation	9.28e−05
in utero embryonic development	0.000126
heterophilic cell adhesion	0.000433
membrane organization and biogenesis	0.000862
positive regulation of phagocytosis	0.0011
lysosomal lumen acidification	0.00143
nitric oxide mediated signal transduction	0.00143
system development	0.00146
vasculature development	0.00168
protein polymerization	0.00204
cell differentiation	0.00227
cellular ion homeostasis	0.00273
positive regulation of developmental process	0.00304
antigen processing and presentation of peptide antigen via MHC class I	0.00326
regulation of cellular component organization and biogenesis	0.00334
detoxification of copper ion	0.00418
tumor necrosis factor−mediated signaling pathway	0.00418
pH reduction	0.00418
anatomical structure morphogenesis	0.0042
protein complex assembly	0.0043
positive regulation of immune system process	0.00481
cellular macromolecular complex assembly	0.00541
humoral immune response	0.00664
leukocyte adhesion	0.00709
ribosomal protein import into nucleus	0.00816
neuron remodeling	0.00816
positive regulation of tumor necrosis factor biosynthetic process	0.00816
regulation of intracellular pH	0.00816
response to osmotic stress	0.00919
regulation of translation	0.00946

↑Age

Overrepresented Biological Processes

GO Term	P−Value
positive regulation of biological process	0.0104
endocytosis	0.0123
positive regulation of cellular metabolic process	0.0126
positive regulation of endocytosis	0.0131
platelet−derived growth factor receptor signaling pathway	0.0133
positive regulation of macromolecule metabolic process	0.0133
mast cell activation	0.0144
negative regulation of RNA metabolic process	0.0147
negative regulation of neuron differentiation	0.0157
negative regulation of cell cycle	0.016
cellular homeostasis	0.0171
protein import into nucleus, docking	0.0175
locomotion	0.0175
developmental maturation	0.018
macromolecule localization	0.0185
microtubule−based process	0.0185
dendrite development	0.0186
macromolecular complex subunit organization	0.0192
cellular zinc ion homeostasis	0.0194
regulation of epidermal growth factor receptor activity	0.0194
oligosaccharide biosynthetic process	0.0194
response to retinoic acid	0.0194
response to vitamin	0.0194
multicellular organismal process	0.0196
cation homeostasis	0.0202
negative regulation of macromolecule biosynthetic process	0.0204
heart morphogenesis	0.0224
liver development	0.0228
regulation of angiogenesis	0.0228
embryonic development ending in birth or egg hatching	0.0231

↑ Age

Overrepresented Biological Processes

GO Term	P−Value
lung development	0.0231
establishment of protein localization	0.0245
positive regulation of lymphocyte activation	0.0247
positive regulation of B cell proliferation	0.0247
negative regulation of biological process	0.0247
chemical homeostasis	0.0255
regulation of protein metabolic process	0.0255
leukocyte activation	0.0257
intracellular protein transport	0.0261
nuclear−transcribed mRNA catabolic process, deadenylation−dependent decay	0.0265
heart process	0.0268
erythrocyte homeostasis	0.0268
regulation of B cell activation	0.0268
positive regulation of cell activation	0.028
response to wounding	0.0282
vasculogenesis	0.0284
leukocyte activation during immune response	0.0288
antigen processing and presentation of exogenous peptide antigen	0.0288
cell cycle arrest	0.0292
monovalent inorganic cation transport	0.0306
blood vessel morphogenesis	0.0309
positive regulation of biosynthetic process	0.0329
phagocytosis, recognition	0.0343
acute inflammatory response to antigenic stimulus	0.0344
regulation of acute inflammatory response	0.0344
positive regulation of inflammatory response to antigenic stimulus	0.0344
regulation of hypersensitivity	0.0344
cellular monovalent inorganic cation homeostasis	0.0344
mRNA 3'−end processing	0.0344
striated muscle cell differentiation	0.0349

↑Age

Overrepresented Biological Processes

GO Term	P−Value
transport	0.0357
regulation of immune response	0.0366
glycosaminoglycan metabolic process	0.0381
negative regulation of cell size	0.0381
cellular protein metabolic process	0.0385
anatomical structure formation	0.0416
regulation of lymphocyte mediated immunity	0.0421
regulation of heart contraction	0.0421
negative regulation of B cell proliferation	0.0432
macrophage activation	0.0432
RNA polyadenylation	0.0432
protein targeting to ER	0.0432
negative regulation of transcription from RNA polymerase II promoter	0.044
regulation of defense response	0.0461
ATP biosynthetic process	0.0487
pancreas development	0.0487
myoblast maturation	0.0487
biological regulation	0.0493
positive regulation of transcription	0.0497

↑ Age

Overrepresented Cell Components

GO Term	P−Value
cytoplasm	5.89e−06
intracellular	0.0025
microtubule cytoskeleton	0.00323
nicotinic acetylcholine−gated receptor−channel complex	0.00496
endomembrane system	0.00674
axon	0.008
protein complex	0.00934
plasma membrane	0.00941
external side of plasma membrane	0.00978
MHC class I protein complex	0.0135
insoluble fraction	0.0152
intracellular organelle	0.0181
adherens junction	0.023
sarcolemma	0.0263
microvillus	0.0271
Golgi apparatus part	0.032
sarcomere	0.0325
cytoplasmic part	0.0349
cytoplasmic vesicle	0.0416
tight junction	0.047

↑Age

Overrepresented Molecular Functions

GO Term	P−Value
GTP binding	0.000818
guanyl nucleotide binding	0.00106
mannose binding	0.00131
protease binding	0.00133
protein complex binding	0.00622
antigen binding	0.0064
protein transporter activity	0.00656
transcription factor binding	0.0069
retinal dehydrogenase activity	0.00759
3−chloroallyl aldehyde dehydrogenase activity	0.00759
UDP−galactose:beta−N−acetylglucosamine beta−1,3−galactosyltransferase activity	0.0124
IgG binding	0.0124
nicotinic acetylcholine−activated cation−selective channel activity	0.0158
unfolded protein binding	0.0172
acetylcholine binding	0.0181
ubiquitin thiolesterase activity	0.0187
GTPase activity	0.0191
small conjugating protein−specific protease activity	0.0217
protein binding	0.0219
glucuronosyltransferase activity	0.0224
histone acetyltransferase activity	0.0262
kinesin binding	0.0322
hydrolase activity, acting on acid anhydrides, in phosphorus−containing anhydrides	0.033
voltage−gated potassium channel activity	0.0355
voltage−gated ion channel activity	0.0394
protein kinase activator activity	0.0404
gated channel activity	0.0447
pattern binding	0.0461
structural constituent of ribosome	0.0465

↓Age

Overrepresented Biological Processes

GO Term	P−Value
cellular localization	0.00182
vesicle−mediated transport	0.00248
lung development	0.00294
glycogen biosynthetic process	0.00712
glucan metabolic process	0.0082
di−, tri−valent inorganic cation transport	0.00909
erythrocyte development	0.0103
energy reserve metabolic process	0.0105
regulation of calcium ion transport	0.012
generation of precursor metabolites and energy	0.013
establishment of protein localization	0.0135
intracellular protein transport	0.0138
protein targeting to membrane	0.0159
ion transport	0.016
sulfur amino acid metabolic process	0.018
response to gamma radiation	0.018
ER to Golgi vesicle−mediated transport	0.0203
cellular polysaccharide biosynthetic process	0.0203
erythrocyte homeostasis	0.0208
establishment of localization	0.0218
macromolecule localization	0.0224
intracellular transport	0.0226
amino acid derivative metabolic process	0.0232
polysaccharide metabolic process	0.0246
transmembrane receptor protein tyrosine kinase signaling pathway	0.0246
metal ion transport	0.0294
post−translational protein modification	0.0298
negative regulation of actin filament depolymerization	0.0302
muscle development	0.0313
nucleotide−excision repair	0.033

↓Age

Overrepresented Biological Processes

GO Term	P−Value
double−strand break repair	0.033
iron ion homeostasis	0.0418
monosaccharide biosynthetic process	0.0449
ATP metabolic process	0.0481
negative regulation of macromolecule metabolic process	0.0484

↓Age

Overrepresented Cell Components

GO Term	P−Value
sarcoplasmic reticulum	1.38e−05
intracellular	0.000133
intracellular organelle	0.000987
membrane−bounded organelle	0.00134
cytoplasmic membrane−bounded vesicle	0.00199
cytoplasm	0.00205
vesicle	0.00617
T−tubule	0.00907
I band	0.0119
perinuclear region of cytoplasm	0.0119
myofibril	0.0128
basement membrane	0.022
collagen	0.0222
cytoplasmic part	0.0263
Golgi apparatus part	0.0273
cell	0.028
endoplasmic reticulum	0.0295
plasma membrane part	0.0368
late endosome	0.0428

↓Age

Overrepresented Molecular Functions

GO Term	P−Value
purine ribonucleotide binding	0.000144
ATP binding	0.000288
adenyl nucleotide binding	0.000353
nucleotide binding	0.000774
copper ion binding	0.00294
adenylate kinase activity	0.00411
transcription activator activity	0.00867
cobalt ion binding	0.00937
cation transmembrane transporter activity	0.0102
protein kinase binding	0.0125
voltage−gated ion channel activity	0.0172
transferase activity, transferring phosphorus−containing groups	0.0198
carbohydrate kinase activity	0.0229
ubiquitin−protein ligase activity	0.0351
substrate−specific transmembrane transporter activity	0.0352
extracellular matrix structural constituent	0.0355
transition metal ion transmembrane transporter activity	0.0355
protein kinase activity	0.0378
P−P−bond−hydrolysis−driven transmembrane transporter activity	0.0397
calcium channel activity	0.042
calmodulin binding	0.0459

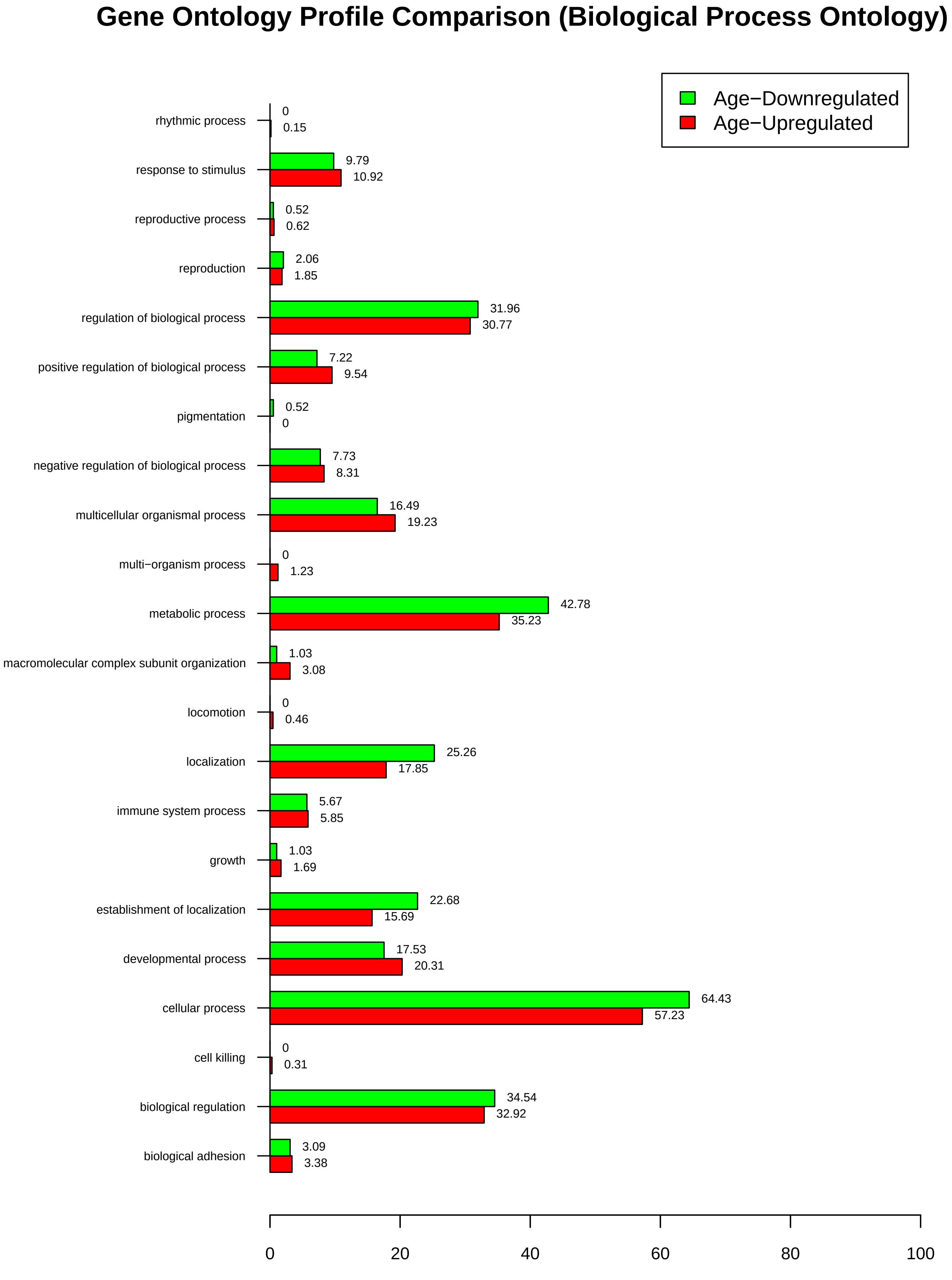

Gene Ontology Profile Comparison (Biological Process Ontology)
Age−Downregulated
Age−Upregulated
rhythmic process
0
0.15
response to stimulus
9.79
10.92
reproductive process
0.52
0.62
reproduction
2.06
1.85
regulation of biological process
31.96
30.77
positive regulation of biological process
7.22
9.54
pigmentation
0.52
0
negative regulation of biological process
7.73
8.31
multicellular organismal process
16.49
19.23
multi−organism process
0
1.23
metabolic process
42.78
35.23
macromolecular complex subunit organization
1.03
3.08
locomotion
0
0.46
localization
25.26
17.85
immune system process
5.67
5.85
growth
1.03
1.69
establishment of localization
22.68
15.69
developmental process
17.53
20.31
cellular process
64.43
57.23
cell killing
0
0.31
biological regulation
34.54
32.92
biological adhesion
3.09
3.38
0
20
40
60
80
100

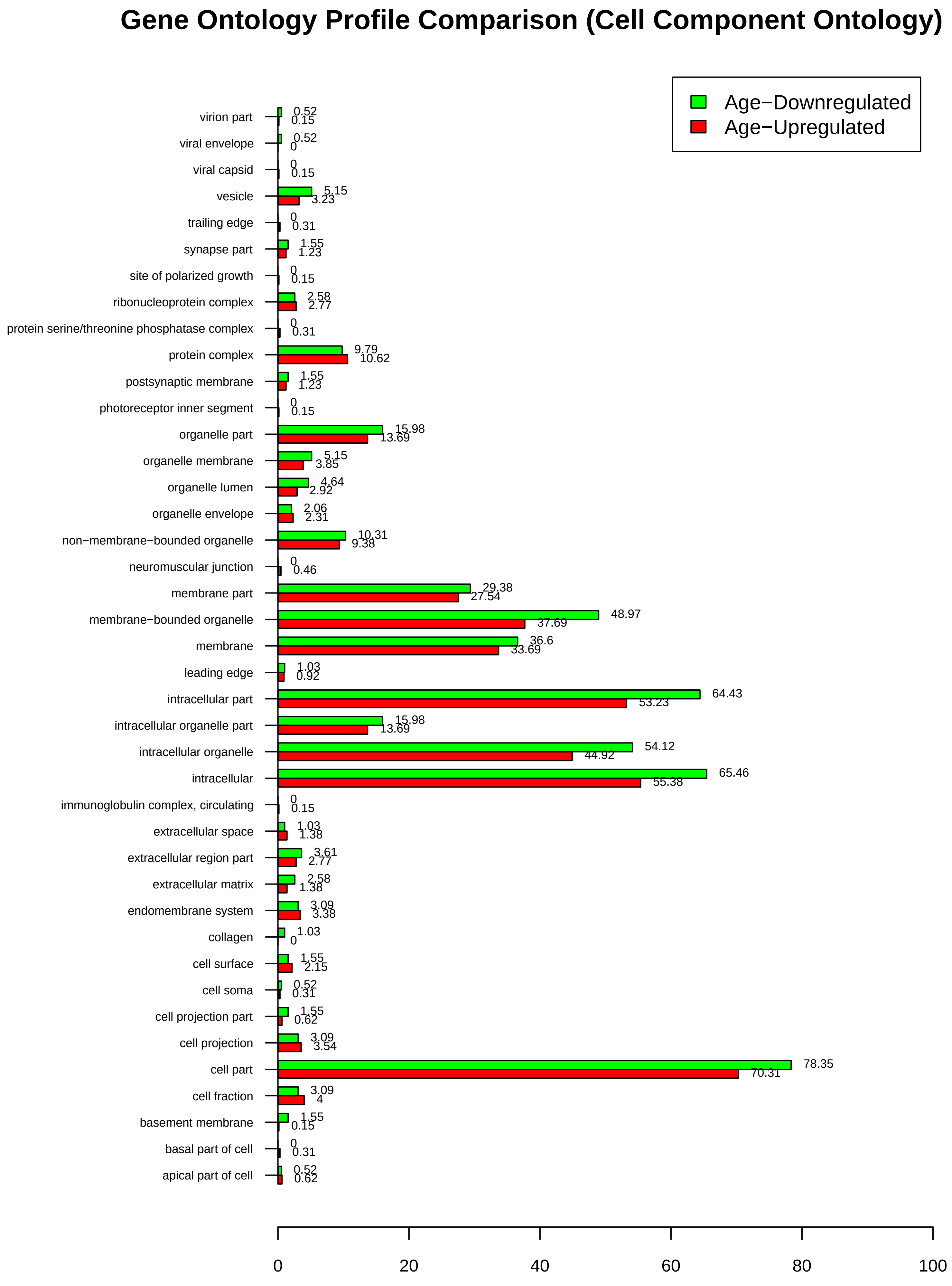
Gene Ontology Profile Comparison (Cell Component Ontology)
Age−Downregulated
Age−Upregulated
virion part 0.52 0.15
viral envelope 0.52 0
viral capsid 0 0.15
vesicle 5.15 3.23
trailing edge 0 0.31
synapse part 1.55 1.23
site of polarized growth 0 0.15
ribonucleoprotein complex 2.58 2.77
protein serine/threonine phosphatase complex 0 0.31
protein complex 9.79 10.62
postsynaptic membrane 1.55 1.23
photoreceptor inner segment 0 0.15
organelle part 15.98 13.69
organelle membrane 5.15 3.85
organelle lumen 4.64 2.92
organelle envelope 2.06 2.31
non−membrane−bounded organelle 10.31 9.38
neuromuscular junction 0 0.46
membrane part 29.38 27.54
membrane−bounded organelle 48.97 37.69
membrane 36.6 33.69
leading edge 1.03 0.92
intracellular part 64.43 53.23
intracellular organelle part 15.98 13.69
intracellular organelle 54.12 44.92
intracellular 65.46 55.38
immunoglobulin complex, circulating 0 0.15
extracellular space 1.03 1.38
extracellular region part 3.61 2.77
extracellular matrix 2.58 1.38
endomembrane system 3.09 3.38
collagen 1.03 0
cell surface 1.55 2.15
cell soma 0.52 0.31
cell projection part 1.55 0.62
cell projection 3.09 3.54
cell part 78.35 70.31
cell fraction 3.09 4
basement membrane 1.55 0.15
basal part of cell 0 0.31
apical part of cell 0.52 0.62
0
20
40
60
80
100

Gene Ontology Profile Comparison (Molecular Function Ontology)

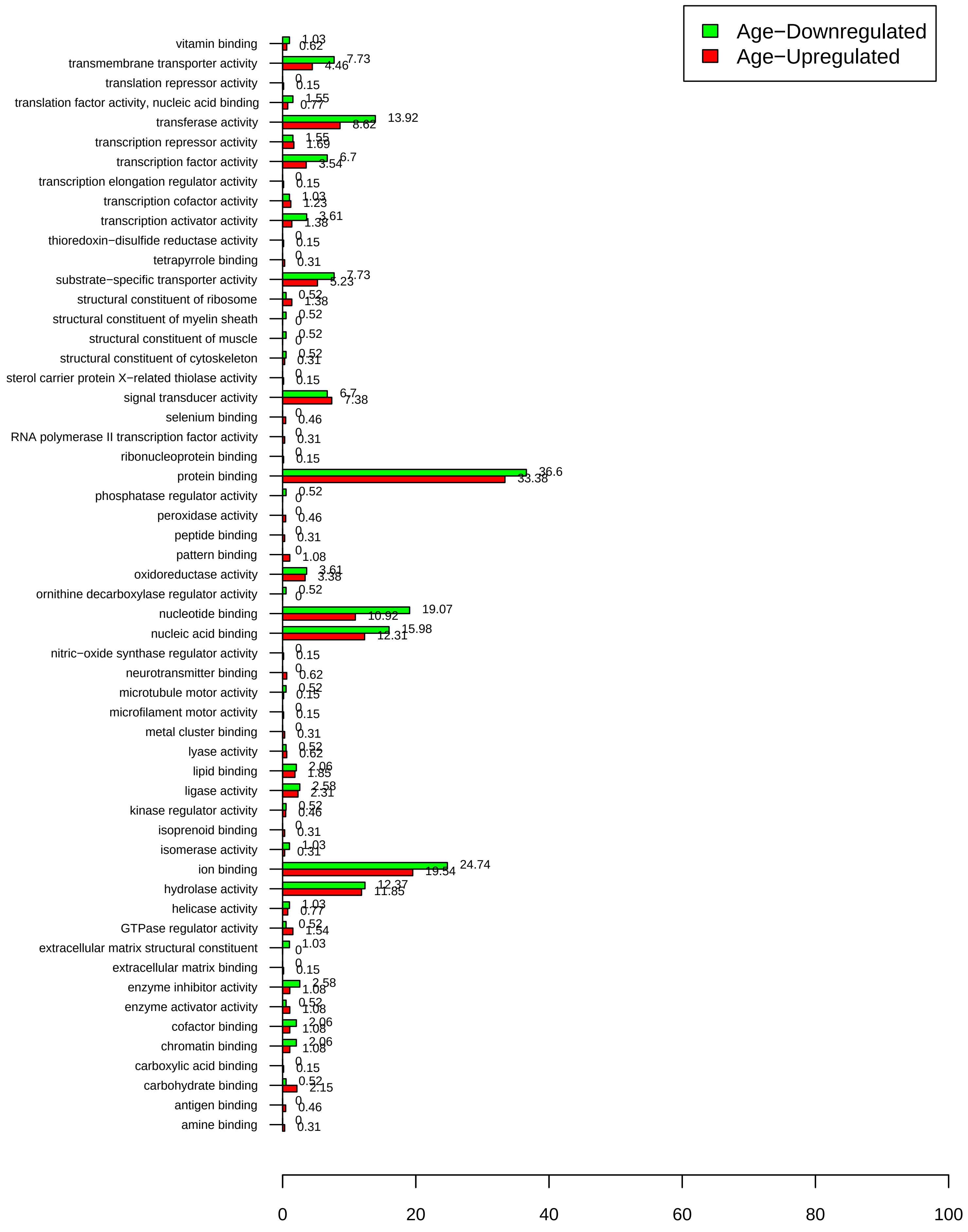

↑Age

Overrepresented KEGG Pathways

GO Term	P−Value
Antigen processing and presentation	0.0167
Ribosome	0.0177
Retinol metabolism	0.0193
Pentose and glucuronate interconversions	0.0241
Complement and coagulation cascades	0.0355
Porphyrin and chlorophyll metabolism	0.0421
Renin−angiotensin system	0.0472

↓Age

Overrepresented KEGG Pathways

GO Term	P−Value
Insulin signaling pathway	0.00624
MAPK signaling pathway	0.00805
Glycerophospholipid metabolism	0.0173
Dentatorubropallidoluysian atrophy (DRPLA)	0.0244
Focal adhesion	0.0335
Fructose and mannose metabolism	0.0371

↑Age

Overrepresented KEGG Pathways

(Based on InterPro Domain Signatures)

GO Term	P−Value
Complement and coagulation cascades	0.0036
Porphyrin and chlorophyll metabolism	0.0061
Bladder cancer	0.0161
Tight junction	0.0273
mTOR signaling pathway	0.0325
Adherens junction	0.0369
Melanoma	0.0407

↓Age

Overrepresented KEGG Pathways

(Based on InterPro Domain Signatures)

GO Term	P−Value
Glycerophospholipid metabolism	5e−04
Adherens junction	0.0023
Thiamine metabolism	0.0031
Insulin signaling pathway	0.0063
MAPK signaling pathway	0.0071
Ether lipid metabolism	0.0072
Tight junction	0.0097
Type II diabetes mellitus	0.0143
Renal cell carcinoma	0.0188
Chronic myeloid leukemia	0.0191
Cell cycle	0.0221
Dentatorubropallidoluysian atrophy (DRPLA)	0.0221
Basal transcription factors	0.0248
B cell receptor signaling pathway	0.0273
Ubiquitin mediated proteolysis	0.0284
Huntington's disease	0.029
mTOR signaling pathway	0.0336
Ribosome	0.0342
Aminophosphonate metabolism	0.0351
Fructose and mannose metabolism	0.0367
Apoptosis	0.0391
Pentose phosphate pathway	0.0411
Small cell lung cancer	0.0435
Adipocytokine signaling pathway	0.0446
T cell receptor signaling pathway	0.0457

↑Age

Abundance of miRNA Targets

miRNA	Freq(Obs)	Freq(Exp)	Obs/Exp	P−value	P−Value(Adj)
miR−93	0.212	0.161	1.32	0.000936	0.202
miR−544	0.204	0.155	1.32	0.00123	0.202
miR−302c	0.188	0.142	1.33	0.00142	0.202
miR−466f−3p	0.24	0.19	1.26	0.00229	0.202
miR−302a	0.208	0.162	1.28	0.00248	0.202
miR−181c	0.192	0.149	1.29	0.00302	0.202
miR−295	0.198	0.155	1.28	0.00375	0.202
miR−543	0.154	0.116	1.33	0.00386	0.202
miR−19b	0.198	0.156	1.27	0.00445	0.202
miR−1−2−as	0.141	0.106	1.33	0.00586	0.202
miR−20a	0.216	0.174	1.24	0.00604	0.202
miR−338−5p	0.162	0.126	1.29	0.00671	0.202
miR−465b−5p	0.218	0.177	1.23	0.00706	0.202
miR−19a	0.21	0.169	1.24	0.00708	0.202
miR−20b	0.2	0.16	1.25	0.00733	0.202
miR−669i	0.135	0.102	1.32	0.0075	0.202
miR−139−5p	0.156	0.121	1.29	0.00783	0.202
miR−302b	0.208	0.168	1.24	0.00784	0.202
miR−181d	0.216	0.175	1.23	0.00799	0.202
miR−466c−5p	0.19	0.152	1.25	0.00842	0.202
miR−138	0.164	0.129	1.27	0.00863	0.202
miR−200a	0.188	0.151	1.25	0.00921	0.206
miR−126−5p	0.17	0.135	1.26	0.01	0.209
miR−128	0.178	0.143	1.25	0.0102	0.209
miR−590−3p	0.305	0.261	1.17	0.0107	0.21
miR−135b	0.216	0.179	1.21	0.0133	0.25
miR−1187	0.202	0.166	1.22	0.0137	0.25
miR−126−3p	0.0158	0.00755	2.1	0.0151	0.257
miR−449a	0.17	0.138	1.24	0.0156	0.257
miR−467f	0.15	0.12	1.26	0.0157	0.257

↑Age

Abundance of miRNA Targets

miRNA	Freq(Obs)	Freq(Exp)	Obs/Exp	P−value	P−Value(Adj)
miR−804	0.111	0.0852	1.3	0.0179	0.279
miR−320	0.216	0.181	1.19	0.0182	0.279
miR−152	0.15	0.121	1.25	0.0187	0.279
miR−101a	0.141	0.113	1.25	0.0214	0.309
miR−146a	0.147	0.118	1.24	0.0231	0.32
miR−669j	0.129	0.103	1.25	0.0243	0.32
miR−682	0.129	0.103	1.25	0.0246	0.32
miR−465c−5p	0.184	0.153	1.2	0.0248	0.32
miR−181b	0.206	0.174	1.19	0.0254	0.32
miR−452	0.168	0.139	1.21	0.0262	0.321
miR−30d	0.162	0.134	1.21	0.0287	0.342
miR−453	0.099	0.0771	1.28	0.0292	0.342
miR−376a	0.0337	0.022	1.53	0.0318	0.36
miR−1	0.168	0.141	1.2	0.0336	0.36
miR−30a	0.174	0.146	1.19	0.0337	0.36
miR−148a	0.152	0.126	1.21	0.0343	0.36
miR−871	0.202	0.172	1.17	0.0345	0.36
miR−494	0.178	0.15	1.18	0.0363	0.36
miR−590−5p	0.117	0.0942	1.24	0.0364	0.36
miR−182	0.186	0.158	1.18	0.0367	0.36
miR−146b	0.145	0.12	1.21	0.0378	0.364
miR−669h−3p	0.176	0.149	1.18	0.04	0.366
miR−105	0.145	0.12	1.2	0.0405	0.366
miR−206	0.158	0.133	1.19	0.0409	0.366
miR−465a−5p	0.23	0.2	1.15	0.0416	0.366
miR−181a	0.188	0.161	1.17	0.0431	0.366
miR−302d	0.19	0.163	1.17	0.0432	0.366
miR−673−5p	0.164	0.139	1.18	0.0445	0.366
miR−466d−3p	0.19	0.163	1.17	0.0446	0.366
miR−667	0.0812	0.0634	1.28	0.0448	0.366

↑Age

Abundance of miRNA Targets

miRNA	Freq(Obs)	Freq(Exp)	Obs/Exp	P−value	P−Value(Adj)
miR−466k	0.178	0.153	1.17	0.0492	0.383
miR−468	0.119	0.0977	1.22	0.0492	0.383
miR−875−5p	0.0792	0.0622	1.27	0.0498	0.383
miR−669a	0.143	0.12	1.19	0.05	0.383

↓Age

Abundance of miRNA Targets

miRNA	Freq(Obs)	Freq(Exp)	Obs/Exp	P−value	P−Value(Adj)
miR−423−5p	0.219	0.141	1.56	0.00202	0.597
miR−337−3p	0.166	0.102	1.62	0.00382	0.597
miR−764−5p	0.124	0.0706	1.76	0.00405	0.597
miR−10b	0.124	0.0727	1.71	0.00561	0.597
miR−10a	0.124	0.0758	1.64	0.00903	0.597
miR−7b	0.225	0.159	1.41	0.00954	0.597
miR−671−5p	0.189	0.129	1.46	0.00985	0.597
miR−762	0.219	0.155	1.41	0.0104	0.597
miR−217	0.178	0.121	1.46	0.0122	0.597
miR−324−3p	0.13	0.0831	1.57	0.0131	0.597
miR−590−5p	0.142	0.0942	1.51	0.0157	0.597
miR−703	0.178	0.124	1.43	0.0162	0.597
miR−298	0.272	0.207	1.32	0.0162	0.597
miR−484	0.154	0.105	1.47	0.0171	0.597
miR−149	0.189	0.136	1.39	0.0195	0.619
miR−666−3p	0.183	0.132	1.39	0.0209	0.619
miR−329	0.136	0.092	1.48	0.0215	0.619
miR−194	0.148	0.104	1.43	0.0267	0.726
miR−542−3p	0.183	0.137	1.34	0.0337	0.738
miR−1−2−as	0.148	0.106	1.39	0.0338	0.738
miR−770−5p	0.0947	0.0624	1.52	0.0354	0.738
miR−669k	0.16	0.118	1.36	0.0387	0.738
miR−877	0.148	0.108	1.37	0.0402	0.738
miR−1186	0.172	0.128	1.34	0.0403	0.738
miR−883b−5p	0.207	0.161	1.29	0.0439	0.738
miR−494	0.195	0.15	1.3	0.0445	0.738
miR−7a	0.207	0.161	1.29	0.0447	0.738
let−7c	0.166	0.125	1.33	0.0463	0.738
miR−871	0.219	0.172	1.27	0.0467	0.738
miR−881	0.189	0.146	1.3	0.0471	0.738

Tests for Chromosome Over−representation

Chromosome	Age−upregulated Genes	Age−downregulated Genes
1	0.826	0.548
2	0.695	0.157
3	0.695	0.795
4	0.268	0.523
5	0.86	0.46
6	0.977	0.393
7	0.983	0.296
8	0.312	0.286
9	0.706	0.353
10	0.798	0.43
11	0.156	0.0704
12	0.439	0.604
13	0.454	0.951
14	0.379	0.929
15	0.649	0.956
16	0.69	0.0203*
17	0.4	0.0919
18	0.982	0.451
19	0.913	0.519
X	0.597	0.47
Y	0.145	1.00

The table lists p−values generated from a test that evaluates whether there exists an over−abundance of identified genes with respect to a given chromosome. The null hypothesis assumes that the set of genes has been selected at random from those represented on the Affymetrix 430 2.0 array. A significant test indicates that a chromosome contains more of the identified genes than would be expected if the gene set had been chosen at random.

* = significant p−value, without multiple test adjustment

** = significant p−value, with multiple test adjustment

Chromosome Locations

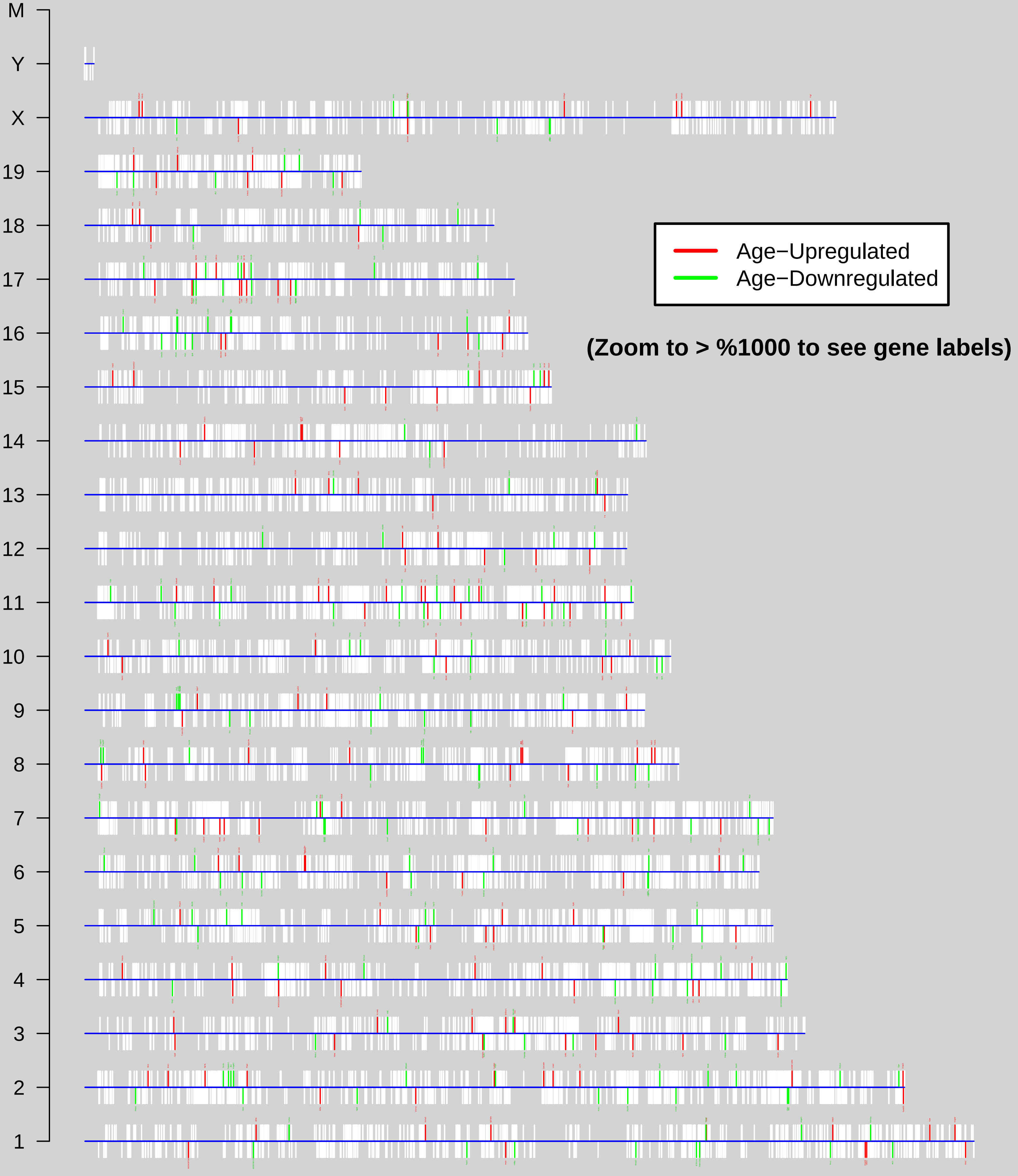

Supplement: Additional file 9 — Genes regulated by aging in muscle. Results from 4 experiments are analyzed to identify genes significantly up and down regulated by aging in muscle. This file also includes analysis of associated gene ontology terms, KEGG pathways, microRNA targets and chromosomal locations of age-regulated genes. [file 1471-2164-10-585-S9.PDF]
