# Supplementary material for: Genes and gene expression modules associated with caloric restriction and aging in the laboratory mouse
Source: BMC Genomics. 2009 Dec 7;10:585. doi: 10.1186/1471-2164-10-585 (PMC2795771; doi:10.1186/1471-2164-10-585)
Supplement: Additional file 10 — Genes regulated by aging in multiple mouse tissues. A gene chart is presented that provides a comprehensive listing of the genes most strongly increased by age across tissues, most strongly decreased by age across tissues, and most strongly regulated by age (in either direction) across tissues. The chart is comparable to those shown in Figure 5, except genes are ranked based upon a p-value generated using Fisher's method, rather than the total number of tissue types in which a gene is up or down regulated by age. This file also includes analysis of associated gene ontology terms, KEGG pathways, microRNA targets and chromosomal locations of age-regulated genes. [file 1471-2164-10-585-S10.PDF]

Additional File 10

Genes and Gene Expression Modules Associated with Caloric Restriction and Aging in the Laboratory Mouse

William R. Swindell
University of Michigan, Departments of Pathology and Geriatrics

---

Common Responses to Aging across 22 Mouse Tissues

This file provides information on genes regulated by age significantly among all 22 tissues included in the analysis. The first set of charts displays differential expression results of the 300 genes most strongly up regulated by age across tissues, while the second set of charts displays differential expression results for the 300 genes most strongly down regulated by age across tissues. An additional set of charts shows the top 300 most significant genes based upon a two-sided significance test for regulation by age across tissues (either up or down in each tissue). Each row corresponds to an individual gene and each column corresponds to one of 17 tissues analyzed. Symbols are interpreted as follows:

- ● Gene is significantly up regulated by age ($P_u < 0.05$)
- ● Gene is significantly down regulated by age ($P_d < 0.05$)
- ▬ Gene is marginally up regulated by age ($0.05 < P_u < 0.10$)
- ▬ Gene is marginally down regulated by age ($0.05 < P_d < 0.10$)
- ▬ Non-significant age effect ($P_u > 0.10$ and $P_d > 0.10$)
- × No data (gene not represented for a given tissue or array annotation was limiting)
- ✳ Evidence conflicts, but favors up regulation by age
- ✳ Evidence conflicts, but favors down regulation by age

The last two categories (✳ and ✳) indicate significant effects with conflicting evidence. This can arise if there is significant up regulation by age in one experiment, and significant down regulation by age in another experiment that has examined the same tissue. Alternatively, a conflict may arise if $P_u < 0.05$ and also $P_d < 0.05$ for a given tissue type. Symbols shown in charts are based upon a comparison-wise type I error rate of 0.05. The final column in each chart lists meta-analysis p-values generated using Fisher's method, which have been adjusted using the Benjamini-Hochberg method to control the false discovery rate among all 21,327 genes.

The remainder of the file includes lists of over-represented gene ontology terms, over-represented KEGG pathways, and over-represented KEGG pathways defined based upon IP domain signatures (see Hahne et al. 2008, BMC Bioinformatics 9:3). Genes were also analyzed to determine if there existed an over-abundance of targets for certain microRNAs (see Betel et al. 2008, Nucleic Acids Res. 36: D149-153), and a list of associated microRNAs is provided based upon this analysis. Lastly, tests for over-representation of identified genes with respect to each chromosome were performed, and an idiogram mapping of identified genes to chromosomal locations is shown.

Contact: William R. Swindell, wswindel@umich.edu

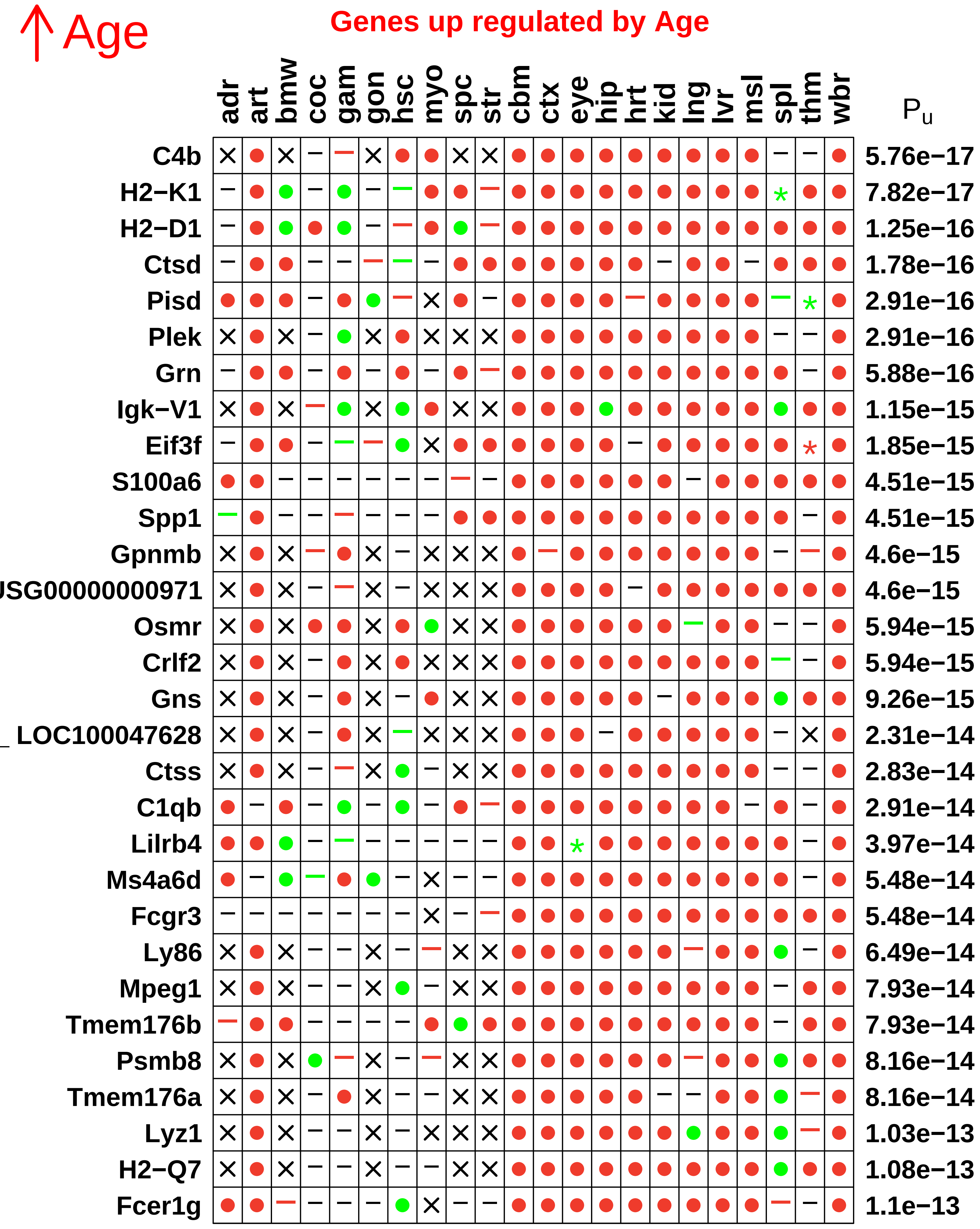
↑ Age
Genes up regulated by Age
adr
art
bmw
coc
gam
gon
hsc
myo
spc
str
cbm
ctx
eye
hip
hrt
kid
lng
lvr
msl
spl
thm
wbr
P_u
C4b 5.76e−17
H2−K1 7.82e−17
H2−D1 1.25e−16
Ctsd 1.78e−16
Pisd 2.91e−16
Plek 2.91e−16
Grn 5.88e−16
Igk−V1 1.15e−15
Eif3f 1.85e−15
S100a6 4.51e−15
Spp1 4.51e−15
Gpnmb 4.6e−15
JSG00000000971 4.6e−15
Osmr 5.94e−15
Crlf2 5.94e−15
Gns 9.26e−15
LOC100047628 2.31e−14
Ctss 2.83e−14
C1qb 2.91e−14
Lilrb4 3.97e−14
Ms4a6d 5.48e−14
Fcgr3 5.48e−14
Ly86 6.49e−14
Mpeg1 7.93e−14
Tmem176b 7.93e−14
Psmb8 8.16e−14
Tmem176a 8.16e−14
Lyz1 1.03e−13
H2−Q7 1.08e−13
Fcer1g 1.1e−13

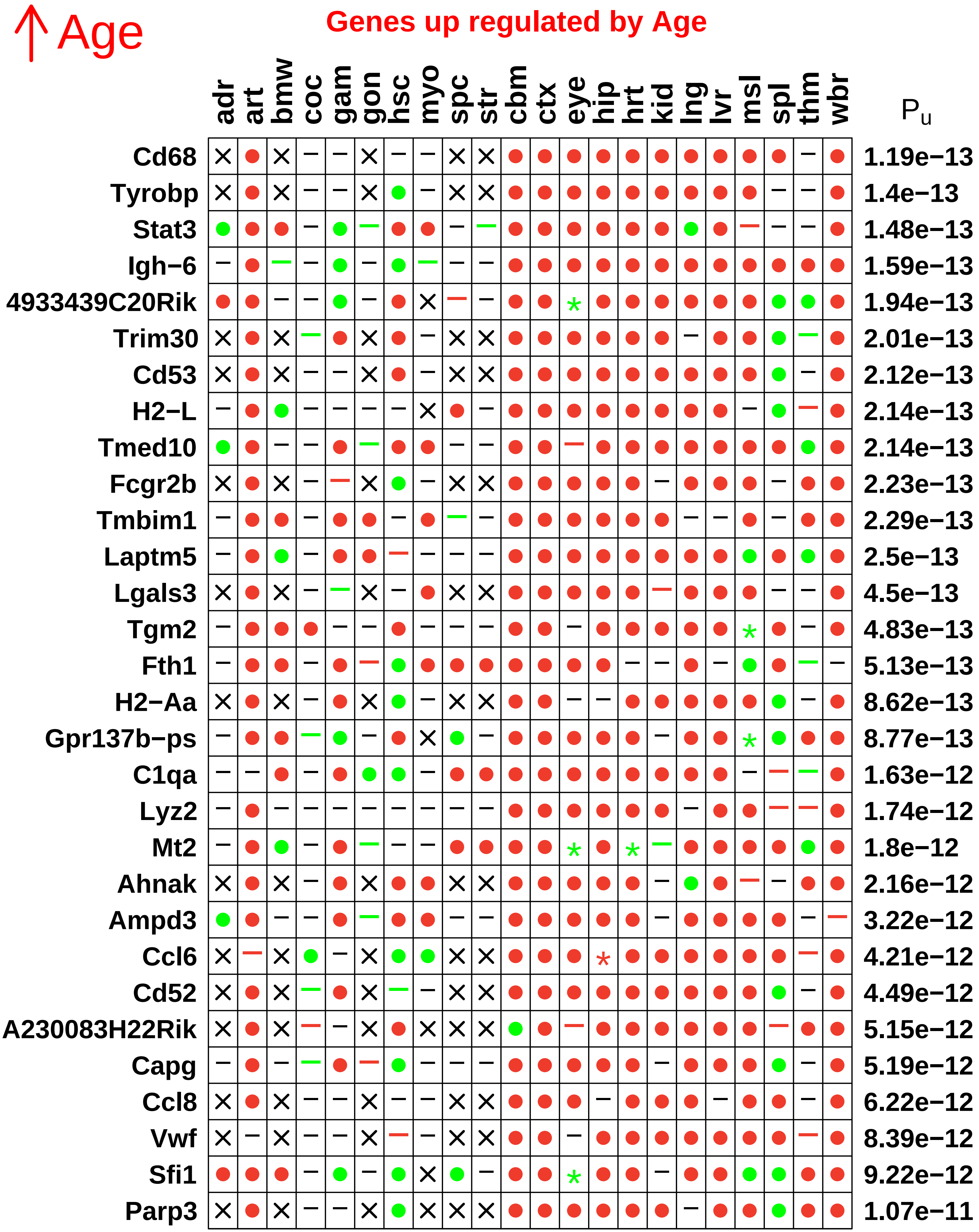

↑ Age
Genes up regulated by Age
adr
art
bmw
coc
gam
gon
hsc
myo
spc
str
cbm
ctx
eye
hip
hrt
kid
lng
lvr
msl
spl
thm
wbr
P_u
Cd68
Tyrobp
Stat3
Igh−6
4933439C20Rik
Trim30
Cd53
H2−L
Tmed10
Fcgr2b
Tmbim1
Laptm5
Lgals3
Tgm2
Fth1
H2−Aa
Gpr137b−ps
C1qa
Lyz2
Mt2
Ahnak
Ampd3
Ccl6
Cd52
A230083H22Rik
Capg
Ccl8
Vwf
Sfi1
Parp3
1.19e−13
1.4e−13
1.48e−13
1.59e−13
1.94e−13
2.01e−13
2.12e−13
2.14e−13
2.14e−13
2.23e−13
2.29e−13
2.5e−13
4.5e−13
4.83e−13
5.13e−13
8.62e−13
8.77e−13
1.63e−12
1.74e−12
1.8e−12
2.16e−12
3.22e−12
4.21e−12
4.49e−12
5.15e−12
5.19e−12
6.22e−12
8.39e−12
9.22e−12
1.07e−11

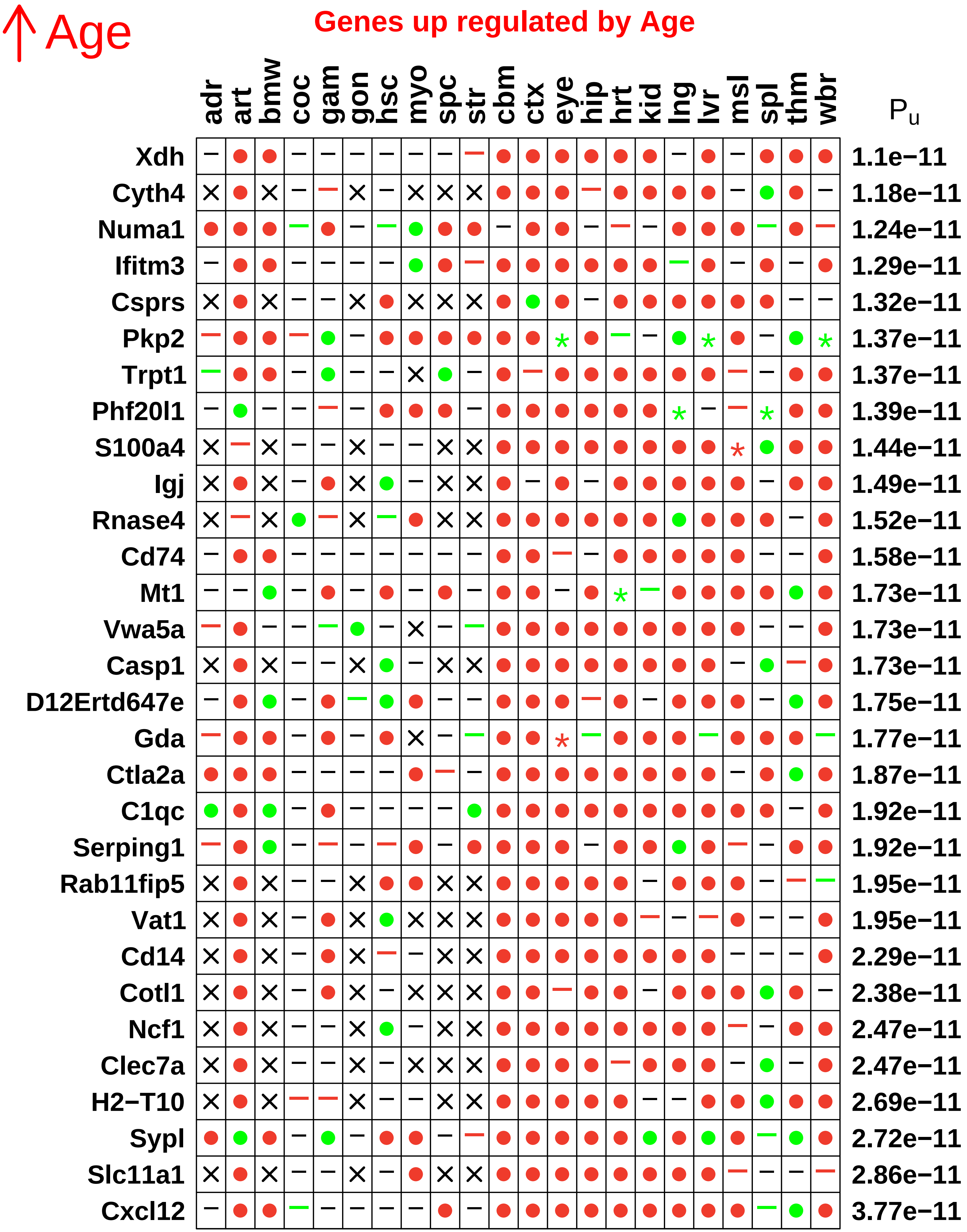
↑ Age
Genes up regulated by Age
adr
art
bmw
coc
gam
gon
hsc
myo
spc
str
cbm
ctx
eye
hip
hrt
kid
lng
lvr
msl
spl
thm
wbr
Pu
Xdh 1.1e−11
Cyth4 1.18e−11
Numa1 1.24e−11
Ifitm3 1.29e−11
Csprs 1.32e−11
Pkp2 1.37e−11
Trpt1 1.37e−11
Phf20l1 1.39e−11
S100a4 1.44e−11
Igj 1.49e−11
Rnase4 1.52e−11
Cd74 1.58e−11
Mt1 1.73e−11
Vwa5a 1.73e−11
Casp1 1.73e−11
D12Ertd647e 1.75e−11
Gda 1.77e−11
Ctla2a 1.87e−11
C1qc 1.92e−11
Serping1 1.92e−11
Rab11fip5 1.95e−11
Vat1 1.95e−11
Cd14 2.29e−11
Cotl1 2.38e−11
Ncf1 2.47e−11
Clec7a 2.47e−11
H2−T10 2.69e−11
Sypl 2.72e−11
Slc11a1 2.86e−11
Cxcl12 3.77e−11

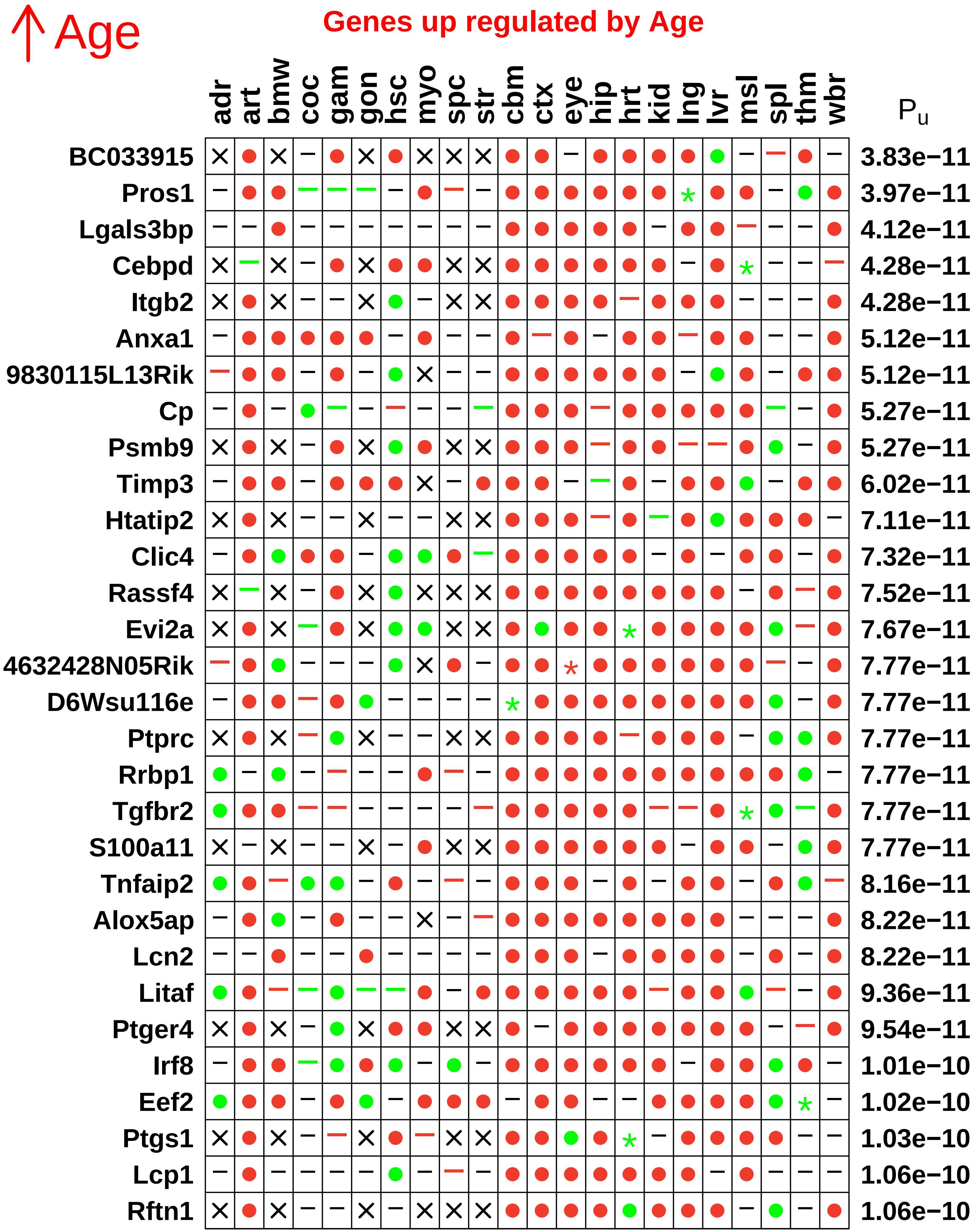

↑ Age
Genes up regulated by Age
adr
art
bmw
coc
gam
gon
hsc
myo
spc
str
cbm
ctx
eye
hip
hrt
kid
lng
lvr
msl
spl
thm
wbr
Pu
BC033915
3.83e−11
Pros1
3.97e−11
Lgals3bp
4.12e−11
Cebpd
4.28e−11
Itgb2
4.28e−11
Anxa1
5.12e−11
9830115L13Rik
5.12e−11
Cp
5.27e−11
Psmb9
5.27e−11
Timp3
6.02e−11
Htatip2
7.11e−11
Clic4
7.32e−11
Rassf4
7.52e−11
Evi2a
7.67e−11
4632428N05Rik
7.77e−11
D6Wsu116e
7.77e−11
Ptprc
7.77e−11
Rrbp1
7.77e−11
Tgfbr2
7.77e−11
S100a11
7.77e−11
Tnfaip2
8.16e−11
Alox5ap
8.22e−11
Lcn2
8.22e−11
Litaf
9.36e−11
Ptger4
9.54e−11
Irf8
1.01e−10
Eef2
1.02e−10
Ptgs1
1.03e−10
Lcp1
1.06e−10
Rftn1
1.06e−10

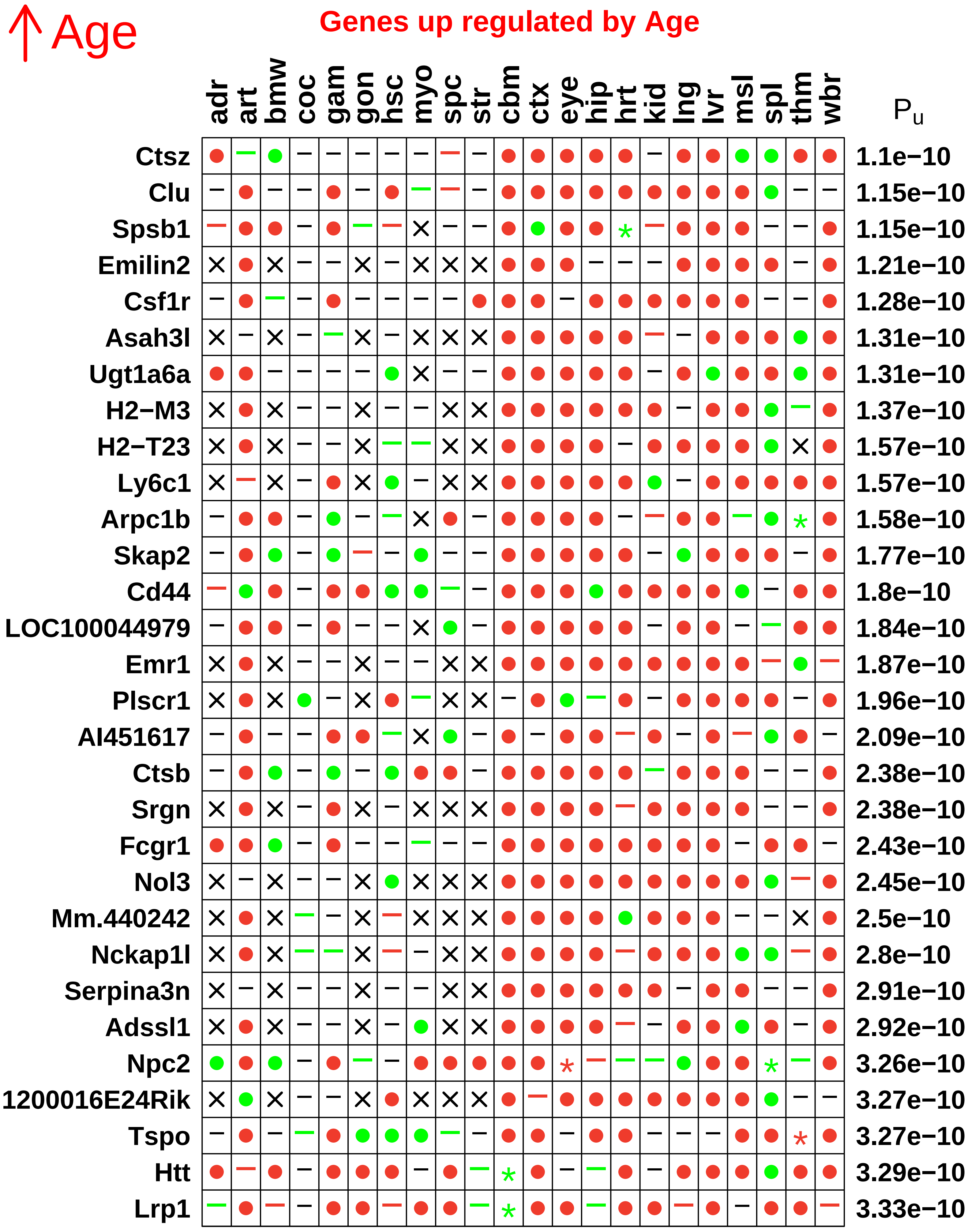

↑ Age
Genes up regulated by Age
adr
art
bmw
coc
gam
gon
hsc
myo
spc
str
cbm
ctx
eye
hip
hrt
kid
lng
lvr
msl
spl
thm
wbr
P_u
Ctsz 1.1e−10
Clu 1.15e−10
Spsb1 1.15e−10
Emilin2 1.21e−10
Csf1r 1.28e−10
Asah3l 1.31e−10
Ugt1a6a 1.31e−10
H2−M3 1.37e−10
H2−T23 1.57e−10
Ly6c1 1.57e−10
Arpc1b 1.58e−10
Skap2 1.77e−10
Cd44 1.8e−10
LOC100044979 1.84e−10
Emr1 1.87e−10
Plscr1 1.96e−10
AI451617 2.09e−10
Ctsb 2.38e−10
Srgn 2.38e−10
Fcgr1 2.43e−10
Nol3 2.45e−10
Mm.440242 2.5e−10
Nckap1l 2.8e−10
Serpina3n 2.91e−10
Adssl1 2.92e−10
Npc2 3.26e−10
1200016E24Rik 3.27e−10
Tspo 3.27e−10
Htt 3.29e−10
Lrp1 3.33e−10

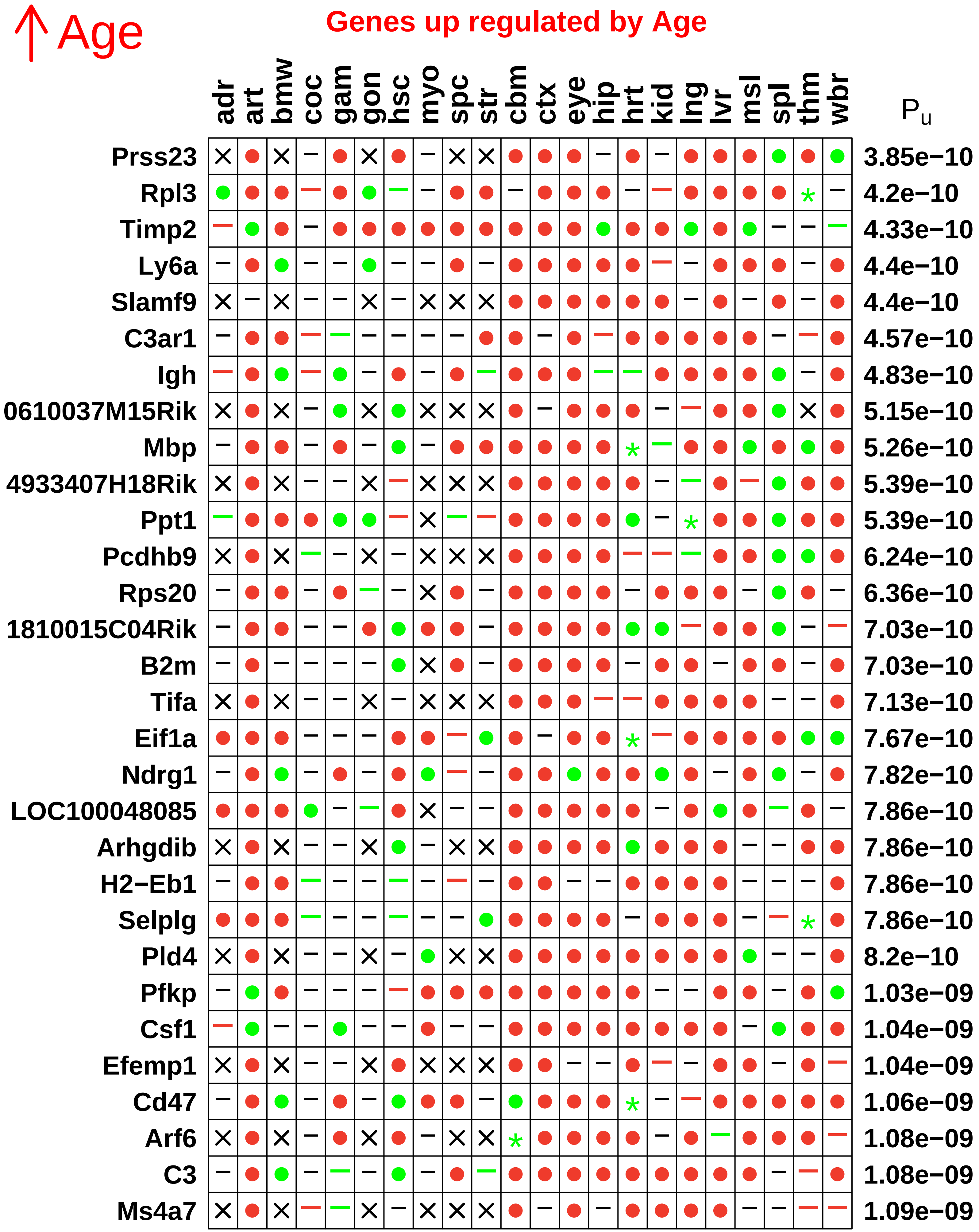

↑ Age
Genes up regulated by Age
adr
art
bmw
coc
gam
gon
hsc
myo
spc
str
cbm
ctx
eye
hip
hrt
kid
lng
lvr
msl
spl
thm
wbr
Pu
Prss23
Rpl3
Timp2
Ly6a
Slamf9
C3ar1
Igh
0610037M15Rik
Mbp
4933407H18Rik
Ppt1
Pcdhb9
Rps20
1810015C04Rik
B2m
Tifa
Eif1a
Ndrg1
LOC100048085
Arhgdib
H2−Eb1
Selplg
Pld4
Pfkp
Csf1
Efemp1
Cd47
Arf6
C3
Ms4a7
3.85e−10
4.2e−10
4.33e−10
4.4e−10
4.4e−10
4.57e−10
4.83e−10
5.15e−10
5.26e−10
5.39e−10
5.39e−10
6.24e−10
6.36e−10
7.03e−10
7.03e−10
7.13e−10
7.67e−10
7.82e−10
7.86e−10
7.86e−10
7.86e−10
7.86e−10
8.2e−10
1.03e−09
1.04e−09
1.04e−09
1.06e−09
1.08e−09
1.08e−09
1.09e−09

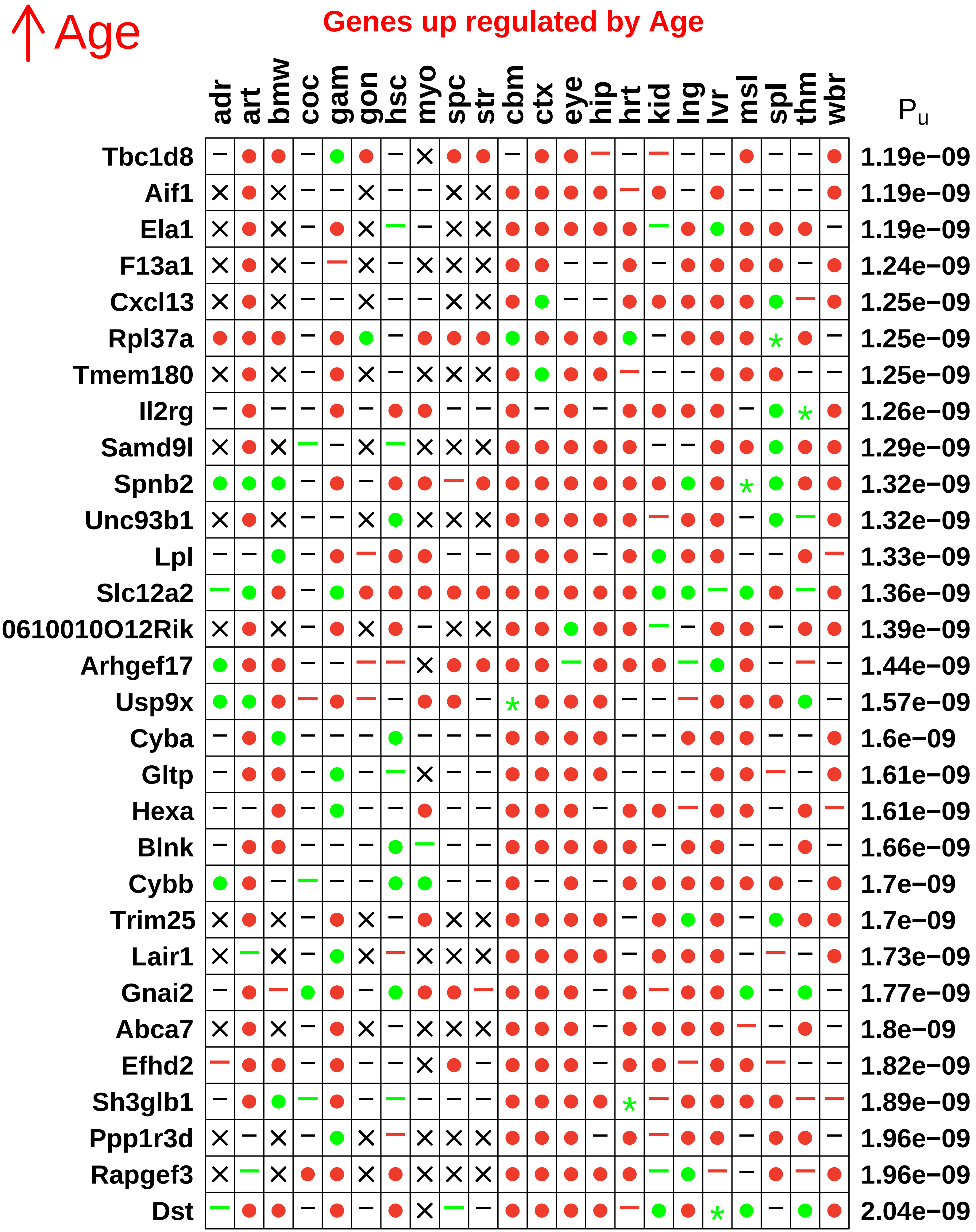
↑ Age
Genes up regulated by Age
adr
art
bmw
coc
gam
gon
hsc
myo
spc
str
cbm
ctx
eye
hip
hrt
kid
lng
lvr
msl
spl
thm
wbr
Pu
Tbc1d8 1.19e−09
Aif1 1.19e−09
Ela1 1.19e−09
F13a1 1.24e−09
Cxcl13 1.25e−09
Rpl37a 1.25e−09
Tmem180 1.25e−09
Il2rg 1.26e−09
Samd9l 1.29e−09
Spnb2 1.32e−09
Unc93b1 1.32e−09
Lpl 1.33e−09
Slc12a2 1.36e−09
0610010O12Rik 1.39e−09
Arhgef17 1.44e−09
Usp9x 1.57e−09
Cyba 1.6e−09
Gltp 1.61e−09
Hexa 1.61e−09
Blnk 1.66e−09
Cybb 1.7e−09
Trim25 1.7e−09
Lair1 1.73e−09
Gnai2 1.77e−09
Abca7 1.8e−09
Efhd2 1.82e−09
Sh3glb1 1.89e−09
Ppp1r3d 1.96e−09
Rapgef3 1.96e−09
Dst 2.04e−09

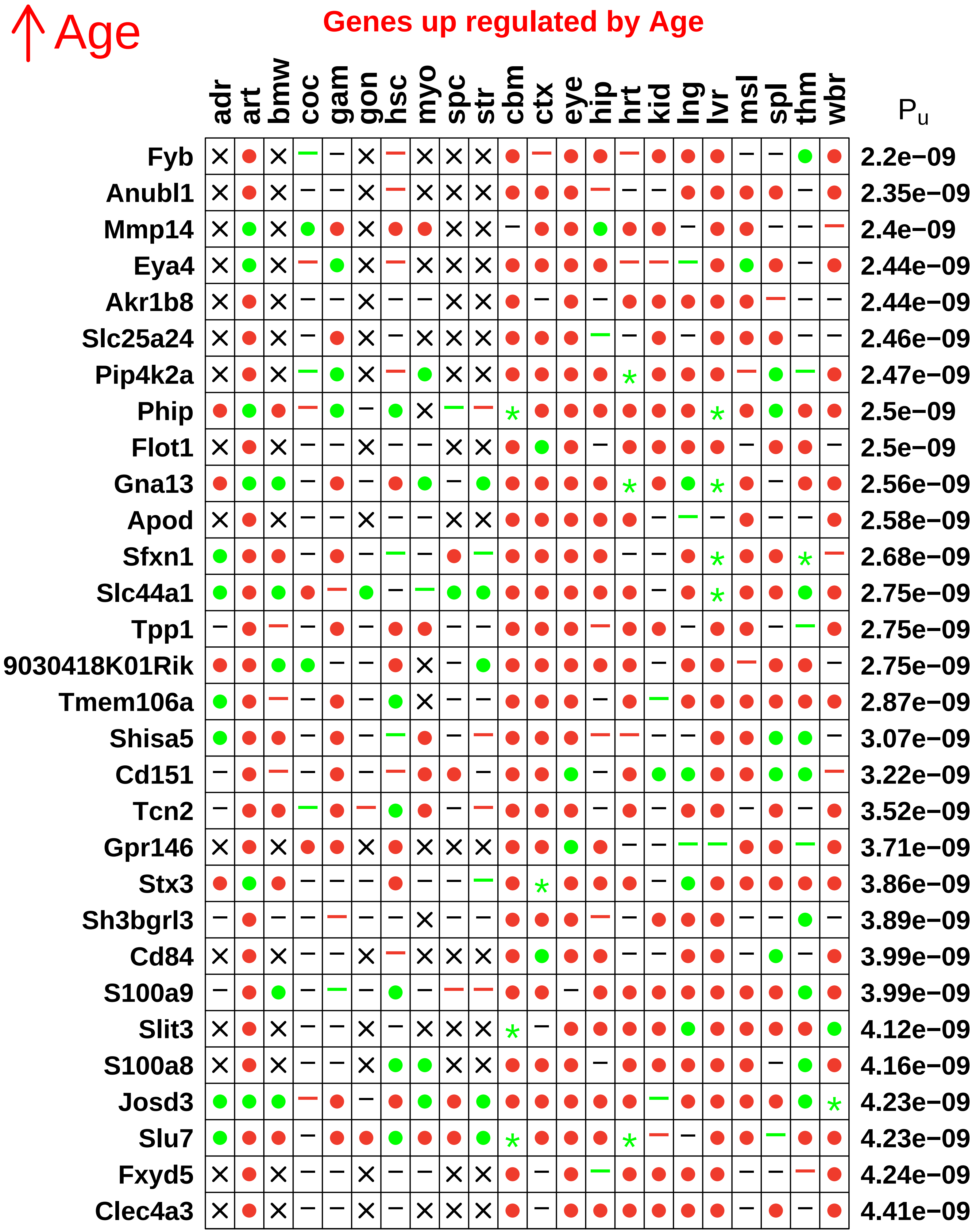
↑ Age
Genes up regulated by Age
adr
art
bmw
coc
gam
gon
hsc
myo
spc
str
cbm
ctx
eye
hip
hrt
kid
lng
lvr
msl
spl
thm
wbr
Pu
Fyb 2.2e−09
Anubl1 2.35e−09
Mmp14 2.4e−09
Eya4 2.44e−09
Akr1b8 2.44e−09
Slc25a24 2.46e−09
Pip4k2a 2.47e−09
Phip 2.5e−09
Flot1 2.5e−09
Gna13 2.56e−09
Apod 2.58e−09
Sfxn1 2.68e−09
Slc44a1 2.75e−09
Tpp1 2.75e−09
9030418K01Rik 2.75e−09
Tmem106a 2.87e−09
Shisa5 3.07e−09
Cd151 3.22e−09
Tcn2 3.52e−09
Gpr146 3.71e−09
Stx3 3.86e−09
Sh3bgrl3 3.89e−09
Cd84 3.99e−09
S100a9 3.99e−09
Slit3 4.12e−09
S100a8 4.16e−09
Josd3 4.23e−09
Slu7 4.23e−09
Fxyd5 4.24e−09
Clec4a3 4.41e−09

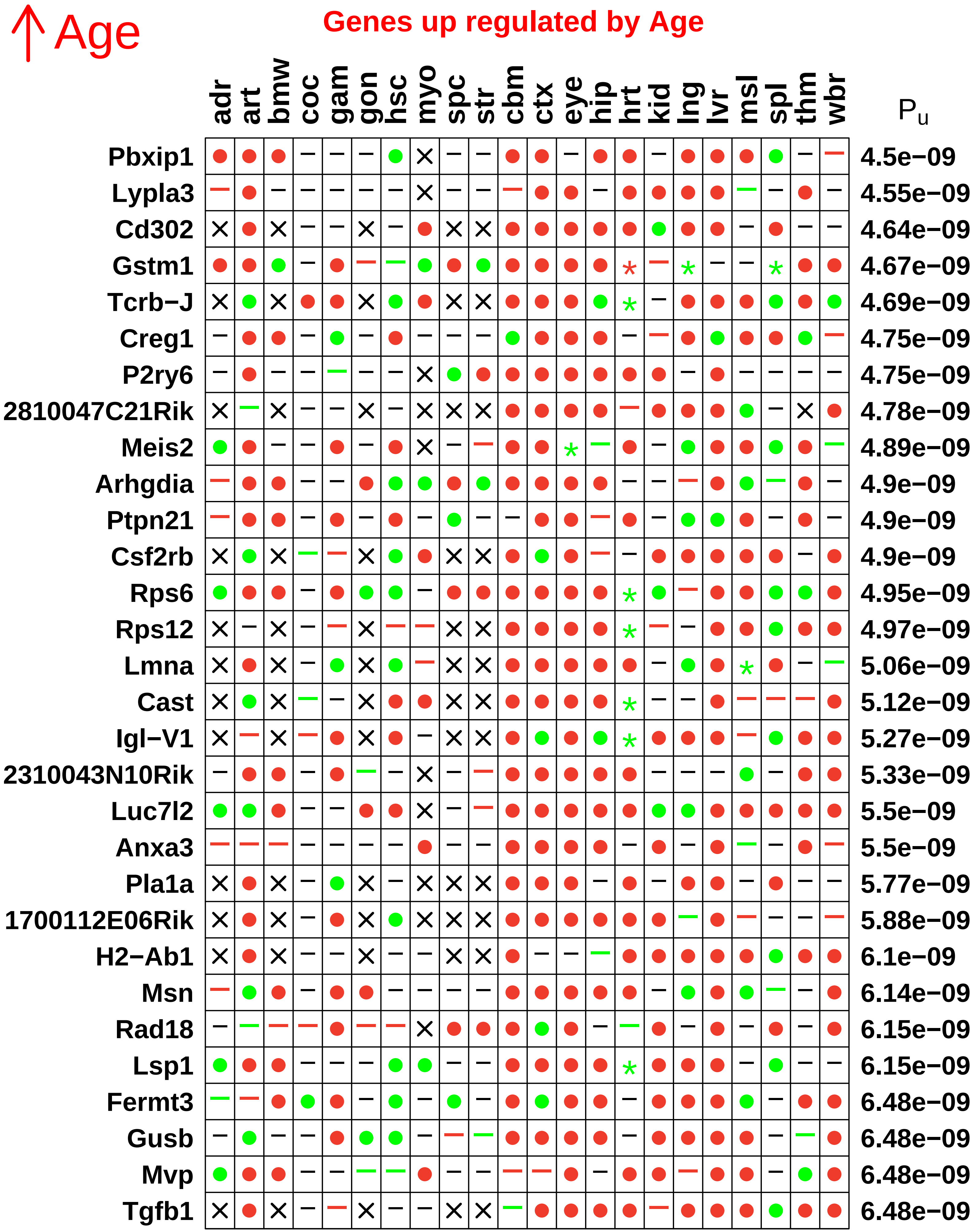

↑ Age
Genes up regulated by Age
adr art bmw coc gam gon hsc myo spc str cbm ctx eye hip hrt kid lng lvr msl spl thm wbr
P_u
Pbxip1 4.5e−09
Lypla3 4.55e−09
Cd302 4.64e−09
Gstm1 4.67e−09
Tcrb−J 4.69e−09
Creg1 4.75e−09
P2ry6 4.75e−09
2810047C21Rik 4.78e−09
Meis2 4.89e−09
Arhgdia 4.9e−09
Ptpn21 4.9e−09
Csf2rb 4.9e−09
Rps6 4.95e−09
Rps12 4.97e−09
Lmna 5.06e−09
Cast 5.12e−09
Igl−V1 5.27e−09
2310043N10Rik 5.33e−09
Luc7l2 5.5e−09
Anxa3 5.5e−09
Pla1a 5.77e−09
1700112E06Rik 5.88e−09
H2−Ab1 6.1e−09
Msn 6.14e−09
Rad18 6.15e−09
Lsp1 6.15e−09
Fermt3 6.48e−09
Gusb 6.48e−09
Mvp 6.48e−09
Tgfb1 6.48e−09

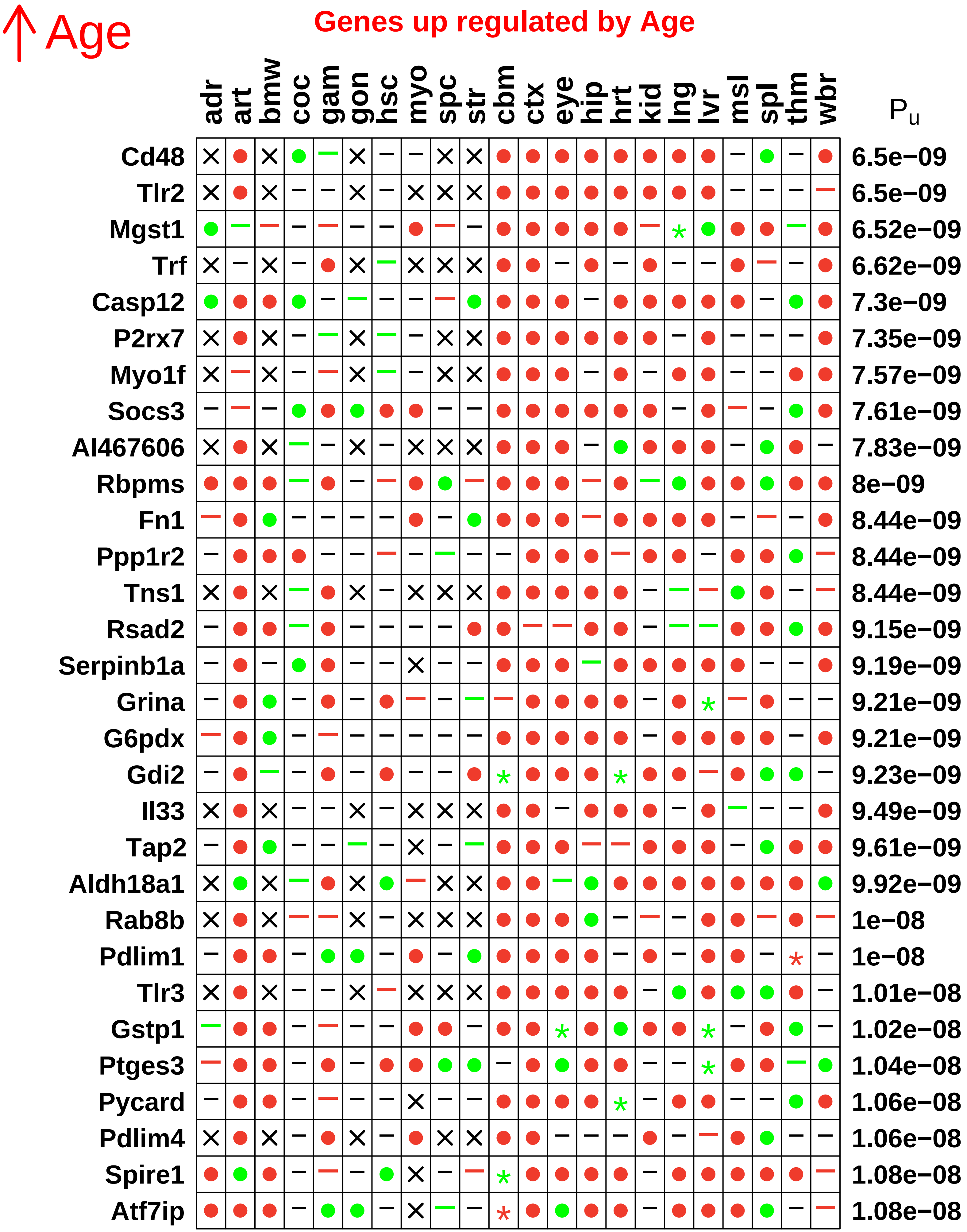

↑ Age
Genes up regulated by Age
adr
art
bmw
coc
gam
gon
hsc
myo
spc
str
cbm
ctx
eye
hip
hrt
kid
lng
lvr
msl
spl
thm
wbr
Pu
Cd48 6.5e−09
Tlr2 6.5e−09
Mgst1 6.52e−09
Trf 6.62e−09
Casp12 7.3e−09
P2rx7 7.35e−09
Myo1f 7.57e−09
Socs3 7.61e−09
AI467606 7.83e−09
Rbpms 8e−09
Fn1 8.44e−09
Ppp1r2 8.44e−09
Tns1 8.44e−09
Rsad2 9.15e−09
Serpinb1a 9.19e−09
Grina 9.21e−09
G6pdx 9.21e−09
Gdi2 9.23e−09
Il33 9.49e−09
Tap2 9.61e−09
Aldh18a1 9.92e−09
Rab8b 1e−08
Pdlim1 1e−08
Tlr3 1.01e−08
Gstp1 1.02e−08
Ptges3 1.04e−08
Pycard 1.06e−08
Pdlim4 1.06e−08
Spire1 1.08e−08
Atf7ip 1.08e−08

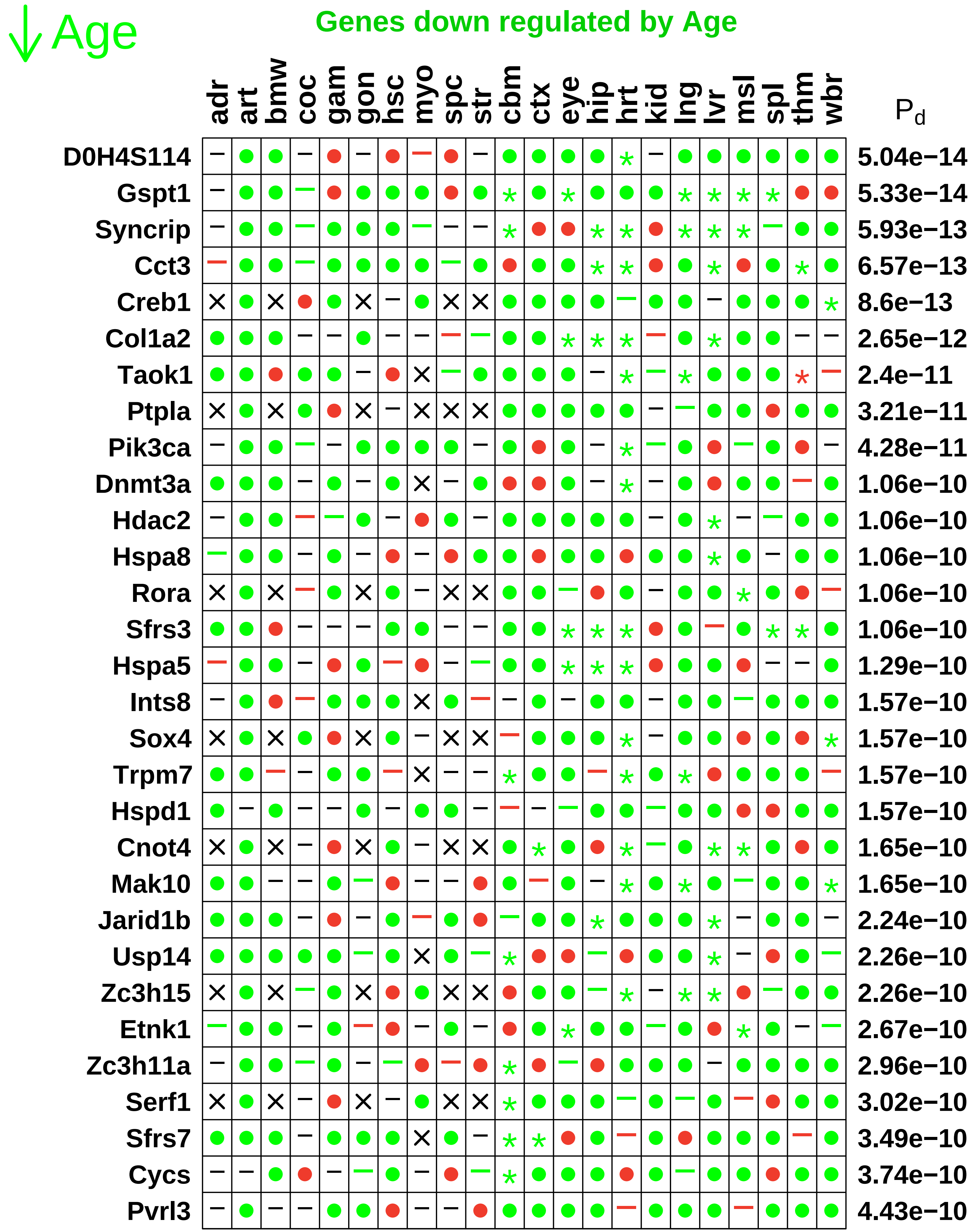
↓Age
Genes down regulated by Age
adr art bmw coc gam gon hsc myo spc str cbm ctx eye hip hrt kid lng lvr msl spl thm wbr
P_d
D0H4S114 5.04e−14
Gspt1 5.33e−14
Syncrip 5.93e−13
Cct3 6.57e−13
Creb1 8.6e−13
Col1a2 2.65e−12
Taok1 2.4e−11
Ptpla 3.21e−11
Pik3ca 4.28e−11
Dnmt3a 1.06e−10
Hdac2 1.06e−10
Hspa8 1.06e−10
Rora 1.06e−10
Sfrs3 1.06e−10
Hspa5 1.29e−10
Ints8 1.57e−10
Sox4 1.57e−10
Trpm7 1.57e−10
Hspd1 1.57e−10
Cnot4 1.65e−10
Mak10 1.65e−10
Jarid1b 2.24e−10
Usp14 2.26e−10
Zc3h15 2.26e−10
Etnk1 2.67e−10
Zc3h11a 2.96e−10
Serf1 3.02e−10
Sfrs7 3.49e−10
Cycs 3.74e−10
Pvrl3 4.43e−10

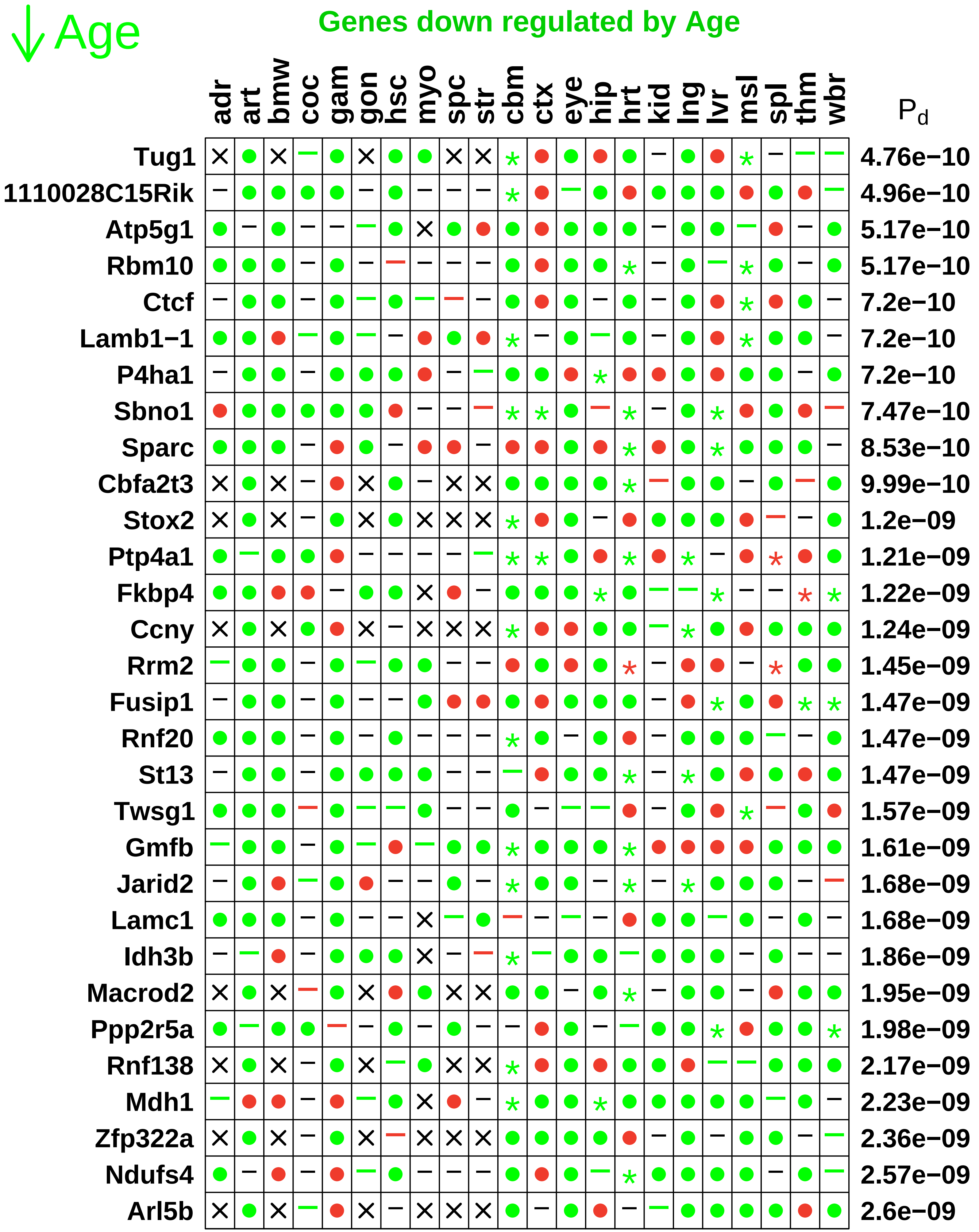
Age
Genes down regulated by Age
adr
art
bmw
coc
gam
gon
hsc
myo
spc
str
cbm
ctx
eye
hip
hrt
kid
lng
lvr
msl
spl
thm
wbr
P_d
Tug1 4.76e−10
1110028C15Rik 4.96e−10
Atp5g1 5.17e−10
Rbm10 5.17e−10
Ctcf 7.2e−10
Lamb1−1 7.2e−10
P4ha1 7.2e−10
Sbno1 7.47e−10
Sparc 8.53e−10
Cbfa2t3 9.99e−10
Stox2 1.2e−09
Ptp4a1 1.21e−09
Fkbp4 1.22e−09
Ccny 1.24e−09
Rrm2 1.45e−09
Fusip1 1.47e−09
Rnf20 1.47e−09
St13 1.47e−09
Twsg1 1.57e−09
Gmfb 1.61e−09
Jarid2 1.68e−09
Lamc1 1.68e−09
Idh3b 1.86e−09
Macrod2 1.95e−09
Ppp2r5a 1.98e−09
Rnf138 2.17e−09
Mdh1 2.23e−09
Zfp322a 2.36e−09
Ndufs4 2.57e−09
Arl5b 2.6e−09

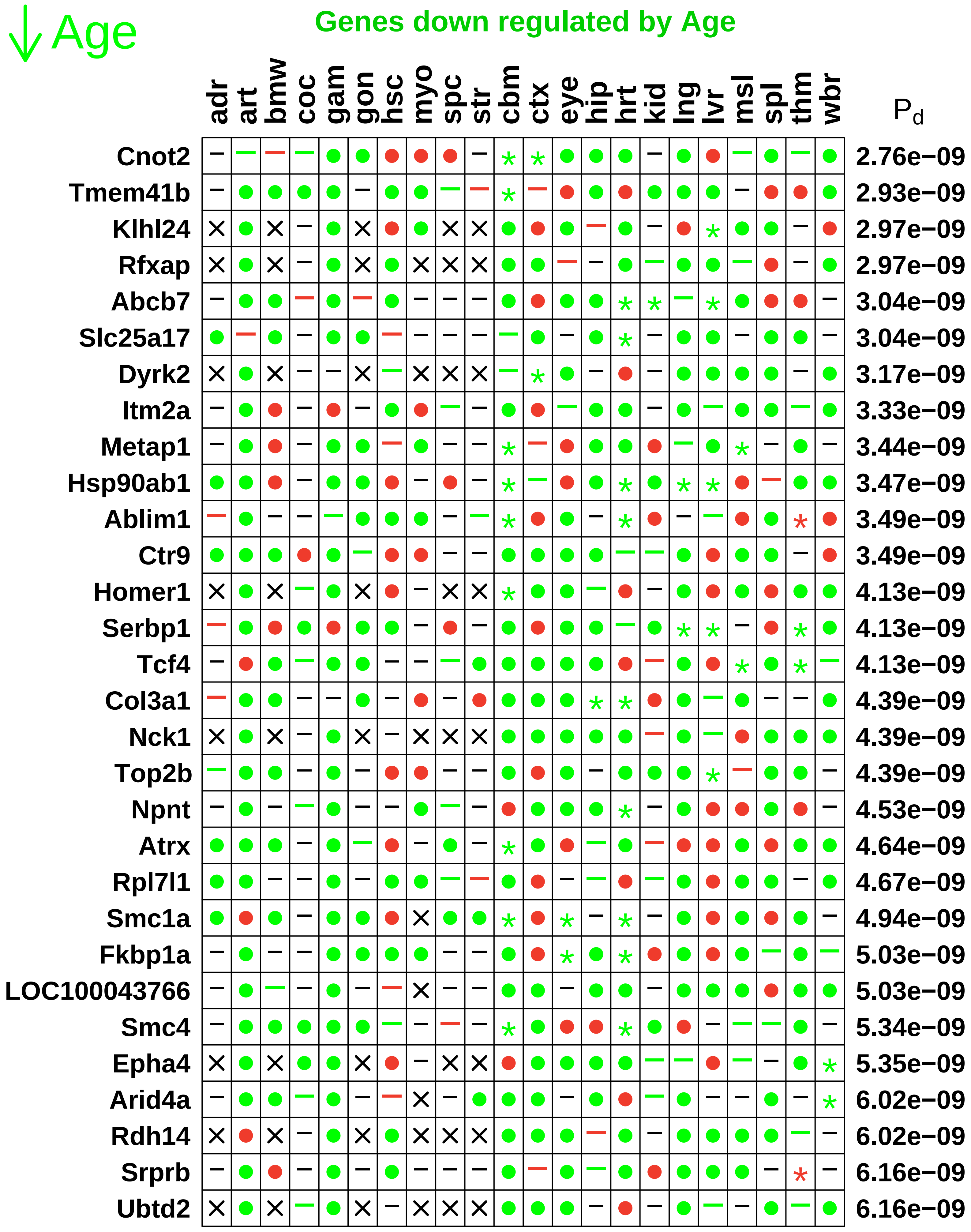

↓ Age
Genes down regulated by Age
adr
art
bmw
coc
gam
gon
hsc
myo
spc
str
cbm
ctx
eye
hip
hrt
kid
lng
lvr
msl
spl
thm
wbr
P_d
Cnot2 2.76e−09
Tmem41b 2.93e−09
Klhl24 2.97e−09
Rfxap 2.97e−09
Abcb7 3.04e−09
Slc25a17 3.04e−09
Dyrk2 3.17e−09
Itm2a 3.33e−09
Metap1 3.44e−09
Hsp90ab1 3.47e−09
Ablim1 3.49e−09
Ctr9 3.49e−09
Homer1 4.13e−09
Serbp1 4.13e−09
Tcf4 4.13e−09
Col3a1 4.39e−09
Nck1 4.39e−09
Top2b 4.39e−09
Npnt 4.53e−09
Atrx 4.64e−09
Rpl7l1 4.67e−09
Smc1a 4.94e−09
Fkbp1a 5.03e−09
LOC100043766 5.03e−09
Smc4 5.34e−09
Epha4 5.35e−09
Arid4a 6.02e−09
Rdh14 6.02e−09
Srprb 6.16e−09
Ubtd2 6.16e−09

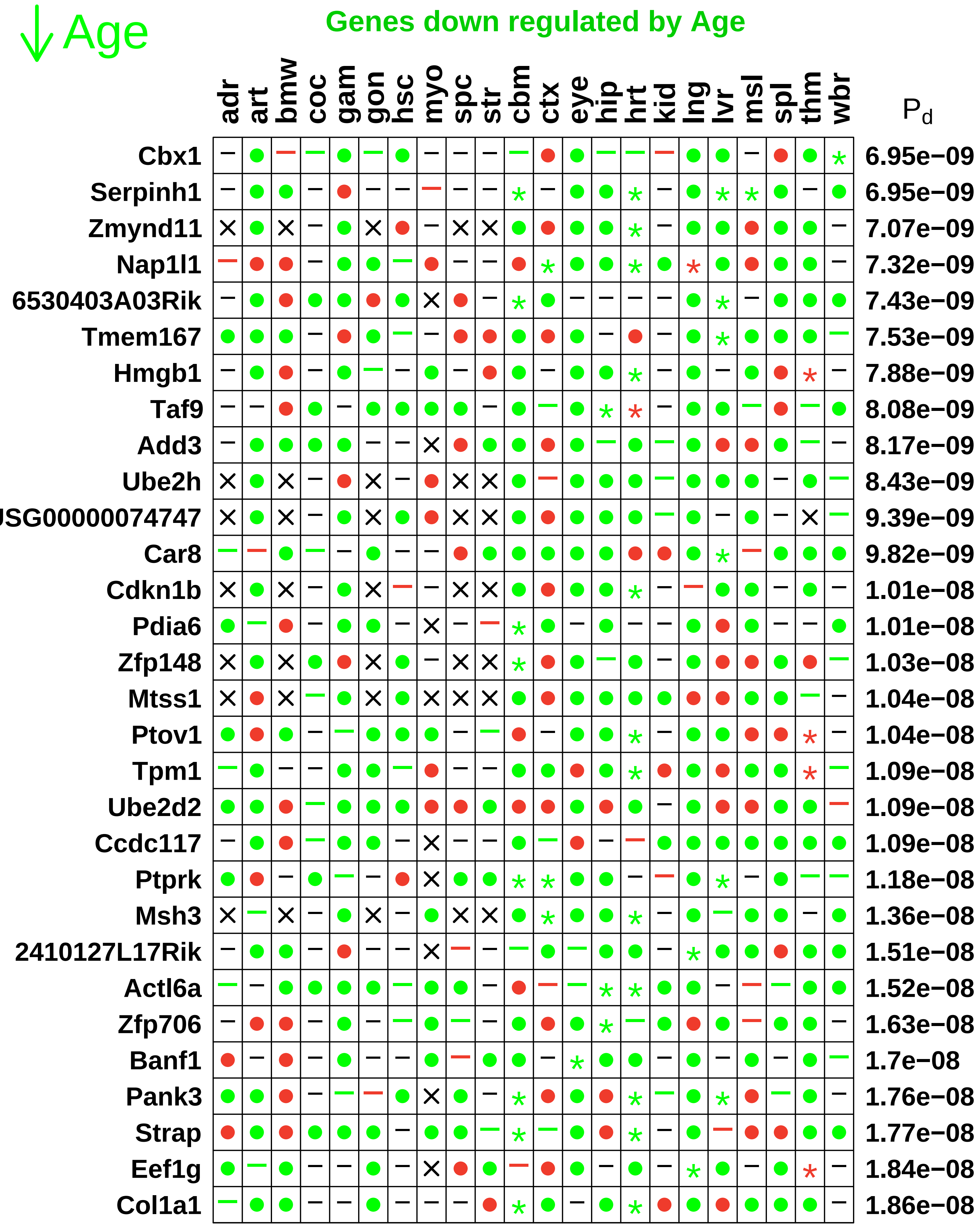

↓Age
Genes down regulated by Age
adr art bmw coc gam gon hsc myo spc str cbm ctx eye hip hrt kid lng lvr msl spl thm wbr
Pd
Cbx1 6.95e−09
Serpinh1 6.95e−09
Zmynd11 7.07e−09
Nap1l1 7.32e−09
6530403A03Rik 7.43e−09
Tmem167 7.53e−09
Hmgb1 7.88e−09
Taf9 8.08e−09
Add3 8.17e−09
Ube2h 8.43e−09
Car8 9.82e−09
Cdkn1b 1.01e−08
Pdia6 1.01e−08
Zfp148 1.03e−08
Mtss1 1.04e−08
Ptov1 1.04e−08
Tpm1 1.09e−08
Ube2d2 1.09e−08
Ccdc117 1.09e−08
Ptprk 1.18e−08
Msh3 1.36e−08
2410127L17Rik 1.51e−08
Actl6a 1.52e−08
Zfp706 1.63e−08
Banf1 1.7e−08
Pank3 1.76e−08
Strap 1.77e−08
Eef1g 1.84e−08
Col1a1 1.86e−08

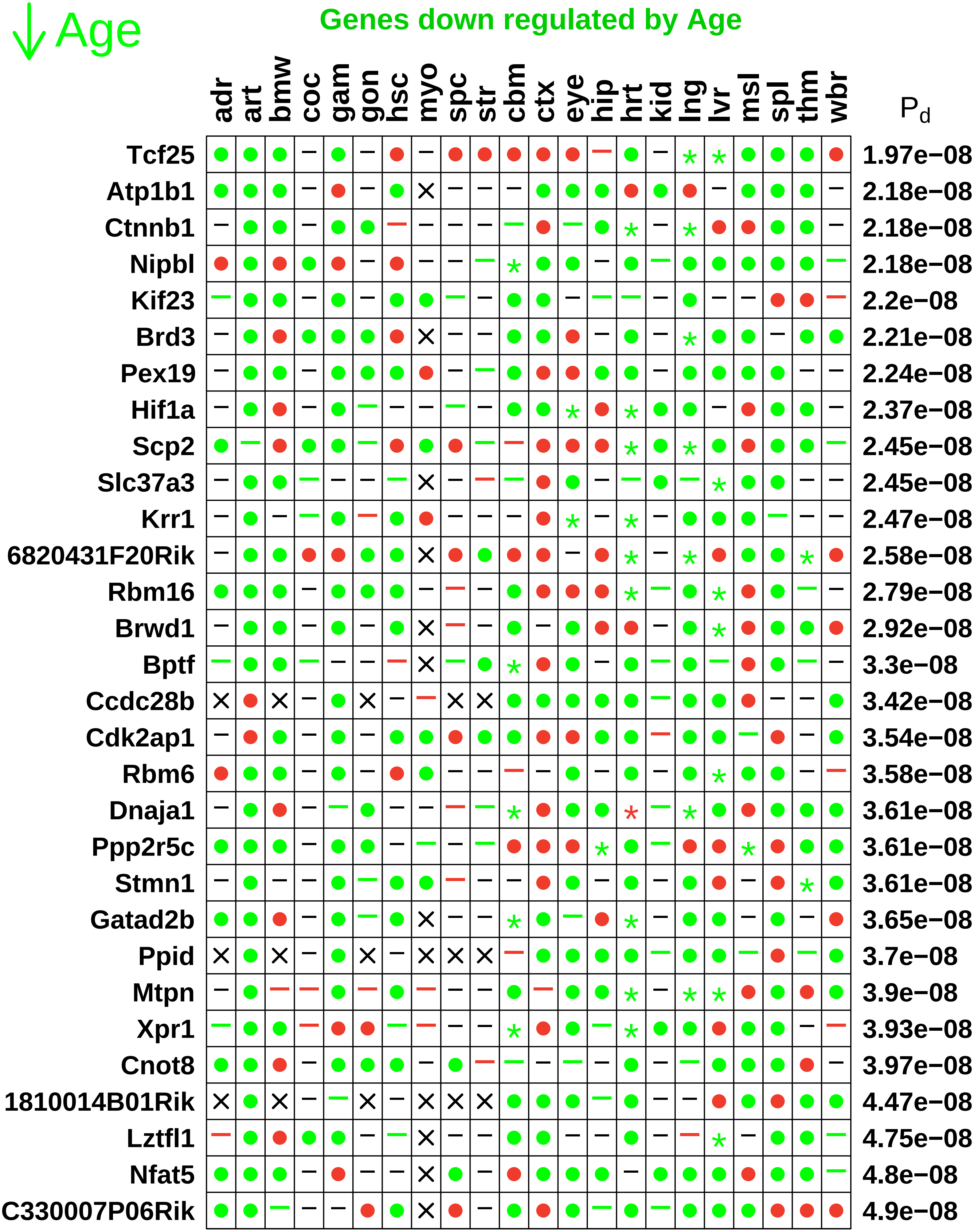

↓Age
Genes down regulated by Age
adr art bmw coc gam gon hsc myo spc str cbm ctx eye hip hrt kid lng lvr msl spl thm wbr
P_d
Tcf25 1.97e−08
Atp1b1 2.18e−08
Ctnnb1 2.18e−08
Nipbl 2.18e−08
Kif23 2.2e−08
Brd3 2.21e−08
Pex19 2.24e−08
Hif1a 2.37e−08
Scp2 2.45e−08
Slc37a3 2.45e−08
Krr1 2.47e−08
6820431F20Rik 2.58e−08
Rbm16 2.79e−08
Brwd1 2.92e−08
Bptf 3.3e−08
Ccdc28b 3.42e−08
Cdk2ap1 3.54e−08
Rbm6 3.58e−08
Dnaja1 3.61e−08
Ppp2r5c 3.61e−08
Stmn1 3.61e−08
Gatad2b 3.65e−08
Ppid 3.7e−08
Mtpn 3.9e−08
Xpr1 3.93e−08
Cnot8 3.97e−08
1810014B01Rik 4.47e−08
Lztfl1 4.75e−08
Nfat5 4.8e−08
C330007P06Rik 4.9e−08

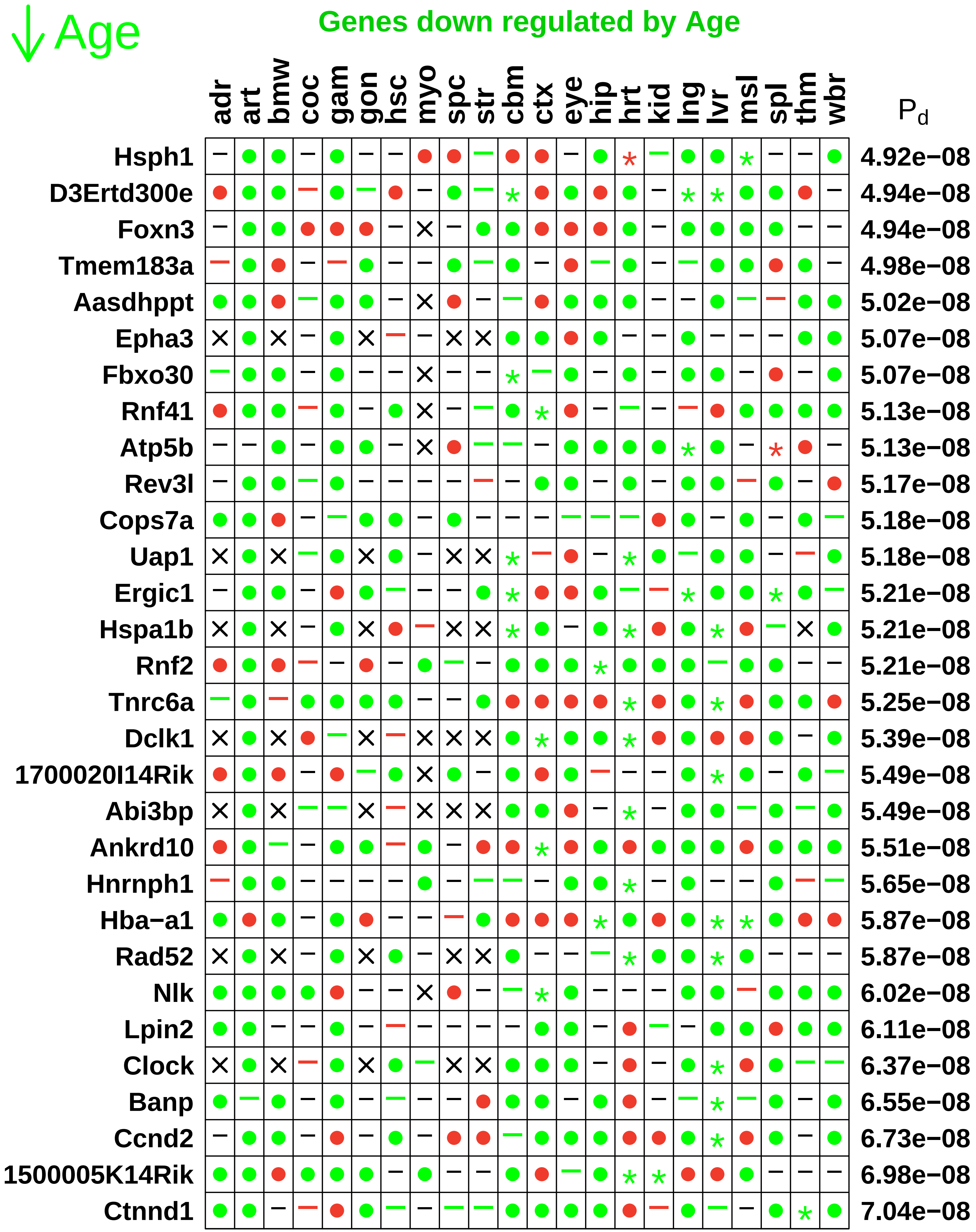

↓ Age
Genes down regulated by Age
adr
art
bmw
coc
gam
gon
hsc
myo
spc
str
cbm
ctx
eye
hip
hrt
kid
lng
lvr
msl
spl
thm
wbr
Pd
Hsph1 4.92e−08
D3Ertd300e 4.94e−08
Foxn3 4.94e−08
Tmem183a 4.98e−08
Aasdhppt 5.02e−08
Epha3 5.07e−08
Fbxo30 5.07e−08
Rnf41 5.13e−08
Atp5b 5.13e−08
Rev3l 5.17e−08
Cops7a 5.18e−08
Uap1 5.18e−08
Ergic1 5.21e−08
Hspa1b 5.21e−08
Rnf2 5.21e−08
Tnrc6a 5.25e−08
Dclk1 5.39e−08
1700020I14Rik 5.49e−08
Abi3bp 5.49e−08
Ankrd10 5.51e−08
Hnrnph1 5.65e−08
Hba−a1 5.87e−08
Rad52 5.87e−08
Nlk 6.02e−08
Lpin2 6.11e−08
Clock 6.37e−08
Banp 6.55e−08
Ccnd2 6.73e−08
1500005K14Rik 6.98e−08
Ctnnd1 7.04e−08

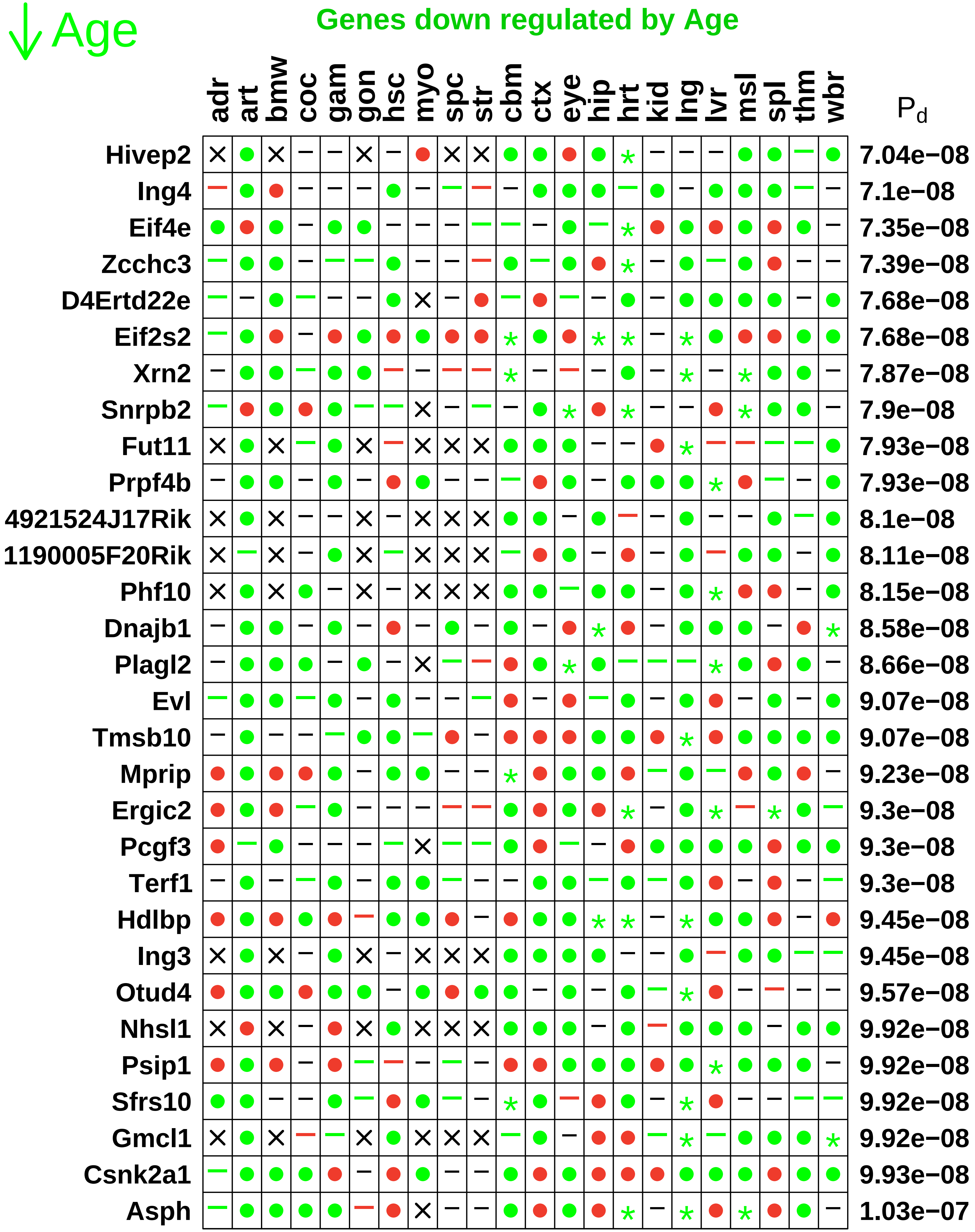
↓ Age
Genes down regulated by Age
adr art bmw coc gam gon hsc myo spc str cbm ctx eye hip hrt kid lng lvr msl spl thm wbr
P_d
Hivep2 7.04e−08
Ing4 7.1e−08
Eif4e 7.35e−08
Zcchc3 7.39e−08
D4Ertd22e 7.68e−08
Eif2s2 7.68e−08
Xrn2 7.87e−08
Snrpb2 7.9e−08
Fut11 7.93e−08
Prpf4b 7.93e−08
4921524J17Rik 8.1e−08
1190005F20Rik 8.11e−08
Phf10 8.15e−08
Dnajb1 8.58e−08
Plagl2 8.66e−08
Evl 9.07e−08
Tmsb10 9.07e−08
Mprip 9.23e−08
Ergic2 9.3e−08
Pcgf3 9.3e−08
Terf1 9.3e−08
Hdlbp 9.45e−08
Ing3 9.45e−08
Otud4 9.57e−08
Nhsl1 9.92e−08
Psip1 9.92e−08
Sfrs10 9.92e−08
Gmcl1 9.92e−08
Csnk2a1 9.93e−08
Asph 1.03e−07

↓ Age

Genes down regulated by Age

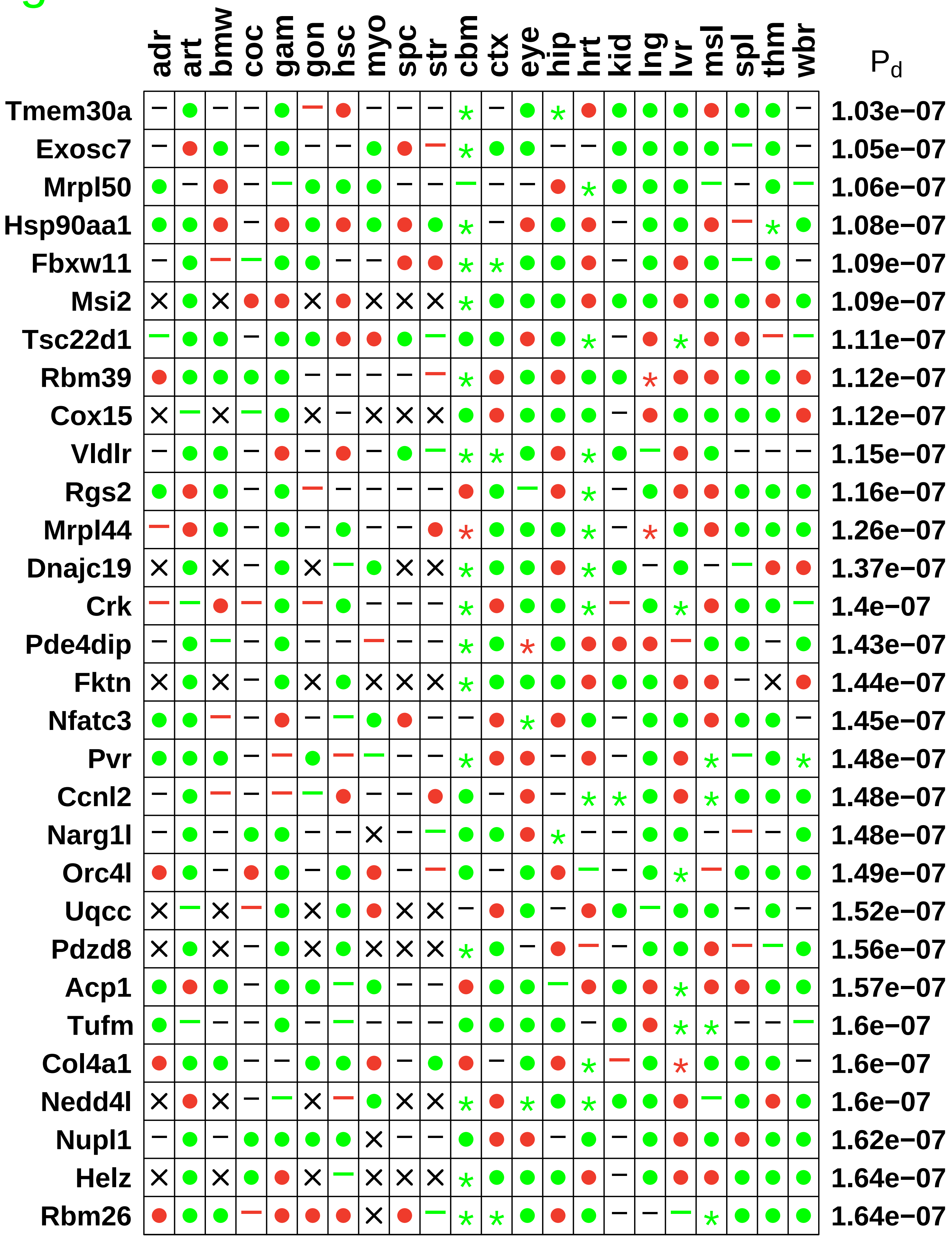

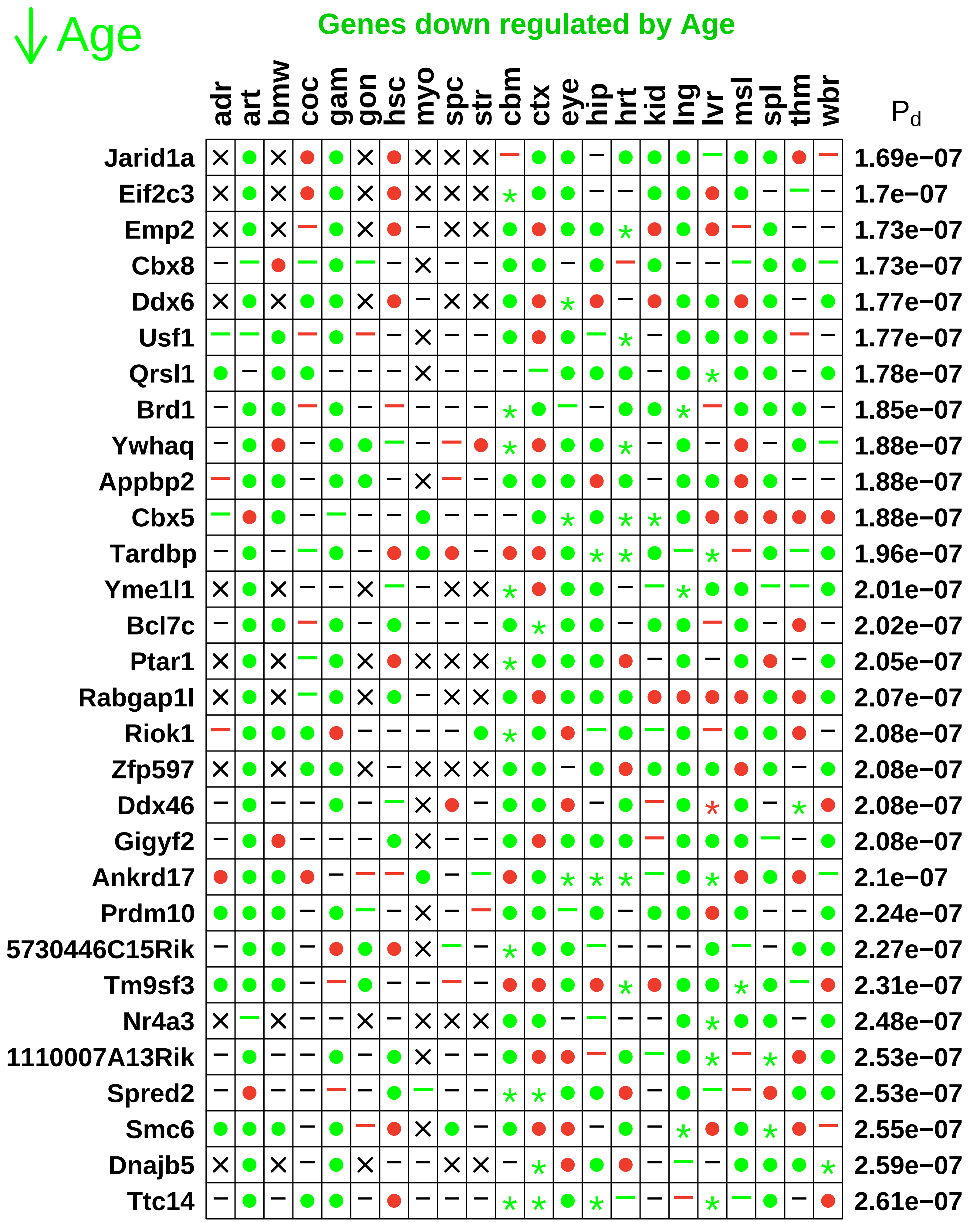

↓Age
Genes down regulated by Age
adr
art
bmw
coc
gam
gon
hsc
myo
spc
str
cbm
ctx
eye
hip
hrt
kid
lng
lvr
msl
spl
thm
wbr
P_d
Jarid1a 1.69e−07
Eif2c3 1.7e−07
Emp2 1.73e−07
Cbx8 1.73e−07
Ddx6 1.77e−07
Usf1 1.77e−07
Qrsl1 1.78e−07
Brd1 1.85e−07
Ywhaq 1.88e−07
Appbp2 1.88e−07
Cbx5 1.88e−07
Tardbp 1.96e−07
Yme1l1 2.01e−07
Bcl7c 2.02e−07
Ptar1 2.05e−07
Rabgap1l 2.07e−07
Riok1 2.08e−07
Zfp597 2.08e−07
Ddx46 2.08e−07
Gigyf2 2.08e−07
Ankrd17 2.1e−07
Prdm10 2.24e−07
5730446C15Rik 2.27e−07
Tm9sf3 2.31e−07
Nr4a3 2.48e−07
1110007A13Rik 2.53e−07
Spred2 2.53e−07
Smc6 2.55e−07
Dnajb5 2.59e−07
Ttc14 2.61e−07

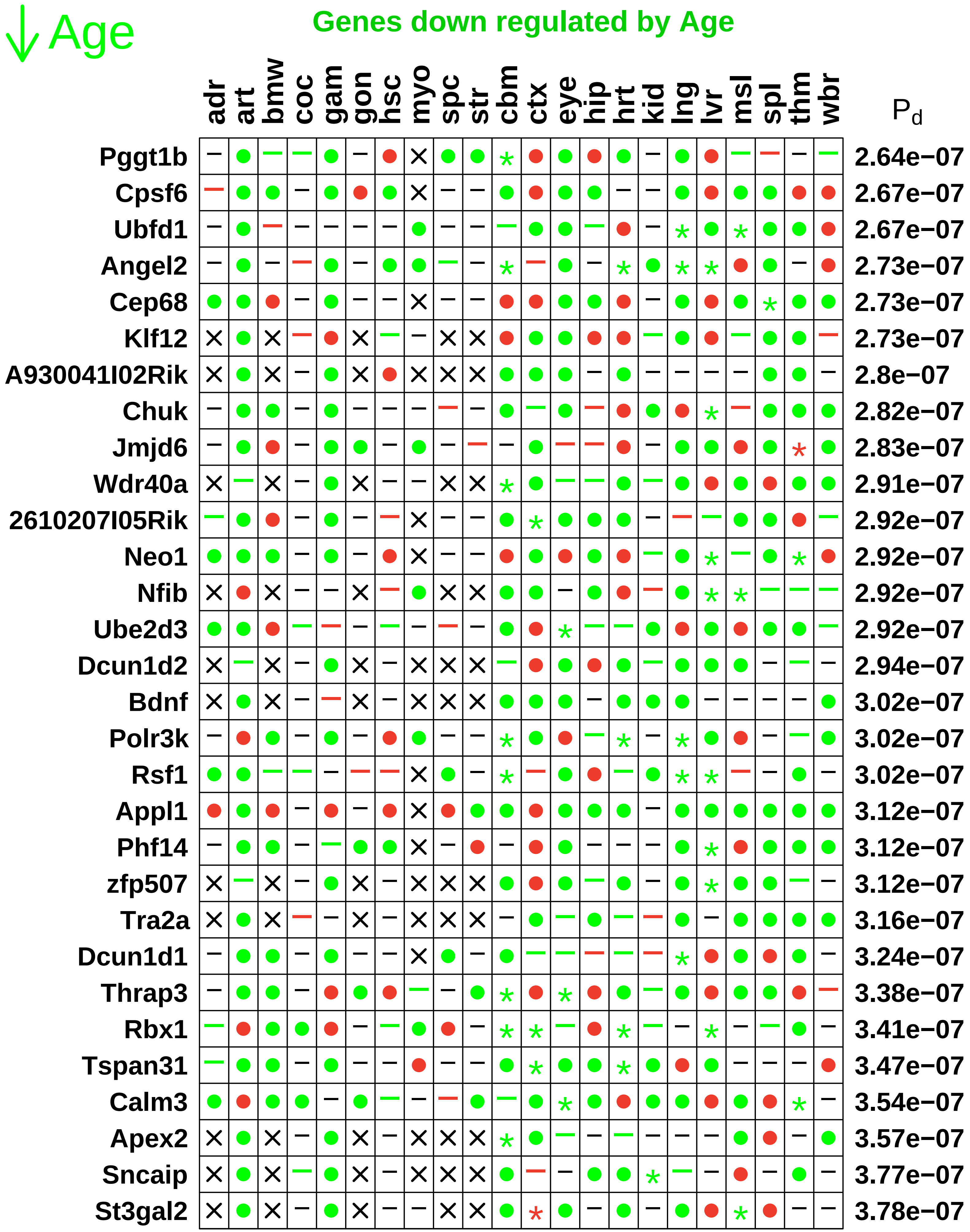

↓Age
Genes down regulated by Age
adr
art
bmw
coc
gam
gon
hsc
myo
spc
str
cbm
ctx
eye
hip
hrt
kid
lng
lvr
msl
spl
thm
wbr
P_d
Pggt1b
Cpsf6
Ubfd1
Angel2
Cep68
Klf12
A930041I02Rik
Chuk
Jmjd6
Wdr40a
2610207I05Rik
Neo1
Nfib
Ube2d3
Dcun1d2
Bdnf
Polr3k
Rsf1
Appl1
Phf14
zfp507
Tra2a
Dcun1d1
Thrap3
Rbx1
Tspan31
Calm3
Apex2
Sncaip
St3gal2
2.64e−07
2.67e−07
2.67e−07
2.73e−07
2.73e−07
2.73e−07
2.8e−07
2.82e−07
2.83e−07
2.91e−07
2.92e−07
2.92e−07
2.92e−07
2.92e−07
2.94e−07
3.02e−07
3.02e−07
3.02e−07
3.12e−07
3.12e−07
3.12e−07
3.16e−07
3.24e−07
3.38e−07
3.41e−07
3.47e−07
3.54e−07
3.57e−07
3.77e−07
3.78e−07

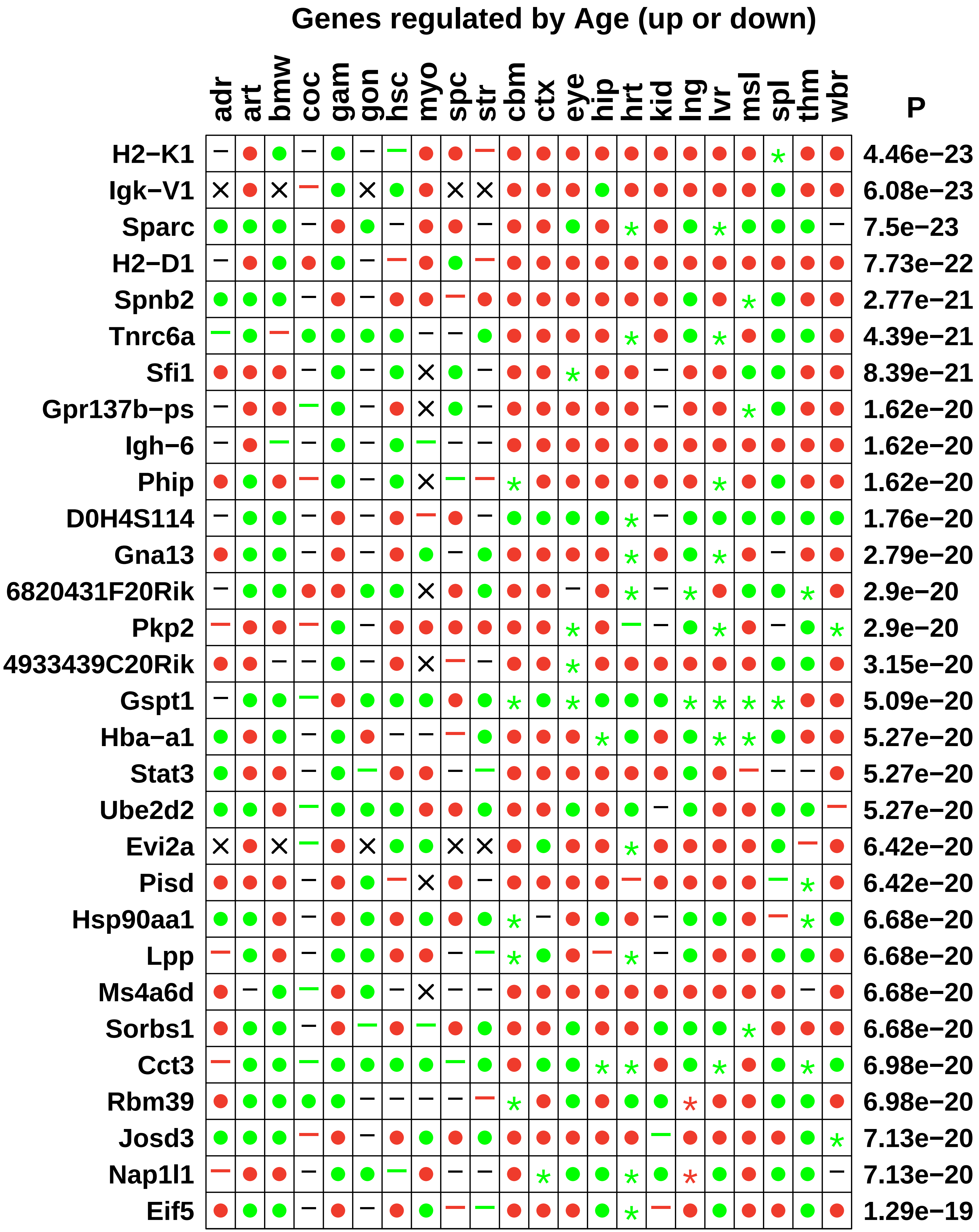

Genes regulated by Age (up or down)
adr
art
bmw
coc
gam
gon
hsc
myo
spc
str
cbm
ctx
eye
hip
hrt
kid
lng
lvr
msl
spl
thm
wbr
P
H2−K1 4.46e−23
Igk−V1 6.08e−23
Sparc 7.5e−23
H2−D1 7.73e−22
Spnb2 2.77e−21
Tnrc6a 4.39e−21
Sfi1 8.39e−21
Gpr137b−ps 1.62e−20
Igh−6 1.62e−20
Phip 1.62e−20
D0H4S114 1.76e−20
Gna13 2.79e−20
6820431F20Rik 2.9e−20
Pkp2 2.9e−20
4933439C20Rik 3.15e−20
Gspt1 5.09e−20
Hba−a1 5.27e−20
Stat3 5.27e−20
Ube2d2 5.27e−20
Evi2a 6.42e−20
Pisd 6.42e−20
Hsp90aa1 6.68e−20
Lpp 6.68e−20
Ms4a6d 6.68e−20
Sorbs1 6.68e−20
Cct3 6.98e−20
Rbm39 6.98e−20
Josd3 7.13e−20
Nap1l1 7.13e−20
Eif5 1.29e−19

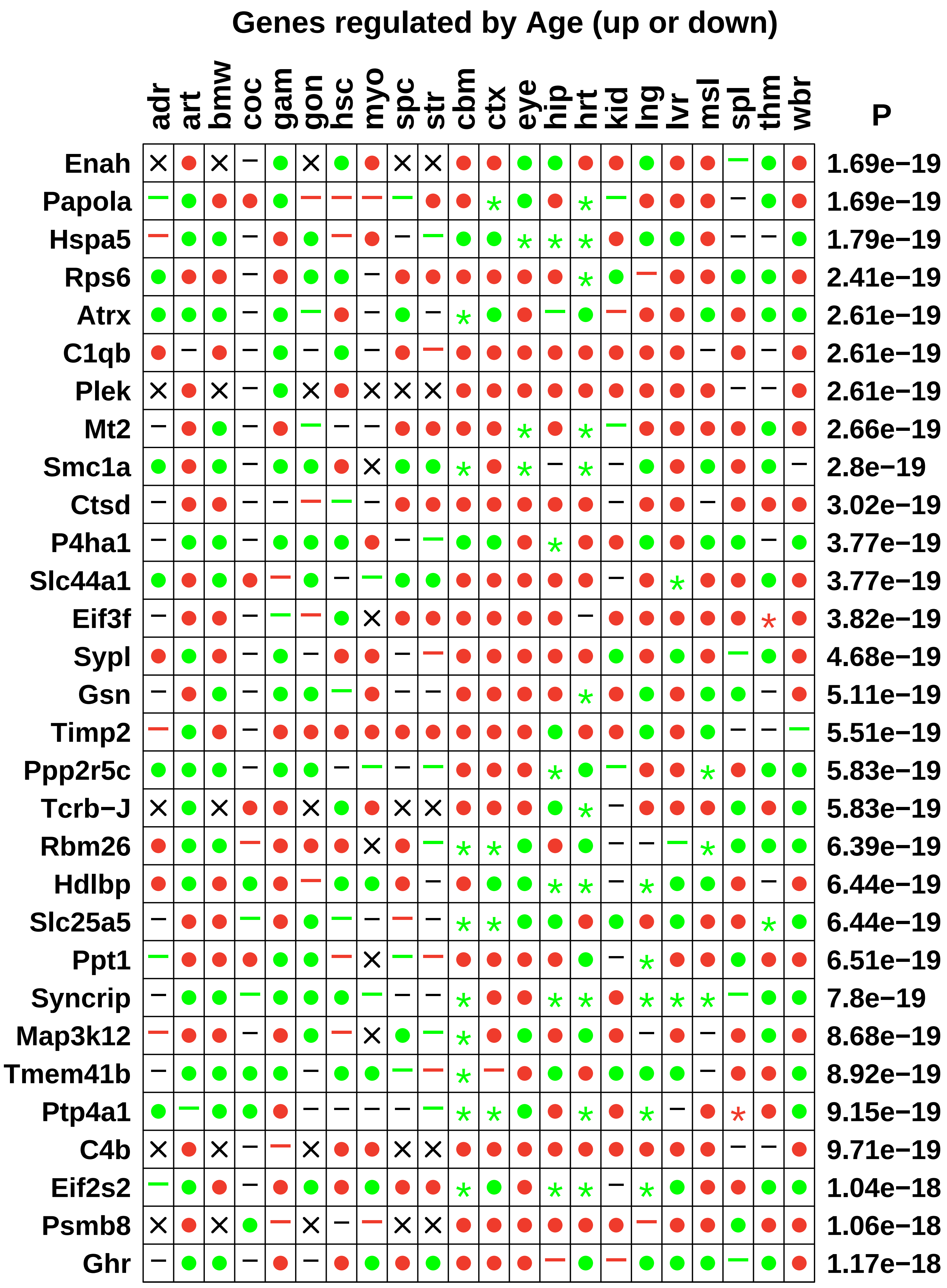
Genes regulated by Age (up or down)
adr
art
bmw
coc
gam
gon
hsc
myo
spc
str
cbm
ctx
eye
hip
hrt
kid
lng
lvr
msl
spl
thm
wbr
P
Enah 1.69e−19
Papola 1.69e−19
Hspa5 1.79e−19
Rps6 2.41e−19
Atrx 2.61e−19
C1qb 2.61e−19
Plek 2.61e−19
Mt2 2.66e−19
Smc1a 2.8e−19
Ctsd 3.02e−19
P4ha1 3.77e−19
Slc44a1 3.77e−19
Eif3f 3.82e−19
Sypl 4.68e−19
Gsn 5.11e−19
Timp2 5.51e−19
Ppp2r5c 5.83e−19
Tcrb−J 5.83e−19
Rbm26 6.39e−19
Hdlbp 6.44e−19
Slc25a5 6.44e−19
Ppt1 6.51e−19
Syncrip 7.8e−19
Map3k12 8.68e−19
Tmem41b 8.92e−19
Ptp4a1 9.15e−19
C4b 9.71e−19
Eif2s2 1.04e−18
Psmb8 1.06e−18
Ghr 1.17e−18

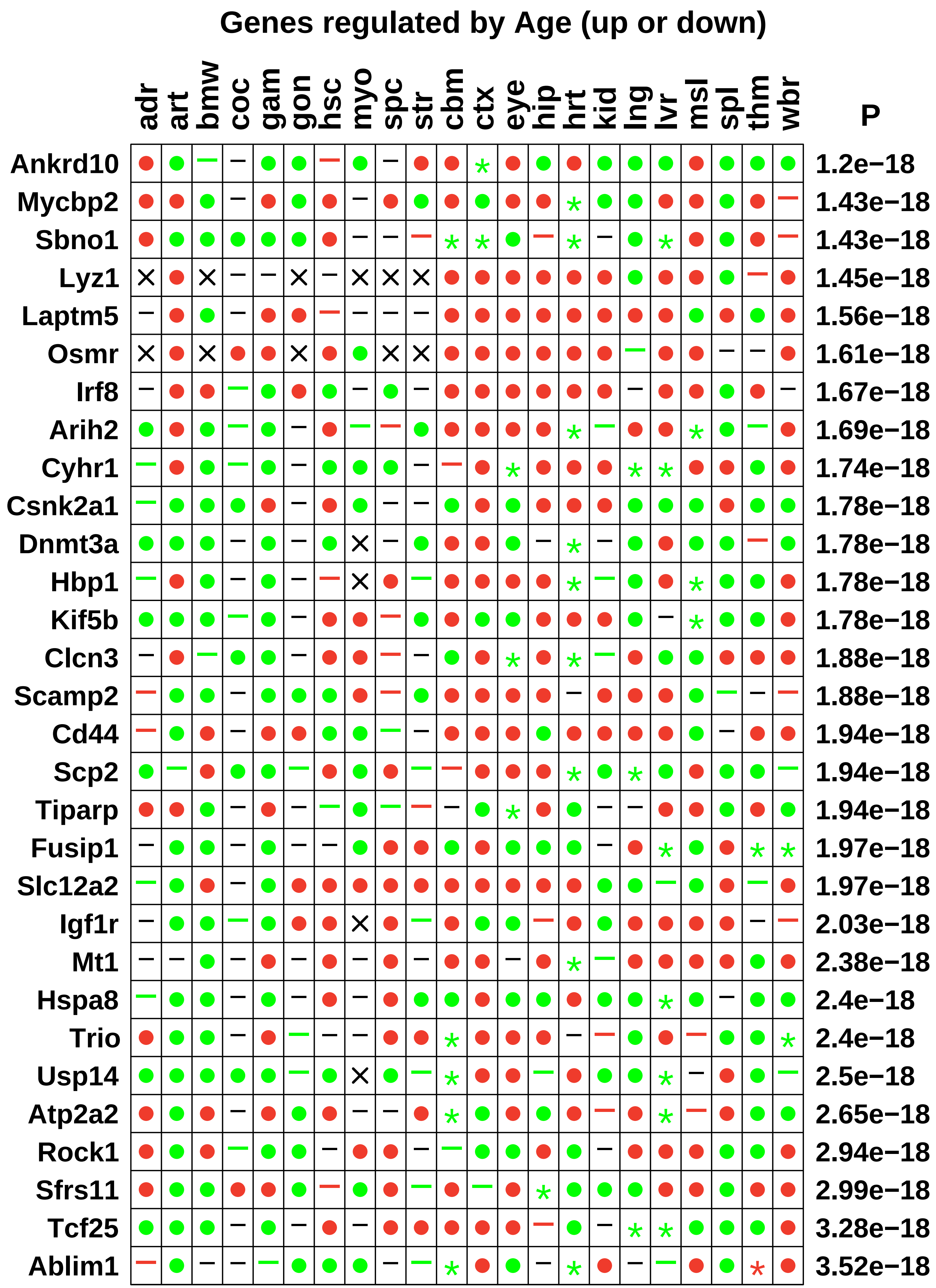
Genes regulated by Age (up or down)
adr
art
bmw
coc
gam
gon
hsc
myo
spc
str
cbm
ctx
eye
hip
hrt
kid
lng
lvr
msl
spl
thm
wbr
P
Ankrd10 1.2e−18
Mycbp2 1.43e−18
Sbno1 1.43e−18
Lyz1 1.45e−18
Laptm5 1.56e−18
Osmr 1.61e−18
Irf8 1.67e−18
Arih2 1.69e−18
Cyhr1 1.74e−18
Csnk2a1 1.78e−18
Dnmt3a 1.78e−18
Hbp1 1.78e−18
Kif5b 1.78e−18
Clcn3 1.88e−18
Scamp2 1.88e−18
Cd44 1.94e−18
Scp2 1.94e−18
Tiparp 1.94e−18
Fusip1 1.97e−18
Slc12a2 1.97e−18
Igf1r 2.03e−18
Mt1 2.38e−18
Hspa8 2.4e−18
Trio 2.4e−18
Usp14 2.5e−18
Atp2a2 2.65e−18
Rock1 2.94e−18
Sfrs11 2.99e−18
Tcf25 3.28e−18
Ablim1 3.52e−18

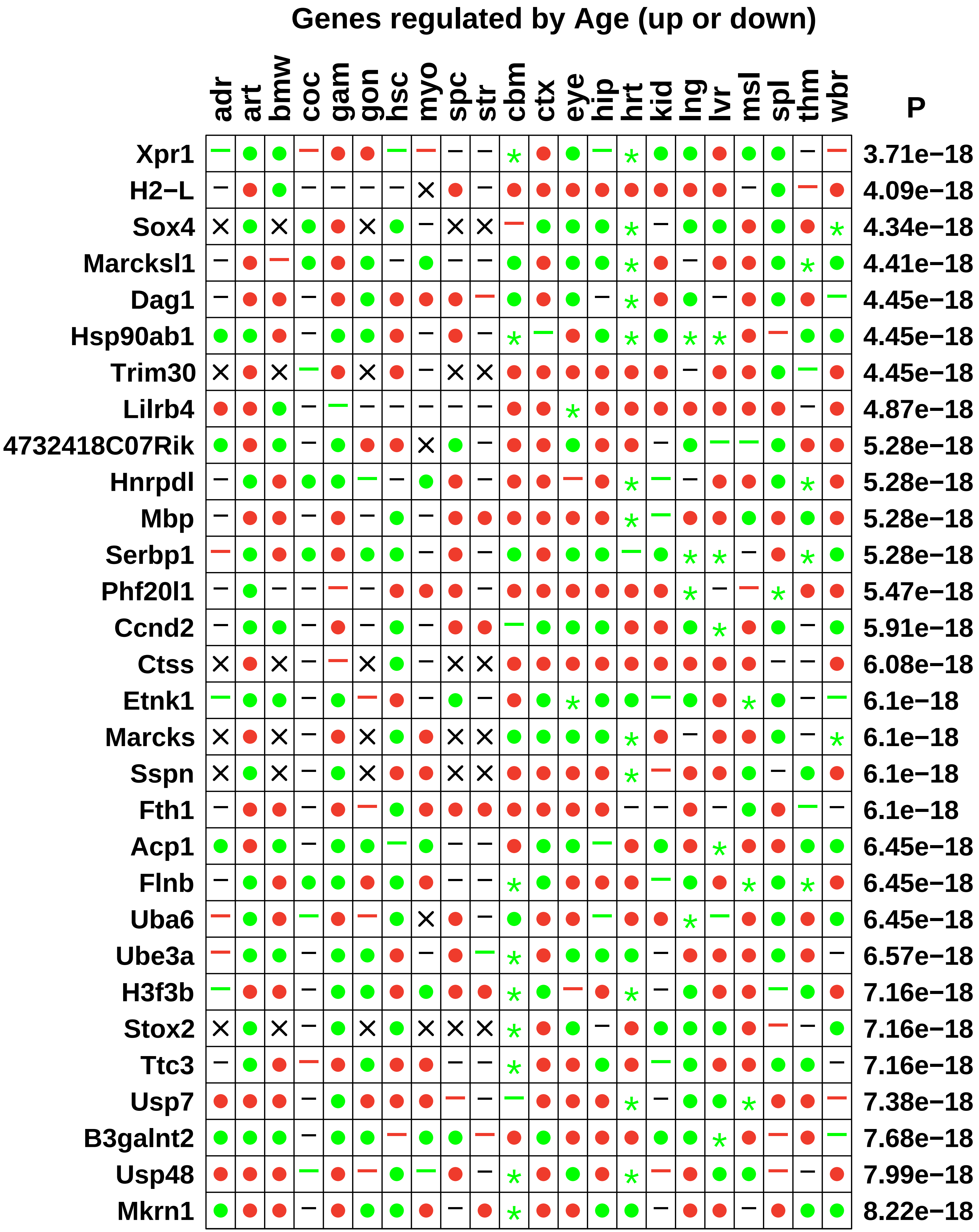

Genes regulated by Age (up or down)
adr
art
bmw
coc
gam
gon
hsc
myo
spc
str
cbm
ctx
eye
hip
hrt
kid
lng
lvr
msl
spl
thm
wbr
P
Xpr1 3.71e−18
H2−L 4.09e−18
Sox4 4.34e−18
Marcksl1 4.41e−18
Dag1 4.45e−18
Hsp90ab1 4.45e−18
Trim30 4.45e−18
Lilrb4 4.87e−18
4732418C07Rik 5.28e−18
Hnrpdl 5.28e−18
Mbp 5.28e−18
Serbp1 5.28e−18
Phf20l1 5.47e−18
Ccnd2 5.91e−18
Ctss 6.08e−18
Etnk1 6.1e−18
Marcks 6.1e−18
Sspn 6.1e−18
Fth1 6.1e−18
Acp1 6.45e−18
Flnb 6.45e−18
Uba6 6.45e−18
Ube3a 6.57e−18
H3f3b 7.16e−18
Stox2 7.16e−18
Ttc3 7.16e−18
Usp7 7.38e−18
B3galnt2 7.68e−18
Usp48 7.99e−18
Mkrn1 8.22e−18

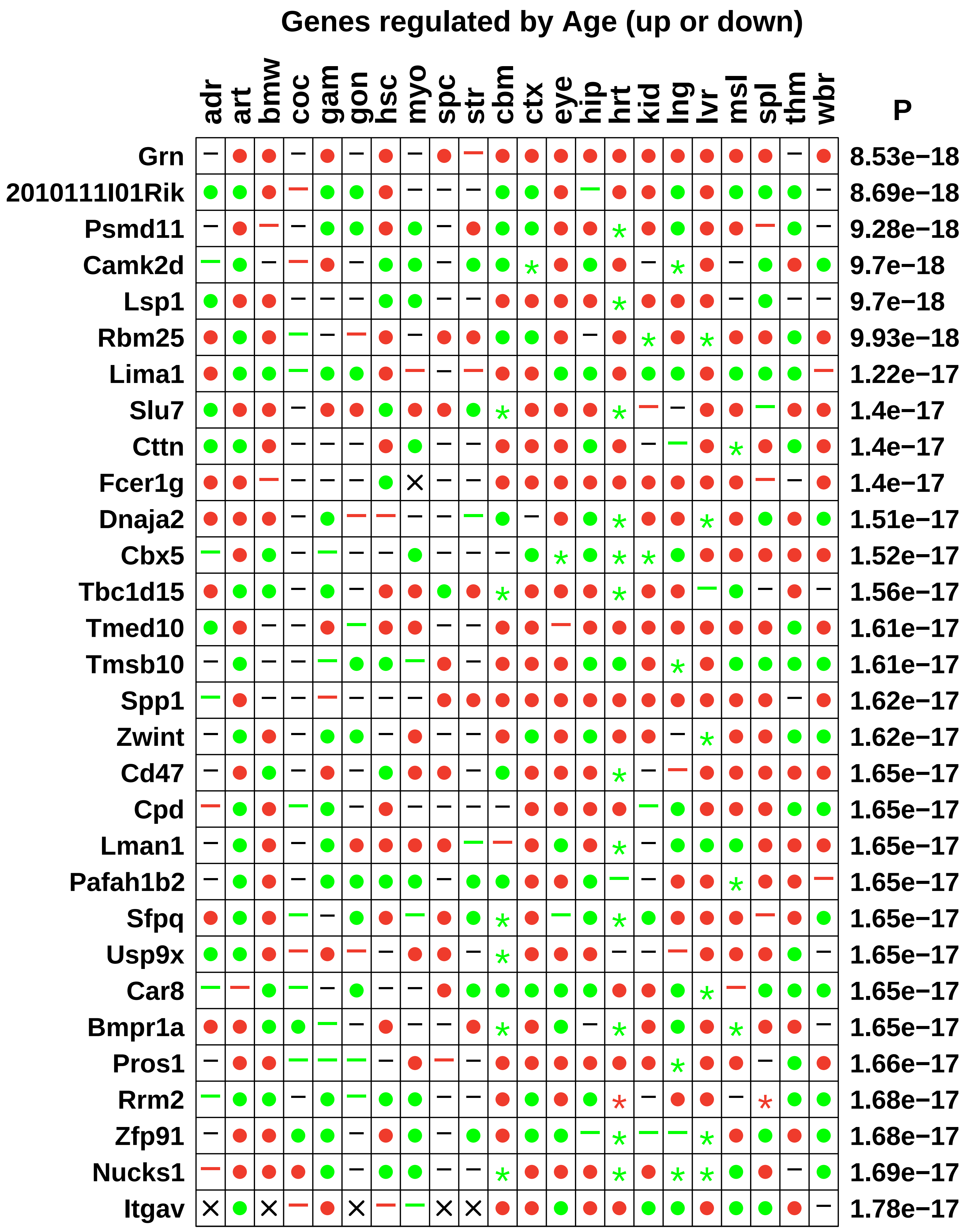
Genes regulated by Age (up or down)
adr art bmw coc gam gon hsc myo spc str cbm ctx eye hip hrt kid lng lvr msl spl thm wbr
P
Grn 8.53e−18
2010111I01Rik 8.69e−18
Psmd11 9.28e−18
Camk2d 9.7e−18
Lsp1 9.7e−18
Rbm25 9.93e−18
Lima1 1.22e−17
Slu7 1.4e−17
Cttn 1.4e−17
Fcer1g 1.4e−17
Dnaja2 1.51e−17
Cbx5 1.52e−17
Tbc1d15 1.56e−17
Tmed10 1.61e−17
Tmsb10 1.61e−17
Spp1 1.62e−17
Zwint 1.62e−17
Cd47 1.65e−17
Cpd 1.65e−17
Lman1 1.65e−17
Pafah1b2 1.65e−17
Sfpq 1.65e−17
Usp9x 1.65e−17
Car8 1.65e−17
Bmpr1a 1.65e−17
Pros1 1.66e−17
Rrm2 1.68e−17
Zfp91 1.68e−17
Nucks1 1.69e−17
Itgav 1.78e−17

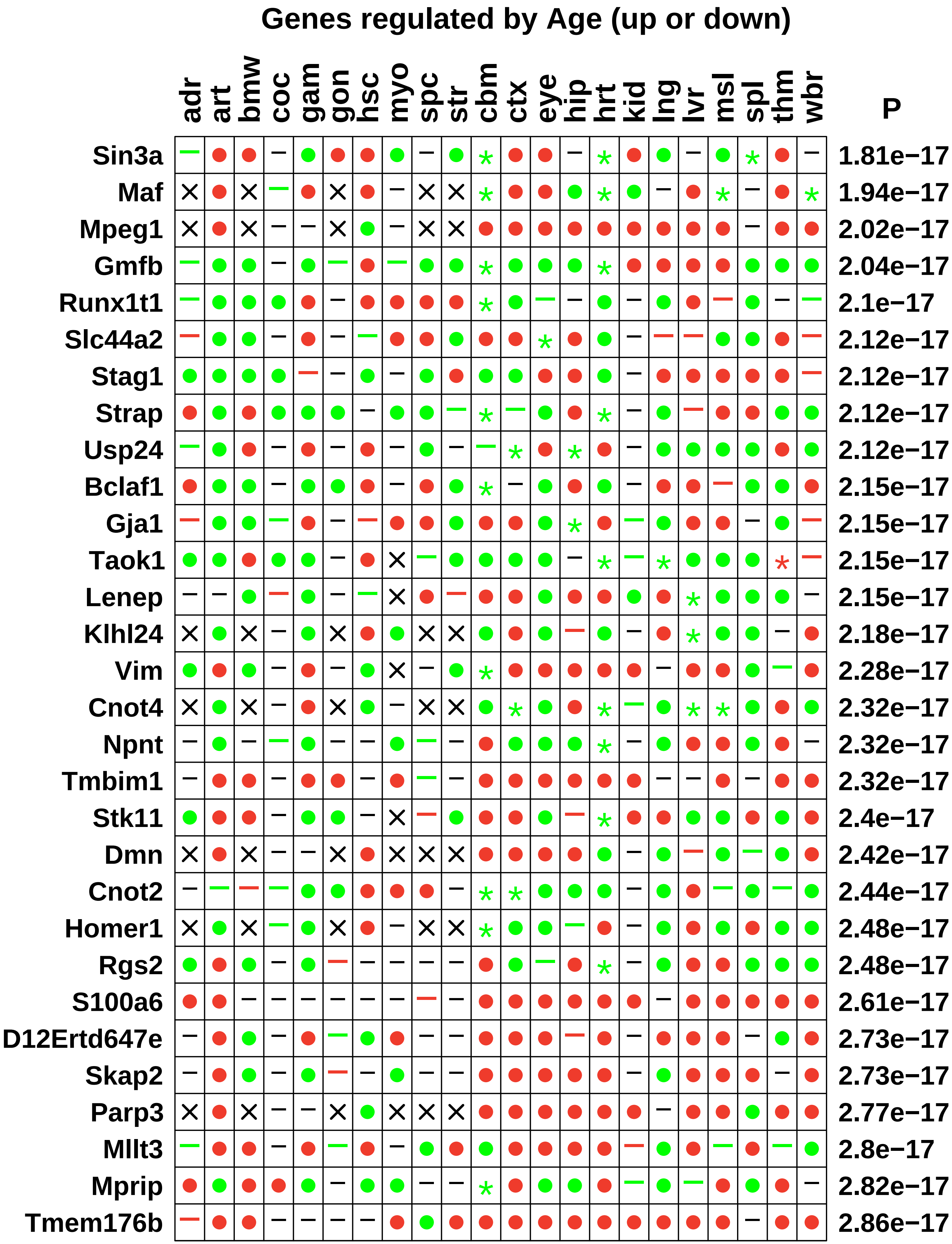

Genes regulated by Age (up or down)
adr
art
bmw
coc
gam
gon
hsc
myo
spc
str
cbm
ctx
eye
hip
hrt
kid
lng
lvr
msl
spl
thm
wbr
P
Sin3a 1.81e−17
Maf 1.94e−17
Mpeg1 2.02e−17
Gmfb 2.04e−17
Runx1t1 2.1e−17
Slc44a2 2.12e−17
Stag1 2.12e−17
Strap 2.12e−17
Usp24 2.12e−17
Bclaf1 2.15e−17
Gja1 2.15e−17
Taok1 2.15e−17
Lenep 2.15e−17
Klhl24 2.18e−17
Vim 2.28e−17
Cnot4 2.32e−17
Npnt 2.32e−17
Tmbim1 2.32e−17
Stk11 2.4e−17
Dmn 2.42e−17
Cnot2 2.44e−17
Homer1 2.48e−17
Rgs2 2.48e−17
S100a6 2.61e−17
D12Ertd647e 2.73e−17
Skap2 2.73e−17
Parp3 2.77e−17
Mllt3 2.8e−17
Mprip 2.82e−17
Tmem176b 2.86e−17

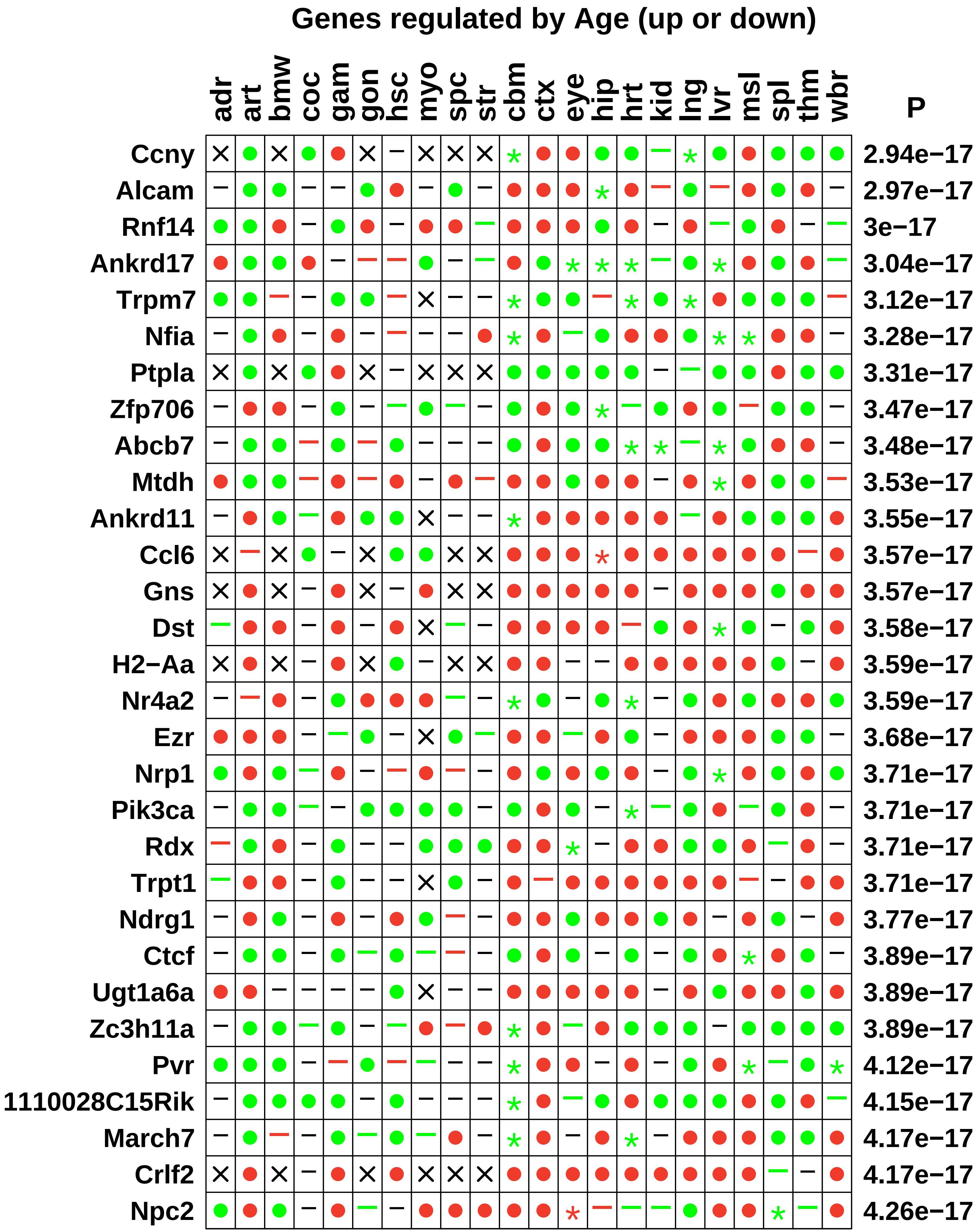
Genes regulated by Age (up or down)
adr
art
bmw
coc
gam
gon
hsc
myo
spc
str
cbm
ctx
eye
hip
hrt
kid
lng
lvr
msl
spl
thm
wbr
P
Ccny
2.94e−17
Alcam
2.97e−17
Rnf14
3e−17
Ankrd17
3.04e−17
Trpm7
3.12e−17
Nfia
3.28e−17
Ptpla
3.31e−17
Zfp706
3.47e−17
Abcb7
3.48e−17
Mtdh
3.53e−17
Ankrd11
3.55e−17
Ccl6
3.57e−17
Gns
3.57e−17
Dst
3.58e−17
H2−Aa
3.59e−17
Nr4a2
3.59e−17
Ezr
3.68e−17
Nrp1
3.71e−17
Pik3ca
3.71e−17
Rdx
3.71e−17
Trpt1
3.71e−17
Ndrg1
3.77e−17
Ctcf
3.89e−17
Ugt1a6a
3.89e−17
Zc3h11a
3.89e−17
Pvr
4.12e−17
1110028C15Rik
4.15e−17
March7
4.17e−17
Crlf2
4.17e−17
Npc2
4.26e−17

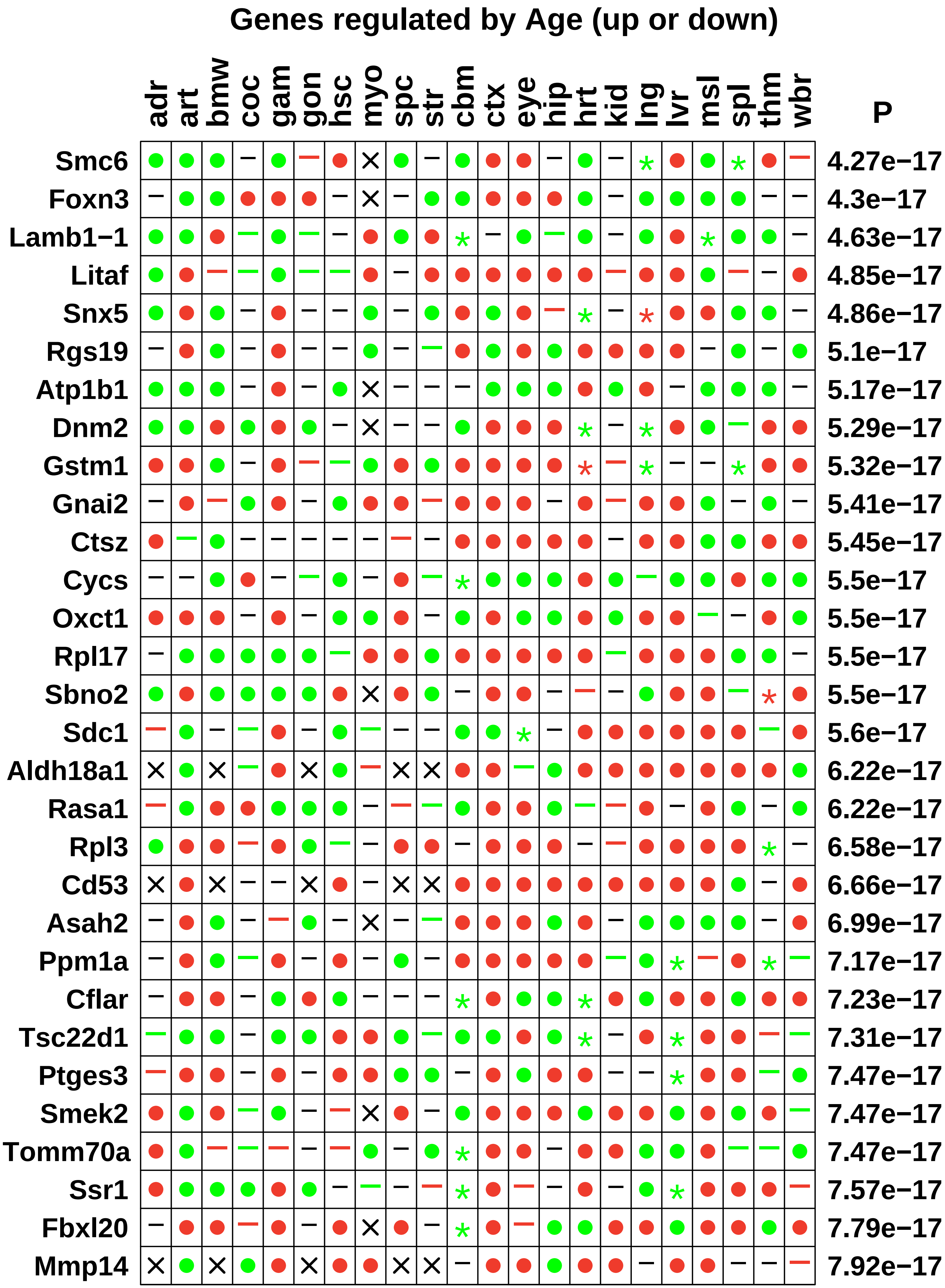

Genes regulated by Age (up or down)
adr
art
bmw
coc
gam
gon
hsc
myo
spc
str
cbm
ctx
eye
hip
hrt
kid
lng
lvr
msl
spl
thm
wbr
P
Smc6 4.27e−17
Foxn3 4.3e−17
Lamb1−1 4.63e−17
Litaf 4.85e−17
Snx5 4.86e−17
Rgs19 5.1e−17
Atp1b1 5.17e−17
Dnm2 5.29e−17
Gstm1 5.32e−17
Gnai2 5.41e−17
Ctsz 5.45e−17
Cycs 5.5e−17
Oxct1 5.5e−17
Rpl17 5.5e−17
Sbno2 5.5e−17
Sdc1 5.6e−17
Aldh18a1 6.22e−17
Rasa1 6.22e−17
Rpl3 6.58e−17
Cd53 6.66e−17
Asah2 6.99e−17
Ppm1a 7.17e−17
Cflar 7.23e−17
Tsc22d1 7.31e−17
Ptges3 7.47e−17
Smek2 7.47e−17
Tomm70a 7.47e−17
Ssr1 7.57e−17
Fbxl20 7.79e−17
Mmp14 7.92e−17

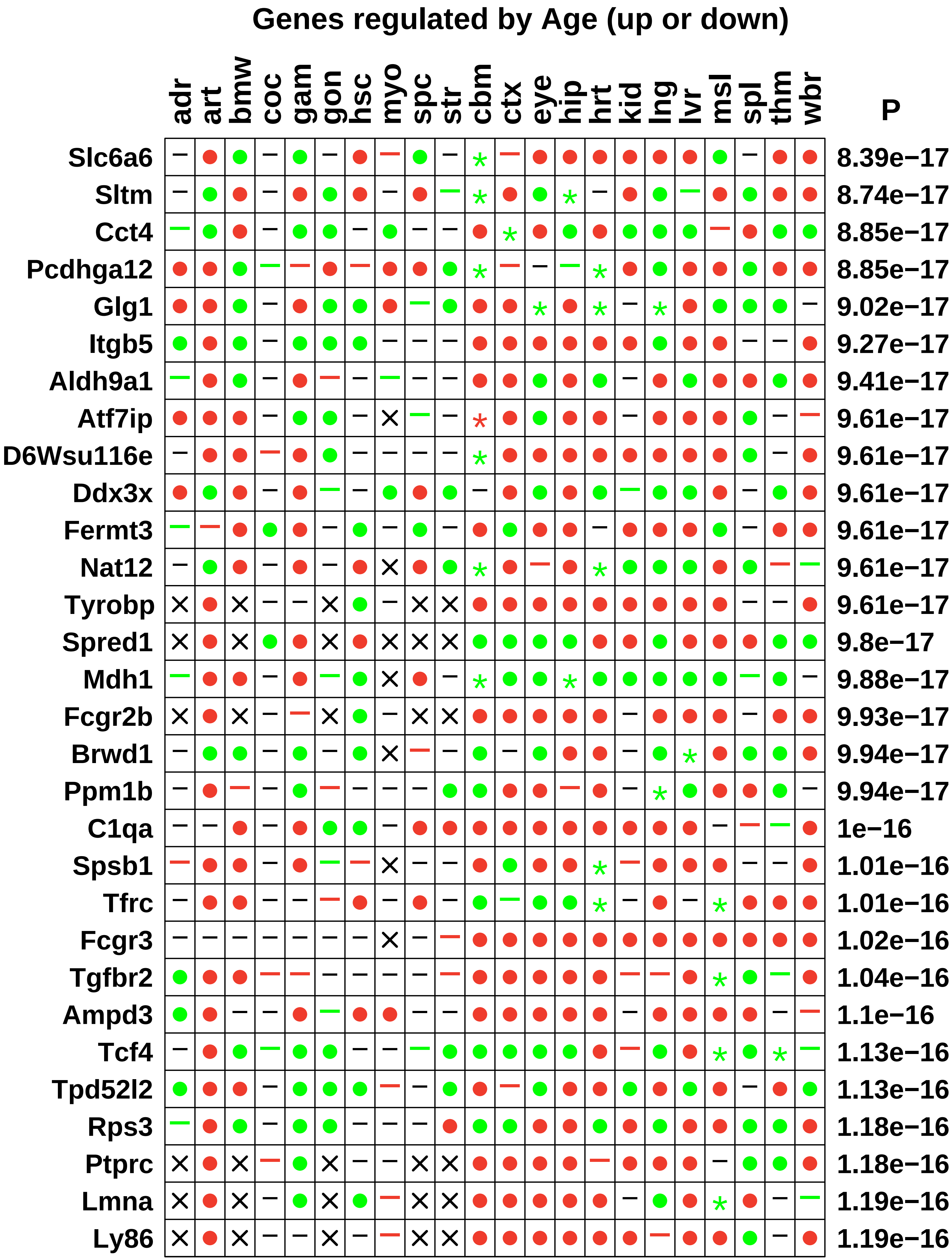
Genes regulated by Age (up or down)
adr
art
bmw
coc
gam
gon
hsc
myo
spc
str
cbm
ctx
eye
hip
hrt
kid
lng
lvr
msl
spl
thm
wbr
P
Slc6a6
Sltm
Cct4
Pcdhga12
Glg1
Itgb5
Aldh9a1
Atf7ip
D6Wsu116e
Ddx3x
Fermt3
Nat12
Tyrobp
Spred1
Mdh1
Fcgr2b
Brwd1
Ppm1b
C1qa
Spsb1
Tfrc
Fcgr3
Tgfbr2
Ampd3
Tcf4
Tpd52l2
Rps3
Ptprc
Lmna
Ly86
8.39e−17
8.74e−17
8.85e−17
8.85e−17
9.02e−17
9.27e−17
9.41e−17
9.61e−17
9.61e−17
9.61e−17
9.61e−17
9.61e−17
9.61e−17
9.8e−17
9.88e−17
9.93e−17
9.94e−17
9.94e−17
1e−16
1.01e−16
1.01e−16
1.02e−16
1.04e−16
1.1e−16
1.13e−16
1.13e−16
1.18e−16
1.18e−16
1.19e−16
1.19e−16

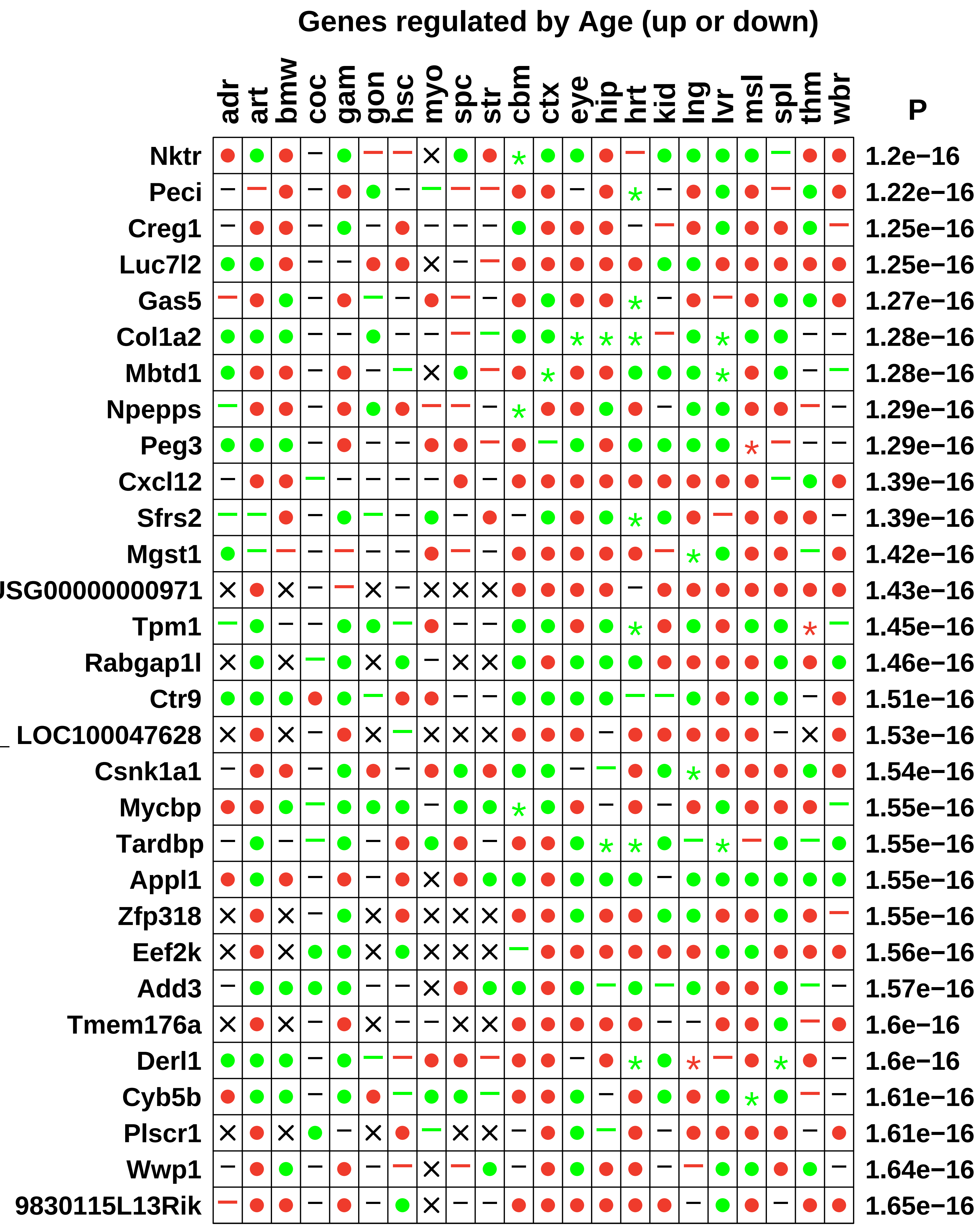
Genes regulated by Age (up or down)
adr
art
bmw
coc
gam
gon
hsc
myo
spc
str
cbm
ctx
eye
hip
hrt
kid
lng
lvr
msl
spl
thm
wbr
P
Nktr 1.2e−16
Peci 1.22e−16
Creg1 1.25e−16
Luc7l2 1.25e−16
Gas5 1.27e−16
Col1a2 1.28e−16
Mbtd1 1.28e−16
Npepps 1.29e−16
Peg3 1.29e−16
Cxcl12 1.39e−16
Sfrs2 1.39e−16
Mgst1 1.42e−16
SG00000000971 1.43e−16
Tpm1 1.45e−16
Rabgap1l 1.46e−16
Ctr9 1.51e−16
LOC100047628 1.53e−16
Csnk1a1 1.54e−16
Mycbp 1.55e−16
Tardbp 1.55e−16
Appl1 1.55e−16
Zfp318 1.55e−16
Eef2k 1.56e−16
Add3 1.57e−16
Tmem176a 1.6e−16
Derl1 1.6e−16
Cyb5b 1.61e−16
Plscr1 1.61e−16
Wwp1 1.64e−16
9830115L13Rik 1.65e−16

↑Age

Overrepresented Biological Processes

GO Term	P−Value
cellular macromolecule metabolic process	1.27e−07
antigen processing and presentation of exogenous antigen	1.36e−07
hemopoietic or lymphoid organ development	6.14e−07
protein metabolic process	1.43e−06
positive regulation of phagocytosis	1.81e−06
membrane organization and biogenesis	1.94e−06
lymphocyte mediated immunity	7.82e−06
immunoglobulin mediated immune response	9.27e−06
humoral immune response	2.27e−05
regulation of endocytosis	2.34e−05
lysosome organization and biogenesis	2.45e−05
regulation of adaptive immune response	2.75e−05
translation	3e−05
intracellular signaling cascade	3.08e−05
immune response	3.47e−05
positive regulation of cell activation	3.76e−05
cell death	4.14e−05
leukocyte adhesion	4.22e−05
positive regulation of lymphocyte activation	6.53e−05
antigen processing and presentation of peptide or polysaccharide antigen via MHC class II	6.65e−05
response to stress	8.1e−05
activation of immune response	9.15e−05
response to oxidative stress	9.56e−05
positive regulation of type IIa hypersensitivity	0.000101
regulation of type II hypersensitivity	0.000101
inflammatory response to antigenic stimulus	0.000109
phagocytosis, engulfment	0.000109
antigen processing and presentation of peptide antigen	0.000123
antigen processing and presentation of exogenous peptide antigen via MHC class II	0.000127
response to external stimulus	0.000196

↑ Age

Overrepresented Biological Processes

GO Term	P−Value
activation of plasma proteins during acute inflammatory response	0.000218
phosphate metabolic process	0.000223
glycolipid metabolic process	0.000243
positive regulation of inflammatory response	0.000243
adaptive immune response based on somatic recombination of immune receptors built from immunoglobul	0.000268
regulation of leukocyte activation	0.000332
positive regulation of B cell mediated immunity	0.000335
leukocyte differentiation	0.000379
hypersensitivity	0.000524
positive regulation of acute inflammatory response to antigenic stimulus	0.000526
protein amino acid N−linked glycosylation via asparagine	0.000526
defense response to Gram−positive bacterium	0.000526
positive regulation of immune effector process	0.000629
response to molecule of bacterial origin	0.000631
antigen processing and presentation of exogenous peptide antigen via MHC class I	0.000637
inflammatory response	0.000708
regulation of defense response	0.000718
positive regulation of cellular component organization and biogenesis	0.000718
iron ion transport	0.000809
complement activation, classical pathway	0.000809
regulation of lymphocyte proliferation	0.000822
response to other organism	0.000842
positive regulation of alpha−beta T cell differentiation	0.000902
regulation of T cell mediated immunity	0.000958
regulation of lymphocyte differentiation	0.001
regulation of catalytic activity	0.00113
immune effector process	0.00114
leukocyte homeostasis	0.00138
positive regulation of tumor necrosis factor production	0.0016
endocytosis	0.00177

↑Age

Overrepresented Biological Processes

GO Term	P−Value
antigen processing and presentation of peptide antigen via MHC class I	0.00177
innate immune response	0.00183
tumor necrosis factor production	0.00186
regulation of leukocyte mediated immunity	0.00186
mannose metabolic process	0.00188
myeloid leukocyte activation	0.00198
organic acid biosynthetic process	0.00198
glutathione metabolic process	0.00201
lipid transport	0.00229
protein modification process	0.00231
positive regulation of immune system process	0.00231
cellular cation homeostasis	0.00235
regulation of small GTPase mediated signal transduction	0.00235
positive regulation of cell−cell adhesion	0.00278
cell adhesion mediated by integrin	0.00278
regulation of B cell activation	0.00297
cellular iron ion homeostasis	0.00302
positive regulation of mononuclear cell proliferation	0.00315
blood vessel development	0.00319
T cell proliferation	0.00329
immune system process	0.00338
positive regulation of transport	0.00349
establishment of localization in cell	0.00351
di−, tri−valent inorganic cation homeostasis	0.0036
myeloid dendritic cell differentiation	0.00371
positive thymic T cell selection	0.00371
ceramide metabolic process	0.00388
positive regulation of type III hypersensitivity	0.00401
response to molecule of fungal origin	0.00401
purine nucleoside diphosphate metabolic process	0.00401

↑ Age

Overrepresented Biological Processes

GO Term	P−Value
purine ribonucleoside diphosphate catabolic process	0.00401
detoxification of copper ion	0.00401
ISG15−protein conjugation	0.00401
homotypic cell−cell adhesion	0.00401
positive regulation of interleukin−1 alpha secretion	0.00401
patterning of blood vessels	0.0044
mononuclear cell proliferation	0.00441
response to chemical stimulus	0.00458
chemotaxis	0.00459
Rac protein signal transduction	0.00501
regulation of response to stimulus	0.00503
Ras protein signal transduction	0.00509
T cell selection	0.00584
positive regulation of adaptive immune response based on somatic recombination of immune receptors bu	0.00613
prostanoid metabolic process	0.00621
regulation of alpha−beta T cell activation	0.00629
cytokine and chemokine mediated signaling pathway	0.00646
macromolecular complex subunit organization	0.00662
actin cytoskeleton organization and biogenesis	0.00686
phagocytosis, recognition	0.00725
positive regulation of T cell mediated cytotoxicity	0.00729
myeloid cell differentiation	0.00741
positive regulation of T cell proliferation	0.00804
locomotory behavior	0.00813
glycosaminoglycan metabolic process	0.0084
negative regulation of apoptosis	0.00875
erythrocyte differentiation	0.00882
acute inflammatory response	0.00919
actin filament−based process	0.00952
T cell differentiation in the thymus	0.00961

↑Age

Overrepresented Biological Processes

GO Term	P−Value
alpha−beta T cell activation	0.00985
response to metal ion	0.01
protein amino acid phosphorylation	0.0102
sphingolipid metabolic process	0.0102
regulation of cell adhesion	0.0103
Golgi vesicle transport	0.0104
leukocyte migration	0.0104
protein transport	0.0105
T cell differentiation	0.0106
protein amino acid dephosphorylation	0.0106
vitamin metabolic process	0.0108
protein localization	0.0111
cholesterol transport	0.0113
positive regulation of B cell proliferation	0.0113
interleukin−6 production	0.0113
homeostasis of number of cells	0.0115
insulin receptor signaling pathway	0.0115
apoptosis	0.0116
cell communication	0.012
positive regulation of protein kinase activity	0.0121
regulation of response to external stimulus	0.0121
cytokine metabolic process	0.0122
cellular lipid metabolic process	0.0123
cellular chemical homeostasis	0.0125
endoplasmic reticulum unfolded protein response	0.0126
regulation of cytokine biosynthetic process	0.013
regulation of T cell activation	0.0134
positive regulation of transferase activity	0.0138
positive regulation of type I hypersensitivity	0.0141
blastocyst hatching	0.0141

↑Age

Overrepresented Biological Processes

GO Term	P−Value
neuron remodeling	0.0141
actin cytoskeleton reorganization	0.0141
gamma−delta T cell differentiation	0.0141
positive regulation of tumor necrosis factor biosynthetic process	0.0141
long−chain fatty acid biosynthetic process	0.0141
apoptotic cell clearance	0.0141
nucleobase biosynthetic process	0.0141
regulation of interleukin−1 secretion	0.0141
positive regulation of interleukin−1 beta secretion	0.0141
response to unfolded protein	0.0143
angiogenesis	0.0145
ganglioside metabolic process	0.0149
innate immune response−activating signal transduction	0.0149
interleukin−10 production	0.0149
norepinephrine metabolic process	0.0149
positive regulation of B cell differentiation	0.0149
regulation of mitochondrial membrane potential	0.0149
regulation of cytokine production	0.015
DNA damage response, signal transduction	0.015
NADP metabolic process	0.0152
mast cell activation	0.0152
actin filament bundle formation	0.0152
positive regulation of NF−kappaB transcription factor activity	0.0152
regulation of programmed cell death	0.0156
positive regulation of cell migration	0.0159
cell activation during immune response	0.016
aging	0.0165
cellular carbohydrate metabolic process	0.017
regulation of kinase activity	0.0173
regulation of organelle organization and biogenesis	0.0176

↑Age

Overrepresented Biological Processes

GO Term	P−Value
leukocyte activation	0.0185
cellular catabolic process	0.0186
regulation of biological quality	0.0188
ion homeostasis	0.0192
antigen processing and presentation	0.0193
glutamate signaling pathway	0.0201
axon cargo transport	0.0201
sphingolipid catabolic process	0.0201
regulation of pigmentation during development	0.0201
positive regulation of calcium−mediated signaling	0.0201
establishment and/or maintenance of cell polarity	0.0211
activated T cell proliferation	0.022
intracellular receptor−mediated signaling pathway	0.022
T cell homeostasis	0.022
response to virus	0.0225
regulation of Rho protein signal transduction	0.0228
cellular process	0.0234
cellular structure morphogenesis	0.0235
lipid catabolic process	0.0237
positive regulation of immune response	0.024
protein complex assembly	0.0244
cytokine production	0.0248
positive regulation of programmed cell death	0.025
regulation of acute inflammatory response to antigenic stimulus	0.0251
positive regulation of myeloid leukocyte mediated immunity	0.0252
G2 phase of mitotic cell cycle	0.0253
antral ovarian follicle growth	0.0253
establishment of T cell polarity	0.0253
antibody−dependent cellular cytotoxicity	0.0253
phagolysosome formation	0.0253

↑ Age

Overrepresented Biological Processes

GO Term	P−Value
lymphangiogenesis	0.0253
angiotensin mediated vasoconstriction involved in regulation of systemic arterial blood pressure	0.0253
negative regulation of acute inflammatory response	0.0253
cell surface pattern recognition receptor signaling pathway	0.0253
MyD88−dependent toll−like receptor signaling pathway	0.0253
negative regulation of hypersensitivity	0.0253
acetate metabolic process	0.0253
'de novo' IMP biosynthetic process	0.0253
phosphatidic acid biosynthetic process	0.0253
lysosomal lumen acidification	0.0253
nitric oxide mediated signal transduction	0.0253
pentose biosynthetic process	0.0253
glucuronate metabolic process	0.0253
vesicle transport along actin filament	0.0253
locomotion during locomotory behavior	0.0253
myelin formation	0.0253
uropod organization and biogenesis	0.0253
phospholipid efflux	0.0253
amino acid import	0.0253
regulation of monocyte differentiation	0.0253
spermine catabolic process	0.0253
glycosylceramide catabolic process	0.0253
positive regulation of filopodium formation	0.0253
L−glutamate import	0.0253
prostaglandin biosynthetic process	0.026
response to exogenous dsRNA	0.026
homeostasis of number of cells within a tissue	0.026
ventricular cardiac muscle morphogenesis	0.026
protein kinase cascade	0.0291
embryonic placenta development	0.0293

↑Age

Overrepresented Biological Processes

GO Term	P−Value
nucleotide catabolic process	0.0293
response to lipopolysaccharide	0.0293
positive regulation of T cell differentiation	0.0297
response to osmotic stress	0.0301
neutrophil chemotaxis	0.0301
vesicle localization	0.0301
organelle organization and biogenesis	0.0303
activation of MAPK activity	0.0303
lymphocyte activation during immune response	0.0305
receptor−mediated endocytosis	0.0305
CD4−positive, alpha beta T cell differentiation	0.0305
oocyte maturation	0.0312
mature B cell differentiation	0.0312
purine base metabolic process	0.0312
nuclear membrane organization and biogenesis	0.0312
cell−substrate junction assembly	0.0312
ribonucleoside diphosphate metabolic process	0.0312
regulation of cell projection organization and biogenesis	0.0312
bleb formation	0.0312
regulation of interleukin−1 beta production	0.0312
lipoprotein catabolic process	0.0312
embryonic viscerocranium morphogenesis	0.0312
positive regulation of cytokine secretion	0.0312
cellular protein catabolic process	0.0313
regulation of tissue remodeling	0.0313
multicellular organismal homeostasis	0.0313
regulation of action potential	0.032
actin polymerization and/or depolymerization	0.0332
protein depolymerization	0.0343
pyridine nucleotide metabolic process	0.0347

↑Age

Overrepresented Biological Processes

GO Term	P−Value
negative regulation of mononuclear cell proliferation	0.0347
lipid homeostasis	0.0347
localization	0.0352
positive regulation of cell differentiation	0.0356
positive regulation of apoptosis	0.0361
cytoskeleton−dependent intracellular transport	0.0371
regulation of cell proliferation	0.0372
cell proliferation	0.0373
regulation of cell shape	0.0379
alcohol metabolic process	0.0381
developmental maturation	0.0385
oxygen and reactive oxygen species metabolic process	0.0386
fatty acid biosynthetic process	0.0391
cellular response to stimulus	0.0394
monocarboxylic acid metabolic process	0.0395
aromatic compound metabolic process	0.0405
regulation of anatomical structure morphogenesis	0.0405
vitamin A metabolic process	0.041
amino acid derivative catabolic process	0.041
negative regulation of B cell proliferation	0.0411
negative thymic T cell selection	0.0411
muscle maintenance	0.0411
defense response to virus	0.0411
neurofilament cytoskeleton organization and biogenesis	0.0411
negative regulation of cytoskeleton organization and biogenesis	0.0414
regulation of signal transduction	0.0416
monosaccharide metabolic process	0.0427
translational initiation	0.047
alcohol biosynthetic process	0.047
respiratory gaseous exchange	0.0484

↑Age

Overrepresented Biological Processes

GO Term	P−Value
cholesterol homeostasis	0.0484
positive regulation of secretion	0.0484
negative regulation of cell activation	0.0494
negative regulation of lymphocyte activation	0.0494

↑Age

Overrepresented Cell Components

GO Term	P−Value
cytoplasm	4.24e−23
vacuole	1.97e−10
lysosome	1.72e−09
cytosol	1.01e−06
intracellular	4.49e−06
ribosome	4.04e−05
external side of plasma membrane	5.39e−05
endosome	8.55e−05
non−membrane−bounded organelle	0.000224
cell	0.000417
soluble fraction	0.00045
oligosaccharyl transferase complex	0.000546
vesicle	0.000663
stress fiber	0.000967
actin cytoskeleton	0.000981
axon	0.00106
uropod	0.0024
MHC class I protein complex	0.00247
plasma membrane	0.0025
cytoplasmic membrane−bounded vesicle	0.00273
Golgi apparatus	0.00273
nuclear envelope−endoplasmic reticulum network	0.00554
MHC class II protein complex	0.00612
growth cone	0.00665
early endosome	0.00665
coated pit	0.00846
ruffle	0.00996
melanosome	0.0107
cell cortex	0.0118
small ribosomal subunit	0.0127

↑Age

Overrepresented Cell Components

GO Term	P−Value
nuclear envelope	0.0138
trans−Golgi network	0.0157
membrane fraction	0.0164
insoluble fraction	0.0166
actin filament	0.018
membrane raft	0.0204
multivesicular body	0.0228
podosome	0.0234
2−acetyl−1−alkylglycerophosphocholine esterase complex	0.0234
flotillin complex	0.0234
nucleolar preribosome, small subunit precursor	0.0234
platelet dense granule membrane	0.0234
anchored to external side of plasma membrane	0.0234
immunoglobulin complex, circulating	0.0234
immunological synapse	0.0242
filopodium	0.0258
cortical actin cytoskeleton	0.0258
cortical cytoskeleton	0.0279
zymogen granule	0.0281
cell−cell adherens junction	0.0282
intrinsic to endoplasmic reticulum membrane	0.0282
adherens junction	0.0324
cell−substrate junction	0.0328
cytoskeleton	0.0341
stereocilium	0.0362
focal adhesion	0.0439
extrinsic to membrane	0.0466
cell projection	0.0478
endoplasmic reticulum	0.0483
intracellular organelle	0.0494

↑Age

Overrepresented Cell Components

GO Term	P−Value
secretory granule membrane	0.0497
SWI/SNF complex	0.0498

↑Age

Overrepresented Molecular Functions

GO Term	P−Value
structural constituent of ribosome	9.29e−07
magnesium ion binding	5.43e−06
actin binding	2.11e−05
nucleotide binding	7.06e−05
protein binding	8.99e−05
GTP binding	0.00011
guanyl nucleotide binding	0.00012
purine ribonucleotide binding	0.000136
enzyme regulator activity	0.000188
catalytic activity	0.000293
lipid binding	0.000447
heparin binding	0.000454
phosphotransferase activity, alcohol group as acceptor	0.000719
protease inhibitor activity	0.000768
Rho guanyl−nucleotide exchange factor activity	0.00126
antigen binding	0.0014
alpha−mannosidase activity	0.00141
dolichyl−diphosphooligosaccharide−protein glycotransferase activity	0.00141
chemokine activity	0.00145
small GTPase regulator activity	0.00169
hydrolase activity, hydrolyzing O−glycosyl compounds	0.00179
rRNA binding	0.00212
1−alkyl−2−acetylglycerophosphocholine esterase activity	0.0025
iron ion transmembrane transporter activity	0.0025
metalloendopeptidase inhibitor activity	0.00328
cysteine protease inhibitor activity	0.00365
opsonin binding	0.0037
xenobiotic−transporting ATPase activity	0.0037
G−protein−coupled receptor binding	0.00411
GTPase activity	0.0044

↑Age

Overrepresented Molecular Functions

GO Term	P−Value
calcium−dependent phospholipid binding	0.00493
phosphatase activity	0.00536
kinase activity	0.00572
immunoglobulin binding	0.00643
oxidoreductase activity, acting on paired donors, with oxidation of a pair of donors resulting in the reductio[illegible]	0.00659
polysaccharide binding	0.00762
adenyl nucleotide binding	0.00904
oxidoreductase activity, acting on the aldehyde or oxo group of donors, NAD or NADP as acceptor	0.00969
nucleoside−diphosphatase activity	0.0112
hydrolase activity, acting on acid anhydrides	0.0127
peptide antigen binding	0.0131
ceramidase activity	0.0135
glutathione transferase activity	0.0136
hydrolase activity, acting on ester bonds	0.0137
protein tyrosine phosphatase activity	0.0154
ATP binding	0.0163
enzyme binding	0.0165
dipeptidase activity	0.018
SNAP receptor activity	0.0194
phospholipid transporter activity	0.0194
phosphoinositide binding	0.0204
antioxidant activity	0.0208
cysteine−type peptidase activity	0.0228
small GTPase binding	0.023
primary active transmembrane transporter activity	0.0234
carboxy−lyase activity	0.0235
ferric iron binding	0.0237
intramolecular oxidoreductase activity, interconverting aldoses and ketoses	0.0237
protease binding	0.0239
5−aminolevulinate synthase activity	0.0239

↑Age

Overrepresented Molecular Functions

GO Term	P−Value
biliverdin reductase activity	0.0239
epoxide hydrolase activity	0.0239
alpha−L−fucosidase activity	0.0239
phosphatidate cytidylyltransferase activity	0.0239
transferrin receptor activity	0.0239
IgG receptor activity	0.0239
structural constituent of myelin sheath	0.0239
heparan sulfate proteoglycan binding	0.0239
eukaryotic cell surface binding	0.0239
integrin binding	0.0256
ubiquitin thiolesterase activity	0.0277
kinase regulator activity	0.0277
pyrophosphatase activity	0.028
protein serine/threonine kinase activity	0.0283
microfilament motor activity	0.0289
aldehyde reductase activity	0.0289
beta−N−acetylhexosaminidase activity	0.0289
phospholipase inhibitor activity	0.0289
GDP−dissociation inhibitor activity	0.0289
translation repressor activity	0.0289
cysteine−type endopeptidase activity	0.029
transferase activity	0.0295
calcium ion binding	0.0314
transferase activity, transferring pentosyl groups	0.0351
small conjugating protein−specific protease activity	0.0368
hematopoietin/interferon−class (D200−domain) cytokine receptor activity	0.037
actin filament binding	0.0376
ATPase activity, coupled to transmembrane movement of substances	0.0394
guanyl−nucleotide exchange factor activity	0.0457
aminopeptidase activity	0.0465

↑Age

Overrepresented Molecular Functions

GO Term	P−Value
protein kinase inhibitor activity	0.0477
lipoprotein binding	0.0477
Rho GTPase binding	0.0477
protein dimerization activity	0.048

↓Age

Overrepresented Biological Processes

GO Term	P−Value
biopolymer metabolic process	4.92e−23
metabolic process	7.37e−13
macromolecule biosynthetic process	3.55e−11
chromosome organization and biogenesis	9.04e−09
nucleobase, nucleoside, nucleotide and nucleic acid metabolic process	1.14e−08
cellular macromolecule metabolic process	8.95e−08
phosphorus metabolic process	2.73e−07
mRNA processing	6.51e−07
regulation of transcription, DNA−dependent	9.75e−07
RNA biosynthetic process	1.13e−06
RNA splicing	1.22e−06
protein folding	1.65e−06
protein modification process	2.09e−06
protein metabolic process	3.29e−06
regulation of macromolecule metabolic process	9.38e−06
mitotic cell cycle	2.66e−05
regulation of biosynthetic process	2.79e−05
regulation of cellular metabolic process	3.48e−05
histone methylation	7.41e−05
DNA replication	0.000104
protein modification by small protein conjugation	0.000114
mitosis	0.000144
regulation of gene expression	0.000166
cell cycle	0.000208
cell division	0.000262
translational initiation	0.000328
covalent chromatin modification	0.000346
cellular macromolecular complex assembly	0.000355
one−carbon compound metabolic process	0.000385
positive regulation of RNA metabolic process	0.000408

↓Age

Overrepresented Biological Processes

GO Term	P−Value
ATP metabolic process	0.000411
macromolecular complex subunit organization	0.000416
positive regulation of transcription from RNA polymerase II promoter	0.000424
biopolymer methylation	0.00045
phosphorylation	0.000462
chromatin assembly or disassembly	0.000465
ribonucleoprotein complex biogenesis and assembly	0.000685
protein amino acid autophosphorylation	0.000787
chromatin modification	0.000953
ATP synthesis coupled proton transport	0.00098
positive regulation of transcription	0.00109
layer formation in the cerebral cortex	0.00139
purine ribonucleoside triphosphate biosynthetic process	0.00143
transmembrane ion transport	0.00151
negative regulation of cellular metabolic process	0.00171
protein amino acid phosphorylation	0.00173
ribonucleoside triphosphate metabolic process	0.00177
proteolysis involved in cellular protein catabolic process	0.00186
ubiquitin−dependent protein catabolic process	0.00224
nucleoside triphosphate biosynthetic process	0.00242
modification−dependent macromolecule catabolic process	0.00283
regulation of transcription	0.00338
positive regulation of biosynthetic process	0.00345
purine nucleoside triphosphate metabolic process	0.00349
purine ribonucleotide metabolic process	0.00389
regulation of protein metabolic process	0.00396
genetic imprinting	0.0041
axonal fasciculation	0.0041
negative regulation of osteoblast differentiation	0.0041
ribonucleotide biosynthetic process	0.00453

↓Age

Overrepresented Biological Processes

GO Term	P−Value
negative regulation of caspase activity	0.0051
transcription	0.00522
purine nucleotide biosynthetic process	0.00544
actin polymerization and/or depolymerization	0.00544
regulation of biological process	0.00548
M phase	0.00599
positive regulation of cellular metabolic process	0.00649
DNA repair	0.00661
protein amino acid alkylation	0.0068
gene expression	0.00681
DNA packaging	0.0073
cerebral cortex radially oriented cell migration	0.00749
protein processing	0.00774
RNA processing	0.00846
negative regulation of RNA metabolic process	0.00917
negative regulation of translation	0.0103
protein depolymerization	0.0104
positive regulation of macromolecule metabolic process	0.0107
cellular macromolecular complex disassembly	0.0108
protein complex disassembly	0.0108
coenzyme A biosynthetic process	0.0117
histone ubiquitination	0.0117
smooth muscle development	0.0117
protein targeting	0.0118
response to activity	0.0125
fatty acid elongation	0.0125
cell cycle process	0.0125
negative regulation of cytoskeleton organization and biogenesis	0.0126
cell motility	0.0133
chromatin remodeling	0.0151

↓Age

Overrepresented Biological Processes

GO Term	P−Value
regulation of actin filament length	0.0152
peptidyl−amino acid modification	0.0156
ribosome biogenesis	0.0174
negative regulation of biosynthetic process	0.0179
negative regulation of protein metabolic process	0.018
nitric oxide biosynthetic process	0.0186
DNA alkylation	0.0187
peptidyl−threonine phosphorylation	0.0187
peptidyl−serine phosphorylation	0.0187
protein catabolic process	0.019
regulation of translation	0.0196
telencephalon cell migration	0.0199
nucleosome assembly	0.02
regulation of actin filament−based process	0.0209
mitochondrial genome maintenance	0.0214
dosage compensation	0.0214
glutamate secretion	0.0214
response to cytokine stimulus	0.0214
rRNA processing	0.0229
proton transport	0.023
electron transport chain	0.0238
regulation of cellular biosynthetic process	0.0246
positive regulation of myeloid cell differentiation	0.0263
response to dsRNA	0.0268
nuclear export	0.0297
nuclear transport	0.0301
negative regulation of transcription from RNA polymerase II promoter	0.0307
response to protein stimulus	0.0341
positive regulation of nitric oxide biosynthetic process	0.0344
cardiac muscle contraction	0.0344

↓Age

Overrepresented Biological Processes

GO Term	P−Value
acrosome formation	0.0346
mitochondrial DNA replication	0.0346
L−serine biosynthetic process	0.0346
nucleolus organization and biogenesis	0.0346
SMAD protein nuclear translocation	0.0346
maintenance of DNA methylation	0.0346
regulation of microtubule polymerization	0.0346
negative regulation of protein ubiquitination	0.0346
gene silencing by miRNA, production of miRNAs	0.0346
regulation of macrophage activation	0.0346
ATP−dependent chromatin remodeling	0.0346
stress fiber formation	0.0346
axial mesoderm development	0.0346
positive regulation of smooth muscle cell proliferation	0.0346
centrosome localization	0.0346
regulation of organelle organization and biogenesis	0.0348
G1/S transition of mitotic cell cycle	0.0365
regulation of actin filament depolymerization	0.0365
inositol metabolic process	0.0368
positive regulation of organelle organization and biogenesis	0.0368
lipopolysaccharide−mediated signaling pathway	0.0368
ATP synthesis coupled electron transport	0.0368
negative regulation of transcription	0.038
negative regulation of cellular component organization and biogenesis	0.0395
negative regulation of macromolecule metabolic process	0.0409
regulation of protein kinase activity	0.041
cellular component organization and biogenesis	0.0418
regulation of intracellular protein transport	0.0428
regulation of nitrogen compound metabolic process	0.0428
axon guidance	0.0436

↓Age

Overrepresented Biological Processes

GO Term	P−Value
regulation of catalytic activity	0.0443
steroid hormone receptor signaling pathway	0.0486

↓Age

Overrepresented Cell Components

GO Term	P−Value
organelle	3.99e−33
nucleus	3.73e−30
intracellular part	6.02e−28
cell	3.05e−16
cytoplasm	7.69e−12
envelope	4.75e−07
mitochondrial envelope	9.13e−07
chromosome	4.31e−06
intracellular organelle part	7.3e−06
membrane−enclosed lumen	9.16e−06
mitochondrial respiratory chain	1.41e−05
chromatin	4.06e−05
cytosol	8.47e−05
macromolecular complex	9.84e−05
nuclear lumen	0.000103
organelle membrane	0.000123
lamellipodium	0.000496
sex chromosome	0.000502
nuclear heterochromatin	0.000572
proton−transporting ATP synthase complex, catalytic core F(1)	0.000624
mitochondrial inner membrane	0.0012
ESC/E(Z) complex	0.00127
chaperonin−containing T−complex	0.00171
nuclear chromosome part	0.0023
mitochondrion	0.00255
collagen	0.00267
Barr body	0.00465
proton−transporting two−sector ATPase complex	0.00481
filopodium	0.00608
endoplasmic reticulum lumen	0.00668

↓Age

Overrepresented Cell Components

GO Term	P−Value
ubiquitin ligase complex	0.0068
spliceosome	0.00752
ribonucleoprotein complex	0.00761
intracellular non−membrane−bounded organelle	0.00819
nucleoplasm part	0.00861
replication fork	0.00881
male pronucleus	0.0107
fascia adherens	0.0107
basolateral plasma membrane	0.015
intracellular membrane−bounded organelle	0.0169
synaptosome	0.0173
female pronucleus	0.0197
collagen type IV	0.0197
cytoplasmic microtubule	0.0197
mediator complex	0.0207
microtubule cytoskeleton	0.0221
protein phosphatase type 2A complex	0.0242
nuclear part	0.0248
ruffle	0.0258
nucleosome	0.0265
intracellular	0.0266
anaphase−promoting complex	0.0317
lateral plasma membrane	0.0317
protein kinase CK2 complex	0.0326
glycerol−3−phosphate dehydrogenase complex	0.0326
unconventional myosin complex	0.0326
laminin−10 complex	0.0326
cullin−RING ubiquitin ligase complex	0.0332
cell−substrate adherens junction	0.0441
PcG protein complex	0.0465

↓Age

Overrepresented Molecular Functions

GO Term	P−Value
nucleotide binding	7.39e−13
RNA binding	2.49e−11
ATP binding	4.59e−10
unfolded protein binding	8.86e−09
purine ribonucleotide binding	1.03e−08
adenyl nucleotide binding	1.53e−08
DNA binding	1.71e−06
protein serine/threonine kinase activity	1.96e−06
phosphotransferase activity, alcohol group as acceptor	4.18e−06
chromatin binding	1.2e−05
kinase activity	1.65e−05
acid−amino acid ligase activity	3.83e−05
ubiquitin−protein ligase activity	4.57e−05
zinc ion binding	7.4e−05
heat shock protein binding	0.000197
monovalent inorganic cation transmembrane transporter activity	0.000965
translation initiation factor activity	0.000979
inositol−1(or 4)−monophosphatase activity	0.00131
histone lysine N−methyltransferase activity (H4−K20 specific)	0.00131
ligase activity	0.00153
histone methyltransferase activity	0.00219
MAP kinase activity	0.00234
microtubule binding	0.00259
cytoskeletal protein binding	0.00308
receptor signaling protein activity	0.00377
nucleic acid binding	0.00449
translation regulator activity	0.00453
stem cell factor receptor binding	0.0048
acetylcholine receptor regulator activity	0.0048
hydrogen ion transporting ATP synthase activity, rotational mechanism	0.00595

↓Age

Overrepresented Molecular Functions

GO Term	P−Value
hydrogen ion transporting ATPase activity, rotational mechanism	0.00595
transcription activator activity	0.00629
ubiquinol−cytochrome−c reductase activity	0.00695
protein phosphatase type 2A regulator activity	0.00695
oxidoreductase activity, acting on diphenols and related substances as donors	0.00695
protein−lysine N−methyltransferase activity	0.00717
protein binding	0.0113
protein homodimerization activity	0.0114
selenide, water dikinase activity	0.012
tRNA (guanine−N2−)−methyltransferase activity	0.012
dimethylargininase activity	0.012
nitric−oxide synthase regulator activity	0.012
ribosomal large subunit binding	0.012
beta−catenin binding	0.0134
hormone receptor binding	0.0134
transcription corepressor activity	0.0142
transcription factor binding	0.0143
ATPase activity, coupled	0.0154
lipid phosphatase activity	0.0174
single−stranded RNA binding	0.0183
specific transcriptional repressor activity	0.0183
protein self−association	0.0203
DNA−directed RNA polymerase activity	0.0231
S−adenosylmethionine−dependent methyltransferase activity	0.0242
N−methyltransferase activity	0.0272
peptidyl−prolyl cis−trans isomerase activity	0.0274
transferase activity, transferring phosphorus−containing groups	0.0284
primary active transmembrane transporter activity	0.0299
ribonucleoprotein binding	0.0312
nucleoside−triphosphatase activity	0.0314

↓Age

Overrepresented Molecular Functions

GO Term	P−Value
protein disulfide isomerase activity	0.0326
nucleoside kinase activity	0.0326
DNA topoisomerase (ATP−hydrolyzing) activity	0.0333
acetyl−CoA C−acyltransferase activity	0.0333
JUN kinase activity	0.0333
eukaryotic initiation factor 4E binding	0.0333
steroid hormone receptor binding	0.0333
transaminase activity	0.0334
ATPase activity, coupled to transmembrane movement of ions	0.0348
protein kinase inhibitor activity	0.0396
extracellular matrix structural constituent	0.0406
calmodulin binding	0.0452
hydrolase activity, acting on acid anhydrides, in phosphorus−containing anhydrides	0.0463
adenylate kinase activity	0.048
signal sequence binding	0.048
tRNA methyltransferase activity	0.048
P−P−bond−hydrolysis−driven protein transmembrane transporter activity	0.048
double−stranded RNA binding	0.0486

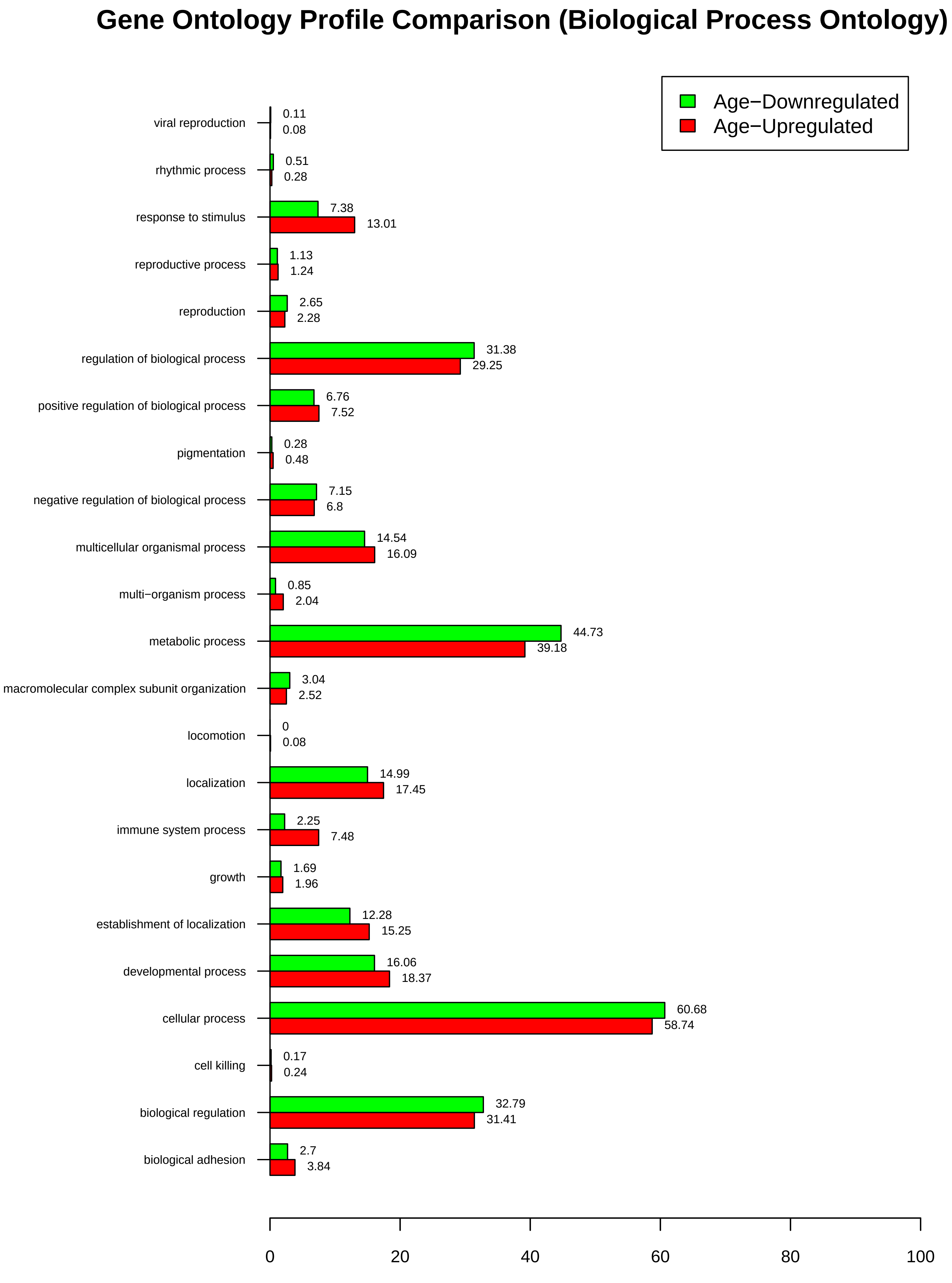

Gene Ontology Profile Comparison (Biological Process Ontology)
Age−Downregulated
Age−Upregulated
viral reproduction
0.11
0.08
rhythmic process
0.51
0.28
response to stimulus
7.38
13.01
reproductive process
1.13
1.24
reproduction
2.65
2.28
regulation of biological process
31.38
29.25
positive regulation of biological process
6.76
7.52
pigmentation
0.28
0.48
negative regulation of biological process
7.15
6.8
multicellular organismal process
14.54
16.09
multi−organism process
0.85
2.04
metabolic process
44.73
39.18
macromolecular complex subunit organization
3.04
2.52
locomotion
0
0.08
localization
14.99
17.45
immune system process
2.25
7.48
growth
1.69
1.96
establishment of localization
12.28
15.25
developmental process
16.06
18.37
cellular process
60.68
58.74
cell killing
0.17
0.24
biological regulation
32.79
31.41
biological adhesion
2.7
3.84
0
20
40
60
80
100

Gene Ontology Profile Comparison (Cell Component Ontology)

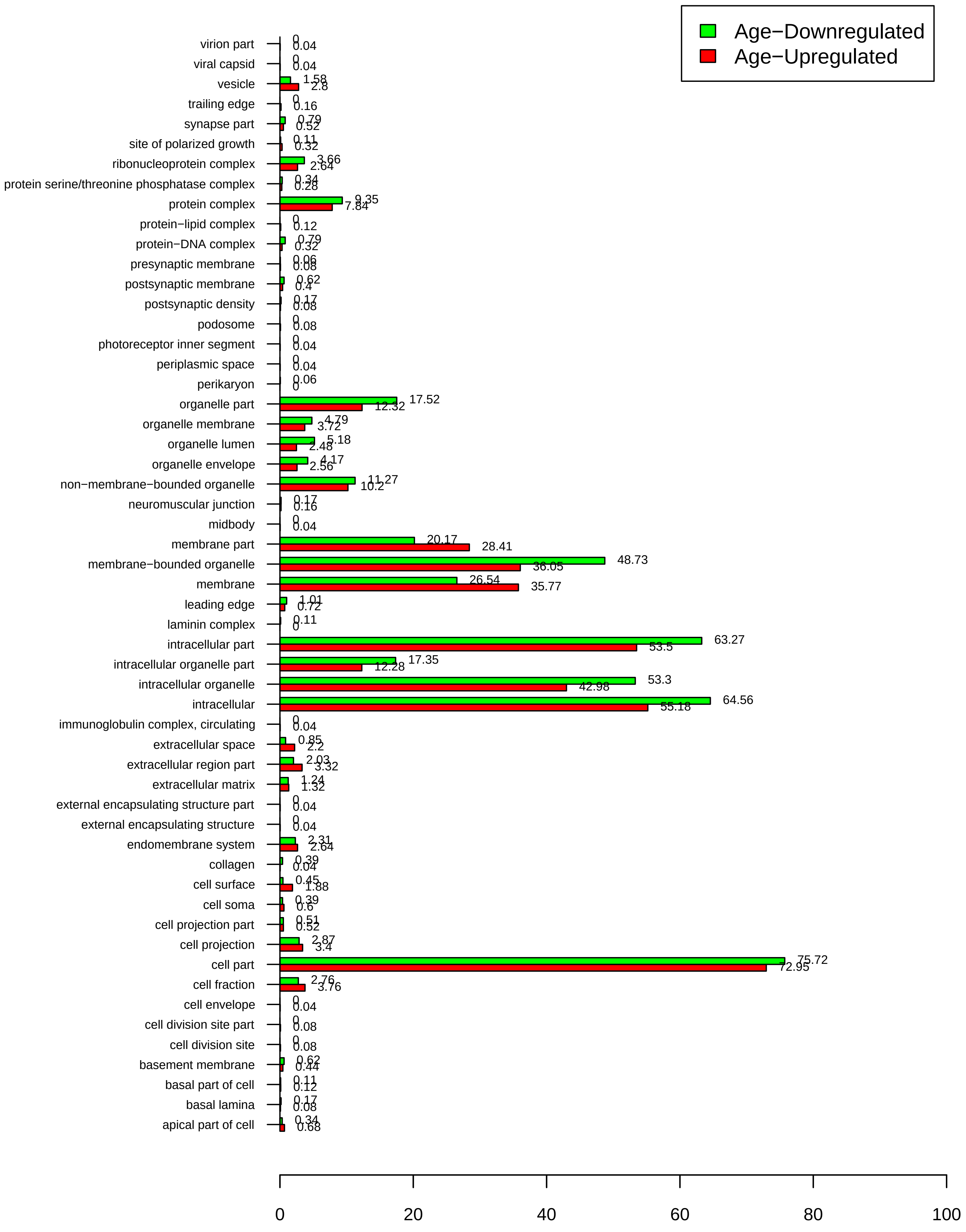

Gene Ontology Profile Comparison (Molecular Function Ontology)

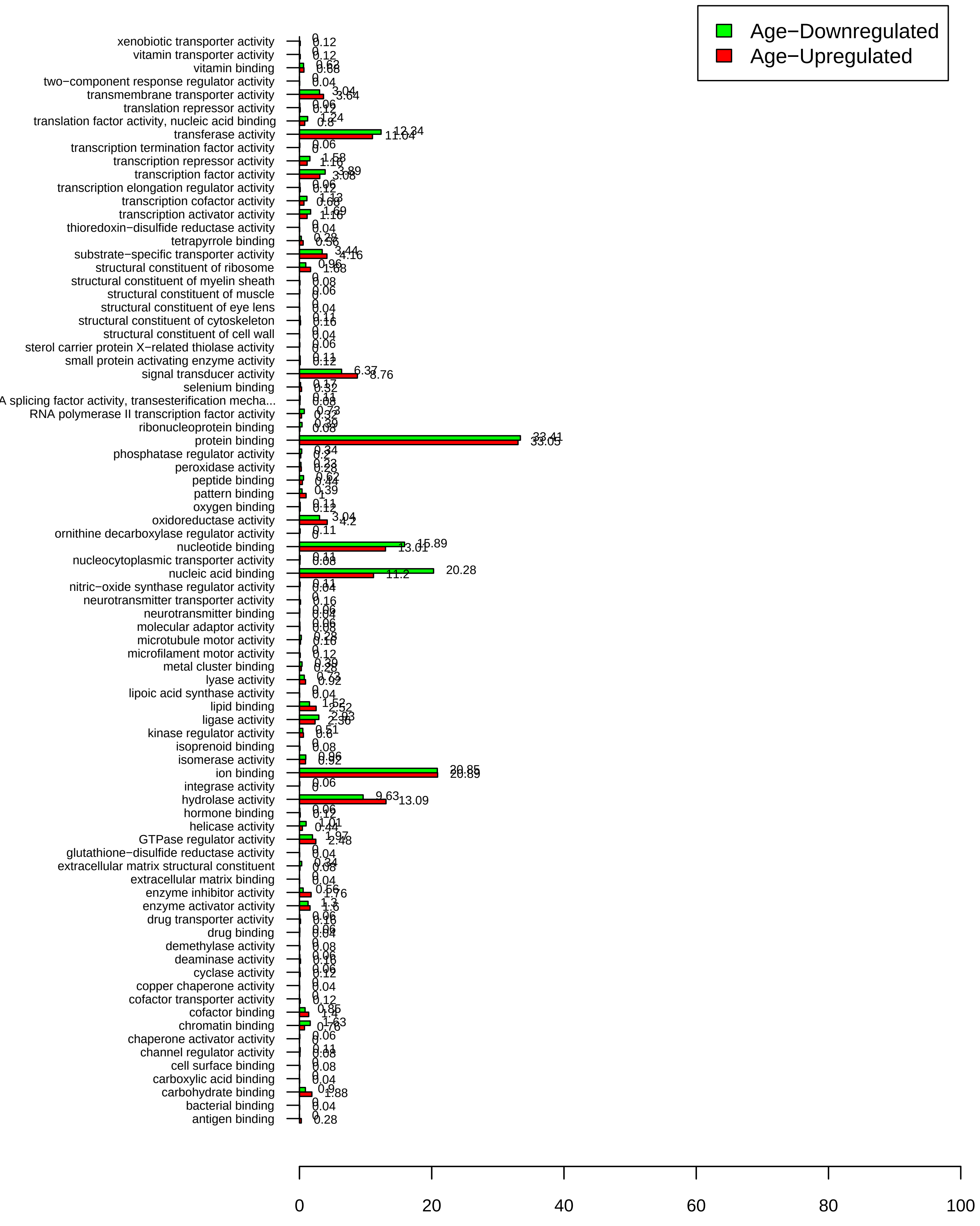

↑Age

Overrepresented KEGG Pathways

GO Term	P−Value
Ribosome	4.56e−09
B cell receptor signaling pathway	0.000237
Leukocyte transendothelial migration	0.000329
N−Glycan degradation	0.0021
Glycan structures − degradation	0.00473
ABC transporters − General	0.00843
Pancreatic cancer	0.00871
Antigen processing and presentation	0.00871
Pentose and glucuronate interconversions	0.0105
Butanoate metabolism	0.0114
Adipocytokine signaling pathway	0.0148
Glycerolipid metabolism	0.0164
Insulin signaling pathway	0.0168
Natural killer cell mediated cytotoxicity	0.0178
Apoptosis	0.0197
Chronic myeloid leukemia	0.0279
Galactose metabolism	0.0287
Glutathione metabolism	0.0307
Pentose phosphate pathway	0.0324
Complement and coagulation cascades	0.0341
Biosynthesis of unsaturated fatty acids	0.0343
Porphyrin and chlorophyll metabolism	0.0439
Acute myeloid leukemia	0.0499

↓Age

Overrepresented KEGG Pathways

GO Term	P−Value
Oxidative phosphorylation	8.62e−08
Cell cycle	1.34e−05
Ubiquitin mediated proteolysis	2.86e−05
Wnt signaling pathway	3.78e−05
Adherens junction	0.000316
Colorectal cancer	0.000779
Endometrial cancer	0.00135
Prostate cancer	0.00145
p53 signaling pathway	0.00149
ErbB signaling pathway	0.00268
Glioma	0.00346
Long−term potentiation	0.00554
Axon guidance	0.00624
Focal adhesion	0.0101
Acute myeloid leukemia	0.0107
Huntington's disease	0.0123
Small cell lung cancer	0.0139
Chronic myeloid leukemia	0.0182
DNA replication	0.0216
Melanogenesis	0.0237
Phosphatidylinositol signaling system	0.0248
Insulin signaling pathway	0.0266
Tight junction	0.0272
Pancreatic cancer	0.0278
GnRH signaling pathway	0.0315
MAPK signaling pathway	0.0403
Renal cell carcinoma	0.0418
Prion disease	0.0498

↑Age

Overrepresented KEGG Pathways

(Based on InterPro Domain Signatures)

GO Term	P−Value
Leukocyte transendothelial migration	0.0036
PPAR signaling pathway	0.006
Glutathione metabolism	0.0095
Porphyrin and chlorophyll metabolism	0.0174
N−Glycan degradation	0.0199
Starch and sucrose metabolism	0.0222
Glycosphingolipid biosynthesis − ganglioseries	0.0223
Peptidoglycan biosynthesis	0.0245
Pentose and glucuronate interconversions	0.0299
Oxidative phosphorylation	0.0333
Pentose phosphate pathway	0.0386
Dentatorubropallidoluysian atrophy (DRPLA)	0.0432
Adherens junction	0.0462

↓Age

Overrepresented KEGG Pathways

(Based on InterPro Domain Signatures)

GO Term	P−Value
Wnt signaling pathway	2e−04
Inositol phosphate metabolism	3e−04
Adherens junction	6e−04
Colorectal cancer	0.0016
Tight junction	0.0021
Endometrial cancer	0.0047
Glioma	0.005
Prostate cancer	0.0072
Cell cycle	0.0076
Basal transcription factors	0.0128
Acute myeloid leukemia	0.0129
Ubiquitin mediated proteolysis	0.0173
Proteasome	0.0192
Phosphatidylinositol signaling system	0.0236
Melanoma	0.0257
Focal adhesion	0.0305
mTOR signaling pathway	0.0367
Thyroid cancer	0.0462

↑Age

Abundance of miRNA Targets

miRNA	Freq(Obs)	Freq(Exp)	Obs/Exp	P−value	P−Value(Adj)
miR−449a	0.162	0.138	1.18	0.000665	0.326
miR−124	0.135	0.115	1.17	0.00233	0.572
miR−449b	0.141	0.123	1.14	0.0086	0.948
miR−699	0.114	0.0982	1.16	0.00864	0.948
miR−34a	0.156	0.138	1.13	0.00966	0.948
miR−764−3p	0.117	0.105	1.12	0.0283	1
miR−671−5p	0.143	0.129	1.11	0.0293	1
miR−681	0.0463	0.0389	1.19	0.0373	1
miR−760	0.145	0.132	1.1	0.0376	1
miR−546	0.0814	0.0715	1.14	0.0386	1

↓Age

Abundance of miRNA Targets

miRNA	Freq(Obs)	Freq(Exp)	Obs/Exp	P−value	P−Value(Adj)
miR−875−3p	0.264	0.19	1.39	9.18e−14	4.51e−11
miR−590−3p	0.34	0.261	1.31	4.34e−13	1.07e−10
miR−17	0.232	0.168	1.38	1.49e−11	2.44e−09
miR−142−5p	0.241	0.182	1.32	1.08e−09	1.04e−07
miR−106b	0.194	0.14	1.38	1.14e−09	1.04e−07
miR−369−3p	0.159	0.11	1.44	1.27e−09	1.04e−07
miR−374	0.177	0.127	1.4	2.27e−09	1.59e−07
miR−186	0.265	0.206	1.29	4.87e−09	2.99e−07
miR−743b−3p	0.27	0.211	1.28	6.08e−09	3.32e−07
miR−106a	0.213	0.16	1.33	6.88e−09	3.38e−07
miR−20b	0.212	0.16	1.32	1.73e−08	7.74e−07
miR−20a	0.226	0.174	1.3	3.52e−08	1.44e−06
miR−302a	0.212	0.162	1.31	6.12e−08	2.21e−06
miR−694	0.243	0.19	1.28	6.67e−08	2.21e−06
miR−465a−5p	0.253	0.2	1.27	6.76e−08	2.21e−06
miR−301a	0.211	0.162	1.3	8.08e−08	2.48e−06
miR−743a	0.253	0.2	1.26	8.68e−08	2.51e−06
miR−302b	0.217	0.168	1.29	1.1e−07	3e−06
miR−465b−5p	0.227	0.177	1.28	1.27e−07	3.28e−06
miR−881	0.192	0.146	1.32	1.53e−07	3.75e−06
miR−381	0.189	0.144	1.32	1.98e−07	4.63e−06
miR−693−3p	0.261	0.209	1.25	2.34e−07	5.21e−06
miR−93	0.208	0.161	1.29	2.61e−07	5.58e−06
miR−126−5p	0.178	0.135	1.32	4.03e−07	8.25e−06
miR−669b	0.241	0.192	1.26	4.35e−07	8.55e−06
miR−144	0.19	0.146	1.3	6.12e−07	1.16e−05
miR−1	0.184	0.141	1.3	6.79e−07	1.24e−05
miR−203	0.214	0.169	1.27	8.94e−07	1.57e−05
miR−301b	0.201	0.156	1.28	9.44e−07	1.6e−05
miR−384−5p	0.224	0.178	1.26	1.03e−06	1.68e−05

↓Age

Abundance of miRNA Targets

miRNA	Freq(Obs)	Freq(Exp)	Obs/Exp	P−value	P−Value(Adj)
miR−146a	0.158	0.118	1.33	1.13e−06	1.68e−05
miR−146b	0.159	0.12	1.33	1.16e−06	1.68e−05
miR−883a−3p	0.168	0.128	1.32	1.16e−06	1.68e−05
miR−302d	0.207	0.163	1.27	1.19e−06	1.68e−05
miR−223	0.156	0.117	1.33	1.2e−06	1.68e−05
miR−323−3p	0.12	0.0867	1.39	1.63e−06	2.22e−05
miR−297b−3p	0.221	0.177	1.25	1.94e−06	2.51e−05
miR−495	0.206	0.163	1.27	1.94e−06	2.51e−05
miR−350	0.199	0.157	1.27	2.05e−06	2.58e−05
miR−802	0.146	0.109	1.33	2.55e−06	3.12e−05
miR−19b	0.198	0.156	1.27	2.6e−06	3.12e−05
miR−883b−3p	0.165	0.127	1.3	3.48e−06	4.07e−05
miR−142−3p	0.137	0.102	1.34	4.04e−06	4.61e−05
miR−19a	0.212	0.169	1.25	4.27e−06	4.75e−05
miR−340−5p	0.243	0.198	1.23	4.35e−06	4.75e−05
miR−105	0.157	0.12	1.31	4.48e−06	4.78e−05
miR−29c	0.146	0.111	1.32	5.77e−06	5.9e−05
miR−335−3p	0.264	0.218	1.21	5.78e−06	5.9e−05
miR−199a−5p	0.169	0.131	1.29	5.89e−06	5.9e−05
miR−34b−3p	0.0951	0.0668	1.42	6.07e−06	5.94e−05
miR−467g	0.217	0.175	1.24	6.17e−06	5.94e−05
miR−217	0.158	0.121	1.3	6.85e−06	6.47e−05
miR−466f−3p	0.233	0.19	1.23	7.54e−06	6.98e−05
miR−669f	0.264	0.219	1.21	7.78e−06	7.08e−05
miR−29a	0.143	0.108	1.32	8.15e−06	7.25e−05
miR−294	0.176	0.138	1.27	8.27e−06	7.25e−05
miR−29b	0.161	0.125	1.29	9.5e−06	8.18e−05
miR−295	0.194	0.155	1.25	1.07e−05	9.08e−05
miR−132	0.152	0.118	1.3	1.09e−05	9.09e−05
miR−181d	0.216	0.175	1.23	1.15e−05	9.43e−05

↓Age

Abundance of miRNA Targets

miRNA	Freq(Obs)	Freq(Exp)	Obs/Exp	P−value	P−Value(Adj)
miR−200c	0.186	0.148	1.26	1.2e−05	9.63e−05
miR−568	0.201	0.161	1.24	1.35e−05	0.000107
miR−291b−3p	0.196	0.157	1.25	1.38e−05	0.000108
miR−26a	0.158	0.123	1.28	1.41e−05	0.000109
miR−30e	0.194	0.156	1.25	1.49e−05	0.000113
miR−202−5p	0.0918	0.0653	1.41	1.57e−05	0.000115
miR−684	0.163	0.128	1.28	1.57e−05	0.000115
miR−466a−5p	0.191	0.154	1.25	1.68e−05	0.000118
miR−466b−5p	0.191	0.154	1.25	1.68e−05	0.000118
miR−466e−5p	0.191	0.154	1.25	1.68e−05	0.000118
miR−465c−5p	0.191	0.153	1.24	2.04e−05	0.000141
miR−135b	0.218	0.179	1.22	2.17e−05	0.000148
miR−212	0.143	0.111	1.3	2.2e−05	0.000148
miR−421	0.179	0.143	1.25	2.25e−05	0.000149
miR−26b	0.163	0.129	1.27	2.36e−05	0.000154
miR−155	0.156	0.123	1.28	2.6e−05	0.000168
miR−499	0.143	0.11	1.29	2.63e−05	0.000168
miR−139−5p	0.155	0.121	1.28	2.77e−05	0.000175
miR−486	0.0866	0.0617	1.4	2.89e−05	0.000179
miR−361	0.137	0.106	1.3	2.91e−05	0.000179
miR−466g	0.169	0.134	1.26	3.07e−05	0.000186
miR−200b	0.184	0.148	1.24	3.1e−05	0.000186
miR−194	0.135	0.104	1.3	3.28e−05	0.000194
miR−300	0.141	0.109	1.29	3.33e−05	0.000195
miR−464	0.161	0.128	1.26	3.37e−05	0.000195
miR−292−5p	0.125	0.0953	1.31	3.56e−05	0.000203
miR−466d−3p	0.2	0.163	1.23	3.85e−05	0.000217
miR−677	0.163	0.13	1.26	4.04e−05	0.000223
miR−466a−3p	0.223	0.184	1.21	4.23e−05	0.000223
miR−466b−3−3p	0.223	0.184	1.21	4.23e−05	0.000223

↓Age

Abundance of miRNA Targets

miRNA	Freq(Obs)	Freq(Exp)	Obs/Exp	P−value	P−Value(Adj)
miR−466b−3p	0.223	0.184	1.21	4.23e−05	0.000223
miR−466c−3p	0.223	0.184	1.21	4.23e−05	0.000223
miR−466e−3p	0.223	0.184	1.21	4.23e−05	0.000223
miR−183	0.137	0.106	1.29	4.4e−05	0.00023
miR−16	0.223	0.185	1.21	4.91e−05	0.000254
miR−320	0.218	0.181	1.21	5.57e−05	0.000283
miR−382	0.158	0.126	1.26	5.59e−05	0.000283
miR−101b	0.148	0.117	1.27	5.68e−05	0.000284
miR−488	0.191	0.156	1.23	5.8e−05	0.000288
miR−30c	0.189	0.154	1.23	6.02e−05	0.000295
miR−1192	0.208	0.171	1.21	6.08e−05	0.000295
miR−1−2−as	0.136	0.106	1.28	6.33e−05	0.000305
miR−467e	0.157	0.125	1.26	6.62e−05	0.000316
miR−466c−5p	0.187	0.152	1.23	6.7e−05	0.000316
miR−467h	0.173	0.139	1.24	6.94e−05	0.000325
miR−759	0.16	0.128	1.25	7.51e−05	0.000348
miR−582−3p	0.114	0.0868	1.31	7.88e−05	0.000361
miR−141	0.199	0.164	1.21	7.99e−05	0.000361
miR−18a	0.155	0.123	1.25	8.01e−05	0.000361
miR−338−5p	0.158	0.126	1.25	8.68e−05	0.000387
miR−30b	0.183	0.149	1.23	8.98e−05	0.000397
miR−878−3p	0.12	0.093	1.29	0.000102	0.000447
miR−384−3p	0.141	0.112	1.26	0.000105	0.000458
miR−181b	0.209	0.174	1.2	0.000108	0.000466
miR−290−5p	0.121	0.0938	1.29	0.000112	0.000477
miR−669c	0.135	0.107	1.27	0.000113	0.000477
miR−136	0.174	0.142	1.23	0.000118	0.000494
miR−302c	0.174	0.142	1.23	0.000121	0.000502
miR−363	0.117	0.0905	1.3	0.000124	0.00051
miR−876−3p	0.17	0.138	1.23	0.000125	0.00051

↓Age

Abundance of miRNA Targets

miRNA	Freq(Obs)	Freq(Exp)	Obs/Exp	P−value	P−Value(Adj)
miR−429	0.184	0.151	1.22	0.000138	0.000556
miR−206	0.164	0.133	1.23	0.000138	0.000556
miR−669g	0.153	0.123	1.25	0.000141	0.000562
miR−7b	0.193	0.159	1.21	0.000144	0.000569
miR−291a−3p	0.17	0.138	1.23	0.000151	0.000593
miR−140	0.135	0.107	1.26	0.000159	0.000618
miR−367	0.125	0.0981	1.27	0.000179	0.000691
miR−7a	0.194	0.161	1.2	0.000181	0.000694
miR−33	0.125	0.0981	1.27	0.000185	0.000706
miR−669h−3p	0.181	0.149	1.21	0.000203	0.000765
miR−15a	0.225	0.19	1.18	0.000226	0.000844
miR−23b	0.173	0.142	1.22	0.000227	0.000844
miR−335−5p	0.172	0.141	1.22	0.000238	0.000879
miR−703	0.153	0.124	1.23	0.000242	0.000887
miR−494	0.182	0.15	1.21	0.000258	0.00094
miR−30a	0.177	0.146	1.21	0.000261	0.000941
miR−669k	0.146	0.118	1.24	0.000265	0.000949
miR−590−5p	0.12	0.0942	1.27	0.000267	0.000949
miR−331−5p	0.0879	0.0661	1.33	0.00028	0.000989
miR−539	0.209	0.176	1.19	0.000291	0.00102
miR−216a	0.136	0.109	1.25	0.000294	0.00102
miR−872	0.117	0.0921	1.27	3e−04	0.00104
miR−23a	0.172	0.142	1.21	0.000308	0.00106
miR−145	0.17	0.14	1.21	0.000336	0.00114
miR−130a	0.17	0.14	1.21	0.000351	0.00119
miR−706	0.16	0.131	1.22	0.000356	0.0012
miR−137	0.143	0.116	1.24	0.000365	0.00122
miR−1194	0.135	0.108	1.24	0.000382	0.00127
miR−1187	0.197	0.166	1.19	0.000406	0.00133
miR−653	0.129	0.103	1.25	0.000407	0.00133

↓Age

Abundance of miRNA Targets

miRNA	Freq(Obs)	Freq(Exp)	Obs/Exp	P−value	P−Value(Adj)
miR−1196	0.084	0.0633	1.33	0.000421	0.00137
miR−201	0.139	0.113	1.24	0.000425	0.00137
miR−871	0.204	0.172	1.18	0.000431	0.00137
miR−181c	0.178	0.149	1.2	0.000432	0.00137
miR−219	0.0671	0.0487	1.38	0.000434	0.00137
miR−742	0.198	0.167	1.19	0.000435	0.00137
miR−466l	0.327	0.29	1.13	0.000438	0.00137
miR−195	0.217	0.185	1.17	0.000446	0.00138
miR−181a	0.191	0.161	1.19	0.000468	0.00144
miR−202−3p	0.102	0.0791	1.28	0.000507	0.00155
miR−679	0.122	0.0972	1.25	0.000525	0.0016
miR−200a	0.18	0.151	1.19	0.000532	0.00161
miR−101a	0.139	0.113	1.23	0.000535	0.00161
miR−292−3p	0.122	0.0974	1.25	0.000581	0.00174
miR−322	0.229	0.196	1.16	0.000595	0.00176
miR−669d	0.16	0.132	1.21	0.000597	0.00176
miR−92b	0.097	0.0753	1.29	0.000599	0.00176
miR−717	0.169	0.141	1.2	0.00067	0.00196
miR−337−5p	0.0273	0.0167	1.64	0.000679	0.00197
miR−1191	0.111	0.0879	1.26	0.000725	0.00209
miR−153	0.122	0.098	1.24	0.000759	0.00218
miR−27b	0.187	0.158	1.18	0.000778	0.00222
miR−410	0.109	0.0863	1.26	0.000784	0.00223
miR−489	0.113	0.0907	1.25	0.000923	0.0026
miR−32	0.128	0.104	1.23	0.000926	0.0026
miR−690	0.127	0.103	1.23	0.000984	0.00274
miR−466j	0.173	0.145	1.19	0.00102	0.00282
miR−505	0.124	0.1	1.23	0.00105	0.00289
miR−450b−5p	0.196	0.167	1.17	0.00108	0.00297
miR−27a	0.182	0.155	1.18	0.00112	0.00306

↓Age

Abundance of miRNA Targets

miRNA	Freq(Obs)	Freq(Exp)	Obs/Exp	P−value	P−Value(Adj)
miR−222	0.0977	0.0771	1.27	0.00114	0.0031
miR−582−5p	0.145	0.12	1.21	0.00119	0.00321
miR−30d	0.16	0.134	1.19	0.0012	0.00322
miR−542−3p	0.163	0.137	1.19	0.00122	0.00325
miR−1190	0.139	0.115	1.21	0.00126	0.00333
miR−669j	0.126	0.103	1.22	0.00126	0.00333
miR−675−3p	0.0645	0.0481	1.34	0.00129	0.00339
miR−21	0.106	0.0851	1.25	0.00138	0.00359
miR−450b−3p	0.184	0.157	1.17	0.00142	0.00369
miR−25	0.117	0.0952	1.23	0.00146	0.00377
miR−433	0.0905	0.0712	1.27	0.00149	0.00384
miR−15b	0.218	0.189	1.15	0.00156	0.00398
let−7c	0.149	0.125	1.19	0.00167	0.00425
miR−669h−5p	0.163	0.138	1.18	0.00175	0.00444
miR−135a	0.215	0.187	1.15	0.00184	0.00463
miR−205	0.118	0.097	1.22	0.00187	0.0047
miR−182	0.184	0.158	1.17	0.00189	0.00471
miR−743b−5p	0.13	0.108	1.21	0.00196	0.00486
miR−376b	0.119	0.0977	1.22	0.00204	0.00501
miR−468	0.119	0.0977	1.22	0.00204	0.00501
miR−543	0.139	0.116	1.2	0.00212	0.00517
miR−409−3p	0.118	0.0967	1.22	0.0022	0.00534
miR−18b	0.14	0.117	1.19	0.00228	0.00551
miR−691	0.181	0.155	1.17	0.00229	0.00551
miR−196a	0.134	0.112	1.2	0.00237	0.00568
miR−509−5p	0.137	0.114	1.2	0.00239	0.00569
miR−669i	0.123	0.102	1.21	0.00274	0.00649
miR−878−5p	0.101	0.0818	1.23	0.00279	0.00659
miR−666−3p	0.155	0.132	1.18	0.00281	0.0066
miR−290−3p	0.115	0.0943	1.21	0.00285	0.00667

↓Age

Abundance of miRNA Targets

miRNA	Freq(Obs)	Freq(Exp)	Obs/Exp	P−value	P−Value(Adj)
miR−873	0.145	0.123	1.18	0.00328	0.00763
miR−362−3p	0.117	0.0971	1.21	0.00335	0.00775
miR−224	0.144	0.122	1.18	0.00339	0.00778
miR−129−5p	0.147	0.125	1.18	0.00339	0.00778
miR−448	0.15	0.127	1.18	0.0036	0.00823
miR−501−5p	0.143	0.121	1.18	0.00364	0.00827
miR−103	0.169	0.145	1.16	0.0038	0.00851
miR−107	0.169	0.145	1.16	0.0038	0.00851
miR−221	0.129	0.108	1.19	0.0038	0.00851
miR−1198	0.142	0.12	1.18	0.00382	0.00853
miR−377	0.194	0.169	1.15	0.00397	0.00882
miR−685	0.112	0.0928	1.21	0.00411	0.0091
miR−130b	0.16	0.137	1.16	0.00438	0.00965
miR−148a	0.148	0.126	1.17	0.00466	0.0102
miR−411	0.0664	0.0519	1.28	0.00468	0.0102
miR−693−5p	0.139	0.118	1.18	0.00481	0.0104
miR−154	0.0924	0.0753	1.23	0.00481	0.0104
miR−467a	0.106	0.0879	1.21	0.00502	0.0108
miR−434−5p	0.0397	0.0289	1.38	0.00506	0.0108
miR−883b−5p	0.184	0.161	1.15	0.00514	0.0109
miR−380−5p	0.0859	0.0696	1.23	0.00514	0.0109
miR−345−3p	0.122	0.102	1.19	0.0052	0.011
miR−710	0.16	0.138	1.16	0.00542	0.0114
miR−544	0.178	0.155	1.15	0.00566	0.0119
miR−654−3p	0.118	0.0996	1.19	0.00574	0.012
miR−451	0.0456	0.0341	1.34	0.00594	0.0124
miR−196b	0.122	0.103	1.18	0.00605	0.0125
let−7g	0.143	0.123	1.17	0.0061	0.0126
miR−466h	0.148	0.128	1.16	0.00702	0.0144
miR−463	0.0801	0.065	1.23	0.00727	0.0149

↓Age

Abundance of miRNA Targets

miRNA	Freq(Obs)	Freq(Exp)	Obs/Exp	P−value	P−Value(Adj)
miR−669a	0.139	0.12	1.16	0.00733	0.0149
miR−455	0.11	0.0924	1.19	0.00737	0.0149
let−7f	0.14	0.12	1.16	0.00751	0.0152
miR−496	0.0938	0.0777	1.21	0.00793	0.016
miR−721	0.135	0.116	1.16	0.00809	0.0162
miR−216b	0.138	0.119	1.16	0.00813	0.0162
miR−467b	0.101	0.0844	1.2	0.0084	0.0167
miR−497	0.211	0.188	1.12	0.00846	0.0168
miR−1197	0.144	0.124	1.16	0.0087	0.0172
miR−471	0.0449	0.0342	1.31	0.00895	0.0176
miR−672	0.139	0.12	1.16	0.00916	0.0179
miR−208b	0.0599	0.0475	1.26	0.00994	0.0194
miR−883a−5p	0.174	0.153	1.14	0.01	0.0195
miR−148b	0.148	0.129	1.15	0.0102	0.0198
miR−452	0.159	0.139	1.14	0.0106	0.0204
let−7i	0.143	0.124	1.15	0.0112	0.0214
miR−682	0.12	0.103	1.17	0.0115	0.022
miR−425	0.0892	0.0744	1.2	0.0116	0.0221
miR−686	0.137	0.119	1.15	0.0118	0.0223
miR−190	0.16	0.14	1.14	0.0126	0.0237
miR−92a	0.0938	0.0788	1.19	0.0128	0.024
miR−877	0.125	0.108	1.16	0.0128	0.0241
miR−376c	0.15	0.132	1.14	0.013	0.0242
miR−324−3p	0.0983	0.0831	1.18	0.0134	0.0249
miR−152	0.139	0.121	1.15	0.0135	0.025
let−7a	0.138	0.12	1.15	0.0141	0.026
miR−466f−5p	0.0859	0.0719	1.2	0.0143	0.0262
miR−151−3p	0.084	0.0701	1.2	0.0144	0.0264
miR−378	0.115	0.0985	1.16	0.0146	0.0267
miR−707	0.0885	0.0744	1.19	0.0148	0.0269

↓Age

Abundance of miRNA Targets

miRNA	Freq(Obs)	Freq(Exp)	Obs/Exp	P−value	P−Value(Adj)
miR−215	0.0651	0.053	1.23	0.0149	0.0269
miR−96	0.133	0.116	1.15	0.015	0.027
miR−509−3p	0.0697	0.0572	1.22	0.015	0.027
miR−329	0.107	0.092	1.17	0.0153	0.0275
miR−879	0.11	0.0944	1.17	0.0157	0.028
miR−9	0.197	0.176	1.11	0.0159	0.0282
miR−574−5p	0.16	0.141	1.13	0.0161	0.0285
miR−297b−5p	0.159	0.141	1.13	0.0171	0.0302
miR−291a−5p	0.151	0.133	1.13	0.0177	0.0311
miR−291b−5p	0.151	0.133	1.13	0.0177	0.0311
miR−466k	0.171	0.153	1.12	0.0182	0.0317
miR−344	0.0592	0.0482	1.23	0.0188	0.0327
miR−615−3p	0.041	0.032	1.28	0.0194	0.0337
miR−383	0.124	0.109	1.15	0.0202	0.0349
let−7d	0.133	0.117	1.14	0.0208	0.0357
miR−1186	0.145	0.128	1.13	0.0208	0.0357
miR−409−5p	0.0553	0.0449	1.23	0.0209	0.0357
miR−342−3p	0.126	0.111	1.14	0.0213	0.0363
miR−467c	0.0553	0.045	1.23	0.0219	0.0372
miR−31	0.126	0.111	1.14	0.0224	0.038
miR−22	0.125	0.11	1.14	0.0227	0.0383
miR−674	0.169	0.152	1.12	0.0231	0.0389
miR−297c	0.17	0.152	1.12	0.0235	0.0393
miR−532−5p	0.111	0.0969	1.15	0.0236	0.0393
miR−882	0.18	0.162	1.11	0.0236	0.0393
miR−127	0.0352	0.0272	1.29	0.0239	0.0396
miR−211	0.164	0.147	1.12	0.0242	0.0399
miR−10b	0.0853	0.0727	1.17	0.0242	0.0399
let−7e	0.156	0.139	1.12	0.0244	0.0401
miR−297a	0.165	0.148	1.12	0.0248	0.0407

↓Age

Abundance of miRNA Targets

miRNA	Freq(Obs)	Freq(Exp)	Obs/Exp	P−value	P−Value(Adj)
miR−467d	0.0462	0.0373	1.24	0.0272	0.0443
miR−327	0.104	0.09	1.15	0.028	0.0455
miR−380−3p	0.107	0.0931	1.15	0.0284	0.046
miR−669e	0.127	0.112	1.13	0.0285	0.046
miR−466i	0.169	0.153	1.11	0.0292	0.047
miR−467f	0.135	0.12	1.12	0.0307	0.0493
miR−450a−3p	0.15	0.134	1.12	0.0312	0.0498
miR−741	0.148	0.133	1.12	0.0316	0.0503
miR−362−5p	0.123	0.109	1.12	0.0372	0.059
miR−338−3p	0.138	0.124	1.11	0.0404	0.0639
miR−592	0.112	0.0992	1.13	0.041	0.0647
miR−574−3p	0.0508	0.0423	1.2	0.0414	0.0651
miR−466d−5p	0.144	0.13	1.11	0.0428	0.0671
miR−340−3p	0.0514	0.0429	1.2	0.043	0.0671
miR−193b	0.0833	0.0725	1.15	0.0431	0.0671
miR−551b	0.0371	0.03	1.24	0.0435	0.0676
miR−191	0.0775	0.067	1.16	0.0439	0.068
let−7b	0.141	0.127	1.11	0.0457	0.0706
miR−199a−3p	0.118	0.105	1.12	0.046	0.0706
miR−199b	0.118	0.105	1.12	0.046	0.0706
miR−10a	0.0866	0.0758	1.14	0.0474	0.0724
miR−98	0.135	0.122	1.11	0.0479	0.073
miR−337−3p	0.115	0.102	1.12	0.0491	0.0746
miR−151−5p	0.0579	0.0492	1.18	0.0492	0.0746

Tests for Chromosome Over−representation

Chromosome	Age−upregulated Genes	Age−downregulated Genes
1	0.665	0.0465*
2	0.972	0.566
3	0.831	0.135
4	0.786	0.155
5	0.484	0.856
6	0.771	0.91
7	0.75	0.975
8	0.489	0.111
9	0.0733	0.364
10	0.286	0.205
11	0.6	0.182
12	0.442	0.847
13	0.953	0.414
14	0.655	0.851
15	0.00436*	0.974
16	0.69	0.133
17	0.249	0.976
18	0.676	0.159
19	0.147	0.543
X	0.677	0.605
Y	0.454	1.00

The table lists p−values generated from a test that evaluates whether there exists an over−abundance of identified genes with respect to a given chromosome. The null hypothesis assumes that the set of genes has been selected at random from those represented on the Affymetrix 430 2.0 array. A significant test indicates that a chromosome contains more of the identified genes than would be expected if the gene set had been chosen at random.

* = significant p−value, without multiple test adjustment

** = significant p−value, with multiple test adjustment

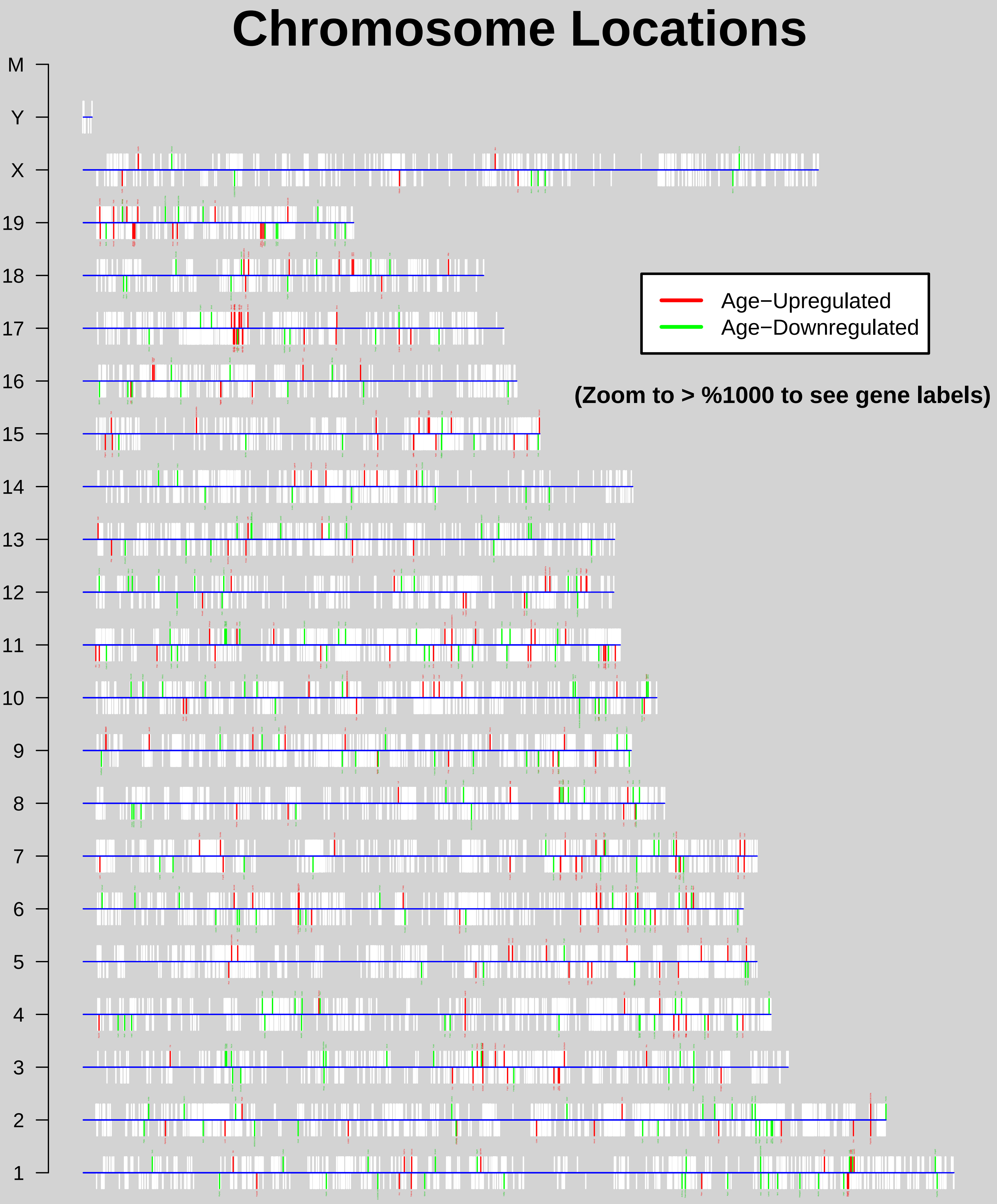
Chromosome Locations
M
Y
X
19
18
17
16
15
14
13
12
11
10
9
8
7
6
5
4
3
2
1
Age−Upregulated
Age−Downregulated
(Zoom to > %1000 to see gene labels)
