# Supplementary material for: Genes and gene expression modules associated with caloric restriction and aging in the laboratory mouse
Source: BMC Genomics. 2009 Dec 7;10:585. doi: 10.1186/1471-2164-10-585 (PMC2795771; doi:10.1186/1471-2164-10-585)

Additional File 11

Genes and Gene Expression Modules Associated with Caloric Restriction and Aging in the Laboratory Mouse

William R. Swindell
University of Michigan, Departments of Pathology and Geriatrics

---

Gene Expression Modules Regulated by Aging

This file provides a description of gene expression modules regulated by age in multiple tissue types. Gene expression modules of varying size are displayed, including those with 2, 3, 5, 10, 20 and 40 member genes. The value of *M* associated with each module is directly related to the overall responsiveness of member genes to age across tissue types (see Methods). The p-value associated with each value of *M* is generated by a simulation analysis, in which modules of the same size are formed at random, without reference to observed co-expression patterns (see Methods).

A dendrogram is shown for each module, which represents the co-expression patterns of member genes. This was generated based upon an average linkage hierarchical cluster analysis, in which similarity between genes was based upon the absolute value of Pearson's correlation coefficient (*r*) (see Methods). Additionally, for each module, a grid is shown that displays the differential expression patterns associated with member genes. Symbols have the following interpretation.

- ● Gene is significantly up regulated by age ($P_u < 0.05$)
- ● Gene is significantly down regulated by age ($P_d < 0.05$)
- ▬ Gene is marginally up regulated by age ($0.05 < P_u < 0.10$)
- ▬ Gene is marginally down regulated by age ($0.05 < P_d < 0.10$)
- ▬ Non-significant age effect ($P_u > 0.10$ and $P_d > 0.10$)
- × No data (gene not represented for a given tissue or array annotation was limiting)
- ✳ Evidence conflicts, but favors up regulation by age
- ✳ Evidence conflicts, but favors down regulation by age

The last two categories (✳ and ✳) indicate significant effects with conflicting evidence. This can arise if there is significant up regulation by age in one experiment, and significant down regulation by age in another experiment that has examined the same tissue. Alternatively, a conflict may arise if $P_u < 0.05$ and also $P_d < 0.05$ for a given tissue type. Symbols shown in charts are based upon a comparison-wise type I error rate of 0.05.

Contact: William R. Swindell, wswindel@umich.edu

Aging−Regulated Modules (2 Genes)

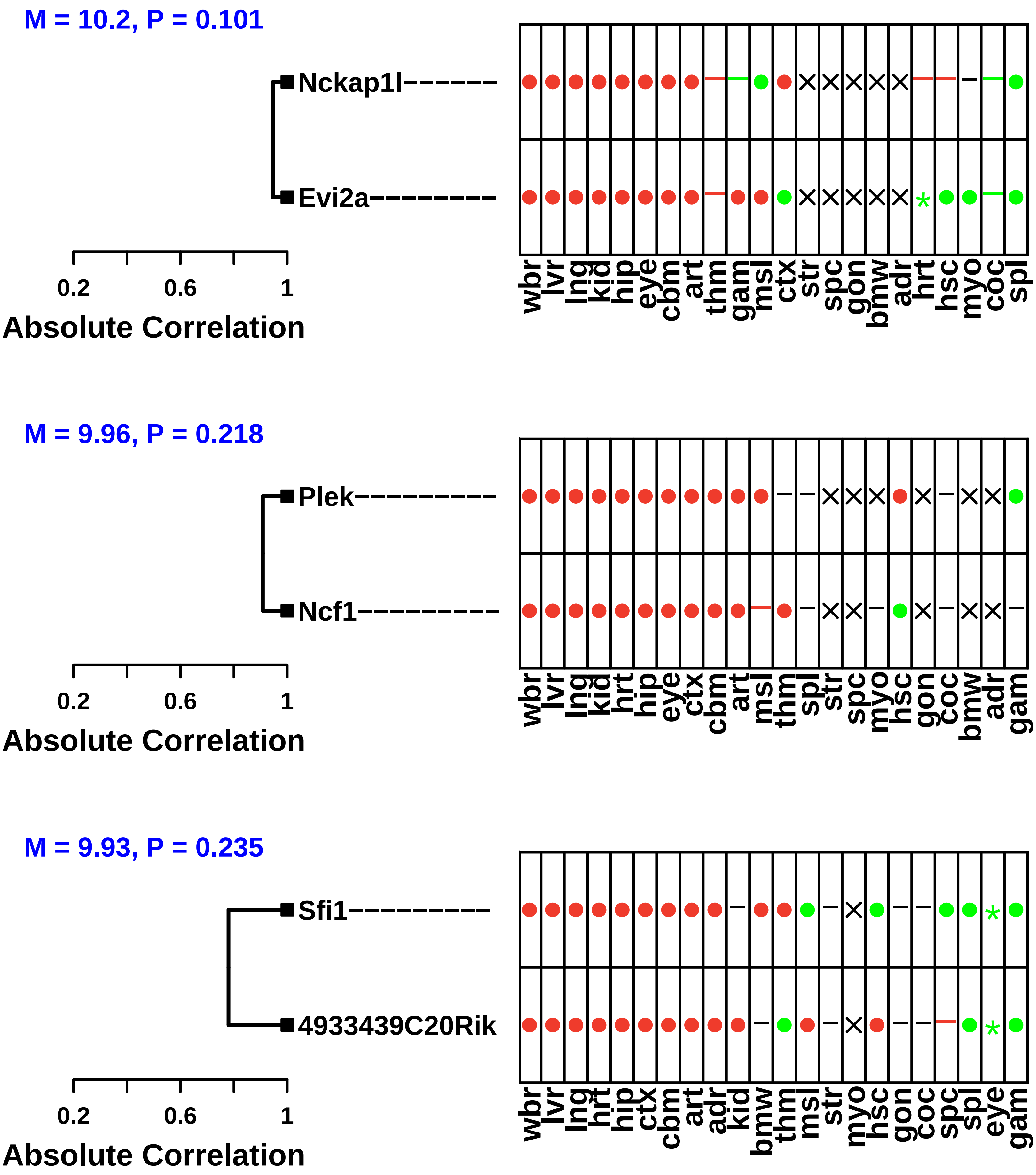

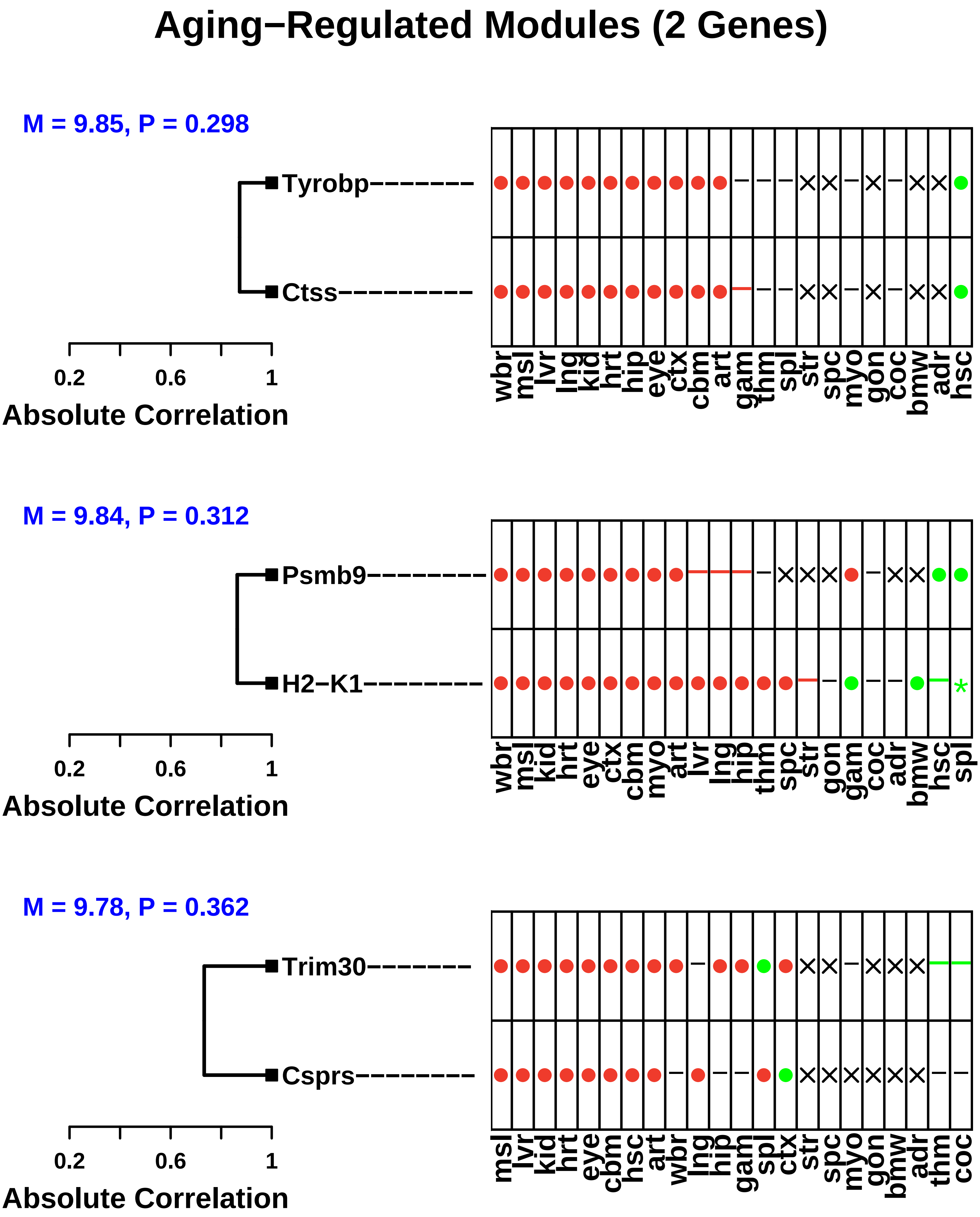
Aging−Regulated Modules (2 Genes)
M = 9.85, P = 0.298
Tyrobp
Ctss
0.2
0.6
1
Absolute Correlation
wbr msl lvr lng kid hrt hip eye ctx cbm art gam thm spl str spc myo gon coc bmw adr hsc
M = 9.84, P = 0.312
Psmb9
H2−K1
0.2
0.6
1
Absolute Correlation
wbr msl kid hrt eye ctx cbm myo art lvr lng hip thm spc str gon gam coc adr bmw hsc spl
M = 9.78, P = 0.362
Trim30
Csprs
0.2
0.6
1
Absolute Correlation
msl lvr kid hrt eye cbm hsc art wbr lng hip gam spl ctx str spc myo gon bmw adr thm coc

Aging−Regulated Modules (2 Genes)

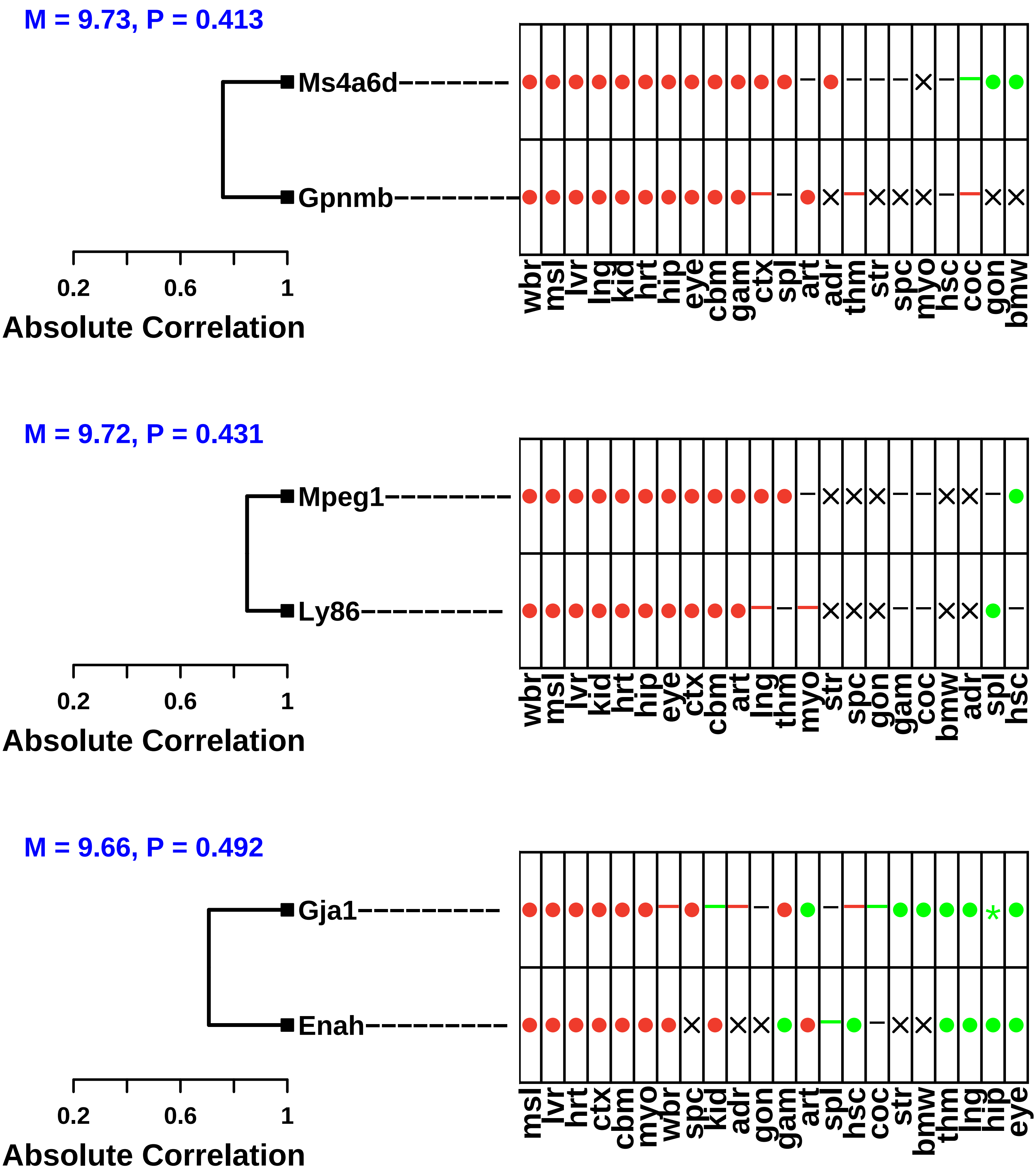

Aging−Regulated Modules (2 Genes)

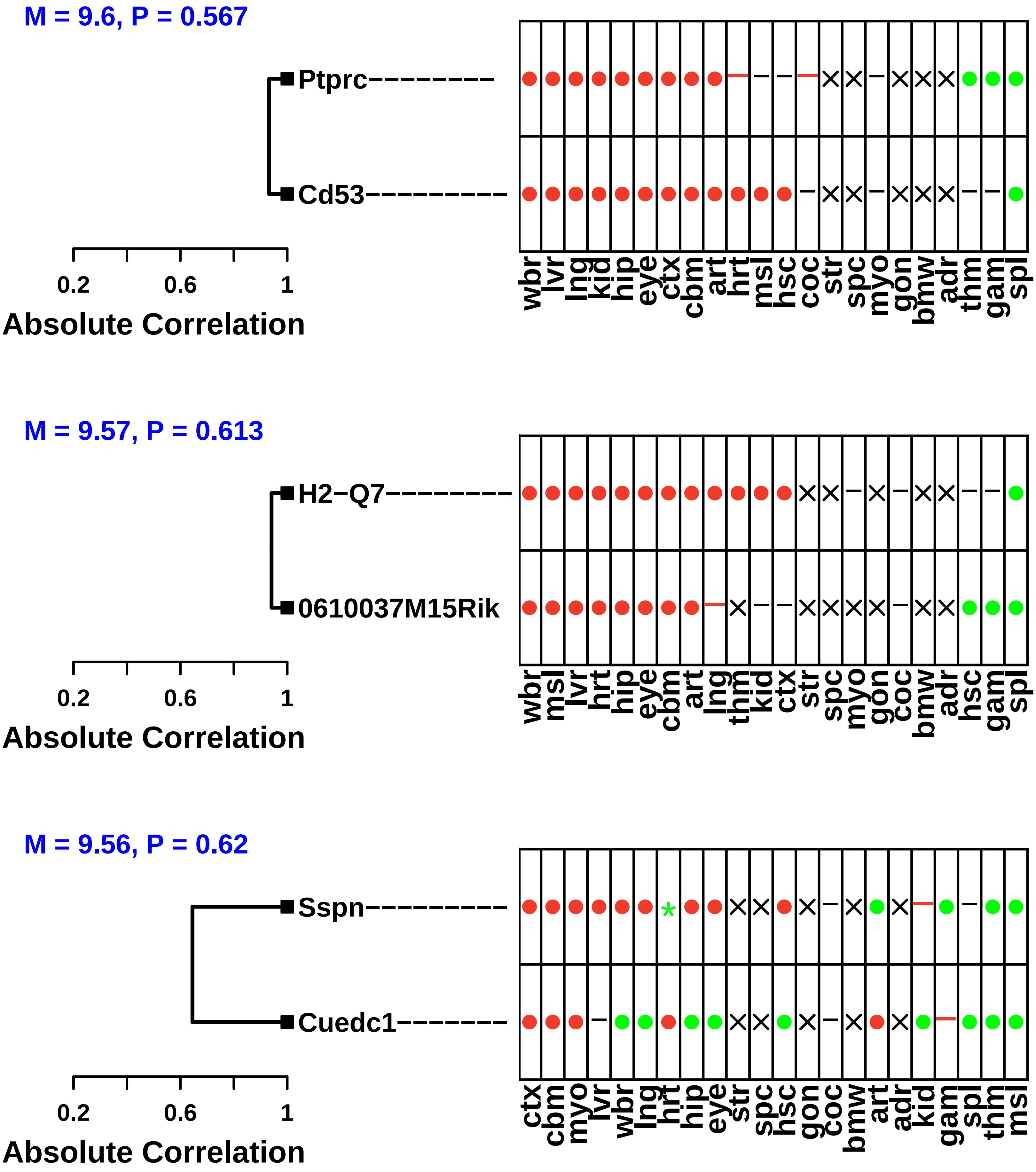

Aging−Regulated Modules (2 Genes)

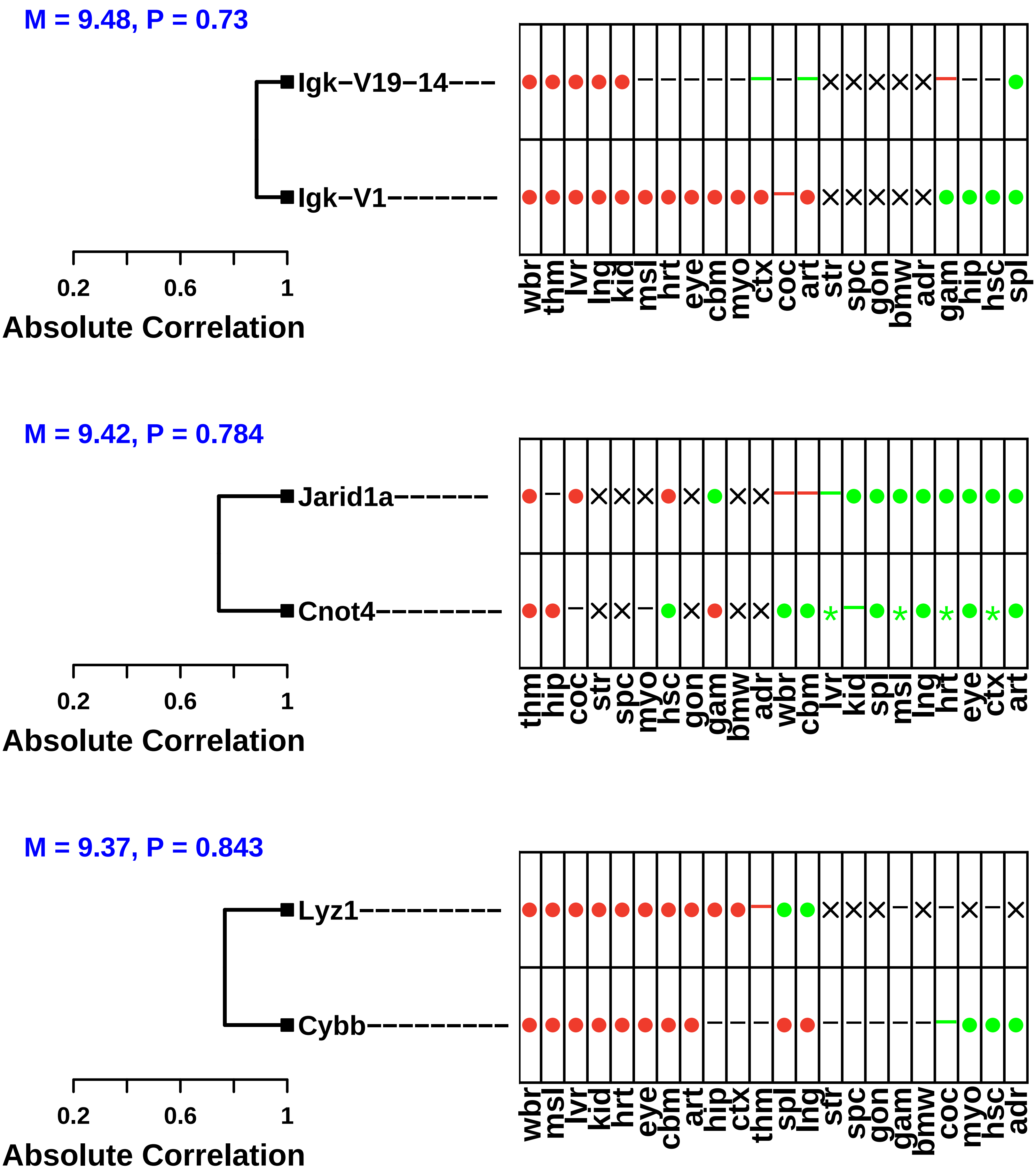

Aging−Regulated Modules (2 Genes)

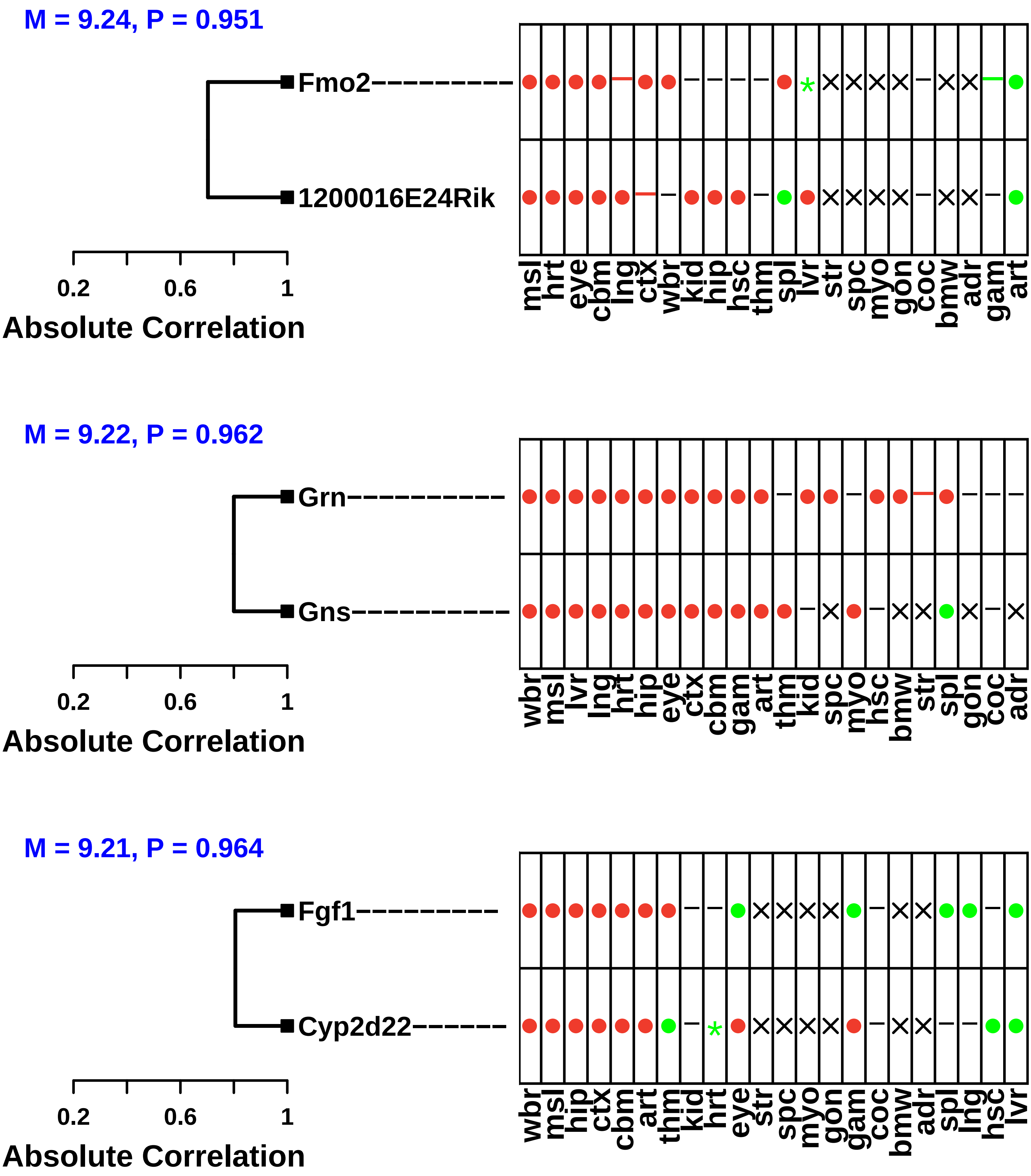

Aging−Regulated Modules (2 Genes)

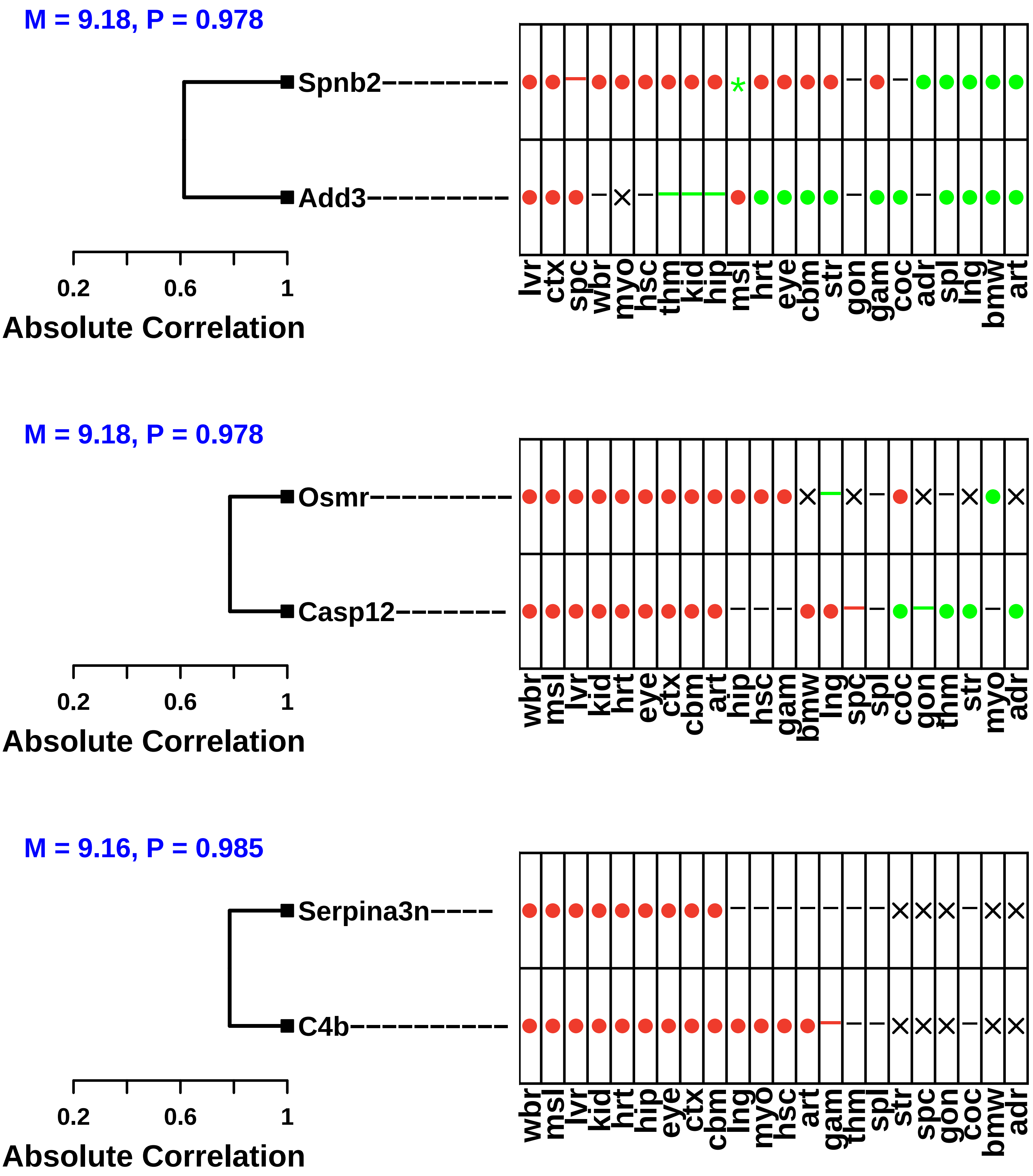

Aging−Regulated Modules (2 Genes)

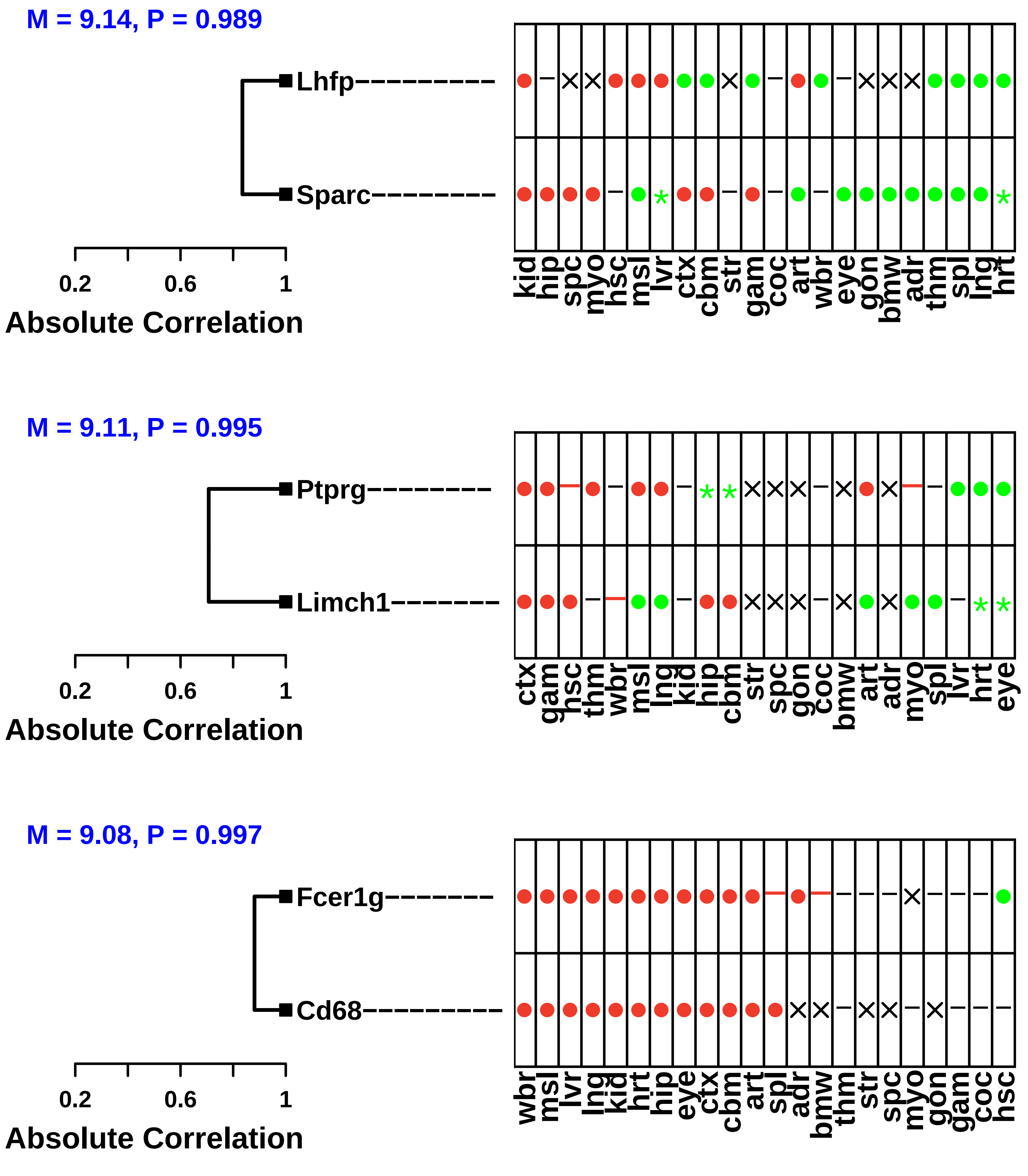

Aging−Regulated Modules (2 Genes)

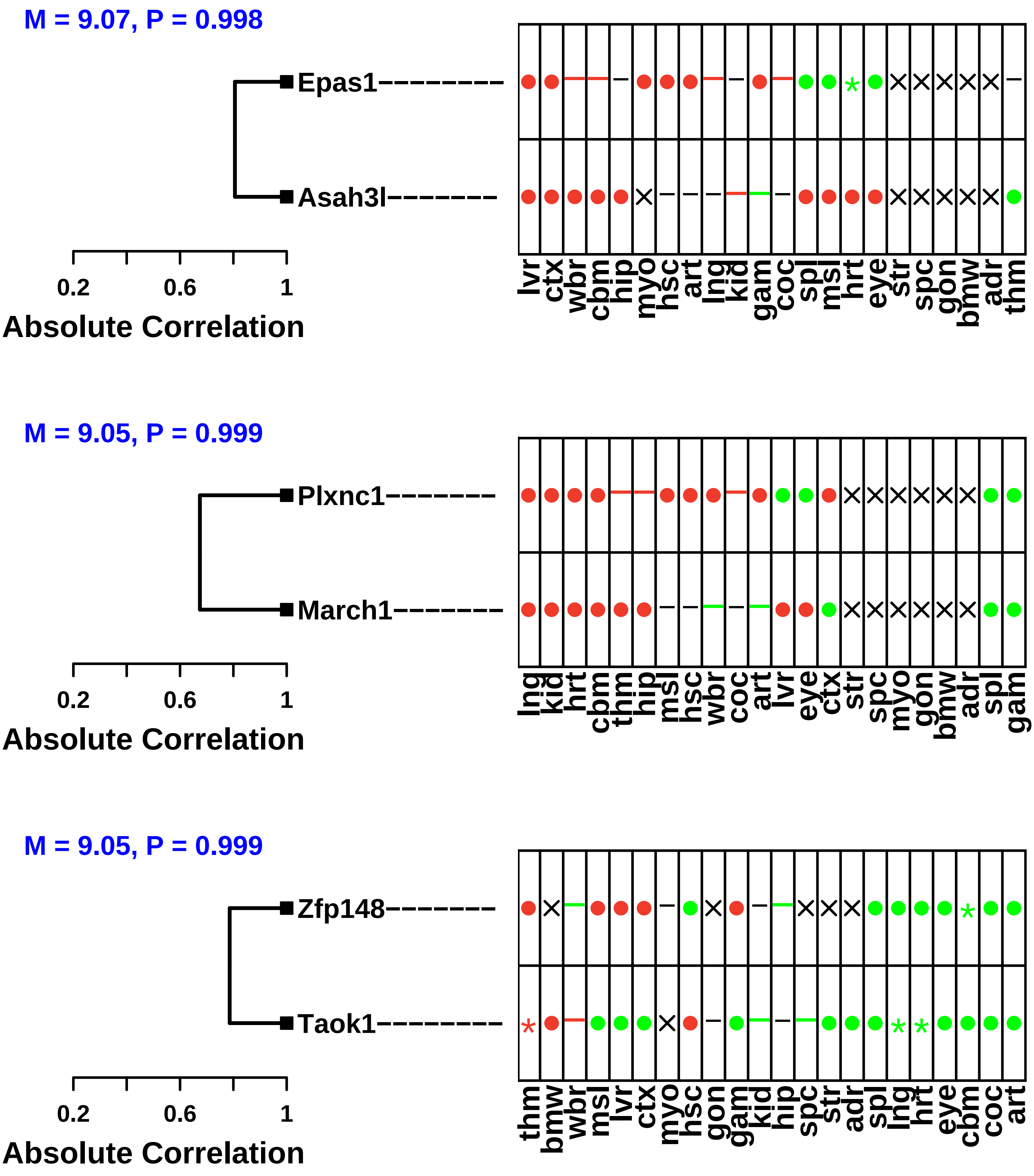

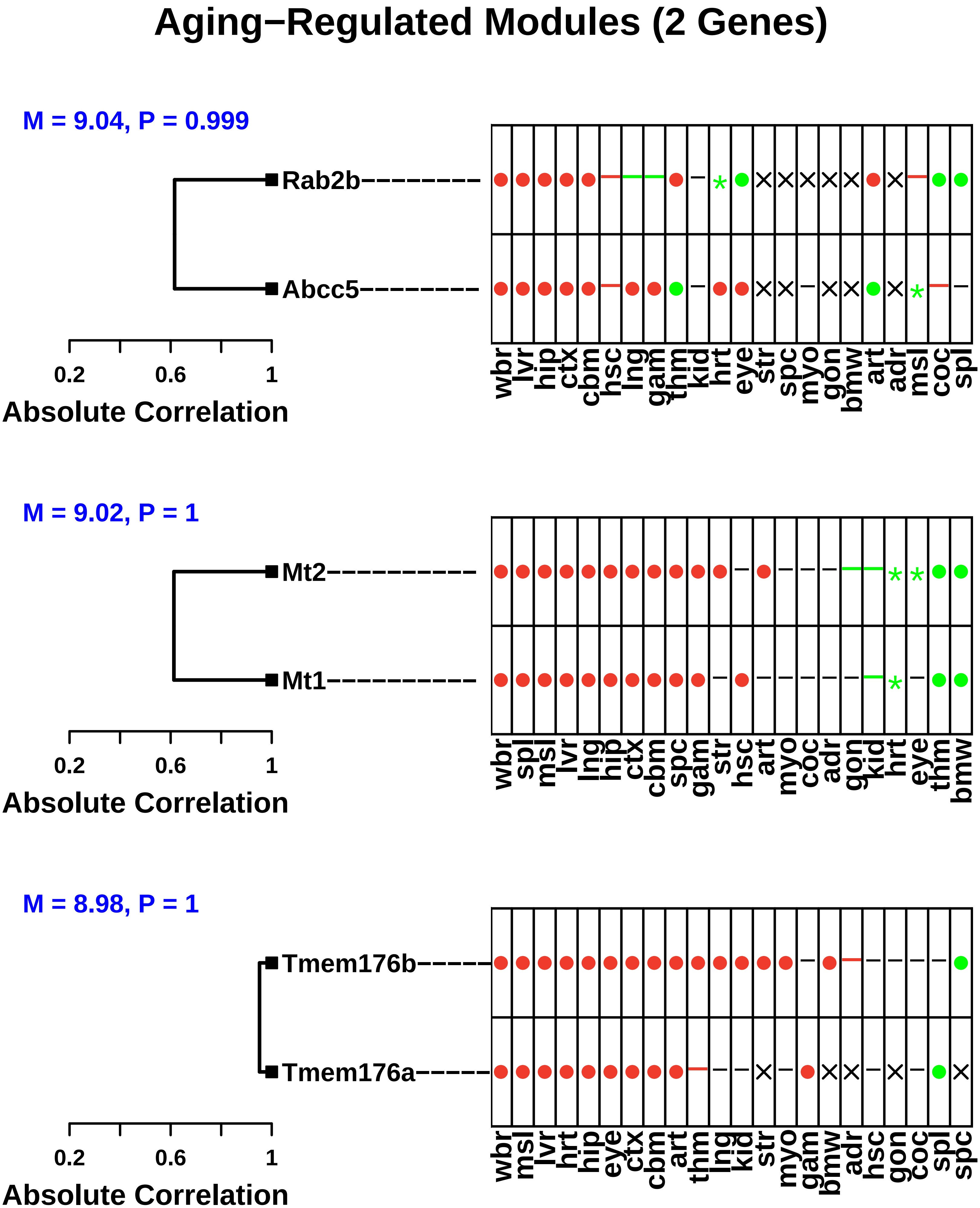

Aging−Regulated Modules (2 Genes)
M = 9.04, P = 0.999
Rab2b
Abcc5
0.2
0.6
1
Absolute Correlation
wbr lvr hip ctx cbm hsc lng gam thm kid hrt eye str spc myo gon bmw art adr msl coc spl
M = 9.02, P = 1
Mt2
Mt1
0.2
0.6
1
Absolute Correlation
wbr spl msl lvr lng hip ctx cbm spc gam str hsc art myo coc adr gon kid hrt eye thm bmw
M = 8.98, P = 1
Tmem176b
Tmem176a
0.2
0.6
1
Absolute Correlation
wbr msl lvr hrt hip eye ctx cbm art thm lng kid str myo gam bmw adr hsc gon coc spl spc

Aging−Regulated Modules (2 Genes)

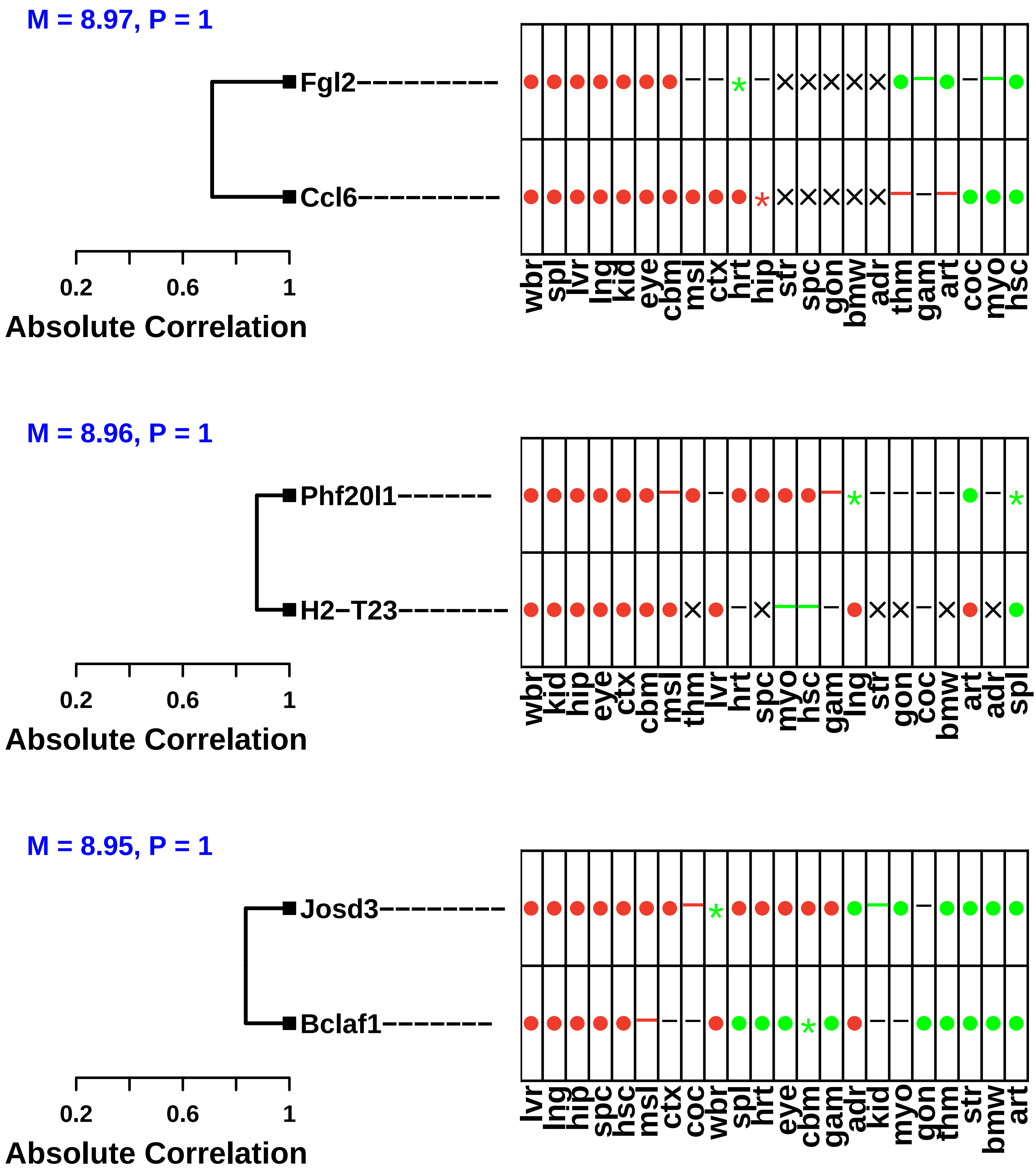

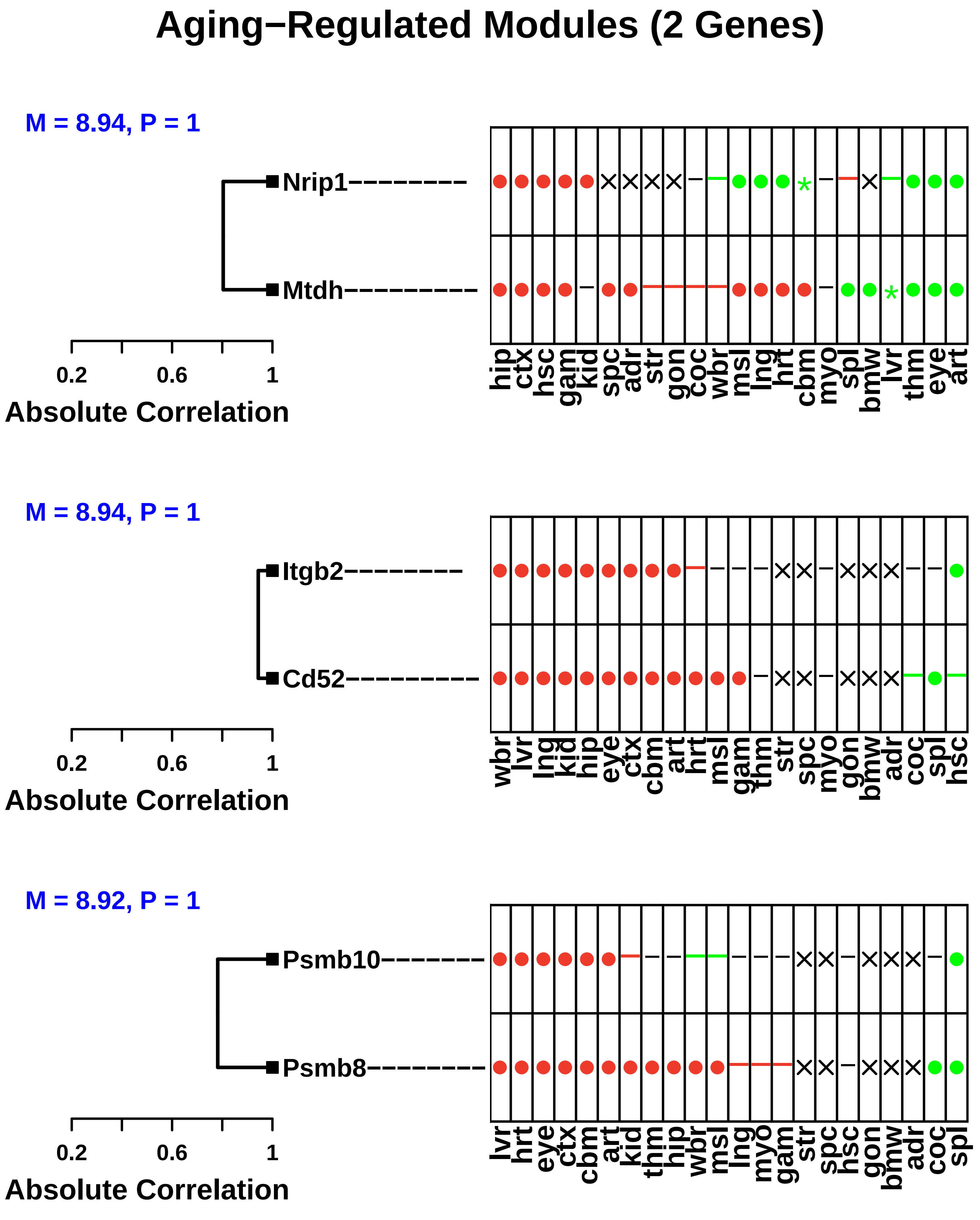

Aging−Regulated Modules (2 Genes)
M = 8.94, P = 1
Nrip1
Mtdh
0.2
0.6
1
Absolute Correlation
hip ctx hsc gam kid spc adr str gon coc wbr msl lng hrt cbm myo spl bmw lvr thm eye art
M = 8.94, P = 1
Itgb2
Cd52
0.2
0.6
1
Absolute Correlation
wbr lvr lng kid hip eye ctx cbm art hrt msl gam thm str spc myo gon bmw adr coc spl hsc
M = 8.92, P = 1
Psmb10
Psmb8
0.2
0.6
1
Absolute Correlation
lvr hrt eye ctx cbm art kid thm hip wbr msl lng myo gam str spc hsc gon bmw adr coc spl

Aging−Regulated Modules (2 Genes)

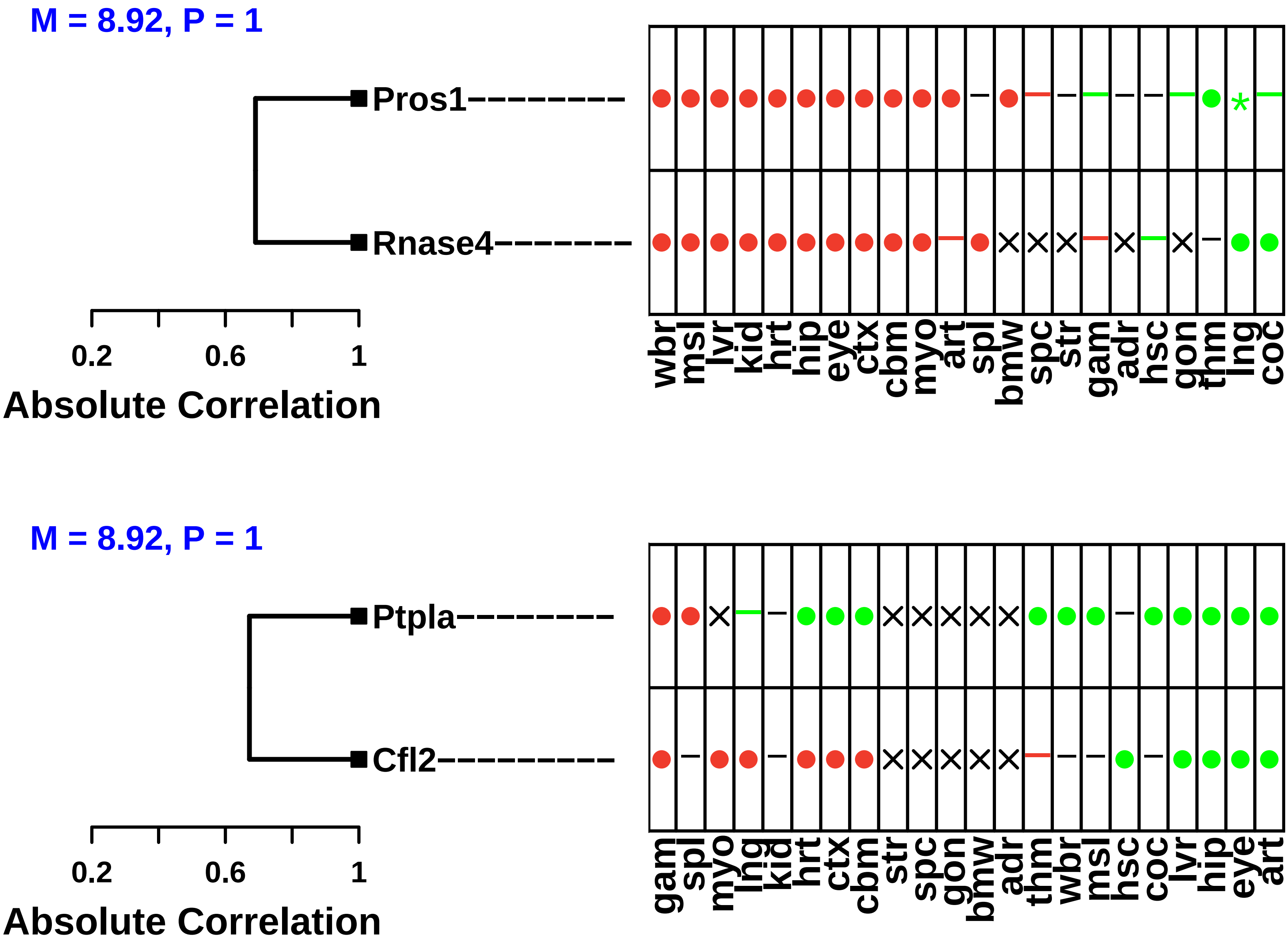

Age−Regulated Modules (3 Genes)

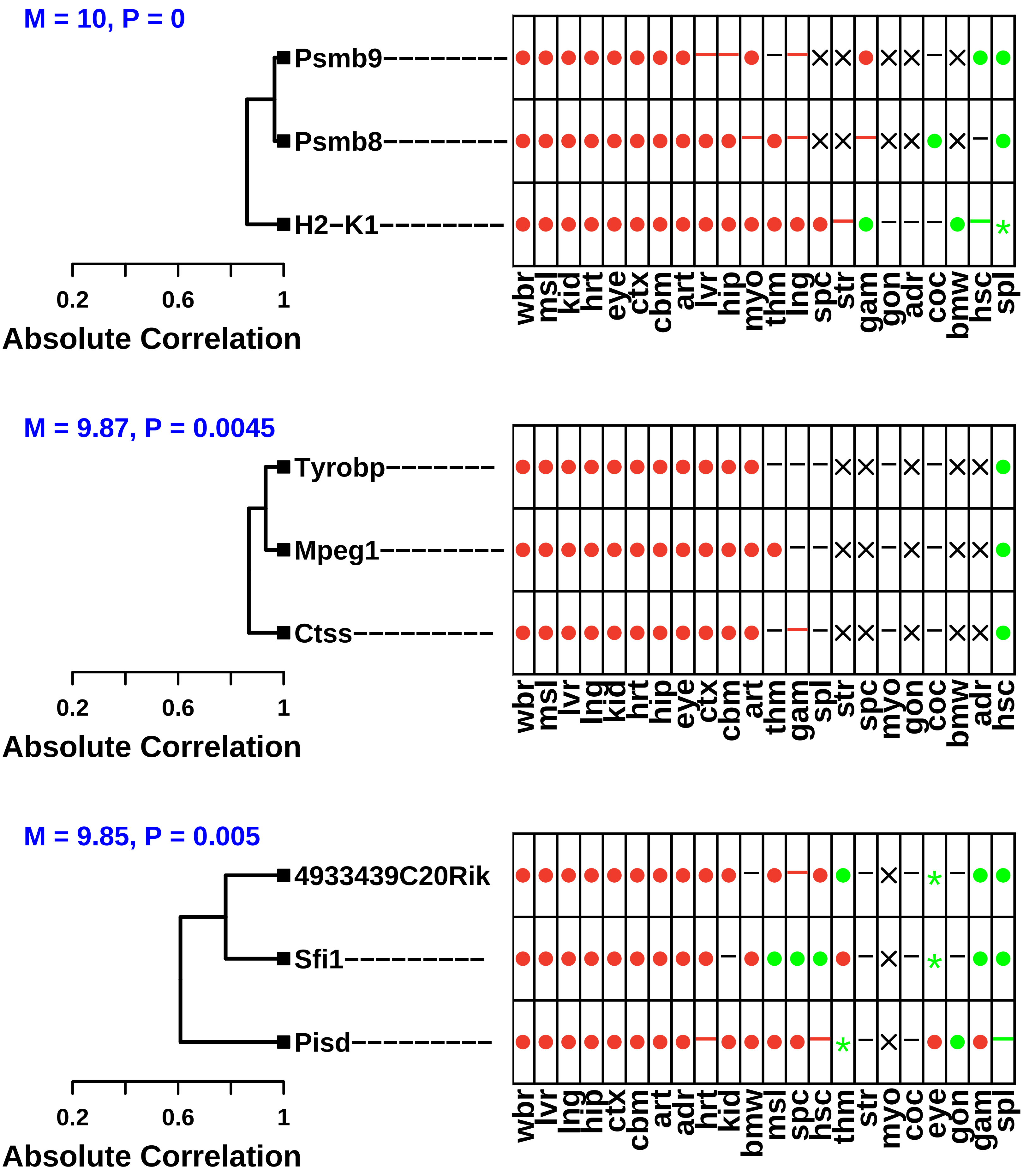

Age−Regulated Modules (3 Genes)

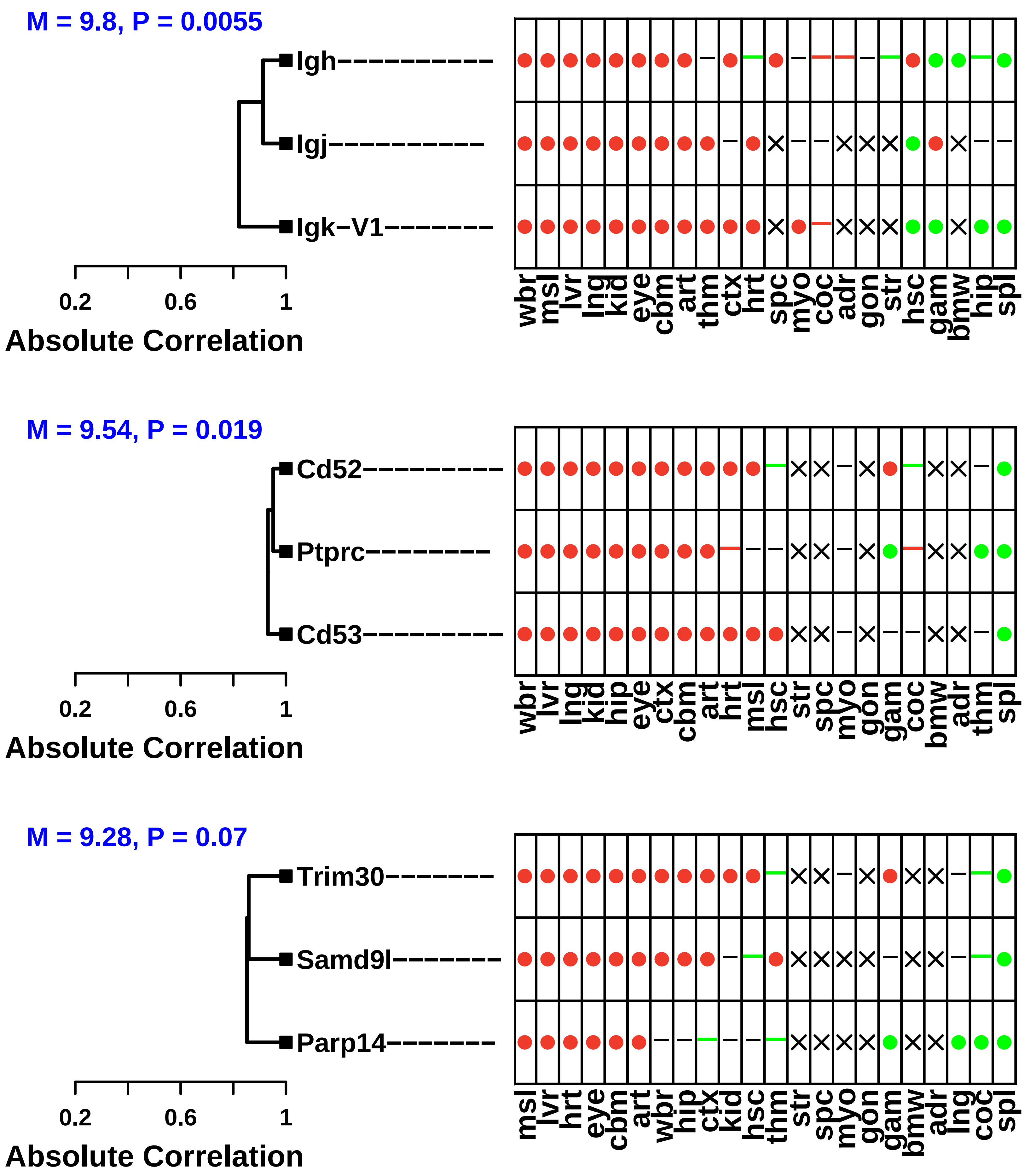

Age−Regulated Modules (3 Genes)

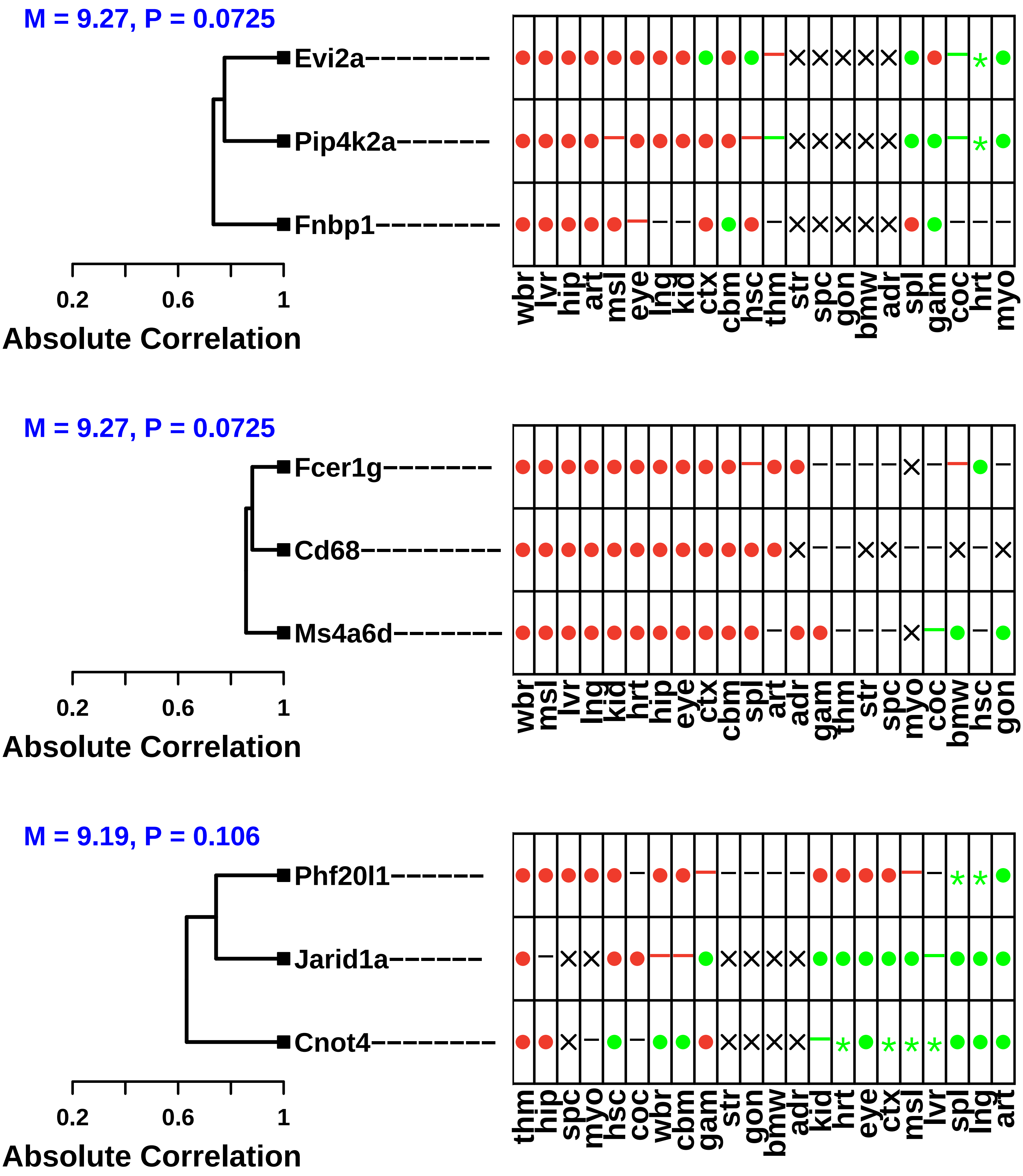

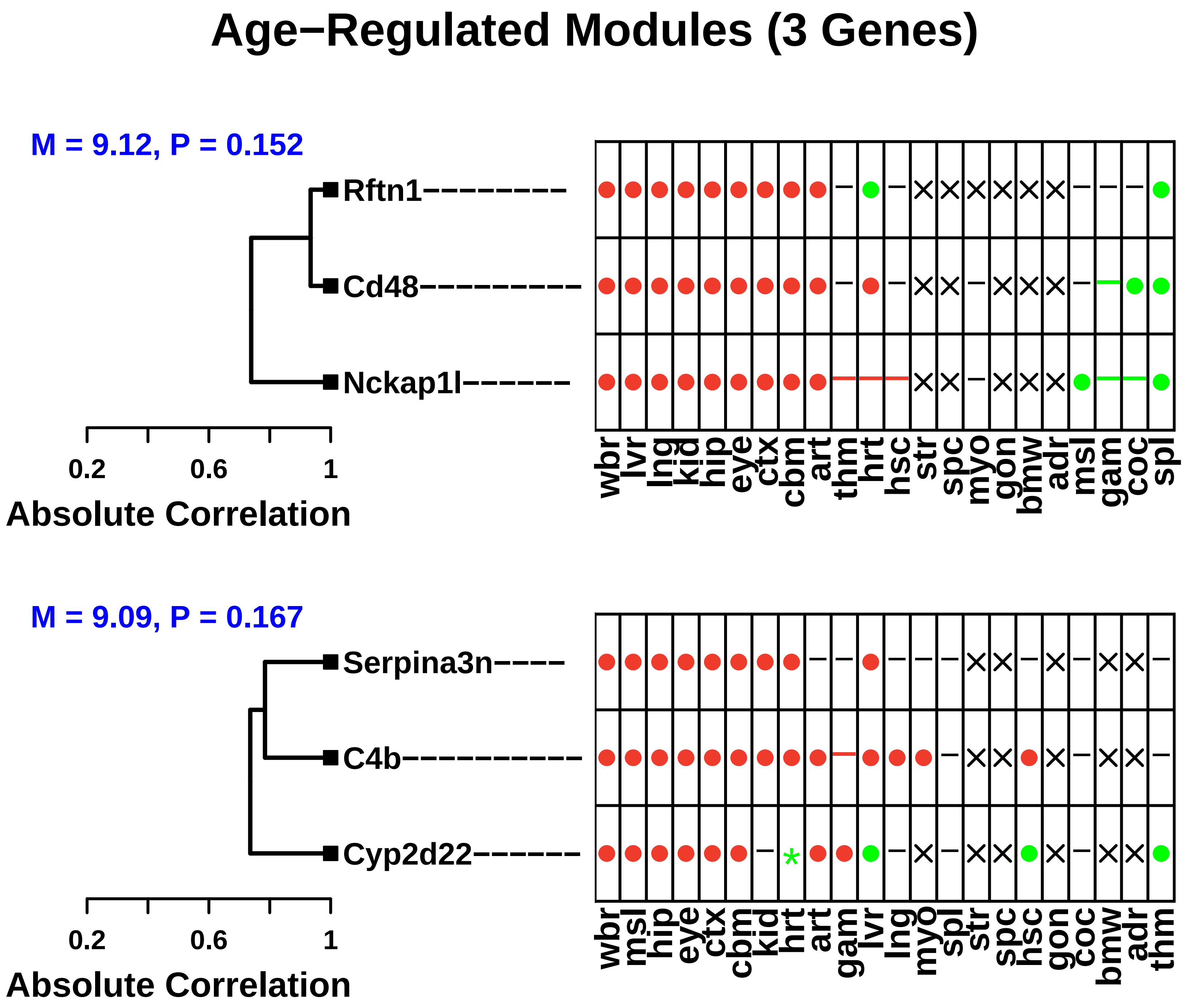
Age−Regulated Modules (3 Genes)
M = 9.12, P = 0.152
Rftn1
Cd48
Nckap1l
0.2
0.6
1
Absolute Correlation
wbr
lvr
lng
kid
hip
eye
ctx
cbm
art
thm
hrt
hsc
str
spc
myo
gon
bmw
adr
msl
gam
coc
spl
M = 9.09, P = 0.167
Serpina3n
C4b
Cyp2d22
0.2
0.6
1
Absolute Correlation
wbr
msl
hip
eye
ctx
cbm
kid
hrt
art
gam
lvr
lng
myo
spl
str
spc
hsc
gon
coc
bmw
adr
thm

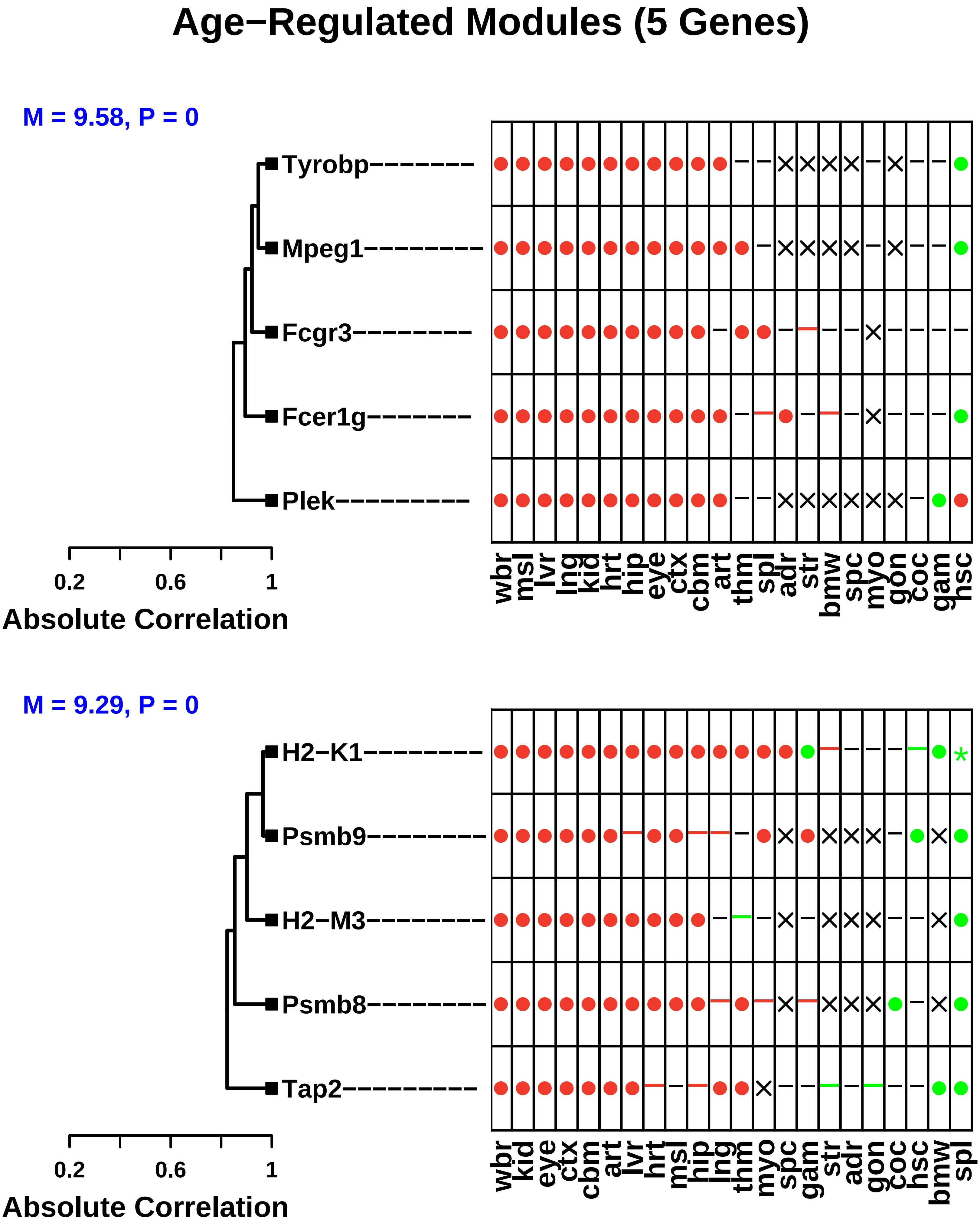
Age−Regulated Modules (5 Genes)
M = 9.58, P = 0
Tyrobp
Mpeg1
Fcgr3
Fcer1g
Plek
0.2
0.6
1
Absolute Correlation
wbr
msl
lvr
lng
kid
hrt
hip
eye
ctx
cbm
art
thm
spl
adr
str
bmw
spc
myo
gon
coc
gam
hsc
M = 9.29, P = 0
H2−K1
Psmb9
H2−M3
Psmb8
Tap2
0.2
0.6
1
Absolute Correlation
wbr
kid
eye
ctx
cbm
art
lvr
hrt
msl
hip
lng
thm
myo
spc
gam
str
adr
gon
coc
hsc
bmw
spl

Age−Regulated Modules (5 Genes)

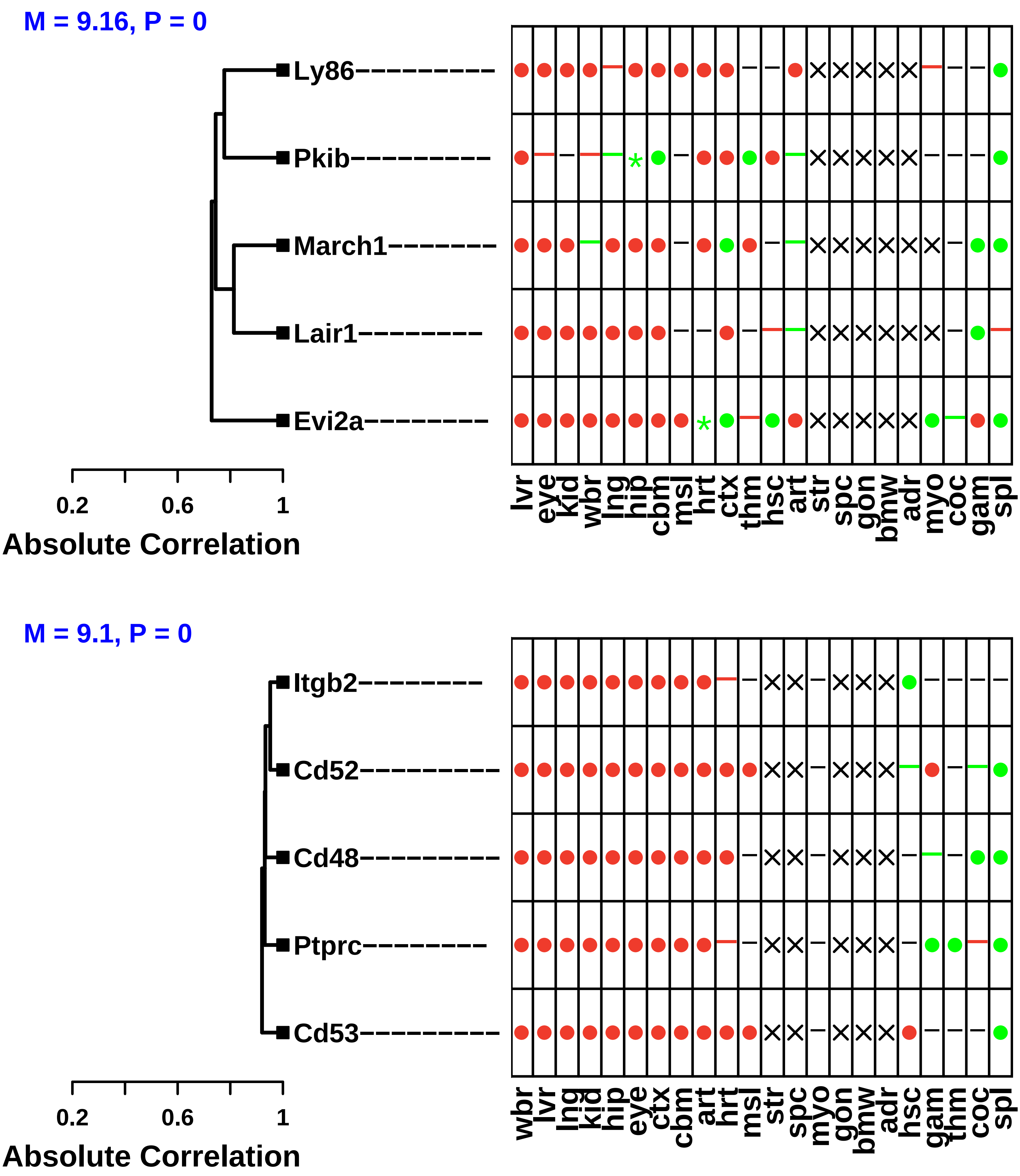

Age−Regulated Modules (5 Genes)

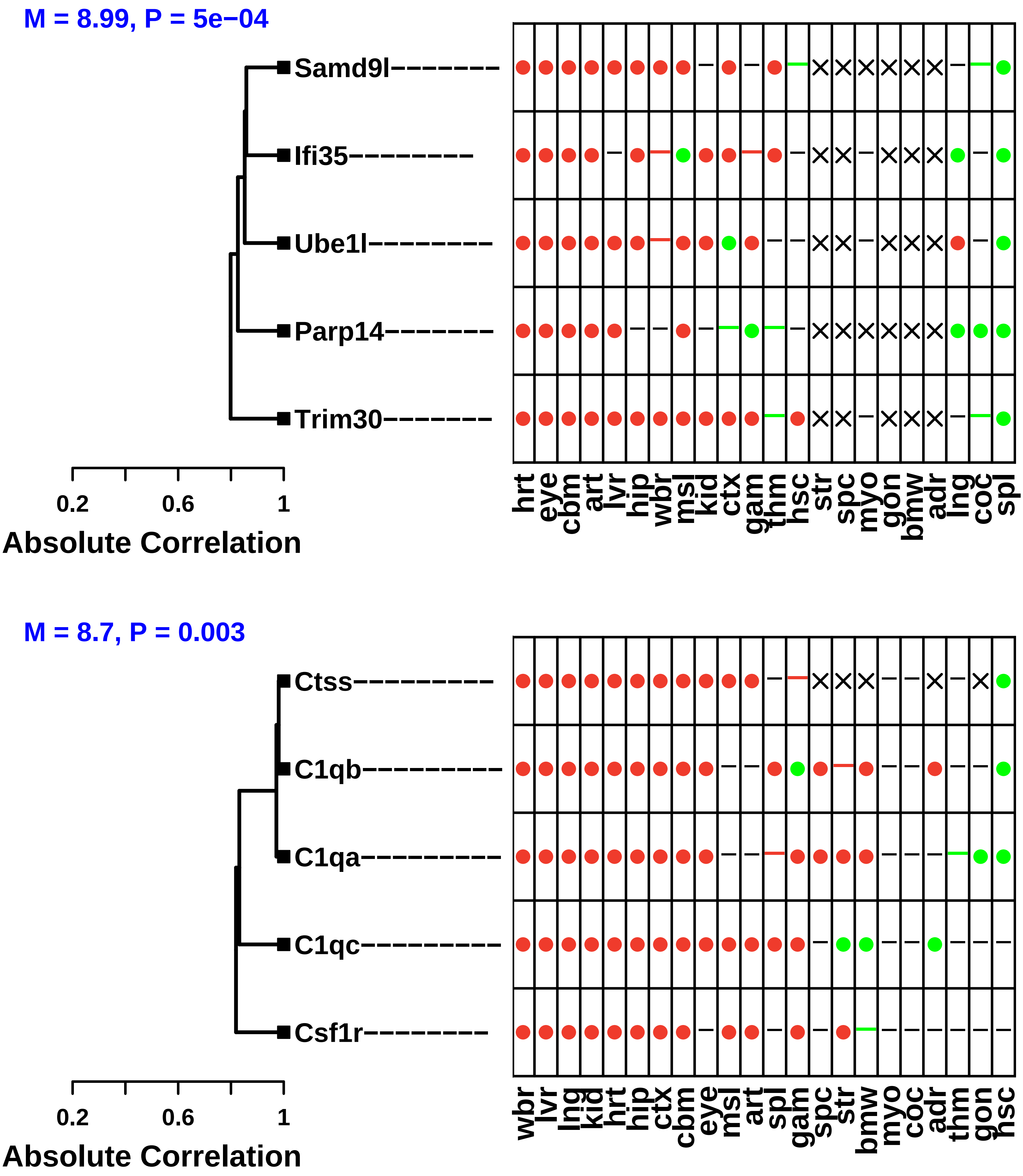

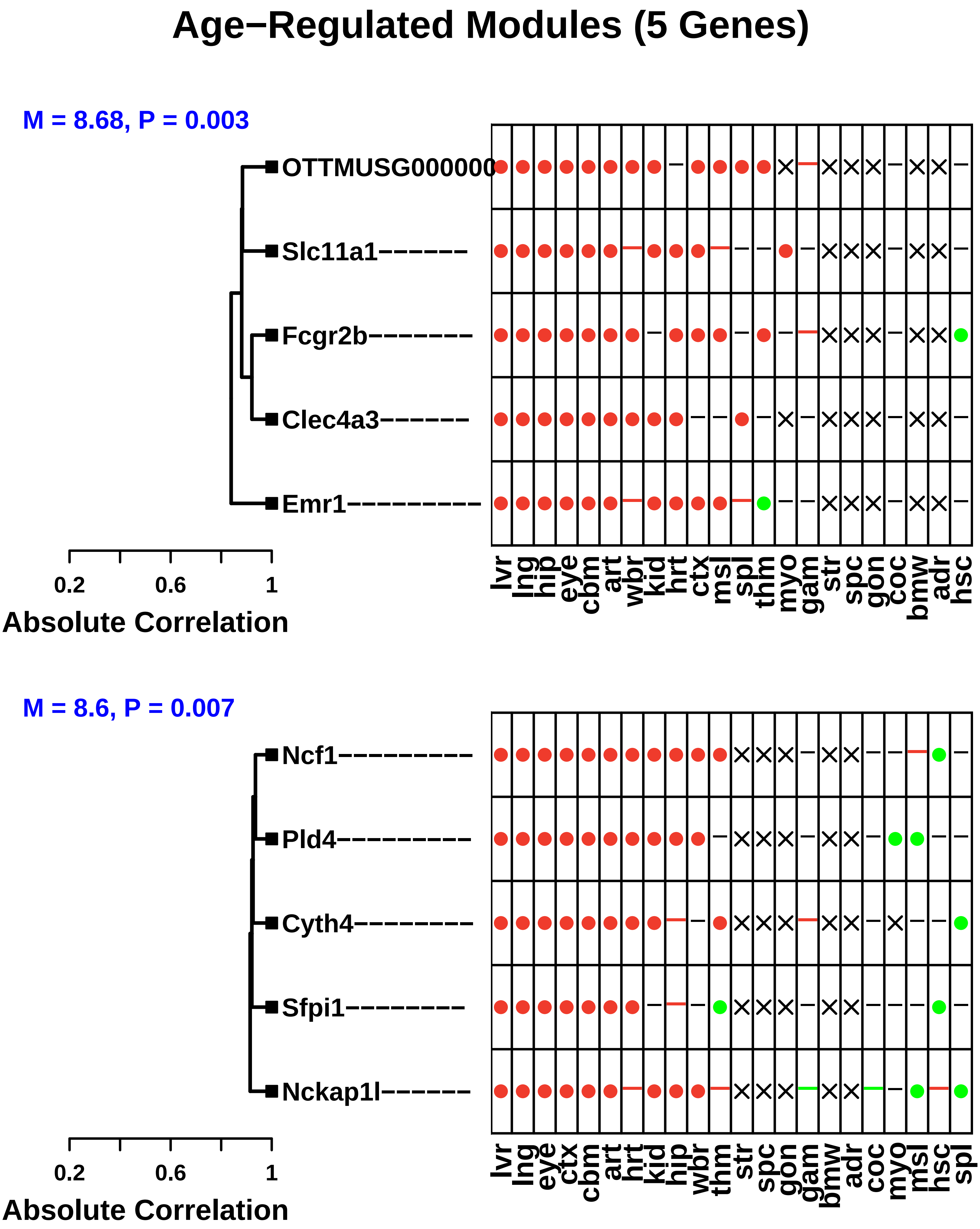

Age−Regulated Modules (5 Genes)
M = 8.68, P = 0.003
OTTMUSG000000
Slc11a1
Fcgr2b
Clec4a3
Emr1
0.2
0.6
1
Absolute Correlation
lvr lng hip eye cbm art wbr kid hrt ctx msl spl thm myo gam str spc gon coc bmw adr hsc
M = 8.6, P = 0.007
Ncf1
Pld4
Cyth4
Sfpi1
Nckap1l
0.2
0.6
1
Absolute Correlation
lvr lng eye ctx cbm art hrt kid hip wbr thm str spc gon gam bmw adr coc myo msl hsc spl

Age−Regulated Modules (5 Genes)

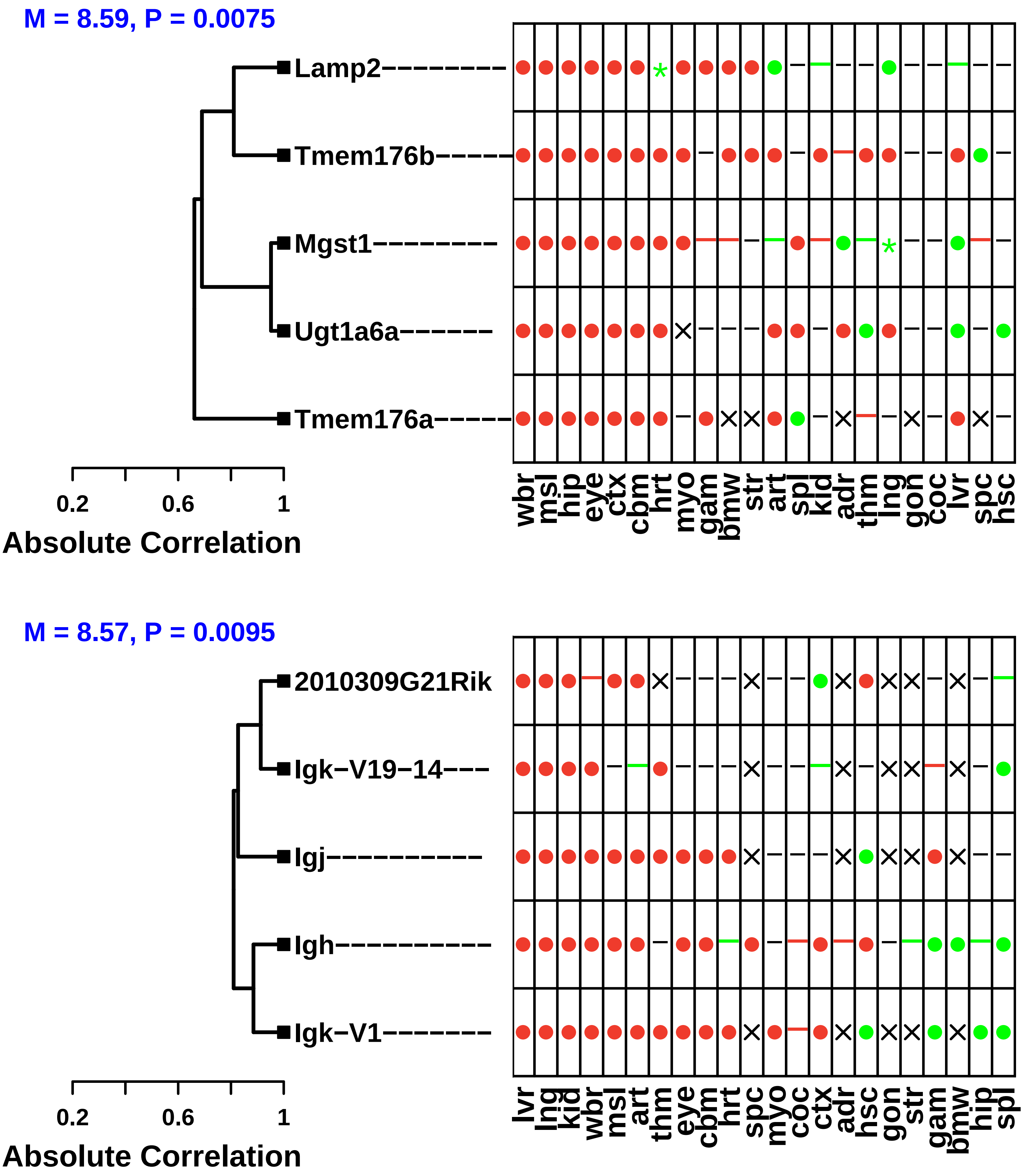

Age−Regulated Modules (5 Genes)

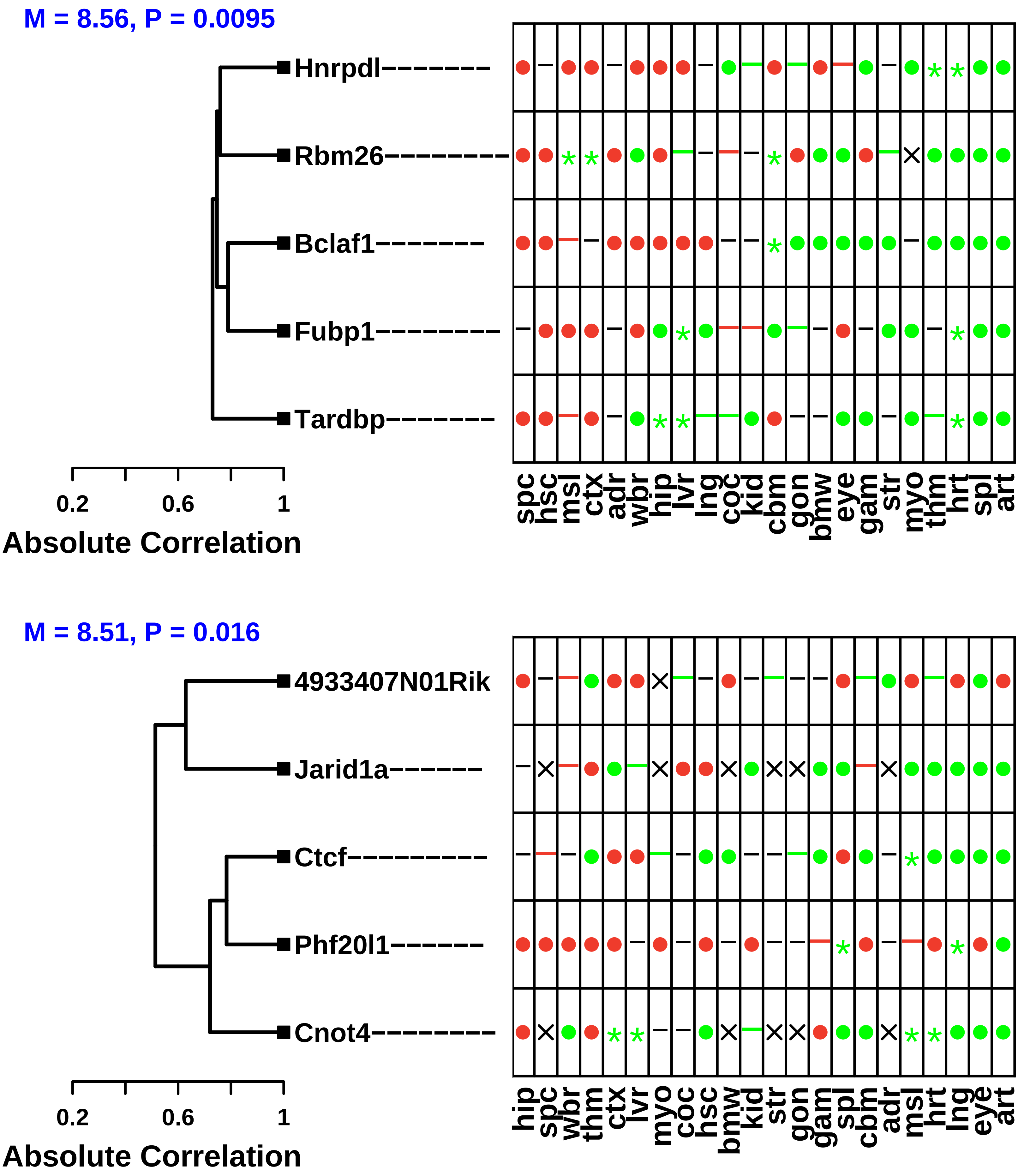

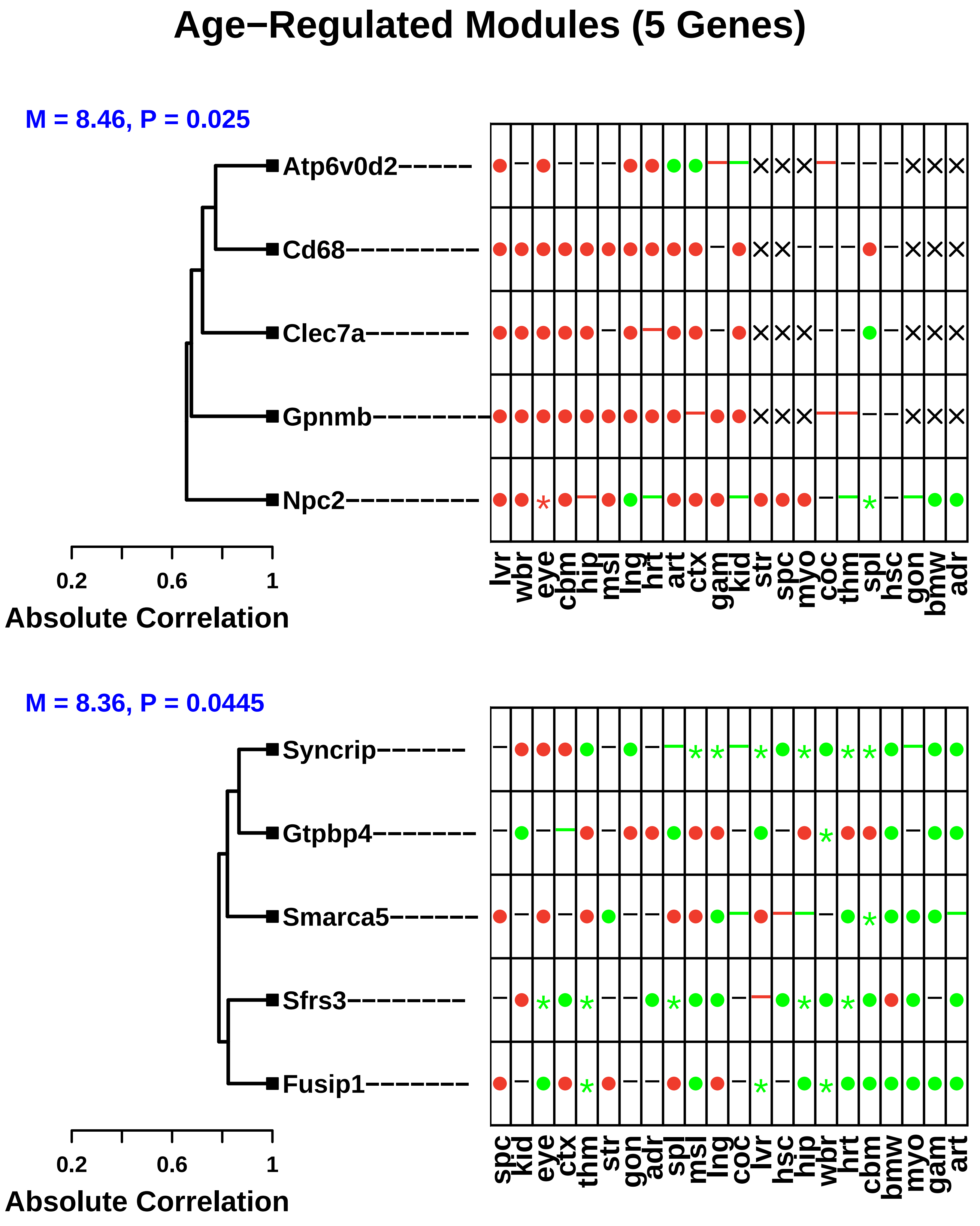
Age−Regulated Modules (5 Genes)
M = 8.46, P = 0.025
Atp6v0d2
Cd68
Clec7a
Gpnmb
Npc2
0.2
0.6
1
Absolute Correlation
lvr wbr eye cbm hip msl lng hrt art ctx gam kid str spc myo coc thm spl hsc gon bmw adr
M = 8.36, P = 0.0445
Syncrip
Gtpbp4
Smarca5
Sfrs3
Fusip1
0.2
0.6
1
Absolute Correlation
spc kid eye ctx thm str gon adr spl msl lng coc lvr hsc hip wbr hrt cbm bmw myo gam art

Age−Regulated Modules (5 Genes)

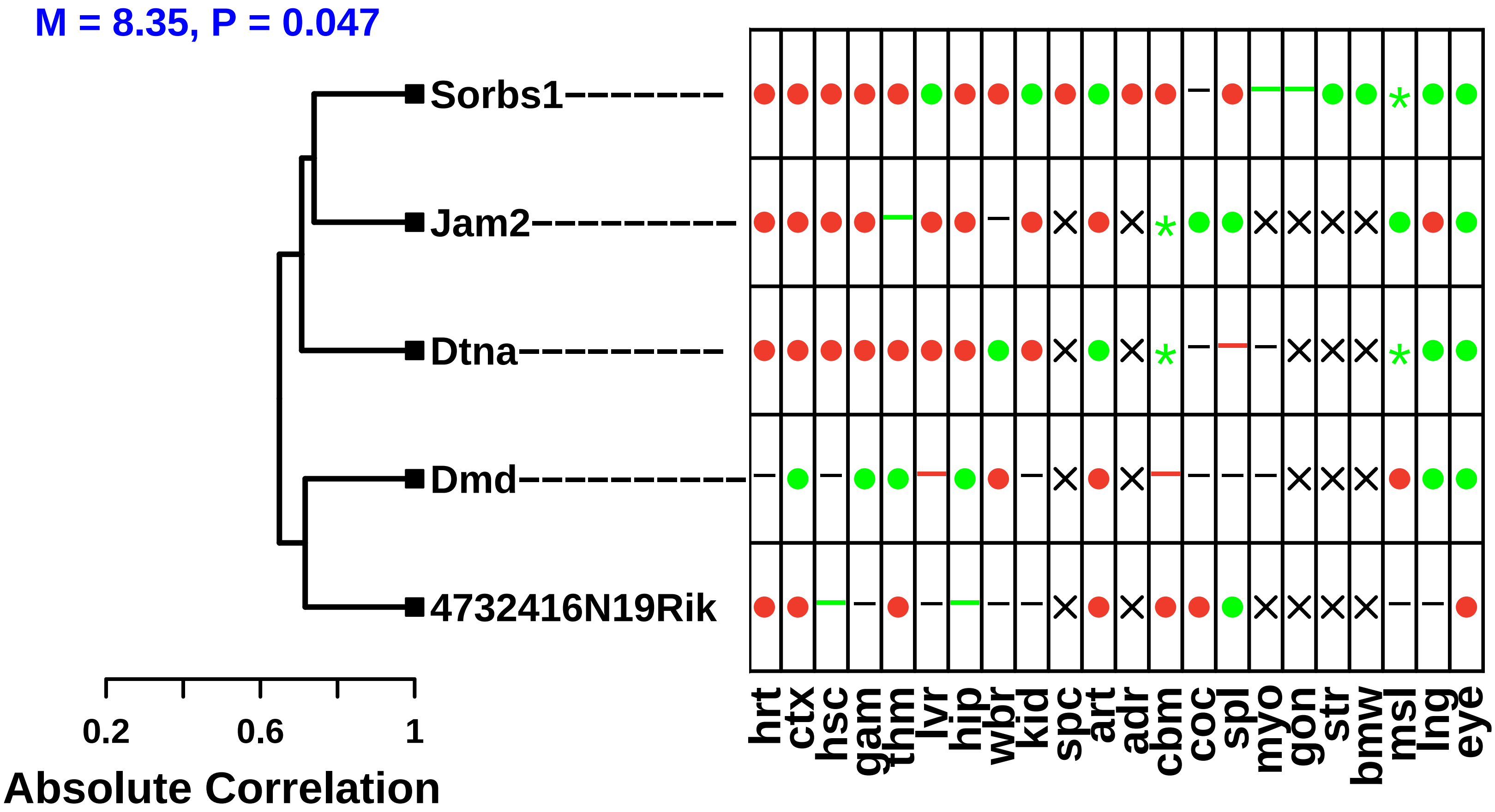

Age−Regulated Modules (10 Genes)

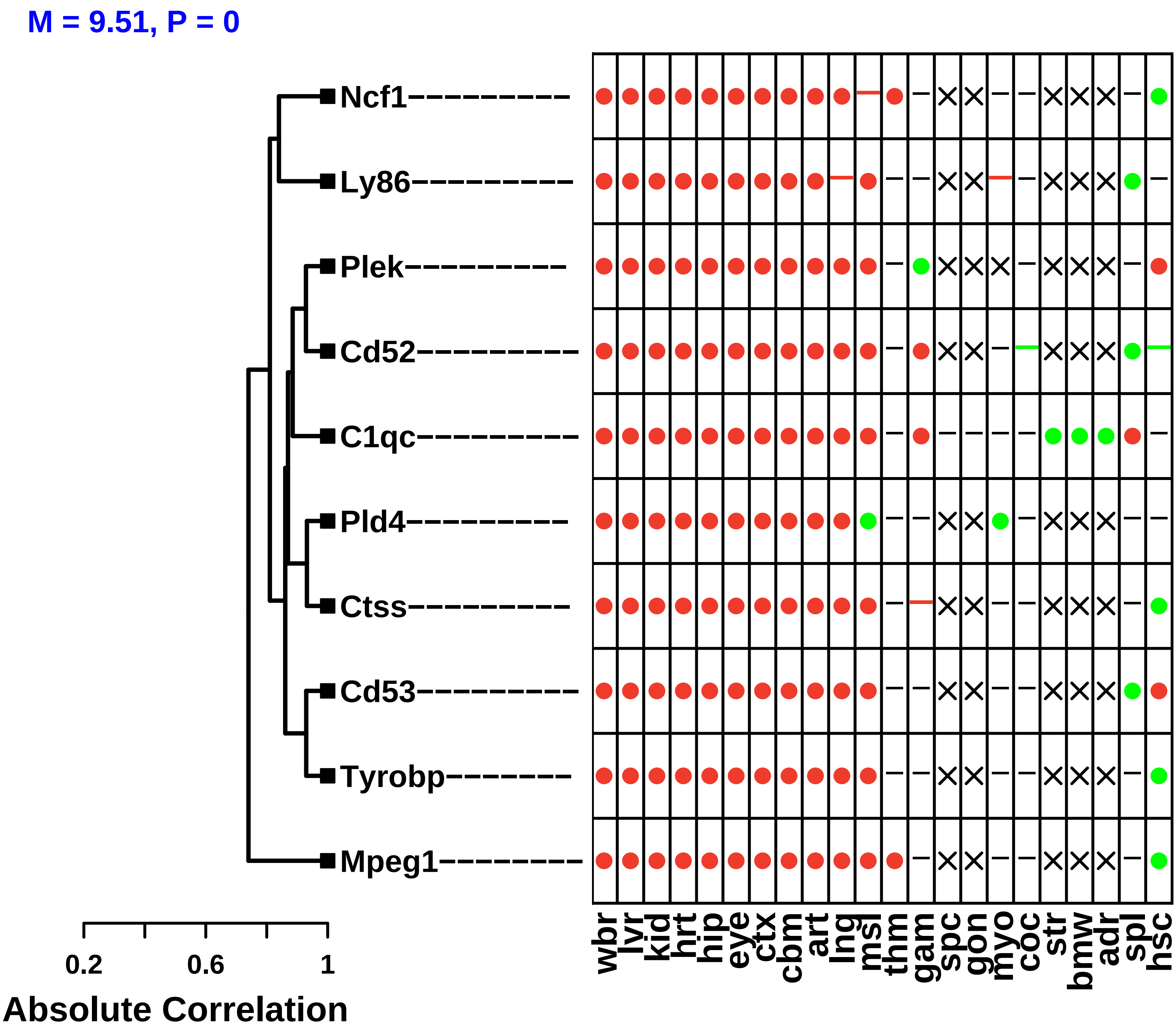

Age−Regulated Modules (10 Genes)

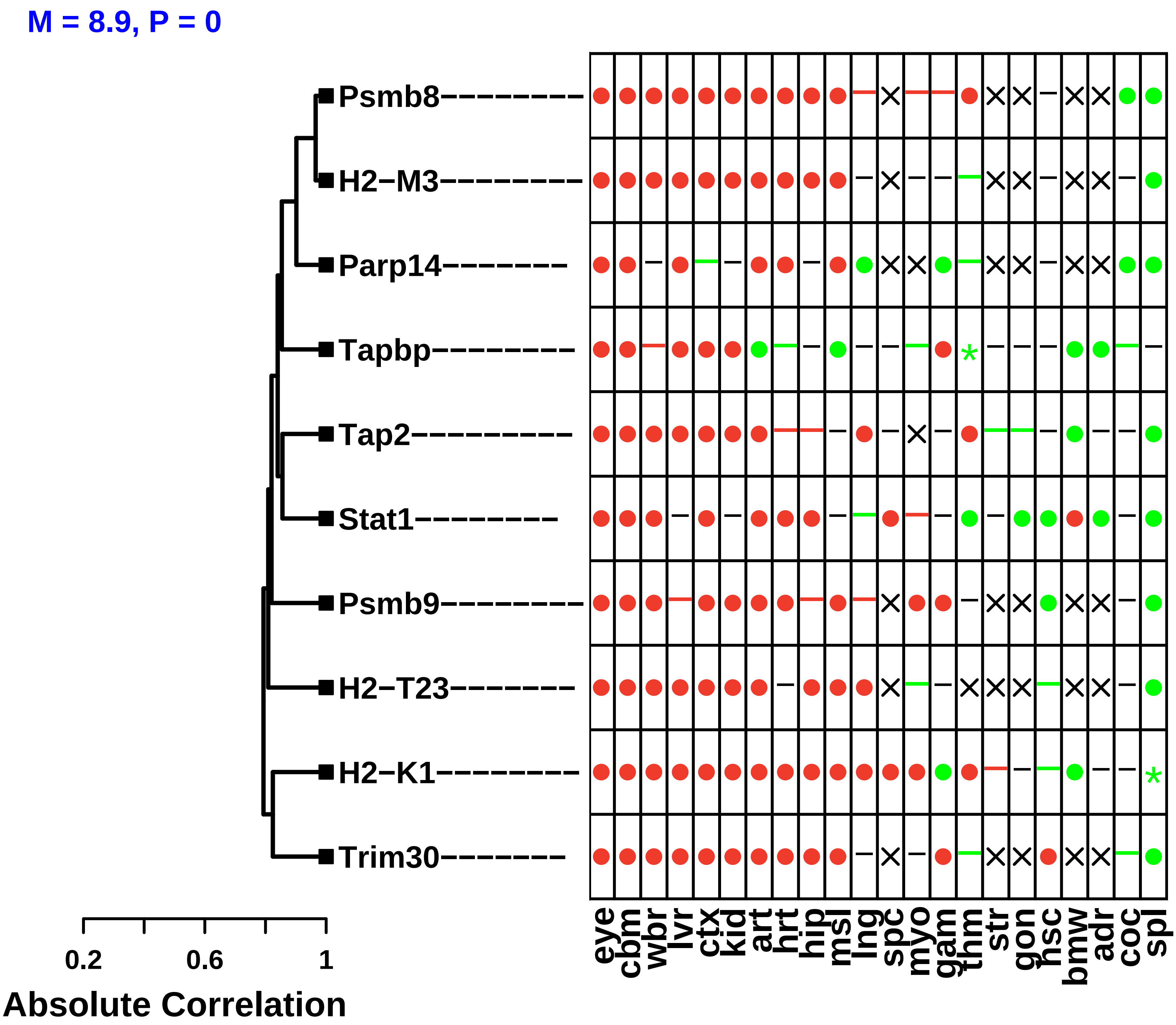

Age−Regulated Modules (10 Genes)

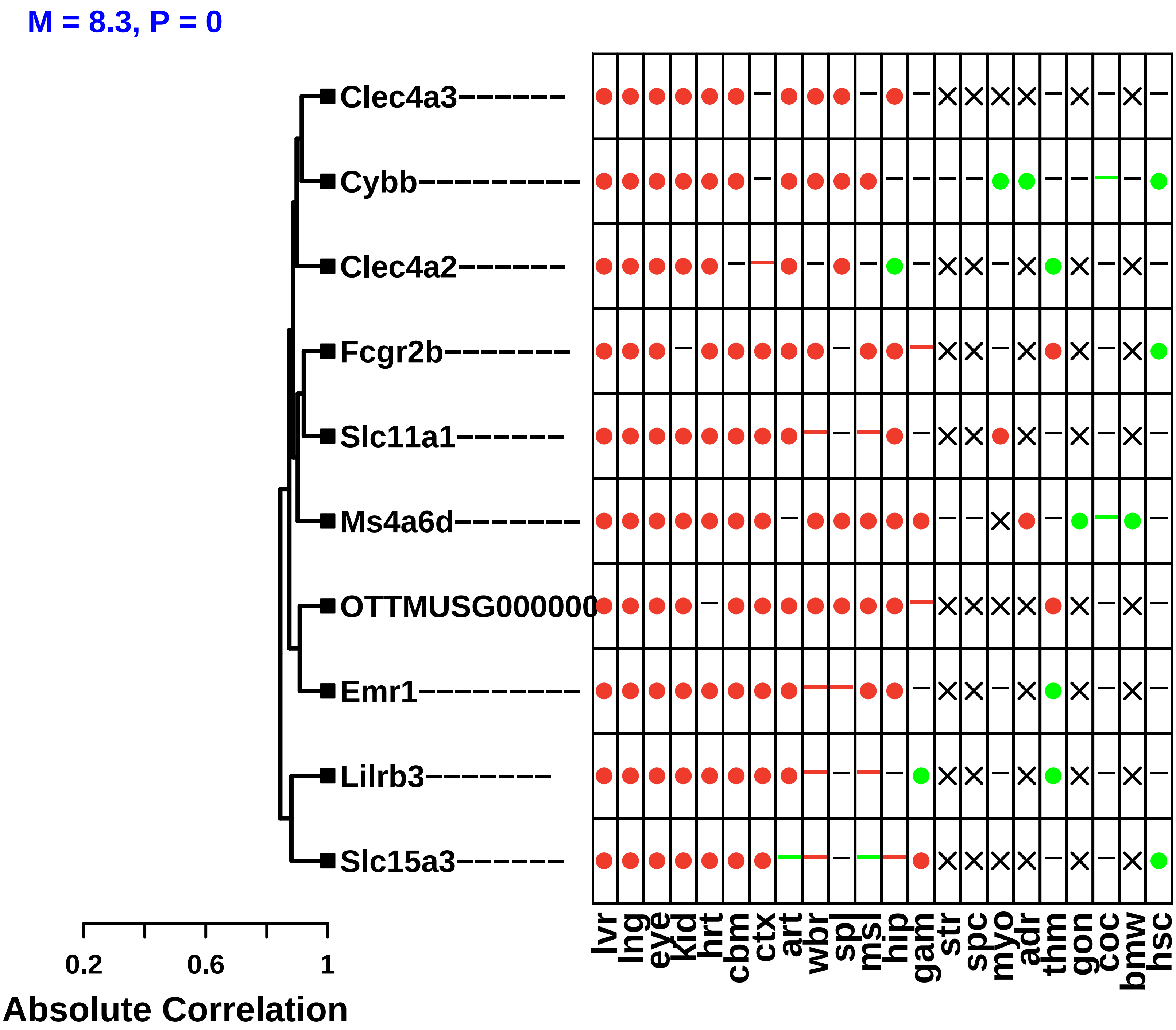

Age−Regulated Modules (10 Genes)

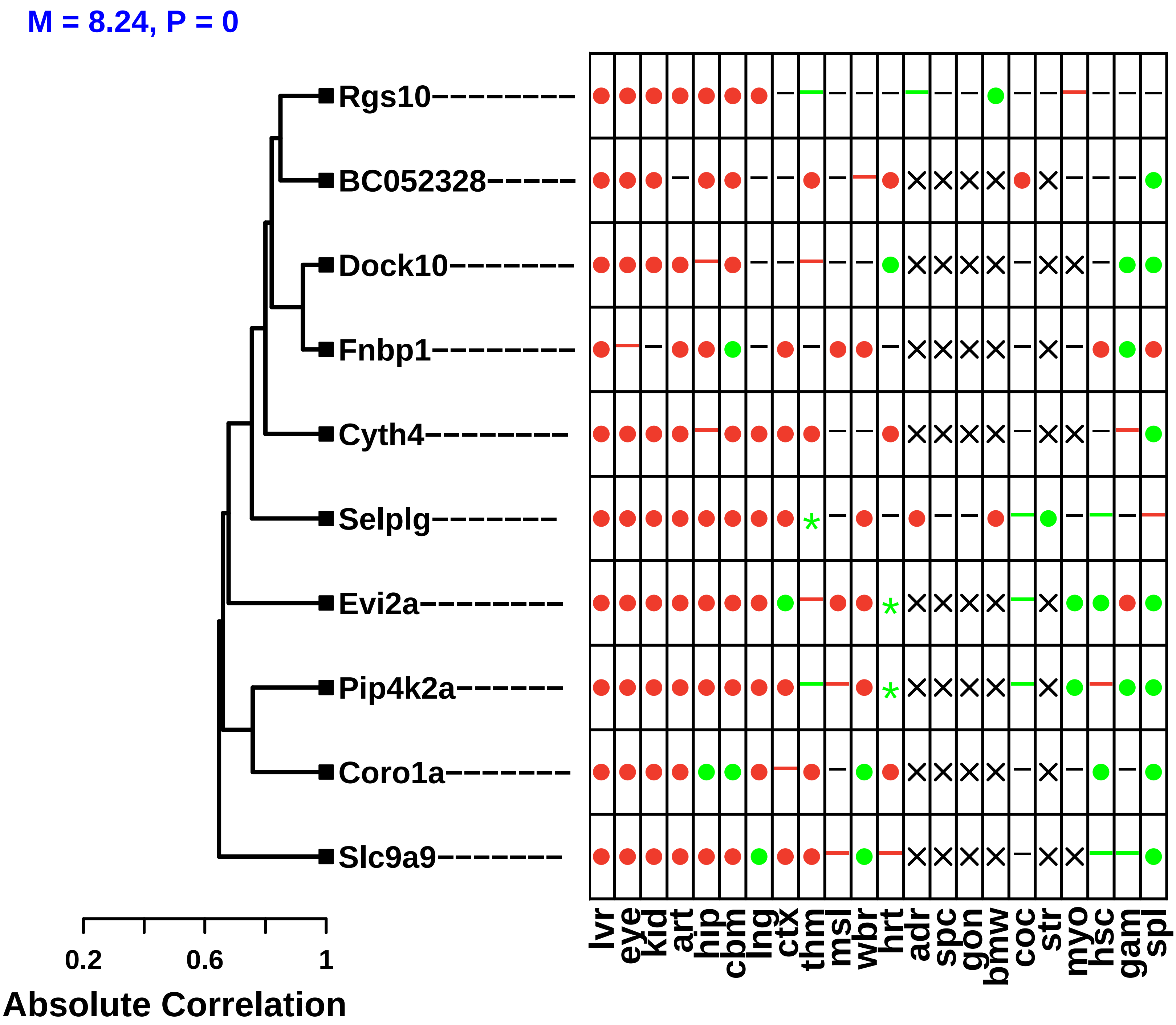

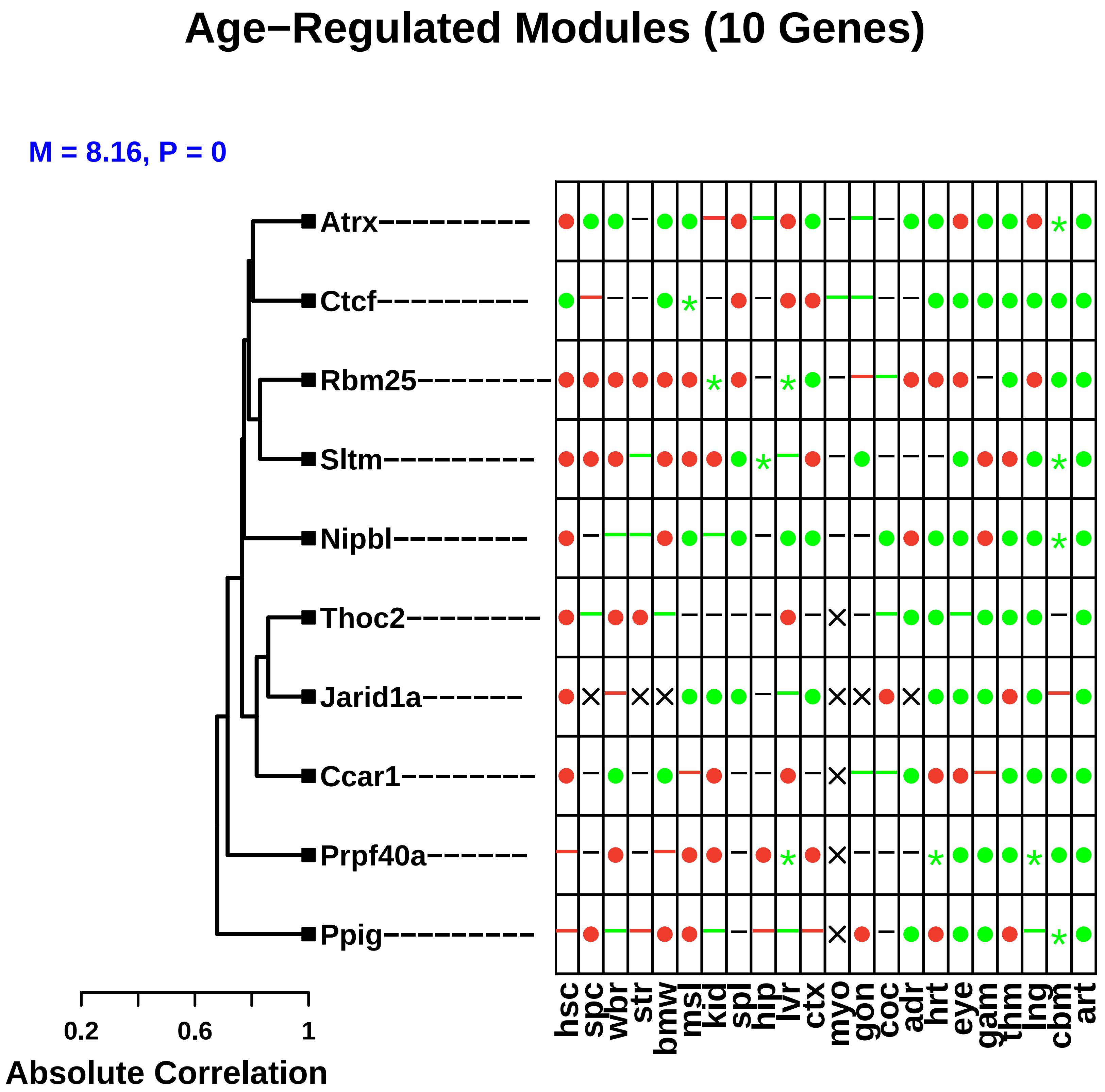
Age−Regulated Modules (10 Genes)
M = 8.16, P = 0
Atrx
Ctcf
Rbm25
Sltm
Nipbl
Thoc2
Jarid1a
Ccar1
Prpf40a
Ppig
0.2
0.6
1
Absolute Correlation
hsc
spc
wbr
str
bmw
msl
kid
spl
hip
lvr
ctx
myo
gon
coc
adr
hrt
eye
gam
thm
lng
cbm
art

Age−Regulated Modules (10 Genes)

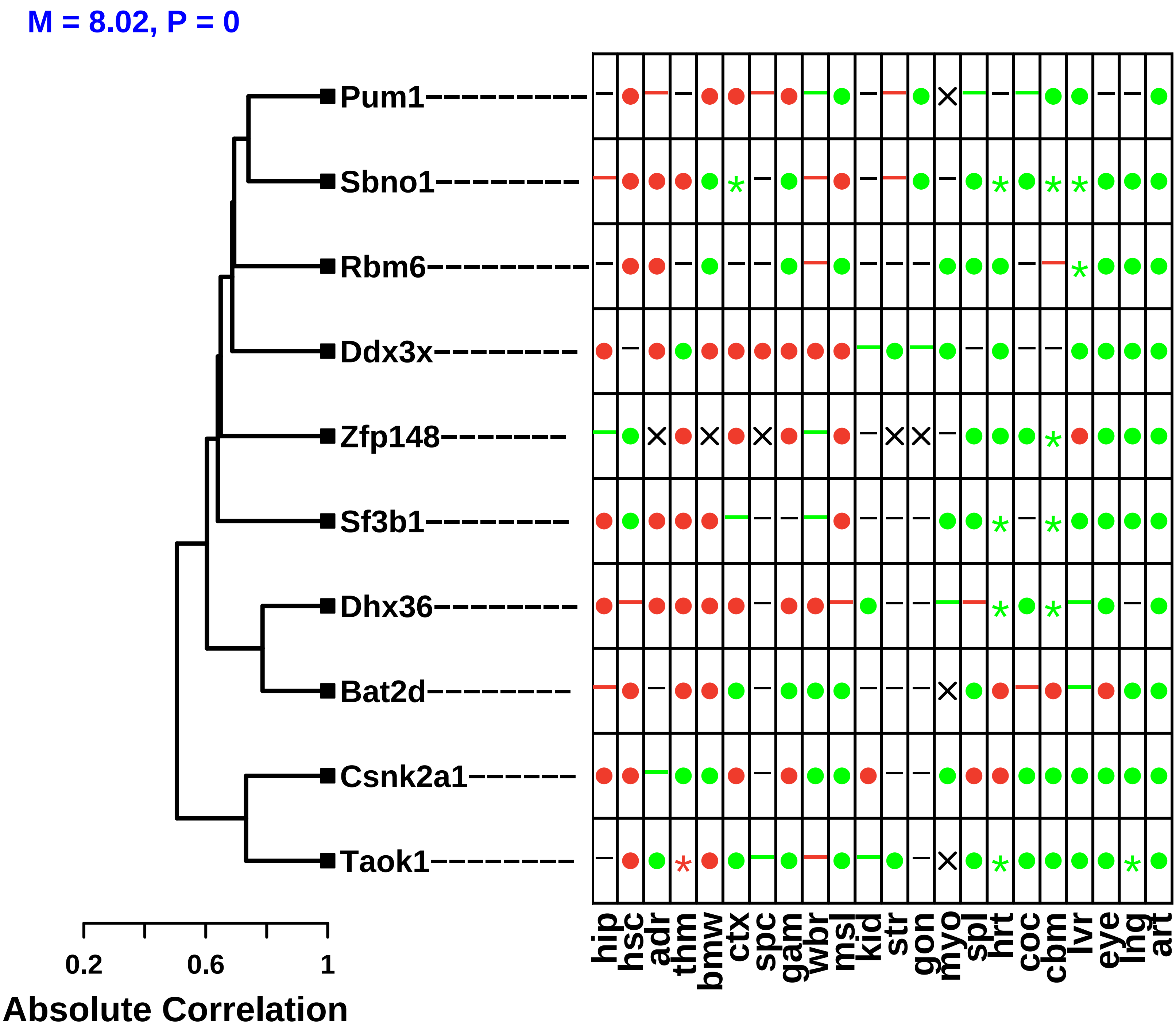

Age−Regulated Modules (10 Genes)

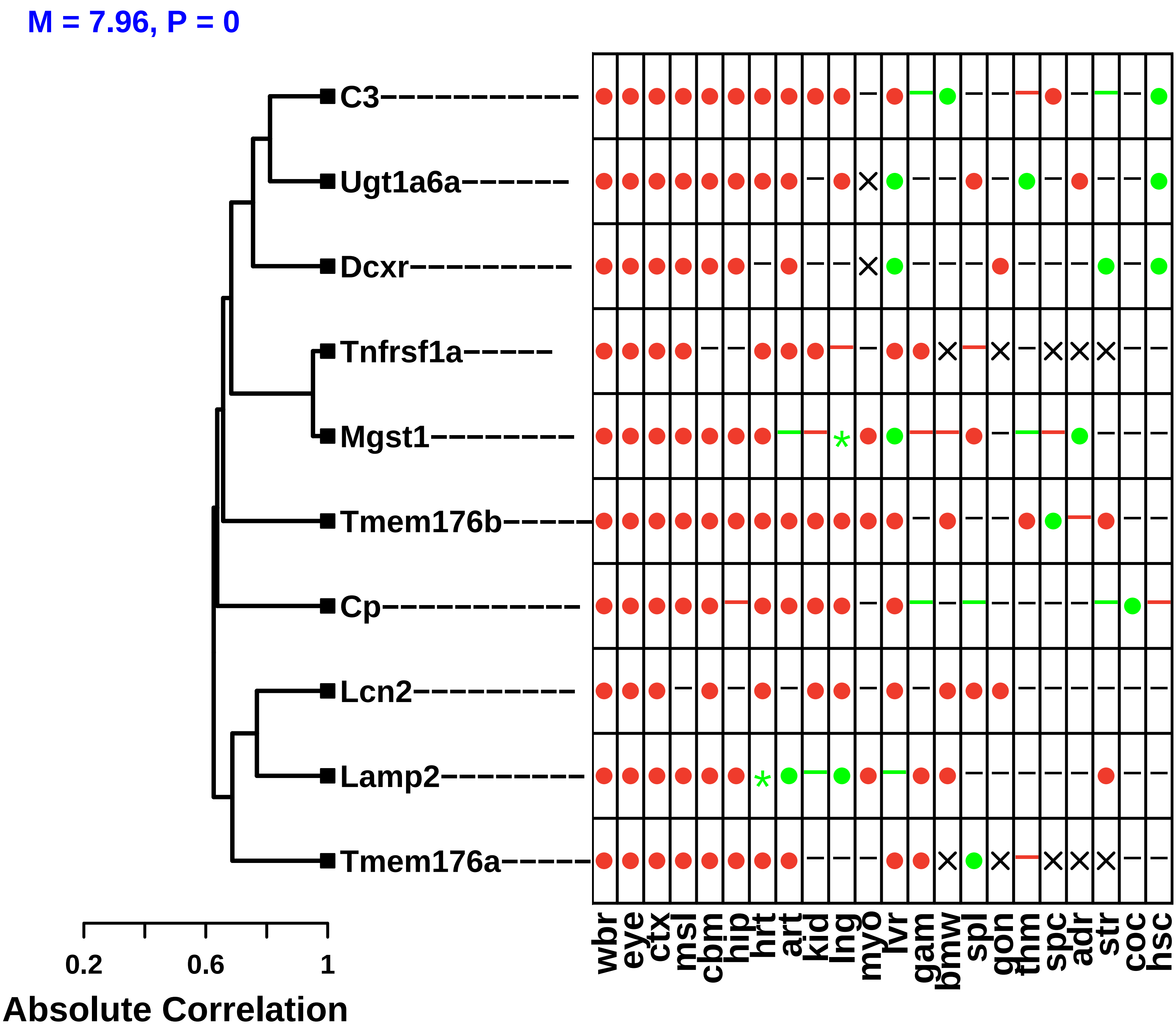

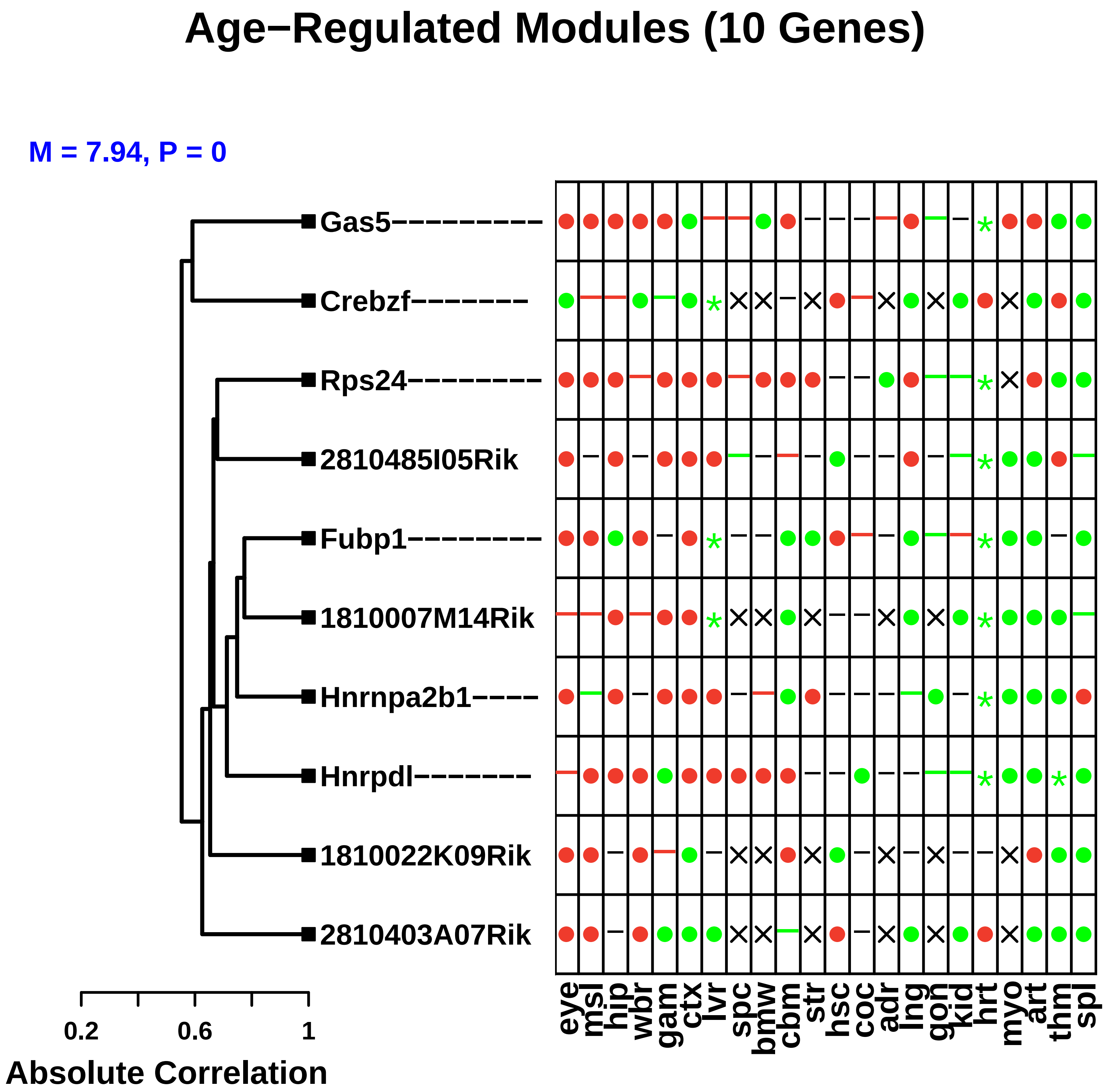

Age−Regulated Modules (10 Genes)
M = 7.94, P = 0
Gas5
Crebzf
Rps24
2810485I05Rik
Fubp1
1810007M14Rik
Hnrnpa2b1
Hnrpdl
1810022K09Rik
2810403A07Rik
eye
msl
hip
wbr
gam
ctx
lvr
spc
bmw
cbm
str
hsc
coc
adr
lng
gon
kid
hrt
myo
art
thm
spl
0.2
0.6
1
Absolute Correlation

Age−Regulated Modules (10 Genes)

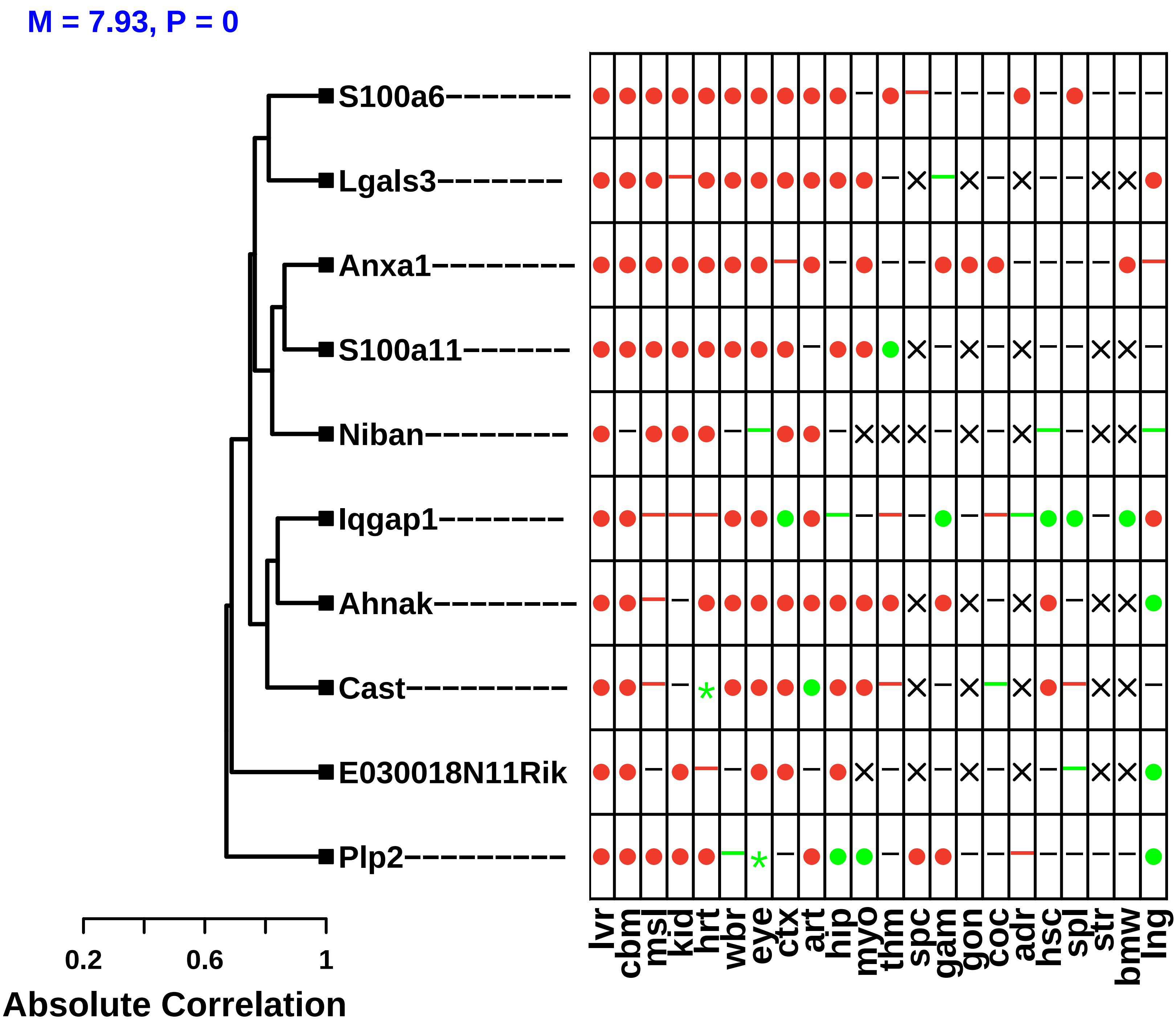

Age−Regulated Modules (10 Genes)

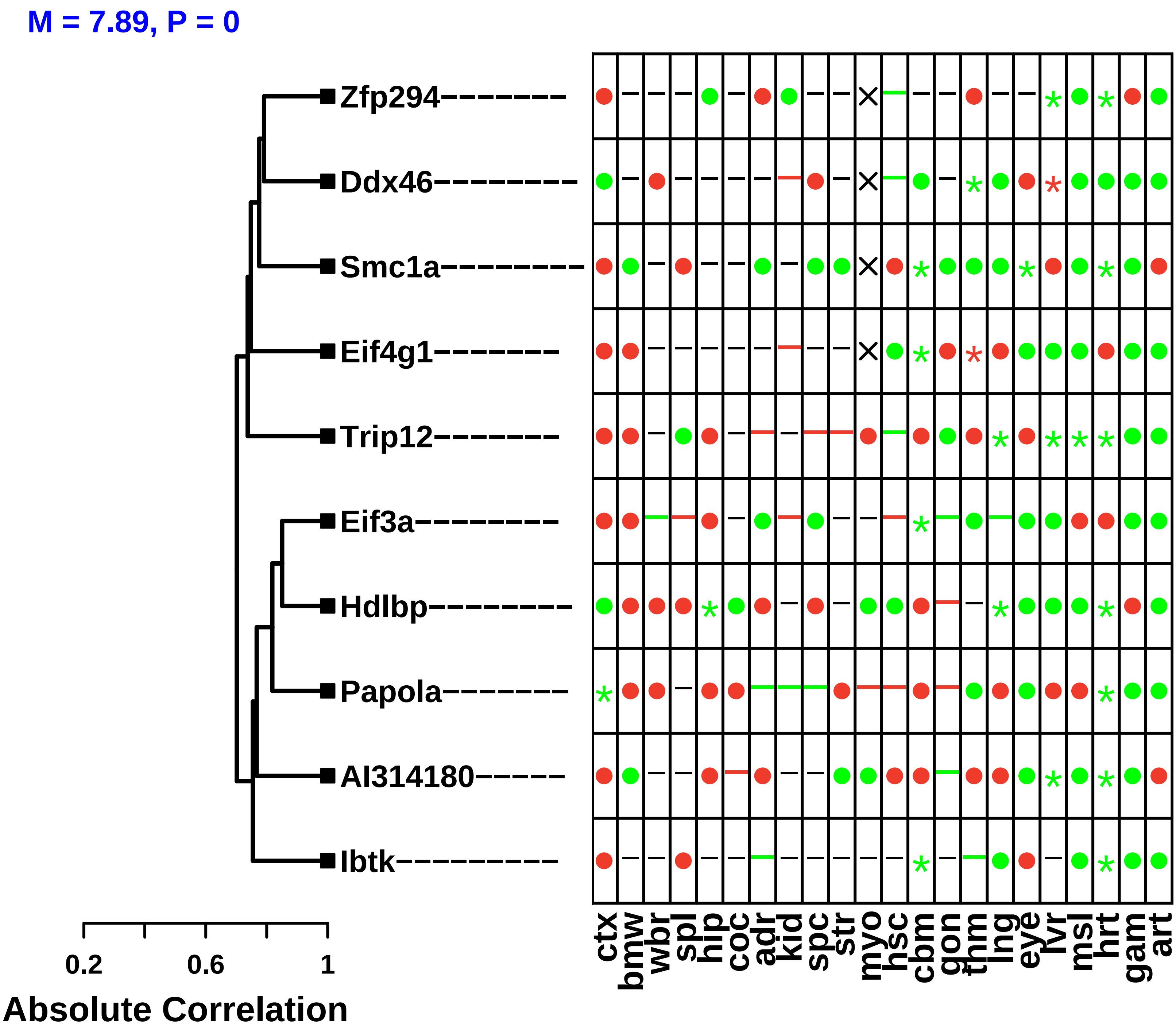

Age−Regulated Modules (10 Genes)

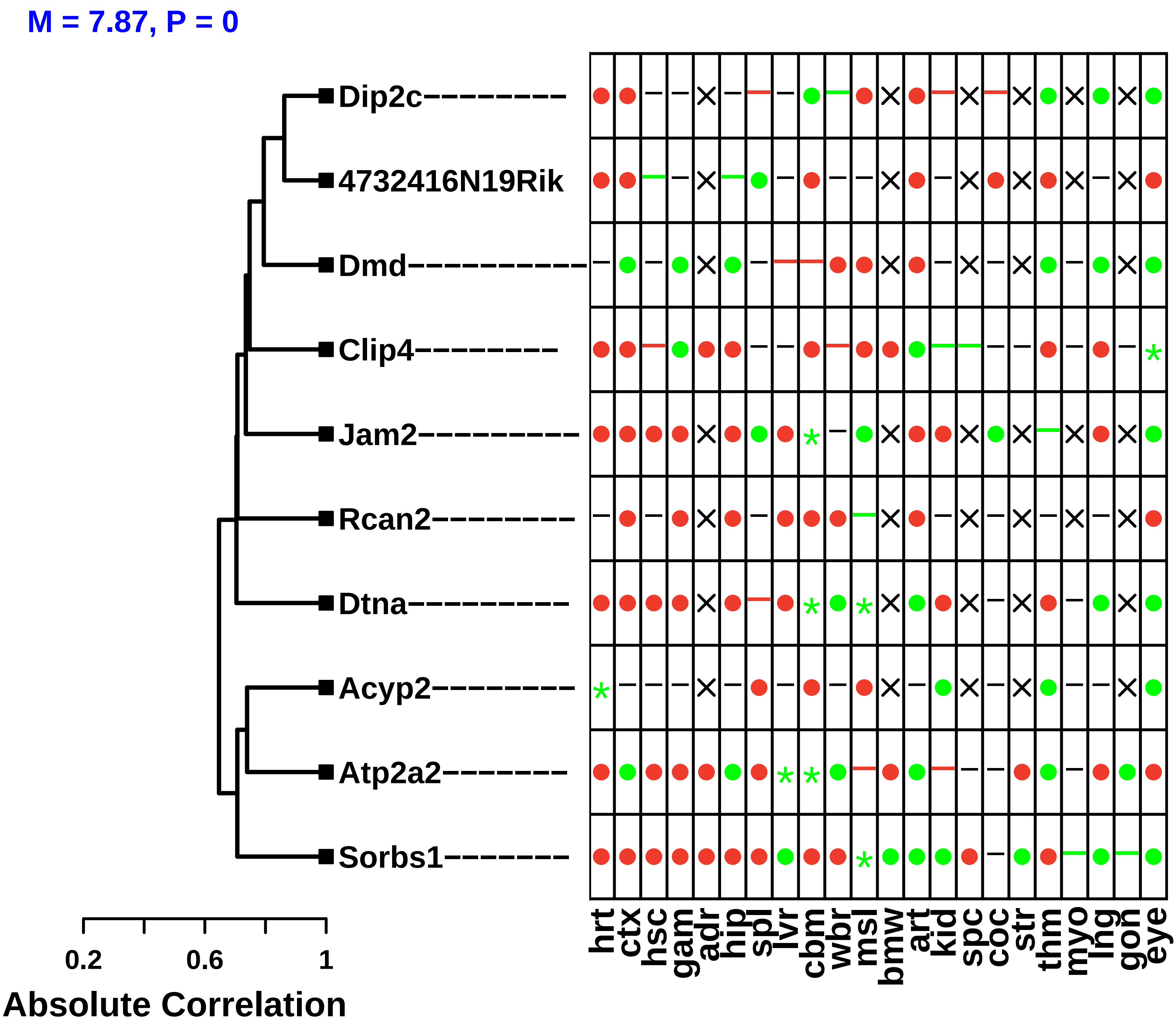

Age−Regulated Modules (10 Genes)

M = 7.86, P = 0

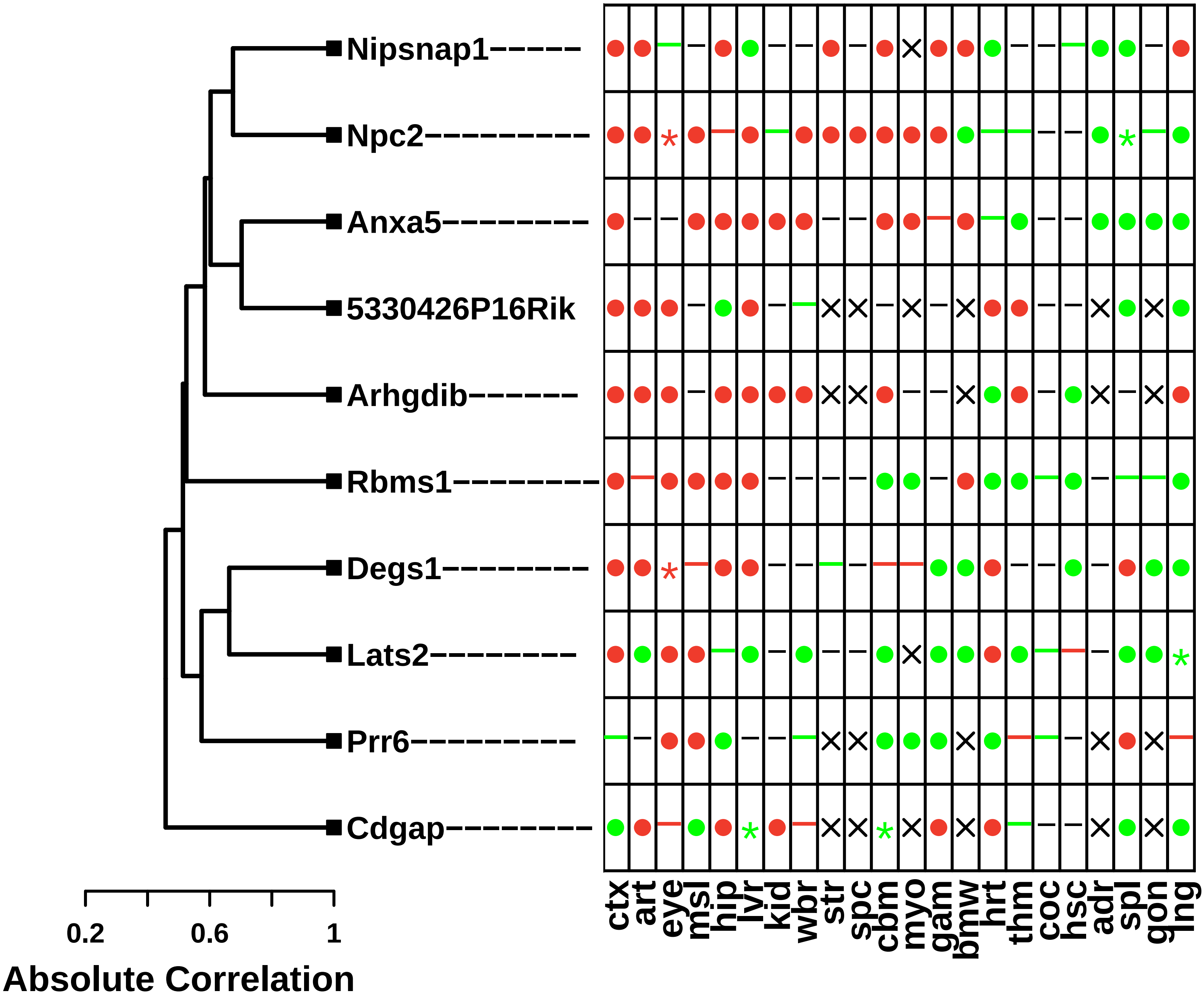

Age−Regulated Modules (10 Genes)

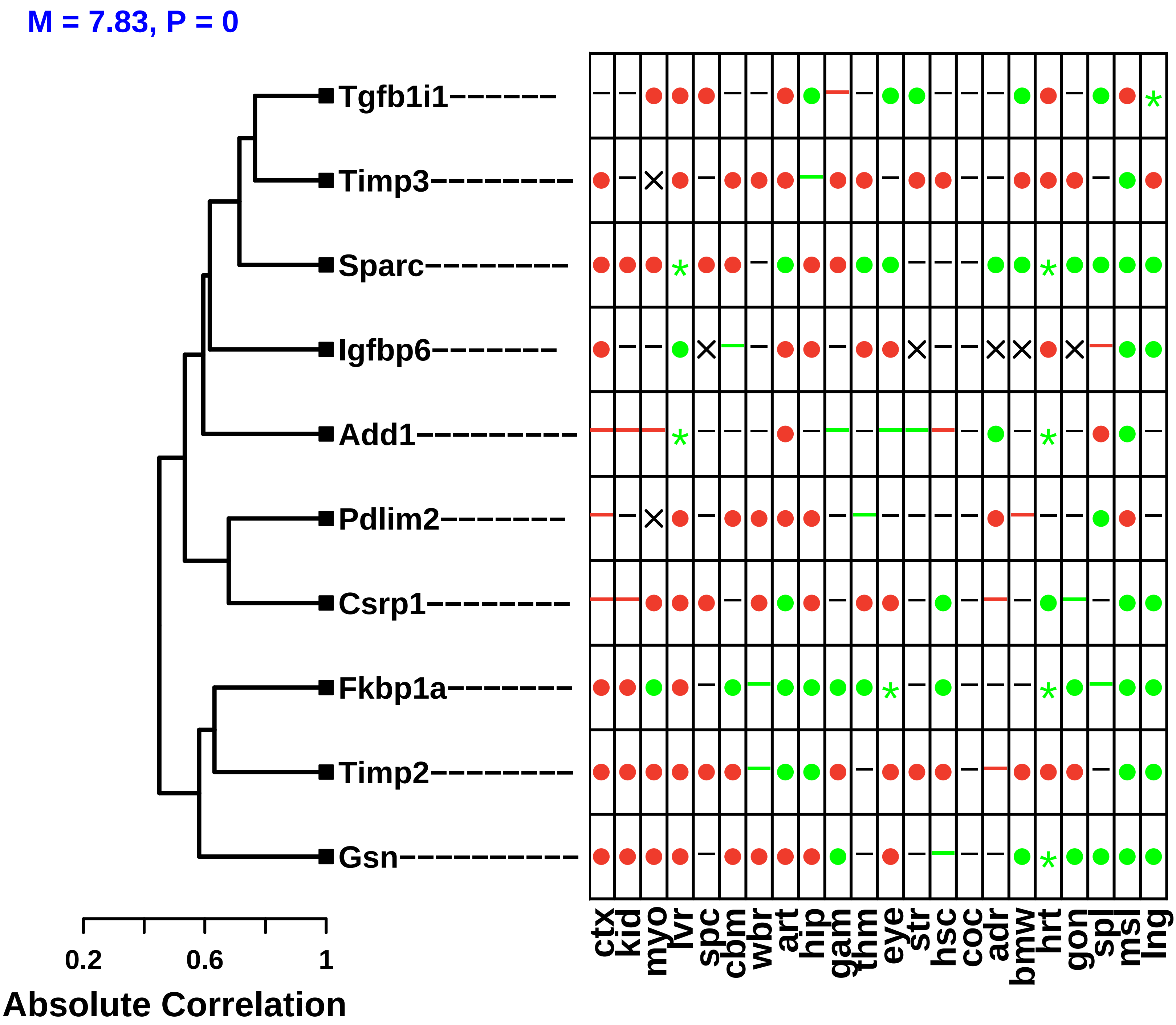

Age−Regulated Modules (10 Genes)

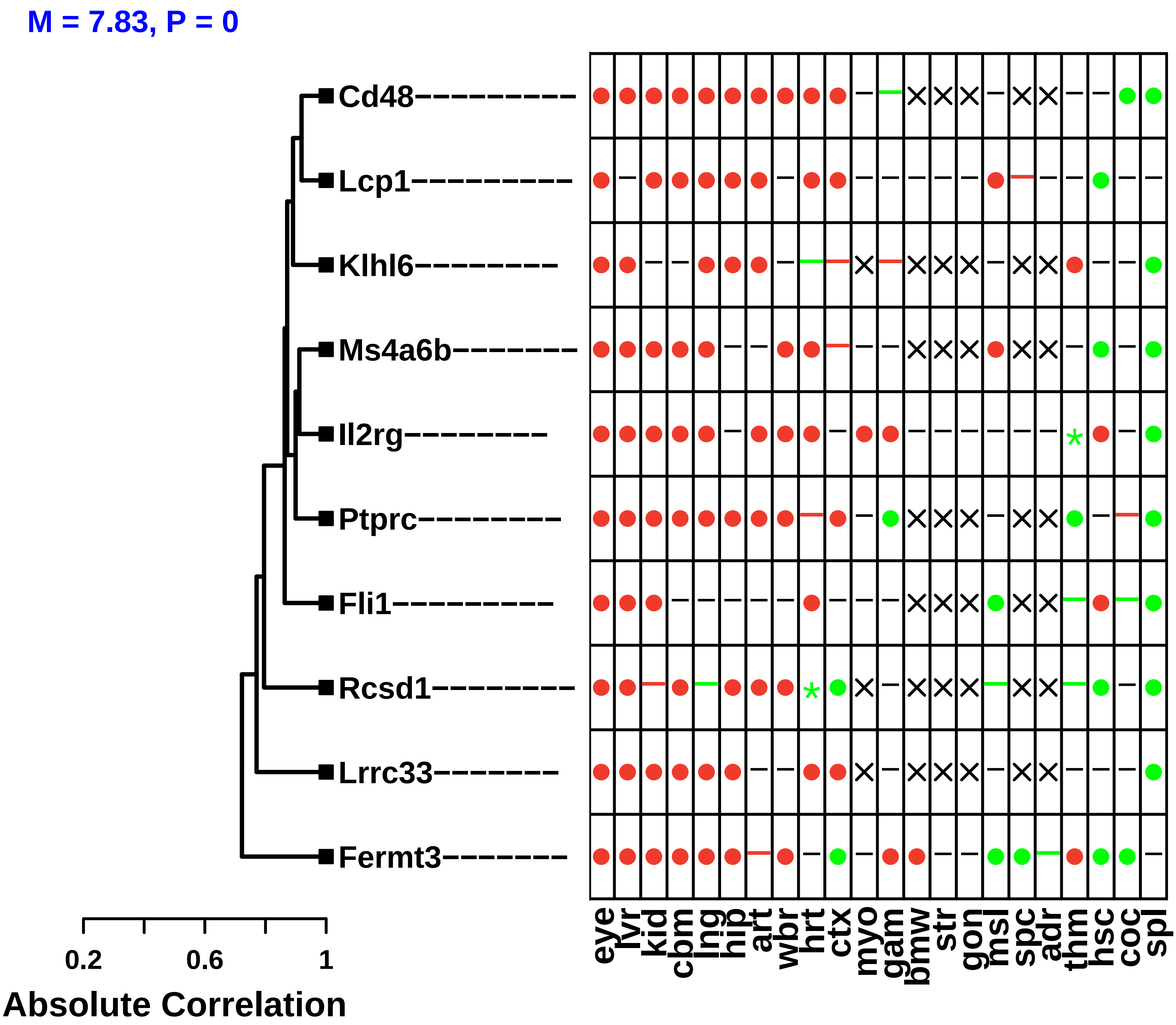

Age−Regulated Modules (10 Genes)

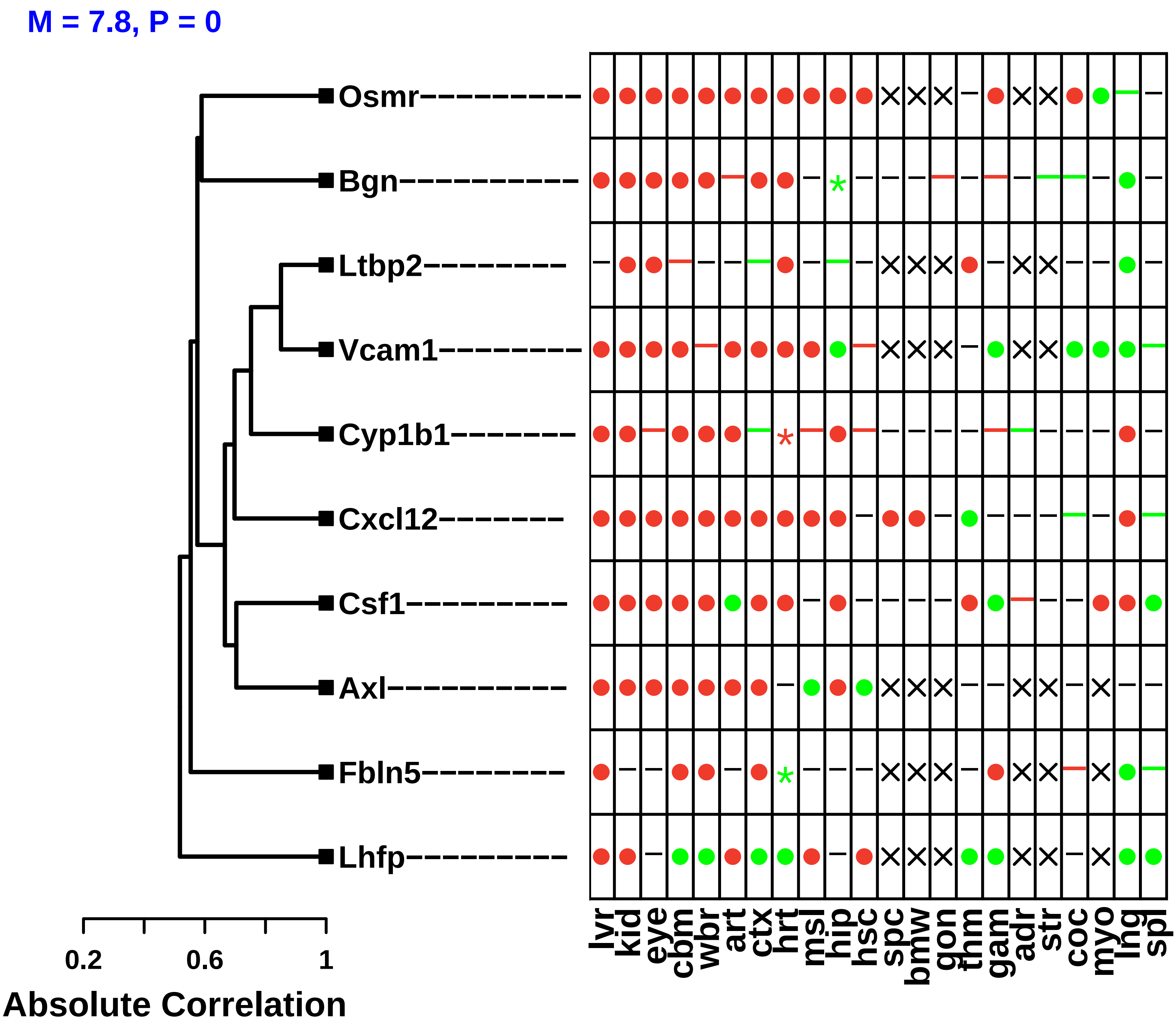

Age−Regulated Modules (10 Genes)

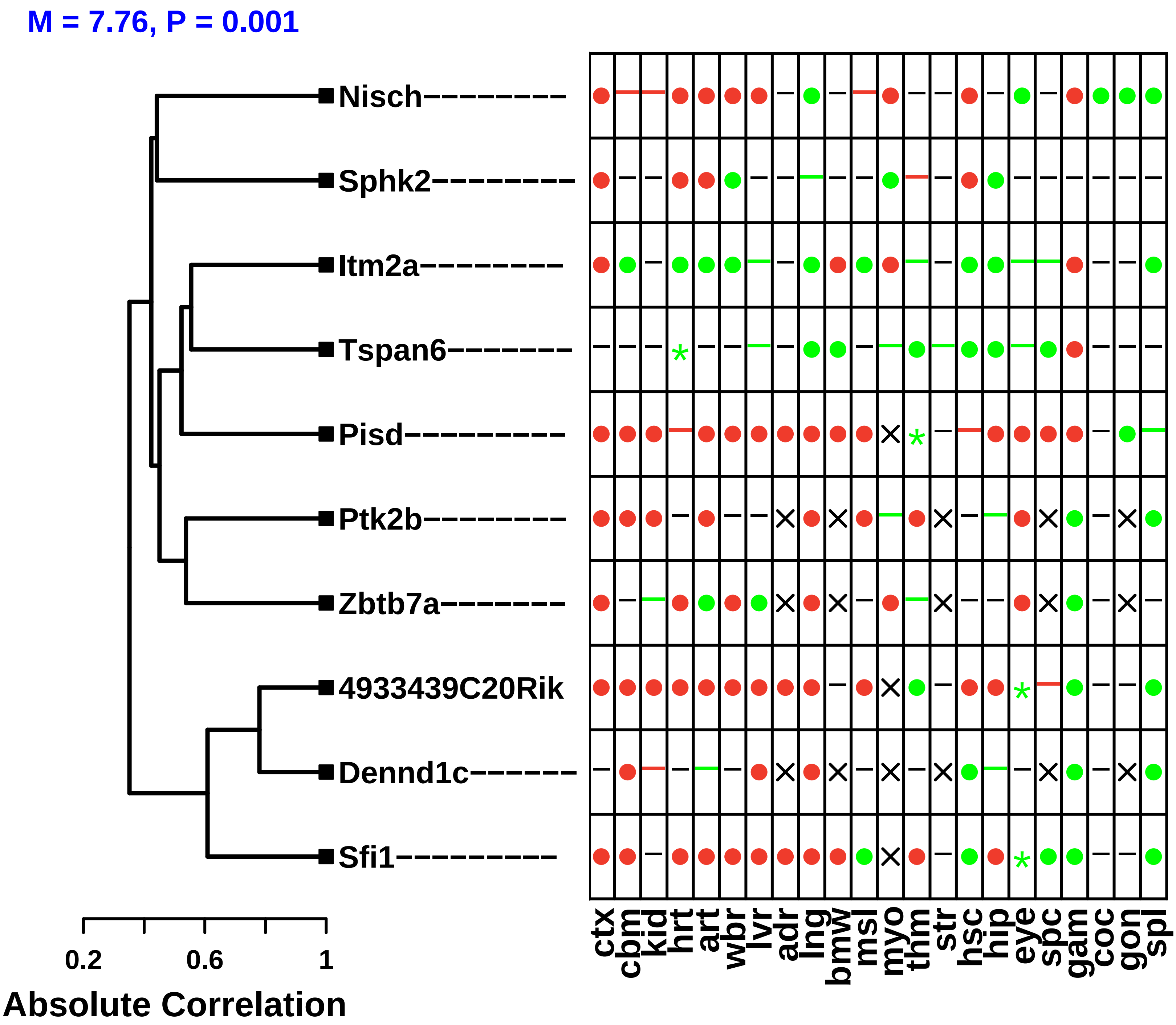

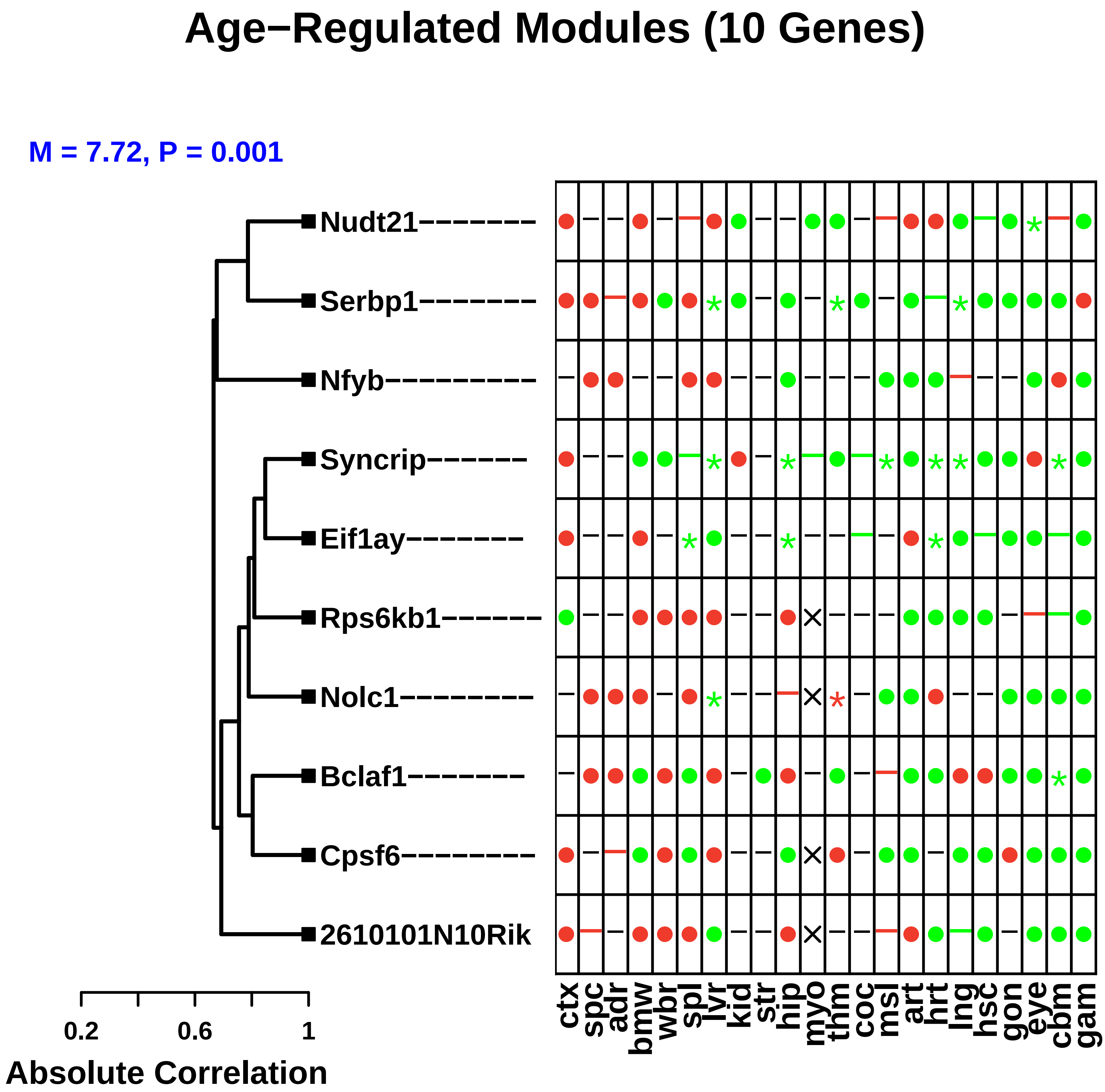

Age−Regulated Modules (10 Genes)
M = 7.72, P = 0.001
Nudt21
Serbp1
Nfyb
Syncrip
Eif1ay
Rps6kb1
Nolc1
Bclaf1
Cpsf6
2610101N10Rik
0.2
0.6
1
Absolute Correlation
ctx
spc
adr
bmw
wbr
spl
lvr
kid
str
hip
myo
thm
coc
msl
art
hrt
lng
hsc
gon
eye
cbm
gam

Age−Regulated Modules (10 Genes)

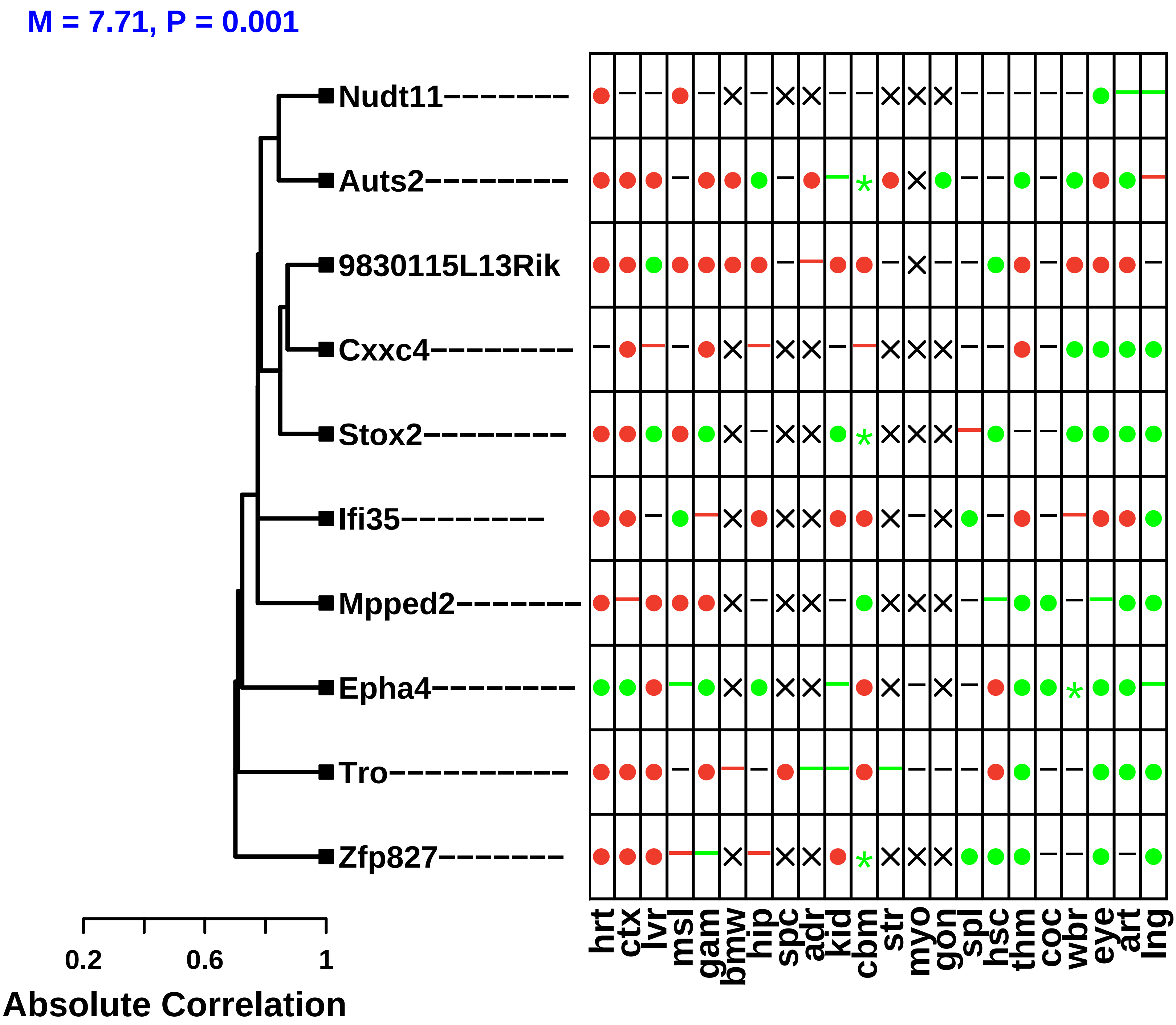

Age−Regulated Modules (10 Genes)

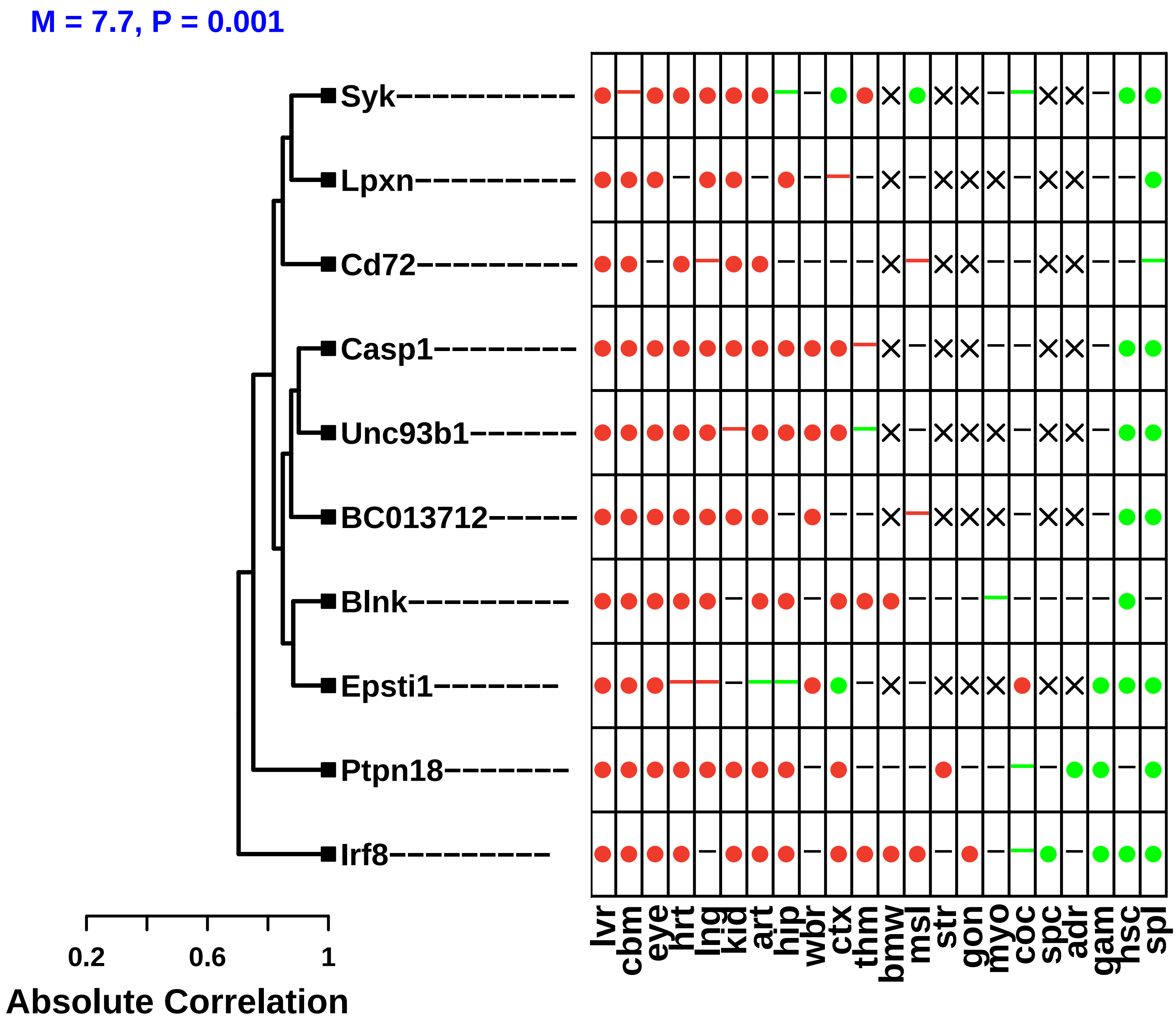

Age−Regulated Modules (10 Genes)

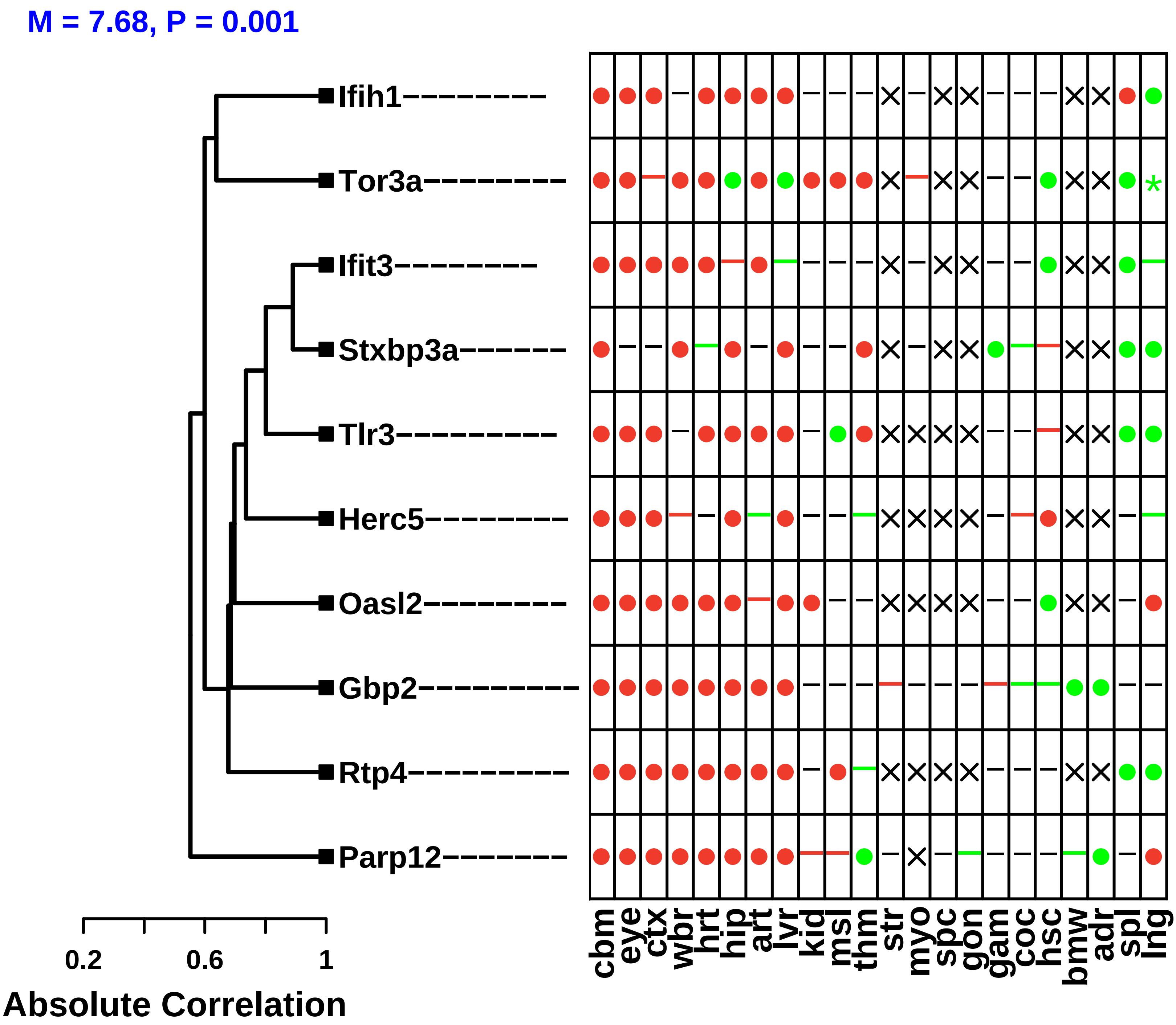

Age−Regulated Modules (10 Genes)

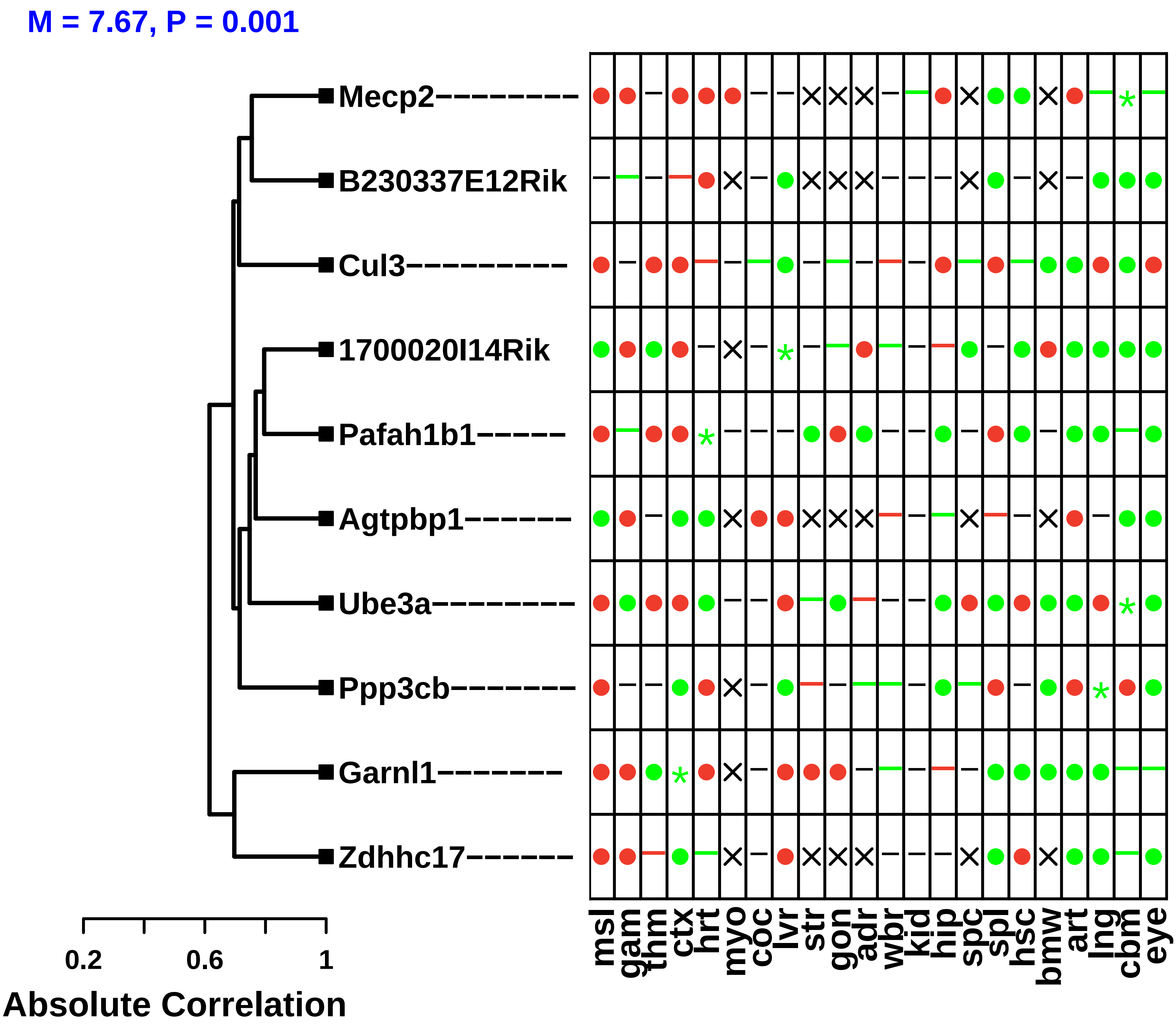

Age−Regulated Modules (10 Genes)

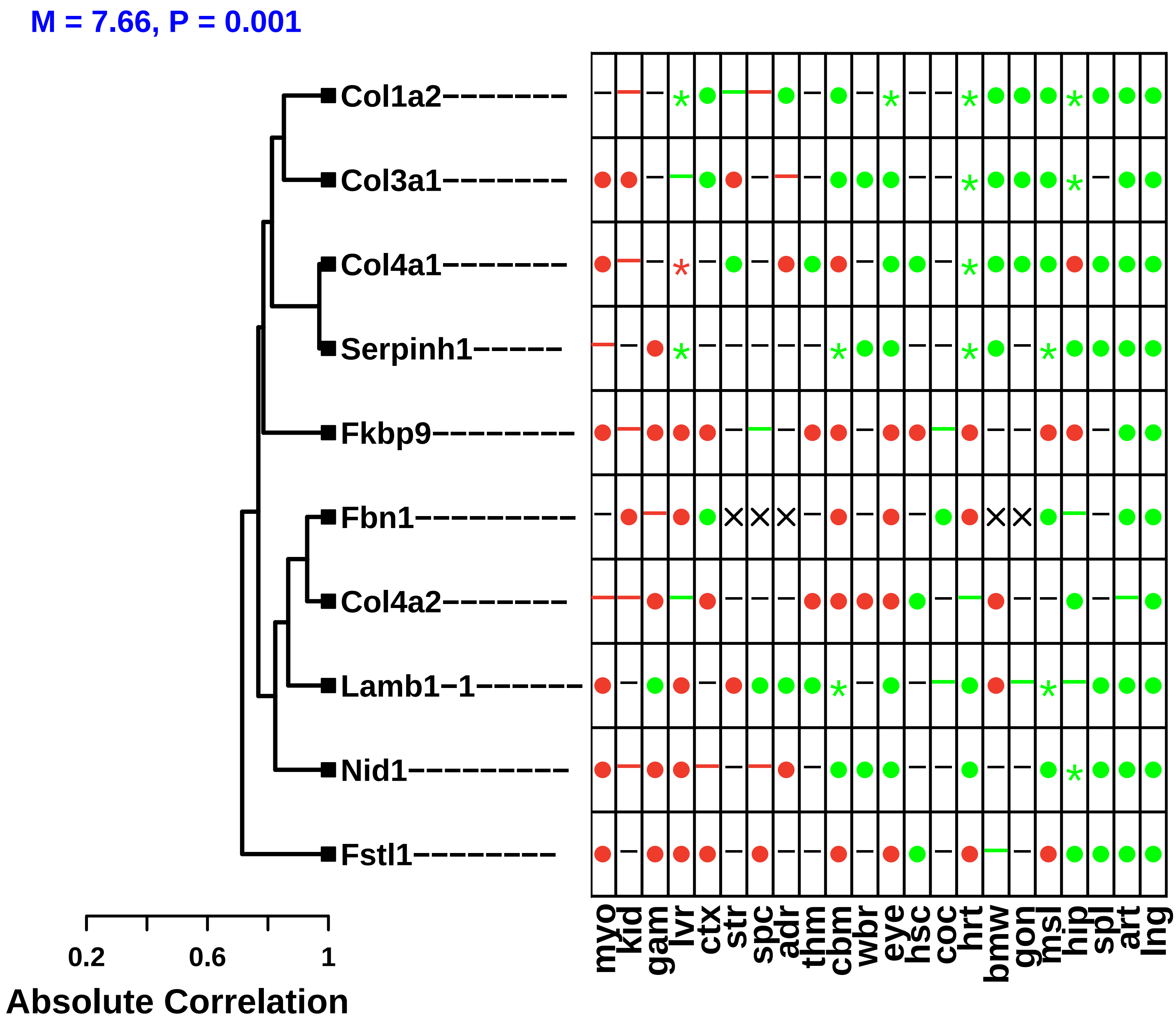

Age−Regulated Modules (10 Genes)

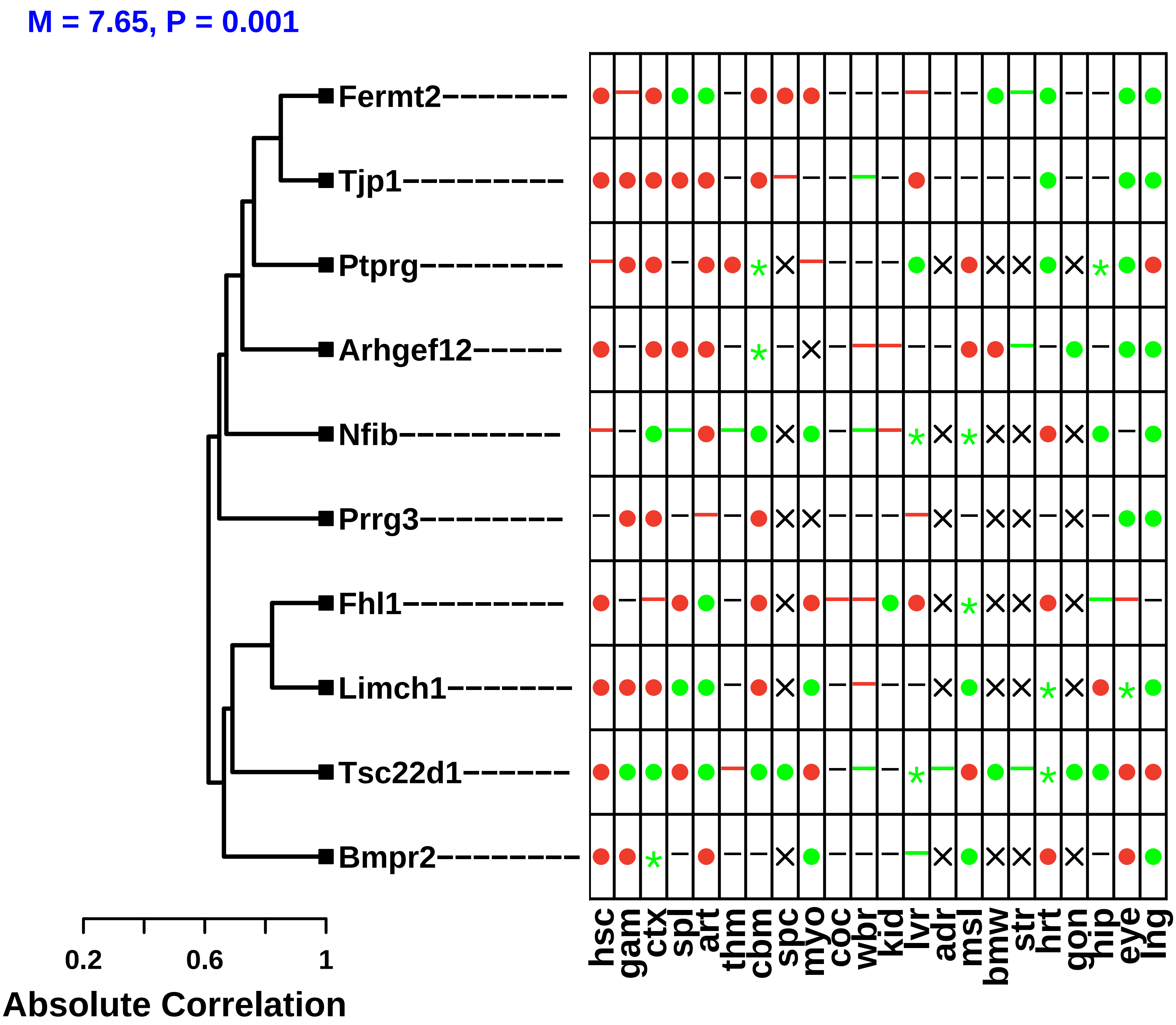

Age−Regulated Modules (10 Genes)

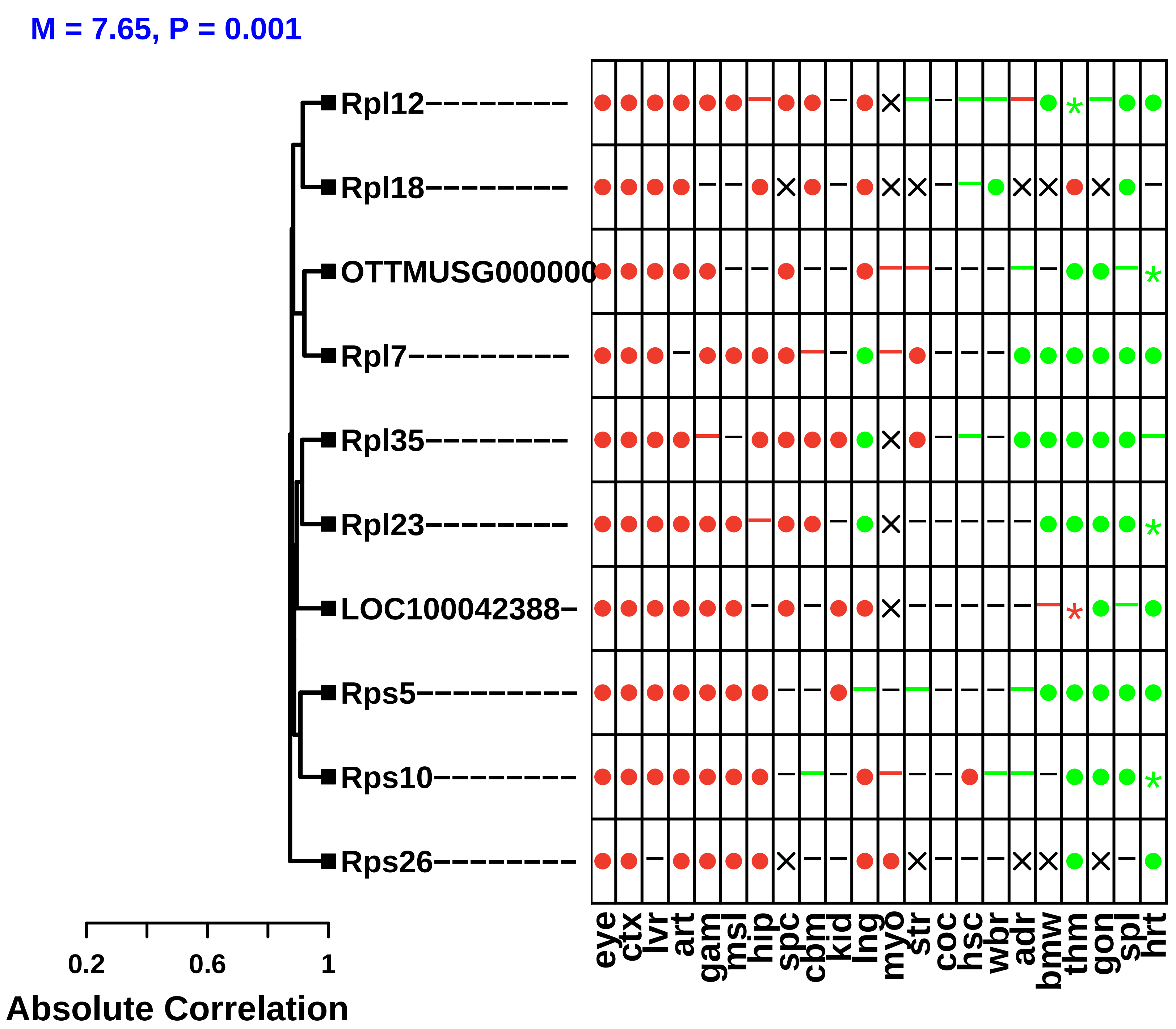

Age−Regulated Modules (10 Genes)

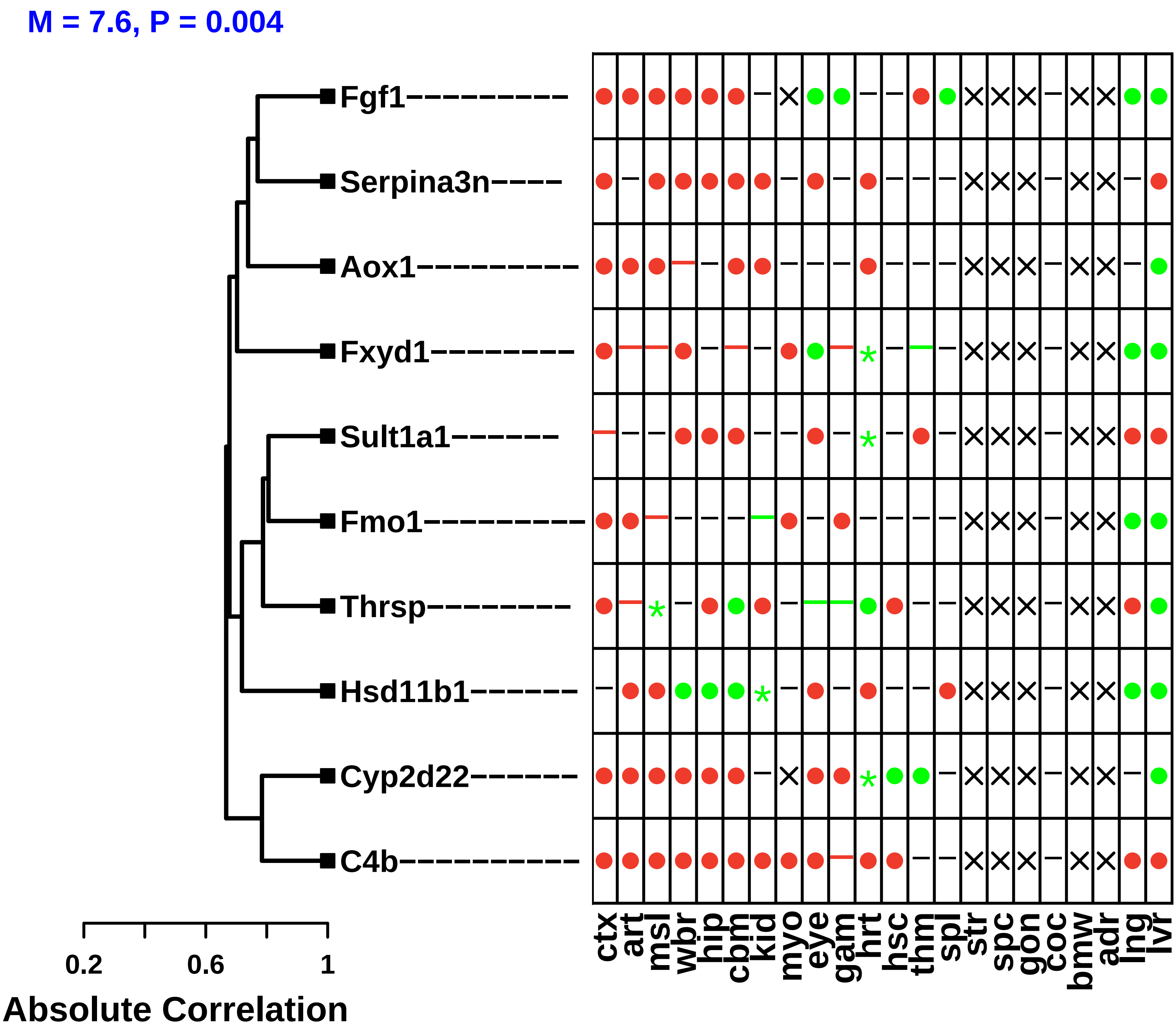

Age−Regulated Modules (10 Genes)

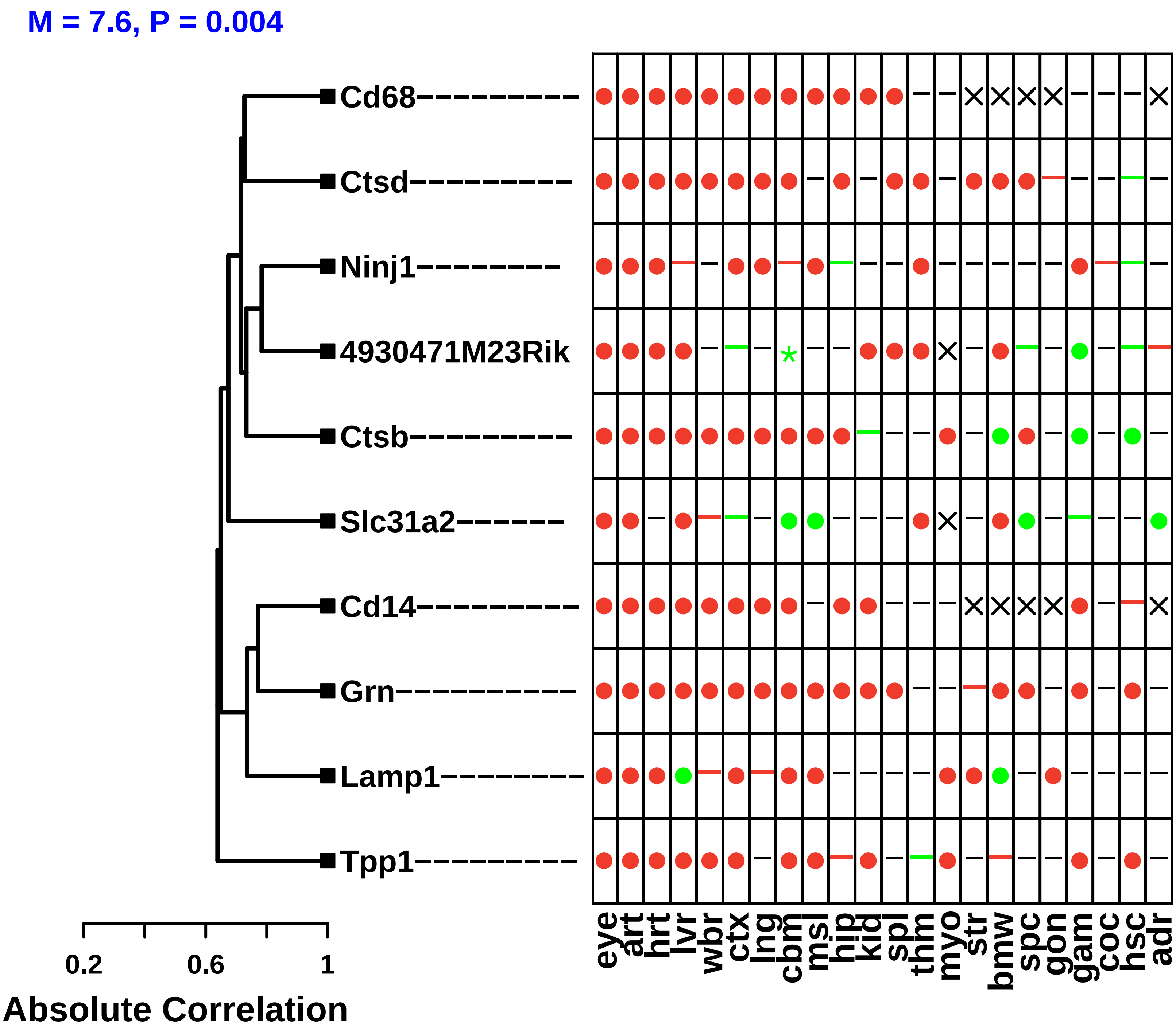

Age−Regulated Modules (10 Genes)

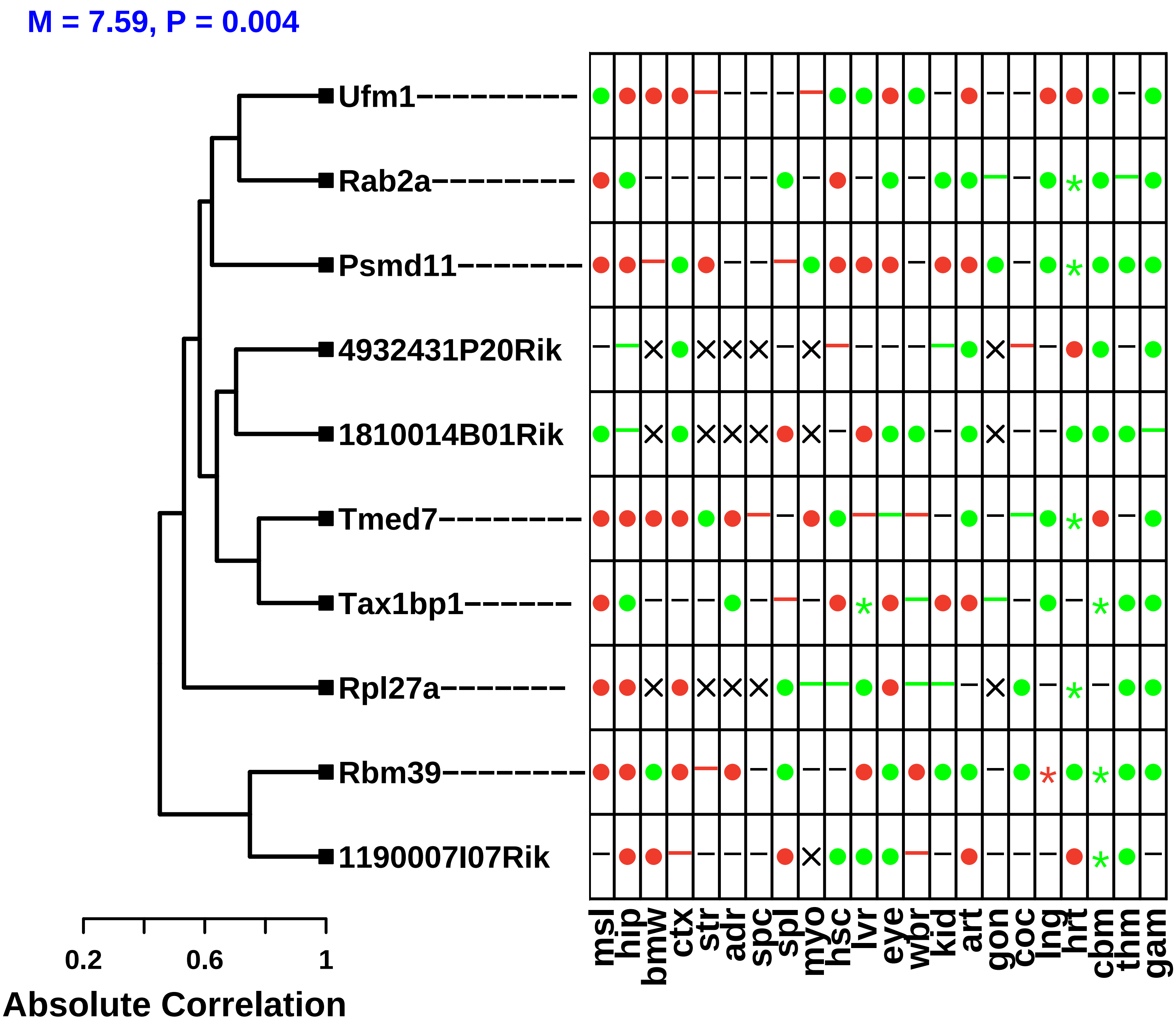

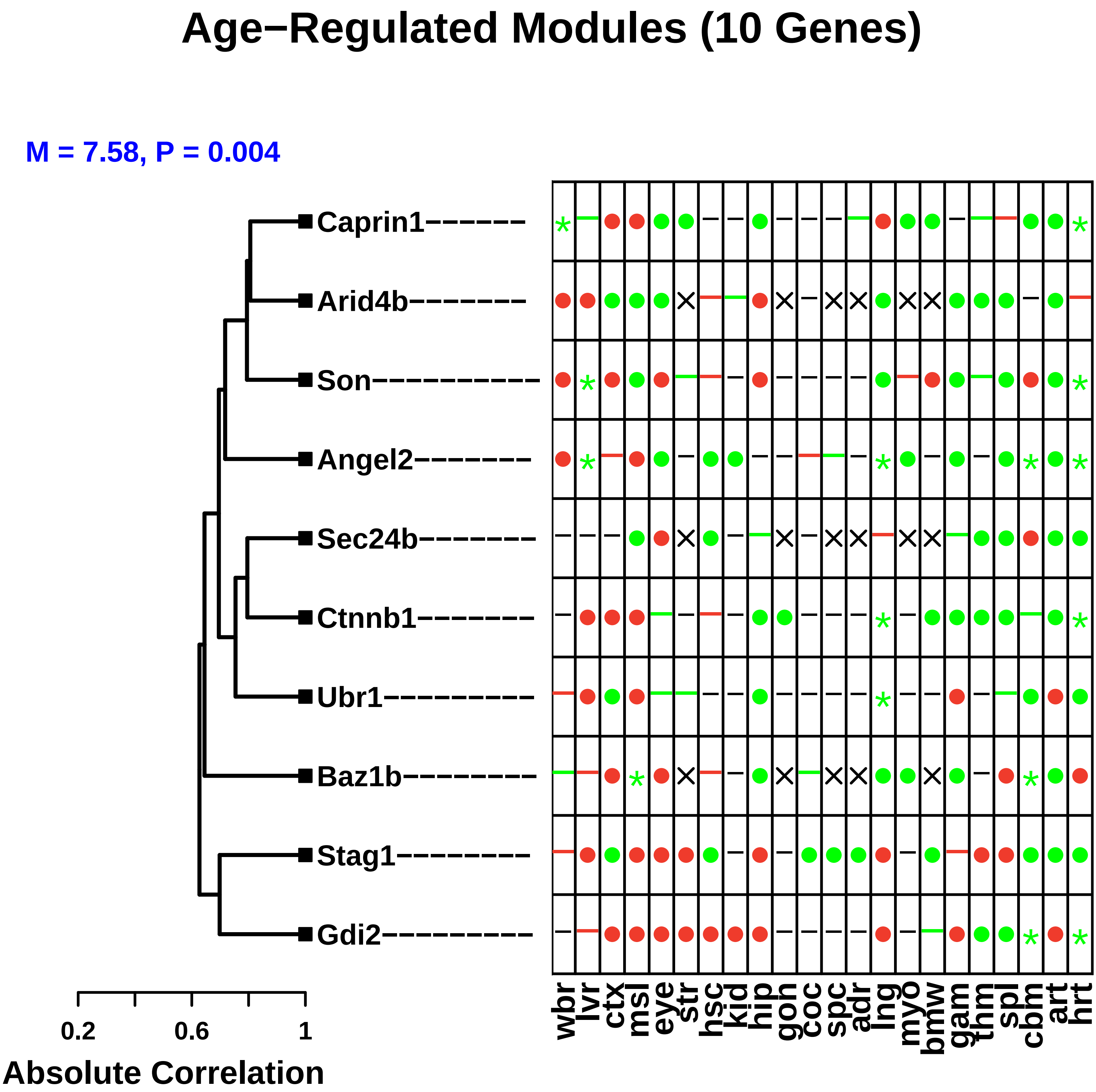
Age−Regulated Modules (10 Genes)
M = 7.58, P = 0.004
Caprin1
Arid4b
Son
Angel2
Sec24b
Ctnnb1
Ubr1
Baz1b
Stag1
Gdi2
0.2
0.6
1
Absolute Correlation
wbr
lvr
ctx
msl
eye
str
hsc
kid
hip
gon
coc
spc
adr
lng
myo
bmw
gam
thm
spl
cbm
art
hrt

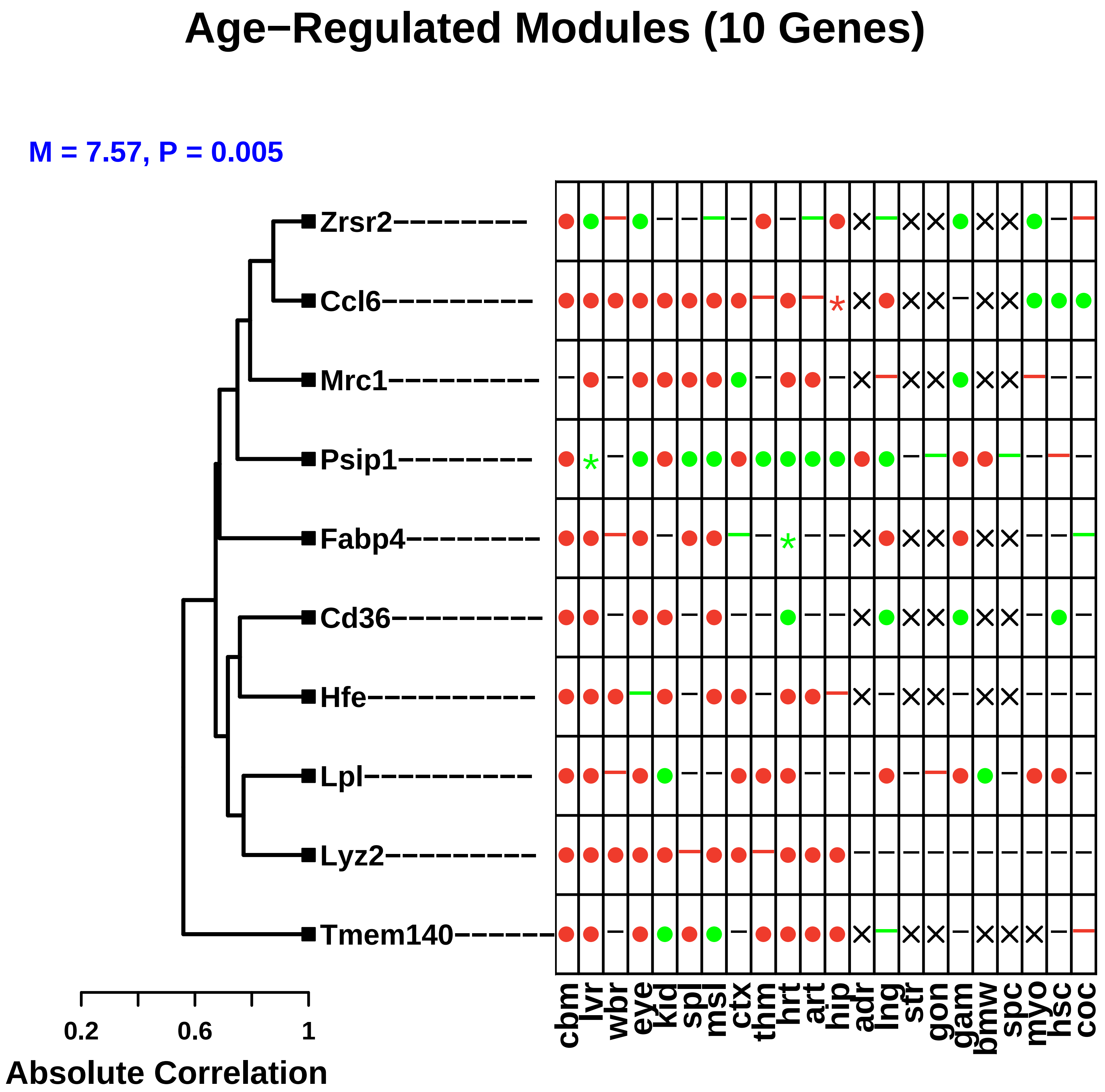
Age−Regulated Modules (10 Genes)
M = 7.57, P = 0.005
Zrsr2
Ccl6
Mrc1
Psip1
Fabp4
Cd36
Hfe
Lpl
Lyz2
Tmem140
0.2
0.6
1
Absolute Correlation
cbm
lvr
wbr
eye
kid
spl
msl
ctx
thm
hrt
art
hip
adr
lng
str
gon
gam
bmw
spc
myo
hsc
coc

Age−Regulated Modules (10 Genes)

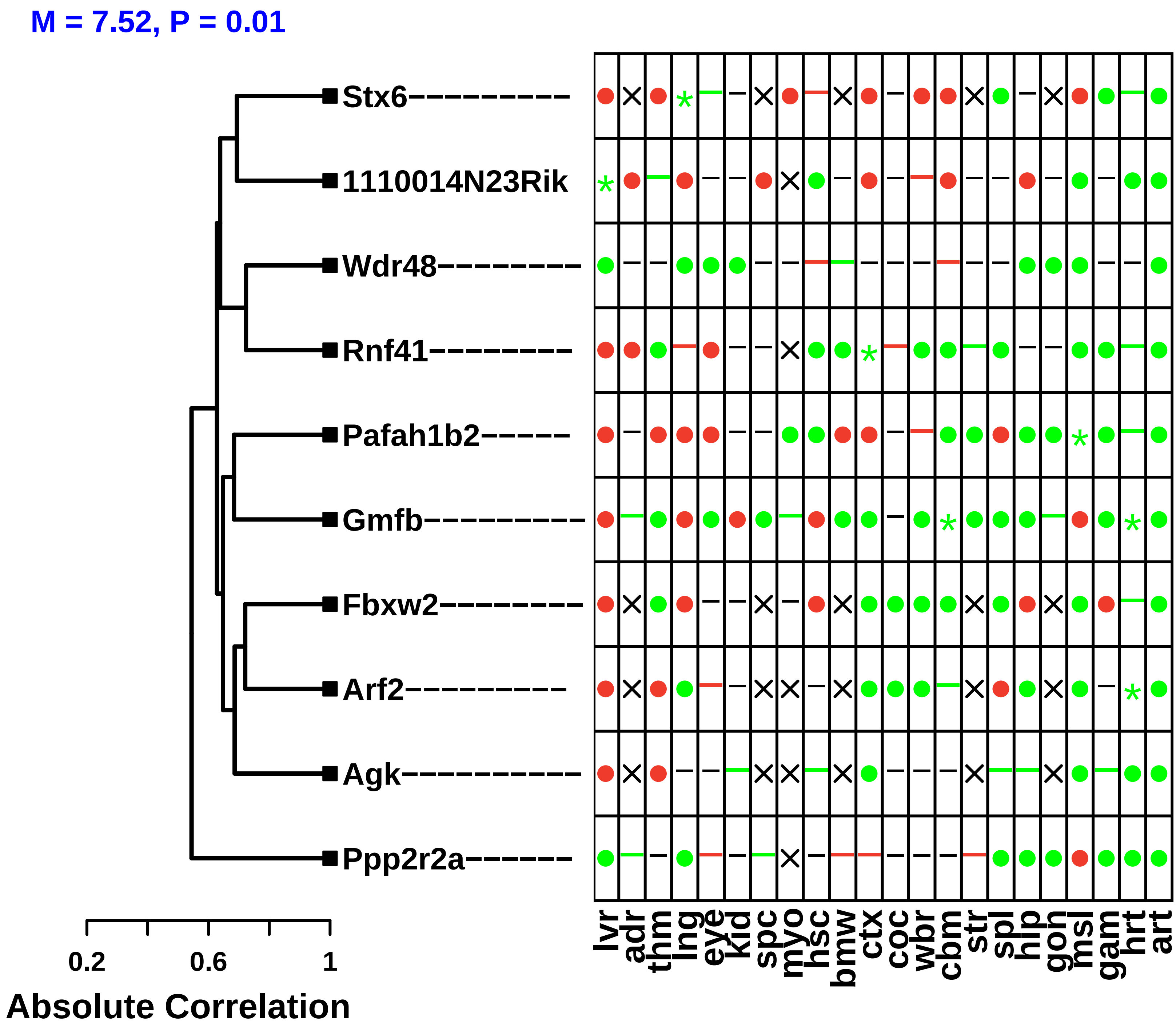

Age−Regulated Modules (10 Genes)

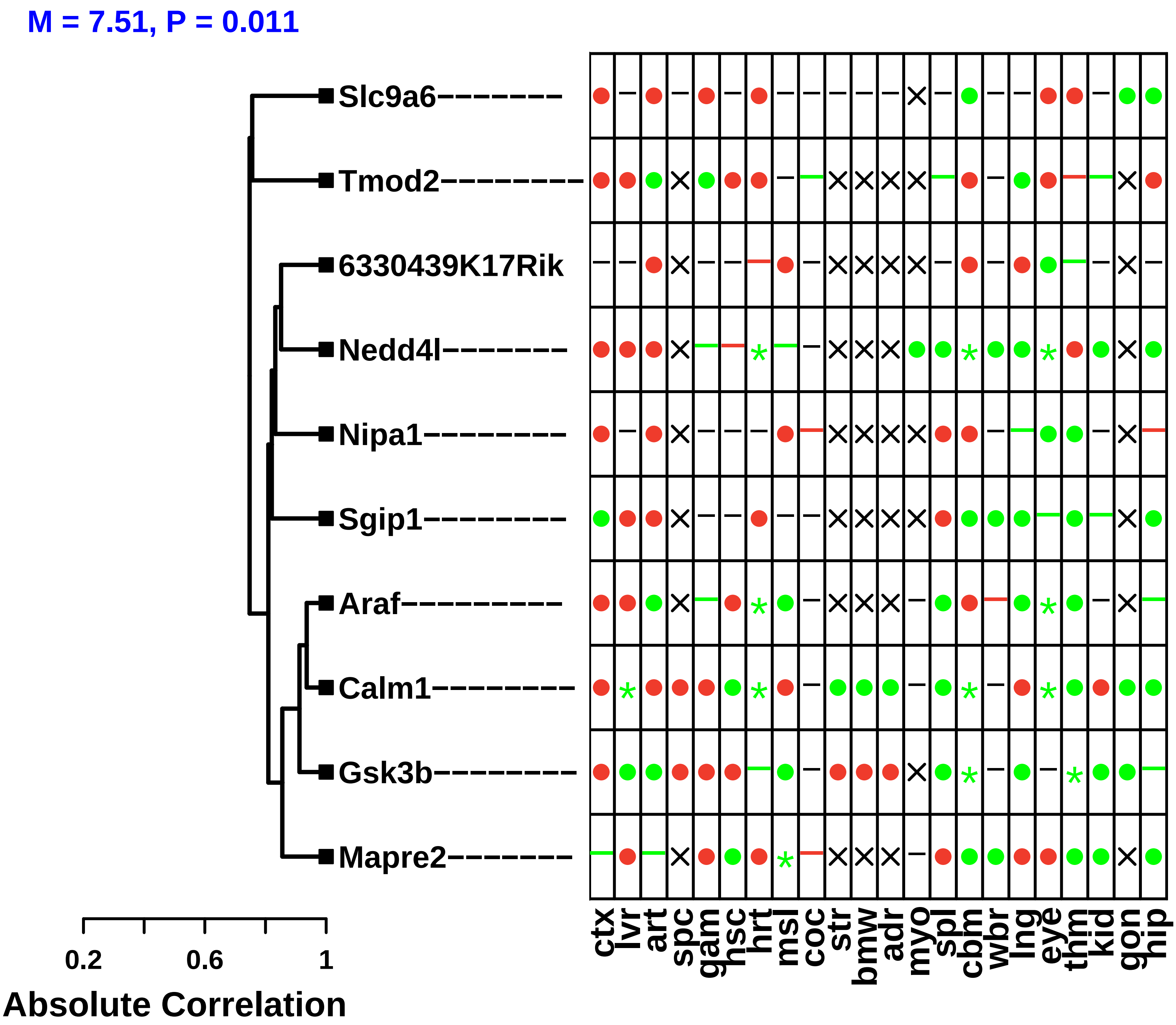

Age−Regulated Modules (10 Genes)

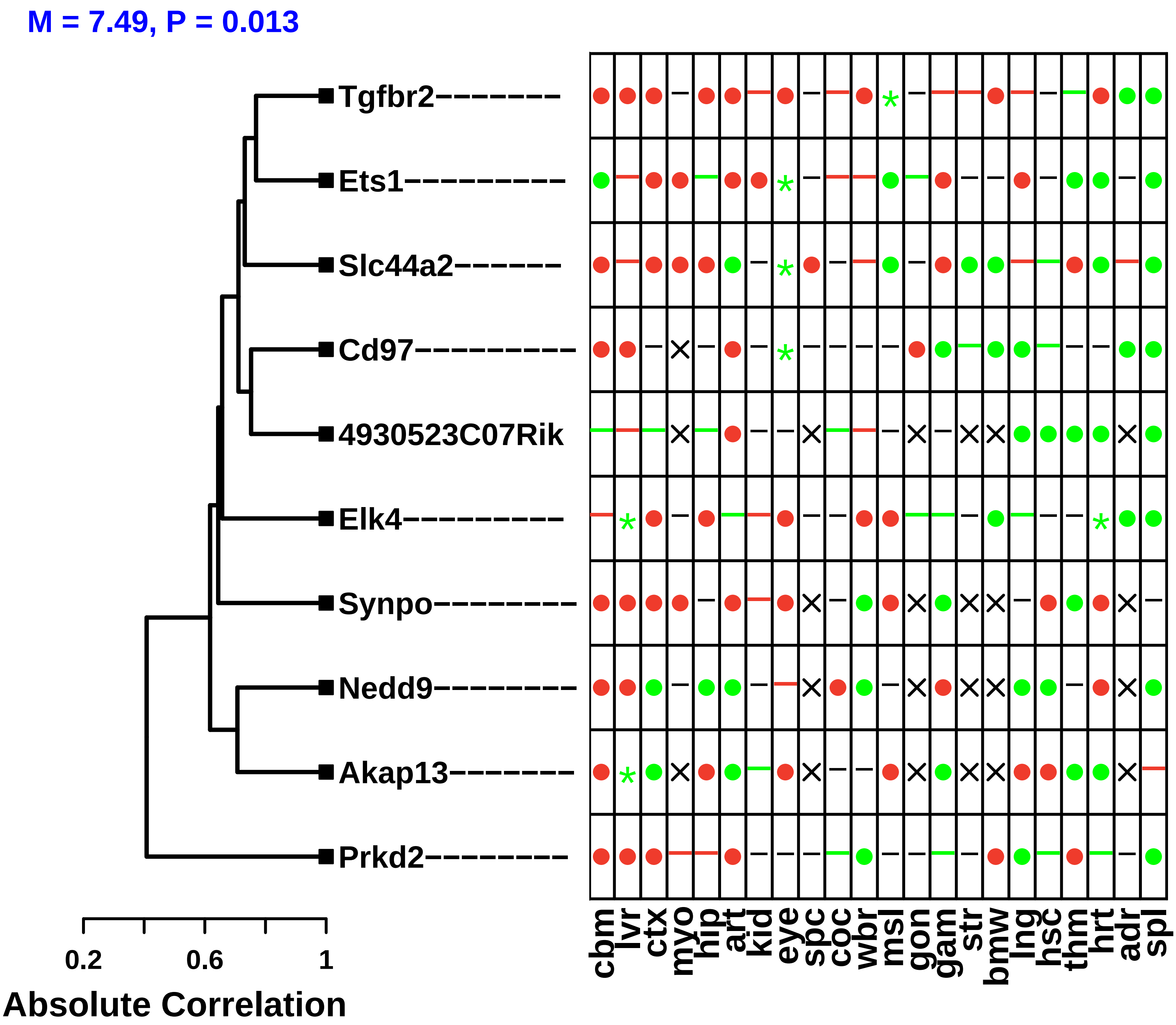

Age−Regulated Modules (10 Genes)

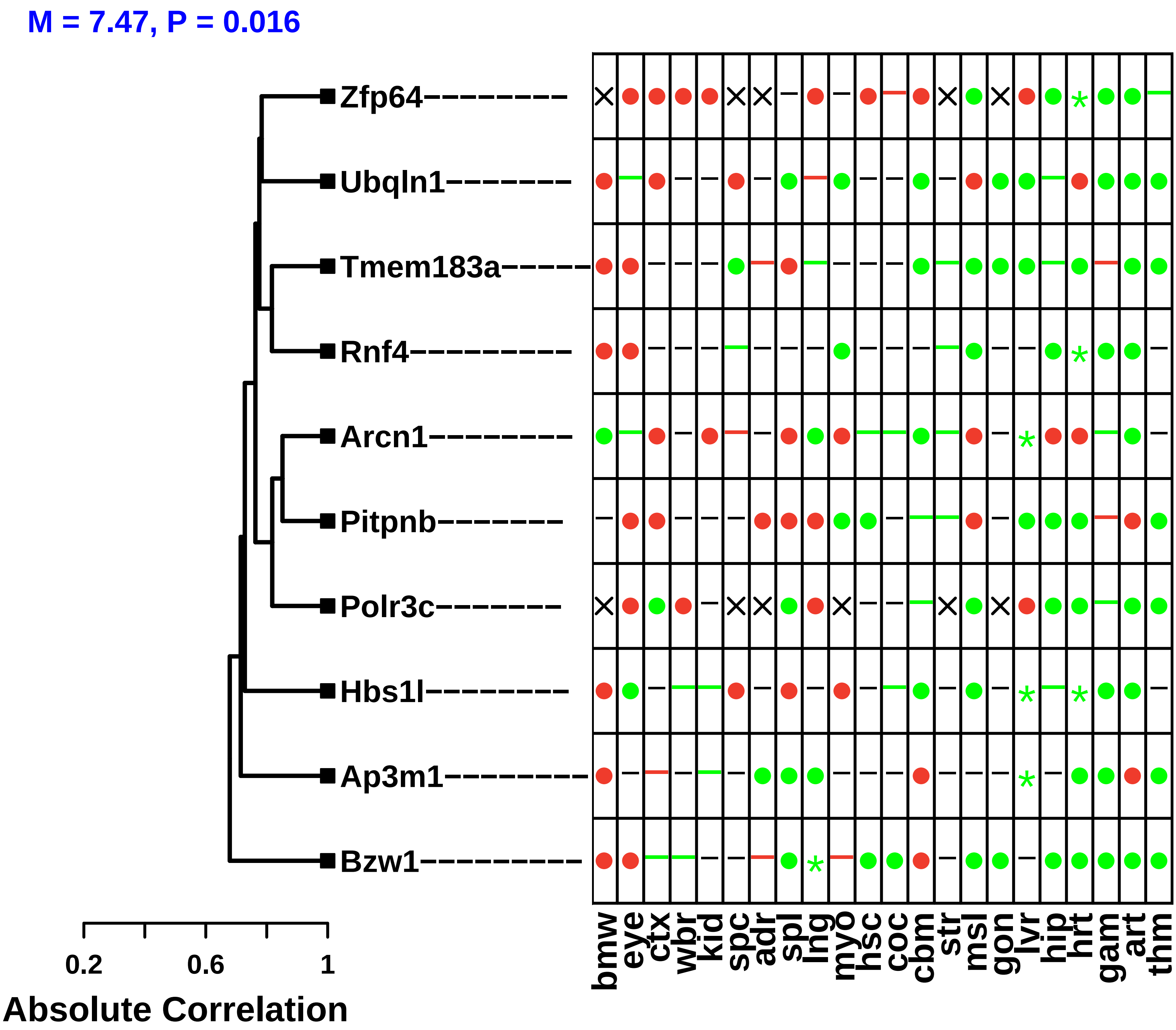

Age−Regulated Modules (10 Genes)

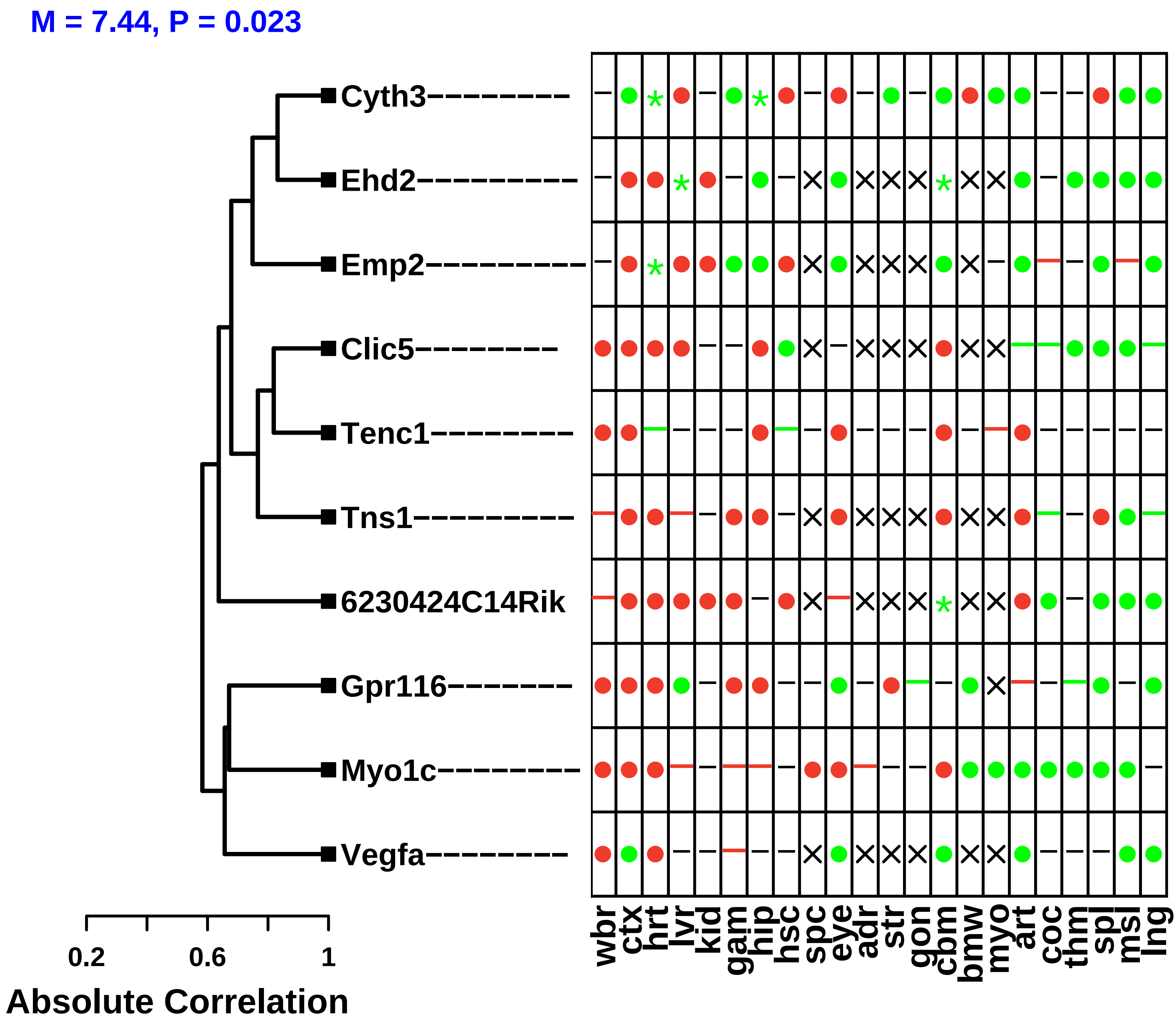

Age−Regulated Modules (10 Genes)

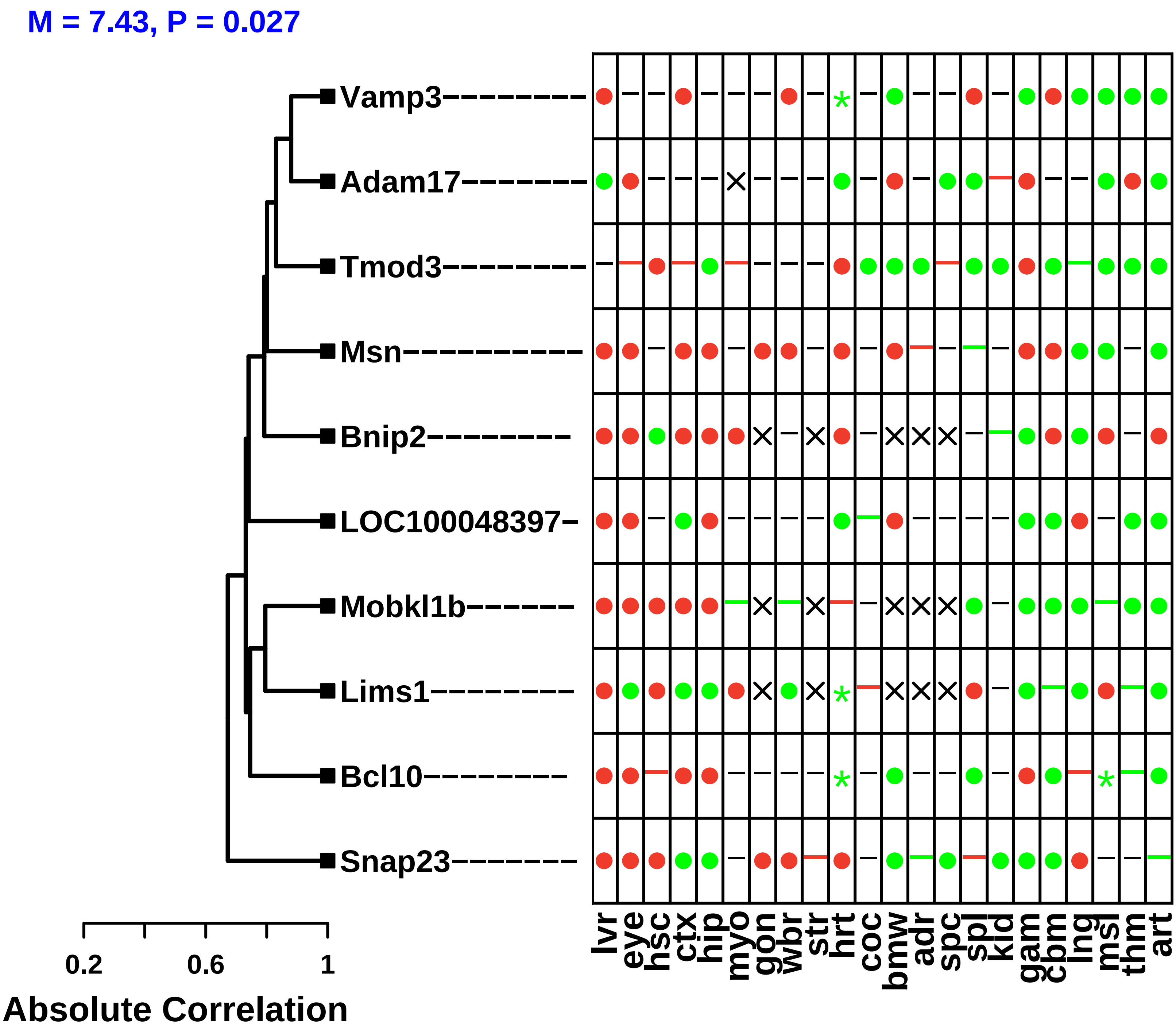

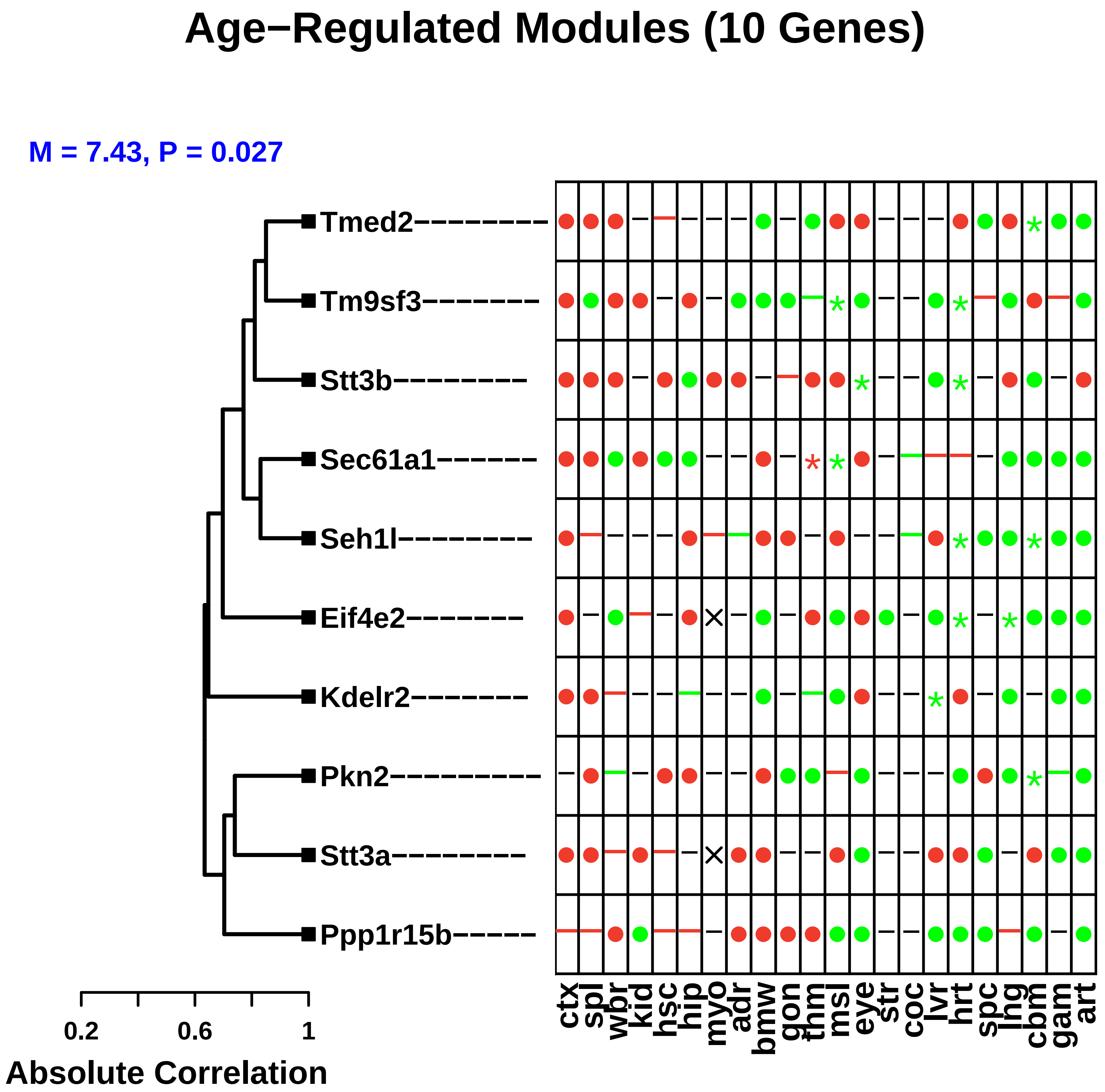
Age−Regulated Modules (10 Genes)
M = 7.43, P = 0.027
Tmed2
Tm9sf3
Stt3b
Sec61a1
Seh1l
Eif4e2
Kdelr2
Pkn2
Stt3a
Ppp1r15b
0.2
0.6
1
Absolute Correlation
ctx
spl
wbr
kid
hsc
hip
myo
adr
bmw
gon
thm
msl
eye
str
coc
lvr
hrt
spc
lng
cbm
gam
art

Age−Regulated Modules (10 Genes)

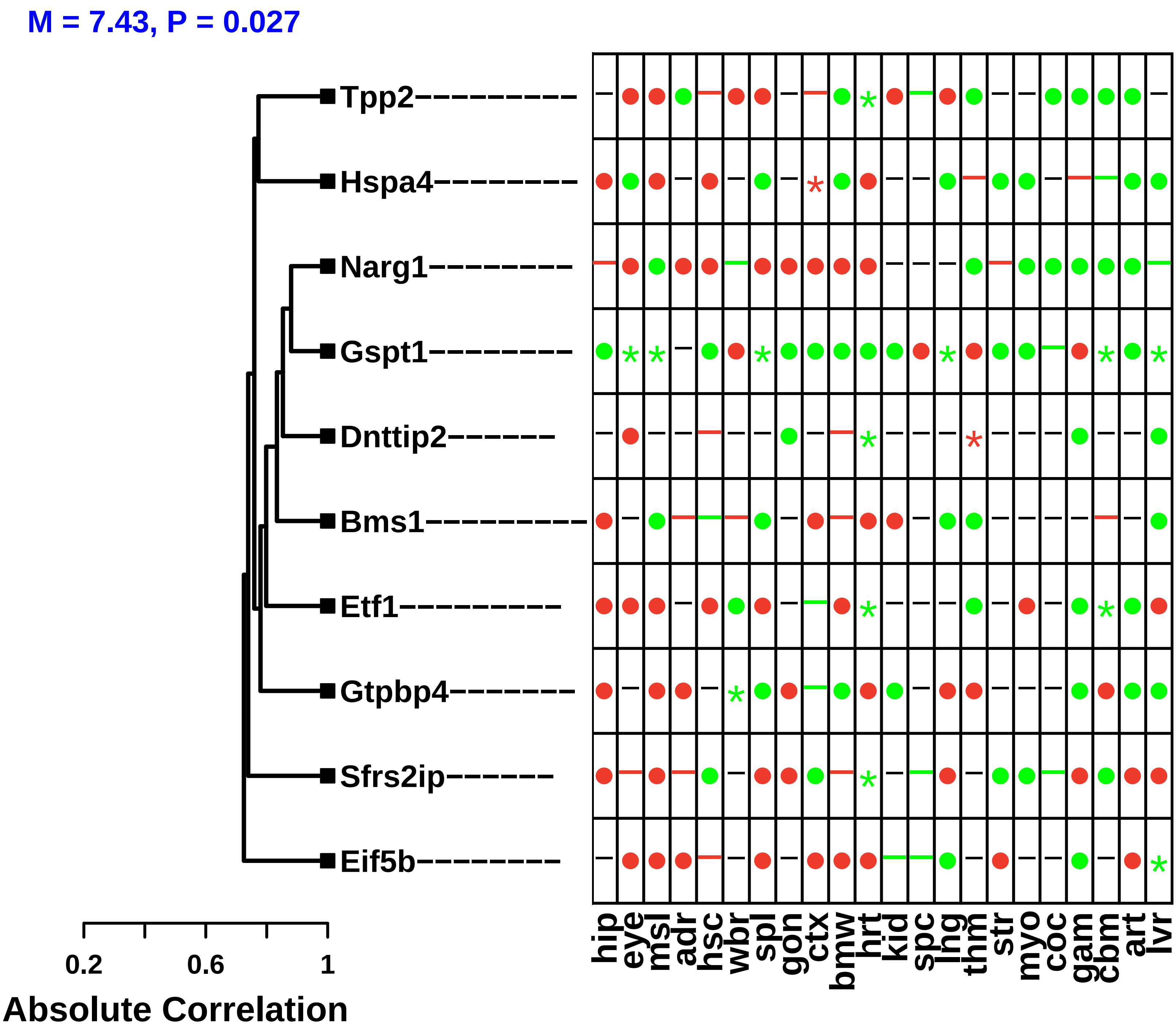

Age−Regulated Modules (10 Genes)

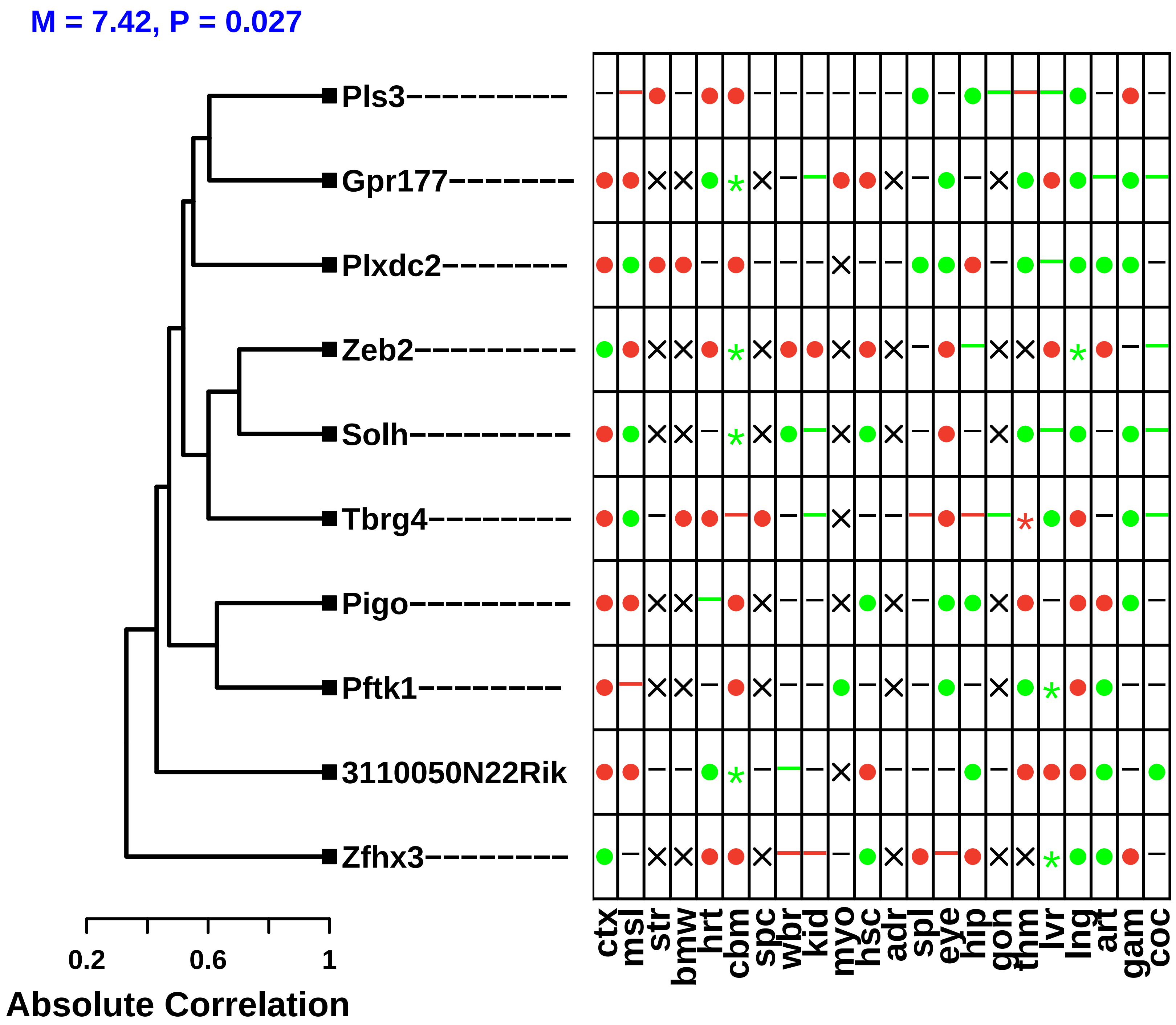

Age−Regulated Modules (10 Genes)

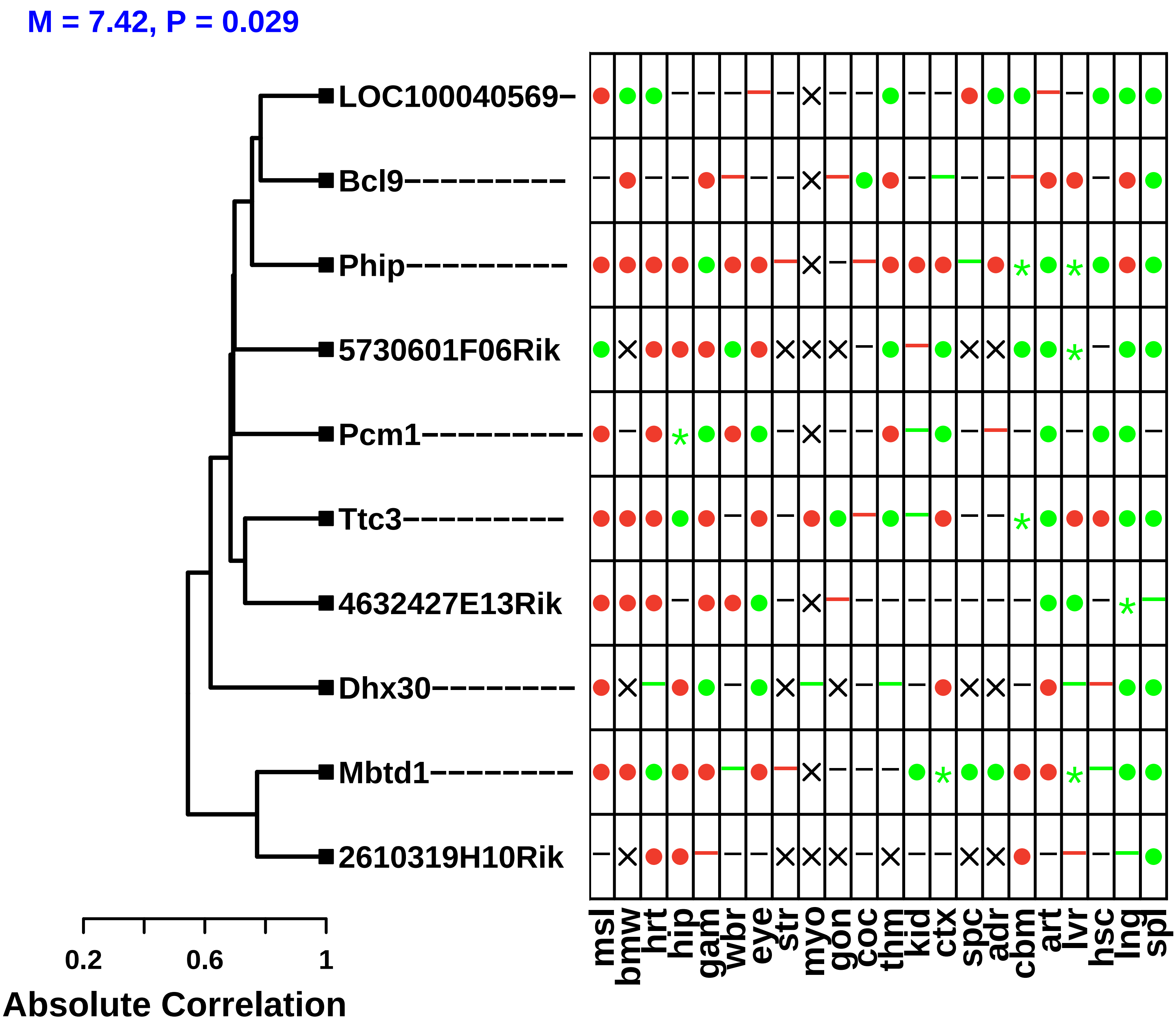

Age−Regulated Modules (10 Genes)

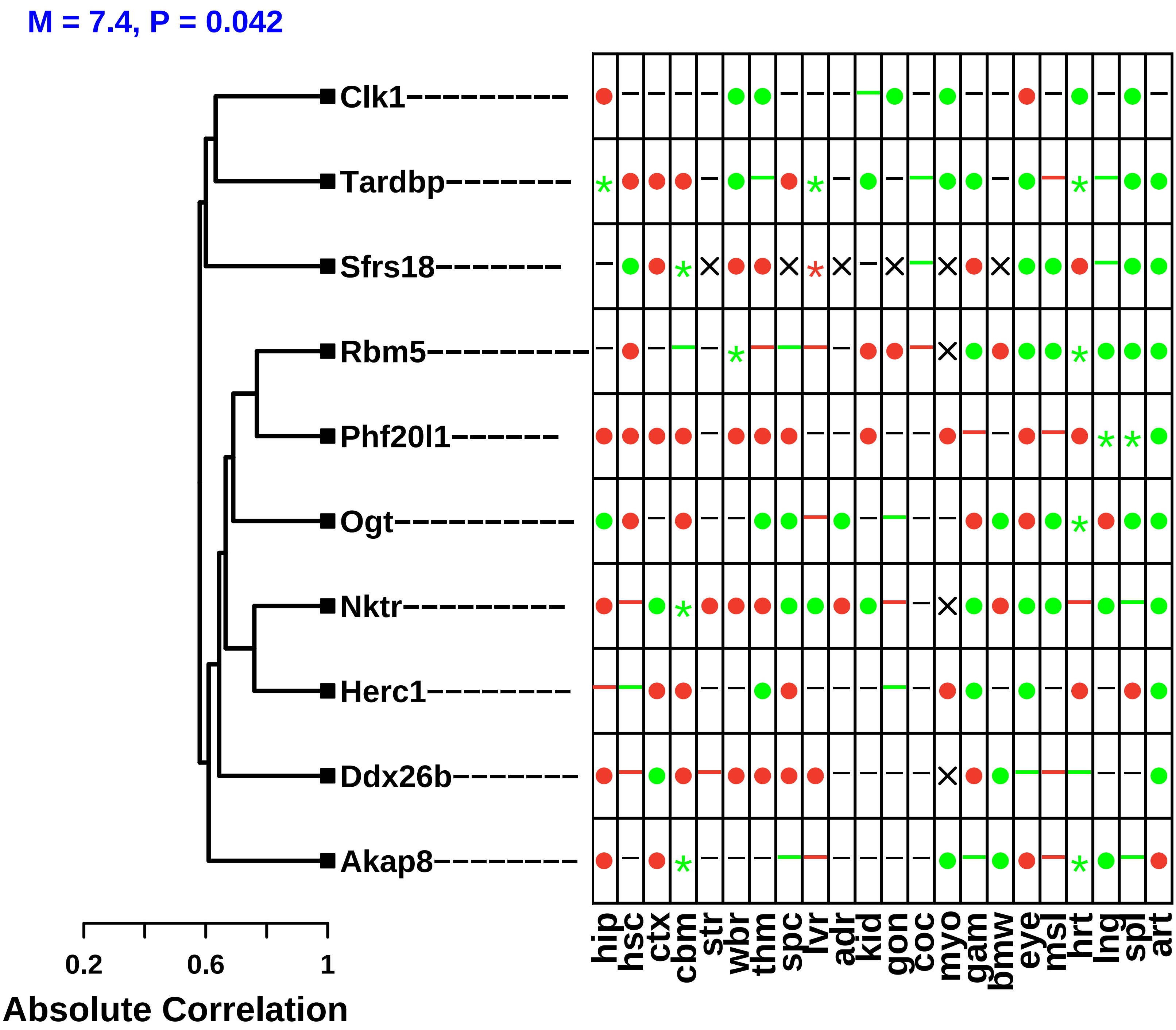

Age−Regulated Modules (10 Genes)

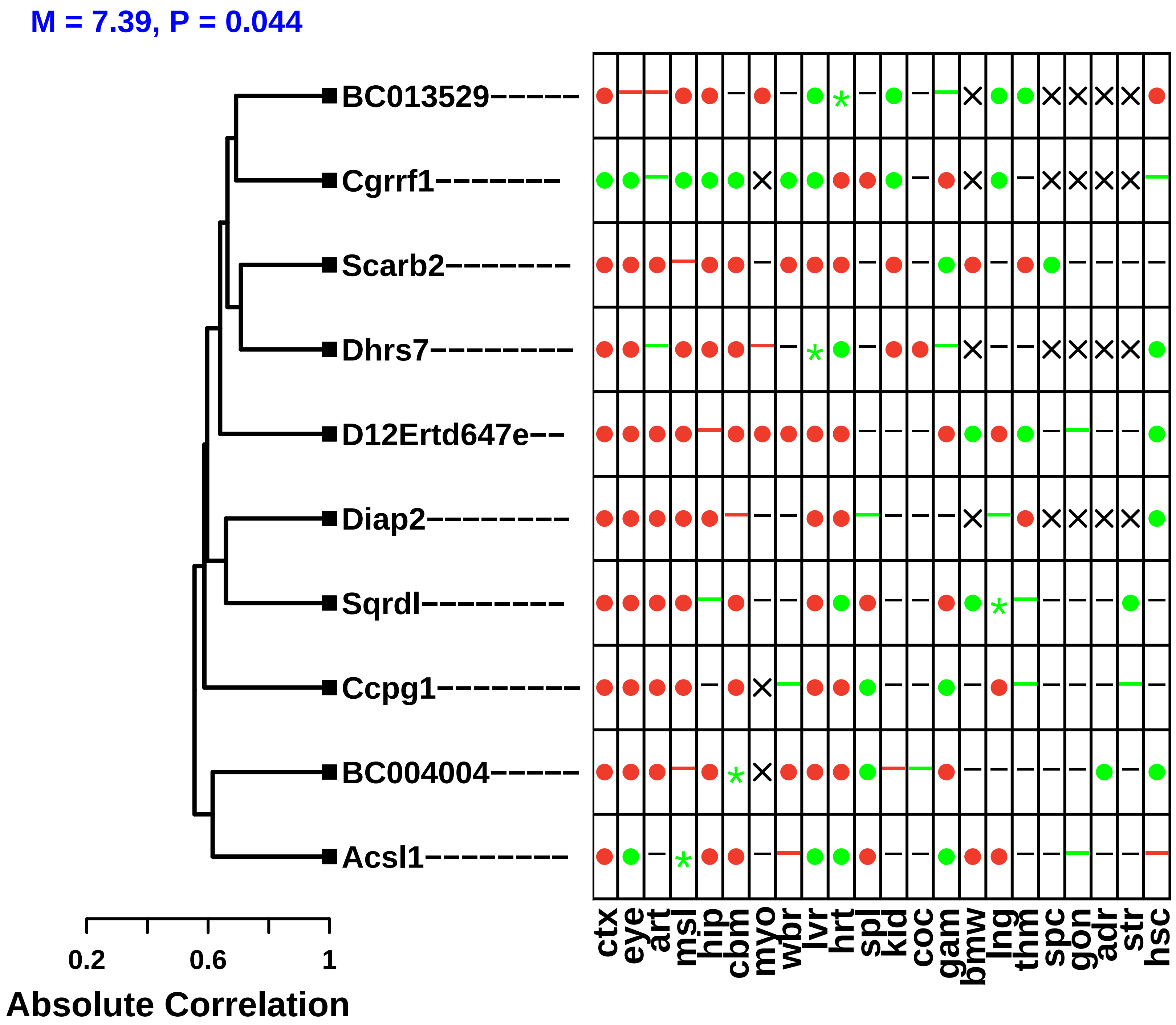

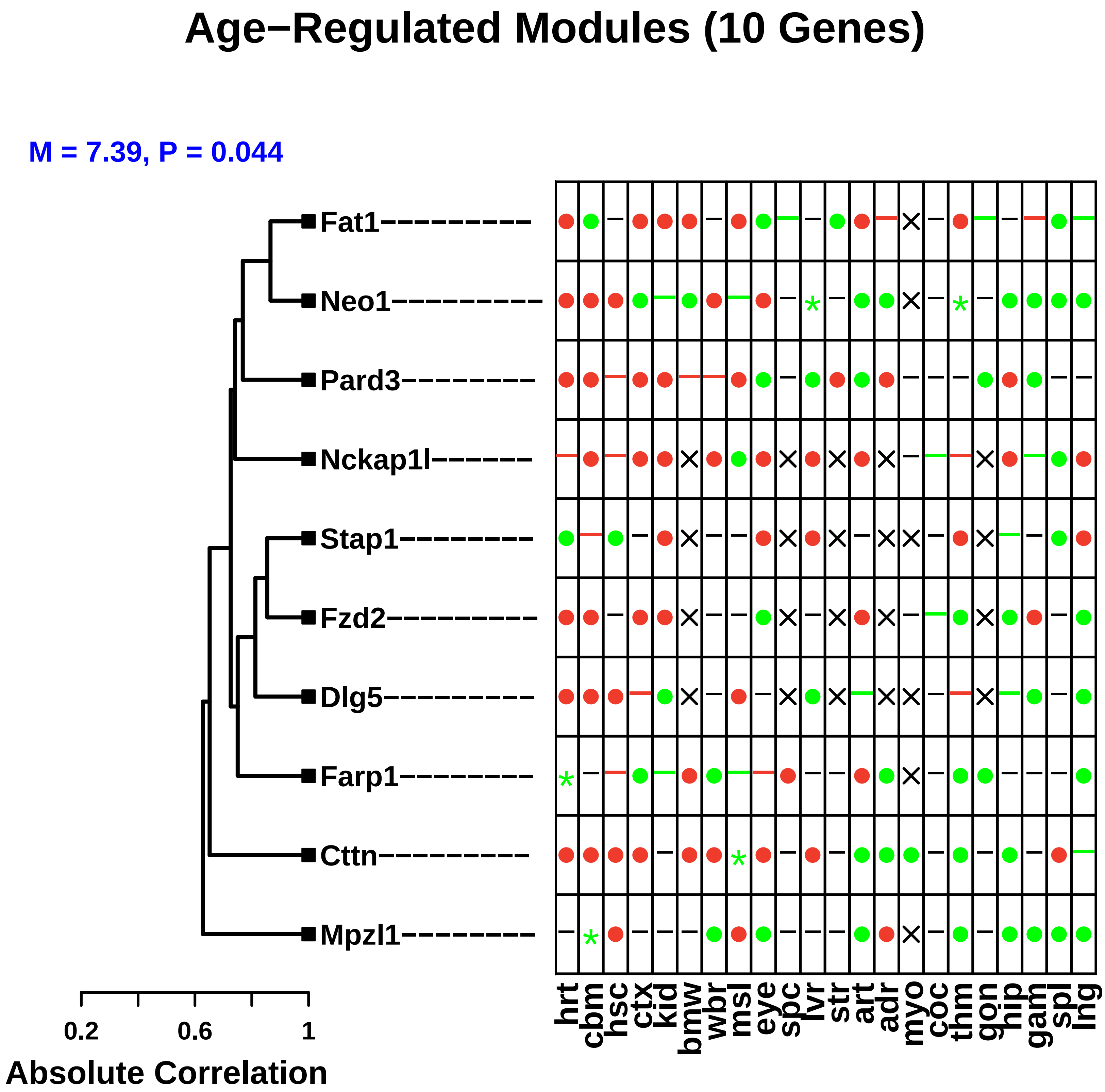
Age−Regulated Modules (10 Genes)
M = 7.39, P = 0.044
Fat1
Neo1
Pard3
Nckap1l
Stap1
Fzd2
Dlg5
Farp1
Cttn
Mpzl1
0.2
0.6
1
Absolute Correlation
hrt
cbm
hsc
ctx
kid
bmw
wbr
msl
eye
spc
lvr
str
art
adr
myo
coc
thm
gon
hip
gam
spl
lng

Age−Regulated Modules (10 Genes)

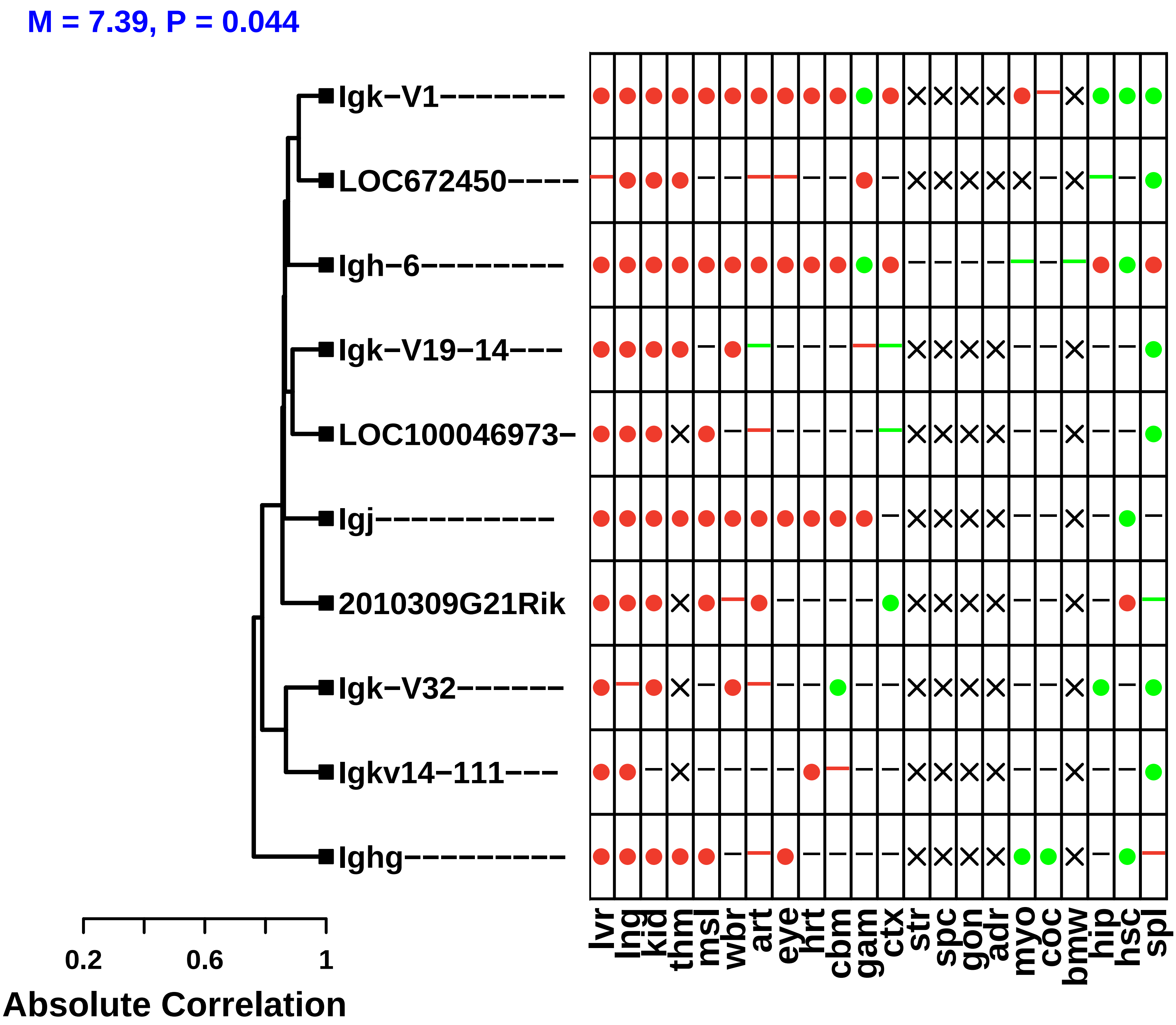

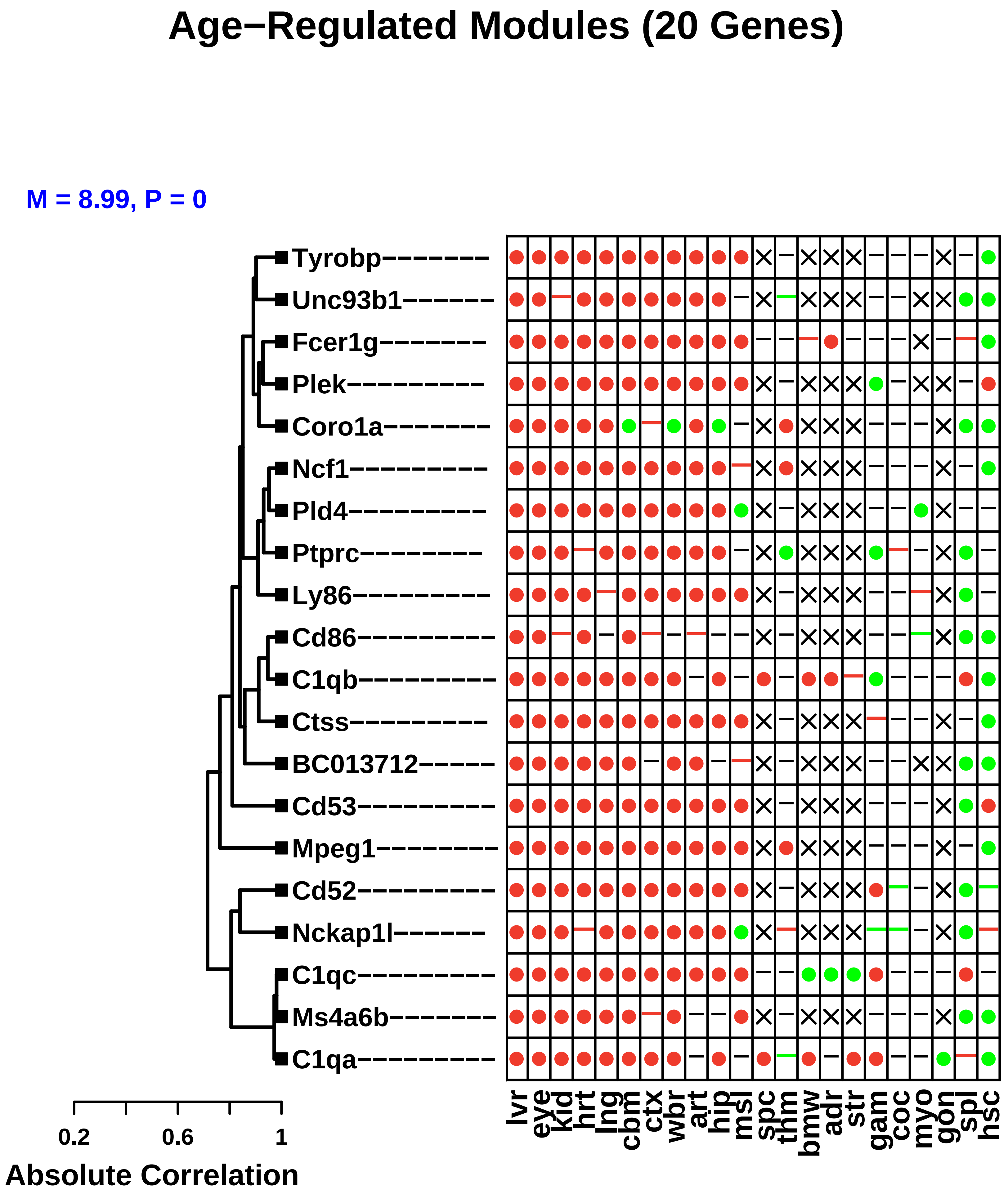

Age−Regulated Modules (20 Genes)
M = 8.99, P = 0
Tyrobp
Unc93b1
Fcer1g
Plek
Coro1a
Ncf1
Pld4
Ptprc
Ly86
Cd86
C1qb
Ctss
BC013712
Cd53
Mpeg1
Cd52
Nckap1l
C1qc
Ms4a6b
C1qa
lvr
eye
kid
hrt
lng
cbm
ctx
wbr
art
hip
msl
spc
thm
bmw
adr
str
gam
coc
myo
gon
spl
hsc
0.2
0.6
1
Absolute Correlation

Age−Regulated Modules (20 Genes)

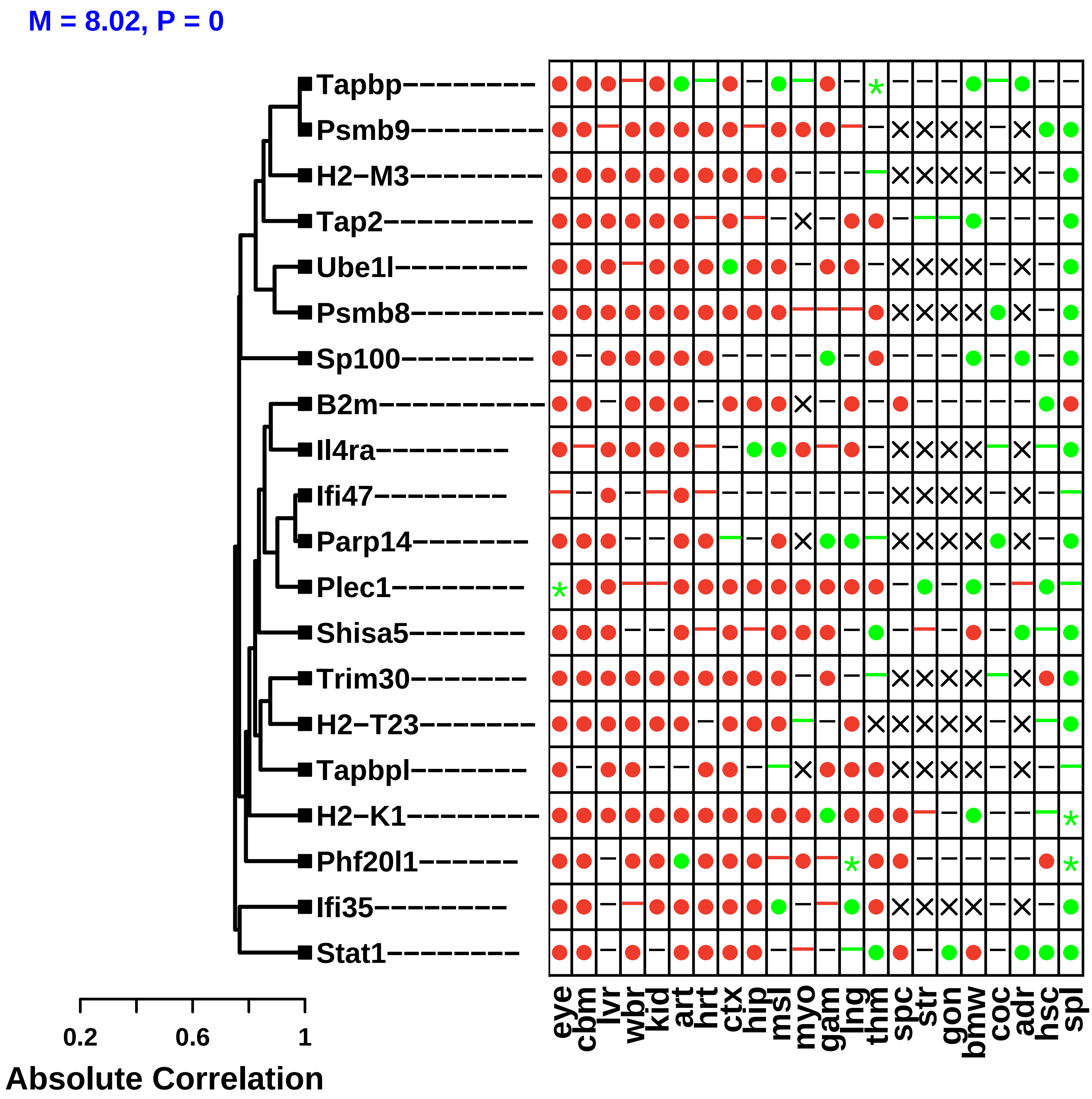

Age−Regulated Modules (20 Genes)

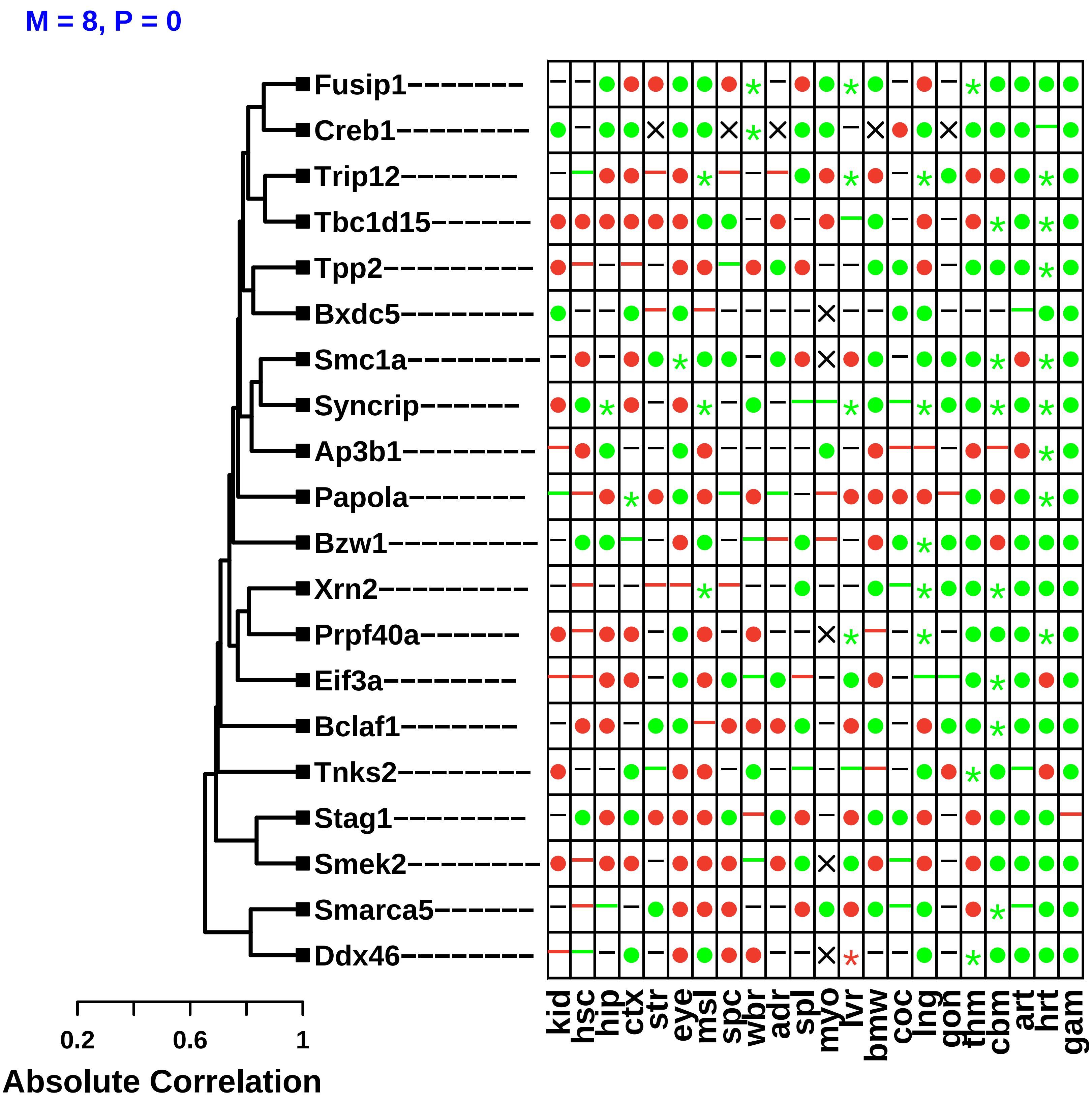

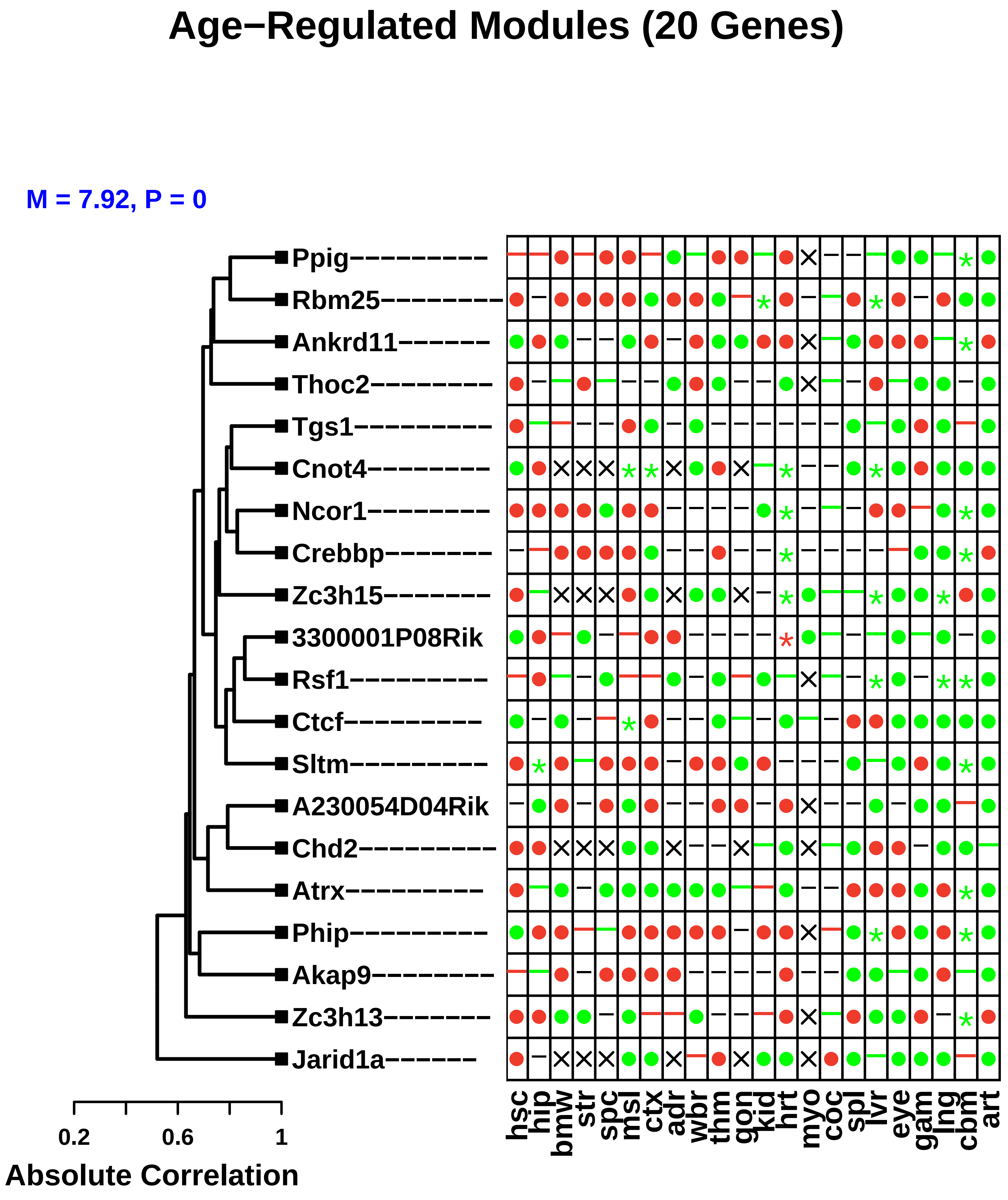
Age−Regulated Modules (20 Genes)
M = 7.92, P = 0
Ppig
Rbm25
Ankrd11
Thoc2
Tgs1
Cnot4
Ncor1
Crebbp
Zc3h15
3300001P08Rik
Rsf1
Ctcf
Sltm
A230054D04Rik
Chd2
Atrx
Phip
Akap9
Zc3h13
Jarid1a
hsc hip bmw str spc msl ctx adr wbr thm gon kid hrt myo coc spl lvr eye gam lng cbm art
0.2
0.6
1
Absolute Correlation

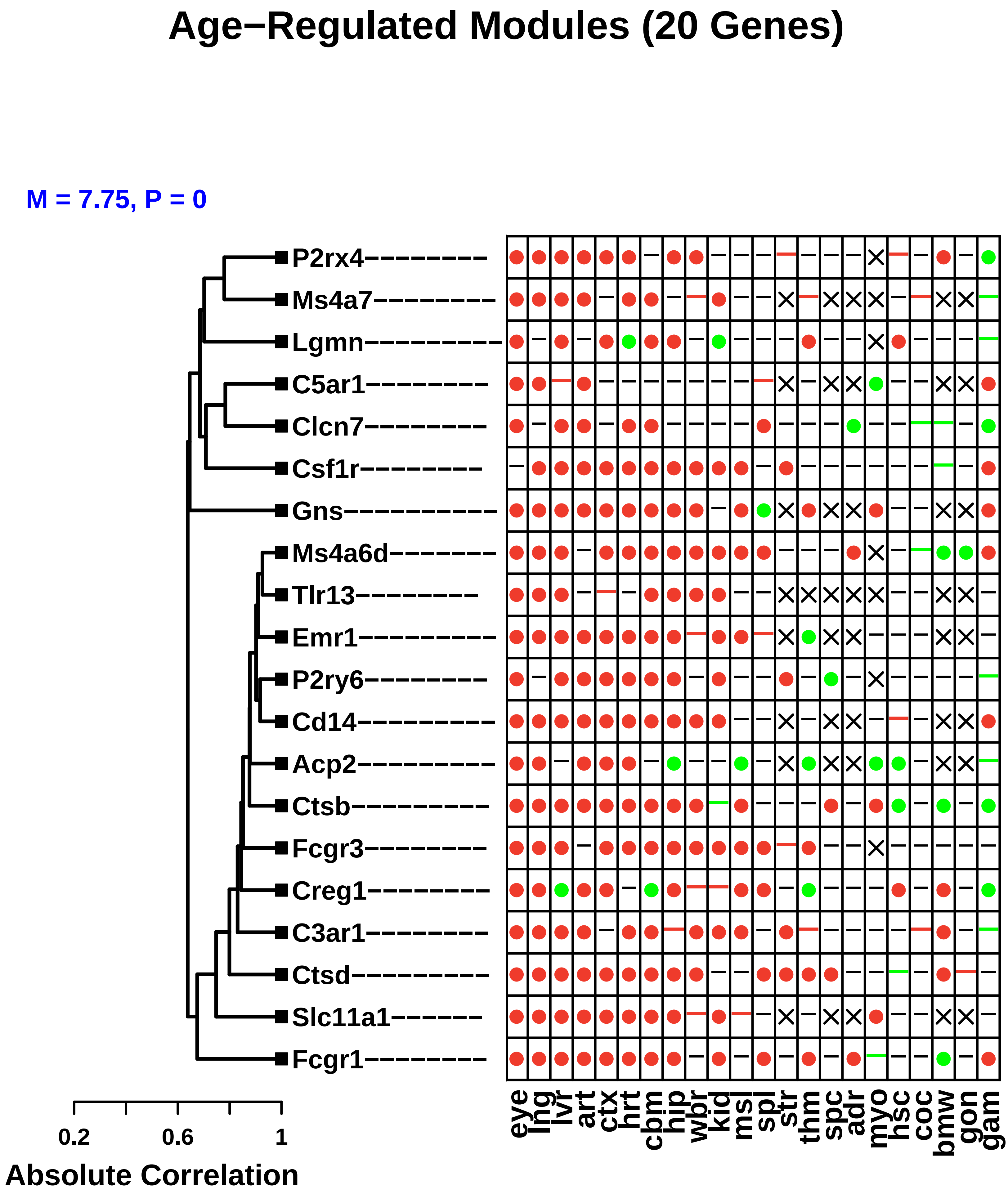
Age−Regulated Modules (20 Genes)
M = 7.75, P = 0
P2rx4
Ms4a7
Lgmn
C5ar1
Clcn7
Csf1r
Gns
Ms4a6d
Tlr13
Emr1
P2ry6
Cd14
Acp2
Ctsb
Fcgr3
Creg1
C3ar1
Ctsd
Slc11a1
Fcgr1
eye lng lvr art ctx hrt cbm hip wbr kid msl spl str thm spc adr myo hsc coc bmw gon gam
0.2
0.6
1
Absolute Correlation

Age−Regulated Modules (20 Genes)

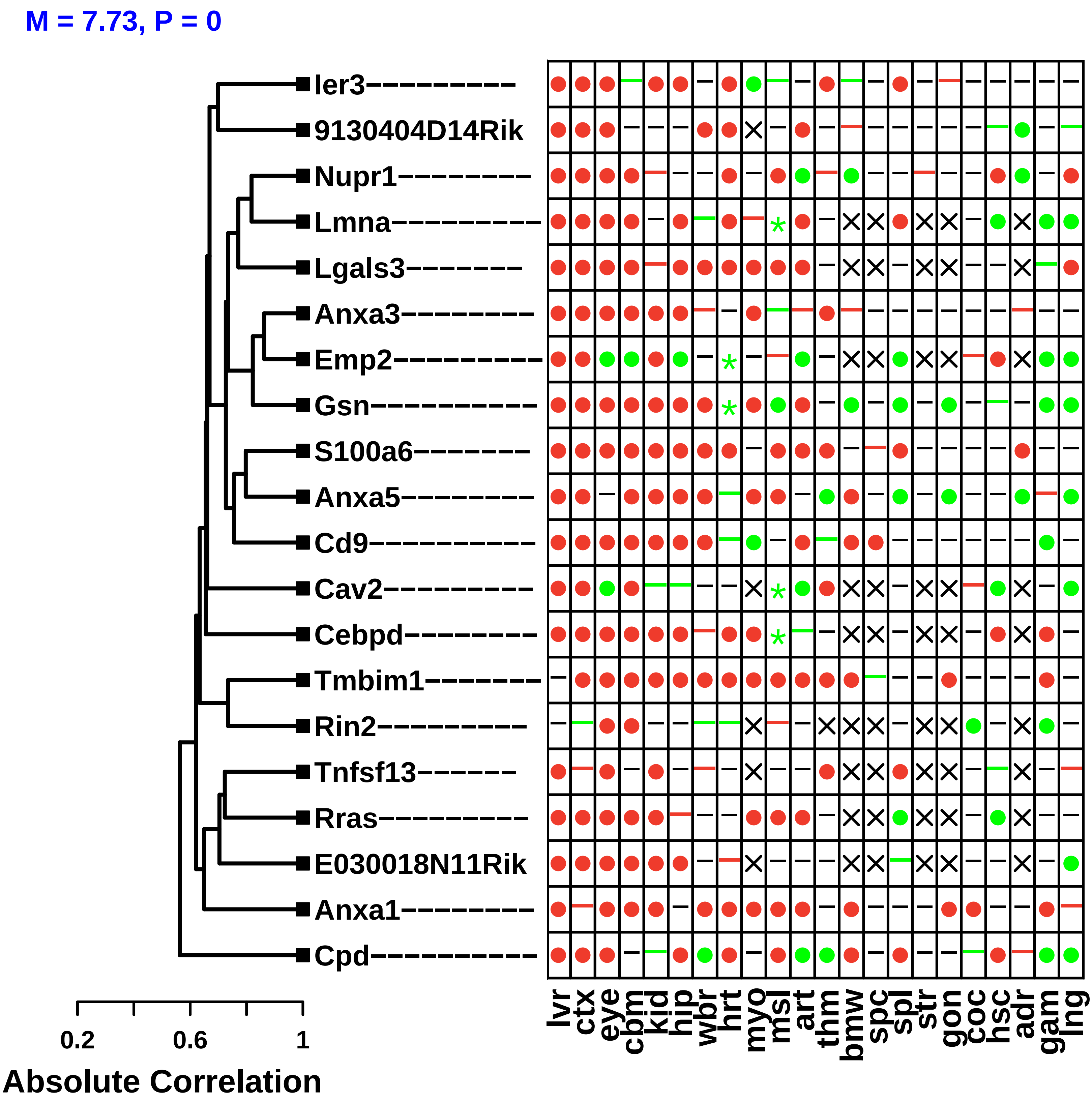

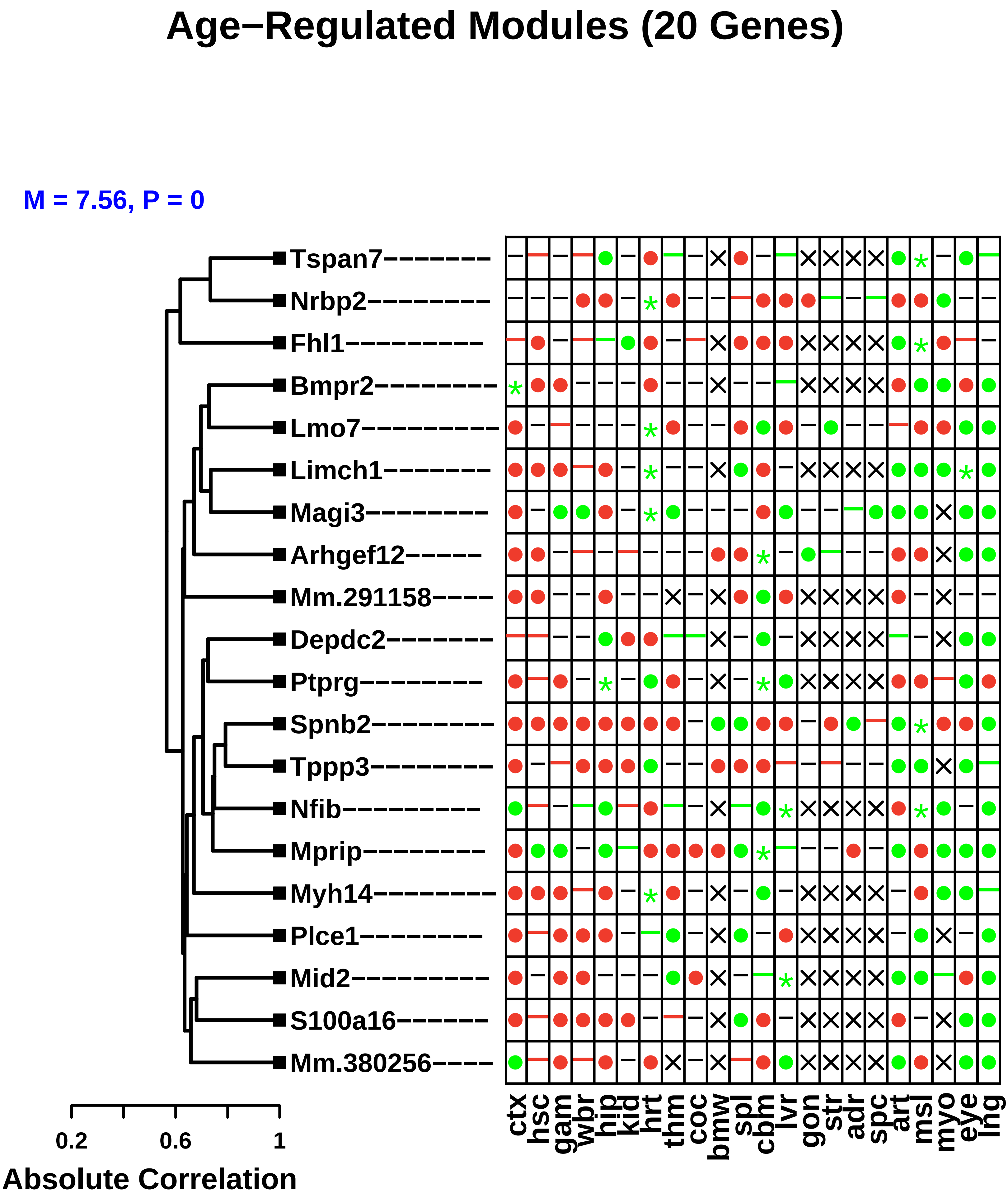

Age−Regulated Modules (20 Genes)
M = 7.56, P = 0
Tspan7
Nrbp2
Fhl1
Bmpr2
Lmo7
Limch1
Magi3
Arhgef12
Mm.291158
Depdc2
Ptprg
Spnb2
Tppp3
Nfib
Mprip
Myh14
Plce1
Mid2
S100a16
Mm.380256
ctx
hsc
gam
wbr
hip
kid
hrt
thm
coc
bmw
spl
cbm
lvr
gon
str
adr
spc
art
msl
myo
eye
lng
0.2
0.6
1
Absolute Correlation

Age−Regulated Modules (20 Genes)

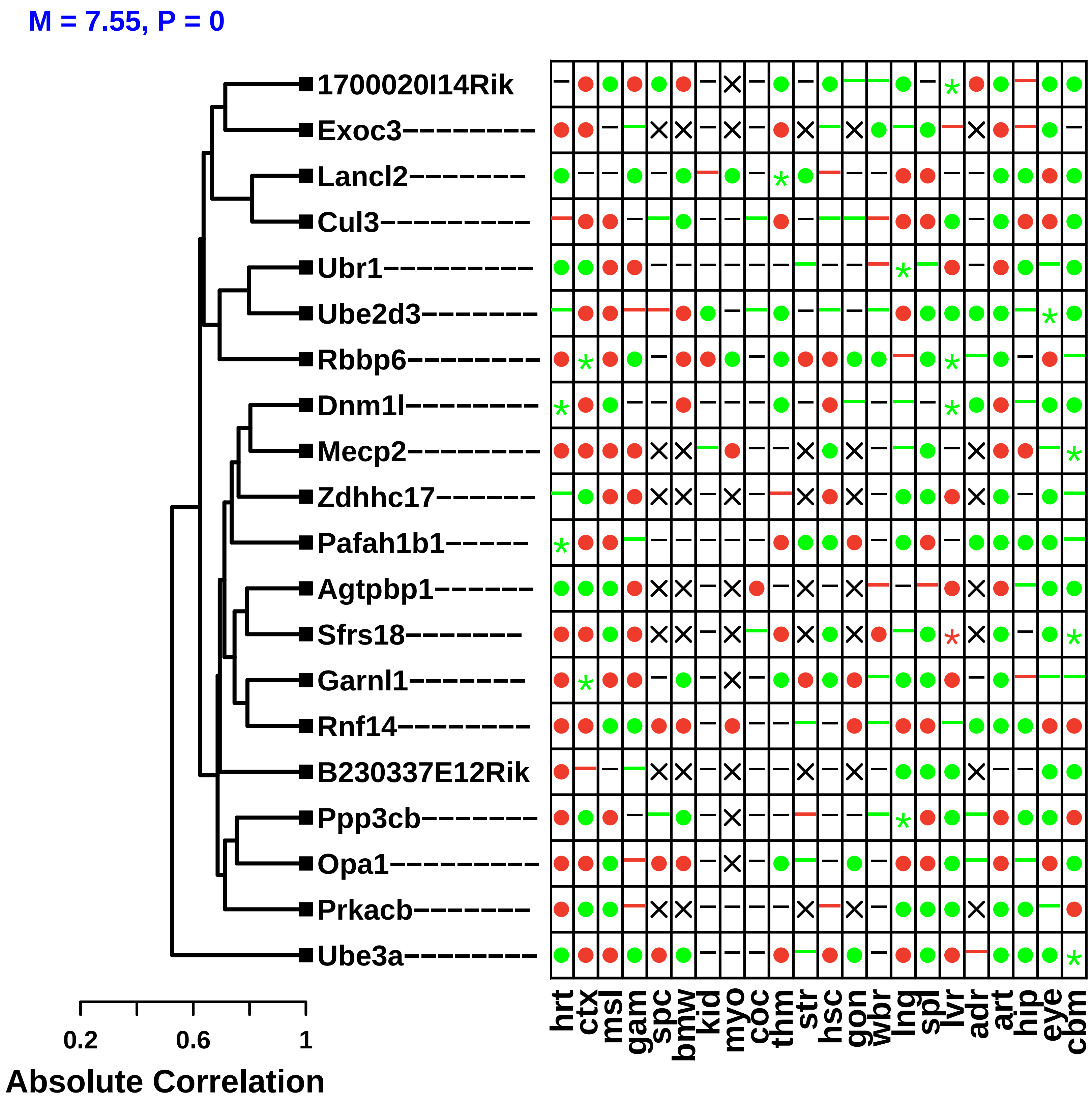

Age−Regulated Modules (20 Genes)

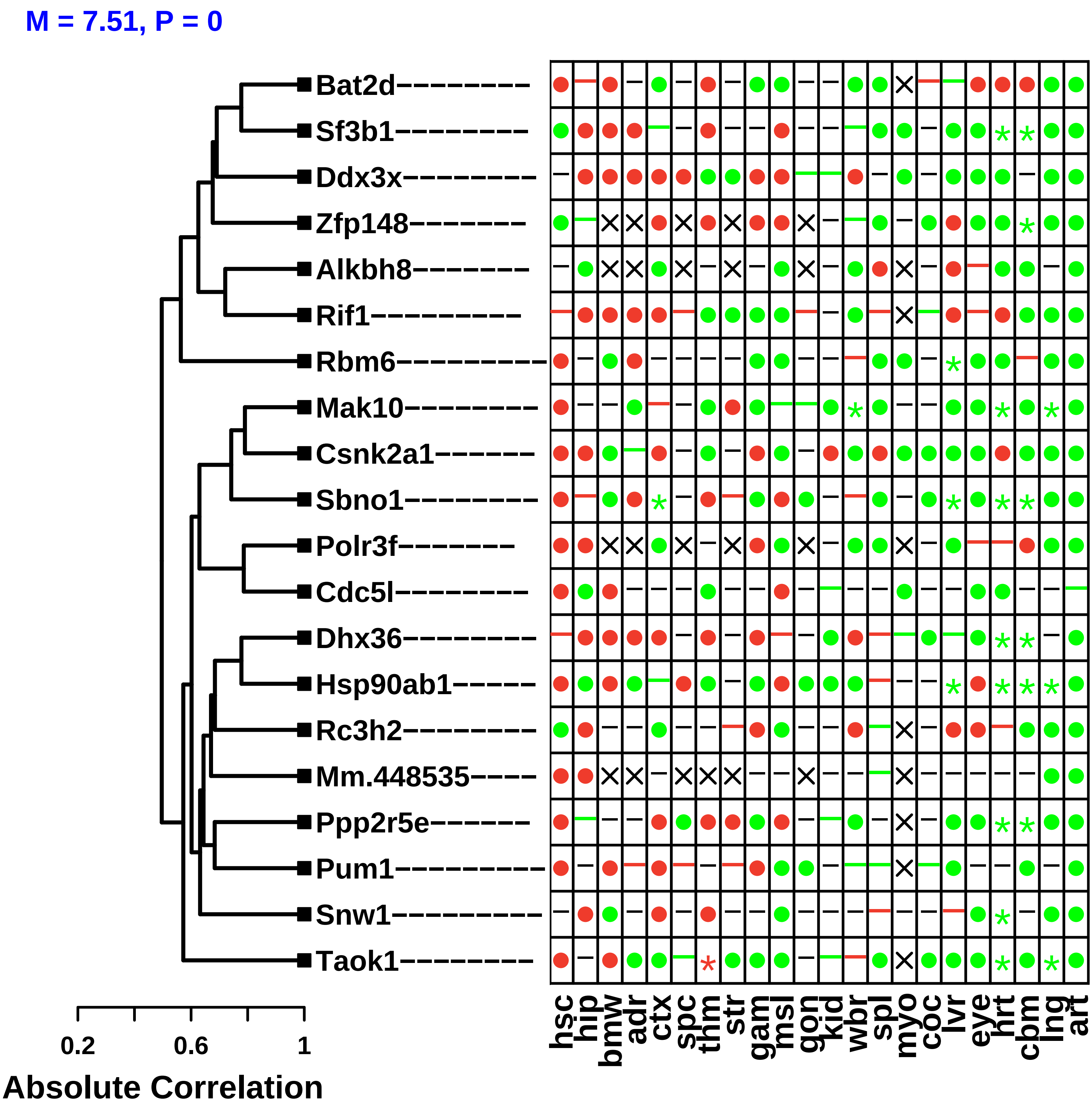

Age−Regulated Modules (20 Genes)

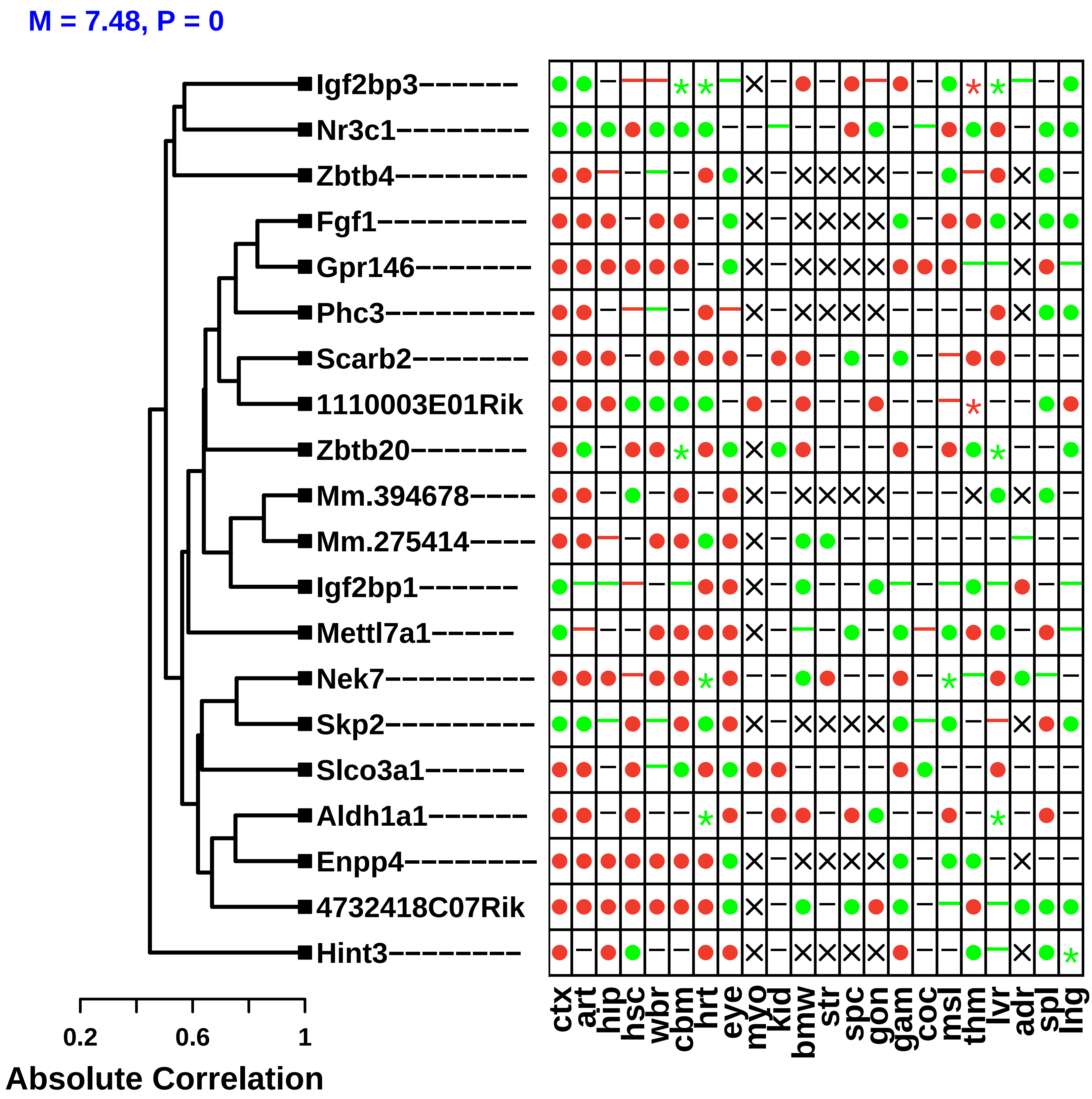

Age−Regulated Modules (20 Genes)

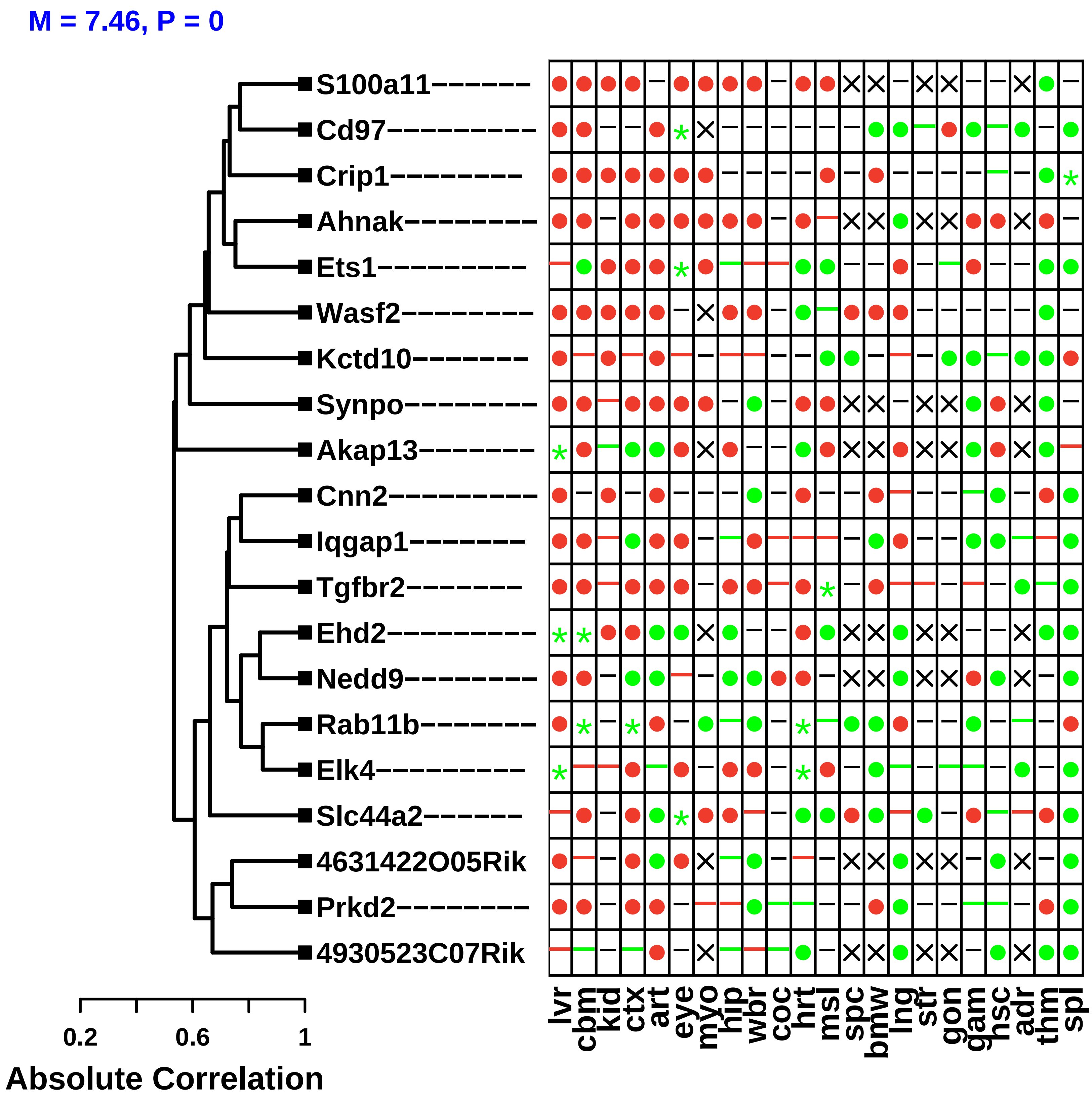

Age−Regulated Modules (20 Genes)

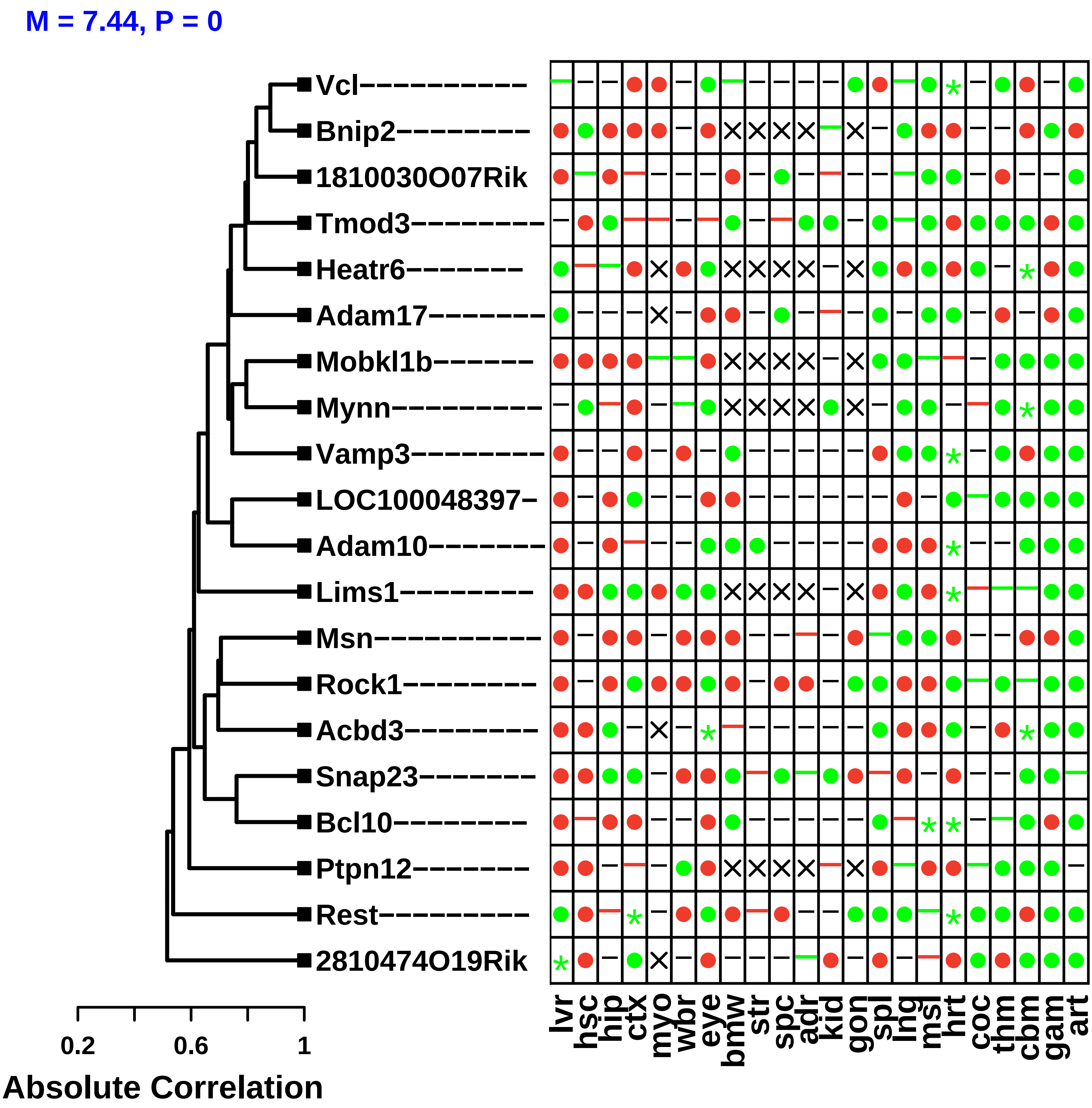

Age−Regulated Modules (20 Genes)

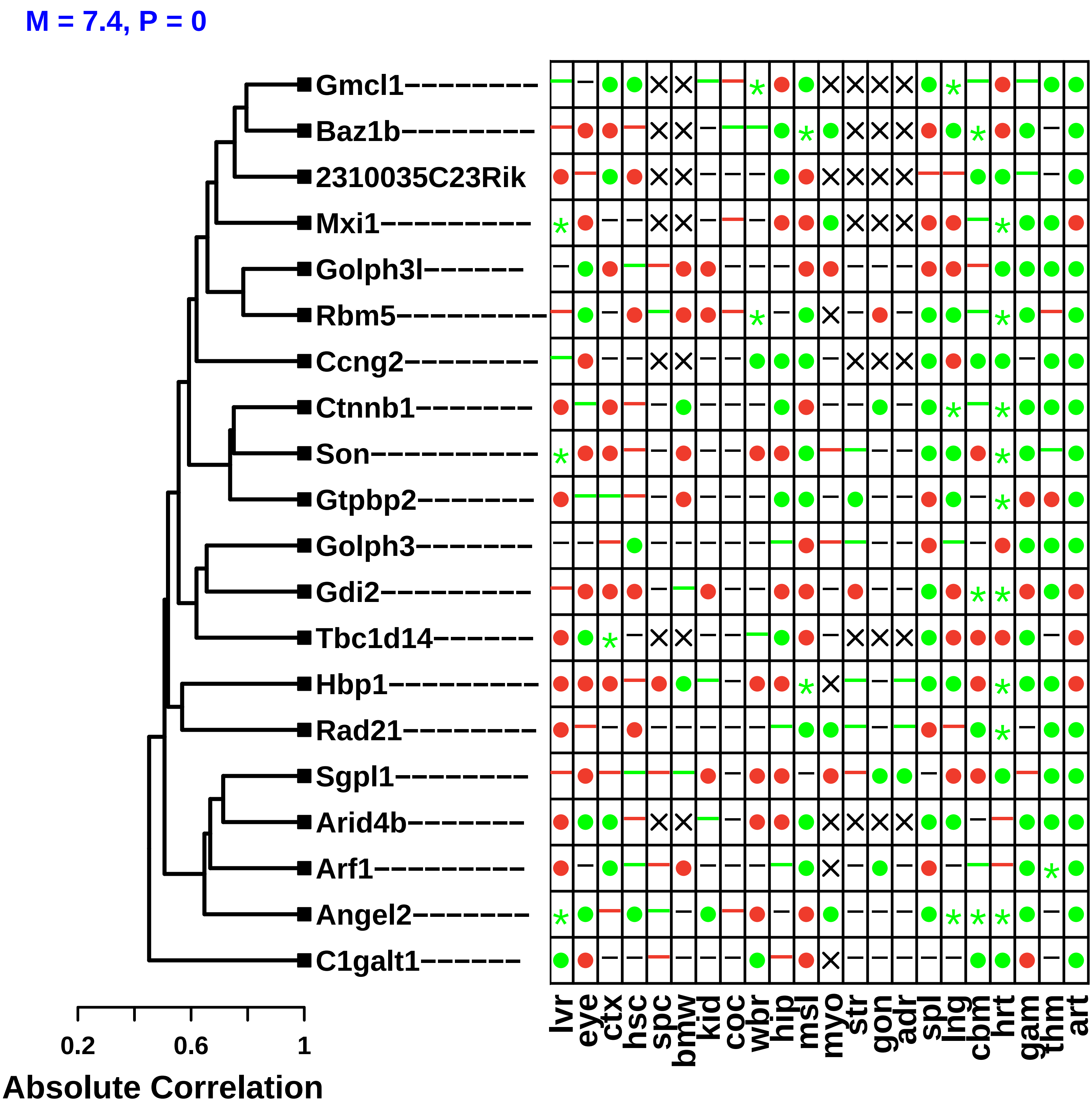

Age−Regulated Modules (20 Genes)

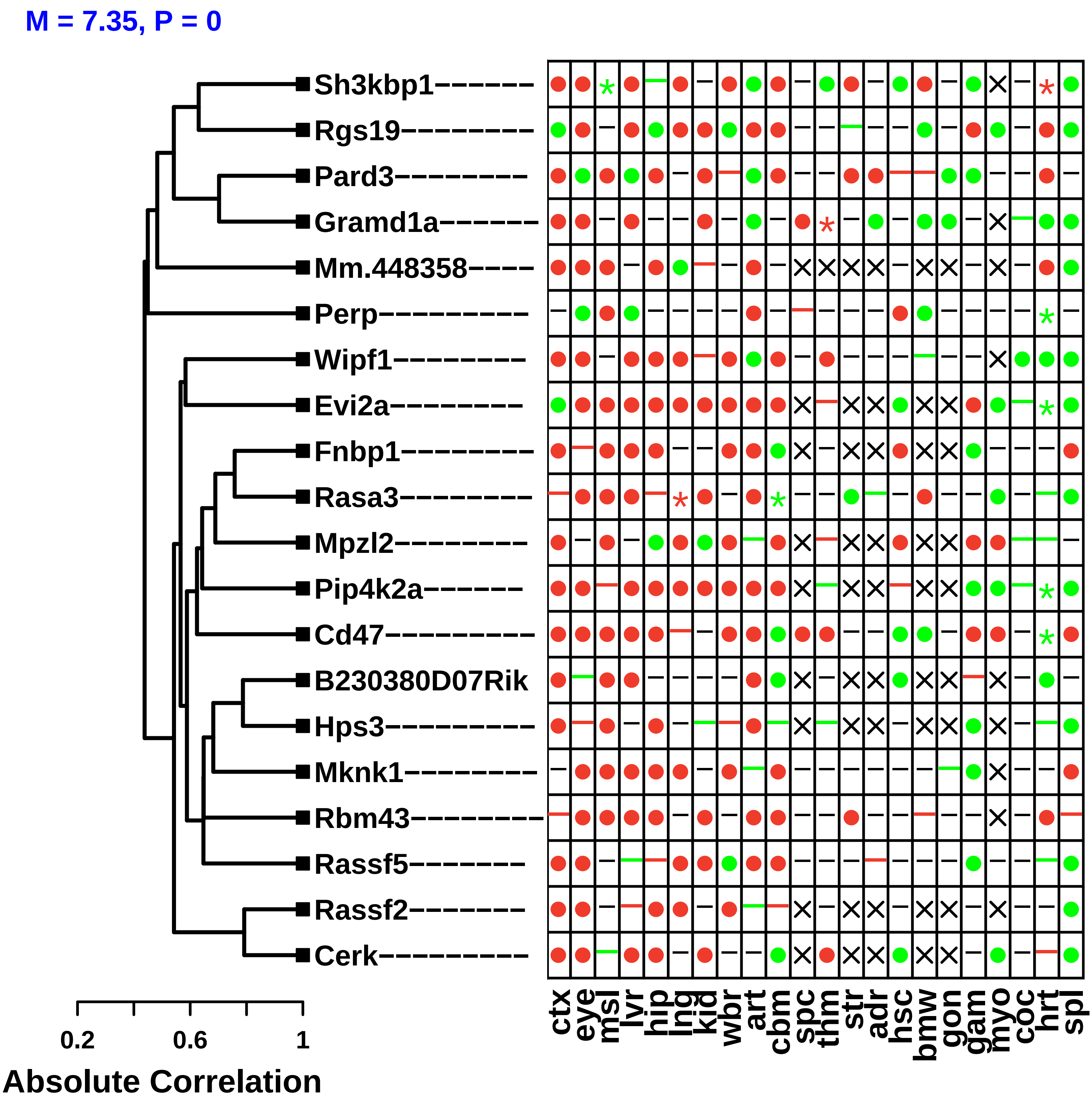

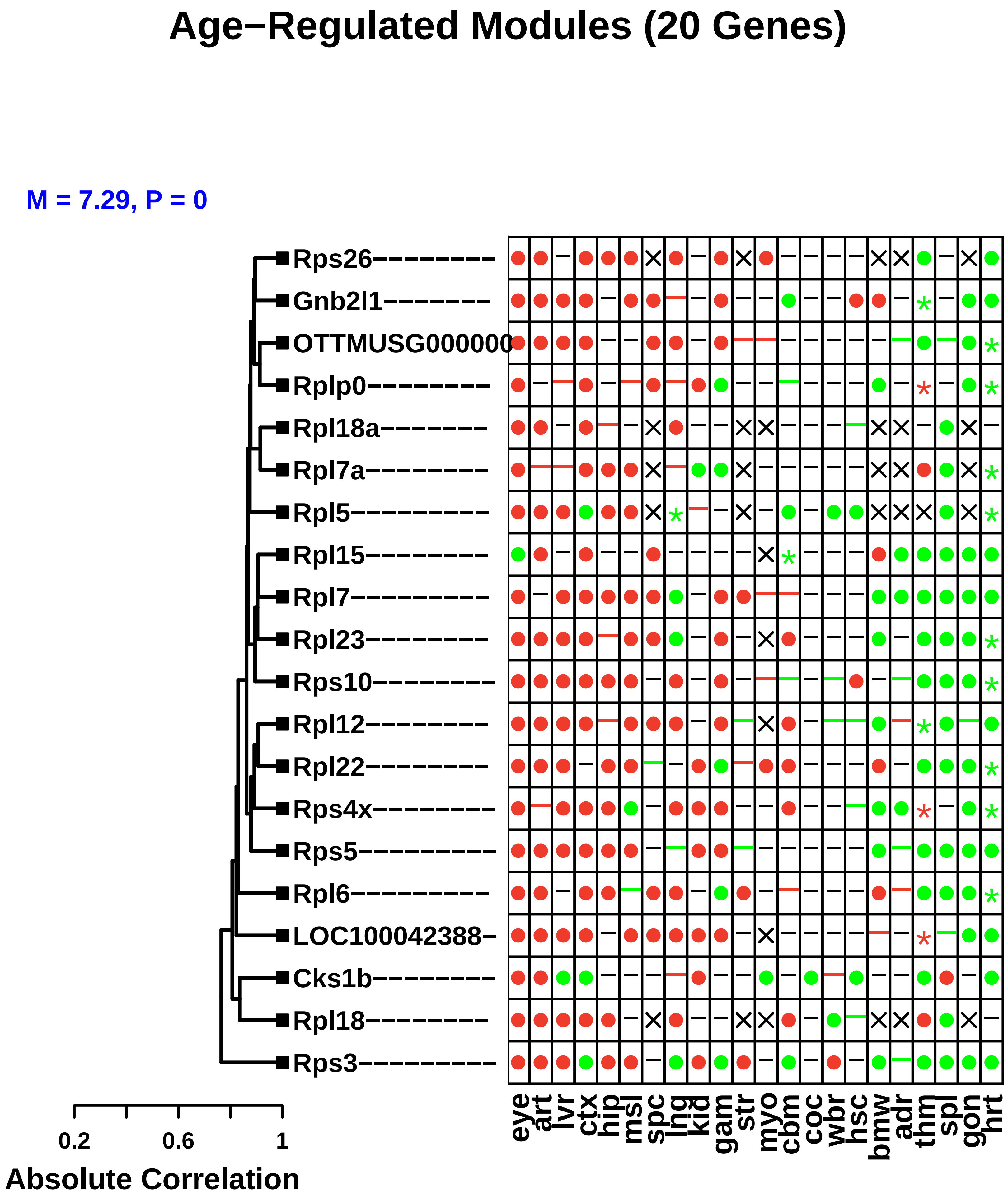
Age−Regulated Modules (20 Genes)
M = 7.29, P = 0
Rps26
Gnb2l1
OTTMUSG000000
Rplp0
Rpl18a
Rpl7a
Rpl5
Rpl15
Rpl7
Rpl23
Rps10
Rpl12
Rpl22
Rps4x
Rps5
Rpl6
LOC100042388
Cks1b
Rpl18
Rps3
eye art lvr ctx hip msl spc lng kid gam str myo cbm coc wbr hsc bmw adr thm spl gon hrt
0.2
0.6
1
Absolute Correlation

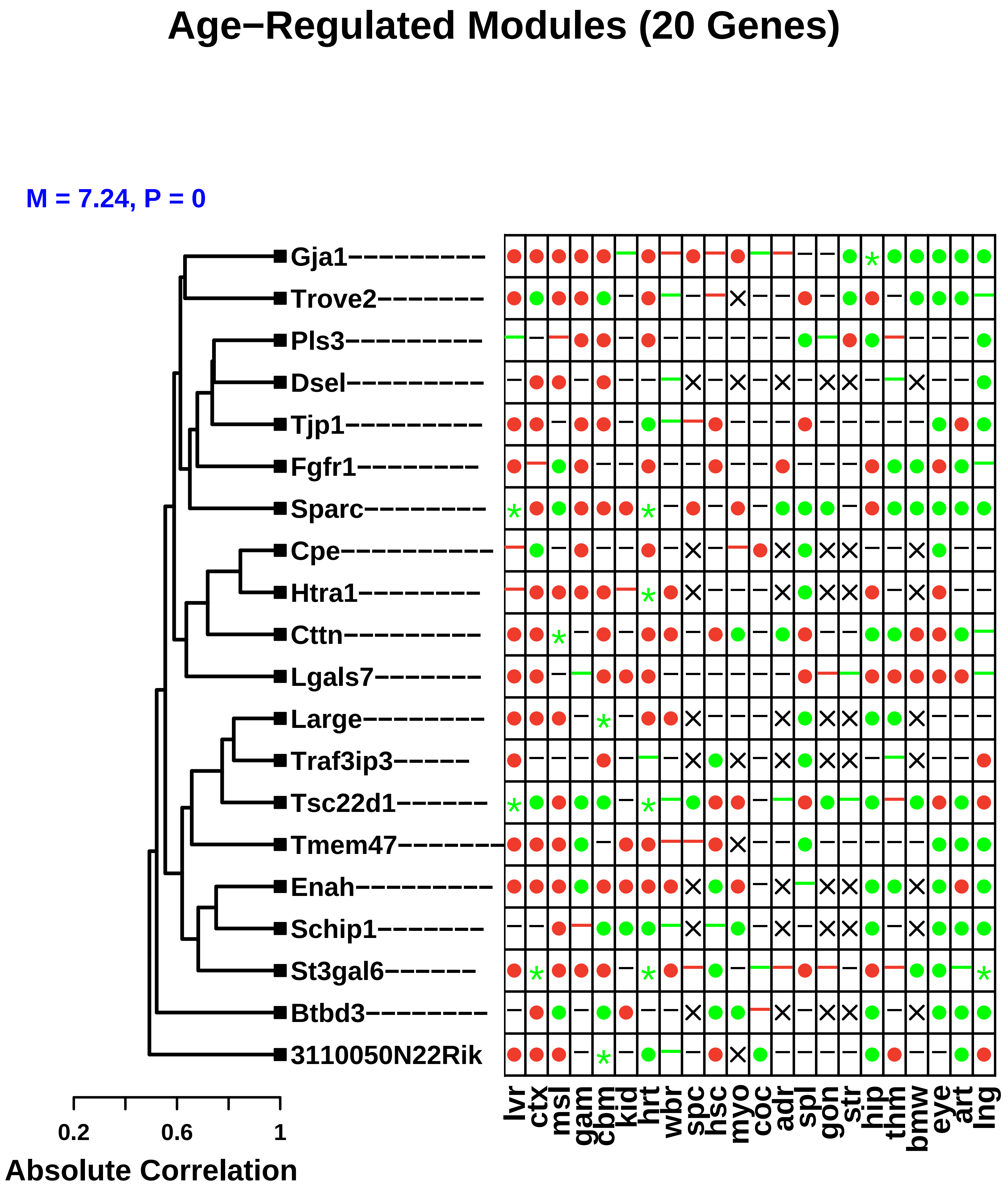

Age−Regulated Modules (20 Genes)
M = 7.24, P = 0
Gja1
Trove2
Pls3
Dsel
Tjp1
Fgfr1
Sparc
Cpe
Htra1
Cttn
Lgals7
Large
Traf3ip3
Tsc22d1
Tmem47
Enah
Schip1
St3gal6
Btbd3
3110050N22Rik
0.2
0.6
1
Absolute Correlation
lvr
ctx
msl
gam
cbm
kid
hrt
wbr
spc
hsc
myo
coc
adr
spl
gon
str
hip
thm
bmw
eye
art
lng

Age−Regulated Modules (20 Genes)

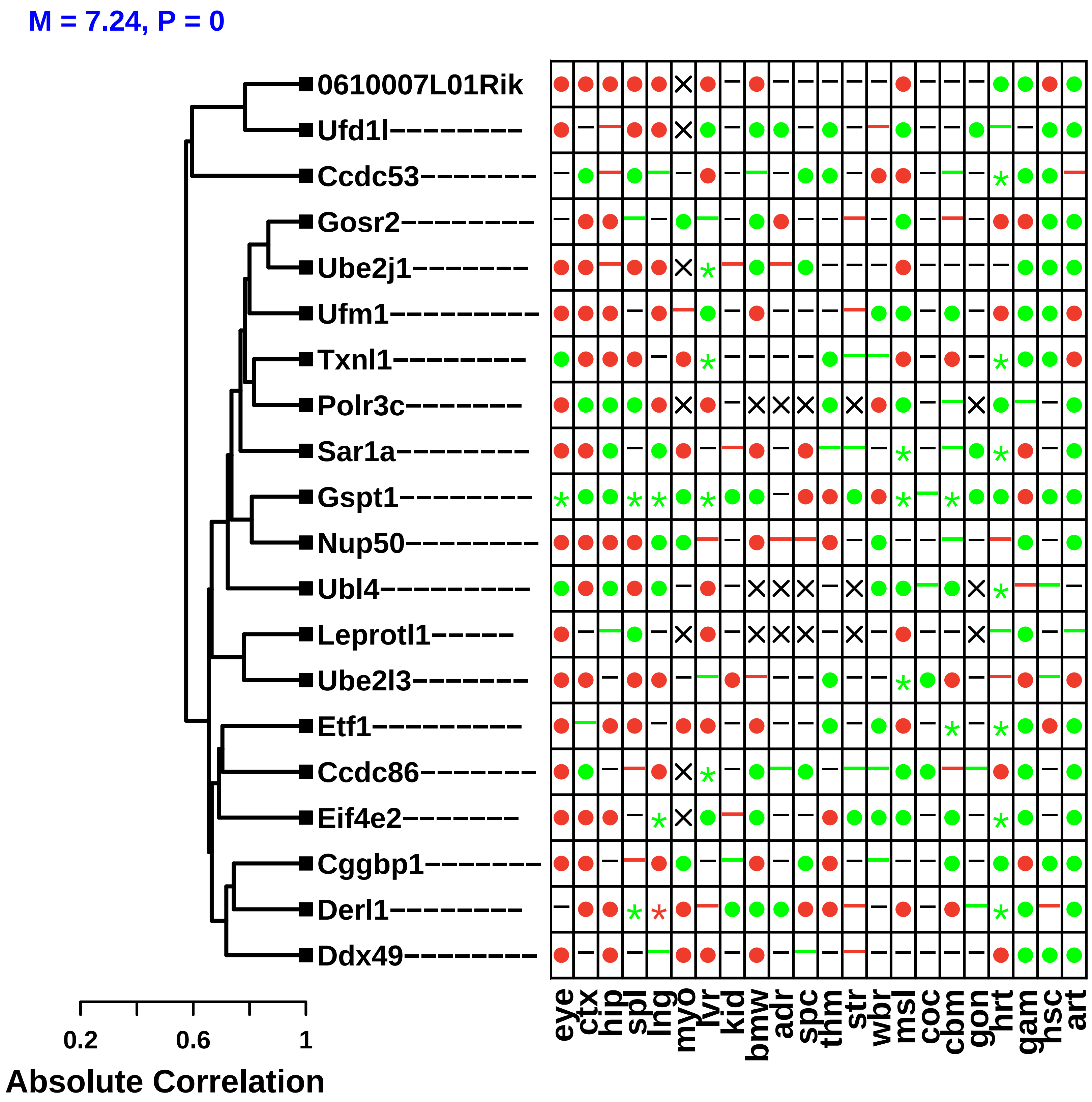

Age−Regulated Modules (20 Genes)

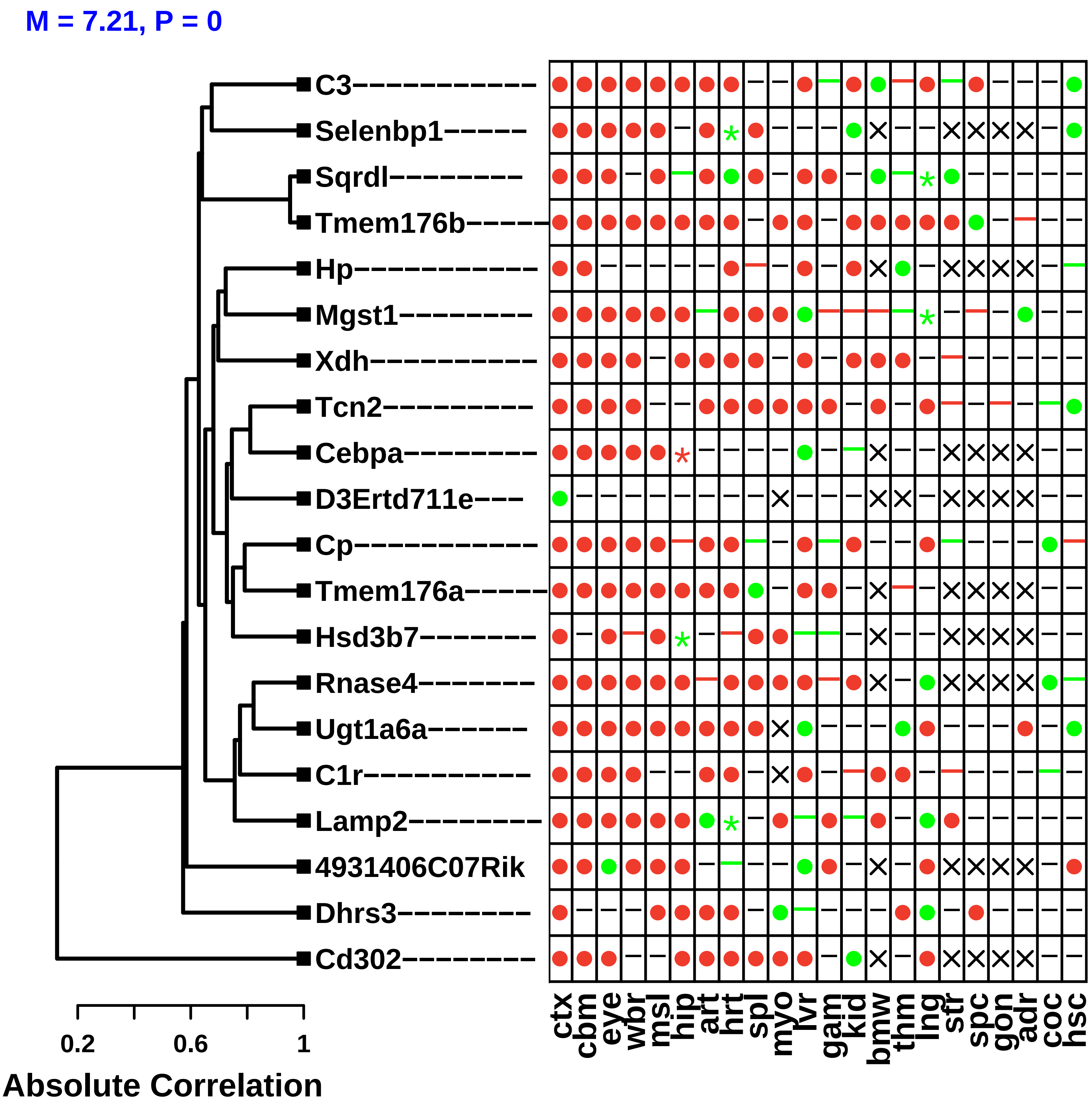

Age−Regulated Modules (20 Genes)

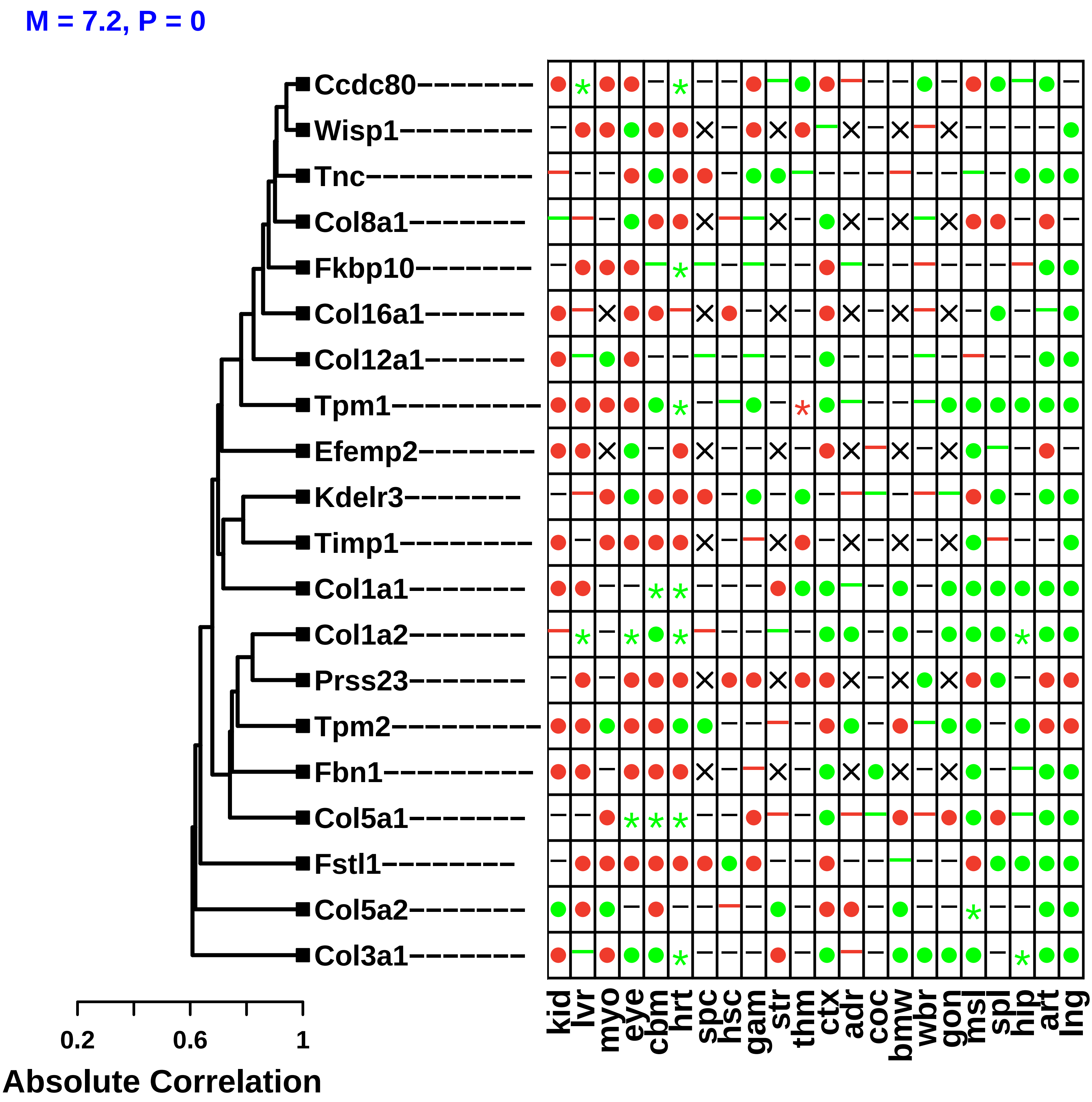

Age−Regulated Modules (20 Genes)

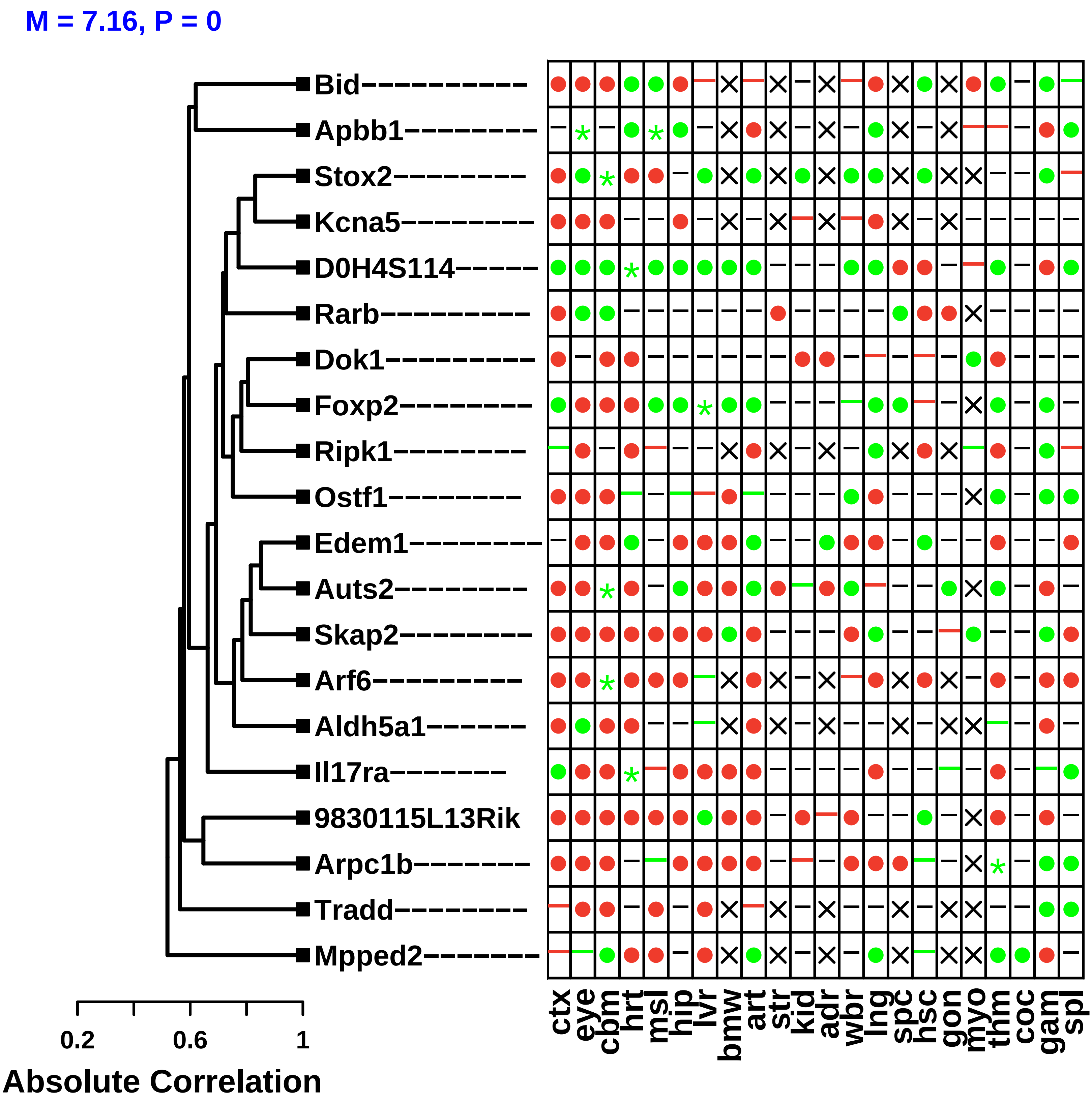

Age−Regulated Modules (20 Genes)

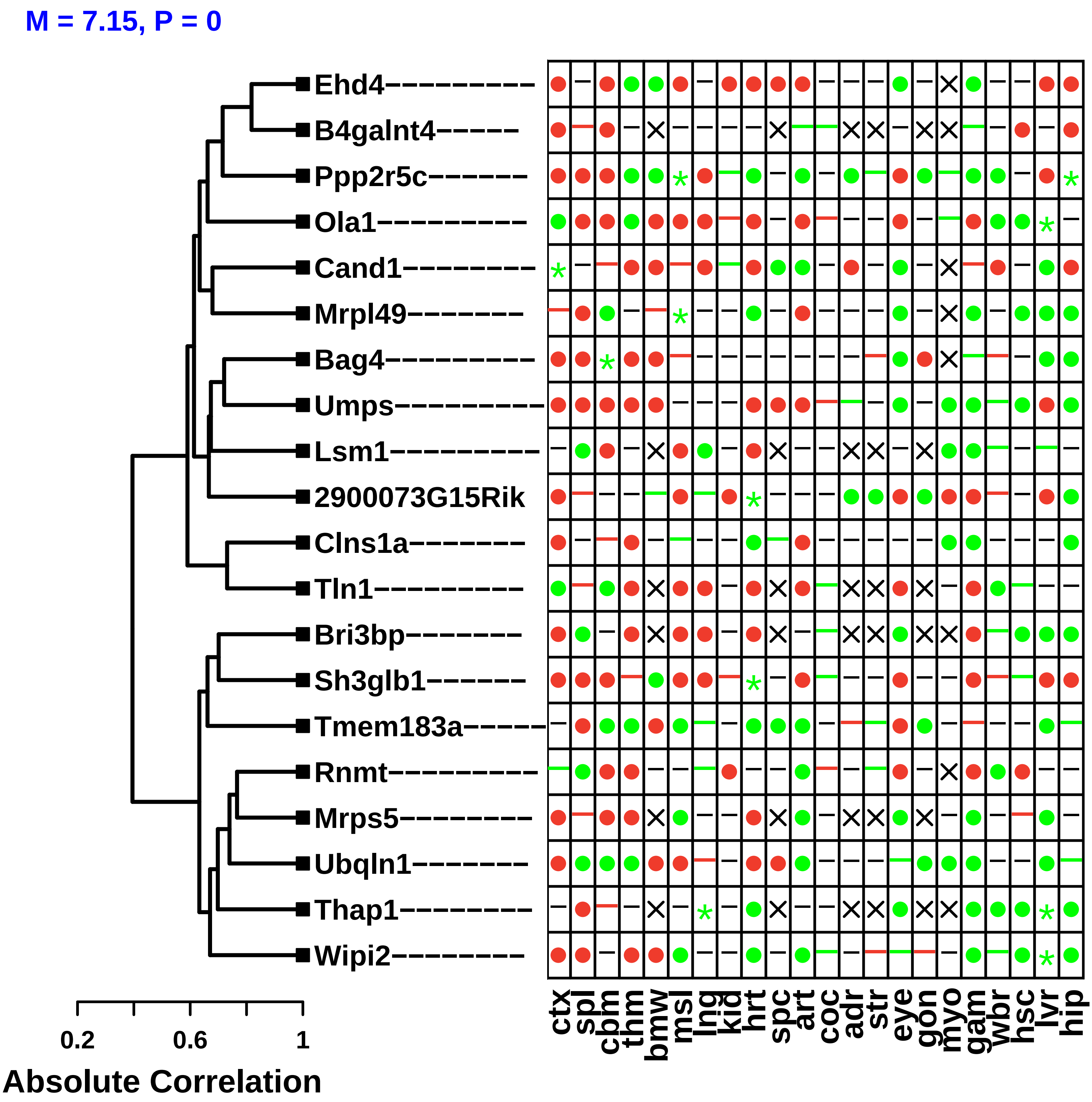

Age−Regulated Modules (20 Genes)

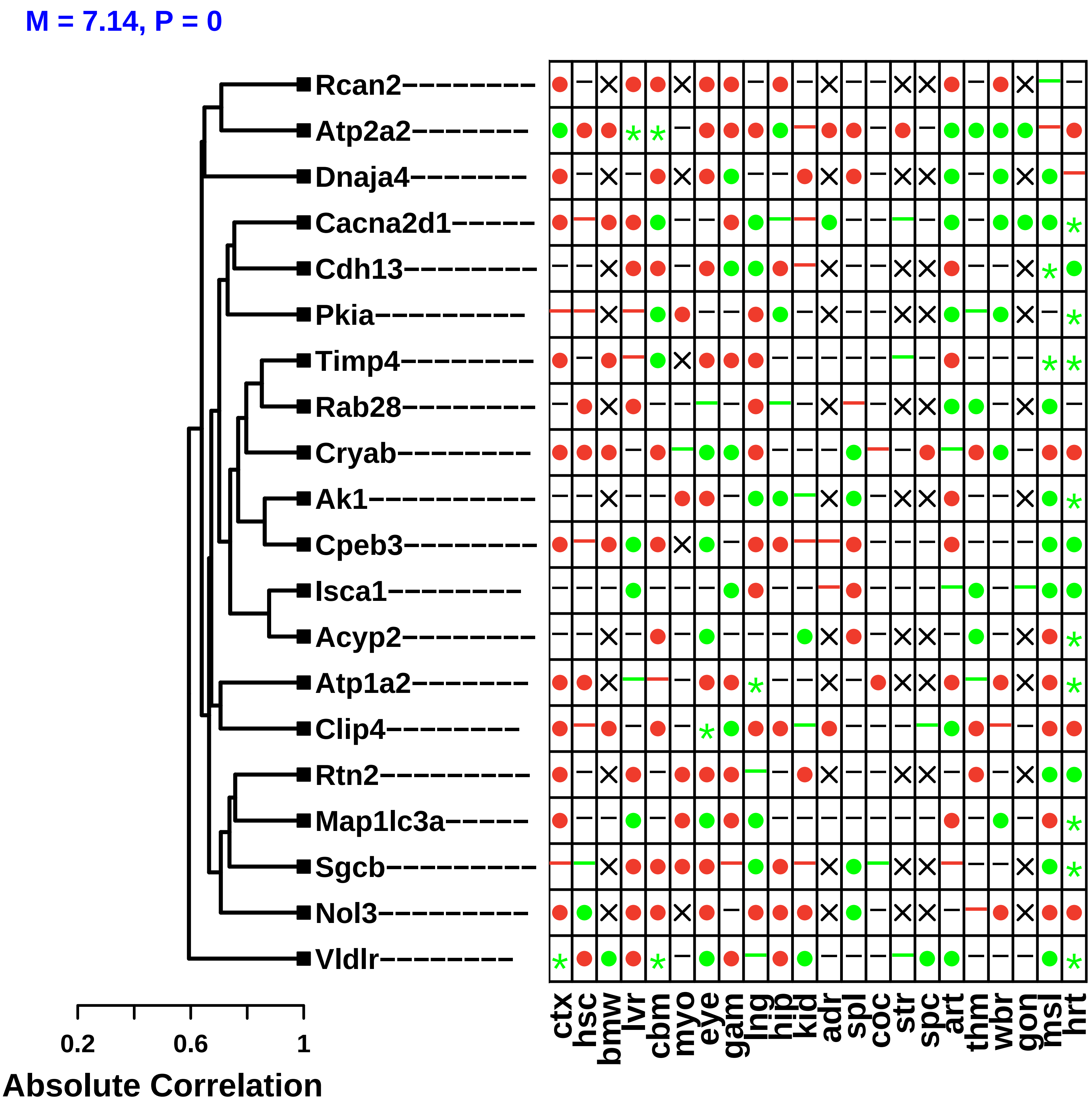

Age−Regulated Modules (20 Genes)

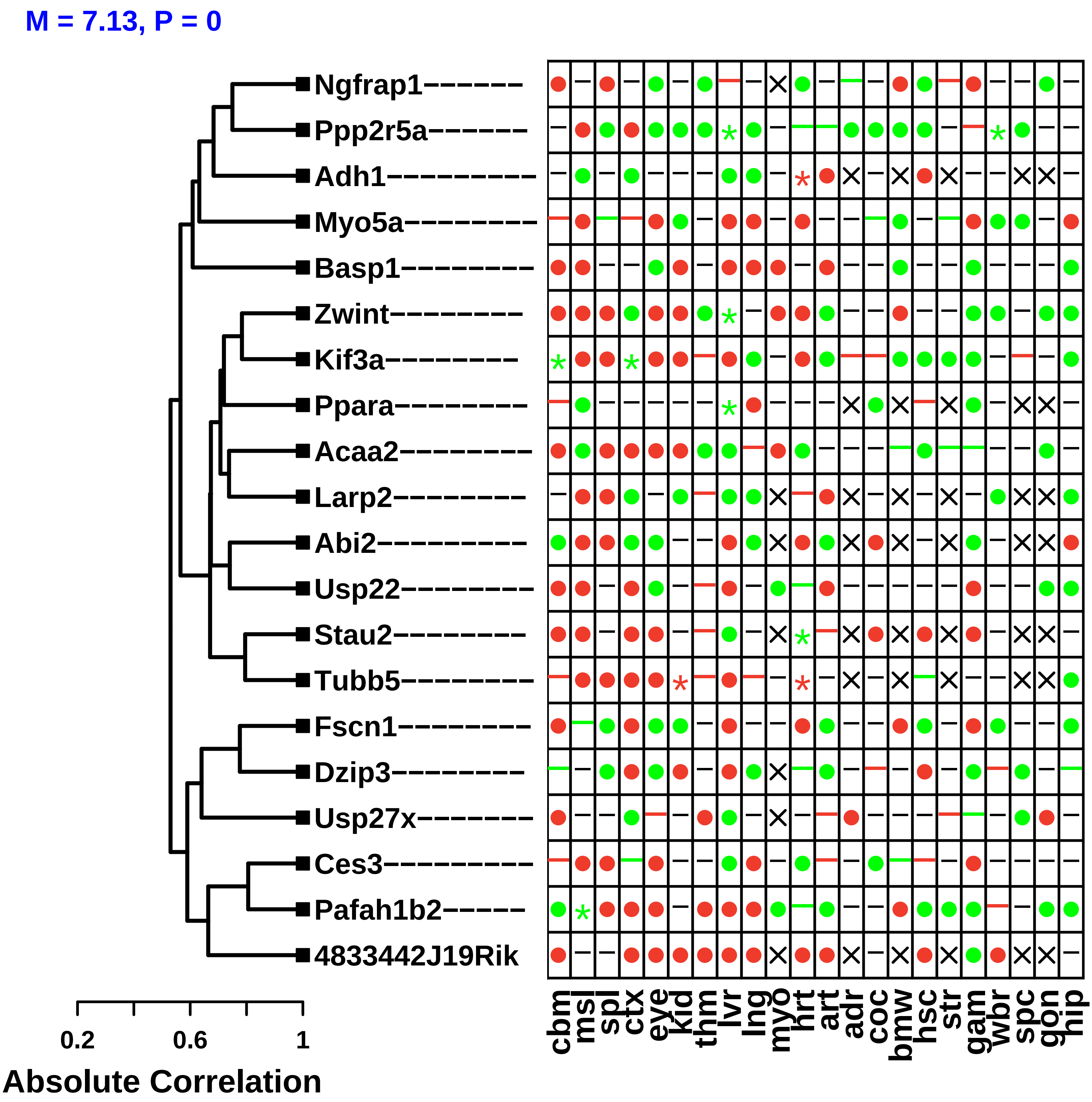

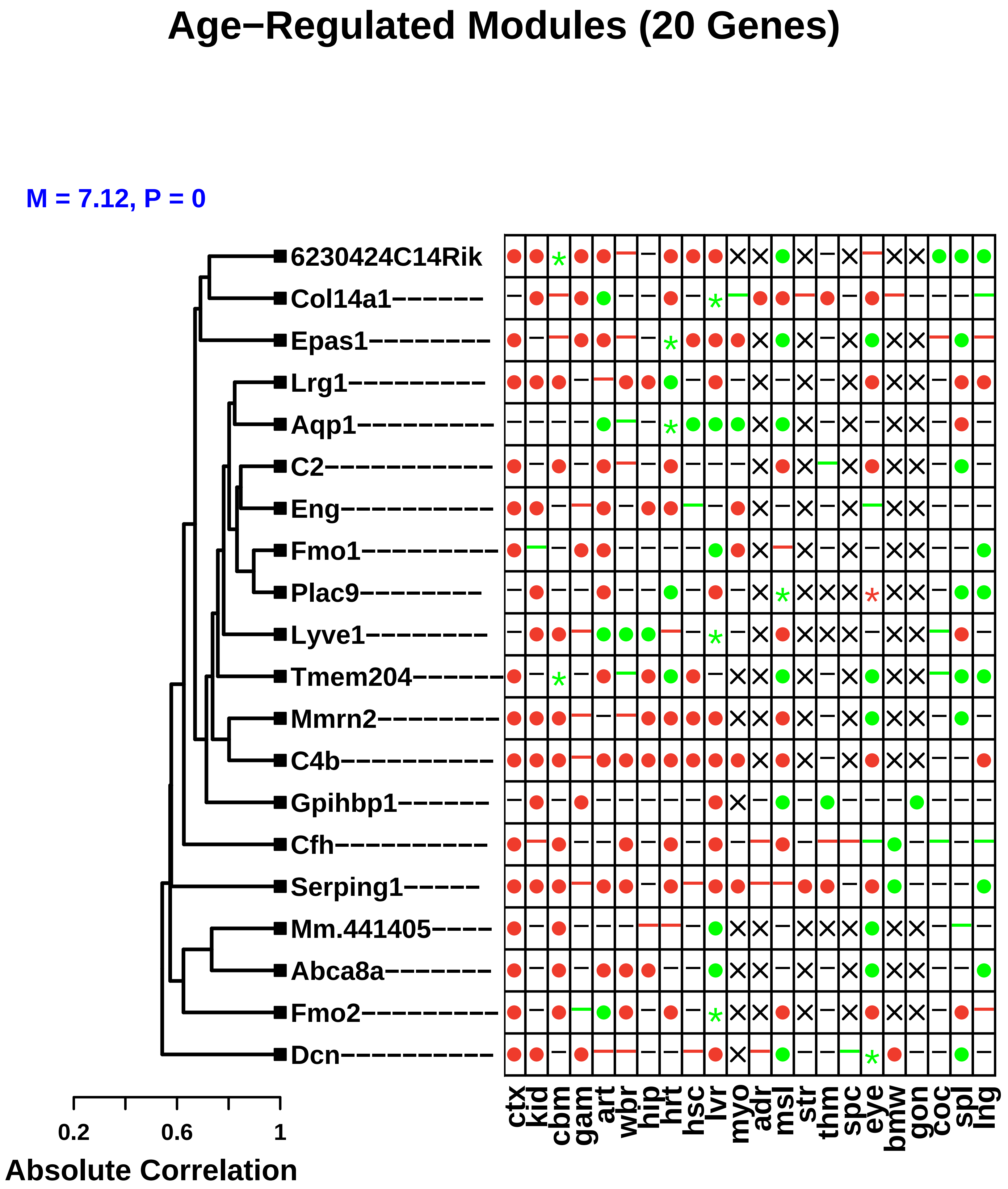

Age−Regulated Modules (20 Genes)
M = 7.12, P = 0
6230424C14Rik
Col14a1
Epas1
Lrg1
Aqp1
C2
Eng
Fmo1
Plac9
Lyve1
Tmem204
Mmrn2
C4b
Gpihbp1
Cfh
Serping1
Mm.441405
Abca8a
Fmo2
Dcn
ctx kid cbm gam art wbr hip hrt hsc lvr myo adr msl str thm spc eye bmw gon coc spl lng
0.2
0.6
1
Absolute Correlation

Age−Regulated Modules (20 Genes)

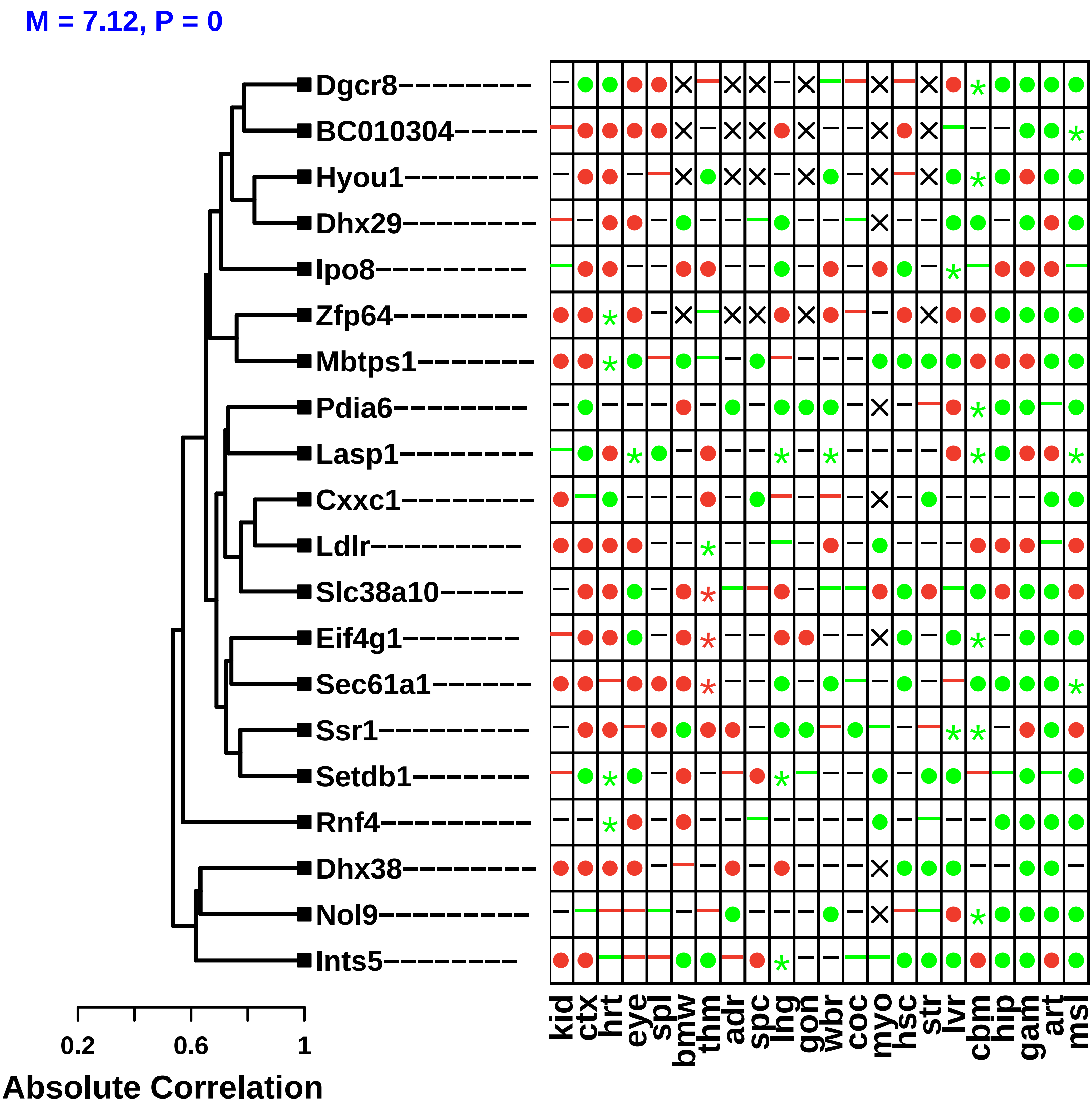

Age−Regulated Modules (20 Genes)

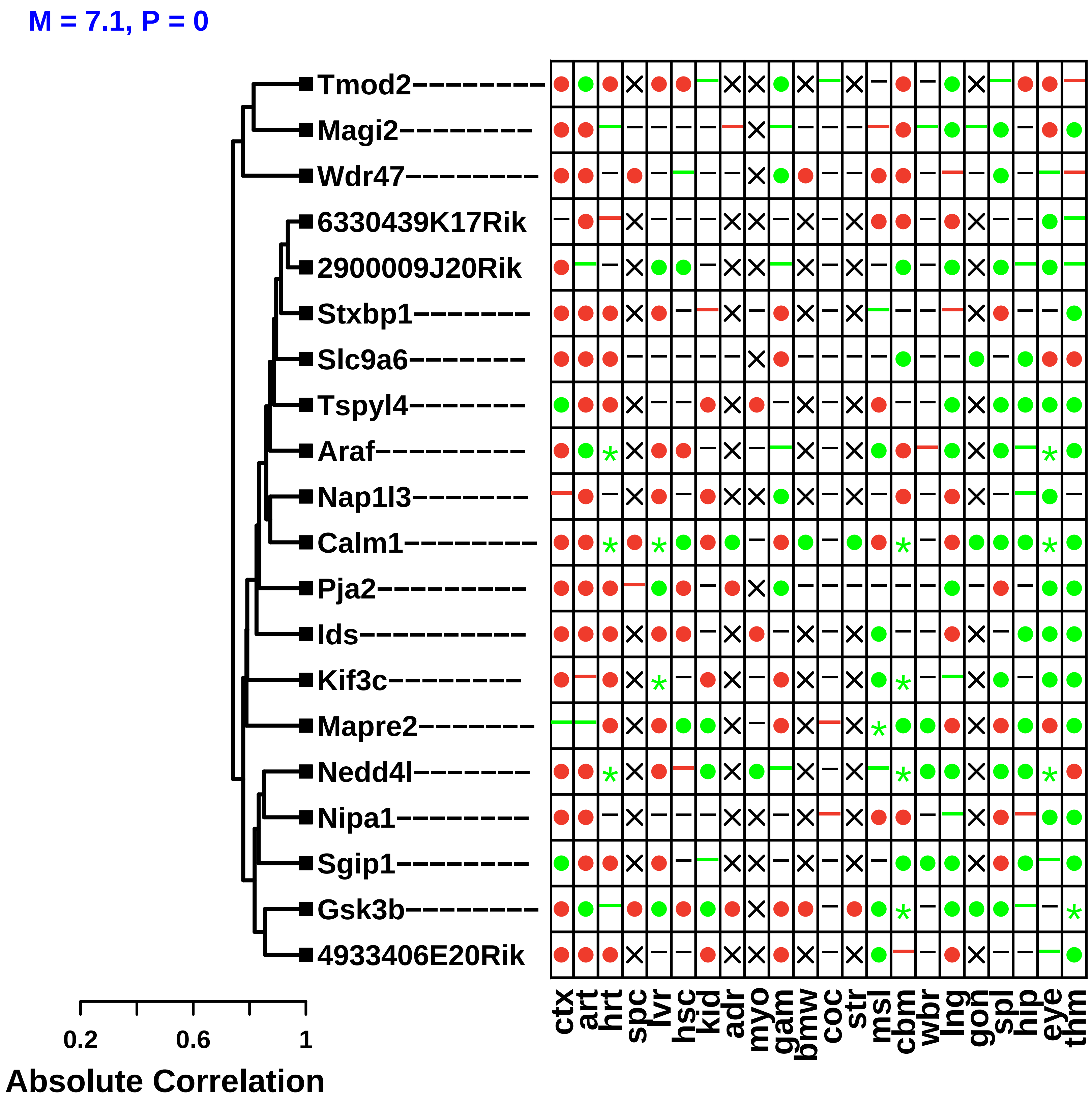

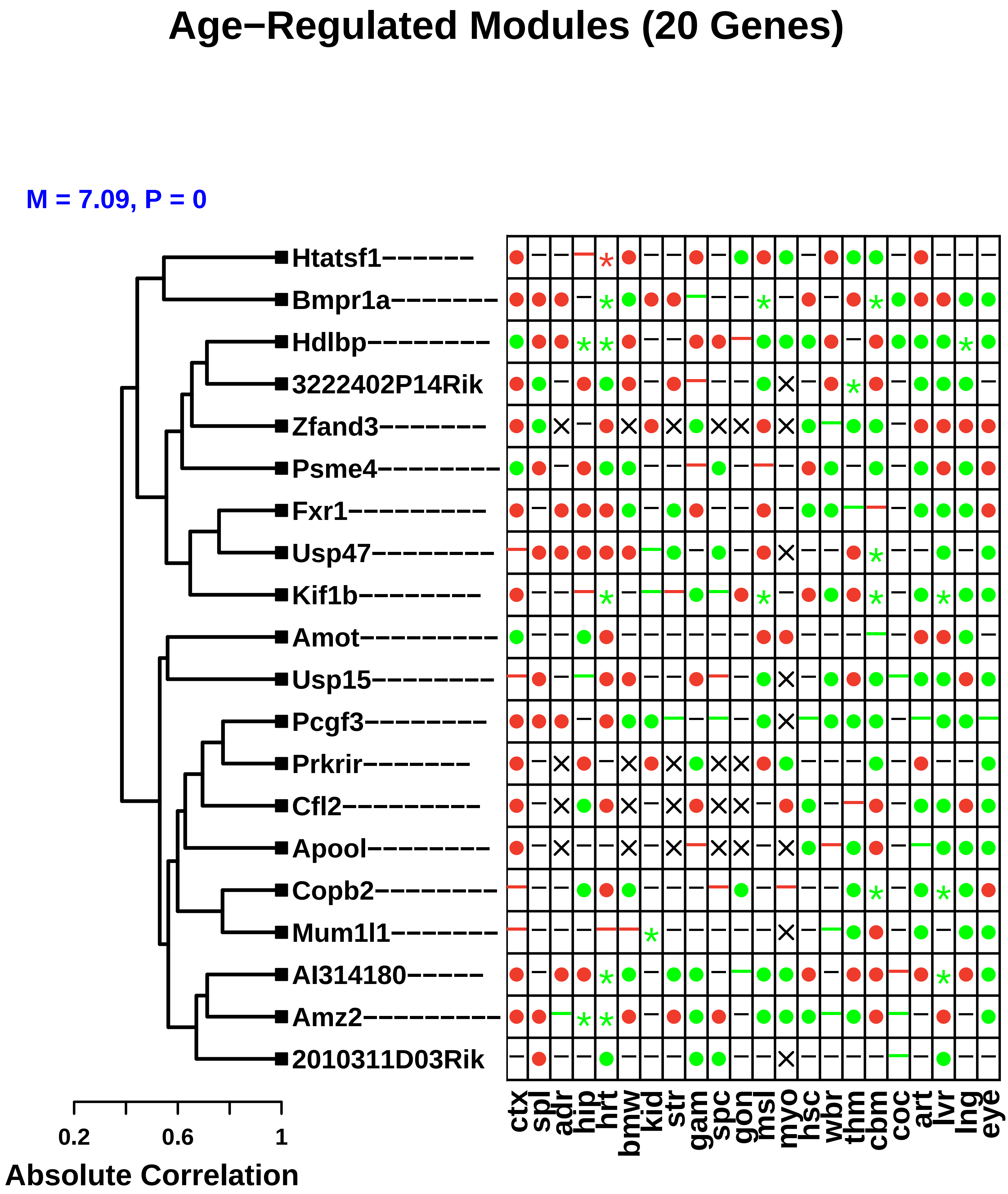
Age−Regulated Modules (20 Genes)
M = 7.09, P = 0
Htatsf1
Bmpr1a
Hdlbp
3222402P14Rik
Zfand3
Psme4
Fxr1
Usp47
Kif1b
Amot
Usp15
Pcgf3
Prkrir
Cfl2
Apool
Copb2
Mum1l1
AI314180
Amz2
2010311D03Rik
ctx
spl
adr
hip
hrt
bmw
kid
str
gam
spc
gon
msl
myo
hsc
wbr
thm
cbm
coc
art
lvr
lng
eye
0.2
0.6
1
Absolute Correlation

Age−Regulated Modules (20 Genes)

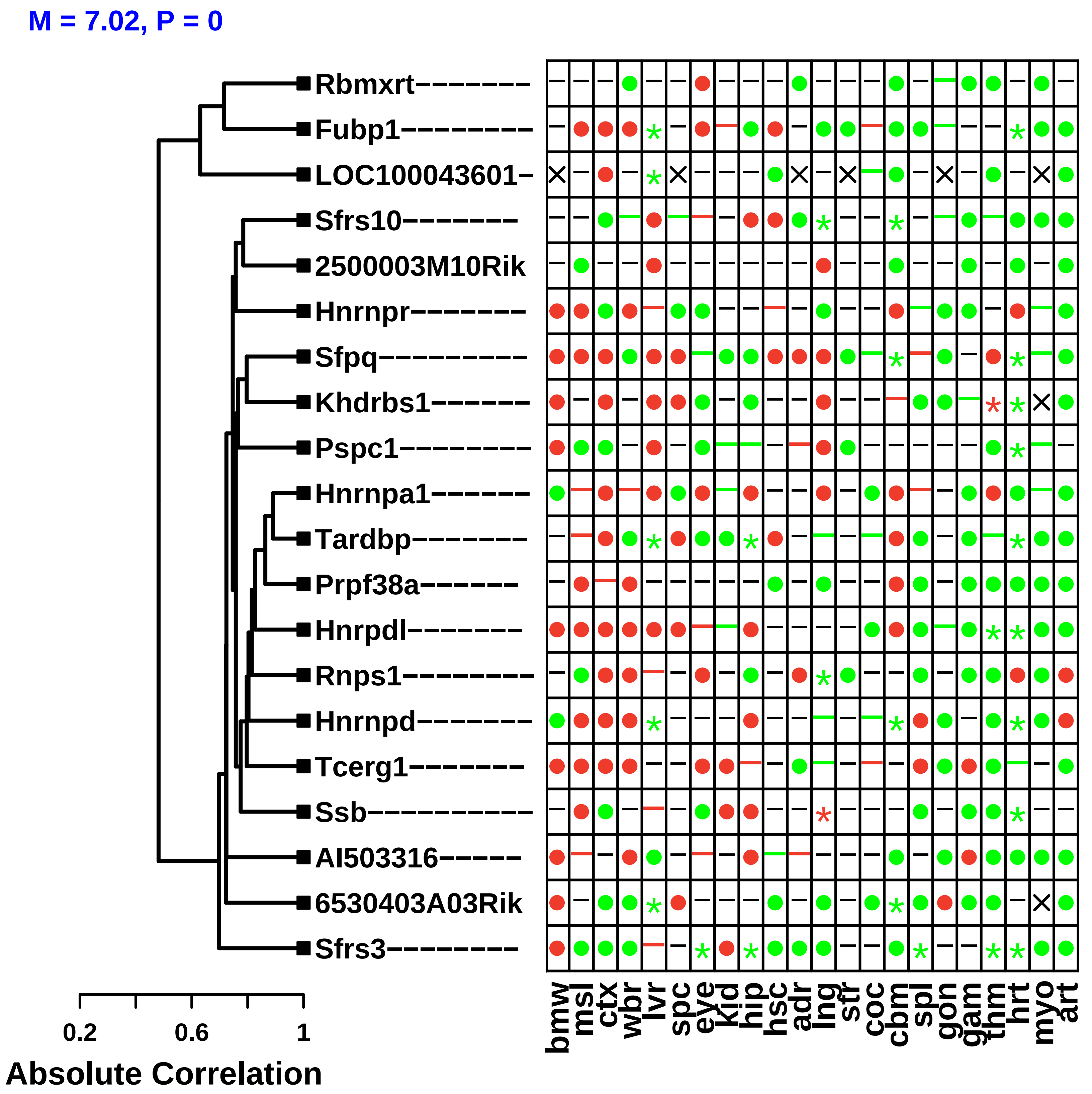

Age−Regulated Modules (20 Genes)

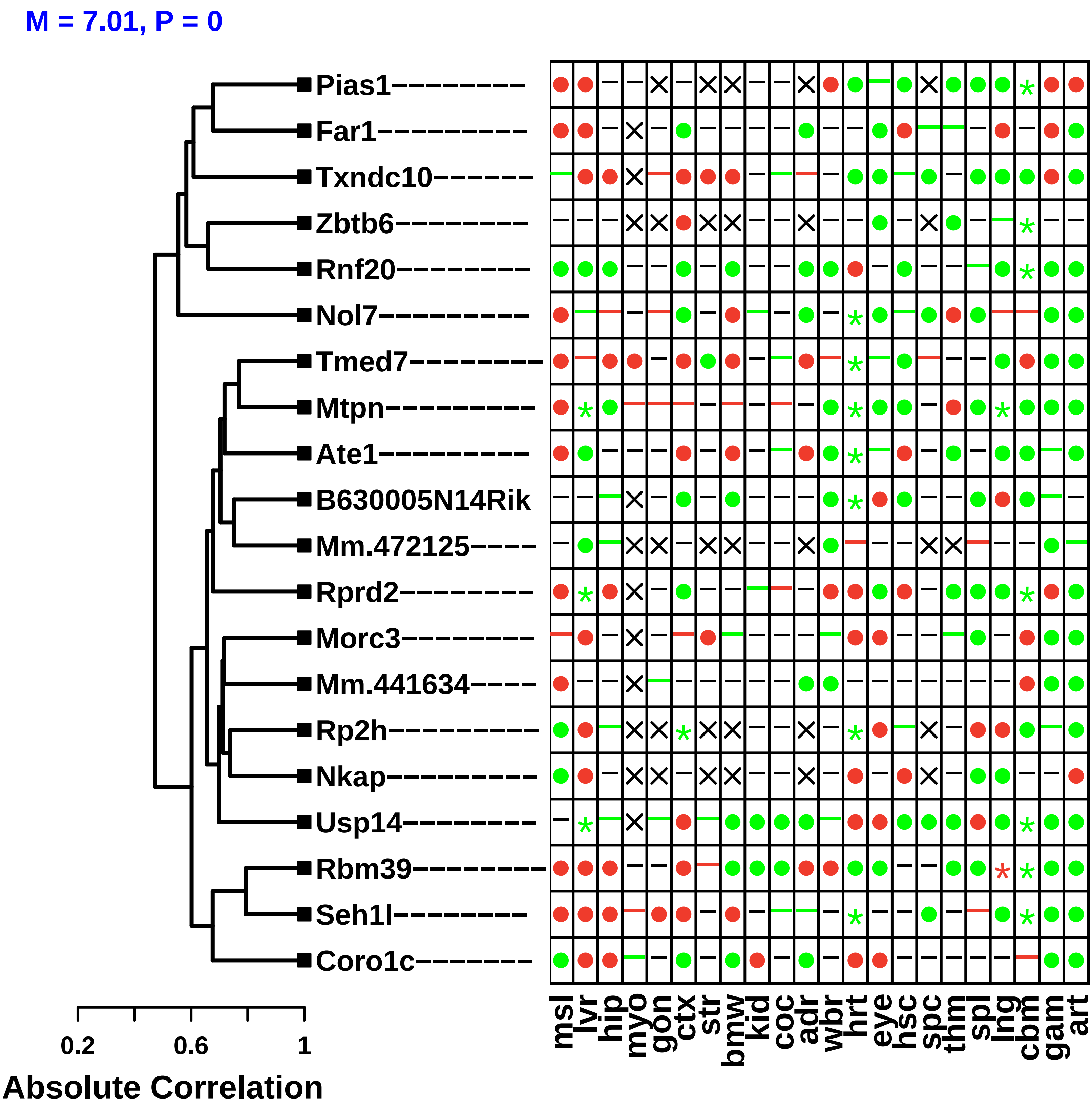

Age−Regulated Modules (20 Genes)

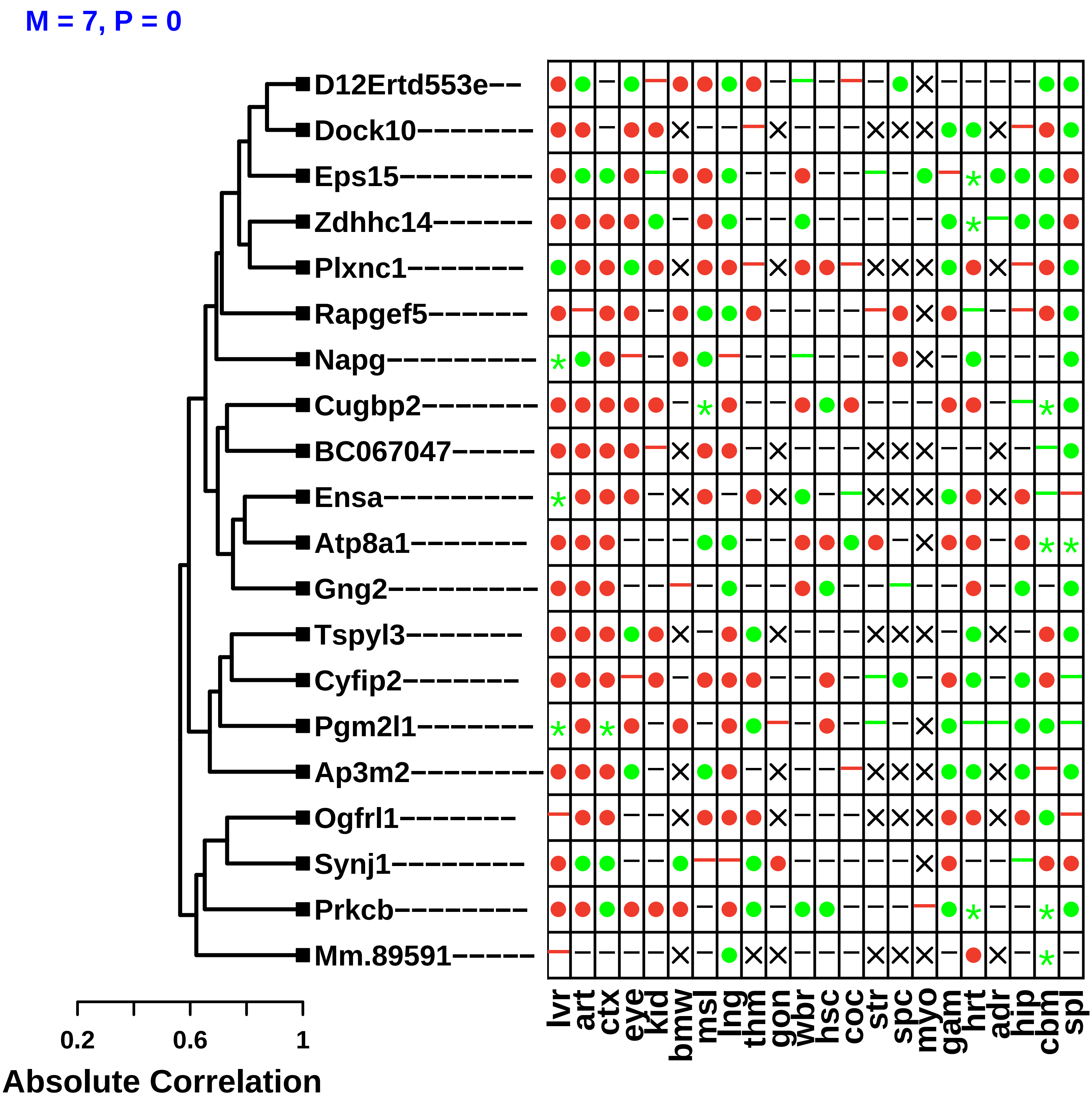

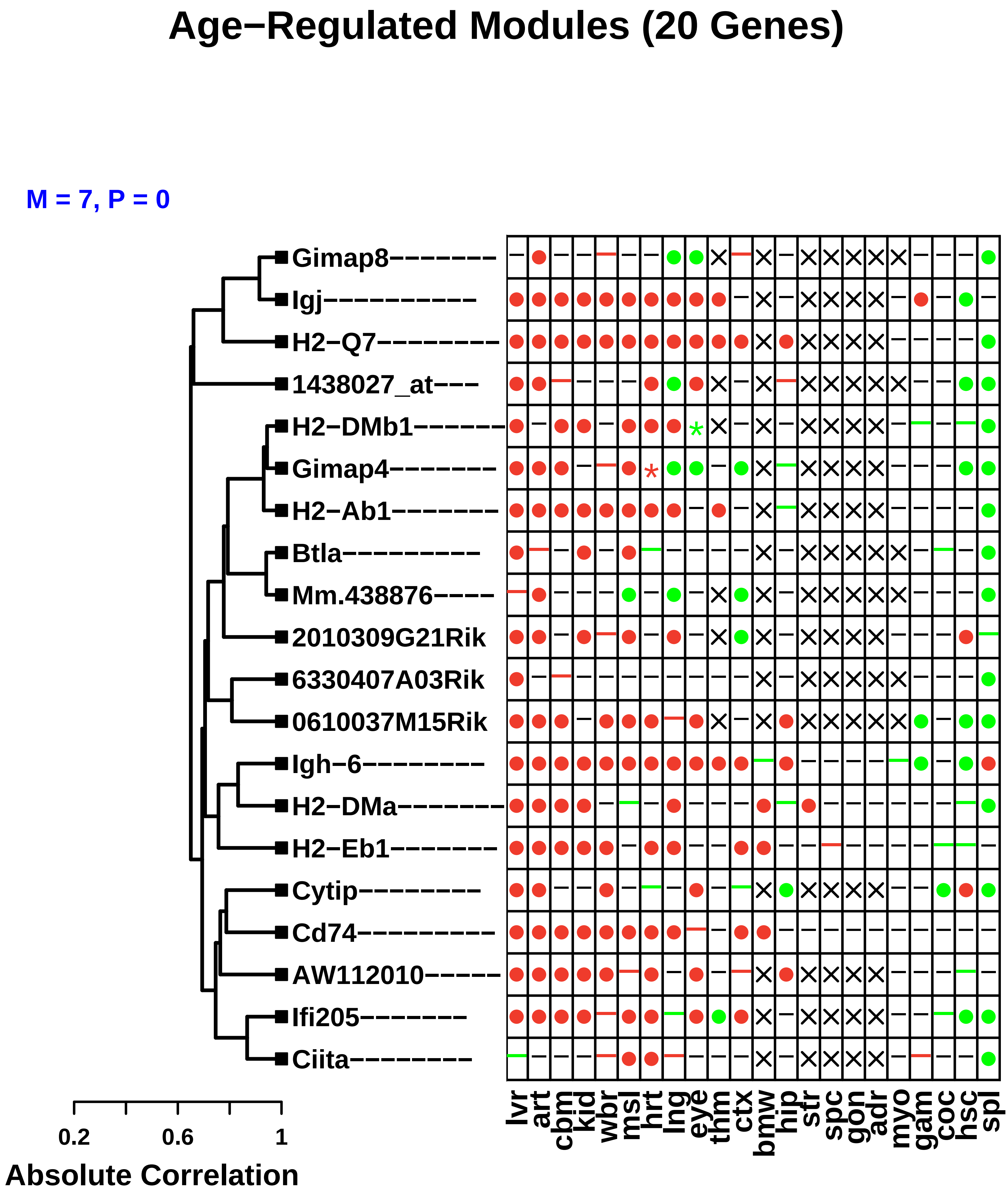
Age−Regulated Modules (20 Genes)
M = 7, P = 0
Gimap8
Igj
H2−Q7
1438027_at
H2−DMb1
Gimap4
H2−Ab1
Btla
Mm.438876
2010309G21Rik
6330407A03Rik
0610037M15Rik
Igh−6
H2−DMa
H2−Eb1
Cytip
Cd74
AW112010
Ifi205
Ciita
lvr
art
cbm
kid
wbr
msl
hrt
lng
eye
thm
ctx
bmw
hip
str
spc
gon
adr
myo
gam
coc
hsc
spl
0.2
0.6
1
Absolute Correlation

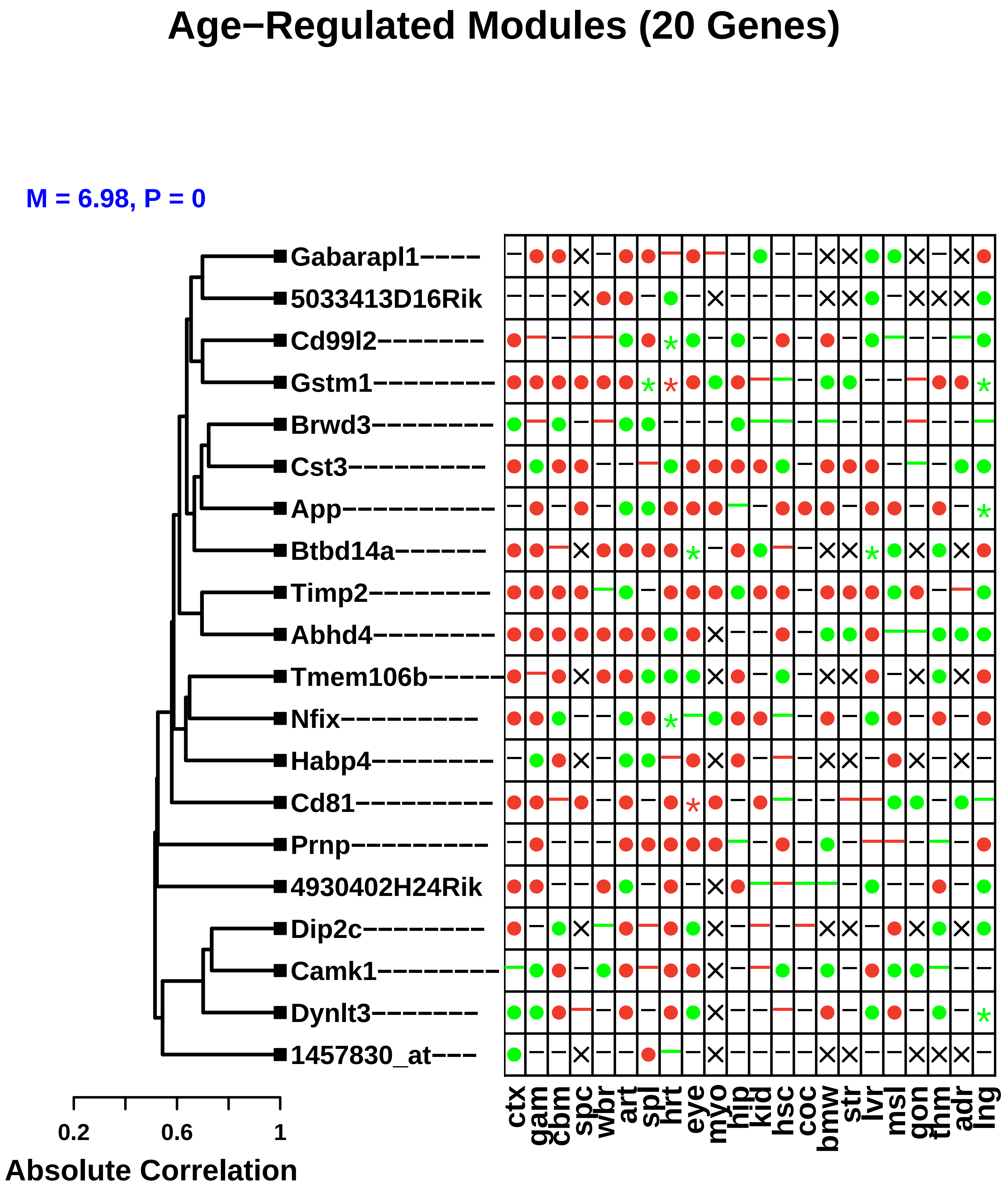
Age−Regulated Modules (20 Genes)
M = 6.98, P = 0
Gabarapl1
5033413D16Rik
Cd99l2
Gstm1
Brwd3
Cst3
App
Btbd14a
Timp2
Abhd4
Tmem106b
Nfix
Habp4
Cd81
Prnp
4930402H24Rik
Dip2c
Camk1
Dynlt3
1457830_at
ctx
gam
cbm
spc
wbr
art
spl
hrt
eye
myo
hip
kid
hsc
coc
bmw
str
lvr
msl
gon
thm
adr
lng
0.2
0.6
1
Absolute Correlation

Age−Regulated Modules (20 Genes)

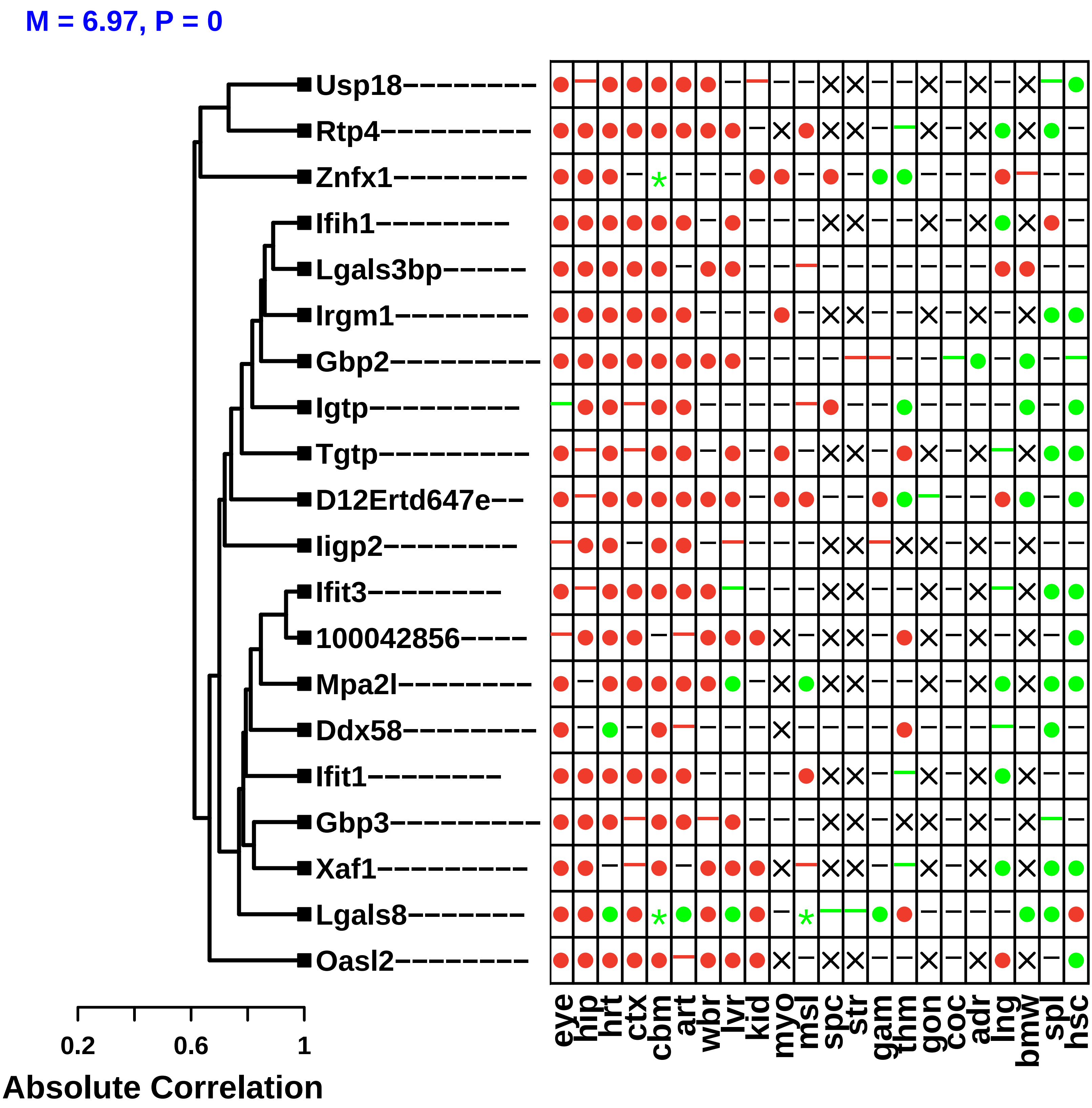

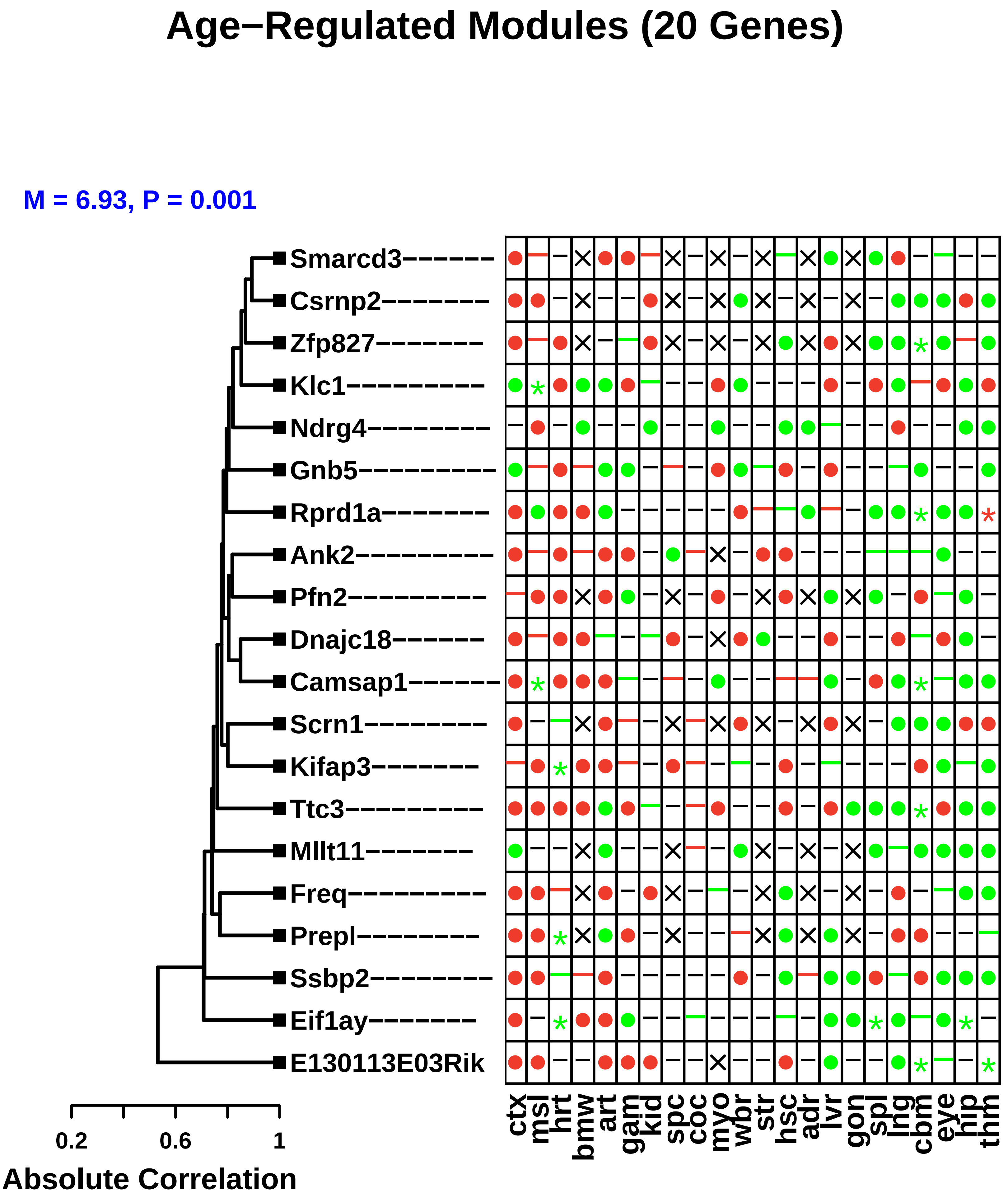
Age−Regulated Modules (20 Genes)
M = 6.93, P = 0.001
Smarcd3
Csrnp2
Zfp827
Klc1
Ndrg4
Gnb5
Rprd1a
Ank2
Pfn2
Dnajc18
Camsap1
Scrn1
Kifap3
Ttc3
Mllt11
Freq
Prepl
Ssbp2
Eif1ay
E130113E03Rik
ctx
msl
hrt
bmw
art
gam
kid
spc
coc
myo
wbr
str
hsc
adr
lvr
gon
spl
lng
cbm
eye
hip
thm
0.2
0.6
1
Absolute Correlation

Age−Regulated Modules (20 Genes)

M = 6.93, P = 0.001

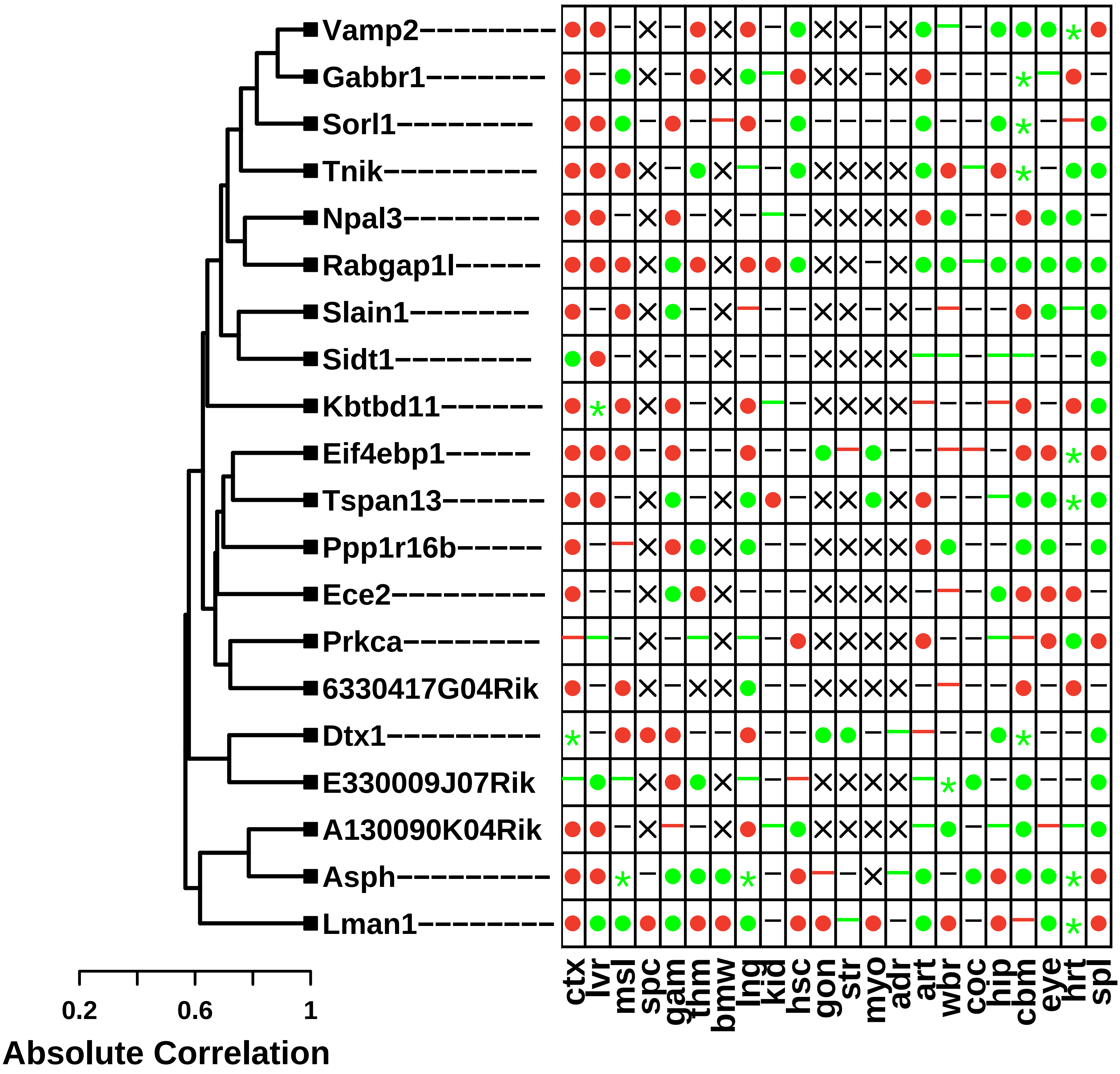

Age−Regulated Modules (20 Genes)

M = 6.91, P = 0.001

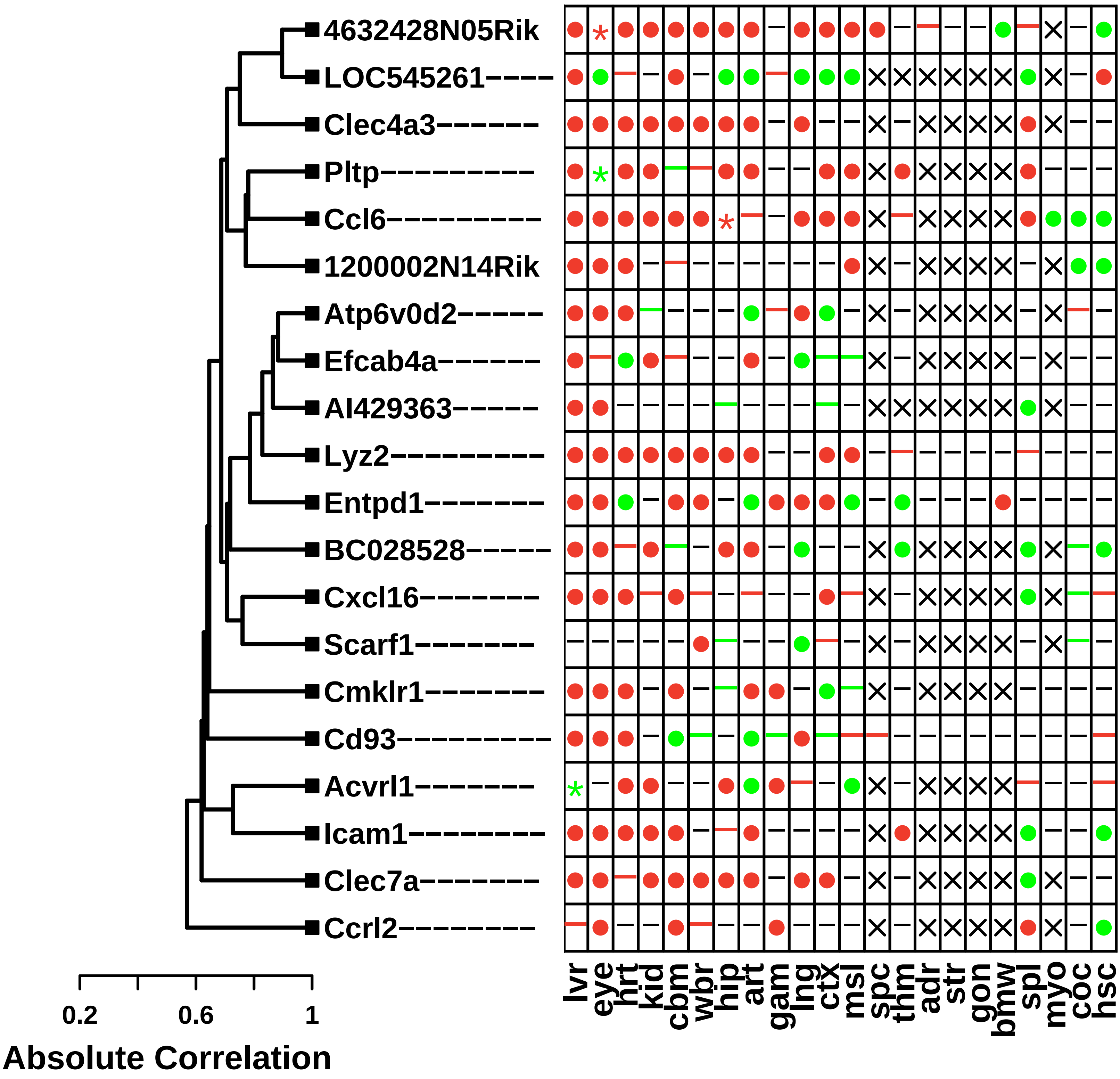

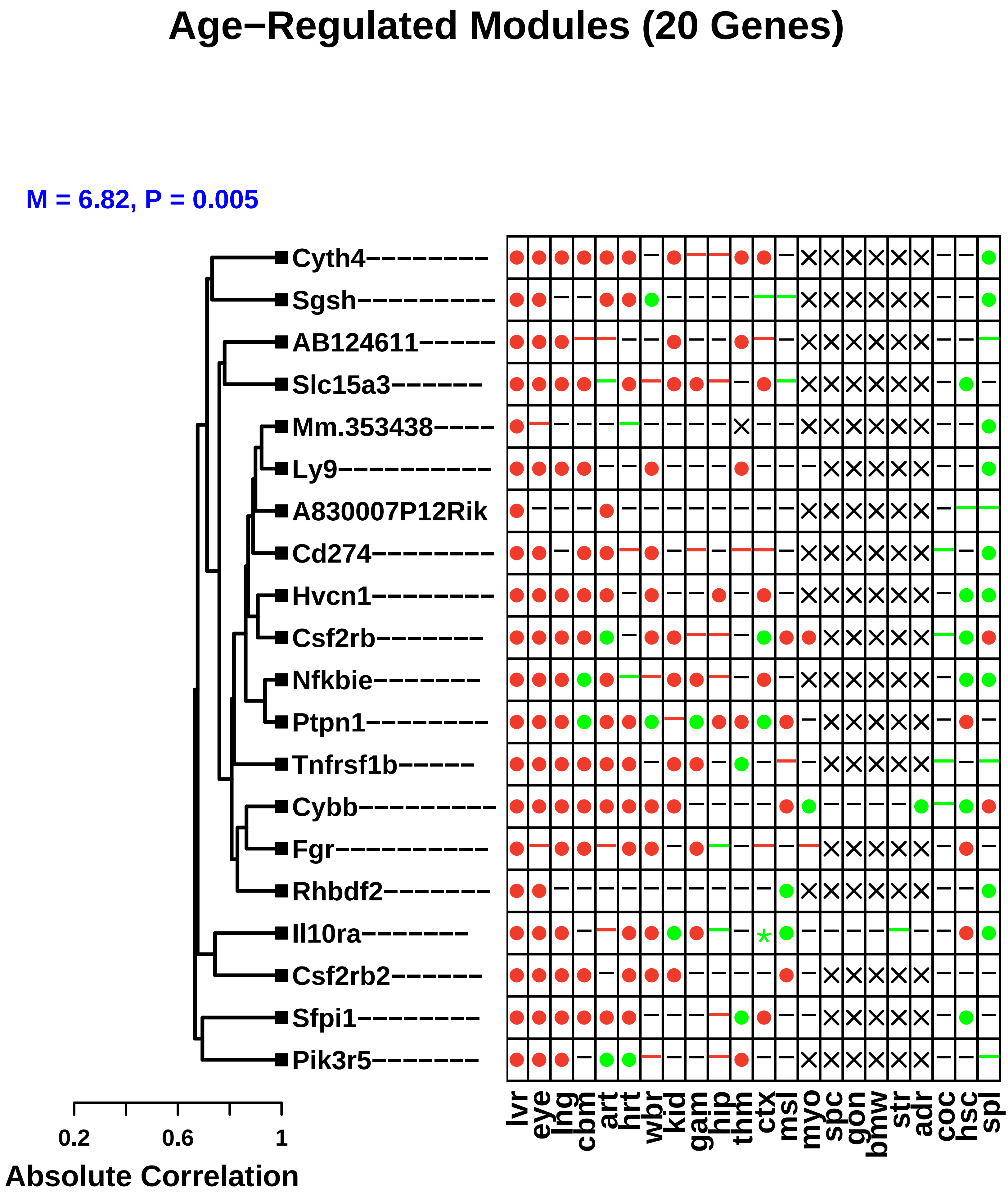
Age−Regulated Modules (20 Genes)
M = 6.82, P = 0.005
Cyth4
Sgsh
AB124611
Slc15a3
Mm.353438
Ly9
A830007P12Rik
Cd274
Hvcn1
Csf2rb
Nfkbie
Ptpn1
Tnfrsf1b
Cybb
Fgr
Rhbdf2
Il10ra
Csf2rb2
Sfpi1
Pik3r5
lvr
eye
lng
cbm
art
hrt
wbr
kid
gam
hip
thm
ctx
msl
myo
spc
gon
bmw
str
adr
coc
hsc
spl
0.2
0.6
1
Absolute Correlation

Age−Regulated Modules (20 Genes)

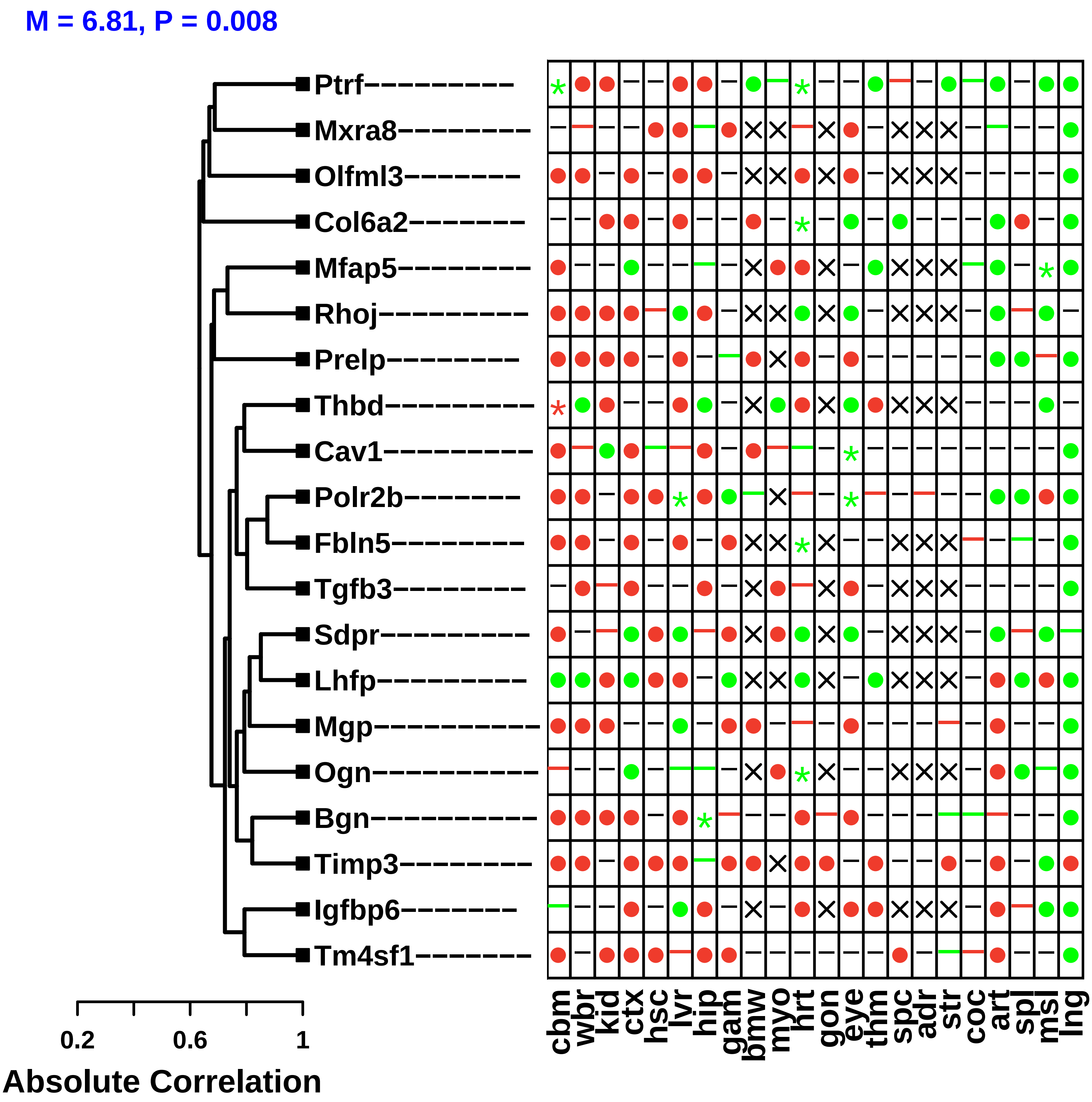

Age−Regulated Modules (20 Genes)

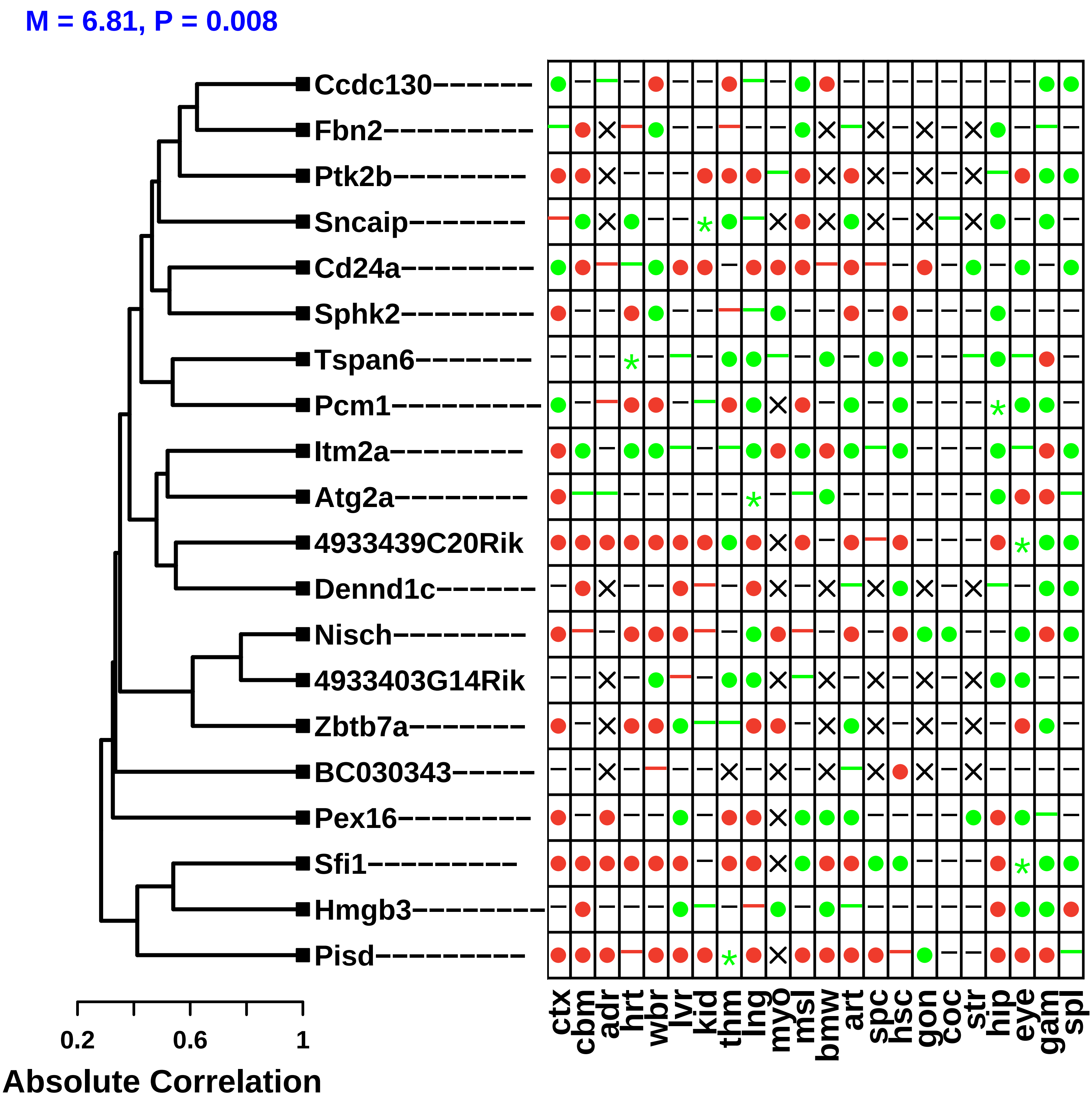

Age−Regulated Modules (20 Genes)

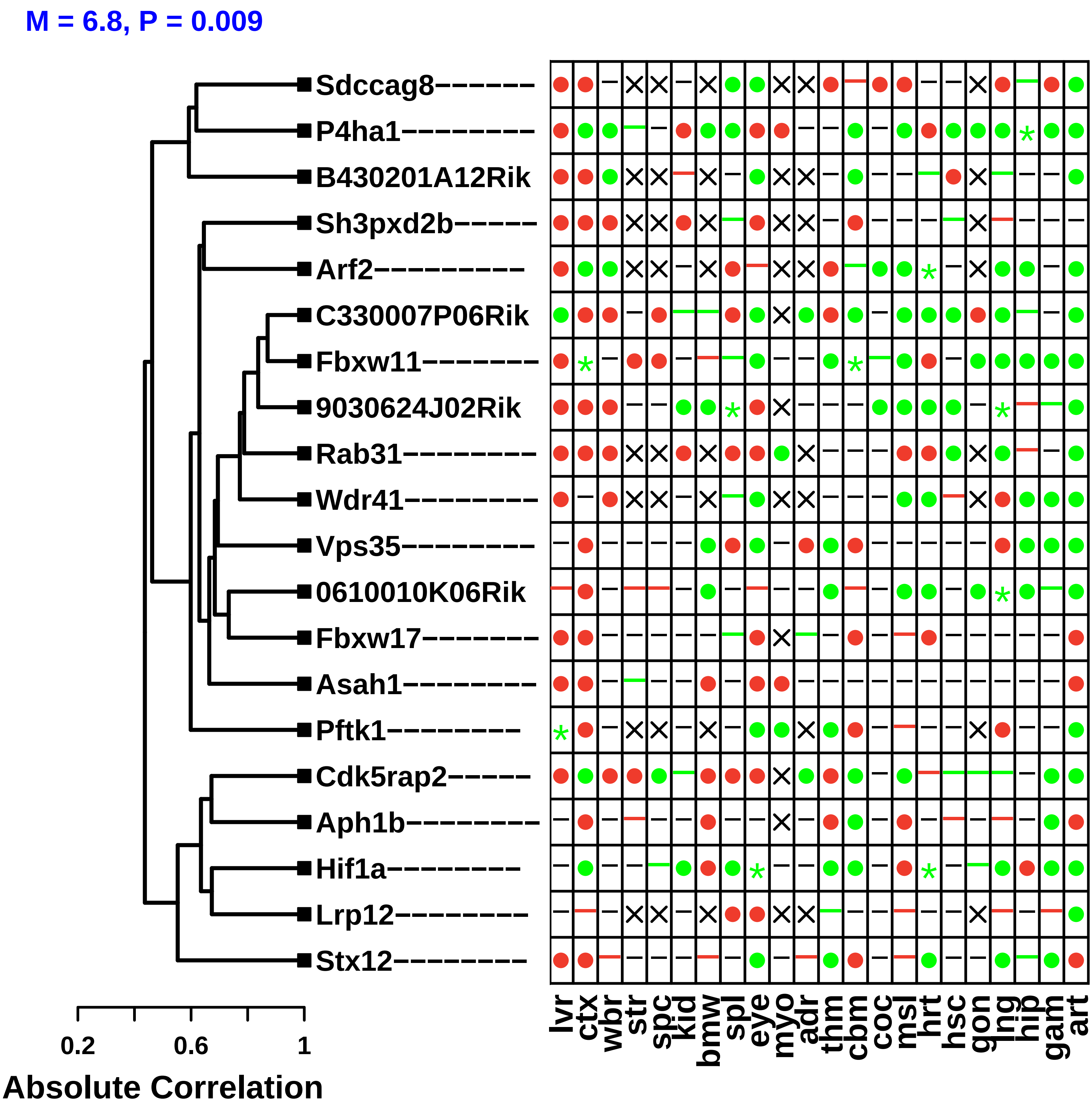

Age−Regulated Modules (20 Genes)

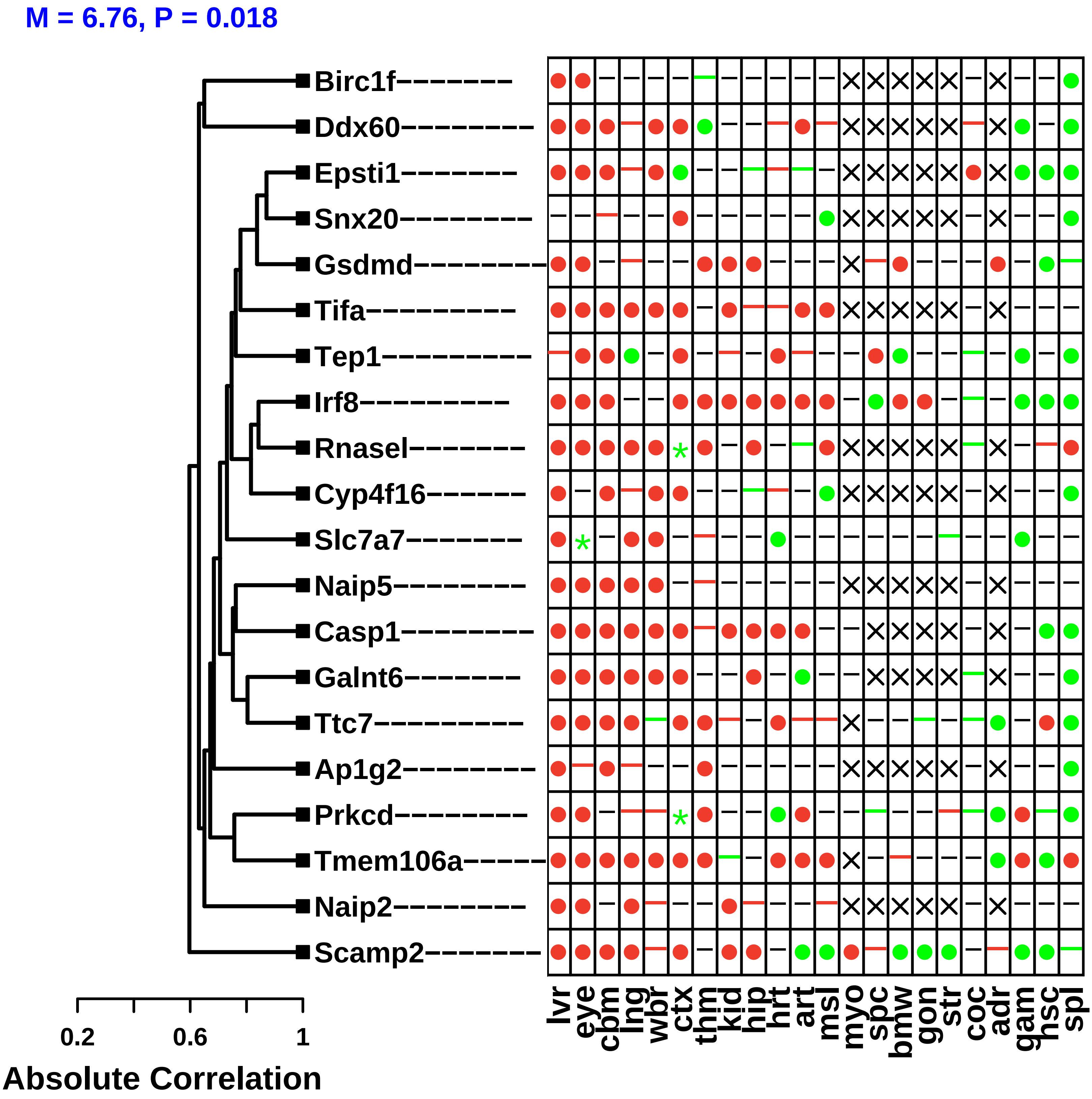

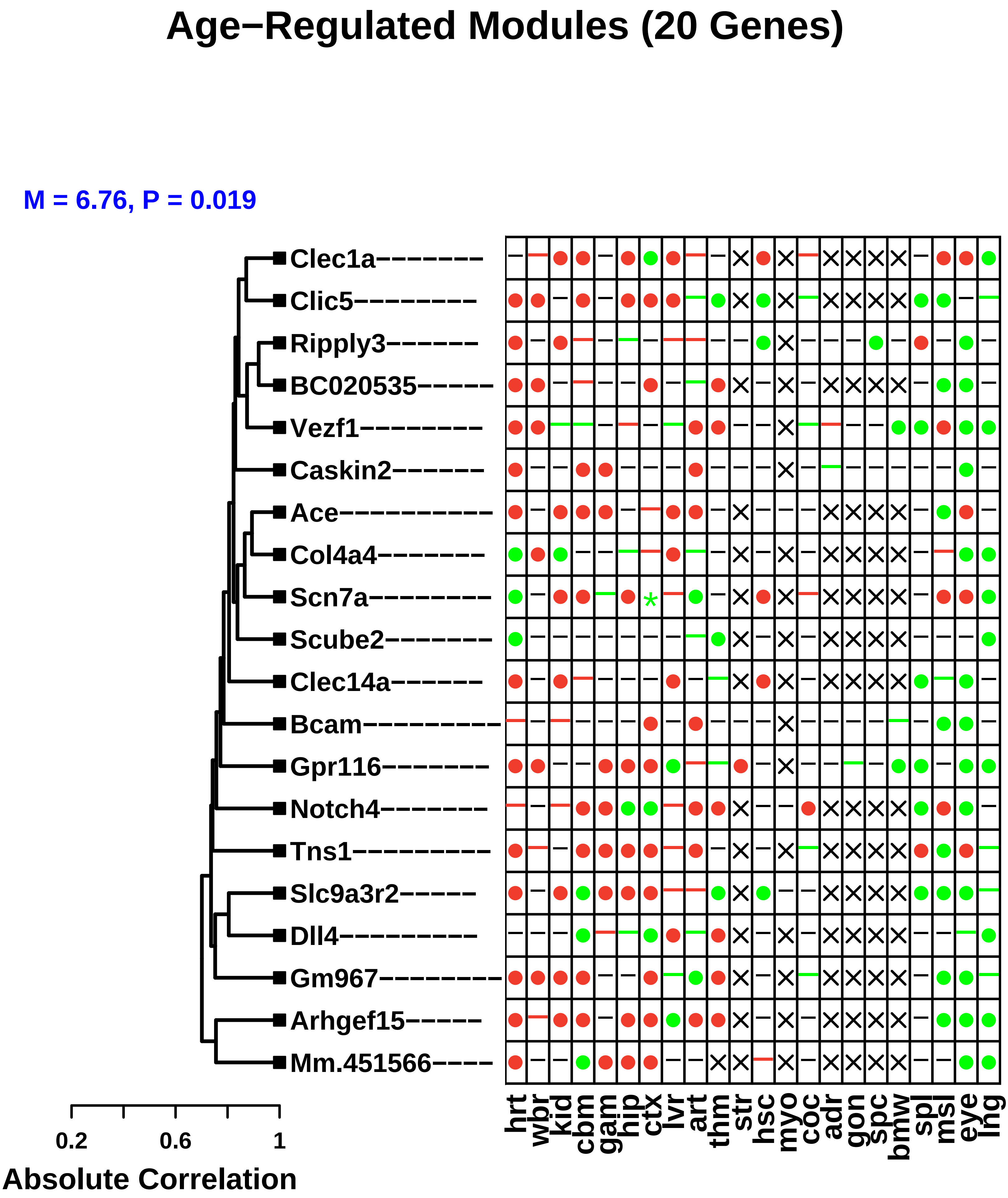
Age−Regulated Modules (20 Genes)
M = 6.76, P = 0.019
Clec1a
Clic5
Ripply3
BC020535
Vezf1
Caskin2
Ace
Col4a4
Scn7a
Scube2
Clec14a
Bcam
Gpr116
Notch4
Tns1
Slc9a3r2
Dll4
Gm967
Arhgef15
Mm.451566
hrt
wbr
kid
cbm
gam
hip
ctx
lvr
art
thm
str
hsc
myo
coc
adr
gon
spc
bmw
spl
msl
eye
lng
0.2
0.6
1
Absolute Correlation

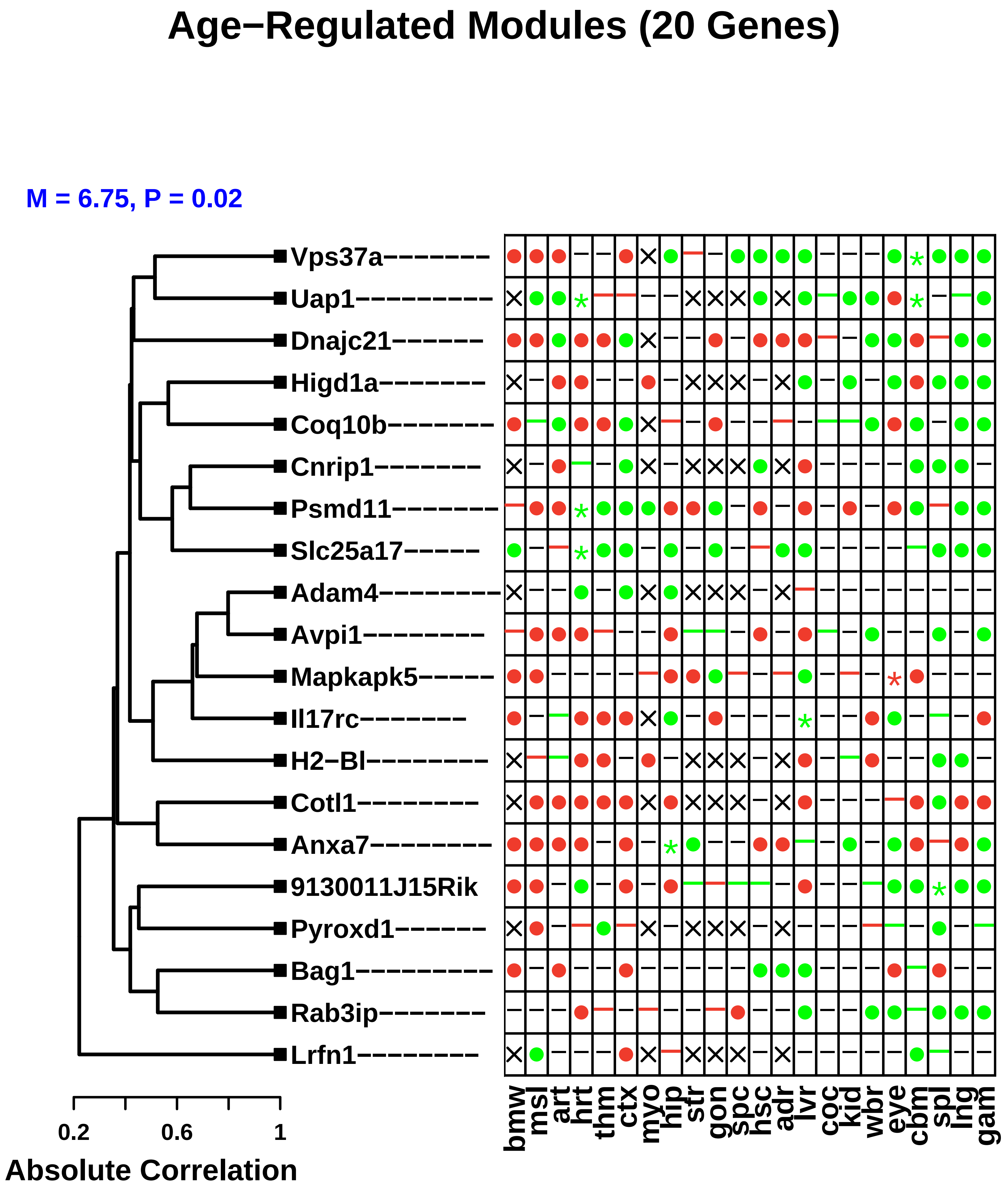

Age−Regulated Modules (20 Genes)
M = 6.75, P = 0.02
Vps37a
Uap1
Dnajc21
Higd1a
Coq10b
Cnrip1
Psmd11
Slc25a17
Adam4
Avpi1
Mapkapk5
Il17rc
H2−Bl
Cotl1
Anxa7
9130011J15Rik
Pyroxd1
Bag1
Rab3ip
Lrfn1
0.2
0.6
1
Absolute Correlation
bmw
msl
art
hrt
thm
ctx
myo
hip
str
gon
spc
hsc
adr
lvr
coc
kid
wbr
eye
cbm
spl
lng
gam

Age−Regulated Modules (20 Genes)

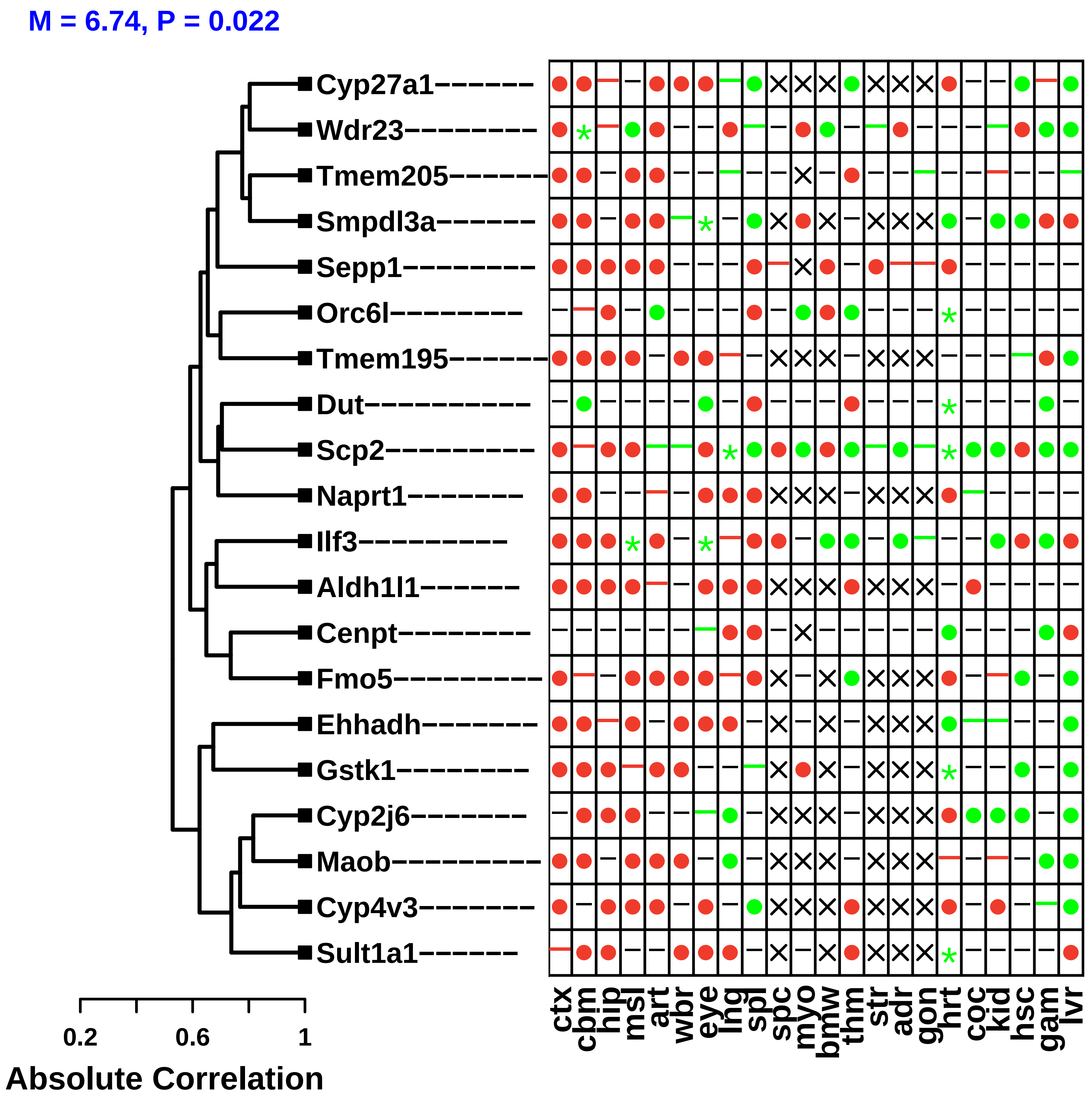

Age−Regulated Modules (20 Genes)

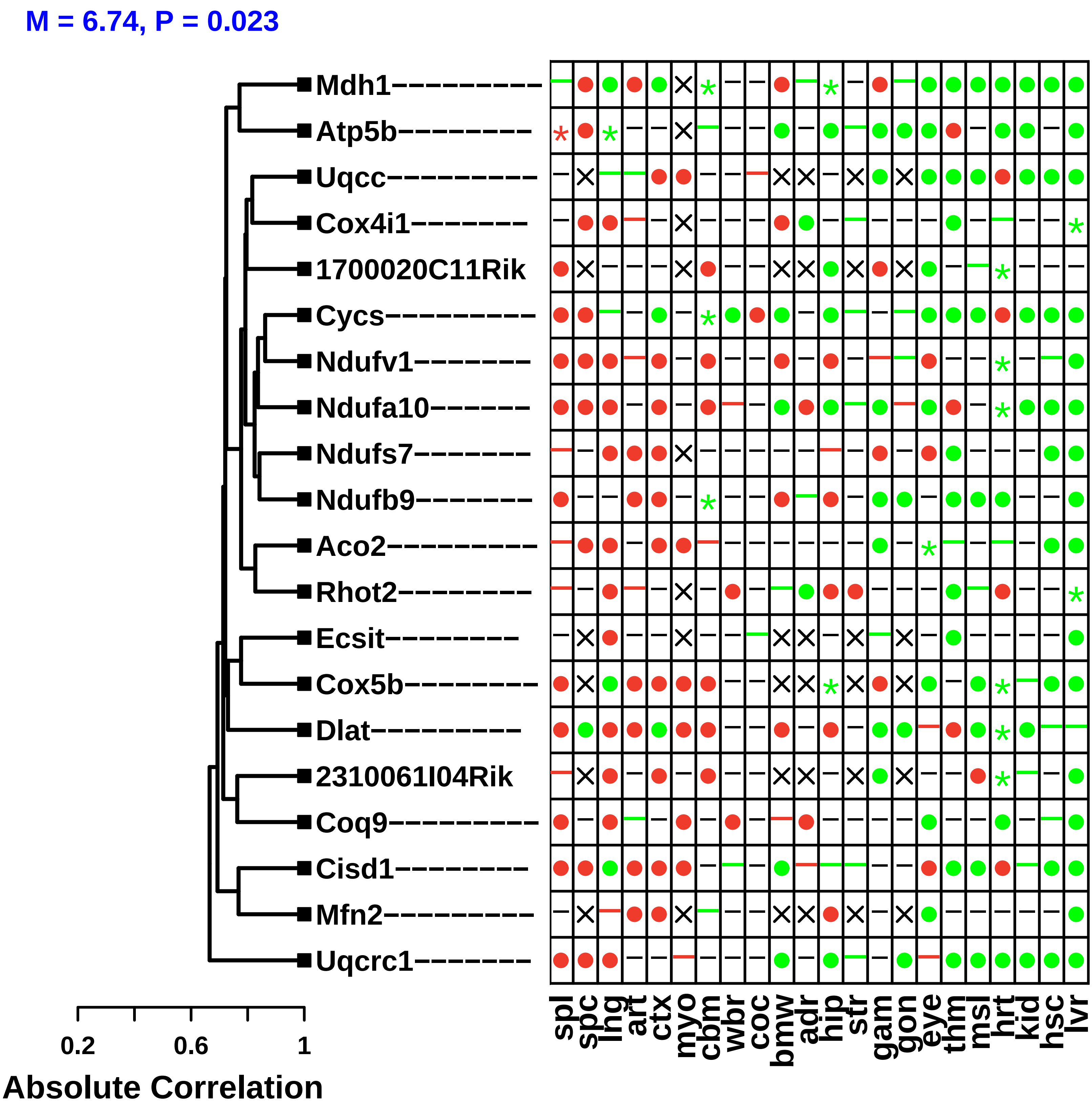

Age−Regulated Modules (20 Genes)

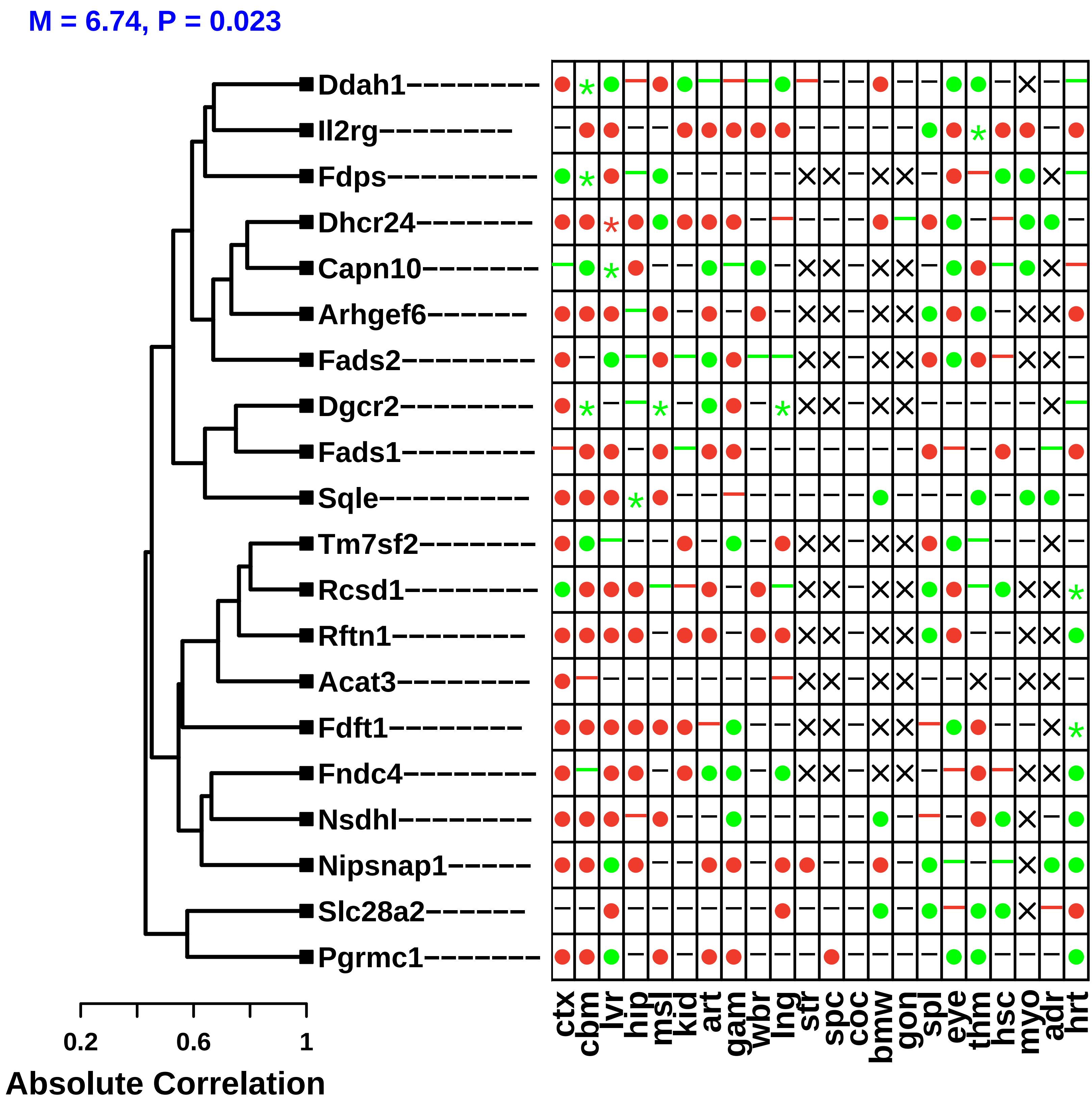

Age−Regulated Modules (20 Genes)

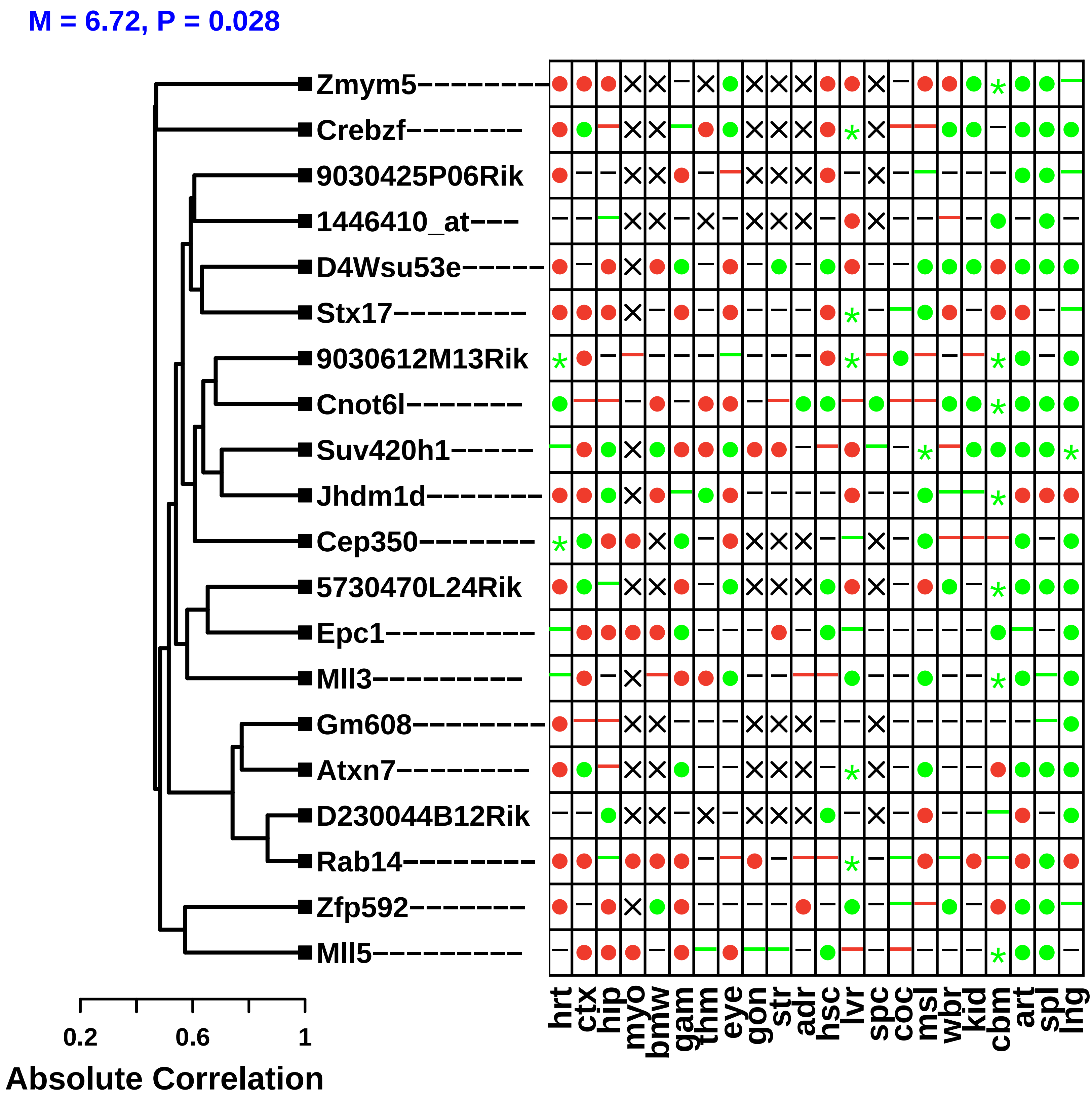

Age−Regulated Modules (20 Genes)

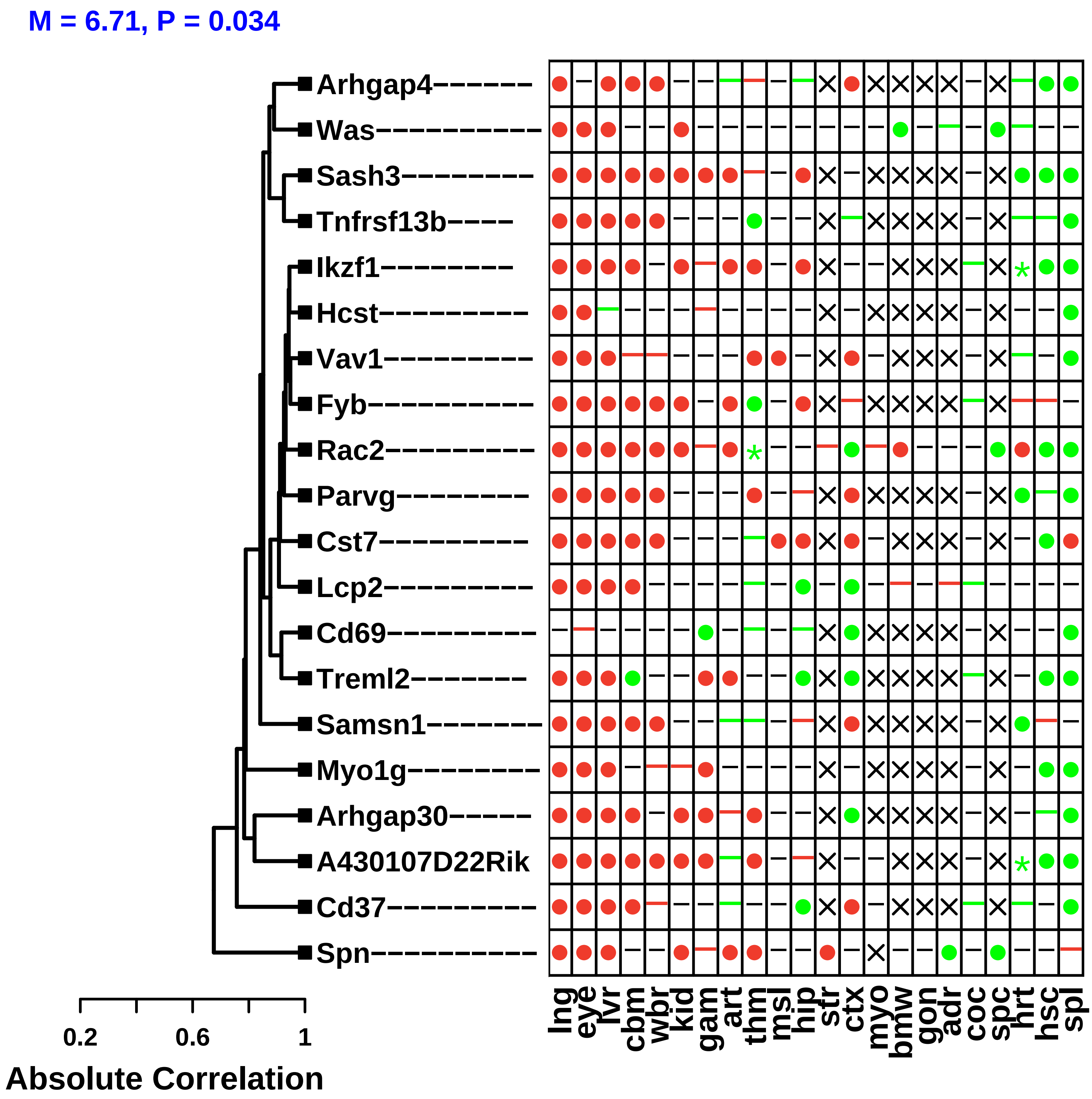

Age−Regulated Modules (20 Genes)

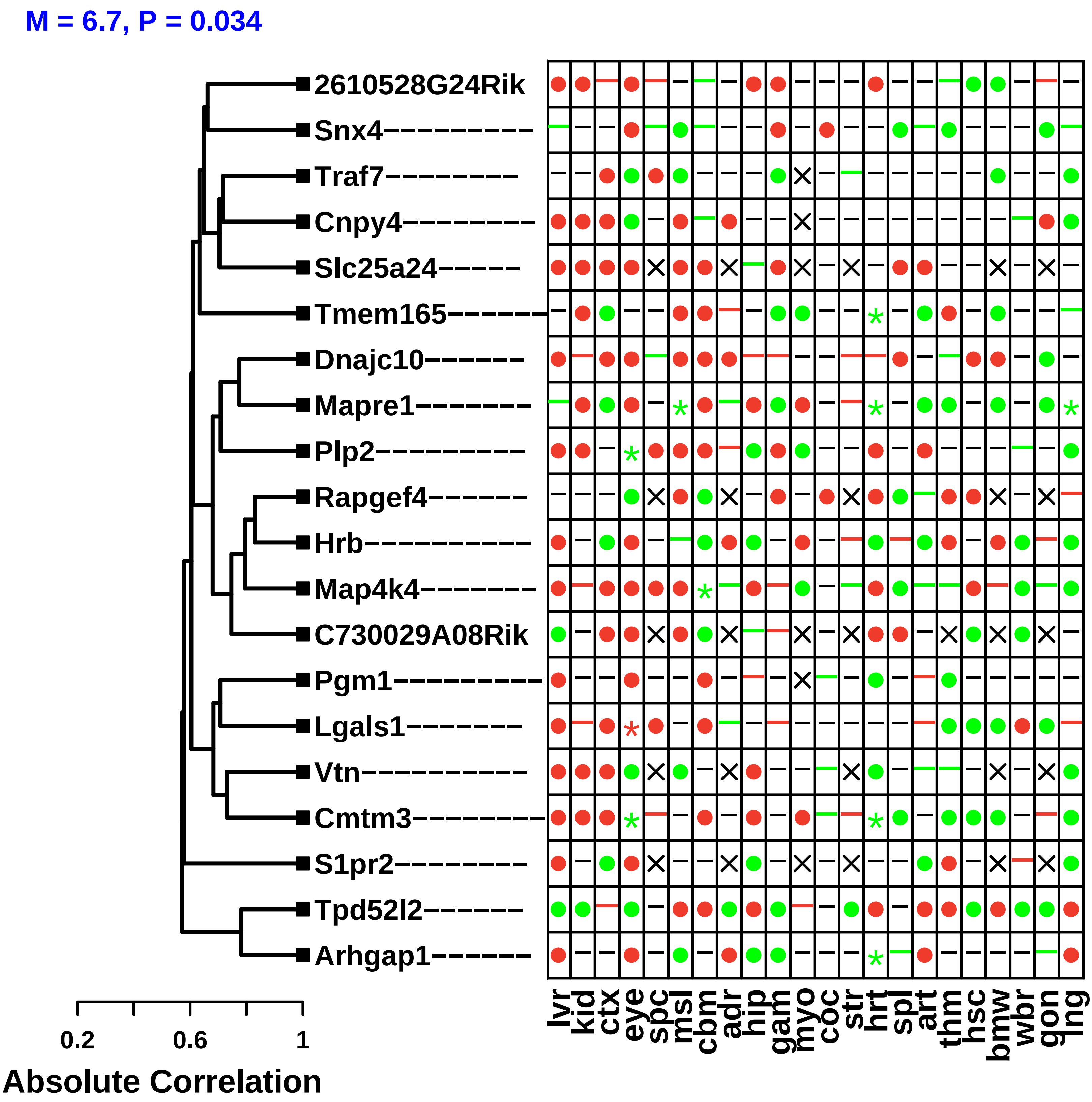

Age−Regulated Modules (20 Genes)

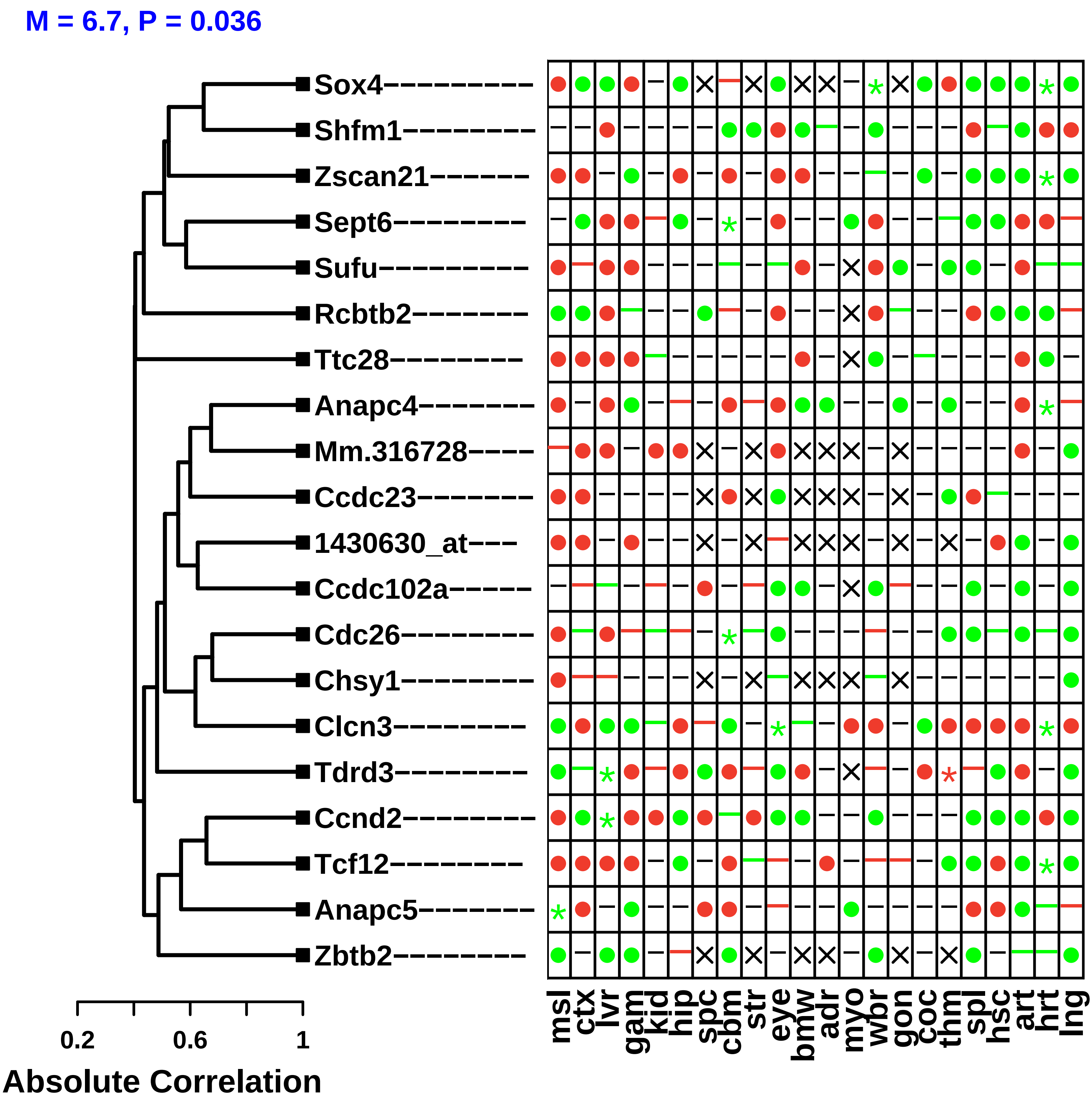

Age−Regulated Modules (20 Genes)

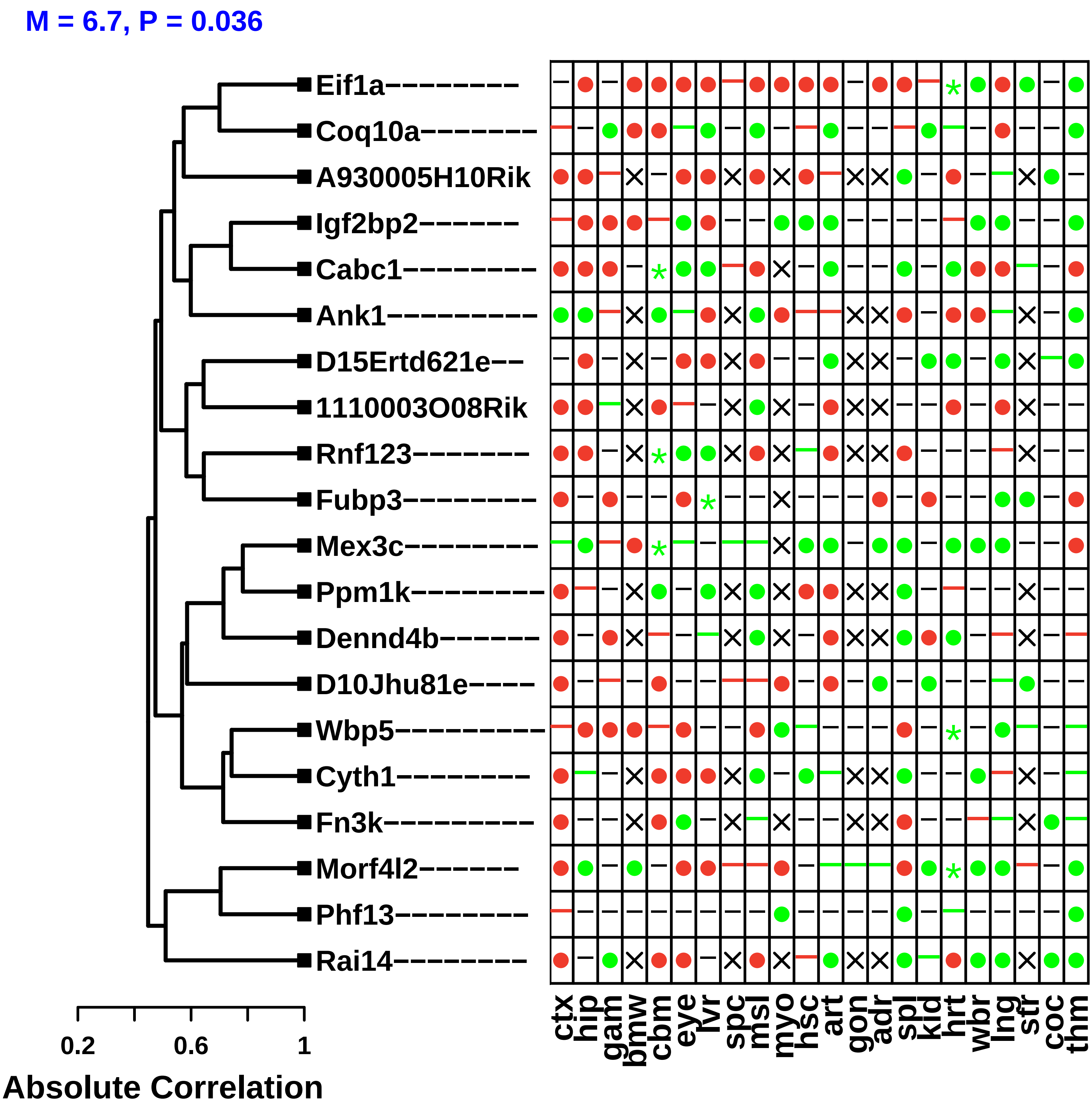

Age−Regulated Modules (20 Genes)

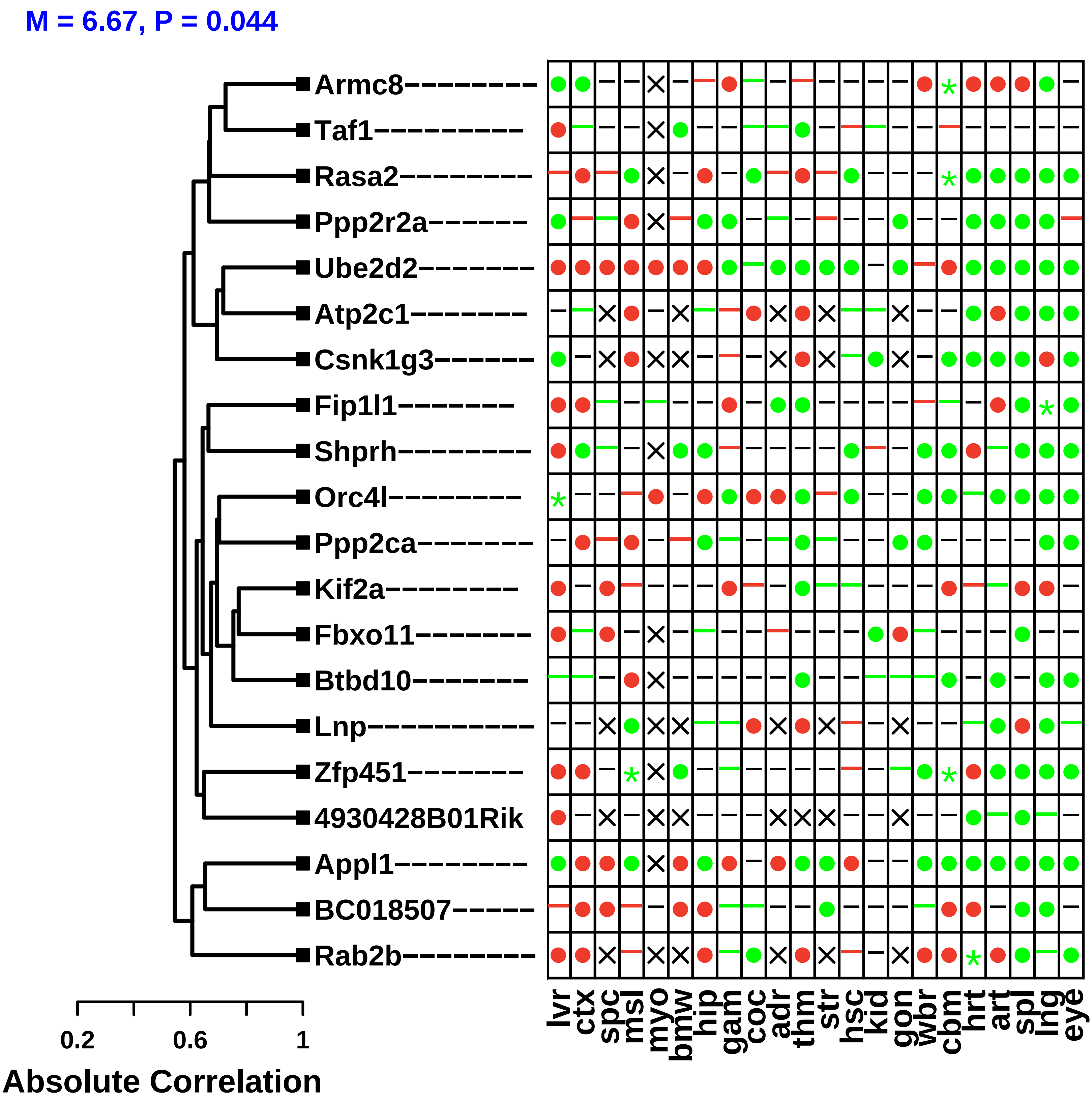

Age−Regulated Modules (40 Genes)

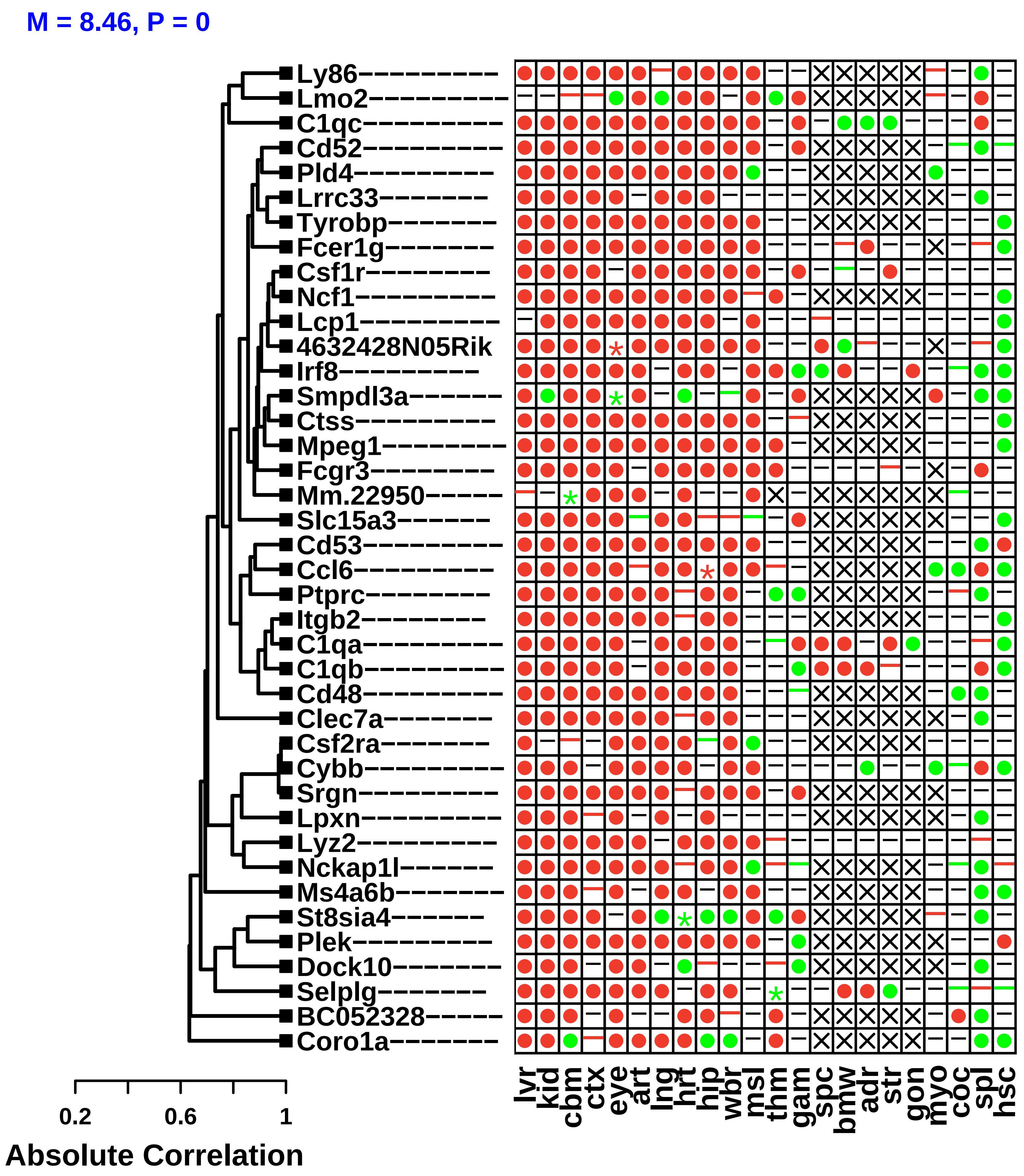

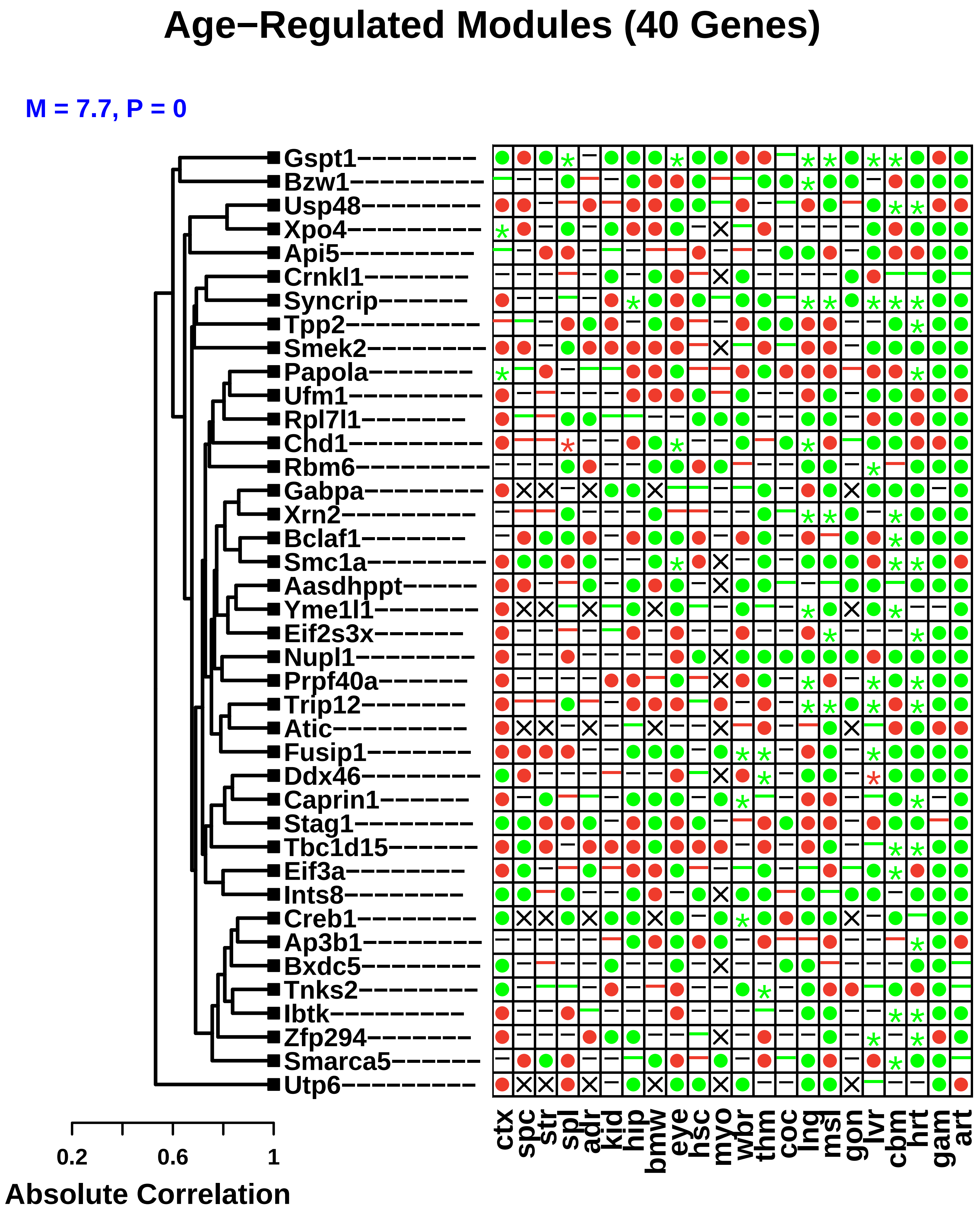
Age−Regulated Modules (40 Genes)
M = 7.7, P = 0
Gspt1
Bzw1
Usp48
Xpo4
Api5
Crnkl1
Syncrip
Tpp2
Smek2
Papola
Ufm1
Rpl7l1
Chd1
Rbm6
Gabpa
Xrn2
Bclaf1
Smc1a
Aasdhppt
Yme1l1
Eif2s3x
Nupl1
Prpf40a
Trip12
Atic
Fusip1
Ddx46
Caprin1
Stag1
Tbc1d15
Eif3a
Ints8
Creb1
Ap3b1
Bxdc5
Tnks2
Ibtk
Zfp294
Smarca5
Utp6
ctx spc str spl adr kid hip bmw eye hsc myo wbr thm coc lng msl gon lvr cbm hrt gam art
0.2
0.6
1
Absolute Correlation

Age−Regulated Modules (40 Genes)

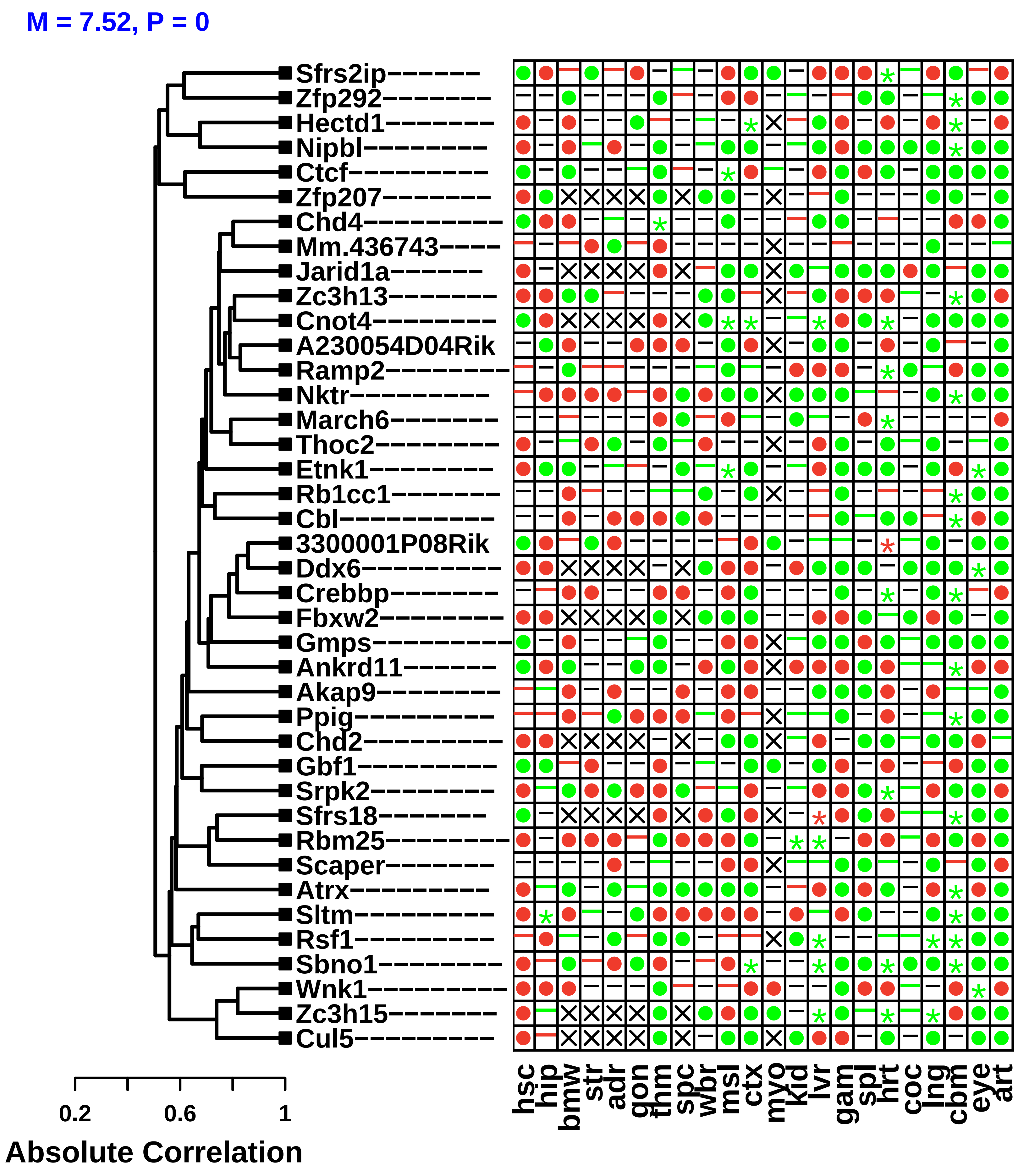

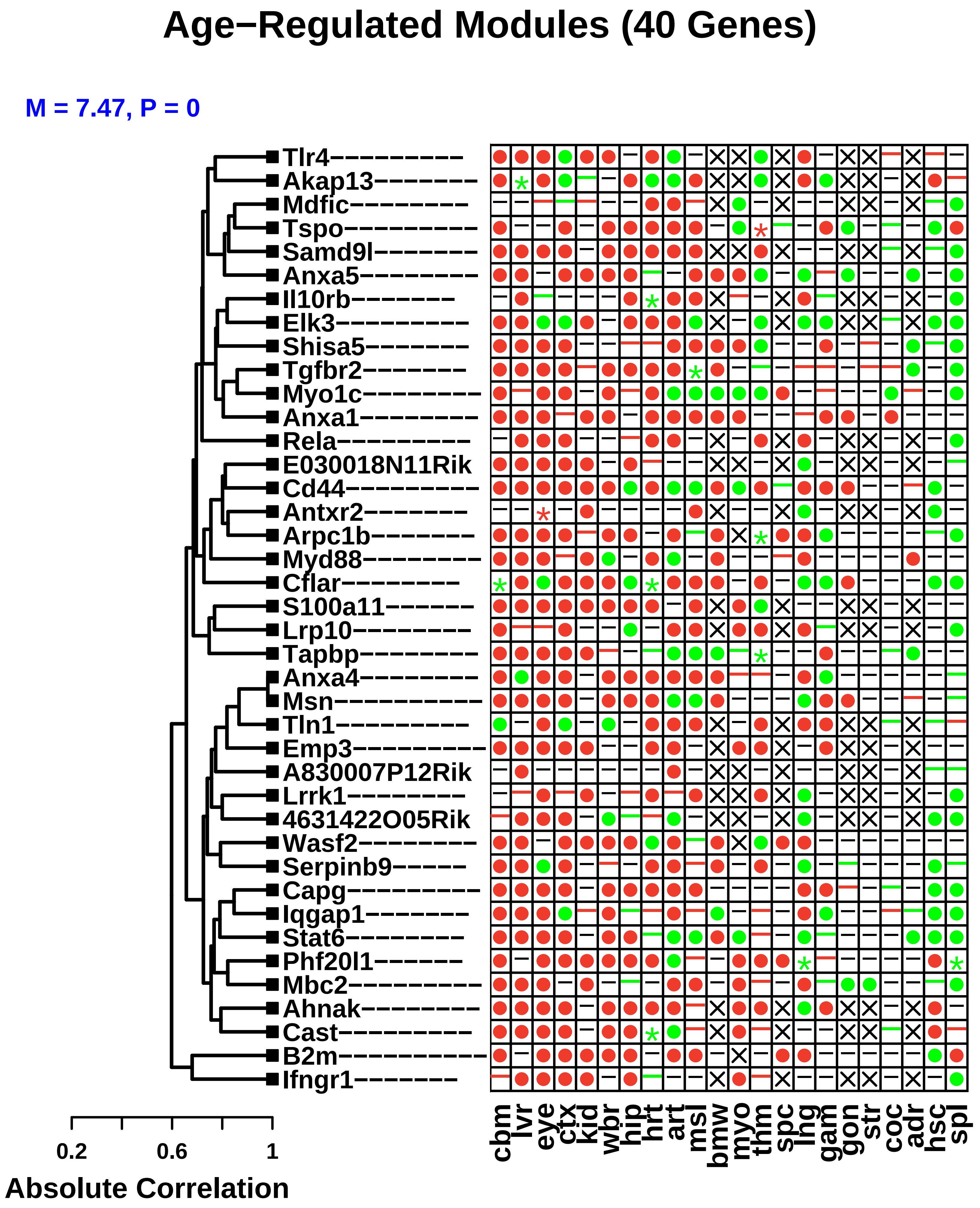

Age−Regulated Modules (40 Genes)
M = 7.47, P = 0
Tlr4
Akap13
Mdfic
Tspo
Samd9l
Anxa5
Il10rb
Elk3
Shisa5
Tgfbr2
Myo1c
Anxa1
Rela
E030018N11Rik
Cd44
Antxr2
Arpc1b
Myd88
Cflar
S100a11
Lrp10
Tapbp
Anxa4
Msn
Tln1
Emp3
A830007P12Rik
Lrrk1
4631422O05Rik
Wasf2
Serpinb9
Capg
Iqgap1
Stat6
Phf20l1
Mbc2
Ahnak
Cast
B2m
Ifngr1
cbm lvr eye ctx kid wbr hip hrt art msl bmw myo thm spc lng gam gon str coc adr hsc spl
0.2
0.6
1
Absolute Correlation

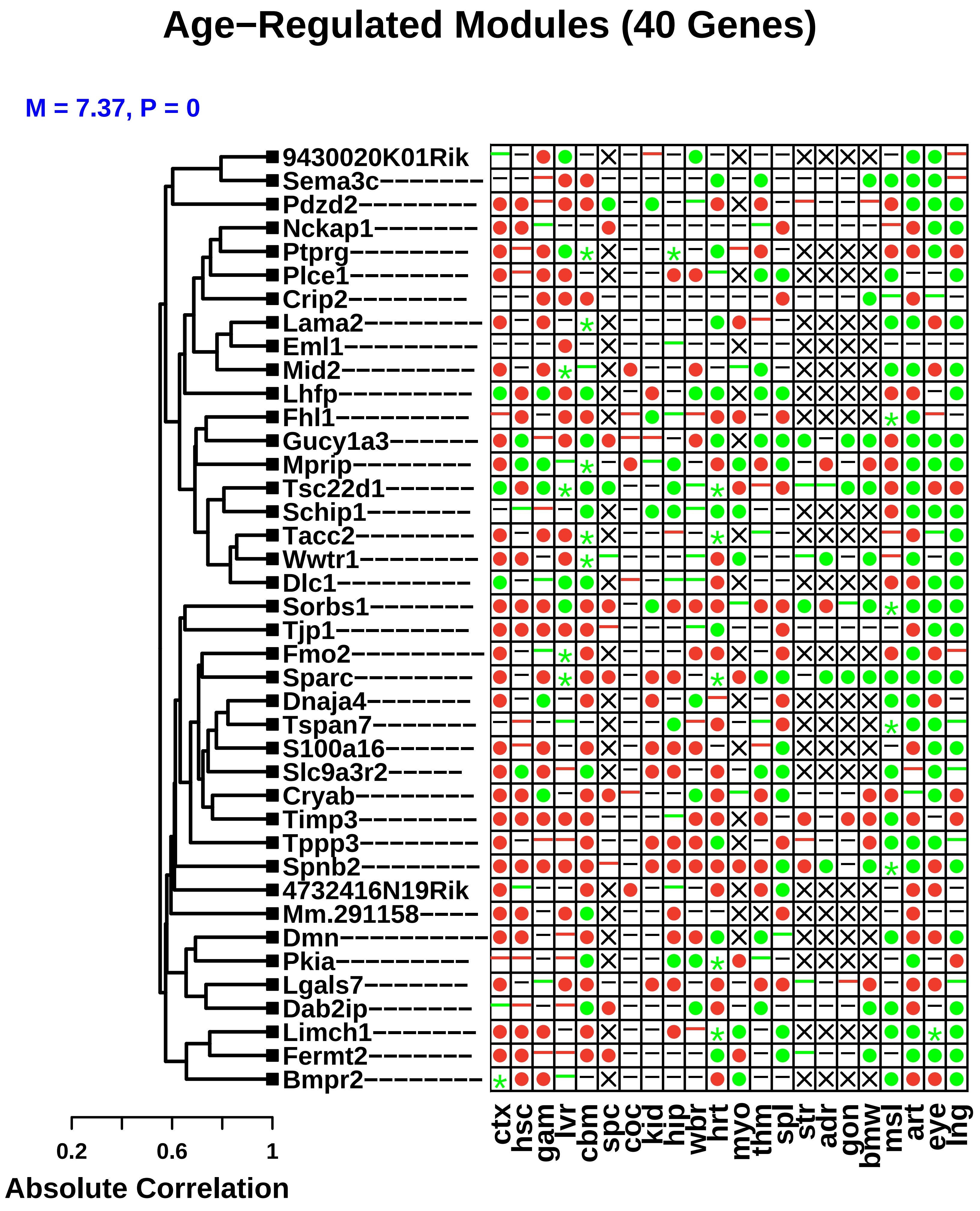

Age−Regulated Modules (40 Genes)
M = 7.37, P = 0
9430020K01Rik
Sema3c
Pdzd2
Nckap1
Ptprg
Plce1
Crip2
Lama2
Eml1
Mid2
Lhfp
Fhl1
Gucy1a3
Mprip
Tsc22d1
Schip1
Tacc2
Wwtr1
Dlc1
Sorbs1
Tjp1
Fmo2
Sparc
Dnaja4
Tspan7
S100a16
Slc9a3r2
Cryab
Timp3
Tppp3
Spnb2
4732416N19Rik
Mm.291158
Dmn
Pkia
Lgals7
Dab2ip
Limch1
Fermt2
Bmpr2
ctx hsc gam lvr cbm spc coc kid hip wbr hrt myo thm spl str adr gon bmw msl art eye lng
0.2
0.6
1
Absolute Correlation

Age−Regulated Modules (40 Genes)

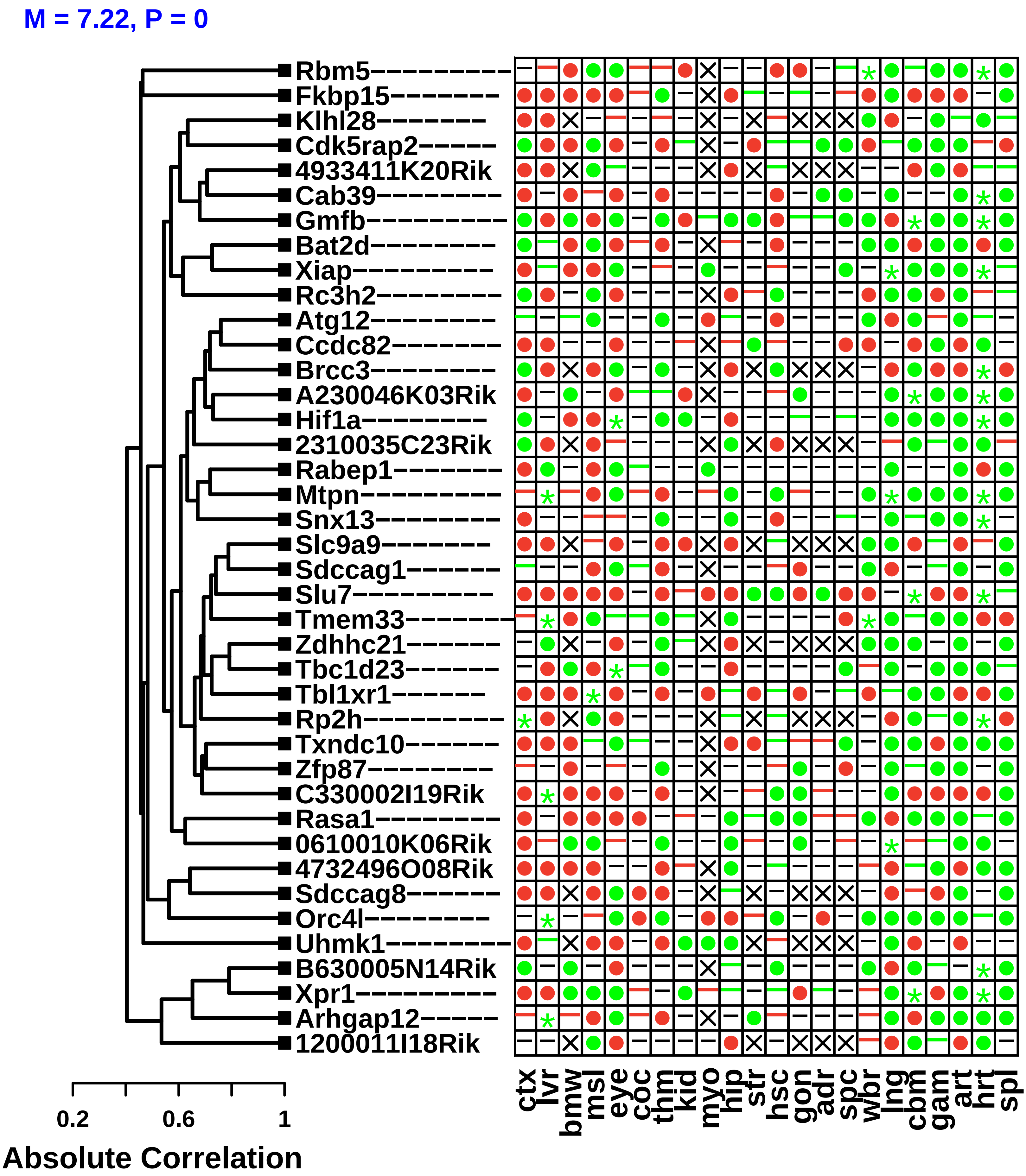

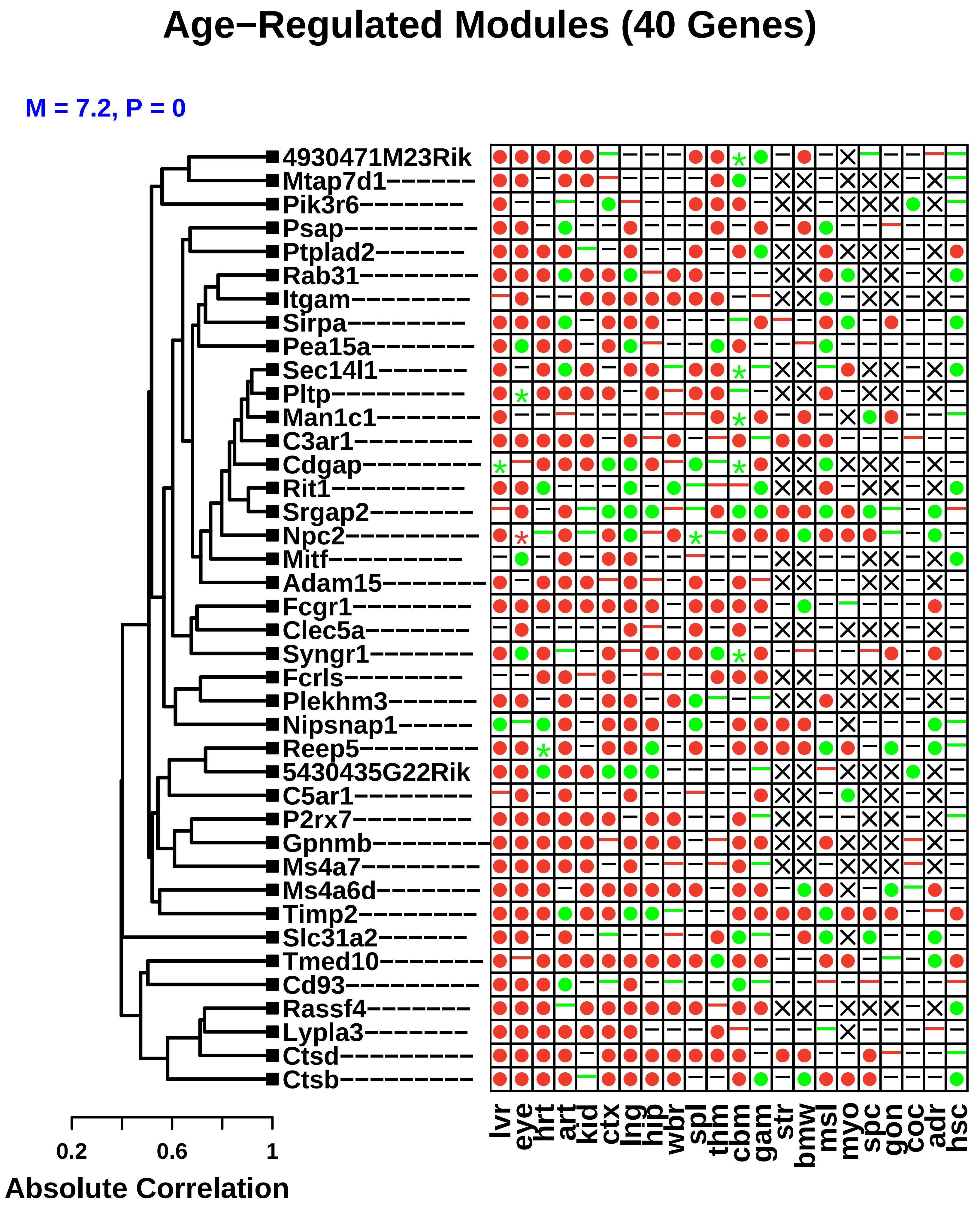
Age−Regulated Modules (40 Genes)
M = 7.2, P = 0
4930471M23Rik
Mtap7d1
Pik3r6
Psap
Ptplad2
Rab31
Itgam
Sirpa
Pea15a
Sec14l1
Pltp
Man1c1
C3ar1
Cdgap
Rit1
Srgap2
Npc2
Mitf
Adam15
Fcgr1
Clec5a
Syngr1
Fcrls
Plekhm3
Nipsnap1
Reep5
5430435G22Rik
C5ar1
P2rx7
Gpnmb
Ms4a7
Ms4a6d
Timp2
Slc31a2
Tmed10
Cd93
Rassf4
Lypla3
Ctsd
Ctsb
lvr
eye
hrt
art
kid
ctx
lng
hip
wbr
spl
thm
cbm
gam
str
bmw
msl
myo
spc
gon
coc
adr
hsc
0.2
0.6
1
Absolute Correlation

Age−Regulated Modules (40 Genes)

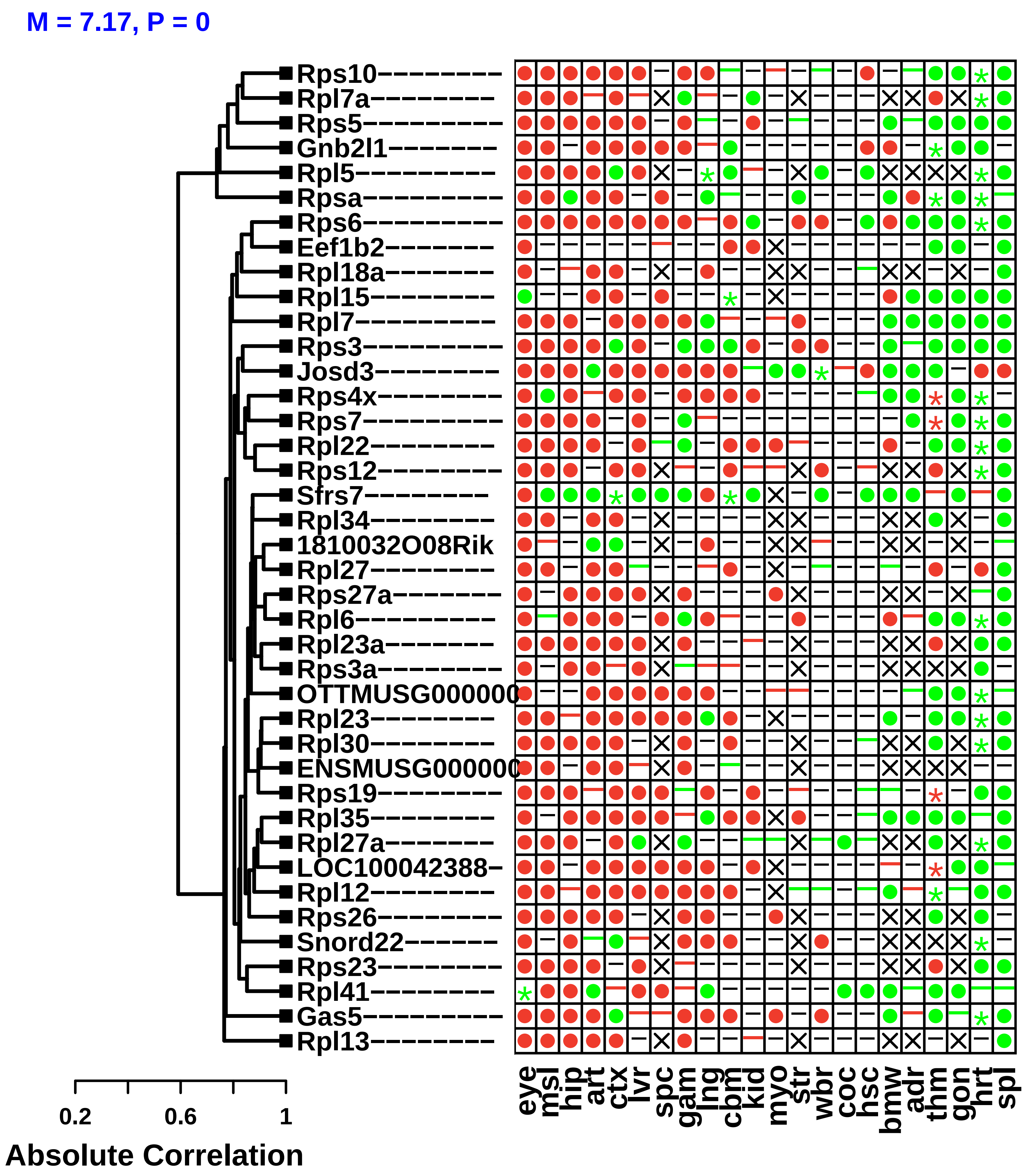

Age−Regulated Modules (40 Genes)

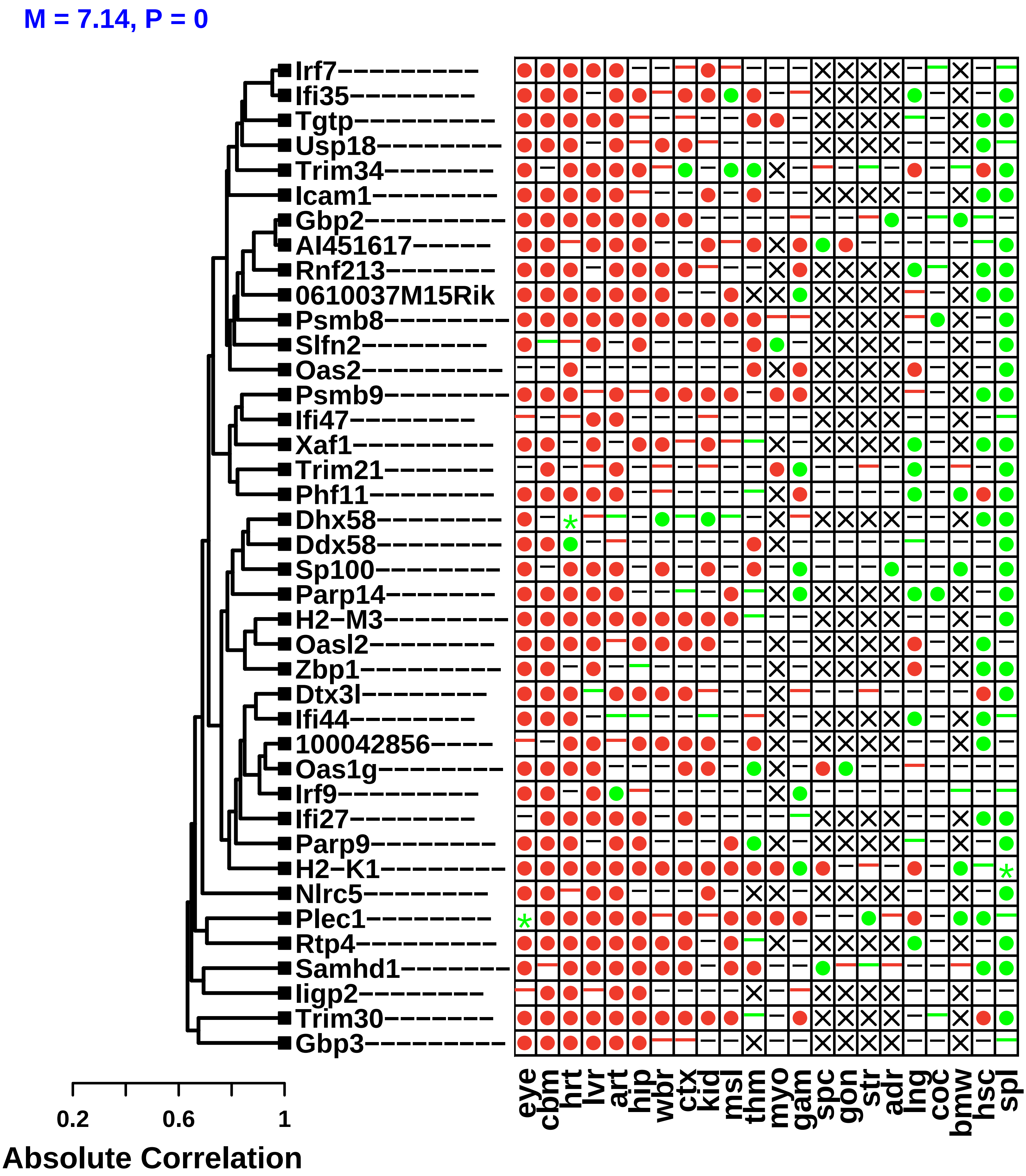

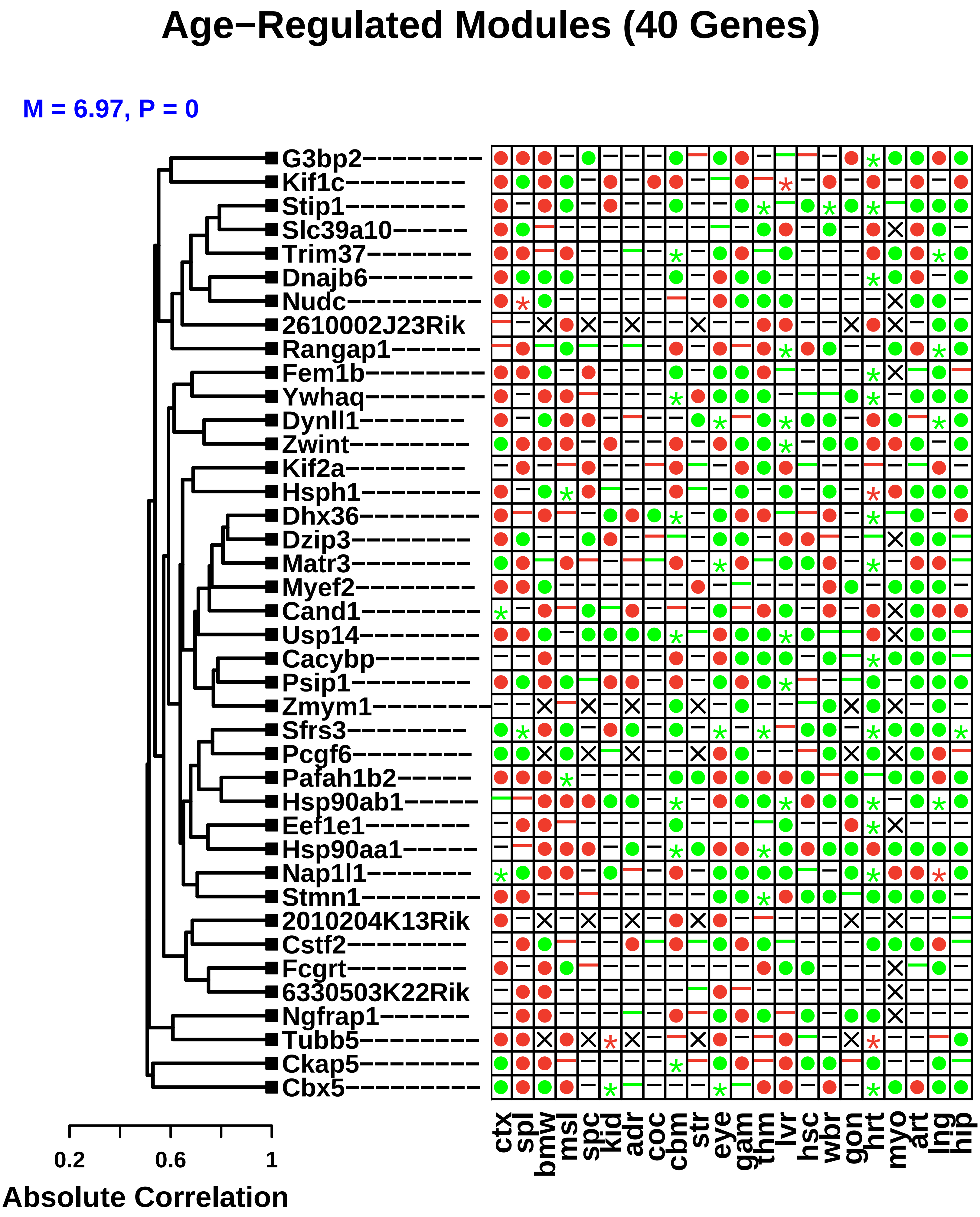
Age−Regulated Modules (40 Genes)
M = 6.97, P = 0
G3bp2
Kif1c
Stip1
Slc39a10
Trim37
Dnajb6
Nudc
2610002J23Rik
Rangap1
Fem1b
Ywhaq
Dynll1
Zwint
Kif2a
Hsph1
Dhx36
Dzip3
Matr3
Myef2
Cand1
Usp14
Cacybp
Psip1
Zmym1
Sfrs3
Pcgf6
Pafah1b2
Hsp90ab1
Eef1e1
Hsp90aa1
Nap1l1
Stmn1
2010204K13Rik
Cstf2
Fcgrt
6330503K22Rik
Ngfrap1
Tubb5
Ckap5
Cbx5
ctx
spl
bmw
msl
spc
kid
adr
coc
cbm
str
eye
gam
thm
lvr
hsc
wbr
gon
hrt
myo
art
lng
hip
0.2
0.6
1
Absolute Correlation

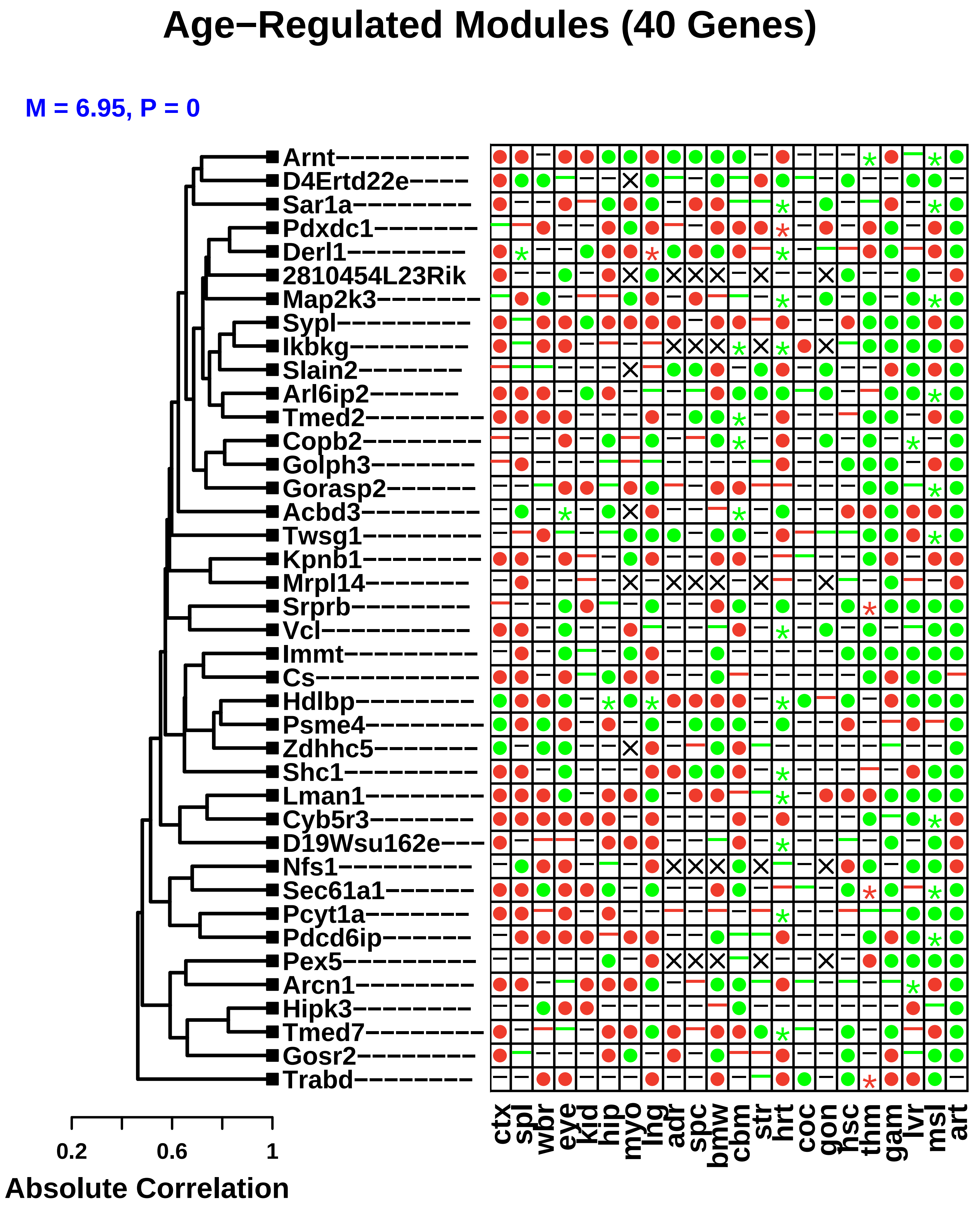

Age−Regulated Modules (40 Genes)
M = 6.95, P = 0
Arnt
D4Ertd22e
Sar1a
Pdxdc1
Derl1
2810454L23Rik
Map2k3
Sypl
Ikbkg
Slain2
Arl6ip2
Tmed2
Copb2
Golph3
Gorasp2
Acbd3
Twsg1
Kpnb1
Mrpl14
Srprb
Vcl
Immt
Cs
Hdlbp
Psme4
Zdhhc5
Shc1
Lman1
Cyb5r3
D19Wsu162e
Nfs1
Sec61a1
Pcyt1a
Pdcd6ip
Pex5
Arcn1
Hipk3
Tmed7
Gosr2
Trabd
ctx
spl
wbr
eye
kid
hip
myo
lng
adr
spc
bmw
cbm
str
hrt
coc
gon
hsc
thm
gam
lvr
msl
art
0.2
0.6
1
Absolute Correlation

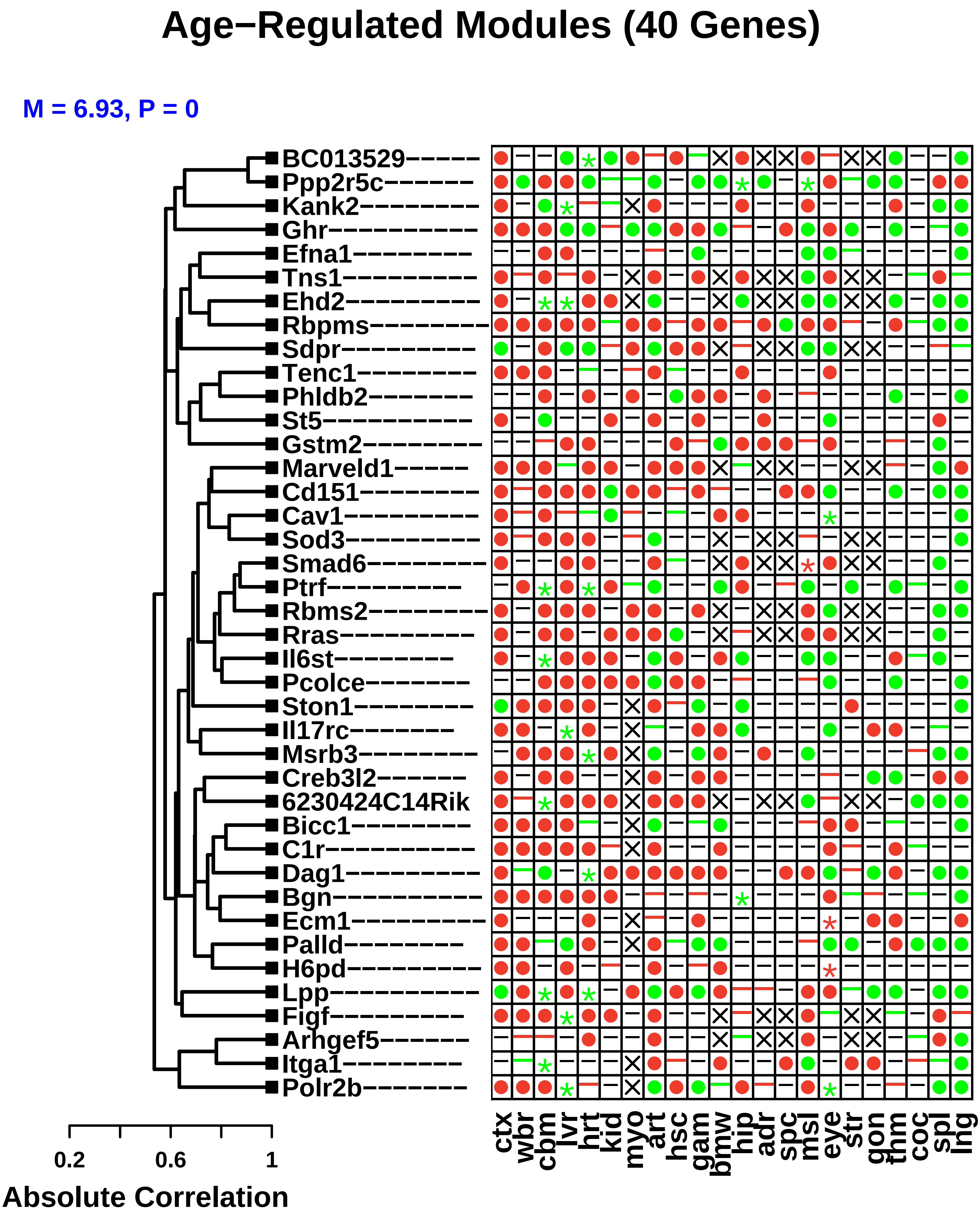

Age−Regulated Modules (40 Genes)
M = 6.93, P = 0
BC013529
Ppp2r5c
Kank2
Ghr
Efna1
Tns1
Ehd2
Rbpms
Sdpr
Tenc1
Phldb2
St5
Gstm2
Marveld1
Cd151
Cav1
Sod3
Smad6
Ptrf
Rbms2
Rras
Il6st
Pcolce
Ston1
Il17rc
Msrb3
Creb3l2
6230424C14Rik
Bicc1
C1r
Dag1
Bgn
Ecm1
Palld
H6pd
Lpp
Figf
Arhgef5
Itga1
Polr2b
ctx
wbr
cbm
lvr
hrt
kid
myo
art
hsc
gam
bmw
hip
adr
spc
msl
eye
str
gon
thm
coc
spl
lng
0.2
0.6
1
Absolute Correlation

Age−Regulated Modules (40 Genes)

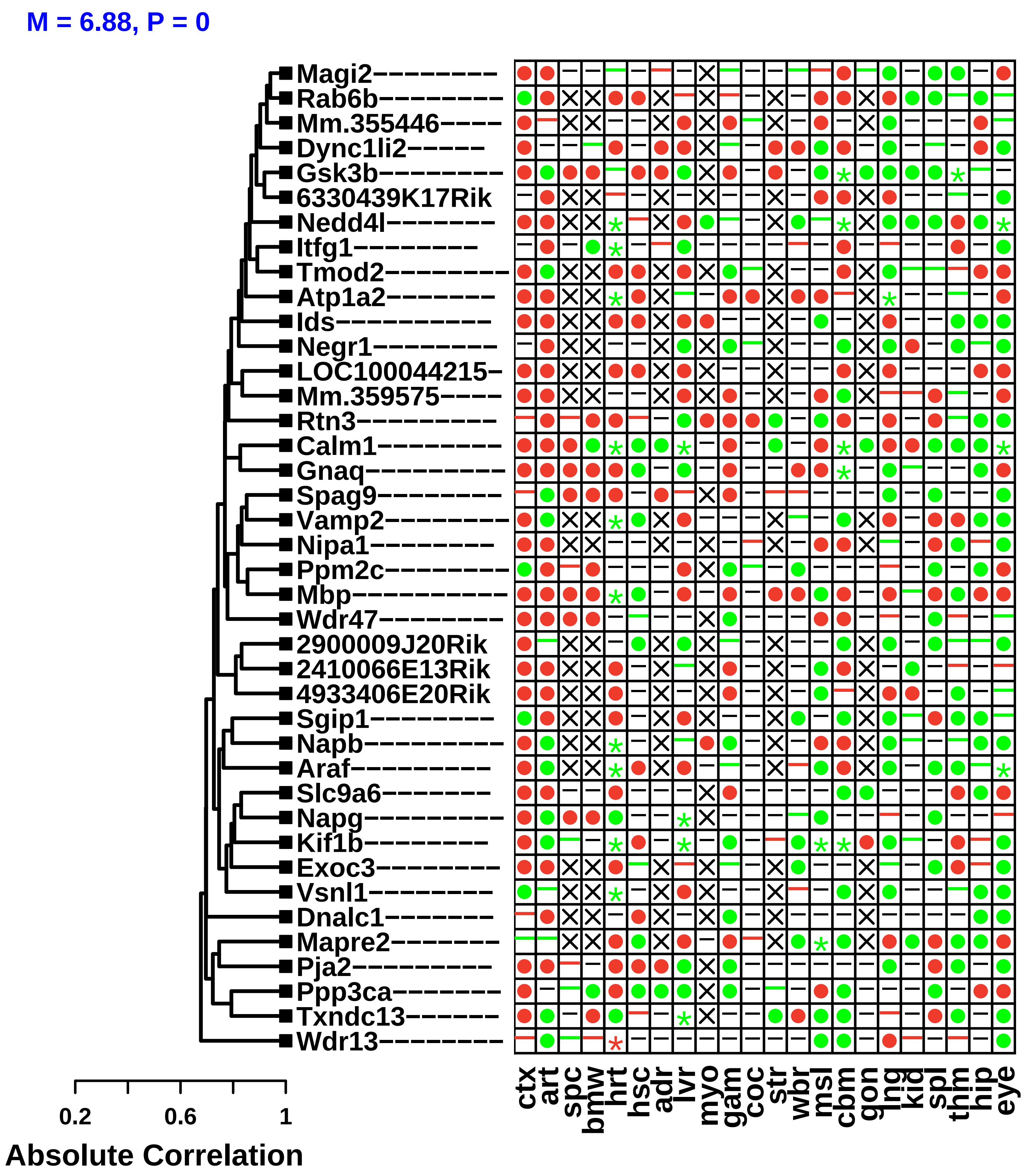

Age−Regulated Modules (40 Genes)

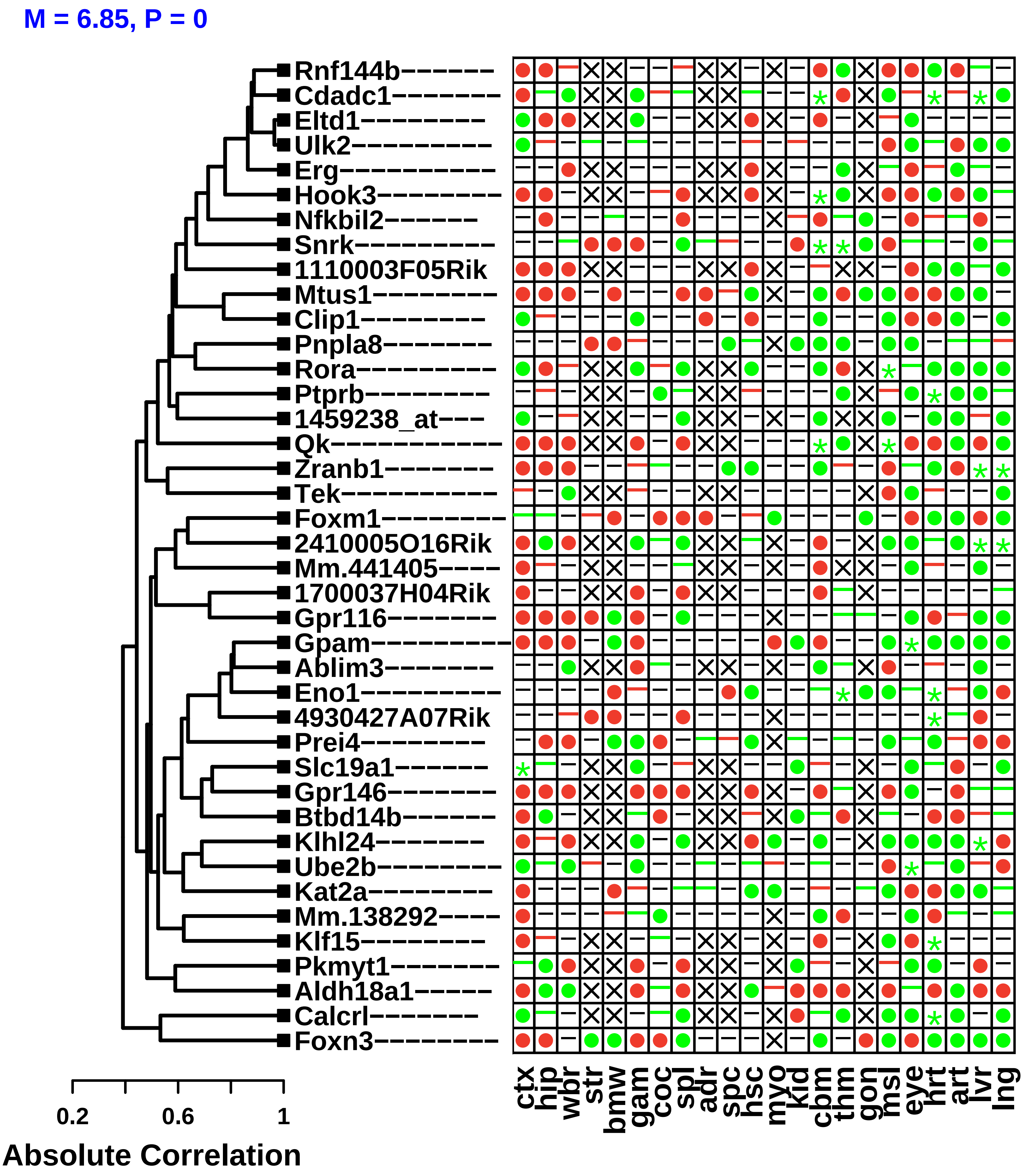

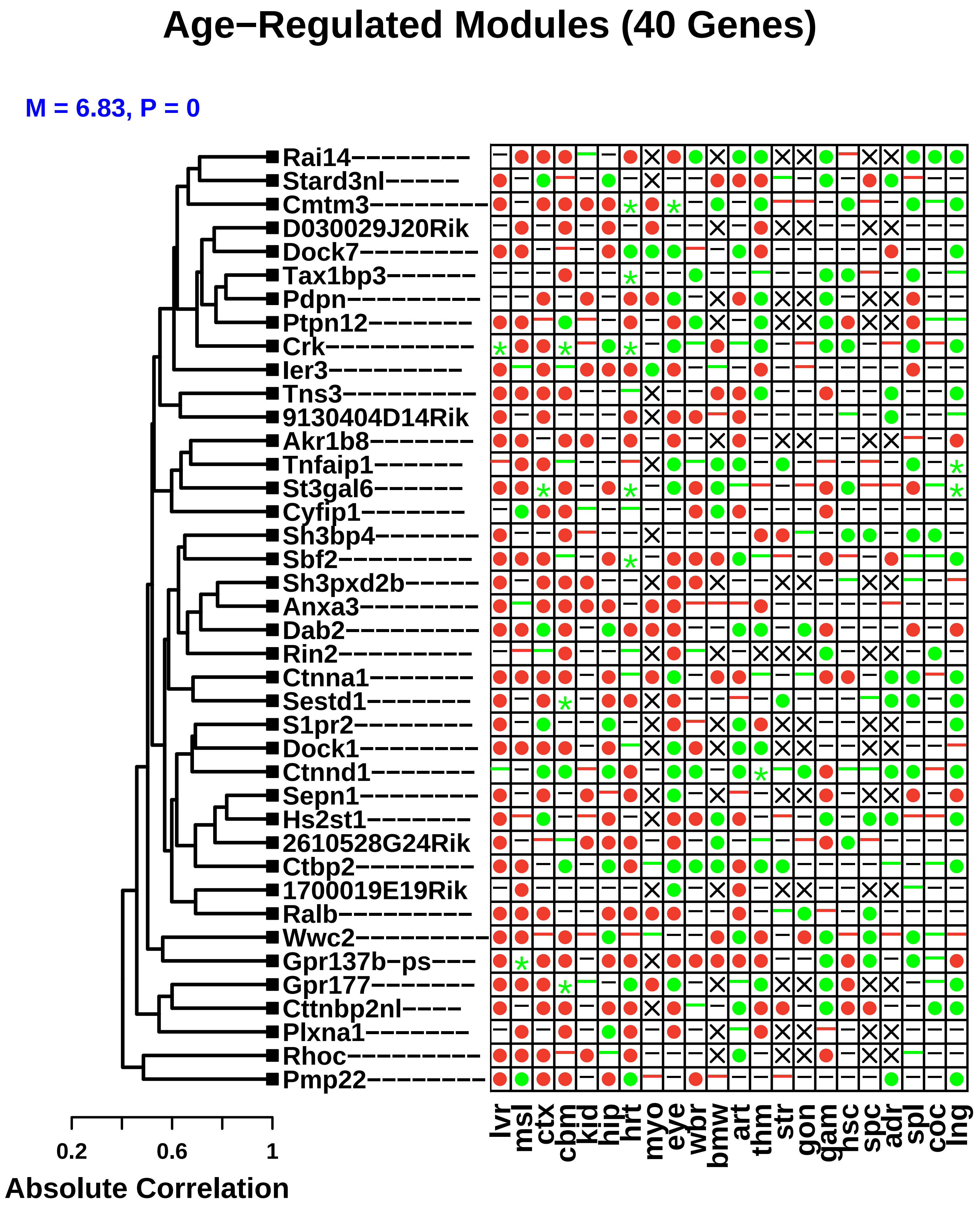

Age−Regulated Modules (40 Genes)
M = 6.83, P = 0
Rai14
Stard3nl
Cmtm3
D030029J20Rik
Dock7
Tax1bp3
Pdpn
Ptpn12
Crk
Ier3
Tns3
9130404D14Rik
Akr1b8
Tnfaip1
St3gal6
Cyfip1
Sh3bp4
Sbf2
Sh3pxd2b
Anxa3
Dab2
Rin2
Ctnna1
Sestd1
S1pr2
Dock1
Ctnnd1
Sepn1
Hs2st1
2610528G24Rik
Ctbp2
1700019E19Rik
Ralb
Wwc2
Gpr137b−ps
Gpr177
Cttnbp2nl
Plxna1
Rhoc
Pmp22
0.2
0.6
1
Absolute Correlation
lvr
msl
ctx
cbm
kid
hip
hrt
myo
eye
wbr
bmw
art
thm
str
gon
gam
hsc
spc
adr
spl
coc
lng

Age−Regulated Modules (40 Genes)

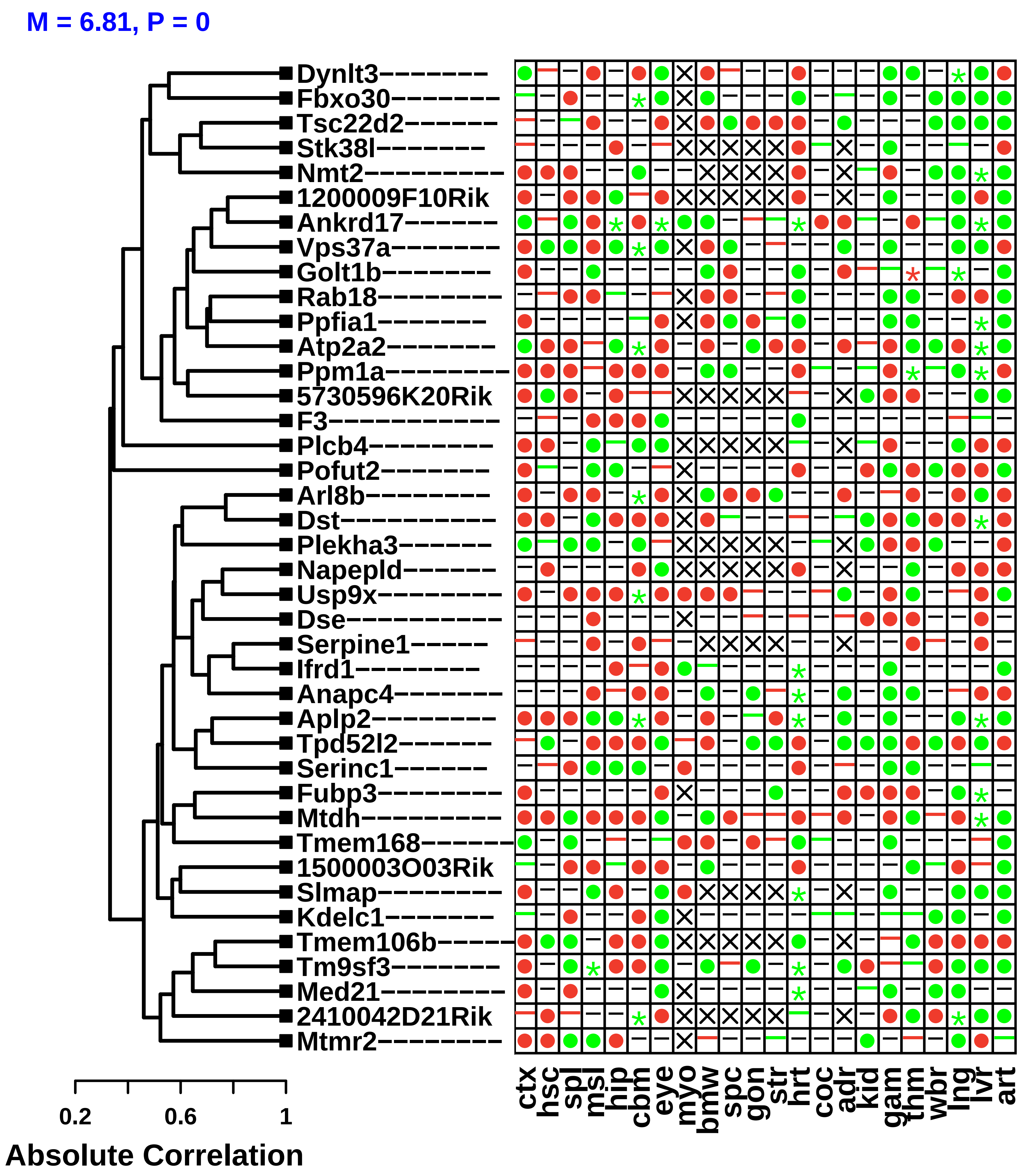

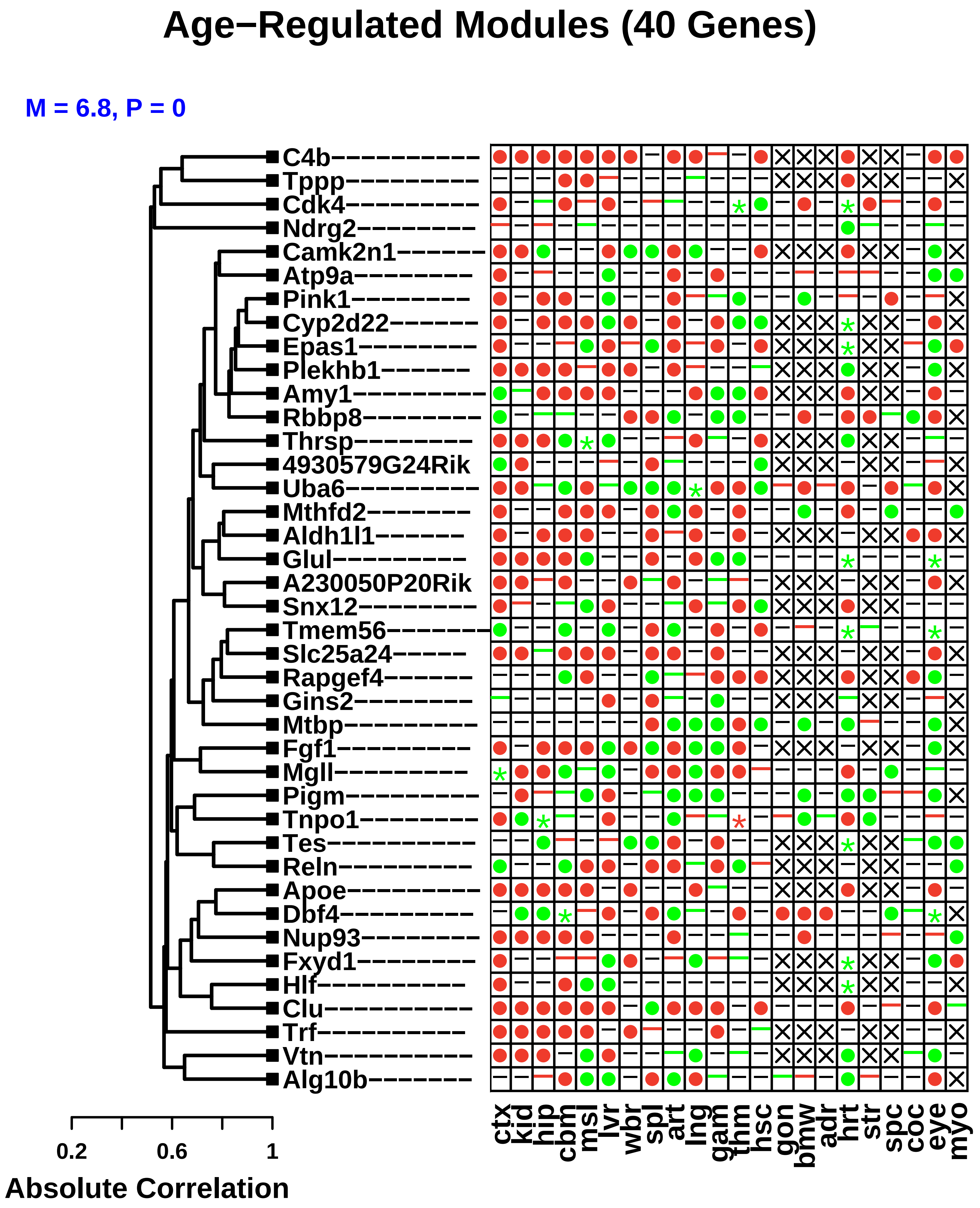
Age−Regulated Modules (40 Genes)
M = 6.8, P = 0
C4b
Tppp
Cdk4
Ndrg2
Camk2n1
Atp9a
Pink1
Cyp2d22
Epas1
Plekhb1
Amy1
Rbbp8
Thrsp
4930579G24Rik
Uba6
Mthfd2
Aldh1l1
Glul
A230050P20Rik
Snx12
Tmem56
Slc25a24
Rapgef4
Gins2
Mtbp
Fgf1
Mgll
Pigm
Tnpo1
Tes
Reln
Apoe
Dbf4
Nup93
Fxyd1
Hlf
Clu
Trf
Vtn
Alg10b
ctx
kid
hip
cbm
msl
lvr
wbr
spl
art
lng
gam
thm
hsc
gon
bmw
adr
hrt
str
spc
coc
eye
myo
0.2
0.6
1
Absolute Correlation

Age−Regulated Modules (40 Genes)

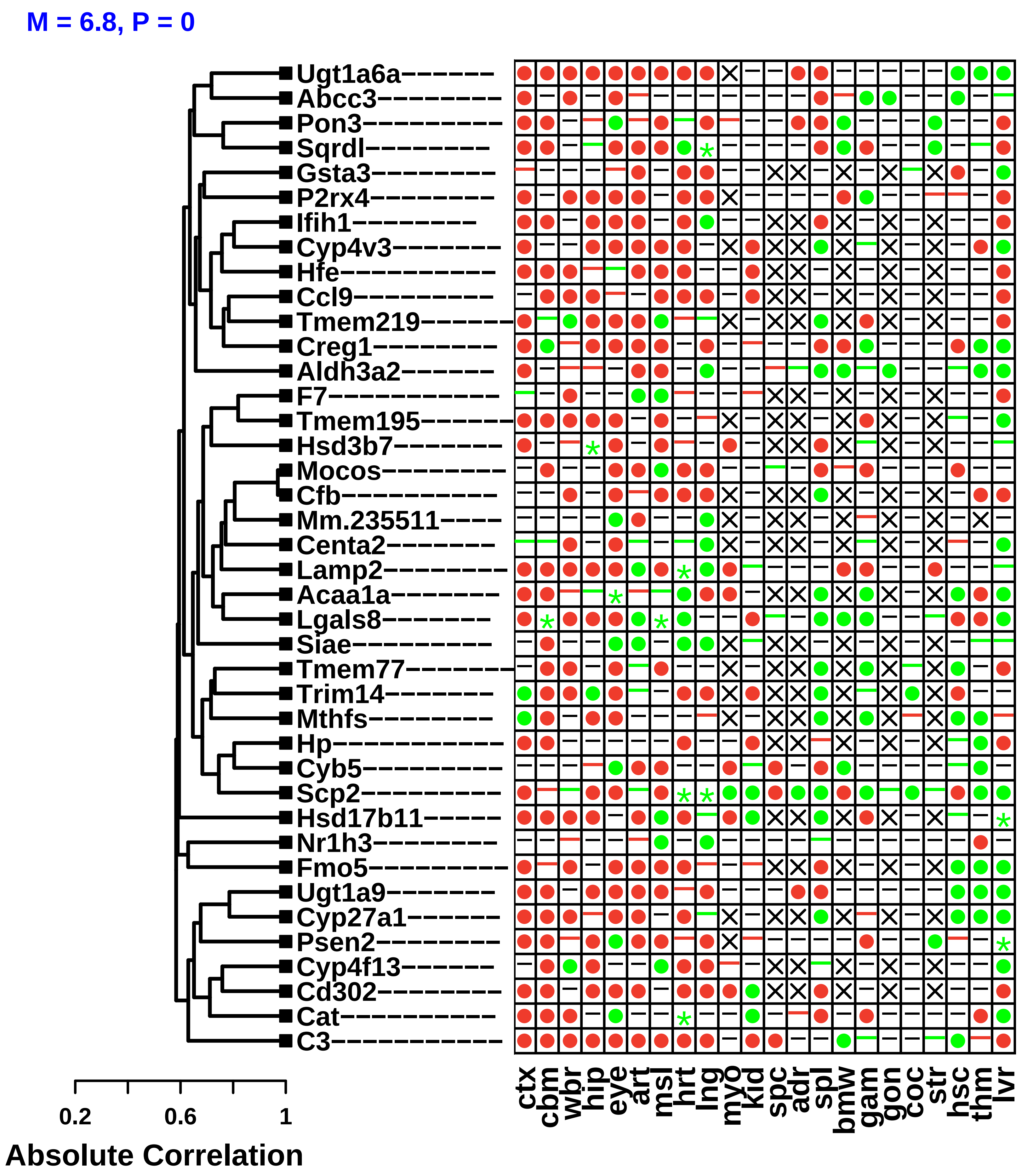

Age−Regulated Modules (40 Genes)

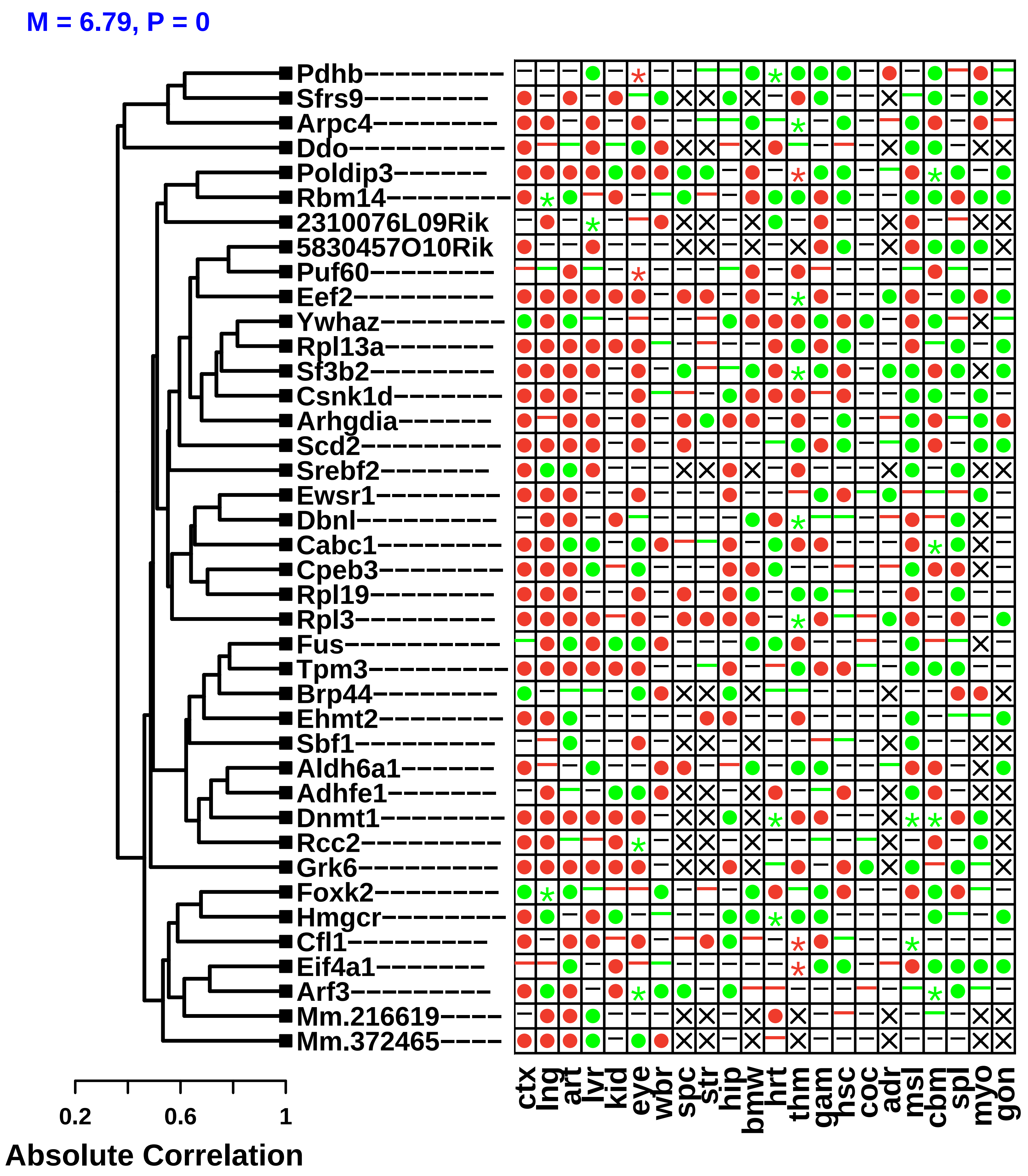

Age−Regulated Modules (40 Genes)

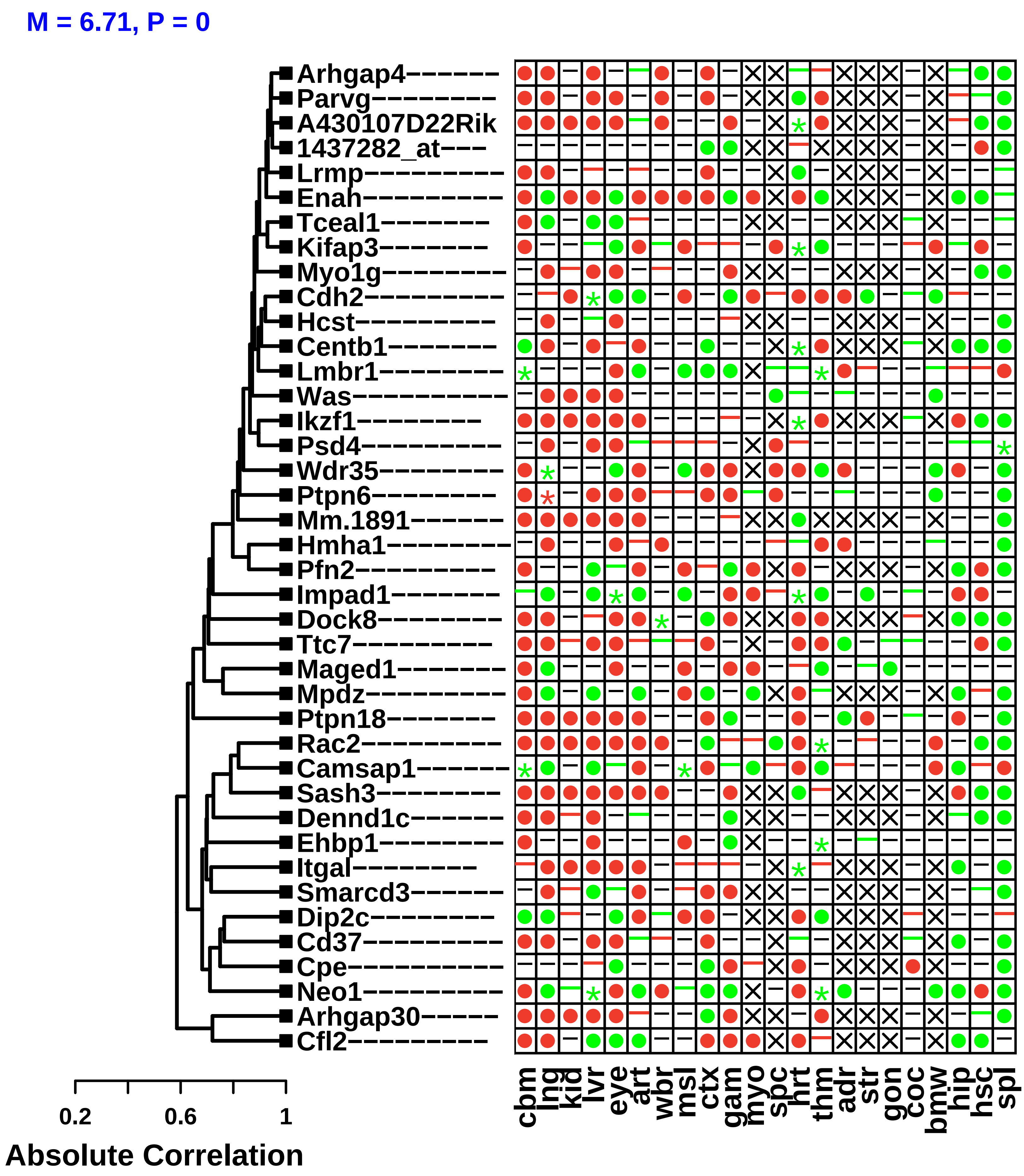

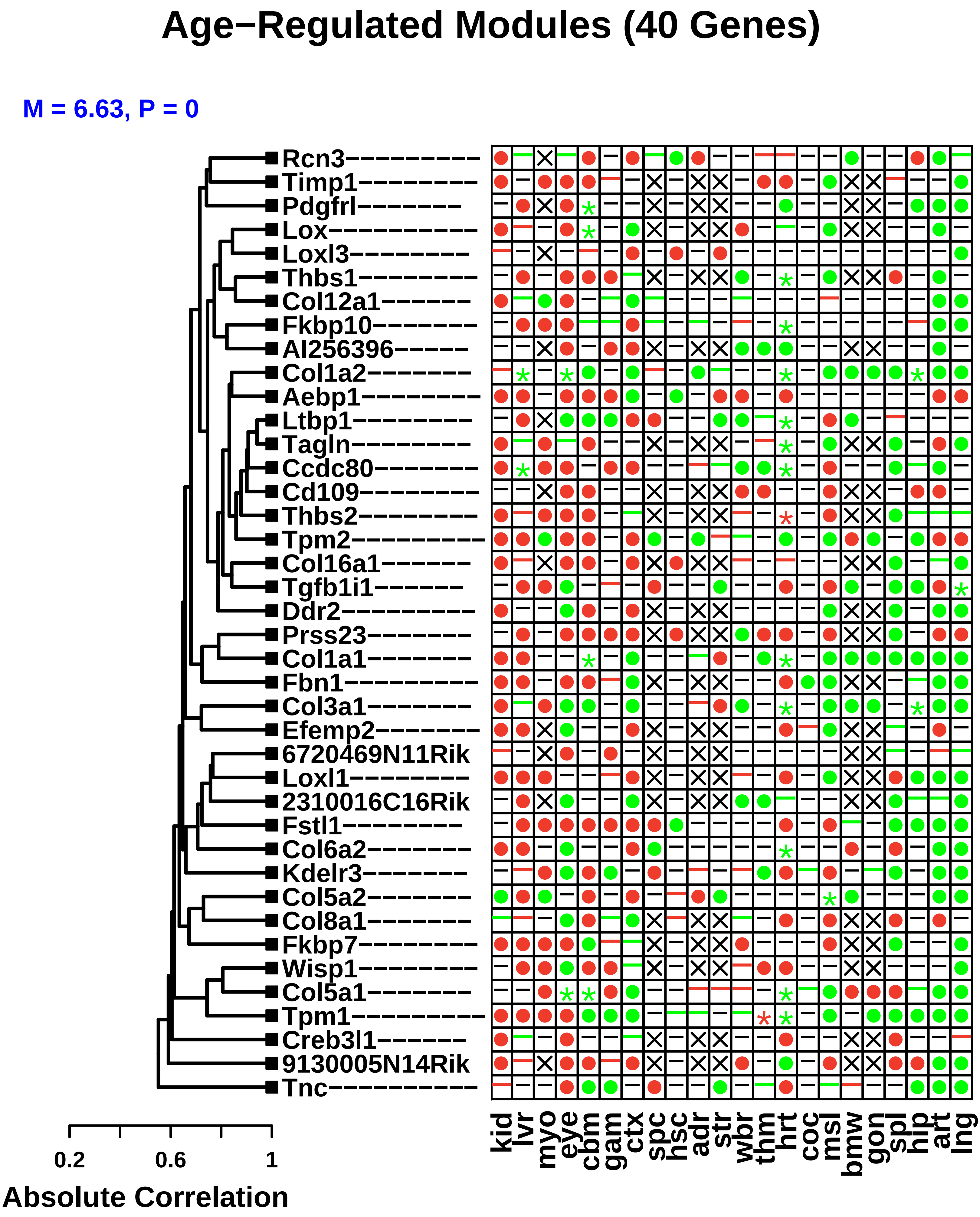
Age−Regulated Modules (40 Genes)
M = 6.63, P = 0
Rcn3
Timp1
Pdgfrl
Lox
Loxl3
Thbs1
Col12a1
Fkbp10
AI256396
Col1a2
Aebp1
Ltbp1
Tagln
Ccdc80
Cd109
Thbs2
Tpm2
Col16a1
Tgfb1i1
Ddr2
Prss23
Col1a1
Fbn1
Col3a1
Efemp2
6720469N11Rik
Loxl1
2310016C16Rik
Fstl1
Col6a2
Kdelr3
Col5a2
Col8a1
Fkbp7
Wisp1
Col5a1
Tpm1
Creb3l1
9130005N14Rik
Tnc
kid
lvr
myo
eye
cbm
gam
ctx
spc
hsc
adr
str
wbr
thm
hrt
coc
msl
bmw
gon
spl
hip
art
lng
0.2
0.6
1
Absolute Correlation

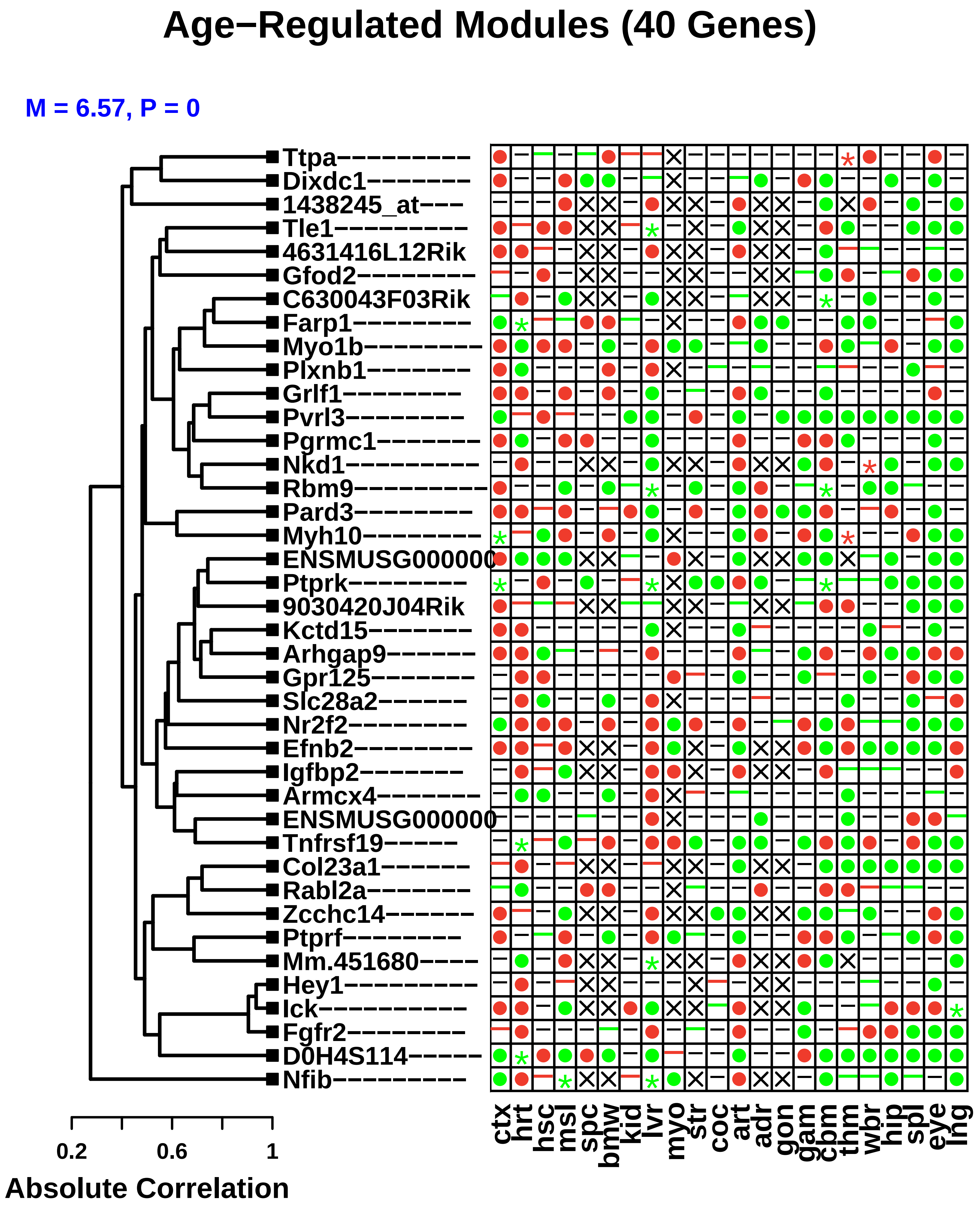
Age−Regulated Modules (40 Genes)
M = 6.57, P = 0
Ttpa
Dixdc1
1438245_at
Tle1
4631416L12Rik
Gfod2
C630043F03Rik
Farp1
Myo1b
Plxnb1
Grlf1
Pvrl3
Pgrmc1
Nkd1
Rbm9
Pard3
Myh10
ENSMUSG000000
Ptprk
9030420J04Rik
Kctd15
Arhgap9
Gpr125
Slc28a2
Nr2f2
Efnb2
Igfbp2
Armcx4
ENSMUSG000000
Tnfrsf19
Col23a1
Rabl2a
Zcchc14
Ptprf
Mm.451680
Hey1
Ick
Fgfr2
D0H4S114
Nfib
ctx
hrt
hsc
msl
spc
bmw
kid
lvr
myo
str
coc
art
adr
gon
gam
cbm
thm
wbr
hip
spl
eye
lng
0.2
0.6
1
Absolute Correlation

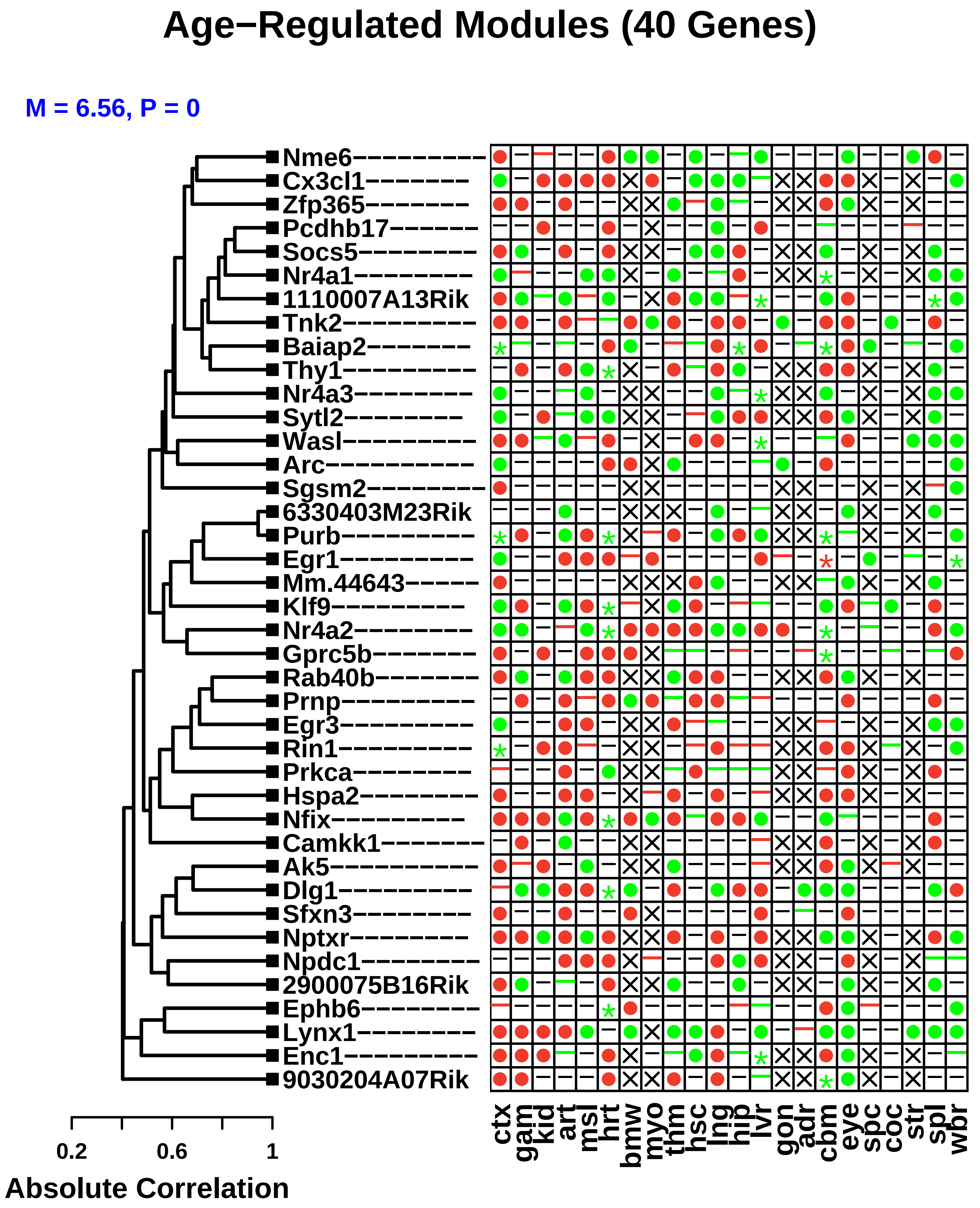

Age−Regulated Modules (40 Genes)
M = 6.56, P = 0
Nme6
Cx3cl1
Zfp365
Pcdhb17
Socs5
Nr4a1
1110007A13Rik
Tnk2
Baiap2
Thy1
Nr4a3
Sytl2
Wasl
Arc
Sgsm2
6330403M23Rik
Purb
Egr1
Mm.44643
Klf9
Nr4a2
Gprc5b
Rab40b
Prnp
Egr3
Rin1
Prkca
Hspa2
Nfix
Camkk1
Ak5
Dlg1
Sfxn3
Nptxr
Npdc1
2900075B16Rik
Ephb6
Lynx1
Enc1
9030204A07Rik
ctx
gam
kid
art
msl
hrt
bmw
myo
thm
hsc
lng
hip
lvr
gon
adr
cbm
eye
spc
coc
str
spl
wbr
0.2
0.6
1
Absolute Correlation

Age−Regulated Modules (40 Genes)

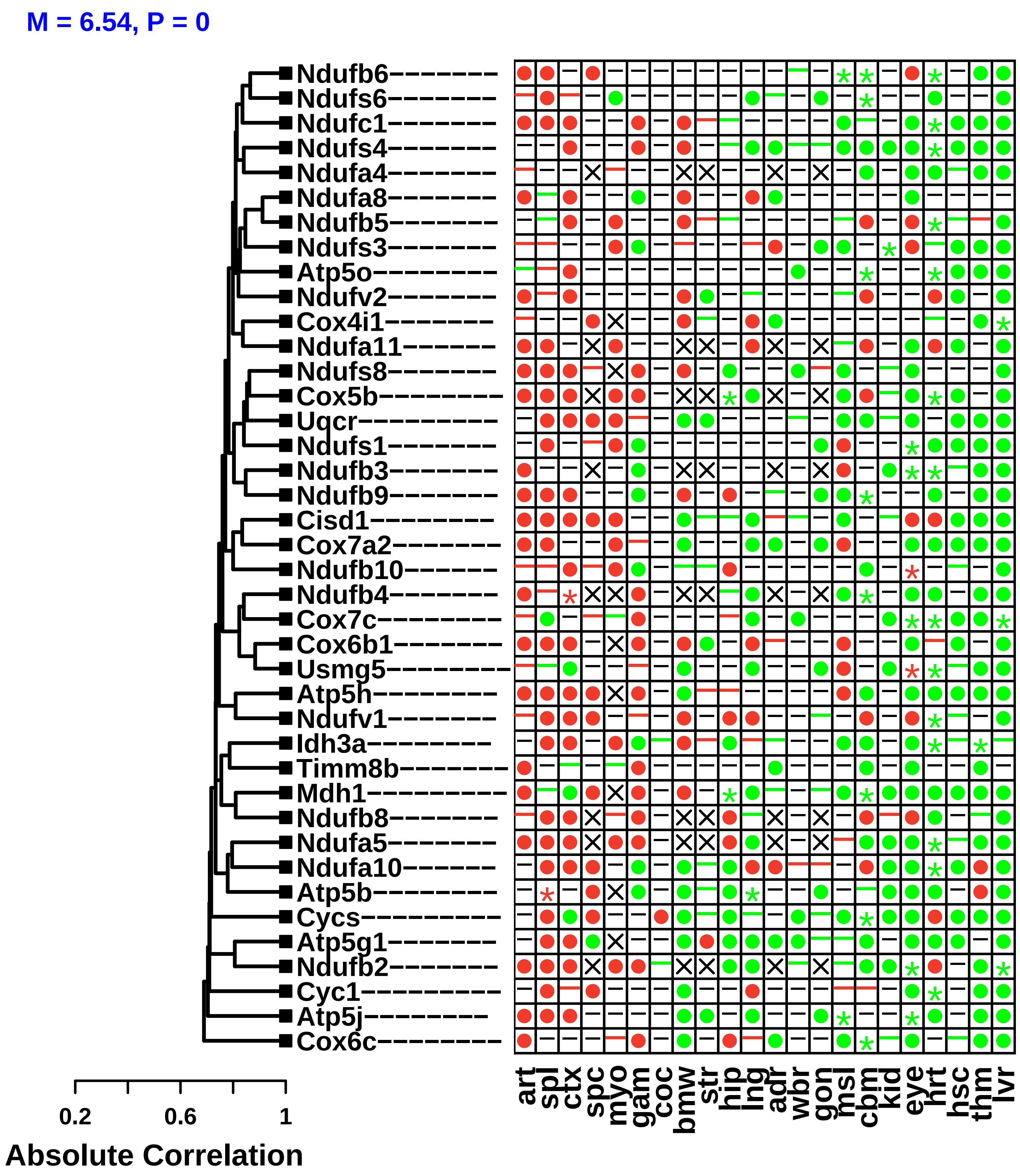

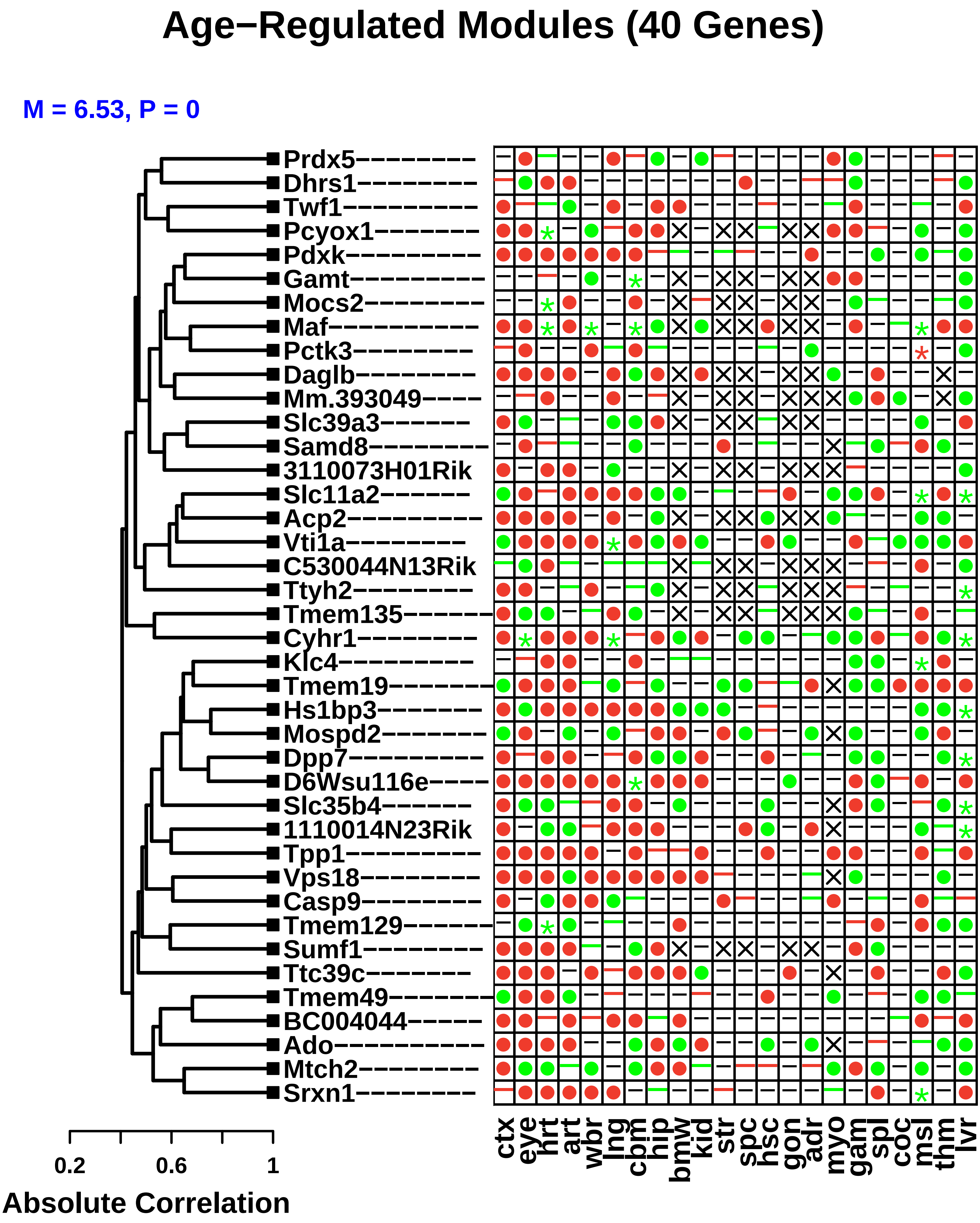

Age−Regulated Modules (40 Genes)
M = 6.53, P = 0
Prdx5
Dhrs1
Twf1
Pcyox1
Pdxk
Gamt
Mocs2
Maf
Pctk3
Daglb
Mm.393049
Slc39a3
Samd8
3110073H01Rik
Slc11a2
Acp2
Vti1a
C530044N13Rik
Ttyh2
Tmem135
Cyhr1
Klc4
Tmem19
Hs1bp3
Mospd2
Dpp7
D6Wsu116e
Slc35b4
1110014N23Rik
Tpp1
Vps18
Casp9
Tmem129
Sumf1
Ttc39c
Tmem49
BC004044
Ado
Mtch2
Srxn1
ctx
eye
hrt
art
wbr
lng
cbm
hip
bmw
kid
str
spc
hsc
gon
adr
myo
gam
spl
coc
msl
thm
lvr
0.2
0.6
1
Absolute Correlation

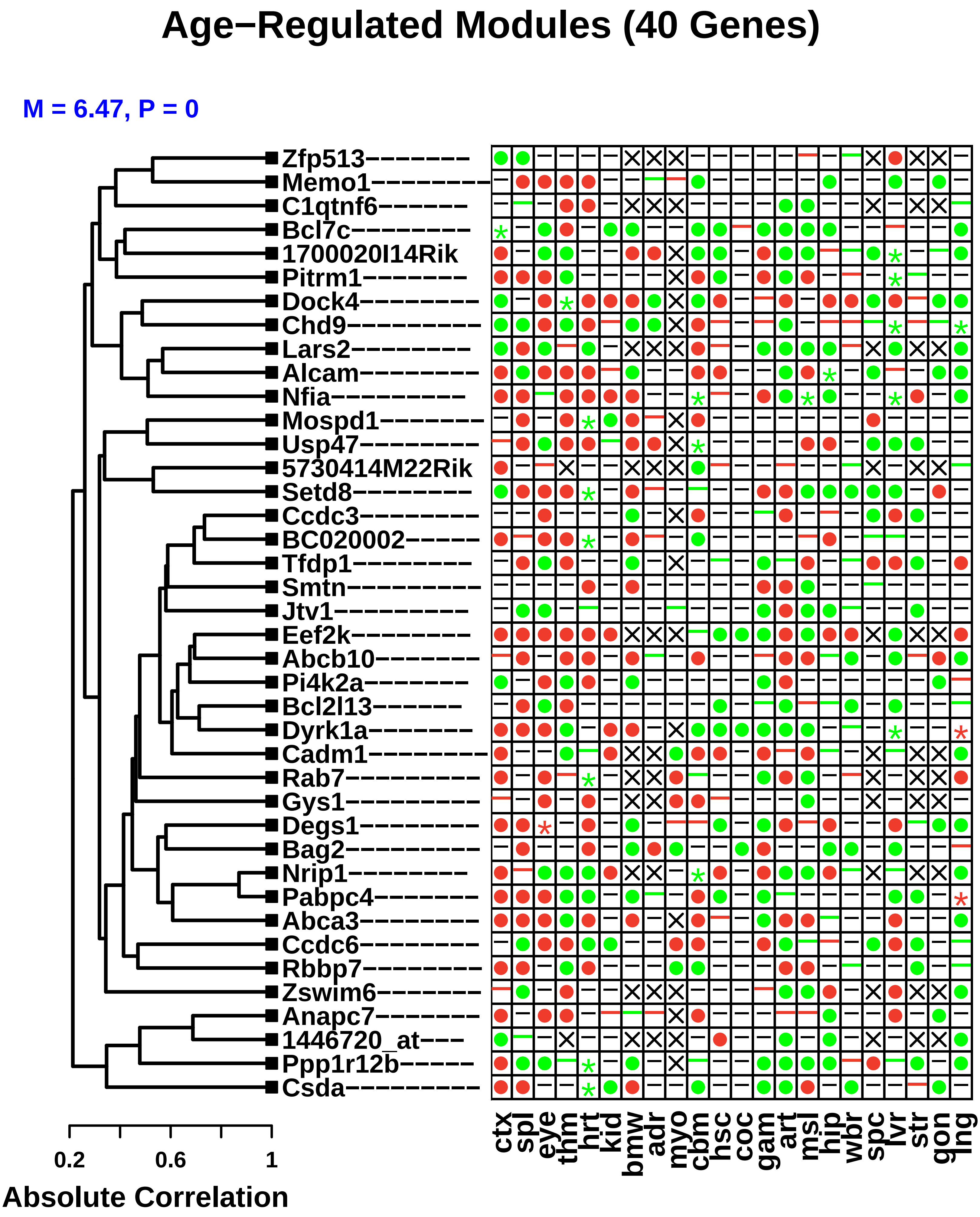

Age−Regulated Modules (40 Genes)
M = 6.47, P = 0
Zfp513
Memo1
C1qtnf6
Bcl7c
1700020I14Rik
Pitrm1
Dock4
Chd9
Lars2
Alcam
Nfia
Mospd1
Usp47
5730414M22Rik
Setd8
Ccdc3
BC020002
Tfdp1
Smtn
Jtv1
Eef2k
Abcb10
Pi4k2a
Bcl2l13
Dyrk1a
Cadm1
Rab7
Gys1
Degs1
Bag2
Nrip1
Pabpc4
Abca3
Ccdc6
Rbbp7
Zswim6
Anapc7
1446720_at
Ppp1r12b
Csda
ctx
spl
eye
thm
hrt
kid
bmw
adr
myo
cbm
hsc
coc
gam
art
msl
hip
wbr
spc
lvr
str
gon
lng
0.2
0.6
1
Absolute Correlation

Age−Regulated Modules (40 Genes)

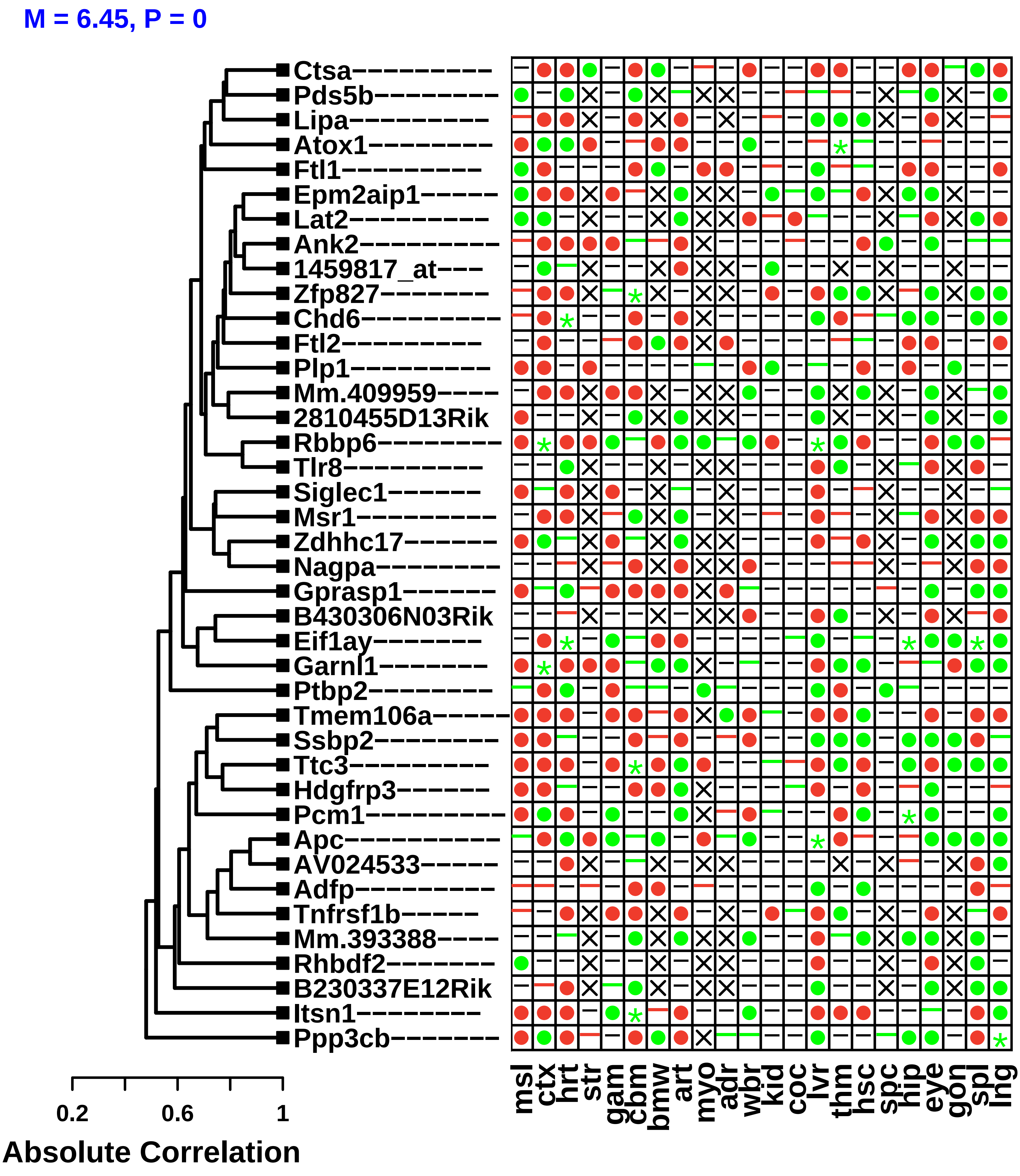

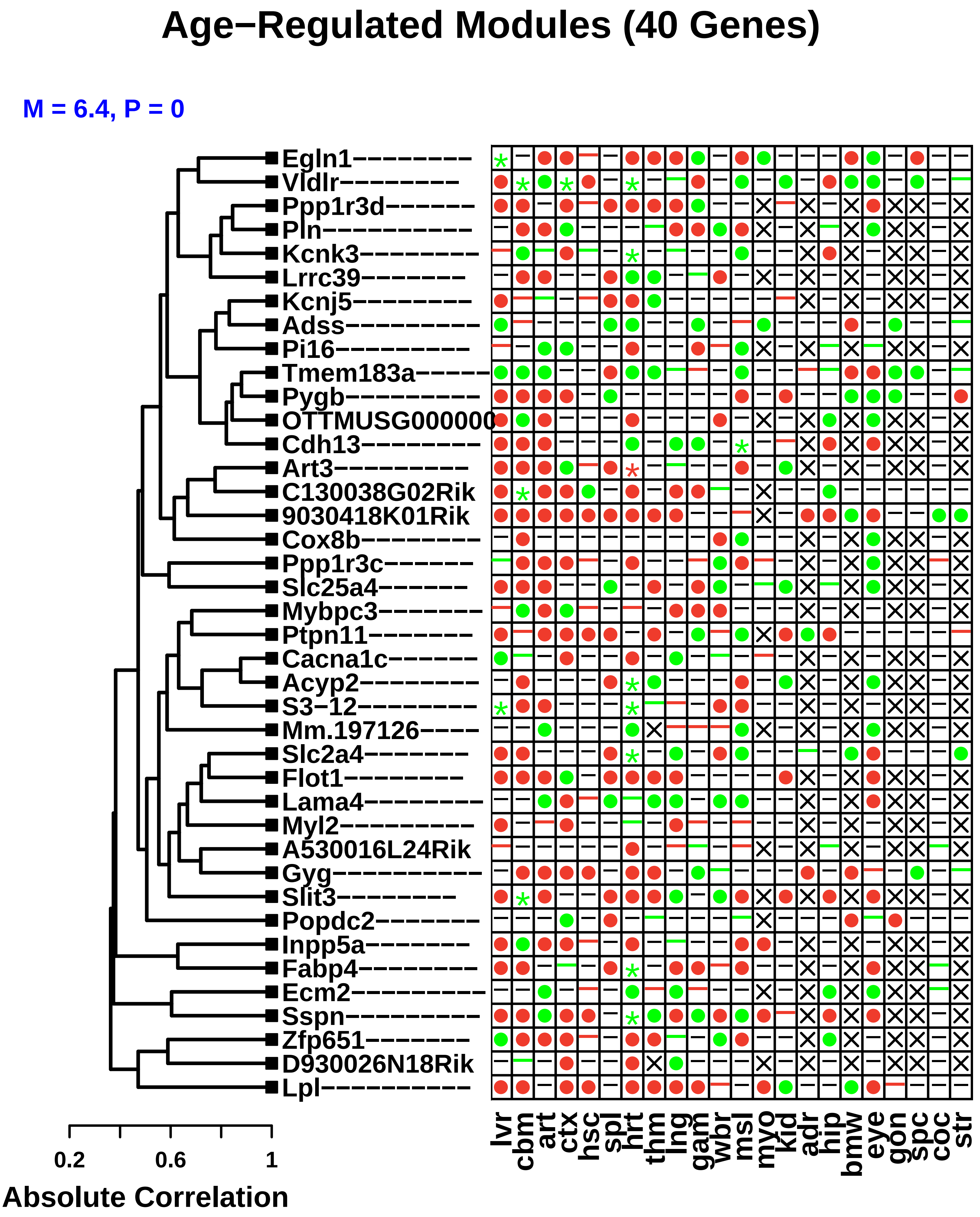

Age−Regulated Modules (40 Genes)
M = 6.4, P = 0
Egln1
Vldlr
Ppp1r3d
Pln
Kcnk3
Lrrc39
Kcnj5
Adss
Pi16
Tmem183a
Pygb
OTTMUSG000000
Cdh13
Art3
C130038G02Rik
9030418K01Rik
Cox8b
Ppp1r3c
Slc25a4
Mybpc3
Ptpn11
Cacna1c
Acyp2
S3−12
Mm.197126
Slc2a4
Flot1
Lama4
Myl2
A530016L24Rik
Gyg
Slit3
Popdc2
Inpp5a
Fabp4
Ecm2
Sspn
Zfp651
D930026N18Rik
Lpl
lvr
cbm
art
ctx
hsc
spl
hrt
thm
lng
gam
wbr
msl
myo
kid
adr
hip
bmw
eye
gon
spc
coc
str
0.2
0.6
1
Absolute Correlation

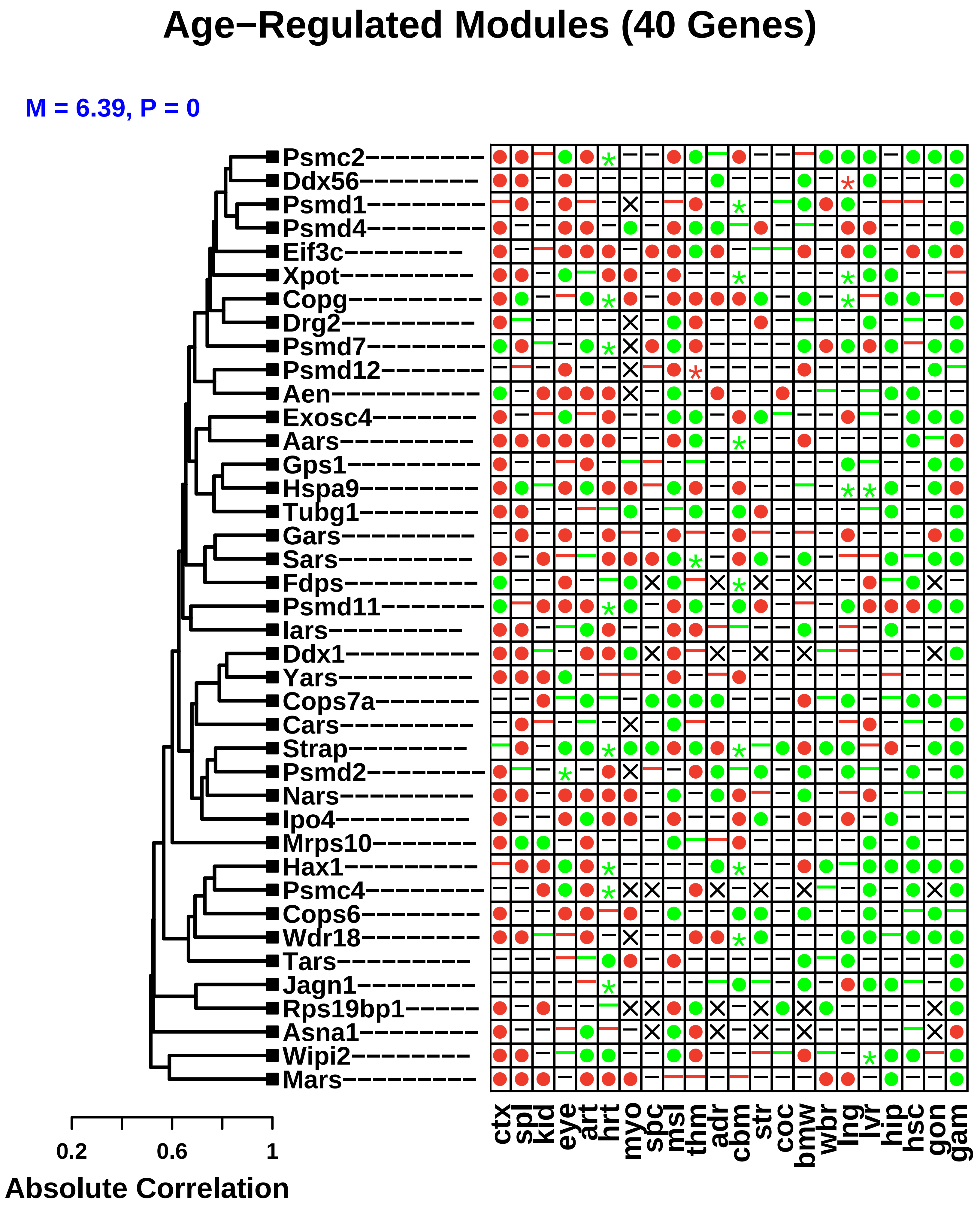

Age−Regulated Modules (40 Genes)
M = 6.39, P = 0
Psmc2
Ddx56
Psmd1
Psmd4
Eif3c
Xpot
Copg
Drg2
Psmd7
Psmd12
Aen
Exosc4
Aars
Gps1
Hspa9
Tubg1
Gars
Sars
Fdps
Psmd11
Iars
Ddx1
Yars
Cops7a
Cars
Strap
Psmd2
Nars
Ipo4
Mrps10
Hax1
Psmc4
Cops6
Wdr18
Tars
Jagn1
Rps19bp1
Asna1
Wipi2
Mars
ctx spl kid eye art hrt myo spc msl thm adr cbm str coc bmw wbr lng lvr hip hsc gon gam
0.2
0.6
1
Absolute Correlation

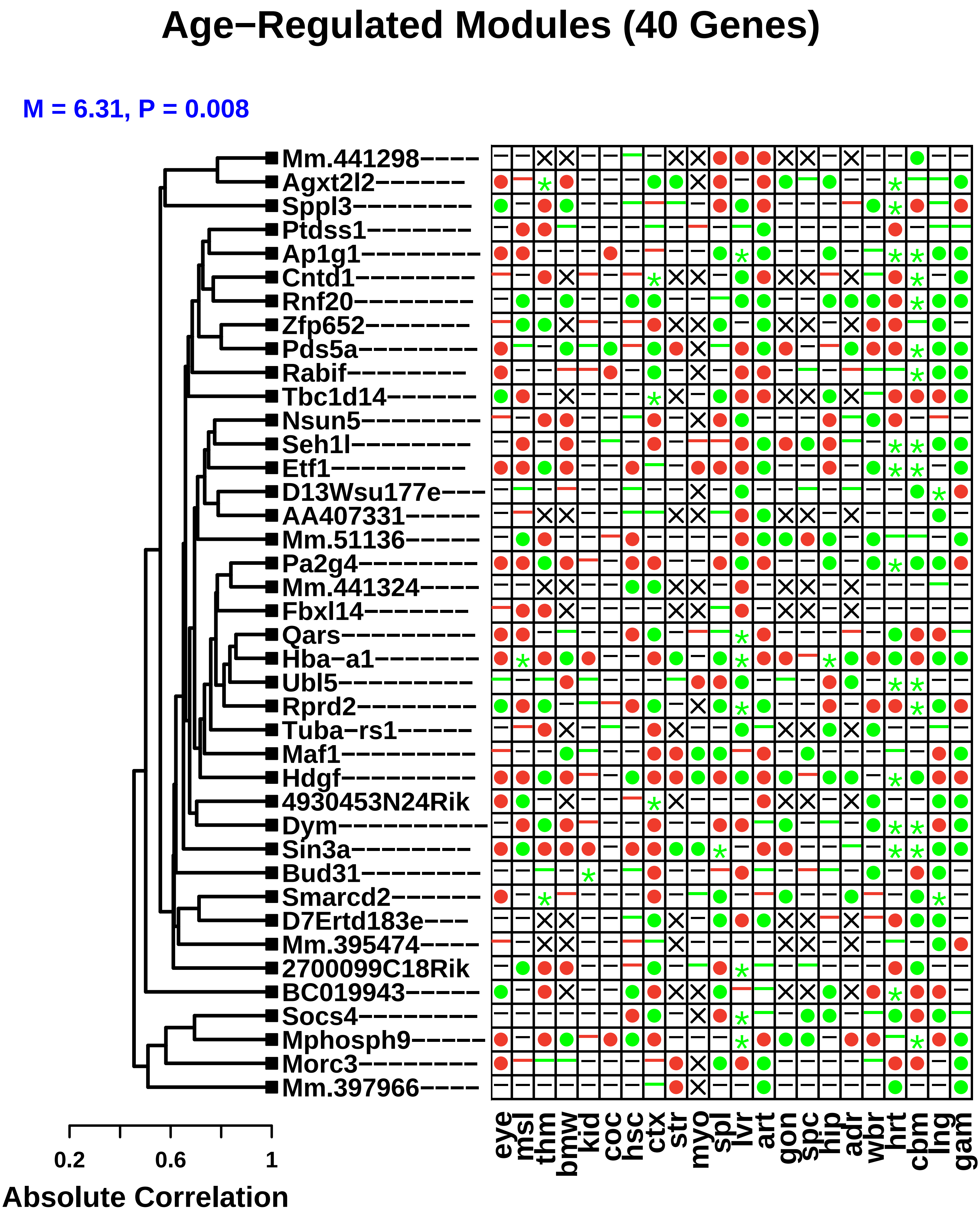
Age−Regulated Modules (40 Genes)
M = 6.31, P = 0.008
Mm.441298
Agxt2l2
Sppl3
Ptdss1
Ap1g1
Cntd1
Rnf20
Zfp652
Pds5a
Rabif
Tbc1d14
Nsun5
Seh1l
Etf1
D13Wsu177e
AA407331
Mm.51136
Pa2g4
Mm.441324
Fbxl14
Qars
Hba−a1
Ubl5
Rprd2
Tuba−rs1
Maf1
Hdgf
4930453N24Rik
Dym
Sin3a
Bud31
Smarcd2
D7Ertd183e
Mm.395474
2700099C18Rik
BC019943
Socs4
Mphosph9
Morc3
Mm.397966
eye
msl
thm
bmw
kid
coc
hsc
ctx
str
myo
spl
lvr
art
gon
spc
hip
adr
wbr
hrt
cbm
lng
gam
0.2
0.6
1
Absolute Correlation

Age−Regulated Modules (40 Genes)

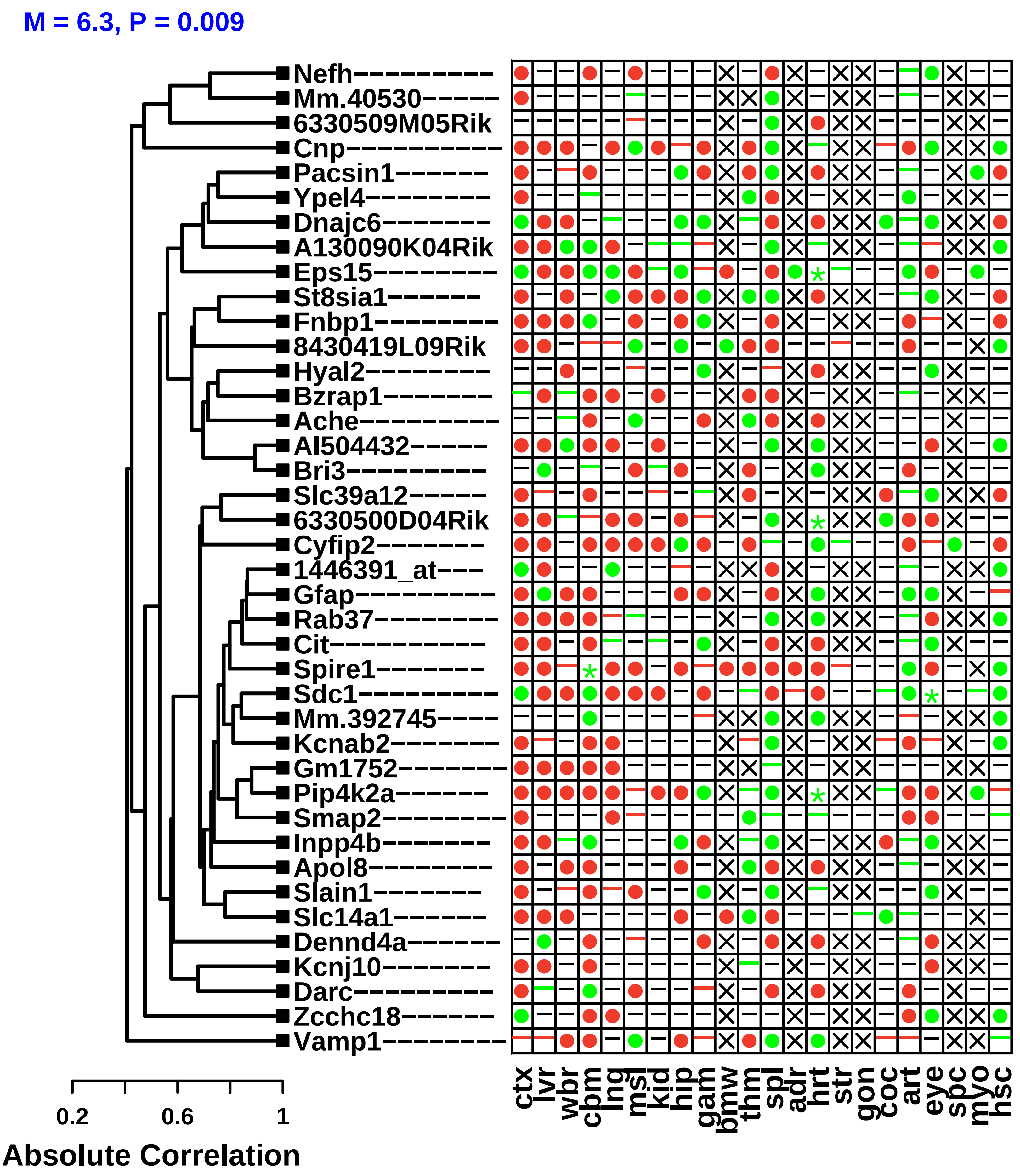

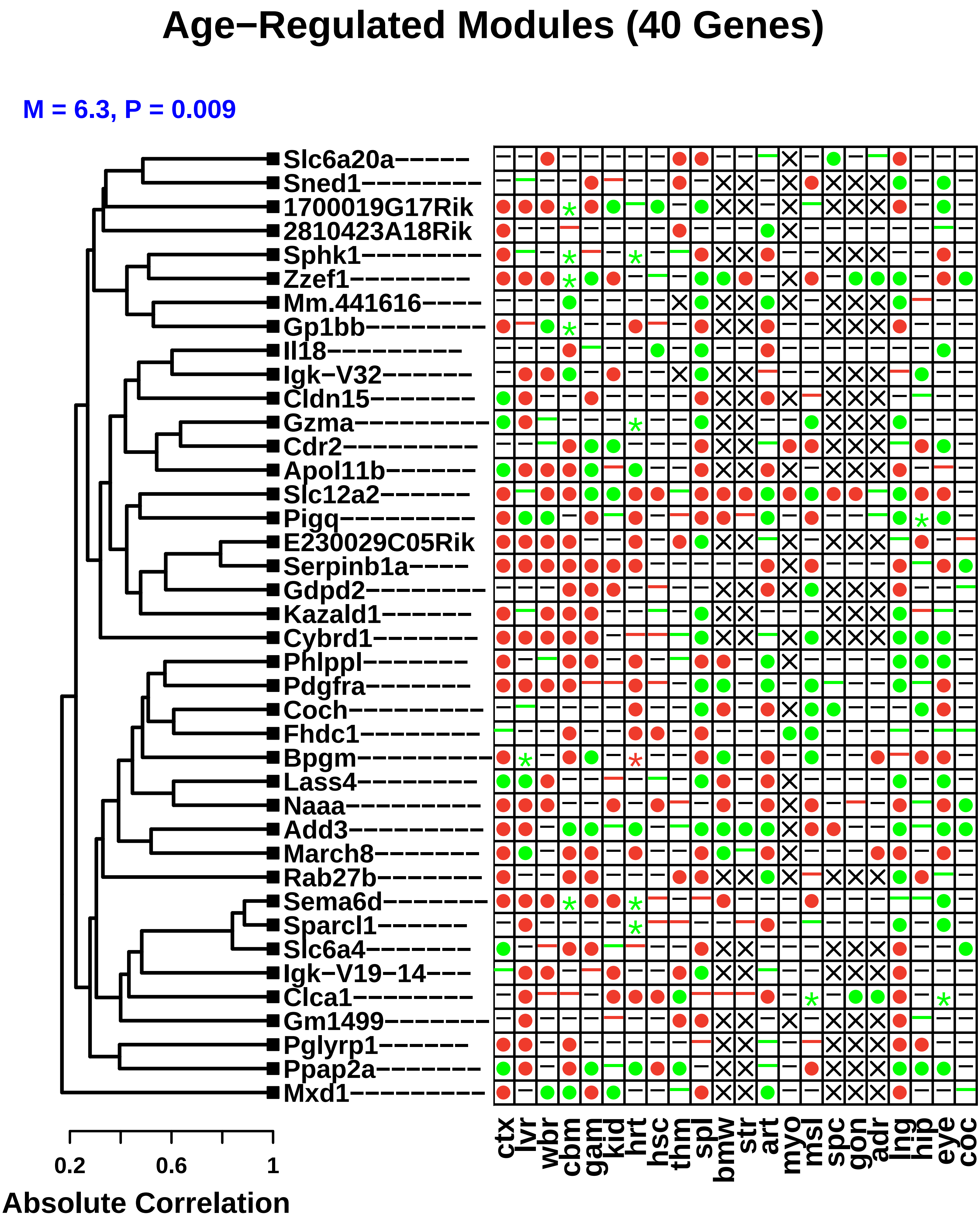

Age−Regulated Modules (40 Genes)
M = 6.3, P = 0.009
Slc6a20a
Sned1
1700019G17Rik
2810423A18Rik
Sphk1
Zzef1
Mm.441616
Gp1bb
Il18
Igk−V32
Cldn15
Gzma
Cdr2
Apol11b
Slc12a2
Pigq
E230029C05Rik
Serpinb1a
Gdpd2
Kazald1
Cybrd1
Phlppl
Pdgfra
Coch
Fhdc1
Bpgm
Lass4
Naaa
Add3
March8
Rab27b
Sema6d
Sparcl1
Slc6a4
Igk−V19−14
Clca1
Gm1499
Pglyrp1
Ppap2a
Mxd1
ctx lvr wbr cbm gam kid hrt hsc thm spl bmw str art myo msl spc gon adr lng hip eye coc
0.2
0.6
1
Absolute Correlation

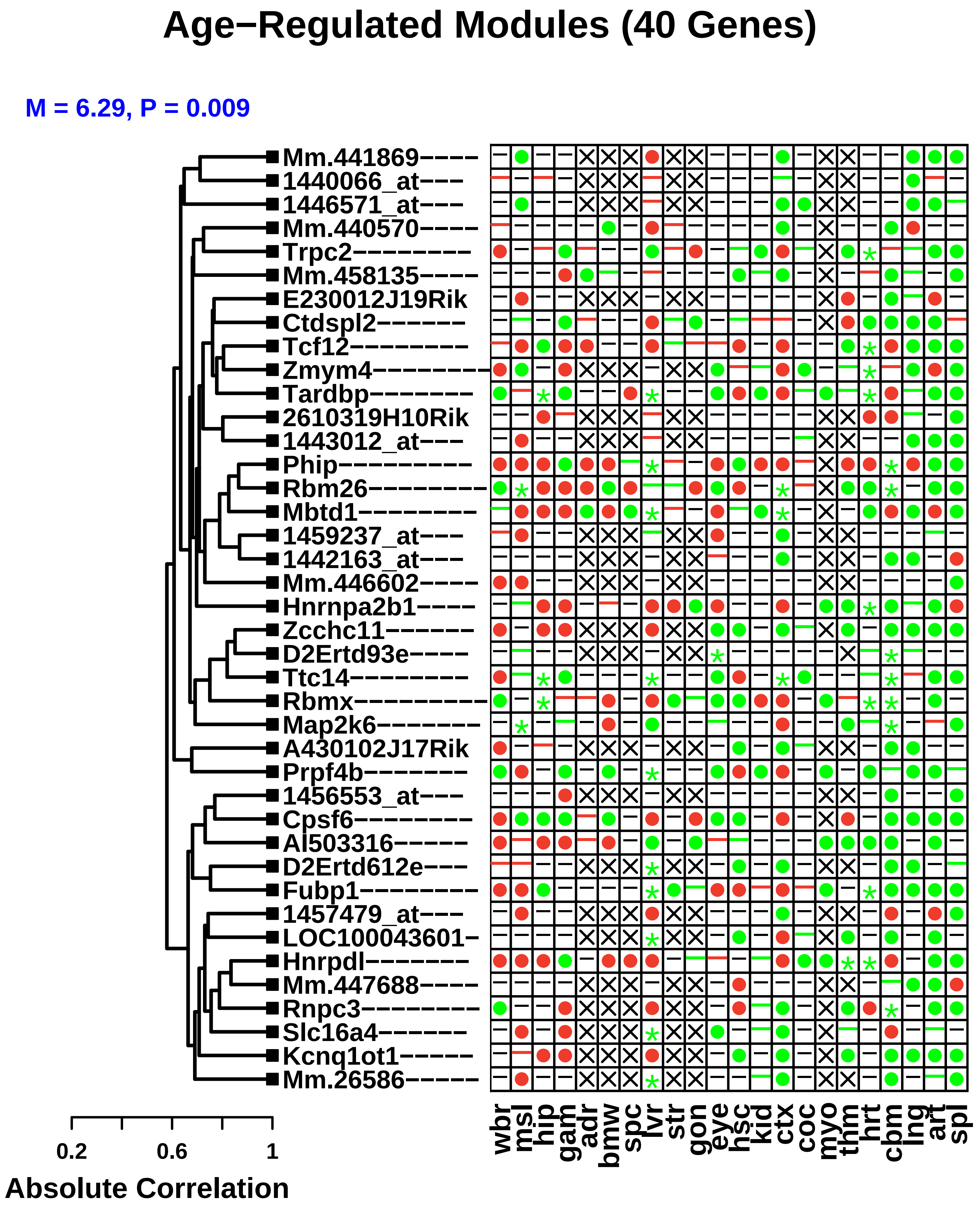

Age−Regulated Modules (40 Genes)
M = 6.29, P = 0.009
Mm.441869
1440066_at
1446571_at
Mm.440570
Trpc2
Mm.458135
E230012J19Rik
Ctdspl2
Tcf12
Zmym4
Tardbp
2610319H10Rik
1443012_at
Phip
Rbm26
Mbtd1
1459237_at
1442163_at
Mm.446602
Hnrnpa2b1
Zcchc11
D2Ertd93e
Ttc14
Rbmx
Map2k6
A430102J17Rik
Prpf4b
1456553_at
Cpsf6
AI503316
D2Ertd612e
Fubp1
1457479_at
LOC100043601
Hnrpdl
Mm.447688
Rnpc3
Slc16a4
Kcnq1ot1
Mm.26586
wbr msl hip gam adr bmw spc lvr str gon eye hsc kid ctx coc myo thm hrt cbm lng art spl
0.2
0.6
1
Absolute Correlation

Age−Regulated Modules (40 Genes)

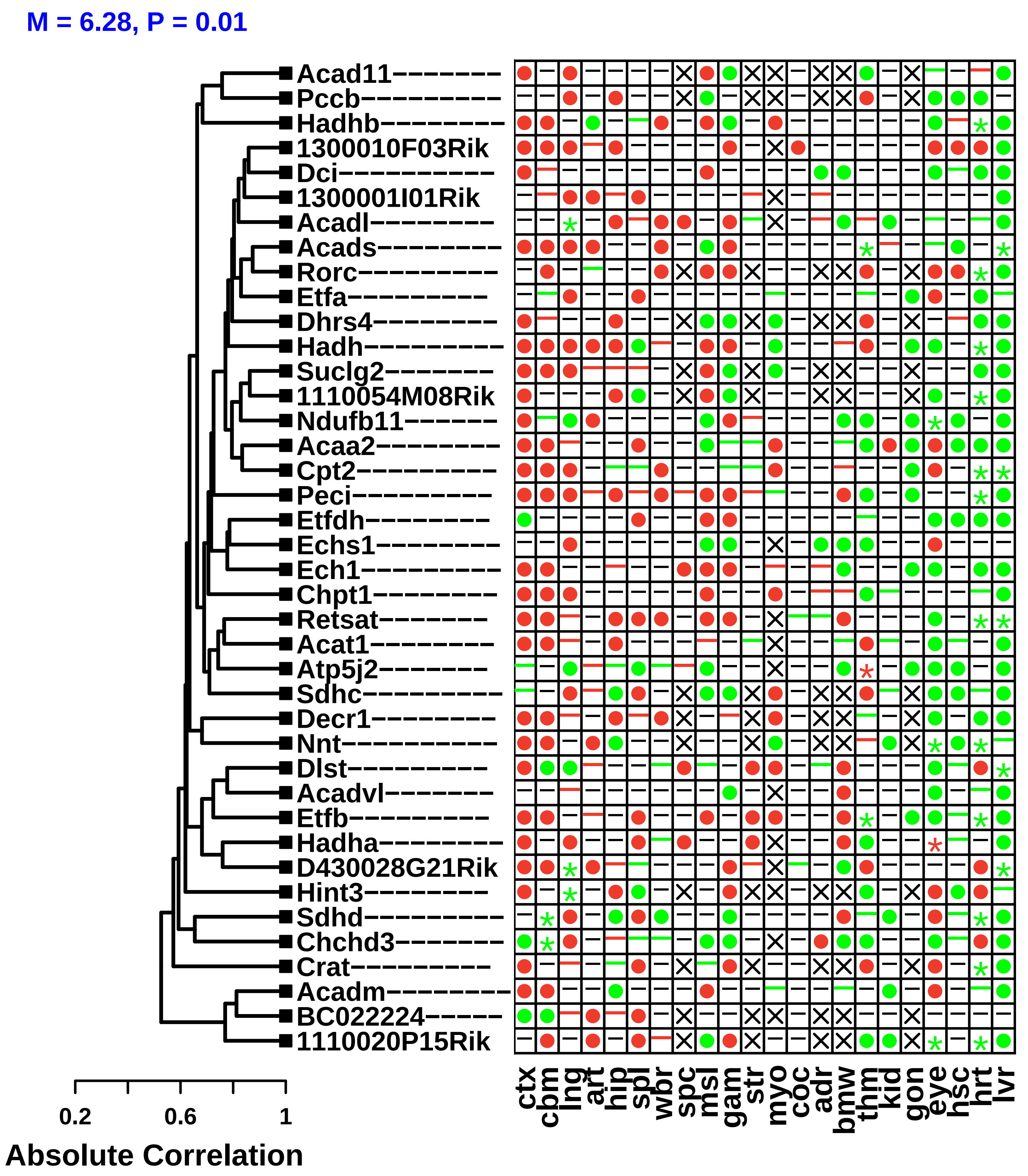

Age−Regulated Modules (40 Genes)

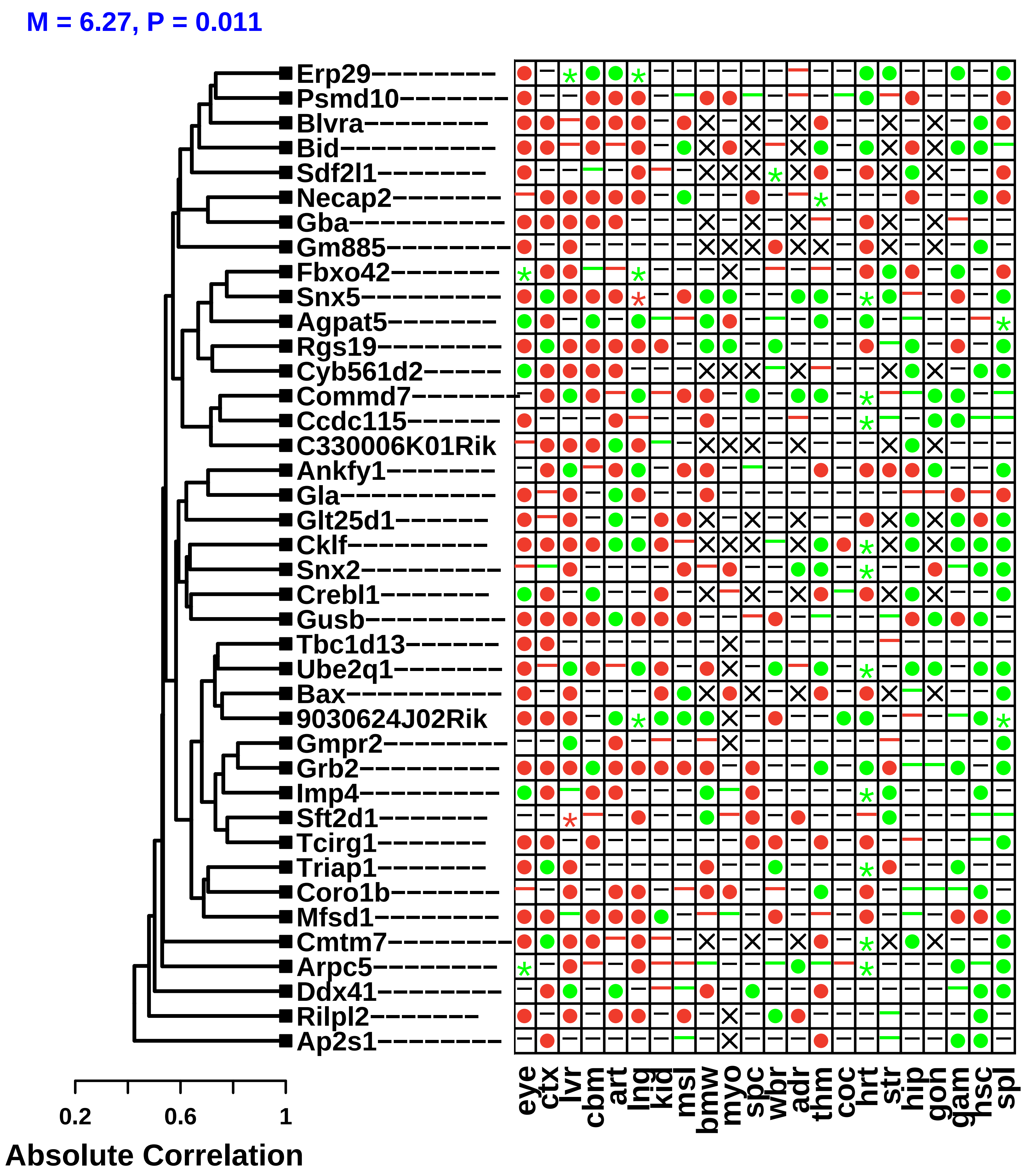

Age−Regulated Modules (40 Genes)

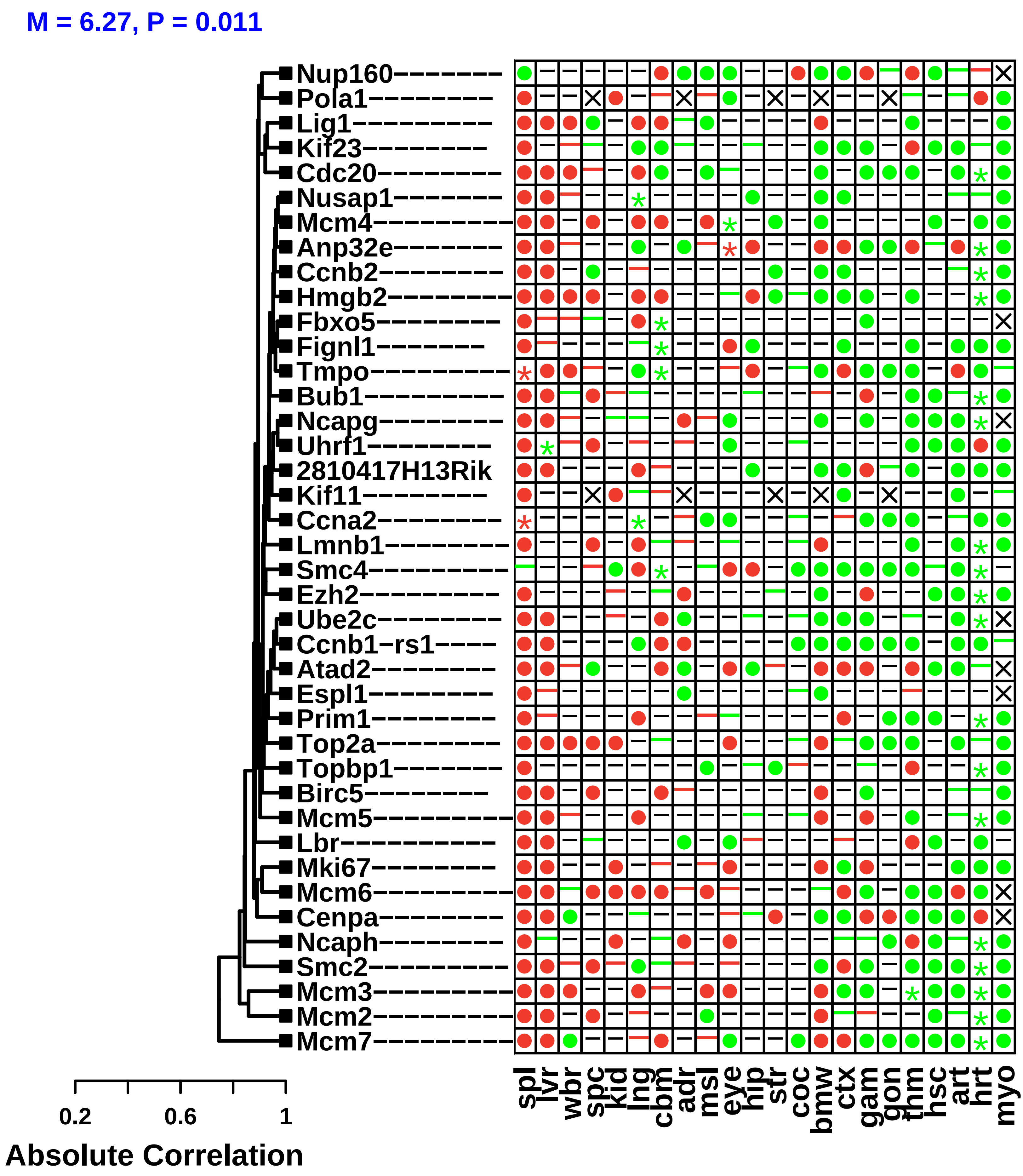

Age−Regulated Modules (40 Genes)

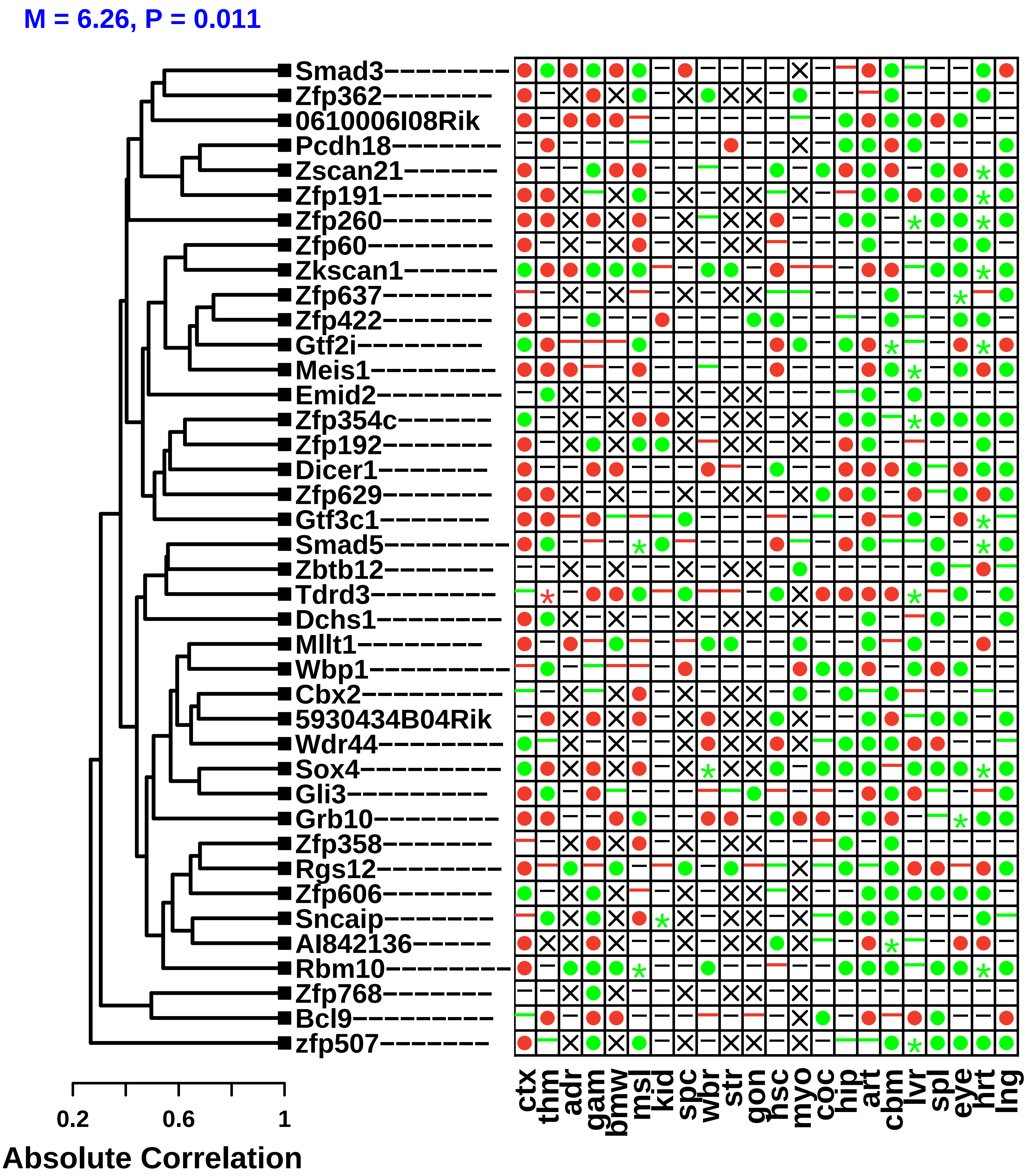

Age−Regulated Modules (40 Genes)

M = 6.25, P = 0.013

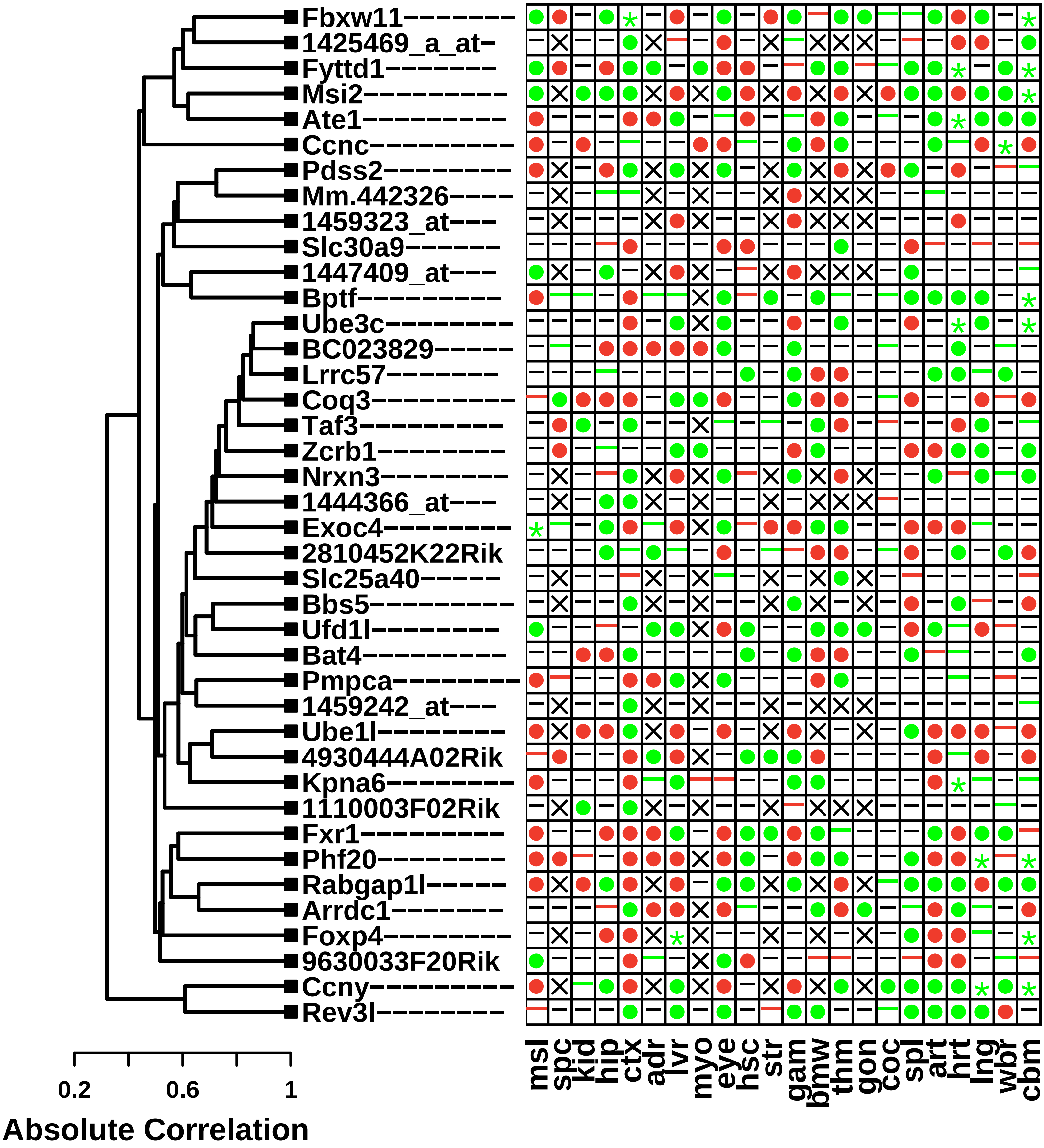

Supplement: Additional file 11 — Gene expression modules regulated by aging in multiple mouse tissues. This file provides a description of the most significant age-regulated co-expression modules. Co-expression modules of varying size are shown (2, 3, 5, 10, 20 and 40 genes), along with their patterns of differential expression across the mouse tissues examined (e.g., see Figure 6). [file 1471-2164-10-585-S11.PDF]
